# Solvent Effects on the Selectivity of Ambimodal Dipolar/Diels–Alder Cycloadditions: A Study Using Explicit Solvation Models

**DOI:** 10.1002/cphc.202500494

**Published:** 2025-10-01

**Authors:** Hayato Matsubuchi, Daiki Hayashi, Daichi Okamoto, Aoi Noguchi, Shoto Nakagawa, Toshiyuki Takayanagi, Tatsuhiro Murakami

**Affiliations:** ^1^ Department of Chemistry Saitama University Shimo‐Okubo 255, Sakura‐ku Saitama City Saitama 338‐8570 Japan; ^2^ Department of Applied Chemistry for Environment Tokyo Metropolitan University 1‐1 Minami‐Osawa Hachioji‐shi Tokyo 192‐0397 Japan

**Keywords:** explicit solvation models, GFN2‐xTB, molecular dynamics, nonequilibrium solvation effects, post‐transition state bifurcation

## Abstract

The reaction between 2‐aminoacrolein and 1,3‐butadiene serves as a representative example of post‐transition state bifurcation (PTSB), leading to both Diels–Alder (4 + 2) six‐membered and dipolar (4 + 3) seven‐membered cycloaddition products via a single ambimodal transition state structure. Previous quantum chemical studies employing an implicit solvation model have highlighted the significant influence of polar solvents on the branching dynamics of this bifurcation, primarily due to the stabilization of charge‐separated dipolar intermediates. Herein, the PTSB behavior is investigated in further detail using an explicit solvation model comprising up to 45 water molecules. Both static reaction path calculations and molecular dynamics simulations are carried out on water cluster models of selected sizes, employing the parameter‐optimized semiempirical GFN2‐xTB method, which accurately reproduces results obtained from density functional theory. The findings reveal that the PTSB dynamics are highly sensitive to the number of water molecules involved.

## Graphical abstract

Post‐transition state bifurcation in the reaction of 2‐aminoacrolein with 1,3‐butadiene is investigated using explicit solvation (up to 45 water molecules). GFN2‐xTB dynamics reveal strong solvent‐size effects on branching to (4 + 2) and (4 + 3) cycloadducts via an ambimodal transition state.

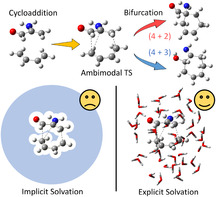

## 1 Introduction

The transition state (TS) is a fundamentally important concept in understanding chemical reaction mechanisms and dynamics.^[^ ^1^, ^2^, ^3^ ^]^ Within the well‐known Born–Oppenheimer framework, the TS is characterized mathematically as a first‐order saddle point on the multidimensional potential energy surface, which is constructed by solving quantum electronic structure problems at a series of fixed nuclear configurations. The geometry of the TS also serves as the local maximum of the minimum energy path, which continuously connects the local minima representing the reactant and product species. This reaction pathway is commonly referred to as the intrinsic reaction coordinate (IRC) in the quantum chemistry field and has been used to understand the detailed nuclear rearrangement through the reaction. In recent years, numerous theoretical investigations have revealed an alternative reaction mechanism termed post‐transition state bifurcation (PTSB), wherein a single TS gives rise to multiple products via a bifurcating region on the potential energy surface.^[^ ^3^, ^4^, ^5^, ^6^, ^7^, ^8^ ^]^ Considerable effort has been directed toward clarifying the nuclear dynamics of reactions exhibiting PTSB since the branching ratios of products in such processes cannot be reliably determined using only the static characteristics of the potential energy surface.^[^ ^9^, ^10^, ^11^, ^12^, ^13^, ^14^, ^15^, ^16^, ^17^ ^]^

In this work, we theoretically study the PTSB dynamics of the cycloaddition reaction between 2‐aminoacrolein and 1,3‐butadiene, a system previously explored in a pioneering quantum chemical study by Houk and co‐workers.^[^ ^18^ ^]^ Using on‐the‐fly molecular dynamics simulations at the density functional theory (DFT) level, this reaction has been established as a prototypical example of PTSB, wherein a single TS (commonly referred to as an ambimodal TS) leads to two structurally distinct products: the conventional six‐membered Diels–Alder (4 + 2) adduct and a seven‐membered (4 + 3) product formed via a dipolar cycloaddition pathway.

A particularly interesting aspect of this reaction is that product branching is significantly influenced by the presence of polar solvent molecules. In the gas phase, the IRC calculation starting from the ambimodal TS preferentially leads to the Diels–Alder (4 + 2) six‐membered product, and on‐the‐fly nuclear dynamics calculations originating from the TS region indicate that the Diels–Alder cycloaddition is the predominant outcome. In contrast, when employing the polarizable continuum model to simulate water solvation (i.e., the implicit solvation model), the IRC connects to the (4 + 3) seven‐membered ring through a dipolar cycloaddition mechanism.^[^ ^18^, ^19^ ^]^ Moreover, corresponding dynamics simulations incorporating the polarizable continuum model indicate a branching fraction of ≈6:4 in favor of the six‐membered ring.^[^ ^18^ ^]^ Thus, the formation of the (4 + 2) product in aqueous solution proceeds through non‐IRC dynamics.

Houk and co‐workers concluded that the polar solvent environment significantly stabilizes the charge‐separated dipolar structures located around the bifurcation region, leading to differences in the potential energy surface feature between the gas phase and the solution phase.^[^ ^18^ ^]^ However, it is important to note that previous nuclear dynamics simulations neglected explicit solvent motions entirely.

In recent years, there has been increasing interest in elucidating how explicit solvent motions influence reaction dynamics, both from experimental observations and theoretical modeling, with the goal of overcoming the inherent limitations of implicit continuum solvation models.^[^ ^20^, ^21^, ^22^, ^23^, ^24^, ^25^, ^26^, ^27^, ^28^, ^29^, ^30^ ^]^ For example, Roytman and Singleton recently demonstrated that solvation dynamics play a crucial role in the nonequilibrium behavior of ion‐pair intermediates in carbocation reactions, highlighting the shortcomings of the implicit solvation model.^[^ ^24^ ^]^ Their study also suggests that the implicit continuum solvation model should be applied with caution in reactions where the timescales of solute and solvent motions are comparable. In this work, we investigate the PTSB dynamics of the cycloaddition reaction between 2‐aminoacrolein and 1,3‐butadiene by explicitly incorporating solvent molecule motions. This approach aims to provide deeper insight into the influence of solvent dynamics on bifurcation behavior.

## 2 Results and Discussion

### 2.1 IRC Calculations with the Explicit Solvation Model

**Figure 1:**
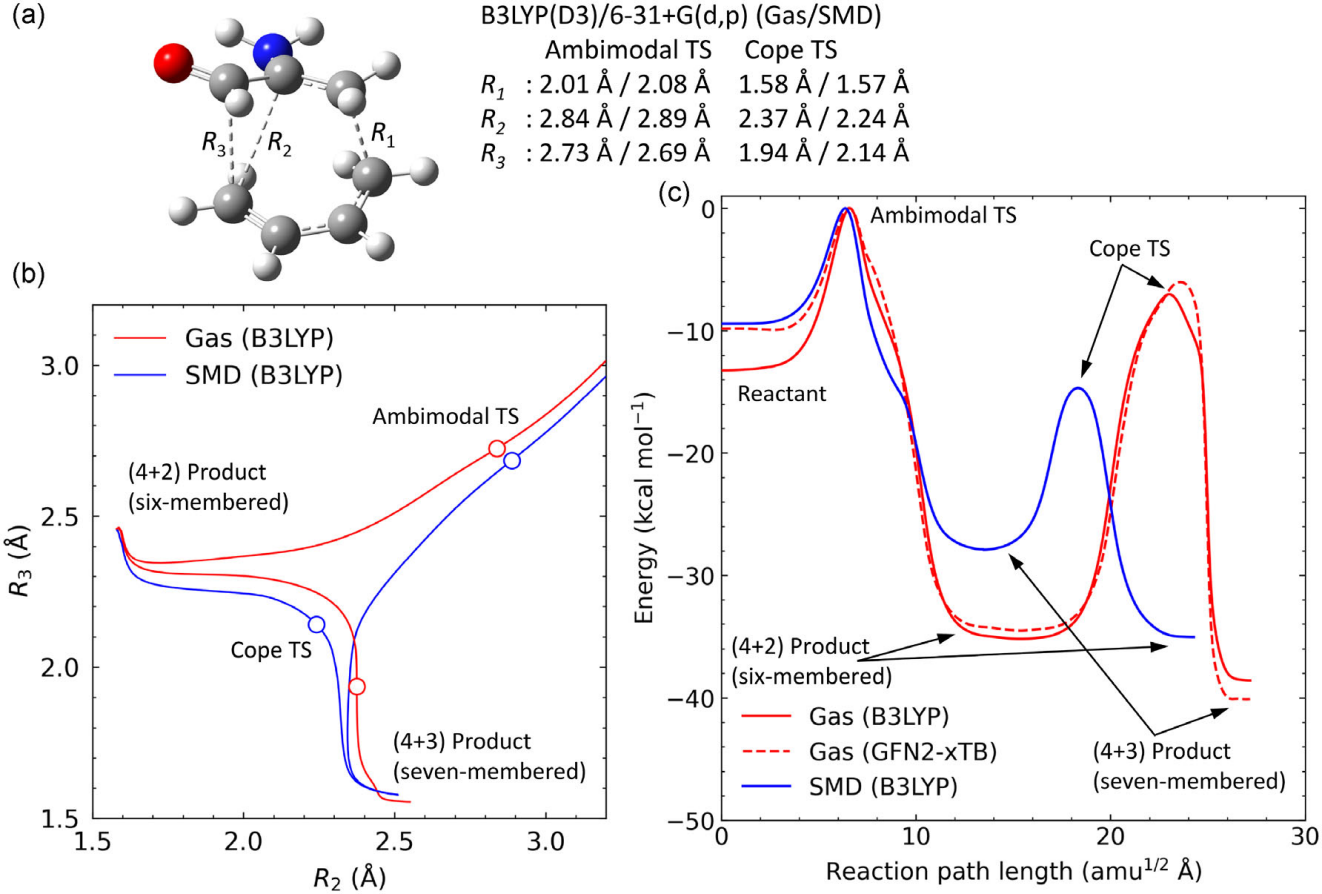
a) Definition of the three key C—C internuclear distances involved in the PTSB reaction of 2‐aminoacrolein with 1,3‐butadiene, along with the corresponding DFT results. b) IRC profiles calculated using the B3LYP(D3)/6‐31+G(d,p) method, plotted as a function of (*R*_2_, *R*_3_). Results for both the gas phase (red lines) and solution phase (blue lines) are shown. c) Potential energy profiles along the IRC pathways obtained from DFT (solid lines) and GFN2‐xTB (dashed line) calculations.

**Figure** **1**a presents three critical C—C bond distances (*R*_1_, *R*_2_, and *R*_3_), which play an essential role in characterizing the PTSB dynamics of the cycloaddition reaction between 2‐aminoacrolein and 1,3‐butadiene. At the ambimodal TS, *R*_1_ is ≈ 2 Å. A subsequent shortening of *R*_2_ results in the formation of the (4 + 2) six‐membered ring, while a decrease in *R*_3_ leads to the (4 + 3) seven‐membered ring. These reaction pathways are visualized by projecting the IRC trajectories onto the (*R*_2_, *R*_3_) coordinate plane, as shown in Figure 1b. The IRC paths were computed at the B3LYP^[^ ^31^, ^32^, ^33^ ^]^ (D3)/6‐31+G(d,p) level of density functional theory, including Grimme's D3 dispersion correction.^[^ ^34^ ^]^ Calculations were performed using the Gaussian09 quantum chemistry package^[^ ^35^ ^]^ with the GRRM code.^[^ ^36^, ^37^, ^38^ ^]^ This level of theory is consistent with that employed in the earlier work by Houk and co‐workers.^[^ ^18^ ^]^ Within the GRRM program, the IRC calculations were conducted using the options “SaddleFC = 1” and “MaxStepSize=0.1’’ to determine the saddle point, whereas “DownSize=5” and “DownFC = 5’’ were applied to generate the IRC path.

Figure 1b presents the IRC results, with red lines corresponding to gas‐phase DFT calculations and blue lines representing those obtained using the SMD implicit solvation model for water. As previously discussed, the IRC trajectory originating from the ambimodal TS in the gas phase smoothly progresses toward the (4 + 2) six‐membered ring. In contrast, the water‐solvated IRC preferentially yields the (4 + 3) seven‐membered ring. These two products are linked via a transition structure known as the “Cope TS.” Although the overall topologies of the IRC pathways remain broadly similar across both environments, the Cope TS position is notably different. In the gas phase, the Cope TS shifts toward the region associated with the (4 + 3) form.

Figure 1c illustrates the corresponding potential energy profiles along the IRCs, with all relative energies referenced to the ambimodal TS. These profiles clearly indicate that solvation in water significantly stabilizes the Cope TS, a consequence attributed to its dipolar, charge‐separated nature—an effect previously highlighted by Houk and co‐workers.^[^ ^18^ ^]^ This figure also includes the IRC energy profile calculated using the semiempirical GFN2‐xTB method (dashed line),^[^ ^39^, ^40^, ^41^ ^]^ for which the carbon atomic parameters were optimized to reproduce B3LYP(D3) energies and gradients.^[^ ^19^ ^]^ The parameters are provided in Table S1, Supporting Information. This optimized GFN2‐xTB level is employed in subsequent computations involving explicit solvation models, including reaction pathway analyses and molecular dynamics simulations, to reduce the overall computational cost. As a benchmark, we compared the GFN2‐xTB results with those obtained from B3LYP(D3)/6‐31+G(d,p) calculations for several representative IRC pathways in a (H_2_O)_5_ water cluster. The results, presented in Figure S1, Supporting Information, show that the GFN2‐xTB method relatively reproduces the DFT energy profiles and IRC shapes for these microsolvated systems throughout the reaction coordinate. These benchmarks confirm that the method effectively captures the key solute–solvent interaction effects relevant to the bifurcation dynamics discussed in this article. It is also worth mentioning that the accuracy of the GFN2‐xTB method in describing the water–water and molecule–water interactions has been extensively addressed in previous theoretical studies.^[^ ^42^, ^43^, ^44^ ^]^

The IRC calculations were performed using a size‐selected cluster model, in which the 2‐aminoacrolein + 1,3‐butadiene system was solvated by a selected number of water molecules. Cluster configurations containing 5, 15, or 45 water molecules were employed. Initially, the TS structure was optimized for each solvation configuration. Initial guess structures were generated randomly using the Packmol code,^[^ ^45^ ^]^ keeping the 2‐aminoacrolein and 1,3‐butadiene structures fixed at the ambimodal TS geometry obtained in the gas phase. These structures were then optimized, including the solvent molecules, using the GRRM code, and subsequent IRC calculations were performed.

**Figure 2:**
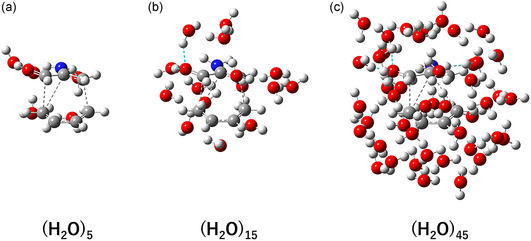
Typical ambimodal TS structures optimized using the GFN2‐xTB method for the PTSB reaction of 2‐aminoacrolein with 1,3‐butadiene solvated by a) 5, b) 15, and c) 45 H_2_O molecules.

Representative optimized ambimodal TS structures are shown in **Figure** **2**. In the system solvated by five water molecules, the oxygen and nitrogen atoms of 2‐aminoacrolein are preferentially solvated through strong hydrogen bonding. Additionally, it is evident that ≈45 water molecules are required to form a contiguous solvation layer covering the organic reactive solute.

**Figure 3:**
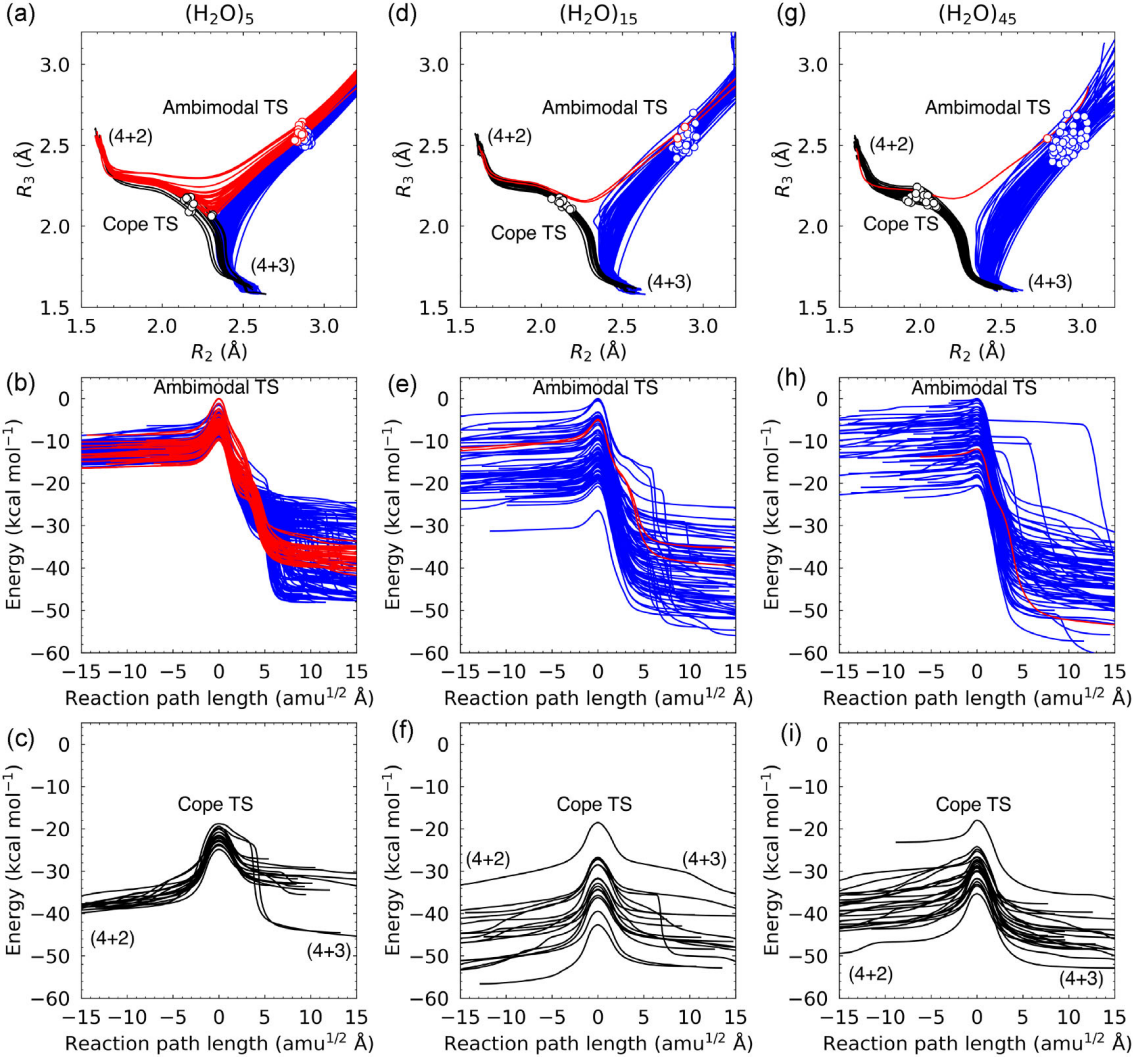
IRC analysis of the bifurcation between the (4 + 2) and (4 + 3) cycloaddition pathways in water clusters of varying size: a–c) (H_2_O)_5_, d–f) (H_2_O)_15_, and g–i) (H_2_O)_45_. (a,d,g) IRC trajectories projected onto the (*R*_2_, *R*_3_) coordinate plane for pathways initiated from the ambimodal TS, with TS geometries indicated by open circles. Red and blue lines correspond to trajectories leading to (4 + 2) and (4 + 3) forms, respectively. (b,e,h) Potential energy profiles along the IRC pathways starting from the ambimodal TS. (c,f,i) Potential energy profiles along the IRC pathways starting from the Cope TS, which leading to (4 + 2) and (4 + 3) forms.

We optimized 224, 70, and 73 ambimodal TS and 19, 16, and 24 Cope TS structures for the 5‐, 15‐, and 45‐water clusters, respectively, and performed corresponding IRC calculations, to qualitatively understand the effect of water solvation on the IRC profiles. The results are shown in **Figure** **3**, where the left, middle, and right sets of three panels correspond to the results of the cluster systems with 5, 15, and 45 water molecules, respectively. The top panels (Figures 3a,d,g) display IRC pathways projected onto the (*R*_2_, *R*_3_) coordinate plane. For the cluster containing (H_2_O)_5_, Figure 3a illustrates that 44 IRCs from the ambimodal TS lead to the formation of the (4 + 2) six‐membered ring, while 180 pathways result in the (4 + 3) seven‐membered ring. This finding suggests that variations in solvation structures can significantly influence the product distribution in the PTSB system. As the number of water molecules increases, the number of IRCs leading to the formation of the (4 + 3) seven‐membered ring becomes more prevalent. For the cluster system containing 15 water molecules, 2 IRC pathways originating from the ambimodal TS lead to the (4 + 2) six‐membered ring product, while 68 trajectories proceed toward the (4 + 3) seven‐membered ring, as shown in Figure 3d. Similarly, in the case of the 45‐water‐molecule system, 72 IRC pathways lead to the formation of the seven‐membered ring, as illustrated in Figure 3g.

**Figure 4:**
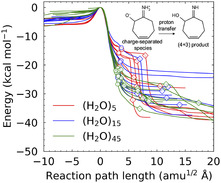
IRC profiles for the formation of the (4 + 3) seven‐membered ring in systems solvated with (H_2_O)_5_ (red), (H_2_O)_15_ (blue), and (H_2_O)_45_ (green). Circular and diamond markers indicate key structural points corresponding to *R*_3_ = 1.80 Å and *R*_3_ = 1.63 Å, respectively. A schematic illustration (top right) shows the charge‐separated species and the subsequent proton transfer that yields the (4 + 3) product.

The middle panels (Figures 3b,e,h) display the potential energy profiles along the IRC pathways passing through the ambimodal TS structure. As illustrated in these figures, multiple IRC pathways emerge due to variations in the solvation configurations. Notably, the overall curvature trends remain consistent across the cluster systems containing 5, 15, and 45 water molecules. **Figure** **4** displays the 6, 8, and 9 randomly selected IRCs leading to the (4 + 3) seven‐membered ring for systems solvated with (H_2_O)_5_ (red), (H_2_O)_1__5_ (blue), and (H_2_O)_4__5_ (green), respectively. Circular and diamond markers indicate positions corresponding to *R*_3_ = 1.80 and 1.63 Å, respectively. As the number of water molecules increases, the IRCs exhibit increasingly steeper curvature up to *R*_3_ = 1.80 Å, reflecting enhanced stabilization of the charge‐separated species^[^ ^18^ ^]^ through more extensive coordination by the explicit polar solvent. It is also noteworthy that the significant stabilization beyond the shoulder region, particularly in the (H_2_O)_5_ system, arises from proton transfer processes.

Another noteworthy observation is the significant decrease in the energy levels of the Cope TS as the number of water molecules increases, as illustrated in Figures 3c,f,i. This result indicates that the explicit water model at the semiempirical GFN2‐xTB level qualitatively reproduces the trend seen with the DFT results using the implicit SMD solvation model shown in Figure 1c. In addition to the reduction in Cope TS barrier height, it is also notable that the position of the Cope TS shifts slightly toward the (4 + 2) six‐membered product region as the number of water molecules increases, as illustrated in Figures 3a,d,g. It should be emphasized that these atomic‐level behaviors depending on the number of water molecules cannot be easily obtained from the implicit solvation model. All Cartesian coordinates of the optimized ambimodal TS structures are provided in Table S2, Supporting Information.

Overall, the findings shown in Figure 3 indicate that molecular dynamics simulations employing the present explicit solvation model, based on the semiempirical GFN2‐xTB method with optimized atomic parameters, would produce results comparable to those obtained from more computationally demanding on‐the‐fly DFT calculations.

### 2.2 Molecular Dynamics Calculations with the Explicit Solvation Model

In this section, we present the results of extensive molecular dynamics calculations for the current PTSB system using the explicit solvation model. All molecular dynamics simulations were performed using the PIMD code developed by Shiga.^[^ ^46^ ^]^ We have slightly modified the PIMD code to interface with the GFN2‐xTB calculations. The newly defined codes are provided in the Supporting Information. Classical mechanics was applied in these molecular dynamics simulations; thus, nuclear quantum effects and quantized vibrational energies were completely neglected in this study. The dynamics calculations were initiated from structures near the ambimodal TS region. Specifically, the initial structure and its associated atomic velocities were randomly selected from a series of structures obtained via constant‐temperature molecular dynamics simulations (at *T* = 300 K), using three appropriate harmonic bias potentials applied to the *R*_1_, *R*_2_, and *R*_3_ internuclear distances. The centers of the bias potentials were set at 2.06, 2.81, and 2.59 Å for *R*_1_, *R*_2_, and *R*_3_, respectively, which correspond to the internuclear distances optimized at the ambimodal TS structure in the gas phase with the GFN2‐xTB method.^[^ ^19^ ^]^ To control the system temperature, we employed the Nóse–Hoover chain thermostat. Once the initial structure and its associated atomic velocities were chosen, we have calculated the production trajectories by removing the bias potentials. A total of 1000 trajectories were integrated for each cluster size with a time step of 0.1 fs up to 500 fs.

**Figure 5:**
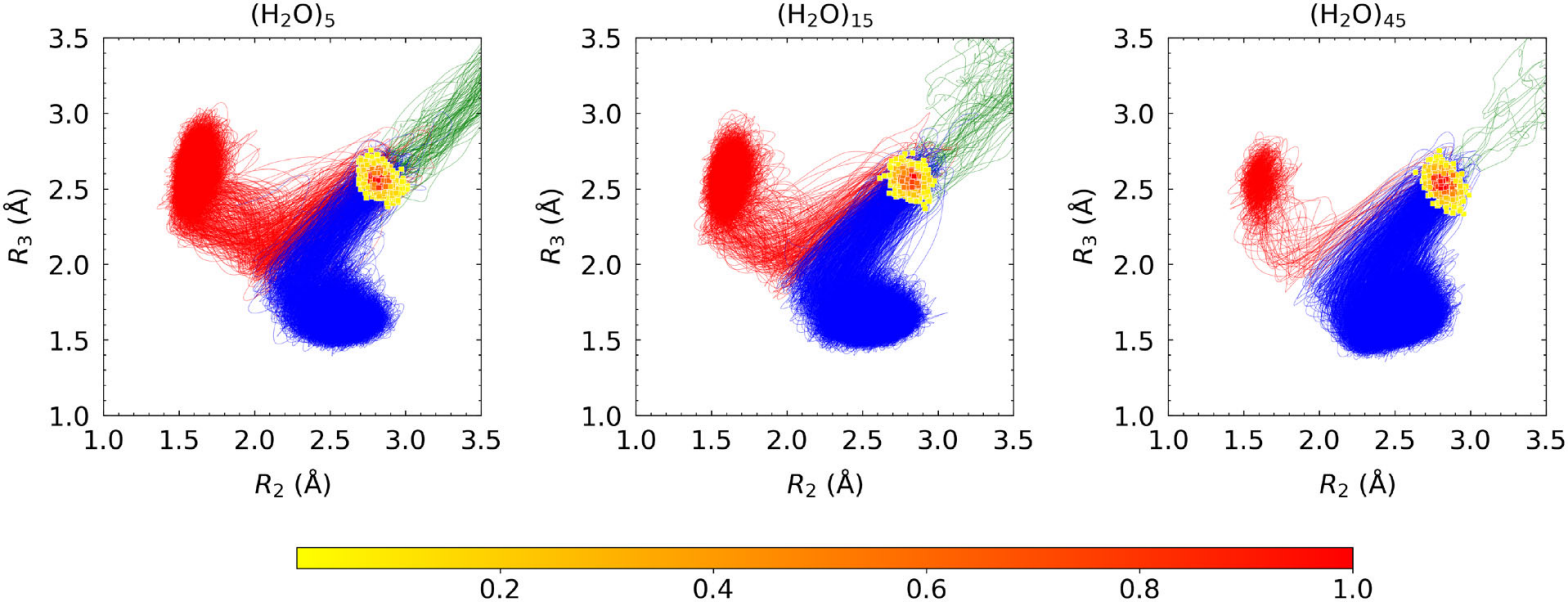
Initial structural distributions from 1000 trajectory simulations projected onto the (*R*_2_, *R*_3_) coordinate plane. The colormap represents the population density of initial structures, with warmer colors indicating regions of higher density. The trajectories leading to the (4 + 2) and (4 + 3) rings, as well as to the reactants, are displayed in red, blue, and green, respectively.

**Figure** **5** displays the 2D distributions of the 1000 initial configurations obtained from trajectory calculations incorporating bias potentials, plotted with respect to *R*_2_ and *R*_3_ for the three cluster systems. These distributions indicate that the initial structures are primarily concentrated in the vicinity of the ambimodal TS regions identified in the IRC analyses shown in Figure 3. Additionally, Figure 5 depicts the 1000 trajectories obtained from molecular dynamics simulations. In this figure, trajectories that result in the formation of the (4 + 2) six‐membered ring are depicted in red, whereas those that lead to the (4 + 3) seven‐membered ring are shown in blue. As anticipated from the IRC profiles in Figures 3a,d,g, the formation of the seven‐membered ring becomes increasingly favored with a greater number of water molecules. It should be noted that this work focuses on the branching between the six‐membered and seven‐membered ring pathways. In the present analysis, the charge‐separated intermediate and the final seven‐membered ring product are not explicitly distinguished.

**Table** **1** provides a summary of the branching fractions for the (4 + 2) and (4 + 3) forms, as well as the number of trajectories leading to each final structure in the reaction between 2‐aminoacrolein and 1,3‐butadiene. It also presents a comparison between the gas‐phase results obtained using the current sampling method based on bias potentials and previous data^[^ ^19^ ^]^ generated via normal mode sampling at the ambimodal TS, involving 2000 trajectories. The branching fractions between the (4 + 2) and (4 + 3) forms derived from both sampling approaches are in good agreement within statistical uncertainty, thereby validating the reliability of the present sampling strategy. In addition, Table 1 reports branching fractions derived from on‐the‐fly DFT simulations performed by Houk and co‐workers^[^ ^18^ ^]^ for both gas‐phase and aqueous systems using the implicit solvation model. It is worth noting that their calculations employed the quasiclassical trajectory method, which wedges quantized vibrational energy into a classical dynamics framework. Although the gas‐phase outcomes are in strong agreement with the present study, a significant discrepancy arises in the aqueous‐phase results: the explicit solvation model adopted here predicts an inverted trend in product distribution compared to that observed with the implicit solvation model. This divergence between the implicit and explicit solvation models closely resembles the bifurcation behavior observed in the HCl + 1,3‐pentadiene system, where similar discrepancies were reported between implicit and explicit treatments.^[^ ^24^ ^]^

**Table 1:** (4 + 2)/(4 + 3) branching fractions and the number of trajectories (values in the parentheses) leading to the three different final structures of trajectory calculations.

System	(4 + 2) form	(4 + 3) form	Reactants
GFN2‐xTB			
(H_2_O)_5_	0.387 (348)	0.613 (552)	(100)
(H_2_O)_15_	0.233 (221)	0.767 (727)	(52)
(H_2_O)_45_	0.044 (44)	0.956 (948)	(8)
Gas [this study]	1.000 (443)	0.000 (0)	(557)
Gas [ref. [19]]	0.996 (841)	0.004 (3)	(1156)
B3LYP(D3)/6‐31+G(d,p)			
Gas^a)^	0.997 (661)	0.003 (2)	(14)
Implicit (SMD)a)	0.609 (255)	0.391 (164)	(27)

a) Taken from ref. [18], where the quasiclassical trajectory method was employed.

**Figure 6:**
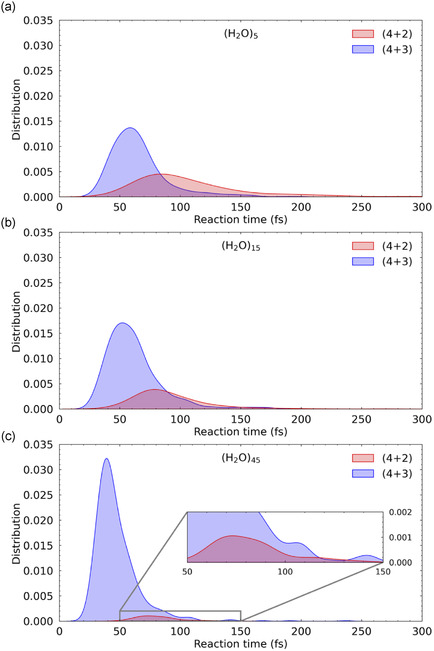
Reaction time distributions for the formation of (4 + 2) (red) and (4 + 3) (blue) rings, obtained from dynamical simulations initiated from the ambimodal TS in the presence of a) (H_2_O)_5_, b) (H_2_O)_15_, and c) (H_2_O)_45_ water clusters.

To gain insights into the reaction dynamics, we evaluated the time required for each trajectory—initiated near the ambimodal TS—to reach either the (4 + 2) or (4 + 3) form. The formation was defined as the point along a trajectory at which the *R*_2_ or *R*_3_ bond distance becomes shorter than 1.8 Å. Reaction time distributions were then obtained by averaging over all reactive trajectories, and the results are presented in **Figure** **6**.

A principal outcome of the current explicit solvation model is the pronounced dependence of reaction time distributions on the number of water molecules. In particular, as the number of water molecules increases, the peak times for seven‐membered ring formation shift to earlier values, and the distributions become significantly narrower. This observation suggests that explicit solvation substantially stabilizes the charge‐separated species along the pathway from the ambimodal TS to the seven‐membered ring, which is consistent with the steeper gradients of the potential energy surface observed with increasing numbers of water molecules, as shown in Figure 4. This inference aligns qualitatively with the IRC profiles depicted in Figure 3. Conversely, although the time distributions for six‐membered ring formation become more localized with increasing solvation, the peak times remain nearly unchanged at approximately *t* ≈ 80 fs across all cluster sizes and are similarly observed in the gas phase (see Figure S2, Supporting Information). This behavior implies that the (4 + 2) reaction dynamics are only marginally affected by solvation, likely due to the absence of substantial stabilization by polar environments in this channel. Across all three cluster sizes, the (4 + 2) and (4 + 3) pathways display distinctly different temporal characteristics: notably, the (4 + 2) product is generally formed later than the (4 + 3) counterpart. By contrast, xTB‐based simulations employing an implicit solvent model reported nearly identical formation times for both the (4 + 2) and (4 + 3) rings,^[^ ^19^ ^]^ implying that nonequilibrium interactions with explicit water molecules facilitate faster formation of the seven‐membered ring.

**Figure 7:**
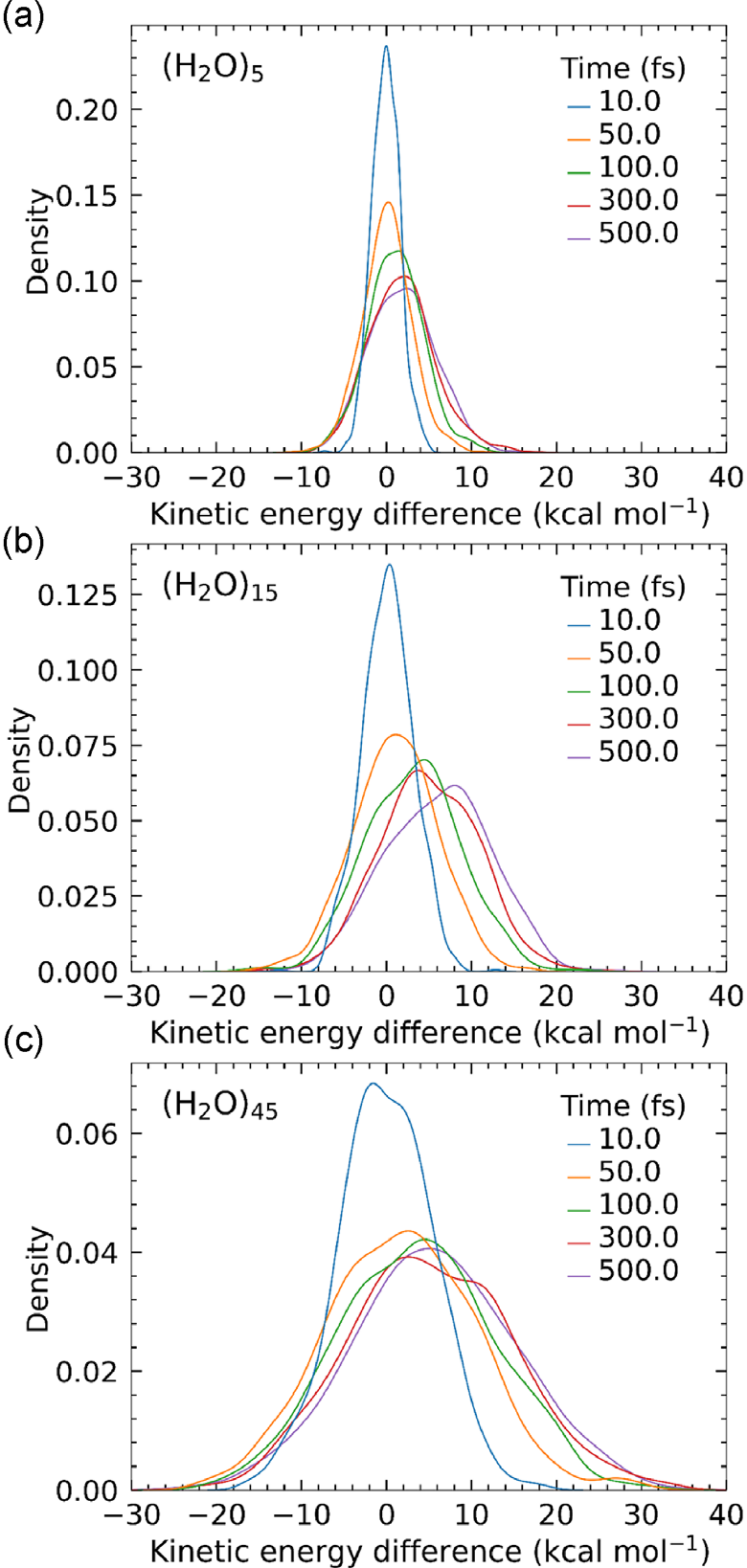
Distribution functions of the change in kinetic energy of water molecules, plotted as a function of energy. Results are shown at five time points (10, 50, 100, 300, and 500 fs) for each cluster size: a) (H_2_O)_5_, b) (H_2_O)_15_, and c) (H_2_O)_45_.

Finally, we discuss the overall energy transfer process from the solute to the solvent molecules—an analysis that is not feasible using an implicit solvation model. For each trajectory, we computed the total kinetic energy of all solvent water atoms (both hydrogen and oxygen), denoted as *E*_k_(*t*), as a function of simulation time. We then evaluated the change in kinetic energy as Δ*E*_k_(*t*)=*E*_k_(*t*)–*E*_k_(0). The positive value of Δ*E*_k_(*t*) indicates a gain of kinetic energy by the solvent molecules, qualitatively corresponding to an increase in solvent temperature due to the reaction's exothermic nature. Conversely, negative values of Δ*E*_k_(*t*) indicate kinetic energy loss. These energy changes were averaged over the trajectories leading to the (4 + 3)/(4 + 2) rings and converted into time‐dependent distribution functions. The results are shown in **Figure** **7** for each cluster size. In all three cluster cases, the distributions at *t* = 10 fs peak sharply near zero energy, suggesting that energy transfer to the solvent is still minimal at this early dynamics stage. As time progresses, the distributions shift toward positive values, indicating increasingly energy transfer from the solute to the solvent as the reaction proceeds. This trend is consistent with the highly exothermic nature of the present PTSB reaction. Notably, the efficiency of early‐stage energy transfer increases with the number of water molecules. This observation is reasonable, as the exothermic energy is initially partitioned into the kinetic and vibrational modes of the solute molecules, 2‐aminoacrolein and 1,3‐butadiene. In the clusters with (H_2_O)_5_ and (H_2_O)_1__5_, 2‐aminoacrolein is preferentially solvated by water via hydrogen bonding, whereas 1,3‐butadiene remains largely unsolvated in these clusters. In contrast, in the (H_2_O)_4__5_ cluster system, the entire solute complex is fully embedded within the solvent environment. Thus, it can be concluded that the water solvent motions are playing some important roles in determining the overall branching dynamics through energy transfer between the solute and solvents. While it is important to note that these molecular dynamics simulations were performed using a purely classical mechanism framework—and may thus lack full accuracy in describing energy transfer processes—our findings offer a useful starting point for future theoretical work. In particular, these results highlight the potential for more accurate modeling using ring‐polymer molecular dynamics (RPMD) and suggest directions for extending the analysis to larger cluster or bulk liquid systems.

## 3 Conclusion

In this work, we conduct an extensive theoretical investigation of the PTSB reaction between 2‐aminoacrolein and 1,3‐butadiene, which leads to both Diels–Alder (4 + 2) six‐membered and dipolar (4 + 3) seven‐membered cycloaddition products via a single ambimodal TS structure. Our study employs an explicit solvation model based on the parameter‐optimized semiempirical GFN2‐xTB method. This computational research is strongly motivated by a previous theoretical study by Houk and co‐workers,^[^ ^18^ ^]^ which used an implicit solvation model to show that polar solvents significantly influence bifurcation dynamics by stabilizing the charge‐separated dipolar character in the Cope TS region of the potential energy surface. By systematically increasing the number of water molecules from 5 to 45, we demonstrate that microsolvation gradually alters the potential energy landscape, thereby affecting the bifurcation dynamics. This conclusion is supported by detailed atomic‐level analysis of molecular dynamics simulation results, including reaction time distributions and energy transfer between solute and solvent. Although the present simulations are based on purely classical mechanics molecular dynamics and thus do not capture nuclear quantum effects including quantum tunneling and quantized vibrational energy, we plan to report results from approximate quantum RPMD simulations^[^ ^47^, ^48^, ^49^, ^50^, ^51^ ^]^ in the near future. Previous study has demonstrated that RPMD can predict significantly faster proton transfer rates than classical MD due to tunneling contributions.^[^ ^52^ ^]^ We anticipate that a similar acceleration effect will be observed in the present PTSB system, particularly in solvent‐mediated proton transfer events. Additionally, we intend to extend our explicit solvation model to include other solvent systems to further understand the role of solvent molecules in the PTSB dynamics at a more quantitative level. Furthermore, while the current study employs size‐selected water cluster models to focus on short‐range solute–solvent interactions, future work will incorporate bulk solvent models with periodic boundary conditions (PBC) to capture long‐range electrostatic and collective solvent effects. Such PBC‐based simulations, possibly combined with QM/MM or machine‐learning potential approaches, will enable a more comprehensive evaluation of solvent influences on bifurcation dynamics.

## Conflict of Interest

The authors declare no conflict of interest.

## Author Contributions

**Hayato Matsubuchi**: data curation (lead); investigation (equal); visualization (lead). **Daiki Hayashi**: data curation (supporting); investigation (equal); visualization (supporting). **Daichi Okamoto**: data curation (supporting); investigation (equal); visualization (supporting). **Aoi Noguchi**: data curation (supporting); investigation (equal); visualization (supporting). **Shoto Nakagawa**: funding acquisition (supporting); investigation (equal); supervision (equal); visualization (supporting). **Toshiyuki Takayanagi**: conceptualization (supporting); funding acquisition (equal); investigation (equal); project administration (supporting); supervision (supporting); writing—original draft (equal); writing—review and editing (supporting). **Tatsuhiro Murakami**: conceptualization (lead); funding acquisition (equal); investigation (equal); methodology (lead); project administration (lead); supervision (lead); writing—original draft (equal); writing—review and editing (lead).

## Supporting information

Supplementary Material

## Data Availability

The data that support the findings of this study are available in the supplementary material of this article.
