# Supplementary material for: Solvent Effects on the Selectivity of Ambimodal Dipolar/Diels–Alder Cycloadditions: A Study Using Explicit Solvation Models
Source: Chemphyschem. 2025 Oct 1;26(22):e202500494. doi: 10.1002/cphc.202500494 (PMC12640666; doi:10.1002/cphc.202500494)
Supplement: Supplementary file 1 — Supplementary Material [file CPHC-26-e202500494-s001.zip › cphc.202500494-sup-0001-supdata.pdf]

Supplementary materials

Solvent Effects on the Selectivity of Ambimodal Dipolar/Diels–Alder Cycloadditions: A Study Using Explicit Solvation Models

Hayato Matsubuchi[1], Daiki Hayashi[1], Daichi Okamoto[1], Aoi Noguchi[1], Shoto Nakagawa[1], Toshiyuki Takayanagi [‡,1], and Tatsuhiro Murakami [*,†,2]

[1]Department of Chemistry, Saitama University, Shimo-Okubo 255, Sakura-ku, Saitama City, Saitama, 338-8570, Japan
[2]Department of Applied Chemistry for Environment, Tokyo Metropolitan University, 1-1 Minami-Osawa, Hachioji-shi, Tokyo, 192-0397, Japan

Contents

S1 Figure **S2**

S2 Tables **S3**

‡E-mail: tako@mail.saitama-u.ac.jp, ORCID:0000-0003-0563-9236
*Corresponding author
†E-mail: murakamit@tmu.ac.jp, ORCID: 0000-0001-8904-8673

S1 Figure

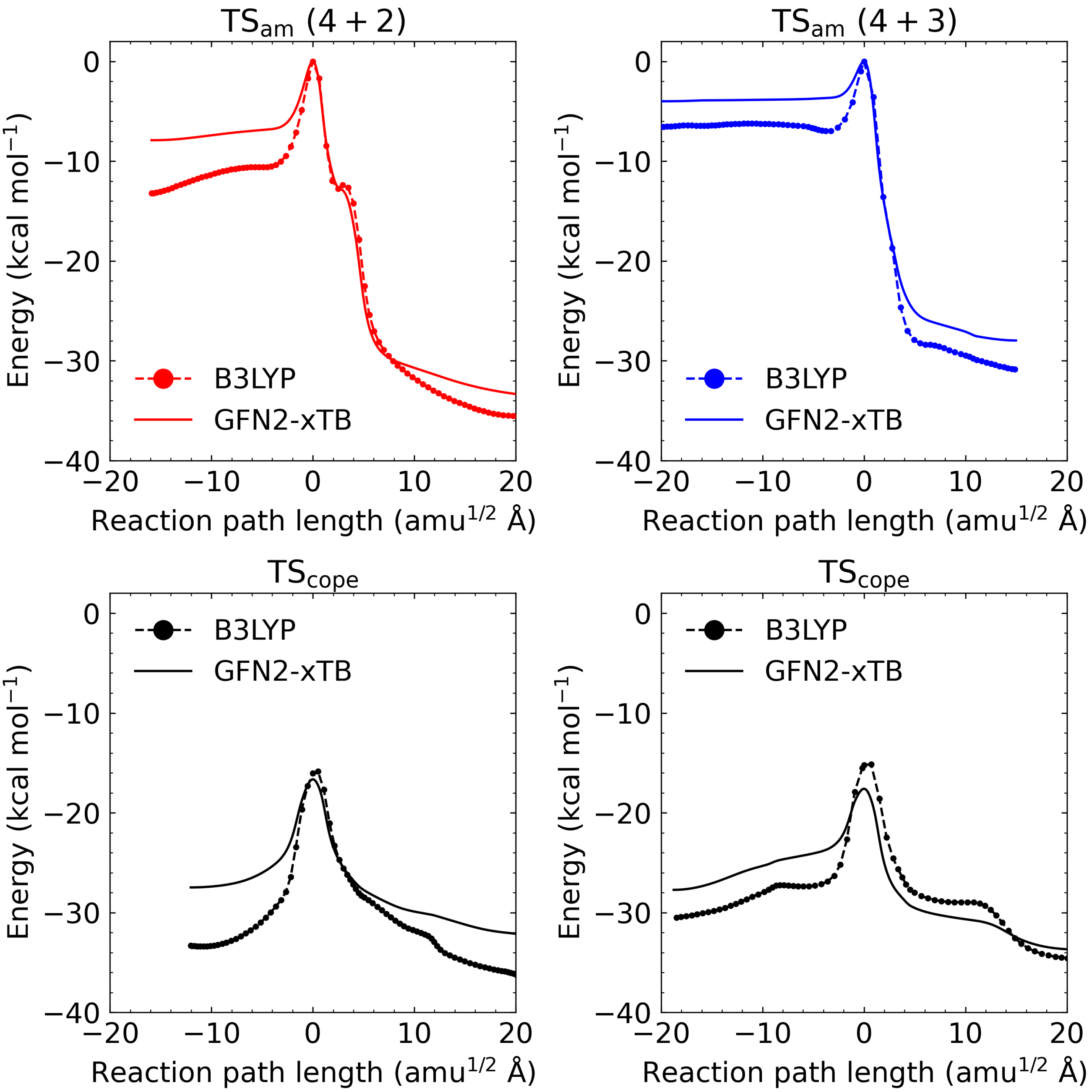

Figure S1: IRC compared with DFT and GFN2-xTB calculations for the (4+2) and (4+3) cycloaddition pathways in the $(H_2O)_5$ water cluster.

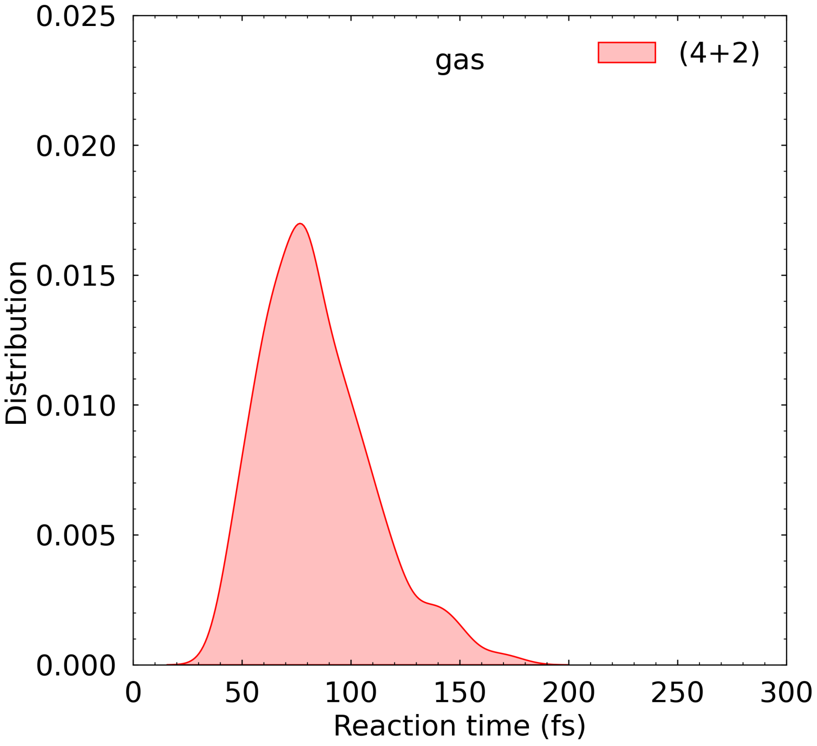

Figure S2: Distribution of (4+2) reaction times for individual trajectories in the gas phase.

S2 Tables

Table S1: Parameters for the 1,3-butadiene with 2-aminoacrolein reaction in gas-phase

```
$Z= 6  Optimized for N-compound Feb. 24, 2023.
 ao=2s2p
 lev  =  -14.854   -9.959
 exp  =    2.002    1.700
 GAM  =    0.400
 GAM3 =    1.418
 KCNS =   -0.102
 KCNP =    0.140
 DPOL =   -0.580
 QPOL =    0.103
 REPA =    1.291
 REPB =    4.203
 POLYS=   -2.039
 POLYP=   -0.276
 LPARP=    1.070
$end
```

Table S2: The xyz coodinates of TS structures

```
Ambimodal TS Gas
20
ANGSTROM
C      0.565370000000    1.524750000000    0.765970000000
C      1.729300000000    1.372590000000    0.054700000000
C      2.373650000000    0.131980000000   -0.163820000000
C      1.826270000000   -1.089300000000    0.251720000000
C      0.146320000000   -1.359890000000   -0.829570000000
C     -0.917890000000   -0.695030000000   -0.197260000000
C     -1.492240000000    0.545190000000   -0.725270000000
O     -2.521360000000    1.046660000000   -0.263070000000
H      2.124930000000    2.238080000000   -0.474870000000
H      0.052540000000    2.480290000000    0.793840000000
H      3.208760000000    0.111670000000   -0.860600000000
H      2.393660000000   -1.990070000000    0.027370000000
H      1.289840000000   -1.139980000000    1.198810000000
H      0.435300000000   -0.983510000000   -1.805040000000
H      0.177790000000   -2.444740000000   -0.749700000000
H      0.194560000000    0.766560000000    1.444100000000
H     -0.994550000000    0.951930000000   -1.623190000000
H     -2.339360000000   -0.694600000000    1.233850000000
N     -1.475200000000   -1.157840000000    0.972440000000
H     -1.430920000000   -2.145840000000    1.174110000000

Cope TS Gas
20
ANGSTROM
C      0.051880000000   -1.363610000000   -0.589860000000
C      1.431360000000   -1.473750000000   -0.117230000000
C      2.257810000000   -0.414550000000    0.027200000000
C      1.795950000000    0.988260000000   -0.219190000000
C      0.549290000000    1.354090000000    0.675100000000
C     -0.725800000000    0.730890000000    0.216000000000
C     -1.254070000000   -0.587780000000    0.610460000000
O     -2.431660000000   -0.898070000000    0.232480000000
H      1.780220000000   -2.454450000000    0.203430000000
H     -0.500080000000   -2.299600000000   -0.630940000000
H      3.256350000000   -0.558700000000    0.432370000000
H      2.593190000000    1.702530000000    0.006200000000
H      1.510570000000    1.148260000000   -1.268770000000
H      0.758220000000    1.043900000000    1.701080000000
H      0.423010000000    2.442980000000    0.676800000000
H     -0.086320000000   -0.800010000000   -1.514370000000
H     -0.830510000000   -0.969020000000    1.557040000000
H     -2.362250000000    0.845730000000   -0.877830000000
N     -1.497130000000    1.352510000000   -0.662400000000
H     -1.247670000000    2.214100000000   -1.122890000000

Ambimodal TS Water5-1
35
ANGSTROM
O     -3.795050886245    1.191342495784    2.050396509351
H     -3.351754252601    1.468857692988    1.224219240104
H     -3.656351326183    1.892462948088    2.687113668046
```

```
O     -4.923885741249   -0.176404942390   -0.161265223726
H     -5.132840317148    0.111381776842    0.734555947602
H     -4.175044999585    0.383843478205   -0.423684777677
O     -5.456500292723   -1.761793711278   -2.478472855821
H     -6.349923934141   -1.689820186862   -2.816767403023
H     -5.370543329567   -1.090587369171   -1.786662039167
O     -4.400148499957   -2.964037014329   -0.130323249475
H     -4.606265616898   -3.051571140298   -1.068700295125
H     -4.530701081173   -2.019610481673    0.051139352906
O     -2.121680656816   -3.835060461686    1.054845758479
H     -2.391761855799   -4.480110883640    1.709575151768
H     -2.932885393410   -3.607134686830    0.555120413818
C      0.199682363764    1.688497718266    0.807951473646
C      1.283735335737    1.525628188426    0.001075903677
C      1.976596202542    0.281266509746   -0.248004589037
C      1.610883760898   -0.937192007561    0.278002847343
C     -0.302593124915   -1.412683294550   -0.800423597999
C     -1.255748509887   -0.605139334681   -0.185848085380
C     -1.604198415862    0.715122655283   -0.734584001350
O     -2.599627442544    1.387714810550   -0.402337291521
H      1.606441552631    2.374901762505   -0.604459290533
H     -0.300878312150    2.644816265684    0.888962275107
H      2.717639206428    0.299752873317   -1.047247987142
H      2.157281997485   -1.832428302829    0.007186198236
H      1.068448963312   -1.015372801243    1.211596636933
H      0.058351330208   -1.168439335761   -1.788647405364
H     -0.243163407431   -2.458056666378   -0.527412881783
H     -0.106706739135    0.952199450154    1.537740396468
H     -1.038228914997    1.031707030651   -1.633271634916
H     -2.592627904987   -0.445985059145    1.391354275713
N     -1.837255564234   -0.988931270916    1.001688693026
H     -1.807542816279   -1.968572309593    1.258617873840

Ambimodal TS Water5-2
35
ANGSTROM
O     -4.671734404309    0.380193206451    0.833934726518
H     -3.883300996650    0.789327453599    0.404140405833
H     -4.957449127633    0.984970740590    1.519142867191
O     -4.855616229377    1.005846651014   -2.023311465764
H     -5.325470938801    0.745548108369   -1.221451875916
H     -4.064833262206    1.470381380517   -1.712230191534
O     -3.602770428473   -1.345099008191   -2.498745005853
H     -3.919377395538   -1.775961917442   -3.293817483162
H     -4.068206317940   -0.481485947469   -2.448462233167
O     -4.598551163611   -2.333392697117   -0.133667250129
H     -4.198246966677   -2.092274976995   -0.990402392137
H     -4.753436065302   -1.493234651297    0.317397400941
O     -2.652906680482   -3.750736058186    1.104432724939
H     -3.045782705277   -4.435985396167    1.645586567511
H     -3.397312824174   -3.325817334123    0.623505573435
C      0.180081718940    1.676155087411    0.817944745306
C      1.299263157496    1.550581267634    0.052539727838
C      2.030233493765    0.326403374990   -0.179945280347
C      1.666187978066   -0.906754800107    0.316883664327
C     -0.177806404118   -1.400049131324   -0.832251902578
```

```
C      -1.182286510544   -0.628915655982   -0.254201196853
C      -1.542002751792    0.685856572273   -0.800371519192
O      -2.555826683146    1.345601849141   -0.495654894534
H       1.624424818805    2.414053426915   -0.531092705629
H      -0.350755426856    2.616577081902    0.888976586329
H       2.802559410308    0.368381217286   -0.948056330112
H       2.245486254863   -1.785931144044    0.061089577820
H       1.095699577899   -1.004151002787    1.231950775977
H       0.213448579543   -1.133888293683   -1.803036112939
H      -0.105369443636   -2.449395214169   -0.578033230529
H      -0.133836558458    0.924759396243    1.528717769228
H      -0.954502281229    1.019574378497   -1.678716190853
H      -2.642164239236   -0.555437812593    1.188362245876
N      -1.797491012047   -1.034194928777    0.915874557174
H      -1.804017205188   -2.022860778879    1.138437382378

Ambimodal TS Water5-3
35
ANGSTROM
O      -3.595928097954    1.495058807703    2.302645799601
H      -3.209798439540    1.548782495381    1.393516509920
H      -3.753831651934    2.399108685286    2.576441258078
O      -5.584778162103   -0.325306074440    2.108862649940
H      -6.167015950955   -0.266713163890    2.866600052816
H      -4.862441405863    0.320748246809    2.255769448954
O      -4.898663273454   -0.124288494844   -0.698108620327
H      -4.213472914535    0.550914304426   -0.633799124029
H      -5.423170267492   -0.046947370738    0.108658509896
O      -4.578203512212   -2.558177970204    0.669310832065
H      -4.562587148507   -1.997659541749   -0.120435849576
H      -4.961467705286   -1.985227004582    1.349247849664
O      -2.236744899552   -3.630214063880    1.505751911497
H      -2.444865046314   -4.417752231324    2.009305875253
H      -3.089089574824   -3.314295356272    1.136263261179
C       0.397176087333    1.742073689652    0.699136875253
C       1.369520910868    1.490244205747   -0.220226180969
C       1.990587006325    0.208622655626   -0.467428761661
C       1.655494577138   -0.960639351637    0.177784006322
C      -0.388312859263   -1.421620055309   -0.630007417846
C      -1.232971022505   -0.545704155012    0.045963109854
C      -1.604635141499    0.752064602794   -0.537165353591
O      -2.529263406875    1.486605506284   -0.135879628435
H       1.639020072434    2.287339147585   -0.916002706646
H      -0.062494230388    2.718874389680    0.776714852847
H       2.629254392585    0.149738321177   -1.348909203750
H       2.138082558980   -1.890654706981   -0.097369156706
H       1.229507145822   -0.960267974145    1.173032485178
H      -0.144056130007   -1.246858876049   -1.667502319414
H      -0.329868510834   -2.452299774726   -0.305801840559
H       0.164201347677    1.066750737706    1.510425516383
H      -1.151509409111    0.985470214586   -1.521128503241
H      -2.373930611160   -0.258641255868    1.744337592370
N      -1.668474205847   -0.838962991764    1.320868302781
H      -1.664707583429   -1.806726131313    1.617352007438

Ambimodal TS Water5-4
```

```
35
ANGSTROM
O     -3.941236748048    1.029019712691    1.849108434520
H     -3.438876728662    1.355894188327    1.072035952655
H     -4.005708103987    1.760241591349    2.463412319865
O     -5.043348555660    0.059865014895   -0.584798219853
H     -5.079966125738    0.086023485489    0.379737177721
H     -4.346791701908    0.691007283501   -0.815509270140
O     -4.157166199481   -2.159879704614   -1.821802555273
H     -4.869900078044   -2.357770806232   -2.431676886821
H     -4.464830081720   -1.381905753608   -1.306688723909
O     -3.198882138043   -4.408374813081   -0.673860798824
H     -2.661016403710   -4.767074640889   -1.381547960982
H     -3.559707762723   -3.562895950508   -1.015894065127
O     -1.746658014069   -3.811853857871    1.526318274065
H     -2.199787216537   -4.184734522849    2.283538331233
H     -2.268290357649   -4.083053609795    0.740811735806
C      0.168182180804    1.633684762631    0.842199018497
C      1.274892885617    1.478329235869    0.065198695337
C      1.959321084647    0.231859949911   -0.193760516053
C      1.559001015901   -0.993838341113    0.289553567119
C     -0.329564294437   -1.417802791025   -0.851155248160
C     -1.298805522543   -0.616696771921   -0.249613206773
C     -1.600215852683    0.726354961521   -0.778770391197
O     -2.573471589229    1.433886773515   -0.460243342906
H      1.626471752439    2.338769404274   -0.507671445041
H     -0.324148260671    2.593106677851    0.933999889006
H      2.723843182074    0.258952376601   -0.970373533574
H      2.101187348686   -1.889999325179    0.012953810450
H      0.990598477382   -1.088352607546    1.205831826217
H      0.064842850837   -1.156049502017   -1.821547083749
H     -0.279999947130   -2.467636066334   -0.595519802992
H     -0.168680199441    0.882469403157    1.542654315485
H     -1.000457633755    1.035489723385   -1.659423830621
H     -2.673746795905   -0.481334349506    1.303203696607
N     -1.940018386735   -1.031616254168    0.887499812580
H     -1.831249688847   -1.986973328077    1.210828103526

Ambimodal TS Water5-5
35
ANGSTROM
O     -3.256827474922    1.579020597795    2.245344205143
H     -3.006854923706    1.614576816725    1.300910449808
H     -3.468764721427    2.475306683684    2.506770969072
O     -5.038823898378   -0.560998306922    2.244628976052
H     -4.450261474694    0.166037952749    2.501069157120
H     -5.157871661944   -0.460743448624    1.283939042303
O     -5.020542701216    0.157127079917   -0.420390593223
H     -5.674482936714    0.767090617323   -0.764256723876
H     -4.166360947784    0.623482978231   -0.449314173436
O     -4.391277553717   -3.096751778604    2.850877839488
H     -5.121948915133   -3.585638642255    2.469803575540
H     -4.601120437374   -2.147567722751    2.699727361009
O     -1.899897663325   -3.639839180252    1.982377796259
H     -1.397420858005   -3.817800978142    2.778703106269
H     -2.827326869844   -3.513089651232    2.280014646825
```

```
C      0.191825080242    1.679738202582    0.812944637565
C      1.300569161173    1.526438574889    0.038533843812
C      2.001726601805    0.284794669443   -0.206309724840
C      1.623014218161   -0.938601124465    0.291762551289
C     -0.277698099691   -1.390335183485   -0.881071288471
C     -1.248185574933   -0.610349158688   -0.258157887952
C     -1.572910892245    0.737839595458   -0.756457891175
O     -2.562009279384    1.411385418628   -0.398398934315
H      1.643118535334    2.383302846226   -0.545307364979
H     -0.304816874110    2.637975222018    0.895087170401
H      2.769775418595    0.315477056867   -0.979399778319
H      2.173758047233   -1.832123343717    0.024588265585
H      1.038243377978   -1.031176701831    1.197769677957
H      0.118951754510   -1.101335209358   -1.842715410038
H     -0.207762540015   -2.443732069367   -0.646192140713
H     -0.138806195542    0.938168154293    1.526672347055
H     -1.006248720872    1.080528807796   -1.644451795386
H     -2.606571861045   -0.482428319178    1.295725371488
N     -1.879937621119   -1.036967840412    0.877255861822
H     -1.797826655847   -1.997331222731    1.199249746773

Ambimodal TS Water5-6
35
ANGSTROM
O     -5.003588470892   -2.427438800057    1.708935931814
H     -4.121708573472   -2.667994839834    2.017639833962
H     -4.924017196027   -1.554847787857    1.301032324766
O     -4.701196562419    0.055547217575    0.283713092584
H     -5.465313140727    0.633293652707    0.265852173651
H     -3.921494334163    0.616940005618    0.028678993190
O     -4.011489698505   -1.741148228313   -1.721736729277
H     -4.561440504865   -1.550947459782   -2.482636401415
H     -4.299310214108   -1.141420212156   -1.009435917580
O     -4.207241338334   -4.119242136102   -0.333748949187
H     -4.039959954308   -3.402595047804   -0.970105786664
H     -4.841308069513   -3.741692747500    0.299287884378
O     -2.345332448734   -3.772167458339    1.538132132838
H     -2.060035396962   -4.605240638959    1.913376054046
H     -2.945632826455   -4.000588695392    0.784938707566
C      0.156011276765    1.645026793953    0.845273205568
C      1.289724115368    1.554080024479    0.094204848290
C      2.034240766891    0.346460722837   -0.163553125172
C      1.663075685401   -0.907523989099    0.286025011253
C     -0.131219798911   -1.387584355412   -0.875024968649
C     -1.161748139912   -0.630925165008   -0.316194939628
C     -1.525964028451    0.693963153986   -0.846464529424
O     -2.557082454250    1.322779418701   -0.554082898697
H      1.614836274728    2.438805349829   -0.456646165453
H     -0.387854731554    2.576054800971    0.934297421561
H      2.816375638586    0.414466249113   -0.919425049810
H      2.259432040248   -1.771765624701    0.016975548535
H      1.098990359495   -1.031160163773    1.202463134427
H      0.266101745434   -1.113974177540   -1.841708258234
H     -0.064187834270   -2.441677205520   -0.638082066610
H     -0.158272260239    0.871257435293    1.531179089307
H     -0.908378212363    1.062589913299   -1.690595685102
```

```
H     -2.616548393308   -0.531188437308    1.129586590760
N     -1.814329482401   -1.054259743111    0.820789623800
H     -1.794463415374   -2.028563658279    1.092842445382

Ambimodal TS Water5-7
35
ANGSTROM
O     -3.914209186159    1.164888597667    2.000530886025
H     -3.395689648259    1.369323683639    1.191807265388
H     -3.769649738998    1.887850938808    2.611258337752
O     -5.124450858558    0.157279337375   -0.393339776738
H     -5.310407817257    0.508954804496    0.485455607948
H     -4.308128395647    0.600446338966   -0.664455085984
O     -4.483826326644   -1.564559605335    2.856514720328
H     -4.346974873106   -0.616373584626    2.744805234356
H     -4.797402811152   -1.888447805855    1.998214682986
O     -4.922606800872   -2.464775480867    0.205062137901
H     -5.709794717845   -2.898981643852   -0.125558660595
H     -4.959649331119   -1.541922556872   -0.129040718627
O     -2.711943384293   -3.528775715671    1.787067458443
H     -3.145764325633   -3.106690699421    2.542074181710
H     -3.371653486019   -3.494106077799    1.082964747838
C      0.226999552188    1.681853451449    0.808793003393
C      1.310437601402    1.539625114343   -0.004566171070
C      2.016521140425    0.306529508272   -0.265551522995
C      1.665650350140   -0.920156908334    0.253337100549
C     -0.247505265077   -1.402913841108   -0.820998340155
C     -1.209155592133   -0.615361887118   -0.192203797638
C     -1.567522110225    0.716169968901   -0.711754049037
O     -2.561412595249    1.375776155985   -0.348459169948
H      1.618528218378    2.397410550294   -0.605599033215
H     -0.283272662004    2.631934918067    0.901863712391
H      2.754065025992    0.338499493950   -1.067545412523
H      2.221076886740   -1.807128721734   -0.026724790888
H      1.130864072375   -1.010751289855    1.190376597719
H      0.107892304104   -1.138149826236   -1.805860386338
H     -0.169282784297   -2.452222195737   -0.568792197759
H     -0.060737514612    0.938391962264    1.539212283803
H     -1.024086598365    1.049294928074   -1.618174120914
H     -2.548721640918   -0.455424469723    1.362163639821
N     -1.797027025464   -1.009570319650    0.983793409161
H     -1.812442430802   -1.987255784967    1.264790963789

Ambimodal TS Water5-8
35
ANGSTROM
O     -4.163506847705    0.851496745828    1.880269332683
H     -3.625916530213    1.194222941144    1.136500043746
H     -5.032301006688    1.256134887457    1.788565301845
O     -6.912550227161    0.628408583691    0.836879485353
H     -6.556749336995   -0.270095047802    0.956552430611
H     -7.864322274785    0.563408351910    0.916695226800
O     -4.985947101781    0.177460843181   -1.263506046258
H     -5.726456071357    0.644684582446   -0.855376528590
H     -4.201677854223    0.713832003446   -1.081011278815
O     -5.082180830873   -1.511749578959    0.884535904333
```

```
H     -4.977831907665   -1.203132384481   -0.036432659990
H     -4.669139754824   -0.803704464275    1.420930095338
O     -3.178126085686   -3.441834459513    1.355054607762
H     -3.575596284128   -4.209738027968    1.766490592821
H     -3.914581596284   -2.829736978927    1.155323521242
C      0.237038666174    1.691407125718    0.792458426369
C      1.285748210088    1.530967417861   -0.063752434395
C      1.985456434216    0.295204362474   -0.320105611722
C      1.656063965972   -0.920379808473    0.245228510271
C     -0.275854645954   -1.436671464407   -0.706818888373
C     -1.214240052661   -0.623080657061   -0.072701159105
C     -1.601153332571    0.677695226360   -0.641384377451
O     -2.598803771023    1.341336373385   -0.301116444927
H      1.562075023796    2.372214891818   -0.702520910463
H     -0.279429904165    2.638503656058    0.877792989174
H      2.686646165798    0.304707091454   -1.154589199102
H      2.207521947359   -1.811237285784   -0.031798399419
H      1.177310102740   -0.981056189875    1.214808767859
H      0.024317626238   -1.219668336462   -1.721850667875
H     -0.198891642449   -2.478021974674   -0.421201015304
H     -0.012808886174    0.971384165473    1.558949853067
H     -1.074205931646    0.971964581914   -1.571922843961
H     -2.456503869305   -0.389726218027    1.552883284224
N     -1.739104055028   -0.974541922399    1.153477921428
H     -1.810480653865   -1.950884426654    1.403000482306

Ambimodal TS Water5-9
35
ANGSTROM
O     -4.403793134771   -1.028676890288    2.467478911592
H     -3.919640717713   -1.877970031184    2.450464832601
H     -4.242480773305   -0.632519461059    3.324101998386
O     -4.900660769549   -0.083347231750   -0.130127586649
H     -4.892860549826   -0.356771061698    0.800025667100
H     -4.207085323597    0.597186402046   -0.214441544269
O     -3.797255346182   -2.006556584110   -1.573526963856
H     -4.163303214989   -1.959595698836   -2.457285609465
H     -4.216675320726   -1.264959513508   -1.063485965941
O     -4.622514109616   -3.882194836762    0.133892132067
H     -4.237093793622   -3.295892752888   -0.557887558144
H     -5.468729971785   -3.487624916631    0.357453788178
O     -3.056999259414   -3.396631711780    2.214364562523
H     -3.101073379142   -4.142178791194    2.814221371493
H     -3.581909235295   -3.656502703386    1.415328059333
C      0.195581276404    1.619988690179    0.876197160334
C      1.310486055804    1.539508917569    0.094490731607
C      2.040801814222    0.336276423687   -0.203328950016
C      1.657067101094   -0.928181113415    0.224674151620
C     -0.130743730658   -1.370801427547   -0.867069236085
C     -1.160172268840   -0.618592902698   -0.292153834722
C     -1.551993712181    0.712214467494   -0.796585953317
O     -2.572641286017    1.317333664270   -0.450619608670
H      1.625389652275    2.434257593581   -0.445904415235
H     -0.346035353769    2.548943710377    0.992434080222
H      2.802442915235    0.409482323497   -0.978904694081
H      2.247691264947   -1.788736708301   -0.070201386662
```

```
H      1.140176267910   -1.059067564334    1.168318739936
H      0.234940183452   -1.093410193381   -1.845851228423
H     -0.078350675173   -2.432010784527   -0.655179644198
H     -0.103915057979    0.835184865535    1.555646587155
H     -0.957630607206    1.098865380890   -1.652632784793
H     -2.617436137664   -0.512268347224    1.141528785052
N     -1.811223565827   -1.041905729932    0.844979023780
H     -1.807469693836   -2.009027842859    1.126468609529

Ambimodal TS Water5-10
35
ANGSTROM
O     -3.405385238217    2.690133976731    1.743060938262
H     -3.031607813154    2.301806058953    0.928951510951
H     -4.164670389666    3.203402178151    1.460541918813
O     -4.860605625527   -0.230988981580    0.012094242695
H     -4.838747392217   -0.209915381547    0.979464860720
H     -4.144044117792    0.363477181844   -0.265315827330
O     -4.330786680817   -2.786577982827   -0.676958951146
H     -4.413937238766   -2.806347105339   -1.630730066457
H     -4.565971336192   -1.871443562096   -0.403086485292
O     -3.992669941023    0.208419816781    2.615335632182
H     -3.777545583719    1.149129657561    2.433438574133
H     -3.976110285310    0.098954418726    3.565966197256
O     -2.293101124954   -3.873478031913    0.781870974079
H     -2.714767141274   -4.477396144591    1.394559667571
H     -3.008212434598   -3.543265006835    0.202920679491
C      0.171628557932    1.648458025734    0.872406120447
C      1.277536716918    1.544097271937    0.085114372836
C      2.005248959746    0.327013914481   -0.197594260971
C      1.659695660221   -0.919200386268    0.275068314636
C     -0.219344688173   -1.407201271068   -0.855403583062
C     -1.201393460274   -0.633815808302   -0.239572982764
C     -1.553834590629    0.695502548330   -0.761110003390
O     -2.574775377989    1.338659975180   -0.449853786691
H      1.594409002019    2.424386244040   -0.478009972290
H     -0.353122108436    2.587958952814    0.987020327505
H      2.761476416437    0.393361506577   -0.980073047398
H      2.234450873772   -1.789965540708   -0.016518742452
H      1.102249202458   -1.043885880391    1.194672183151
H      0.158198317422   -1.128826333687   -1.828555071065
H     -0.146343993690   -2.459281454253   -0.612796476489
H     -0.132958513396    0.876213930635    1.564537605988
H     -0.961962372883    1.047425939880   -1.631175893896
H     -2.573534092862   -0.534886108671    1.314056308567
N     -1.802078066350   -1.057216801747    0.922578291375
H     -1.795029220799   -2.047636235947    1.134958856941

Ambimodal TS Water5-11
35
ANGSTROM
O     -4.977143985114   -0.131779072100   -0.107901669102
H     -4.129355343789    0.339354065921   -0.174775782380
H     -5.268850790556    0.009093840330    0.796025980049
O     -4.446892235314    0.947163124088   -2.668166169123
H     -5.049946369457    0.543442687464   -2.029503732038
```

```
H     -3.867533472762    1.515476922404   -2.146769725611
O     -2.797660468006   -1.186373040008   -3.073628717929
H     -3.037827206341   -1.634646860260   -3.886132358810
H     -3.386947008996   -0.404851586029   -3.014352389681
O     -3.627841590918   -2.579740782985   -0.752648437306
H     -3.248322299446   -2.245023655008   -1.579535893571
H     -4.281986632283   -1.914916128226   -0.496971803633
O     -3.599608452713    1.177234415599    2.106736944637
H     -3.353025362942    1.812060760406    2.779381790420
H     -3.225186674116    1.494561657243    1.265538510923
C      0.182863893595    1.665473654385    0.848407277311
C      1.287941247766    1.545397873635    0.062318063866
C      2.009699471461    0.321762999155   -0.203238534226
C      1.655149480831   -0.917042090341    0.282761112122
C     -0.216243907590   -1.394528678533   -0.847571233864
C     -1.198792950916   -0.623647421790   -0.235545924181
C     -1.556631441001    0.695777297563   -0.764647748927
O     -2.577256129300    1.344893552158   -0.447426828106
H      1.606660092017    2.416132393796   -0.513760701254
H     -0.337546723805    2.610314426367    0.941099502858
H      2.767570216767    0.374363370359   -0.984676376427
H      2.227049596980   -1.793405054321    0.002358204719
H      1.097955306313   -1.027466556769    1.204742596047
H      0.150363507836   -1.116128987270   -1.824040143961
H     -0.140950719052   -2.449094993850   -0.615876937430
H     -0.124644625091    0.906079113636    1.553709661799
H     -0.976686392036    1.041138624852   -1.640872324079
H     -2.573813537214   -0.500780954428    1.304615031062
N     -1.799538798729   -1.040501232778    0.941919358006
H     -1.891724815381   -2.031727776986    1.096386964839

Ambimodal TS Water5-12
35
ANGSTROM
O     -3.440910223120    2.646927778684    1.732598596488
H     -3.054969478460    2.270938071709    0.918372704909
H     -4.198242329392    3.161347536925    1.446949070178
O     -4.862448058869   -0.255523022291   -0.056782747124
H     -4.853488442922   -0.247458830975    0.910989211442
H     -4.144436566355    0.345110618466   -0.316600916465
O     -4.314267245552   -2.799715503334   -0.773106839006
H     -4.384698566236   -2.806871239111   -1.728079314751
H     -4.556382299588   -1.889185856554   -0.490078435821
O     -4.030691110214    0.151631664743    2.563458115469
H     -3.816591051613    1.095452302992    2.397133619937
H     -4.026338783226    0.029377973741    3.512663334635
O     -2.292160635927   -3.899204829664    0.697915686696
H     -2.719713845232   -4.512804892218    1.296747894767
H     -3.000730875541   -3.563662880318    0.114008063022
C      0.151086172651    1.629476648457    0.895633268532
C      1.267702036957    1.539590292015    0.121784558175
C      2.003511063328    0.328960502777   -0.167597205931
C      1.656251438557   -0.924717270165    0.283590019166
C     -0.205858686394   -1.403868052003   -0.878246817689
C     -1.198783792955   -0.642275975937   -0.265164891468
C     -1.549106840053    0.692749580281   -0.773380836687
```

```
O     -2.576411182726    1.328111130302   -0.467090913044
H      1.588810750938    2.428502758846   -0.425161146903
H     -0.378544980341    2.565524847570    1.015909197236
H      2.769795658971    0.408493822878   -0.938983898061
H      2.237978096581   -1.789470469290   -0.012035661817
H      1.087123682155   -1.063751137694    1.193934741957
H      0.183534165761   -1.111056346985   -1.842450985481
H     -0.132259942851   -2.458874762160   -0.648890983822
H     -0.159840391421    0.846896302120    1.573182475245
H     -0.947038277546    1.058448165843   -1.630676283534
H     -2.591742973060   -0.569103313384    1.271269773479
N     -1.813275175837   -1.083425839964    0.883082986548
H     -1.805445218461   -2.076596683593    1.082171499087

Ambimodal TS Water5-13
35
ANGSTROM
O     -3.968603850884    1.210051445803    1.967426014334
H     -3.459585302620    1.341472375054    1.130974911854
H     -4.397392840704    2.046314905465    2.151630462318
O     -4.759956529331   -0.448479449944   -1.082926306686
H     -5.047791859201   -0.736954503681   -0.199955265917
H     -4.173626745665    0.304521340697   -0.939385382097
O     -3.294597451741   -2.655273700138   -1.617767567629
H     -3.776169376828   -3.204855685677   -2.237404463900
H     -3.821571959702   -1.832211822511   -1.519398099721
O     -5.218360886935   -1.150382967853    1.568073738128
H     -4.782451507276   -0.316725320209    1.844151187182
H     -6.010058872604   -1.241072963736    2.099237920443
O     -3.275952264813   -3.290141539605    1.093047847421
H     -4.014682233894   -2.717025307799    1.335240184026
H     -3.216111611465   -3.227088204943    0.123769153118
C      0.270822827702    1.707004565006    0.779497574932
C      1.317403882654    1.530179791584   -0.073525808298
C      2.006007371341    0.284088753440   -0.322833665695
C      1.671015315435   -0.923328439666    0.249279503663
C     -0.279102639032   -1.431801126521   -0.722418570215
C     -1.204185688259   -0.609895311431   -0.086560929459
C     -1.585194093919    0.689195885914   -0.656460633528
O     -2.581431487983    1.357742894262   -0.311824416370
H      1.605283614555    2.365034530629   -0.715668114373
H     -0.233930910897    2.661081512407    0.859582248694
H      2.710070586592    0.285744212814   -1.155096751284
H      2.212603909255   -1.821198328963   -0.023364184129
H      1.175673071606   -0.979083787818    1.210442721689
H      0.026586371674   -1.214566247268   -1.735275776781
H     -0.205033819788   -2.472260701651   -0.435579973512
H      0.006134136106    0.989790500536    1.543657205402
H     -1.061690725228    0.986552192364   -1.586380291878
H     -2.440645048352   -0.351898782448    1.531079394011
N     -1.726610279974   -0.951901405197    1.148102599119
H     -1.878576836290   -1.934176391256    1.357855332027

Ambimodal TS Water5-14
35
ANGSTROM
```

```
O     -3.369442259563    1.956056413439    2.103407116700
H     -3.153139127818    1.839364033430    1.155735216318
H     -2.538203386699    2.113026740354    2.554196581435
O     -4.848778345102   -0.451464991022   -0.377818844938
H     -4.934998340468   -0.510062769486    0.592676621300
H     -4.238896669644    0.283047525539   -0.535624100786
O     -3.704042674240   -2.730470682466   -1.212501988260
H     -4.303218316443   -3.121099669492   -1.849174478884
H     -4.131952599679   -1.893533891788   -0.917063754257
O     -4.764345659782   -0.327238273616    2.362131821068
H     -4.311425425544    0.542908916363    2.392041524734
H     -5.592736628464   -0.215091297884    2.829404470325
O     -2.310870516921   -3.858855765547    0.859040594699
H     -3.003988116518   -4.212650559911    1.418626077891
H     -2.754735607520   -3.547003166113    0.047239573348
C      0.174063730602    1.673270071929    0.825996942105
C      1.281700268388    1.529690852520    0.047895049686
C      2.000387143140    0.297788644215   -0.180808859784
C      1.632824653127   -0.928160919814    0.332626135146
C     -0.219687439380   -1.425575933783   -0.786901091603
C     -1.218057761501   -0.649600766957   -0.203738958454
C     -1.596343509992    0.660260871518   -0.754771768896
O     -2.603942240020    1.309816679414   -0.419305129035
H      1.605970218168    2.385098440493   -0.547681488146
H     -0.344173207244    2.621696186620    0.890134753228
H      2.762492494844    0.326281997964   -0.959448430751
H      2.204097714340   -1.813243416364    0.079531802926
H      1.075390213011   -1.013098673708    1.257028666195
H      0.159512018536   -1.167112171026   -1.764955652275
H     -0.145603265476   -2.472580356536   -0.523922355613
H     -0.137381971948    0.928002743566    1.544805386663
H     -1.032484059707    0.992576689674   -1.650170234808
H     -2.663321350851   -0.571757136547    1.259794687720
N     -1.818951369119   -1.045156895932    0.976839883400
H     -1.810741204716   -2.036356465868    1.196697911133

Ambimodal TS Water5-15
35
ANGSTROM
O     -3.950281021563    1.054391351503    1.998309911736
H     -3.447144687880    1.208493813000    1.162170524482
H     -4.397358588830    1.878146326234    2.194984226657
O     -4.713172644481   -0.581615926545   -1.071433919819
H     -4.991496663965   -0.888204488920   -0.191522124029
H     -4.143836312672    0.182723482390   -0.919654250949
O     -3.199262482426   -2.746920714076   -1.639587445105
H     -3.670072967892   -3.299246912362   -2.265026951235
H     -3.744670330683   -1.937554130299   -1.528839480808
O     -5.146914890159   -1.328644497491    1.571367980096
H     -4.729349262663   -0.488906923055    1.857191198297
H     -5.934662214047   -1.444438452134    2.103526085972
O     -3.157403568817   -3.416740738137    1.062544320355
H     -3.908322736820   -2.864000304873    1.314414824195
H     -3.102117019151   -3.339598937792    0.094014677871
C      0.272749748335    1.664305174418    0.804724845332
C      1.320370549106    1.522798664683   -0.053594687788
```

```
C       2.036581729042    0.296245789040   -0.321328787579
C       1.731219375747   -0.925959900275    0.235766545865
C      -0.209822819678   -1.466320888319   -0.736890563766
C      -1.151478573969   -0.674317929345   -0.087553498790
C      -1.564021028288    0.623042998652   -0.639146463499
O      -2.574224562482    1.263934430540   -0.282825827815
H       1.586956964448    2.372409213532   -0.685493872096
H      -0.253503363123    2.605394208451    0.898861950244
H       2.737748235026    0.325011594896   -1.155538849192
H       2.292390384515   -1.807486435803   -0.050277093023
H       1.240372137856   -1.005701499077    1.197544296409
H       0.087550622256   -1.228835294720   -1.747674898778
H      -0.110978719677   -2.508479681028   -0.464029300636
H       0.027039253974    0.931224500178    1.560114970147
H      -1.050461638643    0.944536191955   -1.566575865072
H      -2.388339001762   -0.466082142512    1.536938408761
N      -1.661953719062   -1.044427782896    1.143999204251
H      -1.790663812167   -2.032606764440    1.341203597936

Ambimodal TS Water5-16
35
ANGSTROM
O      -3.753562024860    2.699259724312    1.638570652073
H      -3.222803552707    2.456464776727    0.865225910802
H      -4.659388188309    2.810592613148    1.293445646845
O      -4.923279465839    0.005576684161   -0.047685266194
H      -4.794793199610   -0.099761094393    0.914553159263
H      -4.124287035973    0.490810827987   -0.334840115455
O      -3.941867520410   -2.523698960225   -0.691670304282
H      -4.605609746884   -3.051663689044   -1.136794184563
H      -4.359245316235   -1.660101001899   -0.518346951705
O      -3.998727586873    0.208159728712    2.492381364890
H      -3.856704451421    1.173739009301    2.306795284770
H      -4.388239061691    0.154614027027    3.364704473762
O      -6.148096709824    2.434217847598    0.300179260592
H      -5.902121103337    1.507262300310    0.106403754266
H      -6.250699039561    2.869625887701   -0.546944489361
C       0.091721997625    1.593775765579    0.951381693069
C       1.227756472557    1.546239193538    0.198532279162
C       1.973557912273    0.356341657413   -0.130500867086
C       1.611076641599   -0.921994131298    0.249522011035
C      -0.190665948048   -1.350819994009   -0.939771797122
C      -1.208657939244   -0.614381858027   -0.331466051277
C      -1.547778485460    0.736740196810   -0.801895694769
O      -2.586365852965    1.356368054878   -0.510287202740
H       1.553908329503    2.461576525482   -0.299348049913
H      -0.447354259092    2.520560516158    1.098633318834
H       2.755026882426    0.470320119284   -0.881645459905
H       2.209595013816   -1.767708582833   -0.068851496605
H       1.045379051134   -1.098324351885    1.156408065617
H       0.203735054825   -1.029795105812   -1.893368540743
H      -0.125811835872   -2.416795536575   -0.761344671126
H      -0.224296675393    0.784061592700    1.593643055156
H      -0.914530301570    1.135339532162   -1.621291024062
H      -2.605674678033   -0.556972142832    1.201693306688
N      -1.850036305721   -1.088498317608    0.792115326024
```

```
H     -1.913657528732   -2.081349709889    0.939167319676

Ambimodal TS Water5-17
35
ANGSTROM
O     -3.361086967103    2.666112949714    1.771614874900
H     -3.006451785551    2.277202187293    0.949101068404
H     -4.120095628816    3.188333792237    1.505313709973
O     -4.873224507347   -0.223538733745    0.021020321558
H     -4.812718827014   -0.214543365853    0.987341898649
H     -4.163548681162    0.367939635510   -0.277497669943
O     -4.404271067954   -2.776950305206   -0.683587607634
H     -5.274374077918   -3.177763505606   -0.658056336740
H     -4.547857636396   -1.825612480972   -0.473523140962
O     -3.963857376355    0.178206947946    2.611191625762
H     -3.744801879381    1.122033728297    2.452292680356
H     -3.847043782966    0.016743446944    3.547315375348
O     -2.436149671502   -3.884625500883    0.841846102342
H     -3.167776972438   -3.507169718725    0.311186275582
H     -2.146532026701   -4.666369383142    0.370204402904
C      0.166917237721    1.633117367390    0.881822254743
C      1.274251030634    1.542416978388    0.093884696944
C      2.010132482719    0.333238820280   -0.194884314697
C      1.668763059837   -0.918654915746    0.270515819417
C     -0.197220829024   -1.413934995166   -0.850771266372
C     -1.192383856837   -0.653490795434   -0.236975254345
C     -1.549688462382    0.677003182719   -0.754845263337
O     -2.569690209082    1.317712021321   -0.438179666739
H      1.584039608070    2.428437632157   -0.464016826522
H     -0.365250631515    2.567647111136    1.001833793336
H      2.765036025638    0.406891984344   -0.977804166438
H      2.252049239585   -1.783133836736   -0.023858073600
H      1.120192010448   -1.049090034410    1.194889826031
H      0.173621197984   -1.131605001993   -1.825180475518
H     -0.112889050067   -2.466711628471   -0.615416301599
H     -0.129905198476    0.855459317797    1.571207974701
H     -0.959709297982    1.032399576126   -1.624574660367
H     -2.569179019732   -0.555977828968    1.308948252498
N     -1.797433750852   -1.080881436281    0.919872822635
H     -1.797465130314   -2.067791839979    1.143223598117

Ambimodal TS Water5-18
35
ANGSTROM
O     -3.251984665892    2.579934946718    2.079373188841
H     -2.918317075051    2.414349217202    1.183974221502
H     -4.152595320732    2.238267380009    2.072671725406
O     -4.785737681412    0.389043023953    0.930562155273
H     -4.020853844568    0.704258142762    0.405377007083
H     -5.408675188122    0.014649010679    0.307312614697
O     -4.038816937146   -1.189342228825    3.172884954763
H     -4.480567641097   -0.723355661194    2.447656645607
H     -3.300691839445   -0.608540105184    3.433670331929
O     -2.022747564198    0.655071187765    3.505623612796
H     -2.414240843715    1.417815068946    3.022209046461
H     -1.783184590766    0.983009190663    4.372610584129
```

```
O     -2.614474569687   -3.358047849034    2.343623571300
H     -2.490493847973   -3.948218485888    3.087472383475
H     -3.215698453446   -2.650472134522    2.660364868969
C      0.168601669495    1.574155144135    0.949796762762
C      1.290893335828    1.489730988834    0.181099526503
C      2.002132576666    0.277492163219   -0.150443729608
C      1.615142140972   -0.985471345524    0.241998124840
C     -0.236648575185   -1.356193163061   -0.969478972622
C     -1.226806053532   -0.617993818455   -0.322964807869
C     -1.552701108220    0.762443956469   -0.725386408985
O     -2.561668489416    1.385501980121   -0.340819272529
H      1.634206246536    2.392152815034   -0.329233198032
H     -0.334098724792    2.521037040714    1.100774050118
H      2.778925648833    0.369092096050   -0.909700790664
H      2.179386702120   -1.851772017499   -0.082152274278
H      1.035820147551   -1.146065161168    1.142632836918
H      0.165450609457   -1.011868195469   -1.911024034073
H     -0.177520921763   -2.424676749902   -0.809010456960
H     -0.159377597822    0.781442596080    1.607876174946
H     -0.963829698337    1.183850889441   -1.563675006874
H     -2.520674830609   -0.515833070004    1.279835594483
N     -1.929616709971   -1.120337744033    0.735214201490
H     -1.816349994933   -2.058163967226    1.089846328825

Ambimodal TS Water5-19
35
ANGSTROM
O     -3.690583454910    1.322260869092    2.126564750082
H     -3.373202794081    1.557861332890    1.232617324300
H     -4.307893234947    0.578079208522    2.006135751377
O     -4.960134281416   -1.076812007282    1.604576101348
H     -4.367051448237   -1.819828595873    1.835732709008
H     -5.100276148398   -1.129697710566    0.658008331581
O     -3.064221070217   -1.457312782830    4.544384081592
H     -3.923761645283   -1.028666265481    4.537978901277
H     -2.421860831001   -0.762086169102    4.279505522218
O     -1.627220639613    0.617614750117    3.630108367228
H     -2.374450247693    0.966957498938    3.082175918195
H     -1.374657783593    1.327819569367    4.220653139262
O     -3.235044370543   -3.051321177718    2.429090106800
H     -3.597042535739   -3.893464473307    2.707735010639
H     -3.074779121289   -2.536421854368    3.258370139874
C      0.278065910244    1.603448315960    0.890149101925
C      1.373395692271    1.478157585958    0.085146933394
C      2.037634107948    0.244898307054   -0.240076080967
C      1.595784813056   -1.002937273922    0.177946012511
C     -0.237690339304   -1.320636354526   -0.907049830249
C     -1.213979784442   -0.534497768553   -0.283778531897
C     -1.535444191552    0.837397526664   -0.730233108781
O     -2.498800529348    1.502032097887   -0.331943879426
H      1.718446023567    2.361681397046   -0.455777685482
H     -0.210502247114    2.559584136336    1.025041125012
H      2.789324412199    0.289667195173   -1.027563013914
H      2.131816131000   -1.890648862297   -0.139539228134
H      1.084462603800   -1.119281812239    1.126560354716
H      0.129874553570   -1.030390048821   -1.881345274327
```

```
H     -0.232487319617   -2.389223715736   -0.727314119581
H     -0.036578481388    0.836065457986    1.583327808544
H     -0.956140420768    1.200942706783   -1.606269326102
H     -2.498475391514   -0.356468320997    1.316885855235
N     -1.927811842453   -0.998060184658    0.791149461660
H     -1.789310357751   -1.909111854855    1.192665486117

Ambimodal TS Water5-20
35
ANGSTROM
O     -3.449533600540    1.826094654818    2.136085520937
H     -3.069698700564    1.700610438508    1.242075366275
H     -4.219166422113    2.385221668901    2.008356552595
O     -5.120712274463    0.325198178564   -0.189278254957
H     -4.250495515857    0.736830935116   -0.334936025854
H     -5.492116148213    0.147504940282   -1.053481573518
O     -4.610914485038   -0.726323125750    2.332769384204
H     -5.012566482672   -0.539935006808    1.470399791808
H     -4.199183503407    0.110362066656    2.597204869840
O     -1.474730526435    3.547240087515    3.134749625934
H     -1.284043718521    3.346210835767    4.051696818834
H     -2.157191305149    2.915529305726    2.854087777262
O     -3.215916403488   -3.117753446446    2.276221090582
H     -3.575147282468   -3.732559760722    2.916653706957
H     -3.759421947954   -2.310772803306    2.346306290973
C      0.156858190286    1.609756923426    0.862917744272
C      1.272225855542    1.526251092737    0.082182721201
C      2.005110489079    0.318242988308   -0.214661896769
C      1.659339086944   -0.936118662231    0.239498696551
C     -0.211818238929   -1.387385653083   -0.910587034021
C     -1.207058205952   -0.633126093344   -0.293342016239
C     -1.547119558689    0.721844278811   -0.757468620452
O     -2.563186524005    1.355210898098   -0.398944572737
H      1.588424326550    2.418018622362   -0.462132222609
H     -0.360032032296    2.549290888283    1.007774100286
H      2.766937512274    0.395489452389   -0.990856486471
H      2.237142589587   -1.801995835884   -0.061501123143
H      1.101730965089   -1.073164870890    1.157464590800
H      0.180248521713   -1.080334034403   -1.868771725200
H     -0.134802968448   -2.446448552275   -0.702077376187
H     -0.135206451305    0.825590827433    1.548657120706
H     -0.980123006887    1.099371773343   -1.630387184591
H     -2.638892489541   -0.544712014312    1.187912405766
N     -1.856940416500   -1.072811486547    0.833731925500
H     -1.843672603907   -2.036980257271    1.126407194545

Ambimodal TS Water5-21
35
ANGSTROM
O     -3.445454951024    1.787371013536    2.101212486756
H     -3.258552781362    1.547679351761    1.171289014193
H     -3.296961458590    2.741529255157    2.111850962827
O     -2.572121660079    4.063458912376    0.637886868277
H     -3.082839347557    4.691880600589    0.126020611726
H     -2.504784446557    3.261167965398    0.092147366854
O     -2.392000904332   -1.922209471792    4.308794427313
```

```
H     -3.347331130886   -1.843322800930    4.294834671121
H     -2.041625174623   -1.038287578985    4.068428605330
O     -1.596339203427    0.561558550647    3.583892055413
H     -2.303900699456    1.015020954131    3.065217651831
H     -1.353402508064    1.161292365532    4.290024358902
O     -1.402079510351   -3.373896003381    2.248459958576
H     -0.534554859914   -3.692388824356    2.501043456572
H     -1.777832356453   -2.954060089159    3.050817323393
C      0.200156288703    1.593979931782    0.873264234409
C      1.331960385754    1.417958196176    0.135470540458
C      1.988380252068    0.155967580648   -0.113103954066
C      1.519977030536   -1.062797234312    0.328466430530
C     -0.306623287718   -1.383850195210   -0.923000928713
C     -1.291222417928   -0.588525847796   -0.337356683237
C     -1.539803452528    0.788874244608   -0.793942796012
O     -2.497351616074    1.500722337614   -0.435771538950
H      1.730177411960    2.274239060872   -0.412981518459
H     -0.263042843884    2.568359204318    0.964488088246
H      2.789483775985    0.167065989438   -0.852381107121
H      2.045188779572   -1.973576018800    0.066199779982
H      0.907068646274   -1.144510440940    1.217251516804
H      0.141623961678   -1.087646800394   -1.859952385367
H     -0.297573598868   -2.447002614688   -0.723276852689
H     -0.173163027082    0.849009713715    1.562436571382
H     -0.924226544209    1.131876436758   -1.650987433885
H     -2.665184343287   -0.436829121004    1.197785006999
N     -2.049288845636   -1.057407588230    0.702781341970
H     -1.849291010754   -1.952773376657    1.134905836565

Ambimodal TS Water5-22
35
ANGSTROM
O     -3.803173558693    2.131128433412    1.967720136033
H     -3.454391193722    2.979005528674    2.244808865406
H     -5.392357967028    0.613483212259    0.744482204010
O     -4.969195133657   -0.043727511976    0.186267601989
H     -3.343133333248    1.903875377480    1.134898113159
H     -4.174481275935    0.387531763656   -0.160790199900
O     -4.693960917711   -2.216736710648    1.927795805279
H     -4.895643876371   -1.664298747644    1.155780069890
H     -4.358946292032   -1.591631172522    2.593103740295
O     -3.743336576012   -0.125403402124    3.504103063089
H     -3.791173968176    0.702024064661    2.987028606657
H     -4.223945953777    0.034173819114    4.316978425936
O     -2.482018071302   -3.710152333779    1.578906909650
H     -2.400429065323   -4.253923148928    2.363212975331
H     -3.367487621058   -3.284568947267    1.640084999516
C      0.237633597627    1.669180439735    0.843783688443
C      1.323837690876    1.533894063359    0.034037316097
C      2.027485491597    0.302350213882   -0.242874006387
C      1.670476192996   -0.931004511290    0.254739440457
C     -0.235454304459   -1.397955340959   -0.838387126107
C     -1.198935699271   -0.621671362094   -0.198922134595
C     -1.562747711693    0.713819076315   -0.700800851070
O     -2.558704955759    1.363505808326   -0.327905772844
H      1.639874157607    2.399761830263   -0.551338782808
```

```
H     -0.266804623927    2.621478354181    0.946638337904
H      2.769442289245    0.345165748017   -1.040255164138
H      2.226097290034   -1.814762802680   -0.034850287124
H      1.128396198913   -1.033760706011    1.186281445624
H      0.121398593449   -1.117849588774   -1.818652141527
H     -0.159804508691   -2.451050287639   -0.602170653871
H     -0.063270560233    0.916453238783    1.558837358778
H     -1.015892748053    1.066712408776   -1.598009856386
H     -2.540668815127   -0.497417239528    1.358141053253
N     -1.776645731523   -1.032014166863    0.977180326738
H     -1.781335170837   -2.016097478488    1.226688958830

Ambimodal TS Water5-23
35
ANGSTROM
O     -2.762465317070    3.882439618537    1.060747706793
H     -2.628648663591    3.186131469852    0.399652669698
H     -3.727600552015    4.006622697964    1.111144974581
O     -5.054952630261    1.145486879209    0.317696473567
H     -4.125755699386    1.277668242510    0.022129104495
H     -5.432215021843    0.481230613824   -0.259752949285
O     -3.507700125708    0.181275594165    2.464505798457
H     -4.271050060028    0.541201337128    1.993316111963
H     -3.046060923373    0.956753642784    2.843464427171
O     -2.437346368344    2.523840550040    3.344286277733
H     -2.478543511043    3.076492592708    2.527963820264
H     -2.974777317042    2.977090521010    3.995157426313
O     -5.517657593944    3.749272840274    1.080712496743
H     -5.485575979725    2.797026376575    0.881459718158
H     -6.016237316525    4.161152537540    0.373836322780
C      0.033091650367    1.428574631931    1.127350242635
C      1.218849137647    1.386058949548    0.453083393222
C      1.916228810132    0.192165777193    0.045730666661
C      1.447128567315   -1.090720838210    0.254239690492
C     -0.286278569041   -1.233411005244   -1.107513591692
C     -1.312671679670   -0.509418092059   -0.492510792643
C     -1.519902815690    0.913331504135   -0.799582516230
O     -2.526147162135    1.582468802196   -0.501179181577
H      1.631206288061    2.325600884190    0.080355307031
H     -0.454179235471    2.369159786314    1.343561911481
H      2.756872998267    0.330013288581   -0.634255064893
H      2.009758463715   -1.939495623936   -0.117184356158
H      0.807555050076   -1.322209430281    1.097589877393
H      0.209133888467   -0.828046696649   -1.978114672703
H     -0.294683870314   -2.315721535889   -1.059535093220
H     -0.370483680920    0.584478347186    1.668927794491
H     -0.799159569068    1.354347645018   -1.518714307552
H     -2.675031852195   -0.543322392563    1.106245799836
N     -2.168703565573   -1.101755960735    0.407976679378
H     -1.980969463764   -2.046705162920    0.700310743466

Ambimodal TS Water5-24
35
ANGSTROM
O     -4.549798753856    0.590806903782    0.992082152452
H     -3.812879516760    0.916247845091    0.404554438600
```

```
H     -5.147143384875    1.332540465165    1.094270444224
O     -5.336649587242   -1.765167637777    0.039745502340
H     -5.030562057531   -0.890587858028    0.380227550615
H     -6.194218351554   -1.617462167343   -0.361205133709
O     -3.468924079749   -3.429337687803   -1.218865172722
H     -4.002343122943   -4.191769837938   -1.449635820088
H     -4.075276994123   -2.778741798641   -0.827037005904
O     -4.649979840588   -3.355582308430    2.227065730153
H     -4.951625639482   -2.855301250095    1.450240139450
H     -4.611371638793   -2.730444514199    2.952639531691
O     -2.104857065809   -3.815504993824    1.177145073621
H     -2.926985006787   -3.863868710336    1.691929429494
H     -2.395336652965   -3.826718059273    0.249731121693
C      0.141270239544    1.677185514855    0.817307451694
C      1.266237748886    1.552709365127    0.060785782802
C      1.998902369964    0.329297806399   -0.168505326400
C      1.626971302584   -0.905971201547    0.320990591293
C     -0.196813749914   -1.407402088068   -0.830773677147
C     -1.212784887135   -0.631785551784   -0.272629963826
C     -1.567486758779    0.679164722847   -0.841186649458
O     -2.582037775440    1.343804974322   -0.569983882783
H      1.596945178523    2.418056557015   -0.517262182315
H     -0.392186471433    2.616194869492    0.884649372444
H      2.776387505294    0.372061827988   -0.931401731660
H      2.210085324328   -1.784050913106    0.069884037088
H      1.055182019992   -1.004278899895    1.235135365992
H      0.199464959436   -1.153586867633   -1.803175190576
H     -0.125459341000   -2.453113310661   -0.562594340206
H     -0.180706202253    0.922530345305    1.520620573867
H     -0.951848974798    1.004252996975   -1.705343901014
H     -2.647878466261   -0.524856177591    1.203468166839
N     -1.844338227768   -1.038127747721    0.882257449639
H     -1.807394668232   -2.024825625222    1.140670115979

Ambimodal TS Water5-25
35
ANGSTROM
O     -1.640173959382    1.416652767138    4.048448610510
H     -2.396701869711    1.426757982776    3.436530642621
H     -1.145817020737    2.221968601359    3.889074572300
O     -3.664525671124    1.435928185129    2.143311618789
H     -4.314268450145    2.139862773072    2.159867532102
H     -3.212253023145    1.501207886321    1.254911952607
O     -4.502902084233   -1.142753536296    2.697034942371
H     -4.732483621448   -1.438886079699    1.813944150265
H     -4.259604447316   -0.203223126818    2.613739271486
O     -0.185819831743   -0.826855365249    3.192978936023
H     -0.592091353056   -0.078863090900    3.658254472185
H     -0.792203869287   -1.567815309258    3.340877426242
O     -2.326435482469   -2.648362228797    3.414425830442
H     -2.457698017850   -3.029779763304    4.283315600418
H     -3.128335488880   -2.120570714464    3.225873245254
C      0.407367023435    1.575562588648    0.847839945334
C      1.447084470377    1.412351275366   -0.021062972641
C      2.049156381713    0.148245615407   -0.357008960190
C      1.600573976059   -1.068405778368    0.124441455663
```

```
C     -0.323295601691   -1.311733126961   -0.914705422466
C     -1.235796378684   -0.511750291574   -0.227055941016
C     -1.512184461214    0.878138054423   -0.621238496076
O     -2.413554594874    1.599216846310   -0.154122506759
H      1.784971652362    2.274332857717   -0.600725940110
H     -0.040716672537    2.548700000759    1.006676497516
H      2.761808417874    0.154195266522   -1.182636498943
H      2.078670875257   -1.984175033180   -0.205251695432
H      1.113661754260   -1.144259397115    1.089897009872
H      0.025138937461   -1.011267668609   -1.892788373131
H     -0.321258463279   -2.380474143595   -0.741817787158
H      0.135449061881    0.815789486867    1.567236392535
H     -0.987433439661    1.229399673327   -1.533163343725
H     -2.319284025925   -0.344720596273    1.510540169363
N     -1.944187952393   -1.008665730923    0.852051028302
H     -1.640953199796   -1.867265969475    1.285364584111

Ambimodal TS Water5-26
35
ANGSTROM
O     -4.687531523493    0.372769282119    1.329204890171
H     -4.016113291985    0.713444403938    0.681784864605
H     -5.391761177730    1.021771030183    1.339509447652
O     -3.771386349923   -2.275631343074   -1.698363663239
H     -4.478390604288   -2.280400296351   -2.345513891318
H     -4.199585644153   -2.240025879684   -0.823108155612
O     -2.316434403569   -4.544691933844   -1.245350287495
H     -2.806590416475   -3.736591349358   -1.493912317311
H     -1.498556417183   -4.528374733456   -1.743489474403
O     -5.032136777172   -2.220576852211    0.750983466000
H     -4.904783802459   -1.281627440213    1.018085462816
H     -5.894289490474   -2.483843916368    1.075312212701
O     -2.672811065841   -3.776370653449    1.416344172071
H     -3.558227457667   -3.405310346379    1.304851202527
H     -2.480404252064   -4.226575689090    0.577584969922
C      0.013152553585    1.625900646960    0.624660155532
C      1.041418527563    1.478314774612   -0.257065237851
C      1.768901648894    0.258420703108   -0.505795365868
C      1.478426753777   -0.955008855946    0.092038378648
C     -0.434513075218   -1.551923115561   -0.774257910283
C     -1.402524739513   -0.756844027405   -0.155811333311
C     -1.856992943581    0.507736558358   -0.758460786740
O     -2.850154103973    1.161418648768   -0.404293717454
H      1.277815224232    2.314888481606   -0.917763124542
H     -0.532638851652    2.556741496292    0.703241052910
H      2.446148781518    0.266322629328   -1.359754231184
H      2.053261057985   -1.834455578678   -0.175585412367
H      1.041134284482   -1.002034389285    1.081856507046
H     -0.173630407233   -1.367196236063   -1.806537445968
H     -0.314878497413   -2.576915249137   -0.446495039886
H     -0.199544100505    0.910248291939    1.405789206268
H     -1.354303881360    0.793145231689   -1.706022646287
H     -2.640889703387   -0.539451834650    1.468339106616
N     -1.890101687877   -1.092943808226    1.088209758584
H     -1.900586044124   -2.070116386040    1.374327620494

```

```
Ambimodal TS Water5-27
35
ANGSTROM
O     -4.332789003654    2.618411461767   -2.075403887991
H     -3.651033854820    2.294801382345   -1.456245446738
H     -3.869027297680    2.950432522820   -2.845076219705
O     -5.481453660106    0.114084505704   -2.083166487020
H     -5.522931900270    0.015782125454   -1.118608345408
H     -5.226168882432    1.043066661337   -2.225801321962
O     -3.320057218016   -1.426590950806   -2.370348382617
H     -3.488196615831   -2.051680740811   -3.077097967747
H     -4.117595096467   -0.842569507864   -2.340079194611
O     -4.829481199876    0.267210239871    0.637473908107
H     -4.019597356380    0.688222251179    0.277303591141
H     -5.329393252298    0.968768392536    1.057251785723
O     -4.023484782981   -2.586512347549    0.084153833363
H     -4.568042078557   -1.866857936029    0.418113268250
H     -3.716668286347   -2.283186361978   -0.787589228392
C      0.192139830598    1.655586657908    0.841290643483
C      1.301069247103    1.540455270396    0.057916359499
C      2.023166771292    0.319063347909   -0.204584918992
C      1.654949608324   -0.922342134912    0.275107929605
C     -0.187221741921   -1.383110645255   -0.844197886964
C     -1.187735584038   -0.618515585770   -0.249368508792
C     -1.556553721640    0.701579596974   -0.765508404592
O     -2.564667223699    1.348502369950   -0.418071188085
H      1.621279625858    2.412873566892   -0.515075869539
H     -0.335706014817    2.595673151328    0.935393788674
H      2.785479507364    0.368585790615   -0.981982985357
H      2.230611919757   -1.798272066636   -0.000453908904
H      1.106955430616   -1.028365549997    1.203296508286
H      0.185548755286   -1.106275120700   -1.819449464837
H     -0.117070363781   -2.438948389989   -0.615609379529
H     -0.112399895893    0.895572382420    1.546740730689
H     -0.986220207479    1.056573296017   -1.646868726170
H     -2.630422636204   -0.520740850603    1.196394680376
N     -1.802196291279   -1.031209748135    0.924581140827
H     -1.900399626180   -2.022961509479    1.084830828752

Ambimodal TS Water5-28
35
ANGSTROM
O     -4.730627180770    0.208528498977    0.331500820483
H     -3.909239727028    0.688077063469    0.037273506655
H     -5.406833158308    0.880176669945    0.430138983029
O     -4.458095961481   -4.001332645077   -0.171401034321
H     -4.453720446179   -3.203397234032   -0.740115631387
H     -4.542958910633   -4.755188835880   -0.755804889253
O     -2.336059421725   -3.704750197114    1.638061987860
H     -2.890283102709   -3.310599972723    2.322748298422
H     -2.967701368364   -3.979031628591    0.953674046959
O     -4.630129093658   -1.713605110983   -1.606318983438
H     -4.653858535469   -1.025833084900   -0.911056885094
H     -5.365687321576   -1.532622989450   -2.193143585054
O     -4.711006640689   -2.118362290695    2.070506023315
H     -4.775117268378   -1.295253392821    1.569955648004
```

```
H     -4.901473734705   -2.822406086870    1.440468799607
C      0.112651677487    1.601435283190    0.909430204407
C      1.268498911151    1.549586345097    0.190119415379
C      2.028124183840    0.357607428240   -0.101588828895
C      1.656241649178   -0.916082534402    0.280234019501
C     -0.116341895717   -1.357189490884   -0.970072256975
C     -1.163595872136   -0.630714414012   -0.401074272327
C     -1.508115494182    0.722660110450   -0.875682786957
O     -2.546043824456    1.341608533552   -0.585851138409
H      1.605841298742    2.460445808658   -0.308645177822
H     -0.436759965915    2.525941611044    1.028076745060
H      2.831979664842    0.466321073891   -0.829658276252
H      2.262503988127   -1.765180273043   -0.012858728338
H      1.057643144129   -1.088491393380    1.165994599411
H      0.313669784726   -1.033187720238   -1.906538201202
H     -0.043103977950   -2.419134511511   -0.774579864454
H     -0.219098937330    0.793949568538    1.546608782185
H     -0.864782522364    1.129202320011   -1.682421321608
H     -2.630933204387   -0.591313877822    1.046334957279
N     -1.859357081418   -1.116931258066    0.673581183877
H     -1.805805379476   -2.091305755024    0.963159309698

Ambimodal TS Water5-29
35
ANGSTROM
O     -3.172695709134    1.866090421262    2.307721592068
H     -2.894535925665    1.778932481431    1.373416248848
H     -3.072832746670    2.790527862596    2.537202678702
O     -4.843971065430    0.098979446112    0.834481097930
H     -4.770035616085    0.620768770732    1.642415529676
H     -4.254297671745    0.537336506804    0.199357079222
O     -5.048478561301   -2.616886012430    1.153526663778
H     -5.655084550603   -2.913204934056    0.473720872845
H     -4.999932645313   -1.645694780886    1.066117367851
O     -1.135983184000    0.293549790628    3.497085468734
H     -1.888575428092    0.855700526197    3.257123080402
H     -1.281437816217   -0.538698832279    3.036585238642
O     -2.590742087715   -3.746487507452    1.538301396739
H     -2.605262228649   -4.168990546219    2.398289175267
H     -3.502606192972   -3.433377294921    1.377395608951
C      0.182785285792    1.761167627387    0.719221933538
C      1.293287049594    1.548916469502   -0.044019561561
C      2.005452109390    0.301257398806   -0.161223820503
C      1.606425092007   -0.879645739723    0.433867339852
C     -0.201652598906   -1.397654228385   -0.725343608415
C     -1.227431837443   -0.634598723882   -0.172184964696
C     -1.599047062752    0.687528273679   -0.694273674580
O     -2.597174939579    1.337610798663   -0.316703062844
H      1.619942049303    2.345123838917   -0.716907625922
H     -0.326136582859    2.717083027799    0.699147478219
H      2.785143066589    0.260772598940   -0.922541187822
H      2.173084744609   -1.787925062464    0.264588606543
H      1.030501496802   -0.881689823322    1.350795824000
H      0.204070985289   -1.133852914466   -1.691238431007
H     -0.124703148740   -2.445974792142   -0.466551831141
H     -0.107819998382    1.106706499857    1.529516632625
```

```
H     -1.076393049031    1.017399671966   -1.613540013124
H     -2.748508400293   -0.587399381613    1.197082667112
N     -1.881187688336   -1.056677623833    0.976021566057
H     -1.891628707731   -2.050869174847    1.187411236434

Ambimodal TS Water5-30
35
ANGSTROM
O     -2.611650623376    3.990401920104    0.663231894757
H     -2.537830404259    3.190549843973    0.115282701903
H     -3.186953614265    4.588686538329    0.184866511711
O     -3.323966541108    1.664917734519    2.162559899114
H     -3.200835446554    2.622297053928    2.182286725637
H     -3.157767987324    1.440471321343    1.225581617110
O     -1.598950708443    0.591879007221    3.902223509351
H     -2.082151422889    0.589705016950    4.729273282787
H     -2.213422385314    0.977211830611    3.234941493498
O      0.231840467818   -1.380235834023    3.632238821341
H     -0.456457518272   -0.690326888648    3.723129233959
H      1.072706635111   -0.931350411163    3.729963952059
O     -0.782138182097   -3.363063120243    2.048247047463
H     -0.327090587790   -2.723041568080    2.626023208230
H     -1.196371466986   -3.999284621615    2.632567320509
C      0.206644055772    1.529574241572    0.915668331027
C      1.327064390613    1.434524517978    0.147229838057
C      2.022124539941    0.212170643097   -0.184123133597
C      1.616177575773   -1.046189218735    0.206412518945
C     -0.235913785061   -1.386387380777   -0.986152231540
C     -1.230659906913   -0.627522169624   -0.370637461418
C     -1.523416358208    0.748316581561   -0.798421422740
O     -2.492194463612    1.431105904139   -0.416993169181
H      1.681217976818    2.333191674344   -0.361813875157
H     -0.291934992452    2.477738364065    1.069322572052
H      2.799483350616    0.293942894363   -0.944315976519
H      2.167138518977   -1.919243710116   -0.122090352711
H      1.038752500404   -1.207077063518    1.107257796467
H      0.180775449969   -1.067433004072   -1.930379155277
H     -0.189348019913   -2.449608789184   -0.792419623411
H     -0.128818561682    0.733577061299    1.565179197939
H     -0.919914268742    1.125831885768   -1.650134495671
H     -2.563537580856   -0.542207627440    1.209887039929
N     -1.965693202923   -1.143705251158    0.669668362560
H     -1.690153091291   -2.022741521568    1.092650160281

Ambimodal TS Water5-31
35
ANGSTROM
O     -4.926106806944   -1.898928521094    2.477628130700
H     -4.187993525604   -2.470991943315    2.201031480093
H     -4.868161989504   -1.110082655862    1.918621767689
O     -4.750585583820    0.120035490184    0.482109998219
H     -5.503776491572    0.693760505458    0.335537246674
H     -3.963006442311    0.617685647024    0.148346318898
O     -4.712023306678   -2.420484870412   -0.734195845390
H     -5.537047404022   -2.795587970230   -0.381939543223
H     -4.713922129777   -1.493522453478   -0.443310435158
```

```
O     -6.742249324062   -3.198461320637    0.964738385824
H     -6.177319211447   -2.775038549762    1.650525429568
H     -7.649248424332   -3.011708351332    1.206253955514
O     -3.149097585967   -3.541280385958    1.105118691925
H     -3.412672128152   -4.458596895600    1.188850041215
H     -3.643538619405   -3.187877164942    0.322012180666
C      0.178857144988    1.644654006984    0.838257041063
C      1.287262912016    1.542717061781    0.049963186912
C      2.019026429764    0.331425877380   -0.217338514710
C      1.653773462312   -0.918164096190    0.257355615844
C     -0.160376323252   -1.399243999509   -0.822422908924
C     -1.174210014658   -0.627166692734   -0.249971732180
C     -1.558007039578    0.686349749618   -0.791888739861
O     -2.584844784354    1.309259721188   -0.478915516143
H      1.594393263701    2.419990725313   -0.522578266072
H     -0.361939458094    2.577030713609    0.931505970169
H      2.776092022467    0.386171912852   -0.999236952068
H      2.241810143187   -1.786200019194   -0.018866362408
H      1.130925012498   -1.025715919078    1.200335396400
H      0.201346993427   -1.148513598478   -1.809668272914
H     -0.097167980088   -2.451349425334   -0.572932566307
H     -0.110756803369    0.882305768897    1.547378958118
H     -0.965164702955    1.045662874296   -1.657843447427
H     -2.581058871369   -0.480287954157    1.233495040954
N     -1.794849014083   -1.026329575268    0.918935574117
H     -1.842838451808   -2.006397497479    1.155209663908

Ambimodal TS Water5-32
35
ANGSTROM
O     -5.358405815572   -1.204515686467    1.042166133757
H     -4.730385545262   -1.947098800679    1.075282052980
H     -4.941638689353   -0.475143923355    1.530957545011
O     -3.936981799179    1.000051999047    1.925464221371
H     -4.393713783208    1.782170021220    2.235695307713
H     -3.496437212733    1.248333497143    1.080814821024
O     -3.304381915624   -2.415109624981   -1.842393588754
H     -3.719362807307   -2.900840023348   -2.556636047257
H     -3.833861041071   -1.579765288469   -1.748820222949
O     -4.783203271555   -0.271537577616   -1.404243307041
H     -4.216628730432    0.475252079904   -1.177247289203
H     -5.166157922918   -0.585172139090   -0.550849252359
O     -3.529796990496   -3.294344541788    0.695617994461
H     -4.010097593366   -4.122462633623    0.656049658750
H     -3.378946897473   -3.015918299950   -0.236177460009
C      0.209034557681    1.678353528520    0.803001412844
C      1.284760167313    1.531500755490   -0.021194802741
C      1.994410686600    0.300702550108   -0.271191693020
C      1.645959489323   -0.922984109049    0.266529783287
C     -0.244558644660   -1.427637051089   -0.748330531473
C     -1.212111111482   -0.631354626331   -0.137121560158
C     -1.589115732101    0.673782961412   -0.701179353269
O     -2.590673058376    1.338514914672   -0.377868329930
H      1.579741913456    2.381977309018   -0.638861410234
H     -0.311975954004    2.623439946011    0.882766492993
H      2.721181677287    0.320963157748   -1.083091558876
```

```
H      2.209180042352   -1.808999582832   -0.002627430692
H      1.141733264089   -0.994521684469    1.222510605810
H      0.081722902362   -1.195467059388   -1.751374513102
H     -0.170994295935   -2.473898485805   -0.480755502319
H     -0.065425253842    0.947792232368    1.550765607702
H     -1.037433635297    0.977312918799   -1.613803582361
H     -2.522403141883   -0.441175520049    1.446966683067
N     -1.775402345339   -1.002448183845    1.068350796485
H     -1.827053535596   -1.978528159972    1.311068181492

Ambimodal TS Water5-33
35
ANGSTROM
O     -3.539903067058    1.520079740139    2.216139854005
H     -3.205458983028    1.567507292072    1.290373239659
H     -3.519014564420    2.416723118311    2.552316381688
O     -4.902336488678   -0.247110766852   -0.539133594356
H     -5.477612963346    0.031677276825    0.200881796694
H     -4.194386040974    0.408088169695   -0.572414524935
O     -4.467797494816   -2.842694743812   -0.012041209274
H     -4.728839885462   -3.283415418341   -0.821503231542
H     -4.586895925753   -1.881464406294   -0.187986947628
O     -6.015449718620    0.452140970995    1.846735576801
H     -5.164430017788    0.868861213722    2.091593644870
H     -6.094841914272   -0.339359903357    2.381563155076
O     -2.260816925176   -3.705675206753    1.319874986406
H     -2.588307596976   -4.263250685409    2.026766306992
H     -3.047267949792   -3.442123973399    0.799751159103
C      0.309225623243    1.719869655790    0.737188172669
C      1.344778354793    1.502090163601   -0.120257956217
C      1.997795023551    0.234952156089   -0.355639216572
C      1.633351192978   -0.954462732794    0.236498779001
C     -0.338877411826   -1.419730728135   -0.714580551362
C     -1.241165026603   -0.571359522698   -0.077720464819
C     -1.593982619950    0.738207668840   -0.648250293691
O     -2.553651565878    1.442855185777   -0.279623635358
H      1.649579091884    2.319545879196   -0.776733493210
H     -0.166703935203    2.689528212428    0.806166030212
H      2.694870221547    0.204791712211   -1.193176973280
H      2.146640571102   -1.871440528010   -0.026891464939
H      1.144984490467   -0.982846840087    1.202329674414
H     -0.029367044157   -1.216693778213   -1.729385638106
H     -0.287917255221   -2.457572167606   -0.412228148989
H      0.032060156198    1.023053983461    1.515762550070
H     -1.083860167010    1.008662917443   -1.594311410835
H     -2.489373185189   -0.326809538062    1.549958408100
N     -1.764151877535   -0.901891948756    1.152800177958
H     -1.767660199087   -1.876334815071    1.431345516141

Ambimodal TS Water5-34
35
ANGSTROM
O     -1.518822937888    0.231320636150    3.676459015036
H     -1.622894352229   -0.709135150656    3.441215767498
H     -2.132605733775    0.714149518881    3.099226192880
O     -3.533830060244    1.387997398788    2.062736243103
```

```
H     -3.940026579217    2.230700983138    2.268989381810
H     -3.213209170312    1.464295525289    1.128082780216
O     -4.575140268047   -0.871950175221    3.439154100490
H     -4.372533858810   -0.601771115369    4.350957429169
H     -4.393513639526   -0.089634225440    2.897708483824
O     -3.287011006954    0.046167512369    5.705154113332
H     -2.521731866699    0.157009473137    5.096505256315
H     -3.253671156534    0.777489160172    6.321466458101
O     -2.369644273202   -2.371541077364    3.218381168245
H     -2.312045440436   -2.893397615909    4.020070702768
H     -3.264101169240   -1.952552632266    3.238066793002
C      0.176686003878    1.631420538571    0.886333126849
C      1.316809909524    1.481603725414    0.152217111660
C      2.002898630956    0.237377761419   -0.079620540120
C      1.543971840226   -0.991421363457    0.362845324212
C     -0.233058325212   -1.338417928173   -0.862387545395
C     -1.260574875290   -0.571811760468   -0.307490182260
C     -1.557436786870    0.800511795882   -0.763188377356
O     -2.525199948295    1.482397580422   -0.388487733526
H      1.690334449044    2.342755476695   -0.405546681106
H     -0.317209799980    2.591321539612    0.962217725126
H      2.805752210687    0.255439589440   -0.816466420332
H      2.094859456072   -1.891057022929    0.112819468314
H      0.954510515788   -1.075987725617    1.267706487955
H      0.210335288874   -1.033638015270   -1.798981369802
H     -0.210867780975   -2.404820889001   -0.677114497875
H     -0.165722975720    0.890184569846    1.594251946334
H     -0.964551439026    1.155981868364   -1.630733183562
H     -2.773358261929   -0.486948941311    1.096830722068
N     -2.023678071200   -1.045037756630    0.729339892883
H     -1.876186189901   -1.938541187337    1.165741596760

Ambimodal TS Water5-35
35
ANGSTROM
O     -4.155043706627   -1.342595392897    3.552689850726
H     -3.734519667571   -2.044378584747    3.028352964770
H     -4.121407869425   -0.552376213627    2.987889009470
O     -4.300984970460    0.366928770609    1.386660981022
H     -4.848892078213   -0.241248397128    0.871597355384
H     -3.812782945750    0.909740760874    0.736206328556
O     -5.731932863761   -1.932828881471    0.484208419414
H     -6.181712159944   -1.934774136854    1.367452388709
H     -6.381978209999   -2.218547551216   -0.158026010381
O     -6.677680335421   -1.887097768706    2.960315705943
H     -5.773783669486   -1.734585497816    3.341890645265
H     -7.199765884145   -1.127671521837    3.223217308701
O     -3.596782513295   -3.281993820392    1.613063634540
H     -4.013428280421   -4.085376767825    1.929619419993
H     -4.269140071027   -2.819600120501    1.077752746867
C      0.135372271282    1.611000631096    0.877964579728
C      1.261896125277    1.553145576787    0.109697885213
C      2.019650283750    0.365233345291   -0.178498620763
C      1.655647484826   -0.906474719795    0.246009681195
C     -0.112688131885   -1.379330287341   -0.865985900817
C     -1.161606639317   -0.644867435696   -0.296662627767
```

```
C     -1.555779905688    0.681374057082   -0.815221507987
O     -2.582968477442    1.295117997140   -0.507382739340
H      1.564228701271    2.453473928117   -0.428758439881
H     -0.425487617027    2.529447732659    0.987021866840
H      2.789490492179    0.452948938215   -0.944556628984
H      2.265865276875   -1.755795549418   -0.041875107169
H      1.130725955013   -1.046500190402    1.183973591311
H      0.259275919041   -1.094318740021   -1.840308147033
H     -0.041427509261   -2.440002686121   -0.654540642572
H     -0.153747215011    0.824307232891    1.559856199888
H     -0.948707567598    1.050833895020   -1.670698473104
H     -2.622794820155   -0.574594537168    1.154369910509
N     -1.816718087286   -1.094406919664    0.826735940916
H     -1.790665431245   -2.064517562550    1.093184242568

Ambimodal TS Water5-36
35
ANGSTROM
O     -5.778749307699   -0.951754690222    0.610513918948
H     -5.224894235535   -1.754048616865    0.473875989379
H     -6.516703925090   -1.212825952263    1.162100088138
O     -3.730598969899    0.589712977280    1.722127526684
H     -4.553912021634    0.183191167361    1.410375375991
H     -3.433844161614    1.179207175947    1.010646786383
O     -3.427151289389   -1.974924830589   -2.267723547606
H     -3.689929657422   -2.261886027811   -3.143112264656
H     -3.888404376345   -1.109731629047   -2.117951034484
O     -4.749175029022    0.252544285217   -1.707300365278
H     -4.053116416252    0.834704467101   -1.360886036341
H     -5.269496284952   -0.025127273446   -0.938805346397
O     -4.284151738046   -3.116088652224    0.007087382700
H     -4.744711681309   -3.900052457517   -0.295923770857
H     -3.848452718509   -2.729817800073   -0.786110100767
C      0.081851635494    1.577962395015    0.991787194226
C      1.268918058301    1.580923684504    0.320989991369
C      2.050241701171    0.417914219561   -0.020341472814
C      1.669808325079   -0.882176036436    0.254154832278
C     -0.041718916834   -1.245126026112   -1.089610984952
C     -1.116464204476   -0.566484482214   -0.508992482986
C     -1.440450380449    0.819066030898   -0.903103050715
O     -2.492856216652    1.417638956592   -0.627962023556
H      1.618354143278    2.525556416774   -0.100558176754
H     -0.478458671558    2.489987616290    1.148204522842
H      2.880141361865    0.579051617054   -0.707994008779
H      2.295381593731   -1.706048542624   -0.069605877515
H      1.045910684833   -1.113627403833    1.108939679988
H      0.416529141625   -0.851712118396   -1.985155496189
H      0.025981827271   -2.321055288708   -0.986433584871
H     -0.271609574058    0.733255347692    1.565629140546
H     -0.761440355376    1.268136887783   -1.658053677497
H     -2.578862743162   -0.598565489277    0.980499245496
N     -1.880458098022   -1.138139183635    0.470742043538
H     -1.775929015547   -2.105279837143    0.717574314736

Ambimodal TS Water5-37
35
```

```
ANGSTROM
O     -4.986539029504   -1.425647160165    2.885247135147
H     -4.674081004744   -1.959765278074    2.125556914212
H     -4.281087381508   -0.794661718835    3.068898635565
O     -3.641025149789    1.056784527917    2.080548003700
H     -4.593557629835    1.187228593051    1.999616932159
H     -3.261245085687    1.441208195702    1.272568180085
O     -4.226191371445   -2.589420219791    0.547206593201
H     -4.833588610073   -3.247296491634    0.206943616220
H     -4.413891193131   -1.760937088528    0.039451738731
O     -4.877781321627   -0.275698897783   -0.606979032667
H     -4.159703508336    0.374672147686   -0.626942800669
H     -5.516202048427    0.057815095945    0.048230089791
O     -6.410948067414    0.410651453659    1.591919975693
H     -7.354568862915    0.409814237342    1.751049583216
H     -6.027866523774   -0.324517408272    2.133552488783
C      0.177656906876    1.656637319669    0.837170312623
C      1.288137212885    1.534851287114    0.053414352078
C      2.000695322722    0.312034652965   -0.214226871120
C      1.617605742039   -0.931279873707    0.258963862805
C     -0.215980232013   -1.383593638750   -0.836775311608
C     -1.209115106636   -0.598866061756   -0.244952216745
C     -1.555969221722    0.733919564350   -0.770025734065
O     -2.570645170412    1.375217231836   -0.448825097125
H      1.611930178947    2.406944995283   -0.518033749079
H     -0.341343500725    2.601306460100    0.931773613944
H      2.759889380157    0.355975476856   -0.994776799639
H      2.189572864484   -1.809433573379   -0.018591022511
H      1.086363374874   -1.031969184771    1.197932604932
H      0.151666260959   -1.122794292236   -1.818921781832
H     -0.165427349837   -2.440555327983   -0.604632632682
H     -0.127792582468    0.904084591665    1.550227363128
H     -0.966266082110    1.078491052726   -1.643792803358
H     -2.575934905579   -0.416521317070    1.294241233499
N     -1.845215845352   -1.000966922669    0.907298178922
H     -1.887165418958   -1.973886619663    1.156095180305

Ambimodal TS Water5-38
35
ANGSTROM
O     -3.721935539232    2.565873941270    3.214494135190
H     -3.695636887751    1.612480020153    3.022901825168
H     -3.094081403499    2.975914964020    2.591908746241
O     -2.730901641435    3.692706570578    0.914986330977
H     -3.606078221317    4.074019528209    0.793141700509
H     -2.696260628579    2.927440508323    0.317016479456
O     -4.010004022204   -0.052996940355    2.319344329977
H     -4.595157529362   -0.625661247306    2.815653617775
H     -4.553292260152    0.313895392421    1.565088504663
O     -5.264513618221    1.036905946256    0.297552578603
H     -4.441041816947    1.164554963307   -0.198580173839
H     -5.507138654301    1.921651043569    0.634420526687
O     -5.648771466361    3.418461059123    1.586508793584
H     -6.466086379703    3.651720104860    2.026281860869
H     -5.012074008898    3.152319801054    2.298520072578
C     -0.019322771185    1.497009210017    1.044861855331
```

```
C       1.154058594482    1.494493931921    0.347939930863
C       1.923045756095    0.329216447302   -0.003276014057
C       1.537327602064   -0.970087936606    0.284599394748
C      -0.180577789905   -1.319220579366   -1.000293689915
C      -1.255894198667   -0.626226085331   -0.431340110474
C      -1.582660666756    0.750022764340   -0.834971458841
O      -2.621698905067    1.359127099599   -0.537478170139
H       1.494130545398    2.436980758733   -0.084390810353
H      -0.570025673490    2.413212104763    1.208984163224
H       2.739184989407    0.479958862673   -0.709204792886
H       2.155397090703   -1.797848395333   -0.044486118447
H       0.944927790000   -1.191169170737    1.164972978002
H       0.264685171072   -0.947598691393   -1.912051095756
H      -0.121501147338   -2.394672694922   -0.881131240324
H      -0.356256946238    0.655504038456    1.634499922529
H      -0.911743184619    1.191700272226   -1.602216395598
H      -2.704730124048   -0.653334984898    1.076556615937
N      -2.034551340347   -1.198492536915    0.545331622261
H      -1.829363303735   -2.119178446185    0.890531444885

Ambimodal TS Water5-39
35
ANGSTROM
O      -3.077025188869    3.128518839931    3.478974945206
H      -3.942094419158    3.466712792660    3.202373567336
H      -3.070893473855    2.223132959133    3.120652246336
O      -3.781166280436    0.997909354617    1.886450710674
H      -4.614012196409    1.484320744307    1.930707944318
H      -3.389457683190    1.263739096597    1.028987451301
O      -5.318441444922    3.398014800725    1.819731481961
H      -6.125889195941    3.881800835628    1.647943885680
H      -4.690161994825    3.648987712538    1.092878940585
O      -3.510068985520    4.003305601366   -0.030536527532
H      -3.237614854136    3.156587456325   -0.414166802114
H      -2.750855590975    4.278984842373    0.519500487952
O      -1.518823377643    4.470921965571    1.784942615154
H      -1.369864453646    5.345554473933    2.145193070124
H      -2.010194730346    3.967636040878    2.480926425039
C       0.113599470573    1.607895154245    0.885531786676
C       1.250227950093    1.512037037677    0.134025156520
C       1.975648704834    0.300518527257   -0.143253561625
C       1.580896353782   -0.955770218500    0.287711437839
C      -0.214370257962   -1.370271613980   -0.869218731608
C      -1.239964291768   -0.605172950559   -0.303118664052
C      -1.552246654531    0.744929807466   -0.801090426840
O      -2.549819719843    1.416850177526   -0.492422375522
H       1.585812969560    2.401205022237   -0.402312993563
H      -0.404009596937    2.551347175725    0.998663228861
H       2.756735612817    0.363241852950   -0.900614018162
H       2.162498720919   -1.826484713173    0.006361890955
H       1.030575819007   -1.078427406115    1.213280984552
H       0.190388872693   -1.085153650901   -1.829415563399
H      -0.170705981447   -2.433321026827   -0.664227921138
H      -0.201827105520    0.834778316054    1.573091064390
H      -0.935050334279    1.084659355585   -1.658247461162
H      -2.665029141667   -0.512067519467    1.222501941559
```

```
N     -1.956896055679   -1.073778918842    0.774086537305
H     -1.804363525084   -1.996983737991    1.137279448034

Ambimodal TS Water5-40
35
ANGSTROM
O     -3.750699989237   -1.725332897304   -2.027252525063
H     -3.333229904257   -2.609484383944   -1.955720026856
H     -4.363375847422   -1.778316403398   -2.762401604789
O     -4.523325586983    0.459499073212    1.613999582060
H     -5.248318664420    1.066614081022    1.763902930971
H     -3.947515487249    0.879562986514    0.928793502268
O     -2.672284186344   -4.172829591195   -1.559260955182
H     -1.736133019264   -4.328308308193   -1.689631337153
H     -2.815985668216   -4.110154611971   -0.588182266559
O     -3.134773798454   -3.765945716945    1.069572520290
H     -3.567235877082   -4.481770922237    1.536730372828
H     -3.850902100136   -3.091953655148    0.889547721331
O     -4.948772977952   -1.980968995950    0.432988914023
H     -4.877508339461   -1.147832376336    0.934724877125
H     -4.608575315991   -1.776144044206   -0.459162780435
C      0.082062951960    1.620038209232    0.763304639069
C      1.081446073985    1.518767782919   -0.159512239620
C      1.762852646861    0.306020105756   -0.525882933104
C      1.437606444714   -0.945863356872   -0.019932404118
C     -0.482668801587   -1.405488462870   -0.843542544821
C     -1.426386999623   -0.634076383066   -0.154051625851
C     -1.861232271195    0.682210855427   -0.654143153091
O     -2.810541033579    1.346674030807   -0.221488546771
H      1.321772923441    2.400000100236   -0.757256966007
H     -0.438008247606    2.553735643588    0.929772048932
H      2.413587555047    0.357820063480   -1.398170546912
H      1.982648261654   -1.815710241759   -0.370920599538
H      1.053296100488   -1.051545455894    0.987989760571
H     -0.254902031131   -1.163724126174   -1.871798738810
H     -0.397057308164   -2.457731316579   -0.599094390563
H     -0.124578912317    0.852820705058    1.495393811141
H     -1.372879445010    1.011050136305   -1.595548531385
H     -2.656404711229   -0.533187113700    1.496165991158
N     -1.902271095878   -1.051740727507    1.073049831369
H     -1.891550292653   -2.034717906642    1.299397246357

Ambimodal TS Water5-41
35
ANGSTROM
O     -6.836486498439   -2.319737920197    1.557267281834
H     -6.074114192144   -2.679858638243    1.074231970193
H     -6.448191351473   -1.904872755228    2.347147604038
O     -5.403588883640   -0.724363372840    3.317082518988
H     -5.788581979385   -0.011235560022    3.827794327237
H     -5.020793597708   -0.317589777894    2.519652206270
O     -4.556958126243   -2.360388141728   -0.043236624558
H     -4.963668095296   -2.297454193950   -0.909301691678
H     -4.519152578898   -1.444386268141    0.297961498978
O     -4.626086987296    0.248328700856    0.870288313356
H     -4.002711306197    0.781311438439    0.332346493764
```

```
H     -5.511047304078    0.336108992533    0.465563188567
O     -7.147783812274   -0.169590900056    0.000095519175
H     -7.984047045569    0.262713827808    0.172918366343
H     -7.152207000959   -1.003074867421    0.534773349356
C      0.087691975714    1.624468016446    0.851842502618
C      1.225756267540    1.544796880139    0.102185633428
C      1.986532610534    0.348836481916   -0.138459952053
C      1.616052008204   -0.909780793985    0.319599490620
C     -0.131344624400   -1.408471950542   -0.800570424118
C     -1.188503972547   -0.656003517235   -0.273497605178
C     -1.575108032108    0.647920205763   -0.842430302388
O     -2.609611267477    1.272115702983   -0.578180163743
H      1.535120113377    2.428308126008   -0.459227427156
H     -0.475733732813    2.545183565986    0.922936556049
H      2.768810189548    0.413295611666   -0.894043238836
H      2.230686082064   -1.766867726925    0.066397368617
H      1.078930611464   -1.021117579426    1.254675832977
H      0.254054139186   -1.156163348244   -1.778850936846
H     -0.064689360453   -2.462099552996   -0.555163663075
H     -0.209354329894    0.859519586931    1.554915970797
H     -0.950794254490    0.992049845779   -1.695138995854
H     -2.673464952089   -0.565207548849    1.151186018728
N     -1.848156823514   -1.071110748870    0.869494530449
H     -1.843943030091   -2.043560735185    1.127249107793

Ambimodal TS Water5-42
35
ANGSTROM
O     -5.582582774720   -0.994967885458    1.516255764552
H     -4.950830031252   -1.733205117065    1.511887622193
H     -5.098860516204   -0.236176221410    1.881786158948
O     -3.872241746275    1.108927333211    2.009707033583
H     -4.057908754862    1.942655722459    2.441789185408
H     -3.473562501140    1.328478603674    1.136303122401
O     -3.820612086790   -2.886070198211    0.383310047491
H     -4.586172213146   -3.439373371968    0.173413519823
H     -3.851924097400   -2.184305411546   -0.287594286454
O     -4.696673929055   -0.596837256987   -1.036116681513
H     -4.191778524524    0.221454279859   -1.051031407832
H     -5.191487449897   -0.582665979715   -0.191233393327
O     -6.475918764113   -3.008918511133   -0.496846844028
H     -6.725285095617   -2.461471646767    0.255264719689
H     -6.205149445840   -2.394248209812   -1.186132531474
C      0.234642467329    1.670615984490    0.807026228781
C      1.293666983977    1.509798872683   -0.037468505058
C      1.973246376454    0.268149276149   -0.309420251180
C      1.607551978283   -0.953913601433    0.224364925420
C     -0.302160292346   -1.405488397047   -0.754193047775
C     -1.243512898698   -0.590489231167   -0.124038719572
C     -1.604365903194    0.725642542146   -0.674427107392
O     -2.591544430957    1.401982892904   -0.333847786501
H      1.593362635529    2.359472026045   -0.653808381357
H     -0.265749656297    2.625403462818    0.902736216348
H      2.686155810786    0.279690449726   -1.133563916879
H      2.148522203058   -1.848392579944   -0.062122245531
H      1.124713821919   -1.022544542865    1.191818805249
```

```
H      0.012577438459   -1.175925189149   -1.762131351515
H     -0.249508511750   -2.455076914860   -0.492733547799
H     -0.036919991836    0.941380466584    1.557312359665
H     -1.059470721453    1.025384878787   -1.592618039908
H     -2.528581181801   -0.384517200139    1.479342075336
N     -1.796382882287   -0.958758870947    1.088676861659
H     -1.866588314083   -1.936758798425    1.318625986798

Ambimodal TS Water5-43
35
ANGSTROM
O     -1.937477660233    1.421346761088    3.696266238946
H     -1.792707484110    0.640341163790    3.149239567810
H     -1.844583785522    2.192710091442    3.099194972709
O     -2.435285082363    3.380688182753    1.891157873994
H     -3.314378844539    3.533361127444    2.250927076533
H     -2.563784076861    2.868742297248    1.075268994284
O     -4.526965166419   -2.148542753020    1.717267312016
H     -4.741458609548   -1.215028217051    1.550835887889
H     -5.363743348084   -2.614290209214    1.720102349772
O     -4.796800465014    0.608130012504    1.326557340383
H     -4.309670411865    1.112764675028    0.657664716478
H     -4.842624945844    1.174833242515    2.120291478898
O     -4.547604280432    2.070923169527    3.614175971324
H     -5.051935654421    1.881296667982    4.405307666829
H     -3.618019398936    1.803309842231    3.802645242480
C      0.266261235763    1.722036212157    0.732285086384
C      1.245500700967    1.352290423436   -0.142512249970
C      1.784874658112    0.024388227732   -0.268859371919
C      1.324156962152   -1.070363034435    0.449472341703
C     -0.632195746822   -1.424098806984   -0.352069070742
C     -1.498126826672   -0.455405604596    0.163238493975
C     -1.780924310554    0.803608988679   -0.536383543215
O     -2.665791333249    1.612096149079   -0.207645102136
H      1.579242053174    2.078134566268   -0.885859223310
H     -0.144180133516    2.722419966433    0.737393973671
H      2.440274265710   -0.156597727104   -1.120559224782
H      1.762219967073   -2.046082308973    0.270157070488
H      0.922739604999   -0.951413679667    1.449048766514
H     -0.342926598204   -1.368877270630   -1.392411148698
H     -0.674931567834   -2.430467881263    0.047798180666
H     -0.003402223913    1.117766269645    1.586527156195
H     -1.272148051777    0.941750101324   -1.512841814786
H     -2.943915425670   -0.074935724468    1.542210431367
N     -2.077072225606   -0.597202357354    1.429476840232
H     -2.184296835184   -1.539699625505    1.778472827172

Ambimodal TS Water5-44
35
ANGSTROM
O     -3.205149765959    3.405647071851    1.386727577337
H     -4.094775996230    3.078821464113    1.582652897596
H     -2.849550644488    2.779814712422    0.733256611316
O     -3.555817107843    0.501085034210    2.290204792183
H     -3.014938345670    1.265280120562    2.510036631151
H     -4.377852641860    0.871187275773    1.921308409195
```

```
O      -5.648229917295    1.978748163399    1.255425321909
H      -6.578823713410    1.772341169672    1.339569732088
H      -5.475882145830    2.113929664324    0.283478228992
O      -4.966472090638    2.485251068647   -1.237304205999
H      -4.092533150274    2.070003317416   -1.201783944068
H      -4.784648009779    3.443428879671   -1.203597337338
O      -4.173991872560    5.033109042657   -0.564563800290
H      -4.722174267771    5.716211035437   -0.177225210840
H      -3.692995392413    4.607661694589    0.177136961780
C       0.141645161940    1.474359606435    1.068551308057
C       1.321404658286    1.399563246081    0.387855458117
C       2.009560067836    0.186727907524    0.026650355765
C       1.531198025151   -1.085158744767    0.289871725625
C      -0.198675166566   -1.298710327928   -1.034248993333
C      -1.226509515498   -0.544483329100   -0.458081791112
C      -1.448287819793    0.865012260115   -0.827196498015
O      -2.439395044265    1.534003745320   -0.501396344648
H       1.735177254950    2.321719288685   -0.023842599688
H      -0.345302226575    2.424626260658    1.244530458694
H       2.842926904359    0.291106316622   -0.667260666637
H       2.091664211005   -1.949743169205   -0.046879086503
H       0.907039482540   -1.278590784510    1.154414898348
H       0.287991165663   -0.936773042572   -1.928448976978
H      -0.212846246472   -2.377737952791   -0.937269107163
H      -0.263806025812    0.647775721669    1.635601851810
H      -0.755755736749    1.276335314798   -1.592755631010
H      -2.687066803842   -0.468103314037    1.032146504965
N      -2.066519966508   -1.069122846659    0.488485806766
H      -1.944813196602   -2.008627389085    0.822677152884

Ambimodal TS Water5-45
35
ANGSTROM
O      -2.132797150947    3.603050347993    2.539283781401
H      -2.751852732328    4.159929492757    3.024784851888
H      -2.515831975433    2.700396731452    2.548656182178
O      -3.456149571358    1.290158344930    2.000610551850
H      -4.349362499491    1.681489073605    2.035474603738
H      -3.182506042773    1.423805320780    1.069011358749
O      -5.526626372883    3.013624018247    1.635478401126
H      -5.581430493966    3.696821420708    2.310222894078
H      -4.943179425933    3.387944009086    0.948516251540
O      -3.396647894489    4.094057304618    0.191813980575
H      -3.187775484614    3.366561898210   -0.407406299341
H      -2.771127662791    3.972094262050    0.940115924480
O      -4.350369268961    5.530278275977    2.530122470256
H      -4.123700431392    5.346679911092    1.607794456412
H      -4.304042074875    6.480313025520    2.640940542073
C       0.216741491603    1.592103728966    0.891568339112
C       1.324583467232    1.466496215917    0.104220177149
C       2.000989598805    0.232055887491   -0.196718964441
C       1.579932902414   -1.010600288883    0.250753567003
C      -0.260072769353   -1.383050772158   -0.832753653185
C      -1.244169945882   -0.561187263310   -0.268954681157
C      -1.508326179529    0.783662490798   -0.807719613727
O      -2.465603308377    1.514986257242   -0.514628830626
```

```
H      1.672248563075    2.345915898444   -0.440544588619
H     -0.275874963641    2.544813572006    1.030943280114
H      2.755943959524    0.268854497530   -0.981480704219
H      2.125145662244   -1.899313825183   -0.046707379255
H      1.064328966930   -1.112614465581    1.198689889138
H      0.127640005958   -1.146507372713   -1.813227748273
H     -0.256210061915   -2.440527292962   -0.595797900735
H     -0.102832152594    0.823608851020    1.581619555154
H     -0.876061658622    1.069372223182   -1.674949490659
H     -2.580834589533   -0.348616714628    1.324171891322
N     -1.978413601689   -0.982322373183    0.816804332786
H     -1.796580912865   -1.873332472793    1.243165389612

Ambimodal TS Water5-46
35
ANGSTROM
O     -3.071617690017    3.399819625381    3.290506867331
H     -5.832553511204    4.285396733508    2.023301013238
H     -3.014802524475    2.454917632716    3.077167512282
O     -3.660820992082    1.034090439852    1.936727951735
H     -4.569130441124    1.349021728628    1.956452350749
H     -3.320258070363    1.305752507920    1.057992548559
O     -5.249133176047    3.569609204535    1.771726505859
H     -4.228175666948    3.867531380910    0.313903057912
H     -4.499991562593    3.579562312538    2.421886154024
O     -3.495451553325    4.021508706102   -0.312667104663
H     -3.212759166937    3.138848492806   -0.597348618085
H     -2.218400652483    4.420611615710    0.680030976177
O     -1.525689910090    4.574231251361    1.373931744994
H     -1.405765235575    5.522925206815    1.422822553184
H     -2.409148062583    3.835443893251    2.717893407057
C      0.098985749252    1.614612735268    0.886372316189
C      1.233671951902    1.523565721854    0.131875510599
C      1.966518105776    0.315786578790   -0.143927973890
C      1.583100564055   -0.941217436515    0.293411599342
C     -0.215771616301   -1.372634262227   -0.859286289838
C     -1.244262279301   -0.608551214650   -0.297503147254
C     -1.563538831273    0.735740830528   -0.805081250186
O     -2.564561089844    1.405171800375   -0.499605674074
H      1.561508980589    2.413319925546   -0.407958741986
H     -0.423415968340    2.555532542029    0.998347668224
H      2.744266262777    0.381312285702   -0.904421261611
H      2.168889299665   -1.809308290505    0.012955488889
H      1.035906449598   -1.064713822794    1.220748544671
H      0.187366974829   -1.092713021447   -1.821694694233
H     -0.165677978217   -2.433593631658   -0.645309656968
H     -0.210285103364    0.840728124851    1.576090983677
H     -0.948393303746    1.074075245581   -1.663704209718
H     -2.635500078922   -0.492237258325    1.256787203154
N     -1.974052588471   -1.081099817782    0.769936615394
H     -1.772896659886   -1.976981243147    1.176086832828

Ambimodal TS Water5-47
35
ANGSTROM
O     -3.150460912096    2.144976791000    2.166399884918
```

```
H     -3.589211502873    1.326447573381    2.443895880104
H     -2.825650221075    1.950312500639    1.272570698070
O     -3.314411201052    4.022931794766   -0.930174846686
H     -3.004527856608    3.108636277078   -0.924853128831
H     -4.053537813489    4.044122144632   -0.307822021472
O     -4.499529227958   -0.262930939908    2.008318887810
H     -5.186798718183   -0.778055812678    2.430373682725
H     -4.926472184361    0.189603680291    1.233949251402
O     -5.422886962628    1.127081755354   -0.046784197139
H     -4.559552082147    1.206004356523   -0.475975564914
H     -5.608762227311    2.013655221657    0.299975650338
O     -5.184063665510    3.596228554165    1.220895653450
H     -5.445588131970    4.344573011297    1.756950461965
H     -4.420641471482    3.168487649137    1.679693190750
C      0.077524751415    1.754674827145    0.653511340123
C      1.196341030069    1.604689450509   -0.114820475795
C      1.960856681665    0.393347186358   -0.257116527644
C      1.625338629946   -0.811612709258    0.336102019087
C     -0.179285583707   -1.419034613639   -0.736486739251
C     -1.224925254956   -0.660780393842   -0.204175772771
C     -1.626271586082    0.623929767597   -0.794913707129
O     -2.634458829921    1.273587867295   -0.459938856204
H      1.482046713361    2.428210534972   -0.771252789243
H     -0.475205644913    2.685212256710    0.656265264668
H      2.724521638770    0.390165483990   -1.034630855119
H      2.232659127441   -1.689893155256    0.148513137618
H      1.088685847052   -0.841651692180    1.276981016521
H      0.189914049860   -1.192685903573   -1.726013829438
H     -0.067033535545   -2.455359202101   -0.441573009791
H     -0.191237243225    1.068522593586    1.445297062650
H     -1.096882803694    0.914028957319   -1.724463913630
H     -2.721081116150   -0.591450491006    1.244396541287
N     -1.854896362077   -1.037109855592    0.965494877481
H     -1.721648175859   -1.966821973964    1.324659365592

Ambimodal TS Water5-48
35
ANGSTROM
O     -2.325408305981    1.647064323320    3.267318711403
H     -2.965556513094    1.115716361740    2.748029808632
H     -2.223631357019    2.462471060333    2.763816327289
O     -2.901657817929    3.608301437772    1.170602474249
H     -2.997986948391    4.397496351155    0.636080626386
H     -2.822764606929    2.861937197514    0.548697565258
O     -4.215177426883    2.495866572306    5.004183937235
H     -3.416547133216    2.191675491980    4.518390170165
H     -4.503803971864    1.753632197310    5.536254363705
O     -4.228886786942    0.547682103167    1.644345238772
H     -3.901416180070    0.877424791679    0.790851835074
H     -4.837796053754    1.251992232929    1.962657668038
O     -5.315750158526    2.839133983319    2.507454921035
H     -5.117889720316    2.827666827000    3.462217114748
H     -4.570872092083    3.309639389952    2.104014459166
C      0.163838692235    1.639056028473    0.852366535215
C      1.283830847076    1.519262968449    0.081778653883
C      1.997295597698    0.296842423203   -0.182465180578
```

```
C      1.602977137572   -0.946585702155    0.279003821243
C     -0.213374113010   -1.366151100873   -0.865458445051
C     -1.229661390092   -0.617115712875   -0.265175042194
C     -1.572263832607    0.729743999077   -0.749638024280
O     -2.568585077644    1.392571396368   -0.410802860033
H      1.617850689187    2.393399960225   -0.481031468573
H     -0.343179009626    2.590170221240    0.948911340031
H      2.770431629970    0.342547592955   -0.949278372209
H      2.174957579377   -1.826449365116    0.007032565464
H      1.052205433897   -1.048546683631    1.206506923545
H      0.171605302926   -1.065505505460   -1.829003010418
H     -0.147420007520   -2.429214537869   -0.668619435175
H     -0.151250452122    0.890526289229    1.565392480683
H     -0.984092347317    1.079410469158   -1.624113779603
H     -2.672953019489   -0.591091440465    1.236903375285
N     -1.875033232184   -1.080634750309    0.859667150659
H     -1.766810382760   -2.036402747911    1.149724029695

Ambimodal TS Water5-49
35
ANGSTROM
O     -5.543137655128    2.334007040742    2.949799236997
H     -5.351659674600    1.386387414657    3.000755032862
H     -4.673552798984    2.730724820225    2.765746987748
O     -2.900261175018    2.315385887894    2.219966164676
H     -3.017809502451    1.430752223795    2.582615294844
H     -2.850815906627    2.189260831267    1.260374046330
O     -4.364704066563   -0.228946714075    2.358798124806
H     -4.804627930360   -1.030893400471    2.641293571125
H     -4.718987543341   -0.028339628652    1.442725915732
O     -5.159913022938    0.369110520820   -0.051559095833
H     -4.340271398433    0.795873756992   -0.346837859717
H     -5.808728322514    1.097968466167    0.039522800884
O     -6.797454431295    2.421336251348    0.584361583797
H     -7.742771273847    2.374612268237    0.728891397600
H     -6.384784565574    2.515007874680    1.474999475967
C      0.189364793033    1.644360156144    0.833875139237
C      1.277985305676    1.541415173562    0.016481668358
C      2.003068217168    0.330336336148   -0.259112651507
C      1.646312338310   -0.915497467684    0.237997386120
C     -0.188471574466   -1.391144006326   -0.779103738868
C     -1.200977827120   -0.620277786655   -0.196265177842
C     -1.584458707997    0.694741222824   -0.733398186586
O     -2.582886958804    1.342462296219   -0.385628838744
H      1.566323242880    2.414894275983   -0.570821179661
H     -0.348717545355    2.576117781094    0.943922849896
H      2.737365813951    0.373672415025   -1.062947846765
H      2.226468078048   -1.787251651083   -0.044158611365
H      1.161722873468   -1.008268334356    1.203139660786
H      0.151482610433   -1.143023057748   -1.774413213540
H     -0.119749855841   -2.445092389995   -0.535511059003
H     -0.071610049522    0.889385862658    1.562207901288
H     -1.022371442003    1.023504218392   -1.632628317728
H     -2.637555430259   -0.533990625035    1.308581327438
N     -1.824723179110   -1.030319149927    0.966323158074
H     -1.727013959320   -1.978881093080    1.283706836595
```

```
Ambimodal TS Water5-50
35
ANGSTROM
O     -2.784642421535    3.744504538509    0.799848042609
H     -3.472135683884    4.260004577077    0.374711753701
H     -2.701598487077    2.922447570957    0.278994701110
O     -4.320904582340    0.405376780544    1.544744851695
H     -4.220210377257    1.097514096473    2.224145904374
H     -3.925262149640    0.782713141010    0.739956393983
O     -2.602595467429   -3.658283331171    1.822827669375
H     -2.442221701103   -4.069971011890    2.673022045108
H     -3.467814576110   -3.209055105834    1.905013973246
O     -4.910056375529   -2.213068830442    2.090152368854
H     -5.657545845059   -2.404823504825    1.521969117473
H     -4.719412991861   -1.263830590496    1.967098034524
O     -3.641638240948    2.553490221794    3.097661558836
H     -2.909185804451    2.497468921276    3.712572422642
H     -3.321770829790    3.079478033394    2.336010385198
C      0.145964794243    1.650197353143    0.879927587143
C      1.253110257538    1.561081660355    0.091110732674
C      1.991383535523    0.354090961471   -0.198625496715
C      1.651152102661   -0.899575926366    0.264589640690
C     -0.206859278731   -1.395134662534   -0.858954627384
C     -1.210220120903   -0.645049336838   -0.244030136038
C     -1.575995356124    0.688677150727   -0.749107967688
O     -2.590083017307    1.330510127163   -0.414651716679
H      1.560773849456    2.448588816726   -0.465405849475
H     -0.386567373516    2.585023740962    0.997230144434
H      2.746767703079    0.429657124899   -0.980856184143
H      2.236829636910   -1.762510915399   -0.029461315786
H      1.103443744815   -1.031571951619    1.189399202235
H      0.163804388379   -1.104570299866   -1.831171447673
H     -0.116064672148   -2.449047971384   -0.629984398464
H     -0.149062351297    0.869836397521    1.567575227818
H     -0.993985879996    1.050960235271   -1.621813847981
H     -2.617289418145   -0.565012670249    1.258359348479
N     -1.811913430332   -1.073144967415    0.914359299415
H     -1.796787252632   -2.054276446247    1.169024303378

Ambimodal TS Water5-51
35
ANGSTROM
O     -2.939654069178    3.226754093990    3.202278928367
H     -6.075127517158    3.421980254696    3.357543754379
H     -2.800290454950    2.278504408788    3.068349163363
O     -3.684542991086    0.778064284914    2.031822319203
H     -4.556380471448    1.139137278160    2.225064044179
H     -3.424875904279    1.153528491871    1.166392764900
O     -5.523096911383    2.974747143906    2.715924255280
H     -5.461711674433    3.786705672628    1.157793813768
H     -4.582068014100    3.151489791076    2.992059584496
O     -5.269800043060    4.214908560913    0.298570023014
H     -5.470997085758    3.564472431601   -0.376248508047
H     -2.495186532686    3.204907934622    0.178914024161
O     -2.552111147734    4.041355537444    0.666269826674
```

```
H     -3.456274162357    4.362893880307    0.492789564290
H     -2.678610808294    3.649884301951    2.352384875682
C      0.110332159743    1.514134693557    1.008260303774
C      1.239964660965    1.465723614858    0.243314856249
C      1.951061231965    0.272731798344   -0.135802529171
C      1.547475162010   -1.010144929230    0.193519520425
C     -0.258600983339   -1.315279937358   -0.994922224476
C     -1.276530564004   -0.600968979828   -0.353834392051
C     -1.582074009989    0.789502190138   -0.727977453942
O     -2.568245850811    1.446117308372   -0.350762203560
H      1.580810167895    2.391497370003   -0.222837795920
H     -0.396640662377    2.452297636111    1.191118976507
H      2.729258756109    0.390454476047   -0.889316737963
H      2.118881244688   -1.860409143963   -0.161077780224
H      0.995853198082   -1.203989029664    1.106104661744
H      0.142779653164   -0.946548979164   -1.927645238528
H     -0.214119359252   -2.392003092374   -0.883473854772
H     -0.214648572251    0.693892443555    1.633964535913
H     -0.967908554634    1.198393828924   -1.556353564277
H     -2.654347993386   -0.627205092872    1.218803139741
N     -1.994122602382   -1.165427520655    0.674792098934
H     -1.807877393691   -2.103947918123    0.979019208905

Ambimodal TS Water5-52
35
ANGSTROM
O     -1.344258594241    3.426778321735    3.811982106705
H     -4.425574494684    3.037768371301    3.805724140563
H     -0.661065396185    2.755707716503    3.772167172840
O     -4.162321334092    0.352984020750    1.594534325086
H     -4.098554193868    1.039686280958    2.273685580424
H     -3.930388940532    0.801390784111    0.764642958509
O     -3.820552388866    2.658205094873    3.167755388759
H     -3.689813457512    3.384363553836    1.481599691330
H     -2.911671957514    2.890108160940    3.473158103060
O     -3.366913659851    3.771677718891    0.649277636277
H     -3.134578644529    3.015155452327    0.088077255442
H     -1.955395707416    4.486313931973    1.125155777127
O     -1.121957857533    4.829391158560    1.544391930157
H     -1.210216650806    5.782804348574    1.564537536380
H     -1.192122403643    4.010228136190    3.032049587189
C      0.133999643971    1.616829936855    0.889347061295
C      1.260624598028    1.523250680974    0.124914321321
C      1.987105120724    0.315957251192   -0.166769055201
C      1.598087211983   -0.945581971414    0.258147537913
C     -0.194984080185   -1.370080041532   -0.869632420810
C     -1.224400199266   -0.629377127157   -0.274063442068
C     -1.587752638896    0.708859511381   -0.775619068132
O     -2.599356613426    1.349998407497   -0.458552322671
H      1.589881930796    2.417454691983   -0.406835157174
H     -0.381533094383    2.561298988567    0.995764985563
H      2.759761983238    0.386556859124   -0.931734611972
H      2.184564148219   -1.810302805771   -0.032084417114
H      1.061121598571   -1.076149627703    1.190772721679
H      0.179444835274   -1.069959683148   -1.838025417436
H     -0.129901622723   -2.434773151759   -0.677884326866
```

```
H     -0.187405318507    0.836683942813    1.565665424279
H     -0.980821365440    1.069176701461   -1.633779631153
H     -2.690858410690   -0.607098261989    1.211110170764
N     -1.859841872951   -1.086276901186    0.858685483174
H     -1.765489614444   -2.051184801773    1.126025610377

Ambimodal TS Water5-53
35
ANGSTROM
O     -4.061174955012    3.490170044182    2.586450891310
H     -7.024143790552    2.747126730797    1.734080336799
H     -3.636047155044    2.636173168016    2.736941483329
O     -3.812021759196    0.764819336526    1.750723082385
H     -4.767189896918    0.894031006980    1.723442069500
H     -3.476397664258    1.153106941771    0.918929716713
O     -6.187366728973    2.359491520690    1.478038921931
H     -5.673585425595    2.719107575539   -0.211854894219
H     -5.483578003045    2.878223555686    1.950058059480
O     -5.217806441689    2.979143492182   -1.033656356094
H     -4.604743482596    2.265391042584   -1.232727022366
H     -3.865120734166    4.094245412700   -0.322656251633
O     -3.021851859220    4.085005370490    0.165539122576
H     -2.612946994508    3.256091517340   -0.120725225100
H     -3.625381514932    3.863496901981    1.787866538021
C     -0.037771499351    1.574809644528    0.992507172790
C      1.153723365926    1.532407134323    0.328747800252
C      1.920294324888    0.345614291513    0.046941057236
C      1.523111001077   -0.935380147346    0.384118247330
C     -0.176517734962   -1.325997569238   -0.953507079677
C     -1.257563002728   -0.617988075408   -0.419785757275
C     -1.553349297159    0.754902151644   -0.861056944428
O     -2.579184548269    1.404034432007   -0.588913940438
H      1.514476212292    2.452410069854   -0.134024231651
H     -0.578105992253    2.505443950580    1.108358970031
H      2.756378387967    0.461913073416   -0.641912704857
H      2.139859007179   -1.781613760827    0.104427411876
H      0.895044590332   -1.117077035448    1.248370561498
H      0.298362613670   -0.973388045623   -1.857351918151
H     -0.114185973644   -2.396822367893   -0.802412085185
H     -0.400869364089    0.765252179610    1.610749769979
H     -0.879634175579    1.151578510458   -1.647789547782
H     -2.744654276324   -0.617471679708    1.052434778843
N     -2.055217364290   -1.169960020032    0.554372796451
H     -1.861593291241   -2.087614111680    0.913768045242

Ambimodal TS Water5-54
35
ANGSTROM
O     -2.750944009929    0.978516378295    6.103989496497
H     -5.751793268033    0.459719702636    5.386622968831
H     -2.190383474121    0.426044668428    6.650115066482
O     -3.502706757268    2.458792279140    1.894740558431
H     -4.002859134213    3.233920658098    1.637621915416
H     -3.149393947005    2.082135043578    1.061392293549
O     -5.029662000828   -0.131880497010    5.171471998224
H     -4.478742498110   -0.309567504097    3.491641850202
```

```
H     -4.229541690840    0.254264061932    5.591444923261
O     -4.029227395481   -0.177758833330    2.634857729497
H     -4.185216126316    0.748185229573    2.391903882111
H     -2.417946752418    0.323082053438    3.283844321380
O     -1.752537701198    0.956826298717    3.620761711809
H     -2.001843059146    1.788555985959    3.200325398415
H     -2.298694363669    1.043999696296    5.228970779555
C      0.203979743804    1.653772662761    0.835430620791
C      1.319312682750    1.519096172220    0.059914048887
C      2.022237440651    0.289637756288   -0.189146771213
C      1.604538169394   -0.946945597824    0.282032357726
C     -0.194403428250   -1.357494128726   -0.832852628558
C     -1.215129408548   -0.596615255875   -0.249467382429
C     -1.563468394135    0.747340150383   -0.746967814463
O     -2.538742626540    1.419502514122   -0.388306588794
H      1.654360941172    2.383695554609   -0.516896285567
H     -0.305748772513    2.605170003109    0.915261668229
H      2.792163938585    0.315962071307   -0.959893500478
H      2.170939147029   -1.834527348888    0.021882543302
H      1.069801128200   -1.029088666343    1.221083685686
H      0.185756563807   -1.073060073597   -1.803629933836
H     -0.147488419996   -2.420960673283   -0.630330074390
H     -0.105748491499    0.915296486584    1.560943293912
H     -0.990454481074    1.077941495455   -1.641398475514
H     -2.686055373731   -0.556151066307    1.226514386237
N     -1.878806492912   -1.045439870377    0.867296066912
H     -1.767224509432   -1.991208225165    1.187296887163

Ambimodal TS Water5-55
35
ANGSTROM
O     -1.534560789458    0.221003512535    3.685364662877
H     -1.634966727542   -0.725154595702    3.480628406457
H     -2.149328991255    0.682202685345    3.090126720007
O     -3.558767064746    1.307585250658    2.054485450943
H     -3.925248692213    2.146657216821    2.336717195173
H     -3.245437206882    1.440380297118    1.124422127519
O     -4.563030164079   -0.925179590996    3.514894522444
H     -4.364059350694   -0.574518953524    4.400639344420
H     -4.431690006786   -0.172679972292    2.920350816290
O     -3.331855597932    0.222263988938    5.697074897224
H     -2.572287839818    0.321534280961    5.079490346148
H     -2.961898702433    0.003982483186    6.552286949065
O     -2.367635718405   -2.403151124878    3.209506960604
H     -2.352463672384   -3.069275505078    3.897469440572
H     -3.257284057936   -1.972824969635    3.268093114758
C      0.172818843753    1.615262341635    0.906165098529
C      1.312753248958    1.472387216547    0.170351441097
C      1.997794773647    0.230304440320   -0.074961115232
C      1.536867928555   -1.002933477617    0.353951759866
C     -0.239321148476   -1.336588978922   -0.870915496509
C     -1.266540407531   -0.571988790962   -0.311411398549
C     -1.559595206237    0.804243426231   -0.759356250235
O     -2.525791836992    1.487394126859   -0.385004005823
H      1.686761319469    2.339190083846   -0.378177114903
H     -0.320931725324    2.574306999237    0.992082223715
```

```
H      2.800012195254    0.255065266545   -0.812237177126
H      2.087160658180   -1.900339401469    0.094558080111
H      0.949632192817   -1.096024742335    1.259497517389
H      0.203669785933   -1.026830931366   -1.806128046215
H     -0.220602839218   -2.404570261434   -0.694003072692
H     -0.170210947991    0.867046119300    1.606360829789
H     -0.960223644029    1.163356655597   -1.621261011269
H     -2.763404159884   -0.484711506181    1.109022948314
N     -2.039050264014   -1.056718523682    0.713043334510
H     -1.872541057500   -1.939458049694    1.164816859939

Ambimodal TS Water5-56
35
ANGSTROM
O      1.928775542144    0.646531334929    3.636393101781
H      1.279170994379   -0.079116371496    3.513845640516
H      2.314754028889    0.807770216576    2.773480831931
O     -3.591414226167    1.211887182121    1.975930848986
H     -4.474085691442    1.577618669611    2.033941720283
H     -3.269169864945    1.402837014335    1.064504398114
O     -1.813482305593    0.622985961925    4.021196943519
H     -1.238791795955    1.407194657851    4.128803250311
H     -2.475580140719    0.868447109237    3.355202308549
O      0.102934539149    2.545390347500    4.289684541859
H      0.837195295685    1.935325891196    4.062892064578
H      0.276673395492    2.851848823648    5.180593598529
O     -0.062049531694   -1.187361649275    3.304500093549
H     -0.026262611910   -1.815497579587    4.027315279579
H     -0.775699276750   -0.535472552674    3.557392903602
C      0.150694904638    1.660315198543    0.845566394396
C      1.264511860166    1.583552138838    0.062265794603
C      2.012588574493    0.381422736295   -0.207728771756
C      1.656067827764   -0.874777740038    0.259051763426
C     -0.168783137259   -1.349342910809   -0.805905738530
C     -1.195496183931   -0.584878721390   -0.240970432298
C     -1.581532621101    0.722538988711   -0.807088222812
O     -2.563222803626    1.400015148965   -0.481732038321
H      1.563308529906    2.467580057536   -0.503662475499
H     -0.402440465294    2.582893569628    0.953033818395
H      2.767517647529    0.446750363453   -0.990978498227
H      2.249024899109   -1.735111463366   -0.031401826988
H      1.138787387395   -1.002312093841    1.202723867283
H      0.191641257588   -1.103002702880   -1.794692900431
H     -0.089579012375   -2.397661147032   -0.546347188864
H     -0.126746139998    0.886868513454    1.546345625973
H     -1.002463371931    1.028146243268   -1.706084707901
H     -2.515622542386   -0.411265099710    1.343278510758
N     -1.889799478407   -1.039658203094    0.860920267456
H     -1.495474070573   -1.771419855786    1.428691093337

Ambimodal TS Water5-57
35
ANGSTROM
O      0.728551666342   -6.328227731882    2.643869936166
H      0.820757267750   -6.046180740822    1.717579450136
H      1.104238553157   -5.605611752518    3.158648776935
```

```
O      1.515201494530   -3.529503024859    2.688258309728
H      2.160388868938   -2.834013535149    2.824249489968
H      1.627788255755   -3.843095273941    1.780658312972
O     -1.178509251158   -3.582252736809    1.734695510947
H     -1.645443741773   -4.351991420043    2.115588992152
H     -0.471219760815   -3.372153586832    2.360649126743
O     -1.928036901604   -5.919073782911    2.845748574358
H     -0.997916275648   -6.235833919298    2.816536173915
H     -2.302090062588   -6.234249634212    3.668605082044
O      0.676810631835   -4.869898140540    0.267526939530
H      0.389472903912   -5.269970529491   -0.553558263025
H     -0.099137114371   -4.386373259320    0.635564015830
C      0.344852676197    1.268545007224    1.069113497972
C      1.416587918548    1.155116556596    0.232222756118
C      1.946806635883   -0.075699507011   -0.283910288636
C      1.350274457815   -1.316804547486   -0.059464528189
C     -0.468985796834   -1.287263562444   -1.095779660114
C     -1.376856080998   -0.495256169870   -0.374765405079
C     -1.612627234749    0.942781943354   -0.649243887955
O     -2.501857219326    1.604981088413   -0.126818808035
H      1.852471939532    2.070852429006   -0.170753669028
H     -0.052233363278    2.233054525476    1.352480131573
H      2.696163158326    0.003016784612   -1.069897308139
H      1.792329359463   -2.200722689132   -0.508427772704
H      0.849077902021   -1.514295894406    0.881579034156
H     -0.099593129759   -0.915432089673   -2.042110709329
H     -0.588752825337   -2.363388147634   -1.055927553525
H     -0.054021343381    0.436608758476    1.631332547805
H     -1.003650550158    1.376218946155   -1.477375017605
H     -2.717082373392   -0.392564344190    1.166259420565
N     -2.112726525289   -1.017329571510    0.658372539217
H     -1.981809087558   -1.958127737977    0.999788633296

Ambimodal TS Water5-58
35
ANGSTROM
O     -1.702227228452    0.130085402744    3.553689046328
H     -1.524301653959   -0.434859178505    2.786361965754
H     -2.377316428720    0.755661078040    3.229751948316
O     -3.527438558355    1.589562843769    2.140499089491
H     -3.896503093528    2.461449395052    2.282536626077
H     -3.209129519089    1.579268353115    1.210217176156
O     -1.118167935383   -3.603851757220    4.259614361403
H     -1.803843705587   -3.006758513174    4.630503855470
H     -0.273364488663   -3.208292889927    4.480444750255
O     -2.777303398730   -1.692443858551    5.182409006865
H     -2.426998612048   -0.961068344732    4.620508828683
H     -2.772259259875   -1.367954384971    6.083382499602
O     -1.744137017021   -3.732022842710    1.637937000595
H     -2.461549382125   -4.365179612201    1.587493063351
H     -1.462999528089   -3.728658988758    2.578720701361
C      0.210797419479    1.673756793465    0.864728146642
C      1.340724790046    1.438003812197    0.138036597266
C      1.990737552253    0.162971887929   -0.007387176334
C      1.491128283359   -1.026035124180    0.507656026256
C     -0.250811964776   -1.401138490106   -0.672187618441
```

```
C     -1.275361096558   -0.589739283958   -0.176012121191
C     -1.579625841314    0.745829224972   -0.712535061746
O     -2.541894155539    1.448325848120   -0.369799552152
H      1.735260329895    2.246846526082   -0.480124828641
H     -0.263557720434    2.646096986598    0.865639064311
H      2.793426277668    0.108756103043   -0.742619614015
H      2.025352397789   -1.951062470171    0.321927824843
H      0.912398614938   -1.037409444386    1.423532493556
H      0.191055429151   -1.168232342486   -1.631052991424
H     -0.261631735574   -2.454094864715   -0.416922349346
H     -0.152780551280    0.994779989234    1.621495111979
H     -0.988938147484    1.049033011335   -1.603439133917
H     -2.885350718393   -0.531197564449    1.090860460773
N     -2.001556837048   -0.996815032427    0.941096881143
H     -1.991069073553   -1.990942797377    1.168459100807

Ambimodal TS Water5-59
35
ANGSTROM
O     -3.008269372774    3.928517115853    0.503665137518
H     -2.927290993701    3.686383690089    1.443789327579
H     -2.848902868519    3.109224634876    0.008984374587
O     -3.897577979344    0.655235755530    1.854806022030
H     -3.447973481045    1.257916136859    2.461391816926
H     -3.711444642832    1.013896044304    0.966980688458
O     -5.735596204337    2.743312863643    2.737220852609
H     -5.867550872768    3.264137217207    1.915813659250
H     -5.634208078630    1.828336401254    2.446430628343
O     -5.672915345333    4.165725321666    0.446695258833
H     -4.689835118386    4.120789471392    0.362219808759
H     -5.900792535135    5.095225780813    0.416928832806
O     -3.155769887372    3.100155222197    3.150409623830
H     -2.946427676002    3.647193825932    3.907262945609
H     -4.149191623507    3.054278082932    3.102149963233
C      0.104181800077    1.619133929322    0.902470379107
C      1.223500557183    1.548850478008    0.124641890156
C      1.965314532341    0.352474484614   -0.174880305619
C      1.603258190787   -0.913901279703    0.256956824155
C     -0.200368722800   -1.372798979955   -0.857730584210
C     -1.231195466994   -0.634142382468   -0.264315291983
C     -1.589190102929    0.706819660975   -0.760719550857
O     -2.593658562644    1.356461282754   -0.433371565842
H      1.529961438930    2.446644268482   -0.415000804850
H     -0.434670661019    2.549345952024    1.024059867108
H      2.727664553430    0.435015154480   -0.948896346233
H      2.198508894016   -1.770054076084   -0.040137250270
H      1.076516842679   -1.051961784265    1.194112853205
H      0.170490533125   -1.077339638646   -1.828640729143
H     -0.124905621633   -2.434833740651   -0.655938894423
H     -0.187550556824    0.836696378965    1.588923659398
H     -0.989654442981    1.062781910914   -1.624728248082
H     -2.659969971675   -0.581915646939    1.259791672530
N     -1.880931398400   -1.098226412637    0.855726783509
H     -1.766134418913   -2.050932703599    1.152992607949

Ambimodal TS Water5-60
```

```
35
ANGSTROM
O     -0.574304081758    3.043441495889    3.908054601046
H     -0.754579005652    2.085894635262    3.812213520864
H      0.179171526710    3.233682253023    3.347453158500
O     -2.428101542522    4.083165796612    0.172601080573
H     -2.517130497619    3.154969901099   -0.114791996735
H     -3.128982479922    4.566953642107   -0.266923487864
O     -3.495601080848    1.244865220409    2.210528973915
H     -3.500530761259    2.171711929630    2.553730535302
H     -3.333347887802    1.348279994989    1.256048065315
O     -3.040509632248    3.778849427688    2.872719385689
H     -2.209447499688    3.679574911880    3.369865951030
H     -2.779680623835    4.051630809644    1.975965283023
O     -1.353422250321    0.443889810292    3.545764978734
H     -1.650493726753    0.155234695576    4.409870401600
H     -2.175957622301    0.714985583683    3.058308578888
C      0.156190839063    1.695930743866    0.788767006063
C      1.286472230543    1.544779562676    0.037846006461
C      2.003950420680    0.311888182157   -0.156159078132
C      1.594384424805   -0.908123827199    0.352572494552
C     -0.200222244437   -1.362110979218   -0.822265574530
C     -1.233666757308   -0.607283189124   -0.261780864662
C     -1.574528137451    0.735668566934   -0.757379624703
O     -2.559969736126    1.404259759105   -0.394803653913
H      1.623149093460    2.391374399468   -0.563722103070
H     -0.362018395631    2.645905789390    0.823646530500
H      2.790219322206    0.320403513701   -0.910727736102
H      2.165971388036   -1.802206479647    0.131277382714
H      1.025748727812   -0.966108826236    1.273204722257
H      0.206098769001   -1.079393780791   -1.782480486294
H     -0.133748891018   -2.419938888835   -0.600222042665
H     -0.154383208751    0.976056579953    1.533506712902
H     -1.004436285422    1.076437550958   -1.645870379741
H     -2.609592843578   -0.483347135971    1.286675870601
N     -1.965387215699   -1.085865285421    0.800556628396
H     -1.702342180863   -1.933734468666    1.269843461467

Ambimodal TS Water5-61
35
ANGSTROM
O     -1.771786441206    2.674448714491    3.872562068390
H     -1.126819693638    3.305991869600    3.551195087484
H     -2.494135872271    2.651616077312    3.203937720523
O     -3.587842846946    2.534933798188    1.891783638667
H     -3.629203452172    3.398694238447    1.459336299303
H     -3.278154436101    1.941914226915    1.183279172514
O     -3.679066184862   -0.261614168467    2.632252573412
H     -2.851231459715   -0.236550453646    3.147442700939
H     -4.013885147058    0.640616069865    2.625880298736
O     -1.260508454039    0.037221391192    3.932487412168
H     -1.358818578386    1.014682909982    3.962537256927
H     -1.258542681334   -0.256098787123    4.844492409431
O     -2.971788562461    4.149712057708   -0.311990405992
H     -2.761446467102    3.216840688074   -0.505190417646
H     -3.549599751603    4.444362089668   -1.016883978077
```

```
C      0.041152030515    1.488824226395    1.028510789056
C      1.231299138745    1.388040916582    0.369175797947
C      1.941974612415    0.163794226803    0.098154085317
C      1.470270049446   -1.091948236580    0.424530278738
C     -0.239588402839   -1.359270241762   -0.976321549748
C     -1.297080096157   -0.653820887342   -0.396329728440
C     -1.556216221050    0.753221279175   -0.749980111141
O     -2.549872723129    1.414514946270   -0.401639056037
H      1.637588626212    2.285771875186   -0.101949276825
H     -0.456185418966    2.445909319358    1.123539528485
H      2.794341925371    0.238698704511   -0.577095527272
H      2.039252049914   -1.972165183169    0.148590537946
H      0.805291059766   -1.240393439342    1.266544014986
H      0.258226139700   -0.962716377055   -1.848926998541
H     -0.202531111763   -2.437528451640   -0.886837643376
H     -0.352933848451    0.704703142079    1.659681627477
H     -0.901778029970    1.168089346351   -1.546583928115
H     -2.792639202275   -0.712069304626    1.085591051529
N     -2.111934969458   -1.234142037670    0.535358396867
H     -1.937062263473   -2.172071772055    0.850433380356

Ambimodal TS Water5-62
35
ANGSTROM
O     -2.955538710520    3.887400457846    0.842459814467
H     -2.655014037749    3.552487840849    1.694621331176
H     -2.882826756266    3.146549430363    0.224472797845
O     -4.032801345665    0.650165306325    1.746098245765
H     -3.745735556433    1.246912291667    2.467045712885
H     -3.639760915374    1.029604161740    0.932881931547
O     -6.428928056421    1.851050055502    1.315728179428
H     -6.530646760819    1.836872167898    0.362991250806
H     -5.659564686170    1.277439867891    1.519509643758
O     -5.254829585185    4.062262846930    2.432208598384
H     -5.842611386639    3.367772217452    2.076708703790
H     -4.670234377826    4.316678410145    1.700740251858
O     -3.356630399118    2.589622070567    3.519825219836
H     -3.439887774733    2.506069197861    4.469581837332
H     -4.099888450920    3.187465589749    3.230102053733
C      0.144611993911    1.654994681423    0.846085422923
C      1.254778267005    1.539851523329    0.060326971163
C      1.971307950075    0.321542860465   -0.211997354592
C      1.591062022794   -0.924858952796    0.257023453955
C     -0.235798950038   -1.365466082737   -0.843529890302
C     -1.247137929609   -0.609830757839   -0.240654662645
C     -1.596001408714    0.729641543720   -0.744591136826
O     -2.585883330007    1.400507541018   -0.406936222941
H      1.572914221713    2.415689493410   -0.507821602171
H     -0.370237517636    2.601639176747    0.943201807547
H      2.730421324237    0.369575956361   -0.992212539332
H      2.165687241604   -1.800573613189   -0.022853729347
H      1.062889550238   -1.029471857807    1.197585937612
H      0.130695806661   -1.079530602054   -1.818767837268
H     -0.167344443044   -2.426184533981   -0.633669447742
H     -0.155857192988    0.901415155559    1.560736273654
H     -1.015600011318    1.064003576247   -1.628874659889
```

```
H     -2.675832060057   -0.563181529175    1.284060121321
N     -1.874977260574   -1.058405550756    0.901597034715
H     -1.769004858066   -2.015806682081    1.188498991633

Ambimodal TS Water5-63
35
ANGSTROM
O     -2.858336924059    3.960181026148    0.591879014694
H     -2.870527263959    3.723822573983    1.537704232556
H     -2.707955991081    3.130025963567    0.113674807960
O     -3.898234191211    0.689751695725    1.855066155493
H     -3.483350677922    1.275497358511    2.501124177241
H     -3.646278050577    1.056446718012    0.985444349303
O     -5.791882182510    2.738391862131    2.600354791897
H     -5.863652077631    3.269124025648    1.780189317609
H     -5.631136974937    1.829413029905    2.314896846896
O     -5.519022821666    4.047979971770    0.240190296501
H     -4.545360373122    4.174988766430    0.325862250082
H     -5.656395401850    3.619700034061   -0.605733911420
O     -3.251820225175    3.141387890473    3.205670041639
H     -3.099959910573    3.756561291612    3.923010951438
H     -4.238306862065    3.089056554612    3.088096528064
C      0.124873970917    1.628355835376    0.892675966547
C      1.238738199360    1.543972892534    0.108454552022
C      1.967538725094    0.339021053959   -0.191339936598
C      1.600074696871   -0.921418153974    0.248906486310
C     -0.222384915036   -1.364435634482   -0.863366866755
C     -1.239925604735   -0.618238531940   -0.258775864906
C     -1.585432131809    0.726882529535   -0.749053213507
O     -2.582670258639    1.389392512417   -0.416671113742
H      1.550532778039    2.436697621187   -0.436379750544
H     -0.400824839863    2.566081645965    1.015365128922
H      2.726904724569    0.412621251665   -0.969298232050
H      2.183962993784   -1.784692541059   -0.049642804411
H      1.071629343593   -1.052672502307    1.185948640271
H      0.148212448346   -1.067915163800   -1.833865690591
H     -0.150898509340   -2.426808383890   -0.663015977773
H     -0.170034978231    0.851634262120    1.584589872726
H     -0.991647490173    1.076351691279   -1.618895036442
H     -2.660152521787   -0.566525207100    1.274764196654
N     -1.882120237705   -1.080421780115    0.867556949186
H     -1.773695555638   -2.036531016232    1.156398091712

Ambimodal TS Water5-64
35
ANGSTROM
O     -1.484556782211    3.274596972713    3.555006110556
H     -1.670645076047    2.832839914033    4.406982869089
H     -0.582153447477    3.039613377180    3.328350832588
O     -1.118240329241    0.052769720782    3.591429549461
H     -1.130348804928    0.376679973816    4.496873165770
H     -1.832521222146    0.536557994497    3.142874803710
O     -4.379908576367    0.871137533227    4.553633425900
H     -4.123858742209    1.162207088681    3.656912858518
H     -4.480738553559   -0.081197729829    4.506818066282
O     -3.113925802731    1.533584742860    2.256288662532
```

```
H     -2.645562310444    2.334617157672    2.567154314482
H     -3.102990976604    1.575086134060    1.278342698751
O     -2.145686138864    1.765217525314    5.753935094601
H     -2.351189111262    2.227543601153    6.567147985585
H     -3.004739900838    1.437226262056    5.399782721067
C      0.167258919208    1.723255304264    0.755353131209
C      1.268754989384    1.591817376501   -0.039393515440
C      2.013572569572    0.376901331624   -0.227401258854
C      1.643703354833   -0.842362637605    0.326225958363
C     -0.148187921063   -1.371135465769   -0.722266147305
C     -1.195552415667   -0.610436523964   -0.189566281271
C     -1.620780298883    0.680450169837   -0.765321942142
O     -2.601702415439    1.335326373340   -0.395220604803
H      1.553348184477    2.432949874427   -0.674305084702
H     -0.390798530515    2.650238006405    0.788135105413
H      2.766896836809    0.381639050968   -1.014503550879
H      2.236662812402   -1.724383614393    0.109631581904
H      1.137586370353   -0.883597657396    1.284228239356
H      0.208916124651   -1.148004740473   -1.718109629957
H     -0.064263575694   -2.413871795657   -0.440649143483
H     -0.085344974273    1.005428052388    1.523632855858
H     -1.087533752962    0.986906697408   -1.691386530788
H     -2.530751999264   -0.393924798858    1.353966514251
N     -1.906572417298   -1.049730076146    0.910032049868
H     -1.522071134524   -1.751158764867    1.519867894870

Ambimodal TS Water5-65
35
ANGSTROM
O     -0.517405456775    6.092286910400    0.276428572498
H     -1.230825437104    5.430229907896    0.260080167981
H      0.286624619817    5.589074588843    0.104097918129
O      0.333377308430    4.203295434061   -1.764733591157
H     -0.528330259445    4.011897990013   -1.348169853154
H      0.190447320925    4.990273734967   -2.297919204426
O     -3.060028602455    5.345382505285   -2.734811534381
H     -2.340164898106    5.999088498259   -2.614919141987
H     -2.869881218953    4.883873871687   -3.552940862182
O     -0.942379158719    6.977614258512   -2.215763514094
H     -0.786873657403    6.746740932535   -1.266251212921
H     -1.102112192093    7.921568958190   -2.235892220214
O     -2.138789272458    4.036386678980   -0.515139936354
H     -2.441813046749    3.115891286003   -0.407656045139
H     -2.678623248467    4.427148128095   -1.227420695835
C      0.205385907734    1.582513527912    0.894098546495
C      1.286563908142    1.524722735650    0.057984175621
C      2.012919460522    0.331470315222   -0.266008882060
C      1.646395508859   -0.939904269502    0.175910324152
C     -0.152264041280   -1.360939167996   -0.824810123931
C     -1.173219931634   -0.594968340725   -0.245024115532
C     -1.556375990323    0.741449898422   -0.717425518312
O     -2.558451991615    1.353075605982   -0.319721404317
H      1.544059910375    2.418694371932   -0.509790175605
H     -0.347149905081    2.501884696704    1.032063067915
H      2.741697169138    0.404983105950   -1.072215266461
H      2.232075546135   -1.796736904767   -0.141552932717
```

```
H      1.188332664218   -1.069956405143    1.150509586321
H      0.184838773708   -1.098679100900   -1.818443674109
H     -0.113735347422   -2.424074567492   -0.612567163153
H     -0.035804663487    0.803746709904    1.605029801565
H     -0.990959103800    1.127680913485   -1.591531803530
H     -2.636207644956   -0.470936788053    1.173188720331
N     -1.837488839876   -1.022841237162    0.898849920303
H     -1.857460022625   -1.995448626797    1.151501931751

Ambimodal TS Water5-66
35
ANGSTROM
O     -3.265511786974    2.227654202211    2.103811403556
H     -3.541603770471    1.321818604722    2.355500152324
H     -2.927441475313    2.123920761139    1.200059037811
O     -4.396549590989   -0.241770107853    2.126705133973
H     -5.114617313519   -0.077233402680    2.760978817505
H     -4.796000767169   -0.012880799668    1.258440505216
O     -5.327459755519    0.901000261011   -0.143656377526
H     -4.435839186582    1.129613393295   -0.445316361707
H     -5.669699506929    1.699401980367    0.281607862698
O     -5.752837883081    3.012897826656    1.730888778843
H     -4.791088290906    2.824515912468    1.910530460086
H     -5.903958742037    3.919672988108    1.997571577561
O     -6.681729204811    0.860970784734    3.190591839129
H     -7.449352530413    0.522718749627    2.726913345880
H     -6.429804400348    1.684500314112    2.730210358704
C      0.149422598537    1.638824447186    0.856930166950
C      1.263748685407    1.540955921038    0.075509133256
C      1.990731307188    0.329441985757   -0.200242404598
C      1.615721137181   -0.921881144930    0.261714688319
C     -0.200137798026   -1.374168602598   -0.839138151989
C     -1.222435290591   -0.619251588767   -0.253267414689
C     -1.578770482775    0.713303840801   -0.769541089764
O     -2.573105352717    1.375928269450   -0.437014649756
H      1.579054737522    2.423078616467   -0.484858593475
H     -0.380216816680    2.576115677636    0.964723628498
H      2.751603058332    0.386095488006   -0.978224581834
H      2.198525334905   -1.791993370962   -0.019139856550
H      1.088630733848   -1.032777137425    1.202071531334
H      0.171671601743   -1.094143382062   -1.814208428410
H     -0.131781362469   -2.433474591858   -0.622146781632
H     -0.147644284127    0.873813410003    1.560352246493
H     -0.992899584053    1.047735464668   -1.652358765443
H     -2.679666906845   -0.585381342924    1.242272926378
N     -1.862621866268   -1.059758806598    0.883416417688
H     -1.759281092244   -2.013301384521    1.184093358002

Ambimodal TS Water5-67
35
ANGSTROM
O     -3.355865355250    2.245917157102    2.059793484858
H     -3.595187158310    1.330157119132    2.322778730613
H     -2.982251497343    2.137807921077    1.171037087585
O     -5.293285976888    0.645823484823   -0.134852520184
H     -5.724294542911    1.419307324835    0.257963577830
```

```
H     -4.418294582138    0.948578454456   -0.420092819671
O     -4.358806922513   -0.261862644981    2.225581505551
H     -5.069351076060   -0.078944813163    2.863177209008
H     -4.788547255220   -0.134621371385    1.350345678689
O     -5.867459812073    2.863116448341    1.547136572042
H     -4.901330058925    2.751103123354    1.760660907744
H     -6.016525654741    3.800732453220    1.426675767777
O     -6.363440527947    1.040104045017    3.596200663946
H     -6.395989158926    1.666955023892    2.852585520755
H     -6.093667819845    1.547201496172    4.363472301418
C      0.136928124872    1.632881881834    0.866277545756
C      1.251726397147    1.543242882774    0.084667360140
C      1.984368636884    0.336236491776   -0.196337730611
C      1.616101664851   -0.918767372456    0.260769874405
C     -0.198472830220   -1.378932290878   -0.840453335368
C     -1.222712506597   -0.627943738731   -0.253118942103
C     -1.583726592277    0.703608523378   -0.768984024514
O     -2.583157007817    1.360103318104   -0.440003918493
H      1.562973108569    2.429182477277   -0.471927679291
H     -0.397237074451    2.567257911281    0.977369860859
H      2.744498200329    0.399970622254   -0.974440036611
H      2.203207444892   -1.784697316819   -0.023876654563
H      1.089862708690   -1.036284501438    1.200814555841
H      0.171307645361   -1.097160617099   -1.815802819179
H     -0.125996784115   -2.438051825456   -0.623933579144
H     -0.157128067274    0.863172344730    1.565810959352
H     -0.995071960231    1.043355378897   -1.647772675801
H     -2.668991932537   -0.591688561388    1.252936298001
N     -1.859967539745   -1.070893114582    0.883713499210
H     -1.758358458043   -2.025110870459    1.182547521533

Ambimodal TS Water5-68
35
ANGSTROM
O      1.487607898638    1.166710204011    3.900259401653
H      0.857169954958    0.414149653424    3.879472111334
H      2.074166674092    1.052604099911    3.151190450414
O     -0.472382821720   -0.675851214243    3.576506830945
H     -1.168986354231   -0.098712467836    3.975601368837
H     -0.831870423964   -0.913336649609    2.712720256814
O     -3.896780231121    0.947860550777    1.858810910219
H     -3.458902709044    1.280565804933    1.041023229398
H     -4.768217370534    1.344090991063    1.881133370715
O     -0.433947330877    3.095572762201    3.867152426013
H      0.316964123666    2.465625890718    3.864179326327
H     -0.349303453818    3.597611356969    4.678986773638
O     -2.329802975982    1.125505329691    4.176551132341
H     -2.925774818584    1.091811203975    3.411388353350
H     -1.768250581210    1.913689717699    4.046843418395
C      0.173152411671    1.668833852590    0.853968642035
C      1.287173781253    1.551601063213    0.069682333535
C      2.000726001173    0.331720614235   -0.183949669910
C      1.601294581914   -0.916692626536    0.291167403209
C     -0.172640676560   -1.341169454109   -0.762192350541
C     -1.202103008583   -0.571198842969   -0.200980493075
C     -1.568418034328    0.751884032907   -0.727591053440
```

```
O     -2.557128807792    1.419757319956   -0.398647909602
H      1.606334211745    2.420195899552   -0.508860385767
H     -0.356314185310    2.607075919028    0.945759801756
H      2.760853807415    0.364301151533   -0.964132661932
H      2.184878307465   -1.790269901196    0.020023728425
H      1.109850716098   -1.011269856749    1.253392582085
H      0.184955937250   -1.088627164152   -1.751252945914
H     -0.132156292071   -2.401891107090   -0.538548786988
H     -0.112961601290    0.925143728000    1.581871560849
H     -0.987953496320    1.069989520060   -1.620530340458
H     -2.662309271580   -0.505277761805    1.266830265402
N     -1.842983693078   -1.016369905535    0.959844608474
H     -1.891076189993   -2.009829154522    1.124404762454

Ambimodal TS Water5-69
35
ANGSTROM
O     -3.535148568895    2.662999280925    1.709177516904
H     -3.602679699158    1.887250152874    2.281177436562
H     -3.122346817729    2.334843744459    0.894835367572
O     -4.016605353928   -0.046567843129    2.316804831835
H     -4.299926875148   -0.892865405516    2.668748890049
H     -4.564342349375    0.091972641608    1.514777558631
O     -5.138778198611    0.308727914176   -0.143396876300
H     -4.275850415464    0.643700327473   -0.442889695696
H     -5.661731605683    1.111477204333    0.057484627849
O     -6.018145264731    2.732796105389    0.664234117107
H     -5.166506014276    2.833398512564    1.149187405749
H     -6.085681375671    3.492308915949    0.084720952170
O     -4.866609706619   -2.368324793986    0.686325455107
H     -5.541966772126   -2.951712184782    0.339092517988
H     -5.015258281433   -1.507048482217    0.264934467391
C      0.062493184011    1.604583996786    0.914500677674
C      1.208922026169    1.571815112025    0.175575650722
C      1.983184145600    0.394886012935   -0.128284469432
C      1.639209403878   -0.886080404373    0.264068155716
C     -0.135865158704   -1.363329150198   -0.937364429488
C     -1.185176987998   -0.646834834773   -0.353769797914
C     -1.544767953680    0.699517257529   -0.831585264107
O     -2.588467414992    1.305786812343   -0.538287832769
H      1.520052363627    2.487491697238   -0.330893576566
H     -0.496132519333    2.522257192846    1.044222756583
H      2.769624876996    0.513026542227   -0.873316758429
H      2.258043585576   -1.724180841026   -0.035316196393
H      1.072867540108   -1.059778555112    1.171280061376
H      0.265719071452   -1.040337865812   -1.886943279962
H     -0.047919711454   -2.426862427166   -0.751225683834
H     -0.240795963614    0.798548491496    1.567869235155
H     -0.918226306133    1.097951858670   -1.656702130843
H     -2.616179735682   -0.605588956177    1.169261096440
N     -1.864248576126   -1.131851716099    0.733990529261
H     -1.803449214847   -2.100192871079    0.993262614733

Ambimodal TS Water5-70
35
ANGSTROM
```

```
O     -3.229210143570    1.740722684225    2.243493432508
H     -3.837485611768    0.977706273203    2.313053327844
H     -2.913264020865    1.703207761072    1.318520467760
O     -4.810067147319   -0.398013972727    1.793427757558
H     -5.598811176120   -0.710638933423    2.236088749227
H     -5.117809930478    0.069445598121    0.972669673743
O     -5.388477273770    1.162946763354   -0.259738094892
H     -4.474873393706    1.263721884200   -0.564880742120
H     -5.575241684479    1.979148845154    0.241934814471
O     -5.276866178712    3.354204602348    1.407260137661
H     -4.537640665330    2.912588548852    1.872429534657
H     -4.921081618434    4.165585333010    1.042776278070
O     -1.150015341059    0.337827048007    3.435396694438
H     -1.865762831248    0.905483186525    3.092972328229
H     -1.102007791459    0.510414471783    4.376061386555
C      0.121514847922    1.769229735177    0.693481959343
C      1.263176401668    1.593955884861   -0.032986116537
C      2.001210150673    0.360667363191   -0.134689605649
C      1.597535490552   -0.827334807724    0.443596483217
C     -0.178104259805   -1.363857912589   -0.767953994190
C     -1.240480599297   -0.629709018343   -0.238794187561
C     -1.606661550504    0.697213892567   -0.765436151856
O     -2.598421547496    1.357696078937   -0.402696720639
H      1.599050118253    2.405309255173   -0.682570558002
H     -0.409332912247    2.712719776346    0.678403170095
H      2.804843086384    0.334289373039   -0.871056307332
H      2.180795904383   -1.728114442157    0.289637624460
H      0.994474214660   -0.836927047950    1.343237166397
H      0.248922167574   -1.079093465218   -1.717860284425
H     -0.079295787980   -2.413634095596   -0.523677407837
H     -0.179254928928    1.088926451344    1.477727859991
H     -1.066435788873    1.018257481973   -1.679352091909
H     -2.766631446120   -0.604624777879    1.183723529202
N     -1.963770539324   -1.096383282183    0.828070212298
H     -1.729346959086   -1.957129280957    1.286659266331

Ambimodal TS Water5-71
35
ANGSTROM
O     -3.215953248112    2.285647694324    2.250195832567
H     -3.740225735665    1.460039339902    2.227745738402
H     -2.746007657475    2.269477008680    1.406524273610
O     -4.564036539967    0.171855372305    1.231576186051
H     -5.482272448557    0.518778962696    1.217599405607
H     -4.166722656834    0.532080885850    0.424407221016
O     -4.989667103812    2.858842490253   -0.941287139318
H     -5.032023794949    3.474078706378   -0.194940435371
H     -4.111705947639    2.459545236521   -0.904882846376
O     -5.232600935366    3.950033496284    1.681800037374
H     -4.475097101048    3.394612427294    1.984474497979
H     -5.214785245830    4.746941800873    2.211175796052
O     -6.760140237581    1.668298732608    0.840645360723
H     -6.424229354067    1.931337965003   -0.033000726887
H     -6.581871085729    2.439038574045    1.395103575572
C      0.176121896647    1.617110511738    0.872374635503
C      1.290602858595    1.512096651660    0.091980691337
```

```
C      1.998757562339    0.293611825572   -0.200370629056
C      1.603768379177   -0.958797966944    0.241037870552
C     -0.209360734871   -1.357314705737   -0.881172694832
C     -1.226588531543   -0.611941365863   -0.277306773870
C     -1.577265057969    0.735762020175   -0.751553563764
O     -2.567977048148    1.389623833168   -0.384738535873
H      1.621787103010    2.397462558098   -0.453721375940
H     -0.331752365551    2.565159781449    0.992981019823
H      2.764635821717    0.351070361169   -0.973304036245
H      2.174769996964   -1.832966408689   -0.051046171667
H      1.068735216204   -1.076947846121    1.176100309484
H      0.168969535372   -1.051592107016   -1.846015764249
H     -0.149221984705   -2.422799752233   -0.694370408750
H     -0.136937033449    0.848746982099    1.565214041828
H     -1.001189788650    1.097574711310   -1.628215888463
H     -2.711852175591   -0.622755155887    1.175116109524
N     -1.866661017476   -1.076085672393    0.857023781674
H     -1.789407197073   -2.047505883333    1.106304716501

Ambimodal TS Water5-72
35
ANGSTROM
O     -3.684344582364   -1.644822509147   -1.896964457902
H     -4.136894610592   -1.812008614518   -1.033469564436
H     -3.970410926077   -2.344656009415   -2.485350419459
O     -5.062701837876   -1.897110813089    0.373507188290
H     -4.711270418836   -1.162410592363    0.917117102705
H     -5.936188552040   -1.579827690521    0.116399549375
O     -6.646824237221    0.444317549322   -0.188951407250
H     -7.407304297501    1.000748073663   -0.355872545256
H     -6.017274290382    0.609884437787   -0.935218331591
O     -4.792747257379    0.853318146141   -2.057081696341
H     -4.403346897097   -0.036028144841   -2.167396503127
H     -4.104390887548    1.355054588670   -1.598024898155
O     -4.382392051888    0.510474375000    1.452496654555
H     -5.277340939656    0.700536450310    1.131619276241
H     -3.786010055470    0.963869587847    0.823664980461
C      0.165930754650    1.644304688341    0.831839456328
C      1.270213212025    1.541075197432    0.037025487795
C      2.003334568322    0.330600304983   -0.227461798349
C      1.642702055686   -0.916292988604    0.257415026533
C     -0.184807528713   -1.398655024262   -0.807716224167
C     -1.197078271348   -0.636425470255   -0.218927365832
C     -1.575373046839    0.679362295994   -0.756579358638
O     -2.580106901450    1.333977085553   -0.435907109082
H      1.569892414510    2.415292960391   -0.543884926300
H     -0.375818001491    2.576219036812    0.923766686478
H      2.753531254177    0.380800737128   -1.016105916550
H      2.228260291600   -1.786124972138   -0.018540650043
H      1.129192493416   -1.017482424543    1.206283934066
H      0.166093481664   -1.136964911370   -1.795370776304
H     -0.112017735095   -2.453939030017   -0.573314929477
H     -0.115158817030    0.889101371635    1.552002250602
H     -1.000229835941    1.006190785831   -1.647995161409
H     -2.607458128257   -0.543001198748    1.292071626273
N     -1.809745217369   -1.054383392108    0.945746952743
```

```
H     -1.771752042556   -2.023212917529    1.211684902044

Ambimodal TS Water5-73
35
ANGSTROM
O     -3.143228249530   -3.529091389955    1.357834522207
H     -3.798029144042   -2.976604078827    1.854173483631
H     -3.388096864069   -4.439550742863    1.528020633448
O     -5.065071971054   -1.962072683478    2.340453139671
H     -4.977098687172   -1.153696932697    1.814418316090
H     -5.791173712110   -2.457076473647    1.922964917987
O     -6.656832930372   -3.291194524306    0.526362119391
H     -7.473490775718   -3.196440729509    0.036501965115
H     -5.928040189482   -3.077082921984   -0.100335347640
O     -4.490488172311   -2.438830826077   -0.828127408986
H     -3.856848154470   -2.874023198580   -0.231415313277
H     -4.548671369376   -1.520569069825   -0.516844284891
O     -4.747689531938    0.089139841656    0.395412910172
H     -5.509000845803    0.636181249098    0.197750872910
H     -3.959589514987    0.603334946402    0.089664188644
C      0.156500830137    1.632786657076    0.857848078426
C      1.281381679529    1.550671369963    0.090806313262
C      2.026746650782    0.348651634396   -0.181159333738
C      1.661179495356   -0.910575995792    0.266341291831
C     -0.130371858892   -1.387503919375   -0.858798988697
C     -1.160469599194   -0.631596422767   -0.293265761007
C     -1.539338694427    0.690672937391   -0.819742198521
O     -2.574795591803    1.304012456739   -0.517044076861
H      1.593948475998    2.439075346887   -0.461260619479
H     -0.391859665794    2.560235117135    0.955713852534
H      2.798651444114    0.420983400302   -0.946924926823
H      2.259976583619   -1.770449002213   -0.012133656044
H      1.119825722931   -1.036863225460    1.196456251753
H      0.249885107764   -1.117215955663   -1.833716943094
H     -0.063968703701   -2.443199618839   -0.625752807228
H     -0.141969953911    0.857478777807    1.549081750372
H     -0.931137402082    1.066122545540   -1.667964812177
H     -2.592336788690   -0.519139102046    1.178398272549
N     -1.806656208053   -1.056427908320    0.849383705200
H     -1.827580037529   -2.033680966806    1.099052966058

Ambimodal TS Water5-74
35
ANGSTROM
O     -4.211952001589   -1.638878328237   -1.403026783422
H     -4.441019080850   -1.225812552738   -0.549878683061
H     -4.774282832292   -2.409163239412   -1.493032015129
O     -0.981146907794    0.083447393115    3.506300443837
H     -0.772152657510    0.327796407607    4.408492518860
H     -1.750491630285    0.623164128077    3.253653918168
O     -4.929962237921    0.195084764013    0.557049128911
H     -5.613798525160    0.544222913196   -0.025321461444
H     -4.106225188117    0.603146420832    0.221860516585
O     -4.966985729362    1.003709755345   -2.176876335190
H     -4.691299008342    0.076354386756   -2.222984121024
H     -4.185709701796    1.490992863705   -1.896400593947
```

```
O     -3.141178495161    1.469643772613    2.422063304244
H     -4.026960529118    1.103868400181    2.310717103524
H     -2.871495271585    1.756268760574    1.536526552459
C      0.158912093244    1.709653763469    0.707698960733
C      1.257417020743    1.532376435725   -0.080930795711
C      1.989697665866    0.298346469006   -0.225688300233
C      1.632853651838   -0.887363519786    0.383165057702
C     -0.222704698546   -1.450785126435   -0.709976790961
C     -1.232530389403   -0.669011802181   -0.153473418437
C     -1.610203806854    0.629996901479   -0.727733349824
O     -2.600459124103    1.305419261039   -0.378994725369
H      1.553952804583    2.342889525494   -0.749860775025
H     -0.373491326964    2.652414848232    0.719205000595
H      2.745033593682    0.272958393069   -1.011252166133
H      2.207264780996   -1.786966797320    0.194863689561
H      1.085974086830   -0.897436436053    1.318256571527
H      0.147914876509   -1.226285147460   -1.699322456912
H     -0.124204456385   -2.487163926668   -0.413814388131
H     -0.102855113086    1.029698587542    1.506841783875
H     -1.077726241692    0.924698651240   -1.653229092265
H     -2.474864362992   -0.415038867657    1.478476694624
N     -1.929933525982   -1.085949377157    0.959715232455
H     -1.585286487802   -1.855078071713    1.507640368917

Ambimodal TS Water5-75
35
ANGSTROM
O     -3.721308538603    0.005228019930    2.624794238379
H     -3.522960466791    0.943880299423    2.892680262735
H     -4.025486700818   -0.436084498790    3.417936539439
O     -3.405532956948    2.535114696589    3.274229130494
H     -2.915721995228    2.964946944208    2.541116050109
H     -4.299776273396    2.884001173125    3.170521857763
O     -5.512096629769    3.432436801114    1.551059550370
H     -6.338416645950    3.810013834408    1.250577828115
H     -5.455709229083    2.532589270540    1.141147663049
O     -5.156968584133    1.010064226833    0.499175391655
H     -4.832598144788    0.510742046025    1.267768183002
H     -4.362258294913    1.149379630967   -0.041309254532
O     -2.771789176335    3.739236329937    0.942985702005
H     -3.717825242442    3.943299920991    0.993952610096
H     -2.703641884401    3.011011321159    0.305386187015
C     -0.020684771925    1.490976739927    1.057929121637
C      1.158358224459    1.474059027135    0.370107505302
C      1.909972364020    0.298944143379    0.016485456673
C      1.497803512433   -0.996376170872    0.290083334335
C     -0.210331755240   -1.305161033803   -1.004647175183
C     -1.277681526511   -0.594209910675   -0.442235208542
C     -1.577646641412    0.789949249586   -0.839478827956
O     -2.608097834089    1.412868968739   -0.544228452044
H      1.516739634877    2.413670847975   -0.053543529151
H     -0.558832055023    2.414422066266    1.223234949394
H      2.733943004497    0.440288271225   -0.682174918301
H      2.105072987723   -1.831751623196   -0.040099304712
H      0.898810775732   -1.213586824548    1.167107179659
H      0.247093325881   -0.939311268026   -1.912947398905
```

```
H     -0.175502803585   -2.382459260475   -0.891354194477
H     -0.374153953245    0.652741699796    1.642367226464
H     -0.893534755659    1.225801678593   -1.598179127776
H     -2.708097096300   -0.597229172595    1.078741190177
N     -2.083391694228   -1.162105942025    0.517422019208
H     -1.883652015969   -2.081116903918    0.871245536682

Ambimodal TS Water5-76
35
ANGSTROM
O     -3.841034306307   -2.412752572834   -1.237543549348
H     -3.661102073537   -2.859417500213   -0.387921317915
H     -4.365754004614   -3.018268870568   -1.762808398858
O     -3.554811993101   -3.487043707767    1.280084684143
H     -3.978734612448   -4.265424881290    1.643526859669
H     -3.962702193755   -2.711771257435    1.729284025061
O     -4.713683665588   -1.280766058723    2.268312400564
H     -5.537558686885   -1.189153027720    2.747320659140
H     -4.837493058129   -0.787522484261    1.412554815249
O     -4.887703417829   -0.128913072926   -0.088354722206
H     -4.589690027482   -0.872402017626   -0.643330906371
H     -4.178985898230    0.535562747554   -0.158642605605
O     -2.395457453940    3.997686815641    0.372249649783
H     -2.497784793701    3.081991860718    0.066421559292
H     -3.268737448704    4.388692177679    0.319691860042
C      0.179419240888    1.621319051095    0.889780628121
C      1.289272381313    1.538433100695    0.099317792539
C      2.022762540670    0.336044578126   -0.200568851855
C      1.658815117361   -0.927017084277    0.237596697265
C     -0.157307408035   -1.374362325397   -0.865694253365
C     -1.170871900752   -0.627068568821   -0.261897313052
C     -1.546142032144    0.712488107078   -0.733867627893
O     -2.571311622787    1.319000955698   -0.372899493837
H      1.594013664644    2.430945101344   -0.449877288295
H     -0.357108581275    2.553719784736    1.005874579866
H      2.781300634226    0.412389186709   -0.979595379451
H      2.247379264951   -1.787157071647   -0.061707666494
H      1.132484590688   -1.061321423617    1.175172952507
H      0.205433953936   -1.081414664620   -1.840550467672
H     -0.091191657146   -2.436178706305   -0.662129255318
H     -0.106587152352    0.841761117836    1.582749471844
H     -0.969926472786    1.109808660996   -1.593596628995
H     -2.580590488734   -0.537358055467    1.230865792181
N     -1.798681007101   -1.071449561430    0.884757775841
H     -1.820511065195   -2.052141334318    1.109412590259

Ambimodal TS Water5-77
35
ANGSTROM
O     -3.638556220885    0.333008494159    2.508421540306
H     -3.348786323596    1.246240450552    2.295735629475
H     -4.361564943986    0.461410475048    3.146544981517
O     -5.741945936453    1.496499614848    3.835476531149
H     -5.985434999087    1.931415064713    2.995570131951
H     -5.424655334894    2.192954243779    4.412497554865
O     -6.132639589846    2.631851612303    1.351658436009
```

```
H     -6.993353223523    2.874524012529    1.010351613503
H     -5.852006122195    1.817599733147    0.850453377283
O     -5.147385105569    0.465994612091    0.223951871578
H     -4.752342117756    0.155738538479    1.061972487858
H     -4.382552251281    0.764562736024   -0.295045455854
O     -3.307066388490    2.880028330476    1.599929970492
H     -4.250402153289    3.086363733567    1.548265881247
H     -3.065518979090    2.557346396791    0.718586866043
C     -0.091368829968    1.498580351193    1.059060883342
C      1.087074246418    1.509477081913    0.371421319975
C      1.871566188561    0.350295502625    0.033777033493
C      1.499460501485   -0.950780469969    0.332731825851
C     -0.206937839752   -1.339677119088   -0.949987696302
C     -1.288398903122   -0.630087110286   -0.412966505310
C     -1.606005419144    0.733169213125   -0.868407655589
O     -2.650037894953    1.354480296452   -0.623400825269
H      1.419612073249    2.453024691417   -0.064869410374
H     -0.661200920739    2.403728533725    1.219757020661
H      2.688782632644    0.504409321423   -0.670064627298
H      2.129146622614   -1.773957902604    0.014434378095
H      0.908640240659   -1.169797797060    1.214720397138
H      0.245559683106   -0.998003358220   -1.870199361601
H     -0.150502465792   -2.411137624286   -0.797041628629
H     -0.419869841065    0.655025830181    1.650001197002
H     -0.909333630438    1.150129957844   -1.626920169191
H     -2.694232839002   -0.607819172508    1.130474716789
N     -2.092022259046   -1.185068298487    0.557014610168
H     -1.863866637148   -2.084380340115    0.944263703088

Ambimodal TS Water5-78
35
ANGSTROM
O     -2.852279378264    2.941440788055    3.465857699917
H     -2.330719100143    3.490324562299    2.831871207592
H     -2.354872862418    2.924143952412    4.283396387167
O     -1.588058684117    4.485102608157    1.693061387111
H     -2.235133350356    4.345675649821    0.944479382377
H     -1.595946771516    5.427371183735    1.865833606622
O     -5.206790294454    3.269137500095    1.855144977680
H     -5.223500923625    2.307535997973    1.753235818908
H     -4.620692038699    3.421124388268    2.607646189094
O     -3.793400924055    0.802340381035    1.906153130001
H     -3.406586040531    1.413552573205    2.553513636292
H     -3.492654650502    1.127486476709    1.034201201269
O     -3.431571983082    4.006987085977   -0.102938695565
H     -4.196839777252    3.845356307233    0.485891641639
H     -3.170819836264    3.133631680537   -0.434674377170
C      0.160945752838    1.618987569883    0.875036543427
C      1.259874500005    1.531283092077    0.069697269584
C      1.982400818519    0.325013490858   -0.238704765665
C      1.623292659152   -0.933336804914    0.217264859261
C     -0.219398947915   -1.385587248652   -0.839827912975
C     -1.222725189127   -0.618078816032   -0.235998907122
C     -1.568592132686    0.716087197441   -0.755337940741
O     -2.562724655380    1.386926777641   -0.435442874719
H      1.562014362514    2.422016715091   -0.483604327313
```

```
H     -0.361538594664    2.556654682499    1.009856997427
H      2.723962600931    0.392515448651   -1.034283921623
H      2.201512587686   -1.798369027314   -0.087647641401
H      1.121069422076   -1.057087869465    1.169724768696
H      0.132411405177   -1.115650923833   -1.825068413406
H     -0.155717101660   -2.444496978988   -0.618385592463
H     -0.117944575788    0.840027076332    1.571525444281
H     -0.977794993544    1.043100837994   -1.635960618766
H     -2.612364084122   -0.520334456156    1.324127451728
N     -1.851776406130   -1.053207631600    0.908974251411
H     -1.748440799852   -2.004894361071    1.213642048963

Ambimodal TS Water5-79
35
ANGSTROM
O     -3.795896795691    0.192797888597    2.570590533915
H     -3.545356686407    1.142692410469    2.401432027968
H     -4.014900514262    0.133994198914    3.500166688039
O     -3.186699957482    2.659276613807    1.900260571539
H     -2.972050997123    2.434859910756    0.982918145217
H     -3.992709581526    3.213708030539    1.846811160853
O     -5.538643335005    3.984355151452    1.662294720147
H     -6.072429720255    3.358439611413    1.121564445356
H     -6.055977838112    4.161257504911    2.448655659740
O     -5.087120876812    0.257122929949    0.129569988040
H     -4.856123859208    0.062248831709    1.051725202633
H     -4.288576797922    0.677068328573   -0.232727206438
O     -6.924582312495    2.182675378835    0.238669994295
H     -7.099422625489    2.420803809282   -0.672687424210
H     -6.302917323949    1.414927960016    0.201788696600
C      0.046278271829    1.502240696665    1.016469330408
C      1.186919858040    1.486903174531    0.267922895984
C      1.933778725434    0.311568322564   -0.097741652374
C      1.556746961805   -0.980205445517    0.234143236085
C     -0.222131074513   -1.354529345183   -0.950636338806
C     -1.263767981856   -0.627094649042   -0.361562358485
C     -1.588884312645    0.739039597948   -0.805887389112
O     -2.611152874343    1.370606639259   -0.503144203629
H      1.511698589922    2.420622564259   -0.194492423463
H     -0.500020523935    2.417473811593    1.198774092904
H      2.715227221748    0.444911325866   -0.845072193293
H      2.155637086878   -1.816768447006   -0.107896634092
H      1.009429032302   -1.180637133337    1.147955147455
H      0.184884248900   -1.022310226481   -1.894914504261
H     -0.172537425126   -2.426117347664   -0.796575579903
H     -0.265102285304    0.668949441285    1.630462422438
H     -0.933948985126    1.143637288474   -1.606507059890
H     -2.660806901324   -0.593126255902    1.196533044649
N     -2.001398462214   -1.151677363603    0.671145187963
H     -1.830355530642   -2.081417997747    1.009425412056

Ambimodal TS Water5-80
35
ANGSTROM
O     -3.636992724955    0.027303286465    2.792473595939
H     -3.182893014930    0.864621428387    3.055257746473
```

```
H     -4.177562534247   -0.220729228686    3.543140243040
O     -2.444070694457    2.300977008649    3.460810610590
H     -2.652839708606    2.873443356085    2.675588806076
H     -1.505915328477    2.405375741553    3.622366541983
O     -5.651630192190    3.461563286107    1.130622078649
H     -6.134503114439    3.811915616948    0.381442434498
H     -5.570826952668    2.491424004105    0.974720766811
O     -5.082791297889    0.893925247711    0.603747562506
H     -4.725253447627    0.534129042234    1.434355048740
H     -4.296170664203    1.061518402788    0.060658154894
O     -2.938221951196    3.629615360621    1.233018666225
H     -3.908680253292    3.750985506792    1.265340773655
H     -2.795730396237    2.959664891636    0.547080698862
C      0.103052581010    1.526503539482    0.982014785303
C      1.255438170376    1.456396098335    0.254162008841
C      1.966242860162    0.251829222906   -0.083908068168
C      1.533125255232   -1.023205931088    0.246475774365
C     -0.227465000662   -1.333144410148   -0.978597382159
C     -1.260238463792   -0.596597148653   -0.386142828651
C     -1.560849682882    0.785405137609   -0.799865692287
O     -2.563276769199    1.429219686785   -0.461612652745
H      1.620300045397    2.371799042438   -0.214693061718
H     -0.409864259780    2.468380552034    1.124602056944
H      2.767976361718    0.350314944842   -0.814760948035
H      2.108910871831   -1.882952901174   -0.077116547745
H      0.959357573210   -1.198612179034    1.149371088693
H      0.198142523258   -0.989753950258   -1.910683190551
H     -0.201539118055   -2.408342318713   -0.846106326873
H     -0.251100388664    0.716291354709    1.604489435860
H     -0.913413936141    1.191909850375   -1.605589151010
H     -2.664962502203   -0.563057087744    1.162411203839
N     -2.015082840900   -1.125159729871    0.631481053326
H     -1.854729953816   -2.057660516225    0.967679458006

Ambimodal TS Water5-81
35
ANGSTROM
O     -2.264025890685    2.267452187854    3.304310352744
H     -2.221926129478    2.910283055856    2.570708076017
H     -2.861127545113    2.680807935618    3.951644461687
O     -4.303949346632    3.785859042766    4.380520002484
H     -4.819769730926    3.540993295346    3.588717786430
H     -4.068870524041    4.708653705233    4.270629287403
O     -5.474993869739    2.975350695060    2.021714866725
H     -6.412511283417    2.790225007836    1.967216439448
H     -5.011768236493    2.099549899573    1.959207952129
O     -3.925928700762    0.828682405015    1.810133619835
H     -3.293117650762    1.210491076048    2.469092856213
H     -3.564844614872    1.100209425631    0.940413866838
O     -2.842071891593    3.798674484524    1.082744241612
H     -3.790825951616    3.801277076661    1.263401866023
H     -2.716902346991    3.144566053818    0.379621934546
C      0.173001631430    1.636092576592    0.843468491407
C      1.297417394054    1.496270924972    0.081567744926
C      1.993752764638    0.263001232046   -0.175787859666
C      1.574298805357   -0.974603393652    0.280886152571
```

```
C     -0.239011903131   -1.360846142014   -0.876393785696
C     -1.247182035333   -0.595065503541   -0.282342298174
C     -1.556418805004    0.762046618010   -0.763884119525
O     -2.537277485290    1.449144783478   -0.428562216329
H      1.646935833351    2.363969694671   -0.481358158944
H     -0.321193869982    2.594334743774    0.932287367484
H      2.773025164268    0.295091062799   -0.937133675941
H      2.133674447207   -1.863847865935    0.012905742688
H      1.017216383723   -1.067859127144    1.205587780503
H      0.158272379582   -1.064693407661   -1.836243339898
H     -0.195620123175   -2.425919275837   -0.683462754038
H     -0.155374344682    0.896433890150    1.560113600171
H     -0.959226901904    1.096736665536   -1.636834584861
H     -2.686066910313   -0.539688271238    1.236541972346
N     -1.921775573986   -1.060869714361    0.823454579637
H     -1.803085118690   -2.009144298272    1.131867964085

Ambimodal TS Water5-82
35
ANGSTROM
O     -3.421685005310    2.000610191800    2.186331045921
H     -3.697533223434    1.115009540874    2.470237097376
H     -3.026667925805    1.881388664876    1.306096024995
O     -5.232721572849    0.501588554603   -0.085202016320
H     -5.656132841575    1.312588220730    0.260240568204
H     -4.353647214317    0.788139291382   -0.379326690997
O     -3.513792963609   -3.100006960666    1.982971444382
H     -3.920821059835   -2.236895549257    2.171396794606
H     -4.220161775395   -3.669920662667    1.676024028916
O     -5.829725892781    2.788456206645    1.267250673656
H     -4.971802823922    2.641083719491    1.726923979303
H     -5.777633130045    3.661372641647    0.876903688142
O     -4.650442354629   -0.547522561844    2.246482529743
H     -4.931553081721   -0.255941814956    1.333042763535
H     -5.429637754304   -0.460740686615    2.797792111884
C      0.171118004421    1.651837532125    0.826174029279
C      1.279806762297    1.546220087385    0.038451746112
C      2.011261650114    0.332690085781   -0.224760029310
C      1.653619779242   -0.912117940045    0.259728508215
C     -0.184434194163   -1.394886399604   -0.840402597009
C     -1.191657334623   -0.631685185950   -0.248841523566
C     -1.565185932010    0.699975473406   -0.755379328403
O     -2.573014133800    1.330239298253   -0.388644157867
H      1.587387456947    2.420612367793   -0.538341527864
H     -0.360918066283    2.588961953919    0.925348931704
H      2.767904232991    0.385109704632   -1.007481071317
H      2.236333350248   -1.783588554332   -0.015851169328
H      1.119227076732   -1.018493905052    1.195972994769
H      0.185434975170   -1.122050230439   -1.818263739431
H     -0.108462197588   -2.448893663962   -0.604422836488
H     -0.116057037319    0.894374345097    1.542089077363
H     -1.011424569602    1.058231129252   -1.646767351364
H     -2.616271180523   -0.505517811440    1.223509106363
N     -1.822919545818   -1.038787855491    0.904677067956
H     -1.838252011045   -2.005115377016    1.192475713703

```

```
Ambimodal TS Water5-83
35
ANGSTROM
O     -4.494100904807   -1.614714092443    2.679644860476
H     -3.904441276322   -2.247922595616    2.233079952896
H     -4.711450153052   -0.938796590049    2.016566038962
O     -4.774618253526    0.333942076236    0.732863231111
H     -5.416423503186    1.038732301112    0.825373253111
H     -4.010445902091    0.728746807537    0.258927773236
O     -3.050916308253   -3.630017323928    1.398085090428
H     -3.380019082078   -4.301970299258    2.037617587879
H     -3.516240056853   -3.812384074701    0.579292187375
O     -6.226636929802   -3.453584822866    3.523159586708
H     -5.676973257211   -2.699248590732    3.201764835759
H     -6.551128776435   -3.194976903452    4.386447910884
O     -4.257815111346   -5.262200467073    3.148915849864
H     -5.066584881812   -4.714714676772    3.259963936499
H     -3.874703275397   -5.355830017656    4.022330755571
C      0.174217347261    1.655723078164    0.811033766458
C      1.287331168306    1.546934118333    0.029434770214
C      2.019381351510    0.334182913117   -0.223037088071
C      1.643860985329   -0.912449931540    0.257865087278
C     -0.153472407987   -1.398332490819   -0.816205365863
C     -1.177213552765   -0.622063547889   -0.261122320359
C     -1.560518323775    0.688093328507   -0.818617196633
O     -2.581393725365    1.317402877408   -0.512284105914
H      1.597733481134    2.419660745917   -0.548336795091
H     -0.367441727549    2.588578954965    0.892468270932
H      2.780065090634    0.379590770985   -1.001875943469
H      2.234075060313   -1.783119716164   -0.006130218565
H      1.124563829622   -1.008779529952    1.204242515423
H      0.211337886522   -1.158782464505   -1.805318043983
H     -0.095314684549   -2.449215703042   -0.558459044251
H     -0.118924686339    0.900557707963    1.526198387607
H     -0.963339852624    1.031812599658   -1.689968576724
H     -2.601790893404   -0.465312661202    1.208845083979
N     -1.815785573705   -1.013595362639    0.896173327579
H     -1.826164576057   -1.981772200448    1.179278071728

Ambimodal TS Water5-84
35
ANGSTROM
O     -4.820380622158    3.021341833581    0.006495959350
H     -4.002236231308    2.633871008886   -0.337093731786
H     -5.014474484919    3.786627671367   -0.535341189946
O     -3.466527152914    1.568269329364    2.173976898478
H     -4.172832405268    2.207937114445    2.043658532579
H     -3.039611919776    1.494687430462    1.306357835619
O     -3.049010115066   -3.215771522238    2.243100328120
H     -3.645268000952   -2.445483442092    2.330061454754
H     -3.486926383401   -3.936263537897    2.697042502870
O     -5.194264293778    0.147323926667   -0.163234624629
H     -4.288378982988    0.373619909313   -0.417872704046
H     -5.619782216884    0.996683973004   -0.001391476601
O     -4.620546133788   -0.962159785659    2.303080703826
H     -5.013769550903   -0.779141793084    1.431363323904
```

```
H     -4.219765334641   -0.120095618445    2.575466359883
C      0.039798908702    1.611126425926    0.861214602986
C      1.202030158014    1.543959434602    0.153439388526
C      1.972501043887    0.346979684424   -0.097771707260
C      1.619663747354   -0.912630038935    0.326677406584
C     -0.177866137959   -1.387899478236   -0.977724559315
C     -1.221075802322   -0.673577023978   -0.395368232872
C     -1.565389602730    0.685032880101   -0.849537646534
O     -2.602456578883    1.295859176518   -0.513480498956
H      1.539241824574    2.440495365448   -0.370961175413
H     -0.502789407674    2.542635444031    0.959782489062
H      2.784225254422    0.443275969337   -0.819133600338
H      2.227172515046   -1.768625875469    0.058972001939
H      0.990605355154   -1.067725950656    1.193958155998
H      0.267437825783   -1.043650755089   -1.899049153765
H     -0.077752812178   -2.447459533499   -0.785135250008
H     -0.292438836163    0.826619221977    1.526999185847
H     -0.968201795637    1.092314922800   -1.688558913207
H     -2.704273946293   -0.642289696148    1.048809830258
N     -1.923499188665   -1.156399057214    0.674006706760
H     -1.862033466847   -2.110756455629    0.991638918865

Ambimodal TS Water5-85
35
ANGSTROM
O     -3.684250218872    0.207158140081    2.488525475189
H     -3.568307611627    1.163681420081    2.306492620866
H     -3.217112779701    0.072941214783    3.318130533431
O     -1.695111993621    1.615805302936    4.005015406117
H     -2.075363568165    2.179267050310    3.312408563037
H     -1.440086213119    2.203077014772    4.716873686201
O     -5.672173316091    3.229499426869    1.116663916564
H     -5.914851241610    3.806471654850    0.391738550882
H     -5.664334177685    2.316035050443    0.748663132091
O     -5.189804995188    0.761700744624    0.148933312657
H     -5.035166283539    0.265387104924    0.961524232302
H     -4.303669716141    0.966672158226   -0.193992434358
O     -3.110856574758    2.825330509752    1.877646880106
H     -4.025480837462    3.149632403797    1.748301377550
H     -2.826884193941    2.525366806898    0.999255818887
C      0.143405708274    1.535235287269    0.960532477130
C      1.284578264450    1.447530973110    0.218213159060
C      1.980368684392    0.231973523583   -0.118956551700
C      1.545294249131   -1.033299731309    0.231602737402
C     -0.248104477385   -1.331407698154   -0.999017615440
C     -1.262761667733   -0.588552414564   -0.387096649725
C     -1.545829941767    0.799217221241   -0.790979763399
O     -2.539379138985    1.458018131758   -0.443332774304
H      1.653703453483    2.353917024089   -0.265898844035
H     -0.349736027965    2.486038796224    1.113332857382
H      2.775511862222    0.318189015168   -0.859082035366
H      2.104940957251   -1.903603021590   -0.091470996703
H      0.965194216862   -1.193831478000    1.132578917167
H      0.179687846327   -0.983726699201   -1.928106998125
H     -0.222525954094   -2.406494874759   -0.868372737520
H     -0.209731903897    0.741321868197    1.603228060447
```

```
H     -0.906532775403    1.198122941235   -1.606168638381
H     -2.654756680014   -0.544270493332    1.186060721328
N     -2.016177800547   -1.113232958506    0.631429607642
H     -1.852551644266   -2.048356669585    0.959318955721

Ambimodal TS Water5-86
35
ANGSTROM
O     -3.343383983495    0.523446130189    2.558309715487
H     -2.809220691261    1.325456755851    2.654276541141
H     -4.026170381579    0.617675900443    3.244061510332
O     -2.756356902916    3.229333663142    3.061077228128
H     -3.031152625530    3.515845185065    2.149121949869
H     -2.315638760175    3.978954983670    3.460571514219
O     -5.191890447147    2.036967904987    3.881502894058
H     -5.735307654604    2.146186404334    3.095625913768
H     -4.426882581664    2.622666982775    3.761221796605
O     -5.230952696956    1.607809656154    0.813436199607
H     -4.556984870259    1.154851089943    1.357706670727
H     -5.013066670342    1.361699323341   -0.088640854510
O     -3.576138375775    3.761890887682    0.616185269600
H     -4.409484158165    3.260243416786    0.695442320361
H     -3.033795316391    3.181345161300    0.058956001271
C      0.004231316713    1.452658247867    1.100685297436
C      1.181761145956    1.434483891685    0.410625654556
C      1.910042206080    0.255233782366    0.029208780178
C      1.467211379388   -1.039080530638    0.272912509349
C     -0.232901255268   -1.296328762382   -1.002779729059
C     -1.290649206895   -0.567269789676   -0.442043863242
C     -1.573313340306    0.826815310658   -0.829977640039
O     -2.588523271099    1.456230741665   -0.520692009569
H      1.556475833875    2.377685233960    0.008990081214
H     -0.522674754851    2.378566928543    1.287803968562
H      2.733117608565    0.395406942079   -0.670199458828
H      2.061321967865   -1.877005758647   -0.074902184105
H      0.879279929034   -1.262836348464    1.156176098765
H      0.227029164708   -0.937027078715   -1.913086415796
H     -0.228421539073   -2.375585835567   -0.900237537805
H     -0.362647920439    0.606462695030    1.663717854340
H     -0.876061578367    1.260343365318   -1.582001864428
H     -2.705786073834   -0.521353394342    1.072202230165
N     -2.125777974206   -1.123368642792    0.500335649655
H     -1.926653535886   -2.031209224621    0.883900018229

Ambimodal TS Water5-87
35
ANGSTROM
O     -2.022739927429   -3.062808180568    1.802436025581
H     -2.303557163330   -2.263466499071    2.271831954995
H     -2.772087784426   -3.662162108331    1.858989204391
O     -3.436320959030   -0.820387234265    2.823080698740
H     -3.800563772255   -0.194725933747    2.134329010285
H     -3.398283727412   -0.318450967529    3.637702461240
O     -5.594852312423   -1.559150460787   -0.188294888716
H     -5.133211379784   -1.906531487716   -0.953365492096
H     -5.421577026745   -2.176097927396    0.547927970090
```

```
O     -4.998329007369   -2.944119652536    2.116612573402
H     -5.789364795335   -3.008685377722    2.653292626854
H     -4.481413141431   -2.190020027517    2.467020998781
O     -4.508251194002    0.684358148247    0.973039465364
H     -5.103195349983    0.073422703767    0.501921597964
H     -3.935772474048    1.084368847610    0.287907386363
C     -0.673697822476    1.000647561373    1.291986942904
C      0.653865645045    1.122235523284    1.006956267873
C      1.526814492314    0.038961461360    0.639701401543
C      1.102629945015   -1.270314412702    0.462193374412
C     -0.121884772561   -1.193894180789   -1.304857541755
C     -1.340888400806   -0.603571650757   -0.957750442421
C     -1.605510544423    0.838457858150   -1.120492681015
O     -2.700403802399    1.383838766540   -0.946480236742
H      1.077669291046    2.125513809617    0.935128830805
H     -1.293507923869    1.866128513219    1.485163196622
H      2.516522199710    0.315545678487    0.276861606099
H      1.818884764797   -2.018123967472    0.139478461343
H      0.282386666261   -1.681254977818    1.039495058324
H      0.572731221481   -0.643244100938   -1.924177016733
H     -0.077296944541   -2.269540728033   -1.420856550505
H     -1.132379391701    0.051746290845    1.530617220097
H     -0.773050517207    1.429534663856   -1.561918672280
H     -3.154769809157   -0.849976291896   -0.057415480749
N     -2.383343342150   -1.365836085905   -0.456597202220
H     -2.170497215062   -2.231353290271    0.026653453608

Ambimodal TS Water5-88
35
ANGSTROM
O     -3.197378569822    0.043611314603    3.080557340761
H     -2.614842910412    0.725531875599    3.490802857235
H     -3.706903863887   -0.331400802320    3.799999104951
O     -1.625459898132    1.865250370293    4.234053515301
H     -1.738496155420    2.710233701775    3.736255194945
H     -0.683150346117    1.690564154517    4.228103590519
O     -3.627107368821    3.792435711047    0.940497514481
H     -3.180580307255    3.325305068430    0.221305279788
H     -4.281233112323    3.137356661029    1.255902720169
O     -4.891284527977    1.420748732370    1.359494089050
H     -4.380182353467    0.963582409210    2.048113375225
H     -4.389382688410    1.271986433106    0.545908376654
O     -1.813358573411    4.112583828342    2.821281159158
H     -2.491258818274    4.008105764652    2.100996646246
H     -2.075618368758    4.894365567795    3.308819747645
C     -0.020277727191    1.497309966648    1.120626011601
C      1.127460348646    1.507734187997    0.381300362843
C      1.849080090749    0.346647065474   -0.062901221529
C      1.429003373115   -0.959205733822    0.165090819560
C     -0.318898243934   -1.204107226438   -1.030443991796
C     -1.357635092186   -0.493833653102   -0.410485456176
C     -1.670268048189    0.900132071708   -0.770490195344
O     -2.680164173412    1.518898946833   -0.424088739284
H      1.477761671356    2.465547131288   -0.006583751747
H     -0.544457360104    2.413120790441    1.357838896245
H      2.639222613090    0.512693914724   -0.793859518619
```

```
H      2.016947399325   -1.780817413273   -0.229292773395
H      0.887550861216   -1.212429011434    1.070215831279
H      0.092561102990   -0.826379119824   -1.956447977384
H     -0.304210762324   -2.285295643515   -0.949242228500
H     -0.357221365527    0.628231596577    1.668567961139
H     -1.001420520669    1.348737442522   -1.538360119205
H     -2.694376864546   -0.510869200104    1.184357819209
N     -2.137231062899   -1.080690902878    0.562592572129
H     -1.910418300983   -1.997707735963    0.907089587827

Ambimodal TS Water5-89
35
ANGSTROM
O     -4.094873919918    0.031046522673    2.536607582627
H     -4.160001951152    1.008375217905    2.645843639025
H     -4.510658375425   -0.354080998671    3.308106471792
O     -4.361960561662    2.686516799392    2.671445696575
H     -4.663209855437    2.833697835322    1.743470214561
H     -3.577440525661    3.225469405062    2.781764549662
O     -3.886838143862   -2.683275399884   -0.499892693256
H     -4.529045031887   -3.231609779565   -0.951675078089
H     -4.312186605426   -1.809441965059   -0.393658204820
O     -4.899526274529   -0.147443116066   -0.070070405681
H     -4.820780659765   -0.175359384066    0.900687861882
H     -4.106361578727    0.339774996820   -0.352055913639
O     -4.910792954109    2.807345438879    0.054454207065
H     -5.453303141712    2.028737557842   -0.119235673944
H     -4.025984884806    2.567620935597   -0.258790444461
C      0.126726410647    1.609430961824    0.928502172704
C      1.260544658768    1.541410431026    0.174286760518
C      1.996026354915    0.341872738357   -0.139509783916
C      1.618144612893   -0.929561526500    0.252349265498
C     -0.178210471978   -1.350273769990   -0.928115348439
C     -1.195536943359   -0.609655058415   -0.323025674928
C     -1.547618505099    0.736949052512   -0.800006824219
O     -2.588894475700    1.342089537878   -0.496471626412
H      1.593331125622    2.447540704521   -0.335963641747
H     -0.404979268265    2.543278589289    1.056207855602
H      2.778601742493    0.439960015155   -0.891533388658
H      2.210339096337   -1.783572261938   -0.055798574556
H      1.053963345383   -1.090738094923    1.163124835814
H      0.213296768836   -1.034542085848   -1.884912899652
H     -0.119140433561   -2.416171630057   -0.746812576520
H     -0.198212201542    0.811886551292    1.581037023160
H     -0.923002226266    1.141196122650   -1.623879992447
H     -2.600755304746   -0.538776645734    1.194305047066
N     -1.835567392628   -1.074203492775    0.806199880811
H     -1.919487426869   -2.067237453557    0.946913932062

Ambimodal TS Water5-90
35
ANGSTROM
O     -4.609496425616    0.207942406026    0.908516928290
H     -5.506941847479    0.334215430649    0.541534877388
H     -4.006597837010    0.753822279780    0.365329471419
O     -7.168310411054    0.130764685607   -0.003875634988
```

```
H     -7.538371832801   -0.438010022175    0.705892078370
H     -7.695753922342    0.930402795205   -0.001494878838
O     -5.449013837704   -1.785677491077    2.806275219185
H     -5.139164255788   -2.309037889893    2.046272406426
H     -5.107409502939   -0.898872201046    2.637735754594
O     -4.627980391551   -2.422927031201    0.237889002490
H     -4.545530008090   -1.451096907562    0.352763553448
H     -5.345653889184   -2.545440223024   -0.385563511121
O     -7.993000550587   -1.398078029578    2.060308310686
H     -7.107261708809   -1.548003505752    2.468143190149
H     -8.409294240920   -2.258574007849    1.999744479789
C      0.093567080389    1.621237042452    0.851589163484
C      1.232398018706    1.549461515082    0.101936837193
C      1.998180311490    0.358169313228   -0.142395162741
C      1.629343581138   -0.904495773277    0.309405603857
C     -0.109183184424   -1.404507532080   -0.807108686875
C     -1.172458558392   -0.657387420851   -0.282489289133
C     -1.567467171154    0.645214908815   -0.849907747642
O     -2.604594624132    1.261021434351   -0.582393687970
H      1.538084561727    2.436527893359   -0.455909847885
H     -0.475003919569    2.538496937845    0.925135085585
H      2.780406702237    0.427616180373   -0.897538827002
H      2.248695676389   -1.757832954117    0.054371132297
H      1.096653109920   -1.019928014153    1.246642708107
H      0.274147796046   -1.150390504676   -1.785858578485
H     -0.041793502374   -2.459128737252   -0.565214282679
H     -0.200109944211    0.852755537916    1.552084360133
H     -0.943825817695    0.994940599349   -1.701512093750
H     -2.663393431905   -0.571450220443    1.132883408510
N     -1.835745629611   -1.077351244422    0.856552984417
H     -1.828596901080   -2.050052248740    1.113076689499

Ambimodal TS Water5-91
35
ANGSTROM
O     -3.156940980375    2.884302435418    2.981060632129
H     -2.569708273080    3.457090454911    2.436167444243
H     -3.071507010174    3.185220314704    3.886009775281
O     -1.752643730593    4.545261299366    1.419996151224
H     -2.238459800500    4.401704645644    0.564895962259
H     -1.772779484734    5.490063826733    1.576933421077
O     -4.910657732317    2.401427529844    0.782360629181
H     -4.250293187419    1.869828185635    0.314764641668
H     -4.481476297852    2.676961858694    1.603710797119
O     -3.706315453132    0.203710841236    2.385866882892
H     -3.359447315506    1.068743830505    2.658334661991
H     -4.556941953309    0.392588151118    1.977039113849
O     -3.199074216539    4.033920760147   -0.734314835072
H     -4.079746852399    3.865528700621   -0.365279034072
H     -2.857226682393    3.138305527042   -0.912800204700
C      0.016011348856    1.543648178892    0.987448625979
C      1.187001583346    1.507429475191    0.287469442796
C      1.945932327647    0.325376957904   -0.028424063992
C      1.552041876479   -0.960717562436    0.303197918644
C     -0.176020627671   -1.342420777174   -0.965239415007
C     -1.242169224360   -0.627004367077   -0.406238814411
```

```
C     -1.560570766910    0.741661134290   -0.846240866084
O     -2.588839268007    1.369008068485   -0.555835719205
H      1.533676873502    2.432643947117   -0.176234211011
H     -0.522748438190    2.471282379610    1.125248192847
H      2.759415793606    0.447010157263   -0.743053908937
H      2.162338662364   -1.803481611626   -0.001317761382
H      0.960550558368   -1.149029902149    1.191700201194
H      0.266463379910   -0.998717913727   -1.889317165436
H     -0.122684677903   -2.414413201819   -0.816425825846
H     -0.327445009087    0.725045357508    1.604632343010
H     -0.895356413538    1.149708958524   -1.638158423670
H     -2.665618477658   -0.588279096855    1.127616287047
N     -2.023999549663   -1.164242822413    0.583706250235
H     -1.818305762257   -2.072606648611    0.960850443902

Ambimodal TS Water5-92
35
ANGSTROM
O     -2.524642004696    2.506354920370    3.280552628378
H     -2.542083096757    3.095896636532    2.485894261331
H     -1.631425084463    2.542685664776    3.622382688226
O     -2.656904001755    3.848987070498    0.984730971149
H     -2.654662670731    3.144548193427    0.320909083889
H     -3.581482560151    4.172815936083    1.009755907113
O     -5.291012586779    4.428102352437    1.280624939160
H     -5.707446880413    3.548160529117    1.407511458849
H     -5.436162536651    4.903367151082    2.100019931698
O     -3.929172064704    0.708458757627    1.823107691896
H     -3.491047743362    1.246587084841    2.511501442259
H     -3.598337589611    1.076577587688    0.977119461966
O     -6.314910113146    1.950314493343    1.669675608094
H     -5.498570081998    1.422765979678    1.821871546386
H     -6.742564472557    1.567154025294    0.902677249346
C      0.162568049184    1.624131704028    0.868701466629
C      1.272310506843    1.515153051197    0.081504767590
C      1.981567548786    0.297095729290   -0.209350055809
C      1.590777279499   -0.954926993238    0.238490509353
C     -0.234571794098   -1.370104054080   -0.859890638848
C     -1.242834543583   -0.611042798830   -0.253869088134
C     -1.581900664796    0.734788432691   -0.749031249925
O     -2.566944561385    1.410069764488   -0.410229545465
H      1.595802980277    2.398209060021   -0.472104230441
H     -0.353543974331    2.568950417298    0.976478749021
H      2.740300295526    0.351743202194   -0.989407177330
H      2.160902599078   -1.829276515519   -0.054779233511
H      1.066795841932   -1.070844857883    1.180230638229
H      0.132249200751   -1.081096168078   -1.834213455020
H     -0.177773990140   -2.433454584982   -0.659295518290
H     -0.141598403567    0.855782263810    1.566375584585
H     -0.991897193236    1.072209910061   -1.626320317578
H     -2.660774246312   -0.551605568495    1.281916025590
N     -1.881374833407   -1.065198940444    0.878722708872
H     -1.775619695246   -2.020935387482    1.170117671902

Ambimodal TS Water5-93
35
```

```
ANGSTROM
O     -2.060857524715    2.257643515420    3.533663268447
H     -1.950786551086    3.072179832024    2.991368402885
H     -1.185066800198    2.026615302073    3.843819573816
O     -1.724373654060    4.417347761384    1.978044731799
H     -2.367920769819    4.298052432872    1.229879269491
H     -1.977318553845    5.238511647797    2.401901114336
O     -5.584378228280    2.892489858444    1.529330664865
H     -5.044227778367    2.122737954898    1.810783347869
H     -6.348038791970    2.535220087913    1.075009435235
O     -3.774552139143    0.909093038964    1.910270115131
H     -3.220166787031    1.333169859191    2.593835801150
H     -3.406208638303    1.209929409697    1.053506738387
O     -3.517840943821    3.991490739081    0.086603313465
H     -4.319307626220    3.741954287426    0.586931458445
H     -3.196418357975    3.170828762243   -0.314786592176
C      0.171071787550    1.631032679507    0.850465134912
C      1.280609297393    1.518589998269    0.062746725956
C      1.988774046296    0.298255583573   -0.221556130939
C      1.603315553305   -0.949991283401    0.241470917255
C     -0.228595995877   -1.382171582690   -0.840206359479
C     -1.232058996493   -0.604567472541   -0.250468084519
C     -1.557485440688    0.735495776144   -0.768430010426
O     -2.542370143234    1.418533096028   -0.445070441787
H      1.604450084458    2.398757596515   -0.494911136532
H     -0.341113930362    2.577158015162    0.966393729421
H      2.743448193215    0.345807208509   -1.006088172399
H      2.172958584372   -1.826477972022   -0.046267934062
H      1.085810569683   -1.057287612605    1.187803137228
H      0.139990602611   -1.115267049558   -1.820076919366
H     -0.179114765898   -2.441758619563   -0.618515144753
H     -0.128747334708    0.862403331457    1.550068296412
H     -0.964780257455    1.054401312068   -1.650348103548
H     -2.645734884726   -0.498337938380    1.287647847053
N     -1.884970848470   -1.036105478069    0.883042993228
H     -1.785742057114   -1.984971626378    1.197342748277

Ambimodal TS Water5-94
35
ANGSTROM
O     -2.776629392677   -1.972109954827    3.474949548267
H     -2.128099741119   -1.238535486651    3.561514843485
H     -2.927316748743   -2.307563422462    4.359487585424
O     -1.178245725276    0.202747862742    3.656983284512
H     -1.783062565965    0.842746354980    3.197348039633
H     -1.004652679176    0.587691637875    4.516730882846
O     -4.772184783249   -0.194427071439    2.678645082039
H     -4.982942721464   -0.293179162296    1.733518968169
H     -4.211390241039   -0.951660391887    2.920195276915
O     -4.946592675016    0.037898811832   -0.052963061132
H     -5.663343532878    0.597140738545   -0.355550802356
H     -4.125198485580    0.543004189426   -0.205839791927
O     -2.939629041486    1.750679342303    2.436236567884
H     -3.726877215597    1.183810421387    2.604397013039
H     -2.847868369032    1.805655203254    1.473503171783
C      0.187126621100    1.657783981779    0.806825745255
```

```
C      1.303705470955    1.528293266413    0.033566339105
C      2.023957918291    0.300156412487   -0.192242810780
C      1.637734676756   -0.926516879177    0.311373400920
C     -0.199191370656   -1.384883265572   -0.844504993388
C     -1.212936363113   -0.623449108602   -0.264029299548
C     -1.561041015738    0.726320597633   -0.744333600645
O     -2.565973115314    1.358791416670   -0.365879930436
H      1.628083821274    2.386012901249   -0.559255858076
H     -0.335112133422    2.602226483263    0.886325472567
H      2.796071996959    0.325409211780   -0.961291997424
H      2.206360382223   -1.815513955598    0.064156247855
H      1.075272959526   -1.003494825806    1.233719364817
H      0.201235877945   -1.097502758153   -1.805399261029
H     -0.131860144725   -2.442284378962   -0.622973242031
H     -0.102785265673    0.917295813756    1.538772755201
H     -1.001089194688    1.097701011479   -1.624614463858
H     -2.620478285227   -0.474245445746    1.237763502933
N     -1.925307917215   -1.066747119688    0.818122774408
H     -1.786148158722   -1.962976127200    1.246964647052

Ambimodal TS Water5-95
35
ANGSTROM
O     -6.251286263504    1.801417021545    1.905946685218
H     -3.000686900941    3.265910514894    3.791932617015
H     -6.765267964282    1.953979533742    2.699503927828
O     -2.428140635475    2.907869303490    3.112281098256
H     -2.616477994545    3.802601668199    1.576251699760
H     -2.858576597592    2.080687951729    2.795152000269
O     -5.547919412378    4.228345611864    1.012456339988
H     -5.898994860754    4.494571918662    0.161986257374
H     -6.032247333225    2.693229836614    1.549387303956
O     -3.714313924295    0.866652479500    1.921757916746
H     -4.670754806691    1.068497657252    1.955733919995
H     -3.421895176082    1.198403802987    1.048028657052
O     -2.916219866290    4.075748100431    0.687081231693
H     -4.562109210151    4.232354825412    0.907596290662
H     -2.765231244571    3.302532021843    0.122077483280
C      0.077533232612    1.353849060230    1.183937952016
C      1.240924456712    1.334854165684    0.470459374175
C      1.932743957646    0.152170672003    0.028102654253
C      1.476162826849   -1.138182609951    0.241869372034
C     -0.284645523902   -1.304624150123   -1.025956797732
C     -1.302390854874   -0.568866906814   -0.407100170123
C     -1.518491908491    0.850104208739   -0.735175262040
O     -2.496949641093    1.535972019756   -0.399411637383
H      1.632186070871    2.283292264815    0.098639399949
H     -0.417216026107    2.281898628343    1.435678726307
H      2.745027381077    0.300581400871   -0.682825968128
H      2.037241434005   -1.977878944951   -0.152466196722
H      0.886221334753   -1.380272580145    1.118217527829
H      0.177659407046   -0.918244193635   -1.922801352140
H     -0.299981466156   -2.386287155214   -0.962592200747
H     -0.297957647244    0.492944440821    1.719363328047
H     -0.822222706198    1.266192894107   -1.492919164418
H     -2.749466750068   -0.584912532250    1.100375798394
```

```
N     -2.115003977100   -1.143639337255    0.542824072255
H     -1.974868139923   -2.096112838272    0.828699538083

Ambimodal TS Water5-96
35
ANGSTROM
O     -2.655257698257   -2.141031402111    3.290136885893
H     -2.024666402329   -1.434764322642    3.506776703098
H     -3.482488598949   -1.686694806286    3.090605583050
O     -1.138145760365    0.194771303749    3.607469437148
H     -1.789694956559    0.820928144527    3.219490756224
H     -0.965252919911    0.506730885788    4.496073007780
O     -4.868757988167   -0.395867071463    2.400858176865
H     -5.019735362099   -0.242146352399    1.442760722087
H     -5.712123940647   -0.652643725889    2.774951560522
O     -5.067262535675    0.416286004608   -0.153017903976
H     -5.651666013605    1.138789450576   -0.388164024644
H     -4.158271666841    0.751548089799   -0.292783808724
O     -3.036413572373    1.704237325508    2.421930657357
H     -3.781203397426    1.093534762184    2.541109438166
H     -2.886171927128    1.772426449840    1.465426827565
C      0.175792630082    1.651184288311    0.818684586183
C      1.305479414124    1.500892058335    0.069863389391
C      2.009055881944    0.257987308929   -0.139079671637
C      1.595648718264   -0.958839076070    0.357666219957
C     -0.240444122491   -1.374583027546   -0.877366408551
C     -1.250270196210   -0.609508793768   -0.300448991305
C     -1.560112160256    0.758683326139   -0.753017972622
O     -2.556480960696    1.407165434140   -0.368883845057
H      1.658165573654    2.351113562949   -0.517906291393
H     -0.328650744857    2.606323587688    0.888756158777
H      2.801227188956    0.271841042830   -0.888159448610
H      2.149156305825   -1.859710337777    0.120625939648
H      0.994769982225   -1.030429533055    1.255530549648
H      0.197884404655   -1.069919927123   -1.815982058742
H     -0.184672551470   -2.434575443363   -0.668148216872
H     -0.140165846192    0.916334360811    1.545791034667
H     -0.987463746879    1.138377775568   -1.620765073708
H     -2.641420832441   -0.444344960851    1.207345677588
N     -2.019753241665   -1.076470951659    0.732048712901
H     -1.835583538248   -1.936375217724    1.223199094114

Ambimodal TS Water5-97
35
ANGSTROM
O     -0.843971176424   -3.318263807241    2.259962511005
H     -0.411622521518   -2.580678170628    2.734682973033
H     -0.141966391505   -3.907055139527    1.980369863166
O      0.246135268938   -1.092040327643    3.380985684842
H     -0.573549661391   -0.582991233928    3.571928011567
H      0.771355774609   -1.049003534850    4.181048540407
O     -2.030690673381    0.184577785612    3.981468955462
H     -2.465389705553    0.797335393796    3.344973153158
H     -2.724512104944   -0.392411331642    4.304616330342
O     -3.321382735069    1.699724169680    2.197045886283
H     -3.117788555586    2.640324573529    2.259248049247
```

```
H     -3.144048477511    1.491015750984    1.258215641003
O     -2.473063016611    4.002017087189    0.694021621864
H     -3.051747803101    4.616942651680    0.241496567883
H     -2.452992386311    3.197968204634    0.148088039340
C      0.256928145765    1.543429020736    0.860723255530
C      1.343482129294    1.437856315067    0.044191939481
C      2.028321944049    0.209709128543   -0.280194270887
C      1.641642896618   -1.033423266376    0.176627144778
C     -0.254464089603   -1.414355105723   -0.931303882497
C     -1.232231317339   -0.635239069015   -0.312436719971
C     -1.516878657050    0.736193078980   -0.763287789832
O     -2.472236250511    1.435875382769   -0.377028945075
H      1.667594435952    2.323649071926   -0.505968500114
H     -0.240986859619    2.491791765056    1.016401144267
H      2.768270982272    0.265696501529   -1.079354269736
H      2.181677304369   -1.917443233916   -0.143384005925
H      1.115977231709   -1.145546583079    1.116358202256
H      0.136182974805   -1.121752210930   -1.894664227012
H     -0.215695098088   -2.475874836318   -0.725775206531
H     -0.030475756322    0.759473367626    1.547029144209
H     -0.933996255092    1.085042329999   -1.640694086014
H     -2.554831366374   -0.491077149785    1.265698852784
N     -1.959673944718   -1.114505024749    0.748263188805
H     -1.722314913206   -1.994040789823    1.189862550002

Ambimodal TS Water5-98
35
ANGSTROM
O     -3.415396684984   -0.532348161562    3.128373331861
H     -2.691674295026    0.072737782819    3.420415077324
H     -3.894883759840   -0.783310768528    3.918410346149
O     -1.578380010093    1.262029584421    3.848791121686
H     -2.050403163506    2.050517897813    3.460026121938
H     -1.416077832684    1.489079545520    4.764931244214
O     -4.609165031281    1.440297447655    1.591563338716
H     -4.042843595049    1.400368218017    0.805492142397
H     -4.364151698101    0.663011415325    2.121367661917
O     -2.381416992614    4.004097664713    0.301195655378
H     -3.147069008192    4.477582704527   -0.028541318094
H     -2.468846718430    3.088889062898   -0.028321648901
O     -3.022816730645    3.222682950455    2.867998843237
H     -3.753616649704    2.677420944809    2.503434154462
H     -2.639761277216    3.655142273959    2.084832248395
C      0.114300025307    1.524622222086    0.949496262281
C      1.255893614160    1.455346994542    0.203087267177
C      1.982652402579    0.255609118042   -0.118779970115
C      1.575465248740   -1.017302289922    0.243388002782
C     -0.201406623033   -1.353302769812   -0.981456167808
C     -1.238402019508   -0.646717916588   -0.364615836155
C     -1.569536779849    0.735002114690   -0.742542353437
O     -2.575265611725    1.359725669032   -0.362543386423
H      1.595974698351    2.364158175561   -0.297053457304
H     -0.400396879498    2.466496823152    1.084182468207
H      2.776559040416    0.351643122770   -0.859308242863
H      2.156858614994   -1.877233791895   -0.069603152295
H      1.002556560816   -1.181262340523    1.148484172931
```

```
H      0.212224730009   -0.987224233499   -1.910042834684
H     -0.146233625962   -2.428275148976   -0.859837638917
H     -0.209383599964    0.738106648292    1.616820232648
H     -0.965072808002    1.160760196725   -1.570559753257
H     -2.611216783114   -0.665765983181    1.204475820135
N     -1.979602956889   -1.215053346550    0.643868648057
H     -1.752816086543   -2.123674849394    1.006379548146

Ambimodal TS Water5-99
35
ANGSTROM
O     -1.830367387468   -2.619638249172    1.787882647099
H     -2.564265736988   -2.025837722851    2.064489516695
H     -1.774546882549   -3.290503689633    2.468889031334
O     -3.906099056005   -1.007222537326    2.362006838145
H     -3.821285634501   -0.040294673588    2.288007614361
H     -4.632868020125   -1.241311672967    1.760513336394
O     -5.625963202245   -1.317920175496    0.207051812706
H     -5.428379250916   -2.001921339541   -0.434290729312
H     -5.571715534594   -0.472134398737   -0.270359199716
O     -5.150930183018    1.031650471400   -1.152801826224
H     -4.196228826630    1.246448322883   -1.120921991889
H     -5.499404831272    1.449426670434   -1.940296488799
O     -3.943876791595    1.627643411484    1.535680458698
H     -4.838253156155    1.682299991970    1.187516307024
H     -3.361870804240    1.776006294851    0.774733168068
C     -0.515111475107    0.880341810160    1.284725345052
C      0.807344989728    0.898574145204    0.956129780818
C      1.560731739435   -0.237845560619    0.484132485112
C      1.021003163482   -1.489847994858    0.269566723198
C     -0.275305209542   -1.168087472853   -1.497585999464
C     -1.402925994535   -0.480114557977   -1.053871988759
C     -1.480909797590    0.983779336541   -1.088093104105
O     -2.513667876652    1.651152835155   -0.880366640879
H      1.326086011019    1.859104334084    0.932682201096
H     -1.036826010656    1.781191505281    1.580409132446
H      2.562392863803   -0.029244240864    0.105915371421
H      1.644370408598   -2.281323780421   -0.131565648338
H      0.160184269670   -1.842467497981    0.825340935292
H      0.470093063996   -0.653766890093   -2.087326611531
H     -0.335955954926   -2.235130080344   -1.666427062692
H     -1.051893765317   -0.038199116984    1.477033622515
H     -0.590082618529    1.511290290238   -1.485252443123
H     -3.180439482765   -0.616922435256   -0.041084505936
N     -2.519877962559   -1.157293335390   -0.582567471367
H     -2.378920254279   -2.085396201212   -0.209212554063

Ambimodal TS Water5-100
35
ANGSTROM
O     -2.891060779323   -3.633278489257    1.522086622082
H     -3.568925560579   -3.102275452038    1.975805161066
H     -3.234298416665   -3.779106433503    0.625808630001
O     -4.871115713876   -1.893502354905    2.328753410773
H     -4.706057641111   -0.978774902005    2.040150430996
H     -5.768514192948   -2.098055280060    2.061813079964
```

```
O      -2.865955755078   -3.938315834555   -1.271731495817
H      -2.731048454684   -3.150543889380   -1.800638997174
H      -1.983786959336   -4.237194706912   -0.992678973317
O      -0.615291965674   -4.564261086649    0.271829619464
H      -0.414660625573   -5.481726020184    0.462517763329
H      -1.251493595229   -4.280166053973    0.949705997995
O      -4.393688962853    0.681999560591    1.443681659397
H      -4.568020444610    1.456190818064    1.980154067208
H      -3.816701460812    0.988747861678    0.704976519930
C       0.208678079165    1.659279504477    0.838428185262
C       1.298296920880    1.534092065883    0.028523044786
C       1.998562464950    0.307317358636   -0.254919387890
C       1.614645116568   -0.934440989035    0.224038662453
C      -0.229271550974   -1.373729938553   -0.828172623835
C      -1.220743619168   -0.594252764548   -0.223519858509
C      -1.592613243312    0.732990870000   -0.748177360679
O      -2.584258221082    1.391092682291   -0.403371303308
H       1.613474378710    2.405909598882   -0.548228979449
H      -0.308438000277    2.603721956429    0.944144897161
H       2.740856211128    0.347733353727   -1.051846864065
H       2.173945675178   -1.816692197913   -0.065126842372
H       1.103969602544   -1.033669852119    1.174305241405
H       0.119532951441   -1.109680769403   -1.817063853121
H      -0.158162954027   -2.426974312372   -0.584946982837
H      -0.085944607183    0.900749538155    1.549560712084
H      -1.018378694717    1.070195538648   -1.637658735505
H      -2.597204980446   -0.458111153601    1.301415349387
N      -1.826961864711   -1.002136261105    0.944666417654
H      -1.836259105429   -1.977985151986    1.212807978336

Ambimodal TS Water5-101
35
ANGSTROM
O      -2.490183000105    2.539493816327    3.252281243099
H      -2.437654043276    3.097967497387    2.436482052675
H      -2.975994728533    3.056329666006    3.896153782310
O      -5.312831974163    4.435509669039    1.134636334815
H      -5.683774464709    3.586899751400    1.462636894020
H      -5.637705432714    5.115946745916    1.724980562793
O      -6.231563852885    2.066904479278    2.047243970873
H      -5.411556877206    1.516976226048    2.031365623154
H      -6.853233457768    1.630526685407    1.463443459272
O      -3.909837109530    0.759229513513    1.836925549746
H      -3.385813641806    1.283372165055    2.482675767552
H      -3.608597159668    1.089053805896    0.964549868743
O      -2.647830778139    3.862488177025    0.946256410566
H      -3.573773716582    4.174337722667    0.942871865543
H      -2.622715031555    3.146756959710    0.294071418885
C       0.156072397163    1.622403401259    0.874188986486
C       1.268897524273    1.512664189099    0.090862272877
C       1.979882055764    0.294426713446   -0.196262879918
C       1.588442854340   -0.956119449804    0.252786662354
C      -0.237050493136   -1.367792671666   -0.859998552884
C      -1.245720717070   -0.606241989247   -0.258868526648
C      -1.575734691483    0.742563339261   -0.749894638137
O      -2.564608810972    1.417731127914   -0.418244831935
```

```
H      1.594667693233    2.394485378510   -0.463941273813
H     -0.355674748271    2.568314293353    0.988147828597
H      2.742202467730    0.347920915104   -0.973283332968
H      2.158133629906   -1.832181769503   -0.036402635424
H      1.057148542609   -1.068807570427    1.190555533459
H      0.138644645466   -1.078175378991   -1.830616412282
H     -0.183660450106   -2.431278411262   -0.659701501019
H     -0.148760513199    0.861381694571    1.578875551122
H     -0.979884412839    1.081584336097   -1.622531984873
H     -2.657813706220   -0.535612565523    1.282953519378
N     -1.904430948251   -1.066721576534    0.857957989115
H     -1.777635418414   -2.011344759371    1.174040488605

Ambimodal TS Water5-102
35
ANGSTROM
O     -1.384285516144    2.980102028004    4.163588866820
H     -1.200582890441    3.348036867128    3.273081321591
H     -2.226479668195    3.390220473284    4.397388019007
O     -3.947258501442    3.972131842951    3.351749838748
H     -4.106274807689    3.057419061645    3.006862501437
H     -4.803178150674    4.399269222808    3.376633678907
O     -4.168456967286    1.496484873171    2.391172270752
H     -3.644948874183    1.558823704061    1.575604113099
H     -3.595072724294    1.000533121164    2.999889688382
O     -2.068105213323    0.460088027897    3.841516362764
H     -1.729292987735    1.389998181909    3.953978670879
H     -2.058465267909    0.069055304428    4.715220005359
O     -1.656475110275    4.055940423371    1.702951706407
H     -2.493088250219    4.330685841454    2.107694249696
H     -1.885120842844    3.303829853982    1.134145713824
C      0.789872058463    1.616246502847    0.866395813704
C      1.599368945804    1.489658143371   -0.225492149431
C      2.085291272804    0.251265380970   -0.774245142324
C      1.744333648953   -1.000356832188   -0.284565903710
C     -0.349998576359   -1.225473276346   -0.787054957158
C     -1.068583942319   -0.419525053741    0.104616147409
C     -1.424820488349    0.968964056868   -0.225340945807
O     -2.219700746372    1.675553281327    0.411784413546
H      1.812397976317    2.385697888862   -0.810744439721
H      0.411786774503    2.581803234127    1.173692889387
H      2.573424178162    0.308206404915   -1.746622355766
H      2.125629264511   -1.888712088912   -0.775572691467
H      1.528772439925   -1.143542364618    0.768133709746
H     -0.268525763043   -0.924751216149   -1.821957400427
H     -0.332195538535   -2.298039702554   -0.632443153999
H      0.650280271759    0.826246826420    1.591352341226
H     -1.072586712995    1.327751406299   -1.215697905739
H     -1.781234107764   -0.266548210161    2.061190953315
N     -1.460347964969   -0.894279882174    1.334962055745
H     -1.187761531819   -1.813202369780    1.636536444151

Ambimodal TS Water5-103
35
ANGSTROM
O     -3.377495106508    0.587725127203    2.505022714058
```

```
H     -2.910474353898    1.426578493297    2.565506611393
H     -4.293218224911    0.823273977895    2.282764238778
O     -5.695259875291    1.796947860082    1.623938545020
H     -5.642652050158    1.728485850309    0.632408852542
H     -6.618141724660    1.931720080453    1.837946335046
O     -5.288900227608    1.848308294527   -0.978224061202
H     -4.362491919866    1.566843695192   -0.971980883239
H     -5.254714034753    2.818272056438   -1.089926080129
O     -4.697847807062    4.527611496009   -0.841945139202
H     -4.056268240558    4.903345243844   -1.445770234635
H     -4.210462767769    4.320733325132   -0.015598473703
O     -3.417491363707    3.389958681860    1.269744047883
H     -4.243522869068    2.983825622370    1.579746333660
H     -3.034120352857    2.743938381387    0.653833973587
C      0.055242268764    1.462282422269    1.013343615453
C      1.225163219994    1.342878625879    0.322044818314
C      1.895241752286    0.107104572917    0.007118777415
C      1.407724274133   -1.146095675275    0.334235420572
C     -0.341480973773   -1.401395094689   -0.958497452350
C     -1.350770021383   -0.607759265930   -0.402990450603
C     -1.559449615937    0.783799775936   -0.838510275971
O     -2.540548181173    1.483047373692   -0.546398351287
H      1.644153954532    2.240417860858   -0.136572364859
H     -0.418903706585    2.425509958018    1.150558735850
H      2.720786312598    0.171047798568   -0.700942431914
H      1.954684010223   -2.031074207984    0.029987825925
H      0.792514206322   -1.293099795608    1.214224237315
H      0.135793011518   -1.090058378217   -1.876728677555
H     -0.366253297494   -2.474129365324   -0.808297978807
H     -0.352078890780    0.668571500112    1.624050412944
H     -0.863285288925    1.148385255905   -1.624755840648
H     -2.731571095725   -0.439507191834    1.150934985107
N     -2.188442818337   -1.082589376986    0.574051149786
H     -2.045952960844   -1.999696432605    0.960558000401

Ambimodal TS Water5-104
35
ANGSTROM
O     -0.181959101255    3.570204738443   -2.121531240809
H     -0.805641969515    4.103824165858   -2.635321048782
H     -0.339968076600    3.830362764845   -1.204452582765
O     -1.348907655493    4.689793369926    0.284942653209
H     -2.157808073290    4.442644381154   -0.208996207144
H     -1.193779104238    5.616100626160    0.094232057039
O     -3.642269475634    3.742886420184   -0.904078737245
H     -4.183565587077    3.827879347433   -0.111140253209
H     -3.274983584939    2.832046379289   -0.851669484942
O     -3.275591182673    3.063200474263    1.871788832160
H     -2.527965196765    3.664381527140    1.782247620674
H     -3.072421186474    2.313831423359    1.295897140949
O     -2.430235463927    5.033277405043   -3.047848030796
H     -2.970087607381    4.637944688989   -2.344836401678
H     -2.853487434997    4.802040474321   -3.875376019830
C      0.188441895961    1.610378385567    0.879165447376
C      1.303466691484    1.522643951687    0.092791606415
C      2.023760792962    0.316052967672   -0.203100848789
```

```
C      1.631135942223   -0.947187164943    0.219951599083
C     -0.158869365624   -1.313457033430   -0.900676549744
C     -1.179293476453   -0.577087099524   -0.294968704292
C     -1.567787374198    0.771110773856   -0.717222520967
O     -2.593109230646    1.342582328893   -0.289802065058
H      1.608524789043    2.408155696556   -0.463331542159
H     -0.330741715913    2.552493949715    0.993787442632
H      2.790219070351    0.384498498960   -0.974377353106
H      2.209599217095   -1.814386173419   -0.080439511925
H      1.103766861216   -1.080400051208    1.157953048607
H      0.215913716694   -0.989817396207   -1.861364484024
H     -0.107961738029   -2.383805385802   -0.736904766742
H     -0.101848649109    0.842192277265    1.584017366786
H     -1.030070604584    1.205576309972   -1.577704323902
H     -2.654345311180   -0.537821736837    1.126518398622
N     -1.839534227206   -1.055325166488    0.834116649778
H     -1.844283569574   -2.038240420015    1.044601325222

Ambimodal TS Water5-105
35
ANGSTROM
O      0.111544514021   -1.567180976496    3.251690868303
H      0.278481792860   -2.123665002214    4.013177805133
H     -0.220621844087   -0.720249766612    3.609085105350
O     -0.905841263006    0.797854896426    4.166667995006
H     -1.679267036396    1.068225151489    3.632804130103
H     -0.403804381850    1.598361093470    4.325443079657
O     -5.522997473465    0.756436545107    2.518692151235
H     -5.684965578928   -0.155511084924    2.764094900209
H     -5.559810630148    0.786929937583    1.539748887297
O     -5.279558407963    0.923304886756   -0.175037142670
H     -5.671273023438    1.702865410020   -0.572607389380
H     -4.314665549478    1.051327062913   -0.228824604803
O     -2.924424986974    1.554851426879    2.523153974669
H     -3.868798592502    1.357325507592    2.677470953152
H     -2.843578348167    1.737786878039    1.572504136938
C      0.266903060626    1.715114206398    0.745343807166
C      1.271789655406    1.538568548134   -0.156267237335
C      1.967236862652    0.296643629756   -0.398955909143
C      1.679184933128   -0.894909261325    0.232406563844
C     -0.315255280050   -1.437764386450   -0.601670719496
C     -1.244319063237   -0.597152844482    0.009661798228
C     -1.649887300026    0.671201232134   -0.613358789680
O     -2.591425029513    1.394522808977   -0.238187835661
H      1.507753826795    2.357934578579   -0.838302447421
H     -0.258134944445    2.657688579570    0.830944092949
H      2.624690313518    0.278544749438   -1.268954987829
H      2.215947604233   -1.793614815940   -0.049859870155
H      1.251727610662   -0.933497373754    1.226824975174
H     -0.046136371662   -1.281845309133   -1.636467650559
H     -0.201372104883   -2.452725619511   -0.245214543783
H      0.070249510253    1.010516863569    1.540876154851
H     -1.167180785481    0.905590285485   -1.585393404109
H     -2.307020142860   -0.199175202919    1.725677787249
N     -1.834339763258   -0.933997731265    1.212644010143
H     -1.359634200285   -1.602540006530    1.803786637426
```

```
Ambimodal TS Water5-106
35
ANGSTROM
O     -1.317817885409    4.674843208726    1.487343944204
H     -1.277656116119    5.656009876445    1.439370329878
H     -1.939398140652    4.476950338984    2.190412599581
O     -4.088515400820    7.660112100970    0.713669088702
H     -4.394149316917    6.796853788655    0.342396255887
H     -4.637144121807    7.823160815234    1.482015156380
O     -4.924180407625    5.315707063931   -0.240348173392
H     -4.093801396495    4.803645421448   -0.436892786914
H     -5.374877297500    5.399772171197   -1.081577030198
O     -2.694437049189    4.029339410985   -0.789272325175
H     -2.106757426795    4.279310094340   -0.049993474005
H     -2.789544447922    3.062970104997   -0.710711743817
O     -1.478571038798    7.349710583519    1.269201633447
H     -2.433296373929    7.500245983357    1.082136574599
H     -1.009258750500    7.682680521500    0.502939835548
C      0.203578356169    1.606337406980    0.879819320835
C      1.304763033466    1.522866001344    0.073473375082
C      2.023365661576    0.321095601682   -0.235871331164
C      1.633333997648   -0.946221353667    0.199609548831
C     -0.137850287443   -1.356940733481   -0.832826373921
C     -1.169004653867   -0.597892637186   -0.260839928083
C     -1.575614563767    0.729915325953   -0.739878886963
O     -2.590601758461    1.316573765366   -0.345416911004
H      1.606838813643    2.417763975015   -0.472437032636
H     -0.321844616735    2.543066447356    1.009088797896
H      2.772903209342    0.384922545312   -1.024037001360
H      2.221881451289   -1.807411152884   -0.100390345759
H      1.157016764345   -1.067090343207    1.166680024723
H      0.206710805220   -1.089342903223   -1.822806498814
H     -0.101114057204   -2.421690608104   -0.628063896132
H     -0.072253041137    0.828982366526    1.579012948327
H     -1.016384664009    1.127002883002   -1.613586796054
H     -2.639130911301   -0.484965401251    1.149151090083
N     -1.832778887918   -1.029490267336    0.881654107436
H     -1.843803685936   -2.001515346553    1.137319435334

Ambimodal TS Water5-107
35
ANGSTROM
O     -3.051564593539   -3.685522259249    1.385909900872
H     -3.753619420168   -3.124707116640    0.948214991978
H     -3.512101869618   -4.251636663365    2.005635237448
O     -3.122556192717   -2.265142303787   -2.105205155001
H     -2.553738111487   -2.978755739409   -1.751247701864
H     -3.374810852373   -2.525721971079   -2.991807632400
O     -1.835148000402   -4.371366114495   -0.920916053098
H     -2.192153697148   -4.169689754493   -0.029055588332
H     -2.166672960699   -5.240928104602   -1.150271389669
O     -4.568894722564    0.378836743750    0.992034853256
H     -5.324918455482    0.966152660177    0.977441447859
H     -3.884533566141    0.798190764304    0.415523659946
O     -4.784923051066   -2.202883001351    0.091827141496
```

```
H     -4.302701942955   -2.128684965252   -0.751767201874
H     -4.791169352250   -1.300475551733    0.463675057917
C      0.174874754020    1.669287125813    0.829227196285
C      1.289565293980    1.556854073468    0.054044971453
C      2.022676861992    0.340917906955   -0.195206990718
C      1.646765819618   -0.903688214762    0.282105652779
C     -0.155507763527   -1.388965321831   -0.818709286488
C     -1.180531081147   -0.626287494837   -0.253353541651
C     -1.570278941637    0.677225571874   -0.817717641710
O     -2.584783242871    1.322118510680   -0.522251649164
H      1.607041119229    2.428815505392   -0.521279937895
H     -0.365825738146    2.602949832548    0.907281802625
H      2.787684530793    0.388263862354   -0.969905589180
H      2.234968178532   -1.775886995009    0.020005851567
H      1.110550119072   -1.002912960641    1.218235354525
H      0.211443006199   -1.127258787874   -1.801086915882
H     -0.088300158296   -2.443185295250   -0.580675724779
H     -0.131701999096    0.910458130812    1.534483242631
H     -0.969097031075    1.012730762669   -1.689484818509
H     -2.613400116784   -0.521370388882    1.223620439383
N     -1.799829979821   -1.028818111626    0.913535691816
H     -1.782550243760   -2.000351187253    1.182641167848

Ambimodal TS Water5-108
35
ANGSTROM
O     -4.232566523339    0.623436834436    1.684391083579
H     -3.684303911103    1.076334190554    1.015673345820
H     -5.114743918187    1.018184360291    1.592981353041
O     -5.171618167440    0.902635565515   -1.460836689714
H     -4.286194337451    1.237994005731   -1.263885873087
H     -5.756597992719    1.314846753697   -0.808883146044
O     -6.748661310210    1.502433603644    0.849959169358
H     -7.150161339128    0.602913414496    0.763998065020
H     -7.396449569673    2.047510228636    1.296686821343
O     -5.040443083899   -1.406740581856    0.001502857381
H     -4.711860930137   -0.884170285873    0.757524405839
H     -5.049446760833   -0.764005906222   -0.735382067825
O     -7.621338535233   -0.990972265747    0.469524840705
H     -8.059881489350   -1.134877883146   -0.370572865402
H     -6.696901295965   -1.326893265992    0.349938645577
C      0.165003906138    1.666618181357    0.793719101519
C      1.253237113192    1.544086735674   -0.020833316315
C      1.984877193875    0.328937459445   -0.266050863058
C      1.639970391401   -0.904694292930    0.262436524543
C     -0.208555163486   -1.421130228384   -0.751349843958
C     -1.211754207175   -0.645584382340   -0.161431512599
C     -1.600996439147    0.657228556386   -0.727587528253
O     -2.598564411147    1.321101592842   -0.405464385496
H      1.537016638790    2.402171400134   -0.632864038902
H     -0.377194886500    2.599708849934    0.870455448109
H      2.718380170852    0.358775902652   -1.071240674876
H      2.222406598338   -1.780384332566   -0.001173399922
H      1.146793596905   -0.980285871654    1.224476404514
H      0.123274330477   -1.184781079262   -1.752148047442
H     -0.125558165159   -2.469170690293   -0.489352095816
```

```
H     -0.097129133326    0.932633723089    1.542608480237
H     -1.044403135235    0.956432316836   -1.640698343004
H     -2.587391961003   -0.523061222599    1.388243676728
N     -1.800260715297   -1.037687765962    1.022935843578
H     -1.727684725513   -1.992501736096    1.328686911078

Ambimodal TS Water5-109
35
ANGSTROM
O     -5.107367181912   -1.454207702332    2.406272121639
H     -4.411933595280   -0.853525749633    2.702051199002
H     -5.814405391258   -0.859997376681    2.103711564504
O     -3.727925392598    1.028412840352    2.009800250003
H     -3.322398595179    1.426862399140    1.221462345686
H     -4.680121768431    1.151588737563    1.892009748430
O     -6.427329054056    0.650135697960    1.195374773679
H     -5.973340197203    0.471434031814    0.329610252962
H     -7.266330655598    1.058051470547    0.982665680390
O     -4.333367270549   -2.378673285124    0.014162626365
H     -4.591665503453   -2.128385955294    0.933756330015
H     -4.953008397165   -3.056038313840   -0.259567311318
O     -4.997488217716    0.099365637064   -0.953905010481
H     -4.214560921113    0.665897940131   -0.880345660888
H     -4.694333674313   -0.805720200095   -0.739911173238
C      0.169310329317    1.646099293321    0.857081547116
C      1.292560161223    1.528066347954    0.091237887526
C      1.998239427485    0.303347644426   -0.186228030760
C      1.594899289753   -0.944351816977    0.257060110183
C     -0.224530578001   -1.356812728784   -0.881338060807
C     -1.221637546807   -0.575746986891   -0.291702583048
C     -1.544387836660    0.771524851016   -0.794957146794
O     -2.554022005777    1.422056485810   -0.477297252471
H      1.634804668711    2.407290124084   -0.458073763684
H     -0.342619125209    2.593713535494    0.960134107136
H      2.771587070157    0.354002010843   -0.952337512445
H      2.162723115086   -1.823191743864   -0.026665976075
H      1.045446543556   -1.056876645640    1.184114036052
H      0.164239312149   -1.078625352856   -1.850425859307
H     -0.187417926699   -2.418762215616   -0.671004566140
H     -0.156136422031    0.883467506931    1.550355985369
H     -0.937270488694    1.123194469831   -1.654402451965
H     -2.623197894109   -0.420342414045    1.221338151632
N     -1.883343152027   -0.995572190184    0.838642228350
H     -1.911870434349   -1.970117929662    1.080505009565

Ambimodal TS Water5-110
35
ANGSTROM
O     -3.250745791267   -3.408184788578    0.633306364377
H     -3.623053450255   -4.226736391419    0.303680117007
H     -3.901356540457   -2.710887380004    0.432441844551
O     -5.105222250802   -1.379840740654    0.186220457493
H     -6.012912859707   -1.563195309142    0.434936779755
H     -4.807472525340   -0.649097118278    0.792928889655
O     -4.363563106881    0.740934227033    1.586055724934
H     -5.188267909273    1.241508177585    1.446370750166
```

```
H     -3.724798087270    1.130745264097    0.955137087918
O     -5.109295428088    0.830453015950   -1.563182435835
H     -5.110016622764   -0.075200019448   -1.212534809253
H     -4.236075564144    1.187998261710   -1.345342585780
O     -6.532511827946    1.942319321023    0.443200591886
H     -6.686187252202    2.874903713316    0.290140541867
H     -6.150269345493    1.585439596253   -0.390364808920
C      0.202341180920    1.686813533409    0.782511589895
C      1.279277937387    1.523301476156   -0.037446765648
C      1.982864524001    0.285447042623   -0.268156263492
C      1.625717903748   -0.929169209840    0.285093316646
C     -0.264220656894   -1.431169941621   -0.728098188276
C     -1.228452905931   -0.619765725895   -0.129055237587
C     -1.596302585702    0.678952343943   -0.712639790492
O     -2.594378186971    1.353714455371   -0.402726091435
H      1.580066204810    2.363425512615   -0.666504079285
H     -0.315198389459    2.634859331754    0.848532904044
H      2.712901091011    0.290683479017   -1.077537182816
H      2.184349404680   -1.822019720224    0.029081580887
H      1.117822936428   -0.985587152387    1.240116824644
H      0.070752241613   -1.213828712539   -1.732347342190
H     -0.200785256250   -2.473585504421   -0.442990177579
H     -0.075987327741    0.967963495965    1.540196668396
H     -1.040144558374    0.964534916723   -1.630066736877
H     -2.560374569675   -0.424941344420    1.431536404554
N     -1.791175208417   -0.975505678668    1.081810453930
H     -1.852880690209   -1.956011796947    1.311709009876

Ambimodal TS Water5-111
35
ANGSTROM
O     -3.979596032792   -0.030284645893    2.526925407918
H     -3.692935926594    0.924720554570    2.506676959422
H     -4.241458467820   -0.210570992144    3.429356097789
O     -3.233463358732    2.460345536051    2.166643667648
H     -2.822025897214    2.282507533320    1.310735622627
H     -4.089013894882    2.895704743238    1.935701602554
O     -5.508687181864    3.275479538347    1.033204992107
H     -5.101703402374    3.702567942985    0.261579020545
H     -5.704507091886    2.367424973942    0.735577661612
O     -5.246682236140    0.666978449018    0.146851304114
H     -5.093284600985    0.231278445883    0.998368795126
H     -4.358419049182    0.879877874384   -0.173789025608
O     -4.054193491458    3.589718480785   -1.324309557470
H     -3.423757110143    2.904388147236   -1.047444672788
H     -4.637600239867    3.175195976611   -1.962880268041
C      0.138182610035    1.589955148783    0.924012476916
C      1.264500860015    1.505606572404    0.158698902977
C      1.983017971389    0.294934644888   -0.153120385128
C      1.601634570694   -0.965724374613    0.260962496519
C     -0.235290950073   -1.357934238364   -0.919509625329
C     -1.243551158191   -0.615279323319   -0.301662271948
C     -1.554949526333    0.750941128001   -0.746433641359
O     -2.554849817625    1.408758173892   -0.399742278227
H      1.599807969610    2.403785962356   -0.362862653529
H     -0.373103692885    2.533138573529    1.065478404656
```

```
H      2.757689688526    0.376035244395   -0.915282569020
H      2.173589555853   -1.832759150882   -0.048128781758
H      1.039123812523   -1.111231529317    1.175289982290
H      0.166888523513   -1.030524495997   -1.866661393169
H     -0.173408055556   -2.425377063243   -0.748281748872
H     -0.180772645552    0.803940318225    1.594559548166
H     -0.953544711091    1.124982024205   -1.599615429081
H     -2.663877372315   -0.590554620448    1.246415184633
N     -1.926411101704   -1.107500444777    0.783270214744
H     -1.811165681959   -2.062889326677    1.070220368275

Ambimodal TS Water5-112
35
ANGSTROM
O     -4.284529536382   -2.686662825419    1.025973651183
H     -5.029915806826   -3.136987240890    1.424764620611
H     -4.524665082384   -1.745469005310    0.996994899700
O     -4.753087489364    0.040297589319    0.622172353070
H     -4.036830207088    0.659825321150    0.380924523305
H     -5.567817220323    0.445838006753    0.263848943476
O     -6.642874442218    1.349771246125   -0.795902401782
H     -5.981070951286    1.405538283111   -1.523888458143
H     -6.942380186034    2.245582715102   -0.637584649799
O     -3.963729429995   -1.260795634918   -1.741482998427
H     -4.297014681011   -0.897620034785   -0.900338382352
H     -3.986699326727   -2.212601185593   -1.630117314628
O     -4.529243706881    1.402096199864   -2.456679247484
H     -3.838972685433    1.668489577694   -1.830529177701
H     -4.386302267501    0.454679306727   -2.590450031953
C      0.190179471788    1.640940842263    0.840932007030
C      1.287663492260    1.531210118520    0.037687068515
C      2.004824589403    0.314156685066   -0.241063988280
C      1.634679356551   -0.932693791934    0.236300594877
C     -0.202815178395   -1.385731210091   -0.822600974566
C     -1.198761161715   -0.611077599717   -0.224401172164
C     -1.572231346565    0.709478542863   -0.749659099022
O     -2.582912015538    1.351910764820   -0.415784269863
H      1.593688277472    2.406366726566   -0.538446597290
H     -0.340347356824    2.578326094485    0.943493798781
H      2.751797279362    0.363171946874   -1.032898307484
H      2.209261296499   -1.806156873975   -0.050886917715
H      1.123058524404   -1.037666042820    1.185758283488
H      0.146703609520   -1.124864000766   -1.811386859433
H     -0.143425732198   -2.442276047258   -0.590266920964
H     -0.094336860270    0.882005220782    1.555914993128
H     -0.999065903019    1.053370816045   -1.635028345834
H     -2.599001321972   -0.479930753028    1.266406735234
N     -1.805684833466   -1.017570718606    0.952703500770
H     -1.886245288738   -2.000949422104    1.156510256703

Ambimodal TS Water5-113
35
ANGSTROM
O     -3.811555631499   -2.472589956376    3.474542219906
H     -3.571950009175   -1.546331161305    3.628572163951
H     -4.371189662045   -2.450599875938    2.675144649691
```

```
O     -3.296374298084    0.287036462901    3.311644627683
H     -3.305089427719    0.945251981616    4.006794120481
H     -3.972704989443    0.549944497132    2.660178646995
O     -4.838172915639    0.858223762867    1.137565862927
H     -4.036544089940    1.039732106053    0.584948215408
H     -5.494033649716    1.504309940833    0.873453883801
O     -1.584118039406   -3.488579459613    2.398432068746
H     -2.363080049685   -3.178611771389    2.920689291349
H     -1.717456447705   -4.427855314181    2.267263880614
O     -4.917864325757   -1.944830147604    1.006413526864
H     -5.085043850008   -0.991108913305    1.065129138603
H     -4.067990164258   -2.023657393228    0.557909080425
C      0.102485159582    1.412853518517    1.029365003427
C      1.189750631195    1.339759448234    0.209720659189
C      1.866945215906    0.128918942313   -0.177986111620
C      1.462597545481   -1.141385281100    0.196131329336
C     -0.403980249981   -1.444089336019   -0.881265939569
C     -1.367495118689   -0.667520544244   -0.231289638559
C     -1.691422466943    0.698035552676   -0.675080128093
O     -2.667991374464    1.367247889986   -0.307471112944
H      1.515823912224    2.250850670712   -0.295499683801
H     -0.401593163041    2.352087928401    1.214887868498
H      2.607892189559    0.223015648547   -0.971586553768
H      2.003619409367   -2.004601320570   -0.174568773230
H      0.956495913612   -1.318003929688    1.137930228236
H     -0.046423054791   -1.146438983333   -1.857399429245
H     -0.378624946588   -2.510504281262   -0.694069546926
H     -0.194827526465    0.605635954113    1.684044605679
H     -1.083567437507    1.078390998480   -1.523492663403
H     -2.480773196267   -0.517991307759    1.495816690309
N     -2.078920835485   -1.174978547672    0.841324905952
H     -1.755192904200   -2.034733977980    1.280893827239

Ambimodal TS Water5-114
35
ANGSTROM
O     -3.519007788154    2.519054046857    1.894426337374
H     -3.061062075001    2.254764334203    1.084572229325
H     -4.426371370567    2.765158083056    1.587816457669
O     -4.003624331862    3.630676875583   -1.330463522129
H     -3.487944613451    2.822504725077   -1.174376663329
H     -3.509278403768    4.351217648108   -0.935541095070
O     -5.841030416194    2.843541947255    0.618430677675
H     -5.906689230712    1.896329171871    0.394305700043
H     -5.465591210063    3.259113298914   -0.174971730394
O     -3.921491140372    0.000614457276    2.540370758087
H     -3.753666422240    0.976141545387    2.432781133468
H     -4.339169836195   -0.106598957668    3.394583353903
O     -5.195348935703    0.221284439867    0.071598220423
H     -4.374727337857    0.540402625695   -0.326928306991
H     -4.946997682196   -0.046649613790    0.970703061343
C      0.042498165490    1.565829706976    0.949548499533
C      1.190319406490    1.525439822334    0.213928547491
C      1.949083086465    0.339317243535   -0.098076343251
C      1.592826061826   -0.937161359068    0.289021217447
C     -0.202498624189   -1.372647025106   -0.937543670367
```

```
C     -1.243767082867   -0.650400883323   -0.350710571130
C     -1.570321221309    0.708204666373   -0.808284926792
O     -2.594645764690    1.344561431915   -0.498253981552
H      1.515764354150    2.440879786849   -0.283530034701
H     -0.500860713864    2.490346003401    1.094803465416
H      2.738641762196    0.453047582554   -0.840321075032
H      2.196265552812   -1.783314421242   -0.017743479066
H      1.014830343996   -1.110955785992    1.188696109343
H      0.216793570483   -1.040255930992   -1.875585190362
H     -0.123695147937   -2.438189926423   -0.761252485108
H     -0.273469262929    0.758469372598    1.595343921424
H     -0.941608855931    1.098391441426   -1.634276468443
H     -2.665982178354   -0.627798215620    1.192525398932
N     -1.960005583465   -1.160122720198    0.702850823777
H     -1.810735656921   -2.099815516374    1.022992434598

Ambimodal TS Water5-115
35
ANGSTROM
O     -3.821991087382    0.374253130149    2.479584109673
H     -3.614452939879    1.335840679721    2.282059715353
H     -4.056085952356    0.340676018699    3.407217764560
O     -3.299146761906    2.839259104853    1.831988522065
H     -2.952403742630    2.629124342643    0.951969006060
H     -4.155159139657    3.287078679621    1.660789738081
O     -5.722019500513    3.721985636302    1.037345719833
H     -6.540471315500    3.438285099215    1.446436106691
H     -5.637976561063    3.193518259839    0.207128705820
O     -5.645875658554   -0.279808187868    0.568659314432
H     -5.048552117728   -0.045380303573    1.307660006005
H     -5.223254604573   -1.003823947960    0.104126994705
O     -5.303409265801    1.985327388043   -0.945329504413
H     -4.337694785509    1.955006747320   -0.971328391442
H     -5.572249437412    1.173450405825   -0.474062380524
C      0.065666646202    1.448216612203    1.067127942860
C      1.233291153024    1.381568787855    0.362977887094
C      1.903997470598    0.171300517208   -0.028230357468
C      1.409499717057   -1.102028008039    0.222055535228
C     -0.323377934367   -1.270948971626   -1.032958900916
C     -1.336821256564   -0.488042297169   -0.464960988723
C     -1.547462689303    0.916007431745   -0.853908328156
O     -2.522011854165    1.606996377643   -0.534094495701
H      1.642454478790    2.307988011760   -0.042913211703
H     -0.422519888782    2.392388884827    1.266947943739
H      2.723814901228    0.275125983905   -0.737809769524
H      1.958539956550   -1.965869997790   -0.136063593536
H      0.822141822031   -1.300964185267    1.111591833923
H      0.147662256455   -0.936487118338   -1.946687252334
H     -0.370447111416   -2.348880621054   -0.926732015900
H     -0.326355402959    0.616091755577    1.635251499112
H     -0.842073463216    1.305691471394   -1.619833538599
H     -2.746463730992   -0.385261491620    1.059207184581
N     -2.198688746643   -1.010355043850    0.479944077590
H     -2.014902031420   -1.916187215291    0.877117865794

Ambimodal TS Water5-116
```

```
35
ANGSTROM
O     -3.754598101543   -1.059941185869    2.006655965249
H     -4.473805218452   -0.830265838605    1.388966559511
H     -3.969064047732    1.084979785307    2.164534852296
O     -5.345384975668   -0.253917215215   -0.101541251621
H     -4.520590668457    0.142399950587   -0.446365902247
H     -5.835473412930    0.484799424424    0.270966519562
O     -4.237034738891    1.847543728500    1.640809119754
H     -3.638462188508   -2.534122159800   -0.905402412299
H     -3.751625283023    1.774674808095    0.809652375092
O     -4.261062256603   -3.750229590443    1.797337718878
H     -3.892212794301   -1.998202293932    2.219403752174
H     -5.012183701354   -4.275250447164    2.075124068097
O     -4.536778965328   -2.849056170537   -0.745742488686
H     -5.071946885038   -2.049638995554   -0.633200712430
H     -4.389158000000   -3.568541555799    0.841091914778
C     -0.562687899991    1.232375549648    1.189909390077
C      0.631837793954    1.603796549532    0.643024664052
C      1.666522990407    0.708030718217    0.200730979754
C      1.557876930307   -0.675354166937    0.226864553845
C      0.083127230294   -1.124496539597   -1.272697173196
C     -1.163334147770   -0.771887472615   -0.740510615347
C     -1.757046059741    0.553014067663   -0.969816102532
O     -2.926849685114    0.872921596335   -0.712537553972
H      0.782157340928    2.658802972138    0.407986617107
H     -1.320114483106    1.966159579129    1.431772592166
H      2.480666320900    1.149973617802   -0.372580300679
H      2.371724671900   -1.281139887902   -0.155817847600
H      0.972480931824   -1.169872055206    0.994089998517
H      0.508533342639   -0.523887172890   -2.065003436254
H      0.360767934396   -2.171911129501   -1.311230844445
H     -0.744559824654    0.249897890394    1.603167697112
H     -1.136500305544    1.253385102372   -1.568081288683
H     -2.565553926216   -1.309756548351    0.708508408649
N     -1.905800798740   -1.673422061020    0.009020680597
H     -1.459198716871   -2.530783029019    0.296920653468

Ambimodal TS Water5-117
35
ANGSTROM
O     -3.220467708341    2.735356016581    1.795535929008
H     -2.950242461261    2.465624181446    0.905148124983
H     -4.137404100111    3.043344600808    1.708931212289
O     -5.294275555608    0.817807371232   -0.063367684198
H     -4.433930906204    1.036721032641   -0.453823396796
H     -5.665623261431    1.656330720954    0.245031267216
O     -5.997931068472    3.025090988524    1.581612325816
H     -6.163685091995    2.367614609804    2.298542732896
H     -6.504529488950    3.805466816882    1.807066736418
O     -4.023023816242    0.194868829664    2.220039942593
H     -3.593187100905    1.077263347239    2.273356244217
H     -4.556725970470    0.243183784267    1.393484077437
O     -6.293400922678    1.055023826033    3.385264101943
H     -6.955885928239    0.405220620179    3.144535752849
H     -5.424088360572    0.647212906279    3.168222852055
```

```
C      0.043697712916    1.556065790566    0.978782865045
C      1.186774578903    1.523677928786    0.235421639507
C      1.941026772421    0.341019177550   -0.093474812404
C      1.572352106843   -0.941088638617    0.276197929120
C     -0.208666681436   -1.360321076100   -0.915891484141
C     -1.255734269449   -0.628990016260   -0.341973302177
C     -1.581091419447    0.725099034745   -0.822830886969
O     -2.601026298080    1.372287751001   -0.543052634985
H      1.510419623847    2.444985692386   -0.252363899701
H     -0.504263293408    2.475293139371    1.134988652901
H      2.725168720233    0.457631874767   -0.840991617777
H      2.175277159020   -1.784923958651   -0.039567907510
H      1.014897956953   -1.118494215245    1.188375000300
H      0.202734751466   -1.042737585544   -1.863029859852
H     -0.146197976629   -2.427087687567   -0.736699804171
H     -0.268964969630    0.738989047482    1.613554546461
H     -0.925947739497    1.101910648651   -1.637564872499
H     -2.688159812915   -0.591881151701    1.198681730486
N     -1.981284730095   -1.133388932210    0.706690669496
H     -1.808239950478   -2.060269784041    1.052135857378

Ambimodal TS Water5-118
35
ANGSTROM
O     -3.600033413963    1.034691730343    1.753123510638
H     -3.232219587317    1.584674165960    1.042747816827
H     -4.562857408356    1.107838272007    1.672466720541
O     -4.985926332711    1.197405483349   -1.438563043932
H     -4.066860638090    1.451810180834   -1.258261549793
H     -5.392566839247    1.931929978366   -1.932691127370
O     -6.629974979156    3.188702459841   -2.352345052867
H     -7.354068168706    2.848153350225   -1.785702295578
H     -7.016608356005    3.359975212179   -3.211375004425
O     -6.183086093369    1.742090255862    0.837238563223
H     -6.002796280680    2.683013140089    0.903610547215
H     -5.746174671847    1.442116574456   -0.007935217909
O     -8.416031756445    1.943513201595   -0.704766387277
H     -8.632348293781    1.061148397831   -1.011147359917
H     -7.760315634073    1.822562101035    0.013123959149
C      0.053351173115    1.483233945646    1.089664472725
C      1.275335921849    1.365649036740    0.494447262840
C      1.918882890116    0.130086605424    0.128091750850
C      1.348772066672   -1.119976005980    0.292627878561
C     -0.286295506441   -1.157532812447   -1.166221973718
C     -1.310709938804   -0.377986233708   -0.618017685180
C     -1.424241544398    1.059498255864   -0.931258638502
O     -2.394972222778    1.783990597901   -0.660424492010
H      1.771345118421    2.277231989401    0.155760635667
H     -0.392279968807    2.452498998556    1.269331273454
H      2.808475445606    0.213630786824   -0.495497265573
H      1.881355049615   -2.002023316219   -0.044202129245
H      0.648480236182   -1.311751365016    1.097018053612
H      0.283385575777   -0.777539619747   -2.002024482750
H     -0.364525404610   -2.237652385806   -1.132422662511
H     -0.443017613090    0.665743251007    1.593102108370
H     -0.648702836591    1.454202422016   -1.621156405152
```

```
H     -2.841912286491   -0.316113941867    0.798775260849
N     -2.238920505778   -0.916664911955    0.236907200467
H     -2.167738429075   -1.877534826705    0.522413364983

Ambimodal TS Water5-119
35
ANGSTROM
O     -3.570941887896    2.681994281663    1.699274432098
H     -3.216484073471    2.279507249790    0.882661072331
H     -3.159465369714    3.542034241971    1.787399115215
O     -5.293758589897    0.495783956183   -0.362730331341
H     -4.484153740098    0.887380800465   -0.712350385663
H     -5.823330843648    1.248067829695   -0.012764147663
O     -6.341908046548    2.471655451612    1.128220178061
H     -6.688901962099    1.910636470494    1.845491664974
H     -5.460912902741    2.744893360759    1.424944093063
O     -4.267023539398   -0.036194596746    2.025633516822
H     -3.994270417103    0.867145376348    2.235139094152
H     -4.685214812856    0.027789030243    1.127809633390
O     -6.805679846830    0.454199789542    2.967113366148
H     -7.391475207036   -0.209999088441    2.600941602964
H     -5.896249703240    0.133228231593    2.809096184409
C      0.143389232429    1.670972233156    0.821893386824
C      1.263172835705    1.553249438285    0.052594008628
C      1.988697543680    0.334055314879   -0.197548724225
C      1.611197507356   -0.906863838019    0.283001491741
C     -0.210541159596   -1.377167465518   -0.840708468882
C     -1.231524148443   -0.621223953316   -0.255842252058
C     -1.583602793635    0.707413684610   -0.787906177156
O     -2.579383914119    1.378669266555   -0.475455063515
H      1.587390723987    2.424548246092   -0.519541734925
H     -0.377708245781    2.615679648960    0.905150271725
H      2.756136964362    0.378053572185   -0.969696557808
H      2.191509397164   -1.783632492197    0.019368906740
H      1.066685778963   -1.004145221790    1.214739133290
H      0.168842640015   -1.098026987230   -1.812831415033
H     -0.133771313113   -2.433965028547   -0.615747993244
H     -0.169335529813    0.920078195372    1.533364131515
H     -0.991820756435    1.028745448268   -1.671825987564
H     -2.695815885044   -0.583865329714    1.248357079688
N     -1.867068502603   -1.052552931198    0.883647681665
H     -1.749455944211   -2.003355811765    1.189048868021

Ambimodal TS Water5-120
35
ANGSTROM
O     -2.878914677517    2.821355326401    2.195241077675
H     -2.795754925801    2.495807921276    1.286591979531
H     -3.838264659608    2.806800648523    2.387515784273
O     -4.894222529111    0.142486016430    0.785892686564
H     -4.043454859390    0.489571943236    0.467120546059
H     -5.458264428399    0.913662670961    0.895480799140
O     -5.491080213179    2.368497258788    2.832798182752
H     -5.306501285660    1.455985962287    3.172926330475
H     -5.939828678208    2.834156152052    3.538283132736
O     -2.088761015244    0.628903906925    3.416787819944
```

```
H     -1.631220487433    0.858186168069    4.225663341951
H     -2.328838082644    1.493722851872    2.982533190235
O     -4.748601118700   -0.105590886261    3.494723776051
H     -4.859980506516   -0.344114382734    2.555340525278
H     -3.790625690290    0.026671060711    3.605022252330
C      0.360288677129    1.592271687344    0.872035336178
C      1.347725872856    1.534279701668   -0.068155451310
C      2.049987879874    0.345657157265   -0.475929706005
C      1.776818730636   -0.922776617191    0.011359384861
C     -0.173524558340   -1.414636771143   -0.804825549736
C     -1.107393059186   -0.662666300401   -0.083090249950
C     -1.552343415238    0.663667731157   -0.541206795136
O     -2.520139779903    1.287061653373   -0.080328519010
H      1.555598052717    2.433320862342   -0.650928393436
H     -0.172068975059    2.511793562920    1.075656178700
H      2.680056544996    0.433392688534   -1.360281988991
H      2.328649802489   -1.773352161276   -0.372390138849
H      1.408781338625   -1.062072119824    1.021398464102
H      0.043102592410   -1.146474082524   -1.829275840713
H     -0.064410321355   -2.470914829836   -0.588149002717
H      0.190088654457    0.807503240293    1.595664468125
H     -1.078761436480    1.032874732574   -1.474715696556
H     -2.090263596537   -0.488591086009    1.738144774704
N     -1.649577825191   -1.128780099875    1.092268138453
H     -1.338479274278   -1.999291382006    1.486725345675

Ambimodal TS Water5-121
35
ANGSTROM
O     -3.328910012481    2.742466093704    1.735631693116
H     -3.014722629708    2.459348115963    0.863081313952
H     -4.233011251286    3.057101715531    1.595796843478
O     -5.295726585851    0.667010041395   -0.084972642007
H     -4.443310766698    0.928312757103   -0.464374213169
H     -5.698768417473    1.484003998142    0.251712854720
O     -6.147151546435    2.862577109782    1.439699246189
H     -6.200463900064    2.320360258203    2.262542227832
H     -6.990293428338    3.307692967626    1.353423967662
O     -4.032728402530    0.174665006404    2.225095772839
H     -3.634373101192    1.072969553914    2.203845153411
H     -4.593966989252    0.146597053786    1.415975578148
O     -6.008765732132    1.330775795340    3.642922744392
H     -5.571622407570    1.762230169437    4.379053186419
H     -5.340497543881    0.723592226058    3.250459285757
C      0.036986117936    1.577514012524    0.958854542480
C      1.178606452222    1.542414838784    0.213322105673
C      1.939977388250    0.360672987408   -0.102270974083
C      1.581415722465   -0.919255373804    0.284221388808
C     -0.199673500375   -1.367982526438   -0.898874167900
C     -1.248959525161   -0.639743834321   -0.325598529226
C     -1.587840001540    0.707271422399   -0.818109187461
O     -2.610908724380    1.348965433545   -0.537464350121
H      1.495016960408    2.459378707537   -0.287272716226
H     -0.516427923432    2.495747266245    1.102360306414
H      2.721642439211    0.473560008127   -0.852915393652
H      2.189788742841   -1.762480344542   -0.022499761368
```

```
H      1.026257603660   -1.089609031545    1.199130990057
H      0.204495858513   -1.055604944416   -1.850828008706
H     -0.126510074151   -2.432319131883   -0.709732213504
H     -0.269569368648    0.767039892741    1.604916537598
H     -0.942543675903    1.079869351741   -1.642579738282
H     -2.685574940071   -0.604530815603    1.210561126443
N     -1.954211891576   -1.133479335862    0.741361831479
H     -1.797056828597   -2.068100283668    1.073282476715

Ambimodal TS Water5-122
35
ANGSTROM
O     -4.586935895235    0.692957357556    1.027809976658
H     -3.918386993878    1.091474570780    0.428790141124
H     -5.442965807627    1.018609331797    0.719768308897
O     -5.106070552461   -1.759935088509    0.173489184083
H     -4.419912693042   -1.944987991585   -0.471601590345
H     -4.863701252114   -0.879878984875    0.566356774214
O     -7.126344559681    0.255229735200   -0.186551422987
H     -6.673685814505   -0.598052098105   -0.251297534700
H     -7.999788064749    0.077090784943    0.164075969742
O     -4.535521174105   -3.335748200633    2.304148864751
H     -4.789587476847   -2.798849436893    3.056552823726
H     -4.828248685221   -2.842208648448    1.508063050891
O     -1.869588979167   -3.573225033668    1.878466402184
H     -1.672311590829   -4.491845782671    1.692216252741
H     -2.822538013598   -3.544245454158    2.108622045941
C      0.022028481991    1.434971861220    1.004928222524
C      1.198796044899    1.403500186838    0.315813318350
C      1.923376681866    0.213760509375   -0.044937211544
C      1.475943231118   -1.073415956543    0.215269013146
C     -0.238039499184   -1.322807062696   -1.067737235007
C     -1.285276712469   -0.577485906162   -0.514295432345
C     -1.568115371471    0.810656737986   -0.928432831806
O     -2.579473903790    1.454579455268   -0.628841897961
H      1.575394064454    2.338424828346   -0.102815247829
H     -0.510031369239    2.361190288725    1.175008729190
H      2.750643020866    0.341874390601   -0.742325507835
H      2.062521776430   -1.918814376183   -0.127088323218
H      0.874630568110   -1.290751556438    1.090425369905
H      0.230122269891   -0.980799643398   -1.980365461130
H     -0.237731075826   -2.397882537355   -0.935169141832
H     -0.341751711058    0.594950849296    1.579945413255
H     -0.873280245892    1.229112146804   -1.688452982625
H     -2.693618578835   -0.503638648085    0.981310947952
N     -2.125198615818   -1.129360941410    0.431585682409
H     -1.883673392425   -2.006067139828    0.882672499299

Ambimodal TS Water5-123
35
ANGSTROM
O     -3.238500588896    2.864070058926    1.595079625746
H     -2.958430835200    2.646806850814    0.693326456106
H     -4.129562807935    3.256755728910    1.508600859385
O     -6.606948332077    0.670225395970    0.295339216473
H     -6.880073313628    0.703520559379    1.222202828834
```

```
H     -7.205297944508    0.073316492431   -0.154421893745
O     -5.922380092998    3.299383512585    1.331495909203
H     -6.196480002786    2.742124976589    2.077239492181
H     -6.093241346472    2.765988729283    0.547445757717
O     -4.143042218175    0.387135946049    1.802898671880
H     -3.696689071126    1.275021271360    1.832566768516
H     -4.651876172711    0.405277746049    0.979773643460
O     -6.358587587810    1.138357110071    3.105165565705
H     -6.286898983743    1.240747308007    4.054218985333
H     -5.491895524672    0.781994048589    2.793774276469
C      0.045329540187    1.526497174065    1.004475198790
C      1.211781991793    1.507711340585    0.296152445676
C      1.969411574857    0.331420933427   -0.033645547431
C      1.565037412679   -0.960218221857    0.286213825735
C     -0.143575393696   -1.336822298696   -0.931372788943
C     -1.218436374215   -0.601816359750   -0.407672052278
C     -1.541585846846    0.756346269499   -0.888305576160
O     -2.564855121716    1.386647158833   -0.614441963216
H      1.553079007663    2.439348204588   -0.158688596175
H     -0.510905113189    2.440621282063    1.159269021090
H      2.776474646514    0.455184543034   -0.754660006342
H      2.178951504185   -1.799025599185   -0.024110954514
H      1.002228500545   -1.149448554781    1.193533200816
H      0.292951967746   -1.023547375568   -1.869505155471
H     -0.112426679857   -2.407922340190   -0.765263700020
H     -0.287243879505    0.696216722543    1.610883484744
H     -0.858748913346    1.147432295838   -1.676693571045
H     -2.731690181050   -0.539205229293    1.028568940887
N     -2.006976362845   -1.106931731257    0.596180454244
H     -1.841996590162   -2.019032296786    0.981884341640

Ambimodal TS Water5-124
35
ANGSTROM
O     -3.804369474811    0.836701391646    1.896996419789
H     -3.540157992992    1.130599062278    1.001350648804
H     -3.274507058749    1.380081473441    2.498584379364
O     -3.289918816533    3.868014845151    0.787369806861
H     -3.051450826350    3.185421206207    0.146252571013
H     -2.857693261760    3.616020661809    1.619120639256
O     -2.421622104826    2.861995788480    3.295877352278
H     -3.249132032407    3.163987812351    3.747383409406
H     -1.694595804900    3.243168523917    3.788587432591
O     -5.637176418586    2.980575018622    1.893346670042
H     -5.303967306748    2.068473949605    1.875501663349
H     -4.985867095850    3.488540604214    1.370434441739
O     -4.769683767868    3.559936657069    4.322343443768
H     -5.233222756236    2.968918528600    4.917202230999
H     -5.219650442379    3.465488023176    3.441831456725
C      0.172694093907    1.623134241508    0.877292522756
C      1.281722279172    1.518634217202    0.088617594230
C      1.990543548524    0.301691565408   -0.206535868259
C      1.600706965875   -0.951332785271    0.240734587728
C     -0.224157218065   -1.371609747750   -0.848623312219
C     -1.230316375566   -0.603409900505   -0.250406420314
C     -1.562852085580    0.740625836510   -0.757106261825
```

```
O     -2.544135235193    1.421738721771   -0.424930345252
H      1.603890083254    2.403257636526   -0.463463334251
H     -0.344400771232    2.566212690097    0.993617404457
H      2.746531597768    0.357853589445   -0.988978069450
H      2.171191552338   -1.824727499138   -0.054695353079
H      1.082023493393   -1.067532164801    1.185402253470
H      0.142135001001   -1.093543486592   -1.826379302203
H     -0.172903770470   -2.433623174261   -0.639140228433
H     -0.130542621624    0.856008535681    1.575801250505
H     -0.967004600619    1.069562664820   -1.634024177869
H     -2.637998893406   -0.514339063699    1.293242120257
N     -1.876132607353   -1.046522746337    0.881846616654
H     -1.776982966990   -1.999742195714    1.183113367157

Ambimodal TS Water5-125
35
ANGSTROM
O     -3.999170199817    3.774170883987   -0.020843498745
H     -3.537774021002    3.014977546175   -0.406387204604
H     -4.634477179264    3.394399693009    0.607377973623
O     -4.647419672593   -0.030443940204    0.773602989138
H     -4.204710447011    0.502088694579    0.098813190630
H     -5.119582665513    0.613543710050    1.318597216544
O     -5.276654920465    2.398250558157    2.101738680905
H     -4.327714291040    2.250608393957    2.353156062687
H     -5.730654978930    2.684685776445    2.893868373078
O     -1.974907652301    4.529799246796    1.562087079400
H     -2.127801145989    5.398310414742    1.934653060675
H     -2.695034928551    4.381146329630    0.902843709603
O     -2.679374558841    2.023963998739    2.388079129411
H     -2.590076927366    1.646112728856    1.499881435408
H     -2.300159160361    2.918160317931    2.308522597172
C      0.185062929083    1.729755532624    0.720501250919
C      1.310967894810    1.544882796104   -0.028824179132
C      2.015050860034    0.299932138697   -0.193921235093
C      1.600098433316   -0.904884070139    0.347494633325
C     -0.195240975131   -1.374380409209   -0.799410890854
C     -1.228041054872   -0.606657660141   -0.254837326009
C     -1.568942376205    0.721634674685   -0.785398831922
O     -2.559654693562    1.387654668227   -0.428023093215
H      1.658561588866    2.373269304123   -0.648838343473
H     -0.313681377160    2.690263061180    0.747674098503
H      2.799242697286    0.282117439789   -0.950861447493
H      2.167431000436   -1.806783756209    0.146825270618
H      1.035055477167   -0.937999973700    1.271356282000
H      0.211546918203   -1.109020329046   -1.764382737410
H     -0.132692908650   -2.429171442632   -0.560828644793
H     -0.136270177804    1.028062867189    1.478487684283
H     -1.015014742535    1.042712499220   -1.689774436512
H     -2.812908709166   -0.606560999853    1.084534227921
N     -1.904119367505   -1.015976825604    0.881013760218
H     -1.812359466452   -1.973638882403    1.177359987410

Ambimodal TS Water5-126
35
ANGSTROM
```

```
O      -3.525315574257    2.942993566636    1.402320800538
H      -3.168751471359    2.696096961919    0.536039130798
H      -4.492831781559    3.011473916540    1.272873385094
O      -5.139949998992    0.632610065298   -0.895323310103
H      -4.209880743060    0.916502641374   -0.879011934328
H      -5.653993037809    1.445247245815   -0.879613282403
O      -6.225885282705    2.709734460654    1.106550531649
H      -6.227522358331    1.755326367742    1.374012546800
H      -6.849644994193    3.152558428719    1.681661960936
O      -3.336786885108    0.576031812889    2.536036170969
H      -3.158079812524    0.680095721172    3.470609749264
H      -3.359514216721    1.497762509856    2.156533967412
O      -5.916409656437    0.120755906050    1.665072848409
H      -5.726775108381   -0.016706175943    0.718033967045
H      -5.042218438181    0.140938066265    2.092575135609
C      -0.123446295341    1.508579554268    1.050594488406
C       1.113019566252    1.434606523791    0.477851877377
C       1.827103296258    0.221750378540    0.175627250623
C       1.321472305689   -1.050658539678    0.390822720523
C      -0.277827125438   -1.257344476095   -1.062258624979
C      -1.343830736051   -0.481612817947   -0.592845653378
C      -1.519722207425    0.920802562604   -1.004314791747
O      -2.540178970567    1.596950316225   -0.806449390981
H       1.563376975495    2.356960087863    0.106971534773
H      -0.628582464502    2.456062902407    1.182879578720
H       2.718783019716    0.324408863528   -0.441731205217
H       1.909289667448   -1.915232156018    0.104041978984
H       0.631260430390   -1.243517080132    1.204116521294
H       0.283522831227   -0.919660323981   -1.922021695747
H      -0.319522970723   -2.334745514204   -0.952502380531
H      -0.577098974908    0.682370441318    1.579788598815
H      -0.739682645711    1.323139471096   -1.683796837772
H      -2.859574977952   -0.374795299116    0.822902936021
N      -2.275138335453   -0.995105266534    0.279900132248
H      -2.172788425322   -1.926239945252    0.644154701250

Ambimodal TS Water5-127
35
ANGSTROM
O      -2.266510476550   -0.083144732238    4.097244797781
H      -2.676570935897    0.519468420257    3.455887613563
H      -2.766118906994   -0.916566330558    4.020914580332
O      -3.529374047535    1.260833371317    2.038877765258
H      -3.145636131686    1.488978085027    1.159769021285
H      -4.441088782569    1.551937041968    2.017104833179
O      -3.296701285084   -2.611608809616    3.692840441288
H      -2.513153647200   -2.948829484917    3.225930524985
H      -3.456763483790   -3.204739637883    4.427917171988
O       0.114673974782   -0.918254770000    3.381603439423
H       0.680488533493   -0.807793067873    4.146450644705
H      -0.764649997930   -0.522991270050    3.638435145253
O      -0.868569906152   -3.243843001161    2.526090584021
H      -0.194206884172   -3.922996527863    2.499191044902
H      -0.429334812227   -2.417856567368    2.840320296274
C       0.310007463558    1.599283886804    0.886373058197
C       1.401030946698    1.477667131522    0.076813369501
```

```
C      2.063868546027    0.239726627500   -0.243466896370
C      1.628381761049   -0.998621333554    0.197167105921
C     -0.233355511688   -1.326808366487   -0.896466986975
C     -1.212346014469   -0.536208506444   -0.284747301239
C     -1.490168814273    0.838955361437   -0.747043410492
O     -2.427586765968    1.556818614756   -0.373305470238
H      1.744527091865    2.358864562152   -0.469083762218
H     -0.181033594455    2.552242734518    1.032006986234
H      2.811477525439    0.278511640682   -1.035927743846
H      2.155630565530   -1.893408475002   -0.115575407062
H      1.108787723764   -1.096417728496    1.142140645204
H      0.153524540750   -1.041575712563   -1.863967157105
H     -0.224500108669   -2.392416433979   -0.703716859690
H      0.004083579658    0.822470185391    1.571929719520
H     -0.894496417722    1.165379284751   -1.626854068079
H     -2.561257265441   -0.405594515677    1.285783890357
N     -1.944489475910   -1.014312521872    0.771458992962
H     -1.732764977855   -1.906925470613    1.186436926107

Ambimodal TS Water5-128
35
ANGSTROM
O     -3.577974818399    1.504560505269    1.975055021356
H     -3.227598946053    1.613664127329    1.058875250853
H     -4.091095133121    2.291841001568    2.158771578327
O     -1.992172961903   -2.194836472201    3.399617461668
H     -1.795365280811   -1.218435409481    3.411746429804
H     -2.939270145195   -2.268533299156    3.533210942261
O     -2.063785118398    0.166382759034    6.242749499079
H     -1.764087647831   -0.766683492530    6.250579449308
H     -1.621833120905    0.599252662233    6.973817721577
O     -1.605502821047    0.408859110886    3.557805488115
H     -1.792596228947    0.507182265711    4.509409302188
H     -2.321212485724    0.871034698547    3.085249327216
O     -1.041991771250   -2.343884433930    5.934046969276
H     -1.354942924344   -2.448683026163    5.009303221231
H     -0.085718435117   -2.395071440491    5.895389794726
C      0.177088543106    1.620408766717    0.930095942974
C      1.339866975281    1.391846563403    0.253673019509
C      1.969447805310    0.108973028803    0.085709883691
C      1.418190175584   -1.082952600751    0.529634942435
C     -0.299150516600   -1.361820725426   -0.763079662972
C     -1.314495182174   -0.534695109222   -0.272133294926
C     -1.531717506932    0.834531805258   -0.777933559823
O     -2.475075316307    1.575111239267   -0.465349512941
H      1.783245178019    2.216844942394   -0.307926893092
H     -0.270792246790    2.605361429072    0.951993576696
H      2.805981098889    0.064401732360   -0.611503310536
H      1.933064555871   -2.016336484093    0.330457208209
H      0.788580049295   -1.110715480204    1.411112749476
H      0.193100789531   -1.104544671390   -1.690166773028
H     -0.341060166329   -2.423908741059   -0.555505094825
H     -0.238260338609    0.921840756424    1.642265227273
H     -0.880465188971    1.134717897507   -1.626510231361
H     -2.787350596039   -0.316465809747    1.152945259819
N     -2.166302434870   -0.977268968630    0.714851758046
```

```
H     -1.944900165655   -1.796548789735    1.259487617014

Ambimodal TS Water5-129
35
ANGSTROM
O     -1.312330335714    0.493404050998    3.864962055534
H     -0.798065320650    1.242881849355    4.168842695717
H     -2.043594808658    0.874334775559    3.325595961156
O     -3.112943530510    1.708734899422    2.291283631713
H     -3.049427116454    1.531050419592    1.335962689009
H     -2.810777166499    2.629904309246    2.383221861184
O     -2.134829195707    4.271563490967    2.078161888202
H     -2.327809609131    4.291608580625    1.115995739251
H     -2.482780419661    5.086282380739    2.442022808980
O     -0.190819691951   -1.837827362722    3.003813155268
H     -0.149457143052   -2.429425003229    3.755957458941
H     -0.571714251435   -1.003395214736    3.343239078657
O     -2.879225372847    4.037715552951   -0.515995697028
H     -2.732070660759    3.070110302774   -0.580616387150
H     -3.813098858140    4.168517525875   -0.689657893226
C      0.184484335674    1.610941345865    0.841520126831
C      1.271738246207    1.531843589651    0.025277362800
C      2.007319415180    0.324919797507   -0.270037072897
C      1.683490382609   -0.924693008613    0.215045708842
C     -0.207936344793   -1.398647860288   -0.861974010612
C     -1.212368143437   -0.639087396884   -0.265025284687
C     -1.576149581171    0.687994047317   -0.773984304295
O     -2.555216217694    1.359230313476   -0.392045110247
H      1.557686634063    2.417003532190   -0.546145887356
H     -0.352292275502    2.540701524247    0.979214683310
H      2.744683502389    0.396449165756   -1.070656494229
H      2.261460480972   -1.787111349590   -0.097479330260
H      1.163516990675   -1.062451486661    1.155176525200
H      0.160780174532   -1.129320076898   -1.841127365418
H     -0.107830597043   -2.444121715867   -0.603837052604
H     -0.070881449803    0.826631955241    1.540473939708
H     -1.023824712578    1.032623147889   -1.672313503729
H     -2.500911575048   -0.460814972619    1.326579060254
N     -1.913774839362   -1.112328638446    0.828307041719
H     -1.478977089082   -1.808835881608    1.418188367286

Ambimodal TS Water5-130
35
ANGSTROM
O     -2.139192661939    2.357185084426    3.557821178327
H     -1.848195980039    3.082742546894    2.962596406909
H     -2.669473410770    2.771592669979    4.240441937507
O     -1.595865796651    4.416316013138    1.898682229567
H     -2.276845200682    4.311400519173    1.182471086597
H     -1.673756257672    5.321490800974    2.201476852118
O     -5.474515038521    3.011113287106    1.697210046259
H     -4.952493932703    2.195675177926    1.878145963434
H     -6.282394060072    2.724222554448    1.270527245422
O     -3.751537206310    0.962094493642    1.906383072291
H     -3.146237151959    1.374959713790    2.559585014519
H     -3.391948874010    1.215839760983    1.029531677669
```

```
O      -3.477750384860    4.016893465115    0.091559085414
H      -4.252020167435    3.787128599462    0.641926838578
H      -3.182184574209    3.184640997428   -0.307228333318
C       0.159921278423    1.639461787674    0.842626486837
C       1.275015676108    1.518686673194    0.063330539438
C       1.987923110891    0.296517960919   -0.202561019596
C       1.601170632995   -0.945608705387    0.270548233258
C      -0.231663851622   -1.376681596492   -0.840339373726
C      -1.236350874266   -0.598696708891   -0.255989044092
C      -1.554854027769    0.743430837624   -0.771531790716
O      -2.543752044913    1.427638140078   -0.455893574481
H       1.600647956279    2.391644560175   -0.505251090468
H      -0.347034807927    2.589109062092    0.948263277966
H       2.750483401714    0.337440575991   -0.980274125438
H       2.170157014096   -1.826756514793   -0.003888043226
H       1.067043924196   -1.042668952591    1.208322739555
H       0.150814353172   -1.106665904274   -1.813763048741
H      -0.181713659052   -2.435848357278   -0.618135910888
H      -0.140377105760    0.889176303420    1.561191664153
H      -0.959632395624    1.061807335610   -1.651489259018
H      -2.642260830330   -0.489000206249    1.291940884252
N      -1.910129118690   -1.040728937792    0.859611256926
H      -1.773394778737   -1.972629330649    1.206931390223

Ambimodal TS Water5-131
35
ANGSTROM
O      -4.012680085921    2.957560307720    0.320852789838
H      -4.397040557940    3.766516674017   -0.018865792783
H      -3.132332273695    2.872422468542   -0.126280471034
O      -6.169545726920    1.670047220969    3.448471481956
H      -5.253003635341    2.033529990082    3.456505558086
H      -6.741872489246    2.372639389398    3.756297846143
O      -3.248859985811   -0.222418400151    2.452706233501
H      -4.031676686789   -0.031373134793    1.881736066586
H      -3.591081310196   -0.703667457857    3.207507898476
O      -5.382346815453    0.606542662419    1.049360838684
H      -5.060917054156    1.450023943135    0.694660358144
H      -5.898587092264    0.849442258336    1.838436104124
O      -3.639073490050    2.482326372272    3.063908333237
H      -3.261447072110    1.589178316515    2.992356874863
H      -3.705753350080    2.797501553163    2.147721600840
C      -0.461200788239    1.636895707332    1.686266840398
C       0.823000387376    1.175070136623    1.710875502776
C       1.209310021416   -0.197553519357    1.516317460801
C       0.313870782746   -1.211705009080    1.220203248837
C      -0.328866342390   -0.736591197194   -0.810231550366
C      -1.259780962218    0.299878289091   -0.707497397353
C      -0.870812575142    1.718085639820   -0.802397470858
O      -1.656445002119    2.677813613221   -0.852252597569
H       1.637620660603    1.899642332059    1.767845018240
H      -0.681361519903    2.691961780619    1.781800243706
H       2.274922027674   -0.393132736279    1.396635523241
H       0.680528126984   -2.217518653846    1.047898267799
H      -0.709647685117   -1.169925894811    1.573480327534
H       0.656139454290   -0.529135984879   -1.202937388477
```

```
H     -0.679194538278   -1.746287283612   -0.984652153985
H     -1.312193937006    0.979930838261    1.796468743014
H      0.201118631813    1.903264223311   -1.023051107442
H     -3.223107421010    0.788672800675   -0.282970690845
N     -2.598552369801    0.035975742869   -0.517733749697
H     -2.912715914186   -0.881759949218   -0.256178073593

Ambimodal TS Water5-132
35
ANGSTROM
O     -1.660593773244    1.502589450676    3.529629686496
H     -1.708835414994    2.476456111550    3.352059342520
H     -1.727589952184    1.407375695136    4.480267183698
O     -2.147017019229    4.059918686546    3.021608627000
H     -3.095454092353    4.101323285735    3.197012507155
H     -2.095395999894    4.177013027872    2.051998340000
O     -4.858031066229    3.481692251043    2.258388954107
H     -4.564691816908    2.542014814460    2.182856773147
H     -5.814704002603    3.465039752163    2.269689384582
O     -3.795640616747    1.040315482992    1.931634796759
H     -3.039161363037    1.146515199959    2.546496352122
H     -3.442067141631    1.255412428899    1.043781507100
O     -2.773082742063    4.083492827612    0.378483465136
H     -2.678656630421    3.226828621658   -0.065953304485
H     -3.657923778791    4.073618521632    0.770373967684
C      0.152205904707    1.702456095963    0.758663299652
C      1.290372092378    1.527911363860    0.023371041970
C      1.995337859096    0.286801670457   -0.156940809581
C      1.567118902058   -0.928415412977    0.350197192201
C     -0.217007936887   -1.365639133845   -0.833513567847
C     -1.242768062128   -0.595364580754   -0.278657649407
C     -1.560430809360    0.750426398695   -0.784169006276
O     -2.546413147479    1.430713181597   -0.446186716026
H      1.644841096854    2.366134866418   -0.579677506332
H     -0.344299089172    2.663677050789    0.784965306175
H      2.790598207697    0.282999742350   -0.902350295008
H      2.133167587845   -1.828633948360    0.138600862458
H      0.989655917810   -0.977069796802    1.265770612007
H      0.196694184618   -1.088676303480   -1.792073754499
H     -0.168009073202   -2.425255942452   -0.613944747395
H     -0.180004696753    1.005898056821    1.515895789179
H     -0.980382116849    1.073080558916   -1.672096856490
H     -2.700981590339   -0.502708126086    1.212199177298
N     -1.947131970692   -1.045086780542    0.814720673566
H     -1.807307037086   -1.970584601214    1.176894565025

Ambimodal TS Water5-133
35
ANGSTROM
O     -2.603571078201    2.569131257563    3.275317897277
H     -2.468082188050    3.103173019614    2.453152110493
H     -3.077956268828    3.143373058296    3.877956150857
O     -2.601591416143    3.861642938460    0.948879926202
H     -3.527287753592    4.170168614396    1.010855146435
H     -2.610957562752    3.138256494315    0.304244894440
O     -6.307287068469    1.976523936437    1.853279512935
```

```
H     -5.489910217190    1.424982843905    1.808856492594
H     -6.669169582440    1.848329879391    2.730841508371
O     -3.935202940514    0.751176624336    1.805340795350
H     -3.457156491579    1.303582350750    2.462270320307
H     -3.610387410043    1.075048926584    0.939282284624
O     -5.274290188414    4.382750190770    1.183195124317
H     -5.685545323223    3.529484244161    1.441564003908
H     -5.726017137678    4.660488035470    0.385488768646
C      0.156720413652    1.620163884517    0.885640295331
C      1.271813060462    1.513112580240    0.105210937600
C      1.981632813755    0.295269211318   -0.187084467470
C      1.587601636151   -0.957265410265    0.253624169388
C     -0.235934282392   -1.360660081833   -0.867163553562
C     -1.244158809969   -0.599799024376   -0.264553065965
C     -1.569075780485    0.751133081864   -0.751718246828
O     -2.557916831495    1.428527197142   -0.423040543541
H      1.601079153723    2.397772179666   -0.442930626750
H     -0.353838545120    2.566373003816    1.003236272193
H      2.746597985473    0.352391554767   -0.961263511777
H      2.156951598451   -1.832353288827   -0.039108840481
H      1.052510937042   -1.074955729196    1.188605764491
H      0.143040894320   -1.067247267781   -1.835367710042
H     -0.184559901651   -2.425078331709   -0.671430842625
H     -0.152003483457    0.854400726406    1.583584508278
H     -0.968663797016    1.091753887180   -1.620573507361
H     -2.664477501191   -0.538343055467    1.269095185426
N     -1.900767779577   -1.062003486009    0.853562371314
H     -1.789271291947   -2.014365018982    1.152301228920

Ambimodal TS Water5-134
35
ANGSTROM
O      0.125255406342   -3.489917444409    5.083303612634
H      0.966191286664   -3.425328769801    4.603170141022
H     -0.068136690370   -2.602051322798    5.395640932097
O      2.128494724421   -2.985581266166    3.144278522429
H      2.184430277386   -2.020724630364    3.325388863934
H      3.026485263975   -3.317075069461    3.159553143735
O     -0.667276927972   -0.719645165714    3.742348105612
H     -0.732715418582   -1.561880447481    3.241325575925
H     -1.357761704266   -0.147251505670    3.404208206685
O     -0.632448360761   -3.156128692353    2.496849153738
H     -0.723717435912   -3.577989729423    3.369675159856
H      0.316076796452   -3.212989389322    2.315934347760
O      2.012816224526   -0.350251372492    3.654034320230
H      1.046148363948   -0.349725273564    3.809034413686
H      2.183885766255    0.324540942922    2.994096690367
C      0.267836100312    1.567024465985    0.914495292484
C      1.360492815865    1.485468449063    0.099508187458
C      2.055365220898    0.276745399156   -0.241531219705
C      1.628563658630   -0.993779378262    0.155483826257
C     -0.151832271879   -1.346116691871   -0.849574777232
C     -1.173669452198   -0.585648289966   -0.259692918652
C     -1.602405463606    0.747842939254   -0.736702798268
O     -2.590399897447    1.337529403539   -0.310633995755
H      1.678287297644    2.387053856727   -0.426502288901
```

```
H     -0.261403636334    2.497826029893    1.061530433894
H      2.799104563390    0.346828969454   -1.033552024200
H      2.197299723273   -1.857623974676   -0.172982617992
H      1.161346160798   -1.136219531170    1.122910113563
H      0.180644972411   -1.071208736042   -1.841899963712
H     -0.133410203668   -2.412926138400   -0.654982969461
H     -0.026572404349    0.769970930736    1.580454776666
H     -1.046939558371    1.146064543220   -1.617018288247
H     -2.664222854083   -0.516178984888    1.126352489450
N     -1.827802090161   -1.022220346606    0.877781985776
H     -1.739958477614   -1.974072176248    1.198933333857

Ambimodal TS Water5-135
35
ANGSTROM
O     -2.748481716765    2.149998318334    3.687565429540
H     -2.908425635355    3.009575323758    3.212960898021
H     -3.269066330466    2.194870399294    4.490829045307
O     -2.820797313405    4.499789604371    2.498934774392
H     -2.749388452528    4.391926312018    1.528936147767
H     -1.932650075203    4.716271330968    2.810707498926
O     -0.327272109826    3.638172184549    3.669026588390
H      0.424693873443    3.208454964293    3.258779565393
H     -0.997124749613    2.949638837997    3.806901394364
O     -3.926910814772    0.463871415605    1.834106859861
H     -3.505533086776    0.991092305993    2.536259975759
H     -3.787185410591    0.966331864290    1.016668270990
O     -2.638657666876    4.038035563560   -0.178482176423
H     -3.394974797744    4.383258334339   -0.655324519892
H     -2.646618939846    3.069833915585   -0.322103490364
C      0.012495308053    1.631675399843    0.920292744423
C      1.172822694954    1.563403865215    0.205054717924
C      1.937898556820    0.370733889831   -0.055395145261
C      1.563221916571   -0.897063075382    0.350802069089
C     -0.197168311912   -1.355666229611   -0.888579470814
C     -1.254791866012   -0.648908577921   -0.305823038246
C     -1.605272021218    0.705440278703   -0.774727038637
O     -2.623725773035    1.344071989646   -0.461073501502
H      1.502593667842    2.462186050022   -0.319441599148
H     -0.537541181222    2.560251815684    0.997110900325
H      2.742115629218    0.462472242216   -0.784778662034
H      2.173535565219   -1.750745487359    0.079662249367
H      0.972168395914   -1.046684421131    1.246636384228
H      0.216836629133   -1.019767428780   -1.828088627939
H     -0.105573166883   -2.420640934071   -0.713393628436
H     -0.317414688848    0.851263748076    1.592280405913
H     -0.986255533143    1.083787834289   -1.615867041930
H     -2.720233400817   -0.640122669175    1.191794085061
N     -1.937244021521   -1.150025083168    0.770849660397
H     -1.799749904772   -2.100890115428    1.064274400172

Ambimodal TS Water5-136
35
ANGSTROM
O     -2.015829869749    2.234085471702    3.537779754121
H     -1.809831841976    3.004281959531    2.965820855381
```

```
H     -2.200009427503    2.585921734638    4.409155159695
O     -1.678140355096    4.392989087797    1.955408974859
H     -2.351376269005    4.270213427904    1.233720765013
H     -1.730983830732    5.315469162508    2.206391780279
O     -5.561892322718    2.952417446831    1.663954642438
H     -5.051802607876    2.121293912929    1.786746731041
H     -5.852689061783    3.225250112357    2.534392004007
O     -3.771809531623    0.946745746423    1.900673087749
H     -3.157863741722    1.341920651053    2.550129146990
H     -3.417257089184    1.206542803815    1.023716957699
O     -3.522965281063    3.991708059468    0.116132383965
H     -4.342664559711    3.743922398065    0.582224553287
H     -3.215495447790    3.177657397030   -0.309784474727
C      0.169584833708    1.644335189262    0.832553797001
C      1.286267507151    1.515867035608    0.056757814281
C      1.995213105751    0.290270446130   -0.201463787599
C      1.598875133861   -0.949367202470    0.273129588015
C     -0.224456132559   -1.377660868058   -0.837546325883
C     -1.231209436618   -0.599189920931   -0.255943384677
C     -1.551333991219    0.742800910256   -0.772326281686
O     -2.536419598576    1.428023773760   -0.451691262572
H      1.616752372426    2.384890565682   -0.515246016047
H     -0.337380348635    2.595064566191    0.928974411206
H      2.760441597936    0.324773613257   -0.976789948051
H      2.166886642774   -1.833131750808    0.004797485022
H      1.064107621004   -1.040242310010    1.211262834419
H      0.157330771327   -1.108926177046   -1.811740377002
H     -0.177880648443   -2.437388099983   -0.616630795887
H     -0.136459844570    0.899510903838    1.554097207539
H     -0.958064375319    1.059514928666   -1.654723256608
H     -2.648756758014   -0.492437310342    1.278513357214
N     -1.901107151153   -1.037141232671    0.863626970206
H     -1.780794506148   -1.976050676999    1.198457445566

Ambimodal TS Water5-137
35
ANGSTROM
O      0.017295809970   -0.655096000585    3.487589993303
H     -0.794797163714   -0.117054673481    3.703399293264
H      0.599789155598   -0.553941373954    4.241138794909
O     -2.215421073298    0.571063770145    4.106324132778
H     -2.778007372024   -0.214141078684    4.246954786525
H     -2.645000700494    1.058072433459    3.381672462167
O     -3.580103451464   -1.800680926409    3.914530127168
H     -2.822052112816   -2.308911118823    3.569019077878
H     -4.206073863131   -1.720314485588    3.192759402176
O     -1.271083649441   -2.915240131049    2.917160189673
H     -0.871028936312   -3.667765946448    3.354010543652
H     -0.714302036385   -2.128893173116    3.135108394534
O     -3.602576033134    1.568506472422    1.949240690899
H     -3.201080971209    1.669174692179    1.052487254719
H     -4.210747725374    2.300726030208    2.051374404699
C      0.325612955070    1.615774791704    0.803113872738
C      1.407082855025    1.385706003350    0.004023204506
C      2.006536091987    0.097482720211   -0.228704889098
C      1.511010137791   -1.085099252947    0.295240389858
```

```
C     -0.360562234942   -1.386983379752   -0.778184381146
C     -1.299295917517   -0.511687793147   -0.223313833445
C     -1.517885474144    0.840628003016   -0.770788630451
O     -2.418384655727    1.623941233993   -0.437457717517
H      1.790603426776    2.208886341478   -0.602258253818
H     -0.117885338391    2.599494840057    0.882129247949
H      2.754260264132    0.044355233762   -1.020215590182
H      1.992642551371   -2.023948731122    0.044820099692
H      0.990664303091   -1.095039187826    1.245105717968
H      0.037281220499   -1.186838150272   -1.762632304148
H     -0.401283918131   -2.436009587612   -0.512289770672
H     -0.011106288846    0.904880361412    1.543534030461
H     -0.914293104939    1.084761719866   -1.671195289367
H     -2.604383709075   -0.204106845901    1.351298566458
N     -2.060620918669   -0.892629944976    0.858111213978
H     -1.844432652150   -1.742022856764    1.354251583939

Ambimodal TS Water5-138
35
ANGSTROM
O     -1.094550635676   -0.203079771138    3.389153823419
H     -1.541948881048    0.658790220100    3.409656227094
H     -0.989917006296   -0.466631661916    4.304082973374
O     -2.518880120041    2.200128063554    3.399005974873
H     -2.904912481516    2.717363945727    4.106898591439
H     -2.497946333231    2.800838979503    2.606206382617
O     -5.494987653279    3.157653750093    1.480008796591
H     -5.224693122655    2.223731452472    1.629332566749
H     -6.087248767041    3.147493785845    0.727520131864
O     -4.243299175161    0.776587022925    1.742647120675
H     -3.682286668488    1.110659871779    2.463021068803
H     -3.737737399344    0.946026826693    0.922859244086
O     -2.808892189418    3.652322983193    1.210470115156
H     -2.673740368753    2.999185236005    0.505485325815
H     -3.778836782673    3.729564981962    1.293609443269
C      0.181151529664    1.622146409223    0.849694981520
C      1.298606851173    1.520062946055    0.073359470856
C      2.021076828569    0.302131590441   -0.191526426710
C      1.630374457354   -0.941444047190    0.270139522992
C     -0.182762567774   -1.358709473952   -0.900023704172
C     -1.214089966619   -0.638021252149   -0.292994942947
C     -1.570417564228    0.720037090467   -0.732660402282
O     -2.561232779917    1.364391017609   -0.343638324284
H      1.623276098010    2.398331445528   -0.488429258068
H     -0.338489194133    2.565345346943    0.956506194268
H      2.795399767973    0.351897189469   -0.957228590076
H      2.204156798998   -1.820949891528    0.000602243749
H      1.071662217638   -1.043657043518    1.192773491672
H      0.209527995967   -1.031556175665   -1.852067923309
H     -0.102452634614   -2.423643670586   -0.721681362821
H     -0.111924256884    0.857699132140    1.555114168203
H     -1.003748373632    1.106050843478   -1.604732200754
H     -2.548377622195   -0.591697077519    1.295497932553
N     -1.940325694161   -1.178256834878    0.744405100708
H     -1.609367096528   -2.001290270118    1.216794088307
```

```
Ambimodal TS Water5-139
35
ANGSTROM
O     -1.916258816162    2.014982918121    3.624463757562
H     -1.734758339186    2.806250693229    3.068568920492
H     -2.228986875821    2.347926702714    4.466670851372
O     -1.809068931387    4.189284414573    2.035544095003
H     -2.189737279367    4.936021658141    2.513159842348
H     -2.519695385388    3.916798085056    1.428402598274
O     -4.244128864987    3.523240241457    0.790150579484
H     -3.903929552729    3.199204992271   -0.050692908189
H     -4.393000353439    2.716195043450    1.319724113097
O     -3.784748625786    1.037628741296    1.889783391195
H     -3.150339763680    1.298220768741    2.585787512209
H     -3.326666330851    1.208621115722    1.040495659437
O     -4.133188085979    5.671635777281    2.637102767063
H     -4.357097120836    4.992776251893    1.982318580810
H     -4.762216009368    6.383261843809    2.516162474402
C      0.211393178570    1.620770271148    0.877875562217
C      1.330825932045    1.483392637389    0.107456106993
C      2.008444225376    0.246828212541   -0.178570267472
C      1.570604910570   -0.994452151442    0.255445954503
C     -0.246717287578   -1.333088135061   -0.882804110965
C     -1.242269525134   -0.550655766667   -0.286234618065
C     -1.527107669128    0.813602449367   -0.764864326006
O     -2.492683505990    1.520029055741   -0.433935789022
H      1.689761783614    2.358539773140   -0.437546336972
H     -0.270601118982    2.582164373986    0.994454078780
H      2.782611942064    0.281340717803   -0.945024242663
H      2.117066830624   -1.885840567180   -0.032125654228
H      1.026157167073   -1.096064152298    1.186947675222
H      0.150379953533   -1.042008386912   -1.844457185314
H     -0.230077327283   -2.400658507400   -0.698335612231
H     -0.119868463343    0.866485258844    1.578173427542
H     -0.917824564789    1.135804635958   -1.635140669357
H     -2.675833202097   -0.482257537244    1.240826965900
N     -1.932863719697   -1.017565227934    0.810206975764
H     -1.818176629135   -1.965355986188    1.121138975907

Ambimodal TS Water5-140
35
ANGSTROM
O     -4.662626804846    0.231745602203    0.961049026430
H     -5.496285842422    0.233966904535    0.453417754146
H     -4.027051719743    0.774724580222    0.453876839138
O     -7.021056752303   -0.376780139888   -0.257980473576
H     -7.235610121492   -1.007341067418    0.460626964089
H     -6.982108663344   -0.888764476750   -1.066905298942
O     -5.499532222714   -0.831491204664    3.347825703930
H     -4.827969304349   -1.522190108057    3.330213030900
H     -5.221583248769   -0.192676791269    2.668296964028
O     -4.375927445372   -2.532046764427    1.263992671157
H     -4.460253784804   -1.589398789904    1.033454651516
H     -5.273377959920   -2.875746386059    1.257472258298
O     -7.344466119238   -2.069544338634    1.849389306169
H     -6.739416472986   -1.606874401616    2.480016083076
```

```
H     -8.205581031699   -2.081223708920    2.268295131427
C      0.183461112458    1.647526557366    0.824916964699
C      1.280608572951    1.534508494775    0.020234788699
C      2.007454806848    0.321476485600   -0.236126059139
C      1.635612544132   -0.923203909088    0.261076342022
C     -0.165876151556   -1.408423081096   -0.755310889809
C     -1.183788306476   -0.624795230068   -0.196332308846
C     -1.584866238051    0.673569000775   -0.768131060695
O     -2.600551986653    1.303951261785   -0.454236050542
H      1.576405042547    2.402644002908   -0.571707447141
H     -0.361199857407    2.578498028605    0.906891180377
H      2.752022308663    0.358003593212   -1.030671816095
H      2.223549840722   -1.794917558417   -0.005945397977
H      1.148092759367   -1.007787788947    1.225758933143
H      0.172980105870   -1.185379931122   -1.757938364477
H     -0.110356935063   -2.458440586169   -0.491432585542
H     -0.090261431465    0.899966043451    1.555511313543
H     -1.002650700386    1.003261549668   -1.655845046419
H     -2.596344053989   -0.448559356623    1.273370086579
N     -1.799530003691   -0.997113394578    0.986667415759
H     -1.854763339148   -1.968810960629    1.244741478870

Ambimodal TS Water5-141
35
ANGSTROM
O     -2.937534031252    2.945169586908    3.241869058413
H     -2.377605861723    3.425059454322    3.851515029176
H     -2.789288595695    3.354731819633    2.347810592586
O     -2.671198755786    3.897148615977    0.788466039971
H     -3.572606643823    4.249559437726    0.643390076986
H     -2.616311454143    3.111620717594    0.221975659189
O     -5.684247379524    2.703946881076    2.629835220706
H     -5.442209212251    1.824625863861    2.300659065316
H     -4.909824090962    2.982561001522    3.140404711391
O     -3.869459162276    0.693485956960    1.872560759653
H     -3.433913685439    1.268653513235    2.519991593111
H     -3.614218732879    1.059390751966    1.003224682129
O     -5.333238410196    4.375533896453    0.541096737850
H     -5.582387140947    3.836178380639    1.325226535395
H     -5.813077484244    4.006997635151   -0.201597605746
C      0.144851973220    1.616594368566    0.895472954600
C      1.256679415113    1.528827308096    0.108671850584
C      1.979243129474    0.321493769794   -0.196170830500
C      1.604501146104   -0.939086959004    0.238718269239
C     -0.219997711883   -1.367302554923   -0.869835375861
C     -1.233543917984   -0.618864404023   -0.260278093852
C     -1.575681317791    0.728727365777   -0.746620562229
O     -2.568352220904    1.395981216378   -0.411068409841
H      1.571610537976    2.421648680976   -0.434224278033
H     -0.379190009118    2.555161589139    1.018122791381
H      2.737743027903    0.393820376588   -0.975101374166
H      2.183839048399   -1.804064383678   -0.063888782064
H      1.078168684427   -1.071213483964    1.176878051977
H      0.148451886766   -1.069304768258   -1.840756658429
H     -0.153868585673   -2.430964317080   -0.674252274810
H     -0.151987488093    0.838055094005    1.584695110952
```

```
H     -0.981243439552    1.076887690829   -1.616828017091
H     -2.646920281488   -0.567627516966    1.280261042465
N     -1.873494201653   -1.083227357281    0.866316465162
H     -1.766248279212   -2.040475442041    1.151796631352

Ambimodal TS Water5-142
35
ANGSTROM
O     -2.872010955427    2.904368193951    3.195314733140
H     -2.256933420233    3.359003744757    3.770216859810
H     -2.770334392675    3.316706179001    2.292879397196
O     -2.747913260544    3.885943189440    0.755632220701
H     -3.668497747769    4.197697942072    0.639541319389
H     -2.680767082694    3.103028254089    0.186175350266
O     -5.629050095983    2.710039737765    2.797759775678
H     -5.523108879580    1.821906335548    2.430486489682
H     -4.764026226658    2.907593695449    3.192309614987
O     -3.885189282398    0.648047480304    1.869713130244
H     -3.427740658309    1.228413206365    2.496130281748
H     -3.681904783051    1.023217234842    0.992343782800
O     -5.406847765026    4.436530674863    0.742991300793
H     -5.611457586148    3.795633556392    1.461703678786
H     -5.798990533314    5.269090692852    1.007507412620
C      0.146445667074    1.617794265160    0.886760882052
C      1.259509571363    1.528936123770    0.101828121248
C      1.984794391496    0.322359748321   -0.197030646216
C      1.608080087716   -0.938208041378    0.239615658230
C     -0.206606595116   -1.373848841536   -0.864723463782
C     -1.225609268666   -0.627803854407   -0.259825693095
C     -1.577129759676    0.717196964390   -0.750060395358
O     -2.570613911217    1.377614635562   -0.410175082262
H      1.573258174340    2.420251793192   -0.444343614125
H     -0.381826881629    2.554930078893    1.001585305269
H      2.744080219431    0.392690363993   -0.975280554283
H      2.190810740133   -1.802691282028   -0.058237667277
H      1.084393910450   -1.067326343840    1.179786281782
H      0.158965385560   -1.078217062162   -1.837610265731
H     -0.139485923753   -2.437325366909   -0.667625982765
H     -0.150509216835    0.841994312147    1.578746860615
H     -0.984800062007    1.067354308414   -1.621508095165
H     -2.644856571519   -0.574394289282    1.273417166913
N     -1.865061830280   -1.089084575331    0.867613438286
H     -1.757263774492   -2.045389481939    1.156343826073

Ambimodal TS Water5-143
35
ANGSTROM
O     -6.358494597087   -3.367740528318    2.389754107455
H     -6.978197018083   -3.995256522120    2.016040063523
H     -5.909646425013   -2.935252821233    1.618620740560
O     -4.932161259719   -2.359657519073    0.394595058635
H     -4.078695552580   -2.779216654996    0.595668482862
H     -4.814920942166   -1.408167245090    0.570942566389
O     -4.105425088557   -4.245766316535    3.517825009933
H     -3.943951167107   -3.680350731224    4.274462077953
H     -4.990518369789   -3.980834622467    3.170440598567
```

```
O     -4.532485927692    0.305885303416    0.982598810004
H     -5.309939494768    0.862979076279    1.028436957668
H     -3.896476820022    0.762452333610    0.382915913586
O     -2.678605125889   -3.782179777017    1.310279068311
H     -3.132701038968   -3.964759954064    2.167360409385
H     -2.587481141977   -4.632708830989    0.878693670389
C      0.142509668798    1.657653786847    0.825096456870
C      1.264770476737    1.554384948192    0.056638989690
C      2.007794080073    0.345529133126   -0.185745883473
C      1.636624670207   -0.902214541571    0.293268014712
C     -0.150677734735   -1.403894682553   -0.801762493897
C     -1.184103642586   -0.635021758731   -0.253773120181
C     -1.562492634542    0.669357791560   -0.828560318416
O     -2.586556206744    1.309120664951   -0.555717769666
H      1.576607617435    2.428070609341   -0.518901463749
H     -0.406132886441    2.587090086199    0.898390118500
H      2.776738159719    0.395300550515   -0.956202159841
H      2.235061128538   -1.769492901715    0.036860345346
H      1.104550305261   -1.001331886935    1.232127335275
H      0.222627516635   -1.162232227290   -1.787042097920
H     -0.086058813103   -2.453603347846   -0.541680882360
H     -0.155970980435    0.900824890879    1.536211065987
H     -0.948055090911    0.999910948776   -1.693317686640
H     -2.622659254215   -0.516444193453    1.215588756166
N     -1.818168489816   -1.037640497114    0.903294663893
H     -1.817055843761   -2.014292779007    1.161094446341

Ambimodal TS Water5-144
35
ANGSTROM
O     -4.670215083947    0.213207907757    0.923426615300
H     -4.032759891725    0.757853006591    0.420417611596
H     -5.499776885073    0.211225883048    0.409126653019
O     -7.016349874305   -0.406824079433   -0.313399536472
H     -7.234145408388   -1.037007818474    0.404563199603
H     -6.968886576208   -0.920029203655   -1.121094400353
O     -5.521997133753   -0.849280807650    3.305272450153
H     -4.847571156960   -1.537320916964    3.294228790297
H     -5.241159029985   -0.210517382402    2.626885238351
O     -4.374930726038   -2.548886094466    1.233506306412
H     -4.461163381701   -1.606983027666    1.000639756327
H     -5.270919563632   -2.896191164592    1.220373575273
O     -7.349905337716   -2.097259437474    1.794256910009
H     -6.751781702929   -1.631096904852    2.428905525634
H     -8.214299280524   -2.111664237891    2.206267470570
C      0.171090263194    1.648195867055    0.823772488733
C      1.275127008754    1.538198117847    0.028145497543
C      2.008866820767    0.327656114407   -0.220222398215
C      1.638019935612   -0.917647722606    0.276161698910
C     -0.153266252880   -1.411858605304   -0.753833774881
C     -1.178777880270   -0.631363811229   -0.204444552066
C     -1.580433872812    0.664388146958   -0.781736734852
O     -2.601130680880    1.291225658298   -0.477135529787
H      1.572202161953    2.406491347651   -0.562924415348
H     -0.377937455623    2.577110979253    0.899722234932
H      2.759657290932    0.365802477875   -1.008813808633
```

```
H      2.231575293287   -1.787451406146    0.015414681315
H      1.143089024875   -1.002523286152    1.237037320148
H      0.192758235321   -1.189187821774   -1.754092927924
H     -0.095669139190   -2.461187044744   -0.487669787950
H     -0.105511469120    0.900804857272    1.553455303851
H     -0.992412528844    0.994882797094   -1.665316081717
H     -2.603819513249   -0.458269229890    1.253531653355
N     -1.802531352693   -1.004109821050    0.974215182123
H     -1.855946405359   -1.975573969898    1.233547286217

Ambimodal TS Water5-145
35
ANGSTROM
O     -1.974904649837    4.529765169038    1.562179097316
H     -2.127797849348    5.398270627888    1.934758504993
H     -2.695041460042    4.381115919134    0.902945316010
O     -3.999187521866    3.774143693933   -0.020728781380
H     -4.634482051507    3.394360486309    0.607498013582
H     -3.537792582133    3.014958194735   -0.406289405117
O     -5.276629554856    2.398188311444    2.101856398569
H     -4.327683960755    2.250549128859    2.353256792217
H     -5.730618877752    2.684610422516    2.893996976284
O     -2.679342211659    2.023914946306    2.388150732929
H     -2.300133856923    2.918114719512    2.308599442869
H     -2.590056289016    1.646075460749    1.499946848695
O     -4.647399800427   -0.030485316955    0.773679997689
H     -5.119558242825    0.613492433745    1.318689867629
H     -4.204704756222    0.502058666075    0.098889852194
C      0.185070614943    1.729745992535    0.720523541546
C      1.310964845923    1.544889954415   -0.028822136859
C      2.015053206024    0.299945915320   -0.193946086215
C      1.600117159049   -0.904879789993    0.347461202327
C     -0.195237474809   -1.374373243944   -0.799421668347
C     -1.228033836074   -0.606664016922   -0.254821989656
C     -1.568952163790    0.721632808526   -0.785361325244
O     -2.559663085348    1.387641910557   -0.427961420304
H      1.658543334045    2.373286515108   -0.648831394053
H     -0.313679448555    2.690249964113    0.747716430638
H      2.799233111602    0.282145808570   -0.950899002545
H      2.167452344248   -1.806773290991    0.146771445728
H      1.035089118860   -0.938010977064    1.271331425263
H      0.211533351082   -1.108998377788   -1.764396643284
H     -0.132678812897   -2.429166881450   -0.560853714108
H     -0.136245906122    1.028041704874    1.478506246363
H     -1.015040995991    1.042725603653   -1.689741699715
H     -2.812880177909   -0.606594458720    1.084574783619
N     -1.904091433990   -1.016001858167    0.881034695768
H     -1.812320642247   -1.973667058397    1.177367391686

Ambimodal TS Water5-146
35
ANGSTROM
O     -2.518831389476    2.199996917728    3.399107297142
H     -2.904852415767    2.717208166754    4.107024093169
H     -2.497896776827    2.800740134425    2.606332201878
O     -2.808843565825    3.652284998534    1.210633269438
```

```
H     -3.778786631863    3.729534744091    1.293783216510
H     -2.673704672029    2.999174629670    0.505620638389
O     -1.094529295639   -0.203226709955    3.389145710803
H     -1.541917661944    0.658647487577    3.409686873769
H     -0.989891186120   -0.466817061318    4.304063275152
O     -4.243279531503    0.776543711312    1.742703259268
H     -3.682257652523    1.110580541046    2.463086612481
H     -3.737722099540    0.946011341834    0.922918455779
O     -5.494942648807    3.157635607029    1.480172283068
H     -5.224657713264    2.223704071621    1.629455640106
H     -6.087209671889    3.147513342433    0.727687761193
C      0.181174063521    1.622089073441    0.849751861568
C      1.298622268357    1.520024596453    0.073403611877
C      2.021076258260    0.302095808290   -0.191537692970
C      1.630363168522   -0.941494255405    0.270080323039
C     -0.182787598487   -1.358690978627   -0.900086112673
C     -1.214102094973   -0.638015813107   -0.293019931703
C     -1.570417508743    0.720064619137   -0.732627030746
O     -2.561222359243    1.364413960397   -0.343570957520
H      1.623297280137    2.398312380366   -0.488351635886
H     -0.338455032116    2.565289591017    0.956605683852
H      2.795393906067    0.351883889013   -0.957243746138
H      2.204133365861   -1.820995637583    0.000502634372
H      1.071656817734   -1.043738631789    1.192714380899
H      0.209499422927   -1.031503172680   -1.852119932724
H     -0.102488494647   -2.423633399103   -0.721788009100
H     -0.111905081081    0.857616262607    1.555141981730
H     -1.003750585111    1.106107603424   -1.604687362599
H     -2.548377087140   -0.591741437565    1.295485031279
N     -1.940336061958   -1.178285573028    0.744363544770
H     -1.609383264466   -2.001342133291    1.216716303144

Ambimodal TS Water5-147
35
ANGSTROM
O     -3.558880048300    1.307500957033    2.054425787608
H     -3.925364973883    2.146561772725    2.336686410952
H     -3.245505116385    1.440343272766    1.124384403451
O     -1.534764917654    0.220804771732    3.685342155437
H     -2.149498653267    0.682044426887    3.090099941683
H     -1.635172480269   -0.725340770673    3.480548613177
O     -3.332157274459    0.221975329739    5.696965526853
H     -2.962244406551    0.003640962821    6.552183187511
H     -2.572558430850    0.321270742763    5.079423196117
O     -4.563240619484   -0.925332426239    3.514662364250
H     -4.431862567861   -0.172801420629    2.920166681576
H     -4.364308228396   -0.574723272638    4.400436228269
O     -2.367849109143   -2.403313368114    3.209299003927
H     -2.352718404241   -3.069475997524    3.897225403922
H     -3.257495104714   -1.972979673859    3.267865977318
C      0.172763282460    1.615195727200    0.906297218261
C      1.312729881595    1.472348677537    0.170527913114
C      1.997768500338    0.230271762518   -0.074822605286
C      1.536808039617   -1.002984827086    0.354000523448
C     -0.239329047939   -1.336552137531   -0.870966331069
C     -1.266564972990   -0.571970507588   -0.311467435021
```

```
C     -1.559582697953    0.804289803767   -0.759350261870
O     -2.525788614808    1.487431413197   -0.385005459008
H      1.686773029272    2.339177933760   -0.377935067097
H     -0.320980294542    2.574241235877    0.992245241958
H      2.800019918660    0.255064581029   -0.812060665642
H      2.087102434111   -1.900382556558    0.094581952343
H      0.949529871996   -1.096119882432    1.259514315478
H      0.203708475005   -1.026747999753   -1.806141584160
H     -0.220631513868   -2.404543360374   -0.694111655712
H     -0.170307368458    0.866944265166    1.606435318858
H     -0.960166697279    1.163443331547   -1.621207348845
H     -2.763493155451   -0.484753400249    1.108902748147
N     -2.039127747100   -1.056747255607    0.712925187176
H     -1.872650244165   -1.939513798633    1.164657596709

Ambimodal TS Water5-148
35
ANGSTROM
O     -2.525294785490    2.506353096460    3.280134867670
H     -1.632117684708    2.542812818182    3.622055214377
H     -2.542717560365    3.095821679149    2.485421844254
O     -2.657463907675    3.848764830664    0.984178975296
H     -3.582080697707    4.172494233569    1.009077550164
H     -2.655075286464    3.144266632106    0.320420564130
O     -6.315333095816    1.949751986547    1.668911926240
H     -6.742866164283    1.566474881454    0.901904166738
H     -5.498950930210    1.422306881341    1.821240194014
O     -3.929474704040    0.708172024730    1.822704097441
H     -3.491481500424    1.246410129725    2.511095462572
H     -3.598592108787    1.076251373773    0.976717513813
O     -5.291667349480    4.427617138479    1.279743223033
H     -5.708018019394    3.547640976277    1.406666161057
H     -5.436956228168    4.902940809692    2.099079483647
C      0.162264866598    1.624208012452    0.868644837638
C      1.272101842872    1.515280573880    0.081574314035
C      1.981522915631    0.297274997983   -0.209097080769
C      1.590822913079   -0.954750475005    0.238814545687
C     -0.234365389230   -1.370225956874   -0.859720882257
C     -1.242774908097   -0.611221011445   -0.253873040317
C     -1.581936630175    0.734528711210   -0.749191229010
O     -2.567090085608    1.409732338737   -0.410553279411
H      1.595555588182    2.398322490391   -0.472079786676
H     -0.353962036213    2.568979773859    0.976283400160
H      2.740331496310    0.351935842565   -0.989079495802
H      2.161074841759   -1.829063724551   -0.054317131505
H      1.066755402818   -1.070641504546    1.180510080188
H      0.132526118628   -1.081265061656   -1.834031085013
H     -0.177472066661   -2.433552294983   -0.659024627291
H     -0.141890546538    0.855887684201    1.566355825072
H     -0.991878128178    1.071936365014   -1.626448602600
H     -2.660882138026   -0.551801705353    1.281758074066
N     -1.881384234336   -1.065345779307    0.878692403593
H     -1.775554862904   -2.021044517538    1.170184068698

Ambimodal TS Water5-149
35
```

```
ANGSTROM
O     -1.417193223886    4.201337479104    2.582084995197
H     -1.259651967780    4.216190937088    3.527043053907
H     -1.787599765870    5.084020458663    2.352006621154
O     -2.684373443471    6.476367580607    1.920926536495
H     -3.552110294393    6.058397816512    1.718483997652
H     -2.392614667177    6.880107021149    1.102139156885
O     -3.523569182281    2.786836861967    1.647544246528
H     -3.240074506665    2.437999442300    0.780943610183
H     -2.721775966389    3.212090562255    2.021172186462
O     -3.765946440858    0.198825014954    2.414687960255
H     -3.680712285008    1.162281801133    2.254363353491
H     -4.681132475807   -0.015861654205    2.228335632154
O     -4.905962835606    5.072127199988    1.355468194057
H     -4.474440515315    4.186868142539    1.386922932522
H     -5.610511757089    5.049389253466    2.004244734145
C      0.178081466901    1.487214769163    1.057389261661
C      1.331319238098    1.433882783434    0.329418657351
C      2.015136012868    0.235292234180   -0.075663169864
C      1.545767289313   -1.046401514978    0.182880011433
C     -0.203005407684   -1.257277552095   -1.037110421137
C     -1.225626583790   -0.505560385442   -0.438826773541
C     -1.481345273480    0.894398992444   -0.828139013602
O     -2.463674837669    1.566511621931   -0.503183383097
H      1.721438368229    2.366900419426   -0.081169767221
H     -0.307847128480    2.429709030860    1.270327704929
H      2.818356420823    0.352934566456   -0.802221130361
H      2.105526505623   -1.900741983878   -0.182143025268
H      0.979312712534   -1.253207745281    1.084001791962
H      0.235699029892   -0.904279105060   -1.959993077303
H     -0.221018333282   -2.336952236326   -0.939931015106
H     -0.197692768529    0.648704287108    1.626927564348
H     -0.795327187848    1.292035239686   -1.610170088995
H     -2.644920811536   -0.472099256137    1.102639787479
N     -2.028129409449   -1.048913332229    0.533960660461
H     -1.847334031431   -1.973768445693    0.883748365025

Ambimodal TS Water5-150
35
ANGSTROM
O     -3.782548446155    2.974561771894    2.334396291290
H     -3.734707470714    3.566365459030    3.084819845713
H     -3.289650908806    3.417599745643    1.591270346686
O     -2.682450064840    4.024295056880    0.183224715466
H     -3.452933218314    4.397596610665   -0.279708414059
H     -2.529987424552    3.169879645183   -0.257883577483
O     -5.232012131880    1.942640145938    0.154014726999
H     -4.405588743280    1.619439769332   -0.237803926349
H     -4.964751579194    2.330749264418    1.001341937290
O     -3.873234565682    0.188247814792    2.134763355944
H     -3.665903362617    1.118045813234    2.324477346400
H     -4.702244724627    0.210755981878    1.646267126165
O     -5.150011437691    4.407139642069   -0.988510656531
H     -5.323427335373    3.482012386677   -0.710575207749
H     -5.949030680976    4.901232359099   -0.804157097141
C     -0.056970718980    1.517692871273    1.038938190736
```

```
C      1.150845445872    1.495156443941    0.404610511683
C      1.920157302507    0.317718757255    0.095827271332
C      1.502031775154   -0.974954557192    0.364613886259
C     -0.153443184060   -1.308308886310   -1.015533332975
C     -1.245798628065   -0.605696485434   -0.493114997016
C     -1.533260238320    0.779740751369   -0.900445838205
O     -2.571746115671    1.403599956084   -0.636685757564
H      1.526711863966    2.431202278384   -0.012216423762
H     -0.601776199718    2.443242104271    1.168439548228
H      2.773629078985    0.456184291779   -0.567088815596
H      2.124247646699   -1.812027733397    0.068908387429
H      0.858176189646   -1.186821285574    1.210367921813
H      0.341509978123   -0.937109061128   -1.901582768941
H     -0.109908535089   -2.384470065207   -0.896967072863
H     -0.440226399210    0.685316645749    1.612535122411
H     -0.826045590153    1.214899921491   -1.638997191790
H     -2.739118014075   -0.595727414765    0.969833175998
N     -2.083771806299   -1.167181341957    0.435772015576
H     -1.899317434942   -2.085360546811    0.800212942739

Ambimodal TS Water5-151
35
ANGSTROM
O     -1.393272992779    1.069469229087    3.851558943272
H     -0.966527308560    1.891351654761    4.094798526215
H     -2.112650330377    1.324005180220    3.207518643142
O     -3.332593627643    1.693928110382    2.200978611350
H     -3.937519187241    0.937280543718    2.291184833510
H     -3.096884807209    1.713337218060    1.253490376455
O     -2.795648502195   -1.313547262875    4.503169006172
H     -2.010196227992   -1.710456026793    4.096707031664
H     -2.540445816145   -0.394457062262    4.661786682955
O     -0.230975257051   -1.358334514852    3.345834597013
H     -0.550246261344   -0.438428481683    3.436358963756
H      0.468466404490   -1.465496194672    3.992163467620
O     -4.467348927223   -0.787603290599    2.469934797312
H     -3.926343533891   -1.069950622961    3.242591923941
H     -5.367715718704   -1.056207276072    2.652326266824
C      0.175011060850    1.702595424502    0.780119883472
C      1.294863337074    1.590020570109    0.010429673325
C      2.033806478453    0.375521349172   -0.210684895741
C      1.651358041723   -0.863428052428    0.285204872585
C     -0.140597477966   -1.351549350454   -0.800414832654
C     -1.188943414635   -0.609126397345   -0.244233745730
C     -1.601413233147    0.703621403759   -0.784727281623
O     -2.582904898412    1.350746270178   -0.408007604144
H      1.607611981967    2.454451026290   -0.578312454370
H     -0.374828598938    2.631895848151    0.845992839776
H      2.802277809829    0.408476351963   -0.982624066997
H      2.243838783081   -1.737164741548    0.035608738707
H      1.122526716569   -0.952819091496    1.226779506555
H      0.226890136594   -1.087546169080   -1.781801360869
H     -0.056161076419   -2.403396974748   -0.556842456721
H     -0.115260639922    0.949728081144    1.497570567569
H     -1.056941706462    1.030998699495   -1.697977852604
H     -2.665629063527   -0.555686263696    1.213619830704
```

```
N     -1.856587173363   -1.047305713865    0.870842480334
H     -1.592720164678   -1.889270588054    1.349371900875

Ambimodal TS Water5-152
35
ANGSTROM
O     -6.646838686635    0.444159626151   -0.188737111670
H     -7.407333099532    1.000559135470   -0.355696248998
H     -6.017319818529    0.609645263727   -0.935048278952
O     -4.792839422573    0.852960785838   -2.056988194670
H     -4.403430619293   -0.036393291223   -2.167210448016
H     -4.104472552581    1.354762555473   -1.598018551435
O     -5.062661351861   -1.897178211554    0.373943521200
H     -4.711219454130   -1.162407289851    0.917451098219
H     -5.936162470066   -1.579938576506    0.116831151113
O     -4.382344223468    0.510547078225    1.452615741251
H     -5.277308171103    0.700557644866    1.131749861812
H     -3.785992895089    0.963874810701    0.823706336088
O     -3.684395001390   -1.645144719645   -1.896611618028
H     -4.136909446460   -1.812232900277   -1.033079117477
H     -3.970474037152   -2.345053381990   -2.484902000697
C      0.165938421379    1.644367178355    0.831646692860
C      1.270191813550    1.541057510842    0.036802754514
C      2.003320286579    0.330561162756   -0.227566590756
C      1.642724207188   -0.916278745278    0.257474598931
C     -0.184819429508   -1.398795441592   -0.807528199791
C     -1.197078423225   -0.636509265339   -0.218792448409
C     -1.575412637213    0.679208196680   -0.756588730099
O     -2.580143519555    1.333847346442   -0.435956896357
H      1.569836217554    2.415209428083   -0.544224696706
H     -0.375820103570    2.576284886983    0.923482252627
H      2.753485952179    0.380677110322   -1.016245594620
H      2.228284255598   -1.786135675954   -0.018398585700
H      1.129252547049   -1.017360978232    1.206375449196
H      0.166039895895   -1.137219539305   -1.795227820774
H     -0.112005564702   -2.454050108969   -0.573002320999
H     -0.115112700006    0.889246781371    1.551911444030
H     -1.000308342922    1.005937292799   -1.648066082586
H     -2.607401529098   -0.542922704956    1.292249474161
N     -1.809694648504   -1.054335338084    0.945955860882
H     -1.771677442230   -2.023132216161    1.212009296261

Ambimodal TS Water5-153
35
ANGSTROM
O     -5.923401701389   -0.565124360790    0.472018816052
H     -6.858428232928   -0.359766702471    0.471049195204
H     -5.554078551381   -0.201907309887   -0.383822982249
O     -4.782535368347    0.301489762867   -1.711407544944
H     -4.316951784958   -0.506587813978   -2.001725434727
H     -4.080006069180    0.890850219982   -1.391866837164
O     -4.444566242591   -2.872312404447    0.366240230749
H     -4.946706007311   -2.050479204717    0.527616081064
H     -5.010276925838   -3.592376237688    0.647607029166
O     -3.729572335026    0.645370144076    1.735124418480
H     -4.590419045240    0.291447970485    1.468221751330
```

```
H     -3.436896062303    1.217632171449    1.007769296335
O     -3.526304229054   -2.101894408311   -2.105422003776
H     -3.720065191662   -2.419577645227   -1.202562784490
H     -3.912224657456   -2.738560526384   -2.708907197328
C      0.074709866539    1.579592373306    0.976813044841
C      1.263271551129    1.560830915567    0.308872150038
C      2.030984647268    0.384907965604   -0.018360180753
C      1.633331281469   -0.907576334302    0.268331726622
C     -0.078151906323   -1.263848801066   -1.074563727023
C     -1.146376680471   -0.566632896556   -0.503421232065
C     -1.453309972502    0.818189217907   -0.913122849075
O     -2.498878040501    1.431929761229   -0.646604695844
H      1.625423540125    2.496648824053   -0.121573145947
H     -0.474610757856    2.500114644339    1.122040894454
H      2.864374741044    0.528496623691   -0.705663948882
H      2.249764122778   -1.742339216585   -0.044995475597
H      1.005173172374   -1.122140804200    1.124429313847
H      0.386011897783   -0.885483117884   -1.973495935204
H     -0.023390170656   -2.339471661029   -0.960252248021
H     -0.290938576453    0.745551054993    1.558500140569
H     -0.767595353305    1.250658699622   -1.671952384343
H     -2.614825874310   -0.567454363709    0.981474803402
N     -1.918856710911   -1.118581766434    0.480621288543
H     -1.830848372187   -2.085430869212    0.734968282897

Ambimodal TS Water5-154
35
ANGSTROM
O     -5.511488202725    3.433597777810    1.550754713898
H     -6.337649725456    3.811472682589    1.250211623460
H     -5.455262326192    2.533840696630    1.140622148369
O     -5.156804902308    1.011401120769    0.498315537152
H     -4.832715332742    0.511794666163    1.266842082131
H     -4.361953700922    1.150643343377   -0.041980726159
O     -3.405495045227    2.535270043201    3.274097643761
H     -2.915428576652    2.965160037894    2.541189201302
H     -4.299625264734    2.884421940270    3.170308457121
O     -2.770982226553    3.739822012305    0.943285739888
H     -3.716973546617    3.944125103817    0.994123572796
H     -2.702906947879    3.011742859476    0.305511984546
O     -3.721821301675    0.005634898210    2.623951316342
H     -3.523274086778    0.944165247515    2.892116889402
H     -4.026269598657   -0.435800376496    3.416921647882
C     -0.020500233535    1.490798508179    1.058178703209
C      1.158670232118    1.473742912759    0.370579038066
C      1.910038528018    0.298518386552    0.016798943444
C      1.497471391625   -0.996762165098    0.289984480240
C     -0.210497653333   -1.304757935844   -1.005153213279
C     -1.277765555629   -0.593666540084   -0.442763216613
C     -1.577284893308    0.790674788971   -0.839708299401
O     -2.607626427210    1.413793650910   -0.544495443963
H      1.517383780570    2.413367950436   -0.052761477795
H     -0.558432714309    2.414344935409    1.223618809407
H      2.734180985667    0.439822209261   -0.681666906772
H      2.104581223223   -1.832214773007   -0.040296506293
H      0.898252332105   -1.214038451693    1.166837451248
```

```
H      0.247199442883   -0.938796705611   -1.913271490181
H     -0.175978035759   -2.382094528586   -0.892130694890
H     -0.374305358328    0.652507576899    1.642333303043
H     -0.892911036232    1.226539676929   -1.598165145212
H     -2.708473855994   -0.596695091281    1.077937813636
N     -2.083811520671   -1.161594245504    0.516593237551
H     -1.884385098106   -2.080749474054    0.870218656828

Ambimodal TS Water5-155
35
ANGSTROM
O     -4.713542923633   -1.280862830435    2.268710736471
H     -5.537393243673   -1.189256751828    2.747763071562
H     -4.837420738037   -0.787665091647    1.412936628315
O     -4.887745867363   -0.129131407702   -0.088002383905
H     -4.589742823510   -0.872638084987   -0.642960366354
H     -4.179054947002    0.535364532237   -0.158368217631
O     -3.554656377530   -3.487149273396    1.280520445211
H     -3.978527825484   -4.265527208326    1.644029321229
H     -3.962545558205   -2.711868522500    1.729706550740
O     -2.395606082476    3.997574674745    0.372236329654
H     -2.497922979879    3.081861141782    0.066460365608
H     -3.268903080221    4.388547629742    0.319718827854
O     -3.841073779046   -2.412993587722   -1.237143499324
H     -3.661072929377   -2.859610148014   -0.387510400772
H     -4.365806691230   -3.018553479463   -1.762344889156
C      0.179380816912    1.621317235054    0.889723854096
C      1.289186589309    1.538427431282    0.099194911859
C      2.022696927577    0.336047624273   -0.200677335589
C      1.658818390547   -0.927003722244    0.237575170203
C     -0.157359412880   -1.374463897677   -0.865578146211
C     -1.170910012585   -0.627172636585   -0.261754807604
C     -1.546253762391    0.712347935992   -0.733769039270
O     -2.571420293913    1.318845513030   -0.372766528082
H      1.593864006070    2.430921562752   -0.450064645935
H     -0.357170029779    2.553706369037    1.005804580907
H      2.781183204880    0.412377517684   -0.979755761400
H      2.247391600760   -1.787139686592   -0.061722945090
H      1.132551601762   -1.061277765871    1.175191546977
H      0.205310668526   -1.081553709299   -1.840472144356
H     -0.091196182399   -2.436267831991   -0.661963643793
H     -0.106556284370    0.841785058661    1.582750271789
H     -0.970105684997    1.109643891073   -1.593554625860
H     -2.580537063826   -0.537432549791    1.231092870439
N     -1.798632020079   -1.071516103201    0.884962546481
H     -1.820416138466   -2.052197279757    1.109668128292

Ambimodal TS Water5-156
35
ANGSTROM
O     -4.668170216374   -1.251481992901    3.222191882790
H     -4.785632738151   -0.715013437116    4.006686065816
H     -4.633391367600   -0.623545504104    2.461687682168
O     -4.608283223587    0.235975591734    1.021332326484
H     -4.910152066754   -0.464058761541    0.418949665772
H     -3.974384115649    0.779144456189    0.510758192538
```

```
O     -6.498178625006   -2.959113368457    2.123398028621
H     -6.123024207474   -3.825598846491    2.298072488579
H     -5.966775686504   -2.328560733877    2.648606503169
O     -3.401212066633   -3.279207749408    1.604555859875
H     -3.703957509741   -2.716423564335    2.328749497921
H     -3.929105090774   -3.016955629172    0.839012344152
O     -5.401697504403   -2.149964924622   -0.188265541518
H     -5.944200093808   -2.434328583783    0.584179786519
H     -5.954173304403   -2.250048118131   -0.963760837001
C      0.186903063625    1.635103257085    0.853293193489
C      1.295268599677    1.545714509248    0.062727271491
C      2.029953342309    0.340952655425   -0.217647506515
C      1.659154466966   -0.916862712727    0.239560638744
C     -0.137690019600   -1.387504912237   -0.828807483558
C     -1.162855408023   -0.625660435165   -0.256230942386
C     -1.559894621282    0.692300278424   -0.788383453437
O     -2.581568650552    1.306864534172   -0.460453883233
H      1.599065113209    2.430921770448   -0.499370553301
H     -0.359188992055    2.563233185613    0.955695030198
H      2.785277316199    0.403717827254   -1.000450107460
H      2.251517065103   -1.779839738969   -0.044487595159
H      1.149048051106   -1.033126436441    1.188778298322
H      0.219458345993   -1.128564468398   -1.816000448905
H     -0.076997115842   -2.443480497247   -0.592919709171
H     -0.099527420588    0.863650488813    1.553567985521
H     -0.971526517601    1.055445757344   -1.658411303276
H     -2.595821668408   -0.493578429849    1.197399781380
N     -1.798386981426   -1.036883697391    0.896802306793
H     -1.841717570747   -2.015364520827    1.149631697889

Ambimodal TS Water5-157
35
ANGSTROM
O     -6.427172951038    0.650700657215    1.196521978715
H     -7.266198047514    1.058584192460    0.983844108058
H     -5.973343644197    0.471733895040    0.330728774904
O     -4.997731105907    0.099261182578   -0.952851385717
H     -4.694572422422   -0.805774490951   -0.738652192222
H     -4.214770484276    0.665786438749   -0.879592645761
O     -5.107074887425   -1.453344777786    2.407788026814
H     -4.411563680944   -0.852606320280    2.703269193705
H     -5.814143391537   -0.859192985434    2.105183473910
O     -3.727608836631    1.029107936061    2.010355429749
H     -4.679821574870    1.152287661016    1.892701794120
H     -3.322208360441    1.427323860524    1.221834666031
O     -4.333539507272   -2.378528648596    0.015807183008
H     -4.591664138196   -2.127967337990    0.935374890628
H     -4.953258286762   -3.055944919295   -0.257619510945
C      0.169431175067    1.646326129677    0.856788295951
C      1.292545501864    1.528044087212    0.090784617210
C      1.998133337981    0.303224568059   -0.186468589759
C      1.594824600188   -0.944339515368    0.257228285513
C     -0.224814695422   -1.357045334707   -0.880745379464
C     -1.221792362962   -0.575782806466   -0.291152023389
C     -1.544581102492    0.771363202501   -0.794718382531
O     -2.554138182294    1.422016425990   -0.477062572580
```

```
H      1.634727415808    2.407105815494   -0.458824911394
H     -0.342446556905    2.593986812647    0.959670441387
H      2.771351530143    0.353642481881   -0.952724080818
H      2.162568428703   -1.823276980473   -0.026355641445
H      1.045526724289   -1.056592117998    1.184406758509
H      0.163798818781   -1.079136346950   -1.849975560560
H     -0.187704053205   -2.418938656278   -0.670128626406
H     -0.155924135093    0.883895068335    1.550326063510
H     -0.937598621381    1.122777604708   -1.654362917341
H     -2.623084825699   -0.419912757860    1.222088870787
N     -1.883319394427   -0.995275552958    0.839420355543
H     -1.911842338383   -1.969754069191    1.081553735749

Ambimodal TS Water5-158
35
ANGSTROM
O     -6.034988024288    2.727990883847    1.363825546136
H     -6.686981738980    3.199963151232    0.846145490105
H     -5.787878341439    1.915976634320    0.843029351643
O     -5.142291882330    0.529494899768    0.218594512910
H     -4.738528710892    0.214405449745    1.053645361711
H     -4.377191864937    0.812864903028   -0.307893541067
O     -6.094564537535    1.073617794816    3.602859783479
H     -6.113524141873    1.801084077971    2.956670231342
H     -6.864265495397    0.532286961472    3.421039800827
O     -3.221918405611    2.821587368056    1.699452936314
H     -4.159976331729    3.062063291699    1.687337614978
H     -3.016761250000    2.520089274954    0.801652733323
O     -3.693695269764    0.279284116636    2.515851215874
H     -3.365294594936    1.194550360699    2.376408319588
H     -4.439339859084    0.387388002334    3.130436010264
C     -0.070688003359    1.468250118028    1.082510447444
C      1.106286731509    1.478602632218    0.392322352060
C      1.879007797844    0.316841572262    0.036952213381
C      1.494695935670   -0.984507409343    0.319267695819
C     -0.218402399781   -1.337251563340   -0.962928883385
C     -1.292212248068   -0.621328839111   -0.418641606341
C     -1.594350973253    0.749738384456   -0.859600664373
O     -2.632105056698    1.380023580796   -0.610450207137
H      1.446741354582    2.425090448704   -0.031248926079
H     -0.632459189522    2.375234861488    1.259386951980
H      2.696203443509    0.472387675326   -0.666659610292
H      2.115393321790   -1.809568699106   -0.011693312716
H      0.904471012580   -1.209347089336    1.200175056838
H      0.237672899606   -0.990824314493   -1.879582997975
H     -0.174872907761   -2.410994128943   -0.822135460470
H     -0.404715028550    0.618847126748    1.661920477885
H     -0.891402298564    1.167249587593   -1.612041733918
H     -2.703254334633   -0.604397104104    1.120938246724
N     -2.104714595678   -1.180290490832    0.542625951076
H     -1.881504302879   -2.083321212986    0.924172710755

Ambimodal TS Water5-159
35
ANGSTROM
O     -6.147897789692    2.434642159631    0.300585009266
```

```
H     -6.250486957760    2.870104883541   -0.546512225881
H     -5.902005775289    1.507676395524    0.106752292508
O     -4.923295107580    0.005916590956   -0.047445573261
H     -4.794790989162   -0.099484364997    0.914783548205
H     -4.124269725535    0.491098949988   -0.334596238984
O     -3.998655528346    0.208283585073    2.492606366323
H     -3.856556099189    1.173860964693    2.307068802766
H     -4.388147249705    0.154723339589    3.364937402436
O     -3.753303530831    2.699409279203    1.638924194022
H     -4.659129893483    2.810837348218    1.293830430163
H     -3.222587047716    2.456611575975    0.865551501876
O     -3.942114107617   -2.523406557150   -0.691595919398
H     -4.359414407979   -1.659782959906   -0.518213699631
H     -4.605912845873   -3.051290783197   -1.136731059514
C      0.091868160945    1.593638408063    0.951568052916
C      1.227877654151    1.546046856515    0.198684416093
C      1.973569554851    0.356104267572   -0.130434060212
C      1.610991036922   -0.922221557012    0.249529561298
C     -0.190820846637   -1.350830904976   -0.939737329880
C     -1.208733773609   -0.614339917136   -0.331363268230
C     -1.547753446911    0.736836265119   -0.801710149662
O     -2.586280412448    1.356535915619   -0.510039099946
H      1.554092855594    2.461383685733   -0.299155337720
H     -0.447125814254    2.520460637492    1.098884976477
H      2.755027216594    0.470057564212   -0.881594236916
H      2.209429198665   -1.767969218475   -0.068906501339
H      1.045303829005   -1.098553262711    1.156421804294
H      0.203580679098   -1.029787536979   -1.893327636457
H     -0.126051680342   -2.416821595206   -0.761369853851
H     -0.224200926712    0.783916048875    1.593794286780
H     -0.914494466960    1.135426638139   -1.621101496835
H     -2.605702999659   -0.556895595260    1.201838092475
N     -1.850120836099   -1.088463247955    0.792210246507
H     -1.913821660630   -2.081317249758    0.939210153978

Ambimodal TS Water5-160
35
ANGSTROM
O     -2.959962995111    4.087129365884   -0.497771369790
H     -2.835060577419    3.156770916831   -0.724310021297
H     -3.791687393976    4.120476550060   -0.005872522850
O     -5.398679101030    1.061665997729   -0.029459983344
H     -5.263217892370    0.498781221175    0.750309084699
H     -4.521877485083    1.119760239879   -0.435509084933
O     -3.182586106549    2.246909758397    2.159250350321
H     -2.815741813548    2.085242298228    1.276797066255
H     -3.875984101504    2.907614375666    2.017171039257
O     -5.357713098337    3.536157556625    0.987882664645
H     -6.172290763041    4.022373770363    0.862320062080
H     -5.489510389616    2.649585963049    0.571233068118
O     -4.336480421862   -0.115380466623    2.251690902846
H     -3.911700073257    0.778736866858    2.344293237013
H     -4.682359931306   -0.341705026423    3.114874341956
C      0.061299565447    1.769269525689    0.642112939043
C      1.191207687370    1.618760272869   -0.110298426632
C      1.962203172062    0.410383229119   -0.240362414183
```

```
C      1.622278282925   -0.796407133562    0.347371588898
C     -0.160056753048   -1.412725245082   -0.753037303401
C     -1.216691663786   -0.665379949396   -0.226366117705
C     -1.620997066164    0.621514977531   -0.810063953530
O     -2.637814760279    1.260428995380   -0.480238205354
H      1.483053573932    2.441879906536   -0.764607778460
H     -0.491123130359    2.700158714275    0.630659567824
H      2.737401335307    0.409723550281   -1.006467527603
H      2.236905045992   -1.671735704520    0.169834199903
H      1.072135410583   -0.828293195314    1.280416296121
H      0.217353420897   -1.177389744007   -1.737428702843
H     -0.044435223869   -2.450566257932   -0.464719028640
H     -0.217215115616    1.086745214800    1.433805605872
H     -1.085159160364    0.921934286517   -1.733000869729
H     -2.709097486648   -0.596726293999    1.226391998499
N     -1.856423974019   -1.056356627140    0.931708183376
H     -1.724757178120   -1.987334106204    1.287300176712

Ambimodal TS Water5-161
35
ANGSTROM
O     -5.652430054525    3.459876544698    1.131825295264
H     -6.135503879718    3.810176781256    0.382750815107
H     -5.571396888316    2.489773695915    0.975816651678
O     -5.082999359240    0.892438664018    0.604614761331
H     -4.725246222734    0.532653880335    1.435134491436
H     -4.296501908699    1.060290207568    0.061426754111
O     -2.444227305675    2.299896683791    3.461430161240
H     -2.653260372871    2.872385892573    2.676295278142
H     -1.506075542045    2.404523960660    3.622859105345
O     -2.939050730376    3.628625487685    1.233841369521
H     -3.909535878466    3.749739604606    1.266317432937
H     -2.796485032914    2.958779677083    0.547816602562
O     -3.636654732290    0.025978013865    2.793043866765
H     -3.182734727916    0.863388573161    3.055844000338
H     -4.177050032057   -0.222269209526    3.543765185173
C      0.102734838518    1.526330797360    0.982185410374
C      1.255032117075    1.456595232182    0.254157116595
C      1.966101111287    0.252246866517   -0.084135568539
C      1.533364050728   -1.022933597078    0.246186104201
C     -0.227324674804   -1.333210182160   -0.978659926419
C     -1.260203268857   -0.596990333143   -0.385981736981
C     -1.561235063855    0.784974299365   -0.799524532962
O     -2.563780327614    1.428494364896   -0.461061425297
H      1.619586876414    2.372139345633   -0.214661204581
H     -0.410406489158    2.468060106938    1.124940471796
H      2.767701956689    0.351013396380   -0.815096055717
H      2.109326264016   -1.882498672857   -0.077575118110
H      0.959774233027   -1.198578190029    1.149148091692
H      0.198056955742   -0.989617111903   -1.910774187895
H     -0.201099292352   -2.408414334357   -0.846278532088
H     -0.251115937473    0.715965109975    1.604632099898
H     -0.914023169199    1.191726977111   -1.605302692013
H     -2.664709340537   -0.563968664338    1.162781128136
N     -2.014760978513   -1.125849714195    0.631700540699
H     -1.854115956953   -2.058341775563    0.967783662655
```

```
Ambimodal TS Water5-162
35
ANGSTROM
O     -5.649594611886    3.418039098801    1.585161668430
H     -5.507631046948    1.921001892358    0.633479787216
H     -6.466984572517    3.651276267880    2.024806679561
O     -5.264829174435    1.036208792881    0.296865484417
H     -4.553591037146    0.313652597010    1.564650727403
H     -4.441337885160    1.163865384707   -0.199232899044
O     -3.722747304396    2.566206598441    3.213528109068
H     -3.094912332210    2.976189656996    2.590885453073
H     -5.012910265088    3.152193202720    2.297294798401
O     -2.731716599014    3.692600779661    0.913804734973
H     -3.606947629443    4.073733341670    0.791787501630
H     -2.696896608576    2.927182857873    0.316039629428
O     -4.010302746447   -0.052949538355    2.319047502446
H     -3.696271882172    1.612766670376    3.022189383885
H     -4.595401146190   -0.625578904277    2.815461731562
C     -0.019787337122    1.497384696742    1.044503239317
C      1.153656584811    1.494884721361    0.347686750478
C      1.922870224312    0.329644885009   -0.003157770956
C      1.537343931717   -0.969649242572    0.285020517234
C     -0.180387954157   -1.319402904286   -0.999934992996
C     -1.255871061637   -0.626441124356   -0.431256803828
C     -1.582832772192    0.749647936929   -0.835274449594
O     -2.621999604921    1.358655097261   -0.538031569467
H      1.493609180278    2.437316091497   -0.084858541687
H     -0.570658166355    2.413538100173    1.208337950867
H      2.739047237980    0.480340601977   -0.709052694066
H      2.155581438615   -1.797391594862   -0.043794768363
H      0.944902641554   -1.190601046978    1.165398581990
H      0.264893648904   -0.947943000765   -1.911749223921
H     -0.121141579420   -2.394814115301   -0.880488261104
H     -0.356633219387    0.655976621923    1.634330183448
H     -0.911921359182    1.191238854368   -1.602574404820
H     -2.704832793269   -0.653400530682    1.076521158683
N     -2.034519178419   -1.198584927083    0.545493957043
H     -1.829205565004   -2.119145936152    0.890952194521

Ambimodal TS Water5-163
35
ANGSTROM
O     -4.929330521914   -0.032074717711   -0.648611421584
H     -4.170167308495    0.572715930392   -0.704548742657
H     -4.545093943234   -0.932967929568   -0.613603630504
O     -3.975499804084   -2.588491262006   -0.796273475722
H     -4.165883540076   -2.680291198309   -1.756924333523
H     -4.557994570822   -3.210861947871   -0.357659167417
O     -5.978031795538   -0.284599971552   -3.094423884208
H     -6.932664664957   -0.246689317721   -3.024260392139
H     -5.628698668350   -0.082738944385   -2.193216098797
O     -3.844713555498    0.686803162141    1.887908820684
H     -3.415807448973    1.349907232116    1.328465414274
H     -4.641675509612    0.436468591860    1.403766101130
O     -4.706476337710   -2.627593862202   -3.381511692142
```

```
H     -5.231677581586   -1.794650533347   -3.361570288191
H     -4.043237216509   -2.502083378595   -4.061425908787
C      0.145371669089    1.608830366799    0.927363731453
C      1.285479894644    1.540650794389    0.181369196837
C      2.016199790561    0.340038952154   -0.135086559863
C      1.623153497019   -0.932007463108    0.244146466210
C     -0.165240323083   -1.329978539162   -0.945949801460
C     -1.188371649321   -0.587517898174   -0.348163819814
C     -1.518350543754    0.773015020215   -0.815385453554
O     -2.544418245663    1.400419008105   -0.512546128420
H      1.625778243509    2.448602580029   -0.320433591960
H     -0.383124067536    2.543545160106    1.060168196508
H      2.803000558584    0.435976586736   -0.882857197066
H      2.211323795086   -1.788759358614   -0.064736921722
H      1.059066859351   -1.092682056604    1.155225958353
H      0.237455763261   -1.009544842412   -1.896199661485
H     -0.116683274618   -2.398660163278   -0.776115905955
H     -0.184895799389    0.810420665403    1.576518830758
H     -0.881634314252    1.162488895248   -1.637581455760
H     -2.609207331820   -0.515443323880    1.177563857529
N     -1.871715786469   -1.064491039856    0.741214340890
H     -1.837002423605   -2.039798079871    0.976835972766

Ambimodal TS Water5-164
35
ANGSTROM
O     -4.052959033660    3.591424417417   -1.322937923980
H     -4.636460179024    3.177348258226   -1.961712141325
H     -3.422742021479    2.905786972879   -1.046333535128
O     -5.246392440597    0.668419961571    0.146936442738
H     -5.093163454469    0.232315328691    0.998277211327
H     -4.358051183687    0.881188608657   -0.173574296382
O     -5.507655997554    3.276626182925    1.034376620415
H     -5.100508512363    3.703917587174    0.262949366207
H     -5.703734481583    2.368755771892    0.736357834133
O     -3.232728659703    2.460332811859    2.167576311260
H     -4.088138039482    2.896045763552    1.936778291439
H     -2.821305221280    2.282731676448    1.311612328715
O     -3.979624128937   -0.030225274911    2.526774367755
H     -3.692677490564    0.924702674705    2.506941840312
H     -4.241582091870   -0.210813892452    3.429116923868
C      0.138715973114    1.589475695249    0.924720948647
C      1.265042298931    1.505112330075    0.159420829934
C      1.983215094442    0.294357788031   -0.152870569909
C      1.601441218150   -0.966360522922    0.260671893061
C     -0.235548949333   -1.357536572431   -0.920042536693
C     -1.243616480139   -0.614841332131   -0.301929346043
C     -1.554592377333    0.751656093519   -0.746145195297
O     -2.554313017142    1.409624149713   -0.399222273946
H      1.600637297515    2.403409289964   -0.361753071111
H     -0.372297648140    2.532751397089    1.066556671347
H      2.757943757620    0.375546209345   -0.914965554077
H      2.173153355905   -1.833435774690   -0.048755021305
H      1.038847845127   -1.112081428248    1.174914538218
H      0.166768030135   -1.029851207276   -1.867040608469
H     -0.173988493750   -2.425068843121   -0.749256429107
```

```
H     -0.180500438896    0.803276632668    1.594927774818
H     -0.953041121395    1.125874277552   -1.599146055933
H     -2.664005176634   -0.590343489160    1.246094206154
N     -1.926667100706   -1.107311968904    0.782770080756
H     -1.811718628228   -2.062855310943    1.069324860640

Ambimodal TS Water5-165
35
ANGSTROM
O     -6.133258269712    2.630953656044    1.351052657364
H     -6.993963046623    2.873523116644    1.009650245195
H     -5.852454882998    1.816757638041    0.849851999007
O     -5.147576741668    0.465269882382    0.223386682183
H     -4.752593205912    0.155036505221    1.061443728441
H     -4.382721389997    0.763959665545   -0.295507507557
O     -5.742713685659    1.495562263378    3.834876277314
H     -5.986158937147    1.930476273300    2.994956377615
H     -5.425588800076    2.192038324401    4.411962710837
O     -3.307748374620    2.879501016511    1.599674231905
H     -4.251105627047    3.085711544726    1.547903871507
H     -3.066051360919    2.556883391007    0.718348594610
O     -3.639005702687    0.332403920495    2.508033758832
H     -3.349333006137    1.245682617476    2.295416091111
H     -4.362109013801    0.460685299741    3.146074297738
C     -0.091799253316    1.498505488900    1.059140541853
C      1.086724789000    1.509585069179    0.371642641316
C      1.871412873361    0.350520919588    0.034050961611
C      1.499446094197   -0.950615703678    0.332914658454
C     -0.206746291920   -1.339695668498   -0.950023386306
C     -1.288367004623   -0.630270101299   -0.413106458538
C     -1.606101903442    0.732959896997   -0.868536892543
O     -2.650247146666    1.354122069325   -0.623633007454
H      1.419188206049    2.453192931432   -0.064574504765
H     -0.661772163697    2.403571389929    1.219800734863
H      2.688692984760    0.504769656143   -0.669687235547
H      2.129280902406   -1.773697190942    0.014663259450
H      0.908549490961   -1.169743914722    1.214824555496
H      0.245815694780   -0.997928210934   -1.870168563068
H     -0.150185362519   -2.411154058609   -0.797108866601
H     -0.420257827530    0.654885723142    1.650011317749
H     -0.909395160786    1.150041289422   -1.626950983623
H     -2.694389201873   -0.608246297608    1.130166860941
N     -2.092032116219   -1.185393908050    0.556758443557
H     -1.863802262087   -2.084689222746    0.944002634252

Ambimodal TS Water5-166
35
ANGSTROM
O     -5.383353390076    3.159854787566    2.084875079291
H     -5.981917381629    3.785412419565    1.677123535804
H     -4.467136510309    3.394191941557    1.768240532871
O     -2.876328029665    3.523276585632    1.365544346091
H     -2.770874118182    2.983181850516    0.569052124603
H     -2.531484010479    2.958438640436    2.097273864372
O     -4.286854064908    0.589987224991    1.563718185562
H     -4.934738483351    1.263205707111    1.812556132718
```

```
H     -3.848830235086    0.938517652466    0.762563904517
O     -4.407966269295    3.013690589772    4.671846402613
H     -4.909617766511    3.023116272844    3.836513172400
H     -4.183056645776    3.925891251765    4.861381429828
O     -2.416762404717    1.691077354761    3.282919529717
H     -2.965850070556    2.067973560577    3.991801366915
H     -3.021450178028    1.098360286982    2.799803945411
C      0.147940643261    1.648892620013    0.857602381566
C      1.279292171367    1.519347744043    0.104448246609
C      1.990662212868    0.294112247569   -0.146338928377
C      1.576220651350   -0.948658924420    0.304901139379
C     -0.207790078595   -1.350473503270   -0.863217142477
C     -1.236385490938   -0.604547263435   -0.277729847785
C     -1.580645829152    0.742324352939   -0.760081526894
O     -2.582331607640    1.398019533376   -0.426773814420
H      1.626240075640    2.390833321708   -0.454617181351
H     -0.352351273068    2.604366182394    0.940615725328
H      2.777637899086    0.334000350143   -0.899380064437
H      2.150383097869   -1.831127193529    0.045132139359
H      1.014767539067   -1.045596581031    1.226726824918
H      0.188279674668   -1.046287676128   -1.821399664271
H     -0.151513347896   -2.415899840563   -0.675015860970
H     -0.184720423975    0.908478057897    1.571026857182
H     -0.985276411389    1.099127735614   -1.626315851700
H     -2.701497435205   -0.591799265997    1.200978902277
N     -1.898531096932   -1.078104936767    0.834649488173
H     -1.789643924105   -2.033964726454    1.123800785263

Ambimodal TS Water5-167
35
ANGSTROM
O     -6.532550984735    1.942590832349    0.444490086875
H     -6.686215927712    2.875194813131    0.291538905045
H     -6.150455335094    1.585772099971   -0.389168740885
O     -5.109697555406    0.830852459785   -1.562221230373
H     -5.110396607058   -0.074832242699   -1.211655358812
H     -4.236429396610    1.188345237013   -1.344488890585
O     -3.250955788850   -3.408053551849    0.633587740885
H     -3.901572353337   -2.710713590740    0.432889860405
H     -3.623348513072   -4.226562117818    0.303950513352
O     -4.363464398734    0.741021130101    1.586889407887
H     -5.188172742578    1.241638676627    1.447381695258
H     -3.724785750749    1.130865162269    0.955903720836
O     -5.105427488664   -1.379599467472    0.186981334748
H     -4.807553105255   -0.648921861611    0.793708083449
H     -6.013085267640   -1.562942117628    0.435826225083
C      0.202346760974    1.686801418326    0.782699588000
C      1.279146042768    1.523322991673   -0.037445998151
C      1.982649120985    0.285462966128   -0.268380052853
C      1.625545429982   -0.929189862319    0.284816930005
C     -0.264574250242   -1.431027966968   -0.728117277582
C     -1.228680033567   -0.619641656014   -0.128846575614
C     -1.596574329643    0.679142984847   -0.712254823794
O     -2.594574913802    1.353914596285   -0.402120299287
H      1.579865165414    2.363492577805   -0.666475535328
H     -0.315146574655    2.634860701117    0.848889472459
```

```
H      2.712556259199    0.290745121455   -1.077877397323
H      2.184102345392   -1.822038228315    0.028635034243
H      1.117801278069   -0.985675039896    1.239916654375
H      0.070245995716   -1.213608550266   -1.732400412905
H     -0.201132397488   -2.473471681612   -0.443113654340
H     -0.075887447330    0.967893356380    1.540364248103
H     -1.040552493023    0.964787582233   -1.629745008844
H     -2.560344437742   -0.424908262657    1.431975941125
N     -1.791221783341   -0.975469891040    1.082077069820
H     -1.852927312205   -1.955994461376    1.311896897139

Ambimodal TS Water5-168
35
ANGSTROM
O     -6.494347375686    2.165883844456   -0.843263670382
H     -6.210777299904    1.263725483567   -1.026750254227
H     -5.721683278325    2.728535243117   -1.054954605816
O     -4.124618505166    3.486452747549   -1.083631144906
H     -3.513537781225    2.755078631789   -0.873820287194
H     -4.078525393334    4.090481799416   -0.340199326608
O     -6.330770001188    1.750419300343    1.801998057770
H     -6.492192196905    2.069477117936    0.884094818737
H     -7.193922560415    1.575730377598    2.178212944010
O     -4.875002187744   -0.075147491188    0.346709091185
H     -5.350942637777    0.442272715938    1.022510946741
H     -4.108711606384    0.471243515655    0.106040157993
O     -4.079407370688   -2.660850712067    0.888067283369
H     -4.407452340838   -1.766440181025    0.673014216989
H     -4.840473782887   -3.238911118904    0.825923101958
C      0.241537251112    1.654660202942    0.824920636679
C      1.316880865690    1.518628037826   -0.003237801936
C      2.009499283718    0.287005506546   -0.286305308289
C      1.641036508636   -0.947680068902    0.216777790324
C     -0.239820113906   -1.391385211307   -0.810020507851
C     -1.204879844424   -0.599422375515   -0.188153346111
C     -1.573943734766    0.729319863273   -0.694624277196
O     -2.573635315062    1.362099597997   -0.306355322832
H      1.623413674918    2.382653417084   -0.595902225723
H     -0.266684653005    2.603807128870    0.933966387712
H      2.739166051309    0.318971846228   -1.095202201686
H      2.194947804217   -1.832598449670   -0.075009728679
H      1.139316268934   -1.037762469307    1.172727301133
H      0.096373692685   -1.136671345781   -1.805243359681
H     -0.183036814316   -2.445637658079   -0.569386153460
H     -0.039642545613    0.907387063791    1.553413452803
H     -1.035772837418    1.087720991746   -1.594648158071
H     -2.560014136118   -0.408585665754    1.324681923816
N     -1.788083565440   -0.978436720309    1.008964807550
H     -1.922299905146   -1.957543807853    1.209200286427

Ambimodal TS Water5-169
35
ANGSTROM
O     -5.535703309306    4.246637358276    1.048113736968
H     -5.677102658887    5.085155208694    1.488752277813
H     -4.560575039937    4.192462360238    0.877208113286
```

```
O     -2.906189212748    4.015868979399    0.801218636540
H     -2.760636909417    3.265207927919    0.205593375467
H     -2.649655788164    3.692460228601    1.687619146669
O     -3.732486100230    0.752774656866    1.948116607212
H     -4.649192894230    1.059542162684    2.103941800055
H     -3.477485824055    1.122031966620    1.078386311669
O     -6.156855414107    1.919180238860    2.219355263366
H     -6.885103914450    1.564146074907    1.708308546270
H     -5.953925378672    2.796282395442    1.820172853249
O     -2.464012094850    2.760935235439    3.197923577061
H     -3.034493074838    3.041713770064    3.914767658994
H     -2.860719221512    1.929482891660    2.848343570854
C      0.056548068738    1.318911154137    1.202758761704
C      1.215403779747    1.340198585572    0.481903801071
C      1.923465275246    0.182824507911    0.001041880180
C      1.487667203247   -1.120734353154    0.178534608501
C     -0.274297444619   -1.277301368706   -1.080061475326
C     -1.301865029435   -0.576319365677   -0.437023364559
C     -1.546151132009    0.847033069493   -0.723183594294
O     -2.534425155802    1.503987629024   -0.361192181358
H      1.588265803097    2.305559447389    0.135726616353
H     -0.451772962772    2.231087748729    1.483574466039
H      2.728671181624    0.364933478932   -0.710048327235
H      2.060231852070   -1.939150348408   -0.243222335673
H      0.909531772343   -1.397982745821    1.052430103181
H      0.175369170749   -0.858626461868   -1.968897733810
H     -0.272958597287   -2.360516441909   -1.047874687004
H     -0.301036121262    0.436799083190    1.715456156342
H     -0.860760523053    1.298351975996   -1.470736335951
H     -2.735842209506   -0.658588673397    1.078409059768
N     -2.103738499734   -1.193289061944    0.496713143792
H     -1.934079752500   -2.145505248088    0.767553698185

Ambimodal TS Water5-170
35
ANGSTROM
O     -5.910750733794    3.045538188963    0.759723301206
H     -5.820026245461    3.788861766826    0.162207554530
H     -5.080269951918    3.007666553125    1.279956914211
O     -3.492343047655    2.539443235714    1.890536495901
H     -3.091697988818    2.282856386686    1.040122529314
H     -3.696691567237    1.687797197457    2.316402014135
O     -5.219972596822    0.537658577477   -0.089909157799
H     -5.685282965197    1.371575835612    0.115330223803
H     -4.349329670842    0.812349465464   -0.418426676929
O     -1.321096947182    4.046056835524    2.745896853459
H     -2.117599301348    3.539165700207    2.511373052458
H     -1.609113936291    4.729466915563    3.352028048516
O     -4.262810376989   -0.062666136388    2.291620439891
H     -4.919859671194   -0.426165679635    2.884985083331
H     -4.717199559749    0.065191157962    1.415324917681
C      0.168567706485    1.571349013065    0.935919433490
C      1.273170213680    1.504341271065    0.136288205603
C      1.987599046516    0.303289435012   -0.211412737689
C      1.619861559946   -0.966225020838    0.201283106030
C     -0.223475980400   -1.363068783098   -0.891023748048
```

```
C     -1.225063989409   -0.606190561897   -0.275052156279
C     -1.558836791166    0.746020498095   -0.744153537562
O     -2.568870645263    1.391569381863   -0.414255333542
H      1.590212412020    2.412549199478   -0.379017799220
H     -0.335374926629    2.510590518061    1.118814020785
H      2.736477578533    0.392808239679   -0.998309549224
H      2.192522524263   -1.825591250286   -0.129130279412
H      1.105773811642   -1.117667206765    1.143082001082
H      0.145635307257   -1.062157624360   -1.860678795105
H     -0.171235314543   -2.429003779420   -0.703806455970
H     -0.121462015338    0.769105017333    1.600770499913
H     -0.955211981505    1.115756575672   -1.598783281793
H     -2.640303739576   -0.555565293032    1.262320248541
N     -1.881611484492   -1.073970576131    0.842232228229
H     -1.790518951998   -2.032964523967    1.125900942904

Ambimodal TS Water5-171
35
ANGSTROM
O     -5.361418049503    3.243132060893    2.115726074869
H     -5.884420711890    4.027016187526    1.947668954552
H     -4.438371079387    3.453149735178    1.799933961386
O     -2.857847941143    3.537897246788    1.355410942618
H     -2.767978898102    2.990993151111    0.561683333429
H     -2.507929016955    2.981415396629    2.088120010777
O     -4.331824084334    0.651792530719    1.523232915909
H     -4.963500452358    1.352432430721    1.730526231067
H     -3.863407314367    0.964404561016    0.723653590383
O     -4.619170361037    2.570302641378    4.677290088567
H     -4.958549356993    2.874527124485    3.813091323493
H     -5.218323607093    1.884701844081    4.975531886193
O     -2.416992724392    1.663597260581    3.252494140784
H     -2.951350415825    2.029844656191    3.976153334100
H     -3.043726968784    1.118690359544    2.741193263481
C      0.143691612255    1.661940575192    0.845121613090
C      1.280125203214    1.520575146536    0.101395740227
C      1.989002056120    0.290675329166   -0.131441064795
C      1.565934281727   -0.946334383930    0.328444611211
C     -0.208026088013   -1.350274191771   -0.849686649137
C     -1.239391990645   -0.597460125974   -0.278474745515
C     -1.579265261196    0.746773100286   -0.769635365581
O     -2.582780983365    1.403201421857   -0.442204799339
H      1.633875344324    2.385029230607   -0.464197470747
H     -0.353288179257    2.620278987618    0.914081694644
H      2.782245142334    0.320027007434   -0.878378855292
H      2.139129812622   -1.833339501042    0.082172572323
H      0.997613871273   -1.031851295302    1.247223021685
H      0.195908920753   -1.054076860628   -1.807096933353
H     -0.156906342717   -2.414648798312   -0.654005477867
H     -0.195737867991    0.931164712940    1.565391284225
H     -0.981345181111    1.099284307923   -1.635425916576
H     -2.715857372008   -0.567828307272    1.186400341442
N     -1.913352318592   -1.062037928084    0.831549670804
H     -1.810361472865   -2.015654629148    1.129659207771

Ambimodal TS Water5-172
```

```
35
ANGSTROM
O     -5.874379555104    2.856397105784    1.536650529183
H     -6.024417871756    3.793768370312    1.415494296223
H     -4.908447986575    2.745686679106    1.751745608060
O     -3.362829506577    2.242565702334    2.053550604552
H     -2.987688182545    2.134480645836    1.165434885297
H     -3.601428989517    1.326639827363    2.316614540548
O     -5.294820143689    0.638988847034   -0.143339179760
H     -5.727402758633    1.412131221225    0.248417457091
H     -4.419758012385    0.942687899763   -0.427356383877
O     -6.371328704534    1.033774338239    3.585826411598
H     -6.403478839956    1.660221299904    2.841853344429
H     -6.103415479954    1.541584107043    4.353279346294
O     -4.362922248076   -0.266373442777    2.219007291608
H     -5.074693235816   -0.084024094243    2.855396222470
H     -4.791445437680   -0.140096732223    1.343035271117
C      0.132591155721    1.633270745640    0.865812688218
C      1.248723257963    1.544626902387    0.085994491949
C      1.983299054718    0.338390498666   -0.193263732121
C      1.615870397106   -0.916842698147    0.263888844496
C     -0.196404880662   -1.379799647958   -0.839947429967
C     -1.222492933287   -0.629790525376   -0.254590980467
C     -1.584346399213    0.701058431759   -0.771681444087
O     -2.585103302758    1.356476996403   -0.444593264427
H      1.559744673052    2.430676333666   -0.470551675372
H     -0.402904160059    2.567038966043    0.975604487441
H      2.744567699392    0.402681920356   -0.970205798290
H      2.204493372500   -1.782185085785   -0.019408030674
H      1.088304690718   -1.034547261497    1.203166161173
H      0.174554668299   -1.098052036254   -1.814856073697
H     -0.122956968121   -2.438721471296   -0.622789045401
H     -0.161607717204    0.863543200255    1.565266314729
H     -0.994736214907    1.041100024848   -1.649715231457
H     -2.671175268470   -0.594583406465    1.249177942589
N     -1.860980011966   -1.072967280322    0.881461360378
H     -1.758658186943   -2.026910765620    1.180927227801

Ambimodal TS Water5-173
35
ANGSTROM
O     -3.925295128234    4.821564921426    1.118640745899
H     -3.083021717158    5.120966204080    1.485149376443
H     -3.738868099918    4.621278157863    0.181653566509
O     -3.335406738431    3.807626485700   -1.364668059186
H     -4.108240479841    3.643867334870   -1.906981066794
H     -3.020510383419    2.931827210789   -1.063742518366
O     -4.228260768201   -0.162989365563    2.228812461200
H     -3.966298083270    0.765435763590    2.384459743833
H     -5.058456516931   -0.124910704901    1.751137074757
O     -3.312364021263    2.378453089915    2.027508372976
H     -3.793759163922    3.201214682313    1.784675324187
H     -3.003803406075    2.018056000624    1.177066233368
O     -1.434554846876    4.333857740344    2.547121372106
H     -1.346146082809    4.548140774439    3.476190102303
H     -1.982022824394    3.526799375840    2.503759067228
```

```
C      0.141703586885    1.688220680352    0.763234879034
C      1.254132066412    1.554818638445   -0.016325526636
C      1.985518696234    0.333851207075   -0.242943536452
C      1.628621755620   -0.895316123907    0.277334682842
C     -0.215703871635   -1.397768751773   -0.831884235452
C     -1.230572242677   -0.642096621946   -0.239263232286
C     -1.586257614473    0.686631260095   -0.759034378708
O     -2.582233400356    1.351615802352   -0.414114225044
H      1.565656259502    2.412564585871   -0.614894700663
H     -0.371719992839    2.637817811620    0.838451598676
H      2.745407837274    0.362622021875   -1.023905928139
H      2.209527238058   -1.776263849018    0.029493505537
H      1.086251194058   -0.973591326008    1.211828149776
H      0.158973989791   -1.120165081161   -1.805909059232
H     -0.125935934275   -2.451269115815   -0.597927815146
H     -0.150883476523    0.955280547082    1.503101682641
H     -1.032973861058    1.005834481904   -1.666114865711
H     -2.684364547233   -0.600069024782    1.275487128374
N     -1.865444832013   -1.073165817981    0.900457133314
H     -1.727385187862   -2.012809795607    1.229189732493

Ambimodal TS Water5-174
35
ANGSTROM
O     -5.748969385377    3.016531013322    1.736321997316
H     -5.899191546693    3.923284892753    2.003583019364
H     -4.787244388028    2.827298988749    1.915198149741
O     -3.262006238876    2.229137321393    2.107111176949
H     -2.924643677781    2.125610234748    1.203070908560
H     -3.538635569931    1.323387747555    2.358518939904
O     -5.326543830285    0.905280127174   -0.139621669950
H     -5.667862133374    1.703727489540    0.286295997379
H     -4.434953475566    1.133350714812   -0.441779989511
O     -6.678527874559    0.864575772748    3.195557545243
H     -6.426279273749    1.688145351075    2.735424973719
H     -7.446750162961    0.527175006353    2.732253906668
O     -4.394965144102   -0.239410488418    2.129497655436
H     -5.112462867421   -0.074639162789    2.764354663869
H     -4.794836694239   -0.009753868299    1.261629528337
C      0.151589125269    1.638328588705    0.857592647385
C      1.265309952296    1.540001185795    0.075367382069
C      1.991168507795    0.328067975999   -0.201498896575
C      1.615502752341   -0.923202625976    0.260067774914
C     -0.201448353405   -1.373518085623   -0.839789154589
C     -1.222765638882   -0.618115678577   -0.252836292183
C     -1.578421994016    0.714980950044   -0.768179842221
O     -2.572018275254    1.378200977901   -0.434635105925
H      1.580918935622    2.422168310861   -0.484759823439
H     -0.377251823627    2.575973332385    0.966226639103
H      2.751557703416    0.384534021055   -0.979965967183
H      2.197443678836   -1.793620276236   -0.021628157484
H      1.088962552824   -1.034175459064    1.200723481756
H      0.169916971851   -1.093275365863   -1.814966012950
H     -0.133763013291   -2.432988578660   -0.623391587250
H     -0.145593211307    0.873185510192    1.560822282312
H     -0.992893307489    1.049416213038   -1.651223032902
```

```
H     -2.678956936597   -0.583894872450    1.243708856094
N     -1.862521767736   -1.058716727975    0.884053268652
H     -1.759712548855   -2.012493676125    1.184168300011

Ambimodal TS Water5-175
35
ANGSTROM
O     -6.021260266339    2.727847808578    0.661572050135
H     -6.089723071815    3.486233936995    0.080693690492
H     -5.169566051580    2.830168754646    1.146069035557
O     -3.537865130375    2.662387970067    1.705849058509
H     -3.125001069962    2.333148484281    0.891976152488
H     -3.604455855467    1.887619811314    2.279286974279
O     -5.139778840450    0.303168553017   -0.141909104796
H     -5.663453336334    1.105772056056    0.057672207495
H     -4.277274191130    0.638436361240   -0.442288901324
O     -4.864732122290   -2.372095564251    0.692618846841
H     -5.014355429154   -1.511736911117    0.269701099725
H     -5.539629724090   -2.956775640048    0.346669347028
O     -4.016477994185   -0.046533818936    2.318579282799
H     -4.298860221420   -0.892463398569    2.672159131561
H     -4.564604624095    0.090005607772    1.516475046708
C      0.060557167068    1.606041046064    0.911960890899
C      1.206783334981    1.573043323392    0.172731705788
C      1.982100652289    0.396318832239   -0.129224289852
C      1.639504121723   -0.884264311553    0.265577671070
C     -0.135483089604   -1.365443758004   -0.934415268768
C     -1.185310735834   -0.648910918337   -0.351796428407
C     -1.546370580262    0.696213408252   -0.831956377451
O     -2.590569874963    1.301996809372   -0.539434577533
H      1.516856828744    2.488096767873   -0.335508778003
H     -0.498925282072    2.523402900577    1.040184124888
H      2.768189256630    0.513866904615   -0.874721752743
H      2.259063370697   -1.722304730076   -0.032472094261
H      1.073620041784   -1.056858232804    1.173285849467
H      0.265483957664   -1.043795606789   -1.884710561384
H     -0.046437810447   -2.428548579200   -0.746361713493
H     -0.241735906035    0.800904551172    1.566897631928
H     -0.920480626697    1.093752165085   -1.657999421625
H     -2.615870407475   -0.606281662846    1.171612676348
N     -1.863562828326   -1.132603339921    0.737064350830
H     -1.801732887093   -2.100408928146    0.998086423583

Ambimodal TS Water5-176
35
ANGSTROM
O     -5.701921115441    1.788495268883    1.367831626183
H     -6.636745600187    1.912668044501    1.530183413538
H     -5.584160994617    1.793019470336    0.379551256444
O     -5.124895439375    2.029149636561   -1.191104661839
H     -4.201242888004    1.742398373778   -1.144906782957
H     -5.082285102130    3.004486265077   -1.228260911761
O     -3.448663849114    0.503673517669    2.307329270366
H     -2.985685357931    1.332961496350    2.460216585217
H     -4.347472225833    0.760470431418    2.043453553133
O     -3.403910626026    3.389518191132    1.282728175183
```

```
H     -4.249054473335    2.966632415098    1.506947352762
H     -2.981539604092    2.788361585108    0.647216192711
O     -4.540969882972    4.687494893245   -0.818500597790
H     -4.109350429331    4.417308189616    0.020485019343
H     -3.860548157752    5.102846954267   -1.349456818731
C      0.075934650582    1.465226330587    1.112938856861
C      1.288730642645    1.390094086467    0.492981125064
C      1.976717297764    0.176900735390    0.132206644174
C      1.467298418827   -1.094058813305    0.333068092834
C     -0.193209413264   -1.242907794916   -1.087009395500
C     -1.236018146026   -0.486340808455   -0.541787414287
C     -1.413981223705    0.934757574779   -0.885984382566
O     -2.411408001462    1.616441021398   -0.607994369441
H      1.738037839173    2.316468612461    0.130470518812
H     -0.405141686513    2.418543052005    1.289582150375
H      2.847195536991    0.287910885716   -0.513477441567
H      2.032123798530   -1.957466683896    0.000776144543
H      0.795275160424   -1.301833392684    1.157500475926
H      0.343883155083   -0.867607179114   -1.946399314919
H     -0.229029849180   -2.323627232149   -1.018484282955
H     -0.371633422581    0.631115967045    1.635235750435
H     -0.667111435169    1.352120599159   -1.595733641423
H     -2.716033613738   -0.424806796086    0.926333385937
N     -2.136784387222   -1.026862959048    0.340383359988
H     -2.021106173242   -1.970799892187    0.666540069188

Ambimodal TS Water5-177
35
ANGSTROM
O     -5.481142692920    3.006384442343    2.047471392112
H     -6.073530084414    3.551379412958    1.524040794917
H     -4.598367829306    3.373289239638    1.864201986815
O     -2.859719246399    3.671581479223    1.202530435024
H     -2.773043187754    3.040724314203    0.471065059371
H     -2.550011949970    3.179797495707    1.989653347561
O     -4.230469832025    0.632947468016    1.589859659488
H     -4.922954750168    1.290384379903    1.784592815148
H     -3.808730878628    0.955551396481    0.769270743659
O     -4.974819275802    5.327003980494    0.305444430193
H     -4.733434768888    6.204802396168    0.009250516929
H     -4.147728817799    4.885236871143    0.550459631950
O     -2.501837858496    1.893596643359    3.240502638259
H     -3.003939935802    2.162447039348    4.011494653759
H     -3.099624319511    1.304391675872    2.725865794690
C      0.172343245574    1.592672066610    0.901578242191
C      1.302877586017    1.480619970437    0.144298057018
C      2.004107358288    0.258576266775   -0.146836202576
C      1.581581234420   -0.994939569445    0.266700546686
C     -0.209011801880   -1.352442813734   -0.900946943957
C     -1.227603420645   -0.603824974103   -0.301285235371
C     -1.551166383221    0.757436128644   -0.757426550787
O     -2.545727835863    1.418578097789   -0.416328245387
H      1.655650112132    2.367415328263   -0.385971215141
H     -0.324865355271    2.546119687464    1.017528750385
H      2.788919124510    0.315952698486   -0.900821713332
H      2.148680751208   -1.872709400604   -0.022685797761
```

```
H      1.024649666339   -1.116816651397    1.188395077760
H      0.188927724912   -1.036259956670   -1.854485549698
H     -0.167555586013   -2.422052229507   -0.733112664061
H     -0.164658096697    0.829246550997    1.588023043076
H     -0.944087763098    1.124947873159   -1.610900353090
H     -2.689638880413   -0.593882249666    1.180697457696
N     -1.896742668544   -1.088810536088    0.802903495961
H     -1.813380733342   -2.055556995711    1.063369141761

Ambimodal TS Water5-178
35
ANGSTROM
O     -5.830412715018    2.787704642153    1.266033501227
H     -5.778356960499    3.660623252267    0.875686444282
H     -4.972559583108    2.640448061573    1.725874442381
O     -3.422448153533    2.000179584103    2.185588819026
H     -3.027245488092    1.880998561290    1.305431621076
H     -3.698237184724    1.114546756959    2.469451989467
O     -5.232852426581    0.500898032947   -0.086276782931
H     -5.656434930371    1.311847227252    0.259074356281
H     -4.353758094939    0.787558521968   -0.380234776951
O     -4.650888819296   -0.548110781711    2.245532581935
H     -5.430201876173   -0.461423021923    2.796690644028
H     -4.931860635282   -0.256576767303    1.332035161770
O     -3.513861976927   -3.100452440264    1.982268094127
H     -3.921036770630   -2.237391212653    2.170606342116
H     -4.220097439843   -3.670457366803    1.675183505171
C      0.170662980109    1.651852779630    0.826130350896
C      1.279517469050    1.546368633157    0.038623510529
C      2.011179197006    0.332929593133   -0.224432691648
C      1.653603772814   -0.911918722421    0.260001372482
C     -0.184175543347   -1.394936384381   -0.840479122412
C     -1.191611041217   -0.631857696051   -0.249121630378
C     -1.565212942717    0.699748984733   -0.755747259695
O     -2.573193030123    1.329887521093   -0.389214271053
H      1.587097171587    2.420793685235   -0.538120608576
H     -0.361512714911    2.588909956447    0.925191451054
H      2.767966234852    0.385437309431   -1.007008159387
H      2.236482605253   -1.783317644586   -0.015455486981
H      1.119043892502   -1.018352411250    1.196143856084
H      0.185847457429   -1.122064162513   -1.818272004705
H     -0.108113654596   -2.448931103742   -0.604472282933
H     -0.116552976793    0.894361077708    1.541998813986
H     -1.011325402958    1.058065373954   -1.647032469687
H     -2.616525454417   -0.505856093929    1.222952310790
N     -1.823043767938   -1.039027983025    0.904279774961
H     -1.838307866759   -2.005354155764    1.192086637936

Ambimodal TS Water5-179
35
ANGSTROM
O     -6.607158662747    0.658609459631    0.279847152338
H     -6.882534590803    0.691582929409    1.206056106613
H     -7.203432207432    0.060622748093   -0.171237808076
O     -3.245520736810    2.858357493290    1.587216344839
H     -4.137039691432    3.249519505224    1.498566656841
```

```
H     -2.962950227175    2.641429062891    0.686163005632
O     -6.366250931789    1.127590081420    3.090180385742
H     -5.498220267885    0.772649626347    2.780899803946
H     -6.296987591612    1.230241545786    4.039385890259
O     -4.146354995352    0.379926660252    1.793285928484
H     -3.701578306513    1.268571723811    1.823871032558
H     -4.653266196953    0.397079008434    0.968954093427
O     -5.929502242060    3.289081736169    1.317209809366
H     -6.204424102527    2.731474908907    2.062390308852
H     -6.097600876863    2.755276674124    0.532841648383
C      0.041963789519    1.526266418648    1.004579665819
C      1.210115971269    1.509345475520    0.299016239032
C      1.970514365761    0.334288649531   -0.028801096386
C      1.567575141135   -0.957983428856    0.290312021079
C     -0.137516888569   -1.337675449242   -0.931245048735
C     -1.214855702397   -0.604410074064   -0.410202494310
C     -1.539170444458    0.753127127284   -0.891817174376
O     -2.564150282921    1.381735772335   -0.620472125245
H      1.550907178575    2.441487507450   -0.155168730301
H     -0.516182849158    2.439471072045    1.157911403205
H      2.779067140916    0.459305919244   -0.747927886968
H      2.183640865227   -1.795798180635   -0.018426201399
H      1.002946324111   -1.148023863193    1.196330518136
H      0.300693201804   -1.023808810761   -1.868394678185
H     -0.104945288839   -2.408694963058   -0.764889755867
H     -0.290633624750    0.695519466554    1.610335627351
H     -0.855137668909    1.145245624686   -1.678654265640
H     -2.731603183755   -0.544138971360    1.022449158922
N     -2.004906135690   -1.110702690980    0.591867854649
H     -1.839294412326   -2.022462869549    0.978105314872

Ambimodal TS Water5-180
35
ANGSTROM
O     -5.105882462868    3.966136658092    2.094782404025
H     -5.380885843491    4.091228541064    3.003749597953
H     -4.320747469887    3.365939268069    2.127297007470
O     -3.149025375461    2.164082884070    2.175308778499
H     -2.902640850479    2.034260540245    1.245504382048
H     -3.578387546276    1.327632179891    2.427691205999
O     -5.228663739157    0.428819588368   -0.114729398272
H     -5.843276703069    1.176825807564    0.040429515824
H     -4.389529045252    0.829876935876   -0.388030332717
O     -6.715510734718    2.634598515675    0.406825198658
H     -6.841068546843    3.212209210238   -0.347141133293
H     -6.164515683177    3.142021074283    1.044384228041
O     -4.387280754624   -0.287581236876    2.258678032141
H     -5.099406362689   -0.625576084309    2.801218906052
H     -4.788181932814   -0.090879465268    1.366394752791
C      0.138022930962    1.645018203882    0.840894525813
C      1.247401747274    1.551582562337    0.051319013218
C      1.985234294340    0.345780941556   -0.218262046244
C      1.625127645031   -0.905665981671    0.257272341870
C     -0.190460016101   -1.384420430835   -0.821539724124
C     -1.216732875201   -0.634017492040   -0.236478989547
C     -1.594015694112    0.690195830765   -0.758506818684
```

```
O     -2.596652896476    1.337364883965   -0.420534828435
H      1.548174940710    2.431446842334   -0.520420831270
H     -0.402549158334    2.576451202669    0.943354705541
H      2.739545309568    0.402338807561   -1.002620611650
H      2.215429572091   -1.772122157821   -0.019546301573
H      1.109228281685   -1.012359552129    1.204369874120
H      0.169574607146   -1.110580930427   -1.802817873845
H     -0.112444518140   -2.441603050886   -0.596868383976
H     -0.143805989206    0.884862723086    1.555742862908
H     -1.020501654296    1.028424358610   -1.647513626591
H     -2.658415056505   -0.589656090137    1.270709505331
N     -1.849186743100   -1.072135608655    0.905940093719
H     -1.737665096319   -2.021797442533    1.215081156724

Ambimodal TS Water5-181
35
ANGSTROM
O     -5.352146741982    2.109289005783    2.801392144165
H     -5.874297905417    2.904932023946    2.900703154552
H     -4.436656343998    2.406977323539    2.563680504622
O     -2.867929434932    2.677718382771    2.065155030998
H     -2.811764698762    2.382435445722    1.143902113606
H     -2.353657596720    2.020134314894    2.573806002113
O     -4.754570219190    0.126411824440    0.876449718692
H     -5.243578670941    0.829586521406    1.328737968727
H     -4.106904290485    0.586847961395    0.315200662604
O     -4.228888676981   -0.385949691192    3.519753133515
H     -4.434143100782   -0.535142010760    2.579298696679
H     -4.740799429666    0.406361663785    3.731499746839
O     -1.750640785080    0.607996038999    3.475557926954
H     -1.481331221819    0.788884962808    4.376623419681
H     -2.622881942524    0.148147770147    3.537657924037
C      0.172128098218    1.663947905479    0.809248560611
C      1.300307004031    1.531558860751    0.052274039892
C      2.021526795084    0.307359023207   -0.176537908521
C      1.617826652324   -0.928672047126    0.300482487433
C     -0.170350394264   -1.366049444326   -0.861217889997
C     -1.202909917330   -0.626950991655   -0.277691464289
C     -1.578199300837    0.714896512763   -0.754853259989
O     -2.585442047752    1.339669605458   -0.380007882326
H      1.636832080348    2.394781348529   -0.525776216483
H     -0.339487099197    2.615463531479    0.875287267106
H      2.805470759236    0.338921259084   -0.933068719230
H      2.196477387970   -1.812483701545    0.056034837113
H      1.055922870788   -1.011941385375    1.223226796873
H      0.219999107523   -1.063529757655   -1.822167430958
H     -0.099854891446   -2.428622261357   -0.663253448817
H     -0.146787260479    0.929293627694    1.535639850933
H     -1.018136638212    1.090230234236   -1.635323183467
H     -2.661988458590   -0.575910995467    1.186537925930
N     -1.871256054443   -1.088118589422    0.831302093901
H     -1.752431333995   -2.026074133664    1.167636629052

Ambimodal TS Water5-182
35
ANGSTROM
```

```
O     -3.711290288481   -3.187199168291    1.166431948555
H     -4.291240484856   -2.400652344893    1.366269371734
H     -4.231143494838   -3.955306329775    1.405952740106
O     -4.907276264524   -0.226323408418   -1.046107467672
H     -4.366638499285   -0.987324158066   -1.329459692103
H     -4.284761933887    0.501028756184   -0.922646134104
O     -3.391457406520   -2.491867433616   -1.439487542779
H     -3.433325708133   -2.791102891512   -0.505283347808
H     -3.845935987985   -3.157446105752   -1.958098007056
O     -3.770965829137    1.178840511690    2.067907722304
H     -4.230588387418    1.980791307453    2.317705814086
H     -3.379282597352    1.349041990740    1.183159713332
O     -5.254539808459   -1.083473706663    1.490895063042
H     -5.338330923621   -0.796029397008    0.555526800607
H     -4.806169344917   -0.339036336733    1.925639532081
C      0.220495452859    1.682040848108    0.800214245340
C      1.296339225311    1.540327467483   -0.025016717287
C      2.007653816843    0.312000511761   -0.280414071549
C      1.659729635785   -0.914669280247    0.251833390711
C     -0.228074751077   -1.416896331439   -0.764151201131
C     -1.198690321338   -0.627520438551   -0.148394022776
C     -1.580443391498    0.682601661553   -0.698677935552
O     -2.579227253671    1.341518218134   -0.356782970789
H      1.589827892917    2.393946932174   -0.639088413399
H     -0.301251211169    2.626310749407    0.884436746343
H      2.734019083430    0.336263652107   -1.092545196246
H      2.224340797750   -1.798785382600   -0.020824825338
H      1.157404780809   -0.990230015120    1.208534285074
H      0.097445972075   -1.177093163303   -1.765741867050
H     -0.151885701603   -2.464883073247   -0.503774713148
H     -0.052432074019    0.948862479123    1.545950670453
H     -1.037737858427    0.995702063803   -1.613597942539
H     -2.509594014683   -0.443318146993    1.433552316087
N     -1.766096917610   -1.005769347413    1.050847808520
H     -1.807956902655   -1.979411954730    1.303782422274

Ambimodal TS Water5-183
35
ANGSTROM
O     -2.699850967674   -3.831976435567    1.106276362271
H     -4.109787094453   -2.742985729403    0.512403532757
H     -2.838999259804   -4.668062284403    1.552073608422
O     -3.161608806471   -2.011228235373   -2.190688052615
H     -2.240204102366   -3.411203903458   -1.782780788127
H     -3.712868957415   -1.854224398121   -2.957928626400
O     -1.919135359991   -4.273384108355   -1.437101403406
H     -2.353122191141   -4.042772322510    0.210348643037
H     -2.391775977115   -4.947443275011   -1.928374872664
O     -4.555721173908    0.331430235583    0.982228055068
H     -5.323986519929    0.902666748265    0.968059826657
H     -3.878484987981    0.766570514835    0.407560312349
O     -4.694761773732   -2.204067839213   -0.044289837272
H     -3.776293355783   -2.030339345550   -1.410726759855
H     -4.722890051368   -1.320658713147    0.370900066557
C      0.170913043539    1.672774630935    0.817678287965
C      1.284075118571    1.558179313405    0.040607788599
```

```
C       2.022243692788    0.343313807117   -0.198897657458
C       1.654576882693   -0.898049810801    0.292582648946
C      -0.149394378857   -1.406793223653   -0.796835141125
C      -1.174834832478   -0.640113226763   -0.238718769527
C      -1.568292011508    0.655151988299   -0.818141955189
O      -2.587310780729    1.298585357111   -0.532109934995
H       1.595764669980    2.425998707772   -0.544071025861
H      -0.374025648517    2.604613890155    0.888228220025
H       2.784553373815    0.386650714211   -0.976449128267
H       2.246490285741   -1.769781353956    0.037404355087
H       1.121272319689   -0.990381237259    1.231096057646
H       0.212619533244   -1.159253749663   -1.784352787471
H      -0.075862292350   -2.456784211937   -0.543079687919
H      -0.130213788693    0.919192572902    1.530879827158
H      -0.967904526680    0.983432256440   -1.692749947869
H      -2.597559453959   -0.512782801841    1.244685929537
N      -1.790816188746   -1.031483676353    0.935213619333
H      -1.793805708207   -2.006287981181    1.195510089737

Ambimodal TS Water5-184
35
ANGSTROM
O      -2.446910097601   -3.944529558913    0.327003101496
H      -3.336416608026   -3.796477459020    0.740850434388
H      -2.292056345314   -4.888988996332    0.365991644617
O      -3.116814211917   -2.546637701806   -1.957572735896
H      -3.399668866050   -3.181951571111   -2.616945568882
H      -2.787630460454   -3.060657710381   -1.195455715971
O      -4.845392105242   -3.416335526995    1.297722697717
H      -5.150041316139   -2.801884497306    0.575694653492
H      -4.913760162853   -2.911916281936    2.109767867668
O      -4.505301546716    0.565096894223    0.853275858507
H      -5.159333709902    1.243076617322    1.025332371170
H      -3.797577799591    0.999415055246    0.315050586200
O      -5.326862028743   -1.628411815313   -0.571496332059
H      -4.582101235161   -1.802514216165   -1.174651582126
H      -5.097887677069   -0.806228805491   -0.098583042961
C       0.121680446410    1.612521589138    1.072544102480
C       1.306549240095    1.596133167148    0.398672388437
C       2.056181868980    0.417664951173    0.046683180353
C       1.631302188463   -0.876457863889    0.302557546542
C      -0.058644977579   -1.169614702139   -1.013853280616
C      -1.132242652515   -0.474595618360   -0.448943407054
C      -1.448213835885    0.907078187977   -0.850497775187
O      -2.475431137016    1.534872143835   -0.560548367230
H       1.678727708327    2.536789184768   -0.011682861303
H      -0.421561938733    2.533933417492    1.234111713459
H       2.891609529807    0.561688102895   -0.637939206739
H       2.240190408139   -1.711511577397   -0.025673712102
H       1.012075014687   -1.099139635421    1.163328302549
H       0.404646268576   -0.787945803127   -1.912372097766
H      -0.022077986192   -2.247000529613   -0.910825751406
H      -0.250211004754    0.766003153441    1.631615091636
H      -0.763673496156    1.343295188412   -1.608914106950
H      -2.714687713847   -0.581367897964    0.869260262637
N      -1.864923353974   -1.037635316363    0.577172309853
```

```
H     -1.850844740057   -2.040063904713    0.689759671242

Ambimodal TS Water5-185
35
ANGSTROM
O     -3.982703535243    1.804054151925    5.087613060288
H     -3.051747243255    2.031455116293    5.203269052966
H     -4.288003103368    2.297986211994    4.320672811620
O     -1.117568345325    2.038594472442    4.547298953856
H     -0.185914888897    2.048021176453    4.766955227229
H     -1.256314744413    2.765628372673    3.894788617472
O     -1.722533251437    3.896119913906    2.736044866469
H     -2.487645271762    3.365176399271    2.385518317499
H     -2.079783921615    4.750775873344    2.979634782683
O     -2.659012982132    0.101639868186    3.326492565714
H     -3.350706970072    0.105745717400    4.002096892791
H     -1.945352492895    0.638672607916    3.717261951225
O     -3.688362289586    2.299089233853    2.015475391596
H     -3.395117492514    1.468156294325    2.440217296277
H     -3.538232663919    2.137534348636    1.070153139540
C      0.150572308107    1.458200245264    1.022017470343
C      1.233715631977    1.456583852635    0.190886812066
C      1.939231903681    0.289988095405   -0.261777338200
C      1.555158849096   -1.011960561181    0.048867087792
C     -0.266746309591   -1.326939434134   -0.980930576395
C     -1.270624674073   -0.603262541816   -0.317460476849
C     -1.646395885186    0.765263933801   -0.721188497964
O     -2.630501671320    1.386429184036   -0.319147919461
H      1.532175707481    2.402635478112   -0.263826916042
H     -0.373508079762    2.373509765648    1.260574129660
H      2.664977157485    0.435783514118   -1.060608427515
H      2.125818999674   -1.840554896036   -0.356873069837
H      1.099355210500   -1.233702886822    1.007991604773
H      0.060535614922   -0.988550404493   -1.954750369591
H     -0.232585907004   -2.403514379914   -0.854477289314
H     -0.121728996839    0.608282882661    1.632017845048
H     -1.052044344033    1.181229973649   -1.566523716802
H     -2.498890003773   -0.567718193743    1.356690572002
N     -1.963175717479   -1.157622436776    0.736892433243
H     -1.684212104791   -2.044031657916    1.120782654671

Ambimodal TS Water5-186
35
ANGSTROM
O     -2.412055941994    2.804709480446    3.224148388619
H     -2.593378782398    3.264135416573    2.353610419076
H     -1.682271896829    3.275324975302    3.627054176504
O     -3.057978080676    3.838318169477    0.904289095676
H     -2.855167336016    3.169659140192    0.234416107078
H     -4.030074708784    3.770738065962    1.042139643850
O     -4.899311453303    3.223894552333    4.315259332242
H     -3.972281142539    3.106793530127    4.030200906475
H     -5.154611836248    2.408237755123    4.748944493404
O     -3.878092359437    0.786897083395    1.854532161687
H     -3.371493982793    1.303244521381    2.496175106157
H     -3.571562528175    1.104036365140    0.980634680778
```

```
O      -5.551789379619    3.105733168831    1.628116126661
H      -5.538611199072    3.356527650546    2.568164984506
H      -5.330651350504    2.162810131025    1.618340619476
C       0.176720551565    1.642887565729    0.848694618072
C       1.290871818970    1.519796799376    0.069696030728
C       1.996755871628    0.295615865593   -0.201209646875
C       1.597191611750   -0.949271163039    0.259295562048
C      -0.220757267298   -1.369877934580   -0.845911421158
C      -1.229473672821   -0.602676956760   -0.251668604718
C      -1.561877524810    0.741376938026   -0.757370117419
O      -2.541508913308    1.423886270605   -0.419929146066
H       1.620357238267    2.393877449933   -0.494767620451
H      -0.336678478427    2.590906956852    0.940617407067
H       2.760454117468    0.337531813319   -0.977112698605
H       2.165759203349   -1.829335944371   -0.019510422402
H       1.067234660994   -1.051060821247    1.199326288882
H       0.151751295617   -1.090289003159   -1.820849659270
H      -0.168477547657   -2.431750904715   -0.636346204091
H      -0.133933103597    0.889789149948    1.559394702577
H      -0.972207679163    1.068354841860   -1.638952874898
H      -2.656669715455   -0.526939329670    1.273118528006
N      -1.873223755836   -1.044381759320    0.883542553624
H      -1.780683277937   -2.001499855121    1.175386947445

Ambimodal TS Water5-187
35
ANGSTROM
O      -2.358168929170    3.367277837874    2.654611292119
H      -2.548968816637    3.661966361693    1.726781694827
H      -2.479717853917    4.137290775275    3.210305334910
O      -3.162859993424    3.874219668330    0.175260475819
H      -2.981504656786    2.986268187482   -0.177992316451
H      -4.122404857080    3.887248124531    0.329983084962
O      -5.724398620752    3.345494947338    1.127764594367
H      -5.300454757235    2.546591045432    1.502268198419
H      -6.244733360117    3.735237710897    1.830688184783
O      -4.093727848022    1.361174607323    2.072995842297
H      -3.468358713317    1.984279979169    2.494251163502
H      -3.713384669170    1.215029788176    1.184799070783
O      -3.554907214175   -1.257375946965    2.453783294766
H      -3.767442191535   -0.300915703255    2.476700932991
H      -4.390470147063   -1.715248304601    2.552940194294
C      -0.214713736626    0.916695336267    1.447950816266
C       0.981882751752    1.181788295320    0.846643708186
C       1.841218054005    0.213276126662    0.216735685264
C       1.537147816176   -1.132677770510    0.098723264387
C      -0.109042727721   -1.183856720494   -1.329048462395
C      -1.243750904424   -0.740313568228   -0.638829013956
C      -1.626609300548    0.678961811397   -0.651283555076
O      -2.702992707013    1.152311991043   -0.252594904472
H       1.272028939118    2.226513759415    0.723141075857
H      -0.828380354158    1.714026069194    1.845973675053
H       2.669903109983    0.607851086012   -0.370678859131
H       2.220886874410   -1.794162817090   -0.421252093822
H       0.927437549814   -1.631987233886    0.842882706809
H       0.346924671719   -0.543410214556   -2.070618837984
```

```
H      0.014493665651   -2.243693670970   -1.518434576172
H     -0.514095379829   -0.076484994375    1.754394699563
H     -0.951501436897    1.337617112936   -1.238446718268
H     -2.666196394978   -1.303687921573    0.792587951890
N     -2.033443972990   -1.622638056542    0.063853810474
H     -1.730467759085   -2.576869099370    0.165346180828

Ambimodal TS Water5-188
35
ANGSTROM
O     -1.840674575266    1.952410020445    3.526899119121
H     -1.822422322072    2.854791286830    3.137695061457
H     -2.041259705973    2.071291690314    4.455998323873
O     -1.996484523127    4.400188788621    2.449430668084
H     -2.547237251939    4.295433080093    1.628321635487
H     -1.284019062761    4.995176191275    2.215225946424
O     -5.497390716632    3.010109045342    1.762566653051
H     -5.029539833068    2.154024202961    1.866862706030
H     -5.479267806809    3.434853889841    2.621524931543
O     -3.767282646593    0.922225936532    1.908241827093
H     -3.095100491036    1.249757967720    2.540207969801
H     -3.441826335990    1.194321470135    1.025507763362
O     -3.428373097076    3.996164497458    0.269066103145
H     -4.293910831815    3.756577551297    0.656185032989
H     -3.113772497662    3.190310790370   -0.166752817531
C      0.157446638402    1.672080623509    0.791585126610
C      1.285405733022    1.524262315931    0.036564978509
C      1.996904006509    0.292397114322   -0.180957247527
C      1.587153142495   -0.935737374866    0.310168395952
C     -0.218672646921   -1.376810826118   -0.836020544921
C     -1.235736526410   -0.605100293581   -0.264537420903
C     -1.558509766011    0.737575884857   -0.782150643473
O     -2.538355534104    1.425811707914   -0.453637552494
H      1.624354249057    2.377565696441   -0.554526914452
H     -0.355031819097    2.623186333501    0.845270637283
H      2.776690884215    0.309979953402   -0.942028059423
H      2.156414580505   -1.826547847270    0.069755474833
H      1.030643444984   -1.007120739669    1.237198145028
H      0.175824028052   -1.103820107761   -1.803893855352
H     -0.165828345163   -2.435884880811   -0.614245079480
H     -0.162108004691    0.953780182025    1.533032047556
H     -0.970883745708    1.051205352134   -1.669785699385
H     -2.665645080636   -0.505147988436    1.259341898413
N     -1.914145189733   -1.046798303994    0.846163679146
H     -1.781011457649   -1.980204210447    1.191461638792

Ambimodal TS Water5-189
35
ANGSTROM
O     -1.398680006553   -2.211652869956    3.679627067730
H     -1.325122321836   -1.230604416205    3.745179024897
H     -1.117252682147   -2.563671644948    4.524915668936
O     -1.270842187819    0.452947692107    3.778119031365
H     -1.989053434973    0.824567327509    3.205086912182
H     -1.293031422843    0.969157664503    4.584385337033
O     -5.317118737901   -0.381808292525    2.700408014724
```

```
H     -4.873129812046   -1.251566288421    2.784880150141
H     -5.969725730823   -0.340559241522    3.399707879900
O     -3.956602651872   -2.719768903600    2.869867441344
H     -3.053433054469   -2.563023942007    3.210657562180
H     -3.850746103879   -3.005844050989    1.960527503584
O     -3.252998601790    1.344282437945    2.275894470684
H     -4.079351621665    0.840557695564    2.397041292883
H     -3.137463777093    1.452495795543    1.312729196702
C      0.172904954262    1.631818936563    0.754512050589
C      1.257587693183    1.520407246988   -0.066579575435
C      2.021090515029    0.321182664682   -0.276353835889
C      1.688136777265   -0.907850874601    0.281875049061
C     -0.118011333888   -1.472934026663   -0.702030664327
C     -1.160921801841   -0.710879149477   -0.158938015796
C     -1.613333944201    0.564027895574   -0.751252841629
O     -2.601812009453    1.205504731128   -0.384999925546
H      1.509386039192    2.366615255179   -0.708622794853
H     -0.403417914443    2.545832987814    0.805285293627
H      2.751551788098    0.340615026764   -1.084442475631
H      2.292910948326   -1.777252581907    0.046984834020
H      1.215988007393   -0.960057393413    1.256488567831
H      0.208894225518   -1.268836960126   -1.712301888955
H     -0.021052453977   -2.510243304358   -0.403332171195
H     -0.049454322880    0.918571333283    1.535334824744
H     -1.088927971042    0.863873684853   -1.685682361800
H     -2.446070832657   -0.469488213796    1.422177270177
N     -1.842085057909   -1.137761362336    0.963547507731
H     -1.440975410613   -1.839767047381    1.564657089155

Ambimodal TS Water5-190
35
ANGSTROM
O     -2.382891819650    2.633539747139    3.077048248636
H     -2.455067181704    3.183111451510    2.267876278970
H     -2.701152510910    3.178241278572    3.797720832155
O     -0.553856862429    5.177447713305    0.475605081153
H     -1.445105331512    4.807491115632    0.556712630457
H     -0.667583609806    6.122429661350    0.367297017599
O     -5.548106100887    3.051832844241    1.378455112077
H     -5.150952104581    2.185999408102    1.619901840844
H     -6.223807708058    2.865897148215    0.725769756687
O     -3.980652042930    0.872576612417    1.762423138343
H     -3.418191484103    1.343992306339    2.408367482120
H     -3.581435484333    1.093124174964    0.893580587956
O     -3.017379385058    3.866644935731    0.723529393069
H     -3.972197771496    3.802819683143    0.919547389277
H     -2.828567686511    3.118887835233    0.132060970227
C      0.175168608203    1.667714907754    0.783865392166
C      1.297253744659    1.502955675210    0.022287031305
C      1.995055565187    0.264438079659   -0.202437733869
C      1.585930534845   -0.960032757844    0.296216159850
C     -0.236712428375   -1.383513320139   -0.839333260514
C     -1.238866792157   -0.600018809041   -0.260607149977
C     -1.555222796151    0.739206527866   -0.778136847156
O     -2.551083971519    1.419944016165   -0.464053259762
H      1.640728390799    2.357679379944   -0.562167796557
```

```
H     -0.300470516662    2.636805921955    0.845593889002
H      2.771094633038    0.275209096925   -0.968046475557
H      2.146163527742   -1.855815924732    0.052478655605
H      1.032108032114   -1.026961151652    1.225176265095
H      0.154462424547   -1.115498742937   -1.809826982925
H     -0.189700278920   -2.442101022810   -0.614127503779
H     -0.152756289437    0.951684695247    1.525242029459
H     -0.966546070223    1.057157878813   -1.661292847831
H     -2.673937073443   -0.506555386487    1.257859869864
N     -1.896753801735   -1.029649185213    0.873837854892
H     -1.798932146083   -1.979187598056    1.186366672254

Ambimodal TS Water5-191
35
ANGSTROM
O     -4.032241946730   -0.244632377222    2.588093726019
H     -4.394101058258    2.888403167719    1.551520594445
H     -4.025227067047    0.722781837523    2.437339585441
O     -3.655113602012    2.339986052407    1.890185624180
H     -2.450758272655    3.635461471062    1.954535138452
H     -3.260022967347    1.947659615819    1.086443205466
O     -2.964365413690    4.111118854252   -0.812500359266
H     -2.824124696350    3.152190824933   -0.889024948183
H     -3.923069241412   -0.366209793175    3.531790019100
O     -5.293364123650    3.965122057478    0.494616192325
H     -5.958842677501    3.606804040823   -0.093571317840
H     -4.504036895735    4.155710588333   -0.069155753815
O     -1.853465216988    4.375640507207    1.713990379307
H     -2.413997756570    4.378194122645   -0.054835186234
H     -2.188157699195    5.147482385536    2.172567480586
C      0.167237481221    1.637554504192    0.847491503607
C      1.267580409885    1.534952983410    0.045933115623
C      1.988151898615    0.322223342389   -0.243336902798
C      1.626958466724   -0.928238012343    0.229374642471
C     -0.215406574259   -1.387939716366   -0.841736135268
C     -1.223018262130   -0.630209893163   -0.234023713963
C     -1.568455878029    0.705855483469   -0.740627879746
O     -2.554719371836    1.382651688462   -0.403748404646
H      1.574326717413    2.416774127514   -0.519119149681
H     -0.349026285224    2.580480723336    0.970837477892
H      2.733859891994    0.377871124118   -1.036149930905
H      2.204160666085   -1.798807919017   -0.061218782780
H      1.114278906574   -1.038766612192    1.177674550772
H      0.141855066816   -1.107222824792   -1.821833068623
H     -0.143208015502   -2.447272182290   -0.626144666944
H     -0.117574571348    0.871155929401    1.555323758969
H     -0.994384530669    1.029059609117   -1.634772945008
H     -2.653271074991   -0.602511084129    1.302846181511
N     -1.843726925730   -1.074196023901    0.911724372880
H     -1.736935059629   -2.033037478520    1.195105328820

Ambimodal TS Water5-192
35
ANGSTROM
O      0.300265064369   -1.345491183765    3.468826359001
H     -0.261719307346   -0.620621093177    3.816679971667
```

```
H      0.871903720165   -1.612439900048    4.189204189088
O     -1.473751540040    0.525224072185    4.284972734727
H     -2.282288567265    0.013606767962    4.086788381010
H     -1.511218496157    1.308989668833    3.732994994767
O     -3.706967354220   -0.825792827450    3.471517187644
H     -4.478772459805   -0.911058238630    4.032724346640
H     -3.948206536776   -0.204763806782    2.752332633811
O     -1.810075681417   -2.949976872155    2.672382891302
H     -2.519405153253   -2.420145012186    3.055449351430
H     -0.984821477936   -2.516205500033    2.952582156310
O     -4.248565864331    0.938518942611    1.499045505775
H     -3.626955943653    1.153068853933    0.758696750914
H     -5.126047579586    1.076747023852    1.140410467875
C      0.005087776611    1.427159440209    0.989138311347
C      1.169475045925    1.427861668552    0.282379828169
C      1.933387470985    0.253237715554   -0.060127292156
C      1.557685805997   -1.034867825004    0.266408207411
C     -0.209548887753   -1.400384486031   -1.020392123322
C     -1.279081664153   -0.690512750981   -0.474462777799
C     -1.586061834476    0.694553290996   -0.884329747998
O     -2.594531314298    1.336193336859   -0.550598923809
H      1.505926635896    2.365773163822   -0.163892445418
H     -0.556080715811    2.337905027315    1.154851357074
H      2.738586719802    0.396264781562   -0.781399520817
H      2.161434031050   -1.870605867824   -0.068712686910
H      0.967739912935   -1.243978537019    1.149856187207
H      0.261946295852   -1.044461241312   -1.924591853949
H     -0.149707679797   -2.468969350206   -0.861337470822
H     -0.313948386292    0.573995161115    1.570076744839
H     -0.932155831289    1.116504284875   -1.675853243055
H     -2.790340516220   -0.655969436617    0.914511849903
N     -2.105026637831   -1.244302069617    0.471256796698
H     -1.878881798797   -2.097390684289    0.972981245779

Ambimodal TS Water5-193
35
ANGSTROM
O      0.121177719136   -1.155506584995    3.337460335100
H     -0.721279055479   -0.671624765307    3.418238874556
H      0.496298311189   -1.113887749257    4.224753348083
O     -0.206302173531   -0.073132099888    5.951047745392
H      0.023175000197    0.764250493278    6.355539713707
H     -1.016910238589    0.078104239045    5.444119999153
O     -2.296575991576    0.017411475226    3.999844398027
H     -2.866499374102   -0.746641544221    4.112033167891
H     -2.743096119191    0.589974592077    3.338940216805
O     -1.008377085644   -3.360729781504    2.248545856598
H     -0.353551903817   -4.024274172237    2.029340471930
H     -0.527946476155   -2.644490571887    2.714258338122
O     -3.624042138526    1.349542220487    2.092564356396
H     -3.204473780348    1.448721235377    1.200444284600
H     -4.087096051058    2.171934988034    2.254245463694
C      0.332296786129    1.523068702030    0.927790161590
C      1.414027514680    1.410125925648    0.105978415384
C      2.058036665460    0.171835327680   -0.253162338420
C      1.618560538839   -1.071962828953    0.160681348983
```

```
C     -0.267958030756   -1.342571907651   -0.944782992711
C     -1.234907151243   -0.570108844649   -0.297691541337
C     -1.502676301829    0.826164016663   -0.694093079212
O     -2.426612566475    1.535933073586   -0.266676457686
H      1.764234932284    2.301448394391   -0.418783531073
H     -0.143139762613    2.478516895146    1.106826720473
H      2.800068872169    0.225360272656   -1.050288162626
H      2.130746457854   -1.962920444906   -0.185485410904
H      1.099487873588   -1.195384942463    1.102896655408
H      0.124659093189   -1.018626889840   -1.897314836608
H     -0.260931114815   -2.413620557163   -0.790026795256
H      0.024070959511    0.727912557104    1.590449785591
H     -0.924245263683    1.189194325941   -1.569501643109
H     -2.617225298974   -0.491314236407    1.234241674796
N     -1.960780250765   -1.076973404599    0.749748007319
H     -1.767703368718   -1.990549882670    1.138870327398

Ambimodal TS Water5-194
35
ANGSTROM
O     -1.499232160092    0.735607882210    3.639246730894
H     -1.851438138330   -0.115312906257    3.983239936107
H     -1.305850634235    1.275667878417    4.406107025783
O     -2.731235449774   -1.521681599765    4.450719190134
H     -3.618739055656   -1.282852438412    4.144746064662
H     -2.475105171937   -2.260970676145    3.884288356836
O     -4.885396623137   -1.076942078631    2.678865575662
H     -5.833178986758   -0.948401579762    2.635461149066
H     -4.484229282167   -0.204494482115    2.420833200038
O     -3.189681326724   -3.235198441825    2.226490942647
H     -3.863389677742   -2.532046139974    2.256638067854
H     -3.663218302908   -4.067725291076    2.219507747174
O     -3.697070240139    1.190994066101    2.072507119953
H     -3.409805588507    1.409195517208    1.162584312334
H     -2.888419599152    1.212005727700    2.620157656192
C      0.202644005562    1.673260051215    0.821256205147
C      1.322772523747    1.530636911576    0.053606007625
C      2.023575204274    0.298946569507   -0.190003598936
C      1.595100042264   -0.937535373062    0.273619151933
C     -0.191385536158   -1.345274479410   -0.855366796618
C     -1.207606548754   -0.578243192357   -0.273527001009
C     -1.562253121691    0.771527817903   -0.760004826712
O     -2.547515385795    1.421036721605   -0.388329434202
H      1.665116468624    2.393048117638   -0.522642519774
H     -0.305546539106    2.626140429790    0.892283232146
H      2.799830256950    0.323347764141   -0.954553978584
H      2.161649032917   -1.826504342273    0.018258783191
H      1.052172930651   -1.019066141590    1.207995214754
H      0.191581123095   -1.063554671670   -1.826078013611
H     -0.154942946652   -2.408915935745   -0.651787049267
H     -0.117835163980    0.941142619073    1.548619064156
H     -0.992078991065    1.122186936864   -1.647110453061
H     -2.617001454799   -0.445722015447    1.220439935634
N     -1.889851259060   -1.022575504975    0.829511641469
H     -1.828731784854   -1.967597440196    1.167673341654
```

```
Ambimodal TS Water5-195
35
ANGSTROM
O     -0.974488236390   -0.020261576554    3.481757514740
H     -2.158168909920    1.210838602588    2.808124337445
H     -1.531780558077   -0.827704701114    3.458105692373
O     -2.957116044706    1.652083574792    2.469051813972
H     -4.170171477604    0.413390920508    2.798614160919
H     -2.833788087905    1.746788235699    1.512907056863
O     -4.642417009991   -0.442646513352    2.717730419324
H     -3.499797462667   -1.556275349623    3.156126061025
H     -4.943116030877   -0.461148263056    1.792737127840
O     -2.720002410304   -2.104171517540    3.436085131744
H     -2.997852628128   -2.560642974661    4.230972579981
H     -0.793193489725    0.159247924347    4.405261096533
O     -4.997703536091    0.124455994578    0.048684952711
H     -4.163399984476    0.595412030200   -0.136651614774
H     -5.699423876864    0.752094646258   -0.129704218002
C      0.176666864633    1.683674905427    0.785843238238
C      1.304083215548    1.534642415171    0.031798215671
C      2.023378258445    0.300100977871   -0.155912714756
C      1.617390591088   -0.915373302082    0.360503738883
C     -0.187472755288   -1.378842051560   -0.835644244372
C     -1.220006095885   -0.630342225843   -0.271376613829
C     -1.570011536261    0.721944619288   -0.744578191717
O     -2.570885850386    1.354285864349   -0.355674951792
H      1.640102168978    2.378491122276   -0.574487974826
H     -0.341066092486    2.633126814651    0.834534316719
H      2.811963640423    0.307447171727   -0.908537810861
H      2.187209901356   -1.811624581389    0.143752892452
H      1.033053672621   -0.971031743118    1.270646250967
H      0.230478837349   -1.081357387534   -1.785891932041
H     -0.116521723582   -2.437226013520   -0.620237902407
H     -0.121354363607    0.960681778019    1.532202964112
H     -1.015623872864    1.095606997062   -1.627534084753
H     -2.682465859721   -0.521329020960    1.181566782690
N     -1.948427838330   -1.088890514917    0.793548518172
H     -1.796571518618   -1.981629391841    1.226020155634

Ambimodal TS Water5-196
35
ANGSTROM
O      0.322027781147    2.848559219476    4.088208252358
H     -1.510230697397    0.329515169062    4.466174932304
H      1.114514102950    2.876917114973    3.549622452931
O     -4.546659110914    0.125439968063    1.275315986344
H     -4.497984460717    0.525288556746    0.400219317714
H     -4.327604537815    0.845771073152    1.886237078414
O     -1.147301737073    0.649269171290    3.639278591309
H     -2.314868733679    1.547652574176    2.689972249065
H     -0.551997008327    1.399501592886    3.871488985256
O     -2.917693841185    2.169132576085    2.229196599019
H     -2.768966059060    2.024321503351    1.276372573795
H     -2.103538107273    3.611947955271    2.563921449248
O     -1.509741269306    4.356487527790    2.828361528564
H     -2.051207151209    4.961495073411    3.336445969166
```

```
H     -0.304913894520    3.484768489431    3.673863931254
C      0.210727361945    1.732388482858    0.757792807575
C      1.339799644439    1.546092179868    0.014282129279
C      2.026729569507    0.293768385941   -0.159015582413
C      1.576786609234   -0.910716034128    0.362529490597
C     -0.197311457523   -1.346037327839   -0.784635822006
C     -1.234356106903   -0.578698762998   -0.240727892811
C     -1.580327255373    0.755579973892   -0.767691617113
O     -2.554809886738    1.429678763258   -0.404380272554
H      1.706275881585    2.378657140309   -0.589878460509
H     -0.281842144592    2.695788729734    0.789356450448
H      2.815095563957    0.269869187661   -0.911030965562
H      2.134731259422   -1.818708821275    0.161445337481
H      1.014637465529   -0.938317530771    1.288741807148
H      0.206855178626   -1.077066088640   -1.750065773065
H     -0.150295767899   -2.405058279237   -0.560459608544
H     -0.127704187128    1.016948184747    1.493495485518
H     -1.017717368518    1.071914312995   -1.671960451063
H     -2.807472660115   -0.554556258277    1.119730613740
N     -1.923686185471   -0.997551016772    0.874441421823
H     -1.804769193203   -1.936497522055    1.213216791920

Ambimodal TS Water5-197
35
ANGSTROM
O     -3.325429107817    1.469213918343    3.862665598691
H     -5.810023146010   -0.334120764702    3.329594345939
H     -2.492818433678    1.182232648972    4.240972533652
O     -3.296262287712   -2.448448978330    1.912901998352
H     -3.914722659507   -1.934199927040    2.451353673773
H     -3.604245194533   -2.313815333386    1.009325462225
O     -4.982961101066   -0.376393635881    2.848807657037
H     -4.785621262449   -0.355156054918    0.976903320716
H     -4.392106142395    0.296537610574    3.253342697738
O     -4.471419925585   -0.446261129008    0.064032431528
H     -4.134271306101    0.415866339005   -0.194096486167
H     -2.527575585422    2.673530637046    1.013413573666
O     -2.640427816330    3.134156687848    1.874285798389
H     -3.158117675758    3.918019963184    1.686758308436
H     -3.100119661117    2.141311226337    3.183571696060
C      0.632905477159    1.937221278047    0.221736552996
C      1.533915162950    1.418730973915   -0.660220699192
C      2.012873608653    0.058929804526   -0.672290505848
C      1.577739070964   -0.928055213212    0.193092114887
C     -0.485542588116   -1.302093094141   -0.433247429581
C     -1.227967665961   -0.229759499466    0.058743889887
C     -1.518055095878    0.960139707971   -0.753390328999
O     -2.349176729816    1.833750741263   -0.446866099200
H      1.845992370996    2.039172940108   -1.502568931566
H      0.269651486936    2.951773983504    0.124297842294
H      2.599192491277   -0.237562914650   -1.541890461878
H      1.960333564419   -1.937166807423    0.092299402637
H      1.228672825211   -0.688070985069    1.190017695450
H     -0.284546267579   -1.368624852969   -1.493102131509
H     -0.525775200741   -2.253122474167    0.083233041043
H      0.379759236651    1.456855726678    1.156164509645
```

```
H     -1.103153456077    0.965029721626   -1.781787097777
H     -2.302227509325    0.532894191936    1.611755449019
N     -1.655491411856   -0.207103704452    1.378599203036
H     -1.892562865786   -1.095584112265    1.811621553897

Ambimodal TS Water5-198
35
ANGSTROM
O     -1.754441460723    3.695737006689    3.644617536007
H     -4.224281473949    2.258115616368    2.435330466086
H     -1.151110280593    2.966773762858    3.793637929431
O     -4.165365253577    0.578465471635    1.562057323739
H     -5.074577915843    0.486342930670    1.272731497379
H     -3.673700453644    0.973839113779    0.804308152147
O     -4.240385107308    3.221864657849    2.536362515474
H     -3.751837687505    3.833800883748    0.981543063490
H     -3.464199769004    3.444234597427    3.081074571545
O     -3.254895249610    4.029180649284    0.154651181857
H     -3.065296139269    3.167204069397   -0.241914462809
H     -1.842914105664    4.573026678397    0.798338318828
O     -1.023124612882    4.827250880397    1.303028570853
H     -1.024681803437    5.784723764848    1.329352386103
H     -1.427983457366    4.159363721185    2.842044788025
C      0.074319041532    1.583909207603    0.909564427047
C      1.218493192867    1.517038467840    0.166657860754
C      1.965308809185    0.323201896542   -0.129154569578
C      1.588967849332   -0.948112786175    0.271291565855
C     -0.191877897353   -1.358716937184   -0.914704805182
C     -1.232441461452   -0.635512683365   -0.323582963946
C     -1.582450752847    0.717276779787   -0.784377475073
O     -2.605416699784    1.349391038791   -0.465759520924
H      1.543319213338    2.423128027034   -0.346503326911
H     -0.449787298782    2.522884125519    1.024155228982
H      2.753737685312    0.415572475265   -0.875717513687
H      2.186661495659   -1.803015064072   -0.024250418138
H      1.027973922529   -1.101737897408    1.185890209036
H      0.208284990642   -1.032137626565   -1.863496774463
H     -0.121176659214   -2.426140970284   -0.743165656317
H     -0.241358226882    0.794812171261    1.579212760584
H     -0.973904244180    1.101355100463   -1.627017809373
H     -2.689454624192   -0.644276909499    1.174652282389
N     -1.908572984063   -1.141437957518    0.767474260200
H     -1.785210106173   -2.098232696814    1.046425489053

Ambimodal TS Water5-199
35
ANGSTROM
O     -3.819179017723    0.274437722411    2.517512398336
H     -3.640736978118    1.232211364896    2.351482917629
H     -4.086540970926    0.204420404703    3.433808813989
O     -3.439612254357    2.801402927278    1.731123647835
H     -3.050480999151    2.480111030507    0.898813702278
H     -4.358022641518    3.052740930271    1.495400598341
O     -5.942401423195    3.002492465723    0.748419098522
H     -6.113802349942    3.507925883515   -0.047052946320
H     -5.830235857147    2.066547945718    0.459666489415
```

```
O     -5.145803385990    0.531906832785    0.060452775240
H     -4.929969873404    0.187923566003    0.939936784032
H     -4.289506764181    0.809342246663   -0.306270798713
O     -1.272543345816    4.434835095999    2.334306740587
H     -2.070548444988    3.896004610707    2.201669258905
H     -1.554541362900    5.216799248957    2.810215060825
C      0.111905333132    1.512915044772    1.010644668290
C      1.256048809174    1.451336842148    0.268506704959
C      1.959961118771    0.250325884003   -0.099761312595
C      1.539170945817   -1.028146509568    0.226312380250
C     -0.247072264458   -1.323872045612   -0.984707988995
C     -1.265615041198   -0.574747804540   -0.386284692609
C     -1.547586360219    0.808106572408   -0.797863302134
O     -2.561399295429    1.453337315519   -0.481565180369
H      1.620526609375    2.374639501073   -0.184140063403
H     -0.376534597891    2.458325852735    1.203623775106
H      2.751651995185    0.358160357239   -0.840837938514
H      2.108677687728   -1.885002125552   -0.115598913160
H      0.973701426779   -1.213061745641    1.132268244692
H      0.179839186910   -0.985404331550   -1.917743384034
H     -0.230122457628   -2.398881543016   -0.849573290155
H     -0.235188930617    0.690427887153    1.621673520419
H     -0.889501563911    1.215255188636   -1.592444195958
H     -2.658420425163   -0.523466659043    1.174304166593
N     -2.034158879321   -1.100430846826    0.627070126775
H     -1.870269536857   -2.030445452171    0.968245788477

Ambimodal TS Water5-200
35
ANGSTROM
O     -3.686112817726   -0.205064030709    2.721473126934
H     -3.612708686558    0.767577530366    2.642301537726
H     -4.622347397809   -0.394239902665    2.796371430847
O     -3.505882483375    2.370822755237    1.940519590437
H     -3.208480233780    1.981170216231    1.099841094104
H     -4.254080231299    2.952316364877    1.701625260632
O     -5.075100051944    4.321647640668    0.877697986443
H     -4.779068161086    5.115342095989    1.328808443087
H     -4.526156054519    4.252395726093    0.073390319445
O     -3.277404630126    3.887330253106   -1.162750172712
H     -3.018289601974    2.960367578658   -0.959180262177
H     -3.556960943980    3.899087358816   -2.078708199350
O     -1.793290435682    4.535265105185    1.294349278705
H     -2.297263430591    3.846445008519    1.755137889954
H     -2.133698765930    4.550693920965    0.393548947042
C      0.057703639937    1.570072622924    0.932802686123
C      1.209557805554    1.501517889476    0.204014242064
C      1.953367047566    0.303081183341   -0.096618745204
C      1.574176297859   -0.966027010309    0.289945052964
C     -0.221977571212   -1.353557339202   -0.970448370875
C     -1.260679008147   -0.642490209454   -0.364866961051
C     -1.576002432379    0.729716536039   -0.784346112984
O     -2.593686223372    1.372483711320   -0.457905452499
H      1.551723324151    2.409369890700   -0.295870953728
H     -0.455679711971    2.513487713526    1.067356057887
H      2.753080762666    0.401499476544   -0.830847622414
```

```
H      2.164558155175   -1.824222032102   -0.009135299255
H      0.979118703506   -1.128741766296    1.180380086004
H      0.208763615269   -0.996050662333   -1.894005773418
H     -0.148110468044   -2.423643914795   -0.822963699095
H     -0.275047418449    0.776898076474    1.588955457448
H     -0.955433304978    1.123771962632   -1.616123443100
H     -2.643199942407   -0.660138758338    1.223584254642
N     -2.007508859023   -1.198944355137    0.642922751131
H     -1.772768979443   -2.108702081121    0.999695355386

Ambimodal TS Water5-201
35
ANGSTROM
O     -4.284437049365   -2.490941637804    1.778813567482
H     -4.422537124319   -1.532974758529    1.699929340769
H     -5.158590587078   -2.875290288152    1.854278875721
O     -4.376731845298    0.301357965594    1.485440508120
H     -4.006272410863    0.728971370194    0.695188369535
H     -4.305041195589    0.966417397027    2.190889354007
O     -5.505470433490    4.129940299175    1.507672473640
H     -5.994405159957    4.813899008968    1.966803889709
H     -5.295482913975    3.450710447653    2.162050772842
O     -3.832741982159    2.428306276668    3.166840108325
H     -3.255585400327    2.350160620949    3.926627533788
H     -3.308910388003    2.883093379992    2.458485114302
O     -2.773560022314    3.640083804585    1.049832518180
H     -2.708815791917    2.903009374765    0.418335397115
H     -3.629268469849    4.057121913024    0.872302048459
C      0.172000261624    1.628700132079    0.876426682970
C      1.269198502863    1.545867408269    0.070141443274
C      2.001980099209    0.343134479945   -0.232727322112
C      1.654245189726   -0.916090389303    0.227095963565
C     -0.181818808097   -1.395335065874   -0.837179385473
C     -1.192108327170   -0.644076825434   -0.228392301722
C     -1.573468933328    0.684475391567   -0.735878226886
O     -2.583217343228    1.318742763541   -0.387392795974
H      1.565406814988    2.436089189828   -0.487844601829
H     -0.364310385529    2.560068174850    1.001593955435
H      2.744972847824    0.415910582922   -1.026657596319
H      2.241579538801   -1.776300900566   -0.073820690794
H      1.144803991889   -1.043096974738    1.174987090506
H      0.165453937507   -1.116930484369   -1.822115999811
H     -0.100129960815   -2.452277958449   -0.614263225311
H     -0.105722526717    0.848444206745    1.571288988208
H     -1.001511965730    1.045497407554   -1.616331073596
H     -2.614748452400   -0.560012228726    1.257001435894
N     -1.791378304630   -1.060363992519    0.941210665608
H     -1.798640277056   -2.035682621482    1.190936083367

Ambimodal TS Water5-202
35
ANGSTROM
O     -4.395929251787   -0.349038779584    2.217454729602
H     -3.434400138973    1.216745970034    2.619690192029
H     -5.079688309991   -0.706058875659    2.783624886954
O     -2.975932709053    2.032883033960    2.366124463536
```

```
H     -2.729179394390    1.895802246565    1.441065537818
H     -4.395030584893    3.146360285685    1.942346309062
O     -5.101767289081    3.455335751576    1.345766154241
H     -4.632103788130    3.891485830304    0.615692851337
H     -5.468186889705    1.937554829979    0.625849682963
O     -5.359866709407    1.049379771041    0.205348100348
H     -4.866803855689    0.106169280125    1.467744066425
H     -4.503018416916    1.096893554403   -0.238045915932
O     -3.551708297760    3.895210541357   -0.932047000397
H     -3.126430593913    3.031030720576   -0.793059236857
H     -4.127267524476    3.796186662723   -1.692317064722
C      0.128549761903    1.687868025485    0.768150182736
C      1.241764801677    1.562570231347   -0.011579008678
C      1.982706348271    0.345826119997   -0.230452509706
C      1.634631064898   -0.882688989804    0.296185962361
C     -0.207001003911   -1.398283667062   -0.818081024125
C     -1.226944161150   -0.647175674457   -0.231627671541
C     -1.590022180750    0.678279170226   -0.751355170174
O     -2.590351162919    1.335566261542   -0.400439730083
H      1.544829753278    2.418368491206   -0.617518375464
H     -0.403010776899    2.628232351188    0.836827426461
H      2.744252523869    0.376846832513   -1.009636838532
H      2.221147133837   -1.760926893858    0.052422837492
H      1.089134386833   -0.960728729559    1.228817541000
H      0.170154824929   -1.121162773767   -1.791250426706
H     -0.109258153125   -2.449857182591   -0.579168670826
H     -0.153799318870    0.959213876535    1.515706869669
H     -1.041079633360    1.004983057699   -1.657712995590
H     -2.688146609655   -0.616370332554    1.263902221918
N     -1.857716222321   -1.077021964891    0.915119849138
H     -1.734937644361   -2.023765851594    1.229596331816

Ambimodal TS Water5-203
35
ANGSTROM
O     -5.599867031144    3.851221097548    1.307162716848
H     -5.121240108845    3.338235390501    1.980698180089
H     -6.175541762518    4.455946121461    1.775581212150
O     -2.085449221132   -0.086295898730    3.040173234134
H     -2.020941211051   -0.192799608550    3.989655779747
H     -2.669560664619    0.686851535151    2.900537321771
O     -3.694278611631    2.055162568155    2.421921270835
H     -3.126091191097    2.690033190887    1.944120897306
H     -4.179165180464    1.584208213149    1.710006820883
O     -4.906471439056    1.231382647295    0.159320728927
H     -5.473251268142    2.006811694975    0.095680017924
H     -4.157747502103    1.385840317430   -0.453263829291
O     -2.828492288316    3.843777602698    0.521520125699
H     -2.719920292541    3.222951145944   -0.215720223947
H     -3.738638919690    4.161735292606    0.473360380183
C     -0.244988157609    1.735937046096    0.828365733864
C      1.028142298443    1.476186606548    0.415356390479
C      1.658471100384    0.179612528757    0.433947532785
C      1.017953646218   -0.980561705843    0.824022554100
C     -0.420038298923   -1.311810313765   -0.817344146841
C     -1.496987148857   -0.455135418446   -0.581947769812
```

```
C      -1.566151516572    0.882928776585   -1.186380567067
O      -2.554059778751    1.641518563049   -1.151082305156
H       1.593888776221    2.275515672173   -0.067739151735
H      -0.669509755157    2.726915853852    0.741004462619
H       2.621728489647    0.098710518093   -0.070513711837
H       1.542150624245   -1.928475099051    0.777553220700
H       0.204608707788   -0.958078156686    1.539380432713
H       0.267877299801   -1.098678253894   -1.622607586705
H      -0.509862649351   -2.360115476003   -0.563748861312
H      -0.803537531165    1.048042104841    1.447773052590
H      -0.728952254335    1.149044609741   -1.863261306774
H      -3.238224191696   -0.166517578030    0.499069573536
N      -2.544771602569   -0.842401896169    0.225452240812
H      -2.435285927525   -1.612444426693    0.862014622528

Ambimodal TS Water5-204
35
ANGSTROM
O      -1.638957521749    0.284397709209    3.633066922256
H      -2.189314894577    0.945666636707    3.140460927864
H      -1.306235988545    0.739591968279    4.407251696503
O      -3.247360811032    1.900751848032    2.287910945400
H      -2.869638291556    2.053101244532    1.408947481420
H      -4.051537390317    1.384063757403    2.088562280490
O      -3.658617248649   -1.466192757400    3.964779856424
H      -4.349156251862   -0.829942284573    4.164395868328
H      -2.833511945660   -0.941014605613    3.868856743812
O      -4.347892030443   -2.430651386692    1.558559512047
H      -5.070540669536   -3.049764232877    1.668179467889
H      -4.055214981203   -2.185941883667    2.466301875357
O      -4.945514450678    0.205498144386    1.026897773915
H      -4.464344173692    0.510284700862    0.247969534016
H      -4.801862646733   -0.757287747646    1.087294983919
C       0.209073367425    1.626079929907    0.861036365575
C       1.319264391326    1.521229806592    0.074339611027
C       2.031365769292    0.304409328797   -0.212862533483
C       1.634836403780   -0.947301659839    0.234207742025
C      -0.172919297029   -1.367823696351   -0.870973223179
C      -1.184623938968   -0.608827584528   -0.274543780721
C      -1.560593147558    0.735022002016   -0.754225197427
O      -2.561989784674    1.353359957506   -0.368347608731
H       1.643809631354    2.404402485808   -0.479748537420
H      -0.307081633430    2.570919099442    0.973286935476
H       2.793218009328    0.358866475182   -0.990034928865
H       2.208974381948   -1.821533884387   -0.052303152084
H       1.107598092886   -1.058843713711    1.174546340287
H       0.194834777216   -1.081213329671   -1.846131959825
H      -0.120871602253   -2.430576326105   -0.667409616845
H      -0.093104975260    0.859087109612    1.560155380213
H      -0.997182103498    1.113463537054   -1.633116697729
H      -2.582633367193   -0.474772164783    1.228683254091
N      -1.844748328150   -1.042460452250    0.847248513537
H      -1.796395401547   -1.989428636156    1.174789827079

Ambimodal TS Water5-205
35
```

```
ANGSTROM
O     -3.816564720327    0.780968586283    1.885376352639
H     -3.572016776513    1.087551411805    0.989914345339
H     -3.328482696324    1.369581038458    2.486117501410
O     -3.410404544701    3.815841111030    0.752938997344
H     -3.143100520157    3.146923616004    0.107930505019
H     -2.922226271794    3.608209335305    1.561878894822
O     -2.505263970570    2.815908759755    3.281253509631
H     -3.324531309558    3.208289967052    3.678558393890
H     -1.864797154065    2.757084889638    3.990298473630
O     -5.725985754298    2.843056345500    1.869335599296
H     -5.389387384658    1.933501639451    1.906854035140
H     -5.073341531885    3.324232397605    1.322371433285
O     -4.794768151028    3.848801109779    4.128466008813
H     -4.890331049104    4.798293798625    4.038906660933
H     -5.300131464181    3.458238649323    3.367698254447
C      0.145478953475    1.601458379261    0.917217412018
C      1.258093675536    1.522483219232    0.131098579640
C      1.977518813791    0.317025190147   -0.186426383357
C      1.598170568776   -0.947341069296    0.236541423562
C     -0.222506614631   -1.368334492838   -0.863068054628
C     -1.235192478660   -0.610931734543   -0.261507944567
C     -1.569225226423    0.736192493250   -0.759679281646
O     -2.558228226113    1.409612259285   -0.436113373610
H      1.576243092118    2.421052182969   -0.400416135681
H     -0.379776317923    2.537106633467    1.054701810246
H      2.734453541092    0.394703626682   -0.966085290501
H      2.176681998513   -1.810106105255   -0.073997108084
H      1.078463522973   -1.085775904506    1.177618726811
H      0.146419543945   -1.079233339365   -1.836623308726
H     -0.166472585514   -2.431964041852   -0.663225947338
H     -0.155396781677    0.814907861958    1.594738433964
H     -0.961327110952    1.077032897126   -1.624117514124
H     -2.644388824968   -0.537725149835    1.283633435834
N     -1.885954060545   -1.067977008923    0.861086458569
H     -1.781718272881   -2.022475216983    1.156406891796

Ambimodal TS Water5-206
35
ANGSTROM
O     -3.717287961414   -0.480701439760    3.086286140319
H     -3.890446851903    1.216956584878    2.336415563139
H     -2.874131059688   -0.324937404362    3.523305912116
O     -3.484900096483    2.076844114668    2.126099707446
H     -3.203407517812    1.998693923220    1.198236850766
H     -2.261229816545    1.865717073190    3.209429644436
O     -1.572544028533    1.619321395540    3.885531083363
H     -2.497148790500    1.581754493916    5.334280108764
H     -0.939522020071    2.338122988342    3.883975970909
O     -5.263547078148    0.300234630098    5.155835194662
H     -4.390874188139   -0.362409916159    3.788177733056
H     -5.891792985020    0.984390583069    4.919255321176
O     -3.048658639356    1.508146988085    6.144930407874
H     -2.535862012691    1.005574654961    6.779141741324
H     -4.525432846432    0.759689966089    5.609729398963
C      0.215540841844    1.615090983625    0.859903435448
```

```
C      1.321215415362    1.506952848648    0.065881325496
C      2.024684949834    0.289533658212   -0.226127424117
C      1.611421896633   -0.964817620667    0.213778162432
C     -0.179764998349   -1.359692509590   -0.850855740277
C     -1.199638060913   -0.601777191193   -0.257569607012
C     -1.575710073509    0.740081264198   -0.745074163833
O     -2.553235295538    1.385911903630   -0.359778428691
H      1.643378930808    2.389763986956   -0.489543977102
H     -0.307008399125    2.556981413921    0.964090738025
H      2.780772583635    0.337943102659   -1.008744815912
H      2.183085016763   -1.839538061874   -0.077736184029
H      1.111346653109   -1.074070705463    1.169751391077
H      0.174245665056   -1.076735244574   -1.832543638612
H     -0.140131658389   -2.425765681239   -0.657223753509
H     -0.083663606997    0.851171876094    1.563189672209
H     -1.010390393409    1.092498642906   -1.636486224334
H     -2.647114075758   -0.547250856173    1.227135320867
N     -1.846401326515   -1.049029818556    0.873502416874
H     -1.769080892146   -2.005421687225    1.171877257186

Ambimodal TS Water5-207
35
ANGSTROM
O     -3.661513773397    4.013137596311    0.380368366677
H     -3.286930182726    3.313627613625   -0.170055185550
H     -3.084365660399    4.070521474057    1.151285477963
O     -4.762165702064    4.610260124646    3.592806957911
H     -4.827850534278    5.412574182147    3.071686256252
H     -3.866067885184    4.257650017327    3.433515914261
O     -2.352238589949    3.427745279816    2.940880382480
H     -2.733909576695    2.546288604332    2.680069208424
H     -1.795109715457    3.266413962075    3.702521582046
O     -5.677318297598    2.707626882586    1.800549483635
H     -5.217463407789    3.215547130013    1.108163571539
H     -5.630920706658    3.278695707610    2.586468070719
O     -3.481246217169    1.226446617460    2.010385983081
H     -3.190118258280    1.405830872224    1.091516831110
H     -4.405987099630    1.571514619463    2.031647635804
C      0.278761807214    1.553025324617    0.906213377164
C      1.387713534395    1.404652585250    0.124689185686
C      2.025212534742    0.155430891909   -0.198138301812
C      1.558491065526   -1.083053853703    0.213641857652
C     -0.282539781174   -1.367614539184   -0.898994668009
C     -1.244294976112   -0.546188626054   -0.298586440629
C     -1.479854033554    0.826212090396   -0.783011758080
O     -2.413974271254    1.568449636681   -0.446595980389
H      1.767752928218    2.283111744132   -0.399491042131
H     -0.184547280275    2.520674248302    1.046462842037
H      2.789060533574    0.186675382230   -0.974160100990
H      2.077331333691   -1.981207601000   -0.101636812939
H      1.027410366057   -1.193010831005    1.152190681196
H      0.112330603944   -1.098247700194   -1.867968787504
H     -0.302274952998   -2.433803044312   -0.706491422130
H     -0.075771172822    0.784133943789    1.578516878789
H     -0.853389277756    1.128868805218   -1.647914002598
H     -2.601485769226   -0.375706245358    1.285849476566
```

```
N     -1.959164862849   -0.981051039000    0.792688504471
H     -1.858301102248   -1.919031614771    1.136222241765

Ambimodal TS Water5-208
35
ANGSTROM
O     -1.349103796263   -3.641314729181    2.083296687859
H     -0.379593236872   -3.760313916422    1.930514396047
H     -1.693011335499   -4.494617115249    2.349907772779
O      1.297324776890   -3.886275028119    1.884225341499
H      1.535450019948   -3.188514972000    2.547149258938
H      1.711145848614   -4.690414945598    2.200676507263
O     -0.975613075150   -1.601687074197    3.911738383547
H     -1.258253270989   -0.812619330257    3.444495089751
H     -1.213934224602   -2.352164024107    3.337429827393
O      2.745593446371    0.455749817971    3.150194956512
H      2.274040201197    0.894812305795    2.435750887431
H      3.683087394959    0.566845180207    2.983466602983
O      1.718122672714   -2.088740804058    3.776264114937
H      0.795002507798   -1.888832644049    4.023288024699
H      2.141031221386   -1.229415361109    3.631400659092
C      0.264589100515    1.615235569004    0.901940315807
C      1.347897790671    1.517198441614    0.076379605427
C      2.047250557011    0.301126612283   -0.243419274787
C      1.636047222922   -0.959172347790    0.195616418863
C     -0.146587341672   -1.365129568950   -0.796377979749
C     -1.169600349881   -0.587184626006   -0.236237254753
C     -1.590370106512    0.731151694796   -0.761911164071
O     -2.584053935974    1.335952913420   -0.374386449998
H      1.655713147628    2.407418855465   -0.475125962012
H     -0.267779278371    2.546914728166    1.030042365587
H      2.777911753761    0.357504176301   -1.048986287090
H      2.202803628534   -1.831169786080   -0.110325553939
H      1.172998018509   -1.078699258970    1.167949494446
H      0.190176032785   -1.125531591744   -1.796461645195
H     -0.110629588423   -2.421033792505   -0.552943501512
H     -0.031828620040    0.829810465970    1.582068605106
H     -1.016966500026    1.102110128515   -1.643584257263
H     -2.656990868873   -0.458300225524    1.141860554246
N     -1.827325042385   -0.987661764038    0.915124371881
H     -1.818825034699   -1.960217216274    1.189155264790

Ambimodal TS Water5-209
35
ANGSTROM
O     -3.431682198355   -0.245468659633    3.458994435226
H     -2.474333012274   -0.382814546256    3.595364473139
H     -3.738807786228    0.248978062305    4.228159056229
O     -0.710713234430   -0.192968704881    3.777779907462
H     -0.794731658677    0.792641291529    3.799858159805
H     -0.225584922421   -0.436018413057    4.566926098617
O     -3.346271817968    2.117875065676    2.103120695528
H     -3.165258605917    2.033738233394    1.150773196824
H     -3.491337835517    1.201469847791    2.415776396406
O     -4.062662230729    2.283745441600    4.829439569696
H     -4.860297854253    2.693239203841    5.165652310363
```

```
H     -4.023478853404    2.490061069660    3.882901639041
O     -1.306518318837    2.401161576205    3.875845135158
H     -1.905205303135    2.442007617936    3.100431651101
H     -1.890411255315    2.514910319456    4.633307734500
C      0.172422928700    1.683402907509    0.799925603046
C      1.291627255864    1.564369364045    0.027527757249
C      2.022566281569    0.345666083122   -0.188276956843
C      1.627413160605   -0.885453127488    0.318993361363
C     -0.161268796292   -1.357081922939   -0.771443239369
C     -1.200606694689   -0.591243612071   -0.230488005169
C     -1.587966707520    0.726637287155   -0.772931218730
O     -2.573459319323    1.378652341489   -0.412739141802
H      1.605344405504    2.423613269617   -0.568231344423
H     -0.366225607277    2.618698460638    0.867694293188
H      2.790533728844    0.364803476157   -0.961079904406
H      2.210721007688   -1.769151585139    0.083484208026
H      1.102690670257   -0.949553104612    1.265312779019
H      0.215546248091   -1.109709358236   -1.753834573419
H     -0.102018636921   -2.409915239742   -0.522799334133
H     -0.107954686389    0.943996374432    1.535536697216
H     -1.026122051875    1.051170063668   -1.676424452682
H     -2.606800601000   -0.436787980773    1.280369133100
N     -1.918002127336   -1.034262833908    0.855736832282
H     -1.643269325700   -1.842977942147    1.382785475497

Ambimodal TS Water5-210
35
ANGSTROM
O     -2.984735969438    1.088657196677    2.455974458549
H     -3.897784322283    1.410075526482    2.302972539579
H     -2.444852787152    1.895986452391    2.465054045634
O     -2.019421074932    3.714794301042    2.346591538263
H     -2.556610339306    3.891553690104    1.554918094138
H     -2.481368584675    4.123489761482    3.080690356208
O     -4.939110276228    0.340583932406   -0.307418608658
H     -4.092083079486    0.767871618832   -0.529760292316
H     -4.706422246050   -0.478179823735    0.136014992104
O     -3.581017856733    3.852951806898    0.070869769262
H     -3.695937686747    4.444876464238   -0.673541089437
H     -3.197052058662    3.030218361596   -0.288203556647
O     -5.320916102863    2.224435814599    1.641127827391
H     -5.464062654186    1.552480981163    0.949571253365
H     -4.912755393006    2.969445932689    1.179851314627
C      0.146723998024    1.617333901556    0.757358242991
C      1.291335721257    1.403112980873    0.045967920201
C      1.929319886600    0.125780622230   -0.148139821534
C      1.437467789451   -1.069874347141    0.344594787740
C     -0.367680529781   -1.431064474845   -0.841886091083
C     -1.332338896550   -0.528848168890   -0.382109517763
C     -1.502811870377    0.790539599420   -1.005153694268
O     -2.484411744369    1.535072804143   -0.837132516080
H      1.708993243331    2.234673551161   -0.524764849248
H     -0.309982818967    2.595549435397    0.819090897526
H      2.729091984110    0.089434481068   -0.887507074407
H      1.956111374409   -1.996368632211    0.126287016153
H      0.855342573208   -1.098544274476    1.258187249567
```

```
H      0.092038917615   -1.274059853480   -1.807569551132
H     -0.424782550639   -2.469859353551   -0.537943603377
H     -0.243375057941    0.902147937544    1.467693930362
H     -0.785884515680    1.040013901436   -1.814773209087
H     -2.558913918291   -0.079205262313    1.243230639109
N     -2.189556375658   -0.842189703901    0.656350572392
H     -2.015245967500   -1.691782675980    1.169986531642

Ambimodal TS Water5-211
35
ANGSTROM
O     -4.735140740290    3.192179031373   -0.628873764040
H     -5.193442518376    2.743141709128    0.098860299559
H     -3.871191312647    2.763716518274   -0.697789778907
O     -3.370460766926    0.931949533096    2.000270705658
H     -3.052990344124    1.441814581220    1.242409967211
H     -4.335404130417    1.015359535000    1.960671733061
O     -5.965943311159    1.298860281650    1.092476641677
H     -6.858236552752    1.057273729836    1.339370510733
H     -5.703232119983    0.698456996090    0.344412721380
O     -5.028879840516   -0.038721521983   -0.977487700378
H     -4.122048086377    0.288599106646   -0.883357119326
H     -5.401896032984    0.470705636810   -1.721874952090
O     -5.911772879992    1.904437708845   -2.678393106511
H     -6.791108649319    2.247357004889   -2.839473848774
H     -5.488156047586    2.518555515052   -2.035680861286
C      0.040114185320    1.618411383880    0.867764177336
C      1.226924615256    1.524297016812    0.201514436160
C      1.980756478662    0.314798260995   -0.014024064782
C      1.573404820061   -0.939399862975    0.397942939971
C     -0.146419446257   -1.386301802871   -0.918764154704
C     -1.210864867021   -0.639595171709   -0.408642019363
C     -1.508453767058    0.713406275693   -0.911965244802
O     -2.547073462226    1.348733276489   -0.654316135120
H      1.594829289799    2.410595194667   -0.318721981153
H     -0.492767433851    2.558311816086    0.929304470514
H      2.814508912893    0.383453145511   -0.712187211847
H      2.177672390719   -1.807757311105    0.162717639883
H      0.942426047687   -1.065744283859    1.269593428333
H      0.326170453656   -1.082694928266   -1.841522249214
H     -0.092000046200   -2.448502704879   -0.713848345262
H     -0.327610815092    0.852780769910    1.536604068259
H     -0.845175232038    1.089419749347   -1.716064506703
H     -2.632650569031   -0.501836401653    1.114759257082
N     -2.020538334664   -1.125127500549    0.588595048740
H     -1.830774623656   -2.020039670949    1.004187161046

Ambimodal TS Water5-212
35
ANGSTROM
O     -3.904759289206    0.186184788450    2.448343701712
H     -3.675462851760    1.147121322524    2.294363157221
H     -4.359135147591    0.159229638052    3.290704881872
O     -3.315171638015    2.669174830660    1.868987478851
H     -2.993785936817    2.461230206679    0.978518985510
H     -4.196608017948    3.077349825338    1.732302704005
```

```
O     -5.756339174200    3.532009885894    1.116714113865
H     -5.923587585596    4.401678682497    0.752613907875
H     -5.733531374329    2.912514855561    0.346437210270
O     -5.434164866115   -0.604788240261    0.323429319244
H     -4.972012648476   -0.322871482831    1.138291740404
H     -4.811583493538   -1.154800170463   -0.155845911579
O     -5.348160194488    1.838346426473   -0.900607837084
H     -4.381909532096    1.844095000969   -0.935642623081
H     -5.580422722986    0.964943122035   -0.529845149331
C      0.034296669533    1.419531023704    1.079165568158
C      1.207913983805    1.389641417659    0.382553000729
C      1.909864606476    0.200181069559   -0.018300249256
C      1.444956908560   -1.087134062288    0.214344174132
C     -0.276811667248   -1.279570815990   -1.056601689663
C     -1.310910728669   -0.525712107156   -0.487082215354
C     -1.548870688325    0.876495462598   -0.864183916516
O     -2.541050718005    1.545702664081   -0.549766660346
H      1.597791829282    2.330219215950   -0.009714279650
H     -0.478030151208    2.348815151108    1.287998041290
H      2.732089786760    0.332012174195   -0.720509150276
H      2.016077807834   -1.933618620460   -0.150507601553
H      0.855149516387   -1.310397913715    1.096387785881
H      0.194064013623   -0.924990208503   -1.962818106589
H     -0.299723684384   -2.359231934473   -0.961189949528
H     -0.341093973789    0.571749141629    1.635243633137
H     -0.844415262361    1.287899489959   -1.619599350454
H     -2.724510649085   -0.476735767790    1.036983221012
N     -2.169809037329   -1.081113409406    0.443658771255
H     -1.961217056687   -1.984589818173    0.834696165954

Ambimodal TS Water5-213
35
ANGSTROM
O     -3.758167743727    0.222713134010    2.578657859833
H     -3.483606649424    1.163291079053    2.393129990173
H     -4.065759639868    0.210582102197    3.484818649873
O     -3.150383438276    2.670199045450    1.847293017193
H     -2.994527473338    2.428861840049    0.921983688189
H     -4.003141339322    3.153830391661    1.859694911412
O     -5.572761219110    3.875782641858    1.689611170033
H     -5.572294537254    4.702232193562    1.205029326078
H     -6.106728696379    3.248900839416    1.151243141911
O     -5.065929524403    0.216643845033    0.147626725957
H     -4.282775058802    0.613308037944   -0.268284746053
H     -4.802529924328    0.089026091248    1.074876416389
O     -6.921959597514    2.123544427642    0.170752025993
H     -6.254355265659    1.400564438737    0.075450268391
H     -7.713248712826    1.707844549738    0.515114196946
C      0.031590659514    1.487871382285    1.022128793035
C      1.178192818380    1.485151947175    0.282600145005
C      1.939142886785    0.317357566268   -0.077801488144
C      1.571080161230   -0.978125012808    0.250712682042
C     -0.194294839490   -1.368341776238   -0.944744621365
C     -1.247179945286   -0.645057317049   -0.370317969012
C     -1.577783289005    0.716164162247   -0.824996756891
O     -2.607894119870    1.341517580554   -0.537556454260
```

```
H      1.497381697291    2.422370896957   -0.176649273107
H     -0.526956219107    2.396125847761    1.201625710956
H      2.724776537308    0.458119726982   -0.819352477116
H      2.180337370386   -1.808977263114   -0.086983324077
H      1.020452040997   -1.183574408016    1.161449995173
H      0.218609490026   -1.038997964898   -1.887546633754
H     -0.139908980862   -2.438858042902   -0.784667697856
H     -0.275328978614    0.650445633599    1.632720329454
H     -0.914619985054    1.122987810024   -1.617840511834
H     -2.647662391549   -0.608687364189    1.181650985871
N     -1.997832781811   -1.173197982290    0.652153066881
H     -1.808575439288   -2.091971048096    1.010501861041

Ambimodal TS Water5-214
35
ANGSTROM
O     -2.881301515451    1.262948261282    3.466122883321
H     -2.630732511049    2.066358447078    2.974425121856
H     -3.603332730047    0.880317574737    2.947877565026
O     -2.600614712789    3.320736908710    1.637014724609
H     -2.588297070933    2.740040089221    0.856756476054
H     -3.441178193259    3.781398587404    1.613191276206
O     -4.499015558559    0.485291755226    1.348011410648
H     -3.879605566464    0.850966541734    0.681274884271
H     -5.152269324913   -0.023065377362    0.867335890717
O     -0.795728969414   -0.372638800838    3.744392639618
H     -0.368787403207   -0.094199776254    4.554809433666
H     -1.588426154275    0.208237162934    3.647015149590
O     -0.693989685996   -2.854210604005    2.603031632138
H     -1.042487191007   -3.467603953144    3.251073102297
H     -0.667910728213   -1.982530920204    3.041950573281
C      0.184336324446    1.430434833271    1.005691031447
C      1.290690057968    1.374681372359    0.213432835825
C      1.985651979161    0.167911800727   -0.171749601121
C      1.592457801207   -1.103668019748    0.182234290900
C     -0.284145210613   -1.376368878023   -1.014096032059
C     -1.273490180994   -0.647939279393   -0.356799767656
C     -1.575704838755    0.740170032548   -0.727659561274
O     -2.560210370250    1.397503167417   -0.330664248765
H      1.631533504443    2.292943143993   -0.269735046908
H     -0.313107842930    2.368913497735    1.211691737947
H      2.754374931685    0.280040163895   -0.937454409973
H      2.140048673993   -1.962475064251   -0.188139156473
H      1.022856172719   -1.300296963971    1.081415747308
H      0.128189590363   -1.013094688309   -1.944145298406
H     -0.228664722911   -2.446909339435   -0.870576160284
H     -0.123650207002    0.605344238383    1.632956293912
H     -0.969445439781    1.164673210384   -1.553227516788
H     -2.572663021720   -0.611165356723    1.243498902027
N     -2.018080409031   -1.216669352151    0.656997035062
H     -1.657245880054   -2.038462262171    1.126207353951

Ambimodal TS Water5-215
35
ANGSTROM
O     -1.950178096684    1.192192099526    3.721181643233
```

```
H     -2.721183804979    1.034043343759    3.140032964934
H     -1.584029796762    2.033660276661    3.441490824176
O     -3.927612204002    0.991882743048    1.879143492434
H     -3.457362301264    1.255263080920    1.046212908945
H     -4.761247072750    1.462646402536    1.881492093195
O     -1.459765177054   -3.827361717584    1.475313288873
H     -0.752285110971   -3.666765556575    2.135232032943
H     -2.121539770120   -4.356576192389    1.922179229359
O      0.131590005995   -0.526769891229    3.777499380682
H      0.464590307031   -0.365857264977    4.661910797572
H     -0.698731474403   -0.007507406618    3.713407391291
O      0.433866050896   -3.171507749659    3.287504228622
H      1.323241379503   -3.205006739885    2.931212128107
H      0.248439967209   -2.225134038837    3.462419463427
C      0.280509470453    1.640152675549    0.862155858927
C      1.371507814961    1.473434588578    0.064342247176
C      2.022496042049    0.214841186058   -0.209346479179
C      1.575093787190   -1.002786839721    0.262949086298
C     -0.291113401353   -1.344532758654   -0.877978505967
C     -1.261459334527   -0.571759469239   -0.236859805802
C     -1.572360941193    0.797551813931   -0.690583396124
O     -2.516037563898    1.502993684285   -0.299273851083
H      1.733493391693    2.330719654129   -0.507196082580
H     -0.194756210555    2.607259670224    0.966404431941
H      2.784376046469    0.224779635983   -0.989002389389
H      2.092625619705   -1.913233119623   -0.016904110120
H      1.014565404101   -1.072160362629    1.186600477665
H      0.096448092918   -1.037286368032   -1.838360387202
H     -0.261254311874   -2.409146510341   -0.685742642598
H     -0.047344724713    0.885068933157    1.561188008589
H     -1.005029449392    1.136365620375   -1.583160371430
H     -2.659056921864   -0.551160103249    1.295843345801
N     -1.888638654679   -1.051253683407    0.885871394516
H     -1.803546609850   -2.035851215533    1.116329771383

Ambimodal TS Water5-216
35
ANGSTROM
O     -3.364261184857    1.590080448565    2.215777240736
H     -3.149810398039    1.691278703997    1.267848927108
H     -3.980513751770    0.832976151185    2.282774550070
O     -3.923334760372    1.504799461153    4.972120814500
H     -3.776584368550    1.840160160121    4.074161379119
H     -4.505185671917    2.130284099607    5.404822983402
O     -4.581611509677   -0.670275169238    3.102484687616
H     -3.867290430715   -1.329306806718    3.157347036379
H     -4.606175247617   -0.242083076339    3.967221389350
O     -1.454041160828    0.458079741907    3.784607584725
H     -1.823832450389    0.725063229167    4.631782978485
H     -2.007045729879    0.927663925203    3.122597015576
O     -2.216495269065   -2.085547588177    3.471688242877
H     -2.043228496275   -2.558790724569    4.286186944781
H     -1.817192785431   -1.188646661775    3.578386034900
C      0.197594181535    1.607935574943    0.873852892660
C      1.317656125597    1.478556544820    0.104882714731
C      2.010414137140    0.245272693311   -0.154659346644
```

```
C      1.571547320024   -0.992449228302    0.294124019359
C     -0.222317084506   -1.362788592768   -0.844586528338
C     -1.242812087245   -0.587843299506   -0.283299499798
C     -1.571869099771    0.772009910042   -0.760011402298
O     -2.540774109296    1.436974789671   -0.373660753206
H      1.663851829246    2.348513886014   -0.456633412213
H     -0.311544213039    2.557675627757    0.969890396159
H      2.786896772453    0.272801477353   -0.918633614935
H      2.127808034262   -1.883901404948    0.025689890696
H      1.028344339270   -1.082299710221    1.227459297322
H      0.182544916964   -1.081196200609   -1.806083041252
H     -0.200971424247   -2.426878201049   -0.643096048453
H     -0.114827035036    0.854829518565    1.582790928571
H     -0.991980440500    1.124864311114   -1.639444907649
H     -2.708975475784   -0.451040999403    1.155590609585
N     -1.987348129488   -1.038997956170    0.776482571515
H     -1.828741253403   -1.914897657266    1.241551685974

Ambimodal TS Water5-217
35
ANGSTROM
O     -2.187548092246    3.567825188499    2.637568226092
H     -2.689021224658    2.743180618753    2.527004007552
H     -2.292324336334    4.038530866646    1.805263025466
O     -3.190150942540    4.007986388675   -0.099065394488
H     -2.951448320263    3.080226396975   -0.299820388109
H     -3.446219356776    4.406628291994   -0.931716604601
O     -3.763947233761    1.292143520128    2.005003347370
H     -4.520519766070    1.906353678675    2.015741989085
H     -3.450286643354    1.297117991214    1.079841688448
O     -5.198755431411    3.603528501233    1.788125916911
H     -4.916130758627    4.088902903337    2.566943072758
H     -4.574564759938    3.851136742812    1.081583986923
O     -1.455741843629    0.543210624535    3.455046198167
H     -1.163659489683    1.403476237004    3.767907380330
H     -2.368378739099    0.680712768563    3.164395223886
C      0.135622171888    1.689369480580    0.755731988015
C      1.260326309834    1.548969474706   -0.004237815419
C      1.991580489903    0.321720298858   -0.196993737480
C      1.618054565815   -0.893562616611    0.339394785732
C     -0.214869489864   -1.386974920986   -0.823638960591
C     -1.236362178003   -0.620930739782   -0.262938165518
C     -1.574952257215    0.709687887132   -0.787608762025
O     -2.572169665565    1.382524597841   -0.457242669971
H      1.584497104460    2.395260990189   -0.613693536531
H     -0.382971991017    2.637612878767    0.812791870655
H      2.768330130922    0.334766002688   -0.961904350291
H      2.194036974589   -1.784483603513    0.117823060093
H      1.050190394382   -0.950429217970    1.260077974593
H      0.189446544467   -1.121693916174   -1.789468084111
H     -0.133254934059   -2.436505747318   -0.571750442248
H     -0.160420255326    0.971854105472    1.509184667172
H     -1.000626807574    1.033632325886   -1.679529831707
H     -2.554731087980   -0.453039407241    1.328326561735
N     -1.968783690627   -1.085740885924    0.807988206152
H     -1.658500060075   -1.893646848033    1.319301397659
```

```

Ambimodal TS Water5-218
35
ANGSTROM
O     -0.531549633499    3.121857800285    3.738207320023
H     -0.736749716993    2.167070610885    3.672252202364
H     -0.337150306296    3.300759539020    4.658758157797
O     -2.643885578718    4.081951422503   -0.109169595881
H     -2.638082687324    3.126589316407   -0.308878684691
H     -3.433788513809    4.434972801582   -0.521331481047
O     -3.512693918372    1.359397525120    2.175462489499
H     -3.468705405283    2.310054426530    2.442605850354
H     -3.342430764898    1.379343635757    1.216280829394
O     -2.949692421719    3.917498741782    2.642398950342
H     -2.065335121742    3.800527016330    3.031992827749
H     -2.793281153237    4.149577993036    1.709887571281
O     -1.451261415389    0.540719591724    3.603645087494
H     -1.807419770234    0.269577240972    4.450899848870
H     -2.239283745700    0.820407528915    3.063533219492
C      0.098703950018    1.710409671574    0.761771810105
C      1.223202182876    1.583908715043   -0.003034877093
C      1.971830022015    0.369451250653   -0.194389039801
C      1.610035930184   -0.853990264363    0.340907172643
C     -0.198284166110   -1.375228841857   -0.788830562207
C     -1.235826092477   -0.617213099093   -0.242127245921
C     -1.599002630207    0.704671106805   -0.772825533742
O     -2.600583922870    1.362336213736   -0.431356044469
H      1.527466293929    2.433095754723   -0.617516820332
H     -0.436477378072    2.649292739810    0.809933098301
H      2.744313572891    0.388687087507   -0.963278883416
H      2.201907068171   -1.735338523150    0.121255377594
H      1.061073548074   -0.913616159273    1.273132293355
H      0.193140443724   -1.118653865068   -1.762391145957
H     -0.113276052598   -2.423950379309   -0.532347240000
H     -0.175303836964    0.993424147530    1.523089899742
H     -1.036744749615    1.031256723246   -1.671212539846
H     -2.593157401292   -0.457240736372    1.316122004034
N     -1.958232965226   -1.076086251774    0.838721935412
H     -1.664006054798   -1.891488555856    1.345771461788

Ambimodal TS Water5-219
35
ANGSTROM
O     -2.060139441496    3.741963932146    3.246388275106
H     -1.762841225607    2.818419329927    3.341720950734
H     -1.946049258134    3.960297051099    2.304785413820
O     -2.589458628852    3.998783233351    0.550596906782
H     -2.591550980918    3.148262732818    0.080876514269
H     -3.504852284504    4.159451983232    0.798612946505
O     -3.959255907158    0.998875052245    1.903283435761
H     -4.381799990059    1.824970745257    2.206097549490
H     -3.607882695936    1.209228931657    1.015485644600
O     -4.664773189158    3.481356257171    2.787696515611
H     -5.212442602718    3.734326079914    3.530436129532
H     -3.726432800905    3.657494644355    3.057913754652
O     -1.775550178152    0.994136761523    3.442621303583
```

```
H     -2.033602702580    0.746217462072    4.331071141497
H     -2.595730202152    0.942572329870    2.892913408881
C      0.143869649003    1.713740000067    0.775597008764
C      1.284898071684    1.541206786824    0.044599966166
C      1.996557987270    0.303276671363   -0.130676937806
C      1.567995472038   -0.913411778498    0.372184119217
C     -0.203985702348   -1.349411631617   -0.830162060528
C     -1.239810080493   -0.595252366066   -0.272239339793
C     -1.578426667551    0.749175243512   -0.764459698202
O     -2.575700937254    1.409109026597   -0.417147303286
H      1.638317534272    2.379753451222   -0.558815777942
H     -0.354522764183    2.674032022393    0.791051795823
H      2.797500500108    0.302921318519   -0.870013512723
H      2.138427476972   -1.811913608973    0.165040784219
H      0.982733267893   -0.963699346027    1.282694355531
H      0.211087472395   -1.058831074521   -1.784157617267
H     -0.145555612865   -2.410491442266   -0.620773165097
H     -0.190162461470    1.019016552041    1.533789114406
H     -1.002786312231    1.093526146156   -1.647089564315
H     -2.680690788197   -0.519118277809    1.227887382009
N     -1.948533587829   -1.066434297223    0.808355764040
H     -1.772988335144   -1.975878843005    1.193959578337

Ambimodal TS Water5-220
35
ANGSTROM
O     -0.342458726504    5.054154465233   -0.020090495278
H     -1.031406280900    5.122351282482    0.654293367273
H      0.067672853821    5.917585265308   -0.083284489684
O     -2.686067967053    3.963694354955   -1.202401055984
H     -2.692827192319    2.987652920225   -1.146205096130
H     -1.754249991207    4.218994346814   -1.232469766901
O     -3.785165915389    0.958058736083    1.635148218757
H     -3.253170508490    1.563583358986    2.181436467879
H     -3.633778206348    1.250910202307    0.722201652678
O     -2.294909278048    2.888583668544    2.933757930491
H     -2.485479943866    3.626272460776    2.320537369348
H     -2.560586965625    3.182254380220    3.805890929661
O     -2.962345649379    4.919317924807    1.233606775360
H     -3.834979270832    5.291557540978    1.366315398547
H     -2.969681812684    4.535971628353    0.313436206936
C      0.210509625732    1.579143826102    0.521506971179
C      1.333129367889    1.305854633763   -0.205008712887
C      1.993155339884    0.029085893496   -0.297212451268
C      1.539882214080   -1.124838680831    0.317612264948
C     -0.290549038256   -1.628786391773   -0.776185253399
C     -1.269605431177   -0.744901027632   -0.307572009659
C     -1.523817617025    0.534531874312   -0.994432591186
O     -2.488767285601    1.288754121873   -0.789067264496
H      1.712628910082    2.085774279150   -0.867882181607
H     -0.245033675246    2.559139202247    0.484962551416
H      2.771417099503   -0.061752312328   -1.054936289188
H      2.073788380472   -2.057242700096    0.172467221671
H      0.980660507019   -1.078579778931    1.244495393455
H      0.115970221944   -1.496251018831   -1.768275146374
H     -0.286192438560   -2.653466651034   -0.424430553352
```

```
H     -0.152945581475    0.944938458032    1.317440517698
H     -0.893846089675    0.717986577349   -1.891159170267
H     -2.683518704313   -0.357220080464    1.189155789881
N     -1.969813433313   -1.003439284357    0.841483670445
H     -1.886902172388   -1.891452956787    1.303118081399

Ambimodal TS Water5-221
35
ANGSTROM
O     -3.225976017011    2.935237637306    1.691564969536
H     -2.953525115891    2.571794247141    0.833211751306
H     -4.209008098767    3.029999729395    1.629223682535
O     -4.193306831843    5.264696928566    0.211135207364
H     -3.586522548780    4.699265951293    0.709441351432
H     -4.392307653903    6.012388551850    0.776595551727
O     -5.829815583440    3.094774690257    1.140451038622
H     -5.818069050011    2.230078075070    0.679454491109
H     -5.711453672332    3.775166126204    0.466264455077
O     -3.593983965814    0.407160344823    2.527743010561
H     -3.358909055306    1.349304263757    2.347244259346
H     -4.018444384392    0.397560102864    3.385901369042
O     -5.141602746446    0.660383239230    0.164341028158
H     -4.330994099303    0.918663496308   -0.301322385047
H     -4.832115219123    0.339357177449    1.024582303035
C      0.057676962269    1.452678476326    1.065453026621
C      1.225011324655    1.428671990482    0.359150760126
C      1.949550261099    0.244941506137   -0.022158285055
C      1.521095678139   -1.045933869258    0.246431200731
C     -0.218023338581   -1.319644117054   -1.016039867498
C     -1.259057338025   -0.581826850957   -0.440093816613
C     -1.530460595105    0.809598052597   -0.838333622752
O     -2.542131972939    1.455871095293   -0.532673298624
H      1.593275204144    2.367768957734   -0.057235174067
H     -0.467213836798    2.378607157644    1.257951589174
H      2.762032379978    0.380766661663   -0.734951767498
H      2.107563213955   -1.887517135571   -0.104571522054
H      0.935902209568   -1.262746228006    1.132809419535
H      0.233793639601   -0.967272875248   -1.932430520603
H     -0.206925054111   -2.397100986629   -0.899460730953
H     -0.300297151157    0.609656836030    1.639728882611
H     -0.842370923174    1.229022280013   -1.602293994919
H     -2.667504083873   -0.538473150099    1.095335769040
N     -2.067854840545   -1.125101987667    0.531122665329
H     -1.895769188028   -2.050391847759    0.882691342038

Ambimodal TS Water5-222
35
ANGSTROM
O     -3.192975954730    2.873973363130    1.727059973133
H     -2.938523381103    2.630208863577    0.825003686116
H     -4.119094187837    3.179569650412    1.654006504539
O     -6.914901297163    2.247315648990   -1.375618148388
H     -6.363512266122    1.561796206726   -0.971771244702
H     -7.446974811790    1.809871854670   -2.040522063313
O     -5.852931500433    3.103234276649    1.229497470484
H     -5.784351643823    2.181656439703    0.923651491332
```

```
H     -6.281338186528    3.561153464531    0.501641328487
O     -3.683624307788    0.386387399477    2.482481349572
H     -3.436441044545    1.331326460766    2.287967805960
H     -3.983686179216    0.375142670431    3.391334997440
O     -5.137547769204    0.613460186556    0.138110700855
H     -4.304025125242    0.913206285140   -0.267101370730
H     -4.865364982541    0.286944838030    1.010615073274
C      0.005228691643    1.473480546507    1.052172858584
C      1.171850395788    1.459561672612    0.344426393513
C      1.910198096676    0.281935760614   -0.029390795421
C      1.497446470170   -1.012063146181    0.250249334114
C     -0.238794753258   -1.317045448452   -1.002660475796
C     -1.288346756265   -0.579216510909   -0.440640333200
C     -1.569294038665    0.801828820029   -0.864767309505
O     -2.589169035134    1.447190927324   -0.585208603137
H      1.528452526706    2.400320284478   -0.078222624055
H     -0.532089891635    2.392619729059    1.241837497000
H      2.719891200987    0.421590066397   -0.744675779406
H      2.093792455336   -1.849323193984   -0.094609574245
H      0.918412751232   -1.228224467600    1.140864938634
H      0.209925328920   -0.976379890532   -1.925063605422
H     -0.219888527254   -2.392565970183   -0.869462151281
H     -0.340570343456    0.628516841938    1.631090573329
H     -0.873072523210    1.214106117584   -1.625613122226
H     -2.692797536600   -0.528809201830    1.101125257647
N     -2.097768302926   -1.118889411775    0.533753933501
H     -1.905456629331   -2.033245381813    0.903830691456

Ambimodal TS Water5-223
35
ANGSTROM
O     -2.507605404812    3.321705238338    1.689764597043
H     -2.400211756572    2.887752823875    0.831313533946
H     -3.306239168213    3.849595068297    1.606876268068
O     -4.689277917586    3.473643986240   -0.287029006412
H     -3.939861745245    2.943834141809   -0.589908485550
H     -5.341751999144    3.442474455119   -0.988694894429
O     -5.715197557191    1.482888786606    1.438484482937
H     -5.652851965828    0.913275596056    0.652368816595
H     -5.490498774467    2.364985786840    1.107610214844
O     -3.275885468383    0.727258398410    2.386403829534
H     -4.223730211240    0.898829461098    2.208602837369
H     -2.869609716319    1.609463064496    2.385584206182
O     -4.747848027805    0.198188444134   -0.792066019467
H     -3.931605722590    0.718528694422   -0.692554313643
H     -4.510614208390   -0.698499468781   -0.545622925123
C      0.185909847376    1.429688781017    1.137797033436
C      1.369505151534    1.278084638063    0.478154627866
C      1.976027832159    0.020715493749    0.115644208427
C      1.410555006007   -1.214873188124    0.365009574962
C     -0.309774811009   -1.277093470067   -1.009696210630
C     -1.286263229380   -0.441043714603   -0.461173737877
C     -1.389861615083    0.969539618115   -0.850008310089
O     -2.372511975557    1.700703771271   -0.610825858552
H      1.859583310676    2.171368515825    0.086709653712
H     -0.231000046457    2.408773507662    1.329471768000
```

```
H      2.830388633550    0.071292172578   -0.559043235590
H      1.911022043993   -2.116152223973    0.030407505343
H      0.749143827218   -1.367319315378    1.209701101436
H      0.231284091782   -0.961148078326   -1.890290273501
H     -0.407816768633   -2.351051638183   -0.904507977945
H     -0.291726245261    0.628212500788    1.684329182263
H     -0.618312429191    1.342835755817   -1.552227917657
H     -2.649299128328   -0.252300489841    1.116886967299
N     -2.224844106269   -0.909822244919    0.441254327727
H     -2.096384228087   -1.837365549402    0.813851346905

Ambimodal TS Water5-224
35
ANGSTROM
O     -3.210387030398    3.111412850199    1.612940178409
H     -2.907902090248    2.956032104365    0.707774319120
H     -4.182522376964    3.253450395941    1.541640724113
O     -5.029891330360    1.210360990443   -0.890809523938
H     -4.089190274778    1.463250561898   -0.803480895000
H     -5.164747274706    0.977391366187   -1.809280342740
O     -5.867607714842    3.037156095914    1.202742225696
H     -6.050987313548    2.175014879691    1.624220052359
H     -5.848275810285    2.832602805065    0.260947969197
O     -3.191549988038    0.653492344367    2.533124007167
H     -3.151178697156    1.605979370509    2.236385714468
H     -3.137500866291    0.677945995172    3.488707834150
O     -5.812804176816    0.343081169872    1.693927988363
H     -5.681043212173    0.373570775077    0.736375195199
H     -4.917228586578    0.326938449002    2.074169615643
C     -0.081495423664    1.463797811431    1.151178844480
C      1.170296194564    1.315877558087    0.627083365032
C      1.785973725398    0.065603857494    0.267393187286
C      1.157510121714   -1.165670423999    0.366851345107
C     -0.374409677323   -1.107077660090   -1.168241317688
C     -1.391082760889   -0.274317753921   -0.688102114901
C     -1.423234660561    1.163821879279   -0.991156348742
O     -2.393414362722    1.913137819533   -0.790729561019
H      1.719127583388    2.215649895358    0.343697264553
H     -0.504744512981    2.442660602970    1.333153178342
H      2.714068728641    0.126163953900   -0.300114168247
H      1.677528479496   -2.059970385515    0.042576910451
H      0.411932564405   -1.348225831175    1.132298076057
H      0.260693178332   -0.759621325937   -1.971189851759
H     -0.516429479288   -2.181324573508   -1.145368723830
H     -0.630933190843    0.649831846218    1.603332714783
H     -0.578169918280    1.546018534234   -1.600155036447
H     -2.926016241905   -0.146368014937    0.696765717904
N     -2.425107774496   -0.773296230559    0.081139632729
H     -2.380035389629   -1.724155268747    0.408163988021

Cope TS Water5-1
35
ANGSTROM
C     -0.060497443723    0.941786392668    0.477586600505
C      1.066347653391    0.847689156237   -0.469792354907
C      1.549806377309   -0.333589511214   -0.896342420870
```

```
C      0.881613939345   -1.642049726504   -0.484958685091
C     -0.677262021316   -1.600903277113   -0.851640605616
C     -1.494014969723   -0.555967871917   -0.086017054612
C     -1.886804196940    0.716185303407   -0.677786520137
O     -2.816137775345    1.457383667646   -0.258633890440
H      1.456856010565    1.783495284810   -0.869852686853
H     -0.402654056083    1.947442331800    0.709921821305
H      2.359574336830   -0.387755056353   -1.617610921292
H      1.337952954934   -2.498486405310   -0.992297281342
H      0.961676289907   -1.815084700960    0.597392161639
H     -0.771339799384   -1.435364432329   -1.931192394761
H     -1.095395838204   -2.587483930830   -0.619832092223
H      0.038488657268    0.353481936303    1.388289526845
H     -1.419155418649    0.975539502893   -1.646228640678
H     -2.719665569033   -0.400215441337    1.576543411790
N     -2.110625431001   -1.000413143109    1.036935010714
H     -1.902717302292   -1.912868361192    1.420853914487
O     -3.735194940414    1.183556012206    2.249070844915
H     -3.416424084511    1.466400410834    1.362038993296
H     -3.573688813590    1.916837849088    2.842576914349
O     -4.997279107602   -0.194599582027   -0.075094978852
H     -5.361209312853    0.004825691445    0.791405954590
H     -4.278834781932    0.459445849445   -0.189894885880
O     -4.033418191243   -1.479351401069   -2.457477780668
H     -4.531415024343   -1.334706603339   -3.263231832989
H     -4.456992643405   -0.940873393374   -1.774860450249
O     -4.323914460278   -2.988502294850   -0.089876644655
H     -4.081051068897   -2.917266587677   -1.021836244072
H     -4.589798038362   -2.087165601537    0.150006731326
O     -2.204425415722   -3.813572312790    1.376905194235
H     -2.484263858328   -4.473449821511    2.012005840037
H     -2.997039178473   -3.606394452914    0.835024069026

Cope TS Water5-2
35
ANGSTROM
C      0.099214687690    0.906359333401    0.402223047813
C      1.292615188323    0.771201311467   -0.453473210394
C      1.809972220050   -0.425954689755   -0.786836241200
C      1.114245087157   -1.719114506672   -0.372908851849
C     -0.410199540157   -1.693715021102   -0.866035927534
C     -1.286786645480   -0.637781182061   -0.192470030548
C     -1.643264836639    0.612371227100   -0.841978320461
O     -2.595664063461    1.371316779510   -0.507564500275
H      1.711992675887    1.688856856967   -0.866158163019
H     -0.260567895963    1.919997597336    0.560853915518
H      2.671959984529   -0.507477369222   -1.442147833174
H      1.610360423299   -2.594005962256   -0.806128523146
H      1.107639274986   -1.844263402999    0.718768306130
H     -0.415551471792   -1.548366398202   -1.952371142805
H     -0.850635281298   -2.675646277775   -0.653646312470
H      0.126297454421    0.359861589442    1.342853047858
H     -1.124726505307    0.835446823830   -1.793750534785
H     -2.719347464602   -0.504493670608    1.279564573541
N     -1.933039330816   -1.035365679883    0.936194498440
H     -1.880834617966   -1.995473775798    1.249348192599
```

```
O     -4.599386097507    0.402867538968    0.907744199539
H     -3.856699391900    0.832464335848    0.405431145595
H     -4.942329099997    1.067216340767    1.505693008623
O     -4.783038497833    0.975609734667   -2.077617885815
H     -5.361439475490    0.763271775478   -1.337855257344
H     -3.993228671190    1.378762364800   -1.672987486319
O     -3.607517153477   -1.414168450086   -2.491218016772
H     -3.959507206914   -1.842929921636   -3.272586593155
H     -4.031036356677   -0.526682695653   -2.451609518788
O     -4.606071521697   -2.306830786292   -0.093975523224
H     -4.220181031077   -2.083353002126   -0.962591848904
H     -4.724942446304   -1.459142250769    0.354814142111
O     -2.643836159017   -3.755030830566    1.065240381500
H     -3.020955140226   -4.476018982920    1.569956968980
H     -3.401561068605   -3.312820822391    0.618320541408

Cope TS Water5-3
35
ANGSTROM
C      0.186491438272    1.027444674713    0.583743855068
C      1.305609518624    0.841739486774   -0.358439115124
C      1.760549217888   -0.376304683056   -0.706781449606
C      1.061802005577   -1.639554920724   -0.213245912092
C     -0.495460616662   -1.580050131907   -0.589074611918
C     -1.284942537800   -0.480929680509    0.126212962102
C     -1.657782029160    0.765916063481   -0.525787659905
O     -2.564010574981    1.553694766253   -0.143709443765
H      1.717173840377    1.738851880943   -0.821050617273
H     -0.129898298389    2.054393997187    0.748980760016
H      2.566010059632   -0.496146253901   -1.424910749255
H      1.497364517969   -2.537364998472   -0.664043546028
H      1.134997056833   -1.744730558485    0.878092129217
H     -0.576489262170   -1.461637604462   -1.676269368192
H     -0.940879586969   -2.543632644135   -0.312932794010
H      0.272879966316    0.498928989970    1.531340855319
H     -1.204884652133    0.953953500332   -1.518121394152
H     -2.493833904832   -0.229559041085    1.784662105096
N     -1.876883149795   -0.851651509959    1.288542711851
H     -1.728023325052   -1.770081227313    1.681338729541
O     -3.581641573222    1.501936057562    2.261533022743
H     -3.213705260429    1.574657894503    1.334026973309
H     -3.771268881315    2.399558387458    2.535803399298
O     -5.562927652881   -0.323657033374    2.119383850392
H     -6.153474679816   -0.217784943504    2.865372827534
H     -4.844629548972    0.335853388227    2.230694243460
O     -4.818258799983   -0.155210479548   -0.691351568110
H     -4.149134087258    0.538953083636   -0.620690664951
H     -5.379211435611   -0.057544808989    0.087410320039
O     -4.534832401153   -2.562384776810    0.700694873871
H     -4.497062064217   -1.979479264680   -0.073719192660
H     -4.929593173425   -2.001599116464    1.383728488582
O     -2.208049930521   -3.644260913797    1.531631901620
H     -2.403972972861   -4.460656059329    1.992232858187
H     -3.067124192344   -3.321833842672    1.178118030926

Cope TS Water5-4
```

```
35
ANGSTROM
C     -0.055557970846    0.816021905249    0.652846300963
C      1.226105426732    0.736857669063   -0.072775594523
C      1.735078629113   -0.425140520748   -0.522824524374
C      0.943684943286   -1.724416003215   -0.406478596559
C     -0.502834090359   -1.526950059889   -1.065186106878
C     -1.419655260305   -0.565013106566   -0.306371389156
C     -1.676765750977    0.792797739172   -0.772754763710
O     -2.629590644800    1.530644450781   -0.406490934826
H      1.730170904889    1.679379311260   -0.288145068143
H     -0.382836610781    1.817370814915    0.922404242760
H      2.669386331833   -0.455860859421   -1.075605644846
H      1.455610759462   -2.547320605409   -0.916699437726
H      0.791261708868   -2.024699514526    0.639243697390
H     -0.364960170130   -1.188020135623   -2.099097569203
H     -0.996329684763   -2.504860516395   -1.090153277975
H     -0.157321347814    0.141797736929    1.501603340094
H     -1.067656825920    1.132964354467   -1.634243226077
H     -2.849969644462   -0.571788295370    1.176403190030
N     -2.202934262098   -1.133046529395    0.639538971096
H     -1.963054075446   -2.040936639853    1.031738915925
O     -3.924995489006    0.952426272038    1.855321343887
H     -3.456721540618    1.322175053375    1.067565438703
H     -4.012744715193    1.672126796367    2.479928619993
O     -4.955183540881    0.043452605000   -0.692037810041
H     -5.113482297993    0.016949044932    0.258241812015
H     -4.236656851407    0.691997560672   -0.794940388569
O     -4.024373698981   -2.193034855576   -1.845520053395
H     -4.683887664481   -2.337409539756   -2.526207557047
H     -4.328860564878   -1.392480503766   -1.359265155850
O     -3.387927966103   -4.435201507124   -0.493356983692
H     -3.048946205058   -4.987288912098   -1.199615153202
H     -3.640793838773   -3.583129136133   -0.912477492944
O     -1.700819677744   -3.783887698430    1.510012787568
H     -2.086937928253   -4.094623995285    2.330001455360
H     -2.294794592079   -4.102829925606    0.796859145001

Cope TS Water5-5
35
ANGSTROM
C     -0.242926877836    0.568254953581    0.894812615943
C      1.118374463248    0.647415847392    0.329380065624
C      1.745682365112   -0.423915024593   -0.189099320742
C      1.020449363356   -1.761361591236   -0.315611861540
C     -0.351726177184   -1.552052418404   -1.112805270120
C     -1.402184530676   -0.707051368144   -0.376949750347
C     -1.663002476557    0.680624347469   -0.734931133720
O     -2.680627898566    1.350566844204   -0.413361677442
H      1.584095540397    1.632316469878    0.295168178634
H     -0.663011841915    1.504398501908    1.253354274115
H      2.738089522772   -0.347824903965   -0.623004946980
H      1.631416562999   -2.496617227837   -0.850291215196
H      0.771108837435   -2.185601324427    0.666938502300
H     -0.113600964793   -1.102653060682   -2.085191955996
H     -0.788345437628   -2.541953142016   -1.293645826355
```

```
H     -0.397132223309   -0.214887643485    1.635656344464
H     -0.963519677697    1.134349361865   -1.466349706931
H     -3.029559857819   -0.930973210236    0.877091549480
N     -2.288725899340   -1.406647675207    0.370573981831
H     -2.028749605459   -2.305589223388    0.766333971224
O     -3.276504063231    1.968586787297    2.133936981468
H     -3.042752261409    1.808116617498    1.194656569759
H     -3.840162713872    2.743286591668    2.137454991564
O     -4.481059872957   -0.480037069252    2.121364329451
H     -4.055898798634    0.362502654929    2.367265324730
H     -4.988849480768   -0.285102697577    1.316550363550
O     -5.213553444029    0.410050518408   -0.357661212575
H     -5.764392481640    1.033646067418   -0.831159318990
H     -4.292085781367    0.736062654708   -0.446138852535
O     -4.110857958276   -3.012910665468    3.049322602552
H     -4.892274081553   -3.456988989344    2.716220264160
H     -4.219334216455   -2.072121117053    2.810047061609
O     -1.749960841404   -3.727183017484    1.939020415343
H     -1.135831618270   -3.899793444240    2.653824218364
H     -2.615507240171   -3.556307540092    2.368789232292

Cope TS Water5-6
35
ANGSTROM
C     -0.530064752166    0.404630298527    0.981925608882
C      0.904699848285    0.590441194137    0.697840296252
C      1.660228922953   -0.377258442478    0.146491327105
C      1.022153827864   -1.680578934973   -0.320709588170
C     -0.163673780054   -1.343100423683   -1.347458469192
C     -1.385209128867   -0.685190895013   -0.706129915821
C     -1.661713417842    0.742127921272   -0.777830308725
O     -2.768159887615    1.279653976881   -0.485267293862
H      1.329304560655    1.572464499540    0.907307555070
H     -1.030374069579    1.260716715643    1.427754094356
H      2.711408689112   -0.221193699011   -0.077134908158
H      1.751293647076   -2.327844842700   -0.819523344852
H      0.582290393600   -2.239782936405    0.516385869684
H      0.243847806112   -0.704807776407   -2.141668628150
H     -0.491524243048   -2.284934213956   -1.804528085620
H     -0.785989572824   -0.509860539984    1.514918681329
H     -0.913069091247    1.365728626426   -1.307487914138
H     -3.205167299221   -1.149966112522    0.109380321247
N     -2.354083115545   -1.530482466384   -0.281178021273
H     -2.110926252536   -2.436717266217    0.094274295350
O     -3.896097006435    1.267428062898    1.936733506352
H     -3.459016732160    1.301037312752    1.055083396182
H     -3.637440796467    2.063405208234    2.401187996788
O     -5.393856390020    0.522215317482   -0.516811511777
H     -5.609008178860    1.014601392881    0.281341525045
H     -4.473930742977    0.781082731401   -0.720871876089
O     -3.732294719715   -1.410954015529    2.681175441375
H     -3.759812899169   -0.446471160004    2.567165694612
H     -4.365820346072   -1.753616406107    2.029610231317
O     -5.305457053878   -1.962400657419    0.477797521185
H     -6.094278652316   -2.459141649850    0.262134664228
H     -5.406751370257   -1.081142798987    0.049097575633
```

```
O     -1.443057564450   -2.723237393309    2.129509096108
H     -2.261213969604   -2.240468795209    2.388761247725
H     -1.314087482339   -3.387640547060    2.806951957132

Cope TS Water5-7
35
ANGSTROM
C      0.116227035169    0.927349452235    0.393376284538
C      1.225375299432    0.618638873520   -0.525681247971
C      1.689631657300   -0.630838701856   -0.720576869110
C      1.010727282788   -1.833506442395   -0.072538102316
C     -0.547915480642   -1.836461029253   -0.456368884568
C     -1.345263453701   -0.691655461126    0.162752869868
C     -1.733852918732    0.491668579855   -0.589527327391
O     -2.650078679177    1.299263535941   -0.272024073806
H      1.628007183212    1.448465436138   -1.106881488635
H     -0.199827478399    1.966855907774    0.424322179932
H      2.488796106489   -0.830973630513   -1.427895484598
H      1.459984239619   -2.773190944233   -0.410884625619
H      1.083458971348   -1.801362999620    1.023546649727
H     -0.626023115734   -1.812294291749   -1.549851105343
H     -0.987488304454   -2.778369030243   -0.104242318522
H      0.203199222053    0.525054375680    1.400418056309
H     -1.321504679599    0.567312321210   -1.615696637325
H     -2.508625564010   -0.287747790055    1.820570469228
N     -1.858045369925   -0.930805176780    1.394839041094
H     -1.774474052739   -1.837084282761    1.825020411107
O     -4.136242321938    0.878230192704    1.894623696450
H     -3.613205621502    1.189643499464    1.114029912439
H     -5.006402376147    1.278919840487    1.804918514242
O     -6.905329855314    0.608504797716    0.864969610170
H     -6.515753403123   -0.279026183467    0.948385699957
H     -7.851942716018    0.506748620351    0.964846012747
O     -4.981199065559    0.206691447878   -1.288367910160
H     -5.719452349094    0.686788845723   -0.894199856726
H     -4.186801425861    0.725770984164   -1.074941643109
O     -4.996090843733   -1.484723471407    0.838243544206
H     -4.928485090202   -1.142280060259   -0.076498024255
H     -4.615001130890   -0.766681523186    1.385569332358
O     -3.120147733509   -3.451410076952    1.267295219753
H     -3.574178064133   -4.278401540513    1.432438796926
H     -3.823370312789   -2.794171514286    1.086118543961

Cope TS Water5-8
35
ANGSTROM
C      0.010653907494    1.064243232626    0.440354141797
C      1.253919554347    0.994171415382   -0.336165122414
C      1.839508313733   -0.162571178265   -0.711665407467
C      1.190306820933   -1.512457827218   -0.432827382135
C     -0.284746674757   -1.552617369987   -1.082410452956
C     -1.291212630790   -0.654607008865   -0.392169179402
C     -1.616181802756    0.721735453042   -0.795521889291
O     -2.629352341185    1.333652733255   -0.360573443935
H      1.670652065723    1.942365947951   -0.677253318860
H     -0.349998353274    2.062570989386    0.670124688065
```

```
H      2.735899411132   -0.163603044291   -1.324462113086
H      1.779777719622   -2.331111265420   -0.859753977974
H      1.081511019176   -1.702311466123    0.644420939302
H     -0.191247779870   -1.281850269048   -2.140347760807
H     -0.649925790537   -2.586209916510   -1.027209454499
H     -0.050464128381    0.420146456033    1.315095304216
H     -1.137228576135    1.083317113403   -1.730925555093
H     -2.730095051074   -0.604361430982    1.036618505946
N     -1.989869744947   -1.165161977565    0.629619761640
H     -1.842597411721   -2.076336776590    1.036072364769
O     -4.367178408499   -1.023769692062    2.422185282564
H     -3.875463119810   -1.864447447497    2.479432063090
H     -4.273040274104   -0.581385794559    3.266197524298
O     -4.863916907211   -0.080908760569   -0.243459004781
H     -4.912089808891   -0.323682067755    0.690608472306
H     -4.129239145878    0.578643635710   -0.303494837630
O     -3.830659322434   -2.070014848825   -1.615995810277
H     -4.256247089282   -2.082080604794   -2.474078906041
H     -4.217550711666   -1.287198377348   -1.133608643011
O     -4.637088009747   -3.780891926075    0.255522524683
H     -4.267303316293   -3.251870532330   -0.491080284165
H     -5.430443008920   -3.311442782969    0.524585547417
O     -2.950802632829   -3.383264137645    2.245547436862
H     -2.972083209474   -4.137849953064    2.835292057558
H     -3.531702346326   -3.620624648297    1.476068976461

Cope TS Water5-9
35
ANGSTROM
C      0.010107795852    0.996636442587    0.644197538720
C      1.262512827852    0.981876805527   -0.132954290466
C      1.874210706197   -0.162999350288   -0.490494272459
C      1.233184161611   -1.514277548154   -0.187275065824
C     -0.253525916890   -1.556086693008   -0.785095303236
C     -1.235896387420   -0.579605490326   -0.128724825653
C     -1.623094093157    0.674173681301   -0.759600232239
O     -2.645096742326    1.350137041420   -0.474989649210
H      1.648909605362    1.943899232753   -0.469980516156
H     -0.423852815552    1.974762880226    0.835228191714
H      2.780079867873   -0.160035870981   -1.089071844740
H      1.810335548960   -2.336060577061   -0.624257133130
H      1.160324166397   -1.695316482225    0.894123119101
H     -0.187504461052   -1.363257165763   -1.863069615682
H     -0.642415127385   -2.571477800897   -0.640074914381
H      0.011524587894    0.399412713838    1.553846302844
H     -1.038170142380    0.974205231655   -1.652522329056
H     -2.690372891876   -0.552422764684    1.357707707651
N     -1.952064701548   -1.078424486426    0.907972233322
H     -1.804048165036   -2.023229946024    1.231491176760
O     -3.296729123718    2.673346149796    1.729848610544
H     -3.002808304599    2.262665695871    0.885309015054
H     -4.022248358210    3.252726875980    1.490737217197
O     -4.875480398625   -0.211141948010   -0.011823216565
H     -4.883794667501   -0.164412204660    0.953392415707
H     -4.154035457600    0.387747498259   -0.283704253375
O     -4.281256212226   -2.758690744998   -0.638123448057
```

```
H     -4.403779808863   -2.799161952075   -1.587047530780
H     -4.525104179436   -1.840308336396   -0.376030013331
O     -3.999927092469    0.246007357336    2.612723221130
H     -3.750894830527    1.175432897108    2.402742397375
H     -4.009639029118    0.171828846247    3.566890661980
O     -2.210324659471   -3.901784367415    0.720424621850
H     -2.582250239527   -4.632055700323    1.216479008534
H     -2.952054009274   -3.530627130457    0.200849862845

Cope TS Water5-10
35
ANGSTROM
C      0.096732810235    0.897671260239    0.464697120304
C      1.276154632541    0.718335922056   -0.408570085797
C      1.796871381919   -0.491200534378   -0.680475083919
C      1.118649415761   -1.764757414441   -0.180552677141
C     -0.415841700037   -1.761500781909   -0.633783217585
C     -1.275152055809   -0.677710896169    0.020992460628
C     -1.682048361763    0.513801703509   -0.703142919300
O     -2.640060924484    1.279561341460   -0.390688623048
H      1.681008616268    1.613766316510   -0.879476377162
H     -0.265856062026    1.919085653314    0.558039910691
H      2.648291427126   -0.604481204006   -1.344230793521
H      1.606446207566   -2.658101639149   -0.584247406963
H      1.151808476853   -1.834074581828    0.916796702067
H     -0.453453629158   -1.653531010725   -1.723544473682
H     -0.859636301663   -2.732796739601   -0.384705885755
H      0.165885733283    0.433732744189    1.447840085515
H     -1.198880304571    0.677285863937   -1.681842358532
H     -2.551430265915   -0.405100240631    1.638565394062
N     -1.903032055100   -1.027866310727    1.175236230946
H     -1.662254285279   -1.870147674633    1.665444548156
O     -4.993911198335   -0.208219651702   -0.074314639427
H     -4.168660689705    0.306040986449   -0.128278264800
H     -5.271352328067   -0.142772528772    0.841601582450
O     -4.364825561645    0.981231857443   -2.622597658022
H     -5.029796931050    0.552042201131   -2.070767627774
H     -3.775282130417    1.430180632949   -1.998618390136
O     -2.765522509304   -1.130171080374   -3.234566685688
H     -3.080074618837   -1.543083107729   -4.040319488839
H     -3.331170308844   -0.338726847977   -3.099851410948
O     -3.543689373388   -2.536455777866   -0.910024057248
H     -3.216666657608   -2.169932882179   -1.745941708778
H     -4.191395020361   -1.892615495020   -0.591594215352
O     -3.506842564885    1.230697068128    2.155863976600
H     -3.446475507023    2.037127502963    2.667776452934
H     -3.215761584344    1.452436934374    1.244233130152

Cope TS Water5-11
35
ANGSTROM
C     -0.005029237714    0.980247317560    0.656535361443
C      1.257610474248    0.980322602981   -0.104018469358
C      1.878146175048   -0.157495086251   -0.468788118358
C      1.238068379638   -1.514955189008   -0.192360596482
C     -0.240426802424   -1.553807194690   -0.810411089026
```

```
C      -1.234948928681   -0.589689025130   -0.154064095569
C      -1.618302000857    0.671163498141   -0.773078961468
O      -2.646434530518    1.339684029604   -0.493002460327
H       1.644944016782    1.948143109081   -0.422879710477
H      -0.445032263402    1.954196961028    0.854942213482
H       2.791851796192   -0.143309677712   -1.055169940228
H       1.823940469645   -2.328756086597   -0.632663208612
H       1.151541230992   -1.710852995458    0.885434751357
H      -0.160825739477   -1.346189834614   -1.884715225170
H      -0.627514165185   -2.572403072664   -0.684273702464
H      -0.013499228182    0.370784437840    1.557991028748
H      -1.022689156580    0.985260942118   -1.654017648418
H      -2.709085934596   -0.587632389761    1.313123119863
N      -1.962977328187   -1.104952035957    0.866199053700
H      -1.815831386376   -2.053529777776    1.178895534721
O      -3.332030130668    2.630696392857    1.720606498041
H      -3.025453427450    2.232492195701    0.874592815844
H      -4.056415858495    3.210750000047    1.479696612140
O      -4.877076513494   -0.235405895642   -0.080625616830
H      -4.898351266051   -0.201763120300    0.884936170074
H      -4.154271245002    0.369592545466   -0.334791109921
O      -4.265357468775   -2.772185003005   -0.733243992291
H      -4.375146906876   -2.800241754864   -1.684172992540
H      -4.515990760100   -1.858278452729   -0.462063575062
O      -4.038047939461    0.189226558510    2.561256534785
H      -3.789631333061    1.122261006736    2.367151524154
H      -4.060134844420    0.102117511663    3.514124030661
O      -2.208475097112   -3.926392020053    0.637199231796
H      -2.584287847515   -4.664584932529    1.118375713939
H      -2.944596382599   -3.550804433961    0.112854435310

Cope TS Water5-12
35
ANGSTROM
C       0.092416098384    1.073442222953    0.570634882530
C       1.315207054347    0.975306893084   -0.245926945971
C       1.868079866589   -0.203533257818   -0.590092258822
C       1.181307388927   -1.521587293735   -0.245645178010
C      -0.318743436267   -1.498366919731   -0.811635399894
C      -1.235498777762   -0.501354787849   -0.107415452705
C      -1.631253917759    0.763880176807   -0.705325388488
O      -2.630982390933    1.449929000150   -0.355224521794
H       1.733776480557    1.909193912443   -0.622036869277
H      -0.289374873120    2.078410560630    0.732995425910
H       2.754897828510   -0.249788638136   -1.215025235869
H       1.713003376108   -2.372976943572   -0.683367254358
H       1.125239798830   -1.681614879371    0.840000802842
H      -0.273493257833   -1.280783775117   -1.885254388914
H      -0.749950244911   -2.499321108239   -0.692878745190
H       0.094432146316    0.510924233556    1.501729624617
H      -1.111631994007    1.057141352282   -1.637229195911
H      -2.582636648307   -0.381034419351    1.437270126017
N      -1.880482299095   -0.954766831468    0.998465865144
H      -1.895068733625   -1.931886706024    1.266219656285
O      -3.931717904589    1.221645649697    1.914190337884
H      -3.447348094875    1.374116062316    1.055280138363
```

```
H     -4.353866953392    2.054565305528    2.125575357040
O     -4.646167090919   -0.493915203337   -1.060080120732
H     -4.960579737094   -0.752218640308   -0.177395566286
H     -4.086950237852    0.284986190461   -0.925917562889
O     -3.282150992999   -2.767169296149   -1.523755514436
H     -3.800446594621   -3.289608518793   -2.137146109049
H     -3.754542993946   -1.908145990940   -1.437841163015
O     -5.200711049629   -1.131245527128    1.599367830358
H     -4.773682506618   -0.280569304752    1.840020083154
H     -6.037020126437   -1.164251357919    2.064793541972
O     -3.273806931462   -3.332512690512    1.195451396560
H     -4.006501690248   -2.755258901244    1.444372439501
H     -3.255710942330   -3.307252200534    0.222437203456

Cope TS Water5-13
35
ANGSTROM
C      0.018334052705    0.982750973366    0.639755686824
C      1.277618006961    0.963722446922   -0.123122054908
C      1.884599840526   -0.180048221712   -0.493562393164
C      1.227216082305   -1.531173305047   -0.233550292609
C     -0.248582642836   -1.537317761536   -0.861722921332
C     -1.237838001739   -0.597339620232   -0.173007720341
C     -1.636020945126    0.682139291535   -0.745652978524
O     -2.653610435829    1.341159958598   -0.413528363584
H      1.677611281462    1.927141594076   -0.440551055865
H     -0.399451635567    1.968289941503    0.830220049308
H      2.797531454672   -0.170739231577   -1.081301141719
H      1.803908216095   -2.347339763797   -0.681116661539
H      1.130209776262   -1.739445828254    0.840802087029
H     -0.163766817215   -1.287020417038   -1.926030960941
H     -0.641876023184   -2.557404173419   -0.780990310982
H     -0.003522340476    0.373527872596    1.540706980076
H     -1.071491300955    1.015685991792   -1.639774817238
H     -2.723680063980   -0.648502922963    1.263546087777
N     -1.929634861867   -1.125579810621    0.865888345908
H     -1.810737028084   -2.094873423888    1.133069234986
O     -3.551008195346    2.167969885503    1.965244771846
H     -3.276523205166    1.957031741055    1.044185473415
H     -2.810157382373    2.617516476486    2.373216249827
O     -4.808384198600   -0.434006984585   -0.350491989314
H     -4.871736985353   -0.486419024834    0.619071306366
H     -4.178231551524    0.287820547954   -0.517352651563
O     -3.771274940058   -2.758009313991   -1.175137861561
H     -4.401096461171   -3.112964339420   -1.803084258489
H     -4.146312137205   -1.894290997555   -0.877924177478
O     -4.527577119282   -0.283198229532    2.403380957199
H     -4.219427678548    0.648643967430    2.360723378610
H     -5.308600216624   -0.281809822426    2.957354523608
O     -2.245650422391   -3.935773045529    0.758982906300
H     -2.851684194202   -4.419396706798    1.321934171706
H     -2.779966467717   -3.601791757938    0.011799439274

Cope TS Water5-14
35
ANGSTROM
```

```
C       0.108267910896    1.029587405123    0.588051141409
C       1.330368528056    0.970324204478   -0.233276712266
C       1.909040499868   -0.190871570007   -0.594587288175
C       1.253798598332   -1.528776109172   -0.265594629910
C      -0.248194201445   -1.532605891124   -0.826878475451
C      -1.185326272987   -0.566270155434   -0.106893745804
C      -1.611920799674    0.697281885748   -0.686886214694
O      -2.626002084991    1.355509699413   -0.324855911443
H       1.726192975244    1.918447631678   -0.598227364231
H      -0.295960511613    2.023293967292    0.764780610627
H       2.794681304418   -0.208513929278   -1.222644608924
H       1.803486377279   -2.361893478719   -0.716065904733
H       1.204893267971   -1.704303325420    0.818000342011
H      -0.211385539674   -1.299950012608   -1.897655811057
H      -0.655937564187   -2.544681448139   -0.720106941998
H       0.126171310787    0.455077006901    1.511623144564
H      -1.102150563813    1.014630824355   -1.616336426374
H      -2.529918678066   -0.497256448815    1.443144719585
N      -1.816192796767   -1.048881623588    0.994765615286
H      -1.807497768726   -2.029512942754    1.249627841190
O      -3.913885607691    1.067585093786    1.945105689065
H      -3.435893720463    1.242422733612    1.086884510320
H      -4.354357318307    1.887731208626    2.168698232342
O      -4.598299574074   -0.624710934271   -1.049486478866
H      -4.903875072430   -0.901750541478   -0.169383837970
H      -4.056675654055    0.165008343760   -0.906666803137
O      -3.183972796106   -2.859721641316   -1.547106621828
H      -3.692105116857   -3.385830165819   -2.165841175798
H      -3.675673798350   -2.012986867089   -1.448481730248
O      -5.129556661102   -1.309513278279    1.602905706796
H      -4.721391847762   -0.452482036714    1.853536662315
H      -5.963393660328   -1.367847523555    2.070277421179
O      -3.153953461907   -3.460409386044    1.164358320867
H      -3.898900179435   -2.903470802572    1.423006814912
H      -3.139557030366   -3.421955127844    0.191714397105

Cope TS Water5-15
35
ANGSTROM
C       0.001701402738    1.022292426673    0.418317602421
C       1.228903770355    0.898992897312   -0.389036584274
C       1.804769487114   -0.285168917984   -0.670825003712
C       1.146752670464   -1.598231930369   -0.254897876986
C      -0.354500933872   -1.640893090864   -0.819391477503
C      -1.302856898394   -0.637732035894   -0.166165119804
C      -1.693182012412    0.604062166536   -0.819538366162
O      -2.683993347678    1.315806453480   -0.509103326639
H       1.632684660404    1.818182566654   -0.813662614341
H      -0.386298210780    2.030789122446    0.536719432120
H       2.692502626377   -0.348152246007   -1.292585477304
H       1.697854813190   -2.459686521100   -0.647142523560
H       1.099921556232   -1.700420656992    0.838951736088
H      -0.312544799718   -1.474584930273   -1.902006103720
H      -0.762578731255   -2.645527962170   -0.654101627715
H       0.010102491866    0.507377656251    1.377049351275
H      -1.178060652136    0.827818666379   -1.775842538048
```

```
H     -2.645360238081   -0.502993573910    1.419660246700
N     -1.943447747630   -1.060776257907    0.946593522841
H     -1.700673985534   -1.927278733039    1.392879539347
O     -3.732943500986    2.662344591217    1.625053564209
H     -3.234053913239    2.368293418717    0.841819566111
H     -4.642808568881    2.790205100624    1.296223688709
O     -4.936125873055   -0.030247295606    0.008938903034
H     -4.816458866133   -0.154192257300    0.965267185621
H     -4.139091101266    0.481334513901   -0.264455880645
O     -3.790012377880   -2.367041622641   -0.981998167042
H     -4.402352201561   -2.798101284655   -1.579780037212
H     -4.258991289892   -1.580094815615   -0.646935874850
O     -3.967298553751    0.231490389155    2.579398529860
H     -3.853520790489    1.191893875730    2.330328169209
H     -4.315533096466    0.224109617684    3.470495883920
O     -6.139269684644    2.416520658830    0.306913113096
H     -5.901201213595    1.481798590244    0.140888072964
H     -6.136862030030    2.846997098310   -0.549215697007

Cope TS Water5-16
35
ANGSTROM
C      0.004420290191    0.958786107925    0.594696600256
C      1.233334578966    0.951302406402   -0.219192078126
C      1.831141923789   -0.190207870966   -0.609488409215
C      1.195689382449   -1.543853080680   -0.305096574345
C     -0.308099755483   -1.574400559968   -0.859288779683
C     -1.268064757341   -0.604324596467   -0.161571744840
C     -1.670305899891    0.658493439542   -0.764382382447
O     -2.681651586225    1.333034002911   -0.440972484132
H      1.612223351272    1.916756882776   -0.554925980991
H     -0.421083916058    1.935329478089    0.811156890865
H      2.718982489426   -0.181505655930   -1.234441379778
H      1.757495146689   -2.361179387658   -0.769570859595
H      1.154241553433   -1.738857563264    0.775533342592
H     -0.273354870417   -1.367634822793   -1.936141316175
H     -0.695282083162   -2.590701002793   -0.716082506862
H      0.031033665734    0.349709537280    1.496060282407
H     -1.111144277625    0.968831100238   -1.670193431843
H     -2.678024233653   -0.593233365802    1.367353859201
N     -1.954711872677   -1.115010076550    0.889225200230
H     -1.799771564767   -2.064306923694    1.195916535109
O     -3.264450061469    2.628786550319    1.799158978738
H     -2.996655582960    2.228512136292    0.941043876267
H     -3.995137095358    3.212920676209    1.589115531075
O     -4.901617630434   -0.229011731230    0.067449328335
H     -4.881354107832   -0.194893634391    1.033016241784
H     -4.186886519173    0.371713191555   -0.217786770235
O     -4.332990372165   -2.769520078232   -0.609189146281
H     -4.483561415065   -2.797301154745   -1.554527607590
H     -4.566525563054   -1.854081310765   -0.328094156494
O     -3.947862664686    0.191735807767    2.670743854478
H     -3.702622133338    1.123250963148    2.465615983312
H     -3.929652703985    0.105103439933    3.623736767528
O     -2.225990990150   -3.935070871578    0.672654013296
H     -2.981713347791   -3.555434288274    0.180071346533
```

```
H     -2.585093801271   -4.670917470767    1.169915803219

Cope TS Water5-17
35
ANGSTROM
C     -0.482093468787    0.244706846210    1.084817014776
C      0.949936540516    0.323922013733    0.734921352863
C      1.602099606537   -0.686376929441    0.131690671124
C      0.847323409020   -1.929890820316   -0.327165129993
C     -0.359322127550   -1.487098824512   -1.286774171428
C     -1.494059276371   -0.751607554440   -0.572140377978
C     -1.640005519306    0.695235087568   -0.597614344343
O     -2.684195958793    1.338426639725   -0.267882001697
H      1.458484843351    1.264307631214    0.945702141758
H     -0.890286734198    1.119414625225    1.583486862382
H      2.651329447901   -0.607596891215   -0.137666213360
H      1.501985569745   -2.617045962722   -0.873837606125
H      0.410275395686   -2.475724293235    0.520539801479
H      0.054732990911   -0.867325253581   -2.092128586955
H     -0.779752346949   -2.393889926349   -1.739382082330
H     -0.777826799346   -0.659761845537    1.614552613109
H     -0.865058121005    1.254995109044   -1.157551739477
H     -3.275596545578   -1.100259411903    0.373157338978
N     -2.498671797416   -1.540636489132   -0.109511624873
H     -2.273908619648   -2.456265449859    0.260374991862
O     -3.318494729559    2.815842840112    1.903059077494
H     -2.975344036320    2.468861260174    1.055569633595
H     -4.220568339519    2.487615111147    1.952573233977
O     -4.675657554963    0.190808153321    0.976765457156
H     -3.956802092823    0.678563711929    0.482169472537
H     -5.435630216933    0.166603826936    0.395297975076
O     -3.292639448933   -1.013792717050    3.138706795923
H     -3.911100308995   -0.656586964332    2.486365336805
H     -2.787288679019   -0.241510254178    3.455718195563
O     -2.098525316824    1.365125934784    3.815083755330
H     -2.500320143650    1.962492339231    3.144063707374
H     -2.263413885153    1.767672620052    4.668182139201
O     -1.673026107710   -2.948505425059    2.176948077694
H     -1.620766805049   -3.647902293518    2.828873382823
H     -2.266821649807   -2.268043885593    2.569064654066

Cope TS Water5-18
35
ANGSTROM
C     -0.132832594150    1.554597352786    0.715590447811
C      1.187873549689    1.577785634303    0.075857127110
C      1.923011713045    0.471597076433   -0.162364110238
C      1.378634118706   -0.919356208031    0.134994787667
C     -0.004390900149   -1.142741761189   -0.663936011226
C     -1.166016518285   -0.322544228262   -0.131278379618
C     -1.576073975493    0.990506362503   -0.646851096199
O     -2.677654986583    1.534672331550   -0.358961381705
H      1.543741887058    2.546290211883   -0.277170340760
H     -0.611818129160    2.521918383896    0.841289577200
H      2.877494749088    0.530050412047   -0.676744153689
H      2.085996823840   -1.697424464795   -0.172023548531
```

```
H      1.166211546230   -1.057267487423    1.204381542016
H      0.179886646808   -0.914487549951   -1.720598852477
H     -0.264191742497   -2.206936139480   -0.584613839022
H     -0.226276842275    0.957382558000    1.620279301624
H     -1.047014788666    1.335352531522   -1.561159028216
H     -2.722562485793   -0.357181978704    1.171521871456
N     -1.930841068631   -0.881772656516    0.818728604638
H     -1.694422058344   -1.718168360696    1.330527066106
O     -3.698783077914    1.426643911264    2.066421766769
H     -3.376649652202    1.593067101584    1.139910948068
H     -4.347438536328    0.711782086773    1.996725318154
O     -4.867354374471   -1.126329221437    1.691496500474
H     -4.266805953315   -1.876339115065    1.861202747215
H     -5.197356001203   -1.233224476956    0.798361048356
O     -3.101768927236   -1.396496519470    4.477693584964
H     -3.981999182073   -1.029090613038    4.361609805641
H     -2.481962911098   -0.673961783582    4.223996030801
O     -1.688697303198    0.690219381126    3.587112787536
H     -2.424308903096    1.032264387338    3.008877096126
H     -1.505725847909    1.388394550360    4.216677505393
O     -2.996334892910   -3.042788675694    2.415879846900
H     -3.234291051630   -3.929358575012    2.689561018069
H     -2.940341633897   -2.506694513573    3.248280043015

Cope TS Water5-19
35
ANGSTROM
C     -1.021546072792   -0.350930141998    1.487078254151
C      0.453842172952   -0.438254848830    1.535484252012
C      1.134836538310   -1.505506049568    1.084552353986
C      0.404905530485   -2.648038832366    0.381801998036
C     -0.460241573015   -2.052925689740   -0.827390255579
C     -1.660426483706   -1.203459494585   -0.396514340885
C     -1.649116188130    0.249331691964   -0.413446579182
O     -2.677761675383    0.995373319434   -0.344473955901
H      0.976015901694    0.434127375645    1.925485116474
H     -1.424584906250    0.576098456272    1.885254712139
H      2.219553562710   -1.546358021962    1.113713808939
H      1.107564742415   -3.389396001893   -0.014077739209
H     -0.284037100694   -3.165004673636    1.064714147281
H      0.211163858690   -1.467664188983   -1.468242617627
H     -0.842068348470   -2.894279127820   -1.419852095614
H     -1.557039061109   -1.206403455356    1.899886899023
H     -0.702970824112    0.731551453407   -0.725921042924
H     -3.674070095299   -1.360336573703   -0.030865232527
N     -2.832070133940   -1.879738674804   -0.255550683918
H     -2.831283568837   -2.814261868729    0.125474239136
O     -3.508754556172    2.109103987248    1.912567386955
H     -3.165267094373    1.775454935725    1.049745546260
H     -4.112322348287    2.821028561054    1.695196536628
O     -5.119011047242   -0.002838793399   -0.260931014598
H     -4.243706247494    0.458836178545   -0.345174834805
H     -5.597792119483    0.170026384193   -1.071143828320
O     -4.702686528355   -0.389516765612    2.453058816819
H     -5.123612984421   -0.368087071619    1.581755333516
H     -4.223919458024    0.452725424874    2.506164699104
```

```
O     -1.228448018320    2.588276887999    3.458210134575
H     -1.486538492192    2.461989354051    4.372247725583
H     -2.041797490983    2.504326069937    2.934323500974
O     -3.206940812873   -2.664231790539    2.458904904784
H     -3.449856258714   -3.166468774444    3.237281220634
H     -3.759486928610   -1.853134472469    2.489924189686

Ambimodal TS Water15-1
65
ANGSTROM
C      0.168240805667    1.740804213712    0.764555576531
C      1.278704238247    1.604569125449   -0.012380132080
C      2.019899224243    0.381704593279   -0.225371393293
C      1.693557184741   -0.838031299278    0.314528436179
C     -0.201438141360   -1.376901268683   -0.845866008573
C     -1.189966050537   -0.618889595167   -0.231430184288
C     -1.537976931368    0.705452981931   -0.747403780861
O     -2.562342554281    1.352877664554   -0.400327895289
H      1.580009432900    2.449750379107   -0.630933505718
H     -0.355850573423    2.686037326388    0.822352784129
H      2.776614938384    0.415533394402   -1.008576303664
H      2.271964351576   -1.720028713533    0.067740250394
H      1.115919018398   -0.925842031786    1.226193278998
H      0.183922937033   -1.080850182090   -1.809174939731
H     -0.092132646269   -2.426061096462   -0.604191905818
H     -0.126436767894    1.013936155119    1.509648095102
H     -0.986228646101    1.051195531629   -1.631200382162
H     -2.614393469262   -0.520613174033    1.272774616341
N     -1.807731945620   -1.030398106979    0.930879749233
H     -1.805541647879   -2.006980232856    1.176603525840
O     -4.678554668612    4.869631519218   -0.348515506099
H     -5.088940193389    4.167702803228    0.240781427591
H     -5.339784163576    5.552499746806   -0.458202273080
O     -1.045531831066    4.358650937138   -1.988841352643
H     -1.927804803523    4.029613831267   -2.255860127299
H     -1.116131196340    4.480492475385   -1.032780297550
O      0.350447682617    2.382149000444   -3.164317718025
H      0.847538323537    2.796071793652   -3.871123102289
H     -0.120064380620    3.113775600728   -2.704507514271
O     -2.926884912645   -1.238757744836   -2.972976991348
H     -2.232403435801   -0.650389509231   -3.353454105103
H     -3.088559450455   -1.896826017981   -3.651028761768
O     -3.823167322677    1.458341814350   -4.265076494652
H     -4.117169703104    0.685281540211   -3.773598314179
H     -3.771332223736    2.181171047978   -3.616388887033
O     -4.094595779005   -0.035553898635    2.456323042682
H     -3.750622533866    0.678716724232    2.997736608656
H     -4.069662112152   -0.867986969084    2.970938902097
O     -5.138576662487    0.502582606310    0.044528684508
H     -4.273669955395    0.812358000983   -0.274924717755
H     -4.964452694734    0.243088998107    0.968364348631
O     -3.378794061335   -3.517392914349    1.011913645576
H     -3.808747170217   -2.938029246640    0.330716257704
H     -3.624034351644   -4.413862947397    0.780870392748
O     -4.529624752331   -2.031127289496   -0.850721096774
H     -3.922461594958   -1.790413369822   -1.568051198541
```

```
H      -4.926451432975   -1.188772645751   -0.560200947077
O      -1.293143357011    0.432697194884   -4.260189428114
H      -2.093063195987    0.936724224498   -4.504488515740
H      -0.678028526661    1.079494636345   -3.872218140931
O      -4.062474369840   -2.529832044890    3.443315145866
H      -3.751698777529   -2.925170233895    2.600509942637
H      -4.941219443659   -2.880779934573    3.596033344453
O      -3.014409658018    2.590122708003    2.021999549113
H      -2.611467935058    3.439950555819    1.793505135803
H      -2.834307366695    2.029727907820    1.246842914376
O      -5.567987614026    2.986894286288    1.196182147237
H      -4.737515013755    2.840047196783    1.696830376997
H      -5.697718675525    2.164604861167    0.690880869067
O      -3.479460735360    3.195136383814   -2.160924200317
H      -3.165208935825    2.532962064874   -1.510420797075
H      -4.028785618306    3.816491224090   -1.656625749550
O      -2.126133304851    4.874607789869    0.636526790081
H      -3.017718951384    4.919369336617    0.233374385685
H      -1.926624978071    5.757255041781    0.950553626429

Ambimodal TS Water15-2
65
ANGSTROM
C       0.299919969196    1.626711504549    0.747027525215
C       1.353952128143    1.438435787109   -0.089169647760
C       2.052453286548    0.183572713441   -0.296325550915
C       1.683815081137   -1.010351730793    0.289263833562
C      -0.225966720062   -1.528193318403   -0.756692308052
C      -1.193583910981   -0.713416093383   -0.181172547721
C      -1.550295779426    0.594160115347   -0.757211956077
O      -2.524566142119    1.272634914280   -0.387495426872
H       1.648390728265    2.254697142324   -0.750925874114
H      -0.219476524959    2.574281143353    0.800133554933
H       2.751342637759    0.159557871720   -1.131380390749
H       2.221486810870   -1.919214605239    0.047495621948
H       1.162994959070   -1.038102800166    1.238550398325
H       0.149698186335   -1.300824419811   -1.742400656050
H      -0.149162600239   -2.564443922057   -0.453612939314
H       0.027875871872    0.915115354278    1.514195677887
H      -0.998689570080    0.902981586892   -1.660742094533
H      -2.461283389566   -0.402970352081    1.417371123950
N      -1.881849563986   -1.080075697461    0.949693435675
H      -1.607703246866   -1.873840585244    1.506832780704
O       0.666507865384    0.237872741610   -3.540088018713
H      -0.283189802200    0.125212076661   -3.727329247864
H       0.964576646607    0.957315105856   -4.098192052727
O      -5.654214352127   -1.037552850282   -3.280172670201
H      -5.262850796338   -0.145777007247   -3.511323624525
H      -6.354481597905   -1.195355091868   -3.912967045770
O      -3.799336791779   -3.227431693419   -0.432014708933
H      -4.153092562808   -4.078297836592   -0.696945972566
H      -4.581957115420   -2.596971819957   -0.448386588839
O       3.035907379661   -0.861725516272    4.078601059486
H       3.379539636853   -1.756133970490    4.057150332905
H       3.521712950585   -0.362581430771    3.394450524226
O      -2.022120197490   -0.111793859196   -4.104431856997
```

```
H     -2.066305880359   -0.392819529761   -5.020844818503
H     -2.353098040773   -0.885014607696   -3.581916168759
O     -3.166216258378   -2.178635425087   -2.928158768364
H     -4.087194946304   -1.899366657173   -3.078676087992
H     -3.139647207045   -2.497524250657   -2.010728789325
O     -4.619402776161    2.577810557476   -1.560016033990
H     -5.271331154793    2.218634656212   -0.951290700480
H     -3.758819802635    2.317839497848   -1.188184319535
O     -4.916977005593    0.544852755979    0.780490226658
H     -4.055878980347    0.795032741564    0.399536503689
H     -4.713852418567    0.135475669376    1.640104947208
O     -1.560993577573   -2.279628267317    3.552683792694
H     -2.303464849284   -1.697068969579    3.751321420115
H     -0.772944797824   -1.709904978809    3.593531230021
O      0.422479506622   -0.372991321877    3.602851055960
H      0.181491294192    0.286061011791    4.255993899304
H      1.355714660865   -0.603209266086    3.791446706040
O     -4.200551330764   -0.842210592999    3.075341910149
H     -4.943266175042   -0.805133372134    3.679165654331
H     -4.177283005285   -1.775952078798    2.736183734405
O     -3.875917900565   -3.347401078904    2.287180647783
H     -2.996919874152   -3.450229753012    2.669682874151
H     -3.760739449599   -3.313213896737    1.315541823152
O     -4.489758192410    1.210234656176   -3.883678439771
H     -3.554268574875    0.951223678144   -3.922120607082
H     -4.576888076896    1.805547271354   -3.108936261889
O      4.178199641211    0.528827964209    2.016082843430
H      4.293642407605    1.473876863266    2.132608749600
H      3.564080816487    0.404408338490    1.276445547604
O     -5.808690624171   -1.592826212437   -0.582319605959
H     -5.567280783202   -0.778172835672   -0.089313685515
H     -5.858780914928   -1.338990100730   -1.518415742441

Ambimodal TS Water15-3
65
ANGSTROM
C      0.231840258208    1.643432458422    0.851221502901
C      1.326487443025    1.538712870139    0.050408911749
C      2.048900451156    0.317299815277   -0.233633612000
C      1.704996611362   -0.921568747175    0.255274092464
C     -0.191343088124   -1.407964236565   -0.865105226327
C     -1.177420144532   -0.637524226338   -0.263269502710
C     -1.530586623083    0.696023448753   -0.763502655124
O     -2.536167291208    1.336427931885   -0.398997842875
H      1.638387445958    2.415735097660   -0.521005731110
H     -0.293014083386    2.582672488888    0.969479516122
H      2.794542313074    0.377684985719   -1.027184750082
H      2.269778138506   -1.797590807702   -0.040674281564
H      1.154301016320   -1.033195313659    1.179978024198
H      0.189704259193   -1.123276279594   -1.833863956039
H     -0.110109450551   -2.461350803475   -0.633440405956
H     -0.061098065070    0.867384453875    1.543008491055
H     -0.958175280783    1.068623711816   -1.634580561386
H     -2.580835377212   -0.484244874343    1.232270107170
N     -1.831057247045   -1.051585904322    0.876658194471
H     -1.807591261136   -2.018888272913    1.166117021987
```

```
O     -5.417479632550   -0.275096425580    2.620079791379
H     -5.460266462989   -0.033910009545    1.679360193754
H     -4.855511375100    0.405913336770    3.034946643378
O     -4.705582923522   -2.389336138532    0.035063156687
H     -4.509766541791   -2.640590839386    0.944906294393
H     -5.012996898834   -1.467437933491    0.067591143530
O     -5.063296551034    0.386296830314   -0.020997648219
H     -4.119928178735    0.627607524862   -0.117277038257
H     -5.382657713137    0.343331006883   -0.947112539867
O      0.403996972308   -0.834323421760    3.412561350988
H      1.139027076240   -0.475447771205    3.911335685967
H     -0.400172388454   -0.349156061676    3.741089322505
O     -4.504016722790   -2.687496593369    2.919712254056
H     -4.848211705036   -1.750336584326    2.833538569009
H     -5.251078631168   -3.209564735627    3.215685771923
O     -2.097477627821   -3.765202651023    1.939498212388
H     -1.466423825603   -3.681629014567    2.677906196753
H     -2.963406805520   -3.520578460986    2.292882667758
O     -5.280234560816    0.167623438617   -2.686149843370
H     -4.568156136935   -0.509173043988   -2.630429788283
H     -4.895082065133    0.910565520357   -3.152869926163
O     -2.184637250980   -3.921368687098   -2.899734322756
H     -1.736425311954   -4.141452349952   -2.062773994530
H     -2.943698749133   -4.504647410630   -2.957827531562
O     -4.155714930095    2.962388270403    1.074733410999
H     -4.935422802418    2.508648771412    0.733489341722
H     -3.426257372252    2.640745943161    0.520156160687
O     -3.797541178230    1.765075017227    3.401855264874
H     -4.063173661966    2.371702365880    4.094031964501
H     -3.860521927890    2.269525961690    2.549677249591
O     -2.763961472090   -2.132979685504    5.052269920714
H     -3.389856335524   -2.338407845328    4.339365636467
H     -1.968673736553   -2.652115990111    4.868192176471
O     -3.271868040278   -1.522957854473   -2.150506118714
H     -2.825410423584   -2.255276584739   -2.609995845517
H     -3.699298277072   -1.940808780534   -1.381586699887
O     -1.170669470758   -4.720387829860   -0.451813481250
H     -1.099483808085   -5.663740512927   -0.301772703793
H     -1.561142784259   -4.344895954102    0.357666227319
O     -1.696567121993    0.290785592325    4.485663472099
H     -2.154955305891   -0.510861478369    4.814438205173
H     -2.368033215957    0.816075436078    4.025678774550
O     -0.404593982899   -3.279888688067    4.012094879625
H     -0.069021305600   -2.383894535900    3.748883138521
H      0.360879690329   -3.805111234274    4.245282115125

Ambimodal TS Water15-4
65
ANGSTROM
C      0.119991342729    1.750176209990    0.792161912550
C      1.289587435172    1.623785686791    0.098777178239
C      2.064451293925    0.415765716545   -0.022732176050
C      1.674260081069   -0.807237500104    0.498096778273
C     -0.038660499540   -1.380873031216   -0.731428456009
C     -1.121252070147   -0.648203032648   -0.231368666739
C     -1.496941548892    0.665651038470   -0.791161328376
```

9743	O	-2.539618829801	1.281676217277	-0.511050963134
9744	H	1.622588091653	2.464962549993	-0.512575829976
9745	H	-0.439508716622	2.676042113087	0.782122586682
9746	H	2.885884232517	0.436571155603	-0.738772327590
9747	H	2.290930542267	-1.683402394911	0.334395933795
9748	H	1.060848974498	-0.863223288221	1.388527680363
9749	H	0.399575359384	-1.105967735942	-1.680311528383
9750	H	0.058447579636	-2.427188461032	-0.465496721202
9751	H	-0.194764684620	1.038832224121	1.541456141077
9752	H	-0.900543568313	1.005081863354	-1.661441701807
9753	H	-2.622628925112	-0.568423582333	1.182946418387
9754	N	-1.834547366942	-1.102028027597	0.845933245923
9755	H	-1.780414950515	-2.068420040176	1.137701927999
9756	O	-2.217504407212	-2.966589838709	-2.840079166876
9757	H	-2.756875095624	-3.189077993193	-2.055935795040
9758	H	-1.330632123371	-2.796325320224	-2.516289666661
9759	O	-2.028861040478	-3.959238834369	1.519980667879
9760	H	-1.232916631200	-3.801204139552	2.060476987741
9761	H	-2.757209366757	-4.046911949408	2.181460550030
9762	O	-0.434596383635	-0.110225801657	3.535291060913
9763	H	-1.370178510230	0.140463557946	3.577041395118
9764	H	-0.218909054819	-0.433037815834	4.431312318015
9765	O	0.538813238738	-1.482582194492	5.701359773273
9766	H	0.612558338424	-2.265844566598	5.138031272139
9767	H	1.378359688265	-1.016840382813	5.603326941954
9768	O	-3.150432382594	0.143202341380	4.014507345051
9769	H	-3.559037425856	0.268867139545	3.124901248738
9770	H	-3.664402912656	0.689712432183	4.609888829806
9771	O	-3.933879705425	-3.920735505019	3.373324299380
9772	H	-3.489455658862	-3.517800952140	4.129463538874
9773	H	-4.642885633360	-3.296601025102	3.127815184899
9774	O	-3.367156903017	-0.733380219098	-3.845958161154
9775	H	-2.940223484506	-1.551791174542	-3.517500209656
9776	H	-3.801746280983	-0.968268516489	-4.666685999007
9777	O	-4.130053021575	0.628998255079	1.608571588988
9778	H	-4.679826942041	-0.032430104321	1.149414298419
9779	H	-3.637753495268	1.095327806645	0.906067941997
9780	O	-5.219016129919	-1.378900833119	0.085935634818
9781	H	-5.229324843619	-0.749548141174	-0.674989816093
9782	H	-4.621245948417	-2.096824926399	-0.181790896256
9783	O	-5.858924313684	-2.121332310168	2.637623482317
9784	H	-6.784493789475	-2.363063447011	2.675220281380
9785	H	-5.686913237027	-1.834412369490	1.723153821770
9786	O	-3.601123079451	-3.551322063319	-0.572770566331
9787	H	-4.150794120660	-4.336039000517	-0.605729646056
9788	H	-2.961495436124	-3.701232385700	0.158274708478
9789	O	-0.331440490846	-2.881322492969	3.326911611282
9790	H	-0.258042697991	-1.946881080056	3.068522784106
9791	H	-1.086113341001	-2.890728387607	3.946202153340
9792	O	-4.852804691938	0.400857400614	-1.848322492849
9793	H	-4.192515397444	0.975491953109	-1.437112921973
9794	H	-4.395904690293	0.011615345889	-2.617455078820
9795	O	-2.366105678388	-2.269193011615	5.063383444036
9796	H	-1.798529523084	-2.016248917937	5.796809691075
9797	H	-2.715903832142	-1.440214259910	4.681196570440
9798	O	2.354661189725	0.053088508974	4.132366055649

```
H      3.056266649681    0.340386463692    3.547790471319
H      1.521763782191    0.258141751596    3.686724511265

Ambimodal TS Water15-5
65
ANGSTROM
C      0.167656436987    1.149916720106    1.428296533746
C      1.437210503077    0.940276000611    0.977700858192
C      2.034312681663   -0.345407366999    0.726798322418
C      1.364203985184   -1.549824298222    0.862186662701
C     -0.075053164426   -1.539063698609   -0.756068937148
C     -1.116399937331   -0.688971756606   -0.374952072025
C     -1.111941318745    0.735292148678   -0.745241386324
O     -2.056922137919    1.526475905984   -0.582169301268
H      2.025362836080    1.804458351778    0.666093285644
H     -0.241657439993    2.146924779893    1.524920330811
H      2.988159785370   -0.342180103754    0.201199836282
H      1.874240100053   -2.475212297321    0.618918321994
H      0.569692603191   -1.666820074266    1.589886839565
H      0.610130062684   -1.217030442030   -1.526420022012
H     -0.222699872291   -2.611665363097   -0.698527814180
H     -0.434565693534    0.376257954060    1.882377807749
H     -0.250649563812    1.055274254079   -1.363154312345
H     -3.001299830657   -0.591385984926    0.471765594378
N     -2.138744817542   -1.132043057648    0.448253345156
H     -2.267152652072   -2.124182724214    0.562330725832
O     -3.699675668667   -0.347551025867   -2.893958192763
H     -3.862502203275    0.567695178150   -3.217134821283
H     -4.229844321082   -0.923576147808   -3.447316719955
O     -4.180058630685    2.148879881328   -3.794567692674
H     -5.086487579788    2.455733364233   -3.823699278939
H     -3.711304627485    2.740512869492   -3.127137028980
O      1.218167570342   -0.695730538141   -3.650671436700
H      1.536473564105   -1.519201219043   -4.024058244891
H      1.731712940963    0.008518540300   -4.113951472972
O     -0.640921880631    3.437440941276   -7.244001689596
H     -1.154092133383    2.859491853308   -6.651215659770
H      0.212845875992    2.984296888821   -7.366692574576
O      1.260017050974    3.161973501312   -1.883780329086
H      1.056049024306    3.754046212754   -1.158960469888
H      0.538264367176    3.286381202489   -2.543997811307
O     -0.632409207405    3.720051564561   -3.656632843069
H     -0.348145984210    4.426444149241   -4.262742531552
H     -0.983712625671    3.007364065617   -4.241325674569
O      0.085566415499    5.396023581885   -5.683647611501
H     -0.300848449724    6.221559306529   -5.974873296349
H     -0.219872394148    4.695825450157   -6.334586677627
O     -2.864940915253    3.613393402650   -2.108780644984
H     -2.060034871349    3.821469788050   -2.619070729119
H     -2.581680838127    2.944380808046   -1.454553781638
O      2.693970937032    1.046240356672   -5.011839377005
H      2.319945815623    1.398976709512   -5.835973452759
H      2.951523512023    1.824318382847   -4.490019562312
O     -4.510391724898    0.307790016170   -0.260409872418
H     -4.369974570084   -0.041688316355   -1.153454087975
H     -3.900590196726    1.059133253694   -0.204801287256
```

```
O     -1.360401606206   -0.498906558587   -4.461904142723
H     -0.454339354148   -0.558680664006   -4.096706199791
H     -1.978147553830   -0.552667257973   -3.717837720305
O      3.335308681916    3.318365305254   -3.528490831531
H      2.586194593126    3.264391874516   -2.888766491871
H      4.141238650799    3.228760725314   -3.019652527451
O      2.713008859938    4.604171313783   -5.926699182005
H      1.847436307006    5.021352669235   -5.769286961298
H      2.998561406170    4.246625455702   -5.071416049099
O     -1.826743304741    1.966837309082   -5.265997669073
H     -1.588052190274    1.026586651374   -5.071199670555
H     -2.740473873564    2.064948742131   -4.959015094909
O      1.935623409537    2.479282875548   -7.266639287108
H      2.447964437266    2.380884960111   -8.069157309987
H      2.277773183498    3.298979272754   -6.813649607083

Ambimodal TS Water15-6
65
ANGSTROM
C      0.237697413474    1.708242858717    0.841821170290
C      1.337669781973    1.549248909882    0.060601571508
C      2.028957408469    0.301436153372   -0.204282225479
C      1.655972822502   -0.926907235704    0.281454436363
C     -0.265765568129   -1.371693483755   -0.856440300309
C     -1.218600950209   -0.589533804551   -0.216425692738
C     -1.571636986835    0.733189364384   -0.745113674900
O     -2.558007690998    1.412436510837   -0.385257145439
H      1.689372550594    2.408498281633   -0.513702348240
H     -0.256865579069    2.666198169143    0.932652782093
H      2.788238974029    0.340843072532   -0.985272378167
H      2.200957376376   -1.816233163209   -0.008646091914
H      1.074937635293   -1.040357824512    1.187007481487
H      0.092397572065   -1.081425089420   -1.832227365000
H     -0.172386266200   -2.423292621563   -0.623086776783
H     -0.116258126621    0.954891886449    1.530367400587
H     -1.012839247300    1.069308112645   -1.635477240975
H     -2.518880633895   -0.419906664471    1.373430349910
N     -1.794966242274   -0.987168653426    0.970671988882
H     -1.808452445438   -1.969833942922    1.239427714946
O     -1.204936759699   -4.647730469148   -0.648354423325
H     -1.512495908079   -5.537334362298   -0.826472978108
H     -1.795357007826   -4.054740061574   -1.178225633818
O     -3.927142079451    1.413549110357    3.697180474963
H     -4.360778846630    0.686253274491    4.160510955133
H     -4.162221358187    1.289867895231    2.760186321898
O     -4.542037897541    0.575170995733    1.137972338257
H     -5.307811980394    0.994859326414    0.734808787600
H     -3.770474331583    0.905706774812    0.605761234287
O     -2.292302857457   -1.522590638101    4.236933452420
H     -1.622171683887   -2.147238300097    3.925958221369
H     -1.868399838520   -0.652822878744    4.256238006421
O      0.098438299835   -2.908485191250    3.592479026657
H      0.494931780312   -2.016454054584    3.580868542789
H      0.449847541417   -3.349252528017    4.367008848167
O     -4.840049491666   -1.372929972489    3.342243404162
H     -4.771628078860   -0.823914611386    2.552945980335
```

```
H     -3.932932993841   -1.499808304345    3.685378604289
O     -2.618585792202   -0.670061310458   -3.268861547932
H     -2.600208933389   -0.839573828385   -4.211921176370
H     -3.572268158248   -0.528468410467   -3.047013349143
O     -5.054001273047    2.072148018744   -1.295830736554
H     -5.180025209137    2.801657581277   -1.903307446027
H     -4.097501387157    2.012075073617   -1.123114330176
O      0.961094303181   -0.323105010581    3.735993148372
H      0.173776032537    0.230122518254    3.874977644307
H      1.570116382235    0.192995763737    3.205325220720
O     -4.690031047316   -3.633683144581    1.765834214094
H     -3.734240775079   -3.784151244626    1.835740795490
H     -4.909546994123   -3.003504383727    2.475215648473
O     -2.889338186565   -3.076092221911   -1.943760220331
H     -2.613524586654   -2.279464421285   -2.429247545964
H     -3.583636291410   -2.769075860288   -1.334612212055
O     -5.175068351615   -0.414694201843   -2.558086190959
H     -5.282993153030    0.443271841065   -2.116035068524
H     -5.220451547207   -1.084615579938   -1.857447186989
O     -4.771040377059   -1.930576618976   -0.265106791221
H     -4.855132066744   -2.654830521643    0.402446683120
H     -4.647448619937   -1.121844000497    0.250604604449
O     -1.341019166945    1.142129359740    4.228934718223
H     -2.270706434774    1.332056040849    3.948274795896
H     -1.243122234419    1.566210066480    5.082917858451
O     -1.890037584631   -3.690481281442    1.752225470557
H     -1.595629033752   -4.113220370822    0.915307314192
H     -1.133048242036   -3.686187140363    2.349612008505

Ambimodal TS Water15-7
65
ANGSTROM
C      0.143563035060    1.737501988760    0.807673719796
C      1.263818981829    1.610031577993    0.046647166640
C      2.031041718031    0.394957722724   -0.147667036574
C      1.725963644223   -0.822446670899    0.397160003492
C     -0.188349296124   -1.390615215573   -0.826162338916
C     -1.173370387717   -0.635627260048   -0.212304830995
C     -1.539215473367    0.688805562556   -0.722798217772
O     -2.570439016244    1.312130623224   -0.352362295696
H      1.566371946575    2.458425722289   -0.569673823817
H     -0.396430133659    2.674700455392    0.854027714208
H      2.807117448383    0.441580684525   -0.912703223973
H      2.316599816318   -1.698601044179    0.160840598347
H      1.109454644819   -0.923908507820    1.280321329012
H      0.217199079193   -1.085783629695   -1.778512813833
H     -0.066599682879   -2.434218548400   -0.573818221270
H     -0.150515815804    1.003660767452    1.546114618988
H     -1.014214770944    1.054197485426   -1.618530991142
H     -2.587017635138   -0.537504551377    1.275319714292
N     -1.784311166581   -1.053195230943    0.952751051622
H     -1.810499830579   -2.050168539341    1.165100968689
O     -1.064096971617    4.895573321192    0.436363990932
H     -0.416391565856    5.485132257628    0.823792839820
H     -0.975159726225    4.978873229906   -0.532902823382
O     -2.473362460914   -3.755191459479    1.062392626381
```

```
H     -2.571993370288   -3.691405536554    0.094493951078
H     -3.341374987630   -3.517995709456    1.419231246270
O     -4.968993698660   -2.656463803070    1.904237471171
H     -4.936388883739   -1.794480620366    2.377695458484
H     -5.526472783333   -3.228733996283    2.432582476875
O     -3.405127738342    1.757371900005    2.211131995326
H     -3.267777906149    2.720998191889    2.365400570555
H     -3.007030000951    1.593429853996    1.336784569905
O     -3.243392079486    4.404153547231    2.082812347999
H     -2.474118199957    4.587354599925    1.520380481895
H     -4.021197273627    4.542626288141    1.518911953414
O     -2.606197062097    4.067511420181   -3.593067360488
H     -2.084083030290    3.277721308974   -3.782377470437
H     -3.295669004806    3.777203997065   -2.956359717424
O     -0.126694876271    2.595339611433   -3.259483657278
H     -0.097562464606    3.411060702104   -2.739171891168
H      0.545647398483    2.678370991872   -3.937150936910
O     -5.973113393563    1.917739613979    1.423525645178
H     -5.067825947465    1.898665460217    1.811307466296
H     -5.904412904810    1.362351721362    0.624657298084
O     -5.374623450689   -2.209580767597   -0.806235089932
H     -5.405056918834   -1.243836889849   -0.882102424061
H     -5.308002690517   -2.398773567982    0.145317750909
O     -3.026060537290   -3.216909697771   -1.588575429145
H     -3.902075001555   -2.816569079450   -1.374896758007
H     -3.208453351866   -3.932504493098   -2.198264291883
O     -4.194771968380    3.099120184325   -1.669968693795
H     -3.494330730295    2.622364041536   -1.187643742432
H     -4.624213397280    3.672204347374   -1.013473741073
O     -5.041018086092   -0.286394931839    3.150089078155
H     -5.802258234584    0.226151741577    2.853414355610
H     -4.284875201974    0.315050764996    3.065124198247
O     -5.509957563672    4.324810381460    0.433422880651
H     -5.797241397394    3.459261282680    0.829696607781
H     -6.267984951223    4.907408309712    0.470750982645
O     -0.693299710962    5.232640902423   -2.245008306939
H     -1.487625935039    4.881564212516   -2.738702370601
H     -0.572062425755    6.132000255370   -2.551360780500
O     -5.160647503590    0.576736460579   -0.811959068593
H     -5.292514934950    1.232779666307   -1.507590690962
H     -4.218765128323    0.665462349242   -0.577893395584

Ambimodal TS Water15-8
65
ANGSTROM
C      0.163823217339    1.737484891490    0.806500941360
C      1.283347687090    1.617483428460    0.039198676920
C      2.042686206288    0.400372823160   -0.166054550370
C      1.727296615348   -0.824789704133    0.370330038601
C     -0.159000472079   -1.415090489570   -0.776762554881
C     -1.155417352072   -0.655336513051   -0.177557853002
C     -1.527855169562    0.659034243753   -0.705830814045
O     -2.565436086086    1.287032280736   -0.380099871983
H      1.594093145749    2.476851001711   -0.557746341084
H     -0.380762230765    2.671579113224    0.856115050912
H      2.823544406044    0.445957052499   -0.927449871359
```

```
H       2.323748687811   -1.696692492065    0.131050947529
H       1.144026813062   -0.920181212141    1.276754305600
H       0.212898449961   -1.142347158475   -1.752198443062
H      -0.045973262994   -2.456938732043   -0.508793783256
H      -0.127351535625    1.002242144149    1.544711592782
H      -0.982030717750    1.009032676453   -1.595776193308
H      -2.543393269561   -0.524253414258    1.326842336007
N      -1.761987418077   -1.065270084962    0.991878225914
H      -1.831127190349   -2.062484974973    1.184723597175
O      -4.731262659409   -1.883742520087   -1.611546532748
H      -4.932715786883   -2.041011933055   -0.676399180866
H      -3.991792937959   -2.480380910776   -1.817273722326
O      -2.159414614762    3.904800730914   -1.301221061617
H      -2.710545740499    4.027846994817   -2.096790614226
H      -2.423827909033    3.054702711511   -0.918411761907
O      -1.310421283371   -0.344183198417   -3.858296340324
H      -0.535704569665    0.236941084787   -3.922980869602
H      -2.081730169757    0.188364974087   -4.118675120842
O      -5.035038130564   -2.113675503674    1.153363117415
H      -5.806627761805   -2.321917906880    1.680859704917
H      -4.915545514363   -1.137661109751    1.207200256798
O      -2.762771199945   -3.797100246586   -1.937696775753
H      -3.183989372892   -4.579505700556   -2.297604731925
H      -2.129758013253   -3.490913164216   -2.629014497292
O      -2.674546186154   -3.706146431163    0.889142811778
H      -2.630165561642   -3.816291832836   -0.072475789711
H      -3.546430115838   -3.324825353433    1.064110520753
O       2.508451475235    4.123077015608   -2.140359613576
H       2.863282313677    3.605120107835   -2.892618932853
H       3.032658588154    4.922261581132   -2.085446805747
O       0.887466788773    1.308239372260   -3.502023762437
H       1.242677263972    1.046098130090   -2.648800392679
H       0.444467242805    2.191799406582   -3.344596679099
O       3.109025523406    2.633304922048   -4.343132452277
H       2.939921137944    3.066638648251   -5.181122258568
H       2.351154162313    2.031835082710   -4.190819615716
O      -4.786652015129    0.554902472222    1.027839819347
H      -3.946518103824    0.882324814953    0.648583451364
H      -5.463868439721    0.839495117947    0.407584465818
O      -2.924860999362    3.866036546045   -3.928560958016
H      -3.146618928616    2.944217361906   -4.144991874853
H      -1.983274294194    3.960694711137   -4.103604076480
O      -1.128000028206   -3.003169703157   -3.878536421971
H      -1.260933406513   -2.031755219635   -3.935736448223
H      -0.184357763330   -3.136400328975   -3.778979799054
O      -4.713116550455    0.761534040337   -2.085888939849
H      -3.992688146254    1.052426453237   -1.508448373943
H      -4.766225111574   -0.213761667285   -1.981297056933
O      -3.533183918782    1.191480573938   -4.392032853186
H      -4.036028760251    1.043331461439   -3.544748035293
H      -4.140202057625    0.977281965290   -5.101069144069
O      -0.080803189925    3.665887057444   -2.964827603227
H      -0.768916592891    3.769889501257   -2.264971882409
H       0.736830967007    4.037749229843   -2.595387080597

Ambimodal TS Water15-9
```

```
65
ANGSTROM
C     -0.043337659291    2.118445492160    0.542531370101
C      1.160784608821    1.937654826395   -0.075884212457
C      1.943961429327    0.733885813944   -0.033062299272
C      1.535575786684   -0.437434911640    0.578908266982
C     -0.081965843899   -1.071630388240   -0.754511959507
C     -1.211584804053   -0.416755816779   -0.268989418435
C     -1.677219070908    0.865880132874   -0.802406640278
O     -2.724563770767    1.438701399342   -0.414751335221
H      1.521504492269    2.717787006290   -0.744653945682
H     -0.596469358744    3.040353540301    0.423515810037
H      2.814860463232    0.694376002302   -0.681589427185
H      2.168136150737   -1.316748667831    0.530084036859
H      0.866591657949   -0.429808185950    1.432485165353
H      0.359291392194   -0.738456024250   -1.682632345478
H      0.082874560857   -2.114163133788   -0.513612398512
H     -0.374862289720    1.487873476397    1.352966334460
H     -1.188917397116    1.219834002016   -1.726862516552
H     -2.636477389556   -0.371190579169    1.231976017744
N     -1.938328293552   -0.943461532626    0.787805587774
H     -1.561145705099   -1.690883024752    1.342570930670
O      1.163993430698    5.966298766402   -1.167356320522
H      0.278086948903    5.630567125914   -1.396407189785
H      1.760606930534    5.589773970123   -1.837963639040
O      4.639133516198    2.515242554897   -0.221790187478
H      4.087875943668    2.696236386928    0.567630136558
H      5.469514250388    2.968115736826   -0.072821612485
O     -3.096503878233    4.123064166171   -0.426159677034
H     -3.090651316382    3.150808265043   -0.526761278984
H     -2.905574962591    4.291084093509    0.511513605176
O      1.838271048053    5.520301853343    1.315683816131
H      1.565382633080    5.640339637620    0.366090519136
H      2.270223332881    6.337385871337    1.567120477087
O     -1.086180273417    0.521035088559    3.523103713680
H     -1.439640249109    0.296596927619    4.384736210543
H     -1.829234872845    0.977923340316    3.044477804652
O     -1.247926669870    4.997347654429   -2.040061663915
H     -1.936552239153    4.647675449644   -1.409426527877
H     -1.695661556739    5.663645134727   -2.563213578221
O     -2.481839519703    4.406373499260    2.278166802540
H     -2.954376068995    5.071509323596    2.779120240296
H     -1.530438272744    4.469297844435    2.564711102854
O     -0.002915020132    4.577273467584    3.106776469358
H      0.376624188205    3.703413257627    3.302913938812
H      0.594714059155    4.969514795677    2.444554942146
O      2.471413849761    4.790180323429   -3.288662688882
H      2.762292089767    5.309097264510   -4.038518962987
H      1.622080820990    4.341225470384   -3.571315677269
O      1.217259690297    1.102658411374   -3.225002907782
H      2.101213605823    1.477926033584   -2.984004735074
H      1.356575274990    0.643382071749   -4.054680778470
O      3.559801235418    2.295557889219   -2.759203326741
H      3.964206329876    2.300805075402   -1.874538452446
H      3.312453300536    3.219617582720   -2.933001648188
O     -3.070820171682    1.727755563064    2.264752253960
```

```
H     -2.938111013094    2.685939116162    2.377119050024
H     -3.069302004544    1.599877497427    1.293095238116
O      1.112877875681    2.091059202542    3.621946086140
H      0.346780494561    1.481191159532    3.541973740442
H      1.497722210238    1.942996350085    4.486832928406
O      3.217371922579    3.221851252203    1.979088014485
H      2.550234862225    2.688399721514    2.428411777269
H      2.760309722822    4.039872345562    1.715251100562
O      0.240307502775    3.562752468456   -3.865036846837
H      0.445941401569    2.644696278254   -3.594902973339
H     -0.364381643561    3.926955535820   -3.195451009436

Ambimodal TS Water15-10
65
ANGSTROM
C      0.172551860366    1.646109376956    0.786068255351
C      1.286700421459    1.524997279026    0.010998587332
C      2.033676369355    0.302827925255   -0.190648774716
C      1.697773259023   -0.910255209636    0.360796513463
C     -0.186398352187   -1.445224662304   -0.817768511594
C     -1.194860198813   -0.695410304663   -0.228371002242
C     -1.551082630788    0.639184732951   -0.720014876106
O     -2.556886715003    1.282514163300   -0.332590306776
H      1.594251115760    2.375999972026   -0.600767971025
H     -0.358564825395    2.585844416377    0.864575532617
H      2.802458949058    0.332951751096   -0.964281240815
H      2.277246016352   -1.795569812432    0.127916014157
H      1.113033841021   -0.982254755563    1.268569914702
H      0.222721674995   -1.149281269906   -1.771105796920
H     -0.076886572519   -2.492600055324   -0.571928927943
H     -0.099526933948    0.901608603184    1.520850778750
H     -1.020925668160    1.001106350699   -1.617738379969
H     -2.646390984192   -0.614962407569    1.236831403830
N     -1.856491907142   -1.133789741097    0.893882558457
H     -1.805037148877   -2.098116927357    1.178101023926
O     -0.112317010572   -0.633439236267    3.309217013956
H      0.122464046432   -1.506795096252    3.675957274381
H     -1.080546764067   -0.572594268976    3.408511510165
O     -3.026480367115   -3.776033287177    1.321775601797
H     -3.159685648461   -4.577038247269    0.814715143976
H     -3.776727559438   -3.178782830136    1.115425016913
O     -6.624580139530    0.019124440259   -2.117075723426
H     -7.506964870032    0.377016034536   -2.021933336037
H     -6.124817045614    0.269828247145   -1.295219682035
O     -2.993551687237   -0.964123059548   -2.730529346804
H     -3.506858020551   -0.183595608189   -3.075912952854
H     -2.550673260028   -1.338196241244   -3.492784701902
O     -4.505418874984    1.001624030477   -3.615040249681
H     -4.328948642975    1.808868068518   -3.092295141436
H     -5.360657578641    0.672959245381   -3.293884001458
O     -4.131703792703    2.973701490184   -1.765657757227
H     -4.873780261731    3.022220029982   -1.159014430736
H     -3.429312640733    2.504366477554   -1.277252639026
O     -5.118881135061   -2.177053529286    0.689486284504
H     -5.122467759836   -2.293164281201   -0.309481209886
H     -5.953815267318   -2.522550858557    1.007624566783
```

```
O      -3.124981709669    1.781488399562    2.355550891174
H      -2.933869119633    1.672962437099    1.410739261386
H      -2.446157169264    2.400450376146    2.691595131731
O      -5.154929281244   -2.334706304110   -1.897030811916
H      -5.816037638928   -1.668320066913   -2.142268543366
H      -4.298438208398   -1.938979166645   -2.172837640473
O      -2.604870942812   -3.151683475066    3.978878399241
H      -2.793512825848   -2.202918452005    3.916409122369
H      -2.746044891159   -3.500444861831    3.084210378841
O      -2.808034709426   -0.350094088402    3.834949465300
H      -2.903392346324   -0.030832775912    4.733304713976
H      -3.002583323809    0.433335941303    3.252206278755
O       0.694845159577    1.662589890251    4.359166604790
H       0.421169499103    0.775969212163    4.016662727956
H       1.553063290133    1.834253163940    3.969400487524
O      -5.218819212354    0.518905366641    0.056657053998
H      -5.159235405469   -0.355691198130    0.476873980061
H      -4.302207487387    0.784221209804   -0.121841998710
O      -1.152773108822    3.344856559659    3.369568927564
H      -1.382213294944    3.939980489097    4.083922799054
H      -0.470086447874    2.733440553080    3.730480860526
O       0.042632058693   -3.125795055568    4.375992413245
H       0.207530497692   -3.294527452854    5.303940437098
H      -0.928127185895   -3.243817335253    4.244485027141

Ambimodal TS Water15-11
65
ANGSTROM
C       0.116366485000    1.590396118136    0.834226291111
C       1.221441858301    1.517166989200    0.034500884787
C       1.964618783518    0.316880356110   -0.243095359545
C       1.625066947974   -0.933045389665    0.240261596223
C      -0.245171816055   -1.398173253544   -0.833508502152
C      -1.241220307143   -0.622033359555   -0.241594705806
C      -1.585395026614    0.713583890423   -0.748436955352
O      -2.579518565806    1.376783201607   -0.395820917103
H       1.493599680715    2.399639178664   -0.544040611658
H      -0.428308147707    2.517953182248    0.944568750460
H       2.711660738708    0.380970355273   -1.034279536120
H       2.205941897229   -1.800170489463   -0.053799994283
H       1.107038087963   -1.049549710767    1.183565169639
H       0.124352485628   -1.134645517455   -1.813268427621
H      -0.180370022498   -2.450566707812   -0.590105131700
H      -0.137391409172    0.823210355956    1.552836271740
H      -1.018548995401    1.047704692293   -1.640403530514
H      -2.656667960377   -0.511163496311    1.259994996228
N      -1.880977946666   -1.043364102669    0.905076062947
H      -1.779942362433   -1.994463015277    1.220998134995
O       0.092851578257    4.331521502984   -1.527831149870
H       0.390144114584    4.987347975123   -2.159395774767
H       0.019550144281    4.806756470848   -0.663596301373
O      -1.219622092007    1.712412281187    4.301619037388
H      -2.008689064150    1.189137689489    4.076348923591
H      -1.142002560521    2.414198113508    3.624281580237
O      -2.605391456856    3.873359488834   -1.562719240229
H      -2.734829307504    2.992828106566   -1.165829693693
```

```
H     -1.637018879128    3.967464758590   -1.663067936691
O     -0.922968595805   -3.667950080031    1.908119959648
H     -0.534334716320   -4.488792873188    1.604898283866
H     -0.225367030963   -3.183543646490    2.384456627355
O     -2.016099239436   -2.253215887674    4.126779667021
H     -2.170944388796   -2.921162761891    3.450085002848
H     -1.053634961176   -2.105805531039    4.107629353338
O     -0.818382562724    4.024271744531    2.915580819332
H     -1.800649922744    4.223984891353    2.833130767652
H     -0.522613003410    4.467545708572    3.715793364470
O     -4.108340521900    2.001631518353    1.819111607843
H     -3.852292684769    1.309699979971    2.441746277509
H     -3.638057538166    1.813116737754    0.989640926283
O      1.974727962375    2.795300263230    3.359360387244
H      1.448663093803    3.112998373114    2.620117899022
H      1.569730821467    3.188553540380    4.152092270425
O     -3.298706984939    0.030029424249    3.688256426786
H     -2.821875447702   -0.824982736911    3.825793549526
H     -3.988442184645    0.056231669742    4.353574581701
O     -0.370974622259    5.656636987277    0.733910695468
H     -0.394978968903    5.063515014746    1.505741795259
H     -1.300464297793    5.888684354072    0.571394548034
O     -3.345731955019    4.468076756466    2.595454121251
H     -3.368740197797    4.995864286719    1.777916470937
H     -3.726421680675    3.597343902075    2.351863823582
O     -3.094252292442    5.755234160306    0.180748654598
H     -2.972213235221    5.020868019976   -0.487370588375
H     -3.718399326453    6.368004210128   -0.207061075446
O      0.717212358097   -2.200850765051    3.591347432003
H      1.296144305803   -2.621802562013    4.228076330284
H      0.894931271999   -1.227261217078    3.684472887128
O      0.432150112690    3.537933522169    5.577152529478
H      0.830883065255    3.293745838046    6.412843119329
H     -0.220765067786    2.849013564817    5.367577759943
O      1.080512841797    0.324402034065    4.075904704489
H      1.627871858028    1.048305925400    3.719011586570
H      0.205790949100    0.738582918832    4.221920480088

Ambimodal TS Water15-12
65
ANGSTROM
C      0.262701822088    1.645342433347    0.635510174774
C      1.289807646319    1.410098682491   -0.227855851301
C      1.958311747894    0.144906295979   -0.422105368272
C      1.625258835224   -1.023523959712    0.228155986200
C     -0.342892402579   -1.553404618914   -0.652224352859
C     -1.256999389693   -0.688228613704   -0.061575478058
C     -1.619436686000    0.570447779387   -0.722330789460
O     -2.605417921969    1.282808455114   -0.440530734204
H      1.569366879986    2.204486863604   -0.922739574576
H     -0.235241357095    2.605537795103    0.671126933681
H      2.632979836046    0.084670027223   -1.275144763730
H      2.146363763402   -1.941773767976   -0.013532744954
H      1.166356701003   -1.015926114349    1.209112616164
H     -0.045981149870   -1.387183651064   -1.675678679900
H     -0.259993212256   -2.574844614160   -0.306329170567
```

```
H      0.007369384299    0.975594680042    1.444487739158
H     -1.083806362948    0.780519136710   -1.669079693253
H     -2.553823493105   -0.372913526139    1.509870314597
N     -1.780921478011   -0.940937750651    1.197729204481
H     -1.836078527475   -1.898128192103    1.511939955416
O     -4.956177231348   -1.723146335754   -0.001710773998
H     -4.818843395020   -1.208608287418   -0.826596615712
H     -4.955644683371   -1.038445796300    0.687496597129
O     -2.883912185000   -1.933015950693   -3.261253121099
H     -2.253072071073   -1.914747697357   -3.995040852168
H     -2.910677456247   -2.865437575989   -2.936054823524
O      1.714539760722   -3.162777951313   -3.345437619140
H      1.423189538063   -2.235575656208   -3.338425936947
H      1.312496057922   -3.549434002190   -4.140260068022
O     -1.891225590994   -5.197208588746   -0.307092566101
H     -0.983695795652   -4.952415360667   -0.528142344838
H     -2.246358602589   -4.527757585591    0.306285472446
O     -4.448973335378    0.605067444418    1.413291570817
H     -5.074046581275    1.308552872878    1.588296514627
H     -3.819314717788    0.954594755492    0.741594530914
O     -5.712155943389   -3.842846318411   -1.846206748442
H     -5.610052431687   -3.227920359603   -1.108991314394
H     -5.880763007644   -3.285982746788   -2.630487548442
O     -5.617498329988   -2.010587324180   -3.890063333503
H     -4.645434499763   -2.046582770647   -3.854343256746
H     -5.846634069098   -1.179640627630   -3.463271496829
O      0.643344038167   -4.738348671714   -1.497702791510
H      1.051139941665   -4.082756936629   -2.118162101175
H      1.335121083967   -5.365517192310   -1.283993579819
O      0.622182040228   -0.642291365078   -3.759344185875
H      1.113088213361    0.017006149685   -4.251293761500
H     -0.079080877152   -0.966158150804   -4.348774279943
O     -3.186304258180   -4.457469036705   -2.511933056549
H     -4.149804193022   -4.407678277072   -2.323918624806
H     -2.779185624170   -4.766695224815   -1.670254307951
O      0.146078304254   -4.234647319680   -5.396303383049
H      0.356125343416   -4.817295462835   -6.126204231159
H     -0.330065133687   -4.804838770075   -4.713603323043
O     -0.994081713608   -5.658098642467   -3.578797276456
H     -1.892438237324   -5.342942857437   -3.371018068575
H     -0.474866224618   -5.470864514495   -2.776259348094
O     -4.477993452899   -0.069292002731   -2.088986478859
H     -3.806318528126   -0.621570502335   -2.530195923101
H     -3.997053266429    0.646996209481   -1.657179136894
O     -3.255828046609   -3.413276055159    1.231007287978
H     -3.850750702909   -3.987910006192    1.715043359517
H     -3.834601488707   -2.826024634658    0.693311440198
O     -1.162460923316   -1.909302344445   -5.456378331051
H     -0.698229988937   -2.776778055691   -5.474540675844
H     -1.678361105935   -1.849830267898   -6.261430192064

Ambimodal TS Water15-13
65
ANGSTROM
C      0.175785212376    1.689110871272    0.713812906160
C      1.279625108163    1.542336985087   -0.070374874646
```

10359	C	2.018496769186	0.314429584462	-0.270058940482
10360	C	1.689286323653	-0.894240870113	0.287504063734
10361	C	-0.246054268127	-1.402189234453	-0.895932220621
10362	C	-1.222926004942	-0.640392883291	-0.274740836237
10363	C	-1.572340150387	0.707186292941	-0.746949326436
10364	O	-2.582877766571	1.327614078649	-0.328786246744
10365	H	1.578305276510	2.382294335166	-0.700262002943
10366	H	-0.341525976493	2.637862197513	0.763509601313
10367	H	2.779551928135	0.338581992164	-1.051462387047
10368	H	2.257944569985	-1.782900654859	0.040032027994
10369	H	1.104618244428	-0.973064606707	1.194840231682
10370	H	0.158799825289	-1.097687911476	-1.848210576796
10371	H	-0.143048435587	-2.452159209039	-0.661267258564
10372	H	-0.101997323271	0.969916737743	1.472089387862
10373	H	-1.071481923867	1.082179465573	-1.652238882395
10374	H	-2.635646712140	-0.536790477391	1.231924275088
10375	N	-1.873441834714	-1.070800249534	0.852654635963
10376	H	-1.766183580346	-2.013310379184	1.189469947953
10377	O	-2.355404239320	2.678279082619	-3.193888562219
10378	H	-3.272809499873	2.332223968211	-3.065266986903
10379	H	-2.320582108330	2.969827591429	-4.105486655355
10380	O	-1.926163439814	0.311767157273	3.826248875971
10381	H	-2.134034514692	1.119938651312	3.331423930595
10382	H	-2.738026416921	-0.227639563286	3.783330748000
10383	O	-3.933093514418	-3.236103635735	1.972012817944
10384	H	-3.026545865441	-3.477905035502	2.206840683191
10385	H	-4.132621303765	-2.437196693638	2.492663539516
10386	O	-4.319777127574	-0.992944115975	3.508992799697
10387	H	-4.752821922469	-1.261824286415	4.319741845378
10388	H	-4.782209691197	-0.143977117507	3.231482554753
10389	O	-5.332259579866	1.305363874701	2.912805268203
10390	H	-4.512340547648	1.804250843825	2.748547725342
10391	H	-5.861039098176	1.394200232540	2.086937421806
10392	O	-2.646728372783	4.824269797938	1.019686034852
10393	H	-2.138589855430	4.630845514194	0.198939662811
10394	H	-2.264373465147	5.612703026133	1.404790343206
10395	O	-2.885073064935	2.321560449234	2.168116215261
10396	H	-2.817477624039	3.270483198974	1.979068633914
10397	H	-2.792799100525	1.891503779821	1.297706976823
10398	O	-4.151928654757	-2.670450308693	-0.628530132683
10399	H	-4.067738151563	-2.875839825910	0.333711931196
10400	H	-4.769422198499	-3.313446017181	-0.978284178868
10401	O	-1.374910769600	4.361172541027	-1.302401117901
10402	H	-1.312773500786	5.138094272791	-1.860082045295
10403	H	-1.735840975087	3.652811153755	-1.878012884559
10404	O	-6.421788258166	1.634807230083	0.496161012064
10405	H	-6.104103813783	0.874572051283	-0.019148855820
10406	H	-5.904393710647	2.388295011822	0.163848504381
10407	O	0.115990411528	-1.287247601886	3.352940902144
10408	H	0.542234630337	-1.337002981979	4.209461703193
10409	H	-0.636021746171	-0.652991944378	3.472081234120
10410	O	-1.158888349122	-3.470173480601	2.446468147961
10411	H	-0.676060250809	-2.700157136481	2.824777297175
10412	H	-0.509419019299	-4.157393256264	2.298410412396
10413	O	-4.875367871882	-0.094748747668	-0.969649075844
10414	H	-4.670399871430	-1.049364873112	-0.901214154722

```
H     -4.082407382354    0.362151609931   -0.649345690716
O     -4.890228995465    1.895271714535   -2.890853107834
H     -5.147239485222    2.584647362256   -2.265124447687
H     -4.981480226986    1.062334579171   -2.395836481271
O     -4.513118331330    3.366809697156   -0.534945228792
H     -3.781709861617    2.727859044053   -0.580362153879
H     -4.201988853475    4.065302038454    0.051100008698

Ambimodal TS Water15-14
65
ANGSTROM
C     -0.190643222818    1.386702826419    1.105640446809
C      0.966467346615    1.461878594511    0.393458589920
C      1.698248995751    0.334443224394   -0.141353705073
C      1.308870933449   -0.976995191350   -0.033914064446
C     -0.561825070913   -1.009647213629   -1.380736305277
C     -1.568931949635   -0.427705279312   -0.619322700666
C     -1.863850702055    1.010031219327   -0.733477714205
O     -2.845469446880    1.582176721897   -0.206272409536
H      1.332381763246    2.444412954744    0.099091656169
H     -0.700434294647    2.281841690050    1.433012711825
H      2.522670107155    0.578662158478   -0.812813631777
H      1.879730300802   -1.757106875541   -0.523584896462
H      0.669069676162   -1.335811152706    0.760793720230
H     -0.112245242818   -0.462472565467   -2.195712347279
H     -0.476274681339   -2.085305138138   -1.433807959285
H     -0.537583084478    0.471976829189    1.563997912692
H     -1.264383443658    1.578290888186   -1.467612376641
H     -2.963421117230   -0.680596676823    0.874216839023
N     -2.309832149306   -1.163898004953    0.274402635875
H     -1.906299670521   -2.000911642756    0.684596125118
O     -2.660103854884    1.164152146726    3.872395208977
H     -1.912191210230    0.540595993609    3.869598653160
H     -2.289268357129    2.041770497971    3.699036098065
O     -4.353757779427    0.543951014911    1.883723765641
H     -3.960659322009    0.981186844802    1.110820064893
H     -3.793218713565    0.784788004733    2.653601258683
O      0.843551306368    3.599549664397    3.550988033388
H     -0.123860484906    3.644690511680    3.532760876953
H      1.137175287297    4.013891397115    2.723361978617
O      1.646467130978    1.051770042690    3.805716901028
H      1.367458044173    1.996186668770    3.799504101645
H      2.184598051937    0.930359329709    3.021653208534
O     -1.935761049985    3.799996464804    3.217884986579
H     -2.451112485509    4.372913622091    3.787031611217
H     -2.278865378405    3.916550122131    2.307199681513
O     -0.089498090973    3.452309469178   -2.555423118999
H     -0.273810862970    4.051087523346   -1.786632267135
H     -0.390820630831    3.928380455594   -3.330512621280
O      1.546727429406    4.971068939760    1.223410090984
H      2.282976936625    4.569848995872    0.705327921037
H      1.853467266639    5.833171081818    1.508928470647
O      2.549118347483    3.061192953102   -2.582987766575
H      2.662262242024    2.123862290682   -2.747106307711
H      1.574971598384    3.217636170321   -2.579187246729
O     -0.911198096286   -2.713578375158    2.147211928794
```

```
H     -0.731061563689   -1.974280931921    2.747229160085
H     -1.670707932556   -3.174972217385    2.547926561972
O     -3.261102368139   -3.737331531160    3.122367500385
H     -3.927463227511   -3.045474799866    2.921682499174
H     -3.564211696867   -4.526608102371    2.672019832644
O      3.526801676336    4.001188819598   -0.279765455280
H      3.173297167942    3.640546646783   -1.126010029729
H      4.260777191233    4.566861879966   -0.522406967675
O     -2.867769825849    4.044811888187    0.684219602613
H     -2.857288189558    3.127742493555    0.301678957477
H     -3.744096021540    4.389348854918    0.505955705617
O     -0.484081592100   -0.538433785695    3.924169644693
H      0.318491209426    0.035315176942    3.875464603178
H     -0.464756916337   -0.950738541125    4.789440367906
O     -0.590343600883    5.082169030480   -0.533844758008
H      0.128824427932    5.033038595743    0.119866536084
H     -1.391847611205    4.773771191682   -0.080961462031
O     -5.191077120787   -1.922913094666    2.561640629053
H     -5.691492032697   -1.725576566361    3.354909091684
H     -4.903823005759   -1.052616145836    2.214981469285

Ambimodal TS Water15-15
65
ANGSTROM
C      0.147917332238    1.655911154571    0.859399091909
C      1.273130785851    1.547108502508    0.103282170073
C      2.014134750375    0.329780352310   -0.146687736529
C      1.665887057878   -0.911132302581    0.333488152012
C     -0.186264368197   -1.428308125677   -0.840064962092
C     -1.185261334383   -0.657865017243   -0.255446880151
C     -1.542330337198    0.656585050747   -0.799772652635
O     -2.568539275009    1.300787396673   -0.496163343093
H      1.595250089894    2.428024753777   -0.454603110711
H     -0.379540021575    2.597187732455    0.940927258434
H      2.795345424139    0.385729150481   -0.906112706984
H      2.252401793724   -1.782005473854    0.067668685132
H      1.081555319425   -1.027800286011    1.236856690041
H      0.204040380648   -1.159650471964   -1.810548903857
H     -0.101122621295   -2.473907042585   -0.574603206486
H     -0.177200405783    0.891815166074    1.551002188189
H     -0.946858604036    1.006220381290   -1.661365749725
H     -2.631288587521   -0.567179678635    1.207989822725
N     -1.802677069274   -1.059686697753    0.912646128957
H     -1.788767266561   -2.044393343443    1.153313142648
O     -4.418291185699   -2.591352719942   -0.196894569486
H     -4.098450529400   -2.281860215850   -1.058498125560
H     -4.660400190269   -1.778606131278    0.275312528126
O     -5.113177986681    1.045512834994   -4.754867414986
H     -5.554020504698    0.774811919745   -3.940760868209
H     -4.212308039987    0.701506745144   -4.716002181639
O     -0.348994711718    3.567492796173   -2.315579809990
H     -1.032374194350    3.356812125939   -2.985675164329
H     -0.415885071140    4.507336474269   -2.143092547507
O     -3.935649291233   -1.276853055513   -2.625379223246
H     -4.621279723308   -0.601058595069   -2.359386187319
H     -4.373006623974   -1.865700312664   -3.243511247222
```

```
O     -4.574932605035    2.858241260087   -1.681384960893
H     -4.602455112816    3.285437995416   -2.571390383790
H     -3.699509131645    2.455177931570   -1.576077709933
O     -2.389973155712   -3.851474147271    1.065717726948
H     -2.752690615799   -4.442015799685    1.726498548571
H     -3.159520881326   -3.501080530368    0.565587265187
O     -5.752001694665    0.482610707747   -1.922823594442
H     -5.426493382128    1.413910416790   -1.868320266420
H     -5.770271514734    0.200898822952   -0.990907811972
O     -4.793643669702    0.046293157430    0.590338910971
H     -4.032981432823    0.526480609511    0.209282075229
H     -5.097692040419    0.599345380493    1.335733552899
O     -3.792012143030    3.457828879684    0.904703824453
H     -3.109727856857    2.814345844812    0.674282440979
H     -4.310986323121    3.545923386689    0.089223697193
O     -4.568204103569    3.674007038655   -4.203555655139
H     -4.951560536372    2.854031278113   -4.568445388516
H     -3.617030400023    3.602436838089   -4.374153408517
O     -5.369009313077    1.922267457633    2.445371918728
H     -4.778229810829    2.574797266898    2.004212608685
H     -6.217129045539    2.353960575563    2.550922543979
O     -1.931965328028    2.844754412108   -4.373111407315
H     -1.399560310016    2.901192019223   -5.168145170192
H     -2.136698825895    1.884602627287   -4.252161555179
O     -2.334913800159    0.239787143608   -4.181369040423
H     -1.397503710647   -0.032958821987   -4.148591759617
H     -2.789181589268   -0.312025353082   -3.514024885305
O      1.563724066218    2.086906059131   -3.510718152059
H      2.288871321303    1.970748864973   -2.894902560576
H      0.910220653275    2.659073446338   -3.055226826832
O      0.336321361344   -0.248478166731   -4.033901194464
H      0.758521406353   -0.616946178181   -4.810918369304
H      0.782557873501    0.610200531438   -3.857765022477

Ambimodal TS Water15-16
65
ANGSTROM
C      0.208272377238    1.807398711254    0.605553478099
C      1.337486970978    1.540779573132   -0.113293635649
C      2.036312056652    0.283994321098   -0.118261948920
C      1.595853531383   -0.847078624087    0.548864645103
C     -0.179170469658   -1.380264115847   -0.577028336191
C     -1.212612562607   -0.553219025638   -0.148250604014
C     -1.560321379010    0.717183899415   -0.779696483206
O     -2.559288014204    1.394007736056   -0.445217190844
H      1.672010911318    2.277387816045   -0.843683932320
H     -0.304627491906    2.752627151330    0.501036900692
H      2.827240966879    0.170893629431   -0.859491552708
H      2.149413383342   -1.774615763365    0.455395157092
H      1.023254475987   -0.770891977220    1.465179624859
H      0.254676207634   -1.221933669539   -1.553186026242
H     -0.150175035764   -2.403455946728   -0.223263141521
H     -0.096353900157    1.210599659505    1.452496684256
H     -1.009033492716    0.997716033423   -1.690249062218
H     -2.849198830842   -0.394506089515    1.036550294804
N     -1.956271675483   -0.871678116715    1.004048173385
```

```
H     -2.036937034404   -1.860094726880    1.206294819471
O     -1.605372426573    4.711858395216    1.335351968319
H     -2.055389937362    5.510531899046    1.614148968450
H     -2.226568784516    3.954498250414    1.559508413631
O     -3.708053091766    3.272056214675   -1.908316682837
H     -4.519858765802    3.481406031761   -1.444897006327
H     -3.277845991572    2.546012469396   -1.390560306152
O     -1.375156223130    4.675103908718   -1.482199184464
H     -1.376157360356    4.701251087207   -0.512191740389
H     -2.242221809075    4.314394808411   -1.738293805663
O     -0.902077992820   -1.659972519529   -3.851667396659
H     -1.064661584281   -2.507777692161   -3.381233599129
H     -1.025693683855   -1.848501444196   -4.784314084863
O     -2.539795318660   -3.687247845902    0.238200425527
H     -2.850204414163   -4.529473661152    0.573378105858
H     -3.237345684354   -3.359353444112   -0.365426557481
O     -2.018504107178    0.883257396613    3.445176858647
H     -1.147172203417    1.236046684337    3.716854076615
H     -1.834793341019    0.152091332226    2.842713990010
O     -1.748863256751   -3.860741922463   -2.538363088942
H     -2.703945161150   -3.735543496093   -2.615181284245
H     -1.582115360803   -3.836171893725   -1.586798035945
O      0.435662367987    1.880540698367    4.022013437615
H      0.597859929659    2.051301777711    4.950627327862
H      0.515129691016    2.749593527265    3.570624948604
O     -3.190291147124    2.717970633735    1.840346845703
H     -3.082172286437    2.206140418829    1.019154595067
H     -2.842018790446    2.132962497515    2.546374953256
O     -1.078419456355    1.246090447929   -4.498207001472
H     -0.812109794195    0.368565701740   -4.205189017851
H     -0.611504214596    1.883076859924   -3.922672402335
O      0.643830895128    4.275931716335    2.792155750622
H      1.351584654885    4.276252563952    2.145603196920
H     -0.180017403385    4.453130017176    2.290306826646
O     -4.291099559731   -2.891216687796   -1.699117714770
H     -3.907522956317   -2.022346660583   -1.999786777471
H     -5.238232529802   -2.757267488680   -1.656857629723
O      0.229258413030    3.060376581353   -2.906853600152
H      0.636126564160    3.664158240429   -3.530123038154
H     -0.352160855778    3.622277470301   -2.344160421317
O     -3.156486639455   -0.695237793818   -2.521191876844
H     -2.357849839976   -0.963955307547   -3.004244705975
H     -3.533713471335    0.052292936869   -3.048415979913
O     -3.760809664792    1.410350585576   -4.005835831318
H     -3.823388653605    2.170779207499   -3.406817036985
H     -2.847414532788    1.435318203126   -4.354303038544

Ambimodal TS Water15-17
65
ANGSTROM
C      0.118893930553    1.947388481575    0.675474257938
C      1.172582121818    1.855634281446   -0.180892322178
C      1.959643204661    0.670018480252   -0.411041550439
C      1.701000703990   -0.550225652666    0.182977128074
C     -0.126936627160   -1.259896895323   -0.721558281287
C     -1.159902488295   -0.492285881140   -0.186756740853
```

```
C      -1.630443523779    0.732570139872   -0.840144723367
O      -2.628499789687    1.392724479989   -0.509872541854
H       1.371532911725    2.704594619219   -0.835278932063
H      -0.481216239307    2.845151282152    0.739226605272
H       2.662900077682    0.710508556436   -1.238309124738
H       2.313393925005   -1.407355273839   -0.061239852441
H       1.227957401100   -0.606351580907    1.154056107218
H       0.185338351643   -1.101689398441   -1.743595270828
H       0.030878273037   -2.260161589321   -0.338212529913
H      -0.078469799450    1.220928752267    1.451429520884
H      -1.099032823309    0.994778435748   -1.779737136669
H      -2.395670786225   -0.175605719757    1.418486437338
N      -1.776441911961   -0.854387645680    1.004654837341
H      -1.280025200429   -1.453518753039    1.648291250410
O      -2.544701975519    3.744449237421   -1.792194239899
H      -2.675375255798    2.868056659735   -1.361498045552
H      -2.901433465358    4.388849228859   -1.179306664693
O       4.488662909259    2.723672611679    0.437391107735
H       3.666329845312    3.112326681839    0.741698489951
H       4.409273983182    2.661786677355   -0.536061496470
O       4.497730532575    0.227977011016    1.438587560080
H       4.603725826729    1.148189964496    1.120603911970
H       5.309584915089   -0.034637480024    1.910132077578
O       2.995379690574   -1.137256228681    3.328766237741
H       3.294990143832   -0.467786486507    2.691960407884
H       2.057854770994   -1.313696196320    3.147383931665
O       0.424991037138   -2.170288394451    2.775065619885
H       0.161336162488   -2.561078041604    3.610044515900
H       0.862400053872   -2.893723964282    2.274265293055
O       1.828288727104   -4.125396927773    1.547160138189
H       2.756266701938   -3.922379706609    1.803812803829
H       1.850515000635   -4.348584390855    0.615682598083
O       4.677055443867   -1.758909016950   -0.302332225183
H       4.559478499791   -0.935414192289    0.234897198489
H       5.609797956987   -1.801870002677   -0.522701908782
O       4.241554148490    2.684151827135   -2.288063706602
H       4.716029439871    3.450178480308   -2.615041318403
H       4.723301211363    1.890242734672   -2.642493775246
O       5.462921915912    0.612324792030   -3.350817114784
H       4.878198749013   -0.179218694171   -3.196929482202
H       5.566033145314    0.671994158384   -4.301387436560
O       6.344944004135   -1.319602639675    2.740372711792
H       6.321996168678   -1.200243072223    3.691071593537
H       5.815028720175   -2.117741597028    2.561118733414
O       0.014252090273    4.052836445460   -2.649027447912
H      -0.113383575038    4.688635237494   -3.355108035309
H      -0.884881251546    3.861637498058   -2.308379831323
O       4.279251548526   -3.112225713983    2.072587238039
H       4.374402516874   -2.732223711985    1.179035158216
H       3.822540641702   -2.423570130937    2.614306172382
O       3.831906552677   -1.401243643327   -2.951066444122
H       3.932421518039   -1.610750424669   -2.009443244038
H       2.915047299585   -1.084058177114   -3.083417243220
O       1.381536943094   -0.367982868878   -3.547016621600
H       1.520480959518    0.610898337879   -3.564202501741
H       1.150511609983   -0.611851118270   -4.444205201242
```

```
O       1.830222495272    2.250745716451   -3.667698433449
H       1.135740727255    2.823078957979   -3.293166344764
H       2.630481071664    2.438747721336   -3.151011876462

Ambimodal TS Water15-18
65
ANGSTROM
C       0.296438997707    1.575530060911    0.803563816434
C       1.378428564527    1.412639960204   -0.009798983030
C       2.041276845449    0.160023187393   -0.299087042417
C       1.653167373454   -1.061981315215    0.191289241364
C      -0.301959363714   -1.423189216849   -0.970228897447
C      -1.225783408581   -0.635152187518   -0.297851370569
C      -1.506836612964    0.751312913475   -0.697149594277
O      -2.477611975466    1.409259430322   -0.252222967072
H       1.720619378937    2.270312496999   -0.593760166609
H      -0.162103120046    2.549057973056    0.925593898367
H       2.789000663289    0.189077595740   -1.092749014613
H       2.169667853720   -1.964935293369   -0.109694942937
H       1.085086691073   -1.158928662827    1.106398483062
H       0.092322102185   -1.108857389403   -1.924649968732
H      -0.254688449153   -2.486094020019   -0.776767263575
H      -0.008276570417    0.837191869141    1.533283924283
H      -0.975273754643    1.141615466771   -1.584122107260
H      -2.420115887125   -0.451997869189    1.372988893333
N      -1.927947868351   -1.098984499180    0.783152189533
H      -1.811066875878   -2.033970121729    1.144428290397
O      -2.822895713968   -1.519107749377    4.241483551404
H      -2.058788265954   -1.036507735918    3.823747818749
H      -2.390998104010   -2.066633112969    4.919375999808
O      -4.763567396522   -2.704762957050    0.623488473202
H      -5.682668420526   -2.967274387516    0.567465514632
H      -4.739829129725   -1.748192954781    0.445413583246
O      -0.077668944490   -3.175426752270    3.194985265341
H      -0.235675475294   -3.289550717894    4.141627801612
H      -0.879235190957   -3.507047548956    2.760236161236
O       0.262406412431   -0.273695982417    5.881526368675
H      -0.377733930162    0.462077485773    6.144676458011
H       1.115320603950   -0.021165264701    6.233676277225
O      -0.579895779726   -0.537961590099    3.265271812425
H      -0.251431615736   -1.454382637101    3.128640841981
H      -0.169771766967   -0.243780801093    4.094637878853
O      -2.668419180567   -3.462954360112    2.263539893747
H      -2.921244834326   -2.870116957351    2.992737560264
H      -3.396901184741   -3.404911925946    1.626005564988
O      -4.833245184020    0.037701317222    0.325508701079
H      -4.008953616005    0.487809587241    0.067859890480
H      -5.550078806858    0.656773886058    0.078037796008
O      -4.596269843006    0.310244335447    3.002696565872
H      -4.842316034547    0.128742346450    2.073356464496
H      -4.168095054880   -0.487188546325    3.337902385623
O      -1.366396259544    1.644811089669    6.414410963353
H      -1.237856161342    2.214935064143    5.629280810623
H      -2.277045369529    1.296828323238    6.335654053626
O      -4.260716608403    3.572105103422    0.135339387277
H      -4.041515161539    3.485337886329    1.075402197119
```

```
H     -3.606884352529    3.016640782393   -0.312244031308
O     -0.961646466163   -2.673476012622    5.891310944490
H     -1.045404707562   -2.981249308852    6.793818115420
H     -0.471320542798   -1.822743869118    5.929876531714
O     -1.123807951142    3.104790236967    4.137555499569
H     -1.783027079850    2.787593129099    3.483978234623
H     -1.276761780397    4.045155419639    4.234233687624
O     -6.451363207855    2.084128775917   -0.307250998035
H     -6.839534220481    2.343079273212   -1.143102646240
H     -5.739444318731    2.737926145130   -0.117147199968
O     -3.001293392502    2.361746366280    2.347714065697
H     -3.607390931235    1.673551808573    2.700556233806
H     -2.637128644373    1.993139295180    1.529059531461
O     -3.781634909741    0.540093810956    5.837921467447
H     -3.488080015908   -0.254428734518    5.357004831929
H     -4.366389254042    0.999079169327    5.229414474790

Ambimodal TS Water15-19
65
ANGSTROM
C      0.151397112811    1.591810194334    0.860699047138
C      1.239566304063    1.523291826237    0.042618206813
C      1.984269397651    0.322549954788   -0.259726513903
C      1.660686496027   -0.931411107930    0.212810869390
C     -0.243644177072   -1.402547305920   -0.866465558171
C     -1.227392896706   -0.636437599250   -0.249131563374
C     -1.582587728223    0.707851274311   -0.725603426448
O     -2.576196033356    1.355636238912   -0.338003730984
H      1.507074116065    2.409278147232   -0.533289334708
H     -0.387618871178    2.520132919328    0.987523354703
H      2.719794334495    0.403775978394   -1.061063519110
H      2.232403114968   -1.797167776855   -0.098840146206
H      1.126382379803   -1.071001364515    1.142450954664
H      0.129181221956   -1.118935800679   -1.839460620239
H     -0.161443738378   -2.452603236224   -0.620263993918
H     -0.109062052978    0.810108840049    1.560687726061
H     -1.028669377378    1.071119291359   -1.614599919278
H     -2.646887655226   -0.549904411178    1.234188887985
N     -1.856874362670   -1.071678762997    0.895851299180
H     -1.759438494848   -2.034427768909    1.192837511818
O      2.760134008938    1.370890195159    3.082914145790
H      3.343393978843    0.910940065692    2.477691703329
H      2.673526106346    2.287704092392    2.744638057641
O      0.467958379595    4.923448080004    1.189945099872
H      0.573099510891    5.876658539394    1.182812869009
H      1.318584334705    4.541775660870    1.482669072607
O      1.244252870379   -0.413914740090    6.405808241026
H      1.642845136878    0.479786798305    6.383316360462
H      0.435392384753   -0.323933841919    6.913283324162
O      0.680757314554   -0.347436580394    3.756075071537
H      1.405071879890    0.211572591998    3.426451046016
H      0.868984366458   -0.473437976161    4.711850841321
O      2.711868807206    4.002923150371    2.441392337576
H      3.569464682892    4.420690929069    2.354242942624
H      2.339092773080    4.311174733969    3.329746182860
O     -0.631582214267    1.859592919995    4.849995725945
```

```
H     -0.761796972070    2.669004276752    4.318030612152
H     -0.499602051947    1.124416671198    4.233053428517
O      1.666206702497    4.697532742103    4.698869630066
H      0.719994829996    4.666167394195    4.458707181846
H      1.813782949401    3.920877589981    5.271443754712
O     -1.151936049618   -3.756009720070    1.656355077721
H     -1.558688681110   -4.446335498752    2.181196137906
H     -0.326560266578   -3.508503230480    2.118282089338
O     -3.358404274818    4.822089238304    3.426200302494
H     -3.592193737141    4.317674164164    2.617116679252
H     -3.938570374616    4.500334201601    4.117187836025
O      0.993248645369   -2.955150073513    3.141995524345
H      0.837171033088   -1.997305597376    3.291974118878
H      1.211568376814   -3.323591197241    3.999272145437
O     -3.928018907869    3.359980317655    1.234287110268
H     -3.780910279686    3.897479768920    0.443788293145
H     -3.522552392559    2.510275333953    1.020034594174
O     -0.760685234482    4.225479989366    3.553203031334
H     -0.462326852240    4.400218084482    2.643234330486
H     -1.690751306275    4.535227489764    3.589732504923
O      1.866616435044    2.137446006546    5.677295867091
H      0.902399592659    2.081222474586    5.444854625940
H      2.339291381451    1.851687580422    4.883256515153
O     -0.188660505862    4.285088642847   -1.544775152548
H      0.027233962071    4.479143303691   -0.623942026477
H     -1.154815393996    4.233594181039   -1.567745873313
O     -2.990717799450    3.807668022275   -1.392282598669
H     -2.823382274043    2.888673350687   -1.081359972363
H     -3.456651560028    3.733536291553   -2.225578954631

Ambimodal TS Water15-20
65
ANGSTROM
C      0.257734380240    1.543013530764    1.089923800753
C      1.414881855436    1.469402227109    0.377095447642
C      2.128215857224    0.255008203397    0.040242977052
C      1.702902679931   -1.015621823957    0.362855789893
C     -0.097910935307   -1.321140566621   -0.933055252888
C     -1.091396457764   -0.549744409278   -0.333438089529
C     -1.375686909696    0.819209469023   -0.796628514738
O     -2.414149044778    1.449663659672   -0.531795087313
H      1.798875017044    2.382137744921   -0.082193884468
H     -0.249684220723    2.482882203748    1.256322317718
H      2.941131093060    0.361949954721   -0.677408496718
H      2.274909147497   -1.875034448852    0.034913749683
H      1.078032452083   -1.205568432648    1.226179514509
H      0.350052814928   -0.983028541339   -1.855228365166
H     -0.091497582202   -2.392457281133   -0.782107483969
H     -0.117372255976    0.723779152351    1.686904562730
H     -0.692521419296    1.222817156868   -1.570270148037
H     -2.446495966198   -0.407087563107    1.227811774400
N     -1.842219858449   -1.023684584569    0.707334960761
H     -1.729970495573   -1.969940765014    1.041244633845
O     -3.684590846073   -3.093427698720    3.237987689898
H     -4.179818520762   -2.372937051749    2.808687139266
H     -3.155182631192   -2.678498995762    3.925487552256
```

```
O     -0.351107579263    0.584434315446    4.751587529285
H      0.596327376782    0.563282007168    4.529371751675
H     -0.693937968710   -0.279367621139    4.473869406582
O     -4.947666786394   -0.881637160084    2.156266119785
H     -4.389147738818   -0.074590093248    2.237577497596
H     -5.819199917657   -0.641759972027    2.472754003754
O     -4.613332033944   -0.444679829398   -0.614354855407
H     -3.974628758241    0.280150737085   -0.556649730743
H     -4.799814355407   -0.713146873514    0.298571044052
O     -0.803127555511   -1.979072129577    3.789302189495
H     -1.102356033079   -2.517122756601    3.046537991765
H      0.140300907712   -2.185927617019    3.884349199598
O      0.123317337586    0.377915326174   -4.021337019653
H      0.391167502789   -0.316177898953   -4.628716036779
H      0.147142023615    1.205881794407   -4.506855450806
O      1.888410375147   -2.686306640817    3.587538810953
H      2.292941747362   -3.178006714491    4.303370997768
H      1.641944265758   -3.348800328939    2.902627564178
O     -3.388631527988    1.245795859110    2.044972380255
H     -3.154905711216    1.547076489701    1.151628574777
H     -2.800070846193    1.741482213144    2.653750683918
O     -1.628914545025   -3.739639035223    1.654811365073
H     -1.825890287683   -4.036786933731    0.736431119313
H     -2.471409356085   -3.757706187592    2.155269688345
O     -2.013682784019   -4.364079009032   -0.893344973269
H     -2.508842711494   -3.613333042734   -1.309624535127
H     -2.523377012512   -5.147217878062   -1.104587657333
O     -3.466595123833   -2.477837832279   -2.019498483768
H     -3.964762633037   -1.844492393038   -1.465742050620
H     -3.097610806824   -1.933341394338   -2.731333300064
O      0.955638523995   -4.482595760400    1.843471231484
H      1.192362746049   -4.560650546084    0.918297725754
H     -0.007955386065   -4.308644664014    1.857832006046
O     -2.571153891703   -0.373159197355   -3.604724735375
H     -3.060354155352    0.284959797818   -3.105109433885
H     -1.642087276040   -0.099269213902   -3.604242215356
O     -1.699025845359    2.572805920717    3.641812255501
H     -2.059825832082    3.068356073194    4.377479355978
H     -1.155035216108    1.851968553993    4.045343356313
O      2.185740361346    0.108694432704    3.756675104894
H      2.172068078152   -0.857998428544    3.684228410082
H      2.273392978098    0.450313773987    2.864460616378

Ambimodal TS Water15-21
65
ANGSTROM
C      0.229192640081    1.707911416539    0.769320913060
C      1.300180722566    1.583750603693   -0.063058124448
C      2.030282234234    0.366702713004   -0.320125015474
C      1.698071231572   -0.866280057255    0.210723176152
C     -0.160988151457   -1.394428422210   -0.802211433434
C     -1.160024004818   -0.633331119813   -0.198190097486
C     -1.604858539831    0.669888113845   -0.716885538576
O     -2.612811369195    1.260069626906   -0.293078508262
H      1.576535181462    2.441675428011   -0.679009210770
H     -0.311942745354    2.640583519966    0.857915478149
```

```
H       2.749832970427    0.400414226591   -1.137720115249
H       2.274498127433   -1.741580882534   -0.064997466576
H       1.202736250865   -0.950811153928    1.170170651574
H       0.177980348213   -1.138064164346   -1.795655369856
H      -0.060525148496   -2.439816454959   -0.538262325517
H      -0.033274679180    0.963811720411    1.507583106974
H      -1.106667431489    1.041858817224   -1.635103058794
H      -2.536580274036   -0.460469304954    1.285171451149
N      -1.752988673588   -1.022421753524    0.987088975304
H      -1.813669099031   -2.001431138184    1.243298348881
O      -3.226978851372   -4.849018410051   -4.268887934174
H      -2.424100003099   -4.450187597601   -3.864382115762
H      -2.960781519013   -5.685154190927   -4.650954986630
O      -1.402402080115    2.043706085423   -4.156659053382
H      -1.452982684188    2.681244263410   -4.869423395210
H      -2.330989434861    1.800275020514   -3.932718633709
O      -4.657988708452   -2.449332913247   -4.425377985220
H      -4.426678420254   -1.855604587622   -5.172000743328
H      -4.340886995779   -3.334071600126   -4.647493160514
O      -4.435138287855   -5.055491736312   -1.755513003599
H      -4.571593943202   -4.151658120653   -1.421656216341
H      -4.143784502936   -4.964781814113   -2.677973770142
O      -0.213450425151   -4.887937682146   -0.815848988261
H       0.446596782848   -5.581189710877   -0.846655897467
H      -1.035957001446   -5.299735846103   -0.493991775688
O      -2.863884878135   -1.625150891962   -2.728752901198
H      -3.586503567127   -1.935770543754   -3.350085903151
H      -2.228908849101   -2.373482255114   -2.701674637175
O      -3.657066009618   -0.521742831035   -6.014384317723
H      -3.982339009370    0.184248586696   -5.439337997815
H      -2.713103728452   -0.604826878102   -5.793269804072
O       0.737589944292   -2.387826948973   -4.432642785179
H       1.022858779828   -2.677491077042   -5.299666767108
H       0.120883223485   -1.635869906396   -4.583357257509
O      -3.825098820017    1.025469016089   -3.635750294025
H      -3.597821365281    0.210696427454   -3.169633529946
H      -4.276073150306    1.583404541945   -2.967634687592
O      -1.209495080221   -0.583681573717   -4.689612701801
H      -1.748554851557   -0.885724481296   -3.931696486419
H      -1.067770094185    0.371758611566   -4.534466170814
O      -4.468776212542   -2.469714423788   -0.741221607428
H      -5.231291899447   -1.891225083916   -0.704329172897
H      -3.833020461504   -2.055980371050   -1.366030175945
O      -1.158532269677   -3.692811293104   -3.039896695425
H      -0.400422179066   -3.295363305922   -3.516912319874
H      -0.792837521117   -4.090211760779   -2.224782284009
O      -2.595807909511   -6.028771040216   -0.160274269668
H      -3.292634946030   -5.698527255276   -0.791670097121
H      -2.990924026455   -6.760759468739    0.313545707348
O      -2.868655934407   -3.615315002666    1.295038954500
H      -2.659398767968   -4.477558017043    0.909618920902
H      -3.530878429542   -3.227438938507    0.704995003958
O      -4.663195625312    2.451079103341   -1.537645445209
H      -3.949128271464    2.055603321412   -0.991344220473
H      -4.561695566389    3.400148187623   -1.456975958587
```

```
Ambimodal TS Water15-22
65
ANGSTROM
C      0.229130242900    1.666035144599    0.810485311174
C      1.320020007909    1.544966165698    0.001552564462
C      2.041497890316    0.317724629054   -0.251293303449
C      1.676305285833   -0.910120561091    0.264662256283
C     -0.190752351783   -1.406240750621   -0.840208843772
C     -1.191193036614   -0.653062101950   -0.230128762959
C     -1.566042531946    0.684544168716   -0.727477146102
O     -2.547312503918    1.336215769946   -0.336486279153
H      1.626995164329    2.407561009146   -0.594210541111
H     -0.303507612182    2.604015000427    0.901549299344
H      2.783562467030    0.354324170768   -1.050229496816
H      2.237536456372   -1.799456782909    0.005274061782
H      1.134107483078   -0.985537256950    1.198504496704
H      0.177824397866   -1.120672157591   -1.814223719217
H     -0.092653904089   -2.458379352754   -0.607353804340
H     -0.056415418704    0.905788308743    1.524156864181
H     -1.025038932223    1.034421270135   -1.630369986195
H     -2.551469498692   -0.520316688870    1.320483451795
N     -1.844499808924   -1.090209058866    0.888871178301
H     -1.689709169548   -2.002736712797    1.274907696130
O      3.389799308657   -3.127980463920    2.365384781838
H      4.045989624469   -3.774620331906    2.627672643456
H      3.886414722443   -2.320146500729    2.110673473663
O     -3.364289958382   -0.457232791215    5.727612092003
H     -4.317281383853   -0.562347735130    5.718889494869
H     -3.197399462039    0.456156692016    5.364665094914
O      3.689434861286    0.451647607139    4.155888552597
H      4.289333746247    0.815801617924    4.807671395050
H      3.090396307115    1.194031006268    3.883762475109
O     -0.086497924949   -0.704267625393    3.253814847880
H     -0.874195377070   -1.232159428777    3.475490029738
H      0.680486751225   -1.167246687598    3.671158051906
O     -5.209920371586    0.628418870367   -0.950906278155
H     -4.323150703965    1.010782745808   -0.908818185672
H     -5.091634751739   -0.314037008151   -0.712656526595
O      4.603106389400   -0.759494629847    1.887966811712
H      4.136422545887   -0.295372978916    1.188527092655
H      4.354133380781   -0.314499184128    2.721633091401
O     -6.241855448499    1.852024421107    1.174129260228
H     -6.468804940912    2.745608710570    0.915569939186
H     -6.009095323794    1.374051136114    0.340373951823
O     -0.184548637001    1.501876432054    4.624134564893
H     -0.181788777480    0.664127025900    4.072788345015
H     -0.077318118246    1.219761496108    5.534234780005
O     -4.817414435796   -1.922538746162   -0.065737385536
H     -5.616483949613   -2.436710768805   -0.186350156374
H     -4.724391849915   -1.795365087731    0.906815926892
O     -2.925568562289    1.861615471624    4.580764710643
H     -1.957865594419    1.927615164544    4.522817587755
H     -3.244266764224    1.707152470202    3.671740323355
O      2.047459950627   -1.816051095724    4.356618844946
H      2.430828062904   -2.401291194345    3.675205022022
H      2.651300096377   -1.058904350938    4.409915147619
```

```
O     -3.827353706409    1.175088714512    2.099360010725
H     -4.739825080125    1.489589453206    1.916110594764
H     -3.338452479737    1.344595715651    1.271426978700
O     -4.536699543970   -1.446195439732    2.557339860698
H     -4.384620649940   -0.483611521008    2.518195281562
H     -3.765169188267   -1.819180041532    3.009227972285
O     -2.278222300651   -2.141948537374    4.045751622769
H     -2.650388711114   -1.509924383308    4.722393360657
H     -2.070341210563   -2.947722915900    4.520061500570
O      2.057634236546    2.402532807236    3.455506583939
H      1.816626983302    2.406171073335    2.524834262314
H      1.224969316882    2.203775973077    3.938619890969

Ambimodal TS Water15-23
65
ANGSTROM
C      0.136388127427    1.772754169579    0.640020623734
C      1.276884445679    1.593441240932   -0.081349167073
C      2.021906382738    0.359698163877   -0.179016435447
C      1.653169781997   -0.823587021757    0.420507195382
C     -0.178984532209   -1.387373884350   -0.739730561151
C     -1.214151929240   -0.614327403216   -0.231040408625
C     -1.595696253563    0.666871561540   -0.835223466831
O     -2.614984755808    1.322136442355   -0.520366505898
H      1.609630074707    2.400848568377   -0.735672592974
H     -0.398808395228    2.711976187465    0.619598753208
H      2.812683922750    0.335094943993   -0.929011100353
H      2.236592028460   -1.720937602337    0.251504637749
H      1.059128551521   -0.847363635021    1.324545892967
H      0.234955696720   -1.156293175095   -1.708423981479
H     -0.084347770931   -2.420167230722   -0.431085354487
H     -0.178182328280    1.086910477847    1.412843056481
H     -1.045347700414    0.964646267273   -1.744679363782
H     -2.816971031588   -0.640422403014    1.065336123384
N     -1.872294925369   -0.978479084879    0.939969838249
H     -1.736205458559   -1.926684195058    1.259092761322
O     -4.756830316659   -0.171722242730    0.290411642567
H     -4.119383349460    0.466358547446   -0.076409540964
H     -5.323390271402    0.334217149022    0.904875789661
O     -0.845942416727    2.690939205200    3.516600808085
H     -1.644310884944    2.572236419381    2.946109144382
H     -1.051415125650    3.405761577865    4.119604667272
O     -6.022859621429   -0.650647033274   -2.136595255137
H     -5.634821188713   -1.538052119458   -2.193708441717
H     -5.885795710082   -0.404747766302   -1.206559852409
O     -0.342320340504    0.265128007731    4.467553867317
H      0.378617354099   -0.166798557294    3.992039495823
H     -0.467520280754    1.166565781135    4.091998786181
O     -3.048792442379    2.174824056149    2.120505838745
H     -3.347856378757    1.393808263001    2.620667565807
H     -2.890209681638    1.891895195531    1.203819721882
O     -0.844782150002   -3.260909921658    2.460505331231
H     -0.030280669067   -2.832123462871    2.791589502555
H     -0.817639674642   -4.175097269168    2.746372249150
O     -4.143885425285   -2.555271221316   -1.321668464914
H     -4.266743252473   -1.941096569450   -0.588486114008
```

```
H     -3.442249186000   -2.174682182059   -1.880051585281
O     -2.435038338284   -1.260798859008   -3.074012016812
H     -2.509810431611   -1.707449951598   -3.918701283495
H     -2.977854040757   -0.438383477781   -3.161131211376
O     -5.921021002379    1.423553317675    2.149309127838
H     -5.524575121609    0.909758498870    2.868055926575
H     -5.378467025375    2.214499868629    2.061457078901
O     -4.034595024926    0.867193189844   -3.122874873361
H     -3.772630770176    1.389984631227   -2.358308612207
H     -4.863790851762    0.397666897842   -2.869259940051
O      1.186061398561   -2.008297665770    3.782762088479
H      2.085426668400   -2.331295779709    3.838392476010
H      0.725326557784   -2.310343504692    4.616588547135
O     -2.233191558670   -1.669773850735    4.242653208938
H     -1.572540733960   -0.949791417913    4.316743853979
H     -1.932744418543   -2.226510773947    3.503622583527
O     -0.220987277125   -2.764749411170    5.872317575170
H     -1.114804178691   -2.623222912913    5.519177232309
H     -0.104491414613   -2.055804741976    6.529728449874
O     -0.000222325131   -0.339155405052    7.105876841464
H      0.745721064277    0.148024921239    7.456405817705
H     -0.170436345518    0.008047818067    6.208694279800
O     -4.048106822140    0.238601508256    3.847328457508
H     -3.499331893483   -0.575729476234    3.916660993282
H     -4.129370804897    0.566617661797    4.744309509712

Ambimodal TS Water15-24
65
ANGSTROM
C      0.093459232730    1.671622188190    0.879589564881
C      1.225591541019    1.613272592114    0.123960227662
C      2.010081896780    0.423398672208   -0.117444075565
C      1.703038945841   -0.822611449535    0.379992183770
C     -0.138842611764   -1.397213472915   -0.782337760249
C     -1.166629434723   -0.608975742599   -0.274600515183
C     -1.486515839416    0.691253414699   -0.866022936821
O     -2.524408708874    1.346936107626   -0.630630196023
H      1.514278981815    2.497475366537   -0.447367218964
H     -0.479746559653    2.585184947052    0.971821568880
H      2.782145562454    0.505205054646   -0.882996574631
H      2.312739814077   -1.679261687380    0.118816094222
H      1.129961885657   -0.943802713195    1.290217743321
H      0.284190120658   -1.173897814985   -1.750167276614
H     -0.051912918109   -2.429913892288   -0.469412256656
H     -0.178066963831    0.895526802731    1.582365351910
H     -0.844292508477    1.021318390679   -1.703459450044
H     -2.448226295395   -0.349428800362    1.343536963681
N     -1.943755743424   -1.031754827108    0.788044845362
H     -1.644116103862   -1.848596131839    1.292924928165
O     -2.383011204424   -3.945663353533    4.923118495603
H     -2.397932343626   -2.989534394882    5.064006946558
H     -3.248578073135   -4.162084579892    4.564816333668
O     -4.147054366188   -2.424052390709   -0.805573228766
H     -4.470740151129   -1.622712632174   -0.370486376234
H     -3.507300747279   -2.830262979660   -0.199266480287
O     -5.374825543944    0.235255119163   -2.728080579059
```

```
H     -5.385598914344    0.022535136222   -1.778850161080
H     -4.622508913300   -0.279476919628   -3.075507418196
O     -0.608008234995   -3.985270438368    2.843014253600
H     -1.125153092141   -4.137541146004    3.658846541995
H     -1.248633950368   -4.064846445028    2.116016686929
O     -4.283359862103   -2.843693651935    2.945447214681
H     -4.993490087273   -2.298135803799    2.603829015988
H     -3.673673454533   -2.228033512052    3.426493485521
O     -2.759244353205   -3.956850677138    1.097064133976
H     -3.109616493342   -4.818797086314    0.868029085982
H     -3.361358893088   -3.582879514921    1.789263130209
O     -7.688540909838    1.783304940528   -2.264893068085
H     -7.754083017700    2.454543796778   -2.945952417267
H     -7.026592661357    1.147654793525   -2.575986138452
O     -4.313715237668    2.717117354569   -2.261715065037
H     -4.691490784955    1.904971028583   -2.651211298023
H     -3.501917844184    2.424366897910   -1.821761149868
O     -3.774508876163    0.540639081966    2.508831287440
H     -3.660576840953    1.501334865879    2.359208872970
H     -4.401942113586    0.268119234668    1.817887986455
O     -3.192810560534   -1.361626039814   -3.068494123513
H     -3.480742900724   -1.820534384082   -2.245313401583
H     -3.173316890899   -2.031253864048   -3.752825746981
O     -0.093067148921   -1.432878172478    3.224134117036
H     -0.176993327233   -2.406720047689    3.044903955853
H      0.555566747489   -1.349062333938    3.924055379999
O     -4.895243706815    0.176414440994    0.041755308645
H     -4.005025951723    0.506649079872   -0.202583897809
H     -5.445643414532    0.999029746932    0.055526605598
O     -2.610932223470   -1.200659254391    4.206071521612
H     -1.696360554758   -1.200704776186    3.852171695456
H     -3.029810100638   -0.412545030535    3.820230566426
O     -6.021794093518    2.567251978292   -0.210813238307
H     -5.407506934481    2.816638909100   -0.942997627067
H     -6.860289951323    2.392214937466   -0.667687696680
O     -3.666866464098    2.982538454445    1.394380998384
H     -4.569109633034    3.113411919658    1.075360963201
H     -3.172435070918    2.640994403856    0.636629185622

Ambimodal TS Water15-25
65
ANGSTROM
C      0.399806043314    1.278068032325    0.426039989341
C      1.510165451574    1.012281331520   -0.317009786304
C      2.244137852747   -0.234223462114   -0.306887035610
C      1.905126962423   -1.325882739688    0.454128072527
C     -0.008520091530   -2.057642968909   -0.576394851432
C     -0.999280780015   -1.163538753109   -0.192172006691
C     -1.281210102248    0.039652301787   -0.980148196859
O     -2.270177831972    0.788729199991   -0.806599025530
H      1.819502826642    1.739995615102   -1.069838294380
H     -0.128899859772    2.218118654930    0.330949137154
H      3.003066566255   -0.349969368033   -1.081360826091
H      2.471063194558   -2.246230366497    0.374656003447
H      1.332727022893   -1.224954209345    1.366752301723
H      0.416970860151   -2.002558635158   -1.565996128125
```

```
H      0.060222789963   -3.025746428089   -0.098678304542
H      0.120937156331    0.681470407990    1.283782101738
H     -0.683389839705    0.177392197357   -1.899094693146
H     -2.362061084766   -0.635369803840    1.279096110981
N     -1.758692590415   -1.361552749589    0.934497976777
H     -1.563895074296   -2.119780004521    1.563596608096
O     -5.157254190733    2.970118019396    0.121572401732
H     -5.385166780482    2.045722323612   -0.074095701993
H     -4.459324270553    3.197460015010   -0.534075031405
O     -3.740348004292   -3.903655341041    1.921722155868
H     -2.945270505721   -3.622655685730    2.404649469355
H     -4.485317452800   -3.650155263386    2.472340873294
O     -3.343876626261    0.416982842469   -3.974291547999
H     -2.702341639243    1.121781025164   -3.861838951360
H     -4.215221528977    0.834745317495   -3.876612853885
O     -1.642350816929   -2.917496448949    3.512823413173
H     -2.354597163136   -2.301019877407    3.914102265045
H     -1.457663205686   -3.576287570668    4.183277400065
O     -3.334497710901    1.531575300032    1.611592577782
H     -3.980658592430    2.226731087192    1.398533536795
H     -2.878476325875    1.345741625349    0.769880014410
O     -4.785361733193   -0.763076393222    2.217705498888
H     -4.758583045363   -1.271049957461    1.390673893421
H     -4.414207701536    0.113959720210    2.020510891207
O     -6.936748417787    3.783448976338   -1.808563226910
H     -6.822123969954    4.715609987397   -1.995783829555
H     -6.448629665544    3.606973744698   -0.979062413699
O     -4.529271913967   -2.246597666627   -0.134547517058
H     -4.204917035072   -2.981780456195    0.413654879720
H     -3.911991783425   -2.164492098342   -0.881625247147
O     -1.995012477370    0.902787728097    3.991545413294
H     -1.189559010860    0.479975200534    3.652220491008
H     -2.455270580779    1.276109600694    3.224373664985
O     -5.057022933944    0.365749137037   -0.822897576018
H     -5.267094622672   -0.551760061138   -0.607103645917
H     -4.088515186874    0.396483609316   -0.886035127078
O      0.112597999785   -0.808011943531    3.323134263549
H     -0.448075960438   -1.607492112607    3.383686931009
H      0.640341374454   -0.777946328001    4.122398135084
O     -5.532494662501    1.811921448322   -3.072491612771
H     -5.578645533151    1.190747949255   -2.321182269431
H     -6.181855057418    2.506754462347   -2.857636781475
O     -3.386435003751    3.073381564931   -1.914367188299
H     -4.089115581854    2.704721252702   -2.485702489374
H     -2.860958342015    2.315528755740   -1.611940650854
O     -3.025959000415   -1.797979411094   -2.422668665381
H     -3.164595010473   -0.990911221748   -2.960707947432
H     -3.145748447974   -2.532031037665   -3.027213790290
O     -3.409154636562   -1.332915936733    4.434777546301
H     -2.943050657470   -0.468709999701    4.490144862814
H     -4.053421964748   -1.200315618605    3.705741149685

Ambimodal TS Water15-26
65
ANGSTROM
C     -0.144987220547    1.988388781784    0.645241973529
```

11255	C	1.037870812261	1.963183074479	-0.032246107902
11256	C	1.980100404758	0.873406511250	-0.035256660006
11257	C	1.768677943586	-0.328236389267	0.618785474234
11258	C	0.168747154616	-1.291024496135	-0.504178349123
11259	C	-1.015562046379	-0.652196795842	-0.128468921043
11260	C	-1.551324488563	0.495135408765	-0.879100921695
11261	O	-2.656192757982	1.019872383865	-0.673864754990
11262	H	1.254813587052	2.781608075853	-0.720829115780
11263	H	-0.831685368170	2.818449078799	0.545312106670
11264	H	2.792720227265	0.934557449928	-0.758141041774
11265	H	2.509543280509	-1.116567227840	0.545491963565
11266	H	1.166263170982	-0.365616983998	1.518077739228
11267	H	0.583431205884	-1.105839759164	-1.484028232690
11268	H	0.388793750810	-2.270161367175	-0.096439056730
11269	H	-0.379308470463	1.305791620684	1.450106702699
11270	H	-0.986648620192	0.783680942049	-1.788928787019
11271	H	-2.620897965325	-0.654550807487	1.160201738205
11272	N	-1.685680295706	-1.009391381595	1.017234292007
11273	H	-1.476596148506	-1.886837087806	1.465928231716
11274	O	-3.230938727156	-1.816082287819	-2.008752064573
11275	H	-3.162178433399	-2.729420523759	-2.289058384112
11276	H	-3.821155796588	-1.370021180511	-2.662181832506
11277	O	-6.064739323304	1.058241959428	0.194052162576
11278	H	-6.797896251494	0.939127116663	0.802504060408
11279	H	-5.387817955947	1.590349558854	0.683025403065
11280	O	-3.686536741453	-3.277607388137	1.901938135018
11281	H	-4.513568320570	-3.713557056124	2.154590555650
11282	H	-3.956053673005	-2.503234391439	1.378077582048
11283	O	0.147334433365	-2.616221008879	3.003200342739
11284	H	1.088005106882	-2.508263460062	2.861469140949
11285	H	-0.185668537722	-1.776386733556	3.356060844339
11286	O	-4.664093073694	-1.304576353871	0.225569699597
11287	H	-5.043812904411	-0.407790885764	0.138124114847
11288	H	-4.122452768444	-1.454318914489	-0.574381181884
11289	O	-2.433338309370	-3.098349085899	4.218513637514
11290	H	-1.596932677518	-3.551080863974	4.075834343674
11291	H	-2.886292853033	-3.139836383553	3.335568587408
11292	O	-3.997072902937	2.078483389409	1.479775877070
11293	H	-3.896523778768	1.372519811694	2.136925278405
11294	H	-3.413744354788	1.830243226090	0.742686507829
11295	O	-6.225573964885	-3.919525868968	2.819453726080
11296	H	-6.623416328224	-3.308121575239	2.131979812880
11297	H	-6.860299705129	-4.620512412008	2.963055154709
11298	O	-5.020123823906	-0.613396777560	-3.575299370098
11299	H	-4.943019953439	0.299712874678	-3.196817424711
11300	H	-5.929422248073	-0.878265444671	-3.426121401059
11301	O	-6.716483308263	-0.081960005429	2.822757302821
11302	H	-5.769592315072	0.066518362784	2.688943449465
11303	H	-6.787298062008	-0.455647822557	3.704612993496
11304	O	-4.841023616938	1.659812317657	-2.235182614122
11305	H	-5.476971463875	1.563078818937	-1.511363604311
11306	H	-3.973482595484	1.572124731913	-1.805656904339
11307	O	-3.830512945097	-0.195614017212	3.037413638199
11308	H	-4.282243428020	-0.889576817171	3.561244602513
11309	H	-2.929426772060	-0.160763757025	3.392680576258
11310	O	-1.411998907946	-0.662939802830	4.334296992442

```
H     -1.341180729248   -0.336291710723    5.231640419517
H     -1.846455397336   -1.554180155295    4.387487658402
O     -5.103188209171   -2.087568374868    4.529116703574
H     -5.564583815455   -2.743841711310    3.973946517437
H     -4.318279677238   -2.542431213621    4.852665766115
O     -7.071076901136   -2.179704168682    1.087850900292
H     -6.242899915020   -1.997353546357    0.600879997484
H     -7.173239849933   -1.400908708610    1.665790629091

Ambimodal TS Water15-27
65
ANGSTROM
C      0.249137735006    1.657265214638    0.833977134891
C      1.351573230042    1.522733934129    0.049010001600
C      2.056434846808    0.286859992621   -0.214366786170
C      1.676061994519   -0.947406792944    0.268214089183
C     -0.195068385660   -1.393858016656   -0.843851862454
C     -1.180849177417   -0.625319208582   -0.238158079414
C     -1.562719734553    0.694821414906   -0.757330941590
O     -2.557439767527    1.342901627938   -0.378046715837
H      1.685680248780    2.387833371817   -0.526944892921
H     -0.266841881085    2.603420513805    0.922965268711
H      2.811774578609    0.328194342354   -0.998881525455
H      2.232130749283   -1.832635829061   -0.014683362274
H      1.119817363424   -1.049946536748    1.190957955861
H      0.167950938842   -1.107804738456   -1.818864258871
H     -0.123702940398   -2.450106374738   -0.618149578178
H     -0.075456295421    0.894566192333    1.525466733347
H     -1.016003079728    1.046860989220   -1.650633061342
H     -2.640211655809   -0.552509125567    1.202771717795
N     -1.791357388603   -1.026792352361    0.940360168135
H     -1.764448833885   -2.002308790419    1.203947217754
O     -1.993117040906    3.732010031720    3.046657963771
H     -1.232602655309    3.339543223427    3.503199589507
H     -1.674249593757    4.516978081502    2.600476673286
O     -3.471175157066    1.583805818789    2.115175518531
H     -3.229479026416    2.477685646765    2.402865055089
H     -3.194013859329    1.530716964676    1.171538248772
O      0.075247011358    2.408079997442    4.441856768585
H      0.276043366066    2.660500820771    5.343983049814
H      0.792859255170    1.782360235356    4.169618209974
O      0.123484701742   -1.443437350156    3.267497863885
H     -0.577763712060   -0.767305216625    3.216734428031
H     -0.336506337822   -2.193611014330    3.703097382378
O     -4.747155750840   -2.290856732949    0.741006990031
H     -5.586254451265   -2.741636318436    0.638888877169
H     -4.845287538634   -1.707735772980    1.537934502417
O     -3.233290287155   -1.408954337088    4.947115077446
H     -2.622895134244   -0.718420583269    4.600593400574
H     -3.499092699183   -1.132733728550    5.824391177907
O     -2.645593682011   -1.146088851356   -3.007170486912
H     -3.001835233906   -1.301645212284   -3.882905155449
H     -3.413543518016   -0.910335462499   -2.450467397904
O      0.851076971044   -4.104928824514    2.389524003742
H      0.915263150905   -3.142903317555    2.444903175007
H      0.231622142578   -4.351468426083    3.085934589936
```

```
O     -1.488978986662   -3.438264718467    4.070283158131
H     -1.920248850965   -3.651979963658    3.219721687146
H     -2.151074425288   -2.913958041169    4.543580821451
O     -0.487336795136   -4.645945951704    0.114180326408
H      0.088678724483   -4.483278498100    0.894487663497
H     -0.338203270934   -5.554430245960   -0.148064869908
O      1.859201595775    0.546064826352    3.803419680638
H      2.302943552445    0.648897383805    2.959376122986
H      1.317928818408   -0.269554581325    3.723826194738
O     -4.933024437469   -0.704541556370    2.838603013068
H     -4.409223133783   -1.050880627988    3.580925096025
H     -4.580981203756    0.175129009823    2.633532128685
O     -4.665598604848   -0.402278365830   -1.306729781582
H     -4.660919826667   -1.081106398887   -0.609159941259
H     -4.200393316898    0.364709524851   -0.950730414533
O     -2.600366721305   -3.802948715248    1.623114439247
H     -3.367740433441   -3.388919269353    1.195074342634
H     -2.017286830572   -4.154821633047    0.927781925147
O     -1.744509439446    0.452517425723    3.778456616218
H     -2.367346461187    0.854141916872    3.128731390424
H     -1.198675413140    1.180967442531    4.117407372859

Ambimodal TS Water15-28
65
ANGSTROM
C      0.159357991458    1.778191666785    0.841427508395
C      1.303625631707    1.603781117441    0.127800919316
C      2.017452865990    0.352405468271   -0.039431772168
C      1.611168431742   -0.854321746998    0.470110469689
C     -0.225465288183   -1.314353667641   -0.830930728982
C     -1.241083157778   -0.565937665255   -0.252470817846
C     -1.567586759224    0.773291159623   -0.756254870319
O     -2.575014885761    1.440133375906   -0.424003346884
H      1.677542257647    2.442247491652   -0.463497246661
H     -0.344324661582    2.735763523485    0.872800235785
H      2.831266321699    0.365467766164   -0.764237346432
H      2.174211389134   -1.754976698805    0.264303525549
H      0.959753439040   -0.931300983615    1.329524853535
H      0.230254897989   -0.986451202267   -1.751758007637
H     -0.131789720873   -2.367798478865   -0.608152900208
H     -0.204760837629    1.047523889211    1.549470514398
H     -0.971149085216    1.127872428829   -1.615972490668
H     -2.763095665693   -0.536333594732    1.145421937610
N     -1.921270940074   -1.006503018518    0.857663808446
H     -1.827977166066   -1.967933408944    1.162521277469
O     -3.009194502780   -3.704615852641   -0.716629040621
H     -3.736107873244   -3.477837556286   -0.121779401176
H     -3.007529518410   -3.027479423175   -1.412002762364
O     -4.528993789264   -3.091034044977    1.699082400914
H     -4.650735144950   -2.143904264453    1.941628327860
H     -5.277928652285   -3.554627403718    2.073950530524
O     -3.048409235967    1.403880982033    2.463628404017
H     -2.818442140381    1.618127840976    1.549518846702
H     -2.240726244150    1.063071483173    2.884959676828
O     -4.962938915789   -0.524939290483    2.274657528250
H     -5.312190614332   -0.145775126321    1.452350671509
```

```
H      -4.318826089087    0.139426050607    2.584866894620
O       1.833862457626    0.579307487772    3.736972286320
H       0.854832009880    0.563258441939    3.776909626498
H       2.061481700693    1.067813009131    2.944066836999
O      -3.241561318057   -1.870303366348   -2.815571830108
H      -3.474521825398   -2.491813616817   -3.507896062205
H      -4.058070521248   -1.307681436356   -2.682807322651
O      -5.301578288873   -0.281708317609   -2.571358169163
H      -4.900829927540    0.546734976123   -2.913819066841
H      -5.481602646305   -0.088666650111   -1.637920158972
O      -1.858447124108   -3.765670620823    1.757172345993
H      -1.912339045813   -4.029609413333    0.824615314150
H      -2.790081187871   -3.662622551995    2.014482074200
O      -0.882069365927    0.265747608609    3.784460692253
H      -0.831318420596   -0.729634181461    3.687727288020
H      -1.172833267584    0.412448866685    4.686553258157
O      -0.531084222779   -2.309973534657    3.707470386691
H      -0.933917935148   -2.890699779976    3.043753299639
H       0.435907022556   -2.394874655336    3.601050352719
O      -5.228541376396    0.880630631742   -0.072334638142
H      -5.677307988882    1.727372469311   -0.086098078797
H      -4.275919457489    1.078341018862   -0.168571105856
O       2.187572103113   -2.105444772800    3.532994794048
H       2.174412972076   -1.128559124908    3.615690255979
H       2.664855390849   -2.432229138144    4.296521114797
O      -1.934974979263    0.186745107851   -4.106863253113
H      -2.304941160822   -0.593951490919   -3.654326480651
H      -1.001958554817    0.225477132561   -3.860633344590
O      -3.781795612519    1.907301857527   -3.041505625243
H      -3.381748592084    2.015358165306   -2.169251265913
H      -3.119666540272    1.428501254232   -3.576013407499
O       0.859543047014    0.490717079266   -3.619091480587
H       1.313732738261   -0.028003788011   -4.287257043210
H       1.021541544347    1.415765604360   -3.818603033318

Ambimodal TS Water15-29
65
ANGSTROM
C       0.073186512693    1.691091493374    0.874487169705
C       1.191545706353    1.595201398093    0.106416172504
C       1.951509241192    0.385556420250   -0.143328337685
C       1.637560246454   -0.853763093687    0.347024597981
C      -0.258418066656   -1.358561662013   -0.889648036241
C      -1.262946487024   -0.630161560885   -0.270499558484
C      -1.611734135207    0.704111100615   -0.752545749075
O      -2.632784061032    1.362004059845   -0.412909789021
H       1.498876710016    2.470370406375   -0.467843402632
H      -0.458322465823    2.629548091677    0.974122602651
H       2.727894995986    0.461214093243   -0.905560567391
H       2.224951642375   -1.721493833128    0.074892098801
H       1.025323449924   -0.989601853195    1.228568006617
H       0.160407391710   -1.024296172347   -1.826264009525
H      -0.142441347471   -2.409658438760   -0.664235646835
H      -0.232452357509    0.918121959101    1.566677198508
H      -1.038663561046    1.075934373242   -1.615487749326
H      -2.757878925748   -0.700945226767    1.148927835065
```

```
N     -1.878277683642   -1.106347017731    0.873873172137
H     -1.791175639088   -2.101076011135    1.060530616199
O     -4.404250127190    2.287694373564   -2.478972028073
H     -3.940914327737    2.930072374920   -3.027298984186
H     -3.718921143395    1.851368943691   -1.951573358499
O     -3.509933293236   -0.960465052467   -2.333999216291
H     -3.539340546951   -1.073094835818   -3.284762174004
H     -4.400716503383   -0.632886755030   -2.073778133454
O     -0.904562149001    4.939336221241    0.944893932920
H     -0.408571654566    5.311953965929    0.195643015927
H     -1.795428897730    4.780570916258    0.612099844185
O      1.820153264509    4.847785339872    1.663590515653
H      2.034085429725    3.935509847835    1.866182456299
H      0.859216891852    4.924315198048    1.756657514500
O     -3.630352971084   -3.401111678000   -1.017208512113
H     -4.381617620872   -3.142263245988   -0.452908919247
H     -3.419566463417   -2.597179791572   -1.522054681039
O     -2.309237915732    4.197142292287   -3.056503635366
H     -2.504763014936    4.282697328560   -2.104152194397
H     -1.699368867864    4.936450713179   -3.267227381689
O     -5.660124013740   -2.140990405371    0.355892009973
H     -5.299625022292   -1.327101471748    0.733019327832
H     -6.204530318397   -1.860780578382   -0.385586541691
O     -1.718013128660   -3.956754970580    0.762325400935
H     -2.051549932170   -4.672171731122    1.304555177843
H     -2.375467652873   -3.842968572015    0.035529149463
O      1.515358285052    3.774939669009   -2.927952415951
H      0.779927607512    3.142378934483   -3.101342188676
H      2.293609432743    3.422741211192   -3.360119768704
O     -0.593807480550    2.262024200186   -3.577298177915
H     -0.677202144886    2.098631416597   -4.517709658341
H     -1.287759644316    2.939671415609   -3.360053947675
O     -5.878066860222    0.139816927926   -1.597877864588
H     -5.669215878755    0.345343732293   -0.672402750700
H     -5.693609096657    0.954533047400   -2.086731201347
O     -0.285597046367    5.943713372345   -3.393239552246
H     -0.101791273846    6.383538575088   -2.555296863349
H      0.427335003117    5.294315857671   -3.481163412630
O     -3.160671348946    4.022944532290   -0.450520222976
H     -4.105548715092    4.027026996622   -0.622645570452
H     -2.905747697515    3.080486053528   -0.375308903313
O      1.035458444234    5.703356739056   -0.895810691551
H      1.581709737372    5.631276442216   -0.098776630084
H      1.307420780145    4.977009130866   -1.474886276833
O     -4.739040569916    0.503297770562    0.939375623644
H     -4.917226334825    1.136785932479    1.635182129381
H     -3.929536856893    0.818997972584    0.464650354947

Ambimodal TS Water15-30
65
ANGSTROM
C      0.210389831677    1.587251725605    0.845782585764
C      1.308420590416    1.507913857313    0.038482334870
C      2.032741500358    0.299639300019   -0.269400470470
C      1.697453279189   -0.950215861123    0.206914950865
C     -0.208271823504   -1.405996979818   -0.885126088297
```

11535	C	-1.172519045247	-0.632698670621	-0.248343030511
11536	C	-1.514391270677	0.720230649665	-0.705215825736
11537	O	-2.536916050249	1.339003877722	-0.328834593036
11538	H	1.604879878177	2.402772967069	-0.509390390925
11539	H	-0.300662937059	2.530105691500	0.980281407474
11540	H	2.776004663476	0.371398118912	-1.064621230071
11541	H	2.264035588436	-1.820409685035	-0.104309659083
11542	H	1.163883723059	-1.072911226303	1.141053110451
11543	H	0.155464087604	-1.116975707738	-1.860193439107
11544	H	-0.133179799092	-2.462823086788	-0.664569002456
11545	H	-0.058617511045	0.809874802714	1.545520620384
11546	H	-0.963401127330	1.110016906734	-1.581250471294
11547	H	-2.520263946969	-0.462502236645	1.281845903620
11548	N	-1.782599860693	-1.047337969739	0.920440822882
11549	H	-1.917898387545	-2.027200326610	1.113523765370
11550	O	-2.642253201314	3.453969853002	5.546339227314
11551	H	-2.975477364652	3.739160746580	6.396622358105
11552	H	-2.020795153317	2.695237344532	5.723505218392
11553	O	-3.756559117703	2.207949152381	3.375119334437
11554	H	-3.483646201687	2.726165005305	2.596586708802
11555	H	-3.511788993146	2.734905525741	4.155958688484
11556	O	-5.781969725501	0.450083009797	2.850975155537
11557	H	-5.212154300460	1.190058250062	3.124012340580
11558	H	-5.554789966353	0.296599676822	1.916832624241
11559	O	-1.334719333887	5.740865895245	0.579507949283
11560	H	-1.319609282484	6.100650370152	1.471511731176
11561	H	-0.430584156575	5.394948319366	0.425517491008
11562	O	0.115621577052	-0.852743833423	3.308631442465
11563	H	-0.829597946887	-0.593335891485	3.365628257990
11564	H	0.214121268109	-1.609586431319	3.888198376851
11565	O	-1.079725320039	1.364793024189	5.830666150219
11566	H	-1.555528678061	0.749987755020	5.240148110230
11567	H	-0.236014430847	1.541494061758	5.382559162383
11568	O	1.229265512862	4.827948778862	0.535460567490
11569	H	1.271858888022	4.475439726287	1.456370635916
11570	H	1.870134798677	5.538850872389	0.499494742711
11571	O	-4.796198324415	0.025771017602	0.311189289643
11572	H	-3.988097707551	0.557528633751	0.126756830458
11573	H	-5.398821287545	0.198972739446	-0.412778754902
11574	O	1.189036359717	1.447907306402	4.222585622726
11575	H	2.094775460255	1.291072491119	4.491265004035
11576	H	0.874275559497	0.627027140714	3.783715097971
11577	O	-4.092765878192	-2.628106591980	1.397450084708
11578	H	-4.266478502743	-2.457329947207	2.334097197004
11579	H	-4.452503581571	-1.864626346742	0.929257539160
11580	O	1.182730032315	4.057439147659	3.089376316829
11581	H	1.190923763468	3.140148079551	3.396026920289
11582	H	0.287622630446	4.393719728284	3.302365737848
11583	O	-1.401049953276	4.771247774436	3.452527009681
11584	H	-1.850363132353	4.294165206937	2.737488956749
11585	H	-1.798670473339	4.445747758686	4.274231082176
11586	O	-2.381563131468	-0.043949242129	3.870786526793
11587	H	-2.846489370276	0.773437678886	3.584258425714
11588	H	-3.082287961927	-0.710965985214	3.980476170441
11589	O	-4.612783724962	-1.602356948029	3.982253288526
11590	H	-5.144788606343	-0.854156480450	3.589791095268

```
H      -5.129898687233   -1.952360986667    4.707253156697
O      -2.739250390375    3.563762717415    1.265214270869
H      -2.379569709082    4.368893164675    0.832775310963
H      -2.680007583802    2.853526947519    0.607263221379

Ambimodal TS Water15-31
65
ANGSTROM
C       0.179917660663    1.621086667248    0.810578484052
C       1.290324845460    1.470412184639    0.034979410006
C       1.989252481859    0.225770027971   -0.194650988725
C       1.609210016444   -0.984195144374    0.334103828265
C      -0.288583935348   -1.393134686743   -0.857782619113
C      -1.286335584697   -0.638742141007   -0.254651604973
C      -1.584372823478    0.711485104396   -0.723733145483
O      -2.560529267699    1.415885176638   -0.343392740524
H       1.625672723136    2.322344323363   -0.558057090074
H      -0.311259289369    2.581077677911    0.901611417162
H       2.752539173357    0.235601329859   -0.971214589188
H       2.153112150989   -1.883890334841    0.072167078860
H       1.026302060542   -1.059395523548    1.243648100102
H       0.143628085563   -1.066041995486   -1.790688350804
H      -0.202229496059   -2.450018569026   -0.642983169441
H      -0.134215908436    0.875854712010    1.528039892804
H      -1.040472918893    1.041057877880   -1.622207693581
H      -2.862801748015   -0.692990477666    1.082986245497
N      -1.959517014035   -1.086825275621    0.874044548976
H      -1.852274948201   -2.048464526131    1.154292143731
O      -2.043337277460    0.648490773922    3.945408353647
H      -2.637941146829    0.981344313851    3.212742587348
H      -2.047942364462    1.335285194790    4.612667739794
O      -3.648953789408    2.940067719091   -2.418151532414
H      -3.379886821007    2.359957929271   -1.686674787432
H      -3.121201562107    3.746410137394   -2.315745068477
O       0.749646203531    0.714656084973   -3.365563309716
H       0.841502401263    1.543027133524   -2.871414005883
H       0.355642974242    0.990165878074   -4.205316493117
O      -4.901156647682   -0.988240703539    2.047446870197
H      -4.378109561832   -1.388691068984    2.785939977157
H      -5.804348092135   -1.281745932486    2.163896705227
O       0.136784121048   -0.900962091017    3.471584968984
H       0.679922037633   -0.769944876126    4.249691376340
H      -0.633068533536   -0.304048499810    3.564140376663
O      -2.371755492435    3.961046808263    0.702490475761
H      -2.432160957114    3.071109805525    0.312623392190
H      -3.139000092431    4.043309360955    1.271513297381
O      -3.729494503734    1.454375571467    2.117260923967
H      -4.344565660149    0.697966454412    2.115445788766
H      -3.316263416378    1.436400918369    1.230773252415
O      -0.513788099524    2.365373988220   -5.283303914655
H      -1.463716340454    2.174172290394   -5.102154511889
H      -0.432064908643    2.466143522083   -6.231680075736
O       3.287003750169   -0.385502139572   -3.354192018262
H       3.249569304530   -1.127944942447   -3.958090610867
H       2.393898210217    0.004879077808   -3.354206686413
O      -1.809470577691    4.917793458536   -1.782986062265
```

```
H     -1.940479645621    4.769602602497   -0.828800241325
H     -1.162253614268    5.631816582637   -1.863989158418
O      0.693349912671    6.117771029761   -2.292239681474
H      1.231011279502    6.579513867145   -2.935759483265
H      0.812738587561    5.164686559290   -2.460541511178
O     -1.166382531985   -3.194154705408    2.831747746998
H     -0.730969982153   -3.942587157206    3.241027066171
H     -0.604146638276   -2.409394115771    2.998599884548
O     -3.097451032337    1.925218101079   -4.800919611902
H     -3.309472778569    2.246692468849   -3.889087315856
H     -3.791287687001    2.273178713739   -5.362062337202
O      0.269196453546    3.431533792610   -2.809522240353
H     -0.565350471449    3.728153383434   -2.400403897641
H      0.050845864618    3.256009959954   -3.737535404268
O     -3.307093091932   -1.853376913090    3.988046094155
H     -2.852464037030   -1.005109643988    4.119791465593
H     -2.649892419838   -2.437872828618    3.572475726731

Ambimodal TS Water15-32
65
ANGSTROM
C      0.164623327417    1.815515267143    0.873352806583
C      1.331064911175    1.937626547250    0.182951463028
C      2.241941097012    0.859702346791   -0.125602689017
C      2.015387488666   -0.462854749032    0.202400253454
C      0.333870691218   -1.072558525697   -1.089090296986
C     -0.821070632983   -0.548936450641   -0.511670256288
C     -1.353941194395    0.765731678439   -0.912615398942
O     -2.441508631117    1.235073418024   -0.538165900275
H      1.564423728896    2.899356450602   -0.277789231034
H     -0.501121196924    2.657413070177    0.999920695289
H      3.037714592620    1.090038971931   -0.833289184443
H      2.729722385855   -1.215716427204   -0.111686374436
H      1.425781733487   -0.734477266442    1.068399954733
H      0.722235571067   -0.629408896883   -1.992985424138
H      0.551056582699   -2.126278478351   -0.970536098916
H     -0.082740761181    0.947381208766    1.465252578490
H     -0.809675484791    1.281425005744   -1.727843514679
H     -2.411352780707   -0.915566416495    0.727016042992
N     -1.459613273225   -1.188061317618    0.530845962471
H     -1.301372065492   -2.178415860346    0.663097839475
O      2.107589450141    0.805529753509    3.693151061001
H      1.401288281919    1.444229172839    3.904806656421
H      2.462821028115    1.077509586861    2.844990610380
O     -4.371161836745   -2.060317965519    0.584887683159
H     -4.941930239281   -2.818076365601    0.460838803779
H     -4.740532293627   -1.554756302570    1.351565955564
O     -1.537591393523   -3.392270288954    3.721206153485
H     -0.750709171048   -2.851269390662    3.864392909957
H     -1.490973646079   -3.656452819886    2.785040391367
O     -3.336757123300    1.263815451672    2.009126339531
H     -3.167511927761    2.170881364132    2.314892490285
H     -3.095283917641    1.251801341378    1.058986575373
O     -4.434700699280   -0.460212272125   -1.631234665187
H     -4.020282861299    0.349392345440   -1.308518436100
H     -4.448113060475   -1.065219950872   -0.863810757352
```

```
O      0.645913592964   -1.508029529255    3.474509193350
H      1.314920954684   -0.832367818313    3.687453856336
H     -0.162345849167   -0.989109116309    3.295151173236
O     -2.288803262890   -1.340439949596   -3.060375470206
H     -2.581450578520   -1.493613421738   -3.959827451517
H     -3.092503605184   -1.062334733620   -2.572080589046
O     -3.805082289308   -2.329177715433    4.534827268136
H     -4.359431920966   -3.064961433383    4.795635919816
H     -2.974062358255   -2.724210660896    4.161361926441
O     -1.464373192403    0.150680046193    3.495780600706
H     -1.873618892229   -0.065916599472    4.359517438626
H     -2.190556052354    0.466813025208    2.905599024024
O     -0.168516615411    2.301791825669    4.221396802483
H     -0.644550824618    1.476977215135    3.919237578298
H     -0.329503467583    2.363006684746    5.164925265870
O     -0.911982679683   -4.040318707444    1.123590326213
H     -1.024484702208   -4.889210734609    0.696141554772
H      0.039291862485   -3.953680725509    1.353410222557
O     -5.154662278965   -0.696438142150    2.711391874211
H     -4.647806072677    0.115741915374    2.548511251634
H     -4.692348958422   -1.173682773344    3.419193092208
O     -2.255953999301    3.621337062038    2.956195093920
H     -2.017023067572    4.439974128079    2.521313598094
H     -1.450974169448    3.280247818354    3.388853934840
O     -2.389888907068   -0.516594046152    5.932698249367
H     -3.061574713915   -1.131296236259    5.578454928500
H     -1.747741699949   -1.058008935848    6.395333169752
O      1.579538003698   -3.534593004260    1.952712077868
H      2.302445660125   -3.170680748569    1.440668168943
H      1.291263073413   -2.824721613260    2.565203953017

Ambimodal TS Water15-33
65
ANGSTROM
C      0.167828665498    1.680982568493    0.794119605679
C      1.317364319969    1.540131064888    0.075495258072
C      2.042499720841    0.308430616068   -0.116406586975
C      1.607164195962   -0.922656675935    0.338967786722
C     -0.132282667227   -1.344271262342   -0.930962819705
C     -1.188691285518   -0.660614031920   -0.330421350665
C     -1.585951778657    0.692129081491   -0.755468998879
O     -2.584640737168    1.306072244356   -0.340767136117
H      1.675703588811    2.395310133359   -0.501842751994
H     -0.356456413124    2.628005453535    0.813684366601
H      2.861123976893    0.337456336525   -0.835746707007
H      2.180405466914   -1.813701999791    0.113374784634
H      0.978179652753   -1.005440264757    1.217345968241
H      0.282108063019   -0.978225394260   -1.859233857814
H     -0.017129242432   -2.407792443551   -0.766426945430
H     -0.172958845655    0.945547580610    1.509688722194
H     -1.041263674228    1.101580248100   -1.631072558357
H     -2.694985305307   -0.761540300665    1.076606629458
N     -1.833896943357   -1.184884225723    0.766602355923
H     -1.716539121111   -2.156708085680    0.997576373207
O      3.387007482823   -1.991481226057    2.889445727359
H      3.323206444291   -1.179692702205    2.382324756712
```

```
H      2.585681888004   -2.027869789752    3.453172408109
O      0.925897342081   -4.459797702610    0.007052733279
H      1.172995779592   -5.034542624842   -0.718273487694
H      1.740281483074   -4.330715072323    0.543379830322
O     -1.220602349946   -4.794097529858    1.582967862181
H     -1.310917338120   -5.746997568170    1.632328401765
H     -0.457120903017   -4.615653717801    0.993864663376
O      1.125961503815   -1.989911039537    4.369352013495
H      0.489925961556   -1.329607494500    3.967269541377
H      1.229386212794   -1.741221047092    5.288733582690
O     -0.637567579040   -0.345794117124    3.390773769221
H     -0.911209427882    0.296771505121    4.063406217630
H     -1.417039531850   -0.930319206274    3.275471597633
O     -4.710084125546    0.086695961434    0.903233074061
H     -4.633827409360    0.303406138149    1.841594002827
H     -4.092654649200    0.668542397822    0.424137385773
O     -2.782682894725   -1.995324070720    3.563758905420
H     -2.248431191954   -2.652669793022    4.052568181180
H     -3.296185601983   -2.544132643068    2.929542097913
O     -3.006610960652    2.787935438412    1.983260538486
H     -2.851520661056    2.427912171051    1.097973583455
H     -3.390048513022    2.062383333594    2.501869605214
O     -1.667340643160    1.512974810286    5.188995340809
H     -1.763155457563    1.804731932063    6.095455002081
H     -1.417049658208    2.314188006687    4.659970092455
O     -1.025079360874    3.513146765061    3.598283332516
H     -0.230746704985    3.322875484719    3.095285847814
H     -1.780390152405    3.387538269752    2.973662924439
O     -3.746435591370    0.570101907967    3.560054053756
H     -3.557040435731   -0.383285371212    3.603366518030
H     -3.177624870033    0.953323644939    4.246535789470
O     -0.946970328855   -3.863889464742    4.242909352414
H     -1.012489677206   -4.218644824797    3.342817822325
H     -0.122499705446   -3.353554688838    4.273426853296
O     -4.596779273349   -2.264639273627   -0.407560866903
H     -4.680196771751   -1.401086962145    0.049624892024
H     -5.474222266973   -2.473765721833   -0.729719438417
O     -3.819444047637   -3.671661415346    1.770961745688
H     -2.992100927049   -4.135803764443    1.587613359703
H     -4.094178469491   -3.237261047886    0.939494317188
O      3.180758427950   -4.226425706574    1.427321549034
H      3.396641149336   -4.925055181520    2.046614419928
H      3.302218364368   -3.385642385612    1.924990389966

Ambimodal TS Water15-34
65
ANGSTROM
C      1.791927862549    1.093258776935    0.795885962808
C      2.969566843737    0.512069893510    0.422865493606
C      3.189724067226   -0.908402980420    0.297643225534
C      2.217856450447   -1.860633928375    0.509785881241
C      0.860618358570   -1.542615578683   -1.274705896920
C      0.081929760415   -0.443810891453   -0.931076734881
C      0.485551482056    0.928005990795   -1.276918023502
O     -0.240837530121    1.935859264859   -1.135923451877
H      3.785943792178    1.152914851123    0.082413326180
```

11815	H	1.684987511580	2.169564970255	0.838032494177
11816	H	4.129544252637	-1.215055719846	-0.163567557163
11817	H	2.438695521512	-2.910235465094	0.354598183443
11818	H	1.374805328432	-1.670245120477	1.161462824694
11819	H	1.665534259463	-1.436075752528	-1.985709898574
11820	H	0.451767302276	-2.540793465134	-1.194340890649
11821	H	1.012620427878	0.535473789741	1.295866263855
11822	H	1.390955018510	1.026226299852	-1.906985829679
11823	H	-1.655404357203	0.225692623437	-0.055347622301
11824	N	-1.054324436391	-0.570789215632	-0.172581902344
11825	H	-1.476301422733	-1.463782470420	0.009441354565
11826	O	-2.337317453686	2.240417058237	0.494897639800
11827	H	-1.462435097265	2.209035675505	0.053360232295
11828	H	-2.359052774128	3.064401925814	1.030805570976
11829	O	-3.752765070463	2.679440948472	-1.704568073145
11830	H	-3.268365169627	2.454212913623	-0.876047024912
11831	H	-3.106776509787	2.603920467268	-2.428426659081
11832	O	-2.967654848560	5.190151305178	-1.021590628595
11833	H	-3.412575795104	4.351504323764	-1.268883873736
11834	H	-3.224662483226	5.843375402222	-1.672326191132
11835	O	-4.071178774942	-0.112471708393	-1.712002643439
11836	H	-4.229048764419	0.845413306415	-1.672994648719
11837	H	-3.241762831798	-0.215579229548	-2.211622541835
11838	O	-0.388459759903	4.627840344320	-3.824966397527
11839	H	0.505582825255	4.491132261752	-4.139963772146
11840	H	-0.311950530945	4.768750032806	-2.843986661570
11841	O	-2.090197306479	2.606254048730	-3.938081375792
11842	H	-2.665289586059	2.832233567786	-4.670776358472
11843	H	-1.422732658825	3.336213660855	-3.881600921216
11844	O	0.141028352556	2.229133346295	3.767970299089
11845	H	0.148528363464	1.329455627619	3.397264902229
11846	H	0.356608259389	2.827332985261	3.039559386076
11847	O	-3.849121955749	-1.459143421519	0.586808999940
11848	H	-3.942193480541	-0.961341916530	-0.259763530733
11849	H	-4.699611761739	-1.870441888389	0.742218421446
11850	O	-2.489658557526	2.571349450478	4.136249366149
11851	H	-1.508280221987	2.480917123354	4.176920026112
11852	H	-2.800817390921	1.745734272857	3.741563894819
11853	O	0.232533231132	4.049555067842	1.555686478839
11854	H	-0.645242211131	4.323933234903	1.859600488139
11855	H	0.322216568803	4.429102149188	0.671461871686
11856	O	-2.850868897407	0.352266736220	2.365064946767
11857	H	-3.259698733930	-0.370342389051	1.853620463008
11858	H	-2.708511791590	1.066035421444	1.710618856925
11859	O	-0.328386538474	-0.374448403954	2.897070435771
11860	H	-0.352818144269	-0.872293777691	3.714900970313
11861	H	-1.266074081895	-0.150431195909	2.686897232788
11862	O	-0.297579661929	4.645730417709	-1.193840237199
11863	H	-1.231182173888	4.871749601096	-1.002060113328
11864	H	-0.241839343345	3.669890806462	-1.181113709623
11865	O	-2.461789395232	4.300622780015	2.181692165132
11866	H	-3.213313602232	4.892280949019	2.171747266631
11867	H	-2.536223390183	3.724461116850	2.995324620819
11868	O	-1.789445928012	-0.050449364856	-3.276721886378
11869	H	-1.785717198545	0.903231541107	-3.474067021498
11870	H	-1.875223048467	-0.504029822235	-4.116212516739

```
Ambimodal TS Water15-35
65
ANGSTROM
C      0.231525271092    1.654298899383    0.899614198539
C      1.297640275129    1.594472846737    0.056210678748
C      2.038048715587    0.397225187239   -0.274343852648
C      1.736096348786   -0.855189402070    0.215136938269
C     -0.155030837243   -1.411135214831   -0.797816656643
C     -1.154698142265   -0.639023731799   -0.215558199182
C     -1.526105788440    0.681585977963   -0.737553756728
O     -2.541304515260    1.320458218724   -0.406698124736
H      1.563444557305    2.490226912001   -0.509971469038
H     -0.321618396250    2.572394059794    1.048245374709
H      2.749561055751    0.483468343267   -1.096779108574
H      2.307208419067   -1.717353149316   -0.107619118441
H      1.236010189784   -0.976674172949    1.166499327258
H      0.203463473337   -1.178164955937   -1.788447526297
H     -0.043493392530   -2.446752042878   -0.505139943427
H     -0.015435523845    0.857564136787    1.586437706839
H     -0.931588168165    1.052889529050   -1.598202224289
H     -2.568368909503   -0.490973581030    1.266097930160
N     -1.794075377401   -1.044760481768    0.939042350643
H     -1.829150644615   -2.023992312364    1.189694581773
O      2.943236653177   -2.733494179577    2.758029562624
H      2.226813559274   -2.154045271668    3.080134560202
H      3.560813433631   -2.162072320266    2.300090083428
O     -3.070124235904    2.031276481833   -4.764385224397
H     -3.562555683596    1.856554795742   -5.567623865405
H     -2.382481137432    1.350182816174   -4.710451809190
O     -4.877978550279    0.001563309245    0.051238833302
H     -4.018937974793    0.469372142143    0.015249376803
H     -5.351858303435    0.326149524273   -0.719985616567
O     -0.148748277734   -2.436675365908   -4.164260750445
H     -0.608323951182   -3.020626622631   -3.520990415834
H     -0.014702390090   -2.959710057802   -4.955660582719
O     -1.476614091030   -4.231102223345   -2.630566617896
H     -2.396834753644   -3.849080684329   -2.498622050018
H     -1.612049318210   -5.059099288925   -3.094641676162
O     -2.700814380705   -3.749809736297    1.557982722092
H     -2.225881756334   -4.362658392866    0.948149721235
H     -3.487788990023   -3.435464324502    1.084865897878
O      0.867173348366   -4.002555872673    1.384598430312
H      1.742231389572   -3.758814215408    1.722276181728
H      0.259963767244   -3.786818941359    2.108079787029
O     -4.465234157692    1.696578571748   -2.388313988518
H     -3.839238630818    1.933352152404   -1.686171703701
H     -4.076904070195    2.032769376758   -3.213593213918
O     -3.865032681045   -3.262115674235   -2.370247284715
H     -3.697390415661   -2.323590633688   -2.592200382803
H     -4.192604380955   -3.229613390529   -1.456363046640
O     -3.234249616124   -0.698123600828   -2.709426605655
H     -2.530256311973   -0.448946931998   -3.322847598561
H     -3.812198809193    0.084830377150   -2.616764133988
O     -1.153329120954   -5.206576761165   -0.048832292833
H     -0.311301745823   -4.937579427032    0.351524330890
```

```
H     -1.166954800043   -4.820437339850   -0.940946956153
O     -0.955152933849   -3.392082351398    3.505566596739
H     -0.984111410160   -4.173719963925    4.058672982069
H     -1.696836760117   -3.479104359435    2.867749228166
O      0.752881490276   -1.227153700698    3.513282342292
H      0.694465503210   -0.844010269636    4.389367611762
H      0.082311820594   -1.931846218337    3.477878100796
O     -1.085836269196    0.058596910319   -4.381056374418
H     -0.769966254793   -0.873319473639   -4.374999889555
H     -0.370435348156    0.573065676586   -4.002009210511
O     -4.815433142779   -2.661760612472    0.158310967188
H     -4.777639584453   -1.674597612275    0.115127932439
H     -5.709410511145   -2.881045818051    0.423063764830

Ambimodal TS Water15-36
65
ANGSTROM
C      0.183576277685    1.544526779987    0.828986418637
C      1.270656243735    1.558165828279    0.005615513185
C      2.170462524832    0.449911223601   -0.203402688035
C      2.019003867282   -0.788471506860    0.384835006907
C      0.220189748148   -1.615271698920   -0.647575593129
C     -0.883184973286   -0.995948883483   -0.068988469860
C     -1.438401180471    0.260072245974   -0.588605358905
O     -2.501266703873    0.769562931916   -0.174050311395
H      1.427085595866    2.426332932467   -0.638417122116
H     -0.477187114493    2.398917340150    0.901772384605
H      2.891139030925    0.559388743383   -1.014592563530
H      2.711338143450   -1.588270388242    0.148406210981
H      1.511451015537   -0.900660819385    1.333918526405
H      0.546357943757   -1.314482998309   -1.630991381403
H      0.468869861318   -2.632907825763   -0.375284792905
H      0.049227902276    0.796690710180    1.596976369027
H     -0.987802154180    0.657782214220   -1.516700351323
H     -2.372467086886   -1.072927619320    1.334238495537
N     -1.485140114451   -1.474249357550    1.070129579416
H     -1.283030015193   -2.392119827006    1.425533016032
O     -4.289812518320   -3.103867290115   -1.138324173333
H     -3.622232886625   -2.519146239173   -1.573672650882
H     -4.355546134192   -3.894058843494   -1.675529620818
O     -0.089532518422   -3.089220702675    4.354760726525
H     -0.706361796680   -2.905404729129    5.071002474532
H     -0.628570642718   -3.491474086196    3.657321204908
O     -4.517559381965    0.078003804031   -3.351195905377
H     -5.287764543022   -0.389523240818   -2.950453222249
H     -4.473811217944    0.936378645930   -2.909897156364
O      0.515719775299   -0.639238819652    3.444495034351
H      1.135855739348   -0.235135176144    4.052717831050
H      0.398374079567   -1.569655179752    3.740469544389
O     -2.647335940270   -1.541608721024   -2.528720076070
H     -3.320123122526   -0.869134947078   -2.843505337956
H     -2.278614955170   -1.920545788659   -3.327693898783
O     -3.156556350869    2.149815471856    2.207159623900
H     -2.736454277786    1.662545505138    2.926613683349
H     -2.947856752685    1.653630975875    1.400534612782
O     -5.773211932115    1.397665949804    2.687780229860
```

```
H     -6.136615862984    1.339025116071    1.792887565353
H     -4.948961867940    1.913522978846    2.603575958601
O     -4.196338725804    2.260254609290   -1.560148274084
H     -3.466890377996    1.783620413178   -1.096968494511
H     -4.122662455159    3.182813797942   -1.313782549348
O     -4.177015499655   -0.716363788683    2.316319939378
H     -4.903742779559   -0.090986743955    2.533372712749
H     -3.448342579260   -0.444260133265    2.906563301214
O     -2.882009142848   -2.460377625456    4.870252863071
H     -2.709077490363   -3.008021300224    4.081950396507
H     -3.834931475220   -2.368749734354    4.926160314796
O     -5.668785135396    0.569449991196    0.029428774223
H     -4.907545041757    0.115437539126    0.414256700532
H     -5.296746280613    1.242537339692   -0.567275258816
O     -2.139145204936   -4.052631287754    2.687652426930
H     -2.982342642639   -3.776513825110    2.211026965576
H     -2.211272758256   -5.000818738908    2.804208035980
O     -4.351991664750   -3.283815187530    1.617456285826
H     -4.364193535717   -3.256756368421    0.646301301858
H     -4.417140278125   -2.346839318423    1.899605481279
O     -6.310433471775   -1.269600304096   -1.877943510872
H     -5.759429786024   -1.999331879716   -1.556750028013
H     -6.326575810323   -0.624233149011   -1.146646811231
O     -2.096312693182    0.020190632862    3.914079709560
H     -2.298410475378   -0.739970492580    4.484814207372
H     -1.173942140416   -0.115130593291    3.626168539816

Ambimodal TS Water15-37
65
ANGSTROM
C      0.205946660281    2.387440739527    0.492330849522
C      1.188637366844    2.318437129938   -0.452566073133
C      2.131111180571    1.238084856734   -0.596072248221
C      2.120032557078    0.088676079443    0.168874759265
C      0.291494985657   -0.903219533351   -0.582977800410
C     -0.775299324857   -0.297691958577    0.068328328568
C     -1.479186558147    0.859587264449   -0.474241882902
O     -2.522412099896    1.347781812379    0.039924536565
H      1.190243118003    3.062683271414   -1.248166210580
H     -0.498230281960    3.209134347269    0.495968463375
H      2.755020598448    1.258148978130   -1.489901096268
H      2.834861091265   -0.701976194713   -0.026979774194
H      1.727542766354    0.089826524533    1.177690595207
H      0.478544260992   -0.675890251822   -1.621676851628
H      0.627433385411   -1.880913461230   -0.261875683874
H      0.220470122355    1.794937672035    1.396565399943
H     -1.199554847545    1.182654492527   -1.489399266257
H     -2.119291862332   -0.503160104716    1.592049461607
N     -1.158558177520   -0.720780999512    1.356391007883
H     -0.902703990732   -1.673398763822    1.587142239014
O     -3.214991884282    0.090710879872    3.738714634755
H     -2.995742569509    0.994914158363    3.375739530994
H     -3.468578084015    0.227434012567    4.651818816599
O     -1.991304058076   -3.270707930299   -0.402095542030
H     -2.763129261009   -3.161395955748    0.218747147317
H     -2.369342091385   -3.487803408097   -1.254126808502
```

```
O      -0.772892353983   -5.121664412249    1.087663275964
H      -1.143738256479   -4.488194901749    0.430717467641
H      -1.514516275549   -5.649279590187    1.390388118882
O       2.140270917168   -1.536698830699    3.467244425568
H       1.779729487089   -2.026951989388    2.720946590918
H       1.498436306046   -0.811483968508    3.628213158005
O       1.156809633798   -3.209480578292    5.276738225347
H       1.669096729774   -2.574943554315    4.714849455982
H       1.769790078093   -3.571018471938    5.916326460770
O      -3.065731468277    3.728720178534   -1.162390734811
H      -2.947242055059    2.885611240206   -0.680576304234
H      -3.966088664298    3.722202356054   -1.490126959356
O      -1.005429204343   -1.509598606925    5.661853282308
H      -1.737977660023   -2.000421172625    5.257684552658
H      -0.266498923410   -2.139190353194    5.709098498113
O      -2.712224872416   -2.614061404759    3.692280431082
H      -2.887541636364   -1.660530782563    3.629427538586
H      -1.819495143035   -2.770489666220    3.320781636626
O      -2.690251705064    2.388738630759    2.646297604953
H      -2.598811829823    2.179843593176    1.704074332454
H      -1.815192515428    2.685557361955    2.963261915913
O      -0.945285570062    3.347964673619   -2.914575451863
H      -0.858938611620    4.132700786438   -3.457452527258
H      -1.669667291349    3.536472487369   -2.290578409213
O      -3.980762749417   -3.014180952464    1.285818651029
H      -4.308436405904   -2.103341281383    1.167748418756
H      -3.657150710920   -3.048931677620    2.206089843320
O       0.024861362134    0.098957166023    3.789338919199
H      -0.409295058578   -0.392449669978    4.528516260217
H      -0.471951664185   -0.133560283287    2.981768195338
O      -0.213488411735   -3.147752972709    2.780537447817
H      -0.319657383376   -3.937033631229    2.203117484966
H       0.180208709710   -3.453159222052    3.608325097126
O      -0.253503016054    2.779968621698    3.775455960469
H      -0.056485687274    1.823742269927    3.866159840808
H      -0.174872478877    3.161334592592    4.649957854533
O      -4.436116842538   -0.323600342599    1.245636083266
H      -3.931055565420    0.334274207860    0.743489069001
H      -4.316825596409   -0.101288500970    2.180105593844

Ambimodal TS Water15-38
65
ANGSTROM
C       0.111608001465    1.294365360835    1.025370419876
C       1.237496133993    1.325752919318    0.264565093337
C       1.976869588550    0.168390908561   -0.206687686114
C       1.634434474435   -1.133982923384    0.043005427189
C      -0.289673166585   -1.376856781161   -1.259866361934
C      -1.280918606076   -0.695202300772   -0.566570180437
C      -1.527549983727    0.725606196350   -0.833479486273
O      -2.514896087826    1.378769723488   -0.419491169421
H       1.568203008693    2.288323979043   -0.129253378146
H      -0.405146487996    2.209408698556    1.284645994219
H       2.759574552712    0.372878600135   -0.939357519578
H       2.202561689635   -1.947564587701   -0.391077626724
H       1.012981343006   -1.421625717463    0.879613081885
```

```
H      0.174563137463   -0.938748599236   -2.129890163018
H     -0.214014747564   -2.451426868778   -1.174443697909
H     -0.208441705891    0.411561617097    1.562767665150
H     -0.890073440934    1.197153545363   -1.605080715954
H     -2.685535666506   -0.789095634364    0.961305668170
N     -2.059621126234   -1.319282745299    0.376774238935
H     -1.786451460303   -2.227779528339    0.730665145712
O     -2.588261294684    4.083114682707   -1.031092181017
H     -2.600721733156    3.116311323674   -0.928324366408
H     -3.515327258986    4.365004087302   -1.001856092354
O      0.344645041361   -1.576132665758    3.284511156145
H     -0.057296465939   -0.909492993656    3.860064207269
H      1.297784196297   -1.373973009022    3.284484423343
O      0.068815317984    4.086691515334    3.663330733804
H     -0.319337871374    4.313912558277    2.803731862718
H      0.732793893056    3.401922987194    3.496559512701
O     -4.530070296404   -0.412538504620    1.977298175715
H     -4.947282894369   -0.181829872227    1.130145026211
H     -5.182441400926   -0.888888274610    2.490756455600
O     -5.331418477897    4.530171341171   -0.797177619029
H     -5.898929158379    5.298784263251   -0.741537162590
H     -5.510690827424    3.985381912504    0.019334565790
O      2.893962522484   -0.533932453433    3.153127317506
H      3.184074482957   -0.492315763281    2.240203440799
H      2.577772883280    0.361657739158    3.381485125609
O     -5.728209683409    2.906271955794    1.214886496726
H     -5.769394083567    2.092083265074    0.675704930093
H     -4.911754167449    2.802478867992    1.736238783198
O     -1.957440810071    2.552637175742    4.535415564809
H     -1.273831954097    3.226902522472    4.317629981291
H     -2.467504580906    2.416783159698    3.719094626704
O     -5.197325424917    0.771369715950   -0.412168099630
H     -4.237305017349    0.932735572455   -0.451064599589
H     -5.542332899206    1.170951206030   -1.232675688903
O     -1.546194706705    4.309915164494    1.450675847540
H     -2.376497074963    4.231494087069    1.926593818552
H     -1.787620654496    4.369871821248    0.502967855676
O     -0.831017862604   -3.501498568989    1.757413365590
H     -0.377009806332   -2.841864933690    2.316044799933
H     -1.118554103965   -4.195300088785    2.351451709307
O     -0.403561721028    0.540286541311    4.961688651241
H     -1.073406440057    1.273125431708    4.795093897851
H     -0.443794717392    0.356103396619    5.900608215333
O      1.747545437155    1.817148265962    3.974551192267
H      2.201868535368    2.172670680791    4.740780988678
H      0.960164502451    1.333906536905    4.319926799069
O     -3.328092548029    2.026582355136    2.184769834613
H     -2.877723604986    1.944056100796    1.328517956858
H     -3.714562511420    1.147040821747    2.343641827658
O     -5.643331709680    2.426346627156   -2.522550108945
H     -5.598747633656    3.240886078591   -1.984230899279
H     -4.948907191756    2.503538284658   -3.178476011797

Ambimodal TS Water15-39
65
ANGSTROM
```

12151	C	0.146739318213	1.771585814712	0.813008609067
12152	C	1.272962976934	1.622773001185	0.058918222722
12153	C	1.992960252475	0.390961824672	-0.145617556215
12154	C	1.621181019286	-0.833324411655	0.371616999413
12155	C	-0.203419942009	-1.307906152249	-0.790526186665
12156	C	-1.220900191951	-0.560283117643	-0.204151069243
12157	C	-1.577816678166	0.762606597019	-0.711564103312
12158	O	-2.596596227153	1.406332550239	-0.349201941977
12159	H	1.610611169851	2.471289889250	-0.532376091842
12160	H	-0.367087264983	2.722597176186	0.865265519712
12161	H	2.763090085088	0.411987230656	-0.915641424764
12162	H	2.196626045156	-1.718981004502	0.128835905216
12163	H	1.059201641192	-0.917643381895	1.294058679041
12164	H	0.192143372935	-1.002848401518	-1.748388908864
12165	H	-0.117261347801	-2.362992719163	-0.563251161764
12166	H	-0.172093297025	1.040737351525	1.541722594568
12167	H	-1.027579594610	1.105256240058	-1.601851720366
12168	H	-2.706212364592	-0.521601576308	1.226079958010
12169	N	-1.852381923348	-1.000298606865	0.958358965683
12170	H	-1.885952098985	-2.003708100920	1.126506254818
12171	O	0.683024513114	1.897926579669	4.200012293636
12172	H	1.156938548719	2.170004048672	3.411557156515
12173	H	0.543481797652	0.942869724249	4.125275119748
12174	O	-3.073204755848	2.974594961141	1.880439137433
12175	H	-2.579895941970	3.752109606714	1.574225753156
12176	H	-2.988755256444	2.323309078576	1.165200495406
12177	O	-3.098480873559	3.660993321170	-1.590867493801
12178	H	-2.917865962027	2.790337081207	-1.138651359710
12179	H	-4.039767004174	3.814270903580	-1.500636104414
12180	O	0.489985832143	1.736655111577	-3.237594707482
12181	H	1.299895929707	2.258808529729	-3.173452646402
12182	H	-0.196133002884	2.361998640626	-3.506706803876
12183	O	0.613889381988	5.397564769386	-3.229864399062
12184	H	1.367798555926	4.793681542279	-3.333282994150
12185	H	-0.170744394307	4.867768309654	-3.454550679369
12186	O	2.704733947189	3.577538093124	-3.046730335138
12187	H	3.545209462248	3.428206345074	-3.480508075972
12188	H	2.901116466012	3.838927605847	-2.122103907990
12189	O	-2.137133497164	1.775953052863	4.067894995571
12190	H	-2.447143752659	2.331297614787	3.315635306316
12191	H	-1.293440928792	2.155059420214	4.351812897152
12192	O	3.067088557850	4.535074971104	-0.544617246433
12193	H	2.204777543975	4.991760612265	-0.451126492487
12194	H	3.738596193787	5.153709788650	-0.255190861917
12195	O	-2.828323944029	-2.318936614906	4.027863456439
12196	H	-3.464155812334	-1.679402720058	3.679147857882
12197	H	-2.002137684053	-1.816266647608	4.133224647071
12198	O	-2.032615992419	-3.697767762427	1.867470366614
12199	H	-2.419491918060	-3.320107040981	2.690980765137
12200	H	-1.420902257351	-4.376911286175	2.153086679776
12201	O	-0.748071243383	-0.522332150715	3.537007952049
12202	H	-1.319408717602	0.224943996724	3.804689260696
12203	H	-1.017382888913	-0.733837485510	2.625010160533
12204	O	-1.507904885078	3.728954079997	-3.763657419367
12205	H	-2.082134299695	3.767818270764	-4.529170087622
12206	H	-2.086505648108	3.715059465282	-2.972842832561

```
O     -3.945600217959    0.015172972211    2.834941070065
H     -3.414546640717    0.593942544062    3.403482956172
H     -4.663184567076    0.562477533320    2.512520625953
O      0.725171258952    5.898868715201   -0.663611341873
H      0.695453335573    6.850766955429   -0.558183686521
H      0.622313658312    5.726051636927   -1.642350880478
O     -1.610691613190    4.845603378853    0.465688926386
H     -0.748537888073    5.180181970390    0.175692072822
H     -2.082191872410    4.591870212821   -0.342974725557

Ambimodal TS Water15-40
65
ANGSTROM
C      0.019995249972    1.580629721769    1.063069694433
C      1.202920537198    1.559436687958    0.386009664667
C      1.991874523085    0.386316979440    0.089636900286
C      1.640156168975   -0.898258510504    0.436107318175
C     -0.117604475091   -1.323739421494   -0.914164633300
C     -1.166438680034   -0.594460256858   -0.363774172701
C     -1.464380709487    0.773162530922   -0.808157548979
O     -2.545888516542    1.355862500336   -0.569289270234
H      1.538075144895    2.485337368606   -0.083584092831
H     -0.533925785449    2.500977341888    1.188193556849
H      2.813256375687    0.524942538048   -0.612775652756
H      2.260099585266   -1.734631608249    0.136924970262
H      1.005110072062   -1.101970996716    1.289419498272
H      0.351299084534   -0.978125570309   -1.822989766725
H     -0.055268788266   -2.390337991430   -0.737556233479
H     -0.321967298578    0.758487773005    1.676882305182
H     -0.780375565573    1.213908305360   -1.550953478013
H     -2.552724978491   -0.487980197972    1.179433758548
N     -1.987334933166   -1.106482414692    0.613412676677
H     -1.861145039644   -2.039123901066    0.965276378721
O     -4.695779612180    0.374087936460   -3.590789177957
H     -3.955880541344   -0.240766882461   -3.789353470482
H     -4.889679229879    0.241872590756   -2.645441740203
O     -2.551907334766   -1.234237813789   -3.790427023629
H     -1.694216236377   -0.767695472015   -3.770683181555
H     -2.607223979943   -1.760155020407   -2.983653993184
O     -3.521466159813   -2.670889977133   -1.533933657833
H     -3.452275133108   -3.026092965494   -0.618250160316
H     -4.147153173095   -3.226967716758   -2.001620372281
O     -3.312738850118    3.657726265693   -1.690029222501
H     -3.608863788012    3.398099915569   -2.582412343951
H     -3.057254018108    2.815166189983   -1.245866063070
O     -4.797512273944    2.400591922150    1.413419835280
H     -4.331487418650    3.262546678257    1.329277920854
H     -5.020235094389    2.127844510104    0.520524344322
O     -3.638146969336   -3.521015029071    1.007072806452
H     -4.121857698969   -4.313281360481    1.242799881020
H     -4.217297181191   -2.756753052701    1.283205700973
O     -3.505329847549    4.696746364977    0.866489988250
H     -3.615342005008    4.561185159155   -0.085591873155
H     -2.544459976521    4.691789192254    1.009197598201
O     -0.188538638336    0.147978437920   -3.898826107990
H     -0.456125735902    1.094378726269   -3.972542764400
```

```
H      0.199167282274   -0.074198754149   -4.746692382899
O     -3.753647885242    2.712736804859   -4.172254102508
H     -4.119806870526    1.803393519700   -3.954813557511
H     -4.259479976645    3.025180565915   -4.922229716597
O     -5.171913593016   -1.458229561769    1.555462653293
H     -5.249601353857   -1.011066452520    0.692167554385
H     -4.648464842348   -0.841566492577    2.097623826440
O     -3.489404887084    0.442793444782    2.585160900579
H     -3.375668238266    0.710127947003    3.496942236835
H     -3.944912859627    1.201812990715    2.135999655975
O     -0.979601373705    2.686421574445   -4.138633111544
H     -1.950299797089    2.675930868406   -4.215371506182
H     -0.773242680569    3.289502695560   -3.407637210144
O     -0.724212515039    4.809249109879    0.688068294387
H     -0.651174870336    4.680940840450   -0.277050042845
H     -0.282230838926    5.633206222255    0.894777626092
O     -4.698964728774   -0.266003739128   -0.892381375968
H     -4.262409395106   -1.101774865664   -1.149066170434
H     -3.970015107387    0.332771458720   -0.639374566610
O     -0.911360862932    4.612833880641   -2.060334099640
H     -0.986588045429    5.463214799797   -2.495860990641
H     -1.846304092316    4.291643927300   -1.912335734319

Ambimodal TS Water15-41
65
ANGSTROM
C      0.195775683237    1.669349827704    0.842624094202
C      1.304314007676    1.515554278850    0.067373547339
C      1.999420234660    0.271136551956   -0.178562960622
C      1.593107585504   -0.955959229820    0.302008440117
C     -0.245396670065   -1.374210476518   -0.860952095565
C     -1.231188179735   -0.603824853924   -0.249506069684
C     -1.601883655845    0.721551145655   -0.762136604299
O     -2.601210784126    1.373324213732   -0.405189875092
H      1.652259560022    2.374069381200   -0.510148708970
H     -0.300286790249    2.627079184736    0.926268675489
H      2.777153409140    0.300101138563   -0.941558134113
H      2.143631900431   -1.853567767217    0.052663808803
H      1.005153691218   -1.043975817747    1.206667377818
H      0.136532510421   -1.083798702651   -1.829092089714
H     -0.171436668906   -2.429590344920   -0.634418634116
H     -0.136307110843    0.917824379662    1.545484620763
H     -1.035055015202    1.076347748537   -1.646761970615
H     -2.652288935398   -0.531751610262    1.233408251908
N     -1.818267377817   -1.010624728376    0.934497617053
H     -1.791649905316   -1.988937081669    1.195143038705
O     -4.647999126934   -2.043463658747    2.439807661371
H     -4.708883119296   -1.176559301519    2.013089827797
H     -4.526489867578   -1.856359835874    3.388444394810
O      1.346214981055   -2.945897383374    3.686735188188
H      1.497808094534   -3.305889962203    2.790663762594
H      1.715787189871   -2.044621380251    3.694731846340
O     -3.712692322301   -1.488323685317    4.956051538498
H     -2.864724302770   -1.921212746404    4.809774222286
H     -3.507735401907   -0.592491554919    5.273621416149
O     -1.102715805903   -2.684721668191    4.443114518729
```

```
H     -0.158985634578   -2.813108232978    4.111078232971
H     -1.123976745922   -3.144256520021    5.284473049271
O     -2.775283196030    3.699753401571    1.080728186313
H     -2.750296080556    3.192976384142    1.913059917154
H     -2.716929688337    3.033164953191    0.378606116699
O     -2.920756497787    1.067467008498    5.675732760583
H     -3.456799827193    1.577841009681    6.282262733399
H     -3.013096160000    1.488568267015    4.789386633641
O     -0.821469966278    0.059574350375    3.983428249672
H     -1.157775484445    0.415106245733    4.815174980633
H     -0.996602791816   -0.896618961634    4.020276994350
O     -0.835807378382   -4.576279121758    0.284318592457
H     -1.175195594525   -5.436862458001    0.036548321386
H     -1.461742749697   -4.222676864008    0.951079660548
O     -6.556124993968    2.224406596598    1.379658108350
H     -7.173866112099    2.126853622938    0.654188923427
H     -6.131384522908    3.104944423525    1.259642226442
O     -5.296339814742    4.551648829537    0.986890250782
H     -5.364343100487    5.239891542955    1.649456369916
H     -4.341337852954    4.297123723997    0.956340192207
O     -4.448413261499    0.592504431386    1.440241192707
H     -3.951285944402    0.899376330263    0.654921785936
H     -5.281142595074    1.115298534901    1.455200270377
O      1.888503072428   -0.249046946946    3.680404600217
H      0.954074502139    0.000182375139    3.813412949967
H      2.213585615022    0.277201387139    2.948560902775
O     -2.823932139884    1.836993683340    3.122999883939
H     -3.500475478956    1.341555565205    2.598563430277
H     -2.014772163012    1.305823667598    3.074217754018
O      1.663576287297   -4.109820549245    1.243616001518
H      0.773568604253   -4.249899704792    0.861472745491
H      2.055291860151   -4.978077996310    1.346389731956
O     -2.451407301821   -3.551047562646    2.156223448303
H     -3.333739169824   -3.117008857660    2.186872262072
H     -2.026928261915   -3.350078398655    3.004616476261

Ambimodal TS Water15-42
65
ANGSTROM
C     -0.729123289626    2.470156358596    1.265277758039
C      0.398224928384    3.232213785546    1.346627163891
C      1.671717817201    2.795439072736    1.867703083817
C      1.933018643598    1.516776375819    2.311086443612
C      1.904547309894    0.324139443021    0.415369833680
C      0.553857390950    0.151300819316    0.123414823952
C     -0.179096931876    1.073927360355   -0.759463463742
O     -1.319492676478    0.849551366661   -1.211768749067
H      0.391267556088    4.219677853389    0.880308204785
H     -1.631458776626    2.849940510938    0.803551729199
H      2.511880405192    3.473207932900    1.715062436727
H      2.929077283532    1.251041004491    2.644624661390
H      1.150177878548    0.882235802407    2.707049488661
H      2.500440031304    1.013838724159   -0.164294545910
H      2.455397445710   -0.485400115708    0.875925218062
H     -0.860673644878    1.557901213856    1.830369229029
H      0.403102947862    1.911409112334   -1.190985938893
```

```
H     -1.159835869510   -0.877560869681    0.640675091648
N     -0.153809598086   -0.909728694630    0.638485572073
H      0.271241765339   -1.494219489271    1.344123224786
O     -1.778143350141   -5.801264324097   -1.423752920321
H     -1.461700184424   -6.387632229209   -2.136382987037
H     -1.117531264184   -5.068381446484   -1.391310837265
O     -0.867155150888   -6.940200751626   -3.707143706586
H     -0.457859402150   -6.091432070242   -3.963507325538
H     -1.419163458954   -7.210992570420   -4.441502397103
O     -1.488262485853   -5.984410842743    1.299463529402
H     -1.660180385214   -6.163624286795    0.360380865996
H     -0.575692170616   -5.611224038351    1.310186936432
O     -3.664328517340   -2.608519254535   -0.418452224215
H     -3.966817851461   -3.283485221890   -1.050325221054
H     -3.414121789277   -3.093285283287    0.395240933381
O     -1.636179606592   -2.464966725760    3.579521883051
H     -1.896133869096   -1.548560472550    3.368746592177
H     -2.144013804875   -3.026716455197    2.964816356176
O      0.832354702418   -4.706647562499    0.983620862576
H      0.690460933044   -4.334524127481    0.101694510221
H      0.882093203373   -3.950782728330    1.595264902616
O     -0.092550473093   -4.391546929577   -4.291157380847
H      0.358605562653   -4.029630380598   -5.053566131979
H     -0.998792487230   -3.971562254796   -4.274904073034
O     -1.675874244667   -1.742791095804   -1.977803751615
H     -1.551479977722   -0.792949716570   -1.812612220918
H     -2.401854368322   -2.028712919929   -1.378609352778
O     -2.435902245770    0.031356714963    2.814211077016
H     -3.046162520887    0.460255133990    3.414161779901
H     -2.868750592833    0.049500210843    1.928237626850
O      0.845941189420   -2.610930451220    2.772718774962
H     -0.082132824798   -2.599500936518    3.138762203281
H      1.422830461599   -2.786934494118    3.515956839715
O      0.007754260863   -3.799957283197   -1.568073850979
H      0.265010931717   -4.013376104422   -2.477717685346
H     -0.513562474099   -2.973189821523   -1.632878736692
O     -2.435987658606   -3.235241976631   -4.101214657153
H     -2.179214007419   -2.528353179141   -3.478160867671
H     -3.087474608883   -3.760032401856   -3.608977106577
O     -3.057931530788   -4.009670175398    1.813949503388
H     -3.799607796573   -4.393244876983    2.282801658530
H     -2.426484408016   -4.776543461019    1.626674343028
O     -3.483767987400    0.061170560260    0.365073848236
H     -2.847739006636    0.427981318208   -0.272117458693
H     -3.728974219786   -0.819712208552    0.043767737460
O     -3.977675796309   -4.644786001119   -2.241411532565
H     -4.670609066887   -5.301657839092   -2.303526133206
H     -3.165906611460   -5.115545752455   -1.903297182502

Ambimodal TS Water15-43
65
ANGSTROM
C      0.110712068206    1.607595485082    0.855327444963
C      1.213736573484    1.540844155304    0.058784988541
C      1.949547741312    0.334279652981   -0.247163772985
C      1.612029543492   -0.912956156131    0.222516349777
```

12431	C	-0.286301526613	-1.387907532726	-0.933859788868
12432	C	-1.282294093230	-0.609287457867	-0.354829341943
12433	C	-1.571045756498	0.746930348906	-0.831882733599
12434	O	-2.571185819239	1.415042021747	-0.496253041757
12435	H	1.516078896833	2.434386763403	-0.493085738646
12436	H	-0.425455917584	2.537473124107	0.998240604527
12437	H	2.702431379375	0.416684820773	-1.032668092648
12438	H	2.181190529519	-1.783673855877	-0.080988705695
12439	H	1.051677377523	-1.034947555005	1.139402926473
12440	H	0.132761713799	-1.113315238203	-1.890070194469
12441	H	-0.215807462449	-2.437654377337	-0.682246356632
12442	H	-0.159847326558	0.816311139785	1.540191295413
12443	H	-0.952159541895	1.117848169712	-1.674237750307
12444	H	-2.570815137251	-0.387798843059	1.228356294115
12445	N	-2.056887889715	-1.070952526234	0.684785886398
12446	H	-1.754904702393	-1.903063150555	1.173433264499
12447	O	-3.723919456966	0.637569761580	2.397016062683
12448	H	-3.742358462940	1.624324564858	2.357502045513
12449	H	-4.641622817758	0.361265184492	2.258146298812
12450	O	0.423622421261	-3.751658517649	3.709378179693
12451	H	1.159066855502	-4.067443735633	4.233613845614
12452	H	-0.377147925091	-3.787952646717	4.286699252324
12453	O	-3.314719267095	3.995440695447	-0.668295774915
12454	H	-2.997697552833	4.357147155444	-1.495870035577
12455	H	-3.005782541063	3.064447983901	-0.634078472430
12456	O	-5.094715217287	0.451053467583	-0.535412166375
12457	H	-5.436062236907	0.055436991241	0.284525045941
12458	H	-4.194031233317	0.781363971452	-0.342813380319
12459	O	-1.909111612965	-3.683389022067	4.991855376674
12460	H	-2.240318934291	-2.818708925891	4.712422056416
12461	H	-2.464069609444	-4.333548545536	4.537192766273
12462	O	-3.486174188821	-5.056567447731	3.168610699348
12463	H	-3.887231160024	-5.824845996317	2.761899096264
12464	H	-4.073443330102	-4.293180713884	2.967645714400
12465	O	-2.893774052344	-1.138429023100	4.115662428913
12466	H	-3.128692026195	-0.401485943816	3.491639648129
12467	H	-3.131458917340	-0.815037045257	4.986291710860
12468	O	-1.450457543109	-3.844997155334	1.613825043858
12469	H	-2.064763056990	-4.357757859015	2.163354857828
12470	H	-0.620490856076	-3.824788297289	2.107310424418
12471	O	-4.114641657945	3.231827823976	2.113758003982
12472	H	-4.914195622451	3.197179377267	1.544674458234
12473	H	-3.466040732417	3.691647475438	1.573131681000
12474	O	-0.187678764122	-1.082740204553	3.065578922761
12475	H	-1.054287742189	-1.050800745799	3.488129709828
12476	H	0.175155405326	-1.955903379206	3.272469787378
12477	O	-3.804425331553	-3.483487900826	0.086287224325
12478	H	-3.868699852866	-2.821477621752	-0.632227240910
12479	H	-2.862167944250	-3.613801562212	0.248967928283
12480	O	-6.108077771951	-0.629053207314	1.807642477989
12481	H	-5.691159144963	-1.491251577994	2.047520953119
12482	H	-7.048955336765	-0.707672625047	1.961662019882
12483	O	-5.869678709852	3.082493904610	0.056349180102
12484	H	-5.781780427900	2.171733364998	-0.270689240036
12485	H	-5.162050410407	3.581189210234	-0.380164808827
12486	O	-4.623197337084	-2.736291121637	2.445039443994

```
H     -3.917876770878   -2.221439576473    2.866025172164
H     -4.317126610133   -2.955235634454    1.517659633223
O     -4.139796362560   -1.665530011282   -1.892878933852
H     -4.514662837313   -0.867293376460   -1.456479633953
H     -4.774099207907   -1.930770628010   -2.559622709615

Ambimodal TS Water15-44
65
ANGSTROM
C      0.220598097015    1.575431541534    0.867204176653
C      1.314871939665    1.500709485801    0.054734145858
C      2.024567359608    0.290600835882   -0.262199757959
C      1.677634275721   -0.963186437755    0.212172882895
C     -0.154866274686   -1.409954473897   -0.839314512135
C     -1.171643416155   -0.648181720364   -0.266259389295
C     -1.535467081465    0.679724654291   -0.754977927408
O     -2.526627160974    1.326420153099   -0.361461566207
H      1.636166567091    2.391771300524   -0.486126650035
H     -0.312484700678    2.505065984633    1.028568957033
H      2.754346956752    0.353958278409   -1.066060012427
H      2.263370675543   -1.820895184217   -0.098165157299
H      1.186099252645   -1.079956913385    1.170751082040
H      0.219730745698   -1.132510962807   -1.812995383247
H     -0.085761002257   -2.466001608450   -0.606347753536
H     -0.053106439597    0.786299897016    1.551972138348
H     -0.977487861738    1.037173842384   -1.643107359516
H     -2.647124161938   -0.523004839470    1.146519165303
N     -1.840284861798   -1.062571851846    0.878189640788
H     -1.825728647518   -2.024559298752    1.165757637997
O     -3.141922340140    3.365017796207   -1.870568145508
H     -2.970970054197    2.593261509805   -1.260145192637
H     -3.940222896631    3.788477462483   -1.554374928724
O     -1.115491556065   -2.098107144781   -4.025361398141
H     -0.942561166915   -2.815864839571   -3.414850708495
H     -1.883576739705   -1.600206628392   -3.650717009620
O     -3.257049589033    1.569724287538   -4.022911942043
H     -3.249346535965    2.275558210971   -3.357897578193
H     -2.429106624310    1.681519901035   -4.538124485707
O     -0.834233877446    1.865617411347   -5.160434647606
H     -0.382945609993    2.659895073782   -4.834947870533
H     -0.290328911616    1.115565936444   -4.866831638509
O      4.998609546460   -1.173376766281    0.376845811088
H      4.960060172911   -0.184843471943    0.349829868948
H      5.928519883391   -1.396028657981    0.315409430594
O      4.931684945829    1.481940411256    0.550917760562
H      4.344984899167    1.593168594726    1.315749511421
H      4.536573450499    2.006437732704   -0.170067002810
O      0.597103323582    3.945635818425   -3.997489452469
H      0.150090549559    4.132818321509   -3.136064838210
H      0.780485420678    4.795612164358   -4.397970986023
O      4.077897775722   -1.276884564142    2.904064618344
H      4.394869808829   -1.360018214052    1.976843580595
H      3.145507876831   -1.521507137970    2.895791450765
O      2.435803223491    1.931504549138   -3.516725393917
H      1.831772389035    2.659998744663   -3.739258275520
H      1.917039782435    1.122604175096   -3.611439860892
```

```
O      3.538280554957    1.310787889425    2.993953845352
H      4.072460096356    1.781566746469    3.634911154751
H      3.860397707452    0.370968625100    3.001942258864
O      3.910810170853    2.977954543092   -1.516630911065
H      3.387360083532    2.528821854322   -2.212849168680
H      4.641794609724    3.399591012911   -1.969897420385
O     -0.636917512503    4.364540671297   -1.687504134394
H     -1.571240995252    4.097963476437   -1.812267364760
H     -0.280536105228    3.782865632647   -1.012325800569
O      1.316529203491   -0.432008675797    3.437203722293
H      1.860329470611    0.332632782962    3.190633615664
H      1.223692753619   -0.399842482690    4.390433601004
O      0.780125075101   -0.250335166087   -4.317258977309
H      0.078386437840   -0.934937117081   -4.205698752695
H      1.264028725379   -0.503704692563   -5.105669011227
O     -3.146573324509   -0.800886582239   -2.914050795002
H     -3.168876221354    0.113753424810   -3.313674476267
H     -3.997578714571   -1.183649544807   -3.132890387706

Ambimodal TS Water15-45
65
ANGSTROM
C      0.235631516824    1.749081705376    0.802891762701
C      1.365873126728    1.562536000521    0.071064400819
C      2.024945060376    0.291577397484   -0.160543215063
C      1.570653174919   -0.919935899053    0.299369474452
C     -0.285751132671   -1.265783543234   -1.002136104319
C     -1.267541685365   -0.513196397396   -0.366393418203
C     -1.553099664322    0.864761602776   -0.790960897391
O     -2.511624528685    1.556725627490   -0.390879398053
H      1.772598139983    2.411248628849   -0.482794170189
H     -0.223754221152    2.725209235447    0.885721280061
H      2.830061418479    0.300888924673   -0.896156802281
H      2.093567710727   -1.832804443051    0.043199255160
H      0.928504238248   -1.002894235237    1.166684931353
H      0.153374774228   -0.914092698076   -1.923791157401
H     -0.230655650525   -2.333307493366   -0.844790628407
H     -0.149681482471    0.997532242544    1.476064792703
H     -0.974099081376    1.231994116356   -1.663559488310
H     -2.630356509142   -0.435803144135    1.203606523433
N     -1.970805282547   -1.003974989737    0.696425434068
H     -1.846979892127   -1.953506437673    1.023252101562
O     -2.446771251633   -5.579010076492    0.090277029453
H     -2.320329510687   -5.009044340945    0.866932230325
H     -2.004171508407   -5.106449373637   -0.636716134114
O     -2.128196626607   -2.143132699115    4.310940670049
H     -1.414399463656   -1.549241980070    3.968170980796
H     -1.809998871503   -2.505020843685    5.137927674753
O     -1.718346100791    1.996646565973    4.015540793363
H     -2.441599781151    1.868534623672    4.636351715183
H     -2.153348193381    2.292579404801    3.186488624646
O     -4.078363754355   -2.621688600821   -1.596818311818
H     -4.442709569562   -3.490663739134   -1.259381630829
H     -4.660975419030   -2.344210439087   -2.303780934601
O     -0.408824072034   -0.365715278914    3.418904115911
H      0.526156986387   -0.191900933933    3.642824710231
```

```
H     -0.884457227011    0.456608460319    3.630754381112
O      2.092305684470    0.600314646644    3.809075610553
H      1.760403144569    1.506145368015    3.973651670435
H      2.530843894123    0.629955959083    2.956929912324
O     -2.587172060028    4.285318119594   -0.211380389474
H     -3.129388667301    4.704440692888   -0.880227808815
H     -2.503624261953    3.346540815868   -0.472542370713
O     -4.930340413173   -4.846768963904   -0.514267943514
H     -4.060838051819   -5.267715500883   -0.290881353356
H     -5.252850492993   -4.467957315981    0.309838957754
O      0.848093311940    2.985599854609    4.246442768207
H      0.916010701229    3.374247991508    5.119577245936
H     -0.077111659358    2.700785518165    4.139821083908
O     -1.716484216881   -3.965124278076   -2.038724038803
H     -2.456933500182   -3.351480428217   -1.868788665860
H     -1.947967742998   -4.457750180965   -2.827510745750
O     -4.653271182449    1.864427822622    4.122474218815
H     -4.310267015752    2.294448196315    3.316445489147
H     -5.590462964454    2.056932380214    4.161436113869
O     -3.919728215917   -0.506530748015    2.854789867354
H     -4.224271353984    0.232416501518    3.403522997933
H     -3.388069193469   -1.076677720425    3.428872039882
O     -2.458440420821   -3.562802780548    1.944100518276
H     -2.313763343932   -3.236686522623    2.844931077596
H     -3.372205566256   -3.295188506612    1.715619026884
O     -3.263771778335    2.722292442567    1.936217876986
H     -3.144323183293    2.052618807304    1.236551502173
H     -3.076013347271    3.557394382128    1.478890175184
O     -4.828196965141   -2.465245111097    1.178188267647
H     -4.527803998982   -2.196498050890    0.297956686194
H     -4.683148203568   -1.698790144557    1.758622780607

Ambimodal TS Water15-46
65
ANGSTROM
C      0.216719662673    1.667615981208    0.709898144840
C      1.331301124927    1.464677336677   -0.043291699019
C      2.036851837742    0.209277313734   -0.189901604940
C      1.673012050889   -0.969365269591    0.404456025356
C     -0.251568969638   -1.461213935764   -0.803748580211
C     -1.221656852855   -0.671906444217   -0.209524039811
C     -1.546598516678    0.648417945885   -0.749651638880
O     -2.559453320205    1.317858633391   -0.388457164452
H      1.667828931731    2.277082792751   -0.686277388324
H     -0.271869252645    2.632631106165    0.722249688716
H      2.804846007310    0.184970180556   -0.961753035688
H      2.219128824249   -1.881204595768    0.196418105501
H      1.062170203835   -1.009444300255    1.297616413242
H      0.172813760822   -1.166816698912   -1.750990039687
H     -0.150761618833   -2.504107680394   -0.534426243695
H     -0.108868642944    0.971779793342    1.470592462052
H     -1.012976737303    0.970339062271   -1.653063850543
H     -2.698836379017   -0.574018420747    1.226109197461
N     -1.845426835480   -1.044223726088    0.972093331820
H     -1.828492877859   -2.020631233027    1.243148356828
O     -0.043213434956    2.896814539512    3.791025834732
```

```
H      0.766804151906    2.809678427833    3.285504191474
H     -0.391679628968    1.987615533952    3.913690397974
O     -2.097096870949   -1.736429277668    5.356597050953
H     -1.346740572564   -2.064493527734    4.838006555023
H     -2.059857986058   -0.778046564409    5.253208475881
O     -3.946810440191   -2.757470526709    3.606048338048
H     -3.435257296538   -2.462425295419    4.382369671249
H     -4.376156557505   -1.953501992011    3.260176709273
O     -2.131692015441    4.065824891432    2.627248474393
H     -1.312973822936    3.706990555803    3.049727971728
H     -2.441133912324    4.770585362110    3.196859367309
O     -4.222506886539   -3.010565070941    0.390806227092
H     -4.415038112884   -2.135872914325    0.029961507732
H     -4.683983492148   -3.056773129940    1.233865434517
O     -4.979203851432   -0.481009454303    2.471654052669
H     -5.146178025229   -0.469154714527    1.518731075363
H     -4.451972037996    0.312588133471    2.647210340084
O     -0.162173441682   -2.271722638285    3.343072980444
H      0.586788821182   -2.815252935927    3.589246947000
H     -0.764264094001   -2.846841724491    2.832225030004
O      0.961848353526    3.236463411811   -3.270787675572
H      1.867338338505    3.547104965576   -3.316283360741
H      0.539123803434    3.742426650574   -2.543986629656
O     -4.807107245207   -0.250528707675   -0.317764213182
H     -4.054364335123    0.369480953659   -0.382645216583
H     -5.449940042314    0.028011372219   -0.970668380820
O      0.654004722468    0.502020944477   -3.358755629692
H      0.757062894260    1.469686784949   -3.292595716433
H      0.567184664455    0.317973399896   -4.295236849642
O     -0.041716567785    4.796196117107   -1.302399977700
H     -0.210775013651    5.671144482974   -1.654981095998
H     -0.897125781413    4.505791425146   -0.907076349463
O     -2.051220749502   -3.729671664050    2.030274856388
H     -2.540698847993   -3.946430007568    1.227597336796
H     -2.761439600689   -3.470692365327    2.672072330187
O     -1.159913119496    0.435219333210    3.759950383956
H     -0.820411527357   -0.399563704965    3.410379381736
H     -1.925796841390    0.693344132143    3.209273580810
O     -3.141383261097    1.557807163510    2.261711785686
H     -2.961165694261    2.483644877563    2.497276114092
H     -2.951171414497    1.500273487792    1.304563880991
O     -2.363437342831    4.075436210962   -0.192152828627
H     -2.320163764631    4.154472911420    0.773205756842
H     -2.523296736320    3.135891313073   -0.384097870956

Ambimodal TS Water15-47
65
ANGSTROM
C     -0.037318247585    1.522039084101    0.603669918345
C      0.972280411786    1.431298219950   -0.308601964595
C      1.770454233072    0.250842115617   -0.548320427266
C      1.596451056027   -0.944022815022    0.106783035156
C     -0.390619804112   -1.664110356104   -0.784342738486
C     -1.374946923829   -0.908651658929   -0.161793582051
C     -1.792696015197    0.382285179049   -0.714522425883
O     -2.781965902273    1.053234215247   -0.319908232927
```

12711	H	1.115231821046	2.258985418956	-1.003898364405
12712	H	-0.625046015186	2.427014034150	0.693030441933
12713	H	2.420384026629	0.279723109428	-1.423448979219
12714	H	2.194894521499	-1.807733985887	-0.157766141387
12715	H	1.145204576246	-0.991174770702	1.089123593940
12716	H	-0.090731815153	-1.435052144887	-1.794349582526
12717	H	-0.210321124092	-2.683294606506	-0.468328239568
12718	H	-0.155832168666	0.816161400674	1.414786600184
12719	H	-1.328961092958	0.671715989559	-1.671262860648
12720	H	-2.730373398117	-0.784717246434	1.400850066693
12721	N	-1.947427138124	-1.296617749453	1.029142708358
12722	H	-1.773242482570	-2.202718359758	1.426836617445
12723	O	-0.911097954790	-1.460133450905	6.051894518707
12724	H	-1.554919871200	-0.928388883078	5.558711302727
12725	H	-1.015528583514	-1.225807263969	6.974154297050
12726	O	-1.579545530485	-0.626126154980	-4.157585469359
12727	H	-2.173676553723	-0.217907950314	-4.797659293517
12728	H	-2.148117255273	-0.953172339440	-3.440888861359
12729	O	-5.587403416935	1.061932361860	-0.526862674502
12730	H	-4.617841418347	1.059591384799	-0.513558829104
12731	H	-5.824525354943	0.547835427619	-1.309980901461
12732	O	-3.460752207993	-1.459092490874	-2.298300168801
12733	H	-3.745539231591	-2.370049684496	-2.219848378859
12734	H	-4.241162242031	-0.942385393173	-2.578753059491
12735	O	-3.051627377875	1.530292665810	-5.666373790460
12736	H	-3.317967392305	1.932193595056	-6.493665668857
12737	H	-3.642585871897	1.890940711580	-4.979124159258
12738	O	-0.470804339800	3.668207187063	-2.581959339273
12739	H	-0.176574878417	4.483728425544	-2.988585172552
12740	H	-0.420734892884	2.979019940676	-3.285915741180
12741	O	-6.056500975162	3.533876715048	-1.671337274794
12742	H	-6.045483251630	2.707645809128	-1.149960169348
12743	H	-5.258230514553	4.003456670989	-1.408819730368
12744	O	-1.695378693434	-2.811698972652	3.568687047671
12745	H	-2.242456160183	-2.025310304808	3.748288570118
12746	H	-1.371781980872	-3.087917618668	4.431160826037
12747	O	0.164707864893	-0.756380978808	3.292109561090
12748	H	-0.315627744238	-1.597153463732	3.186162416603
12749	H	0.497245434190	-0.771529132559	4.193635136089
12750	O	-5.566438319599	0.039125332308	-3.134658402123
12751	H	-5.164427391829	0.924662607350	-3.345781468725
12752	H	-6.151869843532	-0.172715004379	-3.861906628414
12753	O	-4.619705149495	2.448366871240	-3.645027620688
12754	H	-3.900439229688	2.725260903189	-3.014500007751
12755	H	-5.385643512338	2.924404278114	-3.264627669886
12756	O	-2.450220899211	-0.186365111581	4.088574063030
12757	H	-3.125574658124	0.219168280218	3.521817111900
12758	H	-1.599097247793	-0.006463212709	3.654423340801
12759	O	-0.489773294260	1.867605673269	-4.546901541818
12760	H	-1.314542983468	1.955806636688	-5.049868014511
12761	H	-0.505866790082	0.940929542877	-4.258132618731
12762	O	-3.031721298419	3.343793754824	-1.761340674338
12763	H	-2.113811869606	3.555014161134	-2.021061305676
12764	H	-2.952414916996	2.578877056694	-1.160243271322
12765	O	-4.045101655273	0.773222203631	2.075307199862
12766	H	-3.524301056519	1.031919698777	1.289670676634

```
H     -4.962252420394    0.829227888834    1.795818839945

Ambimodal TS Water15-48
65
ANGSTROM
C      0.228009579790    1.665332235705    0.843499005699
C      1.329779885812    1.552597726248    0.059313499609
C      2.062966210546    0.324551534803   -0.199304398694
C      1.717837300779   -0.907555243458    0.295637304065
C     -0.202614329921   -1.412279987579   -0.858896332169
C     -1.209510678416   -0.659036156600   -0.269935107449
C     -1.568673735049    0.661460232704   -0.802502275319
O     -2.538132879005    1.353118795802   -0.426051026824
H      1.652901203170    2.422589446777   -0.516306726549
H     -0.300086235637    2.605298515753    0.939569214931
H      2.823009976706    0.383606258876   -0.978992050231
H      2.282997828245   -1.786715465409    0.012276946852
H      1.122279369333   -1.024076132411    1.191098544622
H      0.204186360625   -1.115411078595   -1.812686034822
H     -0.096386836933   -2.459886257296   -0.616110538151
H     -0.090887764152    0.886953579016    1.522160369722
H     -1.016070438208    0.995984681799   -1.700569546592
H     -2.715322592430   -0.599382836197    1.131728083446
N     -1.875124694579   -1.076838551851    0.854105577941
H     -1.751048534150   -2.012558561442    1.225355374532
O     -3.300859071571   -1.393288421693   -2.051966957197
H     -3.991684251784   -1.208109515297   -2.702018171871
H     -3.814378697354   -1.745512245062   -1.286545159962
O     -3.260333163715    1.726259804524    2.117817242474
H     -3.539166504235    2.638936182662    2.192945986015
H     -2.948187415301    1.601784893462    1.190004490465
O     -3.871086215125   -2.049725811740    3.616278862426
H     -3.294259058182   -1.266723651449    3.765506994800
H     -4.580743992798   -1.726599951522    3.050638227583
O     -2.238694385380   -2.166985829080    5.923927958526
H     -3.028894072008   -2.479612737166    5.464664063614
H     -2.154528275435   -1.240363484383    5.661415586652
O     -1.977546684862   -3.762846847260   -2.155766019643
H     -2.355199604915   -2.856744333369   -2.169972351658
H     -2.383945695226   -4.232094403789   -2.885846596405
O      2.496584085722    0.585080900171    3.477654830378
H      2.769870172488    0.434214478371    2.568229325714
H      2.285379897442    1.517731453966    3.556104398818
O     -3.265395301882   -4.588744143173    0.138711700687
H     -2.698009825451   -4.406863495127   -0.633784703284
H     -2.736843393008   -4.364601843245    0.920132675338
O     -0.261006702096   -3.306684022848    4.554609460845
H     -0.973877117406   -2.931425234882    5.130565827494
H      0.253247178969   -3.897791525292    5.104282121788
O     -5.053365338042   -0.353293026576    1.710162141641
H     -5.454114377638    0.003530598790    0.906182039104
H     -4.514967926586    0.376464334856    2.057620013892
O     -5.266273049254    1.088859451441   -0.701998654876
H     -4.292141917479    1.099213840081   -0.718542319003
H     -5.551879042343    1.897628246478   -1.128490170574
O     -2.205355774036    0.021172037586    4.081309749469
```

```
H     -2.511219318412    0.714877036292    3.480963143781
H     -1.337865124239   -0.277174444810    3.727000191589
O     -4.865101906029   -2.501583984617   -0.180257459970
H     -4.381116668904   -3.346036387234   -0.008390290320
H     -4.898776665007   -2.015311883337    0.650854811119
O     -1.975764886543   -3.501040907372    2.329549822836
H     -1.278612221437   -3.601982261955    2.991107564806
H     -2.731978821914   -3.100838264377    2.806442699789
O      0.161865526599   -0.910211400468    3.256599394365
H      0.973138755091   -0.478072390838    3.564049376681
H      0.143201383050   -1.797403591234    3.651886390444
O     -6.061349775598   -1.075641124432   -2.383072591497
H     -6.014991009194   -1.822128489953   -1.773119636482
H     -5.922801227806   -0.297166544656   -1.824416821954

Ambimodal TS Water15-49
65
ANGSTROM
C      0.210050839118    1.342656105977    0.848399810130
C      1.336729706877    1.312828347365    0.083233324392
C      2.070965246411    0.123066066801   -0.277244993705
C      1.722344503087   -1.154740943460    0.102116075515
C     -0.119152374046   -1.529691529642   -1.093118224459
C     -1.130987966705   -0.801309464111   -0.477226364249
C     -1.461435051733    0.560577764940   -0.903140987585
O     -2.457122124103    1.222922903429   -0.532638473743
H      1.671893505540    2.238156460915   -0.383569106666
H     -0.330346860599    2.263851426647    1.028028975079
H      2.833829400587    0.244546356527   -1.043026729229
H      2.301066548671   -1.999502820467   -0.251849982765
H      1.168880392376   -1.352046288932    1.011572338607
H      0.296335290442   -1.193181759102   -2.032034146491
H     -0.038344962019   -2.591775344788   -0.898797416064
H     -0.095817675030    0.515669364279    1.473957280758
H     -0.864637522520    0.953465568129   -1.747026617052
H     -2.327786566068   -0.702263195739    1.197862150364
N     -1.880784169895   -1.358096496435    0.564827752796
H     -1.474175356608   -2.160417218231    1.049188276252
O      0.752242043276   -1.112694346474    3.530418534716
H     -0.125616124787   -0.691882392136    3.746071027478
H      1.094878563719   -1.439412369594    4.364335175813
O      1.080020417929    0.104056969947   -3.820398603479
H      0.868662957543    0.044305461100   -4.753284071312
H      0.936454666103    1.051064881824   -3.578915235045
O      2.553267811009    0.956057254915    3.206203630043
H      1.990104333864    1.728703950198    3.130883034900
H      1.957219460152    0.183487299550    3.213143920989
O      3.736490869568    0.143098806406   -3.208474009828
H      4.206964000227   -0.073739170943   -4.014235617129
H      2.781819323062    0.022850291820   -3.399024883430
O     -5.066245742565    0.519072943216   -0.295296039875
H     -5.599842517658    0.884691796268   -1.001022328375
H     -4.149133324633    0.817410702610   -0.455482510085
O      3.484525959468    2.870947528921   -2.775174565875
H      3.719688735562    1.931239550136   -2.873866721376
H      3.636477435172    3.088451101830   -1.836667117090
```

```
O      0.888300477116    2.691950172806   -3.242604088895
H      0.460017042998    3.050973185620   -2.463998737360
H      1.859605317997    2.864919580521   -3.128847049048
O     -2.874511459107   -2.689509642064    4.118393266652
H     -3.154077290731   -3.063785925296    4.954429152179
H     -3.694562082099   -2.574134521766    3.576397404716
O      4.581349062219    1.241938943371    1.390781777139
H      3.924913393214    1.141694701751    2.107331385129
H      4.561625453607    0.421967902004    0.893178359229
O     -5.107672550198   -2.447584411344    2.717019159317
H     -4.914817695121   -2.326731318966    1.750161654267
H     -5.718654385755   -3.183617649031    2.765192972541
O      3.908564344816    3.392681724801   -0.110413682848
H      4.153483222484    2.608266993278    0.426271759412
H      4.598674431052    4.039908897804    0.039542536317
O     -4.563731693727   -2.133852379203    0.155650683070
H     -3.592775855835   -2.071870830999    0.126938041421
H     -4.884197529272   -1.235509321377   -0.045264517008
O     -1.644807281386   -0.231191635009    4.106926777019
H     -2.151367611714    0.285304618375    3.460624624973
H     -2.120569731241   -1.079035286382    4.183939355089
O     -3.214898842631    0.822251210903    2.076034925413
H     -2.952378936418    1.204460748076    1.217926701560
H     -4.156273626567    0.642569545617    2.005545829654
O     -0.731060994241   -3.203517899503    2.359911356193
H     -1.465315793455   -3.202494462506    2.991181816782
H     -0.075057608308   -2.586786946418    2.721898215606

Ambimodal TS Water15-50
65
ANGSTROM
C      0.241156192935    1.592762088280    0.819676385933
C      1.321335711307    1.465922519017   -0.004939146377
C      2.014025119820    0.241319876769   -0.287427979874
C      1.637866940707   -0.996025509251    0.214322109979
C     -0.203060652359   -1.428027495492   -0.785305855219
C     -1.195489933028   -0.621681476580   -0.227540835050
C     -1.555567406695    0.700844788777   -0.745681949560
O     -2.550894144378    1.342289973400   -0.366624972494
H      1.625643464915    2.334386144729   -0.588128569452
H     -0.278705231954    2.535903755615    0.926491973593
H      2.748628387436    0.269816335522   -1.089220344442
H      2.200824900463   -1.877262512570   -0.072322056659
H      1.164629143928   -1.076423589989    1.184929201356
H      0.147531921965   -1.208504399690   -1.782197743750
H     -0.169566414455   -2.478987399910   -0.521322564015
H     -0.033678328567    0.843653366784    1.547976804679
H     -0.989896082854    1.050928787629   -1.628989344198
H     -2.653503665590   -0.424068137982    1.188232889430
N     -1.856466114419   -0.991977421139    0.943710624890
H     -1.908746323494   -1.959807393788    1.210963020395
O      1.818799276762    4.197384315252   -2.486778536477
H      0.974994042749    3.731902841724   -2.529386281366
H      2.336014789378    3.845983642110   -3.224282847178
O      5.039744388883    2.048368836264    0.806196158367
H      4.466745514402    1.463939969289    1.335653566733
```

```
H      4.475538687991    2.741523672451    0.438710421838
O      3.717132642077    4.149256478732   -0.598510914009
H      2.936927415444    4.195106901086   -1.193422135870
H      3.865030034086    5.038081292799   -0.274277393370
O      5.230018341784   -1.391740459459    0.501127636054
H      4.813087308575   -1.358007489616   -0.376630291267
H      5.987447562225   -0.801880407001    0.440107367750
O      3.814638295206    0.084452951859    2.287784161467
H      4.264130375731   -0.010823381849    3.128940890418
H      4.222170455632   -0.570617123953    1.683030966986
O      0.176541668853   -0.592950991642   -4.127933745596
H     -0.778326243332   -0.591227619747   -3.867276412245
H      0.191464050125   -0.844571051072   -5.053623989806
O     -2.409264361175   -0.350680302734   -3.620999805953
H     -2.481193351333    0.631211588751   -3.794512316578
H     -3.101347801152   -0.758527720460   -4.142416324765
O      2.970452124807    0.001925917183   -4.093625734979
H      3.539786445527   -0.350144455150   -3.377598379267
H      2.073215252790   -0.301814605903   -3.907332953382
O     -2.432506845752    2.214180559975   -4.165514729102
H     -1.478994402755    2.393474956808   -4.154530841647
H     -2.788053748372    2.715692067427   -3.401906331579
O      4.655821274404   -0.935838714379   -2.197603774057
H      5.218073280818   -0.137205873622   -1.935541750496
H      5.250815164707   -1.527017302234   -2.661412109210
O      0.333110431157    2.168570351344   -3.862786013067
H      0.312595455716    1.196113805326   -3.858482913673
H      1.144294798263    2.408781994168   -4.327793705168
O      6.123113680600    1.026680542731   -1.414023253053
H      5.984764701197    1.790302672749   -2.010936629151
H      5.803224718403    1.359244401344   -0.543752788891
O      5.252760643459    3.207811592047   -2.740309786395
H      4.839468041703    3.587978748400   -1.946739808979
H      4.524052930513    3.009825181362   -3.346775972175
O     -3.272696252369    3.418834219900   -1.895648490239
H     -2.992766077397    2.707262924677   -1.273000855291
H     -4.211487463357    3.539441706725   -1.749383289343
O      3.104033061389    2.588520752497   -4.491660877583
H      3.098657940039    1.607585313053   -4.318715186374
H      3.495050271957    2.705418979399   -5.358412872783

Ambimodal TS Water15-51
65
ANGSTROM
C      0.214645970287    1.390727494887    0.907549408612
C      1.318513650884    1.321401030028    0.108191401718
C      1.995222151593    0.100930190055   -0.255100798876
C      1.583725041426   -1.157628606487    0.133056441785
C     -0.304493046697   -1.430273187583   -1.037268096811
C     -1.268837158368   -0.659796616678   -0.390550661314
C     -1.505213249305    0.738410683444   -0.774397387124
O     -2.462437374318    1.440911096517   -0.381131881515
H      1.642575125482    2.233395057823   -0.392201431417
H     -0.267590863282    2.339701204973    1.097231909866
H      2.756702460484    0.181352219611   -1.031286411678
H      2.114826654128   -2.034932644668   -0.218642179966
```

```
H      1.026228136053   -1.316154593263    1.048121835185
H      0.112704826890   -1.095093970505   -1.974875108998
H     -0.287879784559   -2.501330672217   -0.886514567771
H     -0.083833637297    0.579229009818    1.556739033075
H     -0.901775317616    1.116469385235   -1.620461032786
H     -2.570470524335   -0.565508899092    1.212421755355
N     -2.031820673842   -1.186746594737    0.631087390935
H     -1.755942221304   -2.062348058948    1.056696828150
O     -3.209824919210   -4.584934863091    4.762880778149
H     -3.443230949788   -4.879171334197    5.643137909605
H     -2.212559160846   -4.608933794335    4.711632341131
O      0.546200510532    4.419813209134   -0.900255000611
H     -0.374250010361    4.333618401522   -0.584976215029
H      0.589820864474    5.278924511867   -1.321526865788
O     -0.636640378566   -0.929437543616    3.346508440245
H     -1.448900105589   -1.459301536342    3.245891228281
H     -0.951151518295   -0.032141453894    3.564143204672
O     -0.132155706705   -1.863653186450    5.734618298205
H     -0.191158212421   -1.483095668077    4.815138494048
H      0.752651326082   -1.685199712806    6.052430689621
O     -2.057963211135    4.171340954926   -0.030049690284
H     -2.200313951934    4.186045843342    0.929335074232
H     -2.282167628106    3.269068027209   -0.311510415417
O     -3.669243755194    1.315856076915    1.973867312496
H     -3.231073321631    1.372480136654    1.091115892948
H     -4.550653658888    0.931533978931    1.792870250233
O     -3.666404369084   -4.722373788428    2.016716559108
H     -4.282627236698   -3.984819672423    1.880928853751
H     -3.661173308174   -4.893916115247    2.970874019918
O     -5.840492245445   -0.014860634236    1.114538180206
H     -5.750151449022   -0.056803862517    0.161043319854
H     -5.666211938326   -0.917047633875    1.450554444276
O     -0.635003629325   -4.295597675908    4.483685658018
H     -0.423703320615   -3.504559189274    5.010338881634
H     -0.669362027691   -4.004390527197    3.552769100486
O     -1.205090519535   -3.752010634062    1.829940125964
H     -2.123618251166   -4.124378319611    1.860058061165
H     -0.695206083339   -4.352206788763    1.285141404963
O     -2.880874307827    3.831933235646    2.654122907233
H     -3.310242783458    2.988814431919    2.403373807634
H     -3.584873117592    4.464161681360    2.801840129271
O     -5.246048146625   -2.403845296802    2.269268764150
H     -4.460066109821   -2.133767114828    2.811243338663
H     -5.954373475648   -2.575657883307    2.892376934100
O     -3.145762922725   -2.010899666662    3.799566653679
H     -3.119480276912   -1.427192566880    4.595837481139
H     -3.149182873051   -2.918602999000    4.153492199821
O     -2.631825337880   -0.525640890178    5.932599503518
H     -2.396679629291    0.295689713762    5.472307758911
H     -1.794819016318   -0.968500701672    6.140609237821
O     -1.921408246232    1.372882699404    4.024197402596
H     -2.623502743595    1.243717356214    3.353793267512
H     -1.693692503312    2.304659087426    3.957214183639

Ambimodal TS Water15-52
65
```

```
ANGSTROM
C      0.173452789900    1.662578801574    0.935497130464
C      1.293409971309    1.595319347234    0.165831547480
C      2.042925580594    0.395080294994   -0.125176915597
C      1.693624395006   -0.864944079724    0.315848566564
C     -0.132582914185   -1.350752441950   -0.828621042551
C     -1.159473143695   -0.594959677595   -0.266987134859
C     -1.523042258192    0.724193357011   -0.809640783833
O     -2.547946127295    1.364818996783   -0.530616777019
H      1.605643286712    2.492413147000   -0.373284361982
H     -0.378826393683    2.585619067903    1.051911432652
H      2.808826571134    0.484838296283   -0.896480705469
H      2.288019286668   -1.720639509959    0.017742858907
H      1.142587466517   -1.008746004315    1.236081217107
H      0.258280155283   -1.077476440910   -1.797357918981
H     -0.057275153613   -2.400769244885   -0.583042058815
H     -0.129402091637    0.864756016918    1.598062621564
H     -0.893204428439    1.084536575959   -1.649708434978
H     -2.611870910429   -0.498085714027    1.196951496218
N     -1.827270988143   -1.025694655255    0.855864645697
H     -1.770653775847   -1.991261049395    1.149013731104
O     -2.723473067608   -5.732876578781   -3.061316257688
H     -3.000768700361   -5.941958100666   -2.156182013858
H     -1.802868042888   -5.440383383706   -2.984461787872
O     -1.869014737902   -3.649748219574    2.191575395683
H     -2.817348444387   -3.850224345084    2.125381381708
H     -1.421355475421   -4.400564570956    1.751062317868
O      1.550602590037   -4.208904911771    1.934442282470
H      1.206402889195   -3.383226403454    2.329225783951
H      0.901521116943   -4.889270475277    2.144807832178
O     -3.150889027961   -5.681062616739   -0.291264320037
H     -2.966776879540   -4.725318274793   -0.516091168199
H     -3.806974620077   -5.629654485673    0.411572217134
O     -5.934605583271   -2.325830054226   -2.304178326912
H     -6.587537438599   -2.984188648664   -2.544981390747
H     -5.126236928582   -2.557097425959   -2.799289571940
O     -4.653637083253    0.485357220879    0.831028651525
H     -5.389490650725    1.062113380761    0.623777107593
H     -3.880518132027    0.849772592903    0.330461919421
O     -2.772084389938   -3.173746339307   -0.910858526051
H     -3.478987027015   -2.714930575416   -0.418303632553
H     -3.026337551708   -3.100089057010   -1.848935795927
O     -3.657775055328   -3.250112195337   -3.500484603868
H     -3.459539030646   -4.212929017637   -3.482906350300
H     -2.874839547297   -2.827230406236   -3.895054580999
O     -4.560427597349   -4.264573503287    1.790012937435
H     -5.148719504783   -4.216788906834    2.543679439898
H     -4.777633356428   -3.494516504490    1.223518772090
O     -0.065568987324   -4.717239139566   -2.922440179495
H      0.531718755367   -5.275771362459   -3.422774735839
H      0.366830013870   -4.550995889951   -2.063963243003
O      0.198653700394   -2.182826425012    3.148455229057
H     -0.637346316294   -2.596913899049    2.860935050669
H      0.163894038183   -2.130894561822    4.103997795718
O      1.434060881945   -4.657924910367   -0.653774470882
H      1.506052473224   -4.328870428781    0.280023676147
```

```
H      2.233252428530   -5.166954232918   -0.796272120975
O     -0.748445162277   -5.769929566545    0.898309500323
H     -1.609369055533   -5.932796353593    0.456196181224
H     -0.141817364616   -5.504307942333    0.195519130385
O     -1.141053575807   -2.547878201267   -4.296408878173
H     -0.740148014225   -3.225147995884   -3.723894443409
H     -0.879548798476   -2.752902603107   -5.195058006266
O     -5.003347285469   -2.118359072683    0.229498207364
H     -4.904379532358   -1.177823615162    0.485435786820
H     -5.454266856230   -2.125767650003   -0.637978127353

Ambimodal TS Water15-53
65
ANGSTROM
C      0.180132374501    1.806576602458    0.454181706462
C      1.288382599377    1.585905385872   -0.301170408057
C      2.024189575977    0.339760451502   -0.384257255205
C      1.672738802199   -0.813694792300    0.265508786532
C     -0.228064287201   -1.410859611968   -0.925225770102
C     -1.232909082383   -0.641154776305   -0.358626076767
C     -1.622455871333    0.650459088908   -0.933024479459
O     -2.609657380816    1.321321003963   -0.550527227246
H      1.603599498028    2.368512085119   -0.994244959080
H     -0.335014250455    2.757133988406    0.405550060792
H      2.801728851105    0.296686215553   -1.148014056208
H      2.236107283731   -1.725924009911    0.114020922373
H      1.056963347470   -0.808108486183    1.155183910311
H      0.174103920460   -1.150654244518   -1.892351151676
H     -0.086829019726   -2.437700171851   -0.622229009672
H     -0.121014537985    1.147973428197    1.258463344474
H     -1.119606309679    0.956394428296   -1.869144973965
H     -2.656265153144   -0.508130387896    1.143402207405
N     -1.856189736814   -1.012086807266    0.806580937444
H     -1.670155955009   -1.912195028515    1.220234416855
O     -4.859702296823    0.241260956044    0.645163638580
H     -5.683201322007    0.627090169731    0.283472632256
H     -4.134483548403    0.644051824502    0.135718497858
O     -1.224045194760   -4.615368256504   -0.105773802706
H     -1.873827734174   -4.042296787829   -0.572983736101
H     -1.585839359602   -5.502149060105   -0.120212908962
O     -3.122249660729   -3.125057872971   -1.282540553157
H     -3.742087030433   -2.872717339809   -0.547368329109
H     -3.672765160894   -3.508949014151   -1.965414934098
O     -2.961603590742    2.859092738952    1.803508493656
H     -2.609287893379    2.150946764074    1.244548103952
H     -3.499929316040    3.382652961584    1.181471752521
O     -3.617360174998   -3.131050752087    3.073595787526
H     -4.166564874033   -3.608691213182    3.695192008405
H     -3.347518898308   -2.284519549745    3.541780052307
O     -0.192600556165   -0.999293000148    3.297363320858
H     -0.378487702539   -1.885295614424    2.925814512672
H      0.337338764190   -1.150226677844    4.081301883384
O     -0.987471859494   -3.426008583908    2.329994520071
H     -0.939870336863   -3.915064618605    1.486835356943
H     -1.904796954656   -3.513615403419    2.642217041153
O     -4.382229654989    0.959739981598    3.244330047740
```

```
H      -4.729391745085    0.624126358845    2.398002707764
H      -3.906523075597    1.770589451893    3.011732487405
O      -1.044096678526    4.739547896628    2.142202010227
H      -0.262722063276    4.342377277464    2.529271917297
H      -1.752737584214    4.063055578847    2.188745665177
O      -3.692424109358    3.861827627583   -0.615192149922
H      -2.935512676946    4.476220524060   -0.695114209175
H      -3.339134341985    2.987383613564   -0.852902322449
O      -1.458374581368    5.391253355757   -0.456357594476
H      -1.231063836476    5.213293462012    0.480429802011
H      -1.506222786711    6.344050003945   -0.541139979008
O      -6.262993777167    3.853083456850   -1.440900921515
H      -5.312263180299    3.902122373758   -1.213323000491
H      -6.691290691824    4.554276211569   -0.947460648031
O      -6.976679839470    1.406123685272   -0.544380317304
H      -6.737793036413    2.296748983459   -0.880904217299
H      -7.386152495810    0.942170710167   -1.275220456104
O      -4.786506718249   -2.503037476210    0.655446201726
H      -4.400419318846   -2.745026328994    1.516451619485
H      -4.891748262524   -1.534212741039    0.669189235696
O      -2.810256748130   -0.964877607984    4.245939540972
H      -1.919509867566   -0.783538046657    3.898324765527
H      -3.390085838265   -0.223411545939    3.965201723150

Ambimodal TS Water15-54
65
ANGSTROM
C       0.018074671258    1.200953603659    1.454893671761
C       1.171213052602    1.396116556082    0.754952383412
C       2.008919551645    0.349954953189    0.222234899204
C       1.726405045436   -0.995316083159    0.339143995736
C      -0.038364627310   -1.265443170992   -0.995939161579
C      -1.131285690021   -0.705274906386   -0.335767257974
C      -1.503686727851    0.705377082743   -0.520396393844
O      -2.571076867189    1.215136051441   -0.129539361300
H       1.435599844736    2.415259323018    0.465892889668
H      -0.582657843691    2.039656612649    1.777053663438
H       2.797335298974    0.662102843392   -0.463777110111
H       2.378381281632   -1.727359769887   -0.123635739590
H       1.146200142946   -1.374118615728    1.170975817211
H       0.402998446196   -0.748005141111   -1.834680281345
H       0.082246845221   -2.340837022344   -1.010135343139
H      -0.235779323525    0.251874119178    1.904645210537
H      -0.863072561922    1.294467964138   -1.206623976553
H      -2.616292668949   -0.971864809911    1.078352708879
N      -1.908256438542   -1.431310967792    0.531166977388
H      -1.689995149637   -2.375987247645    0.792982827152
O      -1.426990519088    4.726384795656    2.362223272886
H      -1.726032386412    5.191158777073    3.154679040216
H      -0.522629057984    4.426892597590    2.556297462007
O      -4.631910057393    4.278430638839    3.856078960048
H      -3.896526210066    4.869410628012    4.084863100488
H      -4.332883430759    3.790657429859    3.070787194821
O      -3.119882218730    0.885835732396    3.870748637447
H      -3.612198952455    1.291323489771    4.604193997449
H      -3.112379350726    1.560671990298    3.158272111556
```

```
O     -0.777241317402   -0.018071334418    4.947235641106
H     -0.393692755164   -0.627967870185    4.296340437494
H     -1.636260080857    0.251666524913    4.574721018485
O      0.054644504766    2.460165033897    5.483231660715
H      0.361783162819    2.755294028800    4.615443972112
H     -0.160933378426    1.507973903553    5.377101615691
O     -4.908770391546    0.111628005545    0.782736727299
H     -5.405339353912    0.879474122143    1.071094311811
H     -4.096221861709    0.462661639052    0.370191647546
O     -4.475120125873    2.401930734739    5.697477272812
H     -4.632790674020    3.126216220041    5.034049306456
H     -5.307014362753    2.244570748516    6.142910139348
O     -2.300651941875   -3.298481510259    2.793485305943
H     -3.021479086037   -2.617996694934    2.816714977261
H     -2.552353724350   -3.957363392166    3.441619252125
O      1.050641537510    4.012831411500    3.297350104622
H      1.948440664755    4.085109813840    2.974831185944
H      0.960731453893    4.646392787265    4.050740104610
O      0.020470383146   -1.967440954958    3.135352781593
H     -0.830415482901   -2.441206697884    3.003238618818
H      0.581992046767   -2.557150807849    3.640205127103
O     -3.238517307736    2.816162034148    1.988926497446
H     -2.541260171788    3.509397696360    2.054851022627
H     -3.087941797501    2.375034103864    1.138797412959
O     -2.358966609667    5.639466460236    4.891108437535
H     -2.306772373198    4.854859990082    5.530062481368
H     -2.717136358497    6.372953512062    5.392081463771
O     -4.158522886963   -1.426224165717    2.920393170087
H     -3.777914671167   -0.645553029658    3.369859170137
H     -4.575456079613   -1.068003874085    2.116919618056
O     -2.121338925580    3.636302818751    6.476843676448
H     -2.887871144623    3.054915057133    6.351818587530
H     -1.334226695157    3.132713171082    6.160620902166
O      0.420196027931    5.465301432933    5.427604056323
H      0.387397837138    4.727018083777    6.046038192397
H     -0.505229012074    5.675342249424    5.218270302028

Ambimodal TS Water15-55
65
ANGSTROM
C      0.222080797566    1.696803721039    1.030683564057
C      1.262463629541    1.688125893788    0.153065066286
C      1.938137996079    0.502647223260   -0.331868418572
C      1.608964132084   -0.781500173029    0.014597753133
C     -0.415236416121   -1.028989073059   -1.088559161515
C     -1.343184630229   -0.378256044212   -0.290709961755
C     -1.652347647600    1.034901772380   -0.513126146905
O     -2.583509199631    1.669943149488    0.049633276746
H      1.554909987075    2.623860551383   -0.323792064586
H     -0.270464191836    2.620107087792    1.309640536319
H      2.634940158007    0.660103649326   -1.153801263773
H      2.115071047152   -1.620606144060   -0.446253200730
H      1.094014635278   -1.009019173558    0.938757020216
H     -0.050983213239   -0.564374247751   -1.988545922377
H     -0.318390367001   -2.104553316003   -1.052772704990
H     -0.029745261071    0.844406909325    1.646923852290
```

```
H     -1.158292515501    1.509794873657   -1.375454533587
H     -2.586919599488   -0.505544522093    1.358214849265
N     -2.005533159002   -1.027782604389    0.728102036102
H     -1.720858919090   -1.951511771324    1.025670480177
O      2.469798730303    4.593371774758   -1.410592134652
H      3.029827062971    5.280314753641   -1.047913697440
H      1.599577027440    4.994357411789   -1.565999671340
O      1.979451632551   -1.772657438332   -3.428351384916
H      1.970729243541   -0.805528571194   -3.332084584546
H      2.752643262790   -1.980047097852   -3.954748726802
O      0.344460255194   -1.213692056152    3.236478490616
H      0.908858800459   -1.283987626246    4.007198227652
H     -0.507263429037   -0.841962616797    3.556774573943
O     -2.564895196043    0.077454685960   -3.764194205993
H     -3.229140653897   -0.404284188074   -4.258368349190
H     -1.699856111524   -0.194420249034   -4.149335543373
O     -0.689594133917   -3.346103403328    1.877310113216
H     -0.254848053088   -2.648769255510    2.406158335148
H      0.001636024944   -3.759620850646    1.358266632949
O     -3.437792308304    1.335272962262    2.555250776795
H     -3.148129644641    1.452913917533    1.619365282749
H     -3.531028832285    2.217109872871    2.917290961767
O      1.627916209480    0.989723725457   -3.634287402328
H      0.928993884921    1.561715696343   -3.272672639715
H      2.297889000912    1.630768767449   -3.925604449489
O      2.618940031728    3.518563316904   -4.008278917116
H      1.660006001761    3.571490689377   -4.110104599736
H      2.781126878073    3.811388376493   -3.097617285436
O      0.076607921606    5.736425577546   -2.274149146219
H      0.381808909278    6.293340693984   -2.993124769842
H     -0.908791107802    5.742216170450   -2.340564808171
O     -0.226292171781   -0.675144701647   -4.850680576382
H      0.379774880259    0.066773845230   -4.686978006782
H      0.209889313723   -1.428268685208   -4.429258010171
O     -0.110097120444    3.050059200111   -3.176194168547
H     -0.043143698858    3.881838744711   -2.687233524770
H     -1.048572561684    2.961172676574   -3.445001525540
O     -2.740372921347    2.781788893110   -3.717572038602
H     -2.723296421375    1.806971513653   -3.802108898857
H     -3.209998400056    2.958814237344   -2.886427966703
O     -2.050658427244   -0.379168257285    4.124093904753
H     -2.529189757127    0.283587127048    3.584120498621
H     -2.623355566954   -1.147186934081    4.170032952972
O     -2.525523908204    5.533724861593   -2.765814745581
H     -3.003154819952    5.060175792949   -2.067185449103
H     -2.572801188514    4.946287008782   -3.529297566936
O     -3.825826295470    3.598596116009   -1.254455124904
H     -3.320017885920    2.938362488738   -0.720137280120
H     -4.747911351131    3.478781260051   -1.025565025814

Ambimodal TS Water15-56
65
ANGSTROM
C      0.182084374847    1.721205052015    0.762285335284
C      1.317284954649    1.564078645891    0.029233110043
C      2.047149307450    0.325863663905   -0.156528244249
```

13327	C	1.692264439953	-0.882694412769	0.382624506447
13328	C	-0.213799629046	-1.416575561166	-0.829650363594
13329	C	-1.191029807615	-0.635208972305	-0.231350341387
13330	C	-1.528448804533	0.686520741930	-0.764832915578
13331	O	-2.536385138004	1.354474785635	-0.421455341354
13332	H	1.670757124772	2.415003084189	-0.555527127352
13333	H	-0.323549277776	2.676675123006	0.797988408707
13334	H	2.837441552602	0.346615428092	-0.908299110133
13335	H	2.261259645877	-1.775774235957	0.156816872391
13336	H	1.072282514223	-0.960566590884	1.265847915731
13337	H	0.191839261142	-1.142852777765	-1.791187609996
13338	H	-0.116158439487	-2.457718435708	-0.555816044323
13339	H	-0.158084146292	0.998393116417	1.490959792405
13340	H	-0.984640216427	1.006625983535	-1.669506581464
13341	H	-2.583632211757	-0.495317346653	1.285062875316
13342	N	-1.808880517217	-1.033092389660	0.932687767428
13343	H	-1.796318773856	-2.016879331971	1.179357111530
13344	O	-3.946012666199	4.506848958723	1.732378227124
13345	H	-4.580023863587	5.222244206242	1.690051547370
13346	H	-3.621958080619	4.361700591264	0.816818687072
13347	O	-0.454747919178	4.656786380561	-1.237379007148
13348	H	0.344362887175	4.897238468958	-0.719891155626
13349	H	-0.546117603112	5.295879245229	-1.973424120495
13350	O	-3.006164747189	-3.792893587735	-1.537372966408
13351	H	-2.541034484034	-4.394626364071	-2.120805515513
13352	H	-2.976294299952	-2.917321720335	-1.977589891874
13353	O	-1.026627922953	5.711937585056	-3.638898956580
13354	H	-1.984142822892	5.557399942274	-3.596903218555
13355	H	-0.666979152491	4.952499405910	-4.108253255072
13356	O	-1.997827498813	-3.882716250862	0.986396410244
13357	H	-2.365751481756	-3.856952469949	0.078523048179
13358	H	-2.649827991292	-4.338844631908	1.520000822870
13359	O	-4.207539485891	0.714912890322	-2.647205569870
13360	H	-3.926765709938	1.011473688862	-1.773911510114
13361	H	-3.714706588070	1.275516458090	-3.287143278793
13362	O	-2.682993328329	2.332289898557	-4.177811144492
13363	H	-1.758887700666	2.359769987396	-3.890347910895
13364	H	-3.040881132782	3.214958822377	-3.988191498046
13365	O	1.028298470243	4.405418052680	2.681675284746
13366	H	1.486115278176	3.648427654875	3.049265501718
13367	H	0.071917989621	4.223316322444	2.806958666970
13368	O	-1.553869519095	3.735242372408	3.014440999573
13369	H	-2.268980805114	4.318008031236	2.727310514942
13370	H	-1.923671124611	2.842469385903	2.894114851166
13371	O	-3.605716140609	4.762875585876	-3.204674217150
13372	H	-3.452931974299	4.484001898279	-2.268784807723
13373	H	-4.530287673229	4.999136520158	-3.278355257488
13374	O	-0.106223140064	2.805191950311	-3.167615360112
13375	H	-0.223775561045	3.385128291434	-2.383155709953
13376	H	0.799570679431	2.497011232014	-3.148466997840
13377	O	-3.244829426947	1.715713304351	2.210853864077
13378	H	-3.029905730878	1.573627898609	1.271944317908
13379	H	-3.844017759056	2.470963503659	2.222565975279
13380	O	1.700555948038	5.221115718048	0.223886789685
13381	H	1.933572097679	6.142428050917	0.347053344198
13382	H	1.477892817469	4.880820938973	1.120468452756

```
O     -2.974624968053    3.984961626079   -0.733404928463
H     -2.882785789812    3.008681938704   -0.693026009060
H     -2.048927248144    4.307164092481   -0.783340548717
O     -2.815897788188   -1.524340689430   -3.003216364761
H     -2.916453270291   -1.689870193086   -3.942130280488
H     -3.393592100031   -0.743027975159   -2.819491232179

Ambimodal TS Water15-57
65
ANGSTROM
C      0.131915920395    1.631295300786    0.855033961534
C      1.260261718892    1.528717514536    0.092038182052
C      2.014050470038    0.320751475709   -0.132018325195
C      1.687426450031   -0.909836213455    0.386520793906
C     -0.196527763480   -1.411934565636   -0.804154475002
C     -1.182317148591   -0.654882166172   -0.187394754401
C     -1.509746868634    0.695869998792   -0.657300394979
O     -2.524462639222    1.348712751813   -0.297503390614
H      1.570622682252    2.390517799232   -0.497258167236
H     -0.386421189971    2.576842794735    0.943902255032
H      2.795077306155    0.383752358302   -0.888534917327
H      2.273899697610   -1.785698144956    0.135598993022
H      1.097005149211   -1.010080497236    1.288588164011
H      0.204937228684   -1.110465612172   -1.759957723349
H     -0.099352418270   -2.465064061190   -0.577311420973
H     -0.149355263067    0.889709966516    1.589818841445
H     -0.970099754774    1.046128733676   -1.556847928167
H     -2.609491452481   -0.580464150220    1.288974142055
N     -1.789830101890   -1.082371131383    0.983591402316
H     -1.869900347347   -2.072869999180    1.149179689615
O      3.553179634466    3.322624664824    1.933728951696
H      2.862220689261    4.013043483777    1.981290553654
H      3.209869017624    2.605219285769    2.490250491227
O     -2.700276537288    4.161626268126    2.551983699911
H     -3.155998583695    4.962016461008    2.812559896775
H     -2.618148063157    4.196044858493    1.559010612148
O      2.686933997825    5.194762316304   -0.878839706851
H      3.177765116660    4.345625382815   -0.943075823749
H      2.915438992285    5.550486352468   -0.014821567220
O     -2.466168053985    4.092823712185   -0.059564098627
H     -2.553634775161    3.153416673005   -0.302379179617
H     -1.565600282001    4.361159308956   -0.300959952548
O      1.402406088226    5.015122879100    2.061621341591
H      0.848832061640    5.008104336564    1.264602798864
H      0.882213720663    4.565942427078    2.743278439950
O     -3.531223914513    1.570295799681    2.243718749033
H     -3.123158157209    1.515866092405    1.358182947743
H     -3.367848429085    2.483932062911    2.529006999708
O     -0.300240829566    1.456982055378    5.578508772213
H      0.470084295897    1.687741684326    6.100626865185
H     -0.002661860872    0.775019918919    4.947982967370
O     -4.895212816111   -0.142022026670    0.352514756672
H     -4.973599957075    0.293797482112    1.208759402681
H     -4.254645006875    0.387735342964   -0.142293074259
O      4.105501484911    2.928491260202   -0.618488295989
H      5.054138003910    3.027416000892   -0.706703605038
```

```
H      3.919084567165    2.965316687665    0.354278877273
O     -2.411383500722   -0.064607379526    4.046893464101
H     -2.294631848802    0.476975430832    4.832512906382
H     -2.864275400935    0.506533980263    3.388408763684
O     -0.268391509686    3.430318436254    3.650300945030
H     -0.451305761543    2.869610438793    4.423720481023
H     -1.134178948456    3.677264800891    3.284204318315
O      0.145864548946   -0.535121512901    3.627820370467
H     -0.841868634087   -0.445972784977    3.626312814696
H      0.327112568242   -1.418947064460    3.949001232993
O      1.988122662481    1.676903523347    3.507339642018
H      1.558554242846    0.829876314974    3.342966926835
H      1.282530309048    2.339909797909    3.469664741940
O     -4.022568142007   -2.786467140916    0.447368899595
H     -4.387008570673   -1.885174312656    0.384987080359
H     -4.765842509417   -3.377304419297    0.322863384677
O      0.084507083416    5.132009003364   -0.440313583745
H      1.020802951253    5.050611603230   -0.751640575407
H     -0.167661085581    6.035343479676   -0.640665199516

Ambimodal TS Water15-58
65
ANGSTROM
C      0.199407806418    1.544357248825    0.927177114963
C      1.286293806771    1.540042430998    0.106789605791
C      2.005421220938    0.361984704970   -0.323233703850
C      1.659046547153   -0.927302859875    0.023172867196
C     -0.238733914408   -1.290721194032   -1.072951199508
C     -1.221903650078   -0.549157262119   -0.419519954679
C     -1.545813988888    0.819418113134   -0.846333105294
O     -2.524743142374    1.486873349658   -0.468789555129
H      1.586517139120    2.479362711453   -0.361960657305
H     -0.331039330234    2.456932208835    1.163196036560
H      2.743500540332    0.511533548548   -1.111506153609
H      2.227193786758   -1.763101074274   -0.364437847392
H      1.129408531347   -1.142688382162    0.941742059190
H      0.129789567681   -0.963661558754   -2.033798759290
H     -0.164147453064   -2.355274179020   -0.894645081994
H     -0.080362768009    0.692688901871    1.531517797173
H     -0.939388422875    1.212945309867   -1.689211453887
H     -2.491941878020   -0.456438447426    1.213793305680
N     -1.922191539403   -1.065726027061    0.646178685388
H     -1.595162196802   -1.902454702767    1.097411809102
O      0.764612688277   -4.237858484625    3.252647868096
H      0.917420301654   -4.558486608986    2.343585822960
H      0.019390102557   -4.758086508919    3.592022287476
O     -1.494930259046   -5.770379418800    3.582763227386
H     -1.694630674438   -6.614815856699    3.986371790900
H     -1.476047400586   -5.920410371824    2.598811093039
O     -1.406109218870   -5.931287292582    0.955736602012
H     -0.553383251587   -5.556833348451    0.694233905279
H     -2.088297732617   -5.281532532131    0.696734611088
O     -4.094209597377   -2.171179220813   -1.140959823959
H     -3.747307174320   -2.803291048250   -0.484774374806
H     -4.657205659491   -2.664219001881   -1.738658709444
O      3.293631287654   -2.968693869454    3.173473594303
```

```
H      3.078299268437   -2.029334678064    3.343548119301
H      2.507799555932   -3.458504452148    3.452778065791
O     -3.898911199530    0.287489865419    2.681285423239
H     -3.569571065788    1.201460417565    2.593607564003
H     -4.420599126726    0.125744905460    1.863973736774
O      2.269102188174   -0.469528453231    3.499152415079
H      1.367315612577   -0.815052165308    3.319735711459
H      2.447336933604    0.177852794284    2.815467702933
O     -4.874831242052    0.119755158659    0.195381510814
H     -4.184926816607    0.718183101940   -0.133670043360
H     -4.693988050935   -0.721154872727   -0.257425896189
O      1.279293598258   -5.131471098889    0.693932628755
H      1.667855804581   -6.007723446385    0.665129997340
H      2.028495672379   -4.500952778888    0.553448739644
O     -3.083969125604    2.761482362754    1.871416143337
H     -3.844149751449    3.322739140559    1.709944766123
H     -2.839789452110    2.388860274660    1.004317063166
O     -3.408587409446   -3.799800366148    3.226793616944
H     -3.104541070135   -2.933466923618    3.555611174607
H     -2.747912063324   -4.451057190022    3.516922479290
O     -3.476565348843   -4.176099078384    0.650656709775
H     -3.464871607893   -3.922988711564    1.617769412794
H     -4.255558891853   -4.726815283234    0.553222659289
O      3.442699170302   -3.604426337343    0.626011523573
H      4.310683548889   -3.993065479682    0.512866049566
H      3.412517347254   -3.287540461469    1.570063765675
O     -0.054263451859   -1.679154535666    2.851300230803
H      0.231258032251   -2.596174431440    3.037187273568
H     -0.855908278350   -1.531572510893    3.378991688454
O     -2.487098850348   -1.387846151835    4.107075438565
H     -2.636752089025   -1.127287491416    5.016520107470
H     -3.024428654650   -0.763660637528    3.554248775673

Ambimodal TS Water15-59
65
ANGSTROM
C      0.207744929875    1.441641230398    1.006233730251
C      1.294105874659    1.402057936746    0.185211462076
C      2.021471969261    0.215447544977   -0.208865560189
C      1.666513035965   -1.069468448953    0.151645742729
C     -0.192105483192   -1.463749047991   -0.978821084207
C     -1.182536314761   -0.748976923491   -0.303791402488
C     -1.539120290142    0.620989201291   -0.711671169895
O     -2.535204070634    1.247190989757   -0.320202732346
H      1.586135273013    2.324242123916   -0.317240368591
H     -0.315634415908    2.368620498520    1.194132939114
H      2.758832429387    0.345179393432   -1.000564651564
H      2.240358114209   -1.911081189847   -0.216789667682
H      1.136009389659   -1.269873075398    1.074650329981
H      0.162563576348   -1.102999241273   -1.933210905973
H     -0.120764978265   -2.535457307997   -0.845432309887
H     -0.095158245950    0.615863173224    1.634609504026
H     -0.943087267691    1.043512976868   -1.546482770601
H     -2.512708761555   -0.724537971045    1.284992662777
N     -1.794451925183   -1.254008729286    0.813266651985
H     -1.790562392021   -2.242220804083    1.007204925965
```

```
O     -3.077222604349   -3.886069952389    0.898677473105
H     -3.600169522611   -4.499706820973    1.414678212492
H     -3.735330346718   -3.270230195194    0.456985327571
O     -2.216188366524    3.962245307196   -0.348477192122
H     -2.472907587989    3.024331581279   -0.423467824405
H     -2.177548624387    4.136081891979    0.606689186190
O     -1.819250120341   -4.439851233525   -1.415412069329
H     -2.220229732057   -5.253212450744   -1.725547544936
H     -2.195673548410   -4.275671297075   -0.522199057447
O      2.226659252287    5.095439403995   -0.336483796416
H      1.372754598561    4.816116690192   -0.750782898593
H      2.916758091161    4.750508703707   -0.905146814451
O     -2.272949928803    1.990550341756    3.869778652721
H     -1.349562801349    1.933431082677    4.151259408735
H     -2.315367769697    2.744069382427    3.255577758746
O     -2.927062278379   -2.191273653404   -2.528574610000
H     -3.238719763593   -2.435968125288   -3.400885886028
H     -2.413629075699   -2.951793610114   -2.188101380268
O      0.143793575807    4.721644332236    3.718331905531
H      0.181195536539    3.874777992924    4.196236312386
H      0.930400626997    4.727399469550    3.146844992049
O      0.068730745963    4.253119034799   -1.613944102455
H     -0.768168632444    4.118646230410   -1.093314540530
H     -0.183333222011    4.824203367635   -2.340874390746
O     -2.096707592685    4.327886987843    2.394909573744
H     -1.241834271436    4.556527744957    2.844682193792
H     -2.739359839914    4.971776504978    2.694895485279
O      0.433658085279    2.153526481879    4.714623771097
H      0.672485190936    1.954845439448    5.620431175141
H      1.242219896192    2.002148315254    4.170222379184
O     -4.898381474641    0.121569984003    0.654901126713
H     -4.581406387495    0.030108330818    1.569458339740
H     -4.198963622977    0.623263715765    0.205863162555
O      2.480782849930    4.558112153385    2.238186029644
H      3.085843253409    5.237728482208    2.539115998530
H      2.332875158313    4.730881111459    1.275583270872
O     -4.712385419211   -2.338000747785   -0.409178692551
H     -4.879807272391   -1.456065886584   -0.005075668612
H     -4.178518012291   -2.156272670682   -1.201819647694
O     -3.526469841742   -0.200317671548    3.014419575433
H     -3.059359606348    0.620730510390    3.291437397946
H     -3.954208196952   -0.538852536752    3.800796843756
O      2.612827197089    1.983754276075    3.181863419121
H      2.444443242786    1.432367150890    2.412580970521
H      2.644033995435    2.901384979580    2.851468857526

Ambimodal TS Water15-60
65
ANGSTROM
C      0.189277755036    1.851343477384    0.872938914317
C      1.313192130175    1.652829436831    0.128397225689
C      1.989063704163    0.390229155376   -0.054352238336
C      1.551191553007   -0.811849177503    0.451616249530
C     -0.258724676776   -1.168455029791   -0.845904809612
C     -1.280392350704   -0.435725246058   -0.256459519954
C     -1.557825361847    0.941999759237   -0.652589651864
```

13607	O	-2.532332865787	1.628039850621	-0.231615448321
13608	H	1.678933361342	2.476757691448	-0.485672368301
13609	H	-0.289969058415	2.821685442470	0.912056981378
13610	H	2.783090205808	0.378774349393	-0.800009185069
13611	H	2.086270715014	-1.726164136744	0.225992840823
13612	H	0.926091037360	-0.873178158585	1.333833025281
13613	H	0.214877042647	-0.806468440787	-1.745549355398
13614	H	-0.198098408589	-2.235554296647	-0.673675641558
13615	H	-0.147463347769	1.149801801989	1.623435494272
13616	H	-1.002583011804	1.319690221652	-1.523188610950
13617	H	-2.960753884015	-0.597928434133	0.934817808475
13618	N	-2.008933654741	-0.944662694628	0.821131671667
13619	H	-1.937278108776	-1.947200277041	1.007031338057
13620	O	-5.334438348089	-1.983504687360	-0.472137180658
13621	H	-6.257408624092	-1.830068463410	-0.684128671578
13622	H	-5.100825726730	-1.284211426808	0.177310996233
13623	O	-4.723331021829	-0.384798946794	1.555443465476
13624	H	-4.901297842623	-1.001226954184	2.304219353700
13625	H	-4.577888716721	0.491576969696	1.934959667154
13626	O	0.322809161877	0.916804188994	-3.484408956374
13627	H	0.249614931400	1.814745912008	-3.815979273139
13628	H	-0.519264229368	0.493228541274	-3.708009475999
13629	O	-5.058252590839	-2.273720120165	3.397502804721
13630	H	-5.081267829725	-3.026469991282	2.790453916270
13631	H	-4.197021280220	-2.324159045664	3.847024728423
13632	O	-5.124831490280	1.494189474965	-1.315290735692
13633	H	-5.156632301436	2.302459281741	-1.858107922210
13634	H	-4.294740861647	1.545672413875	-0.819523762445
13635	O	-2.284648800292	2.869219234899	-3.922859220784
13636	H	-2.283878886029	1.896084332576	-3.958333472091
13637	H	-3.210418637413	3.127991126613	-3.982197606084
13638	O	-4.790511061341	-4.039381785921	1.203821469397
13639	H	-5.066921535015	-4.865001301448	0.805455890346
13640	H	-4.962940722925	-3.332196618668	0.543991490767
13641	O	-4.844700932628	3.862780366531	-2.680358549690
13642	H	-5.433351123326	4.609640440564	-2.574284588631
13643	H	-4.021453755169	4.075705470317	-2.171712065104
13644	O	-3.401536187848	1.936342859150	2.303779023074
13645	H	-3.602764824593	2.866958341794	2.404097069787
13646	H	-3.052686352930	1.827061706226	1.391368937085
13647	O	-2.300801221501	0.095976847897	-4.077270785621
13648	H	-2.440280121135	-0.259630229495	-4.956180016722
13649	H	-3.073730347850	-0.213286244895	-3.540035659462
13650	O	-2.479382980615	-2.314394659015	4.363643624999
13651	H	-2.167694985244	-1.417479399798	4.082546035823
13652	H	-2.143958010692	-2.454267370860	5.249077019918
13653	O	-4.459425002277	-0.638724767524	-2.772396477614
13654	H	-4.789497991711	0.173032234716	-2.321631328083
13655	H	-4.533930433383	-1.330779151437	-2.101415268191
13656	O	-1.662354750542	0.010899736588	3.386070547613
13657	H	-2.303869758806	0.733589904264	3.318882924340
13658	H	-1.612527642679	-0.351531134804	2.476276460013
13659	O	-2.104795111450	-3.559125103815	1.845870183531
13660	H	-3.009287139533	-3.831035358501	1.624179940408
13661	H	-2.139570164080	-3.273793755248	2.770876031678
13662	O	-2.499801748390	4.081757464689	-1.450283073038

```
H     -2.511358764795    3.263222875581   -0.919158830877
H     -2.092205341893    3.819462729466   -2.293486225438

Ambimodal TS Water15-61
65
ANGSTROM
C      0.084943636396    1.736973442381    0.697405109394
C      1.186041113430    1.629165639523   -0.097241807978
C      1.975390945661    0.433454538614   -0.291789105029
C      1.699487874948   -0.784401063131    0.280094625340
C     -0.176637321439   -1.408764662707   -0.855552700128
C     -1.196212760251   -0.692015091654   -0.244623754379
C     -1.616610749871    0.614770275270   -0.751613424088
O     -2.628930093260    1.240754779988   -0.347376703988
H      1.442879098443    2.473518340517   -0.739445657633
H     -0.478369624909    2.660699707326    0.741453564657
H      2.723444884772    0.477267757842   -1.083027477751
H      2.309064897282   -1.649084250323    0.048230051863
H      1.134566415393   -0.868675526906    1.199904688037
H      0.190311147941   -1.111968547125   -1.826199881105
H     -0.017522994985   -2.448958008196   -0.602308657205
H     -0.164722108515    1.020865813512    1.468704392141
H     -1.131446321579    0.966185433645   -1.677256657493
H     -2.630896541207   -0.687346967861    1.250856148053
N     -1.782113426302   -1.124394067501    0.929762006630
H     -1.687399434850   -2.085694815165    1.207191135853
O     -1.540060274919    1.746127234069   -4.338996299062
H     -1.618496163192    2.615546543435   -3.877612338880
H     -1.381754625189    1.949487019108   -5.262552110355
O     -3.314523235894    2.438314712599    2.019340049674
H     -4.156612606779    1.946888754894    2.238282097154
H     -3.003991199946    2.026558432939    1.194183093588
O     -3.813362007767    0.193129313787   -4.002476749948
H     -3.048176652900    0.791164839824   -4.050118337427
H     -3.617208728582   -0.525925732351   -4.633971170352
O     -4.150153130538    4.675763886613    0.738170288585
H     -3.742471811377    5.457318297229    1.110535665831
H     -3.897521407545    3.922643153491    1.313799218862
O     -3.489516760480   -0.955642521532    3.404124720896
H     -3.589742025754   -1.455274068882    4.214308985906
H     -2.792620514292   -0.284585951510    3.569082862445
O     -2.026521857328   -3.065464887630   -3.272688713766
H     -2.621137204113   -2.648255471765   -2.622403992828
H     -1.243940217650   -2.496926617915   -3.296132829132
O     -5.352757971296    0.849847996521    2.441840812823
H     -4.865049888628    0.104504262167    2.832060649551
H     -5.538269589278    0.578120872570    1.521912470730
O     -1.946456038765    4.132333792156   -3.236626200902
H     -2.332582388367    4.795365680350   -3.810553632610
H     -2.657311874459    3.887067698573   -2.592451550845
O     -0.466544016162   -0.854385134785   -4.167240732790
H     -0.755461843581    0.063476595103   -4.062160407444
H     -0.929341339967   -1.174060455163   -4.948570706782
O     -2.970800393050   -1.940140961871   -5.483598304190
H     -2.664738728338   -2.465747755376   -4.697992581238
H     -3.601469102013   -2.493461517033   -5.944325538062
```

```
O     -5.174421400477    0.158108877876   -0.178041914382
H     -5.624687284394    0.602019657991   -0.929823145614
H     -4.282982770320    0.544188595201   -0.175419157417
O     -3.575145792712   -1.418639679789   -1.804269522187
H     -3.709212485780   -0.814026368999   -2.560860591474
H     -4.290805921968   -1.213627507322   -1.184339647158
O     -1.790072609834    1.121434276300    3.779403109559
H     -1.826392528608    1.580376029971    4.619357013749
H     -2.272385083856    1.694317538723    3.136613691719
O     -3.847700412377    3.322154076690   -1.591927488579
H     -4.065938302802    3.914997389886   -0.848441610865
H     -3.387566761211    2.561393930297   -1.181478181975
O     -5.774734905876    1.489610500600   -2.432763183675
H     -5.257377801757    1.009280433710   -3.095111917855
H     -5.252906886267    2.282719115714   -2.226065484598

Ambimodal TS Water15-62
65
ANGSTROM
C      0.194657669858    1.733209749647    0.784578382804
C      1.313110175085    1.594310644254    0.018743181303
C      2.054406604357    0.369201595765   -0.166356995089
C      1.690086957735   -0.847221101920    0.372177705967
C     -0.141379990097   -1.375557714271   -0.781165913174
C     -1.160802903400   -0.617681624778   -0.211522749604
C     -1.550576898287    0.703005601842   -0.735492698304
O     -2.572421924013    1.315160333585   -0.371686692112
H      1.628085456852    2.437557284428   -0.599394550931
H     -0.345104290928    2.670006085798    0.830533598010
H      2.829219204013    0.386040824534   -0.932991354505
H      2.273366021597   -1.733436128003    0.151713599399
H      1.115935793085   -0.911355514202    1.287918608712
H      0.254459250061   -1.099567070397   -1.746889126891
H     -0.051482812805   -2.425439465728   -0.534455986257
H     -0.093710837543    1.004703076908    1.528813103842
H     -1.012476651213    1.056578309134   -1.637064082247
H     -2.612311643602   -0.475339660747    1.219493950364
N     -1.838701231038   -1.037799888925    0.907676041578
H     -1.858595168245   -2.009536002630    1.189787377940
O     -4.686782625791   -0.542829659305    2.845947529422
H     -5.517952463346   -0.830087785538    3.227014189517
H     -3.986628537107   -1.095065990755    3.252102852488
O     -3.423819962155   -1.951213255857   -1.469263966867
H     -2.869715187134   -1.484456927415   -2.108967080068
H     -4.135669733920   -2.375811238378   -2.014669458781
O     -5.327696356195   -1.080436495392    0.221510571550
H     -4.526144697310   -1.326073472386   -0.279873870833
H     -5.038589513969   -0.763094736420    1.091633616516
O     -2.554153767729   -3.910202587817   -3.799474473047
H     -2.196506183997   -4.227698140393   -2.963158406110
H     -2.274652505282   -2.982088851827   -3.882903398244
O     -1.206690633492    0.372163551333    3.654865317470
H     -0.924323166951    0.938566894295    4.373646911389
H     -1.855908881722    0.905866681686    3.128979241908
O     -4.738758091290   -0.563536636548   -3.989759903209
H     -4.919632865662    0.037338546016   -3.233500147879
```

```
H     -5.201456538669   -1.388446225028   -3.798526841536
O     -5.262432405823   -3.665881250130    1.175947940719
H     -5.482698920740   -2.789132318906    0.809934990794
H     -5.283144441510   -4.273180930679    0.420202160406
O     -5.054179942746    0.902207007902   -1.731601456095
H     -5.415916330813    0.294141969117   -1.066204586035
H     -4.231584076055    1.242458622935   -1.353656993682
O     -4.798862769917   -5.249424612445   -1.085867859307
H     -3.843414487448   -4.994838672383   -1.022831893510
H     -4.826537706910   -6.205173590951   -1.121437929633
O     -5.074130972194   -3.296422526769   -3.045407246847
H     -5.306556631155   -4.013604064396   -2.436505224754
H     -4.291186700072   -3.624367148968   -3.538102671173
O     -2.198799326348   -1.139333775482   -3.804494499279
H     -3.150413803989   -0.891052191870   -3.987605095625
H     -1.672160385121   -0.632291266586   -4.421645097674
O     -3.076978775353    1.689051457079    2.307107735424
H     -2.949271974624    1.691335542924    1.339577642011
H     -3.854412364708    1.140293316336    2.479749964389
O     -2.317175581242   -4.296178826588   -0.827225365129
H     -2.194806736190   -4.316235960468    0.132035316340
H     -2.627420771818   -3.379491626555   -1.008307365498
O     -2.653134684951   -3.629852525586    1.844065289232
H     -2.579959866720   -3.252764138240    2.728816043053
H     -3.620495542640   -3.666831096706    1.657717511101
O     -2.625131354224   -1.843147857929    4.054230079681
H     -2.585951846936   -2.010410737567    4.996576219120
H     -2.001336827010   -1.098600169884    3.873071545770

Ambimodal TS Water15-63
65
ANGSTROM
C      0.166569005601    1.850186329805    0.713056947695
C      1.310409553318    1.745198427963   -0.017668127057
C      2.101212947375    0.546337489531   -0.168413835227
C      1.778895821031   -0.676255754305    0.377739377062
C     -0.025449648700   -1.258635194104   -0.831132132314
C     -1.096503480412   -0.565283630606   -0.274764051394
C     -1.537667645775    0.734105193391   -0.803180365135
O     -2.547944146111    1.356126901912   -0.418865539106
H      1.607040027953    2.595285749464   -0.635067012498
H     -0.400741796181    2.770164631662    0.728459408802
H      2.895096947804    0.587019014495   -0.914655069868
H      2.399033273887   -1.540517097950    0.175425092302
H      1.181268003923   -0.762539667390    1.276327224409
H      0.377098552774   -0.955659347672   -1.785939848846
H      0.141028381882   -2.294286391822   -0.569956378249
H     -0.115315979584    1.119005504995    1.456245462753
H     -1.022765307197    1.085432902060   -1.720468983039
H     -2.515284869873   -0.531393508591    1.244435830820
N     -1.775685310310   -1.060719206372    0.814121038159
H     -1.384942246058   -1.803251889974    1.370375804017
O      2.749708175426    0.999967611185    4.367009018246
H      2.351583333873    0.166315222779    4.659276041461
H      3.346292376367    0.747005244239    3.653041106719
O     -2.537845185589   -1.587008076667    4.726163329234
```

```
H     -3.225581925672   -1.412958213767    4.064374864311
H     -2.186322022356   -0.714359482313    4.940016842357
O     -0.685036805112    5.244945255031    0.360808076462
H      0.067777669783    5.206648744962    0.985613109434
H     -0.782039129054    6.165790774006    0.111822474497
O      4.564697706845   -2.517123748369    0.641329394366
H      4.405187362990   -2.009570424658    1.455185399553
H      5.290793405080   -2.081189129566    0.193523915685
O      4.221724981102   -1.114831874773    3.035896830943
H      4.942735783263   -1.371389568857    3.613789208407
H      3.404421682821   -1.436143525740    3.465767576106
O     -3.039183482301    1.838566690400    2.191893284460
H     -2.913676878728    1.649112519202    1.238828888645
H     -2.981448189519    2.811272641266    2.267421825276
O     -2.743098492514    4.534832051909    2.048934409250
H     -1.950098147202    4.729005391661    1.510154768369
H     -3.457383854034    4.579850262436    1.396293463690
O     -0.285209503817   -1.613868969779    3.236299854451
H     -0.997678957416   -2.017300858196    3.766459456384
H     -0.484457733955   -0.655490183143    3.346462011985
O     -4.251222472894   -0.529864132279    2.783260816694
H     -3.940322813081    0.388130203660    2.687871517314
H     -5.203823272240   -0.508365293269    2.692786067756
O     -1.139417808156    0.834663897894    3.851288174970
H     -1.841941861623    1.190189077499    3.273638151282
H     -0.474481817661    1.541954630617    3.932320508975
O      2.001407645190   -1.707639596709    4.514006563142
H      1.155833874770   -1.708990970959    3.987130775455
H      1.915204590196   -2.415064872326    5.153542356021
O     -0.910451617524    5.036308255939    4.160179118982
H     -1.713533273558    4.881647616694    3.642969237248
H     -0.594381095484    4.169459368642    4.450605421219
O      1.062316716437    5.247635977928    2.406781833875
H      1.735727643006    5.866798783485    2.689145247688
H      0.345037916957    5.282037249671    3.093183467802
O      0.757931752901    2.773082376691    3.923324023808
H      1.044285642002    3.294430805123    3.163673454646
H      1.517478503178    2.206247761430    4.165588231201
O     -3.319606453814    4.042403154107   -0.594881782368
H     -2.439493218422    4.399988505798   -0.745803210598
H     -3.215771503217    3.078797012877   -0.655467926247

Ambimodal TS Water15-64
65
ANGSTROM
C      0.400931855642    1.564855047327    0.862342945614
C      1.516453892614    1.448402774162    0.091351235677
C      2.155415352264    0.207973225909   -0.292433699781
C      1.716504243950   -1.045383535624    0.093847819563
C     -0.145392073879   -1.422396313003   -0.985013922127
C     -1.123822166622   -0.593506714685   -0.423645502445
C     -1.381833253795    0.745837229402   -0.993142803769
O     -2.304972211150    1.511367771319   -0.719850885574
H      1.932174308733    2.361119233642   -0.348401285701
H     -0.065046035186    2.521363433171    1.056058508396
H      2.916090688396    0.270192148077   -1.070517219051
```

```
H      2.245009715134   -1.920392017877   -0.261545738601
H      1.199181288023   -1.185153537273    1.034183828878
H      0.245359011057   -1.206029892512   -1.968399617521
H     -0.132712622251   -2.467036057427   -0.704394326747
H     -0.010165009009    0.740834022459    1.425702346081
H     -0.719314929869    1.003312732244   -1.854013839835
H     -2.469938695194   -0.338274528464    1.134338108376
N     -1.833237235827   -0.978102852633    0.677131486029
H     -1.688514938999   -1.889950721354    1.090675032748
O      0.686153868790   -1.566236353674    3.457303610024
H     -0.167047678086   -1.330674012151    3.926712032701
H      1.157258592980   -2.151281596727    4.053632657222
O     -3.243956876350    1.295769014057    1.837710529029
H     -2.652473779474    1.886919391480    2.336624880323
H     -3.128445564405    1.533095176706    0.900951930306
O     -1.731449568446    2.900441492748    3.506778834295
H     -1.246580651995    2.373306881799    4.167734593516
H     -2.453759529973    3.319766665589    3.977489861879
O      1.753560307800    0.906514499068    3.784225439399
H      1.480274499093   -0.012140201276    3.577189476985
H      1.684240683236    1.394610386639    2.958717042932
O      0.936862401130   -5.058699298655    0.577261787320
H      0.798596819665   -6.003747571770    0.648523850793
H      1.051348157168   -4.871649563640   -0.377795313815
O      2.948328003555   -2.616524991516   -2.713972890717
H      3.658204943959   -2.874766466575   -3.303311357314
H      2.395661069159   -1.975185103169   -3.199175419312
O     -0.721421525804   -3.353785893532    1.891388188934
H     -0.168816701703   -4.010507990447    1.428737712179
H     -0.099424939936   -2.739919756492    2.317546001739
O      1.409348808972   -0.894645770603   -4.201286164696
H      1.464690268322   -0.896422522013   -5.158077694003
H      1.428657067853    0.041359334463   -3.927616000966
O      1.138260036384   -4.541015793569   -2.076889252822
H      1.852999810689   -3.889839214725   -2.230423355203
H      0.344776583905   -4.152866475349   -2.450885465832
O      1.383011104670    1.722175962870   -3.372138478576
H      1.591242597500    1.691924099027   -2.432003869207
H      0.593374904101    2.260477012693   -3.468215488350
O     -1.441119654092   -0.957579313949    4.830838836996
H     -2.345408691586   -0.746996991136    4.463879109103
H     -1.145495533436   -0.127164732012    5.230801952108
O     -3.827616728722   -0.399532059642    3.917208682291
H     -3.764740804940    0.194387372197    3.153060942867
H     -4.208571112212   -1.235827432767    3.582365413530
O     -4.612711014003   -2.890668850191    3.176790415540
H     -3.766899497576   -3.283341755507    3.498124712013
H     -5.304679837600   -3.275636823655    3.715141005479
O     -2.235007594708   -3.677598525828    4.132236549210
H     -1.974698644946   -2.866515179421    4.586424096895
H     -1.725542977985   -3.689422333110    3.298495462965
O     -0.259859089272    1.522448055162    5.399191365920
H      0.064549565404    1.960218459135    6.186598183902
H      0.533362598457    1.330315743430    4.838398250544

Ambimodal TS Water15-65
```

```
65
ANGSTROM
C      0.248789921380    1.667268941271    0.777589370730
C      1.360476166130    1.557308935840   -0.002334701348
C      2.088764871512    0.335985054652   -0.254822070413
C      1.710852274986   -0.899370458610    0.226234698129
C     -0.132707412878   -1.363742933618   -0.949637817374
C     -1.146225507938   -0.651614906938   -0.309580880340
C     -1.518325464381    0.706633115119   -0.750991927688
O     -2.505044720848    1.351389305734   -0.352082410612
H      1.679929933191    2.427999115437   -0.578733033519
H     -0.280400283376    2.606398775928    0.872727823802
H      2.860510285741    0.383995089639   -1.023328052383
H      2.277679955933   -1.784226196958   -0.038632651686
H      1.128399318409   -0.991806138639    1.133699988576
H      0.248317264092   -1.022821710611   -1.900761867976
H     -0.021480119704   -2.423277642204   -0.760362450773
H     -0.042175081681    0.904320680621    1.484625089028
H     -0.974501681683    1.081111780656   -1.643130522086
H     -2.492100134039   -0.639183221326    1.284512619038
N     -1.805284371743   -1.175495398257    0.769504442189
H     -1.574578000453   -2.089310139234    1.115619386725
O     -2.243259597553    1.103669514411    4.611437954433
H     -2.840576526811    1.465533130561    5.266635302753
H     -2.381559552424    0.137250451916    4.616863023517
O     -0.115981810451   -0.583656199076    3.306217448630
H     -0.972089440738   -1.017208804281    3.532190745874
H     -0.112267673247    0.226453372456    3.822031492858
O     -2.220323345631   -4.230144290633    4.454073383742
H     -1.414002557242   -4.420013797927    3.949956739968
H     -2.925995708919   -4.144773178800    3.793627497264
O     -4.953465981315    0.214949223734   -1.045451797172
H     -4.233057505864    0.833013509832   -0.854445252054
H     -4.514265227198   -0.628680482452   -1.242062067933
O      0.214908073917   -4.620874370935    3.083219171129
H      0.754072056798   -3.823650297379    3.319900323957
H      0.627185172064   -5.361399706457    3.530385877847
O     -6.514325769219   -2.523798238533   -0.906013688435
H     -6.746015454812   -1.601835454008   -1.052547590127
H     -6.501920052341   -2.642125651433    0.065262381792
O     -3.468074368339    0.754170450235    2.140045833408
H     -3.158350942917    1.216735389433    1.340066548683
H     -2.968776619614    1.091027879322    2.900132444988
O      1.696501618504   -2.498145395150    3.622257678219
H      1.049001312670   -1.754931751327    3.576307576564
H      2.366959236373   -2.303277365751    2.965297249284
O     -0.151145788793   -4.467855515774    0.382340825048
H     -0.046467955849   -4.499868802812    1.353783341851
H      0.003084419076   -5.359787715064    0.067697800014
O     -3.988894640226   -2.369845947711   -1.656471414372
H     -3.926687679287   -2.626835609553   -2.577068626897
H     -4.921906238199   -2.568352567260   -1.379424790442
O     -2.891103416350   -4.143080750473    0.164840702142
H     -3.165489965691   -3.489068897270   -0.499563311218
H     -1.921593717781   -4.195408316768    0.135975898262
O     -3.834286375447   -3.106846451189    2.447257402935
```

```
H     -4.771210769984   -2.923081215668    2.276590735800
H     -3.502877988507   -3.521988191118    1.622257765579
O     -6.014572580548    0.141423564701    1.445808772754
H     -5.784414926312    0.216895496870    0.496179199000
H     -5.185954699827    0.384631917330    1.896090906658
O     -2.432355048265   -1.582106641462    4.175237139177
H     -2.246176278166   -2.445196815429    4.599578971261
H     -3.038282852220   -1.833440075162    3.451745902465
O     -6.424076138979   -2.390723904318    1.805925047196
H     -6.277478819041   -1.400272985305    1.724425454275
H     -7.184575983452   -2.507307936167    2.375042907139

Ambimodal TS Water15-66
65
ANGSTROM
C      0.256581057733    1.722514678799    0.836992775106
C      1.319335633926    1.579147975320   -0.003607476941
C      2.025677879017    0.349370552120   -0.256533848225
C      1.667651470344   -0.878372334187    0.276272384429
C     -0.171136369352   -1.339737677087   -0.742043192216
C     -1.180464322010   -0.582605663899   -0.152899539894
C     -1.560248462378    0.731507116028   -0.666114469368
O     -2.549713882801    1.391642125314   -0.298392384510
H      1.600840258556    2.425845278158   -0.632914151671
H     -0.277352846478    2.660181708574    0.917285044429
H      2.739510076312    0.361320138602   -1.079782720574
H      2.235209830608   -1.756206061883   -0.005743733429
H      1.189107814747   -0.953050461477    1.244301381009
H      0.175057396547   -1.064222234627   -1.727109683317
H     -0.092283681913   -2.390371812265   -0.502633807275
H      0.012937403694    0.998452246970    1.599514443222
H     -1.004063065860    1.058365741159   -1.566094300106
H     -2.655124753747   -0.505522227311    1.252316122428
N     -1.815350401340   -1.018137773082    1.020417818333
H     -1.885921536597   -2.022028360008    1.153921672049
O      2.034607783112   -4.060639682721    1.986897733036
H      1.532642439208   -4.130909349345    1.156209310391
H      2.672216323644   -4.801584659761    1.960543494465
O     -2.355689502285    3.686190509694   -1.659548209456
H     -2.493950701260    2.903341624818   -1.074643389698
H     -3.205376525896    3.861375440484   -2.065973651961
O      4.265345631176   -2.444781340364    1.919860369293
H      3.409078907260   -2.876850011765    2.089157784551
H      4.557009472024   -2.788621688552    1.064291030716
O     -0.269460683706   -3.839188500948    3.450474616497
H      0.638885479817   -3.937130943085    3.111193430013
H     -0.357922269383   -2.889826432868    3.686378508202
O      1.656634194671    0.440705688283    3.724840291198
H      2.510129083292    0.331326054344    3.250000476216
H      1.884694780526    0.744327137823    4.603849477099
O     -1.690493453106   -3.982233809843    1.309469285483
H     -1.165393776863   -3.935999703747    2.175816020404
H     -2.429501133005   -4.565085633009    1.485286874135
O      0.423504565763    0.479070595304   -3.738748025400
H      0.265574628390    1.415900250124   -3.503451444305
H     -0.165292319496    0.297436295970   -4.472787621939
```

```
O     -0.529726136637   -1.181167952339    3.652780329224
H      0.271221549225   -0.630183521895    3.730425793479
H     -0.947646744418   -0.940577191212    2.813642970256
O      2.516189220868   -3.735164479814   -1.936597616698
H      1.705554583160   -3.912755957953   -1.441901726073
H      2.424506029329   -2.855588738382   -2.343689671658
O      0.423563441063   -4.633910211139   -0.193687805060
H     -0.413064406369   -4.423460345139    0.284111137187
H      0.476034547140   -5.589443946745   -0.253242697222
O      4.007972699570    0.180345521464    2.439007800224
H      4.172955144377   -0.780580820598    2.323686218204
H      3.927888310383    0.542068897841    1.553911489322
O      4.689502719375   -3.906197548938   -0.452581226704
H      5.410941608519   -4.030817524530   -1.069475311140
H      3.866576078236   -3.847908805364   -1.003664490761
O     -0.225541479124    3.065709030379   -3.201529961401
H     -1.023142064085    3.283117917925   -2.668935102873
H      0.442732432645    3.696243926931   -2.929885310224
O      4.116754804806   -5.722335289792    1.528755389732
H      4.768481859022   -5.555754948579    2.212283366665
H      4.368990118229   -5.156323037093    0.772885258649
O      2.477211361128   -1.288335610692   -3.228762142417
H      1.759012513451   -0.640454154413   -3.337895304648
H      2.920691668354   -1.342546836423   -4.076302437674

Ambimodal TS Water15-67
65
ANGSTROM
C      0.167858236762    1.658655417580    0.691968220626
C      1.281651055555    1.502284651922   -0.075149771894
C      2.009488764470    0.265888378020   -0.273228150259
C      1.648321675966   -0.945242548893    0.264772357254
C     -0.228277802492   -1.436863609099   -0.930645254093
C     -1.227673720096   -0.689872993247   -0.323305743581
C     -1.596252857124    0.636922010682   -0.816667794013
O     -2.572946593003    1.306695434123   -0.396573635102
H      1.606173519118    2.345316601414   -0.687183980913
H     -0.339262278820    2.613869707479    0.740987947661
H      2.786193278422    0.288741549218   -1.037689214857
H      2.213840406868   -1.840599492752    0.042464014702
H      1.045036455181   -1.020058828246    1.159179314197
H      0.172039864445   -1.134087637553   -1.885974193610
H     -0.109016789351   -2.482783022049   -0.684780385267
H     -0.152651750864    0.931461023022    1.426142550394
H     -1.092483871356    0.989111593803   -1.731646249842
H     -2.692022547146   -0.676942220452    1.130080399969
N     -1.837279264668   -1.118391611113    0.837117302722
H     -1.709625130712   -2.073022806552    1.134042110357
O     -3.563924650397    4.181240463471   -2.805902061872
H     -4.122066702990    3.455406529264   -2.419749149702
H     -4.110708171748    4.620209971131   -3.457764454105
O     -0.590590451527   -3.485671471029    2.084052441331
H     -0.871724060329   -4.171924838622    2.691002449548
H      0.370039824973   -3.628733503522    1.929019668218
O     -2.707798740347   -0.061728107194    3.888158930964
H     -2.958836605497    0.643346494535    3.258546804959
```

```
H     -3.055490904582    0.198173812976    4.741613385083
O     -1.442448444353    2.629565056321   -3.541241228956
H     -1.038299058308    3.034905136780   -4.309417881961
H     -2.163854110512    3.227412365797   -3.260047452908
O      2.074103635884   -3.799565413655    2.023091931890
H      2.227020989253   -3.223641225856    2.813007744390
H      2.522026525171   -4.625564947816    2.208663643759
O     -3.541912533677   -1.342173308558   -2.014741287869
H     -3.658936680545   -2.271684931089   -2.214375488381
H     -4.354402225253   -0.897261314229   -2.353152717221
O     -4.878750020725    2.223372516911   -1.609401622965
H     -4.107589083019    1.728324311905   -1.284139830803
H     -5.233543728020    2.678653016755   -0.812511045945
O     -2.857362838112    0.332016601173   -4.253537141840
H     -2.759396899723   -0.291870159008   -3.520976730407
H     -2.336209453441    1.114203070884   -4.006864054068
O      2.230934699790   -2.287222411334    4.209686114693
H      1.300646410265   -2.023043881024    4.275690363043
H      2.690360799245   -1.450046468053    4.052947085922
O     -2.632247635115    4.070356918093   -0.125815913548
H     -2.775494604242    4.478761286598   -0.988169855466
H     -2.498311102584    3.126581879235   -0.322612715706
O     -5.447757374027    0.079135529108   -3.276731956939
H     -4.740888179777    0.257711369425   -3.920454522275
H     -5.503681162432    0.877621968043   -2.730250530863
O     -3.317162692567    1.904135877903    2.111607586509
H     -3.094106295295    1.579003296049    1.211916711487
H     -2.805266572001    2.709477371848    2.222708370139
O     -5.195333061499    3.539216250234    0.696298618509
H     -4.337604708940    3.966615622877    0.501335912497
H     -5.000845440971    2.899918810099    1.392462600763
O     -0.201855175545   -1.086858144368    3.469435692628
H     -0.400723824903   -1.861268565986    2.914550123083
H     -1.070099446440   -0.702938945239    3.682089125102
O      2.181436973075    0.394426656572    3.443301961916
H      2.282259776589    0.841337421064    2.601946320640
H      1.276065221574    0.040631285079    3.461273941336

Ambimodal TS Water15-68
65
ANGSTROM
C      0.230826373350    1.734221308425    0.779093497983
C      1.321146416841    1.603170835173   -0.025151220726
C      2.049511297798    0.379303936475   -0.264088726599
C      1.699862671722   -0.850619560592    0.263520885340
C     -0.135991793068   -1.395272174086   -0.771174661844
C     -1.158560697021   -0.639202426900   -0.200993925781
C     -1.577564539604    0.653830770470   -0.752843242602
O     -2.561233683670    1.308946843331   -0.379071465361
H      1.625284928198    2.466471427959   -0.623077770303
H     -0.309066995014    2.668601073588    0.851724069152
H      2.796780436316    0.403041717841   -1.057565694222
H      2.287391888738   -1.720241105308   -0.000501706611
H      1.186698387249   -0.927187971361    1.214322936608
H      0.211011232726   -1.158612755455   -1.765568791993
H     -0.031764178078   -2.435389889424   -0.495832989718
```

```
H     -0.060261469334    0.987705028584    1.503176163950
H     -1.043324953144    0.973858023342   -1.671765075433
H     -2.605840981900   -0.512773421937    1.220659794592
N     -1.772653320929   -1.025884053059    0.976348577732
H     -1.778160581300   -2.006845310971    1.223139998797
O     -2.456831119168   -0.379508315472   -4.340948200386
H     -1.625708110036   -0.876473616736   -4.427409496762
H     -2.196005453034    0.551970931769   -4.261785376709
O      0.880485666259    1.042901392024   -3.658037521109
H      1.139980151780    1.201594715538   -2.747365730662
H      0.055490102251    1.542608426102   -3.807826330376
O      0.812096368625   -4.788836487869    1.018715130713
H      1.033528526409   -4.659333347379    0.064660678945
H      1.497836205799   -4.298220731159    1.500229833559
O     -1.714421734745   -3.955795250927    1.155933909180
H     -2.226897213815   -4.656679335522    1.560082564721
H     -0.780156774106   -4.270572326152    1.143933101372
O     -3.449812107304   -1.314181280728   -2.040923269736
H     -4.397659209990   -1.389023852200   -2.159959638276
H     -3.097345391068   -0.977447902995   -2.897450204152
O      4.020088662811   -3.605795713213   -0.613584385659
H      4.430090325936   -4.441043202524   -0.865621796075
H      3.133228754521   -3.659069813624   -1.020985134655
O      3.604985362463   -6.375307798158   -1.296550736668
H      3.378907687168   -6.526596624040   -0.348211346159
H      3.795724278765   -7.237693372099   -1.664881297369
O     -0.292521575139   -3.987520812336   -3.212094547976
H     -1.110928697877   -3.991810960595   -2.665797689178
H     -0.222025200830   -3.097105359087   -3.580675831245
O      1.607978372780   -4.416691360375   -1.485323907391
H      2.080368369537   -5.227923332195   -1.721460313305
H      0.906420614435   -4.270568548728   -2.176896706595
O     -3.140496652700    3.175284739154   -2.161142182379
H     -4.073528611706    3.062120749188   -2.349972201060
H     -2.949306522943    2.560714013730   -1.406608640998
O      3.268535612100   -3.853103656448    1.924073727491
H      3.612889590750   -3.720063771073    1.004680154835
H      3.606399485935   -3.125073509629    2.445047676929
O      2.882678441347   -6.608449201859    1.277679248940
H      3.337977969572   -5.893452665582    1.740401874150
H      1.961352874098   -6.302383250644    1.225243368800
O     -2.413402747857   -3.807451770139   -1.574634093461
H     -2.782131035087   -2.916334327426   -1.720990340758
H     -2.185948152607   -3.848425635480   -0.632447309985
O      0.109720357648   -1.420209917876   -4.412750771772
H      0.515060741900   -1.639242788582   -5.253006650233
H      0.494562520152   -0.560515034017   -4.133375105611
O     -1.550718412299    2.262837989500   -4.059268044281
H     -2.114537972673    2.635752554537   -3.331054131118
H     -1.630698747936    2.868738136741   -4.797231364851

Ambimodal TS Water15-69
65
ANGSTROM
C      0.118808854318    1.634071035926    0.811290593236
C      1.240947342783    1.503937568580    0.044120339016
```

14223	C	1.966669544426	0.281004856367	-0.183395784382
14224	C	1.586666336615	-0.950484336563	0.313242080241
14225	C	-0.257934111424	-1.392654303743	-0.840076220965
14226	C	-1.256389106936	-0.623925446875	-0.244637175910
14227	C	-1.588443145257	0.721534133356	-0.731717415188
14228	O	-2.590303898745	1.381633162033	-0.382132578050
14229	H	1.552099971556	2.367642044741	-0.544071897050
14230	H	-0.388294512431	2.587321512304	0.866752244697
14231	H	2.742102278497	0.312115594030	-0.949235282111
14232	H	2.156943217224	-1.836798909383	0.059677261616
14233	H	1.020796614984	-1.037721024642	1.232534971062
14234	H	0.137317169090	-1.107164045569	-1.803335559083
14235	H	-0.194821601256	-2.449780717801	-0.616264816408
14236	H	-0.178145710047	0.905067937652	1.553536574929
14237	H	-1.024103187655	1.067846651644	-1.616990123902
14238	H	-2.689173680135	-0.555478525282	1.244316468044
14239	N	-1.900063784190	-1.068758432688	0.891258779651
14240	H	-1.836308520518	-2.038512472043	1.154274915674
14241	O	-1.290740180523	0.576032607996	3.822595283445
14242	H	-0.994126001884	0.730285562862	4.721407220765
14243	H	-2.279032821256	0.492372303575	3.864352858220
14244	O	-1.135607176012	-2.202181550631	4.092407953605
14245	H	-1.008733551303	-1.279150495860	3.816908237746
14246	H	-2.031607928015	-2.223983870145	4.501074897953
14247	O	-4.458702090996	3.069618820696	4.629816846445
14248	H	-4.627237684587	3.317138426817	3.687375115261
14249	H	-5.145745539448	3.483599261352	5.151258837443
14250	O	-2.552680158605	3.916080276039	-1.586817695472
14251	H	-2.688012111980	3.039030782902	-1.194728836629
14252	H	-3.116764867848	4.531783663210	-1.068523215172
14253	O	-0.198555878155	4.722458823746	-0.692843633103
14254	H	0.043153070661	5.480459095324	-1.226080730315
14255	H	-1.040691494347	4.377440237174	-1.092496505410
14256	O	-1.979026658788	3.177343036577	3.044346146749
14257	H	-1.572369742126	2.298169370589	3.118901851086
14258	H	-2.448327410416	3.334957480717	3.871097597651
14259	O	-1.657157037669	-3.769454773593	2.093835063607
14260	H	-1.377443762691	-3.191785034578	2.865753367965
14261	H	-1.165235192839	-4.586173221572	2.181740494789
14262	O	-3.734690820866	-2.109367177177	4.746413914236
14263	H	-3.933288903514	-1.177893407706	4.557472283632
14264	H	-4.097770853945	-2.610887212249	4.001331304034
14265	O	-4.543265172379	0.762739096878	1.310717291836
14266	H	-4.942241364325	-0.063875630247	0.985808694745
14267	H	-3.837728476071	1.000028964349	0.661780336974
14268	O	-4.302462188551	-3.587465137055	2.410589892248
14269	H	-4.652563720889	-4.476338958491	2.482416593180
14270	H	-3.335435069958	-3.678932204449	2.236381608424
14271	O	-3.928146349245	0.504949986406	3.891867232817
14272	H	-4.182347005538	1.351548989323	4.303045479766
14273	H	-4.196367145898	0.581165117111	2.947105625045
14274	O	-3.850830208749	5.501228973115	0.176846777012
14275	H	-4.360620832351	4.915992474112	0.752011524866
14276	H	-3.040987428797	5.716868269686	0.685186197925
14277	O	-1.499044672810	5.572228638556	1.552434064503
14278	H	-0.921768722962	5.311419666687	0.806706463293

```
H     -1.538242410470    4.800188109196    2.130374145727
O     -5.357129950210   -1.746472695030    0.647841768477
H     -4.969942112540   -2.385188479891    1.273919760502
H     -6.281742712664   -1.976501505708    0.551358110783
O     -4.521423386683    3.422997576009    1.993468102401
H     -3.547494076802    3.400656323203    2.037723431903
H     -4.777085670509    2.562453358004    1.627215216826

Ambimodal TS Water15-70
65
ANGSTROM
C      0.164085726964    1.701436961280    0.788775029339
C      1.302391992004    1.557475140266    0.056012609077
C      2.040189810251    0.323925231382   -0.124983635960
C      1.662879717757   -0.894397950697    0.391449051837
C     -0.177110163545   -1.391834387969   -0.838636796073
C     -1.177196880561   -0.626634737842   -0.254060055622
C     -1.550456251919    0.698756193230   -0.771600915158
O     -2.551524644049    1.340562886569   -0.393431615565
H      1.646755493335    2.404503924529   -0.539632335995
H     -0.354395101103    2.648957961702    0.838606039331
H      2.834549553137    0.347268851172   -0.871353293309
H      2.237985895925   -1.784224296613    0.167700018780
H      1.045720236047   -0.978008530571    1.277468462271
H      0.235715826527   -1.097565377840   -1.791942545143
H     -0.084024458131   -2.442508618232   -0.599030654614
H     -0.163158148831    0.965094266017    1.509817686751
H     -1.014068793384    1.048680566547   -1.674786835679
H     -2.612170634402   -0.499411339940    1.202847251895
N     -1.815298655176   -1.030004595413    0.895142100769
H     -1.785459775766   -2.000498016251    1.193800657222
O     -0.816701832456   -4.793732494339   -0.463916437558
H     -0.764987718725   -5.750417555466   -0.457513415357
H     -1.589777186723   -4.562977362754   -1.017650068131
O     -3.420251747485    1.894936428223    2.195960259668
H     -3.036940965653    1.768987831430    1.312123815854
H     -3.745645221506    1.026243241531    2.489919190570
O     -3.534707497173   -1.626143937053   -1.658489469857
H     -3.968966822229   -1.591340070209   -0.783523026545
H     -4.168507135629   -1.198071520156   -2.260301774263
O     -5.081307911626   -1.393705624455    0.588331336505
H     -5.347413035727   -0.516541171081    0.261445896908
H     -4.774906186080   -1.236180741640    1.502083403649
O     -1.972484348584   -3.722880838627    1.782112180212
H     -1.399041799109   -4.139657013356    1.115390666282
H     -2.886584681769   -3.944773506216    1.504403686087
O     -5.671579906409   -0.270959799529   -2.727808361509
H     -5.603874048256    0.398523670480   -2.028662280563
H     -6.510844107663   -0.715213125242   -2.595332033346
O     -1.451344846639    2.043115553762    4.063192647470
H     -0.782640739841    2.700082040060    3.792279462511
H     -2.174733195950    2.097010044835    3.405607757802
O      0.745501570763    3.546037226774    3.418154149370
H      1.338254877636    2.765566971778    3.390832687878
H      1.075037814808    4.107240393491    4.121151346240
O     -0.104209862090   -0.165950091864    4.049653190666
```

```
H      0.053176465504   -0.346112350908    4.978630133022
H     -0.674836466164    0.665082761984    4.038839383537
O     -2.164267609196   -1.998206798951    3.908521848882
H     -2.028877263107   -2.722249207354    3.275453760049
H     -1.382638335427   -1.419770708162    3.838764906877
O     -4.190256390282   -0.633490754627    3.031839461889
H     -3.409657634622   -1.160129910539    3.379724431693
H     -4.822094098665   -0.605138785928    3.750849082495
O     -4.374422213196   -4.155654194623    0.613928217407
H     -4.050586108819   -4.320842027134   -0.282462475704
H     -4.795589036575   -3.286157539015    0.593991803901
O      2.164383661891    1.222000954069    3.418715892259
H      1.401616759587    0.637651190899    3.612539083077
H      2.471626280778    0.978581719055    2.543565175953
O     -5.237016259029    1.181133932550   -0.383297711146
H     -4.265914291306    1.255178805381   -0.484418693325
H     -5.482401840319    1.903926333319    0.198573294329
O     -3.060081806509   -4.185614116069   -1.907058432491
H     -3.172724355062   -3.197774279701   -1.859751004688
H     -3.124458330657   -4.418476321609   -2.833540327975

Cope TS Water15-1
65
ANGSTROM
C     -0.238009756286   -1.205820245027   -0.817610653143
C      1.073577333779   -1.545980134499   -0.226777628702
C      1.963035965168   -0.604108887837    0.134403173278
C      1.601675498946    0.874869760325    0.042098372746
C      0.239467439961    1.145371727755    0.833293817373
C     -0.993422014064    0.472877169355    0.227903012252
C     -1.604606443113   -0.722585873485    0.784073905351
O     -2.769908391026   -1.139306839521    0.527093134346
H      1.273210289240   -2.600887840870   -0.038328343571
H     -0.895091084558   -2.049583762067   -1.017603043863
H      2.911000057806   -0.865273244307    0.595410364350
H      2.383724675171    1.506142843413    0.474802115449
H      1.445082821262    1.184465391822   -1.001205684059
H      0.381874069935    0.835166981692    1.873441533257
H      0.062168522063    2.227752170276    0.825279520992
H     -0.209440720250   -0.542685832377   -1.681631133981
H     -1.040128931490   -1.239807299301    1.582656064141
H     -2.565216559238    0.911922736958   -1.040423237189
N     -1.711972765427    1.256797784463   -0.623121746091
H     -1.272888381236    2.031506379772   -1.090215194452
O     -2.931491171491    1.526949349291   -3.515853136910
H     -3.437837409394    0.872117606498   -3.009011700991
H     -2.025534640029    1.171633126593   -3.572410386560
O      1.701190749196    4.358383949468   -0.337221575079
H      1.951073095979    5.278339449061   -0.231136970311
H      2.398577627223    3.939804290585   -0.883916762121
O     -1.690319279817    4.040109280962    0.295213910134
H     -2.268602275371    3.473708401196    0.830951926629
H     -2.152877451508    4.152525722173   -0.543607386100
O     -4.083493162375   -0.210780808503   -1.658099286791
H     -4.864704458103    0.116825023121   -1.204381964192
H     -3.615952047085   -0.754902823219   -0.988062682794
```

```
O     -0.330746287981    0.623994813653   -3.625433584072
H      0.299885833587    1.371269113439   -3.680578759632
H     -0.176197405090    0.074501141268   -4.394640476770
O     -1.741431773596    3.218071270391    3.922032329253
H     -0.918345516174    2.692146803313    3.928168613589
H     -2.293870989889    2.846510853129    3.221895994919
O     -2.710893563174    3.880640079310   -2.346985212130
H     -3.268925819132    4.488692326071   -2.832321013621
H     -2.827516143785    2.994782725647   -2.780856400268
O      0.730580201197    2.017806440585    3.883193458169
H      1.090453929250    2.135319628473    4.763206293879
H      1.134326383322    2.738130524623    3.335821031258
O     -0.693540466634    5.343638197777    2.562621444938
H     -1.002206029758    5.065353603377    1.686223555009
H     -1.129692883905    4.728107556805    3.186136454875
O      3.418659979567    3.105970547258   -2.019458512112
H      2.756971349358    2.914982630199   -2.712739896997
H      3.773329579862    2.262526177569   -1.734339720363
O     -0.032300165759    4.465479116844   -2.466446733768
H      0.339945229136    4.455039117015   -1.564724980254
H     -0.987704554590    4.290424007861   -2.386384644255
O      1.652388139482    4.072217346212    2.470717491194
H      0.869647562261    4.658408301196    2.604995497550
H      1.711144316154    3.980647802024    1.509449625755
O     -3.543043472853    2.612534212375    1.806035869974
H     -4.217982167309    3.243608332704    2.062569499008
H     -4.011691383721    1.793855614574    1.537283510079
O      1.351687499947    2.741956441556   -3.785346173776
H      0.794342082394    3.417364339163   -3.283950913183
H      1.512637028765    3.133460117878   -4.645132434989
O     -4.861657616675    0.341673446100    1.189231636675
H     -4.099660848505   -0.245774070891    0.970028722987
H     -5.292989663543   -0.061696282735    1.943778400950

Cope TS Water15-2
65
ANGSTROM
C     -0.164891076497   -1.224203509943   -0.769059732179
C      1.137235915136   -1.511208466122   -0.129298457040
C      2.007480275923   -0.538234621394    0.192114018925
C      1.631813051812    0.930118020715    0.007666593876
C      0.251930077491    1.221272321269    0.762205588418
C     -0.962264094050    0.516344412804    0.153190852241
C     -1.566736710964   -0.658261372660    0.748335525039
O     -2.729409054329   -1.095667825295    0.486928139847
H      1.347652353992   -2.550522546626    0.121875713724
H     -0.804359715571   -2.087571464914   -0.941798658177
H      2.952398634101   -0.758645640540    0.679334982077
H      2.404590271695    1.593262066982    0.410147914650
H      1.494317870656    1.179275234203   -1.054036694890
H      0.365276194691    0.932070855513    1.813495804106
H      0.063184771410    2.300927832170    0.729569302267
H     -0.115383271043   -0.608752300452   -1.667439854961
H     -1.028522437215   -1.130023508497    1.586743478228
H     -2.457367610011    0.804593849494   -1.205704441550
N     -1.666597927167    1.239445502105   -0.758968499098
```

```
H     -1.222365951969    1.952630060854   -1.328125567691
O     -1.106087043033    3.072264889972   -2.861300274813
H     -1.956956665045    3.139045689509   -3.326537288106
H     -0.686829395614    2.281272717804   -3.238879549718
O     -4.115640857847   -0.517510576359    5.998528146639
H     -4.559043960761   -0.058937555629    5.269173872674
H     -3.448072689105    0.088244434633    6.338435114158
O     -2.608836331856    2.868973293815    1.661160475783
H     -3.540072707005    2.605292920836    1.591232709029
H     -2.202721750430    2.205109428713    2.239460358783
O     -3.283726517764    0.205875733164   -3.422369638163
H     -3.487485770332    1.089459419668   -3.772912198011
H     -3.834370535138    0.089845752492   -2.634606645854
O     -2.721439257832    4.215452410794   -0.705413508554
H     -1.985465389970    4.018534870838   -1.296930285107
H     -2.519948435836    3.811994321941    0.157701645018
O     -1.280363082274    0.223541267582    6.095160586776
H     -1.384597155012   -0.665549429850    5.708752722834
H     -0.588483039558    0.161977896802    6.753500173819
O     -4.564537964178    0.095896799265   -0.850626644534
H     -5.291834290202   -0.528444078755   -0.876210868134
H     -3.830002087025   -0.375198726766   -0.331679555520
O     -5.210917282766    1.919260184470    1.149617575225
H     -5.612938554355    2.639296869916    0.655955913815
H     -4.996876144405    1.227119824639    0.496132319649
O     -2.048296365950    0.785983372053    3.409569006095
H     -3.033216487979    0.752896065489    3.478230690113
H     -1.726331971590    0.990896256164    4.296328463564
O     -4.700583429723    0.481267612270    3.485101789862
H     -4.714348191566   -0.430917025588    3.100266815591
H     -5.077928281232    1.059567279051    2.809691423466
O     -2.284259930567   -1.831916289431    4.464637651469
H     -3.040559424122   -1.578516872278    5.033084575930
H     -2.141860091169   -1.079806886917    3.866621667198
O     -3.659990883117    2.877948796691   -4.009380061617
H     -4.061023789225    3.464323444275   -4.650310564636
H     -4.181238765882    2.976665817456   -3.166581591405
O     -0.593437027296    0.465870207585   -3.629657022315
H     -0.337815665253    0.168529310559   -4.503217540353
H     -1.550326024450    0.266855063987   -3.544699346869
O     -4.801974186072    2.901743701147   -1.653631972219
H     -4.082917999065    3.446416997783   -1.239360865736
H     -4.645140221477    1.987471248723   -1.387029768404
O     -4.359528994673   -1.951794435964    2.463050901084
H     -3.781530159836   -1.751862952956    1.700203789062
H     -3.776916824878   -2.346120464189    3.126403639602

Cope TS Water15-3
65
ANGSTROM
C     -0.222538932622   -1.280112516736   -0.756267625367
C      1.095883921660   -1.467827472805   -0.110178295178
C      1.927296337011   -0.445489374806    0.152963904498
C      1.491671668788    0.999614713634   -0.086270882756
C      0.100129528377    1.246851175031    0.660124173681
C     -1.075303806904    0.477942165977    0.049311771809
```

14503	C	-1.671827917289	-0.666979355818	0.703015008239
14504	O	-2.844602795006	-1.095720840877	0.456805529101
14505	H	1.351478630302	-2.481232613466	0.193620334479
14506	H	-0.817068939370	-2.183955568138	-0.871224029590
14507	H	2.884355169517	-0.604090731236	0.640527057053
14508	H	2.234147319446	1.708160624276	0.295796082749
14509	H	1.347981956714	1.211504159284	-1.155367982983
14510	H	0.232169484853	0.963702618427	1.711204001061
14511	H	-0.131166355965	2.318109088510	0.619715294924
14512	H	-0.206678152660	-0.733052309496	-1.698801980110
14513	H	-1.135906255347	-1.102119773990	1.558140137690
14514	H	-2.488846123188	0.614909176922	-1.430558935643
14515	N	-1.771851300010	1.126458985680	-0.928697691627
14516	H	-1.327310183254	1.850525429390	-1.477664766085
14517	O	-3.870061956824	3.374735761747	-0.817437861287
14518	H	-4.200073565577	2.493956190121	-0.549400525762
14519	H	-3.087450844717	3.218158296274	-1.362549411643
14520	O	-1.021167622161	4.256319796868	2.020937335527
14521	H	-0.838853531034	4.998816393821	2.599416864181
14522	H	-2.023089979359	4.219681605304	1.930364560111
14523	O	-3.460733074500	-3.596095800393	0.802069600090
14524	H	-4.417043927751	-3.643291109931	0.846096942252
14525	H	-3.242724253458	-2.625536777088	0.719659851978
14526	O	-1.385334173283	5.437468946649	-0.459207117612
14527	H	-2.338986794851	5.526283502738	-0.367909227269
14528	H	-1.080657030062	5.008473769951	0.361058295895
14529	O	-3.587043040415	4.143724718247	1.704246330091
14530	H	-3.744554866717	3.941073957046	0.750503929825
14531	H	-3.888144485023	3.354299244426	2.178453645779
14532	O	-4.596400981787	1.057099899220	0.441737623223
14533	H	-4.058992562920	0.254913227224	0.334183739671
14534	H	-4.528671512884	1.282681530044	1.377051272534
14535	O	-0.206729097621	-0.195974368056	4.082696503113
14536	H	0.090565316048	-0.282925343615	4.989979229601
14537	H	0.030328848448	-1.046601660490	3.635185518584
14538	O	-3.236466689407	-1.170482762207	-2.272572838499
14539	H	-2.894516494207	-2.044535741075	-2.493019395394
14540	H	-3.347083802347	-1.199197074800	-1.308773895892
14541	O	-2.958939021229	-0.885970244391	3.931413919850
14542	H	-2.049154220305	-0.563865900411	4.029127220486
14543	H	-3.435736335739	-0.197418660514	3.451894408790
14544	O	-3.952757910749	1.728355976512	3.158677473624
14545	H	-3.027094630525	1.929397574029	3.462450255297
14546	H	-4.519741358095	1.862052582812	3.919737544822
14547	O	-2.220126622561	-3.461556111988	3.279389947311
14548	H	-2.638247819310	-2.632156888938	3.574598072332
14549	H	-2.621545273891	-3.666438821578	2.417232877010
14550	O	-2.328450676529	-3.860844512254	-1.718881460176
14551	H	-2.707849207398	-3.875238438203	-0.820362711745
14552	H	-2.433407477981	-4.744475358847	-2.072105689561
14553	O	-1.482651143498	2.270579237398	3.875993971529
14554	H	-0.933018449256	1.474355460507	3.873989203316
14555	H	-1.129908173637	2.872348124651	3.195956076311
14556	O	-1.351587068862	3.547540637696	-2.349081251633
14557	H	-1.243329747376	4.287702314683	-1.710131496206
14558	H	-1.490900566068	3.947581784364	-3.207673514728

```
O       0.237847535307   -2.581401522466    3.025465149761
H      -0.676047206369   -2.977442371564    3.087377186793
H       0.826236071254   -3.214178409744    3.438034873881

Cope TS Water15-4
65
ANGSTROM
C       0.465495653217   -1.125069314081   -0.477050802288
C       1.592517688658   -1.243182209309    0.472119673007
C       2.385211305409   -0.204031409443    0.789286957054
C       2.087387960141    1.197903973669    0.262634061765
C       0.577626229784    1.586509895309    0.624574476321
C      -0.472877943090    0.753670039925   -0.110284133512
C      -1.233761746582   -0.290085119228    0.546250521063
O      -2.330005033421   -0.759637033124    0.118208898980
H       1.728829895258   -2.209622948917    0.957306773714
H      -0.137347589635   -2.021439939875   -0.606107013549
H       3.194220270367   -0.307939021423    1.505833228666
H       2.761847620254    1.939185762993    0.703684936950
H       2.200513103749    1.251320283815   -0.829901959464
H       0.447146266168    1.485589819104    1.707758781870
H       0.416873047143    2.640465337614    0.367927889860
H       0.709572319716   -0.704850778449   -1.451579692954
H      -0.906472168610   -0.588536171745    1.557210832313
H      -1.487789598628    0.646770280439   -1.888128851068
N      -0.925317925039    1.249444189983   -1.294853665604
H      -0.403557267505    1.949789520543   -1.797711050716
O      -5.484191020714    2.570259542780    0.578275954537
H      -5.439351915173    1.841007100879   -0.054883542386
H      -4.825345835769    3.203321783987    0.265922499838
O      -2.021041358467    1.504758397180    2.996181689734
H      -3.002026066439    1.364252665161    2.868794368696
H      -1.905710776946    1.619650369770    3.940642978186
O      -3.998500648819    0.966804208406   -1.232343568435
H      -3.418568834911    0.332856723280   -0.766897676696
H      -3.622632608761    1.848191165078   -1.013292170164
O      -1.029926589297   -1.808875430036   -5.521153363797
H      -1.744001536507   -1.554725181029   -6.149157589104
H      -1.046815132806   -2.766261147493   -5.485397268870
O      -3.924995837376   -2.835422522496   -0.810859108002
H      -4.568497464292   -2.690098330684   -0.108807816924
H      -3.186211643029   -2.244273287973   -0.594832192995
O      -1.630152711111    3.754017949064    1.595692957804
H      -2.095192682050    4.412415148448    2.114687906608
H      -1.721007363169    2.903816788607    2.083301404184
O      -4.779173705257    0.008433375450   -5.383229352879
H      -4.302168941172    0.772972863934   -5.017365195045
H      -4.890691161846   -0.598005697614   -4.636454446151
O      -4.388676019338   -1.412289040789    1.729594061387
H      -3.611570557522   -1.146876021630    1.190276582057
H      -4.050059898116   -1.943282694003    2.451673038461
O      -3.188612035313    1.701293186253   -3.842011120260
H      -3.717542373466    1.622209125267   -3.031313972380
H      -2.587086686052    0.918330663680   -3.795740426787
O      -3.098685431271   -1.159408110583   -7.088753921222
H      -3.780554186238   -0.772172832353   -6.487157923055
```

```
H     -3.008555114046   -0.543105389420   -7.816090402269
O     -4.505881763304   -1.236255449961   -2.896700359986
H     -4.426452862462   -1.983287250989   -2.270485437610
H     -4.619500893174   -0.451758006221   -2.339926471199
O     -1.543247126312    3.631087290849   -2.973631332122
H     -1.645451123957    4.431249864927   -3.489609733621
H     -2.117500489772    2.953242482901   -3.401581420640
O     -1.907626390249   -0.549042199926   -3.321315171075
H     -2.779355971569   -0.978746411730   -3.198961564064
H     -1.489584502498   -0.993699228926   -4.086979844215
O     -4.595782442168    1.159425027624    2.724294843738
H     -4.719941838707    0.252375063925    2.393558752486
H     -4.986473706163    1.742260887928    2.041455762079
O     -3.062666901758    3.431577871497   -0.677871870202
H     -2.482027352381    3.600784705090   -1.441776537355
H     -2.492275407606    3.530715221159    0.110264389720

Cope TS Water15-5
65
ANGSTROM
C     -0.155729798997   -1.200107741252   -0.299589661850
C      1.231745031438   -1.251969072520    0.199405200829
C      2.067224218356   -0.203198491967    0.116723328406
C      1.568858023377    1.143535348475   -0.398664570237
C      0.283702714337    1.586618056910    0.450050045080
C     -0.967447801941    0.728902835519    0.215051718004
C     -1.405681558125   -0.283143196067    1.160634479477
O     -2.549111369869   -0.827718188230    1.177891088470
H      1.544302979702   -2.166760462873    0.694187070114
H     -0.743244945230   -2.100100113846   -0.140395260377
H      3.080742878853   -0.250742921881    0.500950587411
H      2.338285399965    1.918018823073   -0.308703581142
H      1.275729551344    1.081927418822   -1.456170562195
H      0.560457826113    1.572901200478    1.511512052075
H      0.036582002517    2.622234061413    0.182588883467
H     -0.272369358610   -0.839187775299   -1.319170747453
H     -0.737446893160   -0.470439905571    2.022932344764
H     -2.688347195688    0.678927135565   -0.941605178392
N     -1.802349144549    1.163876062309   -0.764275172853
H     -1.436156729980    1.679635907004   -1.547345896173
O      2.766949774340   -1.953388132379   -2.884355339714
H      2.851147060430   -2.652660561210   -2.197436901521
H      3.555274349920   -2.024422337783   -3.423671725182
O     -2.174997275399   -4.018326759391   -4.397547246862
H     -1.271460527123   -3.690157511904   -4.492178533826
H     -2.118764588549   -4.855859498307   -3.907544370998
O      1.023415400328   -2.928703956230    3.292571234974
H      0.162664628965   -3.428444344049    3.322311464119
H      1.325039937630   -2.878134101200    4.199946393591
O     -0.171221705344   -5.754428105231    1.134966918377
H      0.728629113749   -5.385858326336    1.133129103740
H     -0.672589799539   -5.247892343819    0.481464247281
O      0.273403937617   -4.494020266911   -2.046389608049
H     -0.270123206040   -4.095704781754   -1.354802408014
H      0.299466654667   -3.823548457763   -2.775000703274
O      2.860591064982   -4.073000327477   -1.229420271235
```

```
H      2.006654327106   -4.417134555533   -1.547128711449
H      2.794531505833   -4.095393186759   -0.264890022675
O     -1.379517883614   -0.548687518657   -3.298792181071
H     -2.035481341072   -1.167807919821   -2.896940930939
H     -1.797927621794   -0.205449089131   -4.089346854934
O     -4.155034050623   -0.350004112494   -0.962197698915
H     -3.967197745485   -1.104751806226   -1.536347001619
H     -3.792339905311   -0.588514866017   -0.087077719750
O     -3.151577816911   -3.267762082991    1.760466006689
H     -2.890520791380   -2.310054673129    1.618303347590
H     -4.092294810392   -3.255190551882    1.940357965891
O     -1.134759821394   -4.430125966164    3.293744039132
H     -0.832785540649   -5.081799693095    2.626432945210
H     -1.898161226579   -3.990272133722    2.887978072717
O      2.265489798932   -4.469276961899    1.510985713578
H      1.863773413698   -3.842256946819    2.161344731389
H      2.880152518529   -5.017268315664    2.000224058061
O     -2.991039086400   -2.464968908791   -2.390292661448
H     -2.607108336793   -2.986747791410   -1.665025629836
H     -2.873522706077   -3.018891825103   -3.196001295412
O     -1.987359474090   -4.189524361581   -0.450283860443
H     -2.407185803418   -4.960566577552   -0.850086503976
H     -2.487625950723   -3.945832125985    0.362017186807
O      0.339648052937   -2.598843611281   -3.888084298913
H      1.248147834353   -2.313875516500   -3.656818091639
H     -0.220620029469   -1.821391076433   -3.691765534821
O     -1.772783192368   -6.122750582344   -2.609069582927
H     -1.572515834111   -7.040953698579   -2.789268406243
H     -0.916400212152   -5.678944904717   -2.412843776907

Cope TS Water15-6
65
ANGSTROM
C     -0.155797357082   -1.165205588293   -0.814837560417
C      1.106172449717   -1.478800927107   -0.105300876163
C      1.943740582761   -0.501851171969    0.279173058391
C      1.561530540427    0.961808683508    0.062956501236
C      0.131347564232    1.254684832249    0.713248237358
C     -1.034123264677    0.462528986042    0.098269979586
C     -1.598337956814   -0.703023814660    0.740145975308
O     -2.758187459220   -1.165915419973    0.498985391341
H      1.296817943395   -2.525120964330    0.125154863938
H     -0.786927969779   -2.016229785855   -1.058826777910
H      2.865368415593   -0.711941726504    0.813904545030
H      2.297294112377    1.639870812720    0.508141334291
H      1.485894118869    1.192231073237   -1.008101204151
H      0.195690958500    1.050300004569    1.790358994408
H     -0.088841210786    2.321275997345    0.585870228171
H     -0.035235868264   -0.517180220270   -1.683373482772
H     -1.027773927751   -1.167212319053    1.563246550670
H     -2.553990534427    0.648988081914   -1.255790188807
N     -1.802783221320    1.148540668251   -0.804773635555
H     -1.372798456910    1.852369002905   -1.397733979704
O     -4.356675483802   -1.473815152250   -1.728139733601
H     -3.784741370039   -1.353608470581   -0.950812016935
H     -3.780843080893   -1.864567562529   -2.409679156386
```

```
O     -4.798398353018    3.352359317687   -4.324129347722
H     -5.031768333865    2.417545763461   -4.251764145905
H     -4.795632254763    3.676472648594   -3.415851579835
O     -4.611086706387    0.852107535890   -3.075742831308
H     -4.727295365833    0.023182993641   -2.571946827124
H     -4.539693196984    1.580163684923   -2.418542971215
O     -3.443806096446   -3.714411822682    0.472403130110
H     -4.370839784753   -3.707189872144    0.227956143108
H     -3.181321916685   -2.765444765121    0.569883436505
O     -2.471851585796   -2.433139024652   -3.503737732632
H     -2.747412688989   -2.928062094153   -4.276295392896
H     -2.111903747298   -3.100847740232   -2.871999233564
O      0.318039990152   -4.830196395421   -0.057820377239
H      0.701738142981   -5.695573159423   -0.205052117034
H     -0.271613801282   -4.922199562435    0.718909635265
O     -1.702887783587    3.634036162454   -2.238791462611
H     -0.891208378531    4.143443729449   -2.275415362782
H     -1.903038720716    3.412032804221   -3.172076558630
O     -1.522031910046   -5.016426862744    1.935331598842
H     -1.446030357355   -4.486701446399    2.730361135676
H     -2.292949832477   -4.673605139557    1.449672764766
O     -4.282974270022    2.937573805853   -1.413759578472
H     -3.364471634297    3.238587449460   -1.487782614760
H     -4.348281843590    2.430384163705   -0.588122779035
O     -4.692016479925    0.876015319832    0.411619688550
H     -4.008150264347    0.198716340763    0.541121382766
H     -5.444336487367    0.419894004957    0.028072590417
O     -1.794624963873   -4.251808734752   -1.703094804550
H     -2.435561180123   -4.163883197316   -0.976330667930
H     -0.939495330365   -4.425500198031   -1.267329133401
O     -2.187937498500    3.004835483422   -4.865868925319
H     -3.142341250489    3.233696964244   -4.834617770866
H     -2.148072603136    2.037473343891   -4.823019822278
O      0.181904257712    1.371497780954   -2.969567197017
H      0.394439781339    2.204409689668   -3.414163735052
H     -0.595925770014    1.016470806831   -3.429975434154
O     -2.186366292795    0.343840155398   -4.062879713386
H     -3.094583126890    0.546549909303   -3.735181213295
H     -2.106003964935   -0.615963394977   -3.988501173031
O      0.389804636534    3.814916808666   -4.405134858625
H     -0.511163997368    3.664399632718   -4.741407268826
H      0.957177493112    3.900293682143   -5.171452691349

Cope TS Water15-7
65
ANGSTROM
C     -0.166763577795   -1.208238954475   -0.745938754868
C      1.122718250547   -1.498218799504   -0.088748230591
C      1.988102132774   -0.523078503252    0.244070117342
C      1.616063376585    0.942021914435    0.043168609702
C      0.233094822333    1.252155739049    0.794076085941
C     -0.982776058461    0.551949526454    0.196745704780
C     -1.561232554050   -0.651662796115    0.756273217553
O     -2.716884056741   -1.091631503661    0.470437104138
H      1.325839047919   -2.536405453085    0.172247437951
H     -0.797511820933   -2.068961817921   -0.956602646521
```

```
H      2.922169959173   -0.741164011361    0.752902673863
H      2.388097664734    1.609420308769    0.439029773443
H      1.478257940292    1.179872575044   -1.020827297587
H      0.346970140310    0.974271642490    1.848057212723
H      0.063581202778    2.334071521554    0.742391384988
H     -0.105039192392   -0.560817740078   -1.619597201794
H     -1.033555535715   -1.117749693273    1.605756847633
H     -2.473597972936    0.824050490952   -1.184201603310
N     -1.698339369620    1.269851488901   -0.715209224779
H     -1.243071085382    1.994288554034   -1.248476409685
O     -2.918167829012   -0.294616045544   -3.326911559552
H     -2.832867740042   -0.711041590064   -4.210135717132
H     -3.423129859490   -0.935818598131   -2.773762653625
O     -4.396245881527    2.964477134211   -1.097385360063
H     -4.280930802506    2.627164155960   -2.037997855505
H     -5.230081913136    3.437130416628   -1.091238148543
O      0.119184629590   -0.222407481396   -5.728368268481
H      0.816335626591   -0.864154461867   -5.588534498118
H      0.005930439680    0.254432701712   -4.872708813192
O     -4.336349175882   -2.429838799290    2.170312024952
H     -3.658639622840   -2.014262520152    1.597170426837
H     -4.900447219162   -2.937909450606    1.585082950883
O     -2.399807914950   -1.191632701924   -5.809309167304
H     -1.466485239963   -0.892176195839   -5.873801787913
H     -2.863730393149   -0.785026890570   -6.542148432585
O     -0.771103319113    4.392197767606    0.544009305016
H     -1.597190935572    4.018395962557    0.953652375779
H     -0.607192106228    5.222069661899    0.992756228529
O     -3.039620810806    3.404026805111    1.417100154975
H     -2.917333578437    2.574039652189    1.927975356936
H     -3.535493784655    3.160975164295    0.625835211557
O     -2.200195730767    4.645649050601   -1.725284068999
H     -2.989354635848    4.171104308758   -1.418715865841
H     -1.568969778450    4.609322376912   -0.977185862249
O     -0.495373850636    0.950313853366   -3.396099335514
H     -1.337869793064    0.460381221821   -3.319064296452
H     -0.755308950778    1.852687866535   -3.648722651699
O     -5.187864215082    0.111451497688    2.715492654653
H     -4.990463426746   -0.844331452315    2.734990688223
H     -5.388256587271    0.302547097384    1.782517941958
O     -4.292404697352   -1.810180934086   -1.626392925121
H     -4.993764781807   -1.211098002270   -1.338287104521
H     -3.649556015150   -1.788118482641   -0.896125246601
O     -4.941568128741    0.434585667631   -0.007259014210
H     -4.088094801924    0.013572108393    0.216530115778
H     -4.737905792806    1.327868947563   -0.322686784472
O     -4.001955843265    2.137382202689   -3.521719844738
H     -3.714721901442    1.198782877447   -3.487224840191
H     -3.222903054875    2.626245601254   -3.835532267415
O     -1.578683489866    3.414962796618   -3.939325742847
H     -1.329123237204    4.048770244438   -4.611451356412
H     -1.747679530444    3.935692486895   -3.107189962122
O     -2.757469419820    1.134671793915    2.818675405783
H     -3.676192152675    0.748540730179    2.807137479353
H     -2.567778836672    1.304256510263    3.742054529801

```

```
Cope TS Water15-8
65
ANGSTROM
C     -0.287341934863   -1.233500608699   -0.824970161990
C      0.974707249950   -1.592778204809   -0.150259089411
C      1.872246068740   -0.664188650231    0.228663238258
C      1.582946732417    0.820243004481    0.036335922203
C      0.192087517667    1.214085605780    0.727962605773
C     -1.057822415569    0.561184045974    0.136878039202
C     -1.620111555845   -0.659272497856    0.697706806463
O     -2.774225630701   -1.130379785622    0.477085367139
H      1.125038172900   -2.644031585488    0.095305904043
H     -0.952698503087   -2.054458315952   -1.078886060019
H      2.781579084303   -0.935606949829    0.757202698944
H      2.369695720765    1.441266924699    0.476681660718
H      1.502835085529    1.072196253162   -1.029016917418
H      0.267020979946    0.969154495781    1.792579500340
H      0.081560872989    2.300574436599    0.636629667352
H     -0.184340258494   -0.554152048133   -1.669842004992
H     -1.059872585158   -1.092156127500    1.550540860555
H     -2.636264022450    0.966225910813   -1.131058044093
N     -1.758504144716    1.310733777030   -0.748505479462
H     -1.288991709619    2.008131873193   -1.302737249134
O     -4.225857145713    0.004082983510   -1.367154816643
H     -5.026494308859    0.291444805822   -0.909654599725
H     -3.733148184106   -0.535173370259   -0.689341576932
O     -0.160765808819    4.293828932449    2.785515159927
H      0.780723046537    4.100764076207    2.878699466758
H     -0.327874605725    4.358031767730    1.826850375457
O     -1.790081298186    1.095823771617   -4.313229385367
H     -2.276558430314    0.270200556981   -4.118509821573
H     -2.295772912273    1.815358774670   -3.901443212590
O     -3.309405355059   -1.047285082992   -3.656055467514
H     -4.050113054230   -1.295272754431   -4.209836370254
H     -3.690314414835   -0.714487119630   -2.817207782848
O     -4.783682373392   -0.830623792431    2.224653938660
H     -4.082517147752   -1.162392333650    1.629232217943
H     -4.325788951598   -0.180769611128    2.790981148727
O     -6.096676198700    0.692572206958    0.597514218349
H     -5.628309986240    0.053635339940    1.226034623045
H     -7.030576026759    0.614150964686    0.791879660456
O      0.537055962888    1.368621832796   -3.134663399708
H     -0.329820121646    1.197454094269   -3.595159444386
H      1.209741221995    1.247334721119   -3.805222631542
O     -3.087725940062    4.293136159576   -0.493846573037
H     -3.107908962751    3.893275231554   -1.381346512048
H     -3.554145561996    3.679808371004    0.100241881621
O     -0.141198770086    3.923090750923   -2.580477757894
H     -0.089456969678    4.094752439567   -1.624589021026
H      0.254942216748    3.041610146187   -2.741232810587
O     -2.678409216985    3.441885764640   -3.082422077880
H     -3.027391616060    4.057605110283   -3.727467685201
H     -1.719516746530    3.683893359620   -2.962801330869
O      2.030427978037    2.573543707733    3.428960240470
H      2.703343700871    2.228255694497    4.016046343700
H      1.206192627511    2.104136760531    3.634837236001
```

```
O      -4.530714039551    2.762659659506    1.257544729960
H      -5.095657379999    2.028613158418    0.924724923602
H      -5.111803271297    3.361936346833    1.728666622982
O      -3.351736974098    1.245186004310    3.280373748123
H      -2.394741608331    1.335341682833    3.400187756477
H      -3.595275598029    1.843780269930    2.556143188060
O      -0.706611903140    1.955135472318    3.913639121285
H      -0.945209779559    2.122794741214    4.827464972065
H      -0.670471927725    2.843055740107    3.476907776453
O      -0.565319772017    4.600688070240    0.077295761653
H      -0.430066843375    5.548551736269    0.019364224535
H      -1.544801316364    4.465910668903   -0.081010696901

Cope TS Water15-9
65
ANGSTROM
C      -0.260865556595   -1.284741130685   -0.739983122306
C       1.036554772558   -1.586086154594   -0.097804670724
C       1.939335301895   -0.630577069199    0.185458004100
C       1.603066307930    0.842264685667   -0.033558515812
C       0.236274785589    1.173312655297    0.729523674661
C      -1.006494105961    0.515375643272    0.114369292445
C      -1.638370988709   -0.631095790253    0.733602215484
O      -2.808509585163   -1.080643251373    0.497110615565
H       1.222589707266   -2.619185197662    0.184747570741
H      -0.914546069157   -2.138546382256   -0.901164032479
H       2.879598534673   -0.863266668521    0.676544772818
H       2.394368748311    1.495552515526    0.348522082515
H       1.460760293841    1.069038020756   -1.099827972848
H       0.351982875544    0.865624270749    1.775678773446
H       0.085239231205    2.259979691881    0.711670022973
H      -0.199031795127   -0.688087887001   -1.647797038179
H      -1.104702659005   -1.062061510960    1.596052559889
H      -2.503328531782    0.930902046659   -1.254906233547
N      -1.640851989284    1.261031897744   -0.838053950379
H      -1.102603454556    1.877040614105   -1.425855650025
O       2.444434212013   -1.577768547916   -3.062004981383
H       2.865771493584   -1.541289538400   -2.200848212354
H       1.825134531733   -2.338028890362   -3.029058938990
O       1.194520707696   -4.546325473095    1.608967009356
H       0.440503766008   -4.996605386586    2.077889266926
H       1.980447556590   -5.035309587451    1.854451972794
O      -3.497569519708   -3.223818371095    2.073456914862
H      -3.266882367636   -3.967490123192    1.503452446318
H      -3.308682803429   -2.417092795993    1.556247278940
O      -3.484888006393   -1.722267963689   -3.480706747150
H      -3.495607079710   -2.460990003060   -2.851433405125
H      -3.753994191188   -0.956033149632   -2.949446715884
O      -0.089387084076   -5.129612792137   -0.775824037166
H       0.086480864708   -4.507207230418   -1.502959960094
H       0.487046370501   -4.875951472261   -0.033394287639
O       0.553208225402   -3.542065080829   -2.970065696692
H      -0.098810008374   -2.919139622667   -3.409945645251
H       0.727842264660   -4.220311033806   -3.625068112860
O       0.661705691274    0.406867457033   -3.490564923050
H       0.857605037470    0.843852564138   -4.320674616411
```

```
H      1.372946522415   -0.262782150154   -3.368197945497
O      0.103854917794   -2.283331957626    3.055425320356
H      0.661734609540   -2.864308624340    2.524236397829
H     -0.530966311728   -2.878380790799    3.498307972867
O     -2.410751475495   -5.357733492553    0.401238284205
H     -1.538332208394   -5.271116976801   -0.077536989115
H     -2.771381519889   -6.206486411477    0.141137695643
O     -1.924501282413   -0.793281883206    4.496458218302
H     -2.541681860389   -0.708463086047    3.766109374189
H     -1.067530901648   -1.008573261737    4.107169079076
O     -3.299414637720   -3.238379254483   -1.223041764593
H     -3.100316851158   -4.038700862179   -0.719209380139
H     -3.205163672878   -2.496318876205   -0.607053885980
O     -0.892567195875   -5.676592645881    2.756825826528
H     -1.541195054684   -5.621270064579    2.035886728562
H     -1.238724601697   -5.114848579432    3.466517865804
O     -4.194306056774    0.068195700112   -1.466445851830
H     -5.128447829282    0.158057219979   -1.277751831139
H     -3.797209664995   -0.413055419533   -0.699279016423
O     -0.927219305347   -1.816233959733   -4.218790181201
H     -0.561184119552   -0.946126338409   -3.998821343082
H     -1.891268512777   -1.778978786091   -4.018764784598
O     -1.992216560259   -3.625909093977    4.243073290410
H     -2.638757144373   -3.474387068950    3.515163660607
H     -2.128757494934   -2.896685741678    4.859689483840

Cope TS Water15-10
65
ANGSTROM
C     -0.214608421839   -1.215646187002   -0.842689758909
C      1.060904464326   -1.555499239174   -0.187572662881
C      1.942923550380   -0.616873487391    0.205093783547
C      1.627082923372    0.866031226167    0.048087445987
C      0.240430987947    1.226213107935    0.771016076296
C     -0.996497130630    0.557003565905    0.179393885819
C     -1.557492998751   -0.683983003399    0.693578539757
O     -2.737991062040   -1.072057237105    0.452058159717
H      1.238313373276   -2.607402943300    0.037742741606
H     -0.853610980594   -2.054259076876   -1.105567538334
H      2.860140720755   -0.882607418643    0.722288344225
H      2.415188447339    1.489804237396    0.482528486656
H      1.525235328835    1.143620135139   -1.008711625017
H      0.331429692902    0.965828850133    1.832398052797
H      0.109943243549    2.312724395098    0.697540107719
H     -0.146168770382   -0.510917876732   -1.669836326210
H     -1.003216806620   -1.185325168134    1.510447377848
H     -2.543515630231    0.867492285380   -1.137929673445
N     -1.730727005808    1.286434947003   -0.692501051151
H     -1.307433993006    2.043266950541   -1.205153241137
O     -2.460920705369    2.095048526639   -3.846004986708
H     -3.007032780930    1.428641334004   -3.387952860987
H     -2.394953791552    1.770296456919   -4.775719811606
O     -3.982064115204    0.350283981632   -2.445649307467
H     -4.036899565414   -0.591156877483   -2.136611863072
H     -4.689391409801    0.847069641678   -2.007068289940
O      0.024541894133   -0.713374477382   -4.757365960379
```

```
H      0.927352577615   -0.954383357522   -4.966320032866
H      0.078887562693    0.055844597646   -4.143822340191
O      0.568268035642    3.840418625296   -2.060719861969
H     -0.176049613499    4.062011493326   -1.471639830138
H      0.619138556079    4.546573755671   -2.706290967774
O     -2.677051255617    3.212048639258    1.539668915589
H     -3.323205000122    2.521669235353    1.281808592995
H     -2.201374136795    2.820207675759    2.278590356397
O     -2.125868182561    0.709937115945   -6.084007060275
H     -1.318799036031    0.224621606160   -5.869788490119
H     -2.856898245536    0.091022093438   -5.871410038000
O     -1.619313865679    4.453154146975   -0.498010301274
H     -1.976112771011    3.926964261248    0.267434680392
H     -1.687536790617    5.370159084178   -0.227530830729
O     -4.289971537392    1.057892084479    1.085398998754
H     -4.821084760624    1.388557563050    0.343559988924
H     -3.854834646020    0.236918740451    0.771548295474
O     -2.399659901676    0.965874978456    3.244683535962
H     -2.469814218575    0.249728655010    3.876729288826
H     -3.175506776991    0.892714548797    2.670517903539
O     -5.463400891862    2.230427456831   -1.138102750697
H     -6.339224026176    2.611126040604   -1.199124112045
H     -4.836841601131    2.910798417835   -1.501753454164
O     -0.017127381438    1.363523125666   -3.111911567775
H      0.438309358999    2.177356279947   -2.845592039666
H     -0.916073811473    1.663486322607   -3.390227498325
O     -3.737563452046    3.917596825976   -2.223653717810
H     -3.009780240926    4.131823615618   -1.616269966201
H     -3.329342349895    3.407926610053   -2.944779810668
O     -3.887078901251   -0.977441016746   -4.939448438083
H     -3.289843727983   -1.629343280236   -4.541150702655
H     -4.195380510180   -0.443796441526   -4.193118037661
O     -1.806629450726   -2.485538506624   -3.748025737962
H     -1.654225974521   -3.288281712916   -4.248302726231
H     -1.146512627473   -1.833660427693   -4.059242603812
O     -3.848100620964   -2.161130851411   -1.715538163012
H     -3.113305118129   -2.435497441505   -2.278601738907
H     -3.473346069344   -1.940262613629   -0.839005140053

Cope TS Water15-11
65
ANGSTROM
C     -0.194911079187   -1.215936480038   -0.829710429056
C      1.143867766032   -1.534526909456   -0.281178727270
C      2.031184283254   -0.583966787481    0.054797447863
C      1.642070719641    0.892759003840    0.013480904726
C      0.296910453052    1.082450622864    0.856499506777
C     -0.942035977539    0.449963856230    0.213435255329
C     -1.563455909200   -0.737268706007    0.761787698877
O     -2.733262540878   -1.140841372208    0.488077925145
H      1.362871922380   -2.584930883155   -0.098047854783
H     -0.843534271804   -2.074994034878   -0.986482177024
H      2.999870978040   -0.834963272147    0.474307986238
H      2.427850079830    1.523640287724    0.441974974724
H      1.461737171654    1.237943313568   -1.015742862185
H      0.464146966815    0.652046258761    1.849776905158
```

```
H      0.101276995906    2.154057308892    0.981053590532
H     -0.206453957983   -0.582488384513   -1.716709929346
H     -1.016176694223   -1.267917793609    1.550880074318
H     -2.495192376276    0.914154071434   -1.083979447115
N     -1.645894456378    1.254596365170   -0.639996413150
H     -1.185889542439    2.007733197325   -1.122029074422
O     -5.225475144373   -1.803353301870    3.300924426748
H     -5.070111470864   -2.320963901629    2.468626820158
H     -6.026465649964   -2.148738139599    3.694555609569
O     -1.029839993620   -4.999429617358   -0.784902868992
H     -0.995643818934   -5.948123705224   -0.919854553586
H     -0.378580797241   -4.793447642926   -0.070856378716
O      0.535348535393   -1.483303501329    3.364948235445
H     -0.056264625815   -2.242003382858    3.209938468488
H      1.076830920684   -1.722109434422    4.118788209724
O     -6.087946350535   -0.982687791066   -0.417054139990
H     -5.481375219798   -0.745576055419   -1.137607540116
H     -5.937653081954   -0.307372383320    0.260555225185
O     -2.616791244515    1.872094617081    2.591172452376
H     -3.414923807536    1.410660140005    2.252236687427
H     -2.936497188378    2.648251220670    3.053008068195
O     -1.270510150993   -3.553396396391    2.958287621664
H     -1.916561504447   -4.071361217412    2.432993956773
H     -1.828244365688   -3.121295927021    3.652954518945
O     -2.933477637315   -4.940855017985    1.309768822145
H     -2.463560591455   -4.887446718093    0.464889827945
H     -3.726145233419   -4.393227309119    1.207531321094
O     -2.799725926460   -2.203049026059    4.649222852430
H     -3.665772647701   -2.050686838295    4.239971279583
H     -2.374432843100   -1.322410965287    4.700850453250
O      0.601662631788   -4.567459518491    1.285642694831
H      0.860364308041   -5.362490676267    1.754904043098
H      0.002454971083   -4.091427139882    1.894710326921
O     -4.675666774792    0.366744319313    1.682019833835
H     -4.013728726473   -0.139156588771    1.174324886347
H     -4.934187142786   -0.226318528918    2.408257521804
O     -2.108578146980   -1.480108897237   -3.738751247348
H     -2.527306227703   -1.658070874499   -4.581836608155
H     -1.603615040279   -2.286333212751   -3.514164108343
O     -4.727874490700   -2.875948423948    0.935062920345
H     -5.375359435395   -2.386950277452    0.380518161238
H     -3.894827727179   -2.379961562371    0.805097183639
O     -0.613131222454   -3.692510872226   -3.152152780738
H      0.320684420911   -3.485190052164   -3.086922920529
H     -0.851355273713   -4.151606471308   -2.323631322224
O     -1.470031891896    0.152971794575    4.427362031826
H     -1.836778698751    0.764707687925    3.764836305587
H     -0.693218859950   -0.260527353990    4.018092822196
O     -3.780148118472   -0.336694335619   -1.847590892935
H     -3.270647989144   -0.725768864867   -2.577138688721
H     -3.436583816923   -0.760531006667   -1.034030159455

Cope TS Water15-12
65
ANGSTROM
C     -0.256387052947   -1.246100407808   -0.808356707706
```

15119	C	1.047844532481	-1.571505858948	-0.203324089477
15120	C	1.942758660935	-0.617279347946	0.129960965252
15121	C	1.577939703389	0.860204027684	0.003468074856
15122	C	0.219800047632	1.135527825949	0.801787242207
15123	C	-1.021810418197	0.469610719511	0.203934734721
15124	C	-1.609813235046	-0.723945819299	0.797462724719
15125	O	-2.776388002351	-1.156147365237	0.576111326700
15126	H	1.243694432362	-2.619626920291	0.023740899637
15127	H	-0.908225457168	-2.090657218243	-1.015503234077
15128	H	2.882269744235	-0.866182545876	0.617516885912
15129	H	2.360933593069	1.506487904701	0.410582896924
15130	H	1.409217809764	1.154901697220	-1.042166660096
15131	H	0.358289702679	0.807431395919	1.836847248033
15132	H	0.053903361284	2.217530235395	0.803031838948
15133	H	-0.218216584211	-0.579367654523	-1.668690636331
15134	H	-1.031792877265	-1.202997405427	1.608078800974
15135	H	-2.566412484748	0.838893445094	-1.105294489582
15136	N	-1.736823929948	1.229249876318	-0.667763480489
15137	H	-1.260040089590	1.916478695931	-1.237506949227
15138	O	2.638295801259	3.987814362336	0.429529643581
15139	H	3.275010712574	4.690080031634	0.290995567123
15140	H	1.977905389961	4.078819056780	-0.308896155436
15141	O	-0.435827605905	0.267628246539	3.973662230493
15142	H	-0.153284541687	-0.170017581622	4.777742180539
15143	H	-0.289523887715	1.232984902781	4.126834591538
15144	O	-0.232167679849	2.893368252854	4.368900488797
15145	H	-1.156357346279	3.176310050948	4.297868164759
15146	H	0.233607243855	3.365696993539	3.660229662259
15147	O	0.854543738733	4.462305896913	2.362584103522
15148	H	1.550832584041	4.200982221779	1.713601437353
15149	H	1.121795822365	5.307606458173	2.725814080387
15150	O	0.778292850255	4.373897754918	-1.385923886020
15151	H	0.004565751096	4.513466294680	-0.804944923014
15152	H	0.518109231067	3.656550738427	-1.983415973934
15153	O	0.748108327619	0.074743619569	-3.986618396371
15154	H	1.625385576672	-0.200992047458	-3.661776145773
15155	H	0.319743950180	-0.713767651721	-4.322895749819
15156	O	3.118135929754	-0.655711419063	-2.820343394113
15157	H	3.802535699638	0.015397782481	-2.778483943178
15158	H	2.880792422193	-0.878417968039	-1.909490775818
15159	O	-3.475524641020	3.046724118885	1.260515727908
15160	H	-3.792584442887	2.104032764516	1.405155478983
15161	H	-4.185852957203	3.479784362055	0.785304328236
15162	O	-3.147949721076	0.737750152104	4.209294804323
15163	H	-3.072442065804	1.701821517331	4.273194580009
15164	H	-2.238316208534	0.421071595921	4.059223166876
15165	O	-3.156819823951	-3.241240110757	-1.152243989878
15166	H	-3.057013795700	-2.597820446552	-0.426975604024
15167	H	-3.826160632735	-3.860576426543	-0.859369960741
15168	O	-3.892808892615	-0.372684520702	-1.805703623809
15169	H	-3.770878016882	-1.172199116864	-2.324130836866
15170	H	-3.749767989580	-0.660982403327	-0.884669002702
15171	O	-2.902792845189	3.530521582418	3.840469145008
15172	H	-3.600249828813	4.092167644394	4.179075635375
15173	H	-3.077218417607	3.406690568870	2.879654382099
15174	O	-1.204645577375	4.467140950400	0.468939673488

```
H     -0.640666659161    4.489574653315    1.257704363650
H     -1.946478881804    3.878173978729    0.675906380821
O     -0.308610393380    2.356957298488   -2.950954737278
H     -0.871953905595    2.732134494569   -3.629315213272
H      0.088068036223    1.554030530358   -3.336936992447
O     -4.396835308495    0.693275736150    1.834417779149
H     -4.071212035127    0.607782100175    2.758519927011
H     -3.970474053446   -0.030208254659    1.348962660203

Cope TS Water15-13
65
ANGSTROM
C     -0.280700532166   -1.196210146771   -0.827707537391
C      1.035398574785   -1.559204606336   -0.265213208307
C      1.951707681025   -0.632035703114    0.068952864376
C      1.619592557932    0.854504878074   -0.020242818021
C      0.281643138637    1.156534245156    0.803752007380
C     -0.980584414750    0.518933896426    0.217342764745
C     -1.591994365076   -0.660679008675    0.800349899782
O     -2.772460404416   -1.060770503083    0.584443596298
H      1.220503944686   -2.616433680270   -0.073720451937
H     -0.961759520488   -2.020370832419   -1.024552500009
H      2.906326111027   -0.909481746322    0.506326087133
H      2.431220605605    1.468319721992    0.384289545795
H      1.446423951863    1.172103912912   -1.057982201559
H      0.426319442369    0.824364208057    1.837603417334
H      0.132333579486    2.242612446778    0.808877733637
H     -0.255760420754   -0.529166230628   -1.686781168053
H     -1.016403714594   -1.174413531670    1.590560111785
H     -2.473989227066    0.898712975695   -1.143451690025
N     -1.692076009608    1.304451578833   -0.636232553347
H     -1.205076564446    2.022206233648   -1.157740317510
O      0.347578845530    0.388797836776   -4.125366181872
H     -0.481227336169   -0.145304572010   -4.102117963287
H      1.060332478838   -0.216040015611   -3.915047436063
O     -3.812641605684   -0.581362007945    4.204664651563
H     -4.512347014220    0.044812583301    3.951625426510
H     -3.942915610440   -1.355850081409    3.634672771568
O     -0.959058454198    4.267058751954    0.922660919836
H     -0.836769972395    5.164992795900    1.232160909883
H     -1.489258950785    3.808593207908    1.618727183674
O     -1.553394392712    0.662169106350    3.598543364030
H     -2.376522458344    0.155273357544    3.813965315065
H     -1.132487705410    0.834102192299    4.442196507290
O     -6.466877034959   -0.939819572944    1.624277060573
H     -6.440982994866   -0.263933948846    2.313702018583
H     -5.804494529805   -1.603291726553    1.886153522065
O     -3.048148583761    3.036367555909   -3.109703007047
H     -3.214169891868    3.516233217015   -2.272896143736
H     -2.082250189059    2.950426414914   -3.148404785346
O     -4.855200626417    2.446217503715    1.038900225881
H     -5.600243126851    2.955628595167    0.714371593421
H     -4.844864093149    1.591853576760    0.493844556202
O     -5.708610189040    1.240340763120    3.312018386137
H     -5.346139154059    1.714549409898    2.532097930901
H     -5.902070249372    1.904126751753    3.974778018805
```

```
O     -0.277909338420    2.666613577704   -2.829541589401
H      0.228192992185    3.386087848558   -3.207670660531
H     -0.039554249788    1.859501467471   -3.331884821170
O     -3.635859727257    0.498983059502   -2.660279306431
H     -4.368107219887    0.424118255042   -2.034990054418
H     -3.544753101679    1.451256184959   -2.909744477450
O     -1.884614772290   -1.064801394174   -3.930434893278
H     -2.322169408099   -1.232808820287   -4.766297282060
H     -2.525127564743   -0.541622270065   -3.402041831352
O     -4.941257840233    0.223460273504   -0.234205774507
H     -5.632886169320   -0.241645847809    0.289809782172
H     -4.103784056848   -0.246914838827   -0.001649007724
O     -4.245768948692   -2.468171210722    2.231899577134
H     -3.654569203635   -1.985034116863    1.594728604410
H     -4.126394706777   -3.402030509259    2.059509010295
O     -2.544748209220    3.000051996605    2.650601481505
H     -3.307938705796    2.766606771689    2.107869458288
H     -2.150244093874    2.153981213155    2.946432311153
O     -3.220097375075    4.118764739886   -0.635566130171
H     -2.329575304836    4.205045590954   -0.248896751541
H     -3.686942816727    3.504262695947   -0.054483697568

Cope TS Water15-14
65
ANGSTROM
C     -0.217891696583   -1.290411312565   -0.789233821233
C      1.067234610862   -1.582378876036   -0.121293876857
C      1.946211062667   -0.615021604997    0.195310816960
C      1.603376262997    0.854431378653   -0.029163387906
C      0.211574037095    1.200528272743    0.677672143533
C     -1.007723235061    0.489883626206    0.081979836248
C     -1.624234972910   -0.659203725393    0.724929787838
O     -2.786144074874   -1.101449004255    0.495093576738
H      1.252875564441   -2.618118538011    0.162479325080
H     -0.871972607390   -2.144229422770   -0.952509132726
H      2.875925728529   -0.841633183990    0.708451861694
H      2.377797248585    1.509988080978    0.382121534303
H      1.500109989782    1.092571687765   -1.096890636914
H      0.297883926872    0.963727507256    1.744066963412
H      0.051660565000    2.280122686554    0.573962672187
H     -0.144823729221   -0.692055738144   -1.696857837236
H     -1.076550494435   -1.092508537708    1.580153806938
H     -2.531243974119    0.823540894931   -1.263512305951
N     -1.682264832539    1.190771077188   -0.864512025711
H     -1.228359479335    1.949210840195   -1.369706186417
O     -4.426600217911   -1.479141899672    2.639428379791
H     -3.801831131898   -1.523180490907    1.890636614855
H     -5.033687168030   -0.752719867620    2.407651005366
O     -6.222028690186    3.092979850502    3.132444699286
H     -6.212501229705    2.452924689111    2.403946138860
H     -5.886861593408    2.592951393669    3.891624525405
O     -4.141087301396    2.882221004830   -0.329179365060
H     -3.733985323833    2.514080950917    0.467048319941
H     -4.441641158698    2.120489598534   -0.835065135455
O     -4.480398648932    5.093541958758    3.256887345373
H     -5.133196061886    4.354248221902    3.142321302687
```

```
H     -4.909014440077    5.876149956458    2.910274532528
O     -5.520060618498    0.840491678564    1.611100304888
H     -4.639572433488    1.195806961111    1.821346071059
H     -5.478659440712    0.636052545742    0.663695836914
O     -1.165769770785    0.768505874777    3.643890512890
H     -1.024072735338    1.599487995028    4.127669298373
H     -1.745464888375    0.213082243347    4.193058117969
O     -3.169517681478   -0.736904894964    4.815238269148
H     -3.162919048863   -1.480727978339    5.417134317002
H     -3.635442707547   -1.055071683165    3.990135892344
O     -2.037849718173    4.359222301781    2.325892726831
H     -1.499176833051    4.148598352789    3.108279778231
H     -2.869361046313    4.753766703279    2.656661512259
O     -4.332093077402    1.730241237254    4.610944031896
H     -4.047892307264    0.853507699666    4.912225931970
H     -3.904919833653    1.840733793597    3.738170179974
O     -2.176033448729    4.802474523423   -0.457060893850
H     -1.969553195606    4.780882742884    0.487569319405
H     -2.962679549328    4.236647100721   -0.553110443597
O     -3.041530461025    1.913716208799    2.162492825972
H     -2.315308055868    1.366201089167    2.538808891915
H     -2.673451238944    2.829184842927    2.150789337058
O     -3.365868942612    4.028608170344    5.596415488959
H     -3.804550127096    3.166160800338    5.444166937367
H     -3.762022654539    4.616993717918    4.935763304395
O     -1.005029665348    3.376495904698    4.685005211183
H     -1.874876668243    3.623149095340    5.111115971999
H     -0.317729649976    3.716440907625    5.257255984707
O     -4.629934516702    0.106349010605   -0.925547475421
H     -5.123177261417   -0.570328441539   -1.389360940793
H     -3.934221353155   -0.376590963918   -0.395022859106
O     -0.438621059861    3.461117290455   -1.955219799414
H     -0.485976951716    3.838845743911   -2.833792923241
H     -1.007056895011    4.037197224829   -1.387114544356

Cope TS Water15-15
65
ANGSTROM
C     -0.156968491806   -1.194014157194   -0.830228486205
C      1.160249736322   -1.519647517644   -0.244290971241
C      2.054017009446   -0.569084774494    0.078941380859
C      1.690671526446    0.907512173153   -0.049328799348
C      0.333786533671    1.194448709080    0.744134320249
C     -0.909547799016    0.512554920357    0.162445183101
C     -1.522139058762   -0.649771660355    0.775754893155
O     -2.683192051496   -1.074450325462    0.514040235357
H      1.363466534845   -2.569252139022   -0.030836009239
H     -0.815935007252   -2.042407191845   -1.004425754833
H      3.010211025973   -0.816575058450    0.529679247547
H      2.480940219145    1.548165134272    0.355963999344
H      1.533712183885    1.194288056026   -1.099005985634
H      0.468993984774    0.892235761280    1.788324043237
H      0.155097708499    2.275827051201    0.727002449871
H     -0.135887906723   -0.561071018849   -1.716719505756
H     -0.968821758831   -1.129774395058    1.601342926104
H     -2.492656456805    0.912935624631   -1.088460290901
```

```
N     -1.629450810833    1.265519538025   -0.710114892531
H     -1.182714190196    2.000781662903   -1.235222993435
O     -4.983359537028    0.915056923489    2.200078771330
H     -5.569442884597    1.598021401433    2.530312861291
H     -5.499177306117    0.386996889231    1.561347479366
O     -3.754159522761   -3.279223731145    1.375352348866
H     -3.333404255527   -2.410474028661    1.110455562332
H     -3.126814048756   -3.965243953741    1.143227717071
O     -3.441471345647   -0.202798888808    4.263000240629
H     -3.991477552091    0.119940163031    3.531878113491
H     -3.530126840433   -1.172990454368    4.261232237902
O     -6.651926659283   -0.376585446991    0.480187239151
H     -7.592548459736   -0.498525222611    0.610527693812
H     -6.308816279827   -1.254138031867    0.144649368527
O     -3.846017598565   -1.167858311637   -1.899747763319
H     -4.529041929376   -0.454465699126   -1.952013356090
H     -3.437220243092   -1.085583404776   -1.010363854309
O     -1.098551873823    0.893707209988    3.671517469715
H     -0.851798751847    1.383826966521    4.457188865956
H     -1.923706269569    0.407417007802    3.918306027694
O     -5.713535758599   -2.633971682074   -0.457650267945
H     -5.118035071325   -2.301491687535   -1.154316994917
H     -5.129225970382   -2.985857556322    0.236339715290
O     -2.669874965139    4.210927682763   -0.115938498291
H     -2.590345898607    3.613148605189    0.651777492904
H     -1.863735858746    4.092529886066   -0.645549453520
O     -2.600913584360    2.431464822652    1.934744980508
H     -3.390117256647    1.872579422962    1.915356976855
H     -1.969036041175    1.964558662543    2.508852037314
O     -5.632401765075    0.777015841605   -1.831491488649
H     -5.241038246551    1.662348992800   -1.741542393604
H     -6.067739228489    0.564809237542   -0.990954017981
O     -2.062010191297   -0.021606651360   -3.525346321431
H     -2.713269445532   -0.508075466958   -2.964695605014
H     -2.146993874947   -0.389531815537   -4.405162286592
O     -3.950085921917   -2.897428306481    4.059329069377
H     -3.901283772221   -3.064131049149    3.096838811494
H     -4.840066809277   -3.126057638565    4.329995147687
O     -2.510794462192    2.696730216766   -3.614491033700
H     -2.339667911977    1.736007141630   -3.620427135490
H     -3.296079706692    2.826711350608   -3.051833386646
O     -4.455840133172    3.245369707510   -1.734203057428
H     -3.802820694770    3.598576573884   -1.059349683323
H     -5.006340117581    3.988952823432   -1.980418808657
O     -0.682500001512    3.707033914816   -2.017541082701
H     -0.183433378210    4.424279650170   -2.407602995751
H     -1.328750802690    3.404839890994   -2.711540540029

Cope TS Water15-16
65
ANGSTROM
C      0.184617079861   -0.918214149279   -1.007621957079
C      1.347959033006   -1.597201449560   -0.396156747903
C      2.208545818298   -0.962703232844    0.418675570606
C      1.949547509537    0.479465017664    0.851502447012
C      0.479048580056    0.580212663107    1.469797441058
```

15399	C	-0.648282304490	0.365408593940	0.453834082210
15400	C	-1.443603729231	-0.850947585618	0.420075253111
15401	O	-2.571553852693	-0.963221310477	-0.133093504833
15402	H	1.459600165315	-2.663320644786	-0.595364057995
15403	H	-0.465484248147	-1.558291659812	-1.600896339481
15404	H	3.048408298209	-1.480138877157	0.872368563424
15405	H	2.678010528069	0.807077930578	1.600740256451
15406	H	2.009508543768	1.172020592197	-0.000154345610
15407	H	0.400300847263	-0.151887904380	2.282640779352
15408	H	0.343953649587	1.577050023736	1.906909291350
15409	H	0.412997339929	-0.009454056329	-1.563026191167
15410	H	-1.082894085741	-1.701198925220	1.029796285933
15411	H	-1.896849504711	1.434754184646	-0.807244508879
15412	N	-1.121830054243	1.484277040300	-0.151167391615
15413	H	-0.567958148839	2.322260762035	-0.215604341379
15414	O	-6.097388033270	0.715494000641	-1.478675472490
15415	H	-5.649985418460	-0.002563109529	-1.936523206898
15416	H	-5.628437470052	0.808728030327	-0.624910680257
15417	O	-3.851166301934	-1.131279833993	-2.418948101049
15418	H	-4.199644648828	-2.018079506604	-2.520403824126
15419	H	-3.369385218410	-1.126104659316	-1.556176258542
15420	O	-3.188248323742	1.635182702436	-2.114442556978
15421	H	-3.954850577608	2.264856916664	-2.121249974079
15422	H	-3.555021285934	0.766870400152	-2.302988614696
15423	O	0.299268015369	0.992958283107	-3.710381350452
15424	H	-0.488792301970	0.440207536543	-3.891511334334
15425	H	0.902469877811	0.846324742863	-4.439400906898
15426	O	-3.169839118433	6.373242698867	-1.751602748004
15427	H	-3.339178231769	5.851113460639	-2.580747671137
15428	H	-3.214942954270	7.296061271463	-2.000077965047
15429	O	-5.086039699112	4.924094689313	-0.328104227310
15430	H	-4.506791345822	4.378472924348	0.232186366050
15431	H	-4.491887275256	5.573319085377	-0.739146382375
15432	O	-2.020134050026	-0.298895012245	-4.335720301911
15433	H	-2.522550103337	0.385352365842	-4.783853826834
15434	H	-2.613229380684	-0.663579943300	-3.654436524625
15435	O	-3.907141918376	0.799048053730	3.250693393532
15436	H	-4.702113967030	1.080574082700	3.705589405210
15437	H	-4.146244086831	0.761518832266	2.294222547633
15438	O	-3.137866725549	3.340153860951	0.723970141118
15439	H	-2.642572811437	3.162855985337	1.543617601084
15440	H	-2.469070015823	3.671607461693	0.085680762259
15441	O	-1.330659460730	4.419928071177	-0.969240296371
15442	H	-1.829239829762	5.238663335541	-1.111940880358
15443	H	-1.266696441864	4.010376466063	-1.854337862592
15444	O	-1.877257175063	2.564458098374	3.034442404429
15445	H	-2.527721035520	1.841948606804	3.174074395453
15446	H	-1.942061343217	3.135954635818	3.800647692265
15447	O	-1.396929464700	3.123600848077	-3.378538420624
15448	H	-2.045443505417	2.512502957850	-2.967113757691
15449	H	-0.620695554046	2.566505200958	-3.549276415634
15450	O	-5.288143479035	3.193514160679	-2.324481751200
15451	H	-5.343056076603	3.838064362717	-1.574356645936
15452	H	-5.886393880843	2.459227056444	-2.101843152175
15453	O	-4.364206567505	0.939440670106	0.617123962012
15454	H	-3.710308334933	0.279122643098	0.313006058422

```
H     -3.934715957696    1.820134029703    0.529528957303
O     -3.580910332341    4.812883870804   -3.842121912716
H     -4.277268055584    4.233680790976   -3.493933134628
H     -2.770749429360    4.281203981040   -3.865682844513

Ambimodal TS Water45-1
155
ANGSTROM
O     -3.574606472565   -4.532620184885    1.293351869636
H     -2.826735580453   -4.766800229450    0.730268223471
H     -4.048775391524   -3.821187085526    0.838748948772
O     -3.643593145464    3.338670888542   -4.024341424753
H     -3.102696176057    3.607360317934   -3.263295317447
H     -3.927442538100    2.431262816642   -3.833052019721
O     -2.813032692668   -1.822326120873    4.478566132382
H     -3.075784809653   -1.880146016555    5.397246784380
H     -2.656006470965   -2.769934953699    4.184351098595
O     -3.100489861432    3.788643465699    1.055374276329
H     -3.810094587288    3.666047163316    1.690639521236
H     -2.902999641515    2.901933497950    0.707422748122
O      5.344527992345   -3.350144077597   -2.085277354116
H      5.761245602313   -3.277337317789   -2.944367166633
H      4.383280274176   -3.243819302575   -2.233244335656
O      0.394017978661    3.633489066871   -4.915293556117
H      0.233703900211    4.383360583327   -4.331290090896
H     -0.456747185884    3.414407418044   -5.385463298262
O      2.699391422849    3.114917925463   -2.673702315207
H      3.234806346935    3.800627290924   -2.214423174299
H      2.962860494614    3.130447477546   -3.595915298027
O     -1.840978215174    2.772714515924   -5.952278549593
H     -1.786705112326    1.822218628053   -5.751736220023
H     -2.556676176213    3.109106730532   -5.371535738393
O      0.726935527772    0.609989248738    5.123632670520
H      1.681497395539    0.689651984738    5.266023377506
H      0.437724663747    1.486889155642    4.831439835253
O      4.198635632577   -2.007605901313    1.938748549006
H      3.861962248405   -1.155847088729    2.261879586814
H      4.674969708473   -1.795899357721    1.112937524163
O     -1.726545008106    0.303882540844   -4.640649809165
H     -2.565950256269    0.426166874380   -4.171683943252
H     -1.026765073643    0.722135474974   -4.084389804197
O      0.203423650486    1.493072566975   -3.246888604083
H      1.075927474136    1.060691532416   -3.285718666020
H      0.302834854706    2.301562553922   -3.787271926330
O      5.043130779196    2.578821669547   -0.094182749547
H      4.768881992722    1.998916233371   -0.821684445060
H      5.586303634091    2.013769830098    0.500849249748
O      1.543463297758    4.965002564523    0.276155409077
H      0.801293444099    5.112913824862    0.884272233814
H      1.137359023189    4.905299612892   -0.615797660872
O      2.581902412176    0.278965862711   -3.781519562458
H      2.685307697809    0.945142453358   -4.486347014555
H      2.299996845566   -0.557715355964   -4.225843056395
O     -2.192061266983    3.749695371508   -1.709973705747
H     -2.364210343961    2.904308351664   -1.254188323961
H     -2.423569684694    4.437414778999   -1.080703500572
```

```
O       2.383702467800    3.626535518740    4.131910616815
H       2.706733163338    3.544906342538    3.209186397586
H       1.424316876657    3.564418118067    4.071632732056
O      -4.473502075937   -2.331264169496   -0.160838132615
H      -3.822294252077   -2.563244262155   -0.845633172124
H      -4.799960431005   -1.452280302907   -0.420179928289
O      -0.680018078776    4.857933501369    1.941650661538
H      -0.503412453160    4.263301017209    2.682074122295
H      -1.553598485776    4.611402423708    1.597580492283
O       1.701282054501   -4.794760618643   -0.583385041681
H       2.041833741820   -4.507391440394    0.294320333736
H       2.184915879691   -5.588574718402   -0.816165739693
O       1.732679577609   -2.027072465530   -4.776860984382
H       0.767086638646   -2.105080190348   -4.661267434678
H       2.113802748748   -2.610551880344   -4.106890578723
O       2.674633455435   -2.923868879074   -2.318016112127
H       2.455182498827   -2.039654769867   -2.009714001697
H       2.215594323700   -3.557380882814   -1.734920470336
O       6.093688242713    0.750505804094    1.538269647234
H       6.135599009551   -0.020951949855    0.957507499819
H       5.268756267080    0.648668950013    2.047146051893
O      -2.183255018397    1.095788739684    3.303515365988
H      -1.563359174807    0.396737857266    3.001210233656
H      -2.708351450263    0.666949305267    3.985510483705
O      -1.027163438134   -4.516392353983   -0.022100731840
H      -0.746967436455   -4.093643389967    0.818437946905
H      -0.197437618068   -4.830301061358   -0.409145613397
O      -4.235774899942    0.656252860730   -3.460492719916
H      -4.497077255766    0.482413439807   -2.525376041330
H      -4.932912873972    0.302496555161   -4.013285007195
O       3.347619451442    1.454646070983    5.226144194177
H       3.027935259335    2.337488461766    4.890278861114
H       4.123860885544    1.628085926956    5.758041292154
O      -2.287369236519   -4.210064149971    3.639116676669
H      -2.878715449551   -4.420067692520    2.887882021740
H      -1.404407967095   -4.111965866490    3.244847321065
O       5.450309453619   -1.238344586321   -0.362034398156
H       5.574203347832   -2.043035637356   -0.897016907767
H       5.169516698383   -0.548767992581   -0.987380957044
O      -4.441767971794   -0.957735106348    2.353583293659
H      -4.599925116693   -1.630992391103    1.680260842703
H      -3.966699743609   -1.375702468668    3.086438420415
O      -0.497412746849    3.010463377183    4.136812174696
H      -1.147013638622    2.367888071514    3.768426032188
H      -0.945880876824    3.488934964352    4.836181771068
O      -6.010096768119    2.508448980007    0.050784144322
H      -5.635524018542    2.332374580438    0.935091990042
H      -5.565194007342    3.287966210137   -0.288090443363
O       2.595145997268   -4.026620945392    1.834258126258
H       3.009015603690   -4.670983546964    2.409320878622
H       3.195792580860   -3.221300093420    1.861868721193
O       4.716545043225    0.704485954905   -2.221911439124
H       5.367729886763    1.106888175692   -2.797856414447
H       3.904396668027    0.585283852223   -2.767660611801
O       4.024436958713    4.914532295022   -1.194948267131
H       4.567741937648    4.277241193589   -0.708479118040
```

```
H      3.306659229448    5.176644113506   -0.603019032247
O      2.999263814009    3.092884083059    1.582600629009
H      3.791891904740    2.985133779372    1.016126341082
H      2.437361595191    3.741612556930    1.108735982029
O      3.557579460634    0.577393522676    2.652952209989
H      3.209575855539    1.372198894153    2.215350864493
H      3.537629476656    0.774266695842    3.606254579568
O     -2.541866252743   -3.283543503032   -1.921661197000
H     -1.963833098048   -3.724283039444   -1.264808287483
H     -2.914753401580   -3.977573609690   -2.468043990359
O      0.454181495366    4.560581327126   -2.182593248908
H     -0.385510318837    4.089983305225   -2.071591820056
H      1.147455314074    3.903827258520   -2.400641988933
O     -4.862795545391    0.288859407211   -0.899771282307
H     -5.509838807149    0.977676286567   -0.641781970634
H     -4.015082210076    0.636055846404   -0.557131816016
O      2.658416959104    2.339441077501   -5.590344848273
H      1.846938206172    2.854887727587   -5.400147894752
H      2.622260079036    2.130213026899   -6.524104400315
O     -0.920905791662   -2.232391662621   -4.122570354375
H     -1.253913958432   -1.362606641021   -4.423712617921
H     -1.433965195222   -2.457690220561   -3.336824961777
O     -0.050101638198   -3.418585303855    2.242515450983
H      0.899635822188   -3.621890561544    2.189290985706
H     -0.114461090658   -2.488386412185    2.538718742480
O     -4.708374378095    1.759427539245    2.338446839118
H     -3.754587406028    1.691492829353    2.501472439082
H     -4.990785432872    0.832256456101    2.332225570184
O     -0.450353834595   -0.967267841877    3.338602857801
H     -1.195415872750   -1.328685244874    3.850238830270
H      0.118245895698   -0.499490761941    3.990812974749
C      0.190188251353    1.702402780536    0.691983593075
C      1.299100728363    1.500712818535   -0.071224254803
C      2.025897083659    0.252215222177   -0.180245657283
C      1.664099338175   -0.911963746155    0.425000767932
C     -0.269073701793   -1.339562790753   -0.960114816077
C     -1.232671649985   -0.615951287252   -0.297561616780
C     -1.517915455397    0.762907617430   -0.698442611950
O     -2.517535424259    1.427332725726   -0.282758615534
H      1.623206183740    2.281981487279   -0.753755016500
H     -0.300085853364    2.665349306757    0.713781490930
H      2.828238714036    0.243821990374   -0.911933376654
H      2.210243659567   -1.830584425580    0.261677421835
H      0.986070498075   -0.938912315397    1.265545659691
H      0.172788044472   -0.944626280686   -1.860188805176
H     -0.125775479685   -2.393221298389   -0.783910731156
H     -0.094206002366    1.026792602063    1.487099917887
H     -0.998055839371    1.131358754146   -1.588976377084
H     -2.715918651464   -0.605157471206    1.143406719624
N     -1.889912093205   -1.090862114146    0.825752900352
H     -1.881592083618   -2.077128353147    1.016108741884

Ambimodal TS Water45-2
155
ANGSTROM
O     -4.595441945089   -1.360428164080    1.905943093846
```

15623	H	-3.865801818794	-1.314605022504	2.558579547327
15624	H	-4.776541163510	-0.432460566412	1.673019288130
15625	O	3.453872867940	2.560992657250	-3.359066065223
15626	H	3.620349960192	2.928257208180	-2.476189537861
15627	H	3.904955725629	1.699852021355	-3.382901210213
15628	O	1.327699623318	-3.979566581862	-3.100798867545
15629	H	1.963793874501	-3.248895205444	-2.994352638826
15630	H	1.550351919449	-4.424697084143	-3.919128496949
15631	O	-2.968687797199	-0.493903665691	-5.226327489925
15632	H	-2.911095793180	-0.726368544361	-4.284367167760
15633	H	-2.045289424086	-0.550878831093	-5.553211918284
15634	O	4.799676221210	0.124675859153	-3.112908135371
15635	H	5.158837941320	0.498235202623	-2.257034225345
15636	H	5.556220484333	0.002853593191	-3.686735643868
15637	O	-4.370626818941	1.188161560100	0.977196273664
15638	H	-4.949394257278	1.796421335078	0.468144138170
15639	H	-3.632072992647	0.982645878678	0.377751340636
15640	O	1.831115835324	-4.994271870367	-0.516931053085
15641	H	2.500910555943	-4.332151541949	-0.293966409138
15642	H	1.560920252817	-4.790773468973	-1.425010416549
15643	O	-0.415365756947	2.210470295248	-5.938618322577
15644	H	-0.364902832435	2.928164941263	-6.570102387493
15645	H	-1.323088543480	2.236782573413	-5.556618441878
15646	O	-3.981768853981	4.922270151814	-1.583837613827
15647	H	-3.549326646106	4.869070344522	-2.479019335928
15648	H	-4.215519588633	5.842413983550	-1.461648948986
15649	O	0.965082872375	-0.618236682413	-3.696002284283
15650	H	1.006707709007	0.340324037881	-3.520490875703
15651	H	1.807672072509	-1.006091297124	-3.414112787076
15652	O	0.862565833828	2.098526986081	-3.537380965481
15653	H	0.546589260071	2.170015059047	-4.456156861430
15654	H	1.824710060773	2.326874649710	-3.540459170359
15655	O	-0.373686003460	5.059033222452	1.301561615317
15656	H	-0.675921220122	5.968310342838	1.331592041049
15657	H	-0.991369336898	4.570120146639	0.715588280257
15658	O	-1.410666891185	-3.456782617271	-3.076888925385
15659	H	-0.447450583443	-3.553610337734	-2.985393989178
15660	H	-1.577124504471	-3.492987971881	-4.041169956136
15661	O	-1.221319151518	3.804447372408	3.709822987054
15662	H	-0.944528614542	4.214942612134	2.880040795176
15663	H	-1.817686545873	3.074560638167	3.473218864337
15664	O	3.836566672494	-1.485635858300	3.056740874483
15665	H	3.554310441102	-0.726350110652	3.584209809979
15666	H	3.020034513319	-1.919576573942	2.765645983498
15667	O	0.846432080486	2.687399555114	5.066913392291
15668	H	0.433998671510	2.024958320026	5.655248318005
15669	H	0.083508797467	3.115204460353	4.626912401959
15670	O	-0.311160057959	4.232272001264	-2.441782144834
15671	H	-0.855442333266	3.941635838097	-1.684056809898
15672	H	0.091276700915	3.422092495793	-2.829669342423
15673	O	-4.097273711695	1.396704113182	-2.645068383314
15674	H	-3.380732174904	1.250920696864	-2.000537879676
15675	H	-4.314471189922	0.527445577598	-2.991807805504
15676	O	1.363065555623	-2.439276536517	2.132929484796
15677	H	0.974225767324	-2.084639414577	2.954011822033
15678	H	0.839998024248	-3.227184737285	1.896802079628

15679	O	5.632240256798	1.294464666100	-0.954312979915
15680	H	5.596750351401	0.794013753202	-0.114218256273
15681	H	5.045224700391	2.049179259349	-0.822750719265
15682	O	5.249198249834	0.251307649149	1.475087647448
15683	H	4.840178292913	1.061600584189	1.810218156642
15684	H	4.870984385894	-0.468048033776	2.009827879226
15685	O	-2.356487583073	-4.168633625358	-0.487829335520
15686	H	-2.929406871058	-3.383128646681	-0.488578284589
15687	H	-1.987768461342	-4.196991052102	-1.381820786051
15688	O	-0.523465124682	0.614901290237	6.169085051072
15689	H	-0.231800899197	-0.080473931932	5.558416532758
15690	H	-1.486893962278	0.677500913121	6.074720024247
15691	O	-0.189244740231	-4.510534040090	1.231348759608
15692	H	0.512590861673	-4.904626510132	0.675772628005
15693	H	-0.967749181673	-4.437774364946	0.647931928496
15694	O	-4.340161324037	-2.219163222557	-0.585025416503
15695	H	-5.189561146881	-2.635881035559	-0.734548331134
15696	H	-4.360002204235	-1.863597474226	0.338462710619
15697	O	-1.756279199247	-3.117940970177	-5.747207217733
15698	H	-1.079242004848	-2.475609543076	-6.001313522058
15699	H	-2.591947545851	-2.646996038607	-5.838303902425
15700	O	2.127147946262	4.392480623750	3.179436253110
15701	H	1.832312130052	3.940696304259	3.982639711911
15702	H	1.330979733758	4.613693603153	2.687662572510
15703	O	-2.499632736272	-1.029902248403	3.618226582772
15704	H	-2.042185044501	-1.900987240334	3.611752370493
15705	H	-2.783125407646	-0.876915679453	4.525379773342
15706	O	-2.913205276006	2.178655906916	-4.964615909652
15707	H	-3.058156997542	1.271001140301	-5.308719507826
15708	H	-3.303172391437	2.117942008119	-4.072475163730
15709	O	-1.957784678764	3.644547894083	-0.359152212430
15710	H	-2.101678691337	2.686396460401	-0.388772217529
15711	H	-2.770454020140	4.071035222973	-0.700609975170
15712	O	-1.623908323595	-3.528089320031	3.402022929237
15713	H	-2.483875269356	-3.891211733306	3.110597106462
15714	H	-0.976321626293	-3.988858786045	2.851658072028
15715	O	3.660787182164	2.533444343431	1.942136989591
15716	H	3.233880793625	3.275081215935	2.415826516015
15717	H	3.660884764475	2.791497502186	1.009826168824
15718	O	3.871245858867	3.604192433290	-0.737372313774
15719	H	4.552451977615	4.278185506342	-0.689480267621
15720	H	3.013971505071	4.104110125061	-0.722931704869
15721	O	-0.374409850792	-0.637271794504	-5.987523673437
15722	H	-0.257201865603	0.288592832434	-6.239183974078
15723	H	0.136912456482	-0.731842876325	-5.153209690565
15724	O	0.864512152533	-1.139980143774	4.481817201194
15725	H	1.320006710931	-1.626066079934	5.171289800894
15726	H	1.449778253572	-0.386504531973	4.240589271899
15727	O	-2.744447449241	-1.197062891000	-2.545073632873
15728	H	-3.391124601257	-1.514101916672	-1.889850087223
15729	H	-2.173576513430	-1.982218193027	-2.728345982368
15730	O	3.123879050408	-1.973175322950	-2.584393054299
15731	H	3.806520833382	-1.310106118627	-2.792171350673
15732	H	3.182888483673	-2.131567576515	-1.625426302285
15733	O	-3.866430496875	-4.234624984864	2.056014053443
15734	H	-3.446141908630	-4.427736898065	1.209121918073

```
H     -4.311944242114   -3.384379046937    1.969795322546
O      1.658955506314    5.043439708177   -0.719886961011
H      1.034089871674    4.867861995091   -1.450647609874
H      1.129537202089    5.042490567014    0.089771577187
O     -2.823355038979    1.654924769959    3.117909054179
H     -2.461140536315    0.755070987561    3.160099370345
H     -3.487879065498    1.635582868112    2.401903459248
O     -5.596480822452    2.772755637292   -0.794669287693
H     -5.195313093269    3.641341623393   -0.938257704279
H     -5.314610589508    2.253862492253   -1.565580793086
O     -2.592132402608    4.811342986742   -3.818224111371
H     -1.693577442307    4.698202102010   -3.444568901718
H     -2.751299040653    4.051779132530   -4.390825922457
O     -3.267866718344    0.872471451560    5.700846065648
H     -3.677814124082    1.458419661553    6.336879683348
H     -3.235027465720    1.351644955273    4.850658930582
O      2.560291670072    0.888135885337    3.947274872927
H      2.039355728574    1.571734122396    4.416280415276
H      2.904894589759    1.341935636991    3.161711184029
O      3.286996094648   -2.623587195869    0.124291873919
H      2.560005445983   -2.445365840604    0.744732512887
H      4.095692376839   -2.428652130524    0.604527069139
C      0.359672430370    0.899300442823    1.927951195305
C      0.644311483164    1.805245884636    0.995589214519
C      1.407456388170    1.562129289244   -0.261787493187
C      2.325608177796    0.617874767973   -0.450272140327
C      0.056152966064   -1.875115744936   -0.847098974763
C     -0.955244225462   -1.117232424259   -0.369979474222
C     -1.381041780135    0.078832041801   -1.123279866774
O     -2.229112225357    0.904107997272   -0.736422474084
H      0.296389525068    2.828213615525    1.117342089149
H     -0.190531195522    1.140652978707    2.825422395364
H      1.177889371686    2.253787097744   -1.069968159467
H      2.845093153190    0.498493323391   -1.388614776714
H      2.621921608094   -0.068455960669    0.329516612810
H      0.530020895222   -1.631575451902   -1.779685014533
H      0.415486572303   -2.754650457777   -0.342724335670
H      0.668301848418   -0.132413828458    1.841324656223
H     -0.810611544628    0.310235766402   -2.036061653840
H     -2.141423428255   -0.655876775476    1.247622936700
N     -1.699744247036   -1.423449456396    0.771132592821
H     -1.325015565584   -2.119227170636    1.395758448966

Ambimodal TS Water45-3
155
ANGSTROM
O     -1.534122838560   -4.656556546353   -0.259830316400
H     -1.984937438145   -5.501191996061   -0.291808489680
H     -1.782249711619   -4.236619099205    0.608680203594
O      4.369143860097    0.545298885675    1.632802222096
H      4.235686948692    1.484380087308    1.454581437077
H      4.874498083088    0.198700062385    0.875652248830
O     -0.248986448627   -1.175282336058    3.006537979279
H     -1.108390830946   -1.519259653118    3.296544257300
H      0.302177465826   -1.964682716326    2.821025875935
O      5.132293039587    0.507058835300   -2.732062461202
```

15791	H	5.740114807170	0.051547309235	-2.137083833677
15792	H	4.808809701059	1.256078703338	-2.189300082374
15793	O	-4.295101982498	-2.414577807069	2.680456095459
15794	H	-4.040338921298	-1.469709019463	2.695573172612
15795	H	-4.693365497923	-2.544286816167	1.805376849305
15796	O	4.215076623270	2.545686760790	-1.279714079187
15797	H	4.072810774692	2.788313829335	-0.355889151289
15798	H	3.708480238709	3.187798666347	-1.809921649044
15799	O	1.739845705857	-3.685834175671	-3.249827094201
15800	H	0.818923999823	-3.481427770085	-3.487459209100
15801	H	2.216183672460	-2.841876733783	-3.241926668597
15802	O	-0.966453718621	-0.081109078688	-4.645272596552
15803	H	-0.104801661493	0.045932026428	-4.197515152161
15804	H	-1.640130900000	-0.258448066460	-3.951702931018
15805	O	3.341820408020	1.987312123653	-4.481498409916
15806	H	3.324192573089	2.848326289723	-4.047358674203
15807	H	4.115321440127	1.522061582185	-4.138796856202
15808	O	2.627559429428	4.270969883099	-2.675214019538
15809	H	1.751587881650	3.860217399183	-2.806209978931
15810	H	2.522101448784	4.854201853403	-1.910798296004
15811	O	2.330943025710	0.258700619034	3.445600621102
15812	H	1.540028050463	-0.085670792326	3.012488172038
15813	H	3.040555921280	0.268379163442	2.780118899644
15814	O	0.593073339684	-0.736711628229	5.526278470746
15815	H	1.453185264627	-0.317695304924	5.451700012744
15816	H	0.299717417899	-0.865790990971	4.595136526978
15817	O	1.913072988230	5.329069222809	-0.148175658025
15818	H	0.986623985687	5.060008983903	-0.316470899477
15819	H	1.874118076483	6.168495393030	0.310928325490
15820	O	4.053873088520	-2.441348209974	2.146652415106
15821	H	4.148666907283	-1.488882955654	2.245482063137
15822	H	3.152117372904	-2.670791281138	2.439185522964
15823	O	4.234638833363	-2.872151830257	-0.483438245066
15824	H	4.172237448099	-2.811035856074	0.497921700900
15825	H	3.787801624785	-3.703839022571	-0.719173513234
15826	O	-3.194295008231	5.032274662540	-1.661210583212
15827	H	-3.668922132014	5.734536125978	-2.104305608276
15828	H	-3.361302158315	4.199849031537	-2.175675743791
15829	O	0.130859467632	-3.503373589861	5.111751599316
15830	H	0.520837460729	-2.759610111304	5.593285366908
15831	H	-0.788473190696	-3.218235510065	4.973563779456
15832	O	-5.200265928567	2.147860215800	2.188502414319
15833	H	-5.676155427997	2.658899200141	2.842808751280
15834	H	-4.643421630350	2.775892847056	1.682243243294
15835	O	-0.810522460250	-2.902389730565	-3.955748547729
15836	H	-0.877330352470	-2.059436083456	-4.425131393580
15837	H	-1.592034910972	-2.980884334796	-3.400949029535
15838	O	-3.116117228189	-3.026570793468	-1.841446918357
15839	H	-3.733354852209	-2.792408871014	-1.114625159429
15840	H	-2.433660957394	-3.566286979949	-1.407818852884
15841	O	-1.628377377129	2.448238756442	-4.776455937299
15842	H	-1.398374869362	1.475794662983	-4.826534718201
15843	H	-1.689373559579	2.757183812928	-5.679857309972
15844	O	3.437715711777	3.382183434850	1.288469561893
15845	H	2.962132583051	4.086842071532	0.821938556032
15846	H	2.929302065752	3.237640638959	2.106098723893

15847	O	-0.670410655768	-5.624780827589	3.344863384036
15848	H	0.013079789418	-5.705416330000	2.665909966438
15849	H	-0.265640331745	-5.206342352044	4.113974674223
15850	O	-1.955917996217	-3.626930931466	2.093656207726
15851	H	-1.642552596361	-4.352008853186	2.671678368389
15852	H	-2.849507284324	-3.354165797885	2.383096252095
15853	O	0.254096522053	2.925550618146	-2.839469290344
15854	H	-0.383372779701	2.936354834481	-3.573900060818
15855	H	0.679422615192	2.046820235766	-2.886370078797
15856	O	-0.590620180056	4.496988311296	-0.759680287523
15857	H	-1.437026482476	4.858839019770	-1.062741831374
15858	H	-0.332444597718	3.843347584076	-1.435332601140
15859	O	0.901354955519	-5.108271919148	0.972973057577
15860	H	1.603803356287	-5.114929429290	0.298732214497
15861	H	0.083735928021	-4.928247963383	0.472450306467
15862	O	-1.812677772873	0.746743139106	4.990252794839
15863	H	-1.497648910307	1.618040251760	4.676546576897
15864	H	-1.028008825890	0.334825809608	5.381463523616
15865	O	-0.718593160277	3.040917391510	4.032130460308
15866	H	-1.033686087182	3.693178217175	3.383964996000
15867	H	0.239013768899	2.995202968354	3.891964335284
15868	O	-3.545378481840	2.686311885988	-2.816752837042
15869	H	-3.162187720408	2.176540367298	-2.072474358820
15870	H	-2.919190670730	2.583704757852	-3.556563497947
15871	O	1.377578261634	0.521839929368	-3.406625074298
15872	H	1.906673214897	-0.208091545872	-3.033477413693
15873	H	2.025593048845	1.017555434702	-3.957084628896
15874	O	3.190517381917	-1.368991133895	-2.643787339244
15875	H	3.495492379370	-1.862210354411	-1.865226332913
15876	H	3.915298405071	-0.737187925571	-2.834965658592
15877	O	-6.030561797401	-2.266257866466	-2.539395192781
15878	H	-5.226409372175	-2.670522778317	-2.878790419641
15879	H	-5.985212950591	-1.328634211828	-2.767324779519
15880	O	-5.005005566012	-2.310152447615	-0.004032370275
15881	H	-4.931426567387	-1.335937989578	0.056831702978
15882	H	-5.662060773777	-2.459281346013	-0.705176524597
15883	O	-5.364816979829	0.531313234556	-2.680902115365
15884	H	-5.401942798996	0.511056563566	-1.704945369410
15885	H	-5.032436933654	1.412515074155	-2.905911466883
15886	O	-3.528076901490	3.585693526073	0.659508346844
15887	H	-3.586330750560	4.225069481192	-0.069188060521
15888	H	-3.083233999157	2.810459376982	0.272963928680
15889	O	1.478578305782	-3.187216312340	2.748576875399
15890	H	1.271108704120	-3.890493994157	2.094457823529
15891	H	1.177512057828	-3.498697838715	3.621626385965
15892	O	5.910756019996	-0.744882342033	-0.331994558516
15893	H	5.352999201203	-1.530734547744	-0.481494319141
15894	H	6.671715012013	-1.049682839390	0.163544172438
15895	O	2.620968129654	-4.984081941399	-1.162074213690
15896	H	2.976648628594	-5.793368120808	-1.529610179838
15897	H	2.201807971722	-4.503663026375	-1.928783213451
15898	O	-4.948085780710	0.397523210232	0.003114061844
15899	H	-4.017154857807	0.674793837975	-0.139588790773
15900	H	-5.257292537287	0.889843413389	0.773700480213
15901	O	-3.361059706392	0.148918379771	2.818078276766
15902	H	-4.005981680082	0.865120427781	2.724132934412

```
H     -2.787154073047    0.381826189291    3.573267088605
O     -2.286404660694   -2.169981721995    4.677069885222
H     -2.371529483778   -1.287986552330    5.056263509493
H     -3.131109761745   -2.383867069142    4.264275590197
O     -1.418655390186    4.750154501330    1.967324810150
H     -2.290836008311    4.412894205773    1.702951423903
H     -0.897474901051    4.724274122417    1.154747319663
O     -2.871133802507   -0.546724905430   -2.859017111089
H     -2.931972641843   -1.451648661639   -2.494845027986
H     -3.786318111656   -0.211954420433   -2.915655194932
O      2.060907244338    2.874080745288    3.639465991784
H      2.136112029498    1.886468209314    3.620452122837
H      2.533743747087    3.170527058637    4.417829427988
C     -0.031102695373    1.557319229266    0.976837884785
C      1.087027989044    1.571245856196    0.212360340737
C      1.906895266318    0.416225115809   -0.126301047320
C      1.661410487781   -0.854719065206    0.283933600566
C     -0.292074845273   -1.430954338340   -1.008511303724
C     -1.281731528933   -0.666839053508   -0.426068395565
C     -1.531439915588    0.687870839576   -0.906163265164
O     -2.543472648578    1.390144896657   -0.642358073416
H      1.359842066641    2.497309229513   -0.290476006577
H     -0.610904001835    2.453368965517    1.141128011774
H      2.690981605316    0.597090376410   -0.856825139437
H      2.267450130644   -1.683754917710   -0.042889535742
H      1.001072807820   -1.078555484234    1.104449734874
H      0.195803329061   -1.111438866907   -1.911970099480
H     -0.177614625410   -2.476414551336   -0.761710667508
H     -0.319353754152    0.721961251571    1.599703238429
H     -0.852415760142    1.056097211967   -1.688201095159
H     -2.559217145528   -0.498987319136    1.196063529433
N     -2.106246608486   -1.151299370919    0.570068246791
H     -1.847890815855   -2.018958699797    1.011846131313

Ambimodal TS Water45-4
155
ANGSTROM
O     -1.057202824319    2.521425146159    3.454096994279
H     -1.731856157599    2.719645524571    2.780951881118
H     -0.443646239795    3.285497935529    3.439449659449
O     -3.190657795669   -2.370358054879    4.029676527379
H     -3.795436222211   -2.182229663170    3.307767126697
H     -2.779862380611   -1.532655103047    4.272168568306
O     -1.976126537125   -0.127998985278   -3.931212666682
H     -2.638882674129   -0.489719974126   -3.331680185111
H     -1.303171687648   -0.830611607997   -4.015543501049
O     -0.017665807562   -1.270752767832    3.004434409415
H      0.702408263899   -1.358135079704    3.637073101996
H     -0.774001905025   -0.876985419084    3.474510770932
O      1.695353731541    5.234229651817   -1.094411327193
H      0.899270546660    5.118385408528   -0.525760124701
H      1.747057017677    6.166087984980   -1.309477581541
O     -1.734503097943    5.819744940007    2.638155503450
H     -0.905598475686    5.640962149994    3.107805423041
H     -2.361421850867    5.159719343748    2.944797365680
O     -1.240600737757   -4.594929227629   -0.426324754487
```

15959	H	-0.896988039983	-4.401408671704	0.459301665399
15960	H	-2.035494458230	-5.125995168275	-0.309165612551
15961	O	4.182944953667	4.239992330168	-0.288127012036
15962	H	4.053459137386	3.706965196307	0.511974123831
15963	H	3.313857026828	4.578355008111	-0.550398646677
15964	O	-0.318677105310	3.659622569432	-4.794738172115
15965	H	-1.115761178479	3.433038942268	-4.289326154432
15966	H	-0.146005794954	2.864411670004	-5.332789655684
15967	O	1.362495321670	1.280187711365	4.413981809853
15968	H	1.778179060202	0.472944750874	4.056797529026
15969	H	0.604161491110	1.505808305666	3.855845234143
15970	O	4.378146369958	-3.389175523534	0.597510410626
15971	H	4.713182034034	-2.944626810601	-0.199267976460
15972	H	3.542931825570	-3.800406592157	0.303863967827
15973	O	3.919009579445	2.238924978562	1.660639397887
15974	H	3.105411722400	1.767110390894	1.449095266680
15975	H	4.630609012497	1.724253476602	1.239254659904
15976	O	2.939060200552	3.497959064238	3.961222390516
15977	H	3.479096502393	3.183008162286	3.222399876022
15978	H	2.495583308083	2.709379834132	4.317540846512
15979	O	0.125923229046	-3.739448835323	1.896404991277
15980	H	-0.057176109615	-2.810034045665	2.147122467083
15981	H	0.942539151829	-3.956879765869	2.394304292232
15982	O	2.690096878664	-1.052591493584	3.892087553776
15983	H	3.216932161077	-1.109465140520	4.691156194205
15984	H	3.339828400688	-0.977517026689	3.142834800538
15985	O	0.713999544070	-5.883675350605	-1.897059364341
15986	H	0.907162202186	-5.368549303885	-2.685605820094
15987	H	-0.108057897707	-5.520532661143	-1.523862000887
15988	O	-3.902582372933	-0.978270997944	-1.975776122618
15989	H	-4.312974135949	-0.142605072306	-2.245806401121
15990	H	-4.292661532388	-1.140492625876	-1.087184885206
15991	O	-2.256097079757	-4.719131779454	2.931019903120
15992	H	-1.340096726253	-4.497742588267	2.694512763682
15993	H	-2.524853975918	-4.062395440749	3.592038917307
15994	O	-3.981096118404	-5.156932552507	-1.167193367901
15995	H	-3.584999337197	-4.572844852211	-1.846923692616
15996	H	-4.781604677686	-5.522989185296	-1.541586267296
15997	O	0.010690655384	1.053004087806	-5.598709455636
15998	H	-0.881661214795	0.697769663271	-5.642561820569
15999	H	0.271841183797	0.932069736061	-4.648785712224
16000	O	4.466120412112	-1.118267901097	2.007805370491
16001	H	4.447725189849	-1.989274878609	1.550880170718
16002	H	4.760142503750	-0.486962000013	1.341262441456
16003	O	3.160445039084	-0.634004931912	-3.606529062878
16004	H	3.589458799107	0.215794536401	-3.800158857680
16005	H	2.741534155269	-0.917124326331	-4.456482021572
16006	O	3.847906534983	2.030329601269	-3.969513427050
16007	H	4.092757069354	2.361948698525	-4.833708984989
16008	H	3.068631417866	2.557934102407	-3.677950422978
16009	O	1.651134523462	-1.177725108713	-5.732536912253
16010	H	1.175716174000	-0.347912750015	-5.916366924064
16011	H	0.973401651591	-1.781977088895	-5.402468409889
16012	O	-2.412429631569	2.499177942453	-3.381837353051
16013	H	-3.368931004083	2.407509378472	-3.306515581806
16014	H	-2.097975725192	1.589363608996	-3.557134505900

16015	O	5.491191438168	2.430472680507	-1.780878338067
16016	H	4.954028886527	2.254926945716	-2.571307470751
16017	H	5.078277881376	3.196187267956	-1.326716247551
16018	O	4.818438958814	-1.623279973109	-1.486183748049
16019	H	5.085144005296	-0.819137344235	-1.019166553164
16020	H	4.314348973951	-1.337220462479	-2.260429745244
16021	O	-1.900516896576	0.144128010551	4.434206129015
16022	H	-1.641395269062	1.028810227480	4.113127461269
16023	H	-1.447436688364	0.062607223771	5.295952384180
16024	O	5.569714504957	0.665813206239	0.087040723571
16025	H	6.501121736194	0.581734351465	0.297504652072
16026	H	5.529891570268	1.328900244336	-0.672195875911
16027	O	0.857086384749	0.771873615049	-3.106077471324
16028	H	1.164914061491	1.682110275581	-2.956655311744
16029	H	1.655972465660	0.217853260030	-3.180490631502
16030	O	1.700538742650	3.359927853519	-3.120535196062
16031	H	0.984070912037	3.595342929773	-3.762562968846
16032	H	1.711473883832	4.051010097620	-2.439000178315
16033	O	2.549036952413	-3.777937871156	3.035513482503
16034	H	3.273528806792	-3.878933430327	2.409096213298
16035	H	2.589821190921	-2.864153408678	3.355199980313
16036	O	1.944235763434	-4.206608358900	-0.228705253918
16037	H	1.669401917295	-5.022799240313	-0.695976135590
16038	H	1.280319233886	-4.049945264718	0.461669008660
16039	O	-2.519038090420	4.247756638747	-1.246539443139
16040	H	-2.784468825581	4.946752847346	-1.845441469732
16041	H	-2.373277363330	3.461559776109	-1.806683034431
16042	O	0.670432961838	4.556259767834	3.088257257614
16043	H	1.548728563825	4.304922739281	3.461370688710
16044	H	0.770321404888	4.565835049756	2.129986790817
16045	O	1.778349260338	-3.141204552219	-2.785120912018
16046	H	1.896301047764	-3.394623575097	-1.853936081086
16047	H	2.438657210484	-2.468246895015	-2.972748744802
16048	O	-5.044750535630	-1.216552169392	0.447955667607
16049	H	-4.805430267821	-0.566581610615	1.122022255738
16050	H	-4.636955261448	-2.055815887890	0.728312152785
16051	O	-3.627799235372	-3.455628308991	1.008596687926
16052	H	-3.236228277212	-4.000181804956	1.726324476639
16053	H	-3.882078624255	-4.075896374801	0.309566678094
16054	O	-0.439024702186	-2.378223238276	-4.141098382367
16055	H	0.330465143415	-2.660704382290	-3.609368072792
16056	H	-1.196180004895	-2.871781959709	-3.786739724677
16057	O	-2.989674119890	3.080595221904	1.563118741615
16058	H	-3.151546600571	3.911294180627	1.108628519007
16059	H	-2.762370261269	2.459022463577	0.836109880839
16060	O	-0.119418226980	0.270285230478	6.453602849886
16061	H	0.519424041701	0.690978423369	5.841613876977
16062	H	-0.166182096573	0.826723373341	7.230628179570
16063	O	-2.533871811855	-3.305923041920	-2.543511538611
16064	H	-1.958763443500	-3.469809440555	-1.778742165882
16065	H	-3.033093198186	-2.493578439837	-2.351043461531
16066	O	-3.947174878663	0.503404353400	2.429721765360
16067	H	-3.417649813167	0.350802143067	3.222025856371
16068	H	-3.838361672942	1.437342615943	2.210506253600
16069	O	-4.679639100257	1.693509703640	-1.924740849550
16070	H	-3.904402261096	1.676091653111	-1.326131064792

```
H     -5.452217359722    1.692760290312   -1.358379652872
O     -0.475617630905    4.908896336052    0.381417208972
H     -0.935778329943    5.414818676285    1.076107046551
H     -1.176560747164    4.617206496778   -0.234831855379
C      0.272650848708    1.656749392659    0.767715089658
C      1.377622879009    1.538284578485   -0.017922084643
C      2.091807754265    0.305682576058   -0.279020011010
C      1.736637593409   -0.915576376459    0.231795175680
C     -0.219427272662   -1.382774068995   -0.885510143302
C     -1.193650229792   -0.611477960356   -0.273903756407
C     -1.475858639224    0.741900265322   -0.758627622775
O     -2.471304482932    1.418400756155   -0.398972290487
H      1.692254868396    2.406031155659   -0.597766151339
H     -0.236525920470    2.604339483166    0.857221510060
H      2.846244832115    0.350363778132   -1.061340794181
H      2.280039530472   -1.801576136725   -0.059731268426
H      1.163625947615   -1.009672024465    1.142363966247
H      0.191337900230   -1.059196036350   -1.826137984347
H     -0.131998675524   -2.435610372665   -0.664610988608
H     -0.036175338659    0.891421360024    1.465189908908
H     -0.903339739090    1.083117776203   -1.632345505782
H     -2.643013497654   -0.425947777584    1.160230536921
N     -1.899429286329   -1.027820452226    0.833730246330
H     -2.043881407229   -2.008857650557    1.010202715880

Ambimodal TS Water45-5
155
ANGSTROM
O      2.830254465027   -0.626660064448    3.187559894212
H      2.700812265134    0.212042682388    2.705144752891
H      2.732087531326   -0.364098636823    4.124079634098
O      4.818835400548   -1.465546296023   -1.415531838113
H      4.272999135766   -2.191159859601   -1.741996341722
H      4.694174423782   -1.443521830005   -0.445428618877
O      5.408603697751    0.918450497822    3.584099984727
H      5.125228587322    0.014583572426    3.748303797388
H      5.973121301253    0.884792363798    2.795022240475
O      4.695321901970   -1.565072334727    1.303236202718
H      5.343353937539   -0.821979503590    1.294056365795
H      4.002049013130   -1.309465479859    1.930885903122
O     -3.399991527268    0.273656127750   -3.348358284577
H     -2.947457855729   -0.301006115438   -2.715949171890
H     -4.341783850591    0.208112258204   -3.059121425100
O     -1.294687170283    3.540473766785    3.284356124256
H     -0.860047571543    2.953213323656    3.924278017214
H     -0.573112518528    3.982576128356    2.806920674103
O      3.043157996203   -3.533385548256   -2.146298563298
H      3.304901417719   -4.216272269336   -2.765717695732
H      2.190211026809   -3.837527255714   -1.750748187729
O     -0.289639473833   -0.500123153239    3.371968235850
H     -0.016703033159   -1.008143040464    4.155385333337
H     -1.262603550899   -0.556649594522    3.350927017106
O     -0.383247123431    3.724080522208   -2.573975267755
H     -1.334630160470    3.478603393345   -2.605571135340
H     -0.183002028823    4.135341865455   -3.414678536220
O      0.083006720427    1.674399549870    4.806317602788
```

16127	H	-0.585541963945	1.379151028662	5.465897221141
16128	H	0.018082819804	0.977873633181	4.102266487171
16129	O	7.293228898482	-0.223409866046	-1.404441988204
16130	H	6.547040300000	-0.838027613004	-1.491409677292
16131	H	7.159124195455	0.451076602097	-2.073215050969
16132	O	-1.746844719094	-5.226622954434	-1.794100102131
16133	H	-1.866841840762	-4.559189247170	-2.500697160577
16134	H	-1.940123170484	-6.075966787993	-2.192114823008
16135	O	3.167690198715	1.987905007435	2.469420248322
16136	H	4.029179746108	1.739296816742	2.864634382555
16137	H	2.775752448756	2.594076187095	3.133878681321
16138	O	0.216820701578	-6.122819154970	3.071620410797
16139	H	0.953205182297	-5.848070532056	2.484055670972
16140	H	0.178846321450	-7.078238539182	3.044409283311
16141	O	0.363328239166	-4.102031932840	5.077388023119
16142	H	-0.567347199362	-3.794203210559	5.036037400561
16143	H	0.359091888626	-4.964903402946	4.641836565429
16144	O	-4.497407626488	-1.870237163098	1.873236064889
16145	H	-4.810591142659	-1.194147453607	1.247641679588
16146	H	-4.287493907774	-2.656915977545	1.360073407821
16147	O	-2.098958570969	-3.126584434562	4.545248841584
16148	H	-2.473332026109	-2.240823744364	4.564390287193
16149	H	-1.986402787266	-3.347750994906	3.604499235321
16150	O	2.421379084953	0.534458498421	5.604823012564
16151	H	1.604462532446	1.034723387706	5.379797036458
16152	H	3.110355088227	1.193312822114	5.713811508871
16153	O	3.273980064045	2.515545817902	-2.402717444995
16154	H	3.753893757335	1.753230754585	-2.811464142495
16155	H	3.595720879699	3.305357312665	-2.837234352938
16156	O	-3.166714343323	1.826566764838	2.336468043902
16157	H	-2.861853133942	1.745266544965	1.420509041673
16158	H	-2.599658618362	2.518937930656	2.729592705497
16159	O	0.971258523102	4.537640097223	1.972053194096
16160	H	0.579681599637	4.930498905655	1.167674189280
16161	H	1.580562830364	3.863601421571	1.656152559525
16162	O	0.877723058808	-4.506478331218	-1.029592647795
16163	H	1.141749163092	-4.749698070212	-0.117630694701
16164	H	-0.015313322113	-4.840156732468	-1.190580453161
16165	O	-5.628986952448	-0.576660107695	-2.206341134327
16166	H	-5.769246699539	-0.108770230172	-1.372746133400
16167	H	-5.007105685011	-1.291780359148	-1.970761700892
16168	O	-3.415126738186	-2.046903681435	-1.650351473669
16169	H	-2.951005261731	-2.535384083524	-2.353217636647
16170	H	-3.400898528951	-2.641378046074	-0.879423104247
16171	O	-5.002538899193	0.402335097557	0.356727546623
16172	H	-4.131736926323	0.713541736051	0.040143185902
16173	H	-5.235359113094	1.002901443221	1.069377553994
16174	O	-1.035331513782	0.481967132372	-4.905357053175
16175	H	-0.357243647628	0.859153074924	-4.306344279272
16176	H	-1.882396191700	0.562639449493	-4.450298459521
16177	O	-1.101542009096	-3.851078407082	2.088511880024
16178	H	-0.300886306523	-3.304502407734	2.224801787704
16179	H	-0.838057671468	-4.737671058172	2.379047386426
16180	O	2.059747491703	3.565000338121	4.358827708571
16181	H	1.721241113568	4.284817581108	3.813465229714
16182	H	1.285948526395	3.044846018334	4.623256842888

16183	O	-2.977615557350	2.993631129672	-2.522220136450
16184	H	-3.189358879213	2.235099811791	-3.082331513185
16185	H	-2.960526125102	2.630881948128	-1.618694076889
16186	O	-2.999685812272	-0.478307503571	3.688087266699
16187	H	-3.116100510939	0.419419137369	3.299737520526
16188	H	-3.608812036735	-1.044891435618	3.179662197640
16189	O	1.950597872722	-4.952957847973	1.398737002086
16190	H	1.773756481253	-4.116627979645	1.886838304176
16191	H	2.887952512822	-4.872349114862	1.122569456971
16192	O	6.280463259018	0.556394492410	1.014388204515
16193	H	6.867712802449	0.307718601427	0.277979275627
16194	H	5.707909345232	1.269620813859	0.653087877736
16195	O	4.578515361726	2.435049066539	0.115838430540
16196	H	3.951223805313	2.384005476105	0.855759659754
16197	H	4.055665292529	2.453604722174	-0.700844654740
16198	O	-0.173044123037	-1.976570733431	-4.968246543945
16199	H	-0.530686879772	-1.039943397998	-4.958635781499
16200	H	-0.148416796130	-2.229582348139	-5.891866548724
16201	O	4.427250555038	0.347636419351	-3.406460084605
16202	H	3.735029367121	-0.152446029867	-3.859998619081
16203	H	4.656673159326	-0.210829992339	-2.640319429791
16204	O	4.321231941316	-4.194064119960	0.424313688719
16205	H	4.044213187906	-3.973189123163	-0.474189775582
16206	H	4.577425233984	-3.356055514035	0.842721105965
16207	O	-2.153065501263	-3.357064692266	-3.710209833139
16208	H	-2.887341809875	-3.034688274175	-4.271951571722
16209	H	-1.355348379744	-2.917105882084	-4.063588355999
16210	O	0.458531033801	-1.450312831035	5.858102896432
16211	H	0.642776236872	-2.393245389980	5.931260344642
16212	H	1.268967681997	-0.965616705436	6.056076201078
16213	O	-3.157000767745	-3.981722186823	0.268964235373
16214	H	-2.480736329198	-3.965415034139	0.966869489939
16215	H	-2.784425193317	-4.560955523545	-0.414273090116
16216	O	2.240469673780	-1.357117695812	-3.796663059320
16217	H	1.430411548297	-1.656796354454	-4.246927643136
16218	H	2.457550274508	-2.043551381261	-3.147821361459
16219	O	-4.102501810508	-1.938277399741	-4.906142264623
16220	H	-3.803414335359	-1.071816077712	-4.577484186590
16221	H	-5.008132068778	-2.028868265971	-4.602404913152
16222	O	-0.280901322811	5.236024850667	-0.326661178735
16223	H	-1.214837140816	5.416342836056	-0.209227750601
16224	H	-0.211847633645	4.661166820085	-1.116941258036
16225	O	1.286463680369	-2.852023121930	2.868880555533
16226	H	1.108187985170	-3.209457789095	3.762659020919
16227	H	1.792145178871	-2.023338925102	2.969772411432
16228	O	-1.740068476757	0.287550508606	6.154027747237
16229	H	-2.359929335728	0.052690367928	5.449509977562
16230	H	-1.145568141521	-0.469261682883	6.260382478534
16231	O	0.934781285657	1.027661399260	-3.187043347793
16232	H	1.518756832959	0.269206419922	-3.351536945484
16233	H	1.501135479381	1.754790560984	-2.897378128043
16234	C	0.165950848853	1.611141657491	0.804564819935
16235	C	1.257016542941	1.507186994603	0.011457999140
16236	C	2.019136199789	0.283230013279	-0.235952203087
16237	C	1.724523835385	-0.933854200422	0.272257718625
16238	C	-0.343166042842	-1.381291051244	-1.119644275040

```
C     -1.224083022993   -0.641024280041   -0.379674780482
C     -1.552436844223    0.730407097782   -0.784611476226
O     -2.547019921122    1.377673199871   -0.366137962301
H      1.549220002040    2.366660240815   -0.591962874041
H     -0.374600876139    2.542999930682    0.897249708005
H      2.808785932238    0.370259989133   -0.978705828726
H      2.278445275536   -1.819717976155   -0.003159716379
H      1.024460907254   -1.071194169894    1.082367956622
H      0.080407413749   -0.982715117172   -2.024891576605
H     -0.162290825913   -2.427325121509   -0.928239061691
H     -0.116960713555    0.842652998253    1.510629274517
H     -1.003279481758    1.129796077348   -1.651439983048
H     -2.588106733452   -0.610181142093    1.172672259805
N     -1.792592954130   -1.093823904617    0.795858922386
H     -1.708961985877   -2.060069741578    1.051051404198

Ambimodal TS Water45-6
155
ANGSTROM
O      1.579920363103   -0.491398796099   -6.213154145489
H      1.215362432660    0.395326924570   -6.341378207015
H      1.067426455207   -0.888551818438   -5.490510274473
O      5.792347825581   -1.517911476491   -3.249177125498
H      5.929297818803   -0.625731217496   -2.892693852283
H      5.254437275309   -1.378331243061   -4.040059753635
O     -1.587598639025    0.621374598422    3.712562818002
H     -1.011531131250    1.349323981969    4.035001016788
H     -2.228079539332    1.069563170345    3.123516186578
O     -2.355588166171    0.361814751855   -3.708633150489
H     -2.650789249868    0.206667152933   -4.613013740137
H     -2.885453893138   -0.248245676845   -3.145548832248
O     -3.220888343982    3.761485091852   -1.564707528958
H     -2.921738523567    3.747568813762   -2.481160802330
H     -3.001698940960    2.882810690968   -1.203737004752
O     -1.969276566985   -3.423836532460    2.729256197728
H     -1.369630367869   -4.008139075009    3.195970590325
H     -2.297523738621   -2.776734930352    3.392489697123
O     -0.117144534355    1.812024955690   -5.759305247381
H     -0.754453701056    1.108462097664   -5.971401185503
H      0.352673244280    1.543450188614   -4.947510082933
O      1.943848573017   -5.578721441569   -1.667059446126
H      1.913672864149   -4.912391379178   -2.409386436103
H      2.172599629545   -6.416251251148   -2.068982882334
O      1.720847417964   -3.840369012573   -3.610902929907
H      1.917833714507   -2.902701983867   -3.401044373180
H      0.769862191453   -3.875859686082   -3.761153878146
O      0.127247483183    3.204976108726   -2.122540179010
H     -0.543606967175    3.323119812766   -2.831316441775
H      0.879311587516    3.793880273600   -2.298805857610
O     -4.347202503249   -3.310438755168    1.394535709869
H     -3.558579103133   -3.536264688742    1.916680334793
H     -4.573879031507   -2.408843331853    1.647500925581
O     -5.040985204315    0.359105160743   -0.485682452528
H     -5.633290716951    1.050631193116   -0.782934642845
H     -4.144840472904    0.755043906804   -0.464964510774
O     -0.471132066434   -2.801262243507    5.354357634112
```

16295	H	-0.297423306778	-2.333488494699	4.505804973436
16296	H	-1.339207588900	-2.503905279038	5.635946761246
16297	O	-0.316249600338	-4.637059542004	-0.361113179962
16298	H	0.412216069680	-5.141766495483	-0.757177572292
16299	H	-0.022423470840	-4.418150081389	0.536339467932
16300	O	2.203230518240	0.192781685350	3.919887260521
16301	H	3.108075511161	-0.078565328835	3.674478239691
16302	H	2.105835657577	1.109327362623	3.627956638598
16303	O	-1.101645988103	4.730383838395	-0.220532331362
16304	H	-1.987548285397	4.646420293743	-0.630516516137
16305	H	-0.565614530393	4.113451355343	-0.758998849044
16306	O	4.701372196720	1.747921427433	2.203466802743
16307	H	4.753672880424	1.254762898624	1.372978412335
16308	H	4.863881162337	1.080938589642	2.882143323414
16309	O	-0.309295580869	-1.334175224350	-4.276913153749
16310	H	-0.537919505563	-2.166792835313	-3.827551153319
16311	H	-0.875387195020	-0.653058739045	-3.867487844770
16312	O	3.698333286994	2.380124897683	-3.390630084476
16313	H	4.317871470065	1.851780973502	-2.862407006449
16314	H	2.857434116968	1.879377130960	-3.460740191836
16315	O	-2.990474049163	-3.608986367906	-0.914367193125
16316	H	-2.187011977159	-4.022419279157	-0.573967280757
16317	H	-3.600316800343	-3.558551845522	-0.149262442496
16318	O	-3.721046170020	-1.372971538501	-2.258802799051
16319	H	-3.416148942986	-2.175012466731	-1.793119852680
16320	H	-4.355902527796	-0.949080408948	-1.664656363987
16321	O	5.726899680419	1.023583685545	-2.012335124228
16322	H	6.475866468242	1.618411525231	-1.963513086440
16323	H	5.604877304338	0.650623562130	-1.114541325555
16324	O	2.577330229909	4.448883720128	-2.102300151292
16325	H	2.875922553792	5.259086258864	-2.515863488929
16326	H	3.068338489797	3.717284364229	-2.549680411973
16327	O	5.346163504698	-0.230701208175	0.339963021645
16328	H	4.839785094773	-0.937424359714	-0.123447944059
16329	H	5.902688215133	-0.677663791291	0.980369788941
16330	O	-3.257126189231	1.998517494420	2.105695930638
16331	H	-2.891186516249	2.882192665828	2.280338223261
16332	H	-2.994988867349	1.792971763123	1.189014305361
16333	O	2.102592107534	-1.976128671535	5.760586546589
16334	H	2.094446358555	-1.115846031285	5.316802617834
16335	H	1.167910113208	-2.232450402498	5.872235876851
16336	O	0.993522978583	-3.712895463019	1.980268699284
16337	H	0.569249417442	-2.876059333860	2.234277271657
16338	H	1.535376998161	-3.945446745205	2.754922062139
16339	O	-1.026298847248	-3.645120246356	-2.984850592171
16340	H	-1.984688872637	-3.660322208142	-2.900908975191
16341	H	-0.705257746173	-3.889584741554	-2.102563832287
16342	O	2.290730353925	2.900693307248	2.791141517053
16343	H	3.202163777439	2.611259008786	2.598056914847
16344	H	2.113543507717	3.605507112647	2.148324189152
16345	O	0.141345005693	-1.350540333675	3.192614545999
16346	H	-0.588754350552	-0.700684463266	3.242443512920
16347	H	0.932748451093	-0.842020547163	3.466626410392
16348	O	2.668624239876	-3.977306479343	4.148426077193
16349	H	2.490342307485	-3.197109928368	4.742276315136
16350	H	2.660604320892	-4.745724977199	4.718858824594

16351	O	3.125199776457	-4.072823349488	0.261734339349
16352	H	2.342979160095	-3.884903260605	0.817525956678
16353	H	2.817714988666	-4.728900048262	-0.386749337845
16354	O	1.290346835778	1.164463424808	-3.478165354689
16355	H	0.855968679641	1.692331304800	-2.782716264224
16356	H	1.458102906843	0.256665763510	-3.162049621015
16357	O	-1.685953180751	-0.416280208501	-6.401068958737
16358	H	-1.160864793463	-0.971015158118	-5.786570280459
16359	H	-1.496561914472	-0.725685660110	-7.286793763828
16360	O	-1.818930744664	4.341212505304	2.315796187583
16361	H	-1.512673499620	4.502040241340	1.390110570715
16362	H	-2.127925353148	5.183885601914	2.648277942885
16363	O	3.913034926890	-0.277296307725	-4.941554401690
16364	H	3.164061802871	-0.386046405064	-5.570461583041
16365	H	4.025020465579	0.671352814441	-4.815679242094
16366	O	1.753879085683	4.839407538256	0.730294705792
16367	H	2.156664697838	4.726294355527	-0.140340407958
16368	H	0.826079593831	5.038917599216	0.568389628171
16369	O	4.748134551000	-0.719003661731	3.555933748112
16370	H	4.708766742925	-1.563339499293	3.046203438797
16371	H	4.977773710899	-0.967319841299	4.452823120220
16372	O	4.622406327289	-3.111645018277	2.421847979939
16373	H	4.015526752408	-3.503810761205	3.074506494451
16374	H	4.279616984781	-3.409010726024	1.567423405751
16375	O	2.355640383977	-1.271819455807	-2.959282278815
16376	H	2.933250505344	-1.485197023706	-2.200154656467
16377	H	2.964484558944	-1.012083820519	-3.681633794656
16378	O	-0.168704903933	2.848114679933	4.103088573478
16379	H	0.732297270091	2.885482686652	3.748104791067
16380	H	-0.695219800966	3.432138064484	3.538207823090
16381	O	4.210308111904	-2.069515211490	-1.190756427131
16382	H	4.867976783301	-2.162305245610	-1.913808702472
16383	H	3.987897594405	-2.936200521680	-0.807290829505
16384	O	-4.599200966300	-0.461622408038	2.114025961974
16385	H	-4.324164365452	0.419565368423	2.403695868944
16386	H	-4.995620274778	-0.313731027684	1.239992029602
16387	O	-3.036107342355	-1.560262446612	4.364328819627
16388	H	-2.503898139234	-0.744721084841	4.290542368749
16389	H	-3.833109391751	-1.379289241308	3.854664575147
16390	O	-1.788373507003	3.036945828259	-3.974556885953
16391	H	-1.255575006355	2.912542181515	-4.781980962339
16392	H	-2.096428765661	2.136653441527	-3.761404669884
16393	C	0.124499685265	1.662460815384	0.910999412602
16394	C	1.276167456797	1.628212734596	0.194893584141
16395	C	2.099525028557	0.453111595979	-0.023716305480
16396	C	1.846057246833	-0.773350087729	0.500919346299
16397	C	-0.137316129890	-1.382852050565	-0.769169116011
16398	C	-1.164402270474	-0.657476492938	-0.204295376051
16399	C	-1.500916380814	0.668162430412	-0.724750934373
16400	O	-2.533299179162	1.329230594405	-0.426813744667
16401	H	1.583081366735	2.518465789714	-0.346401978346
16402	H	-0.443546248305	2.574299580270	1.009432976581
16403	H	2.903783727029	0.565119896495	-0.749856943153
16404	H	2.459242911161	-1.626259392222	0.262365234966
16405	H	1.177148743720	-0.909980835388	1.332870248794
16406	H	0.337473732902	-1.072686449474	-1.682121686730

```
H      0.039198777640   -2.403841682867   -0.473953896139
H     -0.168532813085    0.871189276411    1.583771617900
H     -0.911196519725    1.024347934034   -1.580037803015
H     -2.704167701822   -0.656502224955    1.194361976580
N     -1.845269002721   -1.098687415441    0.910892828129
H     -1.710800691648   -2.035553835357    1.257719687692

Ambimodal TS Water45-7
155
ANGSTROM
O     -4.818756701534    0.714413697014   -3.306612797169
H     -5.096546977462    0.785001759738   -2.376293502005
H     -4.586792331509    1.603100277742   -3.596378399172
O      1.960791052053   -0.510076997265    3.662792610053
H      2.453002506447    0.203357861148    3.210937292585
H      1.016821553607   -0.351703208202    3.398056687846
O     -5.171249580601    0.836582252725   -0.538506078642
H     -4.211279945076    0.967581505973   -0.427542737776
H     -5.361137895295   -0.018350240284   -0.128864598388
O     -2.437672328128    4.839918654997    2.311476977932
H     -2.479180538463    3.861709308963    2.320908886143
H     -3.242629157467    5.096862363254    1.841266015586
O     -4.743193996703    4.700788218395    0.773629721825
H     -5.505948646226    5.256228939293    0.614539152068
H     -5.098041618227    3.856050189412    1.162564498080
O      1.106234524174   -4.400190542485    0.725020963991
H      1.261006627270   -4.224830012926   -0.234022129110
H      1.456895616380   -5.276056300148    0.891270375951
O      2.925708557416    4.356920024627   -0.545887988188
H      2.502982270311    4.386042561677    0.318746701242
H      2.464156218928    4.991546191031   -1.115718554953
O     -4.828127768720   -1.477371291071    0.999316424339
H     -4.805534936660   -2.254931893613    1.584646470210
H     -4.476108940733   -1.813275170530    0.150765597072
O      5.458124452586    1.618766607091    1.395350123144
H      6.320757230054    1.907832995055    1.693980331530
H      5.514584741794    0.643501698299    1.249496686997
O     -1.040068564495    4.722430151339   -4.420061928738
H     -0.126338512588    4.634754618172   -4.719314097240
H     -0.985328041938    4.940633252759   -3.479630537810
O      1.401965207718   -3.201535027127    3.367722765981
H      1.457196295228   -3.464006486858    2.439709334294
H      1.797251188346   -2.315399449955    3.434324313828
O      3.020652503875    1.775407656991    2.675200563712
H      3.012986407891    2.175939660957    3.568894694737
H      3.920605460758    1.806787514437    2.314061830892
O     -0.303984066978    5.571910010695    0.818151187847
H     -1.082966424393    5.403061240248    1.389946276581
H      0.432239246997    5.089614642449    1.229202523208
O     -1.219674062809   -2.778331133528    3.574033713113
H     -0.263434941217   -2.980688670863    3.696825837461
H     -1.463276117375   -3.275393834966    2.783834076293
O      4.546083275090    2.248170449560   -1.224778500492
H      3.977461982136    3.020329910481   -1.056274177962
H      4.914456939319    2.048329802738   -0.350484297540
O     -3.031386219791   -1.077069724013    4.896854171903
```

16463	H	-2.506998147752	-0.406489996889	5.353638792581
16464	H	-2.397988011445	-1.727512436558	4.557534849905
16465	O	-3.761235847958	-2.359972267429	-1.309426147142
16466	H	-3.390597925193	-1.785797564277	-1.992215044622
16467	H	-3.264759176167	-3.189081686761	-1.355950523430
16468	O	0.972659058254	-1.578742947374	-4.944260786630
16469	H	1.226118353203	-0.794372943775	-4.412599341303
16470	H	0.705164035941	-1.252141789582	-5.802954988151
16471	O	1.514838400752	0.239761084872	6.129138920409
16472	H	1.836936269508	-0.404153496152	6.758815348871
16473	H	1.742087269129	-0.110603139928	5.221345689380
16474	O	-0.494198792277	3.214271305757	3.940383431159
16475	H	-0.982851222541	4.042101350895	3.980524181498
16476	H	-1.006473132312	2.657362452577	3.317258024610
16477	O	1.913567605092	4.138461807961	-4.223393125482
16478	H	1.388882670914	3.532457592854	-3.654579793984
16479	H	2.077175081858	4.916781108378	-3.678986593601
16480	O	-1.072040929100	0.944657987599	5.574610570782
16481	H	-0.970115980348	1.861578199777	5.283466856774
16482	H	-0.202985951435	0.681357101480	5.930727495272
16483	O	-4.305674007871	0.380659058947	2.981634870999
16484	H	-4.554342372695	-0.262477980728	2.295195486756
16485	H	-3.970962256100	-0.148797990418	3.732191984100
16486	O	0.317608914829	2.595202538236	-2.732043447235
16487	H	-0.093788863759	3.304949949311	-2.197382005781
16488	H	-0.354654781542	2.298575168209	-3.376329848677
16489	O	1.463126056176	6.084790302623	-2.177219555187
16490	H	0.593117918239	5.682859391663	-2.016097765886
16491	H	1.435227129843	6.957053354846	-1.782548240738
16492	O	-2.061645625734	-4.556431581594	-1.623486805573
16493	H	-2.403423703361	-5.330773234282	-2.071170882057
16494	H	-1.550277873193	-4.054881480424	-2.288065723960
16495	O	1.532445547811	3.986475194700	2.085094619906
16496	H	0.882068035465	3.803336248239	2.780336202448
16497	H	2.101542161369	3.193838302981	2.092111208938
16498	O	3.770149131628	2.074774093034	-3.933248872778
16499	H	4.174658552180	2.233144206531	-3.067607758744
16500	H	3.272841206788	2.882057090686	-4.158870660580
16501	O	-2.409554517958	2.142821346264	2.321750441489
16502	H	-2.375095839133	1.867148471513	1.390613164083
16503	H	-3.082629695485	1.550952511740	2.716230033669
16504	O	5.719002500198	-0.955123277821	0.791102052589
16505	H	6.555034595632	-1.414359569818	0.872616600795
16506	H	5.558791996405	-0.851828014657	-0.180876817140
16507	O	5.340459802984	-0.494190010214	-1.787063553575
16508	H	4.596209033585	-1.023778161956	-2.121327090837
16509	H	5.072591416745	0.434588270518	-1.826347728080
16510	O	-0.586353512718	-0.157599560322	3.138198211559
16511	H	-0.889423569277	0.262785475768	3.964306595534
16512	H	-0.940352525476	-1.064699958030	3.147088736919
16513	O	3.109970998213	-1.882610993811	-2.723442569045
16514	H	2.584098916743	-1.057787933617	-2.795318175805
16515	H	3.229439563300	-2.186222384961	-3.626463859671
16516	O	-2.610842465336	-0.802798567849	-3.365892219000
16517	H	-3.498695973672	-0.385711632218	-3.433657581469
16518	H	-2.018559244924	-0.115704692023	-3.706843985925

```
O     -5.731587143216    2.423076194802    1.616329195309
H     -5.329804616327    1.864192670310    2.296068925733
H     -5.697958905836    1.895010017778    0.795043586949
O     -0.861125414551    4.724255110508   -1.571032269041
H     -0.642641946491    5.042584379398   -0.653035599024
H     -1.795881017568    4.426420341010   -1.497441149173
O     -3.390229255107    3.250901141440   -3.926158027465
H     -2.829582471952    3.949835400663   -4.294558400790
H     -3.454267259962    3.449677309346   -2.969792116817
O     -1.589736296074   -4.124101148973    1.101916851414
H     -0.619941990500   -4.247481916034    1.063420759553
H     -1.890668825164   -4.338491350477    0.206052658589
O     -4.092299022368   -3.602663657742    2.547045641262
H     -3.332033296541   -3.966240848497    2.076418512828
H     -3.777379113946   -3.300873630123    3.403074412257
O     -0.874769291948   -2.997082231262   -3.497509818751
H     -1.547828089883   -2.296324609023   -3.458654272008
H     -0.176727937988   -2.622700354668   -4.065946920388
O     -3.280904571340    3.692411464459   -1.272587734965
H     -3.837604146885    4.114102702301   -0.589062600707
H     -3.020926800577    2.818450145369   -0.903118058703
O      1.844732221419    0.400026715555   -3.339231698120
H      1.260771833549    1.053563122956   -2.926613463549
H      2.616023902010    0.945325638187   -3.651353721028
O     -1.478791212279    1.507894100527   -4.431714676786
H     -2.266904189032    2.079720666942   -4.237278798085
H     -1.264993228296    1.665439030480   -5.351898215725
O      2.224786976234    2.715854320146    5.080582272448
H      2.072825385799    1.924456715633    5.619330960829
H      1.351147487801    2.995309335580    4.781591443140
O      1.475098698551   -3.967291788260   -1.896541302076
H      2.120457918577   -3.259568362447   -2.077002080901
H      0.708191911503   -3.763800026502   -2.446928704249
C      0.228879666346    1.661298461459    0.598091677304
C      1.400281915708    1.405465931435   -0.043402160854
C      2.113741991399    0.143216762884   -0.049689300182
C      1.684856285520   -1.011131938722    0.523423953804
C     -0.233333636941   -1.405326813979   -0.964781933273
C     -1.212614104737   -0.684206050422   -0.326387902120
C     -1.528498929942    0.679359835651   -0.736092685050
O     -2.512810651102    1.338677990374   -0.283316787485
H      1.816191060725    2.173277411717   -0.692786816396
H     -0.232941353211    2.637120473722    0.521678790770
H      3.006199783565    0.116275202214   -0.668952616488
H      2.246330836974   -1.928615781320    0.409955794083
H      0.898516828325   -1.039003719552    1.261934960768
H      0.262475277148   -1.018748244481   -1.837920543626
H     -0.092608348565   -2.451671607689   -0.754415143454
H     -0.143107037092    1.033171295559    1.394539060993
H     -1.047451955234    1.047407127431   -1.649424925565
H     -2.716445720937   -0.714081181987    1.079745824552
N     -1.870781532173   -1.175429358631    0.786784095026
H     -1.853692375593   -2.171699105725    0.944534795396

Ambimodal TS Water45-8
155
```

```
ANGSTROM
O     -5.157431340288    0.404655093125   -0.120647508877
H     -4.279100533641    0.665748252152   -0.440270864155
H     -5.269356493505   -0.551777341628   -0.334192356294
O     -1.525702707693   -3.272817663161    3.118899098255
H     -1.564435188809   -2.465758948931    3.642410321799
H     -0.580727747421   -3.452771269437    3.003058425660
O     -1.095003631768    4.878309817272    0.895298279442
H     -1.926441084472    4.380113902685    1.084370714729
H     -1.282430801959    5.795908056838    1.097165860922
O      5.319979930291   -0.937222375183   -0.182865473772
H      4.792853799190   -1.528722179328   -0.758088253390
H      6.228568272946   -1.230673056191   -0.260040609699
O     -5.207413541570   -2.235679632905   -0.396265367536
H     -4.529940617575   -2.581838035913   -0.998301962837
H     -4.973585520201   -2.556998937322    0.488012277655
O     -1.402969363011    0.072994578510   -4.641301641614
H     -2.025301660758   -0.124554324412   -3.905860211810
H     -1.523252794090    1.033468725916   -4.827420827086
O      0.830354526647    3.122584214019   -3.145063578311
H      0.229211638674    3.663601280584   -2.575459768026
H      1.552475969907    3.696627383435   -3.400598488724
O      4.799359840820    1.466795829330   -1.558846982840
H      4.432554614909    2.113545683622   -0.939702619983
H      5.090929431462    0.723568298260   -1.008451203300
O     -5.161689196428    1.376837386523   -3.465284689175
H     -5.592976450257    1.523298755137   -4.306309309184
H     -5.819265237150    1.653624878486   -2.757792889880
O     -3.811177821226   -0.035102276827    5.213951968048
H     -4.540644339474   -0.472751243359    4.769216791791
H     -3.010203731967   -0.303466295495    4.733902297116
O      3.938501077280   -4.280951683018    0.401975120237
H      2.998348298668   -4.505632850580    0.325783391766
H      4.143320624769   -3.740860606789   -0.372558608034
O      4.019259703940   -3.279393836254    2.818037733978
H      4.571611904977   -3.859186511910    3.342423300652
H      4.043686813400   -3.637538987834    1.889700156678
O      1.761100337994    4.495528294929    1.452013044163
H      1.624771995753    3.922356904803    2.233248701624
H      0.877405198641    4.644268176632    1.096021695881
O     -0.640067411843   -1.976365867757   -6.206268459999
H     -0.986199073155   -1.214909719852   -5.692951053852
H     -0.505230226879   -1.658851560884   -7.098739554333
O      1.282068374753   -3.175267623653    3.105291319097
H      1.146493210599   -2.546795031628    3.833251485519
H      2.249465950574   -3.266342201247    3.044265602683
O     -0.551562629902   -5.719743417071   -2.438962017926
H     -0.970279533898   -5.191585150264   -3.135879338990
H      0.390846453277   -5.486795764475   -2.457629112646
O      3.684407578516    3.222042972475    4.870342001832
H      4.195458610786    2.771496595237    4.153698421941
H      4.069722311066    4.094287812544    4.956249932161
O      1.424043319844    2.806270080853    3.548497852444
H      2.164643035663    3.046264128524    4.149190243799
H      1.630748036461    1.878820776208    3.281958679030
O      4.899621219123    2.120657075279    2.773416892966
```

16631	H	4.541960187380	2.476765904200	1.939832406027
16632	H	4.790238677133	1.160921100248	2.721396791178
16633	O	-4.226021607911	-2.757069947799	2.200558963227
16634	H	-3.334190476964	-2.961379101192	2.506855335900
16635	H	-4.345574619457	-1.807965229727	2.361631795769
16636	O	3.694904850141	-0.339749013146	-3.402246603116
16637	H	4.149129395463	0.371070676692	-2.920283135263
16638	H	2.772763442137	-0.055667596763	-3.488623944844
16639	O	-1.532786319722	-3.649226268674	-4.045808439483
16640	H	-1.682733847375	-3.460951700241	-4.976786308894
16641	H	-0.732362808544	-3.135731926036	-3.824511670635
16642	O	-3.032187572043	-2.952621026400	-1.889787476420
16643	H	-2.678582881681	-3.258909724847	-2.743048836435
16644	H	-2.471437830620	-3.400127364666	-1.224219607410
16645	O	-1.502752221939	2.713307655069	-4.669092125100
16646	H	-2.136983225262	2.910928418259	-3.959047518903
16647	H	-0.628641064615	2.911775332198	-4.297977179634
16648	O	3.944949551711	3.322546948371	0.453109170943
16649	H	3.093501675103	3.722891503639	0.747952075837
16650	H	4.549550380784	4.057243988936	0.336216143519
16651	O	4.327839764149	-0.590768449739	2.346653443073
16652	H	4.276647481444	-1.517801343846	2.636217456132
16653	H	4.617919365254	-0.631827931507	1.418164639435
16654	O	-3.220637073166	2.977113040007	-2.467529988772
16655	H	-2.959644630014	2.405976522938	-1.714334955038
16656	H	-3.903659336030	2.451556052178	-2.933960197641
16657	O	-2.923169095236	-5.408974877684	1.769074719582
16658	H	-2.414780173652	-4.949600892479	2.449050937568
16659	H	-3.785660759685	-4.983214304649	1.751375149066
16660	O	-0.941064375717	4.557818021691	-1.824763311201
16661	H	-0.944456172035	4.653832756475	-0.855577690627
16662	H	-1.802710170922	4.196138048979	-2.069655707028
16663	O	1.142367571468	-4.171816109080	0.476510532326
16664	H	1.167020804151	-3.782061927766	1.361137916037
16665	H	0.204259837214	-4.392756354711	0.310348983448
16666	O	-6.676495694417	2.179194822342	-1.516139251248
16667	H	-6.268848477872	3.026946780144	-1.243217292805
16668	H	-6.353042083225	1.522901949231	-0.873212069073
16669	O	-3.506369109531	2.270997896819	3.753830932359
16670	H	-2.558998031056	2.377372813138	3.946468132522
16671	H	-3.841536016719	1.724295049849	4.485140165509
16672	O	-0.889711377430	2.122209940000	4.525941640466
16673	H	-0.042506104169	2.458956080597	4.123378842169
16674	H	-0.808408048351	2.251981625627	5.471420830675
16675	O	-5.056177449114	4.254106690971	-0.815156886468
16676	H	-4.404920727446	4.018534487473	-1.496943261217
16677	H	-4.611715249258	4.089248619369	0.028996274921
16678	O	0.972113006198	0.366859892109	-3.440931679355
16679	H	1.040942170549	1.315078538134	-3.253319783961
16680	H	0.124029399336	0.271280797327	-3.931858901085
16681	O	-4.203132400303	-0.004925791928	2.310286128545
16682	H	-4.725218648911	0.220102401007	1.509524916569
16683	H	-3.991588854004	0.839523315096	2.739927484413
16684	O	4.007652792757	-2.551773319535	-1.888757519708
16685	H	3.915925341113	-1.819853993231	-2.538570359002
16686	H	3.350277443653	-3.220004439693	-2.158969057480

```
O       1.896583512827    0.231701079865    3.205721151261
H       2.774633667131   -0.009771457098    2.854721928838
H       1.789355666376   -0.246367574882    4.034726831143
O       0.685175994856   -1.405559397681    5.149786737758
H       0.425620241323   -1.625951703757    6.044175747061
H      -0.103765404289   -1.034426006097    4.697368625836
O      -3.108113066775   -0.311817516082   -2.621015558146
H      -3.220586510249   -1.243339275965   -2.364063103454
H      -3.963726395096    0.040409947447   -2.909719393968
O      -1.408274442681   -0.424177454696    3.780293616523
H      -1.206123336343    0.516116879453    3.974935090581
H      -1.447230472660   -0.501713117334    2.805396400939
O       1.866325079322   -4.247717391858   -2.233825314409
H       1.553989796501   -4.090938306163   -1.329705391542
H       1.461693515932   -3.579171688352   -2.812846916943
O       0.872169312775   -2.362728853537   -3.931357396604
H       1.088118880726   -1.454099696219   -3.679113040786
H       0.700535771149   -2.335377733462   -4.885028364624
O      -1.436411629872   -4.456946718791   -0.275369487145
H      -1.985631891776   -4.958845125598    0.358387866891
H      -1.205993102103   -5.064431968090   -1.018379817809
O      -3.315837305060    3.442858083099    1.249707270049
H      -3.529984854335    3.138887484196    2.143592334629
H      -3.089409175380    2.647490326384    0.732985743955
C       0.173027677012    1.632248787811    0.844676684570
C       1.236468284453    1.528556713715    0.003725220840
C       1.978670272423    0.315587481950   -0.260546007424
C       1.715689001808   -0.909280103910    0.285244902129
C      -0.268200227568   -1.371004217891   -0.816125833257
C      -1.215661893910   -0.596335246861   -0.187840915667
C      -1.552429387321    0.722495629971   -0.699526018359
O      -2.556214160274    1.404238049947   -0.362936084456
H       1.490898864059    2.381698245480   -0.623525993869
H      -0.367927049497    2.559673978129    0.964442139091
H       2.692734612594    0.371755758205   -1.076177258481
H       2.283053485077   -1.777128778538   -0.022367666157
H       1.173933082588   -1.030493361186    1.212197409501
H       0.124178336051   -1.065225254486   -1.770204896236
H      -0.154179268245   -2.413485123695   -0.564783548166
H      -0.041949339409    0.880141708524    1.588629142085
H      -0.983128110517    1.052900337701   -1.582598033276
H      -2.728461367465   -0.596358965772    1.218201482517
N      -1.823250889404   -1.016977745768    1.017273906867
H      -1.826184660198   -2.018956556925    1.154049950856

Ambimodal TS Water45-9
155
ANGSTROM
O      -4.740576407281   -1.367009159216    0.070814546396
H      -4.306533829568   -1.180834568891   -0.770647849654
H      -5.231561425974   -0.556185656359    0.340353382714
O      -4.944675726064    2.345585321775   -1.357649629508
H      -4.052868415294    1.992895425456   -1.190964895577
H      -4.843687070296    3.299744193797   -1.368264167062
O      -1.599300324942   -3.803683119612    4.905735560767
H      -0.696363415123   -3.450357686207    4.924769756768
```

16743	H	-2.158164877691	-3.035597814332	4.711659762825
16744	O	5.330884180965	-1.326902533540	0.736352068338
16745	H	6.153656866943	-1.281831492970	0.241856020665
16746	H	4.875974282686	-0.495263278899	0.535579776888
16747	O	3.399909470950	1.221198798889	2.476427123041
16748	H	3.943925474344	1.282231394589	1.677217737164
16749	H	3.026984979865	2.120575594261	2.584960898521
16750	O	4.695140867600	1.267300726390	-3.842948226554
16751	H	4.410454252384	1.992212193018	-3.264884273070
16752	H	3.916563297385	0.702145914268	-3.952924045874
16753	O	2.069795761231	-4.386446148150	1.477597764362
16754	H	2.517293897827	-4.226972558734	2.323405468052
16755	H	1.271909964938	-3.809587977113	1.504598962070
16756	O	-4.066339534529	-3.262432533957	1.726229365150
16757	H	-4.318575029327	-2.528581468971	1.105749350995
16758	H	-4.877699429112	-3.747582202983	1.885313449580
16759	O	-3.240844925193	-1.148004611296	-2.366881498064
16760	H	-3.774348600615	-0.441836900265	-2.802629919719
16761	H	-2.365683942704	-1.051042601231	-2.803544966384
16762	O	-0.205601449710	-3.084274036985	1.953807046332
16763	H	-0.780238293517	-3.861903573101	2.101784550444
16764	H	0.078954091185	-2.793957039070	2.846800782228
16765	O	4.117062535568	-2.138984545478	-2.419234632686
16766	H	3.969381533243	-2.684878013797	-1.613601871648
16767	H	4.255096110110	-2.760666438941	-3.134994831069
16768	O	-0.676231381512	-5.140539415755	-1.656690233830
16769	H	-0.436962744480	-5.677510469850	-0.873459927722
16770	H	-1.468701565626	-4.640556970543	-1.392582113828
16771	O	3.517067224065	3.095231592261	-2.077926727616
16772	H	3.548764462156	4.041775979872	-2.215682484624
16773	H	2.658946478792	2.782106355719	-2.434441606584
16774	O	2.128155968266	3.598146487477	2.622710773663
16775	H	1.237844187556	3.467961058980	2.990610486811
16776	H	1.991569005066	4.021566962061	1.763210095689
16777	O	4.018668162608	-3.616160626498	-0.211595383222
16778	H	3.316823791822	-3.946245689941	0.388952786915
16779	H	4.526039174879	-2.964513497697	0.296290359217
16780	O	-2.537494704485	4.069430689307	-0.092054655032
16781	H	-1.926204182933	4.417510314121	-0.755398998053
16782	H	-2.501126901747	3.108931696981	-0.238809592286
16783	O	4.828135825974	1.419645881988	-0.007638643941
16784	H	4.374972094105	2.106367021383	-0.511484696273
16785	H	5.370571301348	0.946857545521	-0.677480955079
16786	O	1.143665816478	5.028624021598	0.354096082752
16787	H	1.517966325241	5.908007108160	0.296787930443
16788	H	0.358595243979	5.110804413821	0.961178746309
16789	O	-1.455995505298	0.536437387865	3.462435967511
16790	H	-2.190040348841	0.940939002066	2.964639147442
16791	H	-1.794481503495	-0.328248423320	3.731560536549
16792	O	-2.375362882955	2.753775663333	-3.502190675217
16793	H	-1.863915786126	3.279475707693	-2.876642181194
16794	H	-1.732431515902	2.454587775431	-4.167120197005
16795	O	2.379223224863	-0.396276240797	-3.549314674712
16796	H	1.822712881764	-0.992984803463	-4.066279347143
16797	H	2.944068213836	-0.988035497196	-3.014328373933
16798	O	-2.900877603954	-3.701343199094	-0.944694381373

16799	H	-3.102950515901	-3.680086072317	-0.003256231645
16800	H	-2.890070461458	-2.786035465701	-1.246342992456
16801	O	-0.968340976713	5.108142299335	1.917139728014
16802	H	-1.668111558665	4.768463169957	1.329671630851
16803	H	-0.868729986396	4.486628486235	2.656706439371
16804	O	3.237315192768	-3.447921090120	3.821283786439
16805	H	3.697768457435	-2.584854916892	3.646258792738
16806	H	3.784602157387	-3.916963177925	4.450949158164
16807	O	5.957148381681	-0.135060703663	-1.897876931489
16808	H	5.387683854062	-0.914430021185	-2.018488981183
16809	H	5.756613846876	0.413982484813	-2.681646985183
16810	O	-1.909026351924	-5.059493855969	2.573600044857
16811	H	-2.734596718250	-4.665348168090	2.273036148127
16812	H	-1.815913897718	-4.800053534157	3.520826224943
16813	O	0.773288894163	-2.457358816171	4.372823433727
16814	H	1.642780665994	-2.889988166398	4.286050259628
16815	H	0.963789366974	-1.500470507147	4.382588913377
16816	O	-0.403258584308	3.147607570789	3.806831757194
16817	H	-0.821795253548	2.278759680421	3.734344987250
16818	H	0.155134474179	3.084542341885	4.609105245675
16819	O	0.731899250568	-2.553415083359	-4.285508937870
16820	H	1.140027557643	-2.910447206066	-3.472779652053
16821	H	0.107209909074	-3.266823032514	-4.543249884543
16822	O	1.237598488103	2.004404589830	-3.056493156716
16823	H	1.612657660178	1.101238884361	-3.085091603612
16824	H	0.759795737925	2.080889091825	-3.897199550277
16825	O	-3.328546037844	-1.081774106329	-5.577045264972
16826	H	-3.900275457591	-0.432106900923	-5.147474595785
16827	H	-3.650411271943	-1.955021290725	-5.320443202836
16828	O	-0.492234492574	1.567793447763	-5.119751884221
16829	H	-0.704476565911	0.694154564527	-4.700188822397
16830	H	-0.582426245299	1.444562451099	-6.064700652451
16831	O	1.282467495281	0.189987829579	3.985973563577
16832	H	1.926651240261	0.448000159596	3.305916826811
16833	H	0.394164329172	0.393906816911	3.654359085269
16834	O	0.025177673976	-6.219150620232	0.725641293083
16835	H	-0.643150269354	-6.020166707256	1.393394856072
16836	H	0.834703852073	-5.760087884357	0.997852554624
16837	O	-3.736339498483	-3.358775094078	-3.878021556585
16838	H	-4.058682318194	-4.086709860329	-3.343726110062
16839	H	-3.617184445191	-2.608883460859	-3.259568010976
16840	O	-1.176767021133	-0.749311006265	-4.038787902381
16841	H	-1.850991108910	-0.953190311325	-4.726250692317
16842	H	-0.462120293787	-1.427945414224	-4.128529272309
16843	O	4.525611933568	-1.200401500324	3.363175753804
16844	H	4.088101463020	-0.339470877625	3.285660931638
16845	H	4.966681169950	-1.331734936983	2.508606108006
16846	O	-4.364360982045	0.883473769919	-3.657266709074
16847	H	-3.631459088955	1.530610610722	-3.686888625035
16848	H	-5.014528172842	1.299519608036	-3.076164205535
16849	O	-5.820962787995	0.926768497157	0.849237078495
16850	H	-5.749932747540	1.480155835942	0.053188373206
16851	H	-5.133558717928	1.247516031782	1.455130029633
16852	O	1.609229317725	-3.761065124594	-2.038848106489
16853	H	0.803277795280	-4.299819961617	-1.898456256957
16854	H	2.312778657747	-4.181050463774	-1.540252059314

```
O     -3.129059739074   -1.731143689401    3.814736749932
H     -3.411823503672   -2.269993057295    3.050253281426
H     -3.921726202344   -1.358333571246    4.202727536391
O     -1.166098752035   -4.423153405405   -4.369935722984
H     -2.064435056265   -4.062590569191   -4.298906760045
H     -0.978416385405   -4.853478037666   -3.522961577463
O     -3.426673295263    1.761793935913    1.999975141117
H     -3.381371975151    2.717441580578    2.077266905699
H     -3.069700798875    1.543363747052    1.107789277747
O     -0.323872572698    3.988349013515   -1.788842313205
H      0.240447262763    4.376600572287   -1.097781017343
H      0.198149618264    3.260926380770   -2.158824982327
O      1.545908098289    2.469075237392    5.519458130798
H      1.539587719418    1.558900284296    5.167696612219
H      2.326513260778    2.890231993560    5.151926709924
C      0.215661224249    1.675866831553    0.838892191886
C      1.326260308681    1.524250949683    0.095405382675
C      2.045074414825    0.267595753422   -0.149599937516
C      1.825968797545   -0.909637483483    0.465607567925
C     -0.270344112317   -1.428655520208   -0.917480275807
C     -1.217230747586   -0.650056410868   -0.310074448461
C     -1.577330672369    0.647682512846   -0.869788634388
O     -2.527978401158    1.364914481364   -0.470619173075
H      1.716311806270    2.388922797446   -0.436441010311
H     -0.269163597159    2.637076397081    0.934532201610
H      2.768224281676    0.306818264852   -0.960759681133
H      2.371747720446   -1.798484259994    0.190029703471
H      1.179941771455   -1.019250214280    1.324916061151
H      0.213563547416   -1.119097272524   -1.827100281598
H     -0.054569194770   -2.428942018294   -0.579494876796
H     -0.193163632711    0.895189973202    1.463064608882
H     -1.052505076573    0.959161509486   -1.785968514494
H     -2.734443153357   -0.667863955686    1.116230542508
N     -1.822294569708   -1.016231609264    0.885379125110
H     -1.526412346254   -1.883600313408    1.308956989329

Ambimodal TS Water45-10
155
ANGSTROM
O      4.154332011260    3.505030752419    0.685219520207
H      3.653474988081    4.038733504820    0.058655743598
H      3.533648263143    3.268745547885    1.391075173418
O     -3.693848592156    3.279731658766    1.109391598193
H     -3.266714107613    2.535867159300    0.656844100802
H     -3.582685909293    3.120576525382    2.084183611333
O     -5.762840364309    1.322521691038    3.522537209158
H     -5.105229698890    1.970007162110    3.799825511927
H     -6.098143080436    1.633540235725    2.654698097890
O      0.327810021359    1.846719001743   -3.090141802614
H      0.502084307918    2.794100894884   -3.168331200207
H      1.144134450369    1.372623326883   -3.333033854515
O     -0.053266742820    1.851447990162    4.821101592481
H     -0.882370391420    1.706551211133    5.286078451841
H     -0.007483690474    1.138366887513    4.168713169845
O      2.121304145727   -3.326106081783   -2.783691698592
H      2.776856972509   -2.636904069864   -2.573747401506
```

16911	H	2.493013027118	-3.844779294259	-3.499165619639
16912	O	1.182985764294	-1.647714561515	5.709821964048
16913	H	1.997252250084	-2.125616484416	5.466472952422
16914	H	1.514530465820	-0.781637101947	6.036734173350
16915	O	2.175675107541	3.139729654572	2.643344169129
16916	H	1.563348183157	3.669982505828	2.094809423386
16917	H	2.383309400978	3.670207028729	3.414718417006
16918	O	4.018865765017	-1.775829779743	2.467098346359
16919	H	3.406322258381	-2.473571469367	2.119645853599
16920	H	3.498194700578	-0.965274813189	2.633886486695
16921	O	-1.642247251951	0.849851101264	-4.551377227027
16922	H	-2.406897007787	1.438554755377	-4.495512568364
16923	H	-0.930345120093	1.274452675746	-4.015560840160
16924	O	-0.544637802390	-3.204843815084	-3.671802528232
16925	H	0.309233933101	-3.266664980923	-3.216510245912
16926	H	-1.107864339734	-3.905226251134	-3.304532693648
16927	O	1.321848856336	-4.882161245322	-0.587743111659
16928	H	1.617771583005	-4.338338152767	-1.330362719600
16929	H	1.831330931172	-4.594774113715	0.187772356226
16930	O	5.176467374299	1.542916558649	-0.929736523824
16931	H	4.956159579401	2.234671174104	-0.278199004826
16932	H	5.121882254407	0.701736448475	-0.458053339350
16933	O	-3.841765784379	2.479245637036	-3.825435670892
16934	H	-3.386444846718	2.991315223055	-3.129608004482
16935	H	-4.492840514977	3.059376439501	-4.220747314417
16936	O	-4.915324503458	0.021682576634	-2.900256759645
16937	H	-4.192388083581	-0.579262532913	-3.130870984856
16938	H	-4.629530642242	0.893348839719	-3.213473911379
16939	O	-2.390211289842	3.706268803060	-1.871351946464
16940	H	-2.386719059702	2.854906744403	-1.384586702435
16941	H	-2.389541732581	4.384385277712	-1.175582672448
16942	O	-0.848662669189	4.207166764833	3.607172942752
16943	H	-0.346676777518	4.491318555200	2.836684740716
16944	H	-0.362255614614	3.470348833706	4.013227079079
16945	O	4.073805057920	-1.446805929858	-2.324757897429
16946	H	4.810443580308	-1.170922619401	-2.874005953375
16947	H	4.377284765505	-1.333477820577	-1.401646030559
16948	O	2.595738105501	0.422834029725	3.179823196611
16949	H	1.687182494494	0.058577626426	3.168646964252
16950	H	2.524634143329	1.333616013110	2.859506997577
16951	O	0.448830352200	-1.017951123704	-5.216826002671
16952	H	0.094182504046	-1.830641201162	-4.833706263152
16953	H	-0.285105929556	-0.390458539038	-5.242210069127
16954	O	-1.178312504860	-5.926888661918	-0.309655581864
16955	H	-1.565232182713	-5.732701305406	-1.174154503866
16956	H	-0.239902995197	-5.668458437938	-0.373510638036
16957	O	-2.352039823028	0.343605093371	3.929169046134
16958	H	-3.101222700202	-0.157485072724	3.573561780594
16959	H	-1.557370455107	-0.161944462515	3.692004895838
16960	O	-2.445533788190	-1.280810826230	-3.164798637241
16961	H	-2.150554261012	-0.482899644771	-3.660045984881
16962	H	-1.805723315783	-1.971676950839	-3.408845481786
16963	O	3.478106558894	2.693658462517	-2.748755985984
16964	H	3.178239654111	1.968636374487	-3.309633093595
16965	H	4.171713720036	2.315029785731	-2.178101802412
16966	O	2.434098803970	-3.769224764698	1.702202705519

16967	H	2.527826823929	-4.400615178111	2.443120214859
16968	H	1.515186940594	-3.437726500370	1.822139831286
16969	O	0.148062999852	-0.697425206529	3.474343743093
16970	H	0.448720872269	-1.070007363495	4.342106947597
16971	H	-0.020297798651	-1.489368467653	2.918491549369
16972	O	-0.038716141628	-3.083585517922	2.308336351156
16973	H	-0.773643504848	-3.461994743356	1.790063435920
16974	H	-0.132739467674	-3.476378666203	3.205689358298
16975	O	-4.498562676967	-0.818363748425	2.602607218163
16976	H	-5.058008443144	-0.134332095292	3.045612018009
16977	H	-4.778136088846	-0.802226779729	1.679640195967
16978	O	-2.272183103793	-3.956695837422	1.037409673161
16979	H	-1.917825535340	-4.791421438662	0.639623280197
16980	H	-2.777761649124	-3.528522493654	0.327052180453
16981	O	-2.047854494178	5.202191934970	0.419301552578
16982	H	-2.683994635605	4.529787809524	0.784268384765
16983	H	-2.326722784096	6.049091132210	0.767621954647
16984	O	-2.537028423544	-4.744206207694	-2.480379923259
16985	H	-2.976628628505	-4.059760532301	-1.946599397686
16986	H	-3.215799323634	-5.139991376552	-3.028079706114
16987	O	2.168703270134	-4.953888644003	4.113112667518
16988	H	1.264983713518	-4.843477276334	4.431217713162
16989	H	2.707599545381	-4.310980371059	4.595262564471
16990	O	-0.095731575598	4.591239627952	-3.010787498150
16991	H	-0.923651929467	4.251068990710	-2.607362002100
16992	H	-0.348847594152	5.149190703896	-3.745656781450
16993	O	-3.547777442382	-2.577750551205	-1.038966067408
16994	H	-3.113549355080	-2.059836565504	-1.742285608785
16995	H	-4.177742300104	-1.957577125121	-0.639050338175
16996	O	2.274095609414	0.780543404833	5.913371869803
16997	H	1.517487827377	1.371479350743	5.783469214269
16998	H	2.664802595762	0.681657157081	5.027122366814
16999	O	-3.229535203047	2.865986716543	3.641800137678
17000	H	-2.469295971959	3.467320099909	3.743472270495
17001	H	-2.879274942179	1.961366356004	3.774825552767
17002	O	-2.814605267949	-2.938440563360	3.604847249534
17003	H	-3.525033897469	-2.302654078414	3.451174070294
17004	H	-2.790145760555	-3.493316776514	2.812483613469
17005	O	5.178650311120	-1.109873513848	0.153156756335
17006	H	6.102558552411	-1.351633983822	0.228626909891
17007	H	4.762578465964	-1.346274850910	1.017325355002
17008	O	-0.458318870913	-3.774402163552	4.844008331545
17009	H	-0.087091191697	-3.067029504082	5.389932271786
17010	H	-1.394561551234	-3.557563891834	4.703786157448
17011	O	-4.776681329415	-0.150284246363	-0.242076158715
17012	H	-5.060713059436	-0.112949259609	-1.182347793572
17013	H	-3.971026715222	0.401619967572	-0.234510709010
17014	O	-6.164189567050	2.015813787855	0.940395411554
17015	H	-5.422728219327	2.641652928385	0.889310431869
17016	H	-5.866814144528	1.211083762656	0.486852568437
17017	O	2.090381359060	4.505809867353	-1.263317047461
17018	H	2.641293043508	3.898657056989	-1.799508844432
17019	H	1.362156544794	4.738166229527	-1.856270750314
17020	O	3.610183416095	-2.699728177968	4.972189736640
17021	H	4.326625069672	-2.490092501653	5.571256833485
17022	H	3.873147873120	-2.364424495875	4.084034869446

```
O      2.401122998407    0.282625058129   -3.861187568138
H      1.797762360045   -0.226004511090   -4.442129042373
H      2.831198188578   -0.356455982651   -3.276721954998
O      0.628283422539    4.763979184116    1.094638901180
H     -0.273002654244    4.935437748034    0.769958638859
H      1.190003111506    4.751680206954    0.299477846711
C      0.101744488389    1.577122523686    0.898339580754
C      1.186345585911    1.539979934714    0.079214824449
C      1.970316004500    0.363816181201   -0.246138919485
C      1.763726884136   -0.885765256406    0.252282117169
C     -0.307758298345   -1.485455352873   -0.783456029206
C     -1.226485103739   -0.657136752210   -0.178038912611
C     -1.493430228213    0.667258705338   -0.738361510871
O     -2.504357848696    1.372433938858   -0.455025554316
H      1.434158458713    2.433058337662   -0.488614979025
H     -0.458805839354    2.490772622411    1.041682415959
H      2.685237554453    0.495230948296   -1.053744548615
H      2.339908971363   -1.732234984492   -0.093848582643
H      1.198376447687   -1.063840742624    1.153466780474
H      0.095002553829   -1.229740650874   -1.748738067956
H     -0.183539903259   -2.513350519995   -0.475829389610
H     -0.146134710564    0.787936230062    1.593174120759
H     -0.891219861438    0.967935691577   -1.602516794293
H     -2.626061848610   -0.387579663190    1.312249996013
N     -1.852338071459   -0.962552939659    1.021630990886
H     -1.922889192431   -1.922041276553    1.311983143993

Ambimodal TS Water45-11
155
ANGSTROM
O     -4.711507006397    2.016387029032    1.205748272179
H     -4.446103773863    2.853246206375    1.597690530283
H     -3.874411885106    1.590284807028    0.949216674446
O      0.137163306208   -2.385075691635   -4.010182406499
H      0.864285270519   -2.607370516706   -3.411332165629
H      0.533246461781   -1.777855414554   -4.657396517595
O     -1.422345698381    3.471579496719   -3.268996518941
H     -1.626301329146    3.773192932713   -2.368813847777
H     -2.240700321543    3.035336700885   -3.576090035922
O      4.094810464620   -3.312012873546   -0.115280793725
H      4.766847643522   -3.993741239823   -0.150652716156
H      3.380815847698   -3.653162093391    0.467220619428
O     -0.317349973554   -0.883344211088    3.334273739179
H     -0.933403871023   -0.157870780395    3.147818576719
H      0.580106837739   -0.495458794852    3.400345205732
O     -3.383148564520   -3.580812565887   -1.322955852913
H     -3.555555470156   -2.655567839016   -1.556492769926
H     -3.994465409910   -3.789188521076   -0.600008537062
O      3.144612309438   -2.606153248329    3.547501279632
H      2.598837706634   -3.060451694922    4.243842416699
H      2.971592090336   -3.102855266966    2.741182902990
O     -0.166857138867    2.261142601046    4.828748863711
H     -0.384958186443    3.193974443348    4.881798841796
H     -0.857640816180    1.861768554485    4.254749943932
O     -1.962219476747    1.316757630987    3.077178308441
H     -2.802144655098    0.828772464827    3.137387003169
```

17079	H	-2.157477805006	2.158997654964	2.607393009080
17080	O	5.688609177721	0.337279303478	-1.695936505616
17081	H	5.622792877681	-0.156584971714	-0.861695568029
17082	H	5.103982579812	-0.097898856221	-2.326989496916
17083	O	1.373099706197	0.447055921286	6.253281753171
17084	H	2.160842426675	0.878277203508	6.642902595030
17085	H	0.773183565744	1.156243137185	5.976512994901
17086	O	-0.246659357673	-1.792677384537	5.930279098797
17087	H	-0.428407144953	-1.482272638530	5.024781168729
17088	H	0.285206026229	-1.093350513784	6.332093361018
17089	O	4.405587905550	3.631671090669	3.365126877065
17090	H	3.449830538180	3.388587580361	3.322585649583
17091	H	4.476393275346	4.364869724314	3.977182414541
17092	O	2.883250338690	2.305509920622	-2.956473108554
17093	H	3.435805732424	2.482127092637	-2.148952418954
17094	H	3.170698630264	2.935015201292	-3.618234255022
17095	O	-4.602189772454	-3.834518007862	1.156841071452
17096	H	-3.960874646011	-3.599305989211	1.864847837577
17097	H	-5.127320726882	-4.554686609017	1.505355554146
17098	O	4.023403971079	4.825193566570	0.760399946314
17099	H	4.231432854984	4.408268549666	1.609062521301
17100	H	4.312487087206	4.202819235027	0.077651737692
17101	O	2.506611905640	-2.716299613549	-2.374194811639
17102	H	2.814932947528	-1.870949913948	-2.746752077612
17103	H	3.153299247212	-2.929076756074	-1.684316182483
17104	O	2.226996580288	-0.113401603750	3.791044380715
17105	H	1.964893638514	-0.029605135088	4.737845246289
17106	H	2.585117770124	-1.022063311711	3.676589913960
17107	O	5.145382909790	-0.890496222195	0.750559546638
17108	H	5.479234513910	-1.093074716140	1.655642234347
17109	H	4.720004804676	-1.707378908398	0.443191484850
17110	O	-2.725049640138	3.852653858576	-0.802037272120
17111	H	-2.685853375752	2.875954848305	-0.634019290954
17112	H	-3.604478063338	4.025385588004	-1.141470062692
17113	O	0.072765712773	4.660340770717	2.570643254679
17114	H	0.711243010653	3.968295346393	2.803078141183
17115	H	0.409861505657	4.989290156854	1.716999157411
17116	O	3.999677532480	1.430142511701	1.481023599998
17117	H	4.341964064616	0.544397105120	1.233456978279
17118	H	4.698425709157	1.864613357682	1.977510583716
17119	O	1.386035943582	5.189670958648	0.243408087890
17120	H	2.333161211118	5.049679070443	0.459404667494
17121	H	1.342487668210	6.006929240835	-0.252465309220
17122	O	-3.119076299975	-2.936736925412	3.195922082097
17123	H	-2.215568827809	-3.052335042934	3.503504995506
17124	H	-3.349175499525	-2.000822179228	3.310211938679
17125	O	1.953183581505	2.663538522995	3.169245727947
17126	H	1.337553121206	2.297973268951	3.832257353696
17127	H	2.209498791351	1.942537741862	2.577821733938
17128	O	-1.836138447564	-0.427412923344	-3.658505537201
17129	H	-1.333998994448	-1.247357705409	-3.798554944442
17130	H	-1.156902817465	0.256039324265	-3.551275495615
17131	O	4.598964666334	1.052392121924	4.416548221159
17132	H	3.744691152413	0.720372669183	4.058267313312
17133	H	4.697139628046	1.956580980758	4.077195605761
17134	O	3.754056845953	1.499530522606	6.930031460269

17135	H	4.276857023100	1.010543415742	7.565706154512
17136	H	4.161527255964	1.335074043819	6.049459851361
17137	O	3.372599472227	-0.345522274722	-3.512201215316
17138	H	3.196060884386	0.583049972533	-3.267553520113
17139	H	2.885389387536	-0.471985952593	-4.336212202609
17140	O	4.494267746115	2.597660155585	-0.897681768924
17141	H	4.145357116381	2.198277198528	-0.070794175653
17142	H	5.122104471014	1.922783190094	-1.236067307114
17143	O	-1.363309804712	-4.109868624607	0.471237980946
17144	H	-0.601236347264	-4.418659312379	-0.044764646780
17145	H	-2.091832743585	-4.029796162961	-0.168074324413
17146	O	-4.144064292297	-0.325184243318	3.075176809476
17147	H	-4.877608216785	-0.009056260389	3.602675146599
17148	H	-4.524218809714	-0.516188557165	2.180861586550
17149	O	1.402188918616	-3.767544902331	5.129802734113
17150	H	0.843733911351	-3.103961185218	5.590135472785
17151	H	0.837250311759	-4.097377969194	4.419660338084
17152	O	1.210525909548	-0.218959422172	-5.365336944784
17153	H	0.912522123875	0.050846985675	-6.234009278667
17154	H	0.834682804191	0.429250032953	-4.726708376105
17155	O	-1.252066918978	-4.622625406027	-2.929140377803
17156	H	-2.062008520747	-4.324615390539	-2.492460445146
17157	H	-0.946588673360	-3.890278092399	-3.480946344282
17158	O	-0.126498339043	-3.549056423429	2.784825345146
17159	H	-0.711947183920	-3.811618028644	2.053757902909
17160	H	-0.190467476178	-2.580309845761	2.867141494991
17161	O	2.037978033907	-4.235773817467	1.314026560927
17162	H	1.289738721687	-3.991894272160	1.902732547712
17163	H	1.617703404457	-4.630864361177	0.536773532644
17164	O	0.790815109240	-4.681619025301	-1.187570939457
17165	H	1.394446166443	-4.067054120690	-1.627920175374
17166	H	0.101383129772	-4.873411032911	-1.859638478856
17167	O	0.279154743973	1.459546948072	-3.481483859774
17168	H	-0.298308625868	2.263975871813	-3.474582520437
17169	H	1.137275483567	1.763876961997	-3.146587667369
17170	O	-3.612343262528	1.915527392827	-3.725058292207
17171	H	-3.212457432239	1.050848773353	-3.875629957549
17172	H	-4.189630595991	1.832106019646	-2.953648239521
17173	O	-5.040914867953	-1.156306861206	0.729291917780
17174	H	-5.048330033410	-2.118888224425	0.858806706136
17175	H	-4.547099336101	-1.005188661594	-0.088294961978
17176	O	-2.413263238267	3.749748885034	2.004543089114
17177	H	-1.569183812239	4.162250144862	2.287235507159
17178	H	-2.465342949304	3.923738365008	1.050251133754
17179	O	5.613939881548	-1.347607030816	3.327923171270
17180	H	4.841065599512	-1.902634837800	3.524670568101
17181	H	5.461220170181	-0.512233925237	3.794856576103
17182	O	-5.274610769898	1.481646322482	-1.427988236326
17183	H	-5.108357849897	1.699315182775	-0.484543907470
17184	H	-6.203264987739	1.252922517860	-1.484061528862
17185	O	-3.832030795592	-0.873790957070	-1.822602852736
17186	H	-3.120388685741	-0.736934308280	-2.474616059147
17187	H	-4.331862107463	-0.038994219464	-1.789834117584
17188	C	0.216724831258	1.768489682008	0.701948198822
17189	C	1.322093076829	1.584801254553	-0.067309222270
17190	C	2.042408540297	0.340891689140	-0.225414805931

```
C      1.691697448063   -0.844541324893    0.357350600975
C     -0.157454197773   -1.308861563112   -0.949405816691
C     -1.161515910741   -0.642619026418   -0.280163006451
C     -1.537285347549    0.722994929492   -0.638048467132
O     -2.536716646857    1.317442143446   -0.164593531079
H      1.634982446410    2.412683841574   -0.700340284585
H     -0.282367545864    2.725960278049    0.716896233946
H      2.813613007727    0.325374517705   -0.990923537524
H      2.248848691329   -1.744796668287    0.152978408138
H      1.059115318026   -0.899243358759    1.230591805254
H      0.271633557781   -0.887103739086   -1.842018547788
H      0.001495923722   -2.364832480403   -0.806510077417
H     -0.093341287788    1.075063399818    1.468608757706
H     -1.053050339562    1.155234228366   -1.524815127467
H     -2.612432929343   -0.725571388856    1.169173577686
N     -1.790243577020   -1.191370156297    0.825340730097
H     -1.767448622947   -2.193114705092    0.940677445979

Ambimodal TS Water45-12
155
ANGSTROM
O     -2.257935523681   -1.663529257755   -2.679031001392
H     -2.352634494676   -2.610231887231   -2.905238155507
H     -2.693829073771   -1.154209504410   -3.397778486103
O      2.421985567092   -4.107302305682    1.431022139187
H      2.781645478957   -4.911109246689    1.810325875155
H      1.680205144047   -4.386279494369    0.849946446891
O      3.443337770841    4.561224788836   -3.671456005083
H      3.706096920649    4.791244569096   -2.745708211948
H      4.169414812374    4.830728364576   -4.233879798093
O     -0.051561239117   -2.181281849287   -4.183361568794
H     -0.718419106769   -1.821305598859   -3.566984979198
H      0.231318988449   -3.028476178122   -3.797359090780
O      2.786227810008   -0.225037187406    3.698050565177
H      3.095076391655    0.484062079704    3.095217579257
H      3.277890226075   -0.113276626348    4.514366393115
O      0.267817738671    0.734720338032   -6.805005860825
H      0.972722592977    0.393918687819   -6.235578459221
H     -0.117893428826    1.488537729174   -6.336859407294
O     -2.062335217987   -4.555773480112    0.404228515360
H     -1.930583157970   -4.299345551004    1.333014164597
H     -2.659433025431   -3.860855566031    0.044662090710
O      1.451251329233    5.318415697469   -0.312646105393
H      1.007254761376    4.628648227933   -0.864175817403
H      1.332468095287    6.145215526076   -0.784574988043
O     -3.392921598518    2.486665946704    1.782693577455
H     -2.990323716806    1.996142145230    1.041523086179
H     -2.696038609627    2.616467192397    2.459550038219
O      0.592015582828    1.338517393328   -3.229033531496
H      1.016623809767    0.607139332212   -3.732082557947
H      0.078113021991    1.811569687534   -3.922915484125
O      4.614708207732    2.199569374885   -0.439594632139
H      5.182162276144    1.421379005051   -0.503229550106
H      4.006749226299    2.126064834371   -1.206069415907
O      4.430306996332   -3.321785491136   -0.409764609591
H      3.764114297423   -3.499936183255    0.271511203899
```

17247	H	4.654794009664	-2.383993002441	-0.364785677524
17248	O	4.108960216650	4.902982613411	-1.112401180500
17249	H	4.479954086256	4.070773721476	-0.784480652860
17250	H	3.267284744126	5.022870480478	-0.645410119801
17251	O	-5.456659975904	-1.057299790152	-3.428033803928
17252	H	-5.394604191414	-0.378967203055	-2.746356921126
17253	H	-4.779578619158	-0.824488993761	-4.084207402814
17254	O	-3.698167109047	-2.682778776846	-0.603908024007
17255	H	-4.307449687022	-3.142653504243	-1.233922811238
17256	H	-3.135598081035	-2.134012197183	-1.182705946948
17257	O	4.013019741909	-0.546774494028	-2.940323065353
17258	H	4.602903267667	-0.649208580869	-2.184040349762
17259	H	3.735716094786	0.387106636962	-2.926864053745
17260	O	-2.158359790169	-4.379147414938	-2.864174298005
17261	H	-2.315244885520	-4.714309991362	-1.975991911048
17262	H	-1.218738053115	-4.523793229765	-3.046916489004
17263	O	-5.084438744145	-3.620316267807	-2.630145085998
17264	H	-4.431896301357	-3.981552768831	-3.236385401043
17265	H	-5.345682714692	-2.742731452909	-2.981851711581
17266	O	0.098327588631	3.673063300191	-1.843438740195
17267	H	-0.867672606429	3.830478269103	-1.786599827923
17268	H	0.225272195631	2.749367274477	-2.110764277977
17269	O	0.498881257735	1.234875269365	4.312064261362
17270	H	1.241265372527	0.646043771147	4.099481903852
17271	H	-0.280414249724	0.646259055179	4.317063655647
17272	O	-1.412401490883	3.128912600583	3.474754551927
17273	H	-0.793621030953	2.492851711423	3.859363071065
17274	H	-0.883305280389	3.815825670517	3.055408425316
17275	O	3.910299063150	1.792426441080	2.236572345193
17276	H	4.062696135996	2.021373319646	1.302052015970
17277	H	3.320730900784	2.487268350093	2.593701055659
17278	O	0.700483018056	-4.408290953223	-2.811130391048
17279	H	1.619216193218	-4.093806854526	-2.930478103430
17280	H	0.629776885552	-4.569997672159	-1.855387298013
17281	O	-1.631706185317	-0.372083113003	4.057828986117
17282	H	-2.412496442984	-0.121786384742	3.543570208616
17283	H	-1.923205386530	-1.070189379810	4.647255225877
17284	O	-4.086350515436	-0.015663744853	2.645519210469
17285	H	-4.643990535269	-0.340670146842	1.897846802418
17286	H	-4.066353538214	0.950764540294	2.530983719938
17287	O	0.424967985946	-5.069730297942	-0.133752420088
17288	H	0.451019889020	-6.027686278649	-0.144359175747
17289	H	-0.526972434588	-4.829897286110	0.058314271236
17290	O	-3.196788111237	-0.272346204379	-4.755373535741
17291	H	-2.622720500617	-0.656643304675	-5.473998860045
17292	H	-2.994228396922	0.666531885789	-4.708550779728
17293	O	-5.341848465448	-0.840969198720	0.469502267688
17294	H	-4.800484149830	-1.595546005409	0.156551787477
17295	H	-5.335924404154	-0.206004413319	-0.258979917282
17296	O	1.827771320092	-0.431256170944	-4.776172272199
17297	H	2.690522356764	-0.686771879289	-4.435516337675
17298	H	1.218336465685	-1.188274229632	-4.623391389362
17299	O	0.038803161897	5.085177972033	1.945133503540
17300	H	0.154274391918	5.898556441379	2.439646195336
17301	H	0.584458849127	5.170163008554	1.128802686304
17302	O	-1.452612668287	-3.412694627556	2.801193649323

17303	H	-2.165222255824	-3.096597228799	3.381103925176
17304	H	-0.604207554077	-3.182124338884	3.217413342177
17305	O	3.116233474919	2.038179835543	-2.676736154213
17306	H	2.165314932298	1.827138756771	-2.800995184581
17307	H	3.268112793666	2.854097987879	-3.184221220658
17308	O	0.639897851997	4.864648347069	-4.226181583489
17309	H	1.600020303135	4.818726826148	-4.317244980027
17310	H	0.465152611823	4.529518768668	-3.326779041504
17311	O	-1.470388896100	-1.300708199102	-6.420732658254
17312	H	-0.931437667813	-1.806477079421	-5.792995501409
17313	H	-0.888392727440	-0.575882583075	-6.732652140230
17314	O	5.273501604244	-0.584306387613	2.108464503557
17315	H	5.018196341609	0.341007802210	2.258630387445
17316	H	4.535635547112	-1.111711623569	2.430517110793
17317	O	-4.801035769930	1.121603180229	-1.575018498903
17318	H	-5.273775982614	1.877593641895	-1.222267251858
17319	H	-3.913664796207	1.135997721533	-1.153097562893
17320	O	-3.139709636423	2.426380800764	-3.568422222052
17321	H	-2.915102951495	3.091743039864	-2.899867774519
17322	H	-3.786158318424	1.851497283188	-3.136634147546
17323	O	1.140646524693	-2.789161044429	3.592754017871
17324	H	1.555981839035	-1.927074968161	3.698367953602
17325	H	1.599722911396	-3.215635815369	2.851977392894
17326	O	3.128489819620	-3.239163126155	-2.825692978325
17327	H	3.346923355602	-2.314249977642	-3.002042687076
17328	H	3.631208495577	-3.462789779165	-2.023153756040
17329	O	-2.460650739092	4.420984146292	-1.752917314223
17330	H	-2.623981161879	5.259112280457	-2.187328629769
17331	H	-2.592094254124	4.578939409984	-0.778065139963
17332	O	-3.704321103443	-2.379068096193	4.064774943941
17333	H	-4.444220346789	-2.976627017774	3.951686179097
17334	H	-3.944033480203	-1.561183600397	3.595102028572
17335	O	-0.780321888379	2.698706789950	-5.067199514451
17336	H	-0.371703492328	3.580454243230	-4.956071066076
17337	H	-1.674906980780	2.733476739215	-4.698512397520
17338	O	-2.645373289993	4.889918990875	0.806289793852
17339	H	-3.087399454235	4.107015418163	1.201805456104
17340	H	-1.784437601443	4.956357431669	1.241604131465
17341	O	5.574057286209	-0.588509475556	-0.521324744520
17342	H	6.466496608493	-0.880324813934	-0.714207062075
17343	H	5.488640373894	-0.601332819148	0.463712511706
17344	O	1.977724237903	3.372893862230	3.262235221806
17345	H	1.355383315623	3.874707595331	2.720929444097
17346	H	1.452212161749	2.706697681450	3.732597203462
17347	C	0.196683208835	1.692185695035	0.902277652892
17348	C	1.377783976941	1.473440029957	0.276986729602
17349	C	2.117264269132	0.222897483680	0.265383514913
17350	C	1.716988465333	-0.917991928275	0.890851673842
17351	C	-0.043409441375	-1.574030054363	-0.522075623209
17352	C	-1.074529663095	-0.792804223555	-0.037771506254
17353	C	-1.427843828029	0.472518535912	-0.688775098488
17354	O	-2.436126592428	1.159559972947	-0.404293210066
17355	H	1.782972496303	2.262026469502	-0.354946912623
17356	H	-0.316050344455	2.639967468016	0.810412914321
17357	H	2.959785652780	0.173204545450	-0.418762111152
17358	H	2.287014940637	-1.831376234500	0.810168688642

```
H      0.986223372513   -0.912027322255    1.686730866321
H      0.452200706371   -1.316789196793   -1.443370189537
H      0.097863338389   -2.585607747095   -0.175974904432
H     -0.215359149327    1.021585464878    1.642129323413
H     -0.858082267069    0.735078771274   -1.593193241567
H     -2.585763545153   -0.591301637744    1.359830090860
N     -1.765254756544   -1.114366955084    1.101641343693
H     -1.625714234089   -1.996088759054    1.575755043723

Ambimodal TS Water45-13
155
ANGSTROM
O     -1.084236691494   -3.728418152429   -3.000217487154
H     -0.254355104370   -3.565460307983   -3.501406197579
H     -1.777465292568   -3.481103201017   -3.633025818051
O     -5.739573867213   -0.534049927645    0.989870917349
H     -5.347099105532    0.365351982257    1.128622226193
H     -6.632073408460   -0.500574114752    1.334793869059
O     -4.622101243961    1.851155493211    1.104382288638
H     -3.829601150264    1.632170147996    0.569016033170
H     -5.194741909316    2.351859542460    0.502633352684
O     -2.435859933566    4.943506127037    0.453045940815
H     -1.503106500406    5.166272168148    0.354198336429
H     -2.485600498888    4.292355138428    1.166673167961
O      5.269175643064   -0.323260497278    1.332196584696
H      5.943835028506   -0.066878839762    1.962968986367
H      4.765300179325   -1.046007082654    1.747840699322
O     -2.015480957679    0.348962151041    3.571673030428
H     -2.831216495949   -0.181625422960    3.539920553337
H     -1.320471095827   -0.294078278885    3.343846785711
O     -2.943939364370   -2.471692382036   -4.633973867359
H     -3.071259409889   -1.787269653073   -3.932425120818
H     -3.808597189825   -2.836899216405   -4.820679733105
O      3.869605383304   -2.365186630450    2.456164053222
H      3.644980985934   -2.813853178138    3.307635186525
H      3.488201138935   -2.906619046370    1.754998742311
O     -2.468958775850    2.875614792277    2.515116168719
H     -3.384952608021    2.698945324224    2.254467402000
H     -2.179872011524    2.038995410170    2.923588935464
O     -0.171003544052    1.091697013097    5.603559555854
H      0.331894929642    1.829678805987    5.220206531516
H     -0.911621335916    0.927204110682    4.999314849917
O     -0.820471718424   -5.350448591156    1.309792252324
H     -1.618331222427   -4.789140426804    1.299421872550
H     -0.438981786271   -5.321070892177    0.420950913061
O      0.479967943723    0.557974377531   -6.119948023792
H      1.138972107380    0.455054182376   -6.807625429525
H      0.951240532973    0.836485274469   -5.321073700166
O      0.937327293475   -3.966522313142    2.698161638817
H      1.569349464180   -3.911732039653    1.964590253285
H      0.235011642051   -4.571636329572    2.360236160126
O      1.183326038110    3.314580176068    4.576901175649
H      1.315832378693    3.915300781963    5.310791972206
H      0.629851701065    3.801693430910    3.917533530366
O      3.354589842278    2.232467275410    3.261318730015
H      3.022194156943    1.316621455830    3.267943209473
```

17415	H	2.651333676456	2.737091235992	3.699888794586
17416	O	-3.046197771343	-3.723874000325	1.314976131494
17417	H	-3.093102197359	-3.394398832143	0.382062655814
17418	H	-3.796837331860	-4.309726194539	1.422106982536
17419	O	1.049393140531	-3.188961679298	-4.534898659408
17420	H	1.478973022766	-2.389363287585	-4.178150766772
17421	H	0.504518609626	-2.885302591196	-5.291802974889
17422	O	-1.604080210339	1.146060463934	-4.189745054380
17423	H	-1.453268708205	0.949540816685	-5.118732233213
17424	H	-1.858178942108	2.110478015548	-4.149147440190
17425	O	2.625394218768	-4.084192434664	0.438253434834
17426	H	3.070459075713	-4.932441631172	0.499751922556
17427	H	1.821094352568	-4.259104051786	-0.114063344301
17428	O	-5.530186006255	0.179293678897	-1.745564150533
17429	H	-5.697850542592	1.121336270164	-1.584016561096
17430	H	-5.592012676702	-0.256370608134	-0.880594908895
17431	O	2.459043409882	-5.010066055645	-3.016722461687
17432	H	3.096552936403	-4.331622004641	-2.749597535404
17433	H	1.965224281417	-4.601701802441	-3.744842795416
17434	O	2.276776948141	-1.044822005170	-3.352496249054
17435	H	2.990673876176	-0.616260579329	-3.863431515120
17436	H	2.735874813843	-1.648934547388	-2.733001468343
17437	O	-3.948257828241	-1.512218263043	2.902694877364
17438	H	-4.605340927150	-1.243742166101	2.240533714796
17439	H	-3.525449247377	-2.298743709458	2.528664959450
17440	O	4.434347408852	2.321347396222	0.844849211218
17441	H	4.062362498943	2.453313664914	1.746697689287
17442	H	4.579137489287	1.362623475573	0.816426041946
17443	O	0.369676617018	3.932920735387	-2.624390107463
17444	H	1.083743223834	4.393659050957	-3.070424376055
17445	H	0.531569939147	2.982983575220	-2.756728178744
17446	O	-3.443853090794	3.637058430827	-1.630749717309
17447	H	-3.149602629234	4.283032128841	-0.950477492363
17448	H	-3.052669439802	2.788922972793	-1.313888592396
17449	O	-3.190520416352	-0.624444596463	-2.745902385669
17450	H	-4.064078641778	-0.271335305229	-2.464005462866
17451	H	-2.711724674660	0.092854364772	-3.191461358103
17452	O	5.631480128126	-0.989971382710	-1.328562066040
17453	H	5.601342748359	-0.881222458601	-0.365146439809
17454	H	5.613341239452	-0.076738722974	-1.666565252005
17455	O	2.325277177787	-0.304599290357	3.400794049562
17456	H	2.156284799236	-0.537336927826	4.334448618795
17457	H	2.933353504994	-0.975380160388	3.043682095594
17458	O	-5.901372212104	2.890216736389	-1.090667879825
17459	H	-5.022151496694	3.247998458751	-1.382948383361
17460	H	-6.569482258967	3.483196797708	-1.432586690012
17461	O	1.127667534704	-1.237035081274	5.645156420697
17462	H	0.403019184486	-1.721517628653	5.233473560863
17463	H	0.732892555113	-0.352739803685	5.829621587474
17464	O	3.238266334152	2.849000852426	-3.000337912617
17465	H	2.450397430044	2.288434406013	-3.067648294101
17466	H	3.135212826826	3.382312626934	-2.187489579369
17467	O	3.748496224408	-2.782371162914	-1.878615812126
17468	H	3.527536847356	-3.154628127075	-1.014072126549
17469	H	4.521006721387	-2.189093680069	-1.738401574651
17470	O	5.455500335345	1.681924885639	-1.868823292615

```
H      4.708368530105    2.114102256763   -2.320252049396
H      5.448649059919    2.025705073455   -0.969427981316
O      2.785825042324   -3.379655098114    4.650277374312
H      2.040391726918   -3.771688826862    4.164479259492
H      2.401930542266   -2.710901323835    5.232408690587
O      0.935108172411    1.301861363738   -3.364113334623
H      1.327104768440    0.421807277559   -3.199353588793
H     -0.006695916159    1.149770568149   -3.611842724179
O      0.606020663960   -4.831907861611   -1.114418717696
H      1.265766008293   -5.097798709748   -1.799827012958
H     -0.108444926363   -4.442217769453   -1.642513452611
O     -0.661878667205   -2.032061865887   -6.231468035574
H     -1.501594068040   -2.134848006977   -5.758324889807
H     -0.412362090381   -1.097841726367   -6.181022749819
O     -3.138102169396   -3.047927649530   -1.239868089608
H     -2.377519287172   -3.382527950429   -1.737906806221
H     -3.243015670555   -2.132964836661   -1.530424700476
O     -0.096628229531   -1.490112758014    2.992787643272
H      0.724077812582   -0.973077361707    3.102791153868
H      0.182127058416   -2.423827992006    2.927191429880
O      4.212842928768    0.505547688470   -4.474664228079
H      4.017487001729    1.418671967806   -4.231212998965
H      5.058025119730    0.297605059030   -4.067754732744
O     -2.073449341237    3.703896264373   -3.938864163443
H     -2.713458967994    3.769337073799   -3.201593346974
H     -1.231900331821    3.994529986194   -3.553657709161
O      3.192265179097    4.174960752422   -0.597593408663
H      3.593670485141    3.458095466445   -0.046520052795
H      3.797370250368    4.915752360872   -0.540711730476
O     -0.325228163164    4.612700704652    2.844269456197
H     -1.131935005065    4.073040139921    2.815681583447
H     -0.009466503898    4.700339760172    1.934778577431
O      0.434767190325    4.836773380832    0.043214595036
H      1.396440523653    4.749472108656    0.084098015569
H      0.210161188502    4.544176321469   -0.854633574480
C      0.187543805696    1.611218024371    0.849704335126
C      1.279354152333    1.569711427508    0.043432730155
C      2.055114116876    0.389524459406   -0.267048761442
C      1.760453774881   -0.858947116858    0.200437685848
C     -0.212558208630   -1.365416966089   -0.908748543059
C     -1.188372841484   -0.576624457821   -0.325247190572
C     -1.500592742432    0.761007405643   -0.827126953946
O     -2.495292458666    1.440088561819   -0.493804121375
H      1.539519347895    2.467220208228   -0.511369008823
H     -0.374062615349    2.522960313184    0.985907204593
H      2.813847834346    0.495594990265   -1.040550946667
H      2.333745714218   -1.715886649438   -0.112945201510
H      1.174606539188   -1.006064809904    1.091292290223
H      0.198515214460   -1.118709661518   -1.872256715122
H     -0.114665952194   -2.400561990753   -0.617917339517
H     -0.063473027267    0.821789841247    1.541343847152
H     -0.899225839429    1.120759695270   -1.676298130319
H     -2.508460848496   -0.341745352400    1.238619351752
N     -1.937300819317   -1.008389719842    0.747036105954
H     -1.648896340450   -1.807124656815    1.284565588001
```

```
Ambimodal TS Water45-14
155
ANGSTROM
O     -4.536809055306   -3.562880908577    3.649771755495
H     -4.921079634450   -2.848084879580    3.125281236589
H     -4.349071008011   -4.282228906047    3.033668421386
O      1.655089160106    1.188399968262    4.044700000956
H      1.189028706544    0.525711775786    3.507891100505
H      1.277217428456    2.048534314089    3.786642418848
O      4.279951261515   -1.906310188342   -2.439577317704
H      4.664309276062   -1.212491049216   -3.006576386806
H      3.332223584905   -1.860854599901   -2.675938578575
O      4.740511272675    2.036605255979   -1.400991776452
H      5.068952985244    1.189368176038   -1.040984590605
H      4.477533861095    2.535463458011   -0.609227570758
O     -3.889614298265    3.585359655270    2.010783471943
H     -3.205371919420    3.961187875263    1.404622168324
H     -4.206665757789    4.319329473102    2.538358382407
O      1.862763103671    4.768098688411   -0.422065672307
H      2.538854738213    4.243807666761    0.035366229542
H      1.358657644083    5.187425342374    0.303570963576
O     -3.989138975096    3.293291381041   -1.213103185675
H     -3.286857594272    2.730990930800   -0.797862995025
H     -4.599165703590    3.512531638753   -0.507010471085
O      0.290714910896    5.469516997696    1.693505712144
H     -0.603094557565    5.376095646989    1.329017286449
H      0.393223089266    4.732594785876    2.315282540475
O      5.387064722875   -0.407490902876   -0.359440417393
H      5.048036030517   -1.027305587791   -1.026556437417
H      5.103803207130   -0.742626897508    0.496541841479
O     -3.135471357028   -0.366178019479    3.453113202681
H     -4.032308490617   -0.425086241448    3.783424379174
H     -2.677841989166   -1.177524374979    3.782134158074
O     -6.467237394724    0.231183657138   -0.890343654713
H     -5.904848720844    0.530342446507   -0.157298085133
H     -6.096081353260    0.646716684853   -1.685076821046
O     -2.054855484180   -2.670019337356    4.202389889683
H     -2.930758430905   -3.094324379316    4.280666554696
H     -1.674599878979   -3.054419986511    3.380834932340
O      1.663533852810    1.166062103439   -3.722385003735
H      1.711252802049    0.674706436836   -2.895754251558
H      1.923942963770    2.085098512590   -3.499872605465
O      3.843872474852    0.989198799460    2.390904621475
H      3.958483798262    0.051152199281    2.188348400201
H      3.179545270908    1.040165902493    3.099905161701
O      0.620067484045   -4.134077942922   -3.426678644847
H      0.720633415022   -4.297993854713   -2.468505375171
H      0.798799592756   -4.965351898989   -3.866857812752
O      1.846411958937   -2.575146455963    3.468939748007
H      1.207068783479   -1.882981320891    3.203699508214
H      1.580991932851   -2.761360344550    4.388239289600
O      0.139876437102    3.420077429194    3.618549983023
H     -0.718452020825    2.934576922042    3.598573001649
H      0.140336876052    3.938601736785    4.424424475089
O     -4.733250375132    1.011856982050    1.174212763635
H     -3.863245217586    1.044981779665    0.703919273685
```

17583	H	-4.739497284734	1.834496149126	1.677332700860
17584	O	-0.586362942674	3.738366458457	-1.295001500950
17585	H	-0.982605902631	4.005034053722	-2.147998927333
17586	H	0.287120667414	4.154327743697	-1.204854910751
17587	O	0.721111832770	-4.563596660019	-0.755369119133
17588	H	0.950430103751	-4.607782593168	0.195507619077
17589	H	-0.250702565087	-4.619337136735	-0.784678163404
17590	O	0.301210330974	-2.531099067449	5.714305627580
17591	H	0.216079746087	-1.626526145687	6.033469267467
17592	H	-0.558135668079	-2.734776390004	5.312484353839
17593	O	-0.059295880747	-0.728644756012	3.082286896333
17594	H	-0.462716791180	-0.695379447706	3.965043008430
17595	H	-0.772177940078	-0.892264701751	2.428700606304
17596	O	4.473302624120	0.449847590900	-3.809194594385
17597	H	3.568932921207	0.714136909993	-4.031801352829
17598	H	4.775772108358	1.087716362869	-3.150561374390
17599	O	1.760382966281	-1.648723854698	-3.369372143044
17600	H	1.365050817097	-2.522966856736	-3.508958903951
17601	H	1.713383232107	-1.171581313095	-4.200558412056
17602	O	3.872822892446	-5.431040449891	1.724810624616
17603	H	4.406237403198	-5.001052143826	2.395907543014
17604	H	3.991067657975	-4.914718837540	0.911050437578
17605	O	-2.204007235942	2.159907607428	3.730805851609
17606	H	-2.434149445701	1.219201726520	3.599320461454
17607	H	-2.782176240332	2.641544511630	3.116239928384
17608	O	3.840198402966	3.224449958337	0.929812612057
17609	H	3.784432748642	2.373197443530	1.421570665799
17610	H	4.498337328802	3.745890922207	1.391132268623
17611	O	-1.996040283893	-4.292074770696	-1.214777815081
17612	H	-2.597725161745	-3.810720933528	-0.635369215133
17613	H	-1.983808443123	-3.796596270380	-2.043761566784
17614	O	-0.442674735753	0.304993834553	5.622927904511
17615	H	0.413703784079	0.680142533888	5.346854311680
17616	H	-1.106104972963	0.973247799364	5.431898284790
17617	O	-0.831437717990	0.028241144286	-4.186940562704
17618	H	-1.503860587627	0.695359989108	-4.393656141659
17619	H	0.014534438322	0.503632009109	-4.139420875735
17620	O	-4.112383257383	-2.840746817013	0.125799265607
17621	H	-3.861555836100	-2.184401963326	-0.558515762667
17622	H	-4.570772325071	-2.334593541247	0.821149304209
17623	O	-1.677010842590	-2.480872164508	-3.512376333249
17624	H	-0.909760636811	-3.063838327454	-3.608154447175
17625	H	-1.364766744811	-1.607072565351	-3.816316288005
17626	O	-2.866506843920	1.868979626511	-4.693643832002
17627	H	-3.243567900453	1.855625778539	-5.573337619382
17628	H	-3.582160880143	1.589301301118	-4.073212777371
17629	O	4.031116334309	-1.862986679629	1.869828369939
17630	H	3.929431273490	-2.497839941007	1.146174966598
17631	H	3.365009661822	-2.111685828396	2.531052740759
17632	O	-1.333897722876	-3.796312367177	1.889566180371
17633	H	-2.024618250186	-4.469628177796	1.781529586857
17634	H	-0.475553554602	-4.247172840186	1.904844772876
17635	O	2.752680872621	3.422482851087	-2.790176265140
17636	H	2.454567782827	4.075648921388	-2.145487899959
17637	H	3.546341298924	3.013708234562	-2.407168991041
17638	O	1.263921411986	-4.665790448870	1.900994938323

```
H      2.085541621701   -5.189365869246    1.890797874846
H      1.485590025391   -3.888339959689    2.463331159926
O      3.662430992107   -3.673364817783   -0.404459121741
H      4.064969147533   -3.188850124203   -1.143936517125
H      2.814584419804   -4.004403967687   -0.723594618272
O     -2.133281213438    4.850013922517    0.447685141997
H     -2.554074189694    5.516514572020   -0.097922978750
H     -1.559251767811    4.322492174306   -0.192839768882
O     -3.619314248738   -1.244502636446   -1.992740892027
H     -4.490059113689   -1.605682391382   -2.277580605123
H     -2.952139730996   -1.655797699107   -2.567100347545
O     -3.676723540473   -5.178136691348    1.431109092134
H     -3.639067273746   -5.933541462600    0.843129340293
H     -3.915572542700   -4.408751126346    0.881208460593
O     -5.525197708193   -1.410683412594    1.968417997687
H     -6.448320168987   -1.388957945483    1.710125213703
H     -5.175255938245   -0.509961951247    1.782724275133
O     -2.312681299243    4.235996322615   -3.289855362504
H     -2.347784144811    3.484346253102   -3.901407125273
H     -3.023792473964    4.074713230139   -2.654231727381
O     -4.619497244137    1.181179540408   -2.798027839864
H     -4.414555081139    1.952982539358   -2.233495010859
H     -4.124796391506    0.416420128262   -2.444130892872
O     -6.100415227546   -2.224334104966   -2.019647726065
H     -6.428798291990   -1.400028455802   -1.608593100931
H     -5.975483612539   -2.857364016079   -1.307426886632
C      0.350646272316    1.648022257263    0.783395822598
C      1.380120413926    1.461344341931   -0.079019424420
C      2.042343419665    0.203424722500   -0.360672882309
C      1.688711612524   -1.004768785921    0.163750968597
C     -0.315812576121   -1.342414719639   -0.940709356282
C     -1.224428518680   -0.591790397217   -0.232785018762
C     -1.571636618408    0.761818878557   -0.628714700578
O     -2.510613733965    1.430264122699   -0.126091233073
H      1.681767048438    2.295597745408   -0.705197527176
H     -0.123758134858    2.611507009712    0.886509238365
H      2.786466604151    0.222915542350   -1.154538826591
H      2.180734958279   -1.908065348285   -0.158409784066
H      1.126440235320   -1.096574734457    1.079753219966
H      0.048435331127   -1.004990802816   -1.895750394181
H     -0.187196399707   -2.394282292893   -0.737837992414
H      0.063352675957    0.918596506232    1.523475233699
H     -1.103394727625    1.148514527942   -1.545210391317
H     -2.585872552161   -0.605777288695    1.336422238521
N     -1.772420352750   -1.079685246036    0.972077934214
H     -1.781600820535   -2.084821921903    1.097889820625

Ambimodal TS Water45-15
155
ANGSTROM
C      0.276075165093    1.444066171625    0.440671317088
C      1.397444711762    1.149879842144   -0.274016603921
C      1.995230920997   -0.162297443158   -0.391323244074
C      1.461215473976   -1.302036039817    0.129162875881
C     -0.496338553483   -1.486743173116   -1.296649199435
C     -1.346211870269   -0.688549895453   -0.566852873682
```

17695	C	-1.560702915866	0.721397595117	-0.897113218556
17696	O	-2.463426405269	1.427399544522	-0.352655747611
17697	H	1.836730017564	1.926944286453	-0.896615473729
17698	H	-0.119629386188	2.449302954022	0.456319091473
17699	H	2.852457452456	-0.238414287099	-1.055148610494
17700	H	1.907546248876	-2.265695587701	-0.061302836414
17701	H	0.736739956222	-1.290159285813	0.929719912609
17702	H	-0.041109175221	-1.124426176448	-2.202975493548
17703	H	-0.461238274364	-2.553015386339	-1.133484393040
17704	H	-0.088597952940	0.794798206061	1.223917551298
17705	H	-1.096787587599	1.100746160746	-1.815882805178
17706	H	-2.379451550937	-0.512680952416	1.196568130179
17707	N	-2.043809975369	-1.176934058524	0.519677898248
17708	H	-1.773482219242	-2.077246237641	0.896010330618
17709	O	-2.531142626841	-4.905833380090	-0.708412449402
17710	H	-1.776027001645	-4.983602881175	-1.315786050825
17711	H	-3.035635743008	-4.137396724544	-1.008342682663
17712	O	5.116589305453	-2.209134405503	-3.519561560946
17713	H	4.970027162742	-1.443375000347	-4.113096383574
17714	H	5.416643156193	-1.838022942041	-2.677636004225
17715	O	-0.357658970381	-0.689276299851	2.951041402985
17716	H	-0.247097633042	0.017324256529	3.612702141141
17717	H	-1.268486772332	-1.010308964858	3.064051668039
17718	O	2.591322245431	-2.639017453072	-3.009791570207
17719	H	2.320925245795	-1.719245657559	-2.847945060813
17720	H	3.551427608244	-2.582422753613	-3.267971528137
17721	O	-0.385333348768	1.212035040518	4.925464885327
17722	H	-1.256418954953	0.875966211749	5.227768244384
17723	H	-0.568682559434	2.057907383688	4.485501098983
17724	O	0.285503125638	-2.918451224557	-4.464263319610
17725	H	1.161079675741	-2.886294194702	-4.037969980393
17726	H	0.247674490308	-2.119881640759	-5.009459186228
17727	O	5.260942454649	-1.112574196829	-0.916371018414
17728	H	5.548473439782	-0.585949469181	-0.156026992523
17729	H	4.842348427776	-1.891434745500	-0.518004415344
17730	O	-2.872713537555	0.271595658446	5.322446850741
17731	H	-3.573586280392	0.835146712929	4.982680623888
17732	H	-2.870499348828	-0.507000511003	4.738025785402
17733	O	-3.971911958102	-2.606121400009	-1.476032508556
17734	H	-4.800112896999	-2.949053723226	-1.816915959740
17735	H	-3.977262000965	-1.648022207423	-1.691067326223
17736	O	-2.646838912868	-0.136844988045	-4.876234671492
17737	H	-2.531637450792	-1.058113700662	-4.510960213361
17738	H	-3.101860198228	-0.249090059847	-5.712242794750
17739	O	-1.262497617680	-3.830420275961	4.210313518832
17740	H	-0.351639661973	-3.483544967191	4.059229279693
17741	H	-1.260406566975	-4.222324177189	5.083043294609
17742	O	2.104225099900	4.472569280710	-1.398594305702
17743	H	1.178035435131	4.740406986189	-1.411469353244
17744	H	2.335210443637	4.311006759684	-0.475405121782
17745	O	1.171300185112	-2.767815008346	3.554912110377
17746	H	0.649721671625	-1.982526973853	3.257741931517
17747	H	1.259610171863	-3.359731528868	2.785518873054
17748	O	2.252951726819	1.566459864782	5.237780651915
17749	H	2.640210279852	1.037053581813	5.934729012969
17750	H	1.296675695165	1.360125472632	5.225172373104

17751	O	-0.824532179456	4.566827716878	-1.375979423367
17752	H	-1.784056429757	4.495641766222	-1.488074446671
17753	H	-0.667017371363	4.661827578511	-0.417706274997
17754	O	-1.365822713632	-3.834648399029	1.393045491301
17755	H	-1.852848993327	-4.345188101928	0.702931647294
17756	H	-1.627162839844	-4.157440335176	2.261208794493
17757	O	-2.368400912392	1.653149236602	2.369331762410
17758	H	-2.427547627103	1.689461528383	1.398726205680
17759	H	-3.247646737618	1.394279978057	2.703940447607
17760	O	2.682707266066	2.638065666729	-3.300896017057
17761	H	3.514294269841	2.220205348904	-3.003416789933
17762	H	2.566164794861	3.395187845787	-2.692883428651
17763	O	2.540499115062	3.672065304142	1.426669445878
17764	H	2.477263102449	2.719141082341	1.631664375122
17765	H	2.550063116139	4.081924347500	2.311383934350
17766	O	-4.812344552111	0.742761510372	3.131365582485
17767	H	-4.793665472862	-0.201027643831	3.317516882238
17768	H	-5.079455133043	0.808973153205	2.194106791873
17769	O	-2.579899728393	2.618502294980	-4.208903653751
17770	H	-1.634539963974	2.697563806471	-3.971147141889
17771	H	-2.691102631995	1.694907512880	-4.461845712507
17772	O	3.442040717638	-1.548297589658	2.533768502683
17773	H	3.608071978814	-2.167144680206	1.806305047731
17774	H	2.734682474149	-1.958495048128	3.067175473842
17775	O	-1.075008435242	3.556240556335	3.567976414698
17776	H	-1.735248966806	4.108074921269	3.989009858051
17777	H	-1.572552085867	2.863458972101	3.047961719398
17778	O	2.435956965938	-4.534114941261	-1.266025303709
17779	H	2.477082856949	-3.768458961530	-1.912731294889
17780	H	2.850007967594	-5.275920422971	-1.710306072982
17781	O	4.841115827840	1.358378944383	-2.257397966994
17782	H	4.944177665978	0.462038096631	-1.905603493991
17783	H	4.929991542143	1.931818575617	-1.468467692291
17784	O	-0.119407852319	2.614271712352	-3.144503842712
17785	H	-0.300859081232	3.313443230546	-2.486898699156
17786	H	0.777470219280	2.773764803050	-3.455276856002
17787	O	2.721706594922	1.187687787436	2.509799587389
17788	H	2.617926898655	1.334983998275	3.461404841358
17789	H	2.769455518659	0.229868527912	2.392104412316
17790	O	4.867557866462	2.539421144255	0.165572885315
17791	H	4.965326769925	1.753111091534	0.722911700816
17792	H	4.143439954881	3.054793377595	0.546387131303
17793	O	5.459490614140	0.137157146133	1.575904057862
17794	H	6.049317160527	0.387059498454	2.287424921148
17795	H	4.724131997679	-0.353483486253	1.979550184634
17796	O	-5.682808827511	2.097003383578	-1.799726797398
17797	H	-4.904309837409	2.717876357681	-1.760083759095
17798	H	-6.345827101412	2.532130840560	-2.334813853931
17799	O	-0.265870513524	-4.740793074088	-2.281981116761
17800	H	0.600069018694	-4.739452092080	-1.853795572429
17801	H	-0.152441301122	-4.298900132389	-3.131902439272
17802	O	1.829244906307	4.116194154458	4.018736828019
17803	H	2.133579986580	3.349058127122	4.520705076155
17804	H	0.876762790677	4.004049448230	3.918086538575
17805	O	-4.614420018795	-2.016423835216	1.323492114308
17806	H	-4.208296832867	-2.436392839974	0.553199800760

```
H     -4.843619874341   -1.125081201261    1.009487636751
O     -2.898692126519   -1.733892101127    3.424810832104
H     -2.475709604033   -2.561226488957    3.708617603216
H     -3.499967072549   -1.951919349440    2.681012085916
O     -4.102717240130   -0.105282817551   -2.401887089825
H     -3.647327211272    0.059722829503   -3.236448007267
H     -4.710889578383    0.636826981176   -2.250066339937
O      4.228059846455    0.065568241153   -4.692846756838
H      4.711711320535    0.700887069197   -4.150850925481
H      3.366485430429   -0.046142415485   -4.236101386502
O     -0.043141455773    4.709699568567    1.213573376185
H      0.887756319665    4.427621844575    1.242877475642
H     -0.431411513730    4.402665811393    2.044340880743
O      1.241748474914   -4.443626359691    1.391147305717
H      0.281142859352   -4.266498183325    1.305931930280
H      1.566246260493   -4.570181230427    0.495387874733
O      2.082228950963    1.630112826375   -5.894328785662
H      2.872348054808    1.083815418835   -5.990746310354
H      2.259860276219    2.183383994724   -5.118549493672
O     -2.401654702803   -2.554823271356   -3.915994987077
H     -2.648641550233   -2.662688368475   -2.988041699334
H     -1.464675702006   -2.796648376430   -4.008141575167
O     -3.430395798123    3.466502131540   -1.796430717769
H     -3.006942298019    2.779823547342   -1.236051382092
H     -3.172022931349    3.244106329240   -2.718387800388
O      1.852305902106   -0.047804451260   -3.353787123436
H      1.895164304072    0.897369471165   -3.141297489743
H      1.181611875351   -0.135273414970   -4.062607818670
O     -4.937743362947    0.608020690345    0.442235980084
H     -5.501247657754    1.057365215475   -0.200163653666
H     -4.027555166590    0.802870376738    0.136527962211
O      0.144605213157   -0.229893471891   -5.406331775416
H      0.658833514023    0.504017083339   -5.797437106875
H     -0.758807476097    0.081674291139   -5.251121881176
O      4.211176921046   -3.330730094056    0.457460752065
H      4.770048988593   -3.987077788158    0.874467364105
H      3.555958741899   -3.813380668604   -0.083164583162

Ambimodal TS Water45-16
155
ANGSTROM
C      0.510819100420    1.602320899016    1.153551540817
C      1.505699705494    1.516389747046    0.272519585718
C      1.940378451687    0.273740286985   -0.414379072807
C      1.755309056318   -0.968786057065    0.036451761102
C     -0.724992833467   -1.545064679522   -0.960365405211
C     -1.546672803833   -0.680593452269   -0.303766337714
C     -1.984940924467    0.534305915535   -0.984983313262
O     -2.697419484602    1.430051021180   -0.495233274933
H      2.050348731490    2.414092266535   -0.008676578240
H      0.238954158397    2.533894084486    1.623073545194
H      2.441883519406    0.436431580531   -1.364629258823
H      2.086256174231   -1.837620292831   -0.510861953590
H      1.293414090384   -1.169671479912    0.992342091068
H     -0.352674783951   -1.318489678325   -1.944015409406
H     -0.409654181036   -2.474307338464   -0.513837059279
```

17863	H	-0.070998230452	0.741705099175	1.449149762482
17864	H	-1.677786204105	0.628423178720	-2.039350437353
17865	H	-2.836153855417	-0.536141708868	1.324534297837
17866	N	-1.923747506395	-0.847982015301	1.027723013287
17867	H	-1.606342725736	-1.702808631918	1.464461032206
17868	O	2.715500200084	0.199746164116	-4.883072542219
17869	H	2.499771433375	1.137171632984	-5.071536007098
17870	H	3.688098397657	0.179188769966	-4.778362786234
17871	O	-1.088795867212	0.507847387177	3.834430795808
17872	H	-1.534285738791	-0.352538622958	3.847590520720
17873	H	-0.144364904405	0.337595602167	3.701915208864
17874	O	-1.199869277983	3.291773724583	-4.424628033013
17875	H	-1.173673188641	2.452658258770	-4.937814624237
17876	H	-2.085627933126	3.315379431524	-4.035194298659
17877	O	-5.316680809343	-1.993386774458	1.004944832825
17878	H	-5.166658605272	-1.247922786418	1.607832243275
17879	H	-4.806145198146	-2.728956654899	1.366267285676
17880	O	1.968669976607	2.792769768239	-4.996921441072
17881	H	1.045925123296	3.060269646183	-5.042237409465
17882	H	2.333339158458	3.169675163362	-4.184138145722
17883	O	-3.490607151811	2.898675472331	-2.786820458711
17884	H	-3.969112907398	3.666196605057	-2.465161453872
17885	H	-3.091199213315	2.497303226617	-1.998389821252
17886	O	-3.262827811899	-3.677804959650	2.178226291701
17887	H	-3.017841421350	-3.733945675866	1.225766948254
17888	H	-2.815949675594	-4.442608716808	2.564366532325
17889	O	-3.018139414834	-2.534007636317	-2.706559849185
17890	H	-3.618369402248	-3.215090985366	-3.020539448356
17891	H	-3.567036090243	-1.912606041709	-2.183720607639
17892	O	1.729519728016	-4.540246658356	0.703962364201
17893	H	1.171677130709	-3.770666599947	0.927908952868
17894	H	2.190636317473	-4.355568693559	-0.127321170839
17895	O	-4.348786452311	0.052184325943	2.611537630761
17896	H	-3.934035189383	0.926892047689	2.644914310381
17897	H	-3.862164938236	-0.497609569929	3.249335910239
17898	O	5.335473149657	0.131884739229	-4.195281968235
17899	H	5.265224722900	0.787530438732	-3.482672276829
17900	H	5.512912243287	-0.706013971695	-3.758022865805
17901	O	4.621209552756	1.472835968480	-1.889308399822
17902	H	3.922573571115	2.109308057876	-2.153428788389
17903	H	4.958068093194	1.812173086530	-1.050521244788
17904	O	0.464966879529	0.321345826106	-3.273143753301
17905	H	1.331451961124	0.262197907329	-3.707335112815
17906	H	0.450679565646	1.156081178988	-2.780713019239
17907	O	4.472683450748	-2.040201772187	0.872969955836
17908	H	5.255472100679	-2.571593995419	1.020219573842
17909	H	4.460357843087	-1.827864004566	-0.090625444627
17910	O	4.865445696932	2.569661045154	0.682646579968
17911	H	4.241590264822	3.303897373183	0.503597073352
17912	H	5.339047194966	2.808808559574	1.482985511214
17913	O	-2.507456731941	2.148033655375	2.289503725251
17914	H	-1.946108363224	1.575805029826	2.869112350464
17915	H	-2.385578258978	1.808836978541	1.388763130262
17916	O	-0.508922863497	-5.836972128087	-0.159529529099
17917	H	0.320137507833	-5.476735138372	0.212884812733
17918	H	-0.301264725927	-5.938577961241	-1.110427295044

```
O     2.588706983234    2.839563967829    4.397543170180
H     3.522734538189    2.623341722272    4.181599532279
H     2.595749883693    3.307708104464    5.233069813731
O     2.247562633514   -2.108826168734   -3.278044599873
H     1.313973914751   -2.381657291247   -3.391399767268
H     2.425137473139   -1.505992015923   -4.011807432501
O     0.489199536530   -5.535417257051   -2.641364154935
H     1.386334713887   -5.301246040044   -2.367698138189
H     0.158112974830   -4.749962268576   -3.112904236121
O    -4.610374005807   -0.848916367491   -1.268376897101
H    -4.963253287085   -1.401690550492   -0.526419152245
H    -4.398310213265   -0.009257890185   -0.853309421430
O     5.079439507087    1.985666401454    3.802484684013
H     5.547465516572    1.521245352406    4.497143948308
H     4.740441012873    1.295678513269    3.186807043397
O    -2.587354698681   -4.122149781235   -0.332592792097
H    -2.476455246085   -3.596030253500   -1.131906273981
H    -1.849331715474   -4.767311709930   -0.298044711612
O    -0.159354362129   -2.988716401416    1.936071334598
H    -0.622136466742   -3.805046381819    2.208536852106
H     0.488656594303   -2.795444987337    2.631553766319
O     4.516190655115   -1.326788856130   -1.729352045681
H     3.702730457683   -1.564517841428   -2.201139533491
H     4.539918543278   -0.353368461069   -1.724062220871
O     1.483152007379   -5.331601478893    3.343260620227
H     1.727126618597   -4.477267069189    3.717235230358
H     1.762583558483   -5.284665364729    2.415640922732
O    -1.089569610940    4.409739911399    2.358486316608
H    -1.636173496270    3.584352880604    2.388120640850
H    -1.546501235662    5.062191005858    2.889308851223
O     1.732807207800    4.644740878486    2.448625067053
H     1.998629956888    3.956132250634    3.081967846574
H     0.766884573288    4.597737581069    2.391776315039
O    -0.832074333937   -2.579972390880   -6.207572057234
H    -0.156914406796   -1.956305287888   -6.554042232689
H    -1.650123768126   -2.072756393101   -6.145749020708
O    -2.963857659330   -0.967365441004   -5.048156967532
H    -3.716086347422   -0.391777975990   -4.807376987144
H    -2.820855894667   -1.522221057650   -4.265585881252
O     2.136522921773   -2.474849550077    3.399099941866
H     2.005376221353   -1.552729694113    3.671979653020
H     2.775096283837   -2.452881576976    2.678033852140
O    -1.145871600381   -5.446750903324    2.623844220217
H    -0.273198700343   -5.603533716774    3.036623997225
H    -1.108026576123   -5.872465589630    1.756699139494
O    -2.731396193734   -1.760594639615    3.930515353202
H    -2.880827580402   -2.452666507552    3.239704487958
H    -2.900783822382   -2.182085026116    4.772938778852
O    -0.988012459060    0.834298429186   -5.433713373272
H    -1.762519396849    0.239725852649   -5.421757562320
H    -0.464943913486    0.575858585388   -4.638292626041
O     2.712857190062    3.364840207743   -2.331147363507
H     1.755590259468    3.137518470338   -2.231151237335
H     2.901111693819    3.960694439381   -1.595113216505
O    -3.888087610001    3.948397227521   -0.070686279120
H    -3.626900494277    3.018434028014   -0.019343949206
```

```
H     -4.252989049077    4.172012860952    0.786151670891
O      2.913851337170   -4.268595173599   -1.806671362945
H      2.706294928304   -3.501387757893   -2.375122036726
H      3.794426433779   -4.557978196996   -2.044193059226
O     -0.265174282421   -3.112254394962   -3.707986677595
H     -0.417930764151   -2.999394806578   -4.683551828658
H     -1.105096593201   -2.892465283166   -3.291428036218
O      3.159313814646    4.632390617093    0.216858245268
H      2.561820199255    4.613849322975    1.007171484598
H      3.495511799984    5.528125711215    0.167954757681
O     -4.785417347905    0.579942734011   -3.784991433243
H     -4.938530063824    0.086255356771   -2.969880929491
H     -4.389385887934    1.429402350242   -3.535587101113
O      0.860971685717   -0.592617682117   -6.868467807834
H      1.638906468937   -0.443142392546   -6.313385799297
H      0.212786169570    0.075668393228   -6.580824043599
O      1.751183304699    0.265577064460    3.630447584167
H      2.394031744002    0.315685501453    2.899590197315
H      1.850837265844    1.112918032698    4.086695908536
O      3.982214956885    0.345519533025    2.018172372859
H      4.121575776404    1.008899925815    1.319548108439
H      4.186365918561   -0.526946452900    1.623527681799
O     -1.147809689415    4.482206526963   -0.447734757986
H     -2.114575300788    4.447150322994   -0.492339202594
H     -0.936306474088    4.520791137215    0.495808691698
O      0.122223429622    2.827100847934   -2.178057446181
H     -0.326140252359    3.085492429065   -3.021642224936
H     -0.283486127190    3.395594014713   -1.495559645524

Ambimodal TS Water45-17
155
ANGSTROM
C     -0.192669857593    1.745356029966    1.149662177123
C      0.992022324062    1.873867609046    0.492326976572
C      1.889754518720    0.794480134005    0.153244805343
C      1.645109589005   -0.526378788256    0.420469970745
C     -0.017679264304   -0.871845452631   -1.119487938322
C     -1.130768734668   -0.434910832167   -0.432727085558
C     -1.675955929939    0.905622684099   -0.624218914248
O     -2.766447904033    1.299338884871   -0.145719945268
H      1.253664172671    2.846648768381    0.085100835727
H     -0.820233770750    2.606557396727    1.327131662528
H      2.719675533128    1.056387007465   -0.499348666096
H      2.328736262681   -1.299369740418    0.103408168293
H      0.961487282848   -0.835727126947    1.198498108833
H      0.393462899204   -0.264871622540   -1.911245251832
H      0.257756870154   -1.914468370536   -1.137342346177
H     -0.454379794054    0.869775141312    1.725775952725
H     -1.205399529704    1.515797162140   -1.416037546969
H     -2.685679728749   -0.974260689083    0.771095609153
N     -1.717734597557   -1.198433860100    0.581029158347
H     -1.534514813889   -2.191662241689    0.564593104052
O      0.626651797674    5.970983880789    0.887915727635
H      0.840164430622    5.655597531246   -0.003327776419
H     -0.331896434612    5.982975807934    0.937359544036
O     -1.724290909028    4.395009175372   -0.446639834865
```

18031	H	-2.233525204525	3.995382776899	-1.166348829668
18032	H	-0.800343713643	4.394196394282	-0.738656644707
18033	O	2.997126241927	3.851167097208	-3.831851753427
18034	H	2.576764475555	4.677534839549	-4.075125115846
18035	H	3.419050689391	3.986619458943	-2.969091691645
18036	O	-4.678066854686	-1.272203899723	0.135288142701
18037	H	-4.841836329160	-0.648294561839	-0.598530003486
18038	H	-4.264632577272	-2.040913320069	-0.291466659393
18039	O	-0.746940149033	-5.564215846849	-2.135439583195
18040	H	-1.041058113650	-5.249821443282	-1.260601445372
18041	H	-1.279213226898	-6.328356723977	-2.355971619653
18042	O	-5.116529364994	0.250503476845	-2.084919132236
18043	H	-5.286493323769	1.130216437287	-1.687338560488
18044	H	-4.405997538110	0.399778746624	-2.730284173072
18045	O	4.885419034826	-0.858505656816	-0.107271701659
18046	H	4.373285737971	-1.230147556316	-0.837914175732
18047	H	4.892097075084	-1.565176424311	0.564001703262
18048	O	5.074297657930	1.554912241729	-1.608435496973
18049	H	5.287428515805	0.852147820814	-0.983553882615
18050	H	4.668133367813	1.101938628388	-2.371275520391
18051	O	-4.147411414308	-1.136313451410	2.808525311040
18052	H	-4.028340658764	-0.178793423605	2.873687888267
18053	H	-4.471563267384	-1.297507622521	1.905546032016
18054	O	1.485128615672	4.139995902419	2.729885802262
18055	H	1.249088913013	4.837102676644	2.087609981447
18056	H	0.693249476504	4.094366375716	3.311885194837
18057	O	1.790169517842	-4.873221108924	3.133207290650
18058	H	2.111976475828	-3.999721139725	3.408981815090
18059	H	0.825195324712	-4.804966565102	3.082849376526
18060	O	-1.002013012438	-4.647792410519	2.904728691611
18061	H	-1.521094256585	-5.272441823428	3.411160242454
18062	H	-1.316119772830	-3.743800566183	3.156563583326
18063	O	1.091875862186	0.285213055846	-5.234633688153
18064	H	1.903209618027	0.393902520492	-5.733500951074
18065	H	1.194008441004	0.850503998793	-4.428783947047
18066	O	-1.102915118568	-4.203646553801	0.242701989144
18067	H	-0.193179709631	-3.957961073785	-0.017265331438
18068	H	-1.053059835663	-4.486037187503	1.174161838216
18069	O	-5.041439792557	2.606496951231	-0.784401801737
18070	H	-4.167217731032	2.275598987301	-0.496537201398
18071	H	-5.639660467352	2.370543336365	-0.050721726409
18072	O	4.505117622578	-2.890979350820	1.668825412703
18073	H	5.235444456946	-3.131470448383	2.240473099799
18074	H	3.769874744168	-2.620591061958	2.276995662882
18075	O	1.370263423336	-3.940200186673	-0.900813754617
18076	H	1.080897833926	-4.585454149513	-1.555301113100
18077	H	1.979243350101	-4.420574915963	-0.260183522860
18078	O	-0.859710155916	3.948511420147	3.998252279095
18079	H	-1.548419642024	4.145203570200	3.348962739786
18080	H	-0.977295896124	3.005646554433	4.213860188068
18081	O	3.499516017470	0.066734422116	-3.259345921764
18082	H	3.424323039288	-0.789709927416	-2.820159990222
18083	H	2.711184143384	0.570796250012	-3.019506759780
18084	O	4.208049995638	-0.284983527526	4.473022376017
18085	H	4.526396073285	0.094723643747	3.638844889287
18086	H	3.528880930518	0.330322644133	4.771816789645

18087	O	-5.260108760878	-2.399308787312	-3.087384424632
18088	H	-4.766445426995	-2.862814806588	-2.398472593570
18089	H	-5.491719273942	-1.535871572444	-2.718416977336
18090	O	-1.467618975399	-0.978422894131	5.834364339497
18091	H	-2.000666416291	-1.250794657917	6.580288297859
18092	H	-1.692959786934	-1.567506093159	5.083777983463
18093	O	4.375181222249	0.985240627963	1.986952814416
18094	H	4.526893635065	0.440620523637	1.199308045335
18095	H	4.397386454745	1.914700321245	1.706922922171
18096	O	-6.126565620053	1.326305685496	1.341448746399
18097	H	-6.162883734447	0.408269795618	1.059456988863
18098	H	-5.307652034781	1.414923230822	1.861919111813
18099	O	0.907113790719	4.641096327617	-1.523441490363
18100	H	0.551019534475	4.436933741027	-2.400019204303
18101	H	1.797986957686	4.251250103450	-1.470480442801
18102	O	-3.177339791129	-1.682017522142	-4.642695687543
18103	H	-3.997404691082	-2.023587652891	-4.223660775245
18104	H	-2.447001531591	-2.174041578517	-4.228351911120
18105	O	-0.575644789532	3.657911000529	-3.666584190890
18106	H	-1.464242799313	3.770329648129	-3.303375732484
18107	H	-0.720860758466	3.181122623763	-4.515054435031
18108	O	-3.333676909557	0.834748235488	-4.161232105458
18109	H	-3.182910744123	-0.147553778663	-4.347925815227
18110	H	-3.988223732791	1.105396378108	-4.809586955858
18111	O	2.107672813697	1.440701793919	3.524380557430
18112	H	2.821055900996	1.267088223032	2.886141169222
18113	H	1.787545894959	2.332132412329	3.335275369963
18114	O	2.696315516404	-2.266203191027	3.488426896215
18115	H	1.857177380168	-1.761895920680	3.412815291834
18116	H	3.292097276696	-1.663988732691	3.985743996753
18117	O	3.511609129762	3.708518545810	-1.136902702638
18118	H	3.633161159646	3.866607268523	-0.187036601413
18119	H	4.088522646115	2.937296126215	-1.321118733007
18120	O	-2.882901625897	4.169554762542	1.966022726257
18121	H	-3.595403635911	4.809169444838	1.942209712065
18122	H	-2.453912903102	4.205299212738	1.082422689804
18123	O	3.006744505642	-5.029352658666	0.761548553548
18124	H	2.541986006199	-5.145946192386	1.621630952457
18125	H	3.664017266207	-4.335934876645	0.944682123934
18126	O	0.517103435764	-0.706975794673	3.574124964391
18127	H	0.184051541519	-0.710350714494	4.477827356848
18128	H	0.987154250622	0.154091342687	3.493898294800
18129	O	1.343128517433	1.848952741657	-3.101785837249
18130	H	0.521680485241	2.376232820065	-3.134829767036
18131	H	2.028690720549	2.491772814170	-3.386237185298
18132	O	-3.137678361866	3.287427797118	-2.672176616445
18133	H	-3.981811718883	3.341375172688	-2.204617865897
18134	H	-3.135479700199	2.424117222077	-3.110391548145
18135	O	-1.828086175694	-2.208879665096	3.464724099668
18136	H	-1.155388693108	-1.613856098950	3.096322904842
18137	H	-2.708874797668	-1.864461757322	3.175933225383
18138	O	-3.233930969000	-3.181779882535	-1.255095903730
18139	H	-2.605008430399	-2.939560633605	-1.958280343106
18140	H	-2.693998631277	-3.664673112024	-0.614767174788
18141	O	-1.147098575929	1.908402883520	-5.592597318286
18142	H	-1.866286905507	1.462853227158	-5.117443070241

```
H     -0.433289271033    1.255548796477   -5.665798256689
O      3.892098976937    3.698658004387    1.617999814302
H      4.461264861588    4.348413456854    2.031683662329
H      3.014705074230    3.801329735759    2.043075847681
O      3.262129421094   -2.425461208316   -2.011761506080
H      3.885680662382   -3.083426948598   -2.323601803876
H      2.530086919108   -2.925395908379   -1.571427762397
O     -1.477143652048    1.311765069485    4.223764015722
H     -2.269007537448    1.260712938940    3.668591451911
H     -1.557454176065    0.645715526809    4.919170499242
O     -1.279987915613   -3.018309868365   -3.180651396828
H     -1.104639072843   -3.962618856990   -3.047828001756
H     -0.426777178056   -2.652527567512   -3.496085260492
O     -3.562981195751    1.547373261873    2.357124386982
H     -3.411206343375    2.509581430979    2.404281065111
H     -3.221782217590    1.316721113542    1.461935882699
O      1.115425663794   -2.196798973555   -4.060163914252
H      1.065845074250   -1.356608547374   -4.552038376343
H      1.864456011839   -2.127051801987   -3.458046221581

Ambimodal TS Water45-18
155
ANGSTROM
C      0.174456810483    1.681123912099    0.841409878180
C      1.330159784856    1.494619854042    0.164913431232
C      2.034985548509    0.234553388751   -0.018080138653
C      1.680849788507   -0.959619916203    0.514826030428
C     -0.300739714717   -1.363710049322   -0.863173837170
C     -1.261301953981   -0.521321207484   -0.361049104805
C     -1.464982781278    0.807646364788   -0.933684222350
O     -2.450617460636    1.536135825965   -0.652961773384
H      1.752139589866    2.328163387315   -0.388077134132
H     -0.298632271575    2.651915030972    0.895930685923
H      2.846442050964    0.266404173437   -0.739049845865
H      2.226673156250   -1.863678559738    0.284573892076
H      0.989128753339   -1.046452492785    1.337626283404
H      0.219694220615   -1.103962387544   -1.767176875189
H     -0.214004171332   -2.381484065409   -0.518594837829
H     -0.238203501319    0.936129857071    1.505051476580
H     -0.804326127820    1.122202503616   -1.751079904955
H     -2.647714434457   -0.178582133465    1.133333773333
N     -2.103793319062   -0.895629507028    0.676140962522
H     -1.772623205987   -1.611359849398    1.311525925318
O     -2.022686550426    4.702277832994    0.196919985778
H     -2.742807543346    4.685396395061   -0.445234570070
H     -2.337861676366    4.207944559201    0.963096982579
O      0.938110617125    2.165160710015   -2.838903332066
H      1.731258535980    2.729675149905   -2.743984580995
H      0.725230980139    2.182301072932   -3.795466986601
O      2.795089338411    1.089964082228    3.062294777255
H      3.221306005914    0.409265405148    2.522606898027
H      1.913988523383    0.714473629695    3.295085283219
O      0.632712632202    1.912975147936   -5.511696472665
H      0.553126038748    0.948728846832   -5.593801086364
H     -0.278015531044    2.249342500925   -5.559472629496
O      3.381571520786    2.518840613969   -4.987797716808
```

18199	H	2.486003542448	2.389106676425	-5.331049891382
18200	H	3.320498651064	3.185018846090	-4.289756440195
18201	O	1.214082276156	-3.846164466089	2.252773052665
18202	H	1.975896234468	-3.546896057617	2.761016101158
18203	H	0.509330753776	-3.177655112271	2.433503382890
18204	O	-5.047724113117	-2.176275592236	-1.377362008313
18205	H	-5.735854501184	-2.277333415329	-2.035979613173
18206	H	-4.198105745491	-2.296651279229	-1.841998941181
18207	O	0.316774001002	-0.824198883268	-5.133875281976
18208	H	0.951245128174	-0.715380938762	-4.380655936950
18209	H	0.548270073755	-1.639295256903	-5.578472703460
18210	O	5.193390212469	0.216324904375	-0.626741604659
18211	H	5.109898588430	-0.576144829862	-1.182043963787
18212	H	5.037464368945	-0.081648120972	0.275764354723
18213	O	4.411651696473	0.459435295077	-3.650602587639
18214	H	4.853681464927	0.884444404344	-2.909688150253
18215	H	4.146584181540	1.185457734308	-4.262340758238
18216	O	6.079078927601	0.505148523869	3.321086199338
18217	H	5.565216740846	-0.097565924973	2.757288118276
18218	H	5.896945236741	1.395642658148	3.001099727447
18219	O	2.826959021598	-2.159383141602	4.051895322266
18220	H	1.976943564729	-2.132238713523	4.514982756552
18221	H	3.400959677221	-1.577118894768	4.595314859777
18222	O	4.316434952889	2.840891608141	-0.330888167169
18223	H	4.588370755851	2.981920424800	0.594413721211
18224	H	4.643891766206	1.950114373840	-0.535540596589
18225	O	-1.106387003921	2.292951293381	3.858037021075
18226	H	-1.721556624707	2.467889903647	3.122431360485
18227	H	-0.361752544522	2.911210281344	3.727797912107
18228	O	5.091822198462	-3.043587257220	0.225850661094
18229	H	5.060260829548	-2.753918106624	-0.699986268746
18230	H	5.953339385087	-3.438198384843	0.363214453572
18231	O	2.151703726543	4.652775034757	0.795002820652
18232	H	2.860473519555	4.107763594218	0.435808316525
18233	H	1.471594301545	4.722085598407	0.102870449010
18234	O	4.795206173337	-2.022906444172	-2.321346580683
18235	H	3.932900492890	-2.466360197189	-2.471837767614
18236	H	4.906818414853	-1.421367014874	-3.067113133034
18237	O	-2.907721489179	0.095149720582	4.480866104342
18238	H	-2.380795983912	0.902334478142	4.474820728924
18239	H	-3.534586073381	0.160150304919	3.749851617512
18240	O	0.422486392601	0.129136288054	3.772063634196
18241	H	0.505426015255	-0.306295188098	4.636714738015
18242	H	-0.182829484196	0.900930246032	3.879977177309
18243	O	4.278252253135	-1.102156704928	1.946139112878
18244	H	3.769058203590	-1.543249424928	2.650885545129
18245	H	4.571493557229	-1.832725991697	1.369465880773
18246	O	-3.504329897852	-4.095854210758	3.690464177544
18247	H	-2.809241378232	-4.578885245517	3.205492186120
18248	H	-3.994432422938	-3.595554365344	3.024642134198
18249	O	1.222527733219	-4.344624307516	-0.376498350331
18250	H	1.608337760571	-5.219845005856	-0.431083050301
18251	H	1.236737919744	-4.115247001676	0.582709387592
18252	O	-2.013196883740	-2.410737433774	5.089550570361
18253	H	-2.448418262289	-1.542331189272	5.006688298206
18254	H	-2.654642836020	-3.079708379581	4.755697891407

18255	O	0.255590867103	4.475443913039	-1.263859814777
18256	H	0.235637523183	3.559952062794	-1.579954893009
18257	H	-0.531237604170	4.598612469010	-0.695215932702
18258	O	-4.730837755404	1.493068344472	-3.206853015707
18259	H	-5.063488354857	1.187720529582	-2.345492900363
18260	H	-4.003339784487	0.905895214480	-3.436722587673
18261	O	1.906361618738	-0.302776930613	-3.098253449844
18262	H	2.838603197598	-0.105858727078	-3.338078335025
18263	H	1.516633130187	0.573953883184	-2.872070389885
18264	O	-0.702671405827	-2.106139387009	2.779754566732
18265	H	-1.217320450657	-2.386817109438	3.566406727379
18266	H	-0.288497361167	-1.266144906766	3.060492844925
18267	O	-4.505873812041	0.114901587908	2.094871425928
18268	H	-4.947894830362	0.375849405731	1.268371135303
18269	H	-4.513305914364	-0.859734773519	2.069600752855
18270	O	2.261054350461	-2.967783181167	-2.554700520497
18271	H	2.004338986736	-2.031073031013	-2.565546991174
18272	H	1.992030830707	-3.332798127030	-1.692683080487
18273	O	-1.452706501201	-5.081793294782	2.117383891295
18274	H	-0.532676760099	-4.909848414363	2.345898225526
18275	H	-1.639217849382	-4.546581428766	1.334785299819
18276	O	1.224862254653	3.564049625903	3.172305292420
18277	H	1.792100851841	2.785176937156	3.157663205229
18278	H	1.457448330959	4.076435508465	2.377670249594
18279	O	4.174934269128	-0.123528201126	5.209514680607
18280	H	5.050114121544	0.005436776635	4.803701126072
18281	H	3.607720338020	0.513825885982	4.751419470753
18282	O	-2.961611113855	2.524924923867	1.835559375019
18283	H	-2.784367998187	2.165358382101	0.947338989215
18284	H	-3.708652796319	2.015440002058	2.169704941977
18285	O	4.659931933416	2.972305636514	2.381170071424
18286	H	4.449039565525	3.829091182066	2.756844287923
18287	H	3.949682416113	2.368296875174	2.662565303459
18288	O	-2.620755703220	-2.399518964344	-2.593574569599
18289	H	-2.442122571497	-1.532305708125	-2.990603706869
18290	H	-1.868235978975	-2.969667395088	-2.845587289504
18291	O	-1.562387651015	4.688930832359	-3.439935614189
18292	H	-2.339781817178	4.494666953268	-2.874792516682
18293	H	-0.820480377250	4.822206985972	-2.831884585936
18294	O	-3.546305235865	3.660853339378	-1.948072464772
18295	H	-4.170332162644	3.196883511589	-2.528484353016
18296	H	-3.084494943879	2.934838954271	-1.485737306519
18297	O	-4.984914326138	0.471638669818	-0.609736896920
18298	H	-4.059473559626	0.772916213187	-0.600430599310
18299	H	-4.973013957233	-0.480140918121	-0.816781628688
18300	O	-2.506773399189	-3.758094866484	-0.156719036993
18301	H	-2.969947363892	-4.533503018011	-0.478593146360
18302	H	-2.448509645058	-3.140543058821	-0.905381422547
18303	O	3.023854285533	3.855891233358	-2.497384909608
18304	H	3.586897904303	3.558025190370	-1.757208646092
18305	H	2.573872928248	4.652786320694	-2.203456215399
18306	O	-0.390128998718	-3.974831096607	-2.813606454013
18307	H	-0.235376616593	-4.294018817269	-1.915782071398
18308	H	0.477980879495	-3.658832187831	-3.102937353998
18309	O	-1.935714375893	2.547251266088	-4.904824250811
18310	H	-1.733344141801	3.355242412990	-4.361248732840

```
H      -2.778739561404    2.723516789175   -5.324571742795
O       0.532115732755   -1.771533257058    5.713708856805
H      -0.374921262172   -2.113115781066    5.575864744254
H       0.708457437252   -1.822557518282    6.652875963024
O      -4.449161797371   -2.578013800795    1.438315884712
H      -3.625739818465   -2.859933481145    0.998241703460
H      -5.093384398233   -2.532898121968    0.724651330904
O      -2.013771021326    0.012986039333   -3.836176105128
H      -1.272233289289   -0.372674338653   -4.330064071423
H      -2.017224877815    0.945741616508   -4.122574121532

Ambimodal TS Water45-19
155
ANGSTROM
C       0.196612901575    1.741203246765    0.709509700998
C       1.325016803099    1.562447577316   -0.022055190717
C       2.028559518611    0.301673943107   -0.208961945445
C       1.683468220293   -0.885302726331    0.354241249954
C      -0.346063498890   -1.384459391568   -0.902154356893
C      -1.255789896793   -0.587500070720   -0.243805248608
C      -1.578291314244    0.745830968466   -0.760037425656
O      -2.541189055740    1.449960660112   -0.352692028162
H       1.701226731905    2.399933046777   -0.605689094442
H      -0.296363761873    2.703793799341    0.750921841792
H       2.815402567598    0.309043583406   -0.959338352456
H       2.220799452556   -1.788440465582    0.107203889338
H       1.017915875125   -0.966337208579    1.203701255739
H       0.077867803586   -1.086673703922   -1.845314660839
H      -0.199435218213   -2.414616059219   -0.616870717625
H      -0.174494562001    1.002731036073    1.407948878272
H      -1.068281265357    1.061380961101   -1.682921066068
H      -2.655107204988   -0.506946793748    1.286322004363
N      -1.806387661473   -0.953499354795    0.967974891810
H      -1.692027686659   -1.912265747764    1.280177768885
O       0.535071432275    4.868078986300   -0.784487069719
H       1.305811769827    4.740178763746   -1.395524271831
H       0.326046730998    5.803704236644   -0.816432042897
O       3.834623355290   -3.648705279222   -0.241550163729
H       3.486664085352   -3.645509708721    0.671787911191
H       4.383960472269   -4.429328829893   -0.322119118230
O      -4.037304771119   -2.113328445746   -1.139624223626
H      -4.097739732463   -2.526993115760   -0.230844258362
H      -4.609495167731   -2.636555119042   -1.709056706464
O       4.877885347483    1.371236758742    2.372031052652
H       4.497287972889    1.922585818378    3.086684832403
H       4.761018370398    1.872528139272    1.543310367049
O      -1.396581681913    1.343970871122    4.019778681238
H      -2.040464681317    1.679944141965    3.369087096637
H      -0.765408379246    2.066809970271    4.163859232054
O       1.000111296905    4.906033906859    1.974616825918
H       0.796565962916    4.785376231437    1.033875517556
H       1.973592419304    4.983080064308    2.028073791417
O      -5.790329159271   -0.187779705731   -0.091977115053
H      -5.243871746497   -0.843619205893   -0.554586017838
H      -5.732639910356    0.624322648958   -0.616529807082
O       5.574621863992   -1.461746799578   -0.563575389871
```

18367	H	4.917009055587	-2.169613707457	-0.495327526158
18368	H	5.934817642339	-1.324140317331	0.334292763305
18369	O	-2.111164931563	-3.635252845134	-2.521158584911
18370	H	-2.852469237182	-3.749702468809	-3.137816004547
18371	H	-2.474148212905	-3.062788467955	-1.825975810914
18372	O	3.684186875960	4.637485432741	2.116210288710
18373	H	3.850565603913	4.261992890975	2.987766741328
18374	H	3.893849626458	3.940919876622	1.478001004450
18375	O	1.050365598321	-2.860266065786	4.181110097181
18376	H	0.160871075596	-3.188413756889	4.370400047143
18377	H	0.930710507248	-1.921980387881	3.952607561454
18378	O	3.060924901191	1.048190529500	-5.429051745845
18379	H	2.424530379001	1.564706467044	-5.965128948120
18380	H	2.611108825482	0.223943167068	-5.195145382869
18381	O	0.272330404993	-4.748478209619	-1.845048278297
18382	H	0.273012956099	-5.645656873045	-2.180838110669
18383	H	-0.603833024939	-4.372810233818	-2.057041100967
18384	O	-3.496019039522	4.064022384682	-0.307309943224
18385	H	-4.379341388767	3.873631268488	-0.639835511813
18386	H	-3.040585554203	3.204759312842	-0.306751153127
18387	O	2.997443316680	0.113474491927	5.427885420289
18388	H	2.991864941554	-0.539475889732	6.127995666935
18389	H	3.353268023067	-0.335030595375	4.632126197559
18390	O	-3.268667231637	-0.518702586172	-3.282108348904
18391	H	-3.230210022461	0.456930716175	-3.355891847465
18392	H	-3.451129376144	-0.750203999635	-2.362327094200
18393	O	-1.135448774635	-1.777894292756	-4.384256451670
18394	H	-1.856338688595	-1.224688941050	-4.026475111078
18395	H	-1.192079609248	-2.588192917058	-3.858306886584
18396	O	0.615999821806	-4.735136506210	0.934878781227
18397	H	1.403813579591	-4.283926642538	1.264239824224
18398	H	0.639555153335	-4.713602562116	-0.033629715609
18399	O	2.847679320635	-3.407735924928	2.261244243911
18400	H	3.245669445016	-2.534609527389	2.412946523163
18401	H	2.186111213216	-3.461263229492	2.981907831570
18402	O	0.844229736054	-0.145566966795	3.629475162696
18403	H	-0.049876861103	0.226855857204	3.742957656932
18404	H	1.349596243285	0.139635133054	4.407495613831
18405	O	-1.659176171441	-3.649197617041	1.861282836270
18406	H	-1.600838383716	-3.640646864694	2.831837424336
18407	H	-0.867798279069	-4.128260552371	1.540980006833
18408	O	3.270050381495	2.162127834552	-3.063144428338
18409	H	3.311301461371	1.833037321740	-3.999043378459
18410	H	3.971510937896	1.687581887662	-2.589959278603
18411	O	-1.640969157104	4.157949935154	-2.324546355636
18412	H	-2.379578769763	4.400387619570	-1.741117613556
18413	H	-0.854759324091	4.215000746476	-1.750605739016
18414	O	-2.937467501957	2.122748884837	-3.696061960835
18415	H	-2.371405330526	1.912826655092	-4.459086424051
18416	H	-2.452784886466	2.803694947552	-3.194635003926
18417	O	3.490647692473	-0.811122300362	2.921180478316
18418	H	2.555657656066	-0.593726016580	2.809469281634
18419	H	3.993107706452	-0.026996508545	2.598342072136
18420	O	1.057791444235	2.492129522484	-6.580657126980
18421	H	0.970578691393	3.262529955561	-6.004083471566
18422	H	0.261947596643	1.964690582763	-6.409626136087

18423	O	0.742877593145	1.292428846134	-3.170802232792
18424	H	1.609905099324	1.694134258097	-2.958370844271
18425	H	1.013191109874	0.400498023302	-3.489774022865
18426	O	2.325014717554	-2.998147547575	-2.584838768237
18427	H	2.959810265204	-3.095832445967	-1.865061883958
18428	H	1.596261942599	-3.601151691456	-2.375713431308
18429	O	-1.668581778369	-3.316074139325	4.571032809323
18430	H	-2.107101583533	-2.429422888129	4.565030881408
18431	H	-2.170460362357	-3.859814029344	5.177944811496
18432	O	0.384355400961	3.921895177273	-4.228993479897
18433	H	0.467196856773	3.016010596599	-3.885585101025
18434	H	-0.448960852311	4.246885450940	-3.867323671792
18435	O	-4.409880418547	-0.276902402290	2.208002651080
18436	H	-5.038950375044	-0.257771985744	1.452463724420
18437	H	-4.226656914983	0.653639821693	2.399366800216
18438	O	-3.178652370464	2.223416544727	2.138777105350
18439	H	-3.392893062687	3.146352903644	1.980392336611
18440	H	-2.914010658335	1.857455598370	1.268562187445
18441	O	4.427598464150	2.764521823518	0.111574151112
18442	H	4.702694834283	2.074894833966	-0.518663590124
18443	H	4.646037865430	3.613622264268	-0.325048246403
18444	O	-2.912195257118	-0.980137555487	4.485699804883
18445	H	-2.360596360150	-0.185819134502	4.502112258090
18446	H	-3.520601000416	-0.859382443991	3.742730165019
18447	O	-0.979274018885	1.075980764134	-5.308774226487
18448	H	-1.053724500521	0.130441512066	-5.464785928538
18449	H	-0.388434553947	1.168107869269	-4.536074189967
18450	O	-4.220915447810	-3.100182848381	1.258257375897
18451	H	-3.329035795735	-3.428248851895	1.486643014446
18452	H	-4.379651667947	-2.327416284940	1.812227365886
18453	O	2.440877288336	4.641212924774	-2.596047176909
18454	H	2.866884019917	3.760996218772	-2.698315577815
18455	H	1.816597518368	4.667802573813	-3.345059263603
18456	O	6.335001710285	-0.981066448772	2.011132306406
18457	H	5.760542729622	-1.482296483483	2.595182873265
18458	H	6.090989227482	-0.051536713288	2.139144116161
18459	O	-4.908461644424	1.929133967213	-1.675962946374
18460	H	-4.067451306128	1.702646568157	-1.245153115551
18461	H	-4.682610409940	2.084622482946	-2.599415580558
18462	O	3.521174226270	2.688016605699	4.309360878889
18463	H	3.539624315742	1.993469363595	4.978988733937
18464	H	2.565632197312	2.821744835946	4.128257547514
18465	O	1.476901228841	-1.024372783907	-4.235030897731
18466	H	1.916799094322	-1.688983275394	-3.673286168712
18467	H	0.597873644468	-1.392928589986	-4.437103259152
18468	O	-4.332848283398	-2.979149993345	-3.998486863661
18469	H	-4.051728508112	-2.051158791759	-3.910293202516
18470	H	-4.610753916211	-3.095585979656	-4.906874088055
18471	O	4.596198735944	5.254122338306	-0.909095147439
18472	H	4.280754351497	5.744492405084	-0.145810127573
18473	H	3.884484127330	5.248931432664	-1.566734942928
18474	O	0.918166339275	2.816256450524	3.580014237081
18475	H	1.026058958261	2.108700380673	2.933592213797
18476	H	0.843702745538	3.649568617338	3.053113248207
18477	O	5.269014330678	0.883751829463	-1.685465457838
18478	H	5.316198546509	-0.024150461874	-1.289343337551

```
H      6.122747281730    1.032647864752   -2.093718345844

Ambimodal TS Water45-20
155
ANGSTROM
C     -0.099895138169    1.401567699783    0.984300596716
C      1.070251383594    1.320480361781    0.296292096497
C      1.731739544238    0.098449496335   -0.098857041638
C      1.230111965713   -1.159101259786    0.125857507042
C     -0.516051232107   -1.219306033504   -1.331258477947
C     -1.531497806869   -0.579763288909   -0.639109546702
C     -1.785163519423    0.859058118114   -0.776999450761
O     -2.755223043451    1.450415375262   -0.249324344166
H      1.503121462166    2.239687103002   -0.091504846299
H     -0.555682545301    2.357450756608    1.199836784485
H      2.586799841065    0.210279366525   -0.762582640003
H      1.755016714082   -2.034882147406   -0.226835505100
H      0.525509123801   -1.359059036058    0.920583682555
H     -0.021524332429   -0.723612379284   -2.147276885505
H     -0.469119049864   -2.296545206371   -1.342233406064
H     -0.526436674015    0.564050333619    1.516215146239
H     -1.183204311549    1.403276469214   -1.520063823025
H     -2.978420145348   -0.771424134389    0.815019488449
N     -2.326516298822   -1.272147640435    0.238601303925
H     -2.051714191899   -2.189476151965    0.554596981551
O     -0.339187796504    1.033671948494    5.783887228099
H     -0.751898500358    0.749597156670    6.599564103403
H     -1.065390852670    1.213606076920    5.152401807051
O     -3.088637738395    3.498301034670   -2.691361832193
H     -2.126318733166    3.498475513268   -2.456036025764
H     -3.152158086537    3.950922278644   -3.533428157332
O     -1.016228214506    4.001128178697    3.447588421038
H     -1.578657021448    4.600676513625    3.940124516065
H     -0.862213242267    4.451019493410    2.573411501047
O      1.182355239754   -0.201441748464    3.834696909775
H      0.411640871293   -0.552521915798    3.363469026870
H      0.818297416650    0.218499817764    4.630852583998
O     -1.359155147822   -1.053740913226    3.217900834938
H     -2.219590787532   -1.481733573127    3.022042751261
H     -1.616486136213   -0.157134660491    3.501939788799
O      1.340205655487    4.823108632078   -0.589249150677
H      1.894225112822    4.620887930015   -1.359145418453
H      1.890688019467    4.596249164662    0.181527875313
O      2.737928017220    3.444305398684   -2.509424236054
H      3.385969323263    3.441310556335   -3.243392082384
H      2.181335741232    2.658416737667   -2.653677950279
O     -2.966374684825   -4.100220284890   -1.371809972456
H     -2.082417506906   -4.483655472726   -1.337544629175
H     -2.909495183951   -3.333414122894   -1.963993307068
O     -3.911721384990   -1.746812606616    2.762251139049
H     -4.211422458066   -2.267918212074    1.999502943001
H     -4.230467162914   -0.841447666949    2.623595770047
O      5.283010252513    0.822496629030   -2.342609330493
H      5.479890235381    0.088580977446   -1.743596407987
H      5.071487842465    1.565440036681   -1.759123236966
O     -3.368842864004    0.831451877295   -3.404027817932
```

18535	H	-3.241973232542	1.724988939198	-3.034311686434
18536	H	-4.323832352318	0.666667017993	-3.298212143522
18537	O	-3.012996019734	4.090510955098	0.122331964363
18538	H	-2.880592040964	3.124868655652	-0.019783299064
18539	H	-3.297100986580	4.422862501383	-0.733450167894
18540	O	2.293119003727	-4.972248821767	0.009249867187
18541	H	2.096755363128	-4.675818612223	0.910898504726
18542	H	2.799411392637	-4.258316684512	-0.402521566474
18543	O	-0.969609346405	-3.607842242443	1.376853513299
18544	H	-0.045990331181	-3.560956099472	1.706830477721
18545	H	-1.479305919487	-4.034101257850	2.066811692805
18546	O	2.625123730662	2.006195832729	3.105489308769
18547	H	2.262554745285	2.545964732094	3.844911810697
18548	H	2.082419918864	1.198751240751	3.135804048785
18549	O	2.177199404702	-1.550102423427	-3.240140126158
18550	H	2.557426618809	-1.971115399065	-2.448357886135
18551	H	2.975620435809	-1.316535049949	-3.783060037975
18552	O	0.098349500668	-2.956104624520	4.566114371931
18553	H	-0.510343983372	-2.335988509083	4.117644210952
18554	H	0.662637906252	-2.416429427599	5.122957046316
18555	O	-5.524598573175	-2.029060582652	-1.864616232081
18556	H	-4.704813363605	-2.159505341375	-2.367720553206
18557	H	-5.305545034826	-2.270740807783	-0.950203477022
18558	O	3.683449698175	-2.883513267514	-1.376640772083
18559	H	4.237119931990	-3.388584864336	-1.975663993180
18560	H	4.271617859596	-2.244721448639	-0.923856665636
18561	O	0.298032106190	3.544512261340	-4.861033604117
18562	H	1.186363853610	3.379829366122	-5.184811234814
18563	H	-0.179207311783	2.698271666093	-4.913838765887
18564	O	0.479080362554	-3.809879493274	-3.536155948125
18565	H	0.221172010881	-4.148673633355	-2.658650793231
18566	H	1.090383968079	-3.073970747935	-3.382398642537
18567	O	1.499177306913	1.111171143723	-3.178227695780
18568	H	0.556145563560	1.180169652333	-3.425812713511
18569	H	1.699064682698	0.169309857019	-3.019350873306
18570	O	-5.986553841120	0.399109050935	-2.737292488344
18571	H	-6.771889562439	0.332929002323	-3.280479734635
18572	H	-5.868857785952	-0.488854109050	-2.311196464045
18573	O	1.176877462592	3.286038464340	4.931543757357
18574	H	0.784012309913	2.561560116772	5.436998900262
18575	H	0.450883909929	3.620111838963	4.371228986710
18576	O	1.537537064519	-3.658210472967	2.364631098604
18577	H	1.150294239370	-3.614969376908	3.259174344981
18578	H	2.210123242623	-2.950780670913	2.344860814081
18579	O	0.484842205315	-0.995200359433	-5.556509680676
18580	H	1.071906117604	-1.285975954022	-4.845509163297
18581	H	-0.205037737314	-1.678364401204	-5.609165380919
18582	O	3.249396314150	-1.563697786897	2.604917514249
18583	H	2.634323284057	-1.092889438649	3.186802842690
18584	H	3.637352365240	-0.882852891622	2.042716841173
18585	O	-5.608917280727	2.778189710039	-1.327233688043
18586	H	-4.753273792415	3.012782758242	-1.705515355986
18587	H	-5.825597013906	1.911866918407	-1.701819384234
18588	O	4.342396702143	2.769871681578	-0.502654397988
18589	H	3.720018085886	3.018106443868	-1.227082337074
18590	H	4.163600301793	3.415537516531	0.194600688517

```
O     -4.635607797106   -3.222676063964    0.508559413830
H     -3.935499374908   -3.562583747113   -0.098096777113
H     -5.230908760621   -3.957355784475    0.661472131548
O      2.984995046264    4.129557878504    1.512129515078
H      3.042901025661    4.828668021759    2.164365109421
H      2.778305397966    3.309285965868    2.012795266731
O     -5.371415643949    3.214771660770    1.385927427793
H     -4.577780451660    3.747391481685    1.226336035878
H     -5.684067723934    3.007005205494    0.486488501010
O      4.382765716619    0.770257873121    1.333455735269
H      4.358780896934    1.452402263774    0.632494861541
H      3.961363934549    1.195639615035    2.095026579656
O     -2.137741493124    1.508429909161    3.821711117381
H     -2.929737085408    1.367707534388    3.248307728364
H     -1.760925945415    2.364651152364    3.552351047004
O      2.556275065514    1.205803429173   -5.617842013551
H      1.964622487742    0.659787941094   -6.143460802530
H      2.147338455454    1.239200370082   -4.718302751526
O      5.422468769002   -1.119610005329   -0.267254039166
H      6.161035852248   -1.510311828674    0.201838303641
H      5.017938412927   -0.481050054307    0.354401388236
O     -0.176370936882   -4.646128051739   -1.013840588887
H      0.711655698985   -4.974685882132   -0.762993034793
H     -0.519114783225   -4.270105364820   -0.180880195913
O      4.521607140880    2.847858750411   -4.427662124489
H      4.003911507288    2.357970482357   -5.079327392899
H      5.043227834682    2.201825894509   -3.938143195286
O      4.279368612350   -0.621404049600   -4.496837783497
H      4.704311963894   -0.111394597642   -3.787189505293
H      3.839118083050    0.027785029188   -5.074742744396
O     -3.035896929361   -1.935164971237   -3.117284450998
H     -2.533875162004   -2.235062887171   -3.895127331164
H     -3.039113415951   -0.966052692775   -3.146856574796
O     -0.836601812584    1.076485868726   -4.488635825085
H     -0.505529922025    0.308627776829   -4.998259238700
H     -1.751005601963    0.898173647190   -4.226571928009
O     -0.825691605226    5.253567682757    1.169990151691
H     -1.629707499624    4.900528398620    0.736077771652
H     -0.110166887307    5.187910924471    0.524684389730
O     -0.493208901043    3.503468762429   -2.236717293980
H      0.026994544408    4.009878374329   -1.596206822580
H     -0.106737472902    3.672909547192   -3.114492232144
O     -4.072189056292    0.993954494436    2.057445763513
H     -3.588675184039    1.085106550134    1.207678652826
H     -4.713622757536    1.737845656313    2.004367870754
O     -1.407809654389   -2.918545835501   -5.095338543145
H     -0.730613185505   -3.322049689969   -4.482947999734
H     -1.739297448010   -3.635200655860   -5.636265438490

Ambimodal TS Water45-21
155
ANGSTROM
C      0.217719096219    1.772519337729    0.703705741163
C      1.278789276968    1.578243887895   -0.115579673662
C      2.023446501102    0.336200918766   -0.267606515215
C      1.745558919272   -0.831557970176    0.366485093654
```

18647	C	-0.279807924843	-1.330028198986	-0.893180462750
18648	C	-1.214367573861	-0.556761739905	-0.251837503198
18649	C	-1.559261253710	0.774668765741	-0.738018776834
18650	O	-2.539227344152	1.462322364437	-0.335812641071
18651	H	1.545124525597	2.381570074713	-0.799711144676
18652	H	-0.296214250237	2.722900249947	0.738166664323
18653	H	2.782106147695	0.330129559852	-1.046156108029
18654	H	2.314918386840	-1.723893674624	0.151006662868
18655	H	1.112125260343	-0.895317990593	1.240854987904
18656	H	0.166136828274	-1.020966892003	-1.821466934612
18657	H	-0.123584432160	-2.358143345242	-0.607775418479
18658	H	-0.050111823618	1.076623801203	1.485350672025
18659	H	-1.053461291776	1.106019117301	-1.659277194001
18660	H	-2.641371342701	-0.538139119083	1.254902559760
18661	N	-1.794411872696	-0.977148690614	0.941855330142
18662	H	-1.693653338865	-1.946839751423	1.191908433397
18663	O	2.896050828600	1.095716047157	3.351788011337
18664	H	2.200981753796	1.554476838882	3.854454837130
18665	H	2.986870223659	1.515697269007	2.483644741019
18666	O	-1.013288664471	1.512528663187	-4.391660880442
18667	H	-1.497414653977	2.167041503396	-4.896501714492
18668	H	-1.691323106928	0.893951503411	-4.036651379130
18669	O	5.405036387389	0.075731986120	1.492024058267
18670	H	5.681440139613	0.233081842067	2.412937912789
18671	H	4.849417882588	0.842847081882	1.261122868984
18672	O	-0.574999877930	-3.608203736664	1.874822251348
18673	H	-1.498948944074	-3.909714143049	1.948396574338
18674	H	-0.200593229187	-3.972131737025	1.052262057225
18675	O	3.896369126964	2.297889712920	0.976757279327
18676	H	3.901272415854	2.346854721424	0.001354498358
18677	H	3.950891446410	3.215746870213	1.281559275799
18678	O	-4.155526945154	-4.576706295558	-0.431467010040
18679	H	-3.261612960396	-4.821572732414	-0.784152555907
18680	H	-4.640447490373	-5.401316179589	-0.386068926057
18681	O	2.339177544997	-1.594189921112	3.852011332487
18682	H	1.368295995112	-1.559427882836	3.872243179029
18683	H	2.624546122634	-0.678125597661	3.708374849127
18684	O	-2.954747703745	4.275087622069	2.466692628806
18685	H	-3.311802766506	5.060495424091	2.881413682020
18686	H	-2.064607671619	4.128085376638	2.848759297300
18687	O	1.775793584466	2.013879349483	-3.469702766895
18688	H	0.879975875683	2.302233044916	-3.665035570115
18689	H	1.684711415378	1.047817627463	-3.262552587854
18690	O	-2.905429058148	-2.377332821299	-2.043927659172
18691	H	-2.065115408440	-2.794612601706	-2.361124474217
18692	H	-3.599431408859	-2.965398259796	-2.353422741865
18693	O	-1.794639529085	-5.344681368731	-1.360718484750
18694	H	-1.569793108004	-4.911101924425	-2.199532970081
18695	H	-0.980960697018	-5.284394554539	-0.837650753743
18696	O	-2.260317013272	4.182108359940	-0.255081350276
18697	H	-2.405084386942	3.236608299887	-0.438917495790
18698	H	-2.575115576296	4.314516439714	0.652890001419
18699	O	-0.409822305353	-1.551130952424	3.635084997956
18700	H	-1.183621832783	-1.821762813236	4.170482859482
18701	H	-0.392757907968	-2.187855690061	2.898772937713
18702	O	3.890884876544	2.671803275311	-1.783746065397

18703	H	3.782759083999	3.634816015946	-1.648204139981
18704	H	3.173244315863	2.437949443707	-2.397045290459
18705	O	-2.958539062050	-0.174130587224	-3.616238589924
18706	H	-2.820805908324	-0.948802328788	-3.034971538598
18707	H	-3.715866182335	0.280861827874	-3.201568735367
18708	O	-0.501044030713	3.617431497077	3.405026494236
18709	H	-0.089962371841	3.677486564636	4.271995382086
18710	H	-0.652604416334	2.660046846763	3.272107060276
18711	O	2.320042738241	-2.789500408362	-5.307517763223
18712	H	1.690302696568	-2.217792683498	-5.756917209833
18713	H	3.108068366585	-2.253997341027	-5.136342338647
18714	O	4.143128920332	-1.052168631590	-4.047021876647
18715	H	4.220350677187	-1.688599148084	-3.302056856868
18716	H	4.737587412950	-0.316734252151	-3.765421375569
18717	O	4.032538247627	-5.070019531024	-1.482129728425
18718	H	3.235228372431	-5.172887428160	-2.011380515809
18719	H	3.760857507709	-5.153420755729	-0.553889590192
18720	O	0.907219139940	1.812368417070	5.125315505928
18721	H	1.160585950517	1.228823272998	5.842031101509
18722	H	0.125782771611	1.408720404445	4.708057684617
18723	O	-1.176295617799	1.002297947207	3.537883109627
18724	H	-0.919051539159	0.058886356542	3.461181709582
18725	H	-2.068238419239	1.078163830568	3.148464170916
18726	O	5.636361222667	0.821182991911	-2.905960874005
18727	H	5.952369755588	0.277005907006	-2.169072234248
18728	H	5.122467031024	1.543751960172	-2.508975055172
18729	O	-3.275826315141	-3.773960484510	2.028259795063
18730	H	-3.625559492774	-4.085196500731	1.172015048370
18731	H	-3.684078647568	-2.913324592134	2.185680389557
18732	O	-1.619710487391	-2.054088511531	-5.374752587550
18733	H	-0.858198122421	-1.556964334516	-5.704704279838
18734	H	-2.218805851651	-1.398894688194	-4.994212825361
18735	O	0.686740441731	-4.581525825228	-0.348397914634
18736	H	0.977137692233	-4.275767113088	-1.219813018688
18737	H	1.521318168191	-4.697469706045	0.165689138053
18738	O	5.532702520194	0.957853774420	4.061208550682
18739	H	4.561287281614	0.968796169975	3.961102865050
18740	H	5.805643903268	1.867226030947	4.186688191816
18741	O	0.516088615893	-0.326410364071	-5.772073582744
18742	H	-0.036182297225	0.387418094625	-5.400815053981
18743	H	1.360309570727	0.119130494611	-6.014242925097
18744	O	6.240241393571	-0.963286053781	-0.809814759973
18745	H	7.024788192990	-1.487204103749	-0.646265387831
18746	H	5.984797470824	-0.563064647207	0.047411356697
18747	O	2.858949743099	0.940577977321	-5.797626105135
18748	H	2.639787216397	1.531712609021	-5.054716187179
18749	H	3.536930005383	0.342961241052	-5.463121700962
18750	O	4.064273781712	-2.393697966251	-1.752045191468
18751	H	4.240019757260	-3.351464587982	-1.645410620877
18752	H	4.783813278219	-1.922987327572	-1.301366848875
18753	O	3.628613009921	4.958221775697	1.702878976429
18754	H	4.084335014625	5.685741743782	2.125602623546
18755	H	2.658851912524	5.101086306943	1.863326990767
18756	O	-4.796413368939	0.684892485641	-1.793320518564
18757	H	-5.055037042274	-0.084643900784	-1.270038709505
18758	H	-4.148274300362	1.159520876774	-1.253279559462

```
O      0.176906103370    5.166597442191   -0.672700572485
H     -0.712073306817    4.747410110239   -0.568973659818
H      0.017755140085    5.995396553716   -1.126752761030
O     -2.823431138832   -2.287180106443    4.570017047364
H     -2.929048536681   -3.200788384740    4.290269715505
H     -3.442469793428   -1.774203533580    4.030484894360
O      1.659771693871   -4.450101912707    3.389434684696
H      0.812675609673   -4.198232213946    2.989702772335
H      2.047130864779   -3.635266439271    3.729626337456
O      1.045459110442    5.372817606901    1.912175953132
H      0.734356225887    5.324577368955    0.987710727302
H      0.524305746542    4.719292035162    2.404119564713
O     -3.484623901487    1.525690453944    2.184942028931
H     -3.564586010175    2.469875252321    2.376887251858
H     -3.171494365916    1.489602705157    1.253049925945
O      4.066361087653   -2.363132338859    1.789529478603
H      4.559490178336   -1.545165270255    1.624082358717
H      3.512444245471   -2.178414321867    2.563961264974
O      2.956936784135   -4.700832043076    1.036583834003
H      3.436702736472   -3.858147371961    1.158079670372
H      2.589681909704   -4.875519489032    1.926953196955
O     -4.407821904795   -1.088135861952    2.510520933390
H     -4.287583709386   -0.131214833900    2.599134362754
H     -4.712827059113   -1.225401329396    1.595383322952
O      1.729099778953   -3.552665992179   -2.765458629381
H      2.259949076033   -2.875765342338   -2.318244481579
H      1.954796674242   -3.450755100831   -3.712276572351
O      3.065623496132    5.118123977081   -1.027953667972
H      2.103267146606    5.041918690145   -0.998473819509
H      3.358799985247    5.134484427096   -0.102119308934
O     -0.940978570062   -3.576907505060   -3.273568652103
H      0.016341739064   -3.488688695992   -3.106002204152
H     -1.122570542471   -3.153257004009   -4.139346050444
O      1.599023737969   -0.597197458422   -3.294128894082
H      2.539085467305   -0.776986471308   -3.508556088410
H      1.115147153509   -0.709328708728   -4.132229072612
O     -4.739270653154   -1.726283489509   -0.141587343935
H     -3.903700901560   -1.817681494370   -0.634043485783
H     -5.116581994013   -2.611242864320   -0.167409962195

Ambimodal TS Water45-22
155
ANGSTROM
C      0.199197596914    1.718029649612    0.711691113366
C      1.290604256589    1.567178398417   -0.084620031904
C      2.044155948390    0.347151224773   -0.267870431902
C      1.706668518939   -0.853260312142    0.297209212611
C     -0.175450895216   -1.433271209656   -0.883201878396
C     -1.187743613519   -0.708366652512   -0.281293083188
C     -1.532595446026    0.648602755346   -0.712771172327
O     -2.506514339457    1.290432032697   -0.254654974935
H      1.571463978759    2.393907061787   -0.734765478874
H     -0.330708976694    2.659596065940    0.755379216676
H      2.814943655073    0.369357777218   -1.033566069256
H      2.276590205243   -1.745850994243    0.089227124986
H      1.083140679168   -0.911066302691    1.174338722802
```

18815	H	0.264607749058	-1.098476362786	-1.803685915754
18816	H	-0.050884798417	-2.479250068233	-0.653828242042
18817	H	-0.072563876657	1.001201072272	1.473173304886
18818	H	-1.022593216446	1.032645799044	-1.612369626346
18819	H	-2.684114724613	-0.661510667863	1.132473289810
18820	N	-1.885012679245	-1.180808913540	0.801385215365
18821	H	-1.810245950612	-2.143128949363	1.091027362914
18822	O	-0.710078063440	3.638653601554	3.263385867578
18823	H	-0.921577861661	4.306205034373	3.917052751190
18824	H	-0.409782254117	2.853142382571	3.770183525540
18825	O	-1.194973167883	4.915463939432	0.426887248069
18826	H	-0.467389095029	5.107567354044	-0.207876048683
18827	H	-1.308995383055	5.702967595379	0.957165173431
18828	O	1.425135118063	-0.186095618831	6.091960367952
18829	H	2.122186425843	-0.138598558544	5.426778794297
18830	H	1.041882195931	-1.076898921477	5.997903612784
18831	O	-1.780440280917	1.673710070056	-4.973786068104
18832	H	-2.699899238661	1.764858520327	-4.685893704346
18833	H	-1.534564533058	0.757996104847	-4.753855483952
18834	O	-2.801590469490	3.656790028647	-1.339598746479
18835	H	-2.733926228406	2.756310296595	-0.929858387874
18836	H	-2.336622066921	4.236363694474	-0.705989851112
18837	O	-2.797203312857	-1.006551607677	3.800350369746
18838	H	-3.466496734945	-0.872743997239	3.106702661609
18839	H	-2.867429413967	-0.184385222729	4.348228573958
18840	O	4.660722178631	0.261042414446	-2.693883597840
18841	H	4.348753706547	0.159745119138	-3.602410123960
18842	H	4.508782252917	1.199736304789	-2.477872679543
18843	O	3.744403071288	0.862809526773	-5.333491548727
18844	H	4.212727546563	1.697210326718	-5.266880683679
18845	H	2.924093793757	1.049507022630	-5.804192257425
18846	O	-2.884262908041	1.421272316645	4.788940662126
18847	H	-1.925420431631	1.528732679000	4.933617544380
18848	H	-3.071032181002	1.839063214139	3.931764330906
18849	O	1.722939211089	4.416533322852	2.168950696531
18850	H	0.802695957436	4.156712903945	2.343467655661
18851	H	2.276242627627	3.818996952070	2.715076185889
18852	O	5.070822318206	-1.681587009130	-0.929115017849
18853	H	5.971988817578	-1.937517281556	-1.128262606233
18854	H	4.832924066959	-1.004630530519	-1.604104587381
18855	O	0.498351788267	-2.733574663595	5.423029340376
18856	H	0.915381721071	-2.978774755649	4.586772524454
18857	H	-0.432205861603	-2.964498781914	5.297315878590
18858	O	2.575859259990	0.001165634210	3.487036487888
18859	H	3.139338742354	-0.242890366456	2.739821610642
18860	H	1.661476061636	-0.180015482320	3.230055169810
18861	O	-0.163291804841	1.430870799307	4.680926332876
18862	H	-0.172544445639	0.714584670191	4.020268608517
18863	H	0.394736424560	1.057172305612	5.396475927719
18864	O	3.402893204607	-3.745009120555	-0.076252238074
18865	H	4.047258868994	-3.123288316545	-0.454583108179
18866	H	3.601581764974	-3.746991343408	0.869207856818
18867	O	-1.854159027085	-3.989802555037	1.685957735099
18868	H	-1.010798612689	-4.334507052573	1.369583494311
18869	H	-1.797901116934	-3.902490540819	2.655618248835
18870	O	-2.036241750279	-3.456831550797	4.382155902784

18871	H	-2.708186248568	-3.884477190478	4.912782376697
18872	H	-2.354402327691	-2.536193725142	4.220285823411
18873	O	4.557498852242	-0.473114365511	1.497323612625
18874	H	4.726976952593	-0.888332578534	0.631887892077
18875	H	4.600556106021	0.482800952674	1.326739758423
18876	O	-4.131220047356	-3.660957784705	0.250962070626
18877	H	-3.320791696470	-3.759272952367	0.790505742519
18878	H	-4.765889017252	-4.293404216466	0.587769389077
18879	O	1.647910067009	-1.267726994803	-5.913787749068
18880	H	1.148705134942	-2.018544917978	-5.557511028368
18881	H	2.512883877324	-1.598561577494	-6.159759467034
18882	O	-6.012399848084	1.387528823728	-1.538346575260
18883	H	-5.857964065594	0.504716776840	-1.181283372949
18884	H	-5.806353761623	2.012800725703	-0.819548352059
18885	O	-3.065648629000	-0.647462557776	-2.628032854636
18886	H	-3.468681276851	0.204334196977	-2.895432840895
18887	H	-2.246234430740	-0.714513771023	-3.154620964643
18888	O	1.720447706638	2.562647972931	-3.743707736479
18889	H	1.511579131202	2.362076046351	-4.674553202192
18890	H	0.881454099968	2.889639663491	-3.368688671299
18891	O	3.345232465615	2.625113965966	3.345350556078
18892	H	2.992627203554	1.736547848685	3.538447424280
18893	H	4.062816292363	2.494192059579	2.719100631531
18894	O	-4.912364414802	-1.084946512252	-0.592689234831
18895	H	-4.208470071590	-0.889512584362	-1.231835027698
18896	H	-4.779130158174	-2.005417413348	-0.324148535186
18897	O	4.013048449640	2.885746104318	-2.310098461657
18898	H	3.250956292743	2.845749875555	-2.913506402293
18899	H	3.698288039494	3.396284421649	-1.553042861505
18900	O	2.688275533626	-2.629928449643	-2.845091558184
18901	H	2.515456037412	-1.689782387764	-2.687579010687
18902	H	2.740737498534	-3.059849981827	-1.987309107626
18903	O	0.447975509530	-3.182458460784	-4.237416395161
18904	H	-0.042846369230	-3.750345616921	-3.622203273979
18905	H	1.300359872725	-3.032238488746	-3.772896078088
18906	O	0.884737080966	1.453827925946	-6.095029782228
18907	H	0.917622360786	0.487370811258	-6.158118941116
18908	H	-0.049691474898	1.677435995131	-5.991507204545
18909	O	1.181214307472	-3.131889862061	2.725078816563
18910	H	0.934370985158	-3.625752979477	1.930930688950
18911	H	2.147284648425	-3.028775321592	2.680943855085
18912	O	4.655022319887	2.193772454704	0.711507062365
18913	H	5.165284636558	2.123946470970	-0.099153503135
18914	H	3.958341541109	2.847269647164	0.521825187735
18915	O	1.653943220782	-0.096882240341	-3.377154227796
18916	H	1.777829476972	0.873188659199	-3.357934692041
18917	H	1.942790788144	-0.383222760975	-4.259058689468
18918	O	-5.057926161212	3.210936649741	0.332712935257
18919	H	-4.481271126401	3.050654073071	1.094669138236
18920	H	-4.486859504379	3.615386072534	-0.335807356690
18921	O	-0.184725959119	-0.755014197090	3.051064120523
18922	H	0.189169823864	-1.652896205721	3.049769643758
18923	H	-1.138865133146	-0.873351174259	3.235450897322
18924	O	-0.828752072891	-0.761991197325	-4.142379043323
18925	H	0.011266861902	-0.409169103997	-3.776639338225
18926	H	-0.622985836364	-1.699059890213	-4.311598909313

```
O     -3.013641163192   -3.430378372219   -2.261820907732
H     -3.076365704138   -2.485181822703   -2.468531491350
H     -3.473051279120   -3.551387110051   -1.415322662786
O     -4.475410163111   -0.129505521549    1.877801207209
H     -4.754567107410   -0.376346229881    0.973448850088
H     -4.525359649407    0.827592919251    1.938634481767
O      2.983642654112    4.325036070897   -0.069897601810
H      3.501745673797    5.130452235252   -0.015339930677
H      2.414981209232    4.334463749887    0.763233705712
O      3.922779564523   -2.899274799709    2.597078558082
H      4.302161957741   -3.013174541828    3.469475617256
H      4.192842844366   -2.015008520166    2.290663559579
O     -0.734750530030    3.329383224316   -3.045299648825
H     -1.489298877442    3.504456489444   -2.441492659783
H     -1.121039556442    2.848736586251   -3.802447186189
O     -3.009284682510    2.467746040314    2.160670515136
H     -2.239065316751    3.024856238559    2.362622195000
H     -2.754693233380    1.946581556916    1.377452307316
O      0.663137198310   -4.543668226993    0.309360980088
H      1.598206074680   -4.430676433667    0.086554863885
H      0.201722199215   -4.625197554307   -0.545367836549
O     -0.688779545948   -4.636837156314   -2.131711306961
H     -0.910233341483   -5.533901906683   -2.384519792884
H     -1.546528188601   -4.134309373337   -2.146533887911
O      0.682875421319    5.242148242254   -1.454600765440
H      1.493548098556    4.826790184055   -1.123873973776
H      0.308899093184    4.662907758081   -2.132365224172
O     -4.022458981448    1.834617952439   -3.250854670577
H     -3.638760572057    2.579922946235   -2.772071178787
H     -4.874590396596    1.674506177623   -2.779535341061

Ambimodal TS Water45-23
155
ANGSTROM
C      0.226161163621    1.670854063464    0.782996717594
C      1.329974539743    1.537336267302    0.005253197250
C      1.984477529930    0.283500467242   -0.288628248887
C      1.611779960511   -0.925385290242    0.231761882654
C     -0.334165530778   -1.384015676190   -0.850578455316
C     -1.273983012425   -0.567180614296   -0.245060404995
C     -1.590218545688    0.761379129731   -0.776395678233
O     -2.486142417465    1.512490840127   -0.350473450023
H      1.708080215707    2.406088536264   -0.530941578912
H     -0.272562807936    2.620735795983    0.895868255469
H      2.691600775956    0.304415262601   -1.109473407715
H      2.119227918620   -1.830187813221   -0.067760294024
H      1.068245794054   -0.994491816916    1.162179748787
H      0.053010785891   -1.139626627179   -1.825701140411
H     -0.243854649970   -2.418848312371   -0.556284525883
H     -0.125618557776    0.896234875538    1.448375337230
H     -1.078074513831    1.035794870036   -1.716566777230
H     -2.420155755720   -0.226035087462    1.419731737506
N     -1.952857328411   -0.945884932441    0.890106739806
H     -1.640489371188   -1.734167013636    1.434757863550
O     -2.402481977745   -1.874455184540   -3.140078536052
H     -3.165757808457   -1.272347810508   -3.281789568017
```

18983	H	-2.646558855612	-2.407716050406	-2.368371485921
18984	O	-1.296790521851	-4.868221236122	2.276044263855
18985	H	-1.348488322566	-4.044616366238	2.789018602423
18986	H	-0.536108304294	-4.770875932342	1.691013037192
18987	O	0.150687209059	2.200202723777	5.548124039749
18988	H	0.929802401692	2.257652818417	4.964691363351
18989	H	-0.506130911793	2.785873676338	5.144107460502
18990	O	0.876547134058	4.518731813685	2.104779944807
18991	H	0.233332344905	4.745037020867	1.409254363852
18992	H	1.749274633048	4.646726524677	1.686120211798
18993	O	-0.120188448301	3.652609085905	-2.000906334273
18994	H	0.230114591905	2.757292856074	-2.102736689553
18995	H	-0.760571810302	3.773727971542	-2.738594756504
18996	O	3.860782319142	-0.133674312884	4.084239609216
18997	H	4.276439350533	-0.683021366980	3.392864771947
18998	H	4.115161668777	0.773952802558	3.902809703954
18999	O	-1.729110690294	-2.397315929359	3.391512023556
19000	H	-2.680091031291	-2.237532440438	3.195773757065
19001	H	-1.487829487337	-1.795219568027	4.098633250013
19002	O	2.319237632088	-3.713856953229	-1.671423078621
19003	H	2.993404040768	-3.027617668921	-1.787546994385
19004	H	1.621632902096	-3.552254765006	-2.322671900130
19005	O	5.657816326151	-3.405800519020	0.090523277302
19006	H	5.228990676532	-4.256486406281	0.195486369031
19007	H	5.390897571093	-2.857818747746	0.853793233735
19008	O	0.324824815489	1.112232051500	-6.736443567621
19009	H	1.097883327538	0.663125479818	-7.082485349870
19010	H	-0.083312223153	0.499431772030	-6.106972447560
19011	O	1.827166428656	2.227111227910	3.356388337389
19012	H	1.397405584369	1.413329509662	3.050713995323
19013	H	1.405320866747	2.979774098958	2.901030470045
19014	O	3.263619613971	4.106585285097	-3.696290491814
19015	H	2.532059941687	3.801270464983	-4.247890308797
19016	H	3.704193441446	3.315087477500	-3.346156136952
19017	O	2.160133839115	-2.921162763278	2.618711415516
19018	H	2.289162030674	-3.456849017295	3.402363426872
19019	H	1.711779121172	-2.090726972682	2.914223504756
19020	O	-5.483411255298	2.567529335749	1.309199389031
19021	H	-6.152519165594	3.209171171994	1.546683000505
19022	H	-4.618212277852	3.021931886889	1.360386466996
19023	O	-0.772189520944	-0.290770952485	-4.571256611673
19024	H	-0.232885436609	0.246367160635	-3.930823477814
19025	H	-1.330832106317	-0.891565061558	-4.039320108114
19026	O	-3.839154878390	-4.487534601370	1.365093385994
19027	H	-2.973907042839	-4.775519857468	1.717649897281
19028	H	-4.139659354511	-3.787523730908	1.957659118806
19029	O	-0.900447045893	-5.256168433902	-1.221606091205
19030	H	-1.213477675757	-6.125343856045	-0.967933463637
19031	H	-0.262566001868	-4.983265189335	-0.537837742992
19032	O	0.968145225313	-4.366218189992	0.570548081934
19033	H	1.544388575549	-4.203574194783	-0.203947631308
19034	H	1.362506810609	-3.849674160728	1.292526764068
19035	O	-1.925338208024	3.783790613957	-3.937076239035
19036	H	-2.633613836182	3.829160140260	-3.261293088959
19037	H	-2.162355789329	2.996918120856	-4.460354338928
19038	O	0.837808739763	1.208381038525	-3.144461365919

19039	H	1.650896469175	0.669257419962	-3.196163667644
19040	H	0.952951742808	1.928041855731	-3.805663942253
19041	O	4.499350050339	-1.661143490308	1.877187526814
19042	H	4.404991053935	-0.966600710690	1.203602627337
19043	H	3.633474200731	-2.100535492416	1.979581985574
19044	O	-4.272278135226	-1.908578183241	2.608610108962
19045	H	-4.347829954163	-1.725335698627	1.654414784512
19046	H	-4.542268705910	-1.062529005705	3.022227150431
19047	O	3.206707486499	4.680382120014	0.686843734434
19048	H	2.792580437267	4.852149830291	-0.210828387671
19049	H	3.808899806128	5.409775182553	0.834329865279
19050	O	-4.284002668975	0.008101289393	-3.476473847124
19051	H	-3.906339744056	0.694220096664	-4.042961816368
19052	H	-4.648821294223	0.450693646311	-2.695383194443
19053	O	-1.017283966922	4.990136506267	0.118182545155
19054	H	-0.739463020570	4.426704934852	-0.639941883836
19055	H	-1.081424555958	5.880599992142	-0.228601652100
19056	O	-3.664967174692	3.404005079517	-1.903868223794
19057	H	-3.185203231989	2.769101270543	-1.332550939454
19058	H	-4.522141462140	2.989304062176	-2.032289735223
19059	O	-4.686733604771	-1.485698322127	-0.113587587691
19060	H	-3.921435091310	-1.979729875025	-0.452448886409
19061	H	-5.462062975375	-1.995798422754	-0.355496048718
19062	O	-1.961224895041	0.932501875782	3.176714990393
19063	H	-1.620327575384	0.415107133771	3.916763272910
19064	H	-1.710379036735	1.855314069789	3.351226543986
19065	O	1.291818125461	-0.559498678036	3.475729390947
19066	H	0.664304883168	-0.578437363978	4.212924925841
19067	H	2.180777151759	-0.359938213713	3.846352377968
19068	O	2.936851522150	-0.459131322290	-3.619279007157
19069	H	2.472645164954	-1.033659675790	-4.265834283882
19070	H	3.452033339654	-1.054499076336	-3.055611194521
19071	O	-2.978365399216	-3.462289502070	-0.930829456571
19072	H	-2.229992909509	-4.051529724569	-1.120250525275
19073	H	-3.387693314634	-3.867361422892	-0.128759502192
19074	O	2.082727349894	5.225690425761	-1.585983657326
19075	H	1.249355165314	4.740834037967	-1.717492217307
19076	H	2.633972417064	4.958395459719	-2.355193843311
19077	O	4.303440700930	1.769605629252	-2.607550435215
19078	H	3.952360570513	1.023555062069	-3.113611569879
19079	H	4.554476938997	1.426870314191	-1.742164442896
19080	O	-4.680434681007	0.664790413945	3.286221202629
19081	H	-3.723088913640	0.852193620839	3.255184086852
19082	H	-5.073952950433	1.200588151477	2.583004199882
19083	O	-2.936794588916	3.509652949914	1.417618285556
19084	H	-2.746319970543	2.669424577293	0.959516603551
19085	H	-2.474024813565	4.180209641130	0.888047430983
19086	O	4.381523477105	-1.799231041541	-1.661576263059
19087	H	4.967870431864	-2.459120540870	-1.241546575198
19088	H	4.516473002957	-0.995405093056	-1.141088049667
19089	O	4.043335058870	2.270413583685	1.737420498928
19090	H	3.854877360942	3.102820997419	1.270138556715
19091	H	3.301292802097	2.178497232343	2.364777899973
19092	O	-2.651685605177	1.502261566272	-5.251434455866
19093	H	-1.973612090788	0.821257233212	-5.042291470312
19094	H	-2.750003758793	1.508645469626	-6.203800739699

```
O      -1.318957523397    3.584060270231    3.609927596409
H      -0.541127188329    4.027452718153    3.243502967889
H      -2.025053913315    3.712567923089    2.947416769607
O       4.938778934516    0.451856897091    0.043748253394
H       4.553510844114    1.136541668523    0.648260472727
H       5.889789827893    0.508640634731    0.156989029542
O       1.327906967392   -1.980396638962   -5.144675844444
H       1.087068717850   -2.786652162642   -4.669681105189
H       0.530704869630   -1.424965077054   -5.087142979427
O      -5.269919693102    1.074741821735   -1.083740449053
H      -5.016699298392    0.219543856538   -0.700513164467
H      -5.392991434209    1.646547406550   -0.311400820234
O      -0.697493450625   -0.272576588200    5.395832154582
H      -0.974353634393   -0.596325613063    6.253344552055
H      -0.378198568001    0.662061819780    5.544802781393
O      -0.032313612797   -3.567106631144   -3.291591494680
H      -0.817932149340   -3.003306534991   -3.320400388140
H      -0.299160242215   -4.334411655889   -2.766463263652
O       0.835128360298    3.138960099007   -4.988688165620
H       0.028805977841    3.645863029641   -4.836214858877
H       0.665893394658    2.565879339583   -5.761402545218

Ambimodal TS Water45-24
155
ANGSTROM
C       0.104194846776    1.627583033707    0.853607747168
C       1.170126164281    1.612877647911    0.012016745752
C       1.987177776893    0.448480344547   -0.294222298378
C       1.837972860646   -0.776809565298    0.270691761681
C      -0.270739340576   -1.497112953786   -0.782352699556
C      -1.221575642233   -0.705337894284   -0.183521768104
C      -1.503700867609    0.628109262082   -0.709791310477
O      -2.496198149929    1.339673861235   -0.380608624711
H       1.386792930326    2.512155435914   -0.561757195319
H      -0.467586046442    2.533231533881    1.006284179744
H       2.688804365454    0.566501930279   -1.118820189665
H       2.441453734465   -1.612543320106   -0.047880080935
H       1.268999332352   -0.940397831941    1.172702333066
H       0.166963718660   -1.217756334951   -1.721899980343
H      -0.110085900331   -2.514605562087   -0.461955240372
H      -0.100965265162    0.829251111157    1.553542312013
H      -0.942942935592    0.932329345455   -1.606299423026
H      -2.735646554645   -0.609503446751    1.211366094775
N      -1.870431236914   -1.075477961505    0.982065083289
H      -1.803877304101   -2.048873762682    1.250389757494
O      -2.492808860834   -3.203501942907    4.015414658636
H      -3.337313188862   -3.222297142196    3.544479278335
H      -2.305953024459   -2.261234979631    4.182864642558
O       0.435265327128   -1.306061964650    3.138672144624
H       1.197636295742   -0.793716751312    3.466850824592
H       0.441617978647   -2.147008434305    3.634375857392
O      -4.561652477144   -2.870776041447    2.161794455625
H      -4.304030631059   -2.709736468493    1.235684373530
H      -5.453822078665   -3.217381132608    2.133464108450
O       1.253124262273    0.553323470320    6.391815241112
H       1.909040516931    0.410374403690    5.697655635118
```

19151	H	0.642837834929	1.215778133789	6.034843776005
19152	O	-0.493592358546	-1.481736785128	6.414063504504
19153	H	-1.176990146050	-1.056963785609	5.883650295958
19154	H	0.190471944443	-0.791570263252	6.557322004052
19155	O	-0.325740144643	4.960780083017	-0.160496683855
19156	H	0.337026736276	4.984465855698	-0.862123501127
19157	H	-1.141770865310	4.661013578273	-0.592234138440
19158	O	5.786794336053	-1.165465583202	-0.243711389161
19159	H	5.932864301819	-0.368864674479	-0.795614390108
19160	H	5.464840248889	-0.830057190917	0.604144329852
19161	O	-6.046197572915	-0.379013538160	0.552053232631
19162	H	-5.860854315154	0.338305350223	-0.088384626339
19163	H	-5.673098058943	-1.176096147272	0.157508400108
19164	O	0.476889291345	4.748032201127	2.450420407122
19165	H	0.279516538661	4.887458520609	1.507341828617
19166	H	-0.378585203007	4.564016642784	2.864327520307
19167	O	-0.732569085079	-3.221261493943	-3.853133652162
19168	H	-0.144790204949	-3.955340733752	-3.617812061928
19169	H	-1.483555745877	-3.297742168197	-3.242006545977
19170	O	3.207895969050	2.562936902832	-3.469302412030
19171	H	2.275083904592	2.284189603521	-3.491461022431
19172	H	3.715289916928	1.775585932100	-3.719341685262
19173	O	-1.078756562012	-5.381608743394	-0.423735683635
19174	H	-1.260293525255	-4.909188934278	0.415461757437
19175	H	-0.139705986492	-5.235894581783	-0.588434768323
19176	O	0.519352994088	-0.836113807426	-3.956316198949
19177	H	0.032218216273	-1.687354440828	-3.983778120939
19178	H	1.389209183531	-1.063542503572	-3.591412863371
19179	O	2.647232909046	0.073719435945	3.982769977700
19180	H	3.421384530002	-0.116030299840	3.425680469025
19181	H	2.370350336180	0.970434520696	3.718049378686
19182	O	4.781859345751	-0.153664060095	2.229679430401
19183	H	4.478335257964	0.752189261419	1.928575869157
19184	H	5.609918753178	-0.012178872812	2.691962277110
19185	O	1.611518194207	2.473704486562	3.246572894372
19186	H	1.364668472317	3.369158249154	2.923436324633
19187	H	0.851331323446	2.235030785870	3.810387266341
19188	O	-0.588511758618	1.877638061276	4.777801342724
19189	H	-1.108574176137	2.638143783959	4.472387956540
19190	H	-1.113790620922	1.082613300728	4.571324318922
19191	O	-4.356705466029	-0.768614939065	-2.846707750538
19192	H	-3.531897444375	-0.441354390145	-3.277012989971
19193	H	-4.823835452974	-1.259135220206	-3.523641514688
19194	O	4.057298025837	-3.132686584294	-0.578134096424
19195	H	4.780923687006	-2.465551124413	-0.636660862419
19196	H	4.029928840287	-3.314207234468	0.371109685103
19197	O	2.388125832328	-3.672861312126	-4.789530119496
19198	H	1.985062591218	-4.386749383685	-4.267656196918
19199	H	1.672608317705	-3.280294206234	-5.296088792624
19200	O	-2.260381442211	0.197075425469	-4.129884749999
19201	H	-1.362223546106	-0.152981899435	-4.124273990635
19202	H	-2.196319074790	1.164804462943	-4.088505955496
19203	O	-1.904017886851	2.945462287334	-3.887969048166
19204	H	-2.048519974280	3.648639768840	-4.521235676324
19205	H	-2.156747983868	3.314300318127	-3.003061887812
19206	O	1.735427382783	-4.664982016803	-0.604089000550

19207	H	1.592532664772	-4.567735672834	0.354737628309
19208	H	2.566385995535	-4.194302930505	-0.770274157434
19209	O	2.933772330194	-1.849257809523	-2.902104118156
19210	H	2.880286441020	-2.555292831107	-3.581841450987
19211	H	3.276379391166	-2.268124672834	-2.098846403385
19212	O	-4.270487150325	-0.135200106356	2.499767986486
19213	H	-4.364178293127	-1.044285093379	2.815672793481
19214	H	-4.983310488095	-0.079663565791	1.817651653804
19215	O	-4.882925628828	3.955442815511	-0.204615750399
19216	H	-5.287208132670	3.198543129947	-0.649123835599
19217	H	-4.566271453336	3.615497366022	0.645684313348
19218	O	1.290472788843	4.495653828530	-2.394872579775
19219	H	2.107474852521	4.537004281393	-2.896309214495
19220	H	0.919035973473	3.623746257346	-2.577914855750
19221	O	0.964625373246	-5.316608152779	-3.097681104786
19222	H	1.267471741785	-5.076564909245	-2.199918762898
19223	H	0.767629048964	-6.253601994268	-3.077419890106
19224	O	0.280048094854	-3.428180508770	4.836921856814
19225	H	0.040693673928	-2.766108184576	5.539334579433
19226	H	-0.551747750865	-3.880140280623	4.663977505137
19227	O	-2.657761345312	-4.057860725215	-2.035627580323
19228	H	-3.199394736421	-4.719596730613	-2.466877712077
19229	H	-2.042996608870	-4.558542418586	-1.433221756125
19230	O	-1.234015172866	-3.782391843399	1.704668799292
19231	H	-0.276581915200	-3.709957075344	1.859735922904
19232	H	-1.662297337799	-3.714412767959	2.579716678398
19233	O	5.925651767123	1.107291026400	-1.664904409138
19234	H	5.360166632645	1.801969026299	-1.291739131339
19235	H	5.492352509685	0.824528515054	-2.485466417186
19236	O	4.468400679802	0.125334173318	-3.834550546919
19237	H	3.903805365677	-0.586104077101	-3.449814131706
19238	H	4.972160843756	-0.277912953658	-4.541627400337
19239	O	-5.069280734177	1.347731291874	-1.270125074458
19240	H	-5.023765422882	0.722370841450	-2.009900763019
19241	H	-4.158917334274	1.373097082011	-0.925520672007
19242	O	-1.864123144729	-0.464403465896	4.061402396410
19243	H	-2.526371465759	-0.208660729366	3.408550764144
19244	H	-1.041140173048	-0.726990398053	3.567397740838
19245	O	3.946295053625	2.224244017333	1.670955345440
19246	H	3.107574351113	2.410863515202	2.117986615515
19247	H	3.856477035381	2.500646678007	0.745865707600
19248	O	0.562968017853	1.869365148792	-3.364555919614
19249	H	0.551693161262	0.931240108372	-3.613534060812
19250	H	-0.275474861074	2.253843142754	-3.674864926332
19251	O	3.005151234346	-2.725367744553	4.848704486126
19252	H	2.129799713333	-3.115901213186	4.978968840682
19253	H	2.902345846671	-1.780478733218	5.003773625600
19254	O	-3.557547339303	2.460478177232	1.848094805208
19255	H	-3.941053636921	1.702891645404	2.310763756609
19256	H	-3.076951522877	2.069034587138	1.094471605384
19257	O	-2.509180679929	3.799530638663	-1.455510594984
19258	H	-2.418195753703	2.914458959380	-1.044944484102
19259	H	-3.384603726779	4.107623645854	-1.139186282052
19260	O	-4.217480030712	-2.341277611322	-0.565503436644
19261	H	-3.634620674819	-3.028187681295	-0.934047548418
19262	H	-4.244882595458	-1.679619121781	-1.277883841033

```
O      4.035332061254    3.062663418625   -0.987349276852
H      3.664830482570    2.905592403944   -1.890972256123
H      4.252906608531    3.994175030386   -0.942199290962
O      3.754512332313   -2.779848301254    2.264659737565
H      4.051676996827   -1.874133984668    2.102208810588
H      3.676263807458   -2.853251753021    3.238318022046
O     -2.019110200715    3.877081280527    3.523362432348
H     -2.561252523542    4.590544738316    3.860023606359
H     -2.569182415918    3.390774239477    2.868653339407
O      1.448474207276   -4.074477340203    2.056459386292
H      2.224516681332   -3.470844406570    2.066159780314
H      1.460234055138   -4.525773218455    2.903305518851

Ambimodal TS Water45-25
155
ANGSTROM
C      0.477967099750    1.293196986159    0.850499473111
C      1.204496013082    0.916700552959   -0.233743733718
C      1.543293756441   -0.448153074344   -0.575573517515
C      1.208435677897   -1.550454726932    0.156667133889
C     -1.078848829659   -1.617621326875   -0.290119463422
C     -1.645586495564   -0.630907444029    0.485128118565
C     -1.775395108743    0.754459367002    0.031354046176
O     -2.367326697532    1.650900529737    0.679784356821
H      1.461947331796    1.674732571424   -0.971565276354
H      0.226913599620    2.332564427128    1.010602749701
H      1.953075468830   -0.596931440148   -1.569082392954
H      1.443804463950   -2.541920356225   -0.202215235442
H      0.930003000615   -1.486895649047    1.197271856157
H     -0.872950975957   -1.451719165638   -1.330629166450
H     -1.104117479217   -2.648901616875    0.030538041874
H      0.290199474045    0.634960132568    1.686482885683
H     -1.515656697545    0.962325472583   -1.021419475532
H     -2.737370073479   -0.268440784522    2.183322826135
N     -2.031732018527   -0.878476665900    1.802651908360
H     -2.145005534920   -1.843036119765    2.064226932967
O     -5.383948550195    2.758506173036   -0.104298334873
H     -4.647010637164    3.389229012587   -0.091963969376
H     -5.355870463685    2.327537419442   -0.972715509046
O      1.392776015418   -3.603977290350   -2.416729408730
H      0.481394495356   -3.427932338965   -2.699109913986
H      1.944383135065   -2.966756046030   -2.887730475178
O      2.928608330597   -1.506201629367   -3.655973362129
H      3.486174479747   -1.360119680333   -2.858955595865
H      3.539265627994   -1.514595903229   -4.394831673268
O      3.395357929285    4.028460347787    1.495473692662
H      2.554798538296    4.177206385357    1.001623704181
H      3.459453226996    4.756199318671    2.116763803162
O      4.197708951722    0.269459921594    0.932107461157
H      3.666174615067    0.741138704979    1.611264688396
H      4.782694446421   -0.317753717585    1.416961786757
O     -1.626717882554   -0.610057975940    4.731792815998
H     -2.206080308251   -1.313796920479    4.400624877710
H     -1.976281152615    0.230070559934    4.389019208486
O     -0.109486688749    6.802054099911   -2.178129548074
H      0.817887332101    6.576175972970   -2.053952667136
```

19319	H	-0.403547928611	6.248053382932	-2.911273473242
19320	O	-2.954253728636	3.844239640671	-2.863361108752
19321	H	-2.922640484304	2.974643313108	-3.334564288285
19322	H	-3.778001542487	4.258505320593	-3.124879555306
19323	O	-0.156012085345	-3.616953040154	3.343865300590
19324	H	-0.396730887543	-3.985065008874	2.465538765652
19325	H	0.166012022358	-2.699965113245	3.175553610616
19326	O	-1.038239221045	6.046831950933	0.283882207390
19327	H	-0.865898713782	6.481980384863	-0.571289164289
19328	H	-1.780000353302	5.425992921853	0.142453624902
19329	O	-4.970738521351	1.146738060856	1.999320588476
19330	H	-4.336791214750	1.590728376854	2.568004918567
19331	H	-5.189172566943	1.782506909261	1.278636339505
19332	O	-0.943522561025	-4.563351616304	0.985060221907
19333	H	-0.217660833665	-4.970871321291	0.451556686901
19334	H	-1.754486924988	-5.032497887029	0.787486983351
19335	O	-1.370275388771	-0.320215060165	-3.225760915686
19336	H	-0.468295418388	0.025097452463	-3.351100563957
19337	H	-1.329604264241	-1.266231083880	-3.454478164912
19338	O	-2.705622846164	-3.068149683916	3.801997721667
19339	H	-1.757354418689	-3.348613022150	3.706120719456
19340	H	-3.082973422704	-3.610770268450	4.494452721603
19341	O	0.641136291002	-1.052656858311	3.262231297526
19342	H	1.348030010530	-1.037624591782	3.935002971034
19343	H	-0.164097733246	-0.736987237067	3.715100064015
19344	O	-4.870050940501	-1.038541211395	0.572172797053
19345	H	-5.803008045028	-1.179503886969	0.401359612811
19346	H	-4.835981232170	-0.227126587189	1.143301994682
19347	O	-2.742859888245	1.683803611044	-4.387544050396
19348	H	-2.374969812654	0.895334576263	-3.938834659036
19349	H	-2.035739390544	1.962560332233	-4.986254004618
19350	O	-3.214402340278	-3.620952063298	-1.707475886838
19351	H	-3.270149332025	-3.732590965193	-0.746867795172
19352	H	-3.698156675149	-2.799972136530	-1.901392323488
19353	O	-5.039951657645	1.495480284603	-2.608958681211
19354	H	-4.599952985473	1.654373927627	-3.449137921020
19355	H	-4.851866923319	0.562138803327	-2.408378492956
19356	O	-1.144649320485	6.173231545433	3.095968271521
19357	H	-1.106673011923	6.411882157880	2.158591984949
19358	H	-1.934973044721	5.602909235264	3.175460956254
19359	O	1.176895462080	0.621921572974	-3.782163286256
19360	H	1.702044306400	-0.194669041990	-3.872977333487
19361	H	1.788706668199	1.248441002783	-3.341979753496
19362	O	0.450742321257	2.122114032353	3.915339064335
19363	H	0.767653856441	1.983240890369	4.811532300531
19364	H	0.600454438268	3.084878455586	3.706782087709
19365	O	-3.157663966481	4.357651919448	2.870197777011
19366	H	-3.264374090243	4.321756065079	1.914186574550
19367	H	-2.857523625357	3.478413378125	3.158291278835
19368	O	4.543370623522	-1.070005677701	-1.518421488659
19369	H	4.694406238487	-1.906352823479	-1.036954537134
19370	H	4.264046856822	-0.456444968535	-0.821823436264
19371	O	2.919493879225	1.704455098901	2.805011800768
19372	H	1.951326769253	1.684656225026	2.939693157678
19373	H	3.109612878484	2.573281506475	2.390704751039
19374	O	-2.184295615389	1.791982904328	3.369504151221

19375	H	-1.246797255495	1.955571068517	3.597158307593
19376	H	-2.191150435984	1.687277509510	2.394493258945
19377	O	1.191461904936	-5.415390082523	-0.313290573891
19378	H	1.780145197137	-5.113106727266	0.396599143595
19379	H	1.339941251713	-4.837118506264	-1.079066882142
19380	O	-2.916875854783	4.105540250653	-0.141550809268
19381	H	-2.618355626666	3.216032768548	0.158710656664
19382	H	-2.885487415400	4.063034704508	-1.113348118941
19383	O	0.933251701376	4.561287789085	3.142265084054
19384	H	0.193960760448	5.203245516315	3.292296586900
19385	H	0.956598705573	4.455821244858	2.182463219347
19386	O	-3.991900446918	-0.972371412014	-1.985731904275
19387	H	-3.083406362617	-0.685403754906	-2.138846432425
19388	H	-4.172279060831	-0.924091150693	-1.025280131164
19389	O	-0.501708163123	4.509596830610	-3.866824971685
19390	H	-1.360684903532	4.334774577809	-3.442472367174
19391	H	-0.379540801591	3.792384638627	-4.509490261962
19392	O	2.885310157676	1.429762122514	5.518960284208
19393	H	3.042189671069	1.562320568070	4.560707569591
19394	H	3.615623340424	1.839494353670	5.981800761727
19395	O	-0.199983087156	2.240802532981	-5.456404207626
19396	H	0.272640193688	2.286018008655	-6.287660293381
19397	H	0.305598075002	1.629900112474	-4.877614303622
19398	O	4.588692789148	2.527148500408	-0.572318979894
19399	H	4.288876518953	3.210119386914	0.042297028169
19400	H	4.559110907921	1.700116868986	-0.063986645891
19401	O	0.246590865077	-2.348203671590	6.007433157185
19402	H	0.145938402651	-3.032060723557	5.335111972354
19403	H	-0.417103462208	-1.673769228386	5.802120914620
19404	O	4.658802400524	-3.080961331279	0.291079685655
19405	H	4.174259722737	-3.908782853324	0.250081476769
19406	H	4.409374964317	-2.677232341858	1.133485602892
19407	O	1.064227474590	4.418307683297	0.279518588275
19408	H	1.332967975770	4.416496301236	-0.663771550091
19409	H	0.325440314777	5.068955087247	0.326869291990
19410	O	3.505000091942	-1.917108489239	2.649324637307
19411	H	3.398415268103	-1.809600136622	3.610089017633
19412	H	2.824211202738	-1.357001990140	2.257030700239
19413	O	1.741806843648	4.503963341744	-2.315962093295
19414	H	2.311898778024	3.772986028403	-2.594193056909
19415	H	0.949495569667	4.463538772179	-2.884969172849
19416	O	2.585382430962	-1.269088713148	5.158423545378
19417	H	1.894559528488	-1.692271295284	5.705513364974
19418	H	2.690151155347	-0.355540297314	5.476895142356
19419	O	2.390217719329	-4.369773416504	1.982966744497
19420	H	1.637103802050	-4.479901378887	2.573974359718
19421	H	2.822948603623	-3.546874801191	2.267970843637
19422	O	3.176015448744	2.157852999549	-2.808114973616
19423	H	3.623018421935	2.313111315150	-1.943715921547
19424	H	3.859320340985	1.700752006013	-3.329240556541
19425	O	-3.716088670544	-3.520765726776	1.098060767621
19426	H	-4.156918159114	-2.667439596645	0.957059935514
19427	H	-3.439654139406	-3.517640157735	2.024145399315
19428	O	-1.203763011183	-3.067615026800	-3.349052227011
19429	H	-1.873665947461	-3.327385865805	-2.668319959001
19430	H	-1.375190872963	-3.631125745758	-4.104499435062

```
O      5.288680837087    0.591067321321   -3.603128370595
H      6.064086556188    1.115243587338   -3.397689354114
H      5.171491035497   -0.020351666612   -2.854980396026

Ambimodal TS Water45-26
155
ANGSTROM
C      0.096384077360    1.517207463988    0.714572638524
C      1.260666911549    1.364264907553    0.030854504592
C      2.020680771007    0.137316212943   -0.079439554674
C      1.669605623637   -1.061209146510    0.485427733757
C     -0.110526382609   -1.749845773396   -0.764672157122
C     -1.132296846733   -0.923558552646   -0.329853587647
C     -1.381563316388    0.361990485611   -0.978734023382
O     -2.408562938177    1.052874545187   -0.795840442521
H      1.611146233593    2.189484773056   -0.587112551481
H     -0.454209479060    2.447588778136    0.693500121571
H      2.834422358379    0.153874557320   -0.799344405378
H      2.267197388532   -1.945229533525    0.314990477106
H      1.018409519350   -1.122261717617    1.345523781111
H      0.376889767403   -1.562608472364   -1.705829433847
H     -0.027499132103   -2.761197111375   -0.395121788275
H     -0.255978806390    0.805041415539    1.446801717985
H     -0.710725149009    0.632622617019   -1.808001184430
H     -2.452572457272   -0.514763854307    1.206873501935
N     -1.950750558471   -1.251286604806    0.730000718866
H     -1.777320154632   -2.097091163756    1.246836380152
O      1.956261393289    0.753330492136    6.499474210963
H      1.105048006846    0.274890576472    6.537845009674
H      2.605202340755    0.092286035092    6.239775804455
O     -1.342755751520    2.156147700707    4.055767130011
H     -0.450108954287    2.320217265843    4.411645900265
H     -1.641690729217    2.995034851926    3.662478366651
O     -5.000114216912   -1.561401655711    1.830290936117
H     -4.957215402660   -0.802917929432    1.218786542990
H     -4.302286960658   -1.443708670329    2.500882750952
O     -0.355062295744   -0.899548947156    3.449679779275
H     -1.327467818717   -0.997980367589    3.362933012136
H     -0.018670854656   -1.781225149985    3.700134457921
O     -0.288039912976    5.515916567229   -0.606631511301
H      0.329622679189    5.187908949743    0.071014800226
H     -0.235082164477    4.885495303457   -1.342541355450
O      2.160298645018    0.267013489068    3.337972524723
H      1.214672663389    0.074955616110    3.258313645465
H      2.272591743066    1.220728984003    3.149591648992
O     -0.340706314044   -0.661094870101    6.153041573725
H     -0.317673054884   -0.579204280101    5.172564827662
H     -0.188409153538   -1.591445133161    6.335308369786
O      0.657835813289   -3.102185268357    4.711373926773
H      1.469537727568   -2.630844626990    5.003923612938
H      0.932728517823   -3.628697638688    3.944637639305
O      4.605071102901   -0.128858698078   -2.846228866069
H      4.501183224252    0.773163474470   -2.493323271721
H      4.889150648500   -0.665260963639   -2.091133056407
O      2.535034230809    3.988434609585   -2.440270472867
H      1.580905103585    3.878125736338   -2.534842597625
```

19487	H	2.653749846072	4.823879738475	-1.937484187172
19488	O	3.155361062193	-3.194518569546	-1.665155028091
19489	H	2.673751303593	-2.536447447070	-2.186584613369
19490	H	3.910627665601	-2.720142380482	-1.289610283041
19491	O	1.035435675028	2.875181560384	5.202413149329
19492	H	0.832242819148	3.401579373052	5.978164530053
19493	H	1.485014841226	2.072770734268	5.560752705740
19494	O	-0.307745201342	3.570706411171	-2.580952614463
19495	H	-0.033539447841	2.677809312638	-2.854612566509
19496	H	-1.278155320745	3.564812794905	-2.602599693876
19497	O	-3.320295601126	-0.211580048988	-3.630154103689
19498	H	-3.939746332704	0.490406898883	-3.376525794805
19499	H	-3.487539763092	-0.943200477540	-3.024560839835
19500	O	-3.025790713533	3.231118495675	-2.137712657347
19501	H	-3.868264080293	3.010525238592	-2.547825488676
19502	H	-2.765214907476	2.406089412836	-1.663623166766
19503	O	4.820729803799	1.926769521342	1.097109159770
19504	H	4.607444133484	2.017424379069	0.148820774176
19505	H	4.857442949555	0.983260573557	1.292796909171
19506	O	1.592282084722	4.786350674264	1.245198732133
19507	H	1.995073535106	3.945832421639	1.520053529507
19508	H	1.185400973892	5.137237214377	2.064417037109
19509	O	4.333399346683	-0.778812332142	1.983412380107
19510	H	3.535431561567	-0.338618332635	2.331927126663
19511	H	4.264113075318	-1.685690318265	2.329140191733
19512	O	1.090390021313	-3.888913340161	2.154710637243
19513	H	1.445323582683	-4.369542025921	1.364642023181
19514	H	0.141761370955	-3.824253674486	1.981323652978
19515	O	-1.779377234802	-3.641778036835	-2.710332741113
19516	H	-1.143604210781	-3.172668769057	-3.274805489135
19517	H	-2.428808358162	-2.994989617234	-2.407018922736
19518	O	4.385770131376	2.325169717041	-1.595746248251
19519	H	5.160758839153	2.842773789006	-1.821376297176
19520	H	3.617731315225	2.880811379083	-1.888623305234
19521	O	-1.663545816152	-4.160107194964	1.479943077259
19522	H	-2.601609530288	-4.017198034235	1.245381524393
19523	H	-1.303897187230	-4.632241907884	0.705786828156
19524	O	0.347837693704	5.162354111057	3.582035270772
19525	H	0.658243234440	4.362209933567	4.024930398321
19526	H	-0.565062679291	4.987533324864	3.299041801656
19527	O	0.755445954171	1.254955000855	-3.612699741866
19528	H	1.344037505192	1.723122158919	-4.208764378144
19529	H	0.054998589701	0.823904111449	-4.184357220608
19530	O	2.824205060524	2.746577442907	2.588263911336
19531	H	3.161039789247	3.241119781453	3.337473671248
19532	H	3.605946434407	2.496019929326	2.028720739967
19533	O	-3.875753975775	-2.013578880397	-1.518511242983
19534	H	-3.927952238491	-2.734996196466	-0.867797167538
19535	H	-4.284144402300	-1.260963741651	-1.075953711675
19536	O	-3.041295389221	0.981197518681	2.313875393820
19537	H	-3.634291817503	0.608212546877	2.970931620757
19538	H	-2.274535213118	1.313514755113	2.809918779883
19539	O	-2.987048013603	-1.424685220857	3.699087217477
19540	H	-2.812298774124	-2.339959536280	4.005196222508
19541	H	-3.082627824927	-0.859592377781	4.497898133080
19542	O	-4.742376900043	2.843046114390	1.264950630622

```
H      -4.031328529527    2.326836571570    1.687982703514
H      -4.297402905815    3.563851793131    0.806913105723
O      -4.206998591629   -3.721265389976    0.629564931076
H      -4.538910650167   -2.910347602087    1.115497990828
H      -4.899561286957   -4.377662653694    0.700792317067
O      -2.028184487526   -3.857749309333    4.249576356868
H      -1.142250870218   -3.729286007953    4.612740155134
H      -1.904656085768   -4.164866096733    3.338409401618
O      -2.830631969054    0.432117091693    5.609577978896
H      -2.123856797141    0.070030335663    6.162656314867
H      -2.399054252104    1.151002904224    5.112990146831
O       3.655108376374   -3.276448908004    2.880131157566
H       2.721713356764   -3.363172173370    2.609894909647
H       4.089277983822   -4.068429772805    2.560558428842
O      -2.704229095933    4.661467941653    0.169772900453
H      -1.884887565779    5.152657299828   -0.060345670455
H      -2.901358314101    4.170522071064   -0.651381647258
O       5.130203979316   -1.554460730086   -0.476778323713
H       6.035408343626   -1.814877852861   -0.304928257010
H       4.771917391619   -1.210063206150    0.369863110587
O      -1.001409934306   -0.046007575913   -5.015242297648
H      -0.696035698760   -0.961971875867   -4.920318596257
H      -1.876382073601   -0.022122107072   -4.584197904560
O       2.575591298129    6.188336399001   -0.887059345948
H       1.734696432292    6.607623581074   -1.097599639523
H       2.424769007857    5.752318380637   -0.029003087781
O       2.131601041343   -1.061858368241   -3.226040077405
H       1.638335306242   -0.217587692184   -3.226539389518
H       3.075654117287   -0.786903225195   -3.251649891647
O      -5.256154339724    1.501439410622   -2.559661299560
H      -5.200320757470    1.124660313059   -1.653281298525
H      -6.162186935402    1.394923804411   -2.848460647127
O       0.172646910668   -2.498599181179   -4.362996109642
H       0.948626215764   -2.048901685031   -3.963696527187
H       0.519854563091   -3.128798321491   -4.994843396350
O      -4.858061210887    0.481157001296   -0.068423707030
H      -3.889151431754    0.612993092839   -0.213945334163
H      -5.105178568624    1.287615715667    0.427397556425
O      -2.213497585392    4.399283591489    2.770639266284
H      -2.347074385411    4.410163490299    1.792744287600
H      -2.973767931875    4.855146804051    3.134235221459
O      -0.761918345709   -5.236391468060   -0.876839350478
H      -1.070584429720   -6.105527854276   -1.133703416631
H      -1.118709973353   -4.609777624884   -1.558628881441
O       2.888746923893   -1.648141618044    5.076194926777
H       3.473293047440   -2.227128030096    4.573388695690
H       2.670101831170   -0.912932181349    4.466570960508
O       1.911821135683   -5.009548328172   -0.072431161349
H       2.384807950575   -4.399264781642   -0.672132683764
H       1.050099811402   -5.172285193640   -0.486013192324

Ambimodal TS Water45-27
155
ANGSTROM
C       0.208707063071    1.754897538217    0.669665305351
C       1.341374381142    1.557554405872   -0.054601543836
```

19599	C	2.051983246937	0.303216600703	-0.173243596348
19600	C	1.734832462685	-0.847378999816	0.485999110011
19601	C	-0.188383602911	-1.398595319901	-0.736609909615
19602	C	-1.164616027853	-0.628074176471	-0.148377381201
19603	C	-1.508349380326	0.684498585914	-0.680100478553
19604	O	-2.489382071240	1.391385226081	-0.314500016873
19605	H	1.703620620009	2.367840546528	-0.682351039176
19606	H	-0.274953085574	2.720318266435	0.694457025743
19607	H	2.805167837376	0.259016740300	-0.952988822841
19608	H	2.291766347778	-1.754624221767	0.306910983460
19609	H	1.137488019334	-0.850588286062	1.385941006750
19610	H	0.247289906048	-1.108474505047	-1.677369818750
19611	H	-0.049279835922	-2.428131579761	-0.446411749614
19612	H	-0.123719698572	1.060685713209	1.424067262838
19613	H	-1.004460026952	0.966715799192	-1.618933331307
19614	H	-2.631834029208	-0.577184516294	1.277701650139
19615	N	-1.758835131710	-1.037063373939	1.059306584646
19616	H	-1.825835684079	-2.051629832281	1.172213886638
19617	O	-6.182768103663	-0.123013836071	1.570808355372
19618	H	-5.456381721767	0.399982763021	1.941494155869
19619	H	-5.890028271228	-1.043372174054	1.599363057396
19620	O	2.597408542314	4.346411229897	4.489306785777
19621	H	2.146484698148	3.763021428709	5.119238800562
19622	H	1.914683639748	4.945193966821	4.150211882400
19623	O	-1.308145039508	3.475017696056	-4.307094447253
19624	H	-2.014246521915	3.052149114881	-3.773753761138
19625	H	-1.070695047153	2.822864134094	-4.984960628285
19626	O	1.294804934775	1.361960671224	3.329194832413
19627	H	1.876912175954	0.624756525120	3.612044117197
19628	H	1.863256729653	1.964442712751	2.809955127990
19629	O	-0.344901870730	-1.312259165077	3.457656899193
19630	H	-0.805548518453	-0.984672452646	2.662385884740
19631	H	-0.925245463979	-2.044567847988	3.784888929415
19632	O	-3.076104856680	-0.329477036336	-3.479237800839
19633	H	-2.238444664744	-0.741644100498	-3.731377968439
19634	H	-3.381796624956	-0.785105947142	-2.684870122110
19635	O	0.000623506296	1.424903127581	-5.696201124586
19636	H	0.672360397281	1.726252224313	-6.307935835132
19637	H	0.451290846240	1.261138495691	-4.840902097537
19638	O	-0.151286546508	5.033269069988	-0.953606313174
19639	H	-0.893167449202	4.643610221478	-0.437732767142
19640	H	-0.609009433108	5.558658697955	-1.652124390267
19641	O	1.707835895278	-2.941879430684	3.129305277638
19642	H	1.536144790052	-3.637643562004	3.766762768212
19643	H	0.931687818329	-2.324889505997	3.191746955808
19644	O	-5.264395209973	3.124038294210	0.094783096905
19645	H	-5.467105064073	2.293039446890	-0.368131612676
19646	H	-4.968992796808	2.874420750159	0.973709762877
19647	O	1.934325657134	5.270156061809	0.637250529514
19648	H	2.640508753423	5.696365817343	0.149672786532
19649	H	1.168014390489	5.201710644804	0.019718356447
19650	O	-1.936686432890	-3.357990521724	3.913684413896
19651	H	-1.969468962695	-3.715372046106	3.009886347801
19652	H	-2.745805168155	-2.844086805429	4.032259433691
19653	O	-1.599152582612	4.263082581356	2.946543364001
19654	H	-0.799573231796	4.809905975135	2.990986260526

19655	H	-1.922164897388	4.305903385558	2.031963728134
19656	O	4.226299572672	-2.924837288213	-0.867089809257
19657	H	4.532134367567	-2.021077867054	-1.037947304249
19658	H	4.309645980811	-3.046462916831	0.104667020582
19659	O	-5.977434416228	0.652207609031	-0.927860704145
19660	H	-6.793118654933	0.609469276453	-1.426379946587
19661	H	-6.185307879095	0.365429805383	0.000135611269
19662	O	2.216368037048	-2.178384385109	-4.006235639839
19663	H	2.157877825778	-2.768447531813	-3.237920830369
19664	H	1.307411539716	-1.972814032521	-4.260708576416
19665	O	-1.884249857306	-3.869146590673	1.214753995197
19666	H	-0.962651837236	-4.163229078137	1.099520652926
19667	H	-2.300593130739	-4.003667291589	0.331427776461
19668	O	0.601454109173	-0.314500757270	5.896945297176
19669	H	0.651272794731	0.648700267578	5.998372495041
19670	H	0.234381351860	-0.499096962180	5.015280586779
19671	O	3.646934455880	2.818907906146	-2.251279213895
19672	H	2.806698737453	3.203698404009	-2.539029916656
19673	H	3.790118105019	2.049086885738	-2.813721020550
19674	O	0.718242317936	5.819611155820	2.998952280068
19675	H	0.596255484098	6.765647633205	3.085180975861
19676	H	1.177848294512	5.664924196885	2.149451021496
19677	O	-4.038983588889	-1.299299954981	-1.005365279240
19678	H	-4.697681089367	-0.590040547493	-1.107782826850
19679	H	-4.228843536840	-1.689740086956	-0.134788472836
19680	O	1.990119422671	-3.881140043544	-1.757473942608
19681	H	2.843371887329	-3.482905240572	-1.416647412615
19682	H	2.227991417515	-4.759208551571	-2.061554003181
19683	O	-0.471615912567	-1.146655584333	-4.283893561603
19684	H	-0.618980952818	-0.792958675639	-5.166937775088
19685	H	-0.031461867194	-0.423358030574	-3.793878240440
19686	O	-3.715873023450	1.141668929942	2.093356741102
19687	H	-3.364644335968	1.252494238236	1.184522819991
19688	H	-3.002561493580	1.447480403839	2.682679418347
19689	O	-1.717853755320	5.922671065844	-2.892617152112
19690	H	-1.591958827643	5.250470844651	-3.577047729370
19691	H	-2.602841712279	5.768942446591	-2.533993360205
19692	O	-4.564673739811	-2.449873091317	1.487517756322
19693	H	-4.228983422677	-3.340419820354	1.358861168077
19694	H	-4.231162217886	-2.148166045378	2.350216000090
19695	O	-0.720422764120	-3.662561042566	-2.894631007881
19696	H	-0.702458242570	-2.859626558156	-3.429035233588
19697	H	0.144391595567	-3.744549880092	-2.475543498098
19698	O	4.612232825391	1.950036796032	0.252787390383
19699	H	4.084555665303	2.383639711988	0.944966276110
19700	H	4.342935754105	2.375652836762	-0.579442181928
19701	O	0.944289972032	3.482562906741	-2.841674714610
19702	H	0.632630058630	3.991951080101	-2.066118182279
19703	H	0.213017490881	3.586680018167	-3.487666820326
19704	O	2.824084420573	-0.618933257983	4.304584040384
19705	H	2.310428660685	-0.604152580312	5.132684548273
19706	H	2.629829882370	-1.475082946072	3.892897770660
19707	O	4.773989701338	-0.274380163929	2.218156558418
19708	H	4.274486028831	-0.097489956290	3.023662703716
19709	H	4.741701333148	0.518358326812	1.672543692673
19710	O	5.220275147049	-0.313856972420	-1.192584488611

```
H      6.173999107476   -0.404863412004   -1.187133732916
H      5.014329972924    0.418152449046   -0.579072344032
O      4.258413032276   -2.921479157523    1.795438273326
H      4.573536384172   -2.013160555534    1.954868035424
H      3.426815762042   -3.000037510731    2.276843487397
O     -2.906010437646   -3.905190592766   -1.238325907819
H     -2.166908620421   -3.932825187807   -1.871691654459
H     -3.349132321900   -3.058376721153   -1.378402335153
O     -2.312211428936    0.043919768445    5.698238481894
H     -2.036720793431    0.727411276241    5.062883790253
H     -1.503530969472   -0.300432561342    6.095505898387
O      3.569966277247    0.102688290328   -3.406715573425
H      3.259181135231   -0.761694175210   -3.742107502713
H      4.206575882589   -0.107075524383   -2.703355828295
O      0.748428558671    2.533061710343    5.635834767727
H     -0.133518710211    2.908777538868    5.590661064018
H      0.942833240541    2.158282633034    4.742485789629
O     -3.245665757384    2.296557239741   -2.814923543479
H     -3.096452298156    2.069823400365   -1.880565338889
H     -3.347447233615    1.428332348566   -3.248039649650
O     -4.026075362269    4.722697919679   -1.722757412088
H     -3.875114042304    3.964197777665   -2.310455672410
H     -4.728830600370    4.419276313713   -1.124625809007
O     -3.674800169811   -1.165072993417    3.726229521029
H     -3.784744739517   -0.343213889828    3.224383461788
H     -3.290711467343   -0.860786959637    4.575062667261
O     -2.299266343428    4.050035413493    0.253807811624
H     -2.957988335922    4.468712718316   -0.339536447326
H     -2.413907179743    3.095144208837    0.096257402161
O      0.782710363171   -4.318715977835    0.804457442272
H      1.255603771916   -4.202580077879   -0.032576623427
H      1.285754578218   -3.862463028487    1.493935729099
O      2.944135038240    3.137279454266    2.129251822850
H      2.546555305718    3.816520145829    1.560787156462
H      3.047330813672    3.569274616823    3.008043252618
O     -1.492397762833    1.711993674894    3.637155739505
H     -0.576646968851    1.459116238800    3.443817018446
H     -1.543047769851    2.679746859842    3.468769343613
O      0.984745243968    0.858895338296   -3.216119538680
H      1.930442140321    0.604970380492   -3.248970805726
H      0.967645369814    1.800912521392   -2.937225470335

Ambimodal TS Water45-28
155
ANGSTROM
C      0.175672946724    1.647567322941    0.879317477316
C      1.226331419076    1.568493624951    0.016045608737
C      1.978571267993    0.373215111784   -0.265901301799
C      1.706615962527   -0.857344002725    0.289199752977
C     -0.154239408231   -1.404504104315   -0.746560575927
C     -1.168064894599   -0.627186645555   -0.212804519933
C     -1.492418953175    0.696189806357   -0.715577471869
O     -2.496912960925    1.356048898032   -0.364964450071
H      1.450161789240    2.430248149586   -0.609408305982
H     -0.396298370258    2.557135326713    0.993430376154
H      2.661884716463    0.426768758595   -1.109297373386
```

19767	H	2.279287588396	-1.727234504344	0.002565319931
19768	H	1.224760760899	-0.942915467597	1.252959073302
19769	H	0.252813623090	-1.164080981297	-1.715800103533
19770	H	-0.061119661311	-2.442473460386	-0.459106853187
19771	H	-0.030817873635	0.895236633232	1.624904264894
19772	H	-0.901378195978	1.058889671290	-1.571688857759
19773	H	-2.666494094306	-0.423549969073	1.162624050081
19774	N	-1.872882224462	-1.027501729053	0.953781553970
19775	H	-2.101721769659	-2.008948203078	1.020250511460
19776	O	-0.178809191260	2.307910278969	-3.815997150126
19777	H	0.734000284880	2.128271975016	-4.153744963782
19778	H	-0.543962823765	1.404060359154	-3.687875746733
19779	O	5.315414159209	2.088477795305	-1.072644236648
19780	H	4.940817164476	2.183216445866	-0.180571996213
19781	H	5.583346678670	1.168344889867	-1.138908139374
19782	O	-1.703298543088	2.112377940315	3.830175563363
19783	H	-0.808905681525	2.508051820409	3.721714171617
19784	H	-1.685073846142	1.561387150269	4.618130874046
19785	O	-1.857328027585	-3.999873031803	1.425983420942
19786	H	-2.159851157204	-4.871542617792	1.688973000271
19787	H	-0.920755841952	-4.107817526910	1.156604409991
19788	O	3.688103783396	3.237141042447	-2.843195019966
19789	H	4.256462998895	2.785886022553	-2.176672932264
19790	H	4.249423687911	3.882183900864	-3.274987769949
19791	O	4.037253751895	1.928071949228	1.347312355832
19792	H	3.974265680524	1.478377837745	2.208519417528
19793	H	3.366220985412	2.626893660879	1.358821633980
19794	O	-0.791390278490	-5.639466406245	-1.450740553279
19795	H	-1.641680810646	-5.198406472930	-1.553759847430
19796	H	-0.242619953310	-5.347011095602	-2.192749094574
19797	O	0.635455260977	-4.547520549199	0.544989323289
19798	H	1.398132986757	-4.244377565420	0.005449716998
19799	H	0.165322340010	-5.138060379269	-0.083459897222
19800	O	-0.007196960824	4.909447069245	0.129606903329
19801	H	0.438580317811	4.852836131158	-0.736464308409
19802	H	-0.920541070333	4.608536278472	-0.063976878093
19803	O	-2.677360888929	-0.607577806249	4.595578601295
19804	H	-1.789801915680	-0.719767305893	4.207902776358
19805	H	-3.191303752418	-0.095377336402	3.959418369474
19806	O	-5.046635901577	-1.890273875150	-0.506311002899
19807	H	-5.926658600167	-1.818728167685	-0.877431965889
19808	H	-5.140567421508	-1.660135663019	0.458080716268
19809	O	-0.700041047482	5.522747000318	2.834851045427
19810	H	-1.637937703571	5.289325161978	2.919240837712
19811	H	-0.499816844800	5.506627725472	1.888235123838
19812	O	2.085159997728	-0.825388583584	-3.309598846666
19813	H	1.696653240047	-1.428097308180	-3.953799016488
19814	H	2.864567517013	-1.252517661748	-2.925706210637
19815	O	-3.183528787380	4.354333432643	3.454005087928
19816	H	-2.707548297361	3.515028416399	3.627724357640
19817	H	-3.568454100844	4.634035231048	4.284824298709
19818	O	0.264325604894	-1.877125537504	-5.277255170797
19819	H	-0.452342199009	-2.354481314197	-5.708567960638
19820	H	-0.206184729102	-1.249520344302	-4.677733651610
19821	O	4.957294894868	-0.652475635241	0.293033097410
19822	H	4.705067962942	-1.270318797527	1.008183697142

19823	H	4.585533688920	0.195098783808	0.574112638331
19824	O	0.692223188565	-4.079012296892	-3.342031778947
19825	H	0.943201879456	-3.824051610734	-4.233375864424
19826	H	-0.102984726576	-3.522532619748	-3.143406701009
19827	O	-5.191301240337	1.429032789052	-0.844827077654
19828	H	-4.263931382680	1.279311216180	-0.594284699212
19829	H	-5.498091728576	2.108385315381	-0.238122132344
19830	O	1.311339623659	-3.164216905722	2.809487822530
19831	H	1.540191909846	-3.804610290065	3.484542138299
19832	H	1.103740511094	-3.673676248240	2.004612955404
19833	O	4.298167130630	-1.880542254688	-1.968180688898
19834	H	4.512809164326	-1.401586338085	-1.129560487184
19835	H	5.122720981247	-2.253592300916	-2.281095830031
19836	O	-0.355347647851	-0.969271606121	3.208658503232
19837	H	-0.874200704110	-0.981103652668	2.365162423867
19838	H	0.191487047357	-1.771154667576	3.199727104797
19839	O	-1.534980028963	-2.759858527468	-2.949989721659
19840	H	-2.196428481355	-3.089008394591	-2.317323937098
19841	H	-1.954991538453	-2.728286744103	-3.832562333865
19842	O	-2.596407217445	-2.479266332459	-5.468701416389
19843	H	-2.779966274730	-1.557466346710	-5.705852956253
19844	H	-3.442533615533	-2.807675064089	-5.115493984846
19845	O	-3.501419092000	-0.780769373088	-2.449342622415
19846	H	-3.818754404831	0.057957784470	-2.832073282500
19847	H	-4.028032197221	-0.936373223957	-1.648776781776
19848	O	2.255351069480	1.563499904329	-4.506673810903
19849	H	2.237338322973	0.695473212189	-4.046111181932
19850	H	2.837865120535	2.135803092859	-3.980723724042
19851	O	0.631528693200	3.370532809742	3.652088243815
19852	H	1.271199598954	3.391584765294	2.916706401981
19853	H	0.172689770795	4.238794853907	3.560794093445
19854	O	3.902832138144	-1.941467020685	2.437801048444
19855	H	3.018331040650	-2.329947935587	2.483233614260
19856	H	3.855614988468	-1.109286195832	2.937681827635
19857	O	2.456820902338	-3.856047787302	-1.263795556507
19858	H	3.123504626058	-3.193408311443	-1.499515301624
19859	H	1.886327281280	-3.961937956951	-2.047407548739
19860	O	1.274483099713	0.818252054661	4.578956604142
19861	H	0.664374756521	0.241284615765	4.088066826974
19862	H	1.052468553502	1.735576595380	4.361353588049
19863	O	-4.599731250508	-2.934541137900	-3.704853118480
19864	H	-4.495627619118	-3.632709027749	-3.055497986320
19865	H	-4.247666261417	-2.120048961201	-3.284165829810
19866	O	-1.304654858986	2.116177802943	-6.377493658001
19867	H	-0.608722302240	1.542522577148	-6.755616281599
19868	H	-0.940066196856	2.376999193368	-5.516075119671
19869	O	-5.263958762424	-1.299659631758	2.019605939661
19870	H	-4.824664898533	-0.449905744628	2.165752822497
19871	H	-4.800247651362	-1.955573543550	2.579739781043
19872	O	-1.177195213702	-0.153312776571	-3.824692440148
19873	H	-1.600346856165	-0.721474133444	-3.162124406547
19874	H	-1.884005518050	0.001744639271	-4.498409368583
19875	O	-4.340819298811	1.643002804361	-3.419243493345
19876	H	-4.867353949210	1.682320271792	-2.598850614636
19877	H	-3.707496837341	2.378293280876	-3.358351605746
19878	O	3.722550841718	0.526944242129	3.693410152874

```
H       2.796969344940    0.612359295039    4.046654927591
H       4.309546164706    0.667495832382    4.436575750858
O       0.832218132632    0.467866509532   -6.735533740282
H       0.687785548629   -0.412663934452   -6.350795115134
H       1.462380547954    0.907953350738   -6.151028872792
O      -4.518265484153    3.353194920964    1.203322976069
H      -3.897801040381    3.675989420807    0.527514869073
H      -4.261581097145    3.825415573031    2.008301057042
O       2.172200364896    4.065275271656    1.520660947859
H       1.385055433907    4.314429285538    0.987055427769
H       2.619571083081    4.887961157124    1.725550507384
O      -3.641902110443    0.961715240702    2.260411500506
H      -4.075465025712    1.694275228356    1.779993984483
H      -2.850698116863    1.371085519621    2.643075356651
O      -3.088013021632    0.313650432725   -5.598885110520
H      -2.564739411843    1.022050695975   -6.044822968653
H      -3.750047459749    0.755824770474   -5.050693846988
O      -3.194471043064   -3.807109488522   -1.041278293833
H      -3.915639599072   -3.164997288443   -0.892149994351
H      -2.752198691315   -3.877950535063   -0.180540650217
O       1.139120093725    4.257574482293   -2.244060648096
H       2.054590930026    3.984431797264   -2.406003435257
H       0.590267064298    3.635234141979   -2.743508957183
O      -3.634147755072   -2.901695961250    3.473731857088
H      -2.901536414607   -3.170752652829    2.909103723749
H      -3.298917183357   -2.204289038661    4.070202784214
O      -2.471185921692    3.717088620580   -3.382673581764
H      -2.599184388159    4.253237605322   -4.166595170301
H      -1.616608419295    3.262154031498   -3.499747324233
O      -2.459554750082    3.975060421300   -0.591666761340
H      -2.558591301017    4.142491661820   -1.539141400007
H      -2.422843283803    2.990870120384   -0.523589190247

Ambimodal TS Water45-29
155
ANGSTROM
C       0.212248075117    1.686326479066    0.743637049507
C       1.245065865102    1.510578823220   -0.109142087217
C       2.032473133464    0.288204605262   -0.239654193239
C       1.796859069225   -0.843053103160    0.461609677922
C      -0.277343726538   -1.514538422915   -0.796953521845
C      -1.250864448406   -0.748972333658   -0.204340193758
C      -1.558088075749    0.567317838575   -0.749286836345
O      -2.530095990891    1.300454790485   -0.403319584485
H       1.475968738057    2.296671891622   -0.826946324064
H      -0.334020380775    2.616010856745    0.782320173850
H       2.789354567053    0.288006635698   -1.022395458838
H       2.373652279197   -1.739855591402    0.307840255920
H       1.134259839983   -0.863476926306    1.313412701788
H       0.187619288732   -1.201088078418   -1.715079552513
H      -0.094731367456   -2.530519971326   -0.484893377882
H      -0.009577578620    0.996051553560    1.544480636679
H      -1.003611592168    0.864538308704   -1.650154136783
H      -2.756779345317   -0.682620645855    1.216547332285
N      -1.903029831289   -1.144430251822    0.952035858386
H      -1.858288848558   -2.115071843202    1.223263835740
```

19935	O	-5.075850762685	0.564318640388	0.467556573792
19936	H	-4.150859607267	0.686980290373	0.198877747613
19937	H	-5.384714224283	1.473711323616	0.723673410528
19938	O	1.957576718127	-0.161935166325	-5.980536587241
19939	H	1.184034706956	-0.670805940184	-5.692341108616
19940	H	1.667056547033	0.754471940760	-6.056722794995
19941	O	-0.023858384472	-1.389971998107	-4.474211008737
19942	H	-0.824607604926	-1.921086697695	-4.303116063955
19943	H	-0.225247865521	-0.485960856866	-4.133393309302
19944	O	4.457245079980	-1.242378832995	2.657269295666
19945	H	4.256244919129	-0.299928826619	2.670360111598
19946	H	3.656430101458	-1.691396186658	2.989538617224
19947	O	4.254313865451	3.266891344264	-0.876014270052
19948	H	4.741417656627	2.482277188137	-0.578153504927
19949	H	3.842591866306	3.004114985584	-1.710766800481
19950	O	-3.384881847291	-1.826236518160	-1.683024938250
19951	H	-3.432150570362	-2.511692062689	-0.993790890223
19952	H	-4.296869269943	-1.477822615805	-1.813901635888
19953	O	3.690550183545	1.473812171432	2.037962406433
19954	H	3.378923559775	2.379903330298	1.894609651391
19955	H	4.291227988538	1.285201446279	1.296277328467
19956	O	-2.551297393796	-2.575150597835	4.899053674695
19957	H	-1.985856572254	-3.039342015990	4.250296523228
19958	H	-1.939517922893	-2.169094737783	5.530009905291
19959	O	1.301495247263	2.573024199668	-5.276059951973
19960	H	0.970906647757	3.473112068965	-5.306868337313
19961	H	2.045493505922	2.578381768009	-4.640929483808
19962	O	-4.403421606609	-3.337484303897	2.601501551917
19963	H	-4.953314287406	-2.538736485387	2.593640573369
19964	H	-3.862208131436	-3.288037481264	3.396523420084
19965	O	1.501537691006	-4.111119261631	1.436766719076
19966	H	0.713482975981	-4.431282157890	0.952503262828
19967	H	2.168639502949	-3.962048703638	0.756434769556
19968	O	-0.188130842818	-4.465878969677	-2.646581436803
19969	H	0.578866291966	-3.888314474644	-2.749581300173
19970	H	-0.936430494914	-3.981368936163	-3.031642748170
19971	O	2.082498841347	-2.319494819561	3.456272312212
19972	H	1.369818069315	-1.677704281240	3.328408002687
19973	H	1.916931180763	-3.041164717209	2.825068804492
19974	O	3.322380741375	-0.126679034795	-3.671457795133
19975	H	2.942937285898	-0.182804491954	-4.577467669519
19976	H	2.950480638413	-0.873792772824	-3.185918699222
19977	O	-0.686878571394	-4.813215811564	0.024450002759
19978	H	-0.537125584453	-4.820335075980	-0.938519030392
19979	H	-1.579064347452	-4.460152298427	0.164148481104
19980	O	1.960796225664	-2.620857564059	-3.281947108329
19981	H	1.203510586572	-2.131993179936	-3.707283408333
19982	H	2.438983460145	-3.027394554800	-4.007552260157
19983	O	-5.078560669548	4.100367361701	-1.301262171785
19984	H	-5.032285471389	3.277105158487	-1.822416037351
19985	H	-4.172058547264	4.420612688030	-1.240510338754
19986	O	-0.458755111868	1.122851662662	-3.781094346248
19987	H	-1.324187555999	1.429518719908	-4.092016305012
19988	H	0.193402461237	1.631376755184	-4.298725117799
19989	O	-4.356289093840	1.765232692682	-2.536439428725
19990	H	-3.724360332916	1.522751718526	-1.843528536912

19991	H	-3.829168134600	1.946776156924	-3.334057851311
19992	O	1.106121979248	4.254687625010	-2.696481377292
19993	H	1.863257484224	3.665307051879	-2.790665236365
19994	H	1.042967292485	4.487378012170	-1.758078739888
19995	O	-1.035128610557	-3.350131634789	2.819819624811
19996	H	-0.602557874714	-2.476137603949	2.894492468287
19997	H	-0.346731790155	-3.985054961625	2.611271521153
19998	O	-2.837788220711	3.077865353064	1.970045031157
19999	H	-2.936841771235	2.793040300413	2.928265996550
20000	H	-2.694466846027	2.253909892231	1.487933585029
20001	O	1.898320693397	1.297101331873	4.127580956973
20002	H	1.989400044733	2.273070266919	4.190077165729
20003	H	2.411656173525	1.068320302397	3.341416200427
20004	O	-3.339477310892	-3.841818856977	0.224845132671
20005	H	-3.821846208991	-4.626148864456	-0.040293844189
20006	H	-3.664498055508	-3.633340169575	1.134911195170
20007	O	0.205400518250	5.003056366079	-0.130027941709
20008	H	0.156977014342	5.957361537777	-0.213109995050
20009	H	-0.686924273055	4.652920450874	-0.352182801434
20010	O	-2.939671789232	2.117631231109	4.357380704926
20011	H	-2.062487144453	2.137760294829	4.778100396775
20012	H	-3.084453485378	1.174262469346	4.132664838587
20013	O	-0.293537218483	-0.781746519258	3.299392683400
20014	H	-0.377502811608	-0.800877148076	4.281553814820
20015	H	-1.165560435240	-0.507361866136	2.983821185431
20016	O	-2.177177126606	-2.855712237778	-3.741466889065
20017	H	-2.616080388063	-2.428934420521	-2.951716461712
20018	H	-2.883564795891	-3.175347621321	-4.302873030963
20019	O	3.279313733442	2.449310394615	-3.388661803944
20020	H	3.279382982721	1.450378317672	-3.367016429830
20021	H	4.101476623695	2.690892755592	-3.820707081173
20022	O	-0.541431725420	4.597156336587	2.654310088704
20023	H	-0.194970487314	4.768801383749	1.770520819541
20024	H	-1.360109737131	4.088901322107	2.545479677176
20025	O	-5.732289141591	-0.582930572390	-1.932006830847
20026	H	-5.765502019365	-0.276645031579	-1.009132541952
20027	H	-5.463622130421	0.206840050202	-2.425107040532
20028	O	-3.161207238474	-0.396195647973	3.472191313229
20029	H	-4.095610016325	-0.506139065953	3.233258768793
20030	H	-2.966211884102	-1.154547026512	4.068550582916
20031	O	2.084377113176	-0.848717708265	5.880553298299
20032	H	2.318563780201	-1.560345901053	5.271362879786
20033	H	2.150335395815	-0.030793638567	5.356591858958
20034	O	-0.564703695837	-0.881286778101	5.961303490093
20035	H	-0.755966972905	0.055492744913	6.104160641151
20036	H	0.405713597424	-0.972297203325	6.120683127187
20037	O	1.811907046521	3.966722779678	3.953926554546
20038	H	2.377805859271	4.144671017038	3.192521813510
20039	H	0.915711625099	4.203886405353	3.656876216933
20040	O	-2.706759745108	2.491832994504	-4.584988683507
20041	H	-3.088136346997	2.795988704754	-5.408404977193
20042	H	-2.302473266081	3.284907162565	-4.145556533143
20043	O	2.771676692755	4.049002050924	1.255069924194
20044	H	1.926004040144	4.292604249460	0.866506104562
20045	H	3.377940570078	3.907361293745	0.501302084108
20046	O	5.148887285946	-1.700554199562	0.104744979152

```
H      4.966700799758   -1.654955185187    1.070712507259
H      4.494288430092   -2.309068036905   -0.259890186608
O     -2.279995456241    3.995461557204   -0.547833680440
H     -2.330632986265    3.018702479895   -0.654402545920
H     -2.616883348941    4.128574217183    0.352112944488
O      5.444605642984    0.910032597352   -0.037781233194
H      5.285458128146   -0.065149636188   -0.119960918204
H      6.332491128360    0.987519239789    0.314435721211
O     -1.558129509250    4.460615510991   -3.272542067808
H     -0.592738765387    4.365223105480   -3.183097192862
H     -1.907801769495    4.432388289834   -2.373805214115
O      3.145142909210   -3.546812716021   -0.899059510656
H      3.505595644998   -4.393824102158   -1.166736916817
H      2.671382921311   -3.184749473096   -1.664700833921
O     -5.696960931078   -0.878770499096    2.578635589972
H     -6.520828899657   -0.541332662015    2.930042132729
H     -5.525340866677   -0.389618131423    1.737991172859
O     -0.426121764698    1.858806059344    5.405740220299
H     -0.078997295738    2.656852520120    5.807301532445
H      0.282402393965    1.537045853963    4.814879905133
O     -5.539911689429    3.043810703841    1.157522648950
H     -5.538190126641    3.529703636186    0.308240793974
H     -4.698324005418    3.248440938406    1.595397148507

Ambimodal TS Water45-30
155
ANGSTROM
C      0.177223251693    1.620723810443    0.782716032782
C      1.297823380076    1.505012959458    0.018033388214
C      2.037594861498    0.284885571961   -0.223819042890
C      1.722077568107   -0.943809479277    0.281767511469
C     -0.266476492919   -1.375670683652   -0.920642601672
C     -1.230654060302   -0.623777484709   -0.279648981490
C     -1.565489319415    0.724118425700   -0.734897964742
O     -2.589045093036    1.363034991157   -0.360119951869
H      1.627014762641    2.372619275256   -0.552714453411
H     -0.331921957762    2.568575507743    0.885280388016
H      2.818023415987    0.344930949203   -0.980187184147
H      2.298144127923   -1.813150643926   -0.004971286355
H      1.102897342815   -1.076429360136    1.157236045042
H      0.148612714810   -1.044985952723   -1.857209684091
H     -0.136649772720   -2.419468490840   -0.682440691888
H     -0.124388388631    0.867557216775    1.495922927757
H     -1.019644314321    1.108685129521   -1.605571622144
H     -2.638578070629   -0.526301086437    1.222330561738
N     -1.863587125131   -1.059363089180    0.866521886313
H     -1.844483908104   -2.029334569133    1.145554543533
O     -0.056032838749   -1.953753510596    3.198985081097
H     -0.631474405660   -1.195154405103    2.989307830613
H     -0.312942111094   -2.192694918798    4.119772410648
O      1.931790817774    5.078815496673   -3.580191925595
H      2.481970424319    4.276233655546   -3.490115420563
H      2.325023472709    5.741922106973   -3.010596672598
O     -2.468740922881    4.028687547360   -0.854989933234
H     -2.648601426259    3.072056906906   -0.763379530091
H     -1.707944328320    4.092129584710   -1.455566023604
```

20103	O	2.317177981003	-3.307180280298	-2.577356583960
20104	H	3.169548993331	-3.102893106845	-2.146457820930
20105	H	1.708955220559	-3.485106342771	-1.848919899203
20106	O	0.250781871823	4.285099196064	3.732328436257
20107	H	-0.479404266222	4.312393360266	3.102939426094
20108	H	0.182274796598	3.442560957204	4.208375934689
20109	O	2.874101360724	-4.608327978205	1.262519948575
20110	H	3.281845815808	-3.752679317270	1.492649036419
20111	H	2.235668805393	-4.771426957248	1.980068472650
20112	O	0.240078182632	-5.724789566960	-2.571367848734
20113	H	1.034806277669	-5.920607704273	-3.069382891398
20114	H	0.548456825194	-5.262275579300	-1.745052873600
20115	O	0.462332080137	1.683246928373	4.736863868667
20116	H	0.664803203562	1.159298334789	5.525086349973
20117	H	1.250494360554	1.595002432351	4.162184494168
20118	O	5.425416651889	0.202763005041	-1.606522294211
20119	H	5.026604378093	0.170220636880	-2.492345161638
20120	H	5.463760427942	1.164560028046	-1.406082048992
20121	O	0.733276203350	-4.580617382863	2.936781850053
20122	H	0.058503855769	-5.122749627595	2.509983463286
20123	H	0.412474421006	-3.664939495725	2.853384978887
20124	O	-3.453387840257	-1.284744003137	-3.005331148362
20125	H	-3.141885083634	-1.941485857237	-2.355344495332
20126	H	-3.587459916637	-1.807982759091	-3.822322639919
20127	O	2.205928748998	3.852848796033	1.951359748247
20128	H	1.651402851922	4.119797974113	2.715828335189
20129	H	1.763922872430	4.269481119122	1.180778646013
20130	O	4.475839185090	-2.505320260979	-1.213289045655
20131	H	4.906800332373	-1.657928309080	-1.367415493265
20132	H	5.037307897498	-3.016571146905	-0.602821069978
20133	O	1.384102355420	-0.387230780493	6.341951885406
20134	H	2.017603413251	-0.478306120722	7.053112405848
20135	H	1.833334634034	-0.697324871340	5.519622835459
20136	O	-1.796839811408	0.445940739438	-4.191053092193
20137	H	-2.376196790254	1.074966229931	-4.622315246751
20138	H	-2.377577206467	-0.143652096203	-3.654771841858
20139	O	-1.059276066870	-3.529486332874	-3.510076585812
20140	H	-1.434178113534	-3.250960345989	-2.663719672128
20141	H	-0.534509787215	-4.327712617670	-3.310200604806
20142	O	-2.946092402767	2.181441668086	2.231813145664
20143	H	-2.543299778115	3.060449798415	2.191363249701
20144	H	-2.957265070569	1.864874325203	1.313310210932
20145	O	4.795193802583	3.839538641209	1.436743521945
20146	H	5.158948636916	4.693429569276	1.670724857190
20147	H	3.832259166007	3.874729915642	1.651294433438
20148	O	4.172654188456	0.343509924717	-4.081385408969
20149	H	4.624189559044	0.032132223333	-4.865420525874
20150	H	3.344077266320	-0.187965744046	-4.002406901758
20151	O	3.071134635426	2.670242987378	-3.010452398669
20152	H	2.199780430216	2.241021245679	-2.987167101002
20153	H	3.628941276451	2.047378439157	-3.501179492100
20154	O	5.228837566832	1.530531485783	2.900634912110
20155	H	5.256629497559	2.357772798165	2.391508666017
20156	H	5.415453416224	0.823301629493	2.261609080831
20157	O	5.621094144282	-0.462060115012	0.999949151898
20158	H	6.485358544113	-0.877606445405	1.003175535050

20159	H	5.447809293587	-0.205851751641	0.072869447289
20160	O	-1.725656197306	4.660552293977	1.561424387874
20161	H	-2.209980749523	5.438190412175	1.841379360593
20162	H	-2.053016293995	4.454012143537	0.635604023631
20163	O	2.597518374953	1.384099392691	3.083566100532
20164	H	3.581403655538	1.432376748149	3.163478738695
20165	H	2.370374210215	2.135128768214	2.508117612723
20166	O	-0.434780789296	-2.424192508818	5.815866599431
20167	H	0.288093809298	-3.084609572359	5.842748745402
20168	H	-0.039455308885	-1.613703069674	6.174350509058
20169	O	-1.532923213915	0.290669501472	3.522641803365
20170	H	-0.876113456142	0.804182808470	4.031984337695
20171	H	-2.113756163057	0.958566829306	3.106250107986
20172	O	5.759507992194	-3.983276102011	0.715801230733
20173	H	5.406044315145	-3.477333127484	1.461834135279
20174	H	5.199381687184	-4.763052291541	0.661085421866
20175	O	1.829527584584	-3.812746929493	5.344396296180
20176	H	2.381644238172	-3.114553988249	4.978266860815
20177	H	1.511746190130	-4.321214637429	4.580708429229
20178	O	0.721706670468	5.057464198265	0.088104150143
20179	H	-0.075514167988	5.098095011644	0.634172912609
20180	H	0.454534533872	4.624328144294	-0.743340396029
20181	O	2.336322571916	-1.154586344202	3.948801199864
20182	H	1.465888417032	-1.461884908170	3.586703135444
20183	H	2.461996444646	-0.261664864024	3.576307164444
20184	O	-2.866697947448	-1.952209293693	4.432017143836
20185	H	-2.257912333999	-2.259770827194	5.115138985543
20186	H	-2.532226135966	-1.077571575669	4.161625812731
20187	O	0.837355273790	-4.380505991251	-0.410042387240
20188	H	1.662351988027	-4.504053575219	0.118046369205
20189	H	0.107329452652	-4.661456632493	0.180237111729
20190	O	1.907775512994	-1.003963793218	-3.800717018008
20191	H	2.066846100307	-1.857332784152	-3.324631552780
20192	H	1.269690375684	-1.226361865254	-4.512765530443
20193	O	-3.148262984310	-2.900033334177	-5.148761936944
20194	H	-2.713796606562	-2.510186493182	-5.910551481351
20195	H	-2.437764695120	-3.330191497800	-4.636237396506
20196	O	4.083753695111	-2.358692139976	2.236677743679
20197	H	4.490723442002	-1.612779220548	1.763170040095
20198	H	3.523565543047	-1.960383277037	2.926385587943
20199	O	-2.752756002468	-3.300870913190	-1.271682890774
20200	H	-2.695798468151	-4.269077740491	-1.303804088775
20201	H	-3.421993383118	-3.092553777491	-0.596860328394
20202	O	0.668599654693	1.299412653153	-3.180928747722
20203	H	-0.153868448906	1.140387147402	-3.667981960880
20204	H	1.179388367790	0.470988130960	-3.293495147216
20205	O	-2.794714040094	-3.416918935512	2.185529538212
20206	H	-3.606254458590	-3.163393816168	1.726249630685
20207	H	-2.826167194123	-2.960620694357	3.056290296353
20208	O	-2.263241869352	-6.045532825944	-1.440886178340
20209	H	-2.808701092219	-6.678151978499	-1.909021680080
20210	H	-1.393831338073	-6.047633773029	-1.878628935214
20211	O	-4.824521289704	-0.203352941217	-0.822914987444
20212	H	-4.545939920340	-0.480349775516	-1.709718532716
20213	H	-4.170625120459	0.457386898187	-0.542560531397
20214	O	-0.234090913386	-1.592031839999	-5.287816312821

```
H     -0.785673904902   -0.843166868342   -5.011151696843
H     -0.462762665626   -2.323861426254   -4.687941844072
O     -1.217792915972   -5.287076174881    1.103249671678
H     -1.815978057786   -4.625499972585    1.509280528269
H     -1.722134014988   -5.718285508696    0.403514695497
O     -0.088156964455    3.881812488117   -2.264390753848
H      0.485485632643    4.420223039903   -2.848819152462
H      0.133569964206    2.963527777606   -2.471773172876
O      5.169131575102    2.818627602623   -1.130262054843
H      5.018322423678    3.163396016138   -0.236121785471
H      4.354415364111    2.950371371206   -1.637015673700
O     -4.780084867940   -2.527373904233    0.431272962554
H     -5.684885580777   -2.839327231818    0.447506496689
H     -4.816144436353   -1.609929703864    0.056959192660

Ambimodal TS Water45-31
155
ANGSTROM
C      0.203875482232    1.568944213261    0.835765562124
C      1.304076420801    1.399771548246    0.052841165106
C      2.020583466482    0.157701021126   -0.109374488792
C      1.675085494492   -1.018142774450    0.502247138019
C     -0.206772699259   -1.503303438988   -0.712943438131
C     -1.184834388012   -0.703608234430   -0.165691337776
C     -1.495011900184    0.638272312608   -0.644001743018
O     -2.510787204373    1.283047833202   -0.257798465789
H      1.605443576852    2.218279262751   -0.595393876290
H     -0.298211017837    2.521373757586    0.879358870466
H      2.780378008624    0.135522680183   -0.888268600731
H      2.225853626558   -1.925316599537    0.303530070908
H      1.072180780040   -1.045516638655    1.399738497100
H      0.251144811198   -1.248155061760   -1.655017052998
H     -0.133180581091   -2.542773779127   -0.427202026214
H     -0.073950687231    0.869677656819    1.610200131144
H     -0.952679606984    1.008058717030   -1.522186165617
H     -2.767296593058   -0.634462894469    1.142599315021
N     -1.888514893215   -1.114474428545    0.996300093194
H     -1.994268464566   -2.113126596593    1.111238454101
O      3.704846176924    0.371463879678   -4.117239651542
H      3.392312856532    0.574928072848   -5.012654662402
H      3.157486055459   -0.384754583675   -3.835248628645
O     -4.036459154111   -2.377526696298   -1.294225619118
H     -4.493667461657   -1.580936433718   -0.958057883438
H     -3.571391131759   -2.777976529401   -0.545417935809
O      0.490728591302    5.417585665327    3.512892924337
H      0.971033826695    5.074931116796    4.298148877206
H     -0.360288262955    4.956297323774    3.531040971202
O     -4.903907962319    0.094855701296   -0.534487081913
H     -4.048907124607    0.535571676913   -0.356048270036
H     -5.158003216551    0.486359545883   -1.391164633127
O     -3.761488796925    0.028023350021    3.737539835653
H     -3.815601569023    0.887730968755    3.293482925762
H     -4.222899600520   -0.592330391979    3.150594075169
O      0.387928240805   -2.734626800591    3.918681657067
H      1.291131512665   -2.428089525415    4.057594963788
H     -0.181411062371   -1.944094506721    3.965299129464
```

20271	O	0.140586226062	-4.136648924755	1.650068108272
20272	H	-0.789072182649	-4.069720105252	1.396246492814
20273	H	0.232227356698	-3.669312609315	2.509920069253
20274	O	-3.154459582376	-4.140179479838	-3.325742966053
20275	H	-3.667532250984	-3.526208990765	-2.781758954527
20276	H	-2.819449169918	-4.809170753446	-2.708552766572
20277	O	-1.596334316140	0.945246594582	-4.799364324157
20278	H	-0.776330019530	1.411822470339	-4.538927333562
20279	H	-1.902202469997	0.432835711964	-4.035594483040
20280	O	1.784538064048	3.886082132097	5.289551061740
20281	H	1.276333071546	3.085223829407	5.088036425850
20282	H	2.676504927156	3.724041537833	4.960438983274
20283	O	-1.838443313226	-5.654715180301	-1.381380818145
20284	H	-1.784786919167	-6.607494030451	-1.306744190518
20285	H	-0.958279367777	-5.354760467891	-1.758860470543
20286	O	5.748393331155	0.950751720700	-2.168584501342
20287	H	5.175729612831	0.738961513828	-2.918572570737
20288	H	5.473987982098	1.812315702232	-1.832300510786
20289	O	0.222707473296	6.538822510585	0.905265473047
20290	H	0.347597392528	6.389767738216	1.854224048836
20291	H	1.042736680541	6.260267660118	0.473704131859
20292	O	-1.524798982961	3.581383299400	3.031209784307
20293	H	-2.366543804742	3.102354517042	3.012351834057
20294	H	-1.405986143140	3.887664841439	2.106203610375
20295	O	-0.540492201875	3.374691170793	-1.895906367688
20296	H	-0.815462039628	3.654624175958	-1.006172516595
20297	H	0.187077624128	3.976904491990	-2.138979173414
20298	O	-0.882699495028	-2.833489533619	-3.751748468708
20299	H	-1.716500882855	-3.362835394222	-3.771544964798
20300	H	-0.675297693785	-2.566094322883	-4.653517344251
20301	O	3.700177621185	-2.606210167506	-1.586245404099
20302	H	3.077721996499	-2.174652790047	-2.183726076604
20303	H	3.196492797705	-3.286582674794	-1.119704150190
20304	O	2.592834593303	0.860202275450	3.129202261081
20305	H	1.721127951674	1.185632949941	3.434476259582
20306	H	2.642555648626	-0.091457054867	3.317311987412
20307	O	0.673740383293	2.305487205521	-4.475697122638
20308	H	0.347000534404	3.217609163940	-4.660571722875
20309	H	1.257934851266	2.378303478121	-3.703533365829
20310	O	-0.349523873832	4.749163848024	-4.690042816886
20311	H	0.167185107335	5.183279657364	-3.998874510282
20312	H	-1.238427381177	4.597295321093	-4.338354465885
20313	O	0.263817513627	1.809825560030	4.109084950729
20314	H	-0.256382062812	0.987104012866	4.119934886730
20315	H	-0.320185326197	2.477071702401	3.695527951550
20316	O	-2.530209738019	-3.982712076735	0.592199171079
20317	H	-3.188748510533	-4.426288918577	1.129238956938
20318	H	-2.289495326195	-4.600486614271	-0.134953390163
20319	O	-4.565528050219	1.223065652477	-2.961588641583
20320	H	-3.951256078516	0.524169262444	-3.215623518506
20321	H	-4.089453441131	2.052736892794	-3.118912340475
20322	O	4.392037394541	3.192574895745	-0.779447524524
20323	H	3.942951350000	4.008220465811	-0.515161764626
20324	H	4.419963504002	2.616352939636	0.017119491047
20325	O	-3.639521294312	3.763351976879	-0.789100570548
20326	H	-3.249476060517	2.871798784182	-0.728518466902

20327	H	-3.661922987098	3.960505596102	-1.729993448014
20328	O	0.197579419299	-0.966726300947	-5.579835658862
20329	H	0.867085602953	-0.439813311115	-6.043823931426
20330	H	-0.543406157219	-0.344225702523	-5.423073132189
20331	O	-5.068401202953	-1.404492258116	1.733120589132
20332	H	-5.167184055381	-0.806817326980	0.970813761608
20333	H	-5.954932393018	-1.652736905080	1.995519050733
20334	O	2.085796354030	3.937352726679	1.925900014354
20335	H	2.794132661711	3.662453102182	2.535366239872
20336	H	1.443840561173	4.440506304234	2.471028302485
20337	O	2.020257194667	-4.384804821794	-0.182161877281
20338	H	1.333502469379	-4.263843331298	0.518669376942
20339	H	2.448313337854	-5.219725829801	0.011749260030
20340	O	-2.459378197944	-0.801693085120	-2.888103936612
20341	H	-1.791254253157	-1.452568368654	-3.178359577085
20342	H	-3.099578368246	-1.334024199574	-2.384530786641
20343	O	2.786377849212	2.553332879815	-2.805989788243
20344	H	3.186913826270	1.803953573594	-3.286942188158
20345	H	3.385626153470	2.736577161536	-2.052007955163
20346	O	-3.692692135731	2.216582582603	2.006163067595
20347	H	-4.297153557596	2.852644532009	1.617385346851
20348	H	-3.173835377973	1.871841791099	1.254556643062
20349	O	-1.145951521721	-0.494543089833	3.607858869362
20350	H	-2.068151330580	-0.344029012626	3.907245029599
20351	H	-1.247761261858	-0.681186307143	2.653710792193
20352	O	2.073354871438	0.977110752166	-6.287210230190
20353	H	2.155777005937	1.495520792137	-7.087339162051
20354	H	1.547699441212	1.525524387298	-5.651328077917
20355	O	4.012527517186	3.050021964144	3.684895386344
20356	H	4.802687617985	2.963059835649	3.146519289295
20357	H	3.576296314852	2.172371036674	3.641972028283
20358	O	5.204922871557	-0.790268116919	-0.174542338124
20359	H	4.713432580375	-1.490950786646	-0.640570936528
20360	H	5.593746507980	-0.242943135574	-0.887226521083
20361	O	1.550846961503	4.987680200278	-2.545045650178
20362	H	2.113930294341	4.242500594747	-2.814907546471
20363	H	1.938149107306	5.308734044249	-1.719078562799
20364	O	1.953749206300	-1.711175030618	-3.671068315942
20365	H	1.219812560419	-1.389749573653	-4.243723856101
20366	H	2.254287116507	-2.518978560765	-4.093145102788
20367	O	0.406873208736	-4.805060207411	-2.376147716505
20368	H	1.017402940903	-4.538679421620	-1.666352483156
20369	H	0.119491696789	-3.998487368025	-2.843954778778
20370	O	3.054192144712	-1.862108921532	3.280847174218
20371	H	2.820947587617	-2.464290148377	2.570468309142
20372	H	4.011586551198	-1.682795876257	3.149841441013
20373	O	-2.636064642239	3.273128915230	-3.546312266130
20374	H	-1.884324623707	3.245608009372	-2.908864896605
20375	H	-2.421900495486	2.610518509130	-4.221264554123
20376	O	-1.364724928036	4.446534922257	0.488018834039
20377	H	-2.265744328037	4.543068009417	0.130531350373
20378	H	-0.929797755665	5.325448459897	0.545815410901
20379	O	4.540196064131	1.468590005270	1.271725900611
20380	H	3.722659063486	1.266865680434	1.755770539272
20381	H	4.755744054458	0.664872664385	0.765712842476
20382	O	2.771332845450	5.449598188503	-0.025568922702

```
H      2.500983414791    4.824684781098    0.718436499160
H      3.356710992640    6.102997787577    0.358776448320
O      5.545110541380   -1.217358183575    2.509433304150
H      5.528238892849   -1.215636451436    1.534576609150
H      5.767664062175   -0.318228214463    2.761006605037

Ambimodal TS Water45-32
155
ANGSTROM
C      0.267801758297    1.754407518473    0.686523766879
C      1.403089391028    1.531234754274   -0.023169007424
C      2.085122164236    0.260218992485   -0.137061577650
C      1.650348116884   -0.928148008621    0.388203530567
C     -0.167381926772   -1.346888806921   -0.902644147741
C     -1.173998312734   -0.637616178024   -0.279125612192
C     -1.574279807523    0.695634776948   -0.705282203711
O     -2.556832384930    1.313184212646   -0.214870953561
H      1.795572986274    2.334423063841   -0.644523809629
H     -0.216010281459    2.720999041258    0.670448516948
H      2.927497736105    0.234151471325   -0.821628863541
H      2.201914742034   -1.836755680077    0.184989791895
H      0.998310699555   -0.976439988830    1.248484392373
H      0.253788488361   -0.992743976537   -1.829443740373
H     -0.029848514685   -2.397321142455   -0.694035915050
H     -0.071257364744    1.066523927150    1.445723098132
H     -1.103137097172    1.099104453865   -1.611435737430
H     -2.721357119905   -0.779422091068    1.061359825986
N     -1.780599970741   -1.121269843194    0.892643968049
H     -1.695649418030   -2.119058293143    1.063811906494
O      0.612703715501    2.038905145900   -2.950115828666
H      0.293172862875    1.516976082330   -3.709208847437
H      1.329231670667    2.646833198817   -3.246236256001
O     -0.982205786475   -4.924577014934   -1.839049486193
H     -1.081714452815   -4.205139715376   -2.507961586961
H     -0.322654470383   -4.631001004957   -1.191664600411
O     -2.661190407359    2.022821264095    2.410430680708
H     -2.586575696901    1.748890910730    1.476837452250
H     -1.786395006613    2.349285179525    2.693065377916
O     -4.629026405220   -0.386308542413    0.875926349505
H     -4.615086118204   -1.042745055492    0.156077635580
H     -4.357620361187    0.447324126676    0.472693474353
O     -4.264929928640    0.801065600822   -4.398567927087
H     -4.765659843629    0.538326520925   -5.171101184244
H     -3.596756764080    1.453878745893   -4.720950028163
O     -0.557982224662    0.211249946421   -4.557175945070
H      0.084119279131   -0.507525834075   -4.633600025977
H     -1.272754608012   -0.134216932625   -3.997656375354
O      2.127522787773    5.844055727087    1.615562468843
H      2.149852391497    5.717212032293    0.655177790119
H      2.810751858202    5.258198029817    1.977006476677
O     -3.089553936685    3.799517364422   -0.982443156754
H     -3.509349427920    4.180995379392   -0.189506270038
H     -2.996130207832    2.843090974616   -0.779619770762
O     -2.279552244483    2.329365941271   -5.303997236652
H     -2.003029342510    3.001629527701   -4.655014535136
H     -1.591148496688    1.650721580144   -5.295488242417
```

20439	O	5.289311171094	-1.911523343118	0.482486013422
20440	H	5.989306118991	-2.553997454049	0.361244807694
20441	H	4.873021768532	-1.802216146627	-0.406225660890
20442	O	3.749125974467	2.868156828071	4.832239602957
20443	H	2.921790812575	2.376166882050	4.970341251877
20444	H	4.442197573829	2.193665317560	4.790390398007
20445	O	0.228441509817	-2.529293091554	4.634881764714
20446	H	-0.659894320709	-2.802504885807	4.887260109779
20447	H	0.078687339440	-1.711775332584	4.091318959310
20448	O	-3.584840580623	-0.288418204052	3.452657489033
20449	H	-4.204712018207	-0.492879117869	2.737563854854
20450	H	-3.223443261852	0.582932148810	3.189226261517
20451	O	-0.355791227822	-0.414356402213	3.191032587241
20452	H	-0.783734977269	0.128835197093	3.878945856188
20453	H	-1.033018679263	-0.658269294726	2.532658918727
20454	O	0.978740477163	-5.801682266653	-3.529425876269
20455	H	0.201250033201	-5.708240342293	-2.941853034150
20456	H	0.661564185534	-5.604120397898	-4.412262622672
20457	O	-1.357411861197	3.892966090084	-3.214753761119
20458	H	-2.065867070017	3.842389214145	-2.555545829884
20459	H	-0.674600867613	3.233479568760	-2.959563533507
20460	O	-3.471038214917	4.557824586391	1.614313399323
20461	H	-2.787537703525	5.116467736440	1.995207334283
20462	H	-3.357612178641	3.680961440929	2.021379568008
20463	O	-0.418915839286	3.102984851144	3.496274212772
20464	H	-0.442705567232	4.025571985469	3.122035178356
20465	H	0.505284925523	2.846923190587	3.535974652620
20466	O	-0.463804047916	5.422057131903	2.297757097570
20467	H	-0.669444263162	5.236198778886	1.358279603784
20468	H	0.466188415060	5.719263718637	2.259206150878
20469	O	1.431320374898	-3.622066511866	2.496875497764
20470	H	2.226466121565	-3.053763505008	2.468189400176
20471	H	1.013091996177	-3.377268008195	3.347823121224
20472	O	3.531647130830	-1.888424060383	2.618098304402
20473	H	4.203441564301	-2.023879898630	1.931830030575
20474	H	3.228016155562	-0.964241363678	2.484865800081
20475	O	5.271094529868	0.639477280214	4.104470942059
20476	H	5.271649503918	-0.204977138677	4.558379506046
20477	H	5.809174515463	0.532380246778	3.279397011984
20478	O	-3.078355509158	-4.333835671732	-0.207674705763
20479	H	-2.432336799345	-4.684947888796	-0.851730495510
20480	H	-2.584404772139	-4.223535926416	0.619079277646
20481	O	-0.788193919793	4.908123178021	-0.319004052742
20482	H	-1.632862148310	4.457799392007	-0.559378230603
20483	H	-0.781307647974	5.718419341022	-0.841793242954
20484	O	4.256459362772	-1.828724500461	-1.964923725655
20485	H	3.695474639740	-2.596415431231	-2.156230264691
20486	H	3.833128570169	-1.076873038370	-2.407392887596
20487	O	1.793683668264	5.187516154066	-1.144071158478
20488	H	1.399791696446	5.875246282682	-1.700828397128
20489	H	1.018724434098	4.720681499189	-0.787358423519
20490	O	-1.452240346538	-3.743473417868	1.972902241508
20491	H	-0.501728636183	-3.820976024332	2.120995861360
20492	H	-1.839576325633	-3.433938518573	2.812556878999
20493	O	3.022940788463	0.755563032726	2.606895177642
20494	H	2.262272882121	0.902514249211	3.190977540470

```
H      3.806594936733    0.818463126466    3.193750059333
O      1.610043029926    1.124601162004    4.906139694538
H      0.712141531604    1.101652697895    5.250325979453
H      2.012398099862    0.256632705337    5.140517541809
O      4.677887672154    2.144212970370   -1.919390158894
H      4.531347091548    2.190394053208   -0.949829846460
H      5.624680770945    2.202045524248   -2.049093953600
O     -0.224127012657    6.306461704874   -2.696140616780
H     -0.357901543422    6.918184618393   -3.419066489459
H     -0.609806658349    5.449178209441   -2.985809177528
O     -2.670678427971   -0.901459840822   -3.083219277457
H     -3.311457646176   -0.324801960319   -3.554651861661
H     -3.172267084686   -1.287327380191   -2.343884265128
O     -1.131762282228   -3.065957922570   -3.729477231241
H     -1.750206667022   -2.335039034220   -3.551703664582
H     -0.280903882527   -2.654242420499   -3.930921288702
O      2.698913645882    3.657201976599   -3.222946595586
H      2.444401181000    4.273853095064   -2.510043846962
H      3.475022723405    3.191302348157   -2.880189344327
O     -4.192975932484   -2.168118655784   -1.161385611032
H     -4.968491655287   -2.493631271406   -1.620287197145
H     -3.751649135538   -2.976103279734   -0.777559631131
O      1.371090351975   -1.782413065377   -4.026935023764
H      1.731641341080   -2.498163113492   -3.466474626224
H      2.025459395126   -1.073110344540   -3.969246153814
O      6.306952249998    0.395156390206    1.683846635513
H      5.980375536820   -0.419324171176    1.266005855996
H      5.788018974659    1.115415450358    1.280201060564
O      2.319727602874   -3.752265134546   -2.403024968295
H      2.024171844136   -4.544426238756   -2.898418810895
H      1.940811678245   -3.865040754915   -1.515318503039
O      1.116478630919   -4.472100812122   -0.025425927372
H      1.212723787324   -4.108459540912    0.880079422037
H      1.348652781471   -5.400249246695    0.042810410074
O     -2.556007592961   -2.636250432314    4.228518993559
H     -3.328012356724   -3.078994183934    4.581054844246
H     -2.861079605959   -1.737240758181    3.965691206164
O      2.656483401243   -1.336200982848    5.244169473550
H      3.085031317049   -1.618221809735    4.422431253065
H      1.840432524895   -1.857170102078    5.288343616288
O      2.942948817123    0.365300348852   -3.203433071204
H      3.659317179698    0.976486178079   -2.975125841897
H      2.128994388087    0.840701398633   -2.965126479882
O     -1.339917522952    1.182282166012    5.176170225832
H     -2.290728263023    1.064619064619    5.229244496742
H     -1.179943949399    2.037036444191    4.736964337364
O      4.167540218442    4.216168291411    2.598785379077
H      3.939799813040    3.728521448925    3.432457544279
H      4.876657520508    4.814875655128    2.835883044880
O      4.459998289830    2.215653808398    0.783795340819
H      3.789023975379    1.656119074011    1.215614876390
H      4.438902243296    3.041971379243    1.301617639768

Ambimodal TS Water45-33
155
ANGSTROM
```

```
C      -0.041877333162    1.600385577925    0.966196397304
C       1.047853125534    1.650927898295    0.153469174958
C       1.884514917193    0.530290804298   -0.210209375490
C       1.687705436212   -0.753035033676    0.232021281475
C      -0.236168060332   -1.370350016940   -0.806706355890
C      -1.272900443413   -0.633087446914   -0.254862148967
C      -1.609750482912    0.683891330567   -0.795831771208
O      -2.662640656188    1.313490875162   -0.545485069404
H       1.242912814603    2.581395765521   -0.374998876140
H      -0.654780497476    2.470634388986    1.148112526059
H       2.604463410112    0.705574658373   -1.006313163037
H       2.306903062213   -1.562099589810   -0.127963633137
H       1.179794392220   -0.952831893079    1.161942011222
H       0.154215877369   -1.086962268452   -1.769508929489
H      -0.089778130476   -2.404775884935   -0.531517152022
H      -0.246910922983    0.771075389616    1.624597158601
H      -0.949735998505    1.059478704618   -1.595278774697
H      -2.661021438912   -0.435124997270    1.268689015447
N      -2.046469187351   -1.088033998185    0.799031359617
H      -1.769343490535   -1.901210150143    1.318879678475
O       0.686090530532    0.917712949241   -3.483709760974
H       1.202030309708    1.697080318513   -3.234982204824
H       1.306349150220    0.207551639575   -3.693793024150
O       0.331690127038   -1.565170951222    3.084076794941
H      -0.318880509349   -0.850216443427    3.035742660364
H       0.206660064181   -1.927907653523    3.987446887122
O       1.677050687908   -3.839192766491    2.250137778766
H       1.227702145176   -2.980939963778    2.266074596837
H       1.481054223016   -4.245934916426    1.369478537807
O       5.009358348235   -1.386321593282    0.713520753368
H       4.609030413208   -0.541996328958    0.971626586565
H       4.831167727882   -2.001955739221    1.462338576575
O      -4.074956391448    0.725626048626    2.062851965409
H      -4.554518667960    1.324099812894    1.465449888022
H      -3.854261030659    1.257378919760    2.840525116369
O      -1.108511599907    3.824280837828    4.379008484748
H      -1.928528166042    3.338264564594    4.543372460729
H      -1.197175653973    4.167204518333    3.478298030603
O      -4.716410211720   -2.714431531812    0.283633933379
H      -4.000437670209   -2.846012207955   -0.368989586215
H      -5.425087768501   -3.302833534761    0.022131529511
O      -5.106959184488    3.964109013151   -1.382854433116
H      -5.161219111299    3.367698271999   -2.143869786757
H      -4.154779235723    4.129549009568   -1.233386633894
O      -2.034754657809   -4.220055542069    2.931530385084
H      -1.194450317031   -4.496268758162    3.362207102661
H      -2.681004468027   -4.901886739189    3.120335369011
O       0.247084646125   -4.856650058107    4.183108220879
H       0.556645213667   -5.739542194403    4.384955245378
H       0.866425747811   -4.503451942985    3.480904543648
O       1.697290485961   -3.423453031014   -2.465416562953
H       2.538616703150   -3.240114952768   -2.017282892164
H       1.682970613534   -2.787261860691   -3.197376005211
O      -3.489986078638    2.312559608388    4.291730242126
H      -4.098850983241    2.265356660551    5.028718586159
H      -3.776507922888    3.101299829491    3.729557833573
```

20607	O	-0.910868931740	-1.511080298795	-4.842236423264
20608	H	-0.970641042745	-0.688315932216	-5.382877560585
20609	H	-0.004138514508	-1.490028219793	-4.506637246038
20610	O	-2.901992760224	-2.991251686775	-1.686023373881
20611	H	-2.333019605000	-3.501280315128	-2.302282560987
20612	H	-2.790353660774	-2.065880075839	-1.973445460421
20613	O	-5.522494891739	2.758806888225	0.911222902095
20614	H	-5.371592557277	3.185427061616	0.026494812965
20615	H	-6.464092321773	2.600925783065	0.974128316659
20616	O	4.182642344200	-2.665851599133	-1.394700883786
20617	H	4.476377933279	-2.143541083993	-0.594880167516
20618	H	4.811393444265	-3.383917029363	-1.474892760262
20619	O	4.238823173638	-2.909231954387	2.773835392103
20620	H	3.453000592446	-3.422413792795	2.524845857551
20621	H	3.890426201941	-2.186132172878	3.323550639970
20622	O	4.227813001093	-0.893981757780	-3.458166888896
20623	H	4.289393051766	-1.483189487715	-2.679757236839
20624	H	4.206707758187	0.017988164749	-3.119473890378
20625	O	0.561542381604	1.731027478286	4.550368923742
20626	H	0.084886429163	2.581047234154	4.648345627066
20627	H	-0.112028304146	1.125701959614	4.194448543491
20628	O	2.850190822512	-0.790701174595	3.807675505836
20629	H	2.634771074646	-0.560243270137	4.739603507597
20630	H	1.986376594634	-1.023329233923	3.417229436038
20631	O	-4.146116961318	4.255379133897	2.740293442713
20632	H	-4.643256540763	3.815371856156	2.023672115774
20633	H	-3.307002924669	4.549562027850	2.354077467744
20634	O	-1.591568060124	0.240562262388	3.741067344484
20635	H	-2.187454459319	0.984495750970	3.889935473747
20636	H	-2.076085737287	-0.555520364654	4.053438444622
20637	O	0.994326282719	-4.852932333419	-0.079162603586
20638	H	0.016875864182	-4.894250176693	-0.047313549977
20639	H	1.223512776350	-4.460996553381	-0.930476386732
20640	O	-1.667337021552	-4.628892684046	0.208347901667
20641	H	-2.112733034485	-4.008702033434	-0.388540485299
20642	H	-1.870272522024	-4.360848621839	1.116119007731
20643	O	2.343573615067	2.934860129793	2.844376780824
20644	H	2.898642418188	2.245506960046	2.442917088029
20645	H	1.755431845295	2.472088397263	3.471053645763
20646	O	-5.247615063157	2.294982197792	-3.642186847069
20647	H	-5.856039837333	2.524727028881	-4.344352125895
20648	H	-5.361271762655	1.333928221087	-3.458330874436
20649	O	1.908955050106	-1.441621960182	-4.425843565846
20650	H	2.026753378269	-1.609609613257	-5.362008579546
20651	H	2.830182003375	-1.233667343751	-4.074461565782
20652	O	-4.986226602818	-1.645380515724	2.907482194961
20653	H	-4.889505061457	-2.172812063248	2.103967036324
20654	H	-4.773130111173	-0.734571997428	2.615945508240
20655	O	-0.801271928483	0.986079322365	-5.751804616006
20656	H	-1.590381441461	1.433109704910	-5.416324609504
20657	H	-0.162121664188	1.084692150538	-5.016194200134
20658	O	-1.052199900263	-3.952427352434	-3.371658197756
20659	H	-1.064605117425	-3.269471300742	-4.054305052409
20660	H	-0.164397529384	-3.942798097215	-2.991970162492
20661	O	-2.464454457180	3.947218740054	-0.798626600382
20662	H	-1.822280024457	4.052372041716	-1.520183312370

```
H     -2.569032300611    2.972959894732   -0.680748249924
O      4.022925241894    1.736495699085   -2.671842938255
H      4.795096614458    2.269924271855   -2.860533975527
H      3.252597805181    2.312778100009   -2.811609640677
O     -5.033076780653   -0.059954214891   -0.599019221887
H     -4.206763510829    0.451814860379   -0.492804971356
H     -4.885033538635   -0.910518250574   -0.154914510321
O     -2.538253396230    2.007345985402   -3.872358621739
H     -2.456692897756    1.153209189548   -3.403784013167
H     -3.473154210114    2.257586167650   -3.789614246119
O      1.837912561861    3.410861348107   -2.835176167631
H      2.179054379354    3.910510669433   -2.066625972189
H      0.898590896979    3.654552232222   -2.905588430422
O      4.039792825248    0.870850519197    2.126051340843
H      3.588923525853    0.261079223087    2.761555392372
H      4.871541554818    1.107582996719    2.540193952016
O     -0.883667805287    3.926462737355   -3.057928436424
H     -1.409670145164    3.148473289068   -3.366213691716
H     -1.028757151279    4.619020929245   -3.703644039859
O     -0.261567607103   -2.445016279059    5.552037184793
H     -1.217756744805   -2.313548973236    5.481526727337
H     -0.087880497806   -3.378658933790    5.361189302806
O     -2.820040691282   -2.051652730282    4.393687349853
H     -3.714665182494   -1.873531127976    4.020069898274
H     -2.487883046681   -2.770049255634    3.825151564239
O     -2.659271346942   -0.604097805947   -3.020331958085
H     -2.055879358606   -0.981937227642   -3.699947136215
H     -3.564930199456   -0.659678732842   -3.357023723362
O      2.849057097964    4.527958606613   -0.561428460458
H      3.425459788248    3.877560362998   -0.153524520159
H      2.191366468659    4.762940754054    0.122561023817
O      1.184838134018    4.920634514792    1.575131583133
H      1.413460464656    5.670288928642    2.126778620139
H      1.558266268542    4.127114614002    2.042577278046
O      1.669769658001   -0.239283680303    6.122221902499
H      1.260206345868    0.563423444169    5.752467263356
H      0.986331090781   -0.920110098655    6.140334363471
O     -5.427070236358   -0.324094347544   -3.132245311594
H     -6.231384435528   -0.831560387695   -3.238289494817
H     -5.280200759943   -0.233064286246   -2.137874707583
O     -1.578900047078    4.650641454726    1.671839212001
H     -0.631722259460    4.840156458034    1.560040284347
H     -1.911867282879    4.465566480108    0.770487371749

Ambimodal TS Water45-34
155
ANGSTROM
C      0.169217312578    1.619358840456    0.851149535604
C      1.243423060056    1.546760297631    0.027262792332
C      1.986787614563    0.340421355154   -0.287502255419
C      1.739988020946   -0.875256939661    0.267349873736
C     -0.326212644630   -1.460409932307   -0.817849422997
C     -1.278299198946   -0.682182694241   -0.199177835658
C     -1.555285417768    0.669566808344   -0.689730486189
O     -2.494441955687    1.401530510142   -0.293700161163
H      1.531883392460    2.435239181509   -0.530329068422
```

20719	H	-0.362199715979	2.551525526188	0.985997183581
20720	H	2.690785691213	0.424206214756	-1.110228920750
20721	H	2.277796835046	-1.756211350844	-0.047425664647
20722	H	1.186982988953	-0.963382632704	1.189001611908
20723	H	0.119836191380	-1.164774381713	-1.750224600254
20724	H	-0.195344818174	-2.494707299251	-0.544392201102
20725	H	-0.093050118551	0.835042990069	1.545789090084
20726	H	-1.015048413412	0.979274677086	-1.599791406999
20727	H	-2.771516091753	-0.594479946843	1.221081750826
20728	N	-1.936397095702	-1.085817110742	0.951131878358
20729	H	-1.924375882176	-2.061262177836	1.222377008992
20730	O	1.541654389552	2.589609872996	3.286169033580
20731	H	1.506499583371	2.155822395567	4.151929305057
20732	H	2.232223796798	2.119060455577	2.788709076574
20733	O	0.834527198956	0.754097382951	-3.622421043534
20734	H	0.063261968669	0.152073236247	-3.614527656672
20735	H	0.878352323588	1.071431086207	-4.529272865549
20736	O	-1.175938283573	1.602183953286	5.722450727583
20737	H	-1.571197059977	0.822903614805	5.284060550546
20738	H	-1.284885408749	2.317207168933	5.068543096762
20739	O	-3.235431102323	-2.761950931438	4.254414362789
20740	H	-3.729348550595	-3.186949498344	4.955763327658
20741	H	-2.332892281238	-3.162730447792	4.279052872101
20742	O	-3.058273549487	1.513500134654	2.347579798150
20743	H	-2.869088655634	1.480636976277	1.388625830576
20744	H	-2.430165988766	2.152714650798	2.744207112221
20745	O	4.429640499101	-1.718476751693	-1.682055696598
20746	H	4.298554729949	-0.970088630517	-2.301064354841
20747	H	4.522511907650	-1.315134336049	-0.803855871188
20748	O	4.732082740406	-0.958075759987	0.995785081891
20749	H	4.197949481401	-1.566617592560	1.556650617192
20750	H	5.635548905174	-1.024499740099	1.308784575274
20751	O	0.783053402466	-3.555622101542	2.236392604119
20752	H	0.508894255182	-2.616226138795	2.358882823483
20753	H	0.936483560613	-3.712358676093	1.286659009046
20754	O	3.779536374800	1.451864751371	2.015946407902
20755	H	4.033536523763	0.661825015984	1.508651303753
20756	H	3.912147262219	2.198599126729	1.403153069734
20757	O	-1.240451361678	3.131594542987	3.487754891339
20758	H	-1.596521543065	3.977115383879	3.124395361506
20759	H	-0.299679980820	3.069635874001	3.269528189258
20760	O	-0.044514682540	-3.120003911993	-3.989794513277
20761	H	-0.417118894138	-3.855467849707	-3.458241770170
20762	H	0.830161609002	-2.962084716948	-3.605811650918
20763	O	-2.552214279400	4.023713393861	-0.347027175584
20764	H	-2.887840071017	4.397108095692	-1.204959391408
20765	H	-2.460417550276	3.053474807703	-0.479292822082
20766	O	-1.258847256627	1.080035286693	-5.805564323733
20767	H	-1.853669868516	0.919685331588	-6.537716638513
20768	H	-1.368484813595	0.328030960914	-5.189339636283
20769	O	-5.687064708750	3.252372660474	-2.265587243031
20770	H	-5.573567011398	3.124010181699	-1.291191708927
20771	H	-6.626721902126	3.328386599760	-2.427492817664
20772	O	2.366746945401	-3.143861960303	-2.573714395133
20773	H	2.796916160756	-3.851798040891	-3.056340401420
20774	H	3.095078769947	-2.563766619343	-2.247494753112

O	-5.072367679857	4.058375198383	2.797310548352
H	-5.262081164921	3.673383025132	1.926017283375
H	-5.244708053253	3.365077130198	3.440107472208
O	3.295495317612	-0.283266097670	4.178362600655
H	2.599511125935	0.189461978542	4.653794574886
H	3.600781537392	0.336686427138	3.501896535347
O	1.904496874248	4.727228938484	1.482669175767
H	1.164629957972	4.688831658143	0.853315865610
H	1.702531646308	4.107713830779	2.198874932501
O	3.579018669493	2.987644480243	-2.466220532541
H	3.703409045863	3.172013485184	-1.522067089062
H	2.713597041593	3.340808260529	-2.715603661886
O	-0.001385364915	4.708213425505	-0.550371666551
H	-0.957646080558	4.491139533162	-0.415596190352
H	0.021783291158	5.619931713890	-0.845433388958
O	0.269902271875	-1.100363887814	3.096396068569
H	0.696807263998	-1.262403787537	3.951300386450
H	-0.637308494836	-0.818231876167	3.329197892565
O	1.140111717776	-1.674037213732	5.673886268328
H	1.119354096870	-0.727006311594	5.896659494987
H	2.064189052393	-1.933829614581	5.678524951676
O	3.367893888959	-2.574693850454	2.650055018219
H	2.481833729752	-2.978272780116	2.581129779370
H	3.285723403051	-1.849914127287	3.296725593934
O	-4.692098015126	-3.793009824624	0.891212490447
H	-4.913748062727	-2.935015238917	1.281649099833
H	-3.979939095805	-4.138105193489	1.443696633300
O	1.307771332360	-4.312251432118	-0.365984011824
H	2.164977877626	-4.656022127679	-0.065785004085
H	1.550082664164	-3.809310763437	-1.171376838807
O	-0.853830138566	-4.960972608808	-2.187448089334
H	-1.648676284279	-4.531290641595	-1.833288946713
H	-0.186972399299	-4.941730676253	-1.487771369504
O	3.943347281010	3.585817616575	0.280462189550
H	3.182670180401	4.071523904803	0.700144350078
H	4.706984452606	4.156990909176	0.368758156302
O	-2.557189902646	5.056453200322	2.268988432231
H	-3.460809342682	4.871735469496	2.583967759726
H	-2.568088270327	4.831839111500	1.327399446719
O	-5.104062908481	2.803933829207	0.290655101028
H	-5.019269856556	1.827002802178	0.208932713534
H	-4.206828678451	3.163930188787	0.213553257721
O	-3.914971246259	1.423835019750	-3.341906660938
H	-4.586042119547	2.024682560117	-2.978784911004
H	-3.110773623121	1.955241752499	-3.474829902517
O	-0.744477588067	-3.642741006527	4.659819665097
H	-0.237081388425	-2.994995255932	5.166163562740
H	-0.206001433344	-3.848950019568	3.884602901360
O	-1.918528142284	-3.956962177199	1.526708323042
H	-1.952382764760	-4.086946384893	0.570074664264
H	-1.012277251078	-4.131422866205	1.814976310712
O	-1.752309170690	3.017458225350	-3.951027881926
H	-0.868838578532	3.272653686822	-3.617500008524
H	-1.574697251137	2.470029137916	-4.737985473838
O	-2.095666742341	-0.309012790215	4.077635964127
H	-2.611852019171	-1.121060964243	4.205366410279

```
H      -2.553561903235    0.246481177455    3.416348228948
O       3.978380237043   -4.145405583168    0.240071938989
H       4.335196969941   -3.492483299087   -0.371813248993
H       3.985690049436   -3.745059025373    1.117285537555
O       0.730111541385    3.405437455639   -2.881633075934
H       0.807759649587    2.434863553476   -2.863487126089
H       0.509027121655    3.726366596452   -1.989405794056
O       3.643658859461    0.350056677458   -3.251457686054
H       3.811797825436    1.286183733268   -3.037763251412
H       2.692754953633    0.304057769404   -3.426417679251
O      -3.415148282760    4.802078615812   -2.692494508975
H      -4.297166483796    4.400822480822   -2.715541760579
H      -2.858301348823    4.282461427812   -3.297248180952
O      -4.701833948253   -1.402742440604    2.306938134301
H      -5.142649776040   -0.676612162132    2.787829879077
H      -4.188415406174   -1.904898408943    2.959002768167
O      -3.700134097077   -0.922631098255   -2.260043666412
H      -4.385462121734   -1.417007624788   -2.716056526595
H      -3.755753194736   -0.000526020866   -2.639419724931
O      -5.614090616879    1.064483970692    3.022164600278
H      -4.695584918978    1.325053829244    2.799822002358
H      -6.139148313204    1.259568856430    2.242307944755
O      -2.898726287648   -3.469065745637   -1.089799293922
H      -3.676212336704   -3.647248976527   -0.518863328189
H      -3.020445546891   -2.579659121499   -1.437124987242
O      -5.089335119627    0.126690052948   -0.004909841248
H      -4.535383598688   -0.218130068075   -0.720716555320
H      -4.977689233415   -0.475044183456    0.744153362166
O       1.334647882405    1.105505871625    5.728098719201
H       1.689447425972    1.557573863254    6.494217014724
H       0.335533059747    1.293678245916    5.737570413125
O      -1.336943270001   -0.851562688250   -3.892962427377
H      -2.088635052024   -0.973908329987   -3.297437243718
H      -0.904274589222   -1.740025943351   -3.997431573110

Ambimodal TS Water45-35
155
ANGSTROM
O      -3.083433462306    1.174915809399    2.362235780868
H      -3.065352496391    2.107170343856    2.073757142577
H      -2.403479395872    1.120178486390    3.057841662323
O      -0.603951619103   -3.947966892165    1.887104556345
H       0.128573655655   -4.589882388766    1.917832674612
H      -1.355854995208   -4.322011627170    2.385069027559
O      -5.942440675924    2.251042230094    1.070162518688
H      -5.518927950096    2.431407162730    1.913843006926
H      -5.478821120403    2.817713733060    0.419664565578
O       0.201125927869    4.917937817101   -1.612192814532
H      -0.356406320199    4.276445593137   -2.102180215242
H       0.175076020758    5.732667108203   -2.114822012566
O      -4.937060738583    0.169755880614   -0.279559880517
H      -4.003772241173    0.488687468783   -0.235283672286
H      -5.431203087532    0.828177089955    0.252047753528
O       1.494558774675   -5.644253750301    1.624551609791
H       1.707322217106   -5.678202836854    0.658256269217
H       1.643197430030   -6.527015423111    1.963367540063
```

20887	O	1.288111366886	4.289165580620	2.430918946476
20888	H	1.904838008666	4.388779750043	1.687545950012
20889	H	1.363207759877	3.371203866135	2.735438115354
20890	O	3.921370740254	2.787074956788	-1.967369873480
20891	H	3.005429830359	2.786587479325	-2.312610487835
20892	H	4.362073167060	2.145563951742	-2.559327844409
20893	O	1.527284940220	2.334318738592	-3.131322226723
20894	H	0.651182921105	2.524076834287	-2.777523867417
20895	H	1.603742734078	1.368472786301	-3.209310017312
20896	O	4.818253936121	1.518876130980	0.184684752882
20897	H	5.534457266166	2.066887117332	0.508982672403
20898	H	4.398029736444	2.026970774979	-0.554773988846
20899	O	3.015966744292	-1.566026606373	3.273661320986
20900	H	2.045474618069	-1.583626312419	3.131229576216
20901	H	3.357824398666	-2.355113372135	2.811469280728
20902	O	2.238487890496	-5.609394065415	-0.915364412797
20903	H	2.908156801312	-4.911427485816	-0.939198777443
20904	H	1.576900191060	-5.390340848614	-1.582668416265
20905	O	4.512290657914	0.514245363593	-3.343084107559
20906	H	4.774770810541	-0.094949144891	-2.636094816392
20907	H	3.616472407771	0.260081054838	-3.602484603485
20908	O	-4.521247683201	-2.455447509891	0.286817583252
20909	H	-4.834190010815	-1.550394850864	0.104108713083
20910	H	-3.973239942928	-2.355351339073	1.080560195513
20911	O	-2.992964460623	-2.775179914359	-1.927358099567
20912	H	-3.669230938715	-2.628027030020	-1.233543157075
20913	H	-2.330190341856	-3.328335851025	-1.464115873717
20914	O	-2.941229882129	-4.706106962975	3.061094315879
20915	H	-3.240245378806	-3.755498424790	3.051807539404
20916	H	-3.131551475269	-5.036029926305	3.938965856897
20917	O	3.777683866242	0.946326833535	2.722733978148
20918	H	3.610688240770	-0.012076162604	2.848601658999
20919	H	3.972673682490	1.080732783004	1.784626562432
20920	O	-2.179709676462	-1.084612106829	5.017955663576
20921	H	-1.427743647155	-1.505287511508	5.458044895506
20922	H	-1.879293352137	-0.186406504914	4.806190626197
20923	O	2.187553099389	-0.338140539971	5.587080879800
20924	H	1.858242692760	0.435023486138	5.102180220076
20925	H	2.718566926670	-0.826503222251	4.935150912695
20926	O	-2.667975672021	-0.328847112339	-3.039146043789
20927	H	-2.777253519732	-1.183664795509	-2.581669819961
20928	H	-3.553919133300	0.045543618101	-3.124699682304
20929	O	3.601524315037	-3.767921675339	1.821077026541
20930	H	2.864902501006	-4.393869299994	1.879372312637
20931	H	3.729444450332	-3.589155696339	0.878641511891
20932	O	-1.728990686561	-3.345435849870	-4.296946950946
20933	H	-2.253420828592	-3.077439183503	-3.517404411288
20934	H	-2.318940806017	-3.856175929294	-4.852250075981
20935	O	1.756102837491	-0.335796701712	-3.660976711419
20936	H	1.982835656367	-1.232689474088	-3.365411971354
20937	H	0.908251961609	-0.437006624207	-4.140329895116
20938	O	-0.691436247772	-0.691806240091	-4.761590526085
20939	H	-0.847715561650	-1.635330633445	-4.893360419833
20940	H	-1.393485250866	-0.431755704548	-4.123158462600
20941	O	-1.086385842580	1.338822134597	4.227287924505
20942	H	-1.231486413907	2.297943728094	4.370097386485

20943	H	-0.149768155296	1.268056782554	4.002048929317
20944	O	-1.250093988261	3.148198957496	-3.035190570947
20945	H	-1.033697675674	2.856915398037	-3.944168793934
20946	H	-2.217408424076	3.117983166384	-2.973044919423
20947	O	-4.247372643323	3.487971113235	-0.632421300243
20948	H	-4.347654607923	3.716873339486	-1.561754272486
20949	H	-3.569912853997	2.779712149323	-0.623220094439
20950	O	3.840299688330	-3.238264888070	-0.957934268454
20951	H	4.439483258659	-2.476715976884	-0.904536956994
20952	H	3.284869807042	-3.057186150137	-1.738376864563
20953	O	-1.486586104765	-4.542304541339	-0.576407345336
20954	H	-2.330506145083	-4.981389671283	-0.322011628938
20955	H	-1.105626660662	-4.242860342178	0.274829751595
20956	O	5.311056494954	-0.867874360966	-1.005251397537
20957	H	6.254182861362	-0.988851396126	-0.888648174193
20958	H	5.067319043305	-0.072682937981	-0.483750739747
20959	O	-0.322879022810	2.077647671645	-5.353428363168
20960	H	-0.479388221475	1.123031705916	-5.341306265000
20961	H	0.587714081057	2.205461803910	-5.058014126768
20962	O	0.243859393565	-2.090199157295	5.891197388918
20963	H	0.483324126779	-2.697681915747	6.590033377009
20964	H	0.949694514533	-1.391710004985	5.879008458602
20965	O	-0.845015130476	5.112702891864	0.958261071221
20966	H	-0.534464539968	5.043954518854	0.042316882278
20967	H	-0.070988358448	4.911025197469	1.515778906317
20968	O	1.681099541129	1.909286587719	3.919580573673
20969	H	2.017435096390	2.529848424639	4.569184049658
20970	H	2.473164928937	1.572505624593	3.416616663313
20971	O	0.315388506326	-1.679461376444	3.093438997408
20972	H	0.127147912462	-1.801608464890	4.033165078722
20973	H	-0.001756328865	-2.486180968009	2.651229143215
20974	O	2.597974244854	4.611365219959	-0.044227469242
20975	H	3.228093975579	4.169311010147	-0.623221844867
20976	H	1.819845464251	4.793309675978	-0.590830234371
20977	O	-3.019475964331	3.863440120053	1.811197562725
20978	H	-3.640695662210	3.980571623171	1.081575612842
20979	H	-2.207606494696	4.314592960158	1.483818398827
20980	O	-3.841594994222	-5.212158814256	0.456943527293
20981	H	-4.317833733562	-4.384317610322	0.303019881899
20982	H	-3.609532463772	-5.203225497559	1.398601903278
20983	O	2.230023847201	-2.988696854673	-3.142151479370
20984	H	2.619099158346	-3.389113063502	-3.920880450117
20985	H	1.426311115994	-3.522553367524	-2.924644432866
20986	O	-3.511060693246	-2.171802715365	2.978547120659
20987	H	-2.962971000487	-1.783204168406	3.698487030332
20988	H	-4.333965576918	-1.651166139749	3.020394604663
20989	O	-5.255135859869	0.706207072585	-2.824382883227
20990	H	-6.162984102557	0.523597299350	-3.065719053565
20991	H	-5.176990341361	0.502732828714	-1.849969075168
20992	O	-5.349243012881	-0.200836381485	2.911529000577
20993	H	-6.021875993217	0.117501507466	2.307596774838
20994	H	-4.553266423706	0.351853985437	2.759483895110
20995	O	0.163429073133	-4.567976741995	-2.694626879413
20996	H	-0.440558893241	-4.235046309861	-3.381439156963
20997	H	-0.394144538582	-4.622345212325	-1.892069873525
20998	O	-4.030771511986	3.076777742764	-3.381629014026

```
H      -4.446573025634    2.214514901532   -3.179459728759
H      -4.085154909647    3.193129974733   -4.330819334221
O      -1.311772522025    4.015339063526    4.122969935236
H      -0.522413920933    4.352097262277    3.684974939691
H      -2.036295014981    4.116123414740    3.491632332699
C       0.053817712849    1.564858367979    0.979141956097
C       1.140669061423    1.591406107558    0.160575876532
C       1.938031859342    0.443269063553   -0.200055243581
C       1.688694247420   -0.829601194675    0.249319056676
C      -0.193700155675   -1.355822280918   -0.854419431086
C      -1.207658431567   -0.596743001657   -0.289035418837
C      -1.549467203676    0.733084207603   -0.805717305635
O      -2.590113847662    1.353191652227   -0.511581050374
H       1.366709925391    2.516347540234   -0.365010862170
H      -0.544499749395    2.448459986590    1.149305147926
H       2.643613602770    0.578957480093   -1.015251790220
H       2.292682778402   -1.659896424518   -0.086575072129
H       1.164799445960   -1.000372012692    1.177094096113
H       0.201276471653   -1.082816498654   -1.818905545721
H      -0.074894905346   -2.393848599808   -0.582040641831
H      -0.160283238540    0.736018806574    1.638238962301
H      -0.909618105392    1.134964139246   -1.607212100190
H      -2.466650241018   -0.353612532913    1.316848545265
N      -1.978519218542   -1.053797676176    0.767217358396
H      -1.649246606649   -1.840787547764    1.301404103589

Ambimodal TS Water45-36
155
ANGSTROM
O       4.000854500526   -2.486303603732    2.314149229468
H       3.878004631560   -1.529653520890    2.417123939932
H       4.653904626732   -2.586121637070    1.605384184555
O       3.360729953725   -5.292194563218   -3.077550682211
H       3.003418899471   -4.752598423222   -3.838076901332
H       4.062852380893   -5.831833848712   -3.440321881320
O      -1.652620696379   -3.604518038221    2.282257349313
H      -0.868196210267   -4.180201430236    2.488066898625
H      -2.147566241522   -4.047253018246    1.557773443785
O      -0.159967310844   -4.044238155781   -5.386803756731
H      -0.485887733687   -4.703612924368   -4.718472244008
H      -0.228165627790   -4.479385910246   -6.237657224041
O       0.358796049540    0.139936622303   -3.714221675397
H       0.501602294995    1.056692099400   -3.445760843776
H      -0.604777202574    0.035857248298   -3.839690557310
O       3.635673097250    0.312252347059    2.571341557171
H       2.838005360295    0.287685785749    3.129697875877
H       3.865403032350    1.262235973113    2.570845886308
O       1.445482630934    4.839466392809    0.841856171541
H       2.342245366273    4.616016479371    0.569468168150
H       1.283776626726    4.355580667919    1.666647821935
O      -1.238003613489   -5.695007185102   -3.638457726674
H      -2.180263343321   -5.510719199579   -3.617600852491
H      -0.902050622204   -5.473357938848   -2.740727092989
O       0.021910879520   -1.340919035186    2.979317468332
H      -0.470888303109   -0.564955246119    3.284460229132
H      -0.616535609732   -2.058218281051    2.883698924390
```

21055	O	-2.562311318558	-3.346724914715	-2.248134977124
21056	H	-1.638540069746	-3.509691579355	-2.017094226300
21057	H	-2.578948694712	-3.161256557333	-3.220111084811
21058	O	4.024131860931	4.056545566275	-0.186655057282
21059	H	4.772369810305	4.643460583061	-0.063031381101
21060	H	4.368286260612	3.253366057834	-0.650798248308
21061	O	3.441018593372	1.267863129126	-3.424302333491
21062	H	2.574909048902	1.688922734232	-3.381177198483
21063	H	3.277114556467	0.308170814742	-3.536543811658
21064	O	1.862433884542	-2.927409009909	4.140289991166
21065	H	2.726263715255	-2.873600071489	3.714205266918
21066	H	1.295472757591	-2.282821822732	3.668105313338
21067	O	-0.311751943963	4.361740379127	-1.193387091005
21068	H	-1.180868224917	4.278193859069	-0.771797946496
21069	H	0.298229012065	4.575728924813	-0.461344894261
21070	O	5.484933853642	0.064781052301	0.469271901397
21071	H	4.837079312858	0.154248548787	1.184458242830
21072	H	5.252731223808	0.726880358623	-0.200272425525
21073	O	-0.467052538263	-4.905706442198	-1.181501554405
21074	H	-1.062485925779	-5.238082457853	-0.504129393419
21075	H	0.369983092612	-5.428743031157	-1.092051033910
21076	O	2.509577078904	4.932909742190	-2.421594153875
21077	H	1.751000137299	5.430679660885	-2.102699269274
21078	H	3.012049793733	4.671972138375	-1.634302809984
21079	O	0.895230005269	-1.044117648558	5.983324209709
21080	H	1.276669274760	-1.842085679244	5.586139309679
21081	H	-0.070647946379	-1.147101589022	5.927013094397
21082	O	-2.462296633926	-2.644238089358	-4.797034306632
21083	H	-1.592194627332	-2.956883208575	-5.098205326110
21084	H	-2.397866339943	-1.680418217657	-4.698883410983
21085	O	-3.199616509496	-4.440297762815	0.258972955191
21086	H	-3.934533607767	-3.869348311525	0.558158597650
21087	H	-3.032976055098	-4.149248687647	-0.648150710881
21088	O	-4.250396991843	2.816578105177	-1.851508040563
21089	H	-3.694907354833	2.140271737068	-1.438205680446
21090	H	-3.940156262187	2.864608319768	-2.764653304031
21091	O	0.556931807010	-4.902802700835	2.760757816657
21092	H	1.004276889038	-4.360921234779	3.438683842037
21093	H	1.128114424766	-4.828323382244	1.978463867542
21094	O	-1.817274346275	-1.085843840300	5.468617967487
21095	H	-2.444782894742	-1.649602342104	4.998467426857
21096	H	-1.783730620847	-0.259820077194	4.962274537882
21097	O	1.064982228165	3.305178943781	3.195700208673
21098	H	0.110089233796	3.466710426361	3.194562034729
21099	H	1.171739269259	2.415624239390	3.579653235463
21100	O	2.533379548043	-3.691082044582	-4.967876779604
21101	H	1.573882935297	-3.772067309053	-5.116339789855
21102	H	2.668770018326	-2.799571561807	-4.595892900369
21103	O	3.816189744210	3.020197621425	2.469724778409
21104	H	3.844106710052	3.324604468368	1.552572606433
21105	H	2.935634076140	3.237497188180	2.804412928711
21106	O	-0.591165842449	2.829446345668	-5.292884760916
21107	H	-0.320399494314	1.989689464662	-5.671668641620
21108	H	-0.022972141800	2.962313068954	-4.503853533501
21109	O	-4.793949692780	0.221850679623	0.579168540576
21110	H	-5.421673017648	0.816845440217	0.166735470322

21111	H	-3.914619897901	0.475317197562	0.221070941984
21112	O	-3.593144946684	-2.610075140552	3.742625409002
21113	H	-2.834548767895	-3.013490769419	3.244642674867
21114	H	-3.986383516631	-3.319671858423	4.251786114607
21115	O	-1.736387606867	3.603687229593	2.918191803761
21116	H	-2.259278499345	4.156120675272	3.500143541631
21117	H	-2.054915905166	3.773880107969	2.010305876575
21118	O	1.775746982741	-6.279555596717	-0.980771224872
21119	H	2.166550838258	-5.844689892136	-0.209304597344
21120	H	2.312811713918	-6.022392832738	-1.746821018217
21121	O	-4.309031221247	0.142739950212	3.263708422354
21122	H	-4.604727886977	0.178057221833	2.335499394034
21123	H	-4.260004304363	-0.788533372869	3.511919071579
21124	O	2.675060030524	-1.337982826855	-3.534421451855
21125	H	2.779008945590	-1.849602979506	-2.707964785659
21126	H	1.762642482327	-1.003992637289	-3.572119257181
21127	O	-2.340833866203	-0.008417439940	-3.974981565277
21128	H	-2.699792912658	0.868206468916	-4.199569063649
21129	H	-2.822209364072	-0.286558935384	-3.167513761435
21130	O	3.291421511502	-3.084574915013	-1.496820731413
21131	H	3.395049353910	-3.885688946414	-2.041714806412
21132	H	4.160719225533	-2.898396910189	-1.101143964397
21133	O	-1.743731543266	0.815704215751	3.440971567402
21134	H	-2.712057307408	0.655532682576	3.377812932130
21135	H	-1.631680648727	1.768212655789	3.321413226279
21136	O	5.027818217594	1.985795456806	-1.502259857456
21137	H	4.403591977846	1.700246724584	-2.229837545003
21138	H	5.865120772336	2.156870710005	-1.935444221168
21139	O	-3.697474017807	-0.952796807172	-1.850163389703
21140	H	-4.644655394842	-1.087320754195	-1.902071318420
21141	H	-3.294950051012	-1.847650715556	-1.839338757421
21142	O	-2.610369673055	3.868537917478	0.320128360026
21143	H	-3.400951409907	4.133422236034	-0.162084608308
21144	H	-2.536566523121	2.907383459238	0.132628513172
21145	O	5.548274314566	-2.497691864834	-0.055355715145
21146	H	6.464961203171	-2.764834307970	-0.117625653870
21147	H	5.549787961599	-1.513127335875	0.070112406880
21148	O	1.731324150447	0.927763490752	4.453963563571
21149	H	2.369778630554	1.332252849847	5.044332285340
21150	H	1.331816561994	0.188177668419	4.986051339024
21151	O	-3.112877879445	2.575562511144	-4.482726904987
21152	H	-2.190524641186	2.796177185660	-4.757931334934
21153	H	-3.681227641637	2.856484717714	-5.200300496799
21154	O	2.458788571115	-4.304078538406	0.845750206828
21155	H	2.972642033212	-3.785694105884	1.486203242017
21156	H	2.563027725389	-3.833079044086	-0.000001598585
21157	O	-4.906977579986	-2.539141594396	1.186135018973
21158	H	-4.565161458948	-2.494328895358	2.090063464684
21159	H	-4.830162220119	-1.643542999993	0.825116951048
21160	O	0.907071363561	2.870841524964	-3.052274978729
21161	H	0.304027370123	3.253264257023	-2.375555314988
21162	H	1.611777643563	3.549904029888	-3.087643758739
21163	C	0.242559324147	1.618285195451	0.812724095912
21164	C	1.338657768588	1.538706767784	0.016322434076
21165	C	2.091590914890	0.328712699525	-0.245917344245
21166	C	1.827057004444	-0.885882404022	0.305439291887

```
C     -0.190995276599   -1.472666934583   -0.887623014187
C     -1.158744922156   -0.707197544296   -0.274424114612
C     -1.469092971001    0.651624067688   -0.713144416985
O     -2.476824966783    1.287545836444   -0.306995502060
H      1.625420979637    2.409619284913   -0.571243813764
H     -0.303553851351    2.542786440140    0.925477953294
H      2.831602624856    0.392012811442   -1.041754712518
H      2.394329042072   -1.750183946051    0.006337575537
H      1.238877389414   -1.006512788751    1.202561415371
H      0.236947723742   -1.161384130253   -1.824996564114
H     -0.046551041370   -2.510869045614   -0.624407370269
H     -0.013421846595    0.847462311062    1.523162164801
H     -0.918992905995    1.060105569261   -1.573585781016
H     -2.358996966626   -0.498737756361    1.383117523011
N     -1.925641607783   -1.171580843842    0.774363990233
H     -1.711112567355   -2.065836325431    1.186881891883

Ambimodal TS Water45-37
155
ANGSTROM
O      2.336076908995   -0.886630145824    5.336447074155
H      2.718707140253   -0.521074626335    6.133908340132
H      1.813923128168   -0.173535342122    4.908679496168
O      3.395385397092    1.864497221120    2.586260814466
H      4.006158537244    2.159400083400    3.264933075928
H      3.846390389544    2.112901765176    1.719788666200
O     -4.822998337177   -1.824417705724    0.075043060257
H     -4.669277313282   -1.377612767757   -0.766206006303
H     -4.818039845447   -1.148313631168    0.761842392762
O      1.331784809069    3.967627810029   -3.483032886687
H      0.744731920771    4.381434104983   -2.836608640462
H      0.858670569272    4.045566331159   -4.349242085249
O     -0.312250579626    1.173492758453   -5.784481407839
H     -0.039739451024    1.215241548667   -4.836458625882
H      0.158307771071    0.415163590500   -6.143298800470
O      1.098311864953    0.934042031656    3.838436900847
H      1.803215014829    1.260124961753    3.259815208556
H      0.416278996952    1.646700341210    3.866735853070
O     -0.727801491408    2.864578160153    3.683334825000
H     -1.458632939122    2.872286105911    3.036344672531
H     -0.051685224946    3.466187935283    3.323297267416
O     -2.824008796227    2.840599421168    1.903486158247
H     -2.650634244838    2.211082183876    1.177719637341
H     -3.793560032376    2.731171623659    2.069578764953
O      2.135021263108    4.970998770087   -0.056046725789
H      2.669810618625    4.406038816787   -0.645328321804
H      2.538606820923    5.839537213923   -0.080102881739
O     -0.672008069878   -1.053254911055    3.671210573724
H     -0.592648918753   -1.746720424970    2.990114472374
H      0.079793599810   -0.441419795720    3.573557273254
O      3.488736663168    3.477664059540   -1.922496501764
H      3.882872835863    2.661165173495   -2.278292303920
H      2.826815628616    3.736253789207   -2.589204667642
O     -0.314152007249    3.920355694934   -5.550701612038
H     -0.266098285825    3.011204346128   -5.889851691980
H     -1.076047757894    3.914629935731   -4.950422402592
```

21223	O	-4.507991167428	-2.554215555849	3.959229147258
21224	H	-4.134487627908	-2.187183164681	4.804071171290
21225	H	-5.331862465954	-2.986188209859	4.183620243557
21226	O	-5.311372289029	1.430334533950	-0.725536508106
21227	H	-5.244209067474	2.291966385259	-1.157056988479
21228	H	-4.396425315750	1.211359655254	-0.479950126924
21229	O	-0.938340535527	-1.359073639618	-3.850661700924
21230	H	-1.418695252262	-0.513911653325	-3.858595347061
21231	H	-1.524086523161	-1.989850598065	-3.406208859059
21232	O	-3.739397942889	3.643037878682	-1.365135165785
21233	H	-3.234038418789	2.872426470609	-1.032990571174
21234	H	-3.467785325836	4.377320913053	-0.798333503083
21235	O	-2.578323922169	0.658047783292	4.431494153449
21236	H	-2.170856367793	1.529131199055	4.445496131859
21237	H	-1.896721600627	0.041178686950	4.094277305150
21238	O	-2.380682120113	5.097593302558	0.579495543206
21239	H	-2.531348095443	4.295178360806	1.140544599031
21240	H	-2.548489019087	5.857147371315	1.136219990357
21241	O	5.878400300149	0.557160038270	-0.715040834064
21242	H	5.449410692016	0.663631095885	-1.577690059327
21243	H	5.600098525218	-0.307306201227	-0.375217107053
21244	O	2.471852979087	-0.568707002947	-3.564737852064
21245	H	2.422488798191	-1.382996447503	-3.030211638982
21246	H	2.072189483277	-0.809662628059	-4.423599585475
21247	O	4.443441050880	1.139349890140	-3.055695489781
21248	H	4.921705678343	1.267631854330	-3.875353540333
21249	H	3.726690571907	0.492611740423	-3.244822932208
21250	O	3.784438409113	-0.907690601145	2.920563624014
21251	H	3.628311454549	0.006940344658	2.634026680451
21252	H	3.420088984346	-0.971052750586	3.816583179021
21253	O	-4.191963921588	-0.543139189400	-2.433162888917
21254	H	-4.862474859891	0.097378558205	-2.158394267097
21255	H	-3.635698603610	-0.074093281740	-3.076769946924
21256	O	-0.168106824139	-3.287379740350	2.311618877423
21257	H	-0.065286743521	-3.809862960313	1.499090462014
21258	H	0.505898555029	-3.575534808848	2.945923125115
21259	O	-2.699246383686	-4.352724117333	2.878768608469
21260	H	-3.302032957182	-3.712420905615	3.292028581261
21261	H	-1.812126729031	-3.960744998513	2.894998765088
21262	O	-2.646087672847	-2.971751499813	-2.406396487903
21263	H	-3.349265009454	-2.311218165468	-2.463254472529
21264	H	-2.713223053508	-3.345100530478	-1.514131858168
21265	O	0.971257720769	-1.402000422945	-5.665501342266
21266	H	1.022772261941	-2.277701481466	-6.048289829135
21267	H	0.224946433466	-1.431095599655	-5.017599017732
21268	O	0.533937326876	1.363376017967	-3.239388925397
21269	H	1.262401854167	0.721744772027	-3.232204561867
21270	H	0.925545346719	2.256354098794	-3.265287358610
21271	O	1.496346596343	4.000948368026	2.513399582554
21272	H	2.158790247845	3.295679700052	2.547155569170
21273	H	1.610374861630	4.404857201566	1.641341575786
21274	O	-0.748008549198	-2.292964912768	6.021113751787
21275	H	-0.282151337129	-1.725276135680	6.636183967851
21276	H	-0.716765574702	-1.843829023458	5.142690953169
21277	O	-5.394023787362	2.184832537595	1.958821689500
21278	H	-5.245952969704	1.287456265343	2.299402147294

21279	H	-5.616081174735	2.047190042012	1.022403873891
21280	O	4.713855880436	2.591561598896	0.515000267182
21281	H	5.242064828355	1.841877365432	0.146044262798
21282	H	4.283778612905	2.992447138183	-0.249732767241
21283	O	3.209157492785	-3.612214089152	2.447086945880
21284	H	2.703977018094	-3.872920851110	3.225224189876
21285	H	3.415682136035	-2.673489652552	2.567379013311
21286	O	2.467360554989	-4.208329396591	-0.083230681174
21287	H	2.763558665379	-4.056389529013	0.833982729483
21288	H	1.589613684050	-4.604885755635	-0.013113375902
21289	O	-2.510845948926	0.913234256359	-4.120611413968
21290	H	-2.129398865590	0.943781250071	-5.009398704963
21291	H	-2.282135525357	1.786465972080	-3.741839751236
21292	O	-3.399405664079	-1.446007187137	6.070535333178
21293	H	-2.538428664069	-1.888239611177	6.172366519783
21294	H	-3.213156061565	-0.577931592825	5.677959689237
21295	O	-0.337092850322	4.603506400693	-1.287969773788
21296	H	0.470080495086	4.732444530613	-0.764961821321
21297	H	-1.070005125467	4.855839981793	-0.706063824067
21298	O	-0.164025181337	-4.411666741743	-2.714196406696
21299	H	0.529325601767	-3.735441483001	-2.768594300924
21300	H	-1.010695817102	-3.963504084303	-2.830616335126
21301	O	-0.318678864441	-4.731433708991	-0.051339976069
21302	H	-0.222526525300	-4.763580516648	-1.029580043619
21303	H	-1.272904574643	-4.644616164901	0.082710504181
21304	O	-1.940284259869	3.458063484941	-3.332575464281
21305	H	-2.762356009131	3.663617403414	-2.849034206932
21306	H	-1.237521995216	3.638697957224	-2.692024073908
21307	O	-4.277161616661	-0.263611368892	2.496624635536
21308	H	-4.480617126854	-1.122400369091	2.909218944550
21309	H	-3.712466541137	0.181941161893	3.156282589633
21310	O	1.405519357912	-3.472168492539	4.601871076566
21311	H	1.794012775944	-2.621319252890	4.854997258195
21312	H	0.625270196608	-3.566482798962	5.164915673247
21313	O	-2.964564661212	-3.790444580155	0.273808052973
21314	H	-3.727783770412	-3.182064306175	0.238351891777
21315	H	-2.986171521368	-4.163223332620	1.180836454893
21316	O	4.865257010085	-1.778333715686	0.373778761980
21317	H	4.644856514531	-1.508220882384	1.273357403953
21318	H	4.649266242182	-2.711101839495	0.308164206411
21319	O	2.290553643540	-3.048532431839	-2.476658328577
21320	H	2.373054115889	-3.360379922201	-1.548402545593
21321	H	2.832038641487	-3.649373513199	-2.990512274248
21322	C	0.175158517994	1.765286090612	0.675162465253
21323	C	1.309607868869	1.543357446832	-0.034045016631
21324	C	2.030706825191	0.287175031868	-0.119929839935
21325	C	1.652342742380	-0.874538947211	0.482453551183
21326	C	-0.257747900869	-1.374693542113	-0.826044137067
21327	C	-1.244801352855	-0.618497866653	-0.230685516339
21328	C	-1.597014183516	0.702042552745	-0.743037879118
21329	O	-2.580981456733	1.377251440287	-0.330186768554
21330	H	1.664930154490	2.333671019271	-0.690794614442
21331	H	-0.322881783776	2.723455179365	0.639132321404
21332	H	2.844389323384	0.253587789217	-0.841177690105
21333	H	2.199729195855	-1.788632855011	0.311160237615
21334	H	0.974699777233	-0.904790044323	1.323626077226

```
H      0.182629621103   -1.064348452457   -1.758055700219
H     -0.119593603074   -2.410145571946   -0.554535404132
H     -0.186797666909    1.089459289716    1.436795633737
H     -1.089146007908    1.036589004059   -1.657415685468
H     -2.703080452713   -0.557686601719    1.236774111284
N     -1.862671445593   -1.039283242396    0.944262458134
H     -1.900391351194   -2.035823258833    1.099949766254

Ambimodal TS Water45-38
155
ANGSTROM
O     -1.926733263967   -4.007621430669    3.452318749056
H     -2.152563318813   -4.111275989064    2.505459404524
H     -2.747261567709   -4.100118815636    3.935994583418
O     -3.237239767941   -0.534212717293   -2.722176530656
H     -2.834568912068    0.046155773624   -3.377838827300
H     -4.053242731819   -0.080031942452   -2.437379549623
O      0.152601675919   -2.647869658866   -4.866780103796
H      0.230088870147   -2.955517375138   -5.769394995405
H      1.024202227645   -2.867234483893   -4.423120999346
O      3.113994290687    3.019821486673   -2.201110119414
H      2.829623030873    2.299682376983   -2.794248666274
H      3.797575857289    2.645352199368   -1.630037263485
O      1.191587160405    4.960639109733   -0.625387475263
H      0.293293033631    5.051347506565   -0.275449230145
H      1.097684837402    4.628534864339   -1.528490036477
O     -3.941767190313    3.177425663356    3.121637524121
H     -3.018159100699    3.176121132149    3.436522500945
H     -4.159446673020    2.250007575873    2.934533314503
O     -0.554179754112    1.904807059726    6.018198075521
H      0.346913222587    1.569973776133    5.854050866956
H     -1.137277968973    1.152671671903    5.879945828726
O     -0.281427821594   -1.830104811000    3.382024559718
H     -0.881542135547   -2.560525957238    3.613292767671
H     -0.823467802606   -1.017713386844    3.453042527329
O      0.618474333659   -3.771009558206    1.620534296903
H      0.197935595384   -4.499987257181    2.079440941072
H      0.386933493637   -2.974571740201    2.128548162034
O     -1.267393989133    3.038022863262    3.699008558094
H     -0.991160452218    2.857872747880    4.621646765872
H     -0.730706136083    3.777452872077    3.374537749667
O      4.082163648464   -1.055957570979   -2.899509787996
H      4.875305180305   -1.274925733360   -3.390002476163
H      4.283158345497   -1.278418420617   -1.956182934066
O      3.275100911575   -3.607934236060    1.027645418384
H      2.332291711469   -3.731964945198    1.253337019255
H      3.647086650160   -3.173200270743    1.812489891848
O      4.452715176274    0.155160447381    1.666816010649
H      4.276481205896   -0.635322814046    2.219323567945
H      5.175340078735    0.649838966002    2.141117583202
O      0.530862141874    4.713926077944   -3.399711572227
H      1.417763567000    5.133858037929   -3.451931367583
H      0.549839107235    3.962917280758   -4.015355623071
O     -1.808139862971    1.520390458841   -4.473335962250
H     -2.214444805970    2.127642839952   -3.819844223363
H     -1.099526341790    2.025497051799   -4.894174173564
```

21391	O	3.051376608574	5.528816383007	-3.107682549156
21392	H	3.038076956048	6.047575833528	-2.300339722018
21393	H	3.222625481368	4.607306132807	-2.812606397194
21394	O	0.740738941332	2.644047578842	-5.212540143980
21395	H	1.367624981408	2.051497322017	-4.756422962758
21396	H	1.170845747449	2.920011604472	-6.022580636176
21397	O	-1.883268764072	0.338413420785	3.703000502788
21398	H	-2.712878678955	0.317551784378	3.204167655310
21399	H	-1.546181536438	1.240814537178	3.586859917543
21400	O	-3.991223322093	-2.666227338069	-0.994672300462
21401	H	-4.037682344452	-3.300203739356	-1.714147413208
21402	H	-3.604660404151	-1.870462474370	-1.387965975801
21403	O	4.987867078470	1.759327060065	-0.530348066233
21404	H	5.809064336470	1.368736223005	-0.831813764000
21405	H	4.645147518415	1.171063855580	0.163149473964
21406	O	2.031209310591	-1.720539003744	4.763050594017
21407	H	1.186837850348	-1.830083540838	4.280044197123
21408	H	2.014404866285	-0.802449473812	5.084817166468
21409	O	-5.672858531253	-0.889539139131	0.471994128360
21410	H	-5.207830575345	-1.678839459402	0.166876148891
21411	H	-5.724453814189	-0.312531549831	-0.310667484838
21412	O	-1.957861679934	-3.087471353326	-3.176039108485
21413	H	-1.230466286072	-2.993537645213	-3.817068214534
21414	H	-2.278563371911	-2.188046885326	-3.022334978474
21415	O	2.437896162277	-3.140957241450	-3.744779454924
21416	H	2.922726418468	-2.354329832325	-3.443675930025
21417	H	2.308895425009	-3.692580407073	-2.944921661369
21418	O	-4.052509557336	0.677878061190	1.923283421598
21419	H	-3.603684502102	0.996627238344	1.113841489596
21420	H	-4.796948346683	0.135576459567	1.591210522465
21421	O	-2.482972277072	-4.150525737470	0.815292324085
21422	H	-3.120740518847	-3.636940282735	0.294385400857
21423	H	-1.771733662266	-4.381598864833	0.191370708902
21424	O	5.145532113943	3.767882684406	1.431997747227
21425	H	5.123018030477	3.273185588081	0.599596586565
21426	H	4.232194173231	4.055810587373	1.582644697520
21427	O	2.650008351963	1.845385557707	2.835553200926
21428	H	3.174840783320	1.230324863141	2.288210438637
21429	H	2.490266218504	2.651319767993	2.302867692635
21430	O	-3.559173941075	3.748423275637	0.590721176594
21431	H	-3.181114864643	2.893808778840	0.334053330133
21432	H	-3.793653090598	3.656398727781	1.550169265540
21433	O	-2.867385502093	3.047402873951	-2.517858331541
21434	H	-2.739136748201	2.501127621007	-1.720358459703
21435	H	-2.455104038108	3.914976409487	-2.352306201422
21436	O	-5.575753903604	0.633981373972	-1.829439551195
21437	H	-6.353080152739	0.548236920432	-2.382247387309
21438	H	-5.505743613777	1.603572436427	-1.611203493842
21439	O	4.062040692867	-2.035318800953	3.180370408501
21440	H	3.257547510146	-1.932529634933	3.770221716877
21441	H	4.786899946047	-2.266880651229	3.761448521754
21442	O	2.011710993565	-4.493535646902	-1.462780534696
21443	H	2.519964862018	-4.283772916827	-0.671831406817
21444	H	1.075059659228	-4.470809645306	-1.213180559210
21445	O	0.019014768810	-0.246261287203	-3.568146173540
21446	H	-0.673395865064	0.341239096868	-3.925293976589

```
H      0.028981042710   -1.042537959986   -4.123044685186
O      2.397649940326    4.234879447359    1.652667163524
H      1.696931954058    4.646183405811    2.180404987472
H      2.116517326195    4.396718741745    0.728850616327
O     -1.750479710809    5.554343451600   -2.375286772419
H     -2.146257307997    6.174441135801   -2.987474983504
H     -0.880524885495    5.295442511294   -2.759042103883
O      2.356459758863    0.938891192395   -3.788402203059
H      3.039127193020    0.284824936270   -3.572779793726
H      1.504252760942    0.475402435032   -3.598821397899
O      4.776549019944   -1.712252328857   -0.428262641185
H      4.693376979785   -1.040434499705    0.267490944264
H      4.330659819791   -2.499798674033   -0.076168731506
O     -1.426468407006    5.314457599379    0.349763595470
H     -2.226261284635    4.761834359335    0.519110679358
H     -1.586412144841    5.683131518343   -0.530971572515
O      0.007058009624    5.287138702396    2.660117301240
H     -0.492918552312    5.337837514018    1.821235379966
H     -0.199725733577    6.087930789245    3.143125814157
O      1.920127648703    0.974646259352    5.285148113530
H      2.705645406344    1.369621174074    5.679415641598
H      1.961831975800    1.226227238429    4.342503014366
O      6.127714196593    1.724249945143    2.935144977086
H      5.702836948383    1.889127410826    3.792963075042
H      5.948208211435    2.533885196819    2.413747211395
O     -5.365078480001    3.225521569901   -1.447637597880
H     -4.994630263070    3.504474918957   -0.592328018492
H     -4.620514876880    3.319673491426   -2.067773764649
O      4.292993903499    2.598047159655    4.817981355137
H      4.402735328509    3.549392667514    4.827252171972
H      3.697322864840    2.397189568551    4.058289397059
O     -0.812424582710   -4.641713717658   -1.314517426374
H     -1.215693143729   -4.023660359837   -1.968920188053
H     -0.869292656371   -5.516348745670   -1.701260389385
C      0.019782208972    1.754183293053    0.856869424537
C      1.087034337819    1.619235182706    0.034273752137
C      1.862686969246    0.400753524029   -0.147275237560
C      1.639465880495   -0.767493344067    0.507939227476
C     -0.307223596753   -1.487848837716   -0.695478928443
C     -1.311171864317   -0.740951001253   -0.119173973738
C     -1.639179707567    0.577852352913   -0.660581210409
O     -2.618101983883    1.306784141023   -0.347157591672
H      1.352971788474    2.441796829253   -0.626993676512
H     -0.536995136850    2.679874867639    0.916913168913
H      2.572593232853    0.413514670855   -0.971728997136
H      2.212298925021   -1.652985259325    0.285074010759
H      1.052237283561   -0.822157204004    1.413812826702
H      0.130505022413   -1.196796901483   -1.633573172394
H     -0.116854688276   -2.498685599150   -0.368112052045
H     -0.239129267415    1.024280208370    1.610566603868
H     -1.053305188040    0.868208022787   -1.547197237441
H     -2.793935750052   -0.737327402328    1.342173614726
N     -1.950855848708   -1.185143104580    1.026713559604
H     -1.857817268161   -2.157112218195    1.271066951711

Ambimodal TS Water45-39
```

```
155
ANGSTROM
O     -0.787204144069   -2.881001424178   -3.902033539757
H     -0.832778423694   -3.459338544676   -3.127041469751
H      0.156874723769   -2.874541027949   -4.135518718015
O     -1.592278011304    2.742982974619    5.736945560929
H     -0.865025981425    2.260476535474    6.184455357395
H     -1.166636461071    3.259477823810    5.038313288890
O      2.358853478852   -5.140051575691   -2.608336345394
H      2.766736627354   -4.752326186674   -1.809326191352
H      2.813556106161   -5.964131954142   -2.783944356633
O      0.913906549152    5.427577107579    1.244094642440
H      1.231282548623    5.644523384472    0.330954408455
H      1.460327049110    4.682405011303    1.521051339230
O      2.010107091329    3.214882239880    2.902006447754
H      2.456193559561    3.768549448335    3.546366054207
H      1.041793438813    3.354370716725    3.042961168926
O     -3.026101651626   -4.439598331298   -1.187154442667
H     -2.189939064769   -4.630725664434   -1.633278471987
H     -3.296433847395   -3.547582554402   -1.449917387796
O      1.239280777560    2.745673422128   -3.721668152733
H      0.752735870432    3.164899922567   -2.988485662170
H      0.582136932303    2.710366978015   -4.449787558303
O     -4.458042693204   -0.099392195321    1.846074841984
H     -4.683572434643   -1.049168190469    1.731679620075
H     -4.432450890009    0.083187512658    2.787691214281
O      3.299368158989   -0.957610644246   -3.132224567516
H      2.395141659602   -0.561968467117   -3.079896440540
H      3.859886406867   -0.244197423006   -3.452938022401
O     -4.779232428999   -2.650535066277    1.110248898013
H     -4.629668946695   -2.543397983202    0.154556824418
H     -4.125044243759   -3.292402829934    1.400315113323
O     -3.947195672836    0.964937489962   -3.257720645504
H     -3.121934472408    0.451707032003   -3.391832133720
H     -4.154497356674    0.947565754455   -2.311201106642
O     -0.539369231349    3.827552650875   -1.999552591915
H     -0.921612867829    4.006877108670   -1.122252458160
H     -1.310099346548    3.730316660643   -2.590943382846
O      5.548309374756    0.837023521105   -1.258989142634
H      5.373304276850    1.334430798490   -0.452540325916
H      5.006603304806    1.236985462100   -1.949589487049
O     -1.703384959447   -0.340002557102   -3.920516258134
H     -1.629407421203   -1.312604818867   -3.940898777908
H     -0.841101716191   -0.067215070316   -3.557309977518
O      0.520168218922    1.301684187845    6.624783375259
H      0.126096243793    0.427974953254    6.755321274804
H      1.070832831150    1.208835930043    5.829899828634
O     -2.712238269396    2.838313101013    1.946657599630
H     -2.689404708160    2.122508734995    1.283446583161
H     -2.710220829718    3.643278683395    1.407329767284
O      4.184229948376    2.187496683841    1.122247885861
H      3.485009951323    2.501734884348    1.704739454270
H      4.355235774396    1.260110689939    1.390877596716
O     -0.570418987265    3.773949865347    3.314928893154
H     -1.298485950181    3.328043750795    2.823544623089
H     -0.565776242392    4.684514389329    3.007314319186
```

21559	O	3.520707932897	-4.115135082404	-0.333377075638
21560	H	4.121885831541	-4.603129971452	0.231277258737
21561	H	3.956490991119	-3.258354942577	-0.518410901960
21562	O	-3.934706811651	3.601763491382	-0.929507583078
21563	H	-3.365567794177	2.832262977757	-0.719325924031
21564	H	-4.829193561547	3.259350036903	-0.897325664237
21565	O	-2.866867846884	3.458406894385	-3.414801122911
21566	H	-3.285856408892	2.578838272129	-3.506662354859
21567	H	-3.309447774553	3.823994523587	-2.626665856765
21568	O	-1.518247091141	4.805985825040	0.409701083272
21569	H	-1.964030139200	5.571995001369	0.047596842507
21570	H	-0.638895064721	5.106306308298	0.748838543611
21571	O	-0.971357860223	2.286256156273	-5.168155640525
21572	H	-1.626891894424	2.880121222072	-4.778699072184
21573	H	-1.305392603088	1.392823028827	-5.020223198575
21574	O	0.808839591792	-4.335276773721	0.660818627664
21575	H	0.866916451097	-4.073028307494	1.607537019973
21576	H	1.716561349784	-4.279967307985	0.336006104309
21577	O	-4.065030328200	-1.859083472571	-1.478367068981
21578	H	-4.194482401130	-0.956398976382	-1.144222908919
21579	H	-4.575709737004	-1.880176387038	-2.310047529194
21580	O	4.635887189627	-1.723801000537	-0.904424779115
21581	H	4.067885612545	-1.552298280443	-1.681986533905
21582	H	5.281076014864	-0.996882106299	-0.949269466500
21583	O	-3.297294095474	-1.328638123546	5.237966165517
21584	H	-2.598341485482	-1.278062180887	5.905378317443
21585	H	-3.394996734602	-0.421261902353	4.912799256458
21586	O	4.581608289051	-0.430285624933	1.712466944811
21587	H	4.542310840660	-0.935155073606	0.889664061280
21588	H	3.955632930410	-0.852862373394	2.324038261895
21589	O	0.836321894579	-0.981365123459	-5.895081574537
21590	H	1.305396828046	-1.800431501612	-5.680130799753
21591	H	-0.088251419708	-1.218904668515	-6.001609792017
21592	O	0.926854823002	0.130719909543	-3.465041859620
21593	H	0.939187324711	-0.178819925074	-4.401038360479
21594	H	1.071418102894	1.101331298032	-3.499365974531
21595	O	2.843145085055	-1.479858798918	3.596164383407
21596	H	3.373901794311	-1.708710517816	4.360233636902
21597	H	2.411107218167	-0.614869887175	3.807456404347
21598	O	1.630159378151	0.799433883011	4.120720673436
21599	H	0.729801345810	0.555936982410	3.822834535757
21600	H	1.882340964166	1.581116900670	3.598772823893
21601	O	-2.119138644092	-4.031555735590	1.255395156960
21602	H	-1.213462550215	-4.354771628982	1.203012242971
21603	H	-2.523580258701	-4.270916105562	0.385000486804
21604	O	-0.322327738801	-4.706601154600	-1.832445802397
21605	H	0.472420109274	-5.019361972753	-2.285429812724
21606	H	-0.044854065202	-4.602239282366	-0.906823272993
21607	O	-0.585634272797	-1.179369902968	5.987479086265
21608	H	-0.605858350858	-0.782509984554	5.091189395902
21609	H	-0.309718454539	-2.089240473144	5.867612748389
21610	O	0.959791099982	-3.489859153135	3.195126373335
21611	H	1.588735985278	-2.754789754758	3.284768115802
21612	H	0.077196739495	-3.147485771561	3.407469074187
21613	O	3.653949548956	3.746489726206	-1.126294469441
21614	H	3.894681362414	3.271197511538	-0.314984576699

```
H      3.802071636380    3.135584746774   -1.859156819806
O      1.595571503873    5.560361728741   -1.296937956031
H      0.887563779222    5.042135939148   -1.707838462067
H      2.389760855721    4.996508248999   -1.304311326446
O      3.836175294948    1.972681851371   -3.374578525797
H      4.354095620368    2.422318352449   -4.044164824121
H      2.908964719884    2.247399215709   -3.526823192868
O     -5.095715229195    0.625357422571   -0.690523123175
H     -4.792784148644    0.577550473803    0.235744282978
H     -6.009736722656    0.336028504914   -0.697991078914
O     -0.810650179656   -0.061282148092    3.476380188767
H     -1.095252694159   -0.927850183210    3.131677273635
H     -1.636953690116    0.441106816926    3.661154219950
O      1.970617735524   -3.032955972212   -4.397300194957
H      2.105335495466   -3.848180929787   -3.891065048285
H      2.545405462227   -2.371781456032   -3.974731916916
O     -1.642875959151   -2.616308346741    3.549303521187
H     -2.021447991832   -3.204871584768    2.883244565605
H     -2.370949609926   -2.322177687557    4.136649374518
O     -5.318751860386   -1.323401505303   -3.828550632242
H     -4.979336170132   -0.407383628035   -3.770523585591
H     -4.975411246305   -1.689412442955   -4.644213424387
O     -3.048467154773    1.232014325149    4.155372983938
H     -2.618428999420    1.780394971567    4.861958852877
H     -3.177952746898    1.843409217461    3.414817348108
C      0.181368894194    1.738867226598    0.750962143111
C      1.299126475931    1.544113624279    0.003012511798
C      2.016988939453    0.297743848380   -0.122869982551
C      1.639661547603   -0.881525637828    0.462018590161
C     -0.207462847006   -1.398007441475   -0.806470343881
C     -1.202328406237   -0.631252895644   -0.231617685561
C     -1.527516841208    0.717377470404   -0.702713365967
O     -2.512649279606    1.376679795902   -0.296968022372
H      1.630365110571    2.352650508090   -0.646733107438
H     -0.328025806898    2.690146027007    0.741518782439
H      2.809384579149    0.282002866328   -0.865744994074
H      2.193886561353   -1.790817544442    0.277669651282
H      1.003992112119   -0.916233425854    1.335762215774
H      0.249826686912   -1.105144898757   -1.735382476598
H     -0.102604832682   -2.438239493045   -0.538538225567
H     -0.142205274368    1.052110765413    1.519633656232
H     -0.996678077438    1.073933989583   -1.602357933439
H     -2.706359372192   -0.546891326366    1.162779027368
N     -1.872290285067   -1.060272638296    0.903929581738
H     -1.913984357342   -2.055925148642    1.074551022072

Ambimodal TS Water45-40
155
ANGSTROM
O      5.046179109747   -1.008570086616   -0.809120278831
H      5.305019727067   -0.071334209165   -0.779194220167
H      4.876007397143   -1.254154428967    0.106956495261
O     -6.411308034080   -1.401761942058   -0.108609100638
H     -6.927053848366   -1.199085891929    0.673463622729
H     -6.015459596810   -0.561228929637   -0.407157022422
O     -2.879329367087   -0.319004704612    5.403606811415
```

21671	H	-2.529794841340	-1.222676810950	5.310103236188
21672	H	-2.097717423838	0.246758491659	5.485320169144
21673	O	-4.180389544121	-2.860136495677	0.282965022340
21674	H	-5.052106645258	-2.432705813438	0.163320898237
21675	H	-4.016062493462	-2.880015243706	1.243798845248
21676	O	3.176035472742	-1.133502348209	-2.881305358000
21677	H	2.759295343231	-1.979619137740	-2.666065853988
21678	H	3.936896641772	-1.079074168977	-2.276450967304
21679	O	3.565648046910	5.050713565822	-1.368407977550
21680	H	3.632982777567	4.228797645191	-1.876214333817
21681	H	3.420920778112	4.796772218661	-0.440066481992
21682	O	-0.185135154723	3.882399066504	3.277103953822
21683	H	0.029476420350	4.555690906966	2.612398972611
21684	H	-1.006197396329	3.475558135758	2.946675276991
21685	O	-1.701150835816	-2.718554520319	4.540346638318
21686	H	-1.250954597089	-2.019287179113	4.023513974392
21687	H	-2.461305056473	-2.998703979278	4.002139530741
21688	O	0.293853894385	-3.989152742355	-3.751931096313
21689	H	1.157101866982	-3.889414741431	-3.319048394284
21690	H	-0.069637908614	-3.086790770920	-3.818311725952
21691	O	1.067033507430	-4.393297196077	0.271505418383
21692	H	0.296788422259	-4.105739865827	0.800730340339
21693	H	1.827782560370	-4.249243310664	0.872082913250
21694	O	-4.188766674362	1.625901806216	3.892343510024
21695	H	-3.749447336618	1.043302860043	4.532352756169
21696	H	-3.517555206429	2.230410921871	3.541057587097
21697	O	4.203046700524	-1.085144292776	2.125498699275
21698	H	4.156131464477	-1.153393651021	3.090067878636
21699	H	3.970051992734	-0.155289430549	1.950663788498
21700	O	5.581989875667	3.418142469784	1.653172559921
21701	H	4.979054325971	4.147805143285	1.470729363416
21702	H	5.822080521961	3.043725743430	0.792812469693
21703	O	-0.912006082212	-5.144556689586	-1.688995590111
21704	H	-0.467201026756	-4.790547017368	-2.498005945222
21705	H	-0.229029161154	-5.105069288409	-1.006241014777
21706	O	-0.378491897327	-0.758842991488	3.233055023436
21707	H	-0.503282396890	0.037568074271	3.791242699477
21708	H	-0.968924844871	-0.699846180977	2.447006752637
21709	O	-2.522163901006	2.961360684490	2.162181354396
21710	H	-2.832906668240	3.778615798854	1.762995860742
21711	H	-2.539602584665	2.305322502468	1.439466058081
21712	O	-1.953731105841	4.161913582640	-0.534194263269
21713	H	-2.279988400112	3.246755962365	-0.496457147156
21714	H	-1.366087249397	4.179123012895	-1.301402841401
21715	O	1.271905259853	-2.695538924751	4.114835792538
21716	H	0.787177586340	-1.964032904216	3.681564383618
21717	H	0.635128503233	-3.092920605830	4.715808242019
21718	O	-4.179934575726	0.076046749855	-3.293850271874
21719	H	-4.596196033458	-0.267066167447	-4.086053566517
21720	H	-3.803327163157	-0.706164463669	-2.822775791621
21721	O	-2.319682636643	-3.755288950125	-5.282120868350
21722	H	-2.796353533425	-4.163735536769	-4.543127613091
21723	H	-1.485682519887	-4.230210314834	-5.350268995424
21724	O	3.679629463885	2.623785128152	-2.769781610425
21725	H	4.178695922702	2.641115203647	-3.586689501009
21726	H	2.874458256062	2.078394012316	-2.947414484978

21727	O	2.939317978357	-3.597092380824	2.030859055035
21728	H	2.373827059932	-3.427635856195	2.797760388642
21729	H	3.454338335426	-2.782811749127	1.911199558305
21730	O	2.820558591240	4.274942662023	1.176991638900
21731	H	2.769657055958	3.316386565798	1.292883142525
21732	H	1.906792228562	4.603416633180	1.130469414808
21733	O	5.071236310299	1.754666834547	-0.572451616803
21734	H	4.635005589568	2.081320247010	-1.380445027803
21735	H	4.369684909069	1.619697498007	0.076170311952
21736	O	-3.326667365129	-4.669880744900	-2.840023295279
21737	H	-3.973161833254	-5.292527915232	-2.504578747561
21738	H	-2.471426265373	-4.899670333975	-2.426298771193
21739	O	2.344865779039	-3.652637959512	-1.975796101475
21740	H	1.844343947542	-3.881900436066	-1.162563092225
21741	H	3.200239289369	-4.076234619033	-1.891319633444
21742	O	1.897897802691	0.966401713837	5.742889849260
21743	H	2.432866491824	0.214924343982	5.438986755815
21744	H	2.194142484477	1.725678899698	5.216756022255
21745	O	-4.486299889167	-0.015260905955	1.872356821785
21746	H	-4.383977284492	0.683931694074	2.565909599127
21747	H	-4.914025010022	0.406001973502	1.121157823111
21748	O	-3.174520960562	-2.055170861563	-2.061882763033
21749	H	-3.443549797905	-2.180973601167	-1.130299321163
21750	H	-3.344912888737	-2.925023993101	-2.466673684407
21751	O	1.580583289391	1.060667509847	-3.176369902514
21752	H	1.007936652855	0.950093680486	-3.968267768228
21753	H	2.103544585276	0.234615229938	-3.115932722121
21754	O	-0.151377093612	3.334240902722	-2.628184627727
21755	H	-0.906408734257	3.009197455597	-3.136602337737
21756	H	0.527675678678	2.651662172774	-2.716326485129
21757	O	-2.434450754449	2.126195665030	-3.862893163606
21758	H	-3.023018012608	1.396465302669	-3.587495875992
21759	H	-2.994898201761	2.796717230522	-4.255912795366
21760	O	-0.607940881283	1.289629818725	4.929501927450
21761	H	0.264916319432	1.126780988583	5.362231789940
21762	H	-0.581794260391	2.203728867559	4.631237367804
21763	O	-3.835141612639	-3.402956144021	2.956538476653
21764	H	-4.447794354195	-2.752046352470	3.397694393825
21765	H	-4.229842235314	-4.270931946022	3.045172817488
21766	O	-1.208101608406	-1.688707966702	-3.979657015866
21767	H	-1.685627578835	-2.260255102653	-4.616677872136
21768	H	-1.756299874926	-1.678184117778	-3.180787129487
21769	O	-2.865160962209	-5.263526423095	0.363480728894
21770	H	-3.439623640848	-4.506054570908	0.157108333672
21771	H	-2.249393103442	-5.361301793944	-0.375620940058
21772	O	3.625139592565	1.611610424525	1.969646091277
21773	H	3.090251134511	1.862471349702	2.742351766355
21774	H	4.450259854432	2.139695882158	2.050744528657
21775	O	0.985814175155	5.816559407703	-1.626167869644
21776	H	1.958625660658	5.704426094344	-1.633752929290
21777	H	0.630869267693	5.061800427635	-2.113200889711
21778	O	0.194371464107	5.207030082932	0.850599326695
21779	H	0.549156373527	5.645742623914	0.043413049322
21780	H	-0.668739698760	4.889333790129	0.529914689981
21781	O	-0.304131632235	0.689469367227	-5.024339348684
21782	H	-1.033469417297	1.264981475352	-4.753791804506

```
H     -0.570653867875   -0.206931879822   -4.756286405696
O     -1.133113230498   -3.735960956491    1.716935591511
H     -1.126375043851   -3.843595113820    2.671570167091
H     -1.801627136791   -4.364880958492    1.361649619523
O      2.262057078736    2.975837392129    3.889955873957
H      1.348090682991    3.259887930665    3.669171079482
H      2.793073281502    3.775175775588    3.887267623377
O      3.406678308069   -1.225418525706    4.849736176213
H      3.911625475822   -1.662625226131    5.535981694455
H      2.654615257961   -1.814819097772    4.627013957800
O     -5.276771117941   -1.509200394055    4.002982749483
H     -5.160480994330   -0.942986658889    3.216264738011
H     -4.763696373058   -1.082507031982    4.699479217155
O     -5.106855866016    0.929051475229   -0.816388625942
H     -5.016640060844    0.749749235570   -1.763746169134
H     -4.217614152602    1.221150988068   -0.547091701133
C      0.265307193102    1.759962195213    0.784943928844
C      1.342970164790    1.528669163999   -0.006399892203
C      2.012264841445    0.257401538587   -0.171987706900
C      1.646471603537   -0.920714519769    0.411007911663
C     -0.308665857283   -1.304629956174   -0.821633388211
C     -1.259796380126   -0.545727487383   -0.174355838594
C     -1.577380729509    0.804380043246   -0.606720104950
O     -2.524816648474    1.501623464968   -0.143305489374
H      1.674544806604    2.324749720505   -0.672853894905
H     -0.202303382627    2.731877425394    0.806751063154
H      2.767502702682    0.227549676496   -0.949880246598
H      2.172076874163   -1.831022861289    0.162231903061
H      1.045585560331   -0.975898331940    1.307437799579
H      0.124610955777   -0.967940981340   -1.749762962592
H     -0.197053952946   -2.355685291294   -0.602280805749
H     -0.038329661602    1.083112592998    1.568945011168
H     -1.092746523702    1.148952884140   -1.533090446348
H     -2.792381323393   -0.603463744560    1.223698749067
N     -1.879787491900   -1.000901086248    1.006742916698
H     -1.834297035028   -2.004773589254    1.155747666969

Ambimodal TS Water45-41
155
ANGSTROM
O      1.405529160426    0.510916932244    4.865065016147
H      1.398832214394    1.450745786525    4.544438146277
H      1.279942519522    0.545083537812    5.813803132200
O     -2.745965019760   -2.973800134216   -2.389058001748
H     -2.254362823102   -2.382490473887   -2.991363642652
H     -3.431919758433   -2.433397727331   -1.984430751507
O      2.812025938930   -4.655930454106   -1.757791427373
H      2.161479134722   -4.891264019736   -2.434440988429
H      3.122942687610   -3.758821962961   -1.980968389038
O     -0.988905683939    5.173523540805    0.539895632707
H     -0.106444501449    5.128297982012    0.135005547661
H     -0.920523227512    4.800899991771    1.432810895117
O      0.641854714073    4.182905600409   -3.255782042133
H     -0.328159000695    4.151089197573   -3.308339350387
H      0.949927921542    3.295531967271   -3.497091574054
O      3.311661750286   -2.818146690506    2.830467148998
```

21839	H	2.329523706046	-2.756162540457	2.850601951260
21840	H	3.479773471639	-3.770713345660	2.800230789884
21841	O	1.397133807436	5.176725063239	-0.931405410698
21842	H	1.112781397014	4.779954863592	-1.796311600848
21843	H	1.500280775026	6.114569808127	-1.099702284373
21844	O	-2.254158604980	-4.025289953788	1.164808803237
21845	H	-3.186507540634	-3.855582079251	0.954925270820
21846	H	-1.872328592729	-4.466838922591	0.395802145008
21847	O	-4.277531435654	3.241834259524	1.732640288158
21848	H	-3.552353625870	2.686596308233	2.054057661117
21849	H	-3.934258534405	3.716804129588	0.964868472570
21850	O	-1.116571597435	3.870139589334	3.038571752304
21851	H	-1.582939377650	3.007153048152	3.011559703677
21852	H	-1.610204140289	4.424218782687	3.645210070854
21853	O	3.112257031039	-0.303539029909	-5.160613364464
21854	H	3.842761128538	-0.267384652211	-4.511508645723
21855	H	3.510316549897	-0.480700020521	-6.012904375548
21856	O	-2.389190878334	1.487266124727	2.992225291798
21857	H	-1.678855538305	0.816889753330	2.889120713701
21858	H	-3.028542898560	1.079212647393	3.581976829016
21859	O	-1.666306535743	-3.950417369516	3.862625378494
21860	H	-2.006867874342	-4.130748419089	2.972314405301
21861	H	-0.734208813623	-3.721918970023	3.715165139483
21862	O	4.516467460236	0.419200566567	1.829401331856
21863	H	4.399127286327	0.094044586210	2.760692741059
21864	H	4.485844006099	-0.371193191326	1.277240788626
21865	O	1.537468944307	1.551985955409	-3.710153021233
21866	H	1.901582346461	0.967690274443	-4.389415509742
21867	H	2.300694123626	1.774556626078	-3.145515748438
21868	O	4.651706383394	-2.210005862951	0.545078885593
21869	H	4.148310164095	-2.344320271854	1.376832033230
21870	H	4.964092675625	-3.121994656394	0.343831541736
21871	O	3.534646303232	-2.052785143738	-1.945599299446
21872	H	3.905982610418	-2.047165283387	-1.041345268365
21873	H	4.082067854534	-1.425567461264	-2.453998456827
21874	O	6.472703912154	-0.536738387813	-1.078916983499
21875	H	6.517052047275	0.340182573324	-0.656737099914
21876	H	6.049093025816	-1.127396784242	-0.443175820478
21877	O	-1.705369453181	-4.256428430390	-4.817352315445
21878	H	-2.357891314791	-4.342204312474	-4.114183099171
21879	H	-1.606409710677	-3.301950282636	-4.944304193260
21880	O	-4.820624555125	-3.205279707245	0.545987098617
21881	H	-5.557697058518	-3.777260885684	0.331670731316
21882	H	-4.748661639028	-2.556467068623	-0.181729631513
21883	O	4.921321282280	-0.051460216913	-3.152689746411
21884	H	5.652521611841	-0.262868472009	-2.523425222231
21885	H	4.591516513374	0.815094766089	-2.857397651478
21886	O	-5.099434460690	0.974138285614	0.525099167597
21887	H	-5.203893747718	1.773724957235	1.073136851458
21888	H	-4.147519407322	1.008316119722	0.281092987631
21889	O	3.764140017834	3.888538801795	-0.346258578450
21890	H	2.971853521893	4.427031632055	-0.528657422995
21891	H	3.591656565616	3.494578671863	0.531579994686
21892	O	1.353948494551	2.972003512220	3.944628569914
21893	H	1.981368456364	2.957492332749	3.202232841851
21894	H	0.514817282276	3.309593275591	3.589954522951

21895	O	-2.596848127004	-1.475590874070	4.340237443580
21896	H	-2.364114395232	-2.430685608374	4.311178007556
21897	H	-3.329457124473	-1.366723618489	3.714064798891
21898	O	1.438455558224	-1.903001185984	-3.653342766335
21899	H	2.046607754422	-1.922490214458	-2.892264378186
21900	H	1.927890876834	-1.435351220789	-4.347596790722
21901	O	-1.242660818659	1.245206199319	-3.903251110577
21902	H	-1.559849372194	2.159384674719	-3.803278538994
21903	H	-0.277170868604	1.295042913467	-3.834280108798
21904	O	-4.473109549661	-1.027871591351	2.331812806472
21905	H	-4.655114569538	-1.862444386270	1.878076935002
21906	H	-4.862621791838	-0.326966174740	1.780006499557
21907	O	2.928820889444	-5.448670978687	2.056852539548
21908	H	2.559742200353	-6.213908278998	2.496811915327
21909	H	2.213647739964	-5.071701315559	1.499373675168
21910	O	3.412233367010	2.797090639446	2.112910810422
21911	H	3.669684002307	1.857633301317	1.965941625324
21912	H	4.086408766542	3.160056635512	2.692051801340
21913	O	-3.918495325215	0.428337290372	-3.173365994268
21914	H	-3.014545825822	0.665550447120	-3.407609159011
21915	H	-4.361431170526	1.244256006092	-2.869284728094
21916	O	3.910215440540	-0.521725278983	4.198910887089
21917	H	3.029682260413	-0.184419048071	4.437960089961
21918	H	3.772808109618	-1.436587023876	3.901219791131
21919	O	-2.091353553086	3.804570157088	-3.307581069923
21920	H	-2.414144639550	3.869990134165	-2.370378178364
21921	H	-2.774148099804	4.194998212305	-3.853633680265
21922	O	6.114624560556	1.898449348618	0.158819153709
21923	H	5.629906728064	1.451071183369	0.873023614477
21924	H	5.569237952407	2.638155988022	-0.121957881964
21925	O	-0.493290394091	-0.461912261055	3.171082528086
21926	H	-1.239684633189	-0.880000919383	3.679829529551
21927	H	0.165197719479	-0.170050749952	3.827286806022
21928	O	0.646202276872	-2.901411738528	2.743236550119
21929	H	0.207646410585	-2.026209707621	2.733230972116
21930	H	0.618690060124	-3.263974242543	1.844992899383
21931	O	3.848184343520	2.404726910452	-2.515482119616
21932	H	4.221529932868	3.021283958959	-3.147185355469
21933	H	3.822984473781	2.891380682322	-1.651729944945
21934	O	-4.734586294955	-1.211997612637	-1.261785309626
21935	H	-4.454856948564	-0.690017866337	-2.048431982470
21936	H	-5.121601506781	-0.554773313713	-0.670935774575
21937	O	-5.138613005254	2.514883586366	-1.897835356470
21938	H	-4.534993977172	3.189410935805	-1.563007021212
21939	H	-5.408890527021	1.998744746590	-1.124014336128
21940	O	-2.918829491364	3.902255410934	-0.795835605668
21941	H	-2.720730667863	2.962751220698	-0.552644744284
21942	H	-2.243709593703	4.452730327126	-0.340483281949
21943	O	4.889778275889	-4.839532839419	0.131126616918
21944	H	4.377707891185	-5.176512847671	0.881400783343
21945	H	4.328555047000	-4.985390953644	-0.642633274963
21946	O	-1.246487035155	-1.554069522508	-4.187637945043
21947	H	-0.304650652321	-1.658721297509	-3.971385390347
21948	H	-1.401928401291	-0.602789850265	-4.247048527767
21949	O	1.175269988042	-4.275401476506	0.352209777336
21950	H	0.333188251381	-4.545654866222	-0.054128223320

```
H       1.829355437599   -4.366863413072   -0.375460782428
O      -0.991543421297   -4.693106767395   -1.261934637926
H      -0.377513230040   -4.812608487283   -2.012437068037
H      -1.665651356919   -4.078435464492   -1.614425058226
O       0.626807623527   -4.550731697151   -3.471839860292
H      -0.134590460739   -4.659859558896   -4.075798777932
H       0.895966249744   -3.622555324700   -3.588402351645
C       0.208611222846    1.826642998495    0.721399221751
C       1.277245050025    1.656941468848   -0.096664956435
C       2.015103555483    0.417882822115   -0.249652371486
C       1.663545034453   -0.743768833892    0.373557033775
C      -0.240329568867   -1.309193952051   -0.897180963292
C      -1.247708501356   -0.635751514710   -0.232466747184
C      -1.592811791104    0.730935851538   -0.622219132221
O      -2.565156611565    1.378314785551   -0.160394505297
H       1.543804528238    2.463624429688   -0.776954738355
H      -0.329654951739    2.760615830379    0.758126648870
H       2.777687191928    0.402076768362   -1.025062322503
H       2.189012680399   -1.669224901967    0.201103205774
H       1.031106332868   -0.735631216565    1.247804072302
H       0.173434578667   -0.906057830589   -1.804844160931
H      -0.064570393928   -2.359000428190   -0.725025289267
H      -0.040852189689    1.124240372165    1.501065133494
H      -1.087589003627    1.129223886836   -1.514708754342
H      -2.669185585309   -0.688446067700    1.275036273399
N      -1.895322472523   -1.173285659583    0.851250423930
H      -1.834563899532   -2.162291532400    1.052276318677

Ambimodal TS Water45-42
155
ANGSTROM
O       1.754011217397    6.089861746107   -0.196639736578
H       1.253228108377    6.000938332814    0.627441144888
H       2.616995454214    5.694641526370   -0.037034805132
O       5.250132309660    1.336472679041    0.132428033340
H       6.109750371056    1.021971726371    0.418709501584
H       4.949538722459    0.698782464918   -0.543937728261
O      -1.873435705952    0.640467501443    3.690948355899
H      -2.638427483273    0.034585714441    3.746972245566
H      -2.133587256756    1.387461712648    3.115998026030
O      -4.469320462192   -0.123906541768   -2.443839859890
H      -4.196764289628   -0.857210720828   -1.880869005079
H      -5.057871181088    0.460028904869   -1.912968188496
O      -2.146281447211   -2.999607659365   -3.074357318121
H      -2.852727906771   -2.813274246573   -2.444173915864
H      -1.552415421497   -3.632057123178   -2.635091955309
O       2.976788280293    3.280958676968   -2.502687102977
H       3.679073765869    2.609015190244   -2.593705534434
H       2.803213642595    3.526605294543   -3.422294994195
O       4.136704540285   -1.675751505615    1.990113345052
H       3.909560973160   -0.736451651628    2.029663204305
H       3.428676943599   -2.125707630772    2.500346224789
O      -1.699447675684   -4.131298508830    0.578054055920
H      -1.746652100725   -4.677476798845   -0.213537434830
H      -0.750984700277   -4.214723432014    0.844746723964
O       2.104089698283   -2.919417410338    3.206366758413
```

22007	H	2.405121614070	-2.910840184520	4.134336143777
22008	H	1.354436224002	-2.298919447470	3.189310674344
22009	O	2.017333308537	0.126503305849	4.418070052851
22010	H	2.607486467729	0.371386516666	3.687092673363
22011	H	1.240809597028	-0.302189981023	4.020562721360
22012	O	-3.060129668757	1.544241223591	-3.872621337865
22013	H	-3.589315613223	0.924892247877	-3.301271716968
22014	H	-3.508106413613	1.571295887899	-4.719117636015
22015	O	3.060323608859	2.408778923991	-5.118833846433
22016	H	4.004496506074	2.244489352645	-5.073102072083
22017	H	2.652137519652	1.690808709308	-4.588042845665
22018	O	2.059456321067	-3.975167696473	-1.216628693861
22019	H	2.928295912244	-4.170481125351	-0.828650483685
22020	H	2.205768224988	-3.197188696962	-1.792726380604
22021	O	-3.672944331837	3.381199423161	-1.767405962930
22022	H	-3.539451464447	3.129206136253	-2.687955962047
22023	H	-3.210517269498	2.680372406931	-1.257235229698
22024	O	2.642710624399	-1.889754748327	-2.858748154331
22025	H	2.249555833228	-2.383575656536	-3.607460053512
22026	H	2.288887794127	-0.986917078132	-2.975395106390
22027	O	4.869564254802	-0.620993376702	-1.783694454987
22028	H	4.172962069244	-1.182968591862	-2.163989078019
22029	H	5.426555417102	-1.191124591533	-1.216581760457
22030	O	-5.231589989533	-1.359958415537	1.301682537201
22031	H	-5.006469053540	-0.399150570095	1.304435995579
22032	H	-6.187328392158	-1.409555873713	1.268917719736
22033	O	1.257592844831	2.792915689033	4.649926499234
22034	H	1.820429197727	3.148743387337	3.949588565322
22035	H	1.466266608354	1.845297205863	4.697649836601
22036	O	-1.069948024201	4.117025268652	4.190426051316
22037	H	-1.563061322123	3.808251260854	4.967380864573
22038	H	-0.212349166229	3.652064502765	4.289254128093
22039	O	-0.089130990383	-1.281993204634	3.273844307089
22040	H	-0.676077788106	-2.049450300998	3.355491750193
22041	H	-0.696336820679	-0.514299166866	3.381425077202
22042	O	-0.449664296033	-4.883810069838	-1.934891181957
22043	H	0.447809309258	-4.586842206975	-1.697342457889
22044	H	-0.333318957592	-5.532252917063	-2.630560994312
22045	O	-1.221017950361	-0.632319265116	-3.985898815257
22046	H	-1.896880583623	0.055598636177	-3.954271705089
22047	H	-1.637174804397	-1.463413125399	-3.692303659773
22048	O	-3.674027456846	-1.377197393848	3.628772135841
22049	H	-3.112152426875	-2.162883520480	3.637027074601
22050	H	-4.286554923374	-1.487382537027	2.886629260876
22051	O	2.918400871330	-2.129769395294	5.640556708181
22052	H	2.615667916398	-1.236412116220	5.375548610033
22053	H	3.761027761409	-2.014513137499	6.079120308342
22054	O	-3.752620198258	-2.441089458225	-0.768809244058
22055	H	-3.107049880061	-2.987328367049	-0.302541030391
22056	H	-4.358502363836	-2.123336211471	-0.076517003809
22057	O	6.210279794215	-1.929708535677	0.149685844588
22058	H	5.623319955068	-1.739760986300	0.900103377396
22059	H	6.134865602326	-2.877269011965	0.004537848379
22060	O	-1.847630772054	-3.508788158586	3.264042730542
22061	H	-1.549352934175	-4.246593718651	3.797268459302
22062	H	-1.949915919692	-3.843325642557	2.356914071810

22063	O	1.009401650321	-2.961085987173	-4.722221926176
22064	H	1.005885830533	-2.249119575394	-5.379213336554
22065	H	0.126582190105	-2.955848367595	-4.339735015453
22066	O	4.204893848200	-4.041793709334	0.497059804801
22067	H	4.048287854935	-4.721865302954	1.154046108306
22068	H	4.108972763585	-3.191744297719	0.961707613926
22069	O	5.126854532553	1.700092799883	-3.142184850230
22070	H	5.047824246406	0.797463364720	-2.782112365163
22071	H	5.958645712786	2.050882008431	-2.822416460032
22072	O	-5.806378545929	1.742944656706	-1.047683547020
22073	H	-5.269475253533	2.499124637936	-1.343718197003
22074	H	-5.559228318625	1.612592043029	-0.118921645257
22075	O	2.359904629201	3.584820956603	2.196215863683
22076	H	2.951714892659	3.828383481809	1.461741975492
22077	H	1.548464887227	4.111136991412	2.063862122058
22078	O	0.473746278432	4.334931740877	-1.804090169995
22079	H	0.814521962652	5.137141420116	-1.356589634221
22080	H	1.279130099393	3.833652582143	-2.002497010809
22081	O	-0.381883200398	2.428836520095	-3.683321144836
22082	H	-0.285409585923	3.219335421493	-3.134813069154
22083	H	-1.321401357500	2.199005802841	-3.705106507446
22084	O	-4.464934932181	1.168176221349	1.302556257931
22085	H	-3.709758570463	1.213846300569	0.665202213818
22086	H	-4.217784683872	1.796740192187	1.992592272773
22087	O	0.136624479539	5.188484886388	1.902367196322
22088	H	-0.554356823931	4.941549572707	1.263245829659
22089	H	-0.281764771831	5.140510695925	2.772242643740
22090	O	0.844429649975	-4.430453378725	1.245122396966
22091	H	1.320963211834	-3.960389107047	1.946203013439
22092	H	1.307341599595	-4.245593707084	0.411454683006
22093	O	-1.582305929215	2.151793201380	6.011552288530
22094	H	-0.641874649079	2.135028190223	6.204632554301
22095	H	-1.709900488505	1.535711769658	5.267393673981
22096	O	3.510667083329	1.083773984520	2.280512218722
22097	H	4.161878016373	1.234149074857	1.574210239356
22098	H	2.999573911229	1.913308017335	2.305591663038
22099	O	0.590937401739	-0.491386146381	-5.933227394915
22100	H	-0.217010341171	-0.555582922454	-5.384250088871
22101	H	0.555491966902	0.402260153725	-6.333789005031
22102	O	3.944322002229	3.959942980542	-0.019349447580
22103	H	3.557184379463	3.859584600318	-0.906179814369
22104	H	4.518695417036	3.191248978069	0.086711667447
22105	O	1.685987656918	0.521164107830	-3.707360498495
22106	H	0.951795022111	1.113196730999	-3.478420361341
22107	H	1.381423310034	0.070470123303	-4.530130690692
22108	O	-1.610460035282	4.243936875737	-0.001287046449
22109	H	-2.423198430542	4.361533471224	-0.509170074347
22110	H	-0.900244789871	4.169974416351	-0.666233517907
22111	O	-2.608047869717	2.889749374674	2.350626501963
22112	H	-2.116102631355	3.427615833833	3.007743905216
22113	H	-2.344811292211	3.227483448757	1.482908204918
22114	O	0.535838127081	2.176575828973	-6.326235734777
22115	H	1.466932595223	2.417611350507	-6.228916424808
22116	H	0.141746696550	2.363689595503	-5.458027249672
22117	C	0.204507735471	1.583977115875	0.886878681227
22118	C	1.277817826295	1.507413079571	0.058105082650

```
C      2.005910881682    0.302129717645   -0.258143021966
C      1.700170814141   -0.937033010216    0.236444717543
C     -0.224002690706   -1.398898556426   -0.900378533223
C     -1.180535307661   -0.624497004006   -0.269758921780
C     -1.496595706492    0.709707248121   -0.763154852137
O     -2.494511835545    1.387503904393   -0.424306555293
H      1.566632372925    2.390988073707   -0.504668843286
H     -0.319131547627    2.514636886793    1.034819825618
H      2.726672443247    0.384648964957   -1.066857982800
H      2.264561068201   -1.802546612343   -0.074974015845
H      1.137230090609   -1.064270283261    1.151020787886
H      0.154846133918   -1.101091224108   -1.864100158042
H     -0.125851503876   -2.447272272004   -0.664517925696
H     -0.064937943629    0.797515179014    1.575696385717
H     -0.917321395099    1.050682739266   -1.635796668847
H     -2.590210258672   -0.525880300177    1.241707123630
N     -1.794782201350   -1.038842875366    0.903300687698
H     -1.765531441206   -2.013971091859    1.143106147240

Ambimodal TS Water45-43
155
ANGSTROM
O      0.079783407413   -4.656858249904    0.888626465641
H      0.827501732328   -4.283915297562    1.380547635178
H      0.350958364825   -4.697702257617   -0.036118311963
O     -1.628344650169    3.952787314942    2.376136890926
H     -1.749893575412    3.117030869448    2.860221131198
H     -2.521619920577    4.252642120854    2.152156778274
O     -2.338476343209   -3.788902291374    1.688496212040
H     -2.997316928024   -4.089513547062    1.036538883440
H     -1.496951103459   -4.215359380194    1.435425697016
O     -0.712301923866    4.754642210640   -0.188445414044
H     -1.611748121279    4.670026529715   -0.568682578280
H     -0.827849516832    4.578630468484    0.757127472410
O     -1.404461206768   -0.794340843871   -3.996957947356
H     -0.733069379687   -0.177280045269   -3.639584390679
H     -2.116590785419   -0.176124117671   -4.321360563211
O      0.509684708128    0.950350510393   -3.235492178601
H      1.372165737068    0.510907778605   -3.353957382875
H      0.314267295144    1.417481509320   -4.084099366993
O     -3.121129356681   -1.955952689654   -2.168108397630
H     -2.541851849124   -1.477835913311   -2.785193880320
H     -2.707715164933   -2.832932222230   -2.094622164380
O      2.202255917596    3.545132868427   -5.794045430585
H      2.478124281011    4.233917659850   -6.397883722979
H      2.385815873880    3.885848239433   -4.877150417734
O      3.146038577660    1.331241804863    3.378975799001
H      3.186585021729    2.071087307798    2.758937158865
H      2.413685537344    1.534313890005    3.975351649636
O      0.786074834581    1.631456556251    4.870316140898
H      0.372345512167    0.969948399020    4.289331436452
H      0.136734839777    1.781299319799    5.576658263433
O     -0.165869035214   -0.535688217601    3.383791808049
H     -0.790818730698   -1.279705153869    3.538468150609
H      0.663705982054   -0.855663132269    3.781870771709
O     -1.807626049177   -4.389244411329   -2.336687235460
```

22175	H	-0.899114049667	-4.507405519975	-2.029880624042
22176	H	-1.751433183994	-4.246693139391	-3.296324166828
22177	O	-2.156874566246	1.437677555756	3.516915522591
22178	H	-1.502411632919	0.785090444095	3.217897752275
22179	H	-2.908430834042	1.425926905480	2.881765293353
22180	O	4.484876551363	-1.880620749941	-1.723077151304
22181	H	4.732741729131	-1.353091639168	-0.933044628107
22182	H	3.740517152769	-1.434177088165	-2.141030594370
22183	O	-3.269653340653	0.960122719493	-4.463510881267
22184	H	-2.923772556620	1.851285917517	-4.280834210919
22185	H	-3.919163815153	0.781879791215	-3.753891097930
22186	O	4.527821278242	2.714249094547	-2.322469271392
22187	H	4.780000131375	1.864082414444	-2.754559595912
22188	H	5.281068650607	3.297850403272	-2.428459799176
22189	O	5.217560694097	0.386269013563	-3.418556489488
22190	H	5.591868633739	-0.314612620114	-2.875414931483
22191	H	4.366254433098	0.039569973108	-3.736228753677
22192	O	-4.651233803550	0.359941060538	-2.188621118498
22193	H	-4.359419590409	-0.561394667563	-2.125373548616
22194	H	-4.143451607500	0.839853745374	-1.524193938857
22195	O	-3.939243401958	-4.338946273480	-0.447802271626
22196	H	-3.265638908613	-4.666159574882	-1.060336657276
22197	H	-4.217330670556	-3.487962335149	-0.798815504361
22198	O	3.660068483661	-1.169506688905	2.585966707992
22199	H	4.273561748655	-1.097535558960	1.838825135815
22200	H	3.449139519558	-0.235428836668	2.801644512244
22201	O	-4.295988639061	4.212193489684	1.495233523464
22202	H	-4.389740254389	3.248510912799	1.633283519493
22203	H	-5.058958664040	4.626439820048	1.898059946640
22204	O	2.155039033527	0.774654089251	-6.156665327620
22205	H	1.199899115241	0.800560122252	-6.281544661910
22206	H	2.415670310769	1.706859821128	-6.080289794424
22207	O	-4.654413537259	-1.051938177343	2.334869239202
22208	H	-4.803875040028	-1.976791759126	2.135594599405
22209	H	-4.337242608126	-1.019106019435	3.267668382434
22210	O	1.006996731393	-4.312581387963	-1.793164729190
22211	H	1.890037397214	-4.292249515619	-1.386995556046
22212	H	1.032532584815	-3.664296397298	-2.514904841334
22213	O	2.676271091408	-0.544086168638	-3.916469952869
22214	H	2.581662514637	-0.121948242866	-4.804683996384
22215	H	2.200705978288	-1.388824953156	-3.985605014040
22216	O	0.997223195155	4.049028514667	3.499923298435
22217	H	0.923245851874	3.256413139380	4.058679448735
22218	H	0.117047118752	4.206155034622	3.138034448742
22219	O	4.839171852402	2.080070409380	0.347325579577
22220	H	4.239983065462	2.576709976324	0.927731698336
22221	H	4.618266606671	2.356225640071	-0.557977918797
22222	O	-3.139565819905	3.960728039317	-1.066086303043
22223	H	-3.689551423238	4.231026426551	-0.317689980783
22224	H	-2.943342244066	3.017898673782	-0.902123101271
22225	O	-3.468802211617	-0.796587989744	4.729211031596
22226	H	-2.840616936265	-1.537370433795	4.663524369439
22227	H	-2.971707389582	-0.000916699771	4.488927436326
22228	O	-1.623353076076	-2.673845766720	4.040963896017
22229	H	-1.986677606561	-3.141963797782	3.269243634807
22230	H	-0.828746650783	-3.185078517634	4.304212196142

22231	O	2.115922734768	5.103298242714	-0.472826888889
22232	H	1.162972490548	5.216621226864	-0.361369035294
22233	H	2.314885078593	5.252778671518	-1.402915203319
22234	O	5.271279986146	-0.484328747263	0.439364243281
22235	H	5.041033579705	0.486926654915	0.432360861819
22236	H	6.196903669840	-0.524797207615	0.682487889206
22237	O	0.815560209409	-3.736246854472	4.467734714476
22238	H	1.291438768844	-3.919873236177	3.647623329348
22239	H	1.265598771023	-2.976530802212	4.859765754796
22240	O	-1.768468979621	-3.388233295706	-4.944519433504
22241	H	-2.580655395169	-3.569384325483	-5.418360044201
22242	H	-1.787184997670	-2.441246747363	-4.723247038118
22243	O	-0.186459383829	2.215692765872	-5.471872480441
22244	H	-0.992324788867	2.686064291315	-5.230404274349
22245	H	0.505290348842	2.880626230717	-5.631413440683
22246	O	0.830217468647	-2.588019180333	-4.005038192089
22247	H	0.593779362762	-3.229649394813	-4.680172259715
22248	H	0.053395658623	-2.010758879804	-3.930235632480
22249	O	2.068991143644	-1.256467025099	4.851118706066
22250	H	2.217158702413	-0.422737506225	5.305259682774
22251	H	2.787774359254	-1.352617051419	4.209219384279
22252	O	-2.204583993250	3.426044254542	-3.745496340285
22253	H	-2.835440655713	3.765483767910	-3.100360537890
22254	H	-1.335432713562	3.534457966077	-3.309318571552
22255	O	2.396258136030	-3.591747683901	2.044958691401
22256	H	2.870812503820	-3.862793500175	1.247566255411
22257	H	2.768045548192	-2.723454482492	2.281140346486
22258	O	-4.155952378615	1.525596098104	1.752868087127
22259	H	-4.628205583346	0.689066331261	1.896430865402
22260	H	-3.623260474342	1.411127259623	0.933761161488
22261	O	-1.596235469321	1.728242756422	6.227011480007
22262	H	-1.951973415628	1.805077571902	5.328229451813
22263	H	-2.067597508815	1.005562649136	6.645492963891
22264	O	2.550221908816	4.349225295573	-3.318909449638
22265	H	1.661749750616	4.084811210005	-2.995103076499
22266	H	3.187516925803	3.729696449569	-2.920105145113
22267	O	2.966381398559	3.588428253060	1.697792864027
22268	H	2.309900697983	3.853686139022	2.374559197143
22269	H	2.769574808757	4.159280782536	0.938856359574
22270	O	0.169579185322	3.520176554287	-2.428056686186
22271	H	-0.066080681615	3.853340280454	-1.540238895933
22272	H	0.308841461685	2.558794438424	-2.381020020780
22273	O	3.496494049200	-4.081346719556	-0.574376078615
22274	H	3.917092075153	-3.304587585201	-1.015184945446
22275	H	4.098693073658	-4.817860011214	-0.681951725513
22276	C	0.238418080580	1.599509935683	0.887534737161
22277	C	1.299496324612	1.510511195761	0.054326663603
22278	C	2.013905332843	0.279806958181	-0.258392492872
22279	C	1.753276082335	-0.928151490339	0.301482634189
22280	C	-0.375532208491	-1.473222824018	-0.737290005577
22281	C	-1.323803517387	-0.676747643095	-0.133834226473
22282	C	-1.619218447647	0.639118883837	-0.694596288926
22283	O	-2.608132306890	1.358011618284	-0.402282611234
22284	H	1.620302695263	2.391635386421	-0.499271500474
22285	H	-0.274595111501	2.533366395784	1.053709865917
22286	H	2.716825130942	0.352438955168	-1.088536796623

```
H      2.269029735426   -1.823679955759   -0.012663762452
H      1.184838616764   -1.028295837336    1.213793916084
H      0.069612887785   -1.189012852650   -1.673943060683
H     -0.245430776674   -2.499909155085   -0.433707083163
H     -0.034569750753    0.794327501121    1.552271754953
H     -1.029245949563    0.938658179947   -1.572561888089
H     -2.855417847912   -0.619814860660    1.249881876362
N     -1.972259749128   -1.053446716573    1.028780648447
H     -1.959630102522   -2.038616368756    1.281022369875

Ambimodal TS Water45-44
155
ANGSTROM
O     -4.237139593636    0.774866875068    1.940597998764
H     -4.703870926637   -0.060784697099    2.115079616500
H     -4.555296694569    1.054661768614    1.064248587674
O      4.986137851955   -0.467393455117    0.686831828945
H      4.701795786480   -1.350353822841    0.424424327895
H      5.161648093460    0.013093834223   -0.141500401781
O      4.516943848524   -1.551647620051   -2.658002811196
H      4.938275799927   -0.718472888664   -2.394693356461
H      4.378498108710   -2.058324916424   -1.845769733918
O     -1.375442551440    3.914140121498    2.891337980779
H     -1.166337549698    3.441792959223    3.721214320540
H     -2.350925387372    3.835102887972    2.815957440001
O      2.494073423491   -1.046184700245   -4.356173709367
H      3.268277417665   -1.204942182421   -3.776252225362
H      1.881644618179   -0.456789928041   -3.867791880205
O     -3.135772669298   -2.031097150557   -1.978825412312
H     -2.924198855833   -1.101798639744   -2.177422963785
H     -3.271975987048   -2.426729791406   -2.867229581917
O     -5.359236444821   -0.965795954615   -3.770944952513
H     -5.715148754851   -1.323822479296   -2.947310179115
H     -4.881862825512   -1.682810121670   -4.209730207679
O     -1.500274062452    3.955112948449   -4.116256520852
H     -0.645270085083    4.003739800148   -4.561847360957
H     -1.271884941510    3.976328555152   -3.158536975040
O     -1.272123562155   -4.717812916489   -4.148514564702
H     -1.019193098650   -5.534053745636   -4.579542084619
H     -0.464259119398   -4.172507436954   -4.081342931936
O      4.180648225185   -3.176928264544   -0.301550393300
H      4.900549311696   -3.807905666949   -0.252108252159
H      3.430999208947   -3.629977388600   -0.760574081365
O     -5.480184011715   -1.504984313727   -0.820524096252
H     -5.405897322495   -0.532321478811   -0.817155614283
H     -4.614255872007   -1.822030079900   -1.158181533748
O     -0.220070363085    0.116608294704   -6.578682649464
H      0.675656534631    0.481427324632   -6.493582049008
H     -0.828007401772    0.807667923103   -6.269579524107
O      0.658365125352    0.707418291021   -3.431709719673
H     -0.085276065252    0.152732469974   -3.735176477351
H      0.599241838076    1.541972933318   -3.937171226722
O     -3.226191012046   -2.739068140814   -4.548106225745
H     -2.493021000882   -2.092248982329   -4.647005604428
H     -2.787451851991   -3.600232482577   -4.567789435711
O     -3.337815134305   -2.221713105992    3.836490537847
```

22343	H	-3.043300004698	-1.312508495232	4.028656862279
22344	H	-3.965378554793	-2.156724997520	3.104257326489
22345	O	0.252429634816	-3.164958135347	2.169254709046
22346	H	-0.214729147904	-3.575487414465	2.924763983513
22347	H	1.219724682203	-3.282369903522	2.324570996463
22348	O	2.904656132808	-3.246453722000	2.405592585433
22349	H	3.335281919992	-3.237486480166	1.542791948370
22350	H	3.178780389851	-2.414356375592	2.839228839493
22351	O	-3.242691448992	0.527045036438	-3.036271499540
22352	H	-4.074918190683	0.114003818177	-3.355724574982
22353	H	-2.705326881702	0.687012751808	-3.831969287143
22354	O	0.180729611645	-0.751948096613	3.346306938122
22355	H	0.121297198638	-1.591960106374	2.830480361980
22356	H	0.413417881921	-1.025618162201	4.236371512946
22357	O	-5.297411289330	-1.745842187709	1.824034564213
22358	H	-5.369548998094	-1.750453562156	0.834507555109
22359	H	-6.139044740521	-2.062052386424	2.152727750674
22360	O	2.931744088613	4.364608022949	0.283911242963
22361	H	3.514200415080	3.648827266952	0.585672471607
22362	H	2.262939599059	4.424148706249	0.979803071446
22363	O	-1.255229937401	-3.866766844286	4.256869817817
22364	H	-2.025600432020	-3.270844340326	4.132509007983
22365	H	-1.615609001951	-4.724715035615	4.479851413483
22366	O	-0.980406904063	-1.248748937894	-4.434461030280
22367	H	-0.709993189713	-0.909872930705	-5.319443289576
22368	H	-0.362041575761	-1.961387440157	-4.204839126063
22369	O	3.735016170198	-0.797993738287	3.231964471544
22370	H	4.296284141440	-0.577188658365	2.477015393147
22371	H	3.231499482677	-0.003084052949	3.448095856380
22372	O	1.049600679400	3.064667238751	-4.771487503384
22373	H	1.545196770912	3.553732293666	-4.100023971145
22374	H	1.692553675963	2.586174979854	-5.314573453642
22375	O	1.310864422856	4.044199997945	2.616692284364
22376	H	1.596384242889	4.673296593751	3.280845838185
22377	H	0.335662974129	4.066154952137	2.619023545674
22378	O	-4.912569792419	1.158695902733	-0.687366537259
22379	H	-5.302624021835	1.849647204090	-1.228048983624
22380	H	-3.947303849726	1.228084819511	-0.826788371183
22381	O	1.990220070513	1.494711064475	3.478787069133
22382	H	1.320931037996	0.849922603324	3.207334825305
22383	H	1.705611960640	2.364829133983	3.138202792553
22384	O	4.100445303108	2.165107840953	1.537524625826
22385	H	3.503297521961	1.991498364182	2.276315356503
22386	H	4.447657862780	1.309671167013	1.260208145735
22387	O	2.141316726091	-4.345778990967	-1.569730989859
22388	H	2.388675612789	-5.206861313943	-1.909574501555
22389	H	1.405841388149	-4.530034736731	-0.895391946372
22390	O	0.266232734783	-4.912890903775	0.077992442523
22391	H	0.212984073692	-4.302529030016	0.841730813642
22392	H	-0.585560813059	-4.835821328521	-0.400595694334
22393	O	1.882116892658	4.156823349366	-2.214957050070
22394	H	0.941582881995	4.056478600573	-1.983230975955
22395	H	2.319136452469	4.406636988301	-1.375435684075
22396	O	-1.584369213564	5.420349098483	0.469332192791
22397	H	-2.507665390779	5.163035746519	0.300138068032
22398	H	-1.384069637896	5.069453932824	1.346912479826

22399	O	-2.118046590577	1.650779023044	-5.260585317996
22400	H	-2.873818864864	1.838834253265	-5.818121005554
22401	H	-1.877596674550	2.504673645768	-4.819853476304
22402	O	-3.782193629500	3.907807694371	-0.161703008418
22403	H	-3.209324005283	3.132245153226	-0.064644033949
22404	H	-4.031162578673	3.892342436918	-1.102632466572
22405	O	-4.029250691979	3.522642939693	2.578952331095
22406	H	-4.196133345633	3.832692052442	1.677949807842
22407	H	-4.177368641096	2.566949775905	2.561627508828
22408	O	2.415494736316	0.997399491575	-6.145095686774
22409	H	2.574079774248	0.237718592292	-5.543117880972
22410	H	3.063554174651	0.931223602803	-6.845637575139
22411	O	-0.804794505928	3.861057786219	-1.537484255150
22412	H	-0.979020011458	4.564483528452	-0.869655820966
22413	H	-1.319436089370	3.097840693828	-1.232271551543
22414	O	0.943744646225	-3.173559066456	-3.790150783822
22415	H	1.331309675531	-3.496988700486	-2.957047917073
22416	H	1.589629781011	-2.523948985876	-4.141049355508
22417	O	-1.933794761901	-4.478345367026	-1.408291802856
22418	H	-2.327257246267	-3.592694097492	-1.402842456986
22419	H	-1.765560513591	-4.678262417855	-2.340451699632
22420	O	3.238625096966	1.963722625679	-2.952333217574
22421	H	2.457344494023	1.402554741757	-3.024369418648
22422	H	2.893220480652	2.823714762075	-2.635001234342
22423	O	-0.618929137661	2.302384790963	4.941876715265
22424	H	-1.259398701516	1.588002750715	4.772252368638
22425	H	0.260936258924	1.928836211399	4.827917269400
22426	O	5.309473112822	0.904273688479	-1.692331238816
22427	H	6.025407107405	1.539617570495	-1.689365911931
22428	H	4.542927633522	1.355556533307	-2.127828405651
22429	O	-4.028608912006	3.169017560563	-2.786347869615
22430	H	-3.702883896557	2.247623851406	-2.828556628146
22431	H	-3.415102024595	3.686534901759	-3.316448724242
22432	O	-2.247625420192	0.309445464454	3.913983044980
22433	H	-1.437183367772	-0.056390480948	3.507838520695
22434	H	-2.793635270362	0.672378268010	3.203938695250
22435	C	0.303170844217	1.570790421811	0.842671967446
22436	C	1.381588290943	1.450330388751	0.026925265992
22437	C	2.053070482876	0.207796939796	-0.303616892657
22438	C	1.740005800583	-1.007322323199	0.218517750486
22439	C	-0.345806768388	-1.460047223235	-0.825333221972
22440	C	-1.226039854385	-0.568300210776	-0.256455123863
22441	C	-1.344676572417	0.781253261631	-0.815394994349
22442	O	-2.243894289369	1.619787176661	-0.559308023869
22443	H	1.716465832178	2.326391546930	-0.519875640562
22444	H	-0.180243099654	2.524489992179	1.002476284449
22445	H	2.762256591370	0.257528314287	-1.125556719813
22446	H	2.225518379329	-1.906598874388	-0.124305660473
22447	H	1.190043548007	-1.112221587657	1.137663051721
22448	H	0.100257331729	-1.250881692423	-1.781270252751
22449	H	-0.305627471511	-2.485327996505	-0.495737724379
22450	H	-0.028872196156	0.783962535677	1.504097678577
22451	H	-0.681108757059	0.992014639424	-1.667057978955
22452	H	-2.700187603475	-0.237735214525	1.150469034950
22453	N	-1.933612698577	-0.848116519298	0.903727035754
22454	H	-2.016961937891	-1.802979800417	1.206315937936

```

Ambimodal TS Water45-45
155
ANGSTROM
O      1.218319986855   -4.763406783458    0.579094952516
H      1.073379338910   -4.235817922510    1.382920908029
H      0.350634729777   -5.026827280445    0.240665245239
O      0.293193489739    4.410041374544   -2.398139628659
H      0.346779674116    3.518752057664   -2.780681668326
H     -0.349432075815    4.341365180449   -1.676002227731
O      2.862849689985    4.249871673432   -1.384191053725
H      2.030329347692    4.487365767095   -1.829180674636
H      3.228002318166    3.508301021413   -1.886189854961
O      2.113575582496    4.468150219491    1.279413458596
H      2.479049436002    4.412755427135    0.377874968354
H      1.169001319130    4.648479427147    1.189889896357
O      2.914961640400   -3.939588487806   -1.292927404815
H      3.712456553898   -3.742342704366   -0.776724676579
H      2.273626588738   -4.256074530738   -0.619364721564
O      4.861842614886   -2.900385976005    0.413808938621
H      5.636516233259   -3.395126438753    0.681395363211
H      4.350810542678   -2.726236885744    1.250160727520
O     -4.995686290504   -1.574029105390    1.550871418532
H     -4.942540531417   -0.977500276788    0.781167990435
H     -4.998085909391   -2.468508448289    1.164854164271
O     -2.418274964969    4.254412140829   -3.492260838286
H     -2.447117499773    3.340133646516   -3.804271807216
H     -1.508734181380    4.559375591566   -3.605339733975
O      3.677981268132    1.903479783111   -2.904209082818
H      4.471031832700    1.382741356162   -2.762054000776
H      3.440974324151    1.790119591536   -3.837695798886
O     -1.523110947083   -3.618111149224   -2.561629731299
H     -1.701367533294   -2.663891277793   -2.493957838304
H     -0.629563322622   -3.683361814521   -2.951948551921
O      1.441951377173   -0.320241737151   -4.051001635141
H      1.994930821468    0.150778499300   -4.702116008242
H      2.098032763790   -0.776792144227   -3.474815439085
O      0.071412361513    1.958487426692   -3.547788463966
H      0.590052782172    1.132187854404   -3.608011868476
H     -0.836106070222    1.739772426926   -3.785416927265
O     -4.961288851251    0.611193428072    3.280076883194
H     -5.192086331317   -0.121781177132    2.694576296952
H     -4.858932381043    1.396249401722    2.707526434519
O      5.181512619669   -0.634523656095   -1.128819961737
H      4.995167361800    0.090276469239   -0.528149925074
H      5.153074123566   -1.444478079292   -0.584750851820
O     -2.549611927418   -4.643413665339    4.018835030328
H     -3.253165524670   -3.999789659275    4.183374974523
H     -1.779960897585   -4.337942568267    4.526573443522
O     -4.878179168099    0.206946358568   -0.515183611576
H     -5.471336951486    0.947034454732   -0.372707036991
H     -3.972581936915    0.586114729216   -0.492550719778
O     -0.580824962249   -1.656215377773   -5.179575904399
H      0.181145142885   -1.183461821398   -4.770762601388
H     -0.341523192506   -2.585277990245   -5.196198538707
O      0.900591605755   -3.238042983785    2.844003771967
```

22511	H	0.554092268782	-2.321413362491	2.834096914647
22512	H	0.571850683080	-3.593180986545	3.680810040495
22513	O	-2.173608277058	-3.905799286709	1.512958226972
22514	H	-2.216617085293	-4.265336693770	2.430770000224
22515	H	-1.718622178416	-4.549968073050	0.952989049449
22516	O	-0.754419296320	4.952610644258	1.296792332941
22517	H	-1.260965894537	4.671754919209	0.506669647397
22518	H	-0.896576804252	5.895148445644	1.390017526129
22519	O	3.562397672890	-2.453171250040	2.657849904806
22520	H	2.673960134596	-2.851241612924	2.706879298163
22521	H	3.440389235838	-1.508184432178	2.855470250984
22522	O	-3.956763785366	2.544536918804	1.673855815174
22523	H	-3.355950725782	2.990201999201	2.283760931968
22524	H	-3.387891079070	2.148996745130	0.997559303723
22525	O	-4.801055710901	-1.318755742671	-2.915996319490
22526	H	-4.974855972040	-0.860802482144	-2.080265761173
22527	H	-4.824066968659	-2.279305815042	-2.714504622569
22528	O	2.794377093804	0.145588494392	3.313715819289
22529	H	2.721582614485	0.132764169945	4.275705696422
22530	H	1.888060027367	-0.014778981834	3.005657919961
22531	O	-2.030829104950	3.981544017560	-0.843249103489
22532	H	-2.388264878968	4.212758350904	-1.723440549921
22533	H	-2.234314767753	3.037505814144	-0.715256837138
22534	O	3.280887890324	-1.711232804499	-2.811560235822
22535	H	4.001159093583	-1.240854706998	-2.358891484993
22536	H	3.125166321322	-2.509235203991	-2.272832758485
22537	O	-2.672473209245	1.441719928899	-4.136906976930
22538	H	-3.621101084888	1.485304471533	-4.269966875657
22539	H	-2.510781550149	0.582124059282	-3.661019586095
22540	O	0.133427101371	-0.709560123426	3.221535944512
22541	H	-0.778381533815	-0.404140453201	3.099582194685
22542	H	0.372017775624	-0.506257524837	4.153333813665
22543	O	-4.590225549618	-4.061861882791	0.548799973237
22544	H	-3.656961525986	-3.990746890074	0.897533899300
22545	H	-4.971112404908	-4.848343615605	0.939596101704
22546	O	-4.357554676074	-2.445117587446	4.093588593980
22547	H	-5.175113902745	-2.164941290733	4.508320770218
22548	H	-4.409512197052	-2.125127067951	3.173793361974
22549	O	1.060526592398	-3.654403987494	-3.463883131504
22550	H	1.600924568828	-4.071877369471	-2.779151773870
22551	H	1.557338431661	-2.864383036317	-3.700980272596
22552	O	2.510851532313	2.938405449021	3.450154182909
22553	H	2.783060602982	2.052799049002	3.167105220263
22554	H	2.427064329523	3.493041446388	2.646092197803
22555	O	1.024555411305	0.150565073970	5.583733117559
22556	H	0.774504070433	1.087865597401	5.478102279655
22557	H	0.543791452553	-0.187200308569	6.357312408577
22558	O	-2.229764956633	-0.955714675785	-3.151305571047
22559	H	-1.825993314704	-1.282849732405	-3.979327751770
22560	H	-3.199856940268	-1.170397308294	-3.162749099345
22561	O	-0.690241237929	-0.828958200549	7.506880486794
22562	H	-1.366584686857	-0.995103179244	6.823346144002
22563	H	-1.102704210973	-0.280840815532	8.174470098458
22564	O	-0.464914098611	-3.311351022763	5.270378542387
22565	H	-1.017580253376	-2.500089658379	5.228708363855
22566	H	-0.020406924752	-3.277515430030	6.118377010547

```
O     -1.656609209874    3.237718653792    3.301942281154
H     -0.947138582779    3.126825482143    3.961333476980
H     -1.289322965858    3.839723304107    2.632123092225
O     -2.228013348582   -1.267095753894    5.327638768901
H     -2.139033241264   -0.507312910036    4.711798247591
H     -3.022679995244   -1.731863746193    5.009908940676
O      0.451824480075    2.780286429517    5.004155695064
H      0.615291349842    3.444820373495    5.673857626565
H      1.226440648573    2.852635124145    4.367176887264
O     -4.373645861112   -3.877435779936   -2.212374261339
H     -3.430771983539   -3.965759717270   -2.399519548881
H     -4.476656673480   -3.957708314613   -1.249210394301
O     -1.229375025878   -5.379219265332   -0.578508643572
H     -1.308282464091   -6.235329094067   -0.999124664895
H     -1.361751495307   -4.705063070061   -1.285350406414
O     -2.291424306075    0.565509859989    3.413959301940
H     -2.033009138742    1.503993612398    3.382576599969
H     -3.275746882701    0.564709266098    3.421367565241
O     -1.172515258552    0.812454580892   -6.454410555419
H     -0.977576683057   -0.112140115286   -6.254411659297
H     -1.801336603097    1.101781723783   -5.776762607490
O      2.986844860988    1.296188263359   -5.585201133901
H      3.567981934807    1.229929676150   -6.342160164885
H      2.225048016365    1.880411384591   -5.860078501207
O      0.820880690555    2.652543033941   -6.104354583813
H      0.151116048243    2.035347871138   -6.457062571606
H      0.590216768776    2.748085288110   -5.164846685445
C      0.227471352628    1.652651363646    0.830047014594
C      1.282755751831    1.579315359974   -0.017717522556
C      2.076130684686    0.392963498499   -0.296993332962
C      1.899930059093   -0.826586896787    0.272475500301
C     -0.178528168085   -1.481706746497   -0.820919347218
C     -1.139777098757   -0.709214778695   -0.209772481617
C     -1.440113098104    0.629949419654   -0.717034120092
O     -2.453435522474    1.311096895050   -0.392977259447
H      1.518501844136    2.441056175668   -0.636861424949
H     -0.329217140537    2.569533456009    0.952617115402
H      2.766816432351    0.500603515287   -1.130148910470
H      2.486236399099   -1.682663326001   -0.026686472970
H      1.313227402604   -0.972439763243    1.165760876175
H      0.248415993311   -1.183731414783   -1.762804650436
H     -0.004437075626   -2.502487079623   -0.515298124047
H      0.000445500803    0.885251649052    1.553703279133
H     -0.852601932886    0.974581839266   -1.577535973657
H     -2.547483762853   -0.542208341243    1.292199661481
N     -1.800592937746   -1.111186807895    0.934370439999
H     -1.809392558752   -2.082569148773    1.207970199637

Ambimodal TS Water45-46
155
ANGSTROM
O     -3.211472413011   -1.975182257877    3.069305945765
H     -4.143703284529   -1.720612854156    3.024237476893
H     -3.116497399319   -2.689632415787    3.704277216293
O     -5.465933728324    1.090955202819   -0.857913108877
H     -5.265521834629    1.698439439907   -0.139020539904
```

22623	H	-4.855741573713	1.316389312802	-1.580786575384
22624	O	0.906757698923	0.164410380662	-3.575303663884
22625	H	1.848869556431	-0.090639266862	-3.523363033584
22626	H	0.836814807453	1.030009219161	-3.132605692967
22627	O	4.068019243196	-3.072808950707	0.772317300157
22628	H	3.200640930442	-3.273329538363	1.157571786995
22629	H	4.144451782016	-3.684618242858	0.018111257647
22630	O	1.275047741671	-3.814015094295	-2.681682415626
22631	H	1.555936196146	-3.414683249146	-3.530543055553
22632	H	0.329048122667	-3.608418351654	-2.615407295251
22633	O	-3.888842607241	1.262048334034	-3.119677483438
22634	H	-3.449105172213	0.384930544872	-3.170170695863
22635	H	-4.588582696072	1.242113106240	-3.775214890248
22636	O	-0.044770148927	1.921232931588	5.235659668551
22637	H	0.560680250118	2.372957059476	4.625527675677
22638	H	-0.611216095743	1.369335027269	4.679979163073
22639	O	0.805602195736	-0.208925671425	6.595430776711
22640	H	0.520683385380	0.662685927391	6.231067856004
22641	H	1.493695301598	-0.035862257129	7.236909254894
22642	O	-1.511085602896	0.036640964130	3.619927090877
22643	H	-2.111068126588	-0.671512207027	3.299750824865
22644	H	-1.822809511574	0.860739213221	3.213449790294
22645	O	0.939919166401	-1.165673829465	4.049138778739
22646	H	0.982083307596	-0.965286626936	5.001834985075
22647	H	0.065177883001	-0.849988513356	3.760187771807
22648	O	-3.573999454308	3.879074800365	-0.752963351141
22649	H	-3.122908979258	4.347763418410	-1.468867801270
22650	H	-3.222734909122	2.968894465165	-0.763801294960
22651	O	3.521534423465	-0.498084435800	-3.568873103784
22652	H	3.909311298370	0.397513561244	-3.503966117265
22653	H	3.942046208073	-0.983131007612	-2.832552044259
22654	O	-2.022147880510	5.145955530243	-2.784001964321
22655	H	-1.922288124332	4.361964337264	-3.355282653170
22656	H	-2.404125171307	5.834913728502	-3.327565852899
22657	O	-4.719142350699	-1.373864375994	-0.172721346823
22658	H	-4.957453185730	-0.459127002929	-0.457316102962
22659	H	-5.112437912486	-1.454662470744	0.708703250775
22660	O	1.948344078322	-2.382659930753	-4.877728460864
22661	H	1.190243325638	-1.803473595849	-4.992962673059
22662	H	2.666222805957	-1.808854828373	-4.567604576997
22663	O	-4.967002095855	-2.585541139332	-2.615685889740
22664	H	-4.675195132166	-3.464316684467	-2.361880166713
22665	H	-5.064561800974	-2.102989093107	-1.773343060728
22666	O	0.502957361108	2.753640732206	-2.787457306000
22667	H	1.207533586835	3.004442640308	-3.412753832944
22668	H	-0.340488950424	2.824788469610	-3.280210536478
22669	O	1.335482203473	-3.920246335988	4.071703619280
22670	H	1.550373051146	-4.139254849561	3.150975101347
22671	H	1.354746597148	-2.949952591100	4.101137264865
22672	O	-0.026420709792	4.590685593236	-0.876144070520
22673	H	0.290264147102	3.862387213529	-1.453058293584
22674	H	-0.665842443966	5.061184084554	-1.428180690418
22675	O	1.477250330226	-3.992478836537	1.316640139086
22676	H	0.533453692845	-3.771271301073	1.357049383361
22677	H	1.544346139426	-4.702313499360	0.645095573123
22678	O	-1.339737605970	-3.816977897057	4.296554931743

22679	H	-0.377916675052	-4.016604903266	4.328641014621
22680	H	-1.454039038089	-3.092398609488	4.939392668238
22681	O	-2.190049865017	2.681240070936	3.135729431091
22682	H	-2.008029834347	3.116370006347	3.970169586075
22683	H	-2.035744046510	3.357285375337	2.430137020610
22684	O	-4.153902177589	1.356489239013	1.763348722117
22685	H	-3.657640373446	1.893305326845	2.397247659435
22686	H	-3.572053548920	1.302268344265	0.975822507950
22687	O	4.836590650243	-0.504010227329	1.648173272346
22688	H	4.008994606359	-0.213108699435	2.064139935774
22689	H	4.712259215837	-1.445386588512	1.460719070258
22690	O	3.908370641774	-4.206471520791	-1.719974060887
22691	H	4.512877600174	-3.588629426475	-2.139646892042
22692	H	3.024712026128	-3.985915527128	-2.054894724800
22693	O	-1.508991106745	-3.423777868187	-2.687414545736
22694	H	-1.808934969875	-4.122562129906	-3.270915339545
22695	H	-2.043651065520	-3.502005629460	-1.858774576739
22696	O	-1.306683473671	-4.009540667005	1.646049897523
22697	H	-1.412608299742	-3.822988404873	2.613534512272
22698	H	-1.256473336128	-4.966011821236	1.584472715072
22699	O	4.468163214615	1.946259765831	-2.923197120957
22700	H	4.996821927719	1.638915910826	-2.170756613847
22701	H	3.902479876163	2.643382750729	-2.531776686454
22702	O	-5.585081633966	-0.783409738033	2.409661895764
22703	H	-6.478567469363	-0.510768987941	2.616957533518
22704	H	-5.084156525562	0.043657111315	2.206128826996
22705	O	4.899416814375	-1.439834409122	-1.448957870484
22706	H	4.615405366304	-1.872772690493	-0.629246476796
22707	H	5.289192062668	-0.601630721121	-1.177694742014
22708	O	1.484659296958	4.947684421759	1.346584407604
22709	H	1.030952783798	4.773067775472	0.496896541743
22710	H	1.093840641757	5.753504987490	1.688312763116
22711	O	2.365676914517	2.693691672955	-4.786214507305
22712	H	3.236440594563	2.504861539211	-4.417319937636
22713	H	2.057008547754	1.878736059506	-5.191312192552
22714	O	4.000213295807	3.705611298274	0.919838353726
22715	H	3.201366237277	4.193983084918	1.163026213316
22716	H	4.290509118267	3.253676843978	1.737555581411
22717	O	-1.876399950867	4.497162518741	1.228658716375
22718	H	-1.141740411596	4.480994533059	0.593858633641
22719	H	-2.667096460367	4.364669471706	0.661164844106
22720	O	-2.867453529745	-1.176064542048	-3.550960322443
22721	H	-3.700121370869	-1.661358173215	-3.363808164653
22722	H	-2.171722620630	-1.771983513164	-3.224876813640
22723	O	4.681780095507	2.340872529593	3.179385367949
22724	H	5.529995499802	1.916567363590	3.036582308751
22725	H	4.033488228892	1.610709619925	3.209963504644
22726	O	3.136010435487	3.935087828715	-1.673804869284
22727	H	3.502414408484	3.841691935707	-0.777878645433
22728	H	3.343457146162	4.820353128441	-1.972245541931
22729	O	1.602633287936	-5.757707807653	-0.728569354974
22730	H	1.375309718527	-5.172867154386	-1.471065192637
22731	H	2.531304061501	-5.968487086948	-0.855380711828
22732	O	-1.594934476551	-1.601380860633	5.933899017131
22733	H	-0.809952643058	-1.339885309621	6.429933070712
22734	H	-1.729106219115	-0.904177938482	5.277108068497

```
O     -3.042495345884   -3.584305896604   -0.522284011173
H     -3.543877520576   -2.768309087632   -0.353679256059
H     -2.503308675384   -3.734261091021    0.270767187254
O     -1.862935153512    2.767263041478   -4.109949439491
H     -1.588937776823    2.046911979953   -4.723858115248
H     -2.601471422392    2.361771525850   -3.615053567552
O      1.659409988592    3.091053614827    3.364023511138
H      1.474405092732    3.700267277686    2.626035365048
H      2.555097569753    3.280694647141    3.657712147664
O      5.282576637011    1.281038357069   -0.331658599189
H      4.928971144277    2.112276853052    0.004442232139
H      5.177135968210    0.645178785483    0.407072198897
O      2.667841677637    0.544334341230    2.975832077768
H      2.117793036602   -0.164961497823    3.371910222440
H      2.075193234006    1.301939233846    2.855985668026
O     -1.212815303820    0.431692866395   -5.230898974384
H     -0.402890000135    0.282378864311   -4.707810957943
H     -1.869147704666   -0.164545755933   -4.844757370699
C      0.235203955269    1.562596245527    0.908055874399
C      1.315033848668    1.542944653277    0.086788510521
C      2.033903985148    0.353869455598   -0.317051710477
C      1.742173645074   -0.915932613105    0.101606845617
C     -0.214170474618   -1.363191387379   -0.941477850406
C     -1.194224109855   -0.612271388419   -0.320082187178
C     -1.564223676150    0.716460222767   -0.799232968333
O     -2.546353918063    1.375482007232   -0.400993341504
H      1.614505192870    2.460307694636   -0.413083921756
H     -0.304826559610    2.473535551032    1.115055645218
H      2.740978707505    0.487519286904   -1.131642492893
H      2.278964450414   -1.760941044298   -0.302746179513
H      1.239305718034   -1.112225172845    1.038754350025
H      0.161288518903   -1.071709224268   -1.908403507431
H     -0.082877147293   -2.402309243787   -0.679087600248
H     -0.054792411569    0.719466146846    1.517688030054
H     -1.008524803896    1.091595239605   -1.673386303918
H     -2.706494981358   -0.650432924939    1.075673027553
N     -1.801493151520   -1.033446218493    0.857938210462
H     -1.673944645922   -1.982764588688    1.164894691719

Ambimodal TS Water45-47
155
ANGSTROM
O      3.102824510541    4.118270759031    0.469267756167
H      2.555522385716    4.650804493363   -0.117070948759
H      2.664456512424    4.123139419268    1.328960150906
O      4.851945292502    0.150417831362   -2.169562295882
H      4.855807474257   -0.724418859211   -1.691686601566
H      5.767151066561    0.339101783539   -2.383321019747
O     -2.615031715035   -1.740533546223    6.172044805416
H     -2.798673724524   -1.725640849777    5.213793960522
H     -1.748652400235   -2.137431951632    6.271610690573
O      2.744816227795   -0.990201706684    3.798431079983
H      2.336894508502   -0.255874287726    3.307748843215
H      3.011315860575   -0.593750256487    4.636350920331
O     -0.933479344105   -0.466833538754   -4.155329399736
H     -1.651247381666   -0.637423819179   -3.527197419973
```

22791	H	-0.717574988663	0.472913404039	-4.072873282394
22792	O	1.078970262034	-3.186747763003	3.636207215072
22793	H	1.639102034671	-3.693059820579	3.009763253781
22794	H	1.683955082684	-2.506786462622	3.970148492332
22795	O	4.995196528358	2.389244451106	-0.460331436812
22796	H	4.276207600093	2.956742895725	-0.127954847751
22797	H	4.616651695195	1.694510195005	-1.019542859399
22798	O	-3.919627715351	1.131333615859	3.909654827640
22799	H	-3.560327033080	1.858123300794	3.378160081741
22800	H	-3.534155935136	1.221614392260	4.813811003673
22801	O	-6.065334063000	0.205317583493	2.479834513812
22802	H	-5.494441170213	0.503443144487	3.208225205360
22803	H	-5.886710383735	0.836438250560	1.762358735697
22804	O	-2.598044423307	-2.587830063159	-4.979933730930
22805	H	-2.025219020365	-1.893596761308	-5.324103049485
22806	H	-3.046811465814	-2.184290549119	-4.223569406155
22807	O	-4.847384590872	1.889975819300	0.625587043419
22808	H	-4.003075487794	1.516064654103	0.304344174642
22809	H	-4.575832443153	2.501741535211	1.320523670722
22810	O	-5.255734610190	-0.591927148645	-0.990580584557
22811	H	-5.014432322003	-1.087568376256	-0.184587503007
22812	H	-5.449582230107	0.302331426604	-0.698034236686
22813	O	4.423863159877	-1.926663129530	1.910902875382
22814	H	5.219109516077	-2.198566807005	2.372568126659
22815	H	3.810739849887	-1.580554792012	2.600231953878
22816	O	-3.267526004131	1.911996378216	-3.312915881323
22817	H	-3.384457552258	0.972908261493	-3.142286525544
22818	H	-2.330813285800	2.029650172837	-3.524036203004
22819	O	-2.949889969567	-1.308888695099	3.500198625690
22820	H	-3.308593745618	-0.400471895758	3.635209328825
22821	H	-3.488087854891	-1.692766999162	2.789429540461
22822	O	-3.327698326853	-3.477284401151	-1.178379196908
22823	H	-3.448449105949	-3.664882807710	-0.223201325753
22824	H	-4.150471566961	-3.713381600067	-1.610476181459
22825	O	-2.290758923462	3.876337114754	-0.371530138164
22826	H	-2.223836706618	2.888471327816	-0.408456472239
22827	H	-3.190797314325	4.023830380244	-0.765409387360
22828	O	0.745142957805	-4.884330788578	-0.208029903172
22829	H	-0.022166481754	-4.962854007714	-0.866473344691
22830	H	1.030546353479	-5.787975061109	-0.061569067379
22831	O	-0.322888039456	-0.802601166775	3.188423587234
22832	H	-1.285010363885	-0.980804152459	3.206694330959
22833	H	0.099527924090	-1.660056928354	3.345419790472
22834	O	2.711157743142	-4.183099671124	1.746318516140
22835	H	2.186418251224	-4.243314938967	0.939074765225
22836	H	3.341800368188	-3.457370273322	1.626886135432
22837	O	2.588028273936	-3.401206579556	-1.802516211934
22838	H	1.933960662982	-3.958964132821	-1.358871275503
22839	H	2.119838606264	-2.969206677633	-2.535037165947
22840	O	1.295452242168	4.072915324703	2.653572527072
22841	H	0.625552506234	3.831133907683	3.314388497975
22842	H	0.784321222543	4.586735138772	1.997786015090
22843	O	1.263773690311	-2.046706866997	-3.882636600013
22844	H	2.010264385689	-1.486732319800	-4.146339994045
22845	H	0.472529963049	-1.485456794655	-4.011334965379
22846	O	2.657510901382	1.193296649640	5.584079558748

22847	H	3.020811707210	1.913578444629	6.098050442108
22848	H	2.676993198146	1.480093037877	4.634925236064
22849	O	2.064281859110	2.203296754943	-4.544206040308
22850	H	2.444315418148	1.311178061813	-4.547202697079
22851	H	2.639169821225	2.737356778065	-3.971892742221
22852	O	-3.561926117398	-4.388685208874	1.364520666074
22853	H	-3.620934980224	-5.344582346409	1.333790394465
22854	H	-2.646167159158	-4.187812576944	1.646040791988
22855	O	-4.573831957122	3.638515645006	-1.597315713377
22856	H	-4.218427173663	3.053144911448	-2.293521555861
22857	H	-4.987020662588	3.052808243979	-0.945399565546
22858	O	-3.181463031342	-1.109890100423	-2.588065017784
22859	H	-4.002411891068	-0.824457339553	-2.130568385139
22860	H	-2.941215141652	-1.914406370595	-2.100782790517
22861	O	-2.910056837082	3.216460277865	2.362893938289
22862	H	-2.641553221087	3.576001791688	1.505710972477
22863	H	-2.136404654747	3.309149797876	2.947453659575
22864	O	2.538273960636	1.618810391820	2.994514126997
22865	H	3.413343483777	1.538807246120	2.557298857877
22866	H	2.147872353089	2.472376141811	2.745640728822
22867	O	-0.934074930670	4.665266281372	-2.489194615619
22868	H	-1.572075175425	5.105752476178	-3.052320344044
22869	H	-1.435630748053	4.365291838561	-1.683645704806
22870	O	-1.216038931946	-5.126212087073	-1.826813352965
22871	H	-0.924647411588	-4.884482008409	-2.740395551120
22872	H	-1.948033600929	-4.520145403384	-1.621110613313
22873	O	-0.152728171355	5.352305878274	0.738381070720
22874	H	0.416586342696	5.461238476883	-0.034341654886
22875	H	-0.958613682169	4.917784242231	0.425583540733
22876	O	-0.423567219085	2.188611031327	-3.609719218502
22877	H	0.469366768836	2.193899794407	-4.026005646085
22878	H	-0.525902354307	3.078234098244	-3.237746284083
22879	O	4.841928137499	-2.153581589751	-0.932162545947
22880	H	4.690835923809	-2.076430973811	0.018112505778
22881	H	4.092274863946	-2.668390236326	-1.291883401435
22882	O	-0.063028553147	0.957547842685	5.139875677440
22883	H	-0.117024453636	0.304869195170	4.397841099685
22884	H	0.866532907460	0.979944742950	5.421525004630
22885	O	-0.850945954555	3.294313917803	4.205043526223
22886	H	-1.224864890111	3.718681596852	4.978534191445
22887	H	-0.562048530595	2.393642074028	4.503092442764
22888	O	3.557334703482	3.688805552732	-2.735568224445
22889	H	4.263446322577	3.481377230577	-2.112497488374
22890	H	2.922577419570	4.245300496844	-2.265501325257
22891	O	-4.717469361131	-1.772313201677	1.435561512669
22892	H	-5.343138331299	-1.139624251690	1.881631914039
22893	H	-5.026184428028	-2.668569825276	1.578777193793
22894	O	3.351744529650	-0.272409261492	-4.362672068572
22895	H	3.863493235996	-0.112746369903	-3.538071230846
22896	H	3.983377041811	-0.493507726070	-5.047583205270
22897	O	-0.505999527561	-4.223712661738	-4.202649243982
22898	H	0.205871342409	-3.569107728284	-4.161714638096
22899	H	-1.287398891917	-3.753468301849	-4.548296281569
22900	O	4.883222419945	0.993051977188	1.860195760542
22901	H	5.084597826515	1.522313858253	1.061562894061
22902	H	4.795648779671	0.077293649104	1.572634082782

```
O      -2.608279818127    1.022107353227    6.218039012412
H      -1.675112533253    1.043522913526    5.949707270628
H      -2.774494058130    0.085985975794    6.427190843852
O       1.499539919438    5.385131238111   -1.601899493224
H       0.679113978580    5.076254610372   -2.042140963296
H       1.724804614861    6.226931827992   -2.001438470317
O      -0.945328349508   -3.880237899621    1.788885091212
H      -0.340537670809   -4.241910181441    1.117341439035
H      -0.400551315467   -3.685061855206    2.563766951178
C       0.219529226335    1.586056589540    0.794427749792
C       1.304381569357    1.470524675633   -0.015210247213
C       2.058137442513    0.252205502012   -0.228217906580
C       1.754128961448   -0.950142283006    0.344121956849
C      -0.129628433721   -1.520109246094   -0.910531994985
C      -1.141874032804   -0.779639243054   -0.333767692898
C      -1.439425475667    0.584730374786   -0.773716646993
O      -2.426591900026    1.254636613920   -0.383168540856
H       1.578555096009    2.313776582447   -0.649138069329
H      -0.316172521736    2.520648539413    0.879884212954
H       2.791276325473    0.277171657887   -1.030982445817
H       2.312524615922   -1.837731779913    0.089894179301
H       1.146840296814   -1.025428356232    1.234556830162
H       0.343343406415   -1.198203493618   -1.820804537493
H      -0.014042459112   -2.564206379230   -0.665544003160
H      -0.046454055588    0.845774635282    1.535164985167
H      -0.863561664287    0.964905875502   -1.632718586977
H      -2.689213322563   -0.752411978050    1.025125588930
N      -1.867765345295   -1.248009694069    0.732375099520
H      -1.734275398659   -2.185891100157    1.073751661560

Ambimodal TS Water45-48
155
ANGSTROM
O       2.172199788388   -4.095299794177   -1.777288888545
H       2.145529208703   -3.296506496599   -2.333121778370
H       2.925805155895   -3.978365417585   -1.187854227203
O       3.942753371794    2.882434940613    0.779077357991
H       4.299020828232    2.922564262807   -0.128313092955
H       3.284644601821    3.592179361730    0.804939607267
O      -4.796886926011    2.916402494416    1.907628325693
H      -4.848554805239    2.854473399958    2.869743448306
H      -4.778871230749    1.978011411993    1.620408054997
O      -2.244635391224    1.367851207728    2.513791606737
H      -3.047443143099    1.406550639871    3.062525111132
H      -2.525561531285    1.460387199489    1.589096991102
O       3.088933176158    1.096723203674    2.696621413493
H       3.567867848005    0.265797298410    2.531226468559
H       3.420941869276    1.736714003847    2.041809574081
O       3.389103065483   -2.342757192787    4.719219259374
H       2.874613432627   -1.675984305061    5.222311094006
H       3.921370130008   -2.812992428372    5.360883648122
O       0.831675153653   -0.237901788497    3.467392451422
H       1.095598374666   -0.309631928388    4.408649606892
H       1.540101792382    0.281342032338    3.053034410491
O       0.779618048507    3.358562200549   -2.553786659414
H       0.230384894941    3.630675606971   -1.804294532372
```

22959	H	1.544935583354	3.978785009817	-2.549929301190
22960	O	-1.668665535513	1.980120323955	5.772737014760
22961	H	-0.817319121788	1.892712346259	6.233056104478
22962	H	-1.510739656044	2.644155071162	5.078226494906
22963	O	1.793381586356	4.828345348160	0.515023088294
22964	H	1.654435672021	4.670077909548	1.465131319637
22965	H	0.906236116916	4.826668008592	0.124287890444
22966	O	-1.011445969117	0.195280654513	-4.362570574106
22967	H	-1.767172286455	-0.194588123921	-3.894404957881
22968	H	-1.225797809670	1.146507643356	-4.446545104244
22969	O	5.547254447173	0.353006430025	0.399878770057
22970	H	5.311545915104	-0.195051469955	1.160210844259
22971	H	5.128251352068	1.208700194406	0.541469473142
22972	O	-1.758670086964	-3.617872119880	-2.672553041557
22973	H	-2.117127439417	-4.363774008696	-3.157080396751
22974	H	-1.030240643919	-3.250159601597	-3.241345423524
22975	O	1.634275092169	-0.780724710201	5.996005637936
22976	H	0.891450779115	-1.421425777353	6.128521586794
22977	H	1.380580368919	0.045715317831	6.442682272000
22978	O	-4.241312435191	1.699440716862	4.414994100551
22979	H	-4.528249525188	0.776745107555	4.449918072266
22980	H	-3.605979518031	1.820103075432	5.130925823301
22981	O	-3.968308720473	-3.298702859758	2.355725177240
22982	H	-4.221204798263	-3.210823071382	1.418994620010
22983	H	-3.032777973882	-3.542241426182	2.333005989123
22984	O	-4.216000050618	-2.936099888810	-0.366551899696
22985	H	-3.358106659402	-3.097168608918	-0.765844087194
22986	H	-4.469725365175	-2.025906069844	-0.617019979150
22987	O	-4.240017554478	-1.055621742243	3.781669444972
22988	H	-4.248020833344	-1.948173520686	3.369200159194
22989	H	-3.315170134731	-0.926933713714	4.072104389694
22990	O	2.434374391616	-1.752564363931	-3.149266821282
22991	H	3.325291283550	-1.663531160756	-2.778521993667
22992	H	2.055208583686	-0.858820281580	-3.110132178395
22993	O	-3.094225903775	-1.305455118973	-3.278667891990
22994	H	-2.653115334048	-2.117177061628	-2.950210238532
22995	H	-3.614672828454	-1.562592288611	-4.041794687681
22996	O	-2.774801074255	4.759773644907	-2.545608165363
22997	H	-3.255031012628	5.421636236037	-3.042759392936
22998	H	-3.434390640354	4.283617184239	-1.992930655658
22999	O	0.922516049905	1.733032754531	6.758239328462
23000	H	1.581634781157	2.085039115827	6.097348872187
23001	H	1.152937581304	2.131185136803	7.597537397313
23002	O	-0.124566700598	-4.650909904660	-0.522756499912
23003	H	-0.821838777899	-4.479194413806	-1.169907073338
23004	H	0.711377595145	-4.511036906373	-1.020704643573
23005	O	-4.390139882765	3.372761524276	-0.952648058650
23006	H	-3.725031844193	2.685294167185	-0.748469505596
23007	H	-4.887924681842	3.483619820050	-0.137925941408
23008	O	-2.744366787776	4.601697194820	1.524048145731
23009	H	-3.199654098616	5.435725114011	1.406967071575
23010	H	-3.449558372063	3.914850448658	1.600575974910
23011	O	2.022307819454	-4.623748286151	1.493596013703
23012	H	1.320515903791	-4.872399991314	0.880970859655
23013	H	1.621391373300	-4.022286885109	2.150085202714
23014	O	-1.196193820786	3.517906073342	3.522453677973

23015	H	-1.681640754525	4.181651397826	3.012006033169
23016	H	-1.474382069423	2.659998354768	3.098765521330
23017	O	-4.690647867585	0.246592407804	1.530541851814
23018	H	-5.348574612488	-0.226673077416	1.019224130344
23019	H	-4.593389649473	-0.232572974204	2.390944084169
23020	O	-1.226083336765	-3.602256001068	1.792826269627
23021	H	-0.502935030197	-3.470063737996	2.415647928852
23022	H	-0.862206153159	-4.066499609590	1.020303472157
23023	O	1.375847609354	3.690122069893	2.963867540651
23024	H	1.533202641603	2.827301993488	2.563675751047
23025	H	0.422762642640	3.692213138914	3.219703593386
23026	O	4.201554914717	0.984841980622	-3.311349152332
23027	H	4.470542059395	0.163676981740	-2.882286632923
23028	H	3.236853008760	0.945675092369	-3.431789959881
23029	O	-0.031416668313	-2.492854581596	-4.326508497312
23030	H	0.893588869527	-2.416014040400	-4.056634015361
23031	H	-0.320084917833	-1.591747684778	-4.531128041932
23032	O	1.428723525035	0.812670949133	-3.268382211812
23033	H	1.261991341897	1.719305814416	-2.943662384594
23034	H	0.645083997953	0.600845837055	-3.806244516207
23035	O	-0.391656395097	-2.457412856849	5.841741488157
23036	H	-0.121149987648	-2.973495758223	5.072155114521
23037	H	-1.029416670275	-1.799535961172	5.496150302916
23038	O	-4.693443659941	-0.379866732963	-1.216332034791
23039	H	-4.206341769089	-0.532827746023	-2.039399562988
23040	H	-4.204322623499	0.302471339010	-0.738075529376
23041	O	4.858450715220	3.014448676100	-1.779473165286
23042	H	4.597502324896	2.261574682669	-2.372716726095
23043	H	5.784631739596	3.181507855177	-1.954196498988
23044	O	4.048791303644	-3.420664848741	0.379804408786
23045	H	3.292224829649	-3.898807811783	0.822869425099
23046	H	4.817636346708	-3.981505993913	0.492571171291
23047	O	2.877017763078	4.928426997351	-2.088153129233
23048	H	2.646568248850	5.124936830941	-1.169620600131
23049	H	3.637155138291	4.324635592638	-2.050579948698
23050	O	4.296698636265	-1.339614898216	2.278187138483
23051	H	4.126162491844	-2.015837883942	1.600898398253
23052	H	4.064348028957	-1.737624776515	3.132824265588
23053	O	-1.386375894815	2.871249469468	-4.158769422211
23054	H	-0.521144503341	3.102106459177	-3.778184047235
23055	H	-2.012511367740	3.433884471905	-3.680813677531
23056	O	2.709576187036	2.581212290660	5.038784404220
23057	H	3.028991427410	1.905057769749	4.420747342704
23058	H	2.296025571447	3.244783482415	4.455568859947
23059	O	-1.717307769943	-0.459250107828	4.607676275208
23060	H	-1.123712014456	-0.289323187439	3.861880341012
23061	H	-1.755565869700	0.392538628060	5.092833825257
23062	O	4.706183164601	-1.316566154338	-1.566953255823
23063	H	4.524520750155	-2.073675646795	-0.997477530323
23064	H	5.103868269821	-0.656190598298	-0.962094400505
23065	O	1.043674345868	-2.903575392535	3.367812423653
23066	H	1.864174079579	-2.946354409757	3.884814707102
23067	H	0.895047932033	-1.935109725559	3.251078806116
23068	O	-0.851230891528	4.565404161920	-0.548319859756
23069	H	-1.469656838332	4.495226175496	0.198193711797
23070	H	-1.410720505907	4.779684540017	-1.311579442961

```
C      0.208504141510    1.636800645240    0.716882990461
C      1.341651104306    1.421038024174   -0.001208033512
C      2.002208561528    0.146152044914   -0.176599458051
C      1.527812267869   -1.044908469615    0.292009107432
C     -0.368880631471   -1.263406049742   -1.072100580956
C     -1.336676513090   -0.544661360518   -0.397759360733
C     -1.649535674802    0.844150339885   -0.758663920825
O     -2.610719652107    1.474875854058   -0.255346235488
H      1.748519311677    2.239028841859   -0.590307103495
H     -0.253673271578    2.612475024225    0.745265571844
H      2.838944455406    0.142032165099   -0.869077131807
H      2.028597754908   -1.971786434830    0.053462271113
H      0.827843806103   -1.107591491811    1.111812475438
H      0.092601059008   -0.852255825221   -1.951988279609
H     -0.273284540206   -2.326871170540   -0.935501884541
H     -0.172414660472    0.928409647267    1.437518441546
H     -1.147479450548    1.270261079859   -1.641892305153
H     -2.787539139953   -0.542965032653    1.079259403934
N     -2.032611690932   -1.064682032699    0.664359249145
H     -1.843471579134   -1.986666824346    1.028899481345

Ambimodal TS Water45-49
155
ANGSTROM
O     -3.949076586729    1.951209817152   -2.826267906997
H     -3.603655885337    1.992987948487   -1.921680868186
H     -3.276159052074    2.384842442960   -3.384985476742
O      0.185013459226    3.364530838072    4.299518122422
H     -0.684489037260    3.792654967117    4.299951333061
H      0.015063290511    2.464524862693    3.986594804100
O      1.676930265782    4.895867599251    0.239071472408
H      2.473289903748    4.355465600841    0.071674881922
H      1.383348945090    5.164856831676   -0.643276054282
O     -2.718093773818    2.051606193643    2.252819246984
H     -2.713367278801    1.870704017283    1.296023080789
H     -1.982612057822    1.542678998943    2.654523761488
O      3.929563466396    3.497476785194   -0.462090027232
H      4.619849725771    4.132364354651   -0.660063317997
H      4.304249802346    2.924304125045    0.255159272370
O      1.837173390066   -4.056178660533   -2.129371637721
H      1.110409084740   -3.968585871252   -1.492741845606
H      2.382385336572   -3.250984412966   -2.091184680594
O      1.097364150354   -4.199056767280    2.118500127909
H      1.368718033970   -3.458889303334    2.677290208636
H      1.904160041724   -4.476082701275    1.627873028267
O     -1.048690133897   -1.746102032950    4.106621845194
H     -0.089813705221   -1.935655655188    4.075322542589
H     -1.448299344719   -2.344703768052    3.449919205717
O     -5.036701179584    0.323487172156    0.281651586965
H     -5.198771592971   -0.428228479447   -0.333095339513
H     -4.177065087456    0.690940452458   -0.000475729608
O     -4.358719041015   -1.132126212387    2.420371782119
H     -4.734064497404   -0.639966620888    1.658306212399
H     -4.512934845404   -0.562193821056    3.185133414868
O      1.659451930751   -1.942213552045    3.898026861696
H      1.875510623582   -1.074401465277    3.510078351703
```

23127	H	1.833783815272	-1.820593814865	4.838514904010
23128	O	-3.885124284438	-0.587140074363	-3.522563966726
23129	H	-3.914423657820	0.371608398285	-3.262263009129
23130	H	-4.355043138329	-0.649530967592	-4.354609575281
23131	O	-4.728364646271	1.251434078093	3.807873848326
23132	H	-5.525742365078	1.749230478612	3.620999718766
23133	H	-4.076376266520	1.566655709138	3.138763709440
23134	O	-2.711011718690	1.688785180775	5.590304217384
23135	H	-2.626818307206	2.642004787911	5.456009250380
23136	H	-3.533923312593	1.438485166123	5.129833940799
23137	O	2.236924077352	4.680864380543	3.034758204181
23138	H	1.433384214363	4.296438776945	3.431058989279
23139	H	2.059472493945	4.829639964877	2.098996744559
23140	O	-3.183299772269	4.521294010098	0.621310609965
23141	H	-2.362276886148	4.470168585763	1.139457490161
23142	H	-3.785042197916	3.832600789699	0.945485928078
23143	O	3.711522865753	-2.026277974171	-2.341099378780
23144	H	3.574803375265	-1.144730507901	-2.757082810250
23145	H	4.277900791711	-2.507875308221	-2.946643705095
23146	O	0.746333333500	4.921753628862	-2.379299722792
23147	H	0.568887639428	5.184156375495	-3.302875281888
23148	H	-0.133370712544	4.713554285567	-2.019111327326
23149	O	-2.916432874826	-3.620410288395	-0.568293588210
23150	H	-3.383697606033	-3.745819435728	0.277582576550
23151	H	-1.998510948138	-3.888258517775	-0.406613302583
23152	O	0.385659555856	-3.015597180147	-4.259529522924
23153	H	-0.525707695812	-2.985932209828	-3.915599528326
23154	H	0.912719622232	-3.499428356236	-3.598765260148
23155	O	-0.649320844046	4.708429019670	1.727402743352
23156	H	-0.521701658641	5.407445571138	2.370286647481
23157	H	0.117396143085	4.751533556421	1.128560837608
23158	O	-1.745611520469	3.147849063214	-3.929989687388
23159	H	-1.048609544332	2.472621353325	-3.769492465913
23160	H	-1.807010735643	3.657955311645	-3.099547144994
23161	O	3.673093858463	2.601169315188	3.880121863273
23162	H	3.392742319099	2.587564813136	4.824992818278
23163	H	3.266068765785	3.428930110120	3.536822146927
23164	O	0.222161517151	1.425419997504	-3.369373257790
23165	H	0.442650039226	0.705224783698	-3.983500823853
23166	H	1.054297204332	1.884369048154	-3.168704226487
23167	O	3.070946713195	-4.767305008575	0.412382024139
23168	H	2.790236113262	-4.828270972175	-0.510833643531
23169	H	3.769210646934	-4.080118692201	0.424244942783
23170	O	-1.635143843714	-3.622371378459	2.285703490681
23171	H	-2.466914171670	-4.103186494107	2.345045123640
23172	H	-0.890848623376	-4.219140788115	2.424838377699
23173	O	-4.974734089539	-1.790592223598	-1.322089708337
23174	H	-4.660203490928	-1.409202937125	-2.161433116361
23175	H	-4.299010463892	-2.431574600575	-1.060726326247
23176	O	2.266076695369	2.542879852443	6.129570824714
23177	H	1.532864281755	3.027851636960	5.726579257811
23178	H	1.961982712636	1.618127969115	6.193007883379
23179	O	5.039794739317	2.121940449327	1.486148049680
23180	H	4.974091369429	1.152695743536	1.469293193351
23181	H	4.739917114417	2.387933914007	2.368691597851
23182	O	-1.354270853160	-0.617509094935	6.668202785953

23183	H	-1.471679089284	-1.168039105352	5.879248327391
23184	H	-1.918092896963	0.156670857968	6.549611706160
23185	O	4.733357329560	-2.658338577980	0.170236105684
23186	H	4.665478641608	-1.903215586327	0.773655036965
23187	H	4.464754367662	-2.330968686481	-0.702578529065
23188	O	-0.140644067540	5.084493276533	-4.937082550883
23189	H	-0.813390188470	4.397319928355	-4.736308429283
23190	H	0.374721269135	4.753958640522	-5.673109320402
23191	O	-0.891487361647	0.849710105184	3.795999884555
23192	H	-0.987714302964	-0.132836752744	3.779135711970
23193	H	-1.478973544765	1.127712901055	4.534756677476
23194	O	1.116705967141	0.076900037323	5.802715756043
23195	H	0.736168122426	0.393066121832	4.971385629544
23196	H	0.345687082956	-0.175957764312	6.347586967196
23197	O	1.082079892989	-0.614758893432	-4.975189314522
23198	H	0.983539349781	-0.636121222274	-5.927057437119
23199	H	0.835442091027	-1.532181284135	-4.662020383450
23200	O	-1.922508730734	4.682155307203	-1.694579028355
23201	H	-2.416475854760	4.505372049800	-0.856121642920
23202	H	-2.246241590143	5.529272300356	-2.005561559707
23203	O	-4.337973225887	-3.754524712004	1.804660881810
23204	H	-4.320355268119	-2.814053979463	2.087579771534
23205	H	-5.253059210737	-3.950603118262	1.600458982371
23206	O	2.535512632524	2.883914178659	-2.810807326500
23207	H	1.947203889604	3.661417906573	-2.736955603526
23208	H	3.135211530081	2.965035060006	-2.051863738137
23209	O	4.499730024274	-0.542347174258	1.979734278273
23210	H	3.630062085375	-0.233388395741	2.317408420735
23211	H	5.005771041675	-0.820004189913	2.745260925797
23212	O	3.346956454478	0.242710717791	-3.689556364508
23213	H	2.584059005984	-0.039637833860	-4.225568445141
23214	H	3.176595917852	1.157851746442	-3.432905517141
23215	O	-5.390523390964	2.848884483952	1.367153430722
23216	H	-6.001187526561	3.340142819978	0.816725589867
23217	H	-5.367607169502	1.947461995426	1.001990439459
23218	O	-2.502623937086	4.066914808126	4.052527236010
23219	H	-2.607711032120	3.435129109292	3.310957176594
23220	H	-2.978413763991	4.857088098963	3.797269097394
23221	O	-2.127946576820	-2.770836766009	-3.200930224545
23222	H	-2.441937066566	-3.149645197278	-2.371190965747
23223	H	-2.648377005518	-1.970074369893	-3.352070106689
23224	O	2.289194362405	0.579245628510	3.007065316185
23225	H	2.815153395338	1.330841840859	3.408583080447
23226	H	1.689774505113	0.989370793145	2.378682827288
23227	O	-0.201005848453	-4.436529429531	-0.226160958486
23228	H	-0.155214367557	-5.384657892807	-0.360291655530
23229	H	0.222010251142	-4.260840986916	0.644502882693
23230	C	0.139551664287	1.605619529612	0.725334252483
23231	C	1.244797359160	1.456096766160	-0.049056268798
23232	C	1.956522893550	0.213562214096	-0.294139822019
23233	C	1.599108792974	-1.005275530166	0.200839403524
23234	C	-0.382108644064	-1.329623836199	-1.073415291958
23235	C	-1.304733852850	-0.600698720516	-0.351443449493
23236	C	-1.671464995326	0.738099561386	-0.804821496513
23237	O	-2.652784512557	1.409928141934	-0.383217529953
23238	H	1.581059231989	2.311703000044	-0.625124612289

```
H     -0.348017419996    2.564286563724    0.824425045040
H      2.744437908881    0.261708681627   -1.041698783047
H      2.148581146052   -1.893370720410   -0.074479113223
H      0.951426373181   -1.130063315865    1.058754578896
H     -0.002662430678   -0.954472368286   -2.009815781743
H     -0.255731863324   -2.383472665790   -0.894007261430
H     -0.198849533346    0.844524941174    1.416093144175
H     -1.164549100665    1.095683699432   -1.710555737217
H     -2.652249148007   -0.658691139393    1.237966641322
N     -1.805934808799   -1.053891808361    0.859107288122
H     -1.686140468427   -2.026421319881    1.100150357517

Ambimodal TS Water45-50
155
ANGSTROM
O      0.535300302165    1.418917494897   -3.164434609527
H     -0.320476868334    1.572185600196   -3.606342904930
H      0.772305443394    0.479279375887   -3.281748369294
O      0.180140435965    1.175743437374    4.428377172033
H      1.031032753490    0.877322768269    4.055810433312
H      0.096721877392    2.107923531012    4.186674712971
O     -0.734948381308    5.382219303083    1.118513799916
H     -1.072164650184    4.782426890946    0.406882893495
H     -0.051625290957    5.916174333808    0.712258889114
O      3.207712652638   -2.171020812102    2.688192222232
H      2.585197708042   -2.841293636240    2.290311724180
H      3.700549660544   -2.647871590045    3.356790841898
O     -4.723567740232    0.745079022885   -1.723277634424
H     -5.536666824596    0.818865167031   -1.161691972875
H     -4.002908727976    0.996865983306   -1.117446205400
O     -0.016135240944    3.843520874732    3.331868228205
H     -0.231176753211    4.495671126149    2.644553570004
H      0.959842201266    3.820420179120    3.373063121349
O     -6.494108753383    0.937589042862    0.214587639792
H     -6.045823761591    0.266141069123    0.755620749613
H     -6.096380925474    1.781928151392    0.487438152009
O      2.047786634435    2.374583887999   -5.153325369023
H      1.526410699899    2.076399130426   -4.372742488460
H      1.592685837427    3.167624441452   -5.453784139358
O      0.556806721267    4.059240663967   -2.337324726757
H      0.768983836865    3.117919136370   -2.422500277335
H     -0.279556691252    4.080224427011   -1.832593704934
O     -1.284716712935    0.147781982777   -6.111541778088
H     -1.836817034669   -0.473280381914   -5.628964891234
H     -1.476780372834    1.015474869341   -5.721071303819
O      1.692712069241   -4.002883055804    1.606080143237
H      1.952572176445   -4.063524371836    0.674906239519
H      0.721898889066   -3.902961999646    1.617316649417
O     -4.756421207018    2.977885752582    0.753603104062
H     -3.930309304653    2.475615472529    0.810499779697
H     -4.795588243866    3.518281222043    1.557160025674
O      5.078759677649   -4.071152571564   -0.103095820234
H      5.606845129638   -4.406852633277   -0.827695555517
H      4.144147213275   -4.188389453529   -0.364910072806
O     -4.163437329981    4.914531051740   -1.042303280355
H     -3.997975910412    5.639402184131   -0.403381802988
```

23295	H	-4.550228418511	4.204332993517	-0.488878181558
23296	O	-1.974282422148	-4.638126968639	-0.918248985419
23297	H	-2.147535942740	-3.878064198433	-1.512815778572
23298	H	-2.762938807555	-5.181428859893	-0.940570085269
23299	O	4.839158969842	-1.493365743461	0.630495447852
23300	H	5.087823551709	-2.404119211357	0.371002516487
23301	H	4.174087234652	-1.623803997735	1.336669385905
23302	O	2.570782580805	0.461320138122	3.320168736676
23303	H	2.736040370239	-0.476553583742	3.140331288067
23304	H	2.932462796102	0.948138924596	2.564937142899
23305	O	-2.853012796215	-0.188788432554	-3.525073204197
23306	H	-3.653366688084	0.076353814207	-3.048281965139
23307	H	-2.420141479328	0.637695579368	-3.768336182166
23308	O	6.698406499028	1.536506383985	-1.036324213518
23309	H	6.746742153037	1.260085795458	-0.107044977617
23310	H	6.408913349134	0.748326767424	-1.525305318944
23311	O	-0.180899201437	-3.290276166165	-4.279478831646
23312	H	0.334225483947	-2.472332104329	-4.053502226727
23313	H	-0.180097648867	-3.354474205990	-5.234959795174
23314	O	3.580493160370	-2.395391714022	-3.125897462298
23315	H	3.984646460046	-2.721972313673	-3.930090450663
23316	H	4.230882381094	-1.768413224251	-2.715038853299
23317	O	5.144332927322	-0.606695772174	-1.980848180460
23318	H	4.888638304236	-0.761143294003	-1.053819120119
23319	H	4.591559669601	0.125306870506	-2.331263461107
23320	O	-3.917633581299	-1.760949962878	3.948144300708
23321	H	-4.468665760342	-1.491605812823	4.683409854801
23322	H	-3.215436694459	-1.072565209165	3.861861289080
23323	O	4.557072944313	3.082695628034	-1.037715960394
23324	H	5.408896500772	2.570810270330	-1.067033375686
23325	H	4.788147839431	4.007469361819	-1.124204348049
23326	O	-2.052071222654	0.090974209930	3.526915422081
23327	H	-1.792801861810	-0.247380761525	2.647498776139
23328	H	-1.222076696605	0.377835145788	3.977012336480
23329	O	-4.356685950828	4.507160800269	3.028808250172
23330	H	-4.642430942498	4.609462574376	3.935782055731
23331	H	-3.605481694157	3.863242988679	3.031724447453
23332	O	-1.729587365442	2.290177403313	-4.402710518964
23333	H	-2.546485159872	2.652225570595	-3.989735025853
23334	H	-1.216231005284	3.066924954322	-4.703286267911
23335	O	0.438652570223	-5.153604177503	-2.277311856343
23336	H	-0.354162617905	-5.111319430748	-1.721487245246
23337	H	0.255498714501	-4.581048526817	-3.038633368919
23338	O	-4.601181287760	-2.037972599837	-1.064490371434
23339	H	-3.939742961812	-2.430203018647	-1.643981870393
23340	H	-4.772601622762	-1.151822826372	-1.421678565107
23341	O	-2.401881914167	-2.758012707273	-2.789538907720
23342	H	-1.673904368827	-3.052900845829	-3.365487126582
23343	H	-2.512584579514	-1.815740477459	-3.008825866431
23344	O	3.644275587043	1.362300013689	-2.980419911808
23345	H	3.800497070653	2.117361416447	-2.390647014090
23346	H	3.401918065275	1.716005585679	-3.843661937097
23347	O	-1.720995379237	3.933811576399	-0.871751405219
23348	H	-1.998905346617	3.029537959275	-0.621710597587
23349	H	-2.571738625104	4.396070381774	-1.063517150689
23350	O	-1.033660887099	-3.754637658350	1.512933330705

23351	H	-1.611749746882	-4.037724409136	2.245791714634
23352	H	-1.363774959282	-4.193790381879	0.711340137849
23353	O	-2.749687182389	-4.218218741309	3.565904866851
23354	H	-3.222954140672	-3.372075935009	3.672136064677
23355	H	-2.340238537331	-4.409643784580	4.410595418711
23356	O	1.249604401108	-1.127614202499	-3.786523602022
23357	H	1.404229241481	-0.798273540622	-4.716293113261
23358	H	2.101533123571	-1.488247796549	-3.480339536044
23359	O	2.681120027034	3.450578182345	3.496513077435
23360	H	2.713571061213	2.723066711045	4.124023345382
23361	H	3.141163630742	3.119072479025	2.706933469162
23362	O	6.299945197088	0.528233411466	1.506946860688
23363	H	6.813575747250	0.256146533901	2.267130438188
23364	H	5.807381467439	-0.268321656940	1.191083075313
23365	O	-0.053090369308	4.371064521634	-4.882421057965
23366	H	-0.397471098460	5.247382579780	-5.052442974978
23367	H	0.228879591651	4.357998715361	-3.928065408690
23368	O	-2.485105995001	2.686191288015	2.759992139283
23369	H	-1.625810307643	3.055561057035	3.013464559495
23370	H	-2.487504592202	1.762285869776	3.069617869048
23371	O	-4.763907653748	-0.880697136710	1.401005165727
23372	H	-4.674453427572	-1.381681556488	2.222703154185
23373	H	-4.793184861737	-1.511548203585	0.661075466075
23374	O	-3.398375521273	6.337390197323	1.120172409828
23375	H	-2.453832801661	6.138191834950	1.216191828758
23376	H	-3.834162738204	5.833638714487	1.824278207707
23377	O	3.980670424819	2.069716871329	1.474617359273
23378	H	4.838983053924	1.631105186936	1.590333682644
23379	H	4.004704214457	2.451225793755	0.580621025995
23380	O	-3.978229750765	3.038329871133	-3.176611127826
23381	H	-4.395983938552	2.288287199265	-2.720721357343
23382	H	-4.101190767691	3.806107989834	-2.603291747529
23383	O	2.543671337692	-4.011879003667	-1.099524665198
23384	H	1.819277644222	-4.549003729658	-1.491516388726
23385	H	2.830647719300	-3.423248755826	-1.816706559242
23386	O	1.448005278906	-0.113952892103	-6.177920849560
23387	H	0.492048772352	-0.026409817567	-6.348430771924
23388	H	1.771428090378	0.790291931960	-6.007457726212
23389	C	0.234705675706	1.575978927751	0.954017253062
23390	C	1.281910292971	1.514623642309	0.092265634774
23391	C	1.969034323329	0.300148785767	-0.291020903200
23392	C	1.684955531341	-0.946380485184	0.198580395724
23393	C	-0.295877063667	-1.417690967624	-0.806806314471
23394	C	-1.250193941300	-0.635895675835	-0.183449196045
23395	C	-1.538959987788	0.714331740362	-0.640346007919
23396	O	-2.508179114091	1.424118649737	-0.267786442019
23397	H	1.570494993713	2.414096248445	-0.449659005689
23398	H	-0.286131589673	2.500904013575	1.150530010303
23399	H	2.624684973774	0.388061686967	-1.152548796834
23400	H	2.206074033564	-1.815017680598	-0.176460229341
23401	H	1.198468451706	-1.091547213615	1.153692031378
23402	H	0.092052844486	-1.144313520960	-1.773212672261
23403	H	-0.195941553722	-2.460389466985	-0.542983330985
23404	H	-0.014741834343	0.764187150198	1.620915865063
23405	H	-0.953896735921	1.063529790086	-1.505274769615
23406	H	-2.869439519183	-0.744960970134	1.070572814825

```
N     -1.902474386389   -1.053237452171    0.988599801107
H     -1.763606001285   -2.031927919823    1.225040610491

Ambimodal TS Water45-51
155
ANGSTROM
O     -4.166778508354    0.187510085864    2.415297878984
H     -4.929736503330   -0.336147566861    2.062015928642
H     -4.331203649926    1.112655400966    2.185939893412
O     -1.774738507924   -2.849861772702    5.471533008459
H     -2.234955655159   -3.189185234278    6.238436767422
H     -1.405593768347   -1.968468318629    5.722782737743
O      5.350423736067    0.744865418440   -0.561237244634
H      5.173965500344   -0.212928962168   -0.682855482582
H      6.298087084957    0.847280081998   -0.463363616617
O     -2.859128547756   -1.341756656980   -5.125238171610
H     -3.434110654890   -0.676961407432   -4.723467945501
H     -1.994545504757   -0.915996955954   -5.217533997456
O      4.868453668565   -1.912108864357   -0.628356296548
H      4.635665648244   -1.903482850873    0.342482541153
H      5.568648761044   -2.558565258225   -0.724860009938
O      1.413199255023   -4.224939272979   -1.776587799070
H      1.884532716028   -3.410825107426   -2.053887355594
H      1.584761233679   -4.870863244930   -2.463072143930
O     -1.705785897156    2.010640799456   -4.086311456722
H     -2.545537473369    1.571336309669   -3.830327848826
H     -1.443283038454    2.564756446353   -3.333555295827
O      1.706365200146   -1.266485821755    4.797948798697
H      1.089909109773   -1.498051761648    4.051307994168
H      2.412401535113   -1.911892473954    4.736509991955
O      0.125983193668    2.284298978947    5.530414399435
H      0.689816736611    1.851916932929    4.871871572363
H     -0.263233807643    1.562701883053    6.041138039604
O      4.293748089059    2.997969604513   -1.848589258671
H      3.404986781002    2.904986900539   -2.226410879458
H      4.602117740273    2.111954583735   -1.607468979980
O      0.474850949597   -2.642293211197   -4.298251386537
H      0.229360229918   -1.791013461273   -4.678377944082
H      1.353714930616   -2.530915294232   -3.921487989690
O     -0.361596987823    0.055957514000   -5.141559804705
H     -0.215998633841    0.321322736597   -6.050693091127
H     -0.848975658337    0.816046069736   -4.708693736848
O      1.601169695205    5.326754330297   -0.137344533907
H      1.834059928880    6.254257786694   -0.175310360174
H      2.423748713496    4.842530800679    0.100692815364
O      4.416968600836   -1.725686718892    1.949206395377
H      3.681129388125   -2.228221867781    2.343178688822
H      4.230838541409   -0.781938406730    2.090580976988
O     -0.714921655021   -0.437223309681    5.912225948923
H     -1.113169986164   -0.117407205231    5.073168417547
H      0.196748093768   -0.689511800873    5.696476253550
O     -1.915103602844    4.579860464314    0.173986249644
H     -2.632366218317    4.087403682573    0.609610161724
H     -1.286879463025    4.812818988663    0.865711142180
O      1.914440262261    1.155714408751    3.621185552164
H      2.538464200742    1.036442193875    2.889991290038
```

23463	H	1.936902265826	0.311157107400	4.114217934406
23464	O	2.250665459297	3.818643303610	3.972944251185
23465	H	2.129245113791	2.860009152714	3.816363594857
23466	H	1.865886546742	3.981743572125	4.837451209088
23467	O	2.898353696296	-2.153099676332	-2.672683712159
23468	H	2.582417014428	-1.250977966310	-2.863799597119
23469	H	3.596388492981	-2.052629833029	-2.008366127639
23470	O	-1.454011503634	3.063126374249	3.457430821023
23471	H	-1.039886071784	2.996837000175	4.337492349600
23472	H	-0.866787891514	3.651899266951	2.950098282624
23473	O	0.307519406961	4.706011207728	2.168504790908
23474	H	0.726541502531	4.917076428378	1.310693451551
23475	H	1.044727002473	4.511076963070	2.772620474125
23476	O	2.566736293570	-3.459191256063	3.078954364780
23477	H	2.213005064961	-3.829299283291	2.235977380779
23478	H	3.119271260214	-4.156418222437	3.436543590460
23479	O	-0.021467649222	-4.511781716721	4.041304372233
23480	H	0.769649783136	-3.986771290311	3.861302899739
23481	H	-0.597896954375	-3.959655407298	4.592574960489
23482	O	-6.540633029649	-0.286244707393	-1.072699294675
23483	H	-5.979086288529	0.506617474655	-1.150302909558
23484	H	-6.346279404551	-0.849977192384	-1.824814804776
23485	O	-3.616163996946	-1.381434823309	-1.708261058931
23486	H	-2.896487245510	-1.882498515670	-2.136099484403
23487	H	-4.416325685149	-1.928166503544	-1.854640799744
23488	O	1.820575051744	0.237519297243	-3.430717853489
23489	H	1.084862741758	0.142095555257	-4.055115609336
23490	H	1.809025692939	1.165871194002	-3.150820769829
23491	O	-6.024604273590	-1.353910883190	1.366338055659
23492	H	-6.319846464053	-0.953751400812	0.525379482943
23493	H	-5.474585485461	-2.118234336150	1.121886652723
23494	O	1.673698647239	2.956232384460	-2.893800106238
23495	H	0.834125897657	3.231477759627	-2.465331038753
23496	H	1.732676305954	3.455203985266	-3.709938018164
23497	O	-5.618796857357	-3.175564927170	-1.990328696761
23498	H	-6.063923434207	-3.265026839141	-1.143594363852
23499	H	-5.018960363745	-3.937996339050	-2.054759555094
23500	O	-3.526145517445	2.807886229595	1.614021401632
23501	H	-2.930812738961	2.923794371337	2.370328982865
23502	H	-3.064179993674	2.221990539506	0.984810113360
23503	O	-3.302201960514	-4.753480984141	-1.539652335444
23504	H	-3.516031565965	-4.244368709444	-0.732096107566
23505	H	-2.839915588028	-4.148993757774	-2.148911730178
23506	O	-4.055629122903	-3.111784824653	0.520264784334
23507	H	-3.650812193769	-3.173098272639	1.402558479088
23508	H	-3.658087991219	-2.331599570335	0.096147743662
23509	O	4.092214279463	1.044275389372	1.922610421372
23510	H	4.522325050952	1.773685449328	2.422818355420
23511	H	4.499211685667	1.036308839551	1.039704664496
23512	O	-3.257525881899	-2.551626470399	3.089139125516
23513	H	-2.741818799165	-2.719433101854	3.890814346434
23514	H	-3.733568093801	-1.727780077470	3.219698346883
23515	O	-1.734988168904	0.361812154025	3.554097001008
23516	H	-2.650596084668	0.225744895204	3.253097924174
23517	H	-1.560839076173	1.314517080449	3.435363668712
23518	O	-1.159748452537	-3.776171743578	1.667138824102

```
H     -1.081357389957   -4.383525108523    0.905491410782
H     -0.973962619481   -4.299478066721    2.464065918305
O     -0.785002819814    3.801357199817   -2.122505121281
H     -1.182891358763    3.901767649790   -1.225983951682
H     -0.858104621840    4.677800064212   -2.504875701925
O      0.003516329486   -1.628642091064    2.886476625037
H     -0.713502016892   -0.992615733638    3.047171826502
H     -0.397675785186   -2.421623080359    2.491801177162
O     -1.993387579139   -2.965444473150   -3.160250588573
H     -2.420438021512   -2.580438671295   -3.954506102149
H     -1.050701527431   -3.011077938944   -3.396042813604
O     -4.940337162166    1.965208915982   -1.180550336304
H     -5.235673164224    2.616959403315   -0.542296954920
H     -4.022561907122    1.750228570497   -0.923670276656
O     -0.862360587584   -5.383027279134   -0.470461948856
H     -0.256621900133   -4.954655471264   -1.088788097541
H     -1.744872165860   -5.371979594932   -0.890379980083
O      1.788695437503   -4.775529743748    0.906401176058
H      0.974930258148   -5.259283627051    1.074547241861
H      1.737419178438   -4.514690837888   -0.029002270218
O      4.877567360631    3.357179995657    3.056592377070
H      4.797696483432    3.837087420353    2.219678010389
H      4.095553740596    3.623557266595    3.564550631628
O      3.727470874799    3.863180488055    0.579998182946
H      4.061173801297    3.636241142241   -0.322162194825
H      3.306471602175    3.060657038238    0.910561221175
O     -3.928490245653    0.643366859455   -3.404937482033
H     -3.775615606899   -0.055935242458   -2.723230851227
H     -4.512480452072    1.279632720038   -2.977257995413
C      0.254313877937    1.555918910898    0.868975915665
C      1.357054604331    1.450213341296    0.083980687653
C      2.077156232561    0.225569678064   -0.199744250478
C      1.734362042383   -1.003990947564    0.289230466936
C     -0.223249867250   -1.423876744359   -0.907278896299
C     -1.193633180519   -0.639011426885   -0.311050591742
C     -1.451138477922    0.724859344559   -0.779760373056
O     -2.412406747972    1.434180387071   -0.417114918830
H      1.659935284096    2.325519968103   -0.489364700183
H     -0.254388975904    2.501314671484    0.983252462612
H      2.827430766749    0.290834984184   -0.984738881379
H      2.268346093341   -1.892160891909   -0.016251529890
H      1.149175275029   -1.122772016828    1.188547219874
H      0.234903628612   -1.112039910485   -1.830528450411
H     -0.148630024642   -2.474704573666   -0.674497644826
H     -0.046173953823    0.782684290635    1.558968325203
H     -0.849699403284    1.062998840585   -1.643794306229
H     -2.643493612629   -0.480973420552    1.166048327466
N     -1.961446962260   -1.085435234792    0.735260475833
H     -1.806900995720   -1.993067903888    1.145411642597

Ambimodal TS Water45-52
155
ANGSTROM
O      5.040094382271   -0.286768646906   -3.968531329029
H      5.200618316371   -1.071972017901   -3.418512811463
H      5.348784960886    0.456691898596   -3.445324801432
```

23575	O	2.439935037993	2.455051971644	-2.972472376633
23576	H	3.327914714238	2.654311341545	-2.621346430219
23577	H	1.788469815611	2.865197732737	-2.384206138914
23578	O	-1.742984874834	0.618881092901	4.645344218701
23579	H	-2.266737926930	0.363244984030	5.404844672172
23580	H	-1.865998879480	1.597873311641	4.523374221099
23581	O	-2.585774086886	-4.716215086554	-1.859161008504
23582	H	-3.404686731164	-5.169395232220	-1.573277432388
23583	H	-2.896226885648	-3.839096122266	-2.162804624301
23584	O	-3.697139925949	-2.405103077416	3.446537208402
23585	H	-3.618738840688	-1.513282263018	3.059248334697
23586	H	-2.775672673911	-2.713519466860	3.529682245818
23587	O	-5.050733662409	0.510164641624	-0.480615745355
23588	H	-5.358597258951	1.026149963610	-1.263168296992
23589	H	-4.085028249048	0.646539592534	-0.490374609717
23590	O	5.061601200661	-2.017601701813	-1.755005218827
23591	H	4.138437169851	-2.234425246730	-1.990501047218
23592	H	5.063374884809	-1.121805944365	-1.364382736790
23593	O	4.251816382046	4.687504436201	-0.159897624104
23594	H	3.929454730961	4.272602867348	0.655338622700
23595	H	3.496302930458	5.182575002128	-0.520624234004
23596	O	1.555250495237	1.957137067805	3.724389858861
23597	H	1.095480669335	1.148072079843	3.431367120317
23598	H	0.888397357400	2.637149120814	3.894047783058
23599	O	-2.157355201351	3.134369042990	4.089354714372
23600	H	-1.353274022536	3.673484013564	4.052957760568
23601	H	-2.510000042952	3.125383715446	3.178743363772
23602	O	2.532879689355	-2.481173687712	-2.587281682428
23603	H	1.911283082531	-3.149639651488	-2.886167775578
23604	H	2.343812948737	-1.675062848730	-3.098993104094
23605	O	2.796800222769	3.509881456049	1.861030376385
23606	H	2.456352247163	2.955525528964	2.588028810554
23607	H	2.045216381440	4.065568775760	1.605020684088
23608	O	1.425746006939	3.373808012429	-5.346931824285
23609	H	1.537742827244	2.688558836060	-6.007679130736
23610	H	1.897042159766	3.063158979092	-4.549728733292
23611	O	0.373677511953	-0.548962779449	3.347803444905
23612	H	1.118257312054	-0.927839419114	3.851832268396
23613	H	-0.308353641599	-0.263900429733	3.977043343957
23614	O	-1.038987512549	-2.970386206252	3.067259143596
23615	H	-0.539162422670	-2.140591837213	2.999071089884
23616	H	-1.070176880924	-3.364862100326	2.187286864062
23617	O	5.294437489533	0.499279209513	-0.743442811361
23618	H	5.198087379930	1.360141129043	-1.179419159428
23619	H	5.165832116093	0.673102013533	0.200043520264
23620	O	1.106828412036	-5.054538803358	-0.188487227411
23621	H	1.821542055020	-4.651088337432	0.362325288795
23622	H	1.176706576665	-6.001317572674	-0.061648648848
23623	O	2.318579896596	-0.073328902090	-3.935762696200
23624	H	3.250133533853	-0.202629148001	-4.196817535380
23625	H	2.317855220688	0.793915050193	-3.490215767013
23626	O	-3.096310342559	3.038694725578	1.525647675392
23627	H	-2.844093611566	2.354846306075	0.878456889714
23628	H	-4.035452179943	2.871856328327	1.693182552259
23629	O	-5.319807296267	1.972770368675	-2.683903167650
23630	H	-4.681813155664	1.394083266938	-3.135268583755

23631	H	-4.833596272574	2.797290075458	-2.542152509735
23632	O	5.503248384581	-3.246017547024	0.543063402355
23633	H	6.415744119015	-3.531506323397	0.575676449153
23634	H	5.368594692465	-2.848298134896	-0.356380122852
23635	O	-4.132906827074	-3.619477524278	1.144248621631
23636	H	-4.148862617801	-3.240929494239	2.056099344073
23637	H	-3.203057355308	-3.820443821885	0.983164811461
23638	O	0.335017150408	3.816697022386	-1.801378861332
23639	H	-0.354563596195	4.014263455622	-1.142388473990
23640	H	-0.150613857590	3.586822532061	-2.627467826064
23641	O	1.954956960268	5.833090691136	-1.203572433792
23642	H	1.381945113223	5.096791613424	-1.527339908338
23643	H	1.928530227117	6.516093101885	-1.873547046381
23644	O	0.086156728711	-4.292473208418	-2.676597202888
23645	H	-0.800418018318	-4.667031903323	-2.573741799547
23646	H	0.559646561825	-4.554363635173	-1.870945427209
23647	O	-1.341827160963	-4.001604295598	0.397615286296
23648	H	-0.441302231808	-4.361630769249	0.309577148646
23649	H	-1.800146845427	-4.317567619257	-0.417419395330
23650	O	2.683627055319	-1.611528318820	4.364854169558
23651	H	3.145020764824	-1.604867067245	3.494816140531
23652	H	2.641374420361	-2.527528319706	4.642593180261
23653	O	3.875045415574	-1.493006797020	1.985944727948
23654	H	4.626544047197	-1.975955563300	1.608135747827
23655	H	4.045062784962	-0.545486592520	1.832477051955
23656	O	4.909953009670	2.950929289370	-1.975501249480
23657	H	4.703528384185	3.599510228572	-1.242788996971
23658	H	5.598974137795	3.358203399575	-2.501025990637
23659	O	-1.260166786995	-2.273327156223	-4.168203304124
23660	H	-0.670136670978	-2.935445253560	-3.781772554010
23661	H	-2.090121539950	-2.336834410394	-3.680401122136
23662	O	0.251112144972	4.437907295096	3.763037486645
23663	H	0.656936526213	5.069034281463	4.358046668200
23664	H	0.375606072754	4.784972229829	2.860764619548
23665	O	-3.166285736423	0.041536707198	2.349884666701
23666	H	-2.612375832589	0.296317091822	3.108551736791
23667	H	-3.935170075974	0.633110350437	2.334153010327
23668	O	2.987464231994	-4.066012933478	1.374681861988
23669	H	3.890504067080	-4.067615238084	1.021403626740
23670	H	2.843422596241	-3.149014013916	1.641695141601
23671	O	-1.703804339581	4.694273215233	-0.153218097220
23672	H	-2.259399784942	4.581336361600	-0.951901113712
23673	H	-2.216707514950	4.253547848393	0.546628913089
23674	O	-3.035955507626	3.604842356217	-2.230137102501
23675	H	-2.915144738549	2.795504481945	-1.693187682976
23676	H	-2.487428440825	3.471162350920	-3.015493912532
23677	O	-5.532942422503	-2.206396497958	-0.768390428459
23678	H	-5.512783140459	-1.249453447425	-0.590637047197
23679	H	-5.105027941043	-2.634500940860	-0.002159264038
23680	O	-0.419010680896	0.282588260812	-4.510580569367
23681	H	-0.733218873414	-0.642370946975	-4.483945526026
23682	H	0.545180853309	0.198916568087	-4.485709837768
23683	O	4.009795971924	0.939092432236	4.485006745116
23684	H	3.736520354595	0.032678087126	4.673443126157
23685	H	3.179220099228	1.431897964637	4.399745073306
23686	O	4.496936363708	1.164959420939	1.846485081703

```
H      4.530614618072    1.126725479481    2.827985501579
H      3.951940236910    1.937960582359    1.651994008072
O     -0.900445750548    2.967035741603   -4.014607264609
H     -0.235165444678    3.306132062145   -4.643492889234
H     -0.857986941957    2.000564701245   -4.129030334277
O     -3.427747982547   -2.176103029423   -2.297123126176
H     -4.346949852421   -2.247054694951   -1.927982092230
H     -2.922189109988   -1.850336019077   -1.526058247709
O     -3.181628438516    0.389797797626   -3.363747748281
H     -3.356377532605   -0.538453135997   -3.138715063653
H     -2.445268437221    0.392058134819   -3.981089078316
O      0.647993981453    5.248425080616    1.180469831831
H      1.179525228724    5.706407943660    0.512182097006
H     -0.217783694407    5.114582450123    0.758907545963
O     -5.413625591895    1.632511557591    1.874489406731
H     -6.293463635009    1.490173769442    2.221706888028
H     -5.394429337346    1.222148408008    0.970961480713
O     -5.022888854763   -5.291627940945   -0.846245798131
H     -4.833461906331   -4.886263797766    0.017018183535
H     -5.655872961838   -4.701777030025   -1.263801814286
C      0.083319822391    1.491773186810    0.895719635897
C      1.267387187722    1.462130673670    0.232383901404
C      2.008003528501    0.272872437302   -0.140396962755
C      1.624539597390   -1.006863797661    0.133396831833
C     -0.105247063177   -1.137324250282   -1.439154078281
C     -1.164008329621   -0.520801484165   -0.814594317157
C     -1.451082750696    0.890052320395   -1.014055879557
O     -2.489050191231    1.471592880634   -0.610510789391
H      1.658185127633    2.398410041067   -0.161965756436
H     -0.424366830850    2.427095288586    1.091663722155
H      2.845723548906    0.428688674375   -0.816414171958
H      2.186064416442   -1.848351981809   -0.245636930008
H      0.919937755728   -1.238028250790    0.920874011120
H      0.451543132716   -0.624297954166   -2.204636774657
H      0.006187626725   -2.211092672503   -1.420008533703
H     -0.317117167890    0.640590799777    1.426214986249
H     -0.791863092799    1.425699982862   -1.714079952301
H     -2.556146937734   -0.745079558006    0.710879502680
N     -2.065679034744   -1.251052018282   -0.019313649842
H     -1.746871313750   -2.167894129691    0.279078308038

Ambimodal TS Water45-53
155
ANGSTROM
O     -3.994932296160    1.067207296604    1.832973324125
H     -3.463138602282    1.175046429146    1.012245327289
H     -4.649637438608    1.767618107146    1.819435497061
O      3.306576482808   -0.482553646841   -3.522354875136
H      3.201648043684   -1.416092865661   -3.289859669468
H      2.418207839489   -0.096032146635   -3.485600034291
O     -2.383933180586   -5.382518899311    3.146588458623
H     -2.426508830809   -5.992969579316    3.882136909683
H     -1.775705001901   -5.782929356551    2.474159252040
O     -0.621845346933   -6.063865193061    1.279107057092
H     -1.064909889094   -5.419731954458    0.686380741834
H      0.169335316589   -5.597217299014    1.593226649440
```

23743	O	4.791092067581	1.643861869752	-4.493288913270
23744	H	4.325724755239	0.805936251746	-4.343585188733
23745	H	4.405098928763	2.040136870449	-5.275091327793
23746	O	-6.137187209570	0.439291010802	-1.026660752066
23747	H	-6.001779745540	1.214370477189	-0.466957760462
23748	H	-5.634754177585	-0.292526846031	-0.620302507145
23749	O	0.402784166884	4.653824030465	-1.144881624489
23750	H	-0.538468977082	4.597559843662	-1.351211260715
23751	H	0.445296280122	4.869210199220	-0.191903516585
23752	O	-1.959496478855	-1.722483366337	-5.063014011038
23753	H	-1.935232261263	-0.751943550687	-5.054931583987
23754	H	-1.043990299439	-2.006599030024	-4.948486890026
23755	O	4.769798919220	-2.503265880565	-0.680355997991
23756	H	5.143197771593	-1.705619965979	-1.158136437059
23757	H	5.480787204485	-3.143548833286	-0.639561416513
23758	O	-2.387350151647	-1.375250316909	4.663397596146
23759	H	-1.890891071544	-1.924116770738	4.013462931528
23760	H	-1.866452227684	-1.381436990765	5.470102088520
23761	O	-4.295234174983	0.653926004026	-2.938530484022
23762	H	-4.054264306044	-0.285201120990	-3.017251002173
23763	H	-5.046590909629	0.630528809152	-2.297821968541
23764	O	-0.960590161123	-3.035955226763	3.095435017133
23765	H	-1.498193971031	-3.844000748442	3.176409965606
23766	H	-0.321150375294	-3.040199589079	3.857001525005
23767	O	2.784741508533	-2.451946001526	3.297794701060
23768	H	2.316746130421	-3.006507012155	2.646106288849
23769	H	2.451937638098	-1.548512869518	3.190316051298
23770	O	2.561292222007	1.332201331904	5.510714147614
23771	H	2.724728638590	0.872084514928	4.658260155728
23772	H	3.321753038912	1.167476142172	6.067568942188
23773	O	0.933911205380	-4.655865753211	-1.098321871229
23774	H	0.130157223922	-4.465005662161	-1.619381156843
23775	H	0.886055306006	-5.581897924933	-0.851185853009
23776	O	-4.531968922746	3.383281363575	-3.002373607807
23777	H	-4.444206661622	2.414741196792	-3.076941559814
23778	H	-4.636783366737	3.716190974207	-3.893981173554
23779	O	4.015523725033	2.756255626891	-2.135949690559
23780	H	4.335017035484	2.235152025327	-1.383246203050
23781	H	4.501980397614	2.450937429562	-2.923494346585
23782	O	-1.110551079303	-3.946295163416	-2.788102156166
23783	H	-0.565143647116	-3.248586061019	-3.207759389254
23784	H	-1.504875340367	-4.442347784041	-3.506114135152
23785	O	0.670132611713	0.472176512950	-3.479933548825
23786	H	-0.222566923753	0.587889865137	-3.857864795971
23787	H	0.975702617687	1.388221299329	-3.300533508672
23788	O	3.272714389412	4.913883370248	-0.544219275713
23789	H	3.662788148089	4.318120103745	-1.202475342760
23790	H	2.366160115150	5.070591751638	-0.828574024732
23791	O	4.652171661450	-1.058989467276	1.700787412225
23792	H	4.644649406874	-1.587033414376	0.883267107956
23793	H	4.308654514264	-1.655731544531	2.383885401744
23794	O	2.775452241565	-3.083277095538	-2.481709895374
23795	H	3.497330151067	-2.913453967222	-1.846902817958
23796	H	2.173999942976	-3.678170324531	-2.005420153954
23797	O	-3.997977846220	-3.651976087407	1.533847467475
23798	H	-4.237311307732	-2.873642544184	2.085963642796

23799	H	-3.693591081555	-4.322723373284	2.159352367298
23800	O	4.904851862366	1.333428424596	0.150384490930
23801	H	4.608891770546	2.083815501494	0.709932826086
23802	H	4.933708850433	0.571428003759	0.743741097573
23803	O	-2.898695151291	4.249377636676	1.196175692574
23804	H	-3.739713990816	3.869584789782	0.902645918791
23805	H	-2.458645840053	4.546831264048	0.392633538339
23806	O	0.717503929382	-2.757778889936	5.089376323429
23807	H	1.589942108540	-2.788006039851	4.657484876809
23808	H	0.567970482838	-1.818698330460	5.313182342160
23809	O	-4.380894421994	-1.522460207597	-0.380495767728
23810	H	-4.469906003427	-2.315888406166	0.158208308168
23811	H	-4.057896568494	-1.798063760067	-1.248165185276
23812	O	1.466420919087	3.005140070958	-3.047291293942
23813	H	1.093526571912	3.581387526248	-2.354011240956
23814	H	2.410952654361	2.922307073507	-2.815519190026
23815	O	0.613648795807	-2.264224955155	-4.060043576733
23816	H	0.681376239869	-1.310683289545	-3.892607984740
23817	H	1.427093043526	-2.647964493182	-3.698051222301
23818	O	-1.086685637820	3.486676637235	-4.165500563876
23819	H	-1.427560801610	3.737536810609	-3.289525680752
23820	H	-0.126058166383	3.451374422864	-4.068924077410
23821	O	-4.583971544865	-1.459000588784	2.971177736747
23822	H	-3.974262239593	-1.425861857829	3.721233551361
23823	H	-4.520378261259	-0.595880337203	2.537246727376
23824	O	3.824760057089	3.316829747155	1.596328878169
23825	H	3.639404148273	3.983267191585	0.906289080917
23826	H	3.025087582711	3.233949289458	2.133119546772
23827	O	-1.818792102545	-4.041461257083	-0.014729725733
23828	H	-1.984229069829	-3.921591038776	-0.954836122432
23829	H	-2.654198515422	-3.874167673561	0.465031918241
23830	O	5.613621536092	-0.373779132976	-1.903265753706
23831	H	4.908001372608	-0.230234729963	-2.552105684059
23832	H	5.544588566894	0.339599958557	-1.249894311952
23833	O	-1.073513616624	3.631259468349	3.204248381669
23834	H	-1.777492397353	3.912980396378	2.595068467955
23835	H	-1.345069433716	2.740288592916	3.486914358959
23836	O	-1.865269016200	0.957701470378	-4.374226646886
23837	H	-1.645449468411	1.913613857888	-4.466408882400
23838	H	-2.667929059008	0.925536280144	-3.835086577682
23839	O	1.149796799802	-3.917565692678	1.622773735671
23840	H	1.099583993755	-3.928562627777	0.656151065697
23841	H	0.328642719043	-3.503168091741	1.956872693173
23842	O	-2.238913665225	3.889027127079	-1.635205456913
23843	H	-2.253258979125	3.024305595154	-1.178658396897
23844	H	-3.091370478476	3.930524003305	-2.105293666316
23845	O	-1.857212387998	1.045896102928	3.572679006466
23846	H	-2.582314698806	1.148704177983	2.934583434446
23847	H	-2.099076754422	0.235354290589	4.059422716533
23848	O	-4.942354872522	2.885504382444	-0.225630993191
23849	H	-4.189924492712	2.332843899777	-0.490811820463
23850	H	-5.209448199101	3.347743485742	-1.027221085656
23851	O	-3.692106969247	-2.061911726705	-3.078980836769
23852	H	-2.989629278618	-2.010611037160	-3.772525713167
23853	H	-4.448967784731	-2.471536133835	-3.500873341297
23854	O	1.596133502572	2.909914295249	3.266763432184

```
H      0.652338575344    3.148424074109    3.251573444243
H      1.828850588177    2.769902252368    4.192159934338
O      0.194723504203   -0.096637175029    5.443174334803
H      0.980598352466    0.434401303070    5.647927297503
H     -0.258225177766    0.372158775214    4.733171942088
O      0.573719190669    5.226767181424    1.488088191448
H      1.470466127223    5.202872446997    1.828167564735
H      0.008668869637    4.801906005941    2.148471252284
O      2.589617389417    0.338277505286    3.018932086892
H      2.180077307237    1.180197514336    2.759249331147
H      3.384854673249    0.220782669866    2.479994778397
C      0.168925844193    1.662895250951    0.815947424445
C      1.265641120775    1.531951661792    0.031720745871
C      1.991118851450    0.298114616316   -0.234584814180
C      1.708177157425   -0.911069928441    0.314115937275
C     -0.328580137468   -1.376696284427   -0.890815445922
C     -1.275330771929   -0.624517353608   -0.233730567481
C     -1.596181387522    0.701305864589   -0.754894398172
O     -2.544588218639    1.451276504932   -0.412864690915
H      1.597630195031    2.397095676672   -0.536958349930
H     -0.338399152015    2.613331016701    0.904859308308
H      2.732251327559    0.352212837064   -1.028141849238
H      2.262701032222   -1.795699470921    0.033782120554
H      1.088689024593   -1.014377616259    1.194508210390
H      0.090878913718   -1.033835318330   -1.819069926429
H     -0.164098842732   -2.410423633347   -0.640312386672
H     -0.166411060824    0.909708276700    1.515466647208
H     -1.034832402020    0.991728450150   -1.658508379898
H     -2.739739229051   -0.681088780910    1.217025153997
N     -1.831091845075   -1.032701709918    0.969156693800
H     -1.674599808452   -1.985850312291    1.257649167891

Ambimodal TS Water45-54
155
ANGSTROM
O      4.785956812275   -0.122831593038   -2.469584083813
H      4.565105754237    0.826386167617   -2.511796038980
H      4.752891802593   -0.449118477932   -3.376183626725
O      3.885920241032   -1.612054485558    3.428713058802
H      3.995241195653   -2.054650726000    2.575396094519
H      3.425833866495   -0.778783848688    3.260700590955
O      1.123762805046   -4.783036034283    3.945431658019
H      1.801863896745   -4.910238785943    3.260729772216
H      0.326354702342   -4.547732139289    3.444633735055
O      2.193140248684    0.726283812087    3.430031588943
H      2.123389675819    0.609080153827    4.417885683212
H      1.482551483850    0.127748303646    3.101649443266
O      0.537894165097   -4.562725690235   -0.340266281384
H      1.054762980943   -4.288466590197   -1.126276781452
H     -0.370723151644   -4.660749376727   -0.661469504912
O      1.828356657893   -3.456280267164   -5.277566478491
H      1.016819869665   -2.947878895200   -5.416575624365
H      1.765804674224   -3.789522690509   -4.366873436741
O      3.925955745565   -1.934316166855   -4.584775047803
H      4.376524387922   -2.570191747080   -4.019420046437
H      3.256535739138   -2.468082267310   -5.070938594401
```

23911	O	-4.766672777604	-0.754381801104	1.432223643822
23912	H	-5.327112388629	-0.427993477486	0.724823785482
23913	H	-4.576268434808	-1.685742989758	1.227811883223
23914	O	-4.114475629869	0.817108616363	3.599863009901
23915	H	-4.594479497317	0.159496879437	3.075303989253
23916	H	-4.046527272180	1.582327453977	2.993337463787
23917	O	-2.569099820657	2.102374057502	5.633815470797
23918	H	-3.299274108782	1.665738202932	5.173852544613
23919	H	-2.047425283871	1.397542915752	6.049987095861
23920	O	1.867814671441	-3.556671794110	-2.473883878400
23921	H	1.585845384418	-2.635307850393	-2.564402201525
23922	H	2.819559483143	-3.491682704783	-2.253078150599
23923	O	-5.715391370213	3.096442950231	-0.533108561842
23924	H	-5.768223678884	2.193305513782	-0.876496605668
23925	H	-5.346036513895	3.008000242261	0.363685937859
23926	O	-2.070310705883	-0.859458352151	4.011234217848
23927	H	-2.750351101449	-0.178788459616	3.814235052567
23928	H	-2.486972055030	-1.726296158938	3.807303908406
23929	O	1.612524683107	4.671717792242	0.938922150195
23930	H	0.723631842058	4.814416709021	0.595365436938
23931	H	1.507996005875	4.239522230829	1.800272619465
23932	O	1.458839922952	3.297573535782	3.433906493794
23933	H	1.734700841537	2.354622205092	3.356716176111
23934	H	1.890484664315	3.647879457359	4.214078544529
23935	O	4.251915741836	1.519155807582	1.836474503953
23936	H	4.932326142705	1.719779547755	2.482031920656
23937	H	3.464427142638	1.238985508722	2.342089345737
23938	O	2.594515144824	-4.687488947113	1.613743691441
23939	H	1.939975977719	-4.794067935674	0.910641092528
23940	H	3.225601843835	-4.024952250586	1.315142244129
23941	O	-1.038121667163	-0.124927601401	6.317803975985
23942	H	-1.210473403487	-0.791552708508	6.983248369762
23943	H	-1.400516793059	-0.478169181321	5.466277753290
23944	O	-2.381392717801	-2.000857644840	-3.066965309336
23945	H	-1.649346613129	-2.046627205231	-3.718726912391
23946	H	-2.662843059324	-1.072241721748	-3.103340123734
23947	O	1.683175732359	0.160786864258	5.930178435546
23948	H	0.727036273588	0.227943810931	6.106728886422
23949	H	1.853671612538	-0.792557895453	5.877888842093
23950	O	-1.183334731868	4.797570673365	-0.140813402534
23951	H	-1.989303880994	4.636747315057	-0.668753576406
23952	H	-1.520749625148	4.860592166864	0.769733224597
23953	O	-4.943007357680	-2.280000081130	-1.778528484235
23954	H	-5.080236666470	-1.331885182813	-1.611503529975
23955	H	-4.167727055550	-2.334857022522	-2.352073973062
23956	O	3.888838441125	3.531120359327	-0.101528500976
23957	H	4.022858221793	2.840900170276	0.565837463148
23958	H	3.102060502555	4.027914242145	0.190868864307
23959	O	-4.033974241885	-3.278070348705	0.543148832249
23960	H	-3.207595185330	-3.632637056702	0.180097216547
23961	H	-4.546197462888	-3.042293656290	-0.258533217820
23962	O	-3.006868068692	-3.236830195996	3.213373177984
23963	H	-2.155998229217	-3.578636269270	2.896112532208
23964	H	-3.605428324398	-3.305393066037	2.459321501101
23965	O	-3.307324768845	0.577037501024	-3.577179841446
23966	H	-3.582504427629	0.453588690473	-4.487168555701

23967	H	-2.759869566315	1.394382493304	-3.571252670589
23968	O	0.217265013396	3.819974434600	-2.231274573142
23969	H	0.577089923119	4.643462390636	-2.565282629966
23970	H	-0.222963620342	4.051544278004	-1.388935133210
23971	O	-1.235094627507	2.675280277447	3.405186067349
23972	H	-0.303572604894	2.891475658340	3.557117307393
23973	H	-1.663524749249	2.502792899428	4.274452833959
23974	O	-3.303137853231	3.746989621045	-1.501595156721
23975	H	-4.256243466063	3.760753952333	-1.273046751456
23976	H	-2.984101566295	2.910725399829	-1.099762338965
23977	O	-4.028394115876	2.658938559337	1.611754358754
23978	H	-3.418843994616	2.162668873266	1.042280510805
23979	H	-3.563477606737	3.484839206770	1.832524056528
23980	O	-0.318712133769	-1.633692853146	-4.775978698928
23981	H	-0.536550079068	-0.788503743463	-5.200743646231
23982	H	0.431128560155	-1.400226442111	-4.186238625129
23983	O	0.402688016044	-1.217313645815	3.092844453191
23984	H	0.842057313819	-1.676428818345	3.853612216759
23985	H	-0.501253817565	-0.986027240497	3.391451879627
23986	O	4.384041861361	-2.893254895399	-1.804639724833
23987	H	4.511311581836	-3.042329374321	-0.854570401694
23988	H	4.468158973443	-1.937223404957	-1.931042646018
23989	O	1.808075945248	-2.439687177674	4.973963950223
23990	H	1.561522291839	-3.373909529729	4.772183054198
23991	H	2.678582238069	-2.308881563219	4.551213469489
23992	O	-2.068507987421	-4.094433697028	-1.302523343883
23993	H	-2.096830584098	-3.302723246420	-1.874796651780
23994	H	-2.546260555904	-4.777882166679	-1.774981676520
23995	O	4.685568264163	-2.761540038198	0.957326984114
23996	H	5.442092167939	-3.237092395591	1.305179251763
23997	H	5.015374731583	-1.848577245851	0.725983615671
23998	O	1.785358816535	-0.867673855291	-3.288629180765
23999	H	2.597419076609	-1.133303028424	-3.765657123420
24000	H	1.744233998994	0.104898629427	-3.336932284420
24001	O	-2.365195090931	4.715837999584	2.318058998963
24002	H	-2.589440695437	5.339383640978	3.007964863806
24003	H	-1.898323164170	3.944523289672	2.761486493849
24004	O	-4.908603731769	0.476740888654	-1.336291738861
24005	H	-4.152601827265	0.719277913907	-0.773422192874
24006	H	-4.566849775907	0.592225549772	-2.238469883321
24007	O	-0.465053248083	1.081660271342	-5.280427428414
24008	H	0.381524087164	1.320535370959	-4.880191139791
24009	H	-1.085146296246	1.759368321645	-4.989565275704
24010	O	1.668326051590	1.838505424786	-3.510121606543
24011	H	1.106331452300	2.423933038864	-2.980012635594
24012	H	2.578051415204	2.096049434354	-3.280982398841
24013	O	-1.982574591729	2.914763199253	-3.659628259419
24014	H	-2.634450143579	3.360949866704	-3.080840575370
24015	H	-1.131396217645	3.154374963638	-3.253331440117
24016	O	4.123160833692	2.537849241514	-2.512925995711
24017	H	4.761975128101	3.112731213810	-2.935493414308
24018	H	3.999731429880	2.903006110428	-1.593319514286
24019	O	-0.534565791119	-3.572332073247	2.058634403829
24020	H	-0.092338100319	-2.733829428674	2.298289749473
24021	H	-0.088914170237	-3.921946625410	1.272461183935
24022	O	5.497984743047	-0.384482305945	0.243019099147

```
H      5.022731695543    0.317898423013    0.717896992251
H      5.351322546970   -0.235503099026   -0.702065371863
C      0.176298082303    1.599464507877    0.833973054813
C      1.276293169198    1.526383906199    0.039845362952
C      2.060580074391    0.336454233375   -0.240743729374
C      1.819179205754   -0.903980852702    0.261284650240
C     -0.274563093665   -1.358436015909   -0.887555974622
C     -1.226586621548   -0.603642663320   -0.242454016237
C     -1.531450115198    0.746686617649   -0.715003738183
O     -2.535932655290    1.414120997702   -0.360066209724
H      1.547136350530    2.405264281494   -0.542067037253
H     -0.368132508346    2.524919172524    0.953634357687
H      2.804470969225    0.444501653581   -1.026759873752
H      2.396042437092   -1.759184641524   -0.062450699675
H      1.216577752985   -1.070207917288    1.139264535363
H      0.159142453240   -1.014163807817   -1.807859659771
H     -0.137277873864   -2.398796421089   -0.647698335059
H     -0.101367592104    0.830606416687    1.539783044759
H     -0.967575731400    1.100153335690   -1.595796244228
H     -2.708114482535   -0.550544572255    1.203825531955
N     -1.878777001982   -1.033176701738    0.893559658735
H     -1.804348617189   -1.996828643649    1.173224863550

Ambimodal TS Water45-55
155
ANGSTROM
O      0.334172389991    2.458318029854   -2.985753898038
H      1.054640991704    3.098183345762   -2.858726721625
H      0.778643148274    1.604912223533   -3.134355918067
O     -5.154157517659    1.090449420438   -0.195242028344
H     -4.187210105100    1.102535938356   -0.331660785504
H     -5.522929260022    0.576974345528   -0.929173748930
O      4.855524564330    1.928620605264    0.484617991271
H      4.031543382359    2.082490458552    0.957162157099
H      4.990213300671    0.972854010794    0.454296567536
O     -1.675180641814   -5.076994932939   -0.800774169005
H     -1.569614168137   -4.430942811065   -1.532547417765
H     -2.470669396954   -5.560832464932   -1.042129464491
O     -2.099076678780   -0.799182951419    4.135979447839
H     -2.732273545694   -1.395118659878    3.729052537454
H     -2.411606961972    0.108735262123    3.921200564214
O     -2.973627946555    1.667219246781    3.586971308212
H     -2.813677327482    2.232810010022    2.810106608853
H     -2.627589523977    2.156630854226    4.353389683019
O     -2.598590108665    3.325477958875    1.418428702400
H     -2.676615535042    2.626186292275    0.745936600141
H     -1.994464125256    3.976374213447    1.025404042601
O     -4.446662884918   -0.190104029073    2.084630924604
H     -4.207994458195    0.494831757872    2.721027336412
H     -4.871541635049    0.281266555502    1.341174735059
O     -1.677798052330    2.931154435836    5.630710307113
H     -1.933869102703    2.837025607994    6.548305231589
H     -0.780462567322    2.532539868549    5.554139828477
O      2.937311371871   -2.151478475393   -2.612252110045
H      2.293486638791   -2.802597324601   -2.967421577962
H      3.209847166396   -2.540308150444   -1.770198004481
```

24079	O	-1.627289733985	-3.563599263082	-3.024277970505
24080	H	-2.421370111553	-4.038671436665	-3.298295485386
24081	H	-1.867243048885	-2.622732253587	-2.998790655791
24082	O	-2.931993099950	4.646249084646	-4.155077269834
24083	H	-3.829359748159	4.458318807407	-3.866721769443
24084	H	-2.409412844005	4.657322319892	-3.334681999754
24085	O	-0.255698780282	-0.611724013893	6.283976886100
24086	H	-0.993707575235	-0.779151877428	5.678271197032
24087	H	0.409384633399	-1.288551739251	6.119696662538
24088	O	-1.454941169551	4.835665864131	3.628798649370
24089	H	-1.951685967040	4.392206629673	2.930238633201
24090	H	-1.680300336672	4.370771546174	4.447548166361
24091	O	-0.670370304437	5.132683153398	0.554072470123
24092	H	-0.822301476284	5.882544295718	1.131493304408
24093	H	0.310548561386	5.092452005474	0.417954465694
24094	O	-1.751885993107	2.291149697190	-4.807928451489
24095	H	-1.015807300393	2.384197367998	-4.177094150743
24096	H	-2.158211060234	3.179709820717	-4.836748233275
24097	O	-2.245149510972	-3.858854338823	1.592626391184
24098	H	-3.193611397987	-3.603789089226	1.580281347548
24099	H	-2.093153694956	-4.343371345031	0.761139195343
24100	O	2.518108961503	4.010874421626	-2.385436118240
24101	H	2.809326690730	4.727020226833	-2.950499800409
24102	H	3.285540770209	3.385202567645	-2.305018162069
24103	O	1.115673770218	-4.055613513681	-2.936414411716
24104	H	0.205164890468	-3.853170349121	-3.195462348996
24105	H	1.068595015199	-4.320264797939	-2.007942665844
24106	O	-2.424405706404	-0.920680591651	-2.954922384838
24107	H	-3.032799988749	-0.168201117005	-3.095356798349
24108	H	-1.754823333347	-0.788886702455	-3.670270942613
24109	O	0.365864732938	-0.974923449934	3.166212260761
24110	H	-0.560157460988	-0.872599470975	3.475058328685
24111	H	0.882160403186	-1.314000781553	3.922505255325
24112	O	4.296359656004	-0.918993898083	2.840677632121
24113	H	3.642453242762	-0.233680913940	3.066316746236
24114	H	3.827797499181	-1.770676472783	2.846556262886
24115	O	-1.661057856769	4.055051407489	-1.778918607684
24116	H	-1.291159217184	4.533126762616	-1.021352974855
24117	H	-0.989840986524	3.419503072242	-2.067802353720
24118	O	2.831030757763	3.268807188893	2.179317730020
24119	H	2.154030531829	3.676748703108	2.789354458588
24120	H	3.672801703914	3.634267291736	2.461989937795
24121	O	-5.909141456782	-0.242579500533	-2.557353600789
24122	H	-6.746858430254	-0.034464204571	-2.970806771010
24123	H	-5.224354874990	0.256746947982	-3.049190861268
24124	O	2.109425381946	-1.901442083827	5.034537000124
24125	H	2.516220108838	-2.615800084192	4.527742975905
24126	H	2.772688577768	-1.207774910050	5.103013773693
24127	O	0.735590536813	1.783367515080	5.516006865862
24128	H	0.424106770140	0.952708553121	5.931459107476
24129	H	1.363772469093	1.494177171807	4.827305438784
24130	O	4.591725734522	2.422312384068	-2.152866542706
24131	H	4.800314260049	2.455430836343	-1.198798640473
24132	H	4.673929382305	1.472792055133	-2.364279263420
24133	O	1.159056894430	4.219469122197	3.930258284033
24134	H	0.255929105001	4.537389669434	3.720993739874

24135	H	1.060190698025	3.583034355749	4.647223332092
24136	O	1.935999989969	4.995527341749	0.150457020532
24137	H	2.138652821182	4.671955874148	-0.743893351466
24138	H	2.297316856387	4.345711019498	0.774790911863
24139	O	0.984220150137	-4.659108612092	-0.102832865301
24140	H	0.168829270924	-5.132065282859	-0.322546162753
24141	H	0.782330372315	-4.226992753013	0.755288209228
24142	O	-0.706481242176	-0.365616082551	-4.889770386208
24143	H	-1.038383521890	0.497733689123	-5.167631986038
24144	H	0.183531107733	-0.214381722838	-4.524575734691
24145	O	3.947454789716	1.138028539956	-4.782336252791
24146	H	4.573991003883	0.505939032239	-4.411481952450
24147	H	4.164886056915	1.983350226813	-4.379151419381
24148	O	4.954634926975	-0.277069789300	-2.493280628648
24149	H	4.274530954308	-0.945690379179	-2.676052677256
24150	H	5.246562008088	-0.455074375203	-1.588325529818
24151	O	5.041495519309	-1.010529696378	0.266183378317
24152	H	4.706959204662	-1.887230321589	0.055716177878
24153	H	4.905443528026	-0.927315216604	1.235978687030
24154	O	-3.915513703629	1.253987805629	-3.548661965083
24155	H	-3.191657843672	1.560094444362	-4.135495044162
24156	H	-4.071540194692	2.011432505047	-2.935969170387
24157	O	3.545603476500	-3.740196461778	-0.234729142659
24158	H	4.138186463042	-4.435463744341	-0.526064592342
24159	H	2.648072012763	-4.128827064605	-0.250102083188
24160	O	-4.317546842590	3.291406210648	-1.874758340864
24161	H	-4.704586144012	2.876088471712	-1.093575074477
24162	H	-3.393114952776	3.506090442147	-1.651751852275
24163	O	-4.073461724247	-4.771618025827	-2.376697902931
24164	H	-4.216145661233	-3.884685685775	-1.984616111873
24165	H	-4.941997596754	-5.130347874653	-2.556011430441
24166	O	-4.321202745041	-2.217858399031	-1.469253663799
24167	H	-5.053990666644	-1.676094430872	-1.799615027419
24168	H	-3.526715446093	-1.800470182468	-1.845933738438
24169	O	1.687929651000	0.153226236123	-3.624538861366
24170	H	2.414912632826	0.514070416028	-4.170460201907
24171	H	2.051846340973	-0.644521858297	-3.207900223384
24172	O	3.020814315232	-3.375749937046	2.656644355014
24173	H	3.371875717556	-3.560864193305	1.776243749020
24174	H	2.068570144300	-3.557714037830	2.590726524668
24175	O	-4.669909253052	-2.814450309732	1.238392050032
24176	H	-4.705539649858	-1.944539958488	1.663617180606
24177	H	-4.596040322762	-2.629759887060	0.287820016403
24178	O	2.322764494671	0.894423199104	3.450911561002
24179	H	2.458569885242	1.681077175325	2.892302944085
24180	H	1.571126651239	0.393990790132	3.084251978370
24181	O	0.321600583997	-3.516952330093	2.251851700266
24182	H	0.285433986204	-2.554411988706	2.428156769056
24183	H	-0.610229834203	-3.796020428891	2.226590229896
24184	C	0.267970863018	1.667179017600	0.839153269436
24185	C	1.348325169215	1.539443847865	0.024623431689
24186	C	2.060889444919	0.313508424514	-0.250994571122
24187	C	1.717653255800	-0.905834126311	0.278261023059
24188	C	-0.175006759490	-1.431520005995	-0.799482171714
24189	C	-1.173160513323	-0.658956835843	-0.219868188550
24190	C	-1.499025780246	0.665441157550	-0.752885123615

```
O     -2.497732277365    1.339465769927   -0.419024360568
H      1.655444552677    2.402757847404   -0.561208155239
H     -0.227918692249    2.618359540484    0.953700603014
H      2.790306370573    0.353509876413   -1.054888633963
H      2.270826636012   -1.793977365383    0.018420498830
H      1.173190471255   -0.977380569740    1.205502094890
H      0.209876490534   -1.163252568551   -1.769346060664
H     -0.081822610718   -2.475072245159   -0.537204352462
H     -0.053114342757    0.901966495550    1.530220909727
H     -0.909349376611    1.004358846408   -1.617538797066
H     -2.661034250988   -0.544409094542    1.206053685840
N     -1.836552811029   -1.058959610388    0.922911645651
H     -1.842435185118   -2.039646143409    1.176517730669

Ambimodal TS Water45-56
155
ANGSTROM
O      2.070615213783    3.587942229617    2.249442220898
H      1.531862287478    4.067340754890    1.574102006324
H      2.547712535415    4.265160671173    2.734096321439
O     -2.672019089303    3.924238372948   -0.738891423737
H     -2.651076558401    2.970094254608   -0.476579703479
H     -2.582258016614    4.415081698959    0.093572269863
O      5.772967747067   -0.795106128221   -1.379480176852
H      5.504622441827   -1.733505465958   -1.277005245020
H      5.285329064083   -0.449897160820   -2.140081411941
O      4.874931299189   -3.296378978417   -0.951598728301
H      4.808254277613   -3.447738471828    0.002499207095
H      3.983248358573   -3.452321095274   -1.304274200540
O     -3.552737622307    0.608744468852   -3.340394188048
H     -3.572285217884    0.203910507705   -2.461874801327
H     -3.866270539589    1.529402208524   -3.214671275512
O     -3.361901364265    3.012196378689    2.993256770936
H     -4.029819386154    2.578987443480    2.429390148654
H     -2.867471357315    2.293677057744    3.411046689141
O     -4.872892063341    1.493907167562    1.281982218229
H     -4.037126622052    1.382119044525    0.784609090439
H     -5.140989398609    0.606948158899    1.551974408214
O     -4.855213396351   -3.914229688358    0.199576591478
H     -5.620044114881   -4.487829553318    0.151874180179
H     -4.652790503670   -3.636189449235   -0.714110147163
O     -4.886692501461   -1.391796888328    1.519290496980
H     -5.008206490545   -2.315467136610    1.260710293724
H     -4.422756454223   -1.415057921903    2.383541841218
O      5.545734024394    0.410640199014    0.903074048685
H      6.459594085891    0.561920715214    1.147195395128
H      5.581983235004   -0.038709964835    0.008014771346
O      4.053895284194    2.862733341236   -1.955852066268
H      4.110067032289    2.855741245077   -0.964087945624
H      4.782457845989    3.406040553333   -2.257602798047
O      0.612600564431   -1.153291778403    5.880122839999
H      0.996421324251   -0.277236654379    6.110614834371
H      1.363389174176   -1.738719801623    5.672592086136
O     -4.033971883981   -1.817728987270   -4.644792280009
H     -4.094718060561   -0.909501653206   -4.309654811147
H     -3.177588399868   -1.861267372142   -5.100782107967
```

24247	O	0.028827649738	-3.232445164438	2.531876750680
24248	H	-0.797308784362	-3.549202546135	2.135981020211
24249	H	0.711676110189	-3.607674193373	1.929470506391
24250	O	-2.133995161031	-3.906368890590	0.919514320406
24251	H	-1.824526785651	-3.957485201254	-0.000772407598
24252	H	-3.094614337210	-4.007423761232	0.880690679262
24253	O	0.521932751135	4.963570616581	0.561326908791
24254	H	-0.355585673837	4.986553504798	0.980057079921
24255	H	0.364850800791	4.840322380501	-0.382166972822
24256	O	-1.503978444574	-2.777930937650	4.912679858270
24257	H	-0.910989733934	-2.298319082208	5.505500905424
24258	H	-0.943625852273	-3.165444656823	4.228404216174
24259	O	0.123824724449	-0.620728710268	3.354703092291
24260	H	0.293046999419	-0.851475844151	4.306081615835
24261	H	-0.042750848981	-1.478276751086	2.915800528491
24262	O	-0.471327585903	4.305764904388	-2.221500727169
24263	H	-0.860423567068	4.254612430693	-3.106465071989
24264	H	-1.243010584224	4.145755697992	-1.633592030902
24265	O	4.281059365077	-3.562134290609	1.771218960981
24266	H	4.702890563497	-4.039075814778	2.486461781667
24267	H	4.184883130875	-2.632431324315	2.074478755388
24268	O	-1.627514798048	-3.950451416892	-1.853279268844
24269	H	-2.505954369986	-3.513939155089	-2.015385055818
24270	H	-1.725298729565	-4.856786462194	-2.149550368941
24271	O	4.105239039022	-1.061883696721	2.668047833328
24272	H	3.476564319882	-0.358895306454	2.919934360620
24273	H	4.700465153531	-0.611170872688	2.040063175737
24274	O	-0.056477817568	-3.091366155070	-4.006931298706
24275	H	-0.517466787458	-3.261763547249	-3.166013501752
24276	H	0.708501998128	-2.531293484092	-3.810628665097
24277	O	-4.019201904114	-2.918876549879	-2.222939824382
24278	H	-4.111422453596	-2.697308111943	-3.177974161515
24279	H	-4.118718700474	-2.042206925487	-1.781459371890
24280	O	4.155517137718	0.436474135636	-3.316325864135
24281	H	4.125736187678	1.246250548241	-2.776864081095
24282	H	3.841097325200	0.722142356367	-4.194568445426
24283	O	4.312428539052	2.831070151659	0.650987150931
24284	H	4.685643445391	1.946270892951	0.830428862600
24285	H	3.516472189482	2.929959458914	1.192660630263
24286	O	1.647148416982	1.311149735813	5.921981807513
24287	H	2.227956525334	1.152822852873	5.160603995216
24288	H	1.050248790728	2.015047190742	5.614170535489
24289	O	2.334536428523	-2.664475488097	4.396300438744
24290	H	2.983338645718	-2.160141046050	3.891812595313
24291	H	1.685921556159	-2.997453121604	3.765025934432
24292	O	-4.344957146745	-0.599616952898	-1.006132582476
24293	H	-5.066477286240	0.042175382306	-1.184979548255
24294	H	-4.466916610634	-0.861799057809	-0.073307401390
24295	O	-1.474796393856	0.866731574540	-4.947485663735
24296	H	-2.242208648229	0.749058438868	-4.339489358500
24297	H	-1.437094419343	0.032563677800	-5.442614337460
24298	O	2.639398614322	1.413667446960	-5.372081838582
24299	H	2.306558444881	2.144594590167	-4.828204718893
24300	H	1.915370716404	0.787390127496	-5.446516413548
24301	O	-1.816355843077	3.600026694993	-4.638872656843
24302	H	-1.618858485245	2.659077115192	-4.796029687099

24303	H	-1.736161302853	4.039157731671	-5.485853263155
24304	O	1.702216640565	3.055515324520	-3.319652824010
24305	H	2.508940158454	3.087349227584	-2.775930554504
24306	H	1.023206724076	3.587412538769	-2.868321734949
24307	O	1.802231497058	-4.281422683825	0.860793834805
24308	H	1.832060167206	-4.052008629494	-0.079079617621
24309	H	2.701633170928	-4.130317016699	1.197904657718
24310	O	-2.002910265213	4.873406169758	1.736753930931
24311	H	-2.259843116189	5.684845261518	2.175034001782
24312	H	-2.470165044801	4.146691263431	2.229948106346
24313	O	2.376208385931	0.906620910914	3.288708054386
24314	H	2.261321339748	1.757016576461	2.848449685661
24315	H	1.531239050955	0.421963751855	3.204297191399
24316	O	2.107971808997	-1.405857288434	-3.348812940985
24317	H	1.488533558058	-0.645323984285	-3.234822434200
24318	H	2.982162790430	-0.980473231924	-3.353541779942
24319	O	-1.485354350545	-1.757251504411	-5.781707897732
24320	H	-0.958162869274	-2.262353172882	-5.109935143568
24321	H	-1.350486672343	-2.202959560167	-6.617902741123
24322	O	2.252242514707	-3.577198068488	-1.817794383743
24323	H	2.186874576314	-2.722820908093	-2.305228583290
24324	H	2.046086011534	-4.262584234732	-2.454965704092
24325	O	-5.947457050880	1.538254668251	-1.291628733368
24326	H	-5.765782640314	1.794470504465	-0.371723736815
24327	H	-5.555554309400	2.213714044931	-1.855467162936
24328	O	0.157354611859	3.027605972822	4.337100140757
24329	H	-0.622308317839	2.545585149285	4.033750652783
24330	H	0.695693837045	3.218716780056	3.556814026702
24331	O	-1.829735179304	0.959874300037	4.126954581154
24332	H	-1.710260628586	0.909204014022	5.078093979420
24333	H	-1.135189331145	0.373653944786	3.730968896761
24334	O	-4.085963410814	3.228761046883	-2.955395855212
24335	H	-3.699194673999	3.570832302459	-2.129180128768
24336	H	-3.462560115466	3.504936342624	-3.643080914036
24337	O	0.493849101822	0.659774516129	-3.152650119294
24338	H	0.964206509159	1.516906441806	-3.163927458527
24339	H	-0.213096133130	0.732850933884	-3.825321632267
24340	O	-3.498027254229	-1.275397544558	3.788999731744
24341	H	-2.893343541931	-1.923562010073	4.195407378375
24342	H	-3.077713914348	-0.412095133368	3.910902282130
24343	C	0.152329364407	1.656903937088	0.878944740407
24344	C	1.223336853562	1.597038112404	0.034513106835
24345	C	1.987487583602	0.411897326525	-0.266320458734
24346	C	1.740537936243	-0.829826896774	0.259861941755
24347	C	-0.277174396902	-1.362323499676	-0.720399396123
24348	C	-1.229276493145	-0.609749368277	-0.056787733778
24349	C	-1.575127741780	0.738990282598	-0.510170687536
24350	O	-2.546564657939	1.402141552348	-0.064684120133
24351	H	1.457956347277	2.478887598635	-0.560110304675
24352	H	-0.401819080631	2.574641143413	1.018412207023
24353	H	2.695859480563	0.496284920287	-1.086401385408
24354	H	2.315943059289	-1.683640610252	-0.067382688160
24355	H	1.231716040628	-0.967026435775	1.201109334333
24356	H	0.053014449064	-1.039481482746	-1.691389823975
24357	H	-0.131185134190	-2.406429622246	-0.489145252469
24358	H	-0.062392725511	0.889486202885	1.606618767571

```
H     -1.094804192558    1.086955949701   -1.437595662151
H     -2.591027425913   -0.482497952943    1.464227910820
N     -1.817075043876   -1.030582209054    1.124763615946
H     -1.906024762696   -2.024483095024    1.288327055386

Ambimodal TS Water45-57
155
ANGSTROM
O     -1.477810734822    3.190017531987   -3.081923531032
H     -0.594703851693    3.536688934141   -2.913590654427
H     -2.041671476306    3.507035473980   -2.351248689542
O      1.550701236188    1.912619220978    5.536527526123
H      1.907516061114    1.318917399116    4.841168082420
H      2.282297950236    2.127135376134    6.115315127842
O      0.439958592526   -3.481634720059   -3.551127353682
H     -0.151854178558   -3.637757374719   -2.800940223612
H      1.258622490767   -3.095714511416   -3.206134997998
O     -3.197323922400    3.612458606781   -0.957052863698
H     -2.910540584503    2.720613040474   -0.641409606891
H     -4.039271971304    3.404999832896   -1.407407774894
O     -0.822319263825    4.750759434397    0.068070778864
H     -1.084605468338    5.199242871487    0.907616317730
H     -1.665590548165    4.528627115089   -0.347374205720
O     -3.937672091503    4.071017654072    1.741386792496
H     -3.922932209207    3.146040941193    2.033164690686
H     -3.808859527528    4.048149523864    0.779681568124
O      4.452497533620    0.263553915567    1.868932282227
H      4.709993518143    0.177909616142    0.915780238995
H      5.239334566220    0.581848148922    2.318456347267
O      4.859804856863   -2.331612359234    2.808349599346
H      4.595442570854   -2.307020540874    3.751306064610
H      4.618615147300   -1.459567287842    2.454673496631
O     -4.235292676613   -0.801867475447    3.548476064319
H     -3.986720746523    0.033643402120    3.063628046060
H     -4.977244456359   -0.573705293271    4.108686535410
O      0.928768968968   -2.892275092588    5.133229763849
H      0.902673601307   -3.130636896769    4.196590533395
H      0.263355343722   -3.431225806985    5.564425972578
O      3.304298481090   -4.334251599285    1.945919243399
H      2.424022015674   -4.116876957062    2.281914954096
H      3.918872512654   -3.669927073350    2.319205501159
O     -1.151642947907    1.364008183564    3.716371329285
H     -1.124620212816    1.075933795704    4.637363843130
H     -0.715340178435    2.250583999735    3.692566917101
O     -5.363907653624   -0.278827659149   -0.555944137899
H     -4.572109510363   -0.682067000402   -0.982206113055
H     -6.125173210076   -0.564727406238   -1.063411898649
O      3.474853005132    2.838104482972    1.461789957658
H      3.824570947503    2.917978980156    0.554797460764
H      3.636613697305    1.922254249859    1.719849739571
O      0.577178251394   -4.958672567162   -0.093718489067
H     -0.186466180578   -4.798872950471   -0.682303171947
H      0.546966277065   -5.883487049581    0.154010607677
O      4.408354786558    0.219582863430   -3.157995389529
H      3.780516845811    0.963649650107   -3.200700350659
H      5.025131757463    0.343668614932   -3.880290859323
```

24415	O	2.358332650506	0.027898596518	3.806297275596
24416	H	1.518185375860	-0.417013286683	3.546841967668
24417	H	2.882632667948	0.144819844954	3.005277565429
24418	O	4.453930035381	2.842270523696	-1.069433349035
24419	H	3.804815847562	2.671307651793	-1.776289855561
24420	H	5.029748822212	2.072948044373	-1.022201750384
24421	O	-0.012541029097	-0.993301251776	3.103885348308
24422	H	-0.419454178985	-0.105012405527	3.133115607487
24423	H	-0.633783527286	-1.517116194334	3.647437760450
24424	O	0.994864243998	4.358524289555	-1.993544769613
24425	H	1.756576079658	4.891167304845	-1.692829096912
24426	H	0.324452847538	4.525986347693	-1.308449586841
24427	O	-4.964223397304	-2.311363919513	1.330490407816
24428	H	-5.160687274916	-1.567007574725	0.736610009378
24429	H	-4.734910919085	-1.907307142930	2.182328333068
24430	O	-2.887155640054	-1.253152866536	-4.316621680681
24431	H	-1.965134389452	-1.318648157195	-4.631206697623
24432	H	-3.078042873417	-0.297428434056	-4.289084757352
24433	O	0.092800154597	3.686693541504	3.832531441513
24434	H	0.700907279188	3.387580419350	4.520356833686
24435	H	0.645822108013	4.079854077348	3.115843836956
24436	O	5.667209300882	-2.621704886228	0.004847834339
24437	H	4.931991153314	-3.135924074313	-0.351824417657
24438	H	5.638994122835	-2.742344208732	0.961987766119
24439	O	2.569469105835	2.302568217375	-3.064451773527
24440	H	1.911137187158	1.598195333644	-3.230239571598
24441	H	2.035190245219	3.084713393537	-2.843601900275
24442	O	0.606809479680	-3.580632453661	2.286825878230
24443	H	0.572290983305	-4.010659098771	1.408585890659
24444	H	0.361972323384	-2.652008948227	2.155957228866
24445	O	-1.835154605018	-4.458592919914	3.233488823563
24446	H	-0.915842343080	-4.288377943441	2.966694876623
24447	H	-2.081032331416	-3.715057670100	3.799743365216
24448	O	-2.031058331068	-2.006086446190	4.613053954583
24449	H	-2.830972560450	-1.596268251269	4.229294970372
24450	H	-1.758947606338	-1.442883017264	5.347724817413
24451	O	-2.849904240711	-3.904744593339	0.763192534807
24452	H	-2.606994051162	-4.279518001632	1.631458493820
24453	H	-3.694408672923	-3.430537599128	0.905774584968
24454	O	-0.235943111340	-0.242931530161	5.963332476918
24455	H	0.356164253399	0.522508527820	6.023895242977
24456	H	0.330598789234	-0.998766004308	5.753327237145
24457	O	-3.220807479048	1.447139119285	-4.098291022168
24458	H	-3.538207604858	1.948771109692	-4.850716928899
24459	H	-2.478816941111	1.973447387814	-3.728528040144
24460	O	1.515250549830	4.636849397113	1.766561841444
24461	H	0.847922669771	4.564389900068	1.071465540572
24462	H	2.162001186580	3.915299083382	1.650350017158
24463	O	-5.115515602149	2.110923429819	-2.093553062998
24464	H	-5.171371212042	1.430559374717	-1.404281137787
24465	H	-4.514105129680	1.758956592421	-2.768120558825
24466	O	-0.339796208907	-1.584816899852	-5.185459117657
24467	H	-0.067123711992	-2.340029881970	-4.597474596424
24468	H	-0.149098286674	-1.856047774843	-6.083230995992
24469	O	-3.448843901722	1.367757975594	2.318642751943
24470	H	-3.205405836616	1.247721183725	1.373386198880

```
H     -2.604971933378    1.464155379999    2.804434311765
O      5.413025132493   -0.019114279657   -0.577309065890
H      5.653331594645   -0.957797737743   -0.415836594229
H      5.040609451630   -0.008240863577   -1.473668471043
O      3.072282302084    5.444586638866   -0.615018077025
H      3.773724165619    4.786264585695   -0.654475383828
H      2.732412141913    5.444730325406    0.290874962762
O      2.743921090884   -2.061538960514   -2.736816363549
H      2.970135131200   -2.601320832287   -1.960101852964
H      3.506137005702   -1.494285852900   -2.910742538708
O     -1.530242763755   -4.249323080809   -1.584071041422
H     -2.166015603543   -4.277923800708   -2.318457496957
H     -2.063102545434   -4.097371535909   -0.778045491740
O     -3.328294181127   -3.926194089175   -3.653307918811
H     -3.251919986730   -3.016877638556   -3.987993168826
H     -3.320921073706   -4.502989262478   -4.417068680050
O      1.077670278860    0.088957429469   -3.477825088768
H      1.669370311163   -0.639605848277   -3.234014454521
H      0.522733349677   -0.252294771521   -4.192535523326
O     -1.747840654856    5.510319246130    2.442247660936
H     -1.278253069659    4.999387456823    3.110364033627
H     -2.616847936518    5.076775824173    2.316098256506
O      3.621118472238   -1.961931389633    5.178932767773
H      2.873567838758   -2.536832885507    5.380555060010
H      3.214006725105   -1.152049529366    4.820113061637
O      3.111206818102   -3.814996574206   -0.631146547407
H      3.246752539444   -4.036696482143    0.325525979175
H      2.313383954985   -4.313534669531   -0.844715435664
O     -3.392751074817   -1.650160683012   -1.680014778569
H     -3.171746908593   -1.466363803233   -2.613046970596
H     -3.606761947860   -2.584862624375   -1.628076447098
C      0.119155514073    1.655984678947    0.706542453628
C      1.225499330643    1.554085757276   -0.083145953911
C      2.017282753369    0.364746844608   -0.262760583267
C      1.740558366801   -0.845332269361    0.327786189614
C     -0.120402438826   -1.462581241284   -0.775390650160
C     -1.159679689334   -0.767535097872   -0.179887530118
C     -1.565272703410    0.558861463250   -0.646304222934
O     -2.570695820265    1.167115875345   -0.201812903547
H      1.469238320942    2.393661324477   -0.728976361372
H     -0.450447311477    2.573513330865    0.749551040572
H      2.756223911027    0.395685889281   -1.057955671179
H      2.345911083034   -1.714959795996    0.113274570931
H      1.182088409101   -0.899077102704    1.249572821963
H      0.249801577433   -1.127237391034   -1.728069532332
H      0.041825240548   -2.505360251589   -0.547771464650
H     -0.113144644274    0.941936540570    1.478724121600
H     -1.104714573989    0.923376538610   -1.578941056880
H     -2.657487635471   -0.766301298405    1.189635974194
N     -1.800737381245   -1.246615591570    0.957258199199
H     -1.902049548450   -2.252651774860    1.032880885336

Ambimodal TS Water45-58
155
ANGSTROM
O      5.042262355517   -4.344153809135    0.437452695205
```

24527	H	4.239741098391	-4.382303700652	-0.105608038673
24528	H	4.732727684503	-4.320727787902	1.346841085637
24529	O	4.700626255277	-2.082548908841	-2.905527693942
24530	H	3.758578371769	-2.344677412926	-2.888356052470
24531	H	5.135647536392	-2.651108471770	-3.541591873921
24532	O	-2.777098653743	-2.335315121184	-2.427337060414
24533	H	-2.596670758126	-1.404185952539	-2.620162141077
24534	H	-1.969658420173	-2.825443176554	-2.663423588535
24535	O	-1.910631477311	-5.611887684666	-1.522439581749
24536	H	-1.431409587222	-4.992303650333	-2.108217096599
24537	H	-2.056687914777	-6.408995939578	-2.031698412108
24538	O	2.653228673681	-3.752003487143	2.141355217980
24539	H	1.768920354885	-3.907717892853	2.492260036118
24540	H	2.566916760625	-3.807336557118	1.176933375458
24541	O	-2.974113092482	-3.696829170012	2.317158610225
24542	H	-3.172373656092	-3.662635496284	1.347456793081
24543	H	-3.515905698724	-4.400698867162	2.675160566252
24544	O	1.550295294045	0.018323332040	-3.505833757633
24545	H	1.087762234050	0.869920929146	-3.400036597003
24546	H	2.508945312598	0.265801801685	-3.494984925921
24547	O	-3.532849418270	-3.723221345343	-0.269276538703
24548	H	-3.127818543324	-4.539541928705	-0.599763436157
24549	H	-3.300011746564	-3.071006194239	-0.965178497009
24550	O	-0.761722827805	-3.094969822768	-5.724499820229
24551	H	-1.507549136018	-3.232445001005	-6.308106745998
24552	H	-0.724257826891	-2.106158921220	-5.546761690626
24553	O	3.470622342504	-1.218322585986	2.520901772421
24554	H	4.234958664767	-1.185410701550	3.113464961237
24555	H	3.180729038848	-2.149988128096	2.442143265470
24556	O	5.451740305264	0.307951778715	3.666816599699
24557	H	5.761378128158	0.305785652464	2.746204227678
24558	H	4.744607737522	0.963667369796	3.688381302866
24559	O	3.472787015724	3.256344868704	1.291336253311
24560	H	2.763222720574	3.928087656608	1.374206432892
24561	H	4.294218725600	3.678886939215	1.548039376312
24562	O	0.188885087069	5.462751829197	-0.650714073776
24563	H	-0.680631443429	5.025432630142	-0.670090324484
24564	H	0.569763449786	5.302614191355	0.229474634835
24565	O	0.261362805193	-1.221510127453	3.189291508750
24566	H	-0.542760999241	-0.681068527021	3.266891484440
24567	H	0.826732861808	-0.992570974235	3.943824984564
24568	O	2.015019147339	-2.636962060831	-3.107663491572
24569	H	2.070641141830	-2.878806190306	-4.060217658462
24570	H	1.788882753131	-1.687938334551	-3.109079920008
24571	O	-2.307247088903	2.977291929145	-3.368412548897
24572	H	-2.287183642426	3.528096901251	-2.564427788143
24573	H	-1.370165015119	2.846020791377	-3.589514584540
24574	O	0.016891029234	4.522355978979	3.695131844885
24575	H	0.187033390575	3.622650651079	4.022563145203
24576	H	-0.851191455708	4.474568618147	3.257714780380
24577	O	-0.526522770315	-0.572836617382	-5.214324060443
24578	H	-1.272570742320	-0.256985495332	-4.656883672992
24579	H	0.273752639642	-0.402892265779	-4.688654662171
24580	O	-0.569880981784	-3.821986210754	-3.131520317008
24581	H	-0.668096774458	-3.708755690816	-4.096474332302
24582	H	0.316776625717	-3.487107826002	-2.918512515541

24583	O	-2.275855238944	4.141530780929	2.279874821095
24584	H	-2.753653038984	3.300642278914	2.311906164764
24585	H	-2.866535852137	4.765928490079	1.853394582910
24586	O	6.195586060839	-2.043258569878	-0.511437479518
24587	H	5.732701864530	-1.993893893621	-1.359974603432
24588	H	5.917029180453	-2.889252369651	-0.113917058244
24589	O	1.968091114378	-2.986599039753	-5.750610205222
24590	H	1.024434620936	-3.188268630882	-5.884324336295
24591	H	2.116275343013	-2.127585550978	-6.152164465172
24592	O	-5.460706308034	-0.187798437781	2.677895942975
24593	H	-4.799036199668	-0.717761678856	3.207748351166
24594	H	-6.198762846774	-0.007472812305	3.259219696460
24595	O	-2.503142110199	0.210662101449	-3.571665422029
24596	H	-3.366896998360	-0.104769169207	-3.931061923475
24597	H	-2.547595519206	1.181679331534	-3.532500265787
24598	O	-3.599265378158	-1.512539404559	3.931222874046
24599	H	-3.288598457592	-2.239587773982	3.366549611969
24600	H	-2.884165828764	-0.854399909612	3.953734300099
24601	O	-4.758612311855	-1.060827621959	-4.138585764963
24602	H	-5.362430101783	-0.688658095471	-3.455025078061
24603	H	-4.419951229814	-1.874385994809	-3.748557803038
24604	O	-4.675238419895	2.338583344317	-1.845116764778
24605	H	-4.290024104702	2.679086264488	-2.659291925337
24606	H	-3.936311671467	1.910232785777	-1.375727018706
24607	O	4.111725767345	0.646022615266	-3.298394070582
24608	H	4.370826392555	1.209147886330	-2.540464693897
24609	H	4.541857060084	-0.208328229201	-3.148735214906
24610	O	2.772774129607	1.336551905215	3.081792861009
24611	H	3.000305196685	1.986039285729	2.387235578561
24612	H	2.812353087030	0.460502275733	2.656203231656
24613	O	0.015826395900	-4.411276239153	0.127633441700
24614	H	-0.074534107482	-4.259718763573	1.091894784676
24615	H	-0.669298854604	-5.026157812772	-0.152330710984
24616	O	-2.203771035595	4.097447365599	-0.837890519237
24617	H	-2.150652241055	3.155625571405	-0.592006816744
24618	H	-3.050554266051	4.385856634762	-0.453687311334
24619	O	5.332897514132	0.055348086565	0.872285212158
24620	H	5.793449913162	-0.662008662525	0.380447094708
24621	H	4.516494885670	-0.359420306928	1.193541089067
24622	O	1.587763422491	5.141030639197	1.726169719761
24623	H	0.993724472816	4.873983441497	2.488426663648
24624	H	1.889344442274	6.026617861385	1.932109282287
24625	O	0.453835033339	1.888719354629	4.348887223100
24626	H	1.231612198120	1.710183943096	3.774411083817
24627	H	0.665125188672	1.472833504789	5.188731938965
24628	O	-1.808617283170	0.538044087166	3.595972696741
24629	H	-1.125701937954	1.159224301380	3.905064429076
24630	H	-2.399797930084	1.041031112845	3.006969711402
24631	O	2.027832930947	-0.403965554069	5.220046958628
24632	H	2.556642879971	0.160605074109	4.639370052153
24633	H	2.606038636539	-1.109791005663	5.512021574440
24634	O	-3.673672855063	1.756380950889	1.954491000325
24635	H	-4.388952777557	1.124484775805	2.153228204682
24636	H	-3.292579773976	1.495087863617	1.086058996445
24637	O	-0.216540497633	-3.786981987258	2.723896330846
24638	H	-1.182172789952	-3.842907392391	2.771867670322

```
H     -0.012113230492   -2.837027825091    2.892773510065
O      1.819514062233    4.112688287505   -2.192647840214
H      1.202684905938    4.664884841119   -1.641057981947
H      2.479170721589    4.704626653902   -2.552559825232
O     -4.740500119267   -1.063087706468    0.195437070018
H     -4.717453282852   -2.023120871599    0.143520380091
H     -5.197413829065   -0.821033926452    1.015866509407
O      2.515040340821   -4.083891556782   -0.710498771094
H      2.429938773238   -3.590903024288   -1.536130477305
H      1.601830099784   -4.346088711541   -0.479948814009
O      4.775605720177    1.970755504588   -0.996647365854
H      5.085473530300    1.292712115457   -0.363285497419
H      4.151802782973    2.505465953042   -0.492701365486
O     -4.649088299036    3.991999999321    0.387828908369
H     -4.965397346672    3.555594580143   -0.419860330749
H     -4.524382271319    3.273779827646    1.025541507254
O     -6.052749763255   -0.047079586787   -2.027761372902
H     -5.767280281675    0.880973078335   -2.025253688889
H     -5.602463800323   -0.446454877563   -1.263377282562
O      0.401529031317    2.486384962343   -3.700282098894
H      0.930303892488    3.078441518469   -3.109052818701
H      0.679199182964    2.681254193382   -4.596928577790
C      0.160574105173    1.693657506669    0.882727066222
C      1.223953462646    1.636035607182    0.043561921828
C      1.998517014196    0.440922885119   -0.258989521530
C      1.783756816508   -0.799240327466    0.258716059113
C     -0.216026095021   -1.334835401519   -0.919465272106
C     -1.199581911444   -0.602881596131   -0.298604965128
C     -1.515981879832    0.738221492979   -0.767629264563
O     -2.508889078454    1.423592486242   -0.407509185565
H      1.481709296867    2.515959777667   -0.542506122367
H     -0.390298092879    2.611821119958    1.041231974671
H      2.719689578167    0.550153589997   -1.064528955660
H      2.376506777325   -1.641021797928   -0.072028861891
H      1.205904361723   -0.975047947964    1.155072891082
H      0.222014574408   -0.975897733292   -1.834491739910
H     -0.053241449870   -2.374551444517   -0.675310770606
H     -0.089311642658    0.889660429296    1.560278377209
H     -0.937293375739    1.100549137730   -1.632397656314
H     -2.829188559547   -0.761769533148    0.927099590257
N     -1.864864961137   -1.053759913461    0.845625183419
H     -1.724583526947   -2.022577507591    1.081026401606

Ambimodal TS Water45-59
155
ANGSTROM
O      2.212856269974    0.867878693485   -5.542470432919
H      1.703109127448    0.064185689696   -5.730451689814
H      3.092237329587    0.596015937302   -5.263594373732
O      5.277165635827   -0.534516925096   -0.475913359093
H      6.004883246226   -1.042567492268   -0.074892392073
H      4.688496067009   -0.359221732652    0.295132771609
O      0.033213578744    3.644949836329   -2.324083965477
H     -0.621846996359    3.763009368400   -3.018058065971
H      0.359674665610    2.733357708571   -2.448557994496
O      1.678158400850    0.075154331939    3.103856706651
```

24695	H	1.680591783717	1.031919082013	3.266942440609
24696	H	2.473234424334	-0.080130706138	2.561075512054
24697	O	-4.589267494707	-0.493942672069	-0.371265364693
24698	H	-3.982425275407	0.221791648874	-0.132685361925
24699	H	-4.884133456218	-0.867067607280	0.493636267121
24700	O	-3.523830729873	2.854931032076	-2.525686651619
24701	H	-3.922871856530	3.712037811207	-2.372261524972
24702	H	-3.223272932767	2.533608382433	-1.651181706180
24703	O	2.820400372878	-3.622024708681	2.902708347013
24704	H	2.071434874956	-3.223661952995	3.367633836014
24705	H	2.514356726641	-3.876199489553	2.023472328272
24706	O	1.318651266082	-1.947853862712	4.695827260415
24707	H	2.113912532521	-1.870294593808	5.243421315620
24708	H	1.437551365018	-1.245137550997	4.007791635651
24709	O	-2.559378275648	-4.543139758322	0.563095604385
24710	H	-2.922984559450	-3.915772040530	-0.082916546106
24711	H	-3.048694358865	-4.382001086377	1.383845297477
24712	O	-0.623569134668	2.787109652712	5.960650643084
24713	H	-0.687987949361	1.821035257818	5.913738752711
24714	H	0.274336308918	2.978866334026	6.241568721730
24715	O	-1.770426453860	-0.763865718880	-4.133641269841
24716	H	-2.658493648774	-0.448733209987	-3.870564594468
24717	H	-1.711033772226	-1.653988389903	-3.747090744009
24718	O	2.090727368585	-1.412214495203	-3.257025887957
24719	H	1.701635301719	-0.575617758110	-2.925762455328
24720	H	1.657056486476	-1.555568998172	-4.112469368621
24721	O	4.861857313197	-1.999452331077	3.524883060966
24722	H	4.566572788637	-1.642929041427	4.376976471643
24723	H	4.185419849824	-2.671330963429	3.283732326732
24724	O	5.234128856511	2.296939171786	-0.385996530173
24725	H	4.562392180786	2.488423198995	-1.066316968928
24726	H	5.425318062709	1.350334412611	-0.481574044820
24727	O	-3.708558022412	-2.815787638489	-1.284047748864
24728	H	-4.562597613610	-3.163630002479	-1.545628860835
24729	H	-3.897180385190	-1.917465518797	-0.910400630068
24730	O	6.434430443232	-2.378877464751	1.151974416955
24731	H	5.984358205733	-3.138615000761	0.773295314538
24732	H	6.080670241126	-2.277154622169	2.046450686266
24733	O	4.093654253393	-0.075768277682	1.884876918407
24734	H	4.404287652058	0.805941518075	2.146422374342
24735	H	4.489522370180	-0.739407462740	2.496206616424
24736	O	-1.431507191383	2.142586798416	-4.292059808181
24737	H	-2.234063347498	2.412752125307	-3.823019846982
24738	H	-1.426897687010	1.177919549515	-4.275324691253
24739	O	0.498029048817	-1.293941259892	-5.531578718246
24740	H	0.220359150187	-1.895970830639	-6.222219540894
24741	H	-0.318312876966	-0.991701189177	-5.085664303951
24742	O	2.356090441580	4.417061578186	-3.690512883849
24743	H	1.641714156299	4.500901593443	-3.041106846719
24744	H	1.900640155242	4.128309865931	-4.506380371421
24745	O	4.470844452217	-0.028358323410	-3.410900442808
24746	H	3.697553294642	-0.620233254977	-3.352967824281
24747	H	4.972378548879	-0.185769582192	-2.603158274505
24748	O	2.028791839143	-4.217864648543	0.160544136886
24749	H	2.892345127248	-3.875380085309	-0.125923267222
24750	H	1.548901841619	-4.320745317914	-0.681546586169

24751	O	-5.097705167668	-1.528159049291	2.024758008770
24752	H	-4.851364531053	-0.882666203911	2.708456302972
24753	H	-4.596475207996	-2.327567154829	2.244108866246
24754	O	3.630039717095	-0.899495238683	5.816190078281
24755	H	3.165042488210	-0.027379918784	5.804685234162
24756	H	4.056940116772	-0.962357954990	6.670294659748
24757	O	-0.303917342104	-3.398366435621	1.441228841127
24758	H	-1.013603855258	-3.957062878666	1.057839581936
24759	H	0.531977713266	-3.708391784979	1.058181482949
24760	O	-0.437790531297	0.018077503080	5.518029448099
24761	H	-1.281283430323	-0.426678860087	5.772459255874
24762	H	0.155500104399	-0.722956704106	5.287636198514
24763	O	-3.624896802919	-3.718392652292	3.018124013492
24764	H	-2.760110400532	-3.475730965418	3.393127837817
24765	H	-4.076483551049	-4.236357442219	3.685224221161
24766	O	1.160407459374	1.170657350302	-2.995080511514
24767	H	1.997731898425	1.580525465877	-2.676525573314
24768	H	1.220584555628	1.238712053123	-3.961902597773
24769	O	4.677977031895	2.590281316924	2.161353243335
24770	H	5.441090389175	3.042653325512	2.521362897572
24771	H	4.815999054989	2.568454079408	1.175688406233
24772	O	-4.124175026351	0.152420217579	-3.115851057440
24773	H	-4.432204247120	-0.142995229435	-2.248351269155
24774	H	-4.090013855502	1.117781844432	-3.064578817698
24775	O	-1.724293691865	0.867420493975	3.210330461934
24776	H	-1.146344954861	0.541681387797	3.924646333029
24777	H	-1.628586029040	1.842899862862	3.212880444351
24778	O	2.146322509646	1.336652759803	5.871912897035
24779	H	1.276170231753	0.924432805574	5.779348349619
24780	H	2.262473931208	1.894924865912	5.084081250361
24781	O	-1.444645608456	3.531153042490	3.467757988878
24782	H	-0.648517369199	3.962478026410	3.103700705653
24783	H	-1.271960355006	3.435800082053	4.422394262764
24784	O	-4.136521468556	0.187005321916	3.963047218593
24785	H	-4.587536702180	0.977593324981	4.258946757613
24786	H	-3.246896551689	0.476044961818	3.622672060502
24787	O	3.488467960894	2.370013304207	-2.489239455874
24788	H	3.246590098428	3.187212422866	-2.992544612393
24789	H	4.006061030687	1.785315881757	-3.065992950602
24790	O	2.242112209791	2.727208121663	3.483624043321
24791	H	1.707601376217	3.427117297944	3.073664437471
24792	H	3.087697191158	2.725040322369	3.004053394904
24793	O	-1.753588713064	-3.337267005358	-3.079746375157
24794	H	-2.384282902182	-3.130396630839	-2.358552963493
24795	H	-2.096357000491	-4.113132799355	-3.525463765561
24796	O	-2.655546772032	-1.420450939502	5.662599637876
24797	H	-2.257224813544	-2.067537807874	5.060143920667
24798	H	-3.321991559889	-0.942071546300	5.141333742093
24799	O	-2.728710353775	4.030344944252	0.978202741276
24800	H	-2.502143912586	3.932098564522	1.913054707590
24801	H	-2.761436420019	3.133036435671	0.616190626025
24802	O	-1.128133154304	-3.044360026797	3.942857590255
24803	H	-0.330567929888	-2.842705312657	4.449323543188
24804	H	-0.791768759642	-3.167188935948	3.026760012246
24805	O	0.957800429221	-3.870709823374	-2.355564678970
24806	H	1.389249965893	-3.032915908656	-2.582660325156

```
H      0.025184873977   -3.752704910174   -2.583727185964
O      0.747850419439    4.783013908331    2.379676582733
H      0.934620986041    5.671635840709    2.683615964306
H      0.412463748251    4.883825454875    1.446724545976
O      4.126940933013   -2.951355762719   -1.146673344381
H      4.512704191695   -2.074501992417   -0.956452442772
H      3.696416157885   -2.877935352629   -2.001606146185
O     -0.397082931666    5.086586998306    0.043473596304
H     -0.178396460361    4.592122592634   -0.762163337769
H     -1.310563354513    4.828680339281    0.283336285419
O      0.786943943559    3.287388232867   -5.611601084146
H     -0.042830899643    2.948343222184   -5.242851275294
H      1.310752768076    2.513989271161   -5.854717471752
C      0.283940014405    1.759331099641    0.858284785953
C      1.364207017669    1.624274546272    0.070521730826
C      2.051043447721    0.362551665100   -0.247811410730
C      1.734737533436   -0.862197847919    0.206318448181
C     -0.552854920580   -1.252459395704   -1.096795521646
C     -1.353929256289   -0.485370725833   -0.307485495666
C     -1.677044014605    0.875538079145   -0.738666427725
O     -2.630866704003    1.559237885348   -0.309821721189
H      1.757268069260    2.502736860809   -0.440851279361
H     -0.189936694469    2.716255392353    1.013315090390
H      2.859291401569    0.460484743440   -0.968400883454
H      2.272565967313   -1.739073473762   -0.116675094658
H      1.017774102895   -1.040048428880    0.992807695332
H     -0.192501442340   -0.875072116422   -2.036872743439
H     -0.356726857889   -2.287474942272   -0.872782943578
H     -0.090909582537    0.956776581139    1.474641821070
H     -1.111333098412    1.251314397936   -1.606342597007
H     -2.166970759410   -0.288008415496    1.574134455275
N     -1.960181311473   -0.966512131360    0.854477397617
H     -1.557513386919   -1.817848270538    1.221325576835

Ambimodal TS Water45-60
155
ANGSTROM
O      2.871065281529   -1.051439829489   -2.992496621790
H      2.384208797816   -1.858840385895   -3.222647350837
H      3.204518392050   -0.666275455615   -3.827565711781
O     -1.239153573429    3.499331346698   -3.652095217669
H     -1.477008343408    3.619788128695   -2.715282829144
H     -0.273842579817    3.495440888298   -3.676470575938
O     -5.146938561185    0.502120435139   -0.091635376926
H     -4.273339170129    0.884576984200   -0.270779522470
H     -5.134963207578   -0.354814619195   -0.537509879490
O      3.524177289445   -3.986027541829   -0.843881830767
H      2.896268598844   -3.952225263126   -0.098291080868
H      3.487106602835   -4.888942773349   -1.161405815781
O     -3.679272165711    2.259350156097    2.020216203402
H     -3.118238233170    1.827254387352    1.349343603482
H     -4.340186981936    1.605707529003    2.277722279841
O      0.637703967754    4.747327481802   -0.965825435576
H     -0.272730951633    4.402872990001   -0.863660638436
H      0.551812369696    5.686092269205   -1.139700361243
O     -2.776456116109   -3.028431128947    3.680374783277
```

24863	H	-2.709361096757	-2.053024752315	3.615596835670
24864	H	-3.450424552580	-3.244200320770	3.015849609457
24865	O	1.102397651907	-2.988239264754	-3.963614953679
24866	H	0.483897407844	-2.325080331017	-4.317438879644
24867	H	1.352419882203	-3.547912860580	-4.700019663468
24868	O	3.186126474106	4.538084835926	0.363600501680
24869	H	3.038032404712	4.602417513703	1.328109595351
24870	H	2.309963027449	4.590724028533	-0.038527255050
24871	O	1.284222825862	-2.214729314901	5.092980413382
24872	H	0.407269579919	-2.387053712680	5.507464355387
24873	H	1.702464637962	-1.541293073898	5.632925789816
24874	O	1.274005000790	-6.210477655224	-0.417214725988
24875	H	1.502169684722	-5.534154708995	0.254851638891
24876	H	0.560566217855	-6.719709290976	-0.025052630496
24877	O	-5.078115779694	-0.324759528209	2.437075520639
24878	H	-5.218803370208	-0.007415408849	1.514239313933
24879	H	-5.101357455934	-1.287248796864	2.352324724708
24880	O	1.756064708748	-4.143473137656	1.234314191687
24881	H	0.859964900942	-3.848111131895	1.480188737190
24882	H	2.325982488539	-3.923814527410	2.002915629774
24883	O	-3.312864420736	0.157755789631	-3.012020106767
24884	H	-3.845073164446	0.941724151433	-2.780133143641
24885	H	-2.725355829575	0.475603533368	-3.720455714454
24886	O	4.411347556373	-1.073746615223	1.767265285388
24887	H	5.328074325728	-1.213562438809	2.011950892880
24888	H	4.383916816360	-1.178072417255	0.791785995254
24889	O	-1.950769407531	3.883537339720	-1.002690488053
24890	H	-2.728274188625	4.377263562076	-0.720825774687
24891	H	-2.150921550296	2.956038872261	-0.725662661758
24892	O	-1.624408099962	-5.704147147379	0.331539659065
24893	H	-2.588690316929	-5.640684868530	0.254034348900
24894	H	-1.269208941965	-5.368298207995	-0.501184248850
24895	O	-1.225647836096	-2.230186070031	5.980167796455
24896	H	-1.477738223587	-1.297653501465	6.014303268204
24897	H	-1.833305360467	-2.671701406016	5.372298884422
24898	O	0.107017500609	-0.872404520848	3.060136451530
24899	H	0.473158412729	-1.558981667435	3.645265791769
24900	H	-0.851902164599	-0.869728449096	3.176797697219
24901	O	3.316787145016	0.569161409429	-5.087925135653
24902	H	2.430719910989	0.853107879320	-5.324757715968
24903	H	3.685827669802	1.273277265496	-4.520229457191
24904	O	-4.399511648272	-2.919567130995	1.412140886147
24905	H	-4.518078055961	-3.785608610790	0.999678275490
24906	H	-4.074535833737	-2.361451727590	0.685847527813
24907	O	4.395899176350	2.182518221597	0.656941386885
24908	H	4.080703057008	3.100423581476	0.487251341380
24909	H	3.651684274964	1.609254924325	0.438319240468
24910	O	-0.841370553334	-3.633982817352	1.876092309114
24911	H	-1.147406314392	-4.429692269812	1.395819440224
24912	H	-1.389415643823	-3.575833143332	2.679682179235
24913	O	-4.280351699063	-5.170862599829	-0.304627974227
24914	H	-3.874336445231	-4.752233089724	-1.091516355532
24915	H	-4.963187922100	-5.757550187049	-0.629351167522
24916	O	-4.067274904360	-1.800026232432	-1.185341846623
24917	H	-3.674969299525	-2.632404492576	-1.496581862375
24918	H	-3.727004156026	-1.127076937126	-1.792643316302

24919	O	1.207469392027	1.129071051894	4.393593451958
24920	H	2.117730177162	1.208304928570	4.058919005426
24921	H	0.798834034257	0.436651437688	3.818043478122
24922	O	2.692056456363	4.214899315085	3.003413442482
24923	H	3.144525500194	3.412818332462	3.281110344665
24924	H	1.753197729383	4.083352854941	3.226522476760
24925	O	1.572517579981	3.307952104064	-3.148535157296
24926	H	1.397406311791	3.818465970957	-2.339234833467
24927	H	2.525775740926	3.114867187538	-3.157968019821
24928	O	3.213470813096	-3.163658903412	3.232716329574
24929	H	3.538682088682	-2.369945192217	2.778206370680
24930	H	2.577640593735	-2.877121578978	3.902571721918
24931	O	-2.878650706980	-2.285160806055	-4.328200947996
24932	H	-3.331393947122	-1.527267178976	-3.932350344638
24933	H	-1.980361424144	-1.963834156894	-4.511318167413
24934	O	-4.606403751026	3.831535261138	-0.036816014660
24935	H	-5.557063771026	3.909388778206	0.050446559173
24936	H	-4.305639506997	3.321027790736	0.743414857007
24937	O	-1.446073339122	0.686199302630	5.657464081800
24938	H	-1.856384792446	1.545863602166	5.413730595365
24939	H	-0.505313684091	0.795101589259	5.471363801132
24940	O	-2.878600607856	-4.029556875782	-2.310362699698
24941	H	-1.933492172109	-4.234446407147	-2.211003206111
24942	H	-2.940135625879	-3.524163181314	-3.150319726271
24943	O	-2.609305959978	-0.363324258739	3.471026743757
24944	H	-2.385509626475	0.071290715079	4.315893137816
24945	H	-3.554632087210	-0.208608189869	3.271093739998
24946	O	-4.314752047351	2.651686917980	-2.570483375495
24947	H	-3.630694754604	3.204589592016	-2.956147446352
24948	H	-4.455284592163	2.977057028962	-1.664756449647
24949	O	-0.496913443913	-0.927079997059	-4.854036919786
24950	H	-1.013070128106	-0.335588617604	-5.413112785023
24951	H	-0.099066588120	-0.315611643794	-4.194281434656
24952	O	0.759886695779	0.704318799600	-3.173513500676
24953	H	1.564397817634	0.185971331591	-2.988012338100
24954	H	1.022921816785	1.640787817304	-3.205761968010
24955	O	4.197898974249	2.438245153750	-3.283362858938
24956	H	4.832726359033	3.078777132661	-3.606234788022
24957	H	4.699676759753	1.888525585691	-2.620515880453
24958	O	0.247184054059	3.550712851824	4.056503940153
24959	H	0.373592127610	3.977073064791	4.906176935418
24960	H	0.571517753300	2.615246040228	4.165716266041
24961	O	5.614949376317	1.102441459533	-1.521158312463
24962	H	5.390739696134	1.532421162544	-0.673865513286
24963	H	5.471910112183	0.155521988723	-1.383976338002
24964	O	4.639535713035	-1.502988850697	-0.938638526395
24965	H	4.000982620429	-1.286521583390	-1.645732907504
24966	H	4.552869238333	-2.467400287746	-0.868989701674
24967	O	3.662335049187	1.265147973236	3.133715278840
24968	H	3.788879447425	0.344899475939	2.857028928480
24969	H	4.064604663418	1.765194337585	2.401068929667
24970	O	-2.038464600090	1.392918045670	-5.124915626851
24971	H	-2.757780812394	1.744050863200	-5.651031004736
24972	H	-1.692406251234	2.147703125335	-4.599382783708
24973	O	-2.394552411746	2.867108509374	4.434905134266
24974	H	-1.544237643360	3.248237808968	4.157808350868

```
H     -2.887773573134    2.703621914042    3.615130120284
O     -0.208659629397   -4.619246575288   -2.022874120392
H      0.441317632352   -5.200815422625   -1.574889375954
H      0.300303152620   -4.042191789449   -2.610366952178
C      0.163767818492    1.619819397091    0.983701035464
C      1.211812982711    1.544727912320    0.133271480175
C      1.925214881292    0.327994253913   -0.238855171671
C      1.642418976806   -0.924167833596    0.215736059406
C     -0.340715984670   -1.460364373992   -0.979767943301
C     -1.282615854337   -0.713267423541   -0.302038797278
C     -1.555371799865    0.659418774669   -0.707607827555
O     -2.484638315573    1.389717489169   -0.265004919342
H      1.501596764121    2.443708493740   -0.410372920328
H     -0.345600822396    2.555079946917    1.176990155482
H      2.631239925109    0.436006976923   -1.061288678878
H      2.179856944447   -1.782792954938   -0.155742359363
H      1.075121161214   -1.094972196570    1.118331277197
H      0.061288732838   -1.121964282646   -1.917360096423
H     -0.202403782889   -2.506429103398   -0.749959290201
H     -0.121895218845    0.801278869715    1.624497583408
H     -0.981483279626    1.029113261206   -1.570436268479
H     -2.412227485090   -0.621021049573    1.424126527484
N     -1.981071067416   -1.244949985832    0.764100405236
H     -1.623471548165   -2.111095100717    1.155931941423

Ambimodal TS Water45-61
155
ANGSTROM
O     -0.160242873161    4.657178206091   -0.521052157665
H     -0.574452701403    4.226975651825   -1.291978287786
H     -0.892324737920    4.825319976765    0.105314368026
O      0.144220917442    4.702495027037    3.346142757814
H      0.569516164989    5.259781121641    3.998523736603
H      0.803745477494    4.556764286799    2.640009293247
O      0.665638993153   -4.908477463091   -1.509428312662
H     -0.209986514645   -5.093016748963   -1.150369430374
H      0.541713832428   -4.585199817106   -2.412116458997
O      1.395412810822   -3.632193805145    2.165649456601
H      1.731817930370   -2.835190029375    2.614228771757
H      1.921805519402   -3.720920690169    1.354559420329
O      1.644680472263    0.781124149270    4.550078855350
H      2.210605640531    1.387882112650    4.042628853492
H      0.732812987334    0.997937331220    4.290477789426
O      1.345904468713    1.394838657389   -3.358158299381
H      1.144939707160    2.359811597416   -3.468428311334
H      2.240200957542    1.309144343132   -3.740210056664
O      4.041379900614   -1.904357065191   -1.944255759179
H      3.289759286056   -1.720655253788   -2.539480408219
H      3.709702382206   -2.568726447170   -1.322981712065
O      4.966246270303    2.455529074971   -2.487262681724
H      4.515298254890    3.135877588160   -1.972242065976
H      5.147509099002    1.722699471423   -1.872489700758
O     -0.927082157821   -1.896396165537   -5.853389108997
H     -0.755897563539   -0.979883464166   -5.517590177116
H     -0.549401298889   -1.922684669414   -6.733273402136
O      5.242352918465    2.375590276569    0.931391322554
```

25031	H	4.653913221334	2.317737840915	1.705241986453
25032	H	5.166082972582	1.529101037152	0.463087086993
25033	O	-2.716516509593	-1.014403374106	-2.615819418493
25034	H	-3.037851447613	-1.271094531576	-3.507344295356
25035	H	-3.471140995872	-1.201242345207	-2.015168788748
25036	O	2.024566541568	-0.384868756730	-6.065842063594
25037	H	1.210826512870	0.136010311067	-6.060209783303
25038	H	2.738994155354	0.230919378011	-5.856871182994
25039	O	5.342674515889	0.213566838390	-0.906100396545
25040	H	4.812476824321	-0.555136373910	-1.219995260944
25041	H	6.247212583733	-0.096690262551	-0.855070481998
25042	O	-3.498102776312	-2.152242990711	-4.922382483027
25043	H	-2.665596142909	-2.058261990262	-5.420726786299
25044	H	-3.431082048114	-3.008630214983	-4.480138687874
25045	O	-4.996548482709	0.493457001815	0.215979993413
25046	H	-4.085802714133	0.827449042847	0.049572530272
25047	H	-5.007016741036	0.266866608003	1.157078219634
25048	O	4.109944286742	-0.306037118088	2.085391834362
25049	H	3.524535371663	-0.866343856377	2.627802943556
25050	H	4.936459390476	-0.231952608171	2.564491446856
25051	O	4.113597695398	4.310806730120	-0.411739946248
25052	H	4.567147802390	3.573558389761	0.087963742623
25053	H	4.748262226429	5.028127511504	-0.446203165716
25054	O	-2.885073230498	2.334260984819	2.243685895586
25055	H	-2.102118702713	2.079223765706	2.760818527580
25056	H	-3.489437487874	1.589030454544	2.290136339359
25057	O	2.802123857713	-3.911259724325	-0.266116574030
25058	H	3.392260465490	-4.665062041807	-0.222180597332
25059	H	2.006503770732	-4.229631258207	-0.751757263511
25060	O	-2.585906457541	0.206901804378	4.911063909866
25061	H	-1.778225687139	-0.117081915360	4.435085708838
25062	H	-2.723543079294	-0.399127734830	5.639127830385
25063	O	-0.696366980350	2.130350081249	3.901682675723
25064	H	-0.424657577156	3.060228417918	3.805054454578
25065	H	-1.271804347205	2.054189671972	4.667740946435
25066	O	-4.754740918450	-4.025821805912	0.110649696113
25067	H	-4.770567744008	-3.189936884369	-0.407304472304
25068	H	-5.592783862689	-4.458618205663	-0.052477989214
25069	O	-0.487820979655	0.612201652653	-5.130599178483
25070	H	0.142363319596	0.892713406312	-4.424452353953
25071	H	-1.329033824969	1.023780215616	-4.891489392464
25072	O	-2.746398712600	1.460940266757	-3.699945677855
25073	H	-2.588083337000	0.635881035472	-3.201707689193
25074	H	-3.643920355515	1.720368774512	-3.434500488641
25075	O	-2.046005832135	4.764755861805	1.432715437126
25076	H	-1.453202736681	4.970312895817	2.165890990135
25077	H	-2.435211985580	3.900542822308	1.667593673085
25078	O	3.091311172566	2.165264416119	2.648811660173
25079	H	2.506130199659	2.737027341057	2.125017192160
25080	H	3.136951607823	1.288543780812	2.226092208178
25081	O	0.302627591748	-3.473767660827	-3.956161811496
25082	H	-0.100049994372	-3.095167635876	-4.757417634279
25083	H	0.918106008011	-2.776322584536	-3.659246144474
25084	O	-4.106964018224	-0.326438850169	2.688626150314
25085	H	-4.129479523456	-1.304329687994	2.692695772252
25086	H	-3.699408629117	-0.080634699349	3.540447368056

25087	O	-2.157475242951	-4.935277946699	-0.518785944751
25088	H	-2.170450334003	-4.490507540499	-1.398139780362
25089	H	-3.078384155373	-4.875344949470	-0.225246737030
25090	O	-3.870609726323	-3.041691772271	2.562527788055
25091	H	-4.326336593589	-3.401352185332	1.784920331130
25092	H	-2.950479193120	-3.318717672990	2.446467267567
25093	O	-2.135537723361	-3.649718666357	-2.837099365227
25094	H	-2.222814324163	-2.716143862030	-2.566888101304
25095	H	-1.253672388224	-3.702115545514	-3.253047810482
25096	O	-5.767400204122	-0.791119120624	-3.587087790778
25097	H	-5.642103219023	0.160575642751	-3.486413538631
25098	H	-5.156919824532	-1.097116794417	-4.268864817105
25099	O	3.856266734398	0.902543758515	-4.339113657859
25100	H	4.302919284285	1.601315200914	-3.812007088933
25101	H	4.218937862864	0.071904173159	-4.019761674305
25102	O	-0.368033322424	-3.425334677001	4.443327466529
25103	H	-1.166704619405	-3.799690556859	4.059721382799
25104	H	0.355794964799	-3.747640406043	3.892634145748
25105	O	-0.565530970360	-0.831405038036	3.552950880769
25106	H	-0.444091969489	-1.725717716005	3.923017336525
25107	H	-0.932260917047	-0.939406145288	2.657663874182
25108	O	2.020224825071	5.531015181949	-1.967642160808
25109	H	2.730715761675	5.069288102786	-1.507812382322
25110	H	1.230811847483	5.447167065109	-1.408777498121
25111	O	0.874989742179	3.928978066109	-3.843969141755
25112	H	-0.052870130936	4.055643149975	-3.601099833517
25113	H	1.379204331424	4.568828007889	-3.305063814352
25114	O	-5.157614364863	1.841913111083	-2.360269838235
25115	H	-4.880969848337	2.707545569784	-2.027690340337
25116	H	-5.325976434397	1.331940041212	-1.556686123810
25117	O	-1.305342915063	-3.762373621110	1.746618930344
25118	H	-0.332616579085	-3.764013582460	1.824863158083
25119	H	-1.525985588689	-4.271504978692	0.940743394484
25120	O	-3.652047716726	3.728966660417	-0.891109270587
25121	H	-3.238343887717	2.877903977503	-0.634501084769
25122	H	-3.511006745374	4.319797788991	-0.146818875962
25123	O	1.861245494268	4.282946365639	1.268742003573
25124	H	2.678829144690	4.452925238868	0.774846236894
25125	H	1.144277948157	4.348528451140	0.607059569298
25126	O	2.368773553335	-1.598403187058	3.743193383327
25127	H	1.959205074112	-0.730241189839	4.026415586320
25128	H	2.547133229711	-2.063669887842	4.561284385245
25129	O	-4.886330287805	-1.703651179423	-1.222235377480
25130	H	-5.415148655948	-1.518817214414	-2.025232353119
25131	H	-5.024380892656	-0.928828546441	-0.624357260879
25132	O	-1.614037396029	3.725020126831	-2.663435414061
25133	H	-1.867083151237	2.883595942417	-3.088104627510
25134	H	-2.409360298182	3.974478613001	-2.158115034978
25135	O	1.896426749034	-1.322514702828	-3.545783031956
25136	H	2.019451856085	-1.161173060505	-4.508496595544
25137	H	1.577555052828	-0.473567844459	-3.205279978934
25138	C	0.125452680518	1.675488780181	0.796719347300
25139	C	1.204056422217	1.587993418032	-0.034185784211
25140	C	2.007077457591	0.406397693174	-0.221759485683
25141	C	1.744606507655	-0.797367400925	0.384108836046
25142	C	-0.132309494872	-1.429808452802	-0.708363025938

```
C     -1.139013924833   -0.655005612699   -0.161533124853
C     -1.502242238031    0.650929783276   -0.718880155932
O     -2.539360872729    1.284022861666   -0.425474937464
H      1.408539634140    2.411570797965   -0.716804297396
H     -0.473652896864    2.571382911782    0.849274761917
H      2.736621442593    0.444302464389   -1.025475829771
H      2.348966533568   -1.664194849893    0.167230593706
H      1.197988722663   -0.850955298867    1.315988308218
H      0.256284397795   -1.192592951001   -1.683018659314
H     -0.006128516563   -2.458886626625   -0.402128254502
H     -0.060151580989    0.971793477996    1.594839393449
H     -0.913671735047    0.984822678517   -1.590705674274
H     -2.632333117739   -0.569267397963    1.267851517357
N     -1.782819851078   -1.055434878267    1.010094027314
H     -1.785955095459   -2.051328922290    1.210542877749

Ambimodal TS Water45-62
155
ANGSTROM
O     -2.695543614606    1.910450043101    2.148294315825
H     -2.310380712934    2.805462637530    2.186732041377
H     -2.773824893042    1.683453972708    1.195802380652
O      2.321422685900   -0.052645391345   -6.080426983459
H      2.392858997696   -0.423703939774   -5.169602346944
H      3.164458152796   -0.207682205507   -6.505249603101
O     -2.224707844513    4.099327214513   -3.494326279760
H     -2.267675135714    5.048793715262   -3.605606607325
H     -1.447412033361    3.919640102279   -2.901771152319
O      1.652954024116    4.697130224883    1.863896201665
H      1.225514015648    4.027568195556    2.437725756275
H      2.158793088029    5.260239060414    2.451908125223
O     -5.582235612834    1.670451743088    1.284134837752
H     -5.077725084677    2.428989776942    0.931366007173
H     -5.686285819679    1.853035667163    2.229409262719
O      2.199271764624   -0.977649811552   -3.565807083260
H      1.511463920529   -1.666292799790   -3.557227732557
H      1.758910403023   -0.148374955473   -3.267291099177
O      1.589625643793   -4.348049034240    1.035480697950
H      2.125400915312   -3.699569645919    1.518961503037
H      1.835874447574   -4.261881491730    0.101613452153
O      1.913150874210    5.226251284972   -1.970263459271
H      1.445110898145    5.848266793881   -1.369100624840
H      2.631085774451    4.857566675963   -1.438064608222
O      3.672350725344    2.409759002189   -2.415792884004
H      3.694106298956    2.942289713979   -1.602034121887
H      4.387599854058    1.749309611632   -2.314189266102
O      2.296312099064   -4.116092431200   -1.680374080317
H      2.456144735006   -5.012289098292   -1.978923467596
H      1.610735414751   -3.746316639897   -2.272256139266
O      2.888147771067    4.116562852363   -4.409881153512
H      3.366224046060    3.507717541290   -3.819340016942
H      2.476736082292    4.753591079135   -3.811447524704
O     -4.714862154563   -2.597613920630   -0.033162968888
H     -4.280459168996   -2.048344294064   -0.712826295826
H     -5.626571342499   -2.691482988049   -0.313512026697
O      0.827121150766   -1.042943761625    3.616207054186
```

25199	H	0.171151061634	-0.306025645117	3.641431081344
25200	H	0.280921822005	-1.809928692357	3.858688777679
25201	O	-1.593789024370	4.348215041607	2.521766559442
25202	H	-2.479350077169	4.659616670032	2.830466076411
25203	H	-0.996189592687	4.313490010800	3.272559535176
25204	O	-3.674128269108	0.538002975141	-4.216344277854
25205	H	-2.929247792838	0.946616079963	-4.679463822904
25206	H	-4.047440830176	1.252227465230	-3.670306326622
25207	O	0.455066400901	6.378992320783	-0.059239640441
25208	H	-0.451428892129	6.055881914807	-0.187131429770
25209	H	0.819590092065	5.848898384916	0.667157698412
25210	O	-4.359567604262	-0.716194110943	2.032136385908
25211	H	-4.805219122299	0.084195874614	1.705082300236
25212	H	-4.574605945788	-1.436123199260	1.419634288320
25213	O	-2.638058153812	-2.047258176787	-5.110113113818
25214	H	-1.787458341080	-1.564264227953	-5.134425082177
25215	H	-3.306554902418	-1.360647168371	-5.008607648703
25216	O	-1.816233968482	4.738540678885	-0.157020630272
25217	H	-1.289279823694	4.107694832232	-0.676449051042
25218	H	-1.597536272463	4.604017036789	0.779829051781
25219	O	-0.377966660707	-0.550218690838	-4.933770526768
25220	H	0.320829423011	-0.467109464107	-5.590269959863
25221	H	-0.776700474064	0.332397976124	-4.864693745641
25222	O	4.778914605884	-1.047917790985	0.761665209487
25223	H	4.550615891691	-1.380014557745	-0.122150813659
25224	H	4.201512718999	-1.520434796426	1.376922249414
25225	O	-1.046863718120	0.833711307855	3.913871274410
25226	H	-1.689939765097	0.251866978188	4.372487163565
25227	H	-1.576070875118	1.159424762655	3.153845596121
25228	O	-1.092625048653	-2.991332396622	4.072068274498
25229	H	-1.144989094012	-3.789394112985	4.597665983156
25230	H	-1.125817278480	-3.280238121861	3.127629636082
25231	O	2.727351040704	1.007942465730	3.626684837576
25232	H	2.110152453588	0.260315176398	3.555407522599
25233	H	3.287665513802	0.833160448156	4.383893029355
25234	O	4.379644827427	1.608292138186	1.361581672359
25235	H	4.573161089986	0.689115209983	1.098403066511
25236	H	3.776417084435	1.525298750685	2.111622117440
25237	O	-4.406294488079	3.906304214292	0.181553492939
25238	H	-3.488333597804	4.228841730981	0.074849665015
25239	H	-4.670390692941	3.650503989406	-0.712313102392
25240	O	-0.154682218280	3.526951387666	-1.991281556850
25241	H	0.236749548067	2.682509032151	-2.296550389411
25242	H	0.581933861684	4.177786550076	-2.036859136303
25243	O	3.576346770275	3.830089760226	-0.026321269545
25244	H	2.893577620624	4.139990339455	0.591302153153
25245	H	3.994913660494	3.080329108371	0.439615086500
25246	O	4.081596103733	-1.927328268445	-1.845429405077
25247	H	3.454761724716	-1.513003998628	-2.473430020538
25248	H	3.715946828623	-2.811214944230	-1.704839864718
25249	O	-2.700189789574	-4.533317525327	-0.364795399104
25250	H	-2.432403772219	-4.214790678361	-1.239579245374
25251	H	-3.474931697009	-4.002061892263	-0.124088978181
25252	O	-0.992171637588	-3.717946047591	1.545680690563
25253	H	-0.085291822646	-4.026099305991	1.346289835140
25254	H	-1.592669896552	-4.148159700478	0.901382914434

25255	O	-1.471938605557	1.998538219386	-5.112829338333
25256	H	-0.608990466697	2.269620781046	-5.465094943177
25257	H	-1.793679703019	2.765794653161	-4.610627686344
25258	O	0.446597601813	-3.151000974355	-3.456094711531
25259	H	0.324941577067	-3.386218181427	-4.377397040037
25260	H	-0.465904104609	-3.081107814391	-3.083112710858
25261	O	2.968668994931	-2.411149834586	2.562789794903
25262	H	2.198013675747	-1.891445068180	2.861988580498
25263	H	3.344617537599	-2.822973894114	3.341886935915
25264	O	-2.987608012045	-0.914973054192	4.464877273956
25265	H	-3.514028437923	-0.867526993797	3.648720653917
25266	H	-2.427998891465	-1.700563911031	4.367691344192
25267	O	-4.172510007155	4.692215402090	2.986482366182
25268	H	-4.482135545733	3.901921201354	3.455324880166
25269	H	-4.475807336184	4.596590239879	2.073766648517
25270	O	-4.163225153414	2.552277672500	-2.315882127033
25271	H	-3.590453521813	3.185559319481	-2.782691570094
25272	H	-3.578067574235	2.145390027387	-1.639512561945
25273	O	-3.698563215517	-0.884948355474	-1.898595489373
25274	H	-3.592231426391	-0.552769602177	-2.809025352516
25275	H	-4.470608075382	-0.366680697485	-1.573492107559
25276	O	-5.850086557562	0.538118640235	-1.197152636383
25277	H	-5.894525993784	0.851983885169	-0.280540928596
25278	H	-5.638430185162	1.316685008731	-1.725066838685
25279	O	1.228610006018	1.466071670295	-3.043382385489
25280	H	1.107787834048	1.771067336279	-3.971591624076
25281	H	2.112931861047	1.804102234613	-2.780216328624
25282	O	0.748150141277	2.964095125091	3.763568571045
25283	H	0.032979351339	2.318105713328	3.890508395816
25284	H	1.546822203687	2.413299016470	3.733070170924
25285	O	-4.722072980896	2.070848835349	3.871096609804
25286	H	-3.952576009442	1.893985594021	3.283320348030
25287	H	-4.677052847040	1.404220493074	4.557887334118
25288	O	-2.121685420560	-3.054093720973	-2.708209886721
25289	H	-2.415881436828	-2.889140038896	-3.634389630280
25290	H	-2.471357968517	-2.291402355407	-2.223981602213
25291	O	5.516167104931	0.525118139759	-1.834168938547
25292	H	5.729229574295	0.603390640103	-0.900058619864
25293	H	5.204573536044	-0.381140877675	-1.960128550696
25294	O	1.187976206270	2.412668608427	-5.545581508961
25295	H	1.830959375901	3.116409278569	-5.297021147338
25296	H	1.687749440978	1.679956335878	-5.940219923328
25297	C	0.249955516750	1.664247543317	0.847955706712
25298	C	1.344767921299	1.515178610001	0.070038086530
25299	C	2.009364758250	0.264400992386	-0.231380935099
25300	C	1.611913706370	-0.958937734423	0.228528310735
25301	C	-0.310257909865	-1.349874312990	-0.919680824611
25302	C	-1.244260435481	-0.576051737039	-0.248310944284
25303	C	-1.636063054054	0.705675136036	-0.818493352068
25304	O	-2.567015965835	1.449176459167	-0.437755813003
25305	H	1.721488764931	2.386123770573	-0.456909711467
25306	H	-0.213691584647	2.631064723319	0.977141806544
25307	H	2.781033114179	0.310213595227	-0.994838582093
25308	H	2.128874992276	-1.854330930332	-0.083837458810
25309	H	1.013150198089	-1.069013697260	1.121319893711
25310	H	0.032980554696	-1.042736233777	-1.893250195894

```
H     -0.183222252155   -2.393769517376   -0.677146813618
H     -0.120357556694    0.884356345905    1.493180108231
H     -1.142799885423    0.958714795064   -1.774479547729
H     -2.586612025696   -0.566186026425    1.341188492993
N     -1.722065897341   -0.956825344125    0.990372587861
H     -1.550303480912   -1.913446823034    1.272930876402

Ambimodal TS Water45-63
155
ANGSTROM
O      0.162645813196   -4.829136491420   -3.202180634219
H     -0.744892439845   -4.508601080267   -3.219548175534
H      0.632147546895   -4.262296662747   -3.825323829122
O     -2.045866683640    0.845337869867   -3.945320883099
H     -1.319335099727    0.488604077969   -4.495685649041
H     -2.651736288626    0.129066593643   -3.740751989098
O      0.836653669447   -2.431630725714    5.628370664241
H      1.656619620398   -2.154906552508    5.178938762354
H      0.992323756979   -3.307869168176    5.980300982056
O      0.413857323279    5.331933104662    0.246443343335
H      0.943866949831    5.436952939376   -0.570520954556
H     -0.430437380877    4.958610870900   -0.044753129706
O      1.392678782875    1.098000181610   -3.075676079779
H      0.783250687687    1.817093431162   -2.833036543385
H      2.274796731327    1.529444508831   -3.112121008870
O      2.635966409077   -1.513809151806    3.716994161504
H      1.761898224757   -1.340352189035    3.326602325046
H      3.060303261669   -2.132976125472    3.102626914420
O      5.465037492471   -1.309536679232    2.459507424569
H      5.581886628063   -1.403257064584    3.405769823384
H      4.892575172877   -2.044087713105    2.183944521322
O     -2.201920867496   -0.266480617096    3.761658909194
H     -2.488930964647    0.559673627866    3.344888487557
H     -1.596617002336   -0.011204610073    4.478807465814
O     -0.074107209706    3.334425572083   -2.492614407202
H     -0.625650996109    3.455795287116   -3.301503506471
H     -0.672905949450    3.556860945734   -1.757561483683
O      3.804207601184   -2.864852656620   -1.058380166327
H      4.480150579676   -2.200040359715   -0.842998488695
H      3.342772290796   -2.478536903038   -1.826282772677
O      0.968287265682   -4.072260527814    1.903117636891
H      0.636283463962   -3.190743686511    2.147634798527
H      0.973876572251   -4.124625846005    0.928142657838
O     -0.008190518088   -1.751831543030    3.063743587424
H     -0.004904186108   -2.136389523378    3.953877637992
H     -0.832818793610   -1.235631680697    3.019617291234
O      0.205075857962    0.333218579473   -5.335041718139
H      0.253993227909    1.139033272667   -5.869472396314
H      0.731252127104    0.553093977762   -4.532699230679
O     -4.641587071905   -1.630641434093    0.158337681779
H     -4.923622150911   -0.708803065113    0.268688642925
H     -4.508020475867   -1.939191099784    1.081090679476
O      1.971787253796    4.959498606476   -1.957290821484
H      2.725295033638    4.455225954145   -1.631890137580
H      1.284370119286    4.308482821497   -2.223407156509
O     -0.112132466648    0.332299406600    5.506263740172
```

25367	H	0.839736608841	0.584499998797	5.446232698475
25368	H	-0.080906199137	-0.553017019277	5.884130525998
25369	O	4.994684359666	-0.069958789480	-3.092029584626
25370	H	5.307968672467	-0.295129886360	-2.203937807108
25371	H	4.264771679274	-0.681503102699	-3.279047875350
25372	O	-0.725883275498	5.263273629746	2.956706727555
25373	H	-0.467714112347	4.396718761479	3.311641440819
25374	H	-0.213002179159	5.407245708485	2.153184089219
25375	O	3.781150266468	2.287516782288	-2.966837443596
25376	H	4.351788914947	1.494548433143	-3.099794549851
25377	H	3.990334025040	2.625852350579	-2.081638359365
25378	O	2.672918786364	4.725332290041	1.583395047698
25379	H	2.966308920993	5.560894009613	1.947991876990
25380	H	1.814336281670	4.896488734738	1.144466313754
25381	O	-4.003897982991	-0.882182700780	-2.597882691106
25382	H	-4.341948233032	0.029297224143	-2.568719147816
25383	H	-4.245476158510	-1.258814203807	-1.741599164233
25384	O	-5.178260502163	0.987128280162	0.939596200251
25385	H	-5.221616112299	1.937733494115	0.732784454390
25386	H	-5.058610106825	0.932802588751	1.889784054191
25387	O	2.570567996292	-1.476385786488	-3.096639933952
25388	H	1.910248283684	-1.937959769154	-3.647986043398
25389	H	2.163559034934	-0.618563880868	-2.907619518870
25390	O	-4.674422006405	1.708416142824	-1.923875060355
25391	H	-3.962035240797	1.620169058032	-1.259762661039
25392	H	-5.490972957316	1.746807189144	-1.420154147966
25393	O	3.586083613942	-3.304049010447	1.781298367969
25394	H	2.729139853796	-3.762838980029	1.913424742585
25395	H	3.713749349761	-3.293252884103	0.820337907097
25396	O	-2.735639263810	2.047409436477	2.375612498383
25397	H	-2.720678499745	1.726598205724	1.453804802170
25398	H	-2.992810865967	2.992138102813	2.304414560255
25399	O	-2.002090107136	-2.766727379650	-3.139599625360
25400	H	-2.175414373342	-3.151158539741	-2.264284365744
25401	H	-2.693971431319	-2.098303222372	-3.233729292324
25402	O	4.047613297245	3.220138209574	-0.348989144962
25403	H	3.664966933392	3.825752895283	0.305057994732
25404	H	4.311197280958	2.448969519091	0.172574806831
25405	O	-3.090635942269	-3.707449413434	-0.775839011921
25406	H	-3.601962570407	-2.937018570565	-0.463058962252
25407	H	-3.736097011822	-4.378714420019	-1.001696874448
25408	O	-3.707214263548	4.189120625258	-2.625642322925
25409	H	-4.114391056934	3.304020612244	-2.562296791737
25410	H	-3.155205449532	4.158916758020	-3.419651768623
25411	O	-1.871975324285	-3.933869380028	1.713007878199
25412	H	-0.980930331912	-4.283941006922	1.855971983479
25413	H	-2.122528752557	-4.131400972381	0.798997304191
25414	O	2.518331080323	0.813649295644	5.202074130662
25415	H	2.589454057361	1.493894914490	4.513199220543
25416	H	2.755321775709	-0.014850416362	4.760277001335
25417	O	-3.065176683977	4.647646349009	1.791383380985
25418	H	-2.376646489939	5.036834828684	2.370015375064
25419	H	-2.647337591532	4.604375684314	0.910489468404
25420	O	0.444032734987	3.012551325150	-6.134991928721
25421	H	0.622359705632	3.363505631227	-7.007475336664
25422	H	1.171751358458	3.349742054087	-5.559203158073

```
O      5.406486541415   -0.701211931743   -0.386006177870
H      6.096459911232   -0.986632153621    0.217749947328
H      4.888384462793   -0.051893363523    0.119101173117
O      2.443516727659    4.008482382090   -4.662847872468
H      2.258247839734    4.678325789240   -3.992739974350
H      3.036029185478    3.371585237398   -4.229063388422
O      2.316641333731    2.357540325498    2.953785529681
H      1.352614922659    2.375138374489    3.092284382999
H      2.537864465204    3.223581464725    2.577060429751
O     -0.359243053720    2.578630211855    3.662032894059
H     -0.349564274929    2.058854109035    4.474244412042
H     -1.188388597105    2.342786108625    3.199938284253
O     -1.978455315807    4.033291731478   -0.650257068602
H     -2.207996525519    3.092688096207   -0.465290892475
H     -2.611745528838    4.284297784218   -1.368284952736
O     -3.895896422175   -2.280660277467    2.688183977434
H     -3.258506738808   -2.993242386285    2.531158551403
H     -3.412337333002   -1.613101404544    3.195679476881
O      1.221841061900   -4.162684762333   -0.777615611351
H      2.147585242918   -3.968499497383   -0.962547642930
H      0.850558771871   -4.531724369206   -1.601524020049
O      4.323775454667    1.051587073334    1.493498618201
H      4.733971859231    0.355546330491    2.031895155359
H      3.578432680307    1.393159007629    2.014208417716
O     -1.765067479697    3.430222506571   -4.533661847725
H     -2.023430903313    2.499276683403   -4.371575733805
H     -1.118575750575    3.388034012082   -5.258060957447
O      0.407819474736   -2.322109192583   -4.505614470789
H     -0.433366449442   -2.394493060790   -4.022425444755
H      0.330576075499   -1.528507524939   -5.053179813277
O     -5.182787364261    3.663357413976    0.276082598757
H     -5.004683542113    4.100950969004   -0.561189453308
H     -4.663701017542    4.132233001150    0.945035592513
C      0.185759082908    1.733670169303    0.764014182027
C      1.303716703477    1.589747326131    0.017780378086
C      2.025263470014    0.342669869839   -0.184149203426
C      1.677389148051   -0.849432810686    0.356941780782
C     -0.300182824115   -1.311078368082   -1.037537962642
C     -1.239952082883   -0.591058682668   -0.342065189178
C     -1.571596763793    0.762466997081   -0.759558330081
O     -2.516632925823    1.457798975062   -0.280927375301
H      1.663635225754    2.441335850548   -0.554141378947
H     -0.307290094302    2.690980509275    0.855532033586
H      2.833394825938    0.376978339075   -0.910718245540
H      2.220079309837   -1.749460743349    0.119582531824
H      0.976121388871   -0.935172904884    1.173152539626
H      0.123021012923   -0.911897195242   -1.943712825021
H     -0.101677741272   -2.353084201824   -0.839167181466
H     -0.158547126421    0.974917274178    1.451485411228
H     -1.093740128893    1.123980804913   -1.679521481642
H     -2.661101904229   -0.590403344361    1.138983425487
N     -1.812490866724   -1.040813525637    0.839074233296
H     -1.756670284796   -2.022440762171    1.071362225725

Ambimodal TS Water45-64
155
```

```
ANGSTROM
O     -5.207701185447    0.176544488774   -2.249080674272
H     -5.148183568547    1.087306321616   -2.581365800789
H     -5.292689320771    0.249261214038   -1.290570236614
O      5.305963321609   -0.188233681406    3.007657015769
H      5.438457917168    0.795104578145    3.062958255609
H      5.998342711662   -0.591791764247    3.530694097875
O      2.794087253465   -4.325128040545    0.905201043216
H      2.103257449604   -4.391751682814    0.219251881453
H      3.069143165105   -5.224083987156    1.089217871902
O      4.008880650154    3.196768612502   -2.124489033816
H      3.361990519924    3.918417098429   -1.978241605805
H      4.369747666212    3.017876114032   -1.236635906196
O      1.401202648245    0.887499806888    5.667485596625
H      1.924455500236    0.359990235871    5.037106976828
H      1.693217350965    1.802272043902    5.566625695664
O     -0.468860161051   -4.180197493364   -3.429132032229
H     -1.444892501198   -4.078104756965   -3.457055236108
H     -0.245828532310   -4.911926208247   -4.006507128509
O     -3.019672216077   -1.252984764224   -2.729256636059
H     -3.856206197828   -0.751809489551   -2.584574706675
H     -2.452451541678   -0.695619826687   -3.287938859059
O     -1.147100026873    3.693748428836    4.187375692480
H     -1.351904073466    2.865464388442    4.651020627711
H     -1.683352037295    3.678336639370    3.372009988423
O     -4.791693974799    0.614995331292    0.548641296859
H     -3.867525272143    0.776750909026    0.226225119196
H     -4.731871367281    0.676765698327    1.506696633483
O     -1.383081651396    0.139541383114   -4.488958468633
H     -1.698414014099    0.293401051897   -5.381389239961
H     -0.743840667393   -0.628650350574   -4.565252234632
O     -4.922491526064   -2.162675135718    0.974748708169
H     -4.981237867164   -1.262037322810    0.621592379065
H     -4.274278542011   -2.628817482446    0.422425190748
O      0.809565176366   -4.590324466741   -0.960588259685
H      0.085476745134   -4.709433869107   -0.302906585822
H      0.359162883213   -4.505637323346   -1.814700896063
O     -3.118731147231   -3.731095268419   -3.658201095086
H     -3.130685344970   -2.783358516279   -3.374526708794
H     -3.543334632116   -3.766375752609   -4.515300080976
O      1.528989923331    3.495187039827    4.685102548685
H      0.568551968156    3.521655457650    4.493450025151
H      1.799262622082    4.413750263041    4.755819927972
O      2.345222346689   -0.742815667639   -3.342779299401
H      3.259758992754   -0.418849044607   -3.225950965251
H      1.784822584152    0.028907908542   -3.131349325753
O      2.960736118937   -3.295192792215   -2.451744138357
H      2.222643835753   -3.689533254309   -1.973378129404
H      2.672539301444   -2.418842714305   -2.752039874954
O     -1.218512230008    1.079718910842    5.116080968160
H     -1.655112549705    0.438141708852    5.718457847970
H     -0.281361246425    1.088437527872    5.421333597758
O     -2.920012624919    1.022601987126    2.752750866164
H     -2.769654287252    1.879684688946    2.311259915184
H     -2.415275199274    1.065135800776    3.574630148462
O      5.272455449409   -0.322869318161    0.169506266579
```

25535	H	5.106068522760	-1.242800517993	-0.135278512114
25536	H	5.347911321818	-0.401664827090	1.131399251582
25537	O	2.641178312230	-0.446699484144	3.567052277974
25538	H	3.602054113442	-0.386005913568	3.407652591320
25539	H	2.454588825571	-1.414186008309	3.485785546663
25540	O	2.683955454429	2.255241280509	2.615504284000
25541	H	2.272368377722	2.679509389567	3.397642089410
25542	H	2.559881083319	1.302279497871	2.738244597325
25543	O	-2.350448865467	2.855702160208	-3.817668675740
25544	H	-2.103730436391	1.927088417278	-3.878646784664
25545	H	-3.270656210853	2.872831361133	-3.509260900678
25546	O	-3.113516150250	-3.299216353480	-0.795228473442
25547	H	-3.407752196341	-3.907691704114	-1.479068658835
25548	H	-2.881275617881	-2.479361484134	-1.259388282374
25549	O	1.631461568252	4.614497566523	1.325044518295
25550	H	1.412601741744	5.121801610251	2.125748504044
25551	H	1.985417555079	3.765814541907	1.623467771915
25552	O	-1.218818996216	-4.629403646038	0.755617285618
25553	H	-1.040239997827	-4.125791163332	1.567478455413
25554	H	-1.945083266109	-4.176026187674	0.297499987924
25555	O	-0.161928699744	-0.448979416425	3.141820098675
25556	H	0.783264534074	-0.394162057794	3.329794196022
25557	H	-0.614195519037	0.047186939354	3.848136527358
25558	O	-0.751734809988	-3.027227572315	2.959965485175
25559	H	-0.585046129072	-2.057678883024	2.987889464949
25560	H	-1.390265297031	-3.184567000084	3.674271003593
25561	O	-4.220380010751	-1.406703940355	3.432308677197
25562	H	-4.509481568893	-1.788900793727	2.572229307894
25563	H	-3.789117931777	-0.567357929820	3.228278938993
25564	O	0.815186133650	1.513102712399	-3.254894550237
25565	H	0.111192854786	1.248793333826	-3.861469040187
25566	H	1.537500428877	1.906764076470	-3.801262053332
25567	O	-2.579031533075	3.601709809013	1.868182907013
25568	H	-1.937114200312	4.023049735574	1.238751245760
25569	H	-3.442477307605	3.852951597860	1.524641512424
25570	O	4.665999342846	2.305077429272	0.365923732277
25571	H	4.911134948965	1.372931440227	0.207107962099
25572	H	3.826136576791	2.276154702063	0.838036161376
25573	O	-4.439021593249	3.273334915438	-0.298648882361
25574	H	-4.948119395301	2.533814373284	0.066423396256
25575	H	-3.545381248105	2.897459345882	-0.417752315527
25576	O	4.664593836506	0.508581618187	-2.695786195015
25577	H	5.104747375996	0.156245528618	-1.914743177611
25578	H	4.498943431347	1.447541261682	-2.512448507716
25579	O	4.766136370425	-2.863064228423	-0.488983054900
25580	H	4.237899642195	-3.324139827677	0.177596402150
25581	H	4.281149956681	-3.007686864103	-1.326454075969
25582	O	-0.325817887352	3.804676769741	-2.234503705931
25583	H	0.175684460703	2.990011659385	-2.423524690683
25584	H	-1.101911770637	3.735426511582	-2.817140684231
25585	O	2.019282548273	-3.019226842656	3.247387288390
25586	H	1.059242626506	-3.168606099718	3.211003794393
25587	H	2.371727818546	-3.416496073673	2.434934667752
25588	O	5.409656635636	2.445642725510	3.015511004090
25589	H	4.440861322558	2.546889887699	3.042477481126
25590	H	5.633895968887	2.636351357513	2.092110727671

```
O      2.106971054802    4.997839214026   -1.471761153232
H      1.238583299920    4.758844683503   -1.828673380312
H      2.023663103800    4.987509378680   -0.508847713899
O      0.284184872214   -1.845606551609   -4.728035879088
H      0.071064374279   -2.645133511392   -4.215374157448
H      1.142784508634   -1.535428514706   -4.389788057952
O     -4.959184892995    2.874464547261   -2.862786099044
H     -4.803147819064    3.152961216657   -1.924672304797
H     -5.560164422727    3.511890362616   -3.248036542130
O     -0.899187663724    4.793414286165    0.241498041498
H     -0.816440615837    4.456613242606   -0.670750842001
H      0.008381349451    4.802257938781    0.593163414499
O      0.478835102830    5.891986628615    3.484240704856
H     -0.227559470662    5.283250795336    3.750605579758
H      0.049531042673    6.662936404618    3.110768029121
O     -1.963237166225   -0.989058879329    6.618394581399
H     -1.168531451554   -1.293288906623    7.059930768099
H     -2.243382916993   -1.710771672500    6.018577722019
O      2.985244963161    2.339438143207   -4.540015621366
H      3.400662644334    2.820954342010   -3.804667092492
H      3.553863041912    1.582012543530   -4.702412382979
O     -2.707670170230   -2.940536864108    4.873838644343
H     -3.266902538801   -3.643641244846    5.204429147850
H     -3.302554840975   -2.359601306955    4.315550843163
C      0.217008879898    1.609108936859    0.699561555777
C      1.349545880706    1.447054966866   -0.041262075322
C      2.056587515995    0.200992147931   -0.218597915030
C      1.665008613225   -1.004578695128    0.302656557424
C     -0.248946125936   -1.398668632407   -0.910136040599
C     -1.206143022330   -0.600705963870   -0.311381395833
C     -1.500521983450    0.745685427477   -0.805706143526
O     -2.492016122601    1.425188115361   -0.449282269370
H      1.697931510087    2.280114675998   -0.651018406433
H     -0.290013179792    2.559510589522    0.748066617984
H      2.858455759237    0.208305802264   -0.950257854578
H      2.214525507022   -1.904433077629    0.066341451739
H      1.039919698080   -1.081358733695    1.179390810278
H      0.172013992107   -1.117247107856   -1.861305246824
H     -0.167044179651   -2.444150667969   -0.656121610693
H     -0.096037969829    0.892095992531    1.443014939847
H     -0.930524522891    1.082803636040   -1.682944174517
H     -2.314584369482   -0.330746714682    1.405728633783
N     -1.962241942139   -1.036531622896    0.771899044427
H     -1.674269116149   -1.884014596149    1.233486218075

Ambimodal TS Water45-65
155
ANGSTROM
O     -2.987996797776   -3.620049351503   -1.136346402089
H     -2.644105008154   -3.070194772684   -1.856031566980
H     -3.773237128529   -3.169377633889   -0.794120159722
O     -0.229112561127    2.959829552188    3.824055564102
H     -0.454207613862    3.514436185858    3.072809828293
H      0.683916272201    2.636259954636    3.673450448343
O      0.967575710298   -4.351840619704    0.526153527287
H      1.751495330966   -4.361710744234   -0.046494600916
```

25647	H	1.305664551028	-4.157075835623	1.408816559968
25648	O	0.212463937925	-1.133824982864	-6.095945807262
25649	H	-0.044044220729	-2.042856722325	-5.821150759921
25650	H	-0.511622060374	-0.777001688145	-6.619690542462
25651	O	-1.679372881036	4.349375174891	0.937664451990
25652	H	-2.217901802026	4.322620797376	0.131702385417
25653	H	-2.195341836029	3.836580141053	1.583083176977
25654	O	0.378371549523	-1.071841433604	2.918284410227
25655	H	-0.422003005203	-1.185648863313	2.363314945717
25656	H	0.072867426811	-0.961473815570	3.839273996866
25657	O	-2.156097892120	-1.836471256739	-3.196026200396
25658	H	-2.923079518156	-1.330464372130	-2.853662741304
25659	H	-1.448241871334	-1.189941887208	-3.299601689624
25660	O	4.125785589522	-3.843356122932	1.550714067617
25661	H	4.599592484565	-2.999455982215	1.444648514066
25662	H	3.364876421461	-3.658642075229	2.121842713281
25663	O	-0.479619232776	-0.881236475920	5.459979967535
25664	H	-1.014589572664	-0.078742656263	5.341070292618
25665	H	-1.113370631920	-1.630404017596	5.410981398370
25666	O	0.119782361988	-0.020843907863	-3.714027830557
25667	H	0.167929206564	-0.378320668637	-4.638852252227
25668	H	0.777789129174	0.713031542956	-3.683290312016
25669	O	-5.187848070226	-2.452599142476	0.285214531782
25670	H	-6.105597426586	-2.716368596243	0.221206917738
25671	H	-5.180164362277	-1.466078079916	0.375202018450
25672	O	-5.060548398676	0.173915243398	0.711282277345
25673	H	-4.291938328281	0.385864097108	0.152712737923
25674	H	-4.710823594600	0.120174079976	1.617155083436
25675	O	-3.648198910642	-0.414617646950	2.986810868441
25676	H	-3.166389613376	-0.120294429805	3.766404732053
25677	H	-3.745530292225	-1.383625884098	3.046388124357
25678	O	-5.357474253036	3.022464357381	0.746096844771
25679	H	-5.603450116343	2.095670086107	0.615431380689
25680	H	-4.727257874073	3.012318998277	1.482663181403
25681	O	3.305374914981	-4.108969369456	-0.975367733172
25682	H	3.715037088628	-4.209817241351	-0.086950772328
25683	H	3.722401907538	-3.314021047734	-1.343058216044
25684	O	2.203161848783	-1.648821005983	5.290489910366
25685	H	2.672067964403	-1.479683044460	6.107639656026
25686	H	1.280010883860	-1.379288141543	5.441428967248
25687	O	-2.041772390103	1.283839173937	4.713831313220
25688	H	-2.439480026311	1.754388432885	5.447742875731
25689	H	-1.319038945187	1.876515325302	4.376107731978
25690	O	1.496906621957	1.413826912036	-6.445997607694
25691	H	1.721934154178	1.698084073178	-5.540023232142
25692	H	1.638203090173	0.460962911576	-6.466831057720
25693	O	4.340251056293	1.224063342129	-2.840811417735
25694	H	4.484634138778	1.751270956190	-2.029013417417
25695	H	4.509294592669	0.310710460479	-2.581450938871
25696	O	-4.339056324543	1.804948381176	-3.162977957719
25697	H	-3.554227227399	1.763769749570	-3.737255213604
25698	H	-4.149911822951	2.500958263993	-2.509414454269
25699	O	-3.832255556946	-3.107833285609	2.605611754523
25700	H	-3.088665124293	-3.626058773901	2.265710739016
25701	H	-4.409346519006	-2.980691570987	1.830797000596
25702	O	4.800321281195	1.676206867911	4.087258895017

25703	H	3.892559831265	2.003228278037	3.903328268724
25704	H	5.225952380987	2.319061277079	4.653723504666
25705	O	1.757931640247	-3.310833578705	3.069986790077
25706	H	2.140625396605	-3.195496174927	3.946964303780
25707	H	1.273383003583	-2.456396549779	2.914195248098
25708	O	2.273396073542	2.129359503353	3.230340609002
25709	H	2.311729448582	1.206265210540	2.920602822959
25710	H	2.470680159151	2.698446964924	2.455646689433
25711	O	-3.417437513295	3.397852173138	-1.078762209956
25712	H	-3.036739574739	2.548800931029	-0.759476235084
25713	H	-4.233085989684	3.492695345465	-0.542862817502
25714	O	1.934010759084	1.904421213086	-3.789288186530
25715	H	1.677615222404	2.714753684804	-3.313504858090
25716	H	2.845884467962	1.675134252957	-3.494441072700
25717	O	-1.571735142370	4.020634632888	-3.104783660622
25718	H	-2.228514079180	3.969861250163	-2.396309887875
25719	H	-1.698690273548	3.214872854541	-3.645053570316
25720	O	0.874514184573	5.047837022836	0.106947851317
25721	H	-0.023434169797	4.882558149999	0.436463931088
25722	H	0.891088124931	4.738209866926	-0.816804023886
25723	O	-0.624762100032	-3.399862121800	-4.938410534730
25724	H	0.005715197581	-3.832965840192	-4.351287522991
25725	H	-1.314802607389	-3.027754888094	-4.368381100765
25726	O	-1.112731738534	1.358770874210	-6.945746659191
25727	H	-0.137436208323	1.447649445406	-6.795784578983
25728	H	-1.331630535902	1.964410571772	-7.653259406339
25729	O	-2.182626730927	-2.869950087492	4.968273415053
25730	H	-2.964141586979	-2.952989033573	4.412160627782
25731	H	-1.584360100995	-3.585230162515	4.662924330672
25732	O	2.274174379149	-1.853210301386	-3.504587694768
25733	H	1.570270507262	-1.187930879622	-3.515905259737
25734	H	1.839079312913	-2.705171103701	-3.345198890932
25735	O	1.090550790616	-4.243961610570	-2.707670109040
25736	H	1.913531387263	-4.450615906473	-2.237493071486
25737	H	0.383253514188	-4.405715765356	-2.077797780585
25738	O	-1.903731921871	1.664472118941	-4.392881143940
25739	H	-1.767617334982	1.620989169882	-5.359606240001
25740	H	-1.238566965506	1.049125541469	-4.026243424097
25741	O	2.876069990092	3.841302526525	1.264439057309
25742	H	3.455927700550	4.556150845943	1.530758845268
25743	H	2.091885415097	4.277703645863	0.831723525603
25744	O	4.394762487837	-1.646109952949	-1.978834456088
25745	H	3.604962253773	-1.666618650229	-2.580665748369
25746	H	5.144475925376	-1.921330358449	-2.508432092553
25747	O	-3.100492983796	2.434846145403	2.279954863282
25748	H	-2.903250043820	1.951329011833	3.091499985852
25749	H	-2.889986555940	1.831177308058	1.545906973178
25750	O	6.033694566209	1.223337834374	1.556033724112
25751	H	5.682573440633	1.385431159292	2.443480043500
25752	H	5.878400533100	0.284081820755	1.366611899745
25753	O	3.272806493763	-0.424551192457	2.970968944295
25754	H	4.017581005519	-0.005094578400	3.418168261718
25755	H	2.799127745294	-0.917371622328	3.660679387445
25756	O	-4.162547794106	-0.529668921846	-1.988825841510
25757	H	-4.268862540389	0.361002342565	-2.417882851428
25758	H	-5.011607454136	-0.963450715968	-2.077660180203

```
O     -1.690285628090   -4.211422538919    1.127352692370
H     -2.192978781249   -4.167693763851    0.286887810637
H     -0.771116606687   -4.381113814904    0.861578053318
O      4.579858783258    2.396737941845   -0.434937347199
H      5.198774142558    2.019149553964    0.217859158411
H      3.914424602205    2.877185286530    0.077197481774
O      0.964613722934    4.160960315417   -2.516647796807
H      1.293523345995    4.912017981046   -3.012941766684
H     -0.009715991916    4.106032566431   -2.722439216025
O     -0.594851226149   -4.655062170894    3.738742241740
H      0.281292368131   -4.286779621391    3.533041733587
H     -1.043201089765   -4.763304883333    2.891957446678
O      4.932545793883   -1.276888253290    0.939162233942
H      4.698832372636   -1.211710714465    0.004579005917
H      4.177337633831   -0.948168017642    1.459608572861
C      0.261240841914    1.581197004857    0.694737823173
C      1.216290916926    1.492042854343   -0.266991836499
C      1.981628803699    0.303536933195   -0.580050222437
C      1.859230634859   -0.899432202951    0.055250015406
C     -0.183358497330   -1.566876971681   -0.808753915200
C     -1.137812249752   -0.849971628369   -0.122818937724
C     -1.598893576274    0.453555068312   -0.570231548469
O     -2.561668789463    1.078660628138   -0.055872324978
H      1.350961340178    2.330865928303   -0.947173148602
H     -0.316207203830    2.486103075384    0.823348226786
H      2.568653082078    0.353189358902   -1.491558000550
H      2.410528373207   -1.761815502146   -0.289707782486
H      1.456225347492   -0.986995630925    1.052136382948
H      0.112196400502   -1.269372012994   -1.798142256228
H      0.045260017298   -2.583913710914   -0.527293042252
H      0.152456370875    0.849101979933    1.482523811665
H     -1.198786312270    0.821537262989   -1.528892716789
H     -2.445590818986   -0.831286049180    1.471247333471
N     -1.630222065923   -1.314578840581    1.112500807255
H     -1.686668968884   -2.325169464787    1.208641091971

Ambimodal TS Water45-66
155
ANGSTROM
O     -0.747115314901   -0.497868129778    3.432607105951
H     -0.936276540109   -1.425807600068    3.654911659587
H     -1.582840475945   -0.120962622863    3.122132600236
O     -2.700903788933   -2.540087077506   -2.500958913173
H     -3.454942769315   -1.926761809444   -2.554482725723
H     -1.989665344959   -2.178297492350   -3.043872385800
O      4.374914464617   -2.217384352279   -1.169763141335
H      4.786938507659   -3.038973451550   -1.442116910121
H      4.452215603807   -2.181776433114   -0.188213928465
O      3.443666565270    2.793477048163   -4.437543417860
H      3.915177125402    2.882631828969   -3.593874505890
H      2.571799170102    3.192180297147   -4.295980957256
O      5.004915175128    0.096228881391   -2.559510922621
H      4.450020082772   -0.016526970636   -3.344700900706
H      4.842420089949   -0.675686793073   -1.992129559958
O      4.531881770641    0.225366878183    2.376835211210
H      3.620198773525    0.261561065615    2.707779649526
```

25815	H	4.625224098744	0.985355790043	1.764127785823
25816	O	-0.932393401685	0.755025212531	5.883628517131
25817	H	-0.879685219511	0.160371292768	5.113981381161
25818	H	-0.230697446615	1.402940136763	5.710216039476
25819	O	-2.800461871999	2.365995898581	4.839326256497
25820	H	-2.252669921895	3.085845214653	4.511860016754
25821	H	-2.207566803035	1.797966158838	5.385210363109
25822	O	2.408685487576	3.262595118601	2.421968829854
25823	H	1.752204441692	2.554277086954	2.420556187202
25824	H	2.922580061487	3.106318748785	3.243661076533
25825	O	-0.643690726966	2.588942753842	-5.691744321915
25826	H	-0.646052423353	1.633601198559	-5.552393777346
25827	H	0.010353951961	2.966235038589	-5.072657194640
25828	O	1.050978988778	5.063188365639	0.819662351098
25829	H	1.555551644370	4.968017121622	-0.008671614697
25830	H	1.537957352965	4.516347942093	1.457774701853
25831	O	-2.919426923463	3.067395841978	-4.382703640140
25832	H	-2.100450610403	2.950847010274	-4.928997861713
25833	H	-3.517916683259	3.592033374113	-4.915057892850
25834	O	1.305542648241	-4.403642331420	-1.430490078529
25835	H	0.423641721886	-4.651661480469	-1.762592485471
25836	H	1.615225887158	-3.684373666575	-1.990223026231
25837	O	1.033156216205	-4.359206921647	1.266940572967
25838	H	1.218571999838	-4.433733851197	0.311325692347
25839	H	1.489914844464	-3.563152871725	1.586955034985
25840	O	-1.309005493620	4.605399740595	-2.725813705971
25841	H	-2.032326010448	4.052050006580	-3.058292848883
25842	H	-1.442337246991	4.745574583704	-1.774904695354
25843	O	3.602080756495	2.185602204098	4.564942840073
25844	H	2.979374817140	1.441061727844	4.474014356523
25845	H	4.450826354091	1.846139216747	4.268593732938
25846	O	-5.283998448992	2.308686726779	1.883823217282
25847	H	-5.654130884821	2.634271344432	2.705448451518
25848	H	-4.541223464196	2.902097805274	1.658816023608
25849	O	-4.884631190911	0.227118240991	0.024653206175
25850	H	-5.423746491578	0.857812029599	0.515459656906
25851	H	-4.014392108209	0.673357023693	-0.088618175656
25852	O	-1.428734481003	5.384787933442	-0.073066806073
25853	H	-0.510731146578	5.225891389263	0.252850681755
25854	H	-1.506208725892	6.333792643268	-0.177526350126
25855	O	-4.884619781854	-0.878437648294	-2.412071971887
25856	H	-4.931672486411	-0.454829227993	-1.526416545453
25857	H	-5.666452281221	-1.427165169587	-2.489461305766
25858	O	0.865548535687	-4.398527046819	4.115717822597
25859	H	0.834262298569	-4.862411931135	3.269954419710
25860	H	0.015224742907	-3.947201971296	4.203826627865
25861	O	4.671721813032	2.630147901332	-1.923448030545
25862	H	5.554282995558	3.004781165883	-1.932603385191
25863	H	4.792333601365	1.659256123443	-2.094658530104
25864	O	0.649903792254	2.541819838607	4.594870611696
25865	H	0.055462774122	3.121298449583	4.084721754001
25866	H	1.349054012522	3.087558092447	4.957383006818
25867	O	-1.654544344556	-2.957233155898	4.301912282999
25868	H	-1.671095514813	-2.982438291958	5.260144001270
25869	H	-2.565916909670	-2.608528386531	4.043936613626
25870	O	4.763137658844	-2.207736434712	1.489787721840

25871	H	5.651831197283	-2.465796136656	1.737447440550
25872	H	4.670080968481	-1.256134582282	1.785648325982
25873	O	-2.929516042305	3.527257057664	1.238317638089
25874	H	-2.766064180795	2.753501690147	0.663911899379
25875	H	-2.629455329367	4.289306712586	0.716684565322
25876	O	-3.553228269308	-4.004604754594	-0.239911113572
25877	H	-2.829657479467	-4.019949630753	0.409752071636
25878	H	-3.260661832156	-3.422578108853	-0.953126978568
25879	O	-3.907018299958	-1.893530843362	3.644654851223
25880	H	-3.738583501602	-0.945497116660	3.492681367483
25881	H	-4.385539558790	-2.189190181595	2.837643960303
25882	O	-5.074451092636	-2.319708289971	1.280229679686
25883	H	-4.985397909529	-1.474000818452	0.817336721893
25884	H	-4.624009573084	-2.989760082357	0.729310720236
25885	O	-3.386832962289	0.351656802546	-4.437845184841
25886	H	-3.944339631304	-0.018482212107	-3.739430448984
25887	H	-3.351654808268	1.313176249145	-4.292781438622
25888	O	0.727086440785	0.831689681537	-3.169664279971
25889	H	1.596969074432	0.555852634552	-3.514792776316
25890	H	0.078555951710	0.465069046584	-3.800677872830
25891	O	2.299591982504	-2.478189579393	2.900273538340
25892	H	3.216601245395	-2.648899777872	2.653332279391
25893	H	2.000617014756	-3.151537081644	3.543731976019
25894	O	-3.266860146831	0.658232926137	2.880377552895
25895	H	-3.111591051785	1.286577788074	3.629218595532
25896	H	-4.035907435654	1.030024011339	2.419784524427
25897	O	4.530072573375	2.438200163516	0.887444257744
25898	H	3.763110325054	2.875858201339	1.297938535531
25899	H	4.421933725773	2.506775680580	-0.070566226488
25900	O	-1.529416254630	-3.738213510414	1.574800090900
25901	H	-0.598432975426	-4.021056994635	1.432437061599
25902	H	-1.674587336008	-3.723085370831	2.526240538068
25903	O	-1.024385464087	4.107127581077	3.107596312338
25904	H	-1.740602985056	3.813261197547	2.507515292862
25905	H	-0.463565508797	4.689023916780	2.586706047143
25906	O	2.026884551713	0.140246730909	3.567878162139
25907	H	1.081426326424	0.327000790846	3.631490212361
25908	H	2.077521669693	-0.824032417548	3.422311120290
25909	O	2.436535178689	4.440673906083	-1.479250486688
25910	H	1.896709854051	4.204339362569	-2.255297944235
25911	H	3.252919635937	3.935166527493	-1.554784945987
25912	O	2.999151126018	0.184208270201	-4.531743983226
25913	H	3.242672728405	1.143894499766	-4.573861465008
25914	H	2.558900669118	0.018261391123	-5.388574859858
25915	O	2.284304028060	-2.126794601085	-3.027990879019
25916	H	2.920320365353	-2.092723857890	-2.295537199045
25917	H	2.554962192724	-1.424895843794	-3.635643244944
25918	O	0.901635846101	3.441556902449	-3.557211914246
25919	H	0.106553957607	3.936422148826	-3.245706413384
25920	H	0.790792219137	2.509695440445	-3.249945965553
25921	O	1.354899403447	0.134020002300	-6.681599066736
25922	H	0.500913084248	-0.100097272016	-6.285415015804
25923	H	1.238116864278	0.993031557108	-7.093198911587
25924	O	-0.890445863830	-0.227333736477	-5.073333466598
25925	H	-0.732773232572	-1.161116281017	-4.851941691562
25926	H	-1.849642140203	-0.070876042949	-4.902594490622

```
O     -0.270449387713   -2.633875944456   -4.009499620790
H     -0.429969590138   -3.580124709877   -3.959713768642
H      0.617776170818   -2.483420679479   -3.645502777366
O     -1.258672056152   -4.903884366509   -2.315232590634
H     -1.833607466357   -4.121478436733   -2.355015582624
H     -1.714732888015   -5.523114821131   -1.741797719869
C      0.143915825499    1.686244836747    0.785178963371
C      1.259381287389    1.520427302180    0.029324477676
C      1.974039225134    0.273041257204   -0.168977985362
C      1.598394997820   -0.924431278774    0.357783903353
C     -0.361533733143   -1.366531588738   -0.943446498280
C     -1.335186929093   -0.593575184173   -0.346099065619
C     -1.594189811431    0.777540195499   -0.803086750350
O     -2.577522700317    1.466175136539   -0.440388689157
H      1.603968948509    2.362296611278   -0.568673765880
H     -0.353221809474    2.644379357560    0.835548328745
H      2.769696271964    0.297574715062   -0.910932929067
H      2.136993914168   -1.828615391213    0.115322595534
H      0.938992265367   -1.003490179394    1.209135550066
H      0.131989928551   -1.028591973261   -1.836736878527
H     -0.270188098765   -2.415561874431   -0.707027679605
H     -0.200759461455    0.954625678587    1.501569742795
H     -0.987652400860    1.147850600096   -1.642548549293
H     -2.673377777417   -0.439842482709    1.230981820784
N     -2.108394289035   -1.067835334123    0.686038332439
H     -1.934655088750   -1.989336871767    1.064713308391

Ambimodal TS Water45-67
155
ANGSTROM
O      1.639347603333   -4.655806137415   -0.147938830167
H      2.027727052524   -5.438392195603    0.245952089860
H      1.372821619008   -4.084724174717    0.597636240281
O      3.496427398821    1.301975167476   -3.907362171580
H      3.896980378713    1.921745907900   -3.266996554088
H      3.787886304410    0.411297592132   -3.664777089678
O      1.853950595033    0.922822779838   -6.094202050041
H      1.583786460133    0.084553992616   -5.689689526210
H      2.598128229727    1.224880797380   -5.551553342303
O     -1.855016909157   -4.044357350353    0.708683101751
H     -2.797351254683   -3.888011609034    0.448583638025
H     -1.446786760884   -4.397790198914   -0.104079840246
O      1.597543077212    2.938290835104    3.085958331216
H      1.873282573635    2.877991580718    4.010678498031
H      1.061406188568    3.769411345143    2.989914853314
O     -4.588968240455   -2.015140369532    2.026692638463
H     -4.691596947895   -1.198519211808    1.499018941539
H     -4.667355009587   -2.737960685295    1.390022556606
O     -1.743732785712    5.161542776805   -2.612185516871
H     -2.254647705467    4.304586437461   -2.603807967067
H     -2.340319433323    5.821915735255   -2.963613207828
O     -0.484350176255   -2.943700502235   -3.743340460377
H     -0.678286667333   -3.384725947683   -4.571942518718
H      0.268224905875   -2.332097021387   -3.910347175746
O     -0.510427550157    1.505833110093    4.027305190548
H      0.158101643024    1.892463298882    3.424774169488
```

25983	H	-0.026423251931	1.454584189824	4.869949268809
25984	O	1.378853496911	-1.057338843829	-4.111780102952
25985	H	1.066292571039	-0.254005917387	-3.669802186114
25986	H	2.253640809674	-1.237306493060	-3.719031422339
25987	O	-0.683353437723	-4.796231717727	-1.652729844641
25988	H	0.208215389433	-4.888471686281	-1.278836974959
25989	H	-0.612372730947	-4.130371081542	-2.356887564506
25990	O	-0.635167695598	5.018255325439	-0.074721754617
25991	H	-1.413048145474	4.535627759594	0.284660891814
25992	H	-0.945861034420	5.305114767576	-0.947746620550
25993	O	-2.534443788213	-1.166760128127	-3.248693661117
25994	H	-1.772306387236	-1.770263997743	-3.313406525555
25995	H	-2.286724982585	-0.357073275258	-3.719202335926
25996	O	-0.261347971045	-1.273129914651	3.583251150136
25997	H	-1.125544290489	-1.725573052520	3.719447836483
25998	H	-0.443977676361	-0.323894537859	3.632732098600
25999	O	-5.105880685652	2.431469796443	1.989967643018
26000	H	-4.371651069259	1.925461606708	2.382801223142
26001	H	-4.691026366905	3.135863085384	1.478791253350
26002	O	0.566765986269	4.150394701156	-3.631362025687
26003	H	0.394505919293	3.857862910681	-4.542831044023
26004	H	-0.256796937335	4.568100109883	-3.317926082723
26005	O	-2.721632515982	3.685569114402	0.960106692809
26006	H	-2.670375229606	2.803448615051	0.556251036608
26007	H	-2.541399898169	3.588713107859	1.911234088285
26008	O	0.586451482359	-3.309717020208	1.975115085919
26009	H	-0.263356063815	-3.693698586896	1.717823501020
26010	H	0.343156570814	-2.562740156267	2.548340385447
26011	O	1.684334901133	1.579151199752	5.595785722118
26012	H	1.957703625708	1.704667372745	6.504132596894
26013	H	1.904639270171	0.636043101531	5.383898641173
26014	O	-3.473244275801	-5.365172436402	-2.102903931113
26015	H	-3.680958878074	-4.774771352521	-2.852024409622
26016	H	-2.511984283648	-5.363208761989	-2.017268538368
26017	O	-4.814555995492	-1.581718653663	-1.655330568451
26018	H	-4.014404851950	-1.303348247878	-2.125130367983
26019	H	-5.001816694302	-0.895459494567	-0.995407650285
26020	O	4.233838905958	2.953641922009	-1.899560078495
26021	H	3.519800776846	3.572305353814	-1.700519168397
26022	H	4.543646271419	2.614867722745	-1.050662098378
26023	O	3.820284478505	-1.281626828760	-2.845555676826
26024	H	4.314349389467	-0.890699904532	-2.108934028562
26025	H	3.782189580378	-2.234049958464	-2.600835417346
26026	O	-2.174736652837	3.652567787783	3.670305355675
26027	H	-3.009944113830	3.592116046777	4.137112347128
26028	H	-1.644128172979	2.871896039129	3.922648876323
26029	O	0.005659221866	2.832970052461	-6.019659474138
26030	H	0.002386130376	3.239102169394	-6.886069767475
26031	H	0.697888182041	2.124611316786	-6.055433354232
26032	O	-2.999116209864	2.867835654188	-2.689403918778
26033	H	-2.557445584035	2.266834556129	-3.317607422339
26034	H	-2.973848879311	2.416495124738	-1.829975661515
26035	O	-2.597171775003	-2.536343919541	3.760677431849
26036	H	-3.245567152913	-2.345219761776	3.047369144977
26037	H	-2.465530936114	-3.502445352776	3.730585867896
26038	O	-3.023950636852	0.821707236962	2.819537241734

26039	H	-2.169059091540	1.002720317900	3.222031985433
26040	H	-3.533679524290	0.308307204671	3.485664839828
26041	O	4.996193622697	2.091216692486	0.761120861362
26042	H	5.823930784965	2.362302084824	1.160799482054
26043	H	4.356269501962	2.847564635275	0.933145478861
26044	O	3.745933950014	-3.684758863903	-1.704126615774
26045	H	2.919554195440	-4.037118619409	-1.343465439723
26046	H	4.283934425945	-3.466393478227	-0.918310217006
26047	O	-4.157789837830	-3.381362828235	-3.889550660430
26048	H	-3.586168342330	-2.603952602809	-3.786128018934
26049	H	-4.984887476122	-3.131183075623	-3.468492061109
26050	O	3.338990866155	4.014078965058	1.197085523117
26051	H	2.849427329942	4.314249078441	0.416918104222
26052	H	2.683636520084	3.675644366187	1.831069829341
26053	O	3.297813008801	0.613203778972	2.528705613234
26054	H	3.932739189690	1.013097969362	1.920513408110
26055	H	2.631506351965	1.285652460507	2.705665768794
26056	O	5.358170247535	-0.214468938677	-0.726012797718
26057	H	6.278832070378	-0.159083297793	-0.986006037075
26058	H	5.157364059918	0.573119120118	-0.194798839562
26059	O	-4.330065687989	-3.752634297738	-0.152472103724
26060	H	-4.189303220231	-4.480519201119	-0.797685442735
26061	H	-4.529861804498	-2.970113439373	-0.714029641217
26062	O	-4.832325973447	0.202145440620	0.427972073830
26063	H	-3.970566364622	0.582304627724	0.169108266724
26064	H	-5.271637567327	0.918961602284	0.918083255542
26065	O	-1.476116873323	1.210515584392	-4.306302247065
26066	H	-0.618204011839	1.242379607929	-3.837503990820
26067	H	-1.310710659752	1.711177622257	-5.116580385661
26068	O	3.297947156261	-2.127699005323	2.787411066537
26069	H	3.238850848564	-1.170237792275	2.618630117164
26070	H	2.464410156878	-2.526363069459	2.513368838750
26071	O	-4.358050586361	-0.633300789546	4.629585735456
26072	H	-5.073815627473	-0.969782858121	4.081493397299
26073	H	-3.719490310050	-1.363144166620	4.686134673796
26074	O	2.003574579464	-1.012808997219	5.120634606476
26075	H	2.705319828563	-1.391800242787	4.573745262068
26076	H	1.172190056890	-1.247842520881	4.670129659870
26077	O	1.871801556804	4.547841550500	-1.163529130809
26078	H	1.627480365098	4.557402191393	-2.099838350721
26079	H	1.035430613148	4.732344935030	-0.698103512405
26080	O	4.889420887573	-2.713051579707	0.577787306335
26081	H	5.063171287445	-1.822227039365	0.245056781359
26082	H	4.418892861241	-2.602494346401	1.423291350128
26083	O	0.934300558018	1.539247361992	-3.091989346250
26084	H	0.764660440893	2.503317821620	-3.117276424360
26085	H	1.868118330943	1.477102115856	-3.388678413518
26086	O	0.075590150278	5.039006702130	2.661266184945
26087	H	-0.066410391149	5.213020625209	1.718095782892
26088	H	-0.792303484636	4.830484795945	3.032710207920
26089	O	-2.389744680429	-5.163807782651	3.104628746337
26090	H	-3.202059058101	-5.666521431401	3.037919205394
26091	H	-2.153910328636	-4.900181358400	2.192038911081
26092	C	0.236321608133	1.574516481491	0.829099338902
26093	C	1.305829147825	1.531540408988	-0.001986672093
26094	C	2.064510357264	0.344478109007	-0.353720705513

```
C      1.837618643748   -0.900729572452    0.139466882683
C     -0.245031784399   -1.443831696421   -0.931800793108
C     -1.180310316826   -0.679754481820   -0.268078954378
C     -1.494375567181    0.663633553376   -0.756468628256
O     -2.498523269201    1.336535163183   -0.416115694711
H      1.566786774720    2.431758376404   -0.551132825170
H     -0.302469274152    2.496084833622    0.994571251117
H      2.769484318813    0.467904102634   -1.170904901872
H      2.397064800338   -1.755097180567   -0.213020426761
H      1.269642841037   -1.062423040308    1.042793506987
H      0.149458463737   -1.121913997857   -1.877225984259
H     -0.090746297752   -2.480298016461   -0.682478197774
H     -0.031382082556    0.756342790969    1.481010951075
H     -0.901270716663    1.022854468209   -1.610658775643
H     -2.469594938044   -0.512344703592    1.351810559590
N     -1.792473916510   -1.100984290760    0.889837069604
H     -1.799000899751   -2.082504007865    1.115111415998

Ambimodal TS Water45-68
155
ANGSTROM
O      4.866950393447   -0.110604610537   -4.027155174922
H      5.690251878706   -0.467049595678   -4.360406979310
H      4.334222607868   -0.889467446655   -3.707508178299
O     -1.741937598906    1.416693201604    5.929133193018
H     -1.568061037250    2.232210653028    5.435284989129
H     -0.873608938095    1.075219722437    6.180290812881
O     -0.596388553008    4.290241381149   -1.742324893845
H     -1.569142793770    4.227001388617   -1.702465987720
H     -0.298701079200    3.479403560459   -2.187229741741
O      0.614536946351    2.173787618033   -3.076326241713
H      0.786500837326    1.210750803006   -3.118100237092
H      1.496311956697    2.592335113170   -3.053994145697
O     -0.930413454205    3.466770365863    4.218853512611
H     -0.080283895019    3.054131392575    3.965873579997
H     -0.791625211683    4.414737309691    4.217109258483
O     -2.668078412710    3.258970059709    2.036229263497
H     -2.276957546165    3.180699451208    2.918162246774
H     -2.058170832021    3.863405559693    1.574338130121
O      1.625790623087   -4.181498492338   -1.995319972098
H      2.262119065336   -3.689418836229   -2.526265416428
H      0.771037280103   -4.100959718860   -2.469546677084
O      3.057016499131    3.352901895126   -2.917131082053
H      3.706540798489    2.779460226937   -2.483978144450
H      2.811660022400    4.063884511800   -2.287296422244
O     -2.173735730983    1.070269568802   -3.953712150601
H     -3.029795293520    1.275296151880   -3.538408225501
H     -2.007769339568    1.716528861041   -4.644757326013
O     -1.276251429135   -5.448090151441    0.972830447310
H     -2.005536010186   -4.813953669672    1.105056029896
H     -1.494490728036   -5.941289270313    0.180463423323
O      1.993532573289    5.138190580975   -1.221436393962
H      1.998030079139    5.103570659626   -0.260074838262
H      1.065946699305    5.037978558463   -1.490296982805
O     -0.561079843644   -5.363851365953    3.696518402416
H     -1.382600693371   -5.055285510019    4.091558898944
```

26151	H	-0.778718488993	-5.630090818134	2.790792082225
26152	O	1.374440349758	2.101751639130	3.642695089674
26153	H	1.414830140151	1.700434437002	4.544019176767
26154	H	0.936596576837	1.405002049844	3.125182718061
26155	O	4.660134963962	-2.932313265213	-0.878080780948
26156	H	4.300462009587	-3.235343038738	0.000723822041
26157	H	5.165258518393	-3.668802307140	-1.222518374036
26158	O	-2.651679916914	-3.375691354712	-1.330187844245
26159	H	-3.205571395035	-2.626281093751	-1.589550424016
26160	H	-2.861679104034	-3.540861840364	-0.403281841695
26161	O	0.541523427152	-3.014243434389	3.076642962261
26162	H	0.783823173541	-3.203662566577	2.153988697133
26163	H	0.330209532547	-3.900327378800	3.447341476252
26164	O	3.352458279184	-2.064640852056	-3.104531352421
26165	H	2.539955440955	-1.522328415139	-3.047598800139
26166	H	3.686611256355	-2.238115745145	-2.207348374809
26167	O	0.099201285193	0.030613128113	-6.631217664332
26168	H	-0.129974711850	0.944303450460	-6.361330302359
26169	H	1.060701138439	-0.011592526799	-6.573879156946
26170	O	-1.052680611082	-1.235045913182	-4.607005434508
26171	H	-1.523006277184	-0.436852892858	-4.275835801949
26172	H	-0.723208871677	-0.956019952849	-5.493328586081
26173	O	-0.726021304567	-3.724627426119	-3.229426343339
26174	H	-1.387571484028	-3.642389305251	-2.520993799090
26175	H	-0.754738915024	-2.892923598895	-3.720627483369
26176	O	2.723474931172	0.599382598768	-5.630866454910
26177	H	2.640716058651	1.567791100742	-5.690413101102
26178	H	3.582031930829	0.424193623968	-5.211522821481
26179	O	3.941989340424	1.610276889810	2.757194200281
26180	H	3.065214181873	1.842839086434	3.109744063532
26181	H	4.040138824106	2.077753591306	1.911168363104
26182	O	-4.362460540770	1.585182787852	-2.396164396683
26183	H	-4.572777961756	2.522878102456	-2.392720739029
26184	H	-3.810521338324	1.454082512011	-1.598851657430
26185	O	-3.231666127385	-3.582520665028	1.432413501017
26186	H	-3.014712841336	-3.416934408143	2.375253138668
26187	H	-3.938226648515	-2.941041270071	1.213688914293
26188	O	4.830534014518	1.503829251113	-1.760230428443
26189	H	5.094392597973	0.821756640939	-1.103320991972
26190	H	4.886525656275	1.040339312228	-2.614033011719
26191	O	-4.823349603412	-1.532361629796	0.618054584590
26192	H	-4.763853849724	-1.441628537576	-0.345049514027
26193	H	-4.638269032657	-0.660458511198	0.990691687026
26194	O	2.568774057904	3.352906203726	-5.587405978006
26195	H	2.755988556321	3.448605350415	-4.632673603818
26196	H	3.160556571166	3.949950646841	-6.044671519381
26197	O	1.928826431428	4.229152556536	1.714533562386
26198	H	1.752684574409	3.623354778071	2.444464108128
26199	H	2.671112794154	3.837046330457	1.230482003376
26200	O	-2.629175726937	-0.264192709156	4.046428584028
26201	H	-2.474178889721	0.338606205724	4.806878100610
26202	H	-3.216088560380	0.189408503499	3.415703549498
26203	O	-4.088857868978	0.932726500711	2.058435193861
26204	H	-4.909194213462	1.347053457019	2.329207120006
26205	H	-3.502571910077	1.665434829090	1.774191681470
26206	O	1.292019967416	-0.379332546282	-3.575660449082

26207	H	0.479423991585	-0.792653594174	-3.936064242329
26208	H	1.788469153623	-0.059662772428	-4.362521533079
26209	O	-3.174134855054	3.855388890687	-0.991226882158
26210	H	-2.926732767008	2.968497080504	-0.652652658222
26211	H	-3.342656294027	4.388039950087	-0.212048387046
26212	O	4.245572769119	-0.973698859602	2.572458179851
26213	H	4.099923150767	0.005886231196	2.664508404740
26214	H	5.079548735101	-1.156389454980	3.010160996275
26215	O	4.158250056768	3.081390999222	0.412040020821
26216	H	4.911886898705	3.672757707928	0.447838061645
26217	H	4.298871155887	2.510813842480	-0.363345125658
26218	O	5.388466287844	-0.451460859063	-0.004643426788
26219	H	4.929755909156	-0.528182715607	0.844480733736
26220	H	5.260617804708	-1.316400874949	-0.429420108161
26221	O	-0.221869753351	2.541020985393	-5.603245047933
26222	H	0.536100916666	3.020871563743	-5.951669007640
26223	H	-0.012176783406	2.422084274864	-4.649710176147
26224	O	-2.251507745076	-2.947816161798	3.838034483979
26225	H	-1.309186593414	-2.856139813788	3.634220299927
26226	H	-2.556913288390	-2.049704457052	4.050961196367
26227	O	-0.717781067953	4.883796043168	0.968822284699
26228	H	0.188203013475	4.779083034459	1.294580170445
26229	H	-0.665182874874	4.776914280366	0.003451051242
26230	O	2.477673374013	-1.666145315534	4.682428745906
26231	H	1.834410304970	-2.264172269598	4.277316203394
26232	H	3.085217948061	-1.422287335747	3.969800990617
26233	O	-4.326161294112	-1.267817463638	-2.153930496367
26234	H	-4.199034042878	-1.698648672966	-3.024440019838
26235	H	-4.500165213619	-0.339927399305	-2.348107538948
26236	O	1.152422054086	-4.120031405895	0.645214441006
26237	H	0.408055274411	-4.733355745032	0.756005962381
26238	H	1.379330256082	-4.157073596790	-0.310177678735
26239	O	0.970246800817	0.446758147250	5.672222897822
26240	H	1.685079175469	-0.192001190748	5.800190268320
26241	H	0.484218904672	0.068023428376	4.904715644871
26242	O	-0.073266909835	-0.306984870245	3.321328750665
26243	H	0.172336395215	-1.236667469136	3.211182660031
26244	H	-1.045605730777	-0.295730546428	3.473103167611
26245	O	3.706923647856	-3.594683440002	1.465648869614
26246	H	2.789565757022	-3.880367587989	1.302668738773
26247	H	3.670766619188	-2.752212193858	1.935852947323
26248	O	-3.489443453194	-2.571649307533	-4.377494645352
26249	H	-2.653688555215	-2.123454290309	-4.598572794588
26250	H	-3.245083835813	-3.455273743015	-4.091397840939
26251	C	0.164272889267	1.636615759182	0.722089402138
26252	C	1.272536468985	1.556751475808	-0.063215758877
26253	C	2.027142258284	0.352341277012	-0.322614952008
26254	C	1.750734607066	-0.869483161952	0.231192984146
26255	C	-0.206622704513	-1.428037281724	-0.815479622518
26256	C	-1.179736981729	-0.656548159339	-0.210860616884
26257	C	-1.578115279650	0.645399464103	-0.746112705267
26258	O	-2.594420906771	1.275119611287	-0.365417356664
26259	H	1.556804990859	2.432016161830	-0.643424272764
26260	H	-0.390706092704	2.556224971884	0.816403242631
26261	H	2.742163908417	0.406292548751	-1.139580074455
26262	H	2.312352352631	-1.746296972121	-0.056659317926

```
H      1.222729173426   -0.960531370469    1.169056065326
H      0.161694163308   -1.163116164688   -1.789927281692
H     -0.071162994507   -2.458580305210   -0.525960077551
H     -0.116164966157    0.867389630343    1.423907441595
H     -1.071168955549    0.980546875247   -1.664670641120
H     -2.562436791719   -0.489494128488    1.281514515620
N     -1.763158664470   -1.031245553080    0.991325248354
H     -1.817024074027   -2.011874563583    1.216025980006

Ambimodal TS Water45-69
155
ANGSTROM
O      2.641697802196    3.883727232201    1.843534245815
H      3.381979060458    4.278310459310    2.308531592651
H      2.228927584818    4.615597384820    1.328398622771
O      0.175431325444   -3.098473721158   -3.551984773032
H     -0.158275425923   -2.267638482142   -3.925753327759
H     -0.609518318092   -3.667881339380   -3.461574870033
O     -2.167432797356   -4.535218891950   -3.162217158370
H     -2.758968263024   -4.585937945277   -3.913801881057
H     -2.652334721283   -4.041303676044   -2.471243614178
O     -3.358631066414    3.500721266077    0.968459568256
H     -2.995483405371    2.712043039654    0.535382032167
H     -4.138876688011    3.157744653529    1.465419295620
O      1.236520782387    0.963461213418   -3.277257532139
H      1.248115931793    0.732604687900   -2.341431798154
H      2.134700947514    0.676847176808   -3.616612381257
O      5.077369447408    0.759039813338   -1.667800405512
H      5.089937582756    0.502115553444   -0.715086320651
H      5.955691868201    1.088466669977   -1.857183297365
O     -3.462952278951   -2.467702646375    3.248871220426
H     -3.350722182759   -3.442888378958    3.226903309138
H     -2.572292214329   -2.133037648739    3.445348473448
O     -2.787877392241   -5.029579000999    2.838020569341
H     -2.521238270277   -4.925334472443    1.905997212687
H     -2.005333718447   -5.357852295554    3.297537196197
O      1.555439176738   -0.423646500136    3.368225887548
H      2.003149890030   -1.284520684728    3.263788816064
H      1.518197144699   -0.233058184658    4.331257968382
O      4.509804461637   -2.661812690575    1.105352830920
H      5.262872997786   -3.118169002592    1.482968160433
H      3.771933322525   -2.757727333504    1.748227197185
O      0.768406383229    3.571767893562   -3.019291319614
H      1.427276767299    4.065267943054   -3.508498257154
H      0.964597488551    2.616269895345   -3.161870819512
O      0.866888890009   -6.375153677863    1.097322721800
H      1.076022332957   -5.597004100704    0.564220327226
H      0.261311371991   -6.892628498208    0.559760666969
O     -2.317289231241    1.437416208854   -5.407451957410
H     -2.359651963222    1.833281881064   -6.277984609794
H     -2.178790204399    2.183983347163   -4.776135737879
O     -0.881509901291   -1.639791385553    3.612842416268
H     -0.261044790302   -0.901753672285    3.495430436649
H     -0.545147394653   -2.342118035024    3.009262076538
O     -0.832772941462   -6.280345508067   -1.383279492259
H      0.000655911271   -5.771312448191   -1.399787383047
```

26319	H	-1.336594912528	-5.961191994686	-2.143415541257
26320	O	2.492978876636	-2.926883868892	2.840117075978
26321	H	2.452079094805	-3.485940698679	3.636707870468
26322	H	1.682518433651	-3.148080317064	2.338279818016
26323	O	-3.079864961238	0.460187156363	2.976149118948
26324	H	-3.418203900255	-0.355513231970	3.358050523902
26325	H	-2.317295591954	0.734235760915	3.513855185076
26326	O	-3.412684403873	-3.032154622730	-1.273791451322
26327	H	-4.013024198568	-2.780290376624	-0.549526982832
26328	H	-3.194678151531	-2.186422471584	-1.730732628902
26329	O	-4.838065404373	-2.300912187482	0.968719901932
26330	H	-5.609685074723	-2.825702411059	1.183374253716
26331	H	-4.281228680136	-2.296099315998	1.785167862500
26332	O	-3.296537161437	4.517889353250	-1.558241762122
26333	H	-3.358969639671	4.319231600319	-0.601907889636
26334	H	-4.048044067348	4.037515777925	-1.933103580191
26335	O	-1.019825716063	1.523545454073	4.538844033323
26336	H	-0.439859010767	2.154318402028	4.049822705225
26337	H	-1.562804812957	2.069153812189	5.110469249108
26338	O	2.567047025056	2.405121829013	5.140432454150
26339	H	1.858864818510	2.937808896001	4.756136324827
26340	H	3.073622580968	2.053822448597	4.386298810208
26341	O	0.024358580895	-3.516577139785	1.989118903211
26342	H	-0.542368981045	-3.794667186205	1.237797981951
26343	H	-0.046953348140	-4.274429143985	2.616747203677
26344	O	1.084759691749	0.300248257565	5.889352505678
26345	H	0.234064956514	0.666536134854	5.589066410247
26346	H	1.710313397086	1.056223370840	5.858915523746
26347	O	-4.765948499501	2.195955885395	-2.066578698602
26348	H	-3.906178446409	1.938036788443	-1.687886545969
26349	H	-5.410700354268	1.773561363744	-1.487871677016
26350	O	-4.599055434391	0.429143019932	-4.156241373809
26351	H	-4.803811455370	1.140061383856	-3.520355183728
26352	H	-3.904340329655	0.785196835204	-4.732080816117
26353	O	-5.197150865475	2.052560123223	2.199671838404
26354	H	-4.505696777737	1.549105972899	2.660518712921
26355	H	-5.426081893574	1.484664271394	1.439700333474
26356	O	0.316336568755	-2.407231916464	5.924477589649
26357	H	-0.277388939423	-2.197058325990	5.174912540904
26358	H	0.705224169383	-1.559205481929	6.178731133531
26359	O	-1.882010351100	3.513145863366	-3.815881551531
26360	H	-0.949091358549	3.583433227485	-3.559161454336
26361	H	-2.400925912965	3.840737954275	-3.065420527616
26362	O	3.460515240539	1.338385809238	2.699246552297
26363	H	2.720251525319	0.701599221184	2.812547354981
26364	H	3.118959962331	2.071658427289	2.171220508037
26365	O	1.863709259704	-4.345377462373	5.074040079813
26366	H	1.265589857391	-3.631807931386	5.431593167063
26367	H	2.334540610522	-4.697682886567	5.828823818963
26368	O	-0.061774843361	-5.651396285740	3.553304879208
26369	H	0.344188830037	-6.139454544828	2.812248937666
26370	H	0.649211863798	-5.404333094374	4.166624103631
26371	O	0.468394739401	3.456810795292	3.458436457005
26372	H	1.226767705417	3.461141110367	2.845887755797
26373	H	-0.201396673141	4.013560090417	3.006441805697
26374	O	1.141673087649	-4.453736564950	-1.129497821478

```
H      2.122617567784   -4.398708555974   -1.124673259085
H      0.857947669625   -3.917928607158   -1.881698019115
O     -2.833014182165   -0.842346410266   -2.631792333478
H     -3.587081601928   -0.479637139465   -3.153868394246
H     -2.079926193311   -0.861568588600   -3.245629988556
O     -1.803893686651   -4.452589948660    0.337984306340
H     -1.513699989599   -5.209856734601   -0.211966983211
H     -2.364348322368   -3.894543552770   -0.237955889907
O      3.580025812590    0.024195233001   -3.836668737348
H      3.423738055473   -0.930123542041   -3.737235853770
H      4.152531754815    0.283885095119   -3.090635158081
O     -5.123757489375    0.322740346335    0.090259951249
H     -5.129305481253   -0.604140198169    0.374550527337
H     -4.189452610812    0.528118608392   -0.080996784555
O      3.756740087694   -3.960000139175   -1.227424251697
H      4.065854782155   -3.449578792940   -0.459821434981
H      3.685527991315   -3.345826242168   -1.972542999323
O     -0.539881368655    4.820099355722   -0.883012538521
H     -1.454332546688    4.904655960611   -1.191039390109
H     -0.068041242433    4.327748871472   -1.571431921465
O      5.167937101784    0.060602936209    0.909051650872
H      4.912693335253   -0.873167006560    0.969851996837
H      4.630646081069    0.535444028780    1.568868602973
O     -1.216665625676    4.850140051067    1.881564107332
H     -2.099824729958    4.444300150832    1.796526684109
H     -0.896217226243    4.871815617729    0.967768919454
O      2.884329405424   -2.650837488354   -3.557788327998
H      3.221543792928   -3.174698264879   -4.286116783294
H      1.919780357125   -2.797766413464   -3.546192979399
O      1.393079587511    5.962419158497    0.714657899175
H      0.815525398183    6.279622989098    1.414807747523
H      0.799153895222    5.668561566617    0.004076537212
O     -0.724579131198   -0.583446556155   -4.400399792764
H     -0.011249136435   -0.009598044500   -4.062280561117
H     -1.288012746022    0.010344052239   -4.924898194045
C      0.064885839674    1.690184730905    0.841445983571
C      1.188891261105    1.680562642448    0.080706645320
C      2.009050784177    0.515040264510   -0.200039086137
C      1.737886472103   -0.752599072473    0.244395089002
C     -0.180030835372   -1.351885382108   -0.875466412084
C     -1.201272032584   -0.595637318805   -0.324069284913
C     -1.585894608047    0.705060361329   -0.881467712745
O     -2.609069491533    1.337916589390   -0.558664891076
H      1.458549948910    2.588932574013   -0.458663896657
H     -0.533671153691    2.581689131898    0.947280523697
H      2.811126879615    0.657193048033   -0.921912775589
H      2.360503482366   -1.582589495165   -0.058894914164
H      1.151922047728   -0.929186340357    1.131763221190
H      0.214474922043   -1.112777890802   -1.847732739920
H     -0.042974487552   -2.376940054505   -0.565593892176
H     -0.188483086165    0.890017520676    1.520848964867
H     -0.992779642036    1.082926112664   -1.733854962089
H     -2.479633108356   -0.390094195105    1.308265795615
N     -1.926645011556   -1.033491499205    0.757917136208
H     -1.705716627705   -1.912923177135    1.187534132427
```

```
Ambimodal TS Water45-70
155
ANGSTROM
O      -3.583975023199   -0.536110536564   -2.474609722110
H      -3.393513193406   -1.491291130433   -2.431856751192
H      -4.239607986592   -0.374036054128   -1.780251301396
O      -2.894621347558    3.105178543478    1.678162057480
H      -2.647561184852    3.918915169787    1.184071498870
H      -2.731503713427    2.381319121852    1.053214590299
O       1.331010081399   -4.157798190130    2.910894938775
H       1.267993342383   -5.079057074149    3.165694363267
H       0.400508069426   -3.815786392426    2.847871314644
O       1.146240873044    5.392797490783   -1.325523404792
H       0.341985454849    4.995431346094   -1.705842261300
H       1.049920560955    5.338787546113   -0.358219516229
O      -0.901462965926    3.908311865728   -2.323843915748
H      -0.428539125473    3.420463630221   -3.013033329183
H      -1.855866186765    3.706838308213   -2.424840360612
O       2.537180389095    0.819649564478    3.080507149459
H       1.703811554381    0.345330721705    2.893170040089
H       3.229956785320    0.322658241177    2.608419370598
O      -1.394962325952   -4.803063484510   -0.532234118186
H      -1.434369096563   -5.637490072308   -1.000935158591
H      -0.452533374697   -4.681206712101   -0.279360655629
O      -4.492916163899   -2.827387997769    3.666353906261
H      -3.804702891882   -2.947524492845    4.336795057801
H      -4.320262499670   -3.480891166216    2.977290478332
O       1.854090007280   -2.246843948364    4.733932423818
H       1.789402055417   -3.009786124784    4.118219208906
H       2.776513879556   -1.990501040815    4.758964211137
O       4.722009409842   -2.720629319360    0.251563248740
H       4.055205361676   -3.136034476439   -0.307270084271
H       5.382892643815   -2.367661830964   -0.387842144676
O       3.906918840561   -1.226316947385   -3.317301210699
H       3.864443915097   -0.285276306919   -3.075444125405
H       3.619623754903   -1.256954073459   -4.240082925479
O      -3.060886309324   -3.192080190167   -2.106455727080
H      -2.383567563436   -3.643184961477   -1.579995005892
H      -3.845043958207   -3.142963402212   -1.514882665695
O       3.333206070518    1.839603488217   -5.304487976542
H       3.880708151657    1.733081870308   -4.511531664307
H       2.482293357193    2.194467958428   -4.984822329418
O      -0.491518561312   -1.666493251459    6.365653792273
H      -0.763180062879   -0.790854908073    6.030493728222
H       0.366591131879   -1.862073250815    5.967633676308
O       5.552458765130    0.992796357644   -0.390118430783
H       5.099670053895    0.507964094315    0.320264239913
H       5.415138708542    1.943083510540   -0.183748559532
O      -1.089292883185    0.638604592422    5.046294362088
H      -0.500242520370    1.390321906861    5.193704792353
H      -1.955188870584    1.009115631536    4.721632633467
O       0.222450412242   -0.691362795335    3.205472585192
H       0.811969518464   -1.210811900816    3.783951741762
H      -0.348241207225   -0.169930789379    3.823783265569
O       1.102717645805    2.450378044787   -3.761410565061
H       1.702361164066    2.796891361034   -3.083642344447
```

26487	H	1.021432755339	1.486559574486	-3.630407591885
26488	O	2.986779391531	3.327210274110	2.181503470090
26489	H	2.775031456908	2.402775151715	2.433759146488
26490	H	3.425706752802	3.734520347631	2.929259882624
26491	O	0.967372311790	-3.000690239884	-3.907475425005
26492	H	0.955728919587	-2.021232791293	-3.820053938629
26493	H	1.444582711949	-3.187377930551	-4.717696170868
26494	O	6.160449379710	-1.480720570536	-1.635203246499
26495	H	5.513886436763	-1.469350727152	-2.357601037314
26496	H	6.200319690998	-0.576642344007	-1.295323718453
26497	O	0.845536066869	4.957168856477	1.350833625668
26498	H	0.243740277502	4.758724051574	2.099564214631
26499	H	1.635993423124	4.430124384090	1.533954030245
26500	O	2.822130058618	-0.673763790555	-5.880747895184
26501	H	2.935810864853	-0.868621092958	-6.810637000353
26502	H	3.077648327659	0.280858165673	-5.764752445326
26503	O	-3.845097957923	-0.544889968392	2.426749132462
26504	H	-4.168134531182	-1.335223065752	2.910193753459
26505	H	-4.514018550588	-0.321385207741	1.760167444733
26506	O	-3.281316108947	1.566891744981	3.984913118597
26507	H	-3.229726213270	2.261502600505	3.313608005922
26508	H	-3.568809805017	0.770073371357	3.492819666481
26509	O	4.210318411679	1.423171976261	-2.693190712220
26510	H	4.830934357098	1.246446778221	-1.956762301172
26511	H	3.763428376601	2.245966187989	-2.446164843600
26512	O	-1.760855862375	-3.005951626109	-4.566109942082
26513	H	-2.332459093531	-3.175058829279	-3.801895772152
26514	H	-0.854811067625	-3.185534278504	-4.268089486477
26515	O	2.776577198510	-3.272006633644	-1.834951990230
26516	H	3.275299793830	-2.541211764423	-2.243111311971
26517	H	2.057303564537	-3.418132796615	-2.471455822465
26518	O	-1.058303006224	-3.053497984432	2.924053794118
26519	H	-1.478255791852	-3.180045929585	3.797201356886
26520	H	-0.766655237917	-2.116037620938	2.897355935687
26521	O	-3.467257996713	3.126413353691	-2.122307001002
26522	H	-3.779426499752	2.664393899034	-2.923780517219
26523	H	-3.155599230660	2.434069983011	-1.509951626831
26524	O	-2.242157334076	-3.318852253317	5.325309435296
26525	H	-1.656439136665	-2.670525790208	5.799818830296
26526	H	-2.414780469127	-4.029425997917	5.942841611968
26527	O	-0.746573541739	4.043527398126	3.302284758893
26528	H	-1.532451654356	3.637667607353	2.904336659485
26529	H	-0.288303140295	3.370541498812	3.819842090417
26530	O	-5.333730165152	2.825591349034	0.033998237445
26531	H	-4.909616324427	3.167442232322	-0.767714478390
26532	H	-4.760702463248	3.092263152751	0.764662709875
26533	O	3.009713362914	3.882827090425	-2.212879548969
26534	H	3.443599945099	4.414681177653	-2.882786878030
26535	H	2.292744826697	4.488754388169	-1.829468191810
26536	O	1.026352912543	2.395574756988	4.841467178330
26537	H	1.640782628662	1.861331127158	4.314242873637
26538	H	1.542834491868	2.928795788965	5.445569209331
26539	O	-4.881906807063	0.162426980482	-0.016353703601
26540	H	-3.954081792721	0.472863058643	-0.058301396015
26541	H	-5.381851683930	1.001915728375	-0.017914581223
26542	O	0.844194398581	-0.273373353156	-3.993047968442

```
H      1.379834797169   -0.326784630402   -4.799508523176
H     -0.108121101365   -0.227859510057   -4.274108182103
O     -3.170709762041   -4.102206821015    1.512848258602
H     -2.438344629435   -3.719906436230    2.026222607610
H     -2.741450556652   -4.527108436946    0.756097824757
O     -4.074382668652    1.529793671775   -4.246432288771
H     -4.133299349343    0.717813530887   -3.718240017642
H     -3.380813927467    1.367558720310   -4.891539762015
O      4.686963422477    3.491925266448    0.007456340874
H      4.137818162703    3.722942253295   -0.758315835569
H      4.080613240510    3.471742538388    0.765542923159
O     -4.882683648995   -2.703807744721   -0.222799675755
H     -4.982743155322   -1.756675627212   -0.059217517158
H     -4.408670753039   -3.083642964437    0.533664916869
O      1.185575553443   -4.538550204132    0.111708957064
H      1.458172696638   -4.281706668540    1.004058205819
H      1.809217969797   -4.136261381514   -0.510246483712
O      4.538502026877   -0.553260728482    1.742799025078
H      5.364314092599   -0.626294028851    2.224704694507
H      4.477495796982   -1.389031580480    1.208469667509
O     -1.709959159961   -0.337686651335   -4.562492305809
H     -1.792650294061   -1.299332304288   -4.759028325550
H     -2.234798580838   -0.221613493011   -3.758619687778
O     -1.859820472336    5.071262855849    0.201882917693
H     -1.005796068002    5.324097186539    0.570001296837
H     -1.694433361225    4.751473261600   -0.692543696400
C      0.203022651729    1.653099521067    0.744962068041
C      1.332269613302    1.534221173865    0.001410090006
C      2.071977711148    0.307560574191   -0.233152769916
C      1.729434358718   -0.919929042169    0.251826961148
C     -0.128013908040   -1.386251307060   -1.137834278171
C     -1.138273792988   -0.726654191470   -0.468016079844
C     -1.477952683330    0.654423826183   -0.810999871572
O     -2.473465225676    1.274175949684   -0.363158070326
H      1.668290655718    2.404940753294   -0.560497067829
H     -0.312155487018    2.599820579575    0.817770460336
H      2.859192987974    0.361846604966   -0.979581471347
H      2.297103333170   -1.795981577985   -0.017181163673
H      1.066870935015   -1.052093196401    1.094942848587
H      0.331037970265   -0.946771835598   -2.004227397596
H      0.025437737381   -2.446623589504   -1.008795176688
H     -0.137548216416    0.891897983639    1.432327558899
H     -0.937717718483    1.094934731879   -1.665316169151
H     -2.483597863688   -0.845380101301    1.151972685691
N     -1.844926368861   -1.335454949962    0.539584533482
H     -1.631984871267   -2.286053343464    0.777266160514

Ambimodal TS Water45-71
155
ANGSTROM
O      5.764010535112    0.529090508572   -3.501958806268
H      5.247955533628   -0.251075106184   -3.739156739263
H      5.182296280047    1.273425043418   -3.702730577736
O     -0.577805180632    4.868888316444    1.325303962457
H      0.303894522947    4.776235567711    1.728385452634
H     -1.040409532398    5.543184993164    1.823635028640
```

26599	O	-3.702751956357	-0.289869730773	3.289695815404
26600	H	-2.756835978821	-0.099009371708	3.421679497995
26601	H	-3.984663370896	0.252712180353	2.524032188531
26602	O	-4.175213377553	1.080820660633	0.997326506268
26603	H	-3.320741883622	1.362908662767	0.614713759847
26604	H	-4.551426330834	1.905232941254	1.376396627359
26605	O	-2.098277577529	-2.255615935282	-3.290840541537
26606	H	-2.749393446147	-1.737223860045	-2.811070898810
26607	H	-2.002289517111	-1.860934618843	-4.171371990486
26608	O	2.180703599459	4.775858816376	-0.418452671302
26609	H	2.107938047463	4.758267264101	0.548827232545
26610	H	1.312471802310	4.519266726821	-0.761981085730
26611	O	-4.520920284954	3.500814976325	2.066034068330
26612	H	-3.709066883572	3.381512878151	2.583284073515
26613	H	-4.259350449653	4.003016328584	1.278544303246
26614	O	4.299638425458	3.303501082908	-0.930486940545
26615	H	5.057425398504	3.876502228895	-1.059610480789
26616	H	3.519520116238	3.903749184029	-0.788023572444
26617	O	1.958174708166	-3.793156810945	1.707629430841
26618	H	2.191931437149	-4.589965527623	2.185075735225
26619	H	1.815446697529	-4.060001931569	0.764595021219
26620	O	4.003333006846	-2.252598028897	4.814010966465
26621	H	4.307848489684	-1.378655319663	5.115100650243
26622	H	3.055431940959	-2.294340095272	5.057746364479
26623	O	-1.764124830878	-1.240755265613	-5.912959449934
26624	H	-2.286373717188	-1.689633986084	-6.577770555133
26625	H	-1.995076202665	-0.279717486863	-5.985009423054
26626	O	2.670786025744	0.402627509850	3.012957713599
26627	H	2.968829217082	1.137008212441	2.450215852652
26628	H	3.006487176195	-0.430942228506	2.617493590373
26629	O	-1.877994970717	2.850719209175	2.826297017387
26630	H	-1.533517440074	3.295051524452	2.037399053088
26631	H	-1.645416235144	1.912142181833	2.763563849039
26632	O	-0.103775737322	0.641905783343	-3.701406979743
26633	H	0.361891030426	1.476864113891	-3.860706078743
26634	H	0.268752177836	-0.030127713013	-4.295861532895
26635	O	0.883343030672	3.239809771878	-3.677410391267
26636	H	0.294658409069	3.490068793464	-4.412519139883
26637	H	0.478658846308	3.596139831286	-2.875467498568
26638	O	1.932215532474	4.292987334560	2.352028959641
26639	H	1.452161285146	4.014168017627	3.165209883073
26640	H	2.686772276254	3.690186968569	2.290759958410
26641	O	4.564618168428	0.448171913313	4.977920456574
26642	H	3.890647201682	0.527041461694	4.273637439944
26643	H	5.405818115710	0.602099430960	4.543264241084
26644	O	-4.241413182710	-2.708932911291	2.547372969305
26645	H	-5.090269459093	-2.911867674643	2.940616682755
26646	H	-4.016110737278	-1.778303214248	2.847552428877
26647	O	-0.560372979960	-4.381743831508	-2.449682534671
26648	H	-1.172469116623	-3.656260073638	-2.673595225190
26649	H	0.074668633518	-4.385468683704	-3.180275190659
26650	O	0.369290684891	3.395371270183	4.330889458232
26651	H	0.603453671935	2.533449825245	4.707362143965
26652	H	-0.519206669815	3.283161748027	3.945821507531
26653	O	-1.643502627058	-3.946523913836	0.152637180057
26654	H	-1.307363663531	-4.449025851524	-0.598390095523

26655	H	-1.609920131488	-4.493439529623	0.943167858712
26656	O	5.633635385010	0.631315371865	-0.812164788528
26657	H	5.086792433410	1.424369373520	-0.782334671756
26658	H	5.878374283748	0.535544285769	-1.757445252205
26659	O	3.376564106964	-2.899420466397	-2.376274461239
26660	H	3.298320041859	-2.038725746982	-2.828863532829
26661	H	4.077462511263	-2.760048872725	-1.707777394059
26662	O	5.704107014485	0.345902479132	1.981372473060
26663	H	5.204441952101	-0.474724837004	2.095149641307
26664	H	5.980909019269	0.355730098373	1.054136555130
26665	O	1.603074708309	-3.925038060357	-4.187020228091
26666	H	2.261940268815	-3.628879623440	-3.530856741359
26667	H	2.068924799142	-4.498450694927	-4.796288204966
26668	O	-3.919102664615	-0.474462477490	-1.445927835639
26669	H	-4.266543489809	0.098738183055	-0.750083365243
26670	H	-4.054035760875	-1.383778443748	-1.095106704109
26671	O	1.187884468051	0.825780243187	5.170406402574
26672	H	1.851215334983	1.025085751566	5.835131747617
26673	H	1.688303338207	0.666687912114	4.338643809547
26674	O	3.395978318197	2.126456177006	-3.330820092270
26675	H	3.697400798070	2.516510259005	-2.495313139720
26676	H	2.569503700035	2.581457248194	-3.555140996052
26677	O	0.878292842582	-1.434146284890	-5.237166333866
26678	H	1.008744128262	-2.318431232888	-4.854253958126
26679	H	-0.012387084431	-1.448167804860	-5.627593906590
26680	O	-2.556898471174	3.819861625484	-3.190949448568
26681	H	-2.731555751532	2.854726474488	-3.153999242570
26682	H	-1.852659452278	3.996500326738	-2.544785949401
26683	O	-4.138231605518	-2.855072902240	-0.247915128909
26684	H	-3.266737755850	-3.283353257149	-0.292411569560
26685	H	-4.311625395974	-2.752748429895	0.700864739526
26686	O	-2.038446544178	-4.193589692712	2.958345274101
26687	H	-2.148064663876	-4.576599644859	3.829281111474
26688	H	-2.854903592454	-3.678405533081	2.773014662287
26689	O	-1.155749646300	3.719556094701	-5.427198554346
26690	H	-1.767974253802	3.816554105781	-4.649557845705
26691	H	-1.344046792639	4.450101665095	-6.015788241155
26692	O	-0.022471342773	-2.342875214183	2.913973540878
26693	H	0.672788930201	-2.789606611356	2.398113438829
26694	H	-0.766972545100	-2.972081350917	2.930812719571
26695	O	1.322847749786	-2.029262468246	5.269938796234
26696	H	1.179491451207	-1.091435037072	5.452814405211
26697	H	0.830901938637	-2.207385468168	4.445744440239
26698	O	3.874480187216	-1.884055708516	2.234565912960
26699	H	3.195850682222	-2.532483050535	1.971095198328
26700	H	4.045104875097	-2.103181881127	3.193665303144
26701	O	-2.754730791864	1.139161828569	-3.318514040619
26702	H	-3.205683803767	0.548835202064	-2.691291721542
26703	H	-1.827849800787	0.833247162406	-3.399329680739
26704	O	1.675028247252	-4.495718646941	-0.815092163106
26705	H	2.312022873773	-4.005203345094	-1.359257533320
26706	H	0.837414489573	-4.478473070565	-1.310490181498
26707	O	-2.527410858891	1.294070195866	-6.012166247831
26708	H	-1.896410430821	2.028918671546	-6.010248433059
26709	H	-2.887007485596	1.280084616677	-5.106769630342
26710	O	-3.363720255683	4.243280582081	-0.354509785630

```
H     -3.668230445719    4.382601955418   -1.255406733448
H     -2.946124460397    3.369933720313   -0.344605501365
O     -0.517514969586    4.055669744209   -1.269136536226
H     -0.732851434516    4.717761903738   -0.593905589182
H     -0.908131061637    3.242944346848   -0.899447465030
O      4.006859500402    2.427426851405    1.765336261549
H      4.106783374066    2.690094150063    0.839045014816
H      4.746515809326    1.809631502246    1.946080721872
O     -0.995174960165    0.206963472465    3.359342190572
H     -0.496143463319    0.389134287457    4.162700088063
H     -0.684011530954   -0.658746001441    3.034731985393
O      3.174641004983   -0.596641236968   -3.844864961159
H      3.116049631703    0.354113559622   -3.648015273791
H      2.461371663361   -0.793799653880   -4.465857055532
O      5.056187437037   -2.106767763081   -0.390607568523
H      5.280018682134   -1.179945063077   -0.566001655304
H      4.728692342668   -2.135875116686    0.518493362699
C      0.812322971160    1.597063903636    0.805550173809
C      1.760600328900    1.374247915747   -0.128864672208
C      2.319450750275    0.073627614307   -0.483699036207
C      1.922315129837   -1.102525082712    0.050873070792
C     -0.239891066793   -1.291513985988   -1.130149959355
C     -1.046810975017   -0.472159922838   -0.380489114042
C     -1.119417380253    0.942968173372   -0.727223442840
O     -1.877569409950    1.828175230640   -0.251257216778
H      2.084690334224    2.204005481556   -0.752330048357
H      0.428652666442    2.589562673230    0.989811189629
H      3.009925864913    0.067781897763   -1.323120168618
H      2.329279251605   -2.038264154537   -0.292962239345
H      1.310678804965   -1.164378258456    0.938532603210
H      0.217024975393   -0.948548213112   -2.041093281414
H     -0.221302959771   -2.353474162232   -0.951995985708
H      0.483023332851    0.847499531242    1.509616801871
H     -0.522997944842    1.216372194697   -1.610047904034
H     -2.518219666618   -0.456581745710    1.063638302017
N     -1.687258676110   -0.938971142202    0.765514691540
H     -1.751482280694   -1.943266905151    0.841260961764

Ambimodal TS Water45-72
155
ANGSTROM
O     -0.026271259446   -4.728344594072    0.877403936228
H      0.050817872948   -5.668012690860    1.052975927485
H     -0.867593928259   -4.451628934879    1.347595023950
O     -4.480178122497   -0.850874231247    3.191273961050
H     -4.571055463105   -0.137775064262    2.534834185870
H     -4.468639335455   -1.669175614169    2.685952945395
O     -5.005077644691   -0.146734043069   -2.724928497572
H     -4.934397660162    0.815596438165   -2.762282835219
H     -4.063328914072   -0.463009313291   -2.653755604330
O     -1.123290423633   -4.873108356982   -1.757672733294
H     -0.672731042646   -4.759423354879   -0.909003402520
H     -0.654118601285   -4.318616775996   -2.407862249461
O      0.902891941419    2.432215627411   -5.417664405756
H      0.238923527439    3.057864085394   -5.106077283899
H      0.430192756100    1.602674487894   -5.613896202336
```

26767	O	0.549176512403	3.902261074358	3.378259588193
26768	H	1.309093571499	3.289372400987	3.212777341346
26769	H	0.781945811320	4.396098406933	4.165374146697
26770	O	4.418018784569	-0.481516029302	1.711632475221
26771	H	3.708616721500	-0.487806610823	2.376327851172
26772	H	4.546549690672	0.460569935161	1.500876822642
26773	O	4.096363123008	2.746394848742	-1.499744776694
26774	H	3.390574555147	2.462562737347	-2.112383762442
26775	H	4.870933791704	2.201167014122	-1.756937251440
26776	O	2.598816999832	2.277905218989	3.077639622937
26777	H	3.199230669397	2.326748757172	2.315553877763
26778	H	2.467379194738	1.339957345022	3.259534697374
26779	O	-0.692698334703	6.735633711401	-1.222079330067
26780	H	-0.429773329267	6.363800384818	-0.365332252738
26781	H	-1.468917222404	6.227119317049	-1.501947913296
26782	O	1.274768889043	-0.739070669961	-3.300959148061
26783	H	2.132150466309	-1.206551893182	-3.294579040247
26784	H	1.522852717456	0.201881117898	-3.186877726166
26785	O	4.481809345671	0.217173154372	-4.568897808965
26786	H	4.228378789748	-0.645930143887	-4.187265100263
26787	H	4.455862536569	0.125431695201	-5.521670456445
26788	O	0.215747087130	-3.313924193992	-3.576482775272
26789	H	1.066015456708	-3.781255140827	-3.564418167390
26790	H	0.429384911090	-2.385183890150	-3.403590453373
26791	O	0.146482913449	5.099659362417	0.902771604035
26792	H	0.989219058375	4.946345394713	0.432456237843
26793	H	0.304044789904	4.800676482203	1.812983626351
26794	O	-0.946124462592	-1.911414842207	3.421704922061
26795	H	-1.448799960918	-1.429099205926	4.115157552892
26796	H	-1.115268362585	-1.395222639200	2.600704703023
26797	O	-3.730564557709	3.120150402386	2.536589524149
26798	H	-4.169069953663	2.387427602472	2.059782411971
26799	H	-4.402195088242	3.548145701602	3.067997758381
26800	O	-5.935204467504	-0.807611074288	-0.371786556539
26801	H	-6.832974687932	-1.114406426651	-0.495379784100
26802	H	-5.591343804128	-0.577769670081	-1.282507559494
26803	O	-2.918048217540	5.081154904038	-2.006822021111
26804	H	-3.626068466874	5.725397056325	-2.047402498464
26805	H	-2.831543450056	4.826874138996	-1.049145967865
26806	O	2.451386350240	4.843768736411	-0.544942727717
26807	H	2.104659428615	5.159622701690	-1.395572037523
26808	H	3.120252582846	4.185674601362	-0.770653870005
26809	O	-1.993081286750	-2.229691997279	-4.935085100422
26810	H	-1.318730597580	-2.853992601606	-4.632322647838
26811	H	-1.499302614254	-1.485992596450	-5.336140608192
26812	O	2.441467641413	1.683810152392	-3.389677228077
26813	H	1.898480025410	2.097983668029	-4.101777618605
26814	H	3.205015489755	1.295371102901	-3.851215399739
26815	O	2.680304337798	-4.467410086919	-3.033077255989
26816	H	2.591221566439	-4.397147896631	-2.053133653237
26817	H	2.930903079030	-5.371870702542	-3.220662675364
26818	O	3.895690983853	-3.317236401370	1.701000979902
26819	H	3.153219325992	-3.357857726960	2.321058225276
26820	H	4.236893284963	-2.416844425023	1.767157457993
26821	O	4.461740536448	2.177325971215	1.060052368361
26822	H	5.223481008633	2.727439657149	1.246638998453

26823	H	4.215695953419	2.362927620205	0.121547481923
26824	O	5.867284518946	0.848237442665	-2.180800547961
26825	H	5.593407065410	0.684106352798	-3.092778611416
26826	H	5.602271668063	0.063854657428	-1.674665958131
26827	O	1.170585449903	5.788044350918	-2.811116829124
26828	H	0.503282116120	6.242107016167	-2.229650962921
26829	H	1.550390408571	6.460531674427	-3.376243036193
26830	O	-3.420771113545	-3.545778894079	-1.584695029010
26831	H	-2.656988136434	-4.155103485232	-1.688219464118
26832	H	-3.946286713644	-3.585107805971	-2.407505194889
26833	O	-2.445610322606	-0.310960409165	4.918524562613
26834	H	-2.202650485125	0.567918299204	4.590956195118
26835	H	-3.276662331799	-0.534267368857	4.458765880587
26836	O	4.914692931585	-1.427068743667	-0.774973727790
26837	H	5.486942227831	-2.159500627980	-0.539364693576
26838	H	4.608691593811	-1.052117086278	0.078162064960
26839	O	-0.815835701337	4.148967494913	-3.774998841971
26840	H	-1.547415385220	4.509107030356	-3.257238285628
26841	H	-0.018289500769	4.610539015224	-3.460127522280
26842	O	-1.923293553736	1.636154993428	-3.977620777714
26843	H	-2.792074779870	1.763325807148	-3.566289697377
26844	H	-1.475868708441	2.493287255531	-3.909184992938
26845	O	-4.123420119606	-2.560802845968	0.798828764853
26846	H	-4.811913306738	-1.943657675303	0.491757730293
26847	H	-3.882110753440	-3.041208834117	-0.018140825037
26848	O	-0.420378513769	-0.052610037568	-5.364592828765
26849	H	0.197180574161	-0.359078145425	-4.676450975627
26850	H	-1.074246765452	0.498289172332	-4.886885438665
26851	O	3.696699404940	-1.953609172969	-3.175173040624
26852	H	4.057907381080	-1.802067781921	-2.282484130453
26853	H	3.462836863212	-2.895893466357	-3.226500048214
26854	O	0.421491677593	0.407588467882	5.162082638208
26855	H	-0.114587955246	1.023448013813	4.644843721580
26856	H	-0.207169497195	-0.172888612630	5.599035994992
26857	O	-2.600471230570	-1.149124617750	-2.505529036966
26858	H	-2.829644740022	-1.987695107904	-2.054062606959
26859	H	-2.355243833529	-1.413967267809	-3.413735959324
26860	O	2.461315486991	-4.200891779273	-0.419137678601
26861	H	1.603996240099	-4.344790100962	-0.000959474176
26862	H	3.078409692156	-3.937135019880	0.292916389721
26863	O	-1.455989185049	2.048242420614	3.719429436066
26864	H	-0.779241517920	2.738872031774	3.596552544264
26865	H	-2.258226096623	2.413617116502	3.313028688863
26866	O	1.347810844726	-3.231006452510	2.904533560878
26867	H	0.994478453147	-3.775803844509	2.185966835165
26868	H	0.555945537871	-2.805303446129	3.282407264979
26869	O	-4.036203482218	2.488994471884	-2.420069996917
26870	H	-3.515508027423	2.166549079660	-1.655096483535
26871	H	-3.803795494974	3.424898436656	-2.485034084638
26872	O	-4.608561656981	1.007440069209	1.149060567605
26873	H	-3.854766576608	1.166878146013	0.531232106359
26874	H	-5.255138395489	0.501449289243	0.628909624913
26875	O	-2.121974654064	-4.026458826619	2.249380223492
26876	H	-1.767614702059	-3.308619014999	2.818517405865
26877	H	-2.901689113279	-3.669190932127	1.806967638889
26878	O	-4.592237971318	-2.930682791948	-3.957568936150

```
H      -4.971789433119   -2.063732829500   -3.774536401590
H      -3.799840976732   -2.767447379899   -4.490201731686
O      -2.564579040860    4.727859398959    0.549257012674
H      -2.923270952379    4.098356976610    1.192340819395
H      -1.609646769152    4.798047260839    0.721006899098
O       2.319416679137   -0.598606076041    3.516393101152
H       1.706684685116   -0.275746096373    4.207177322129
H       2.087158336959   -1.528364391497    3.388947729159
C       0.209993197226    1.711021778587    0.871484588398
C       1.239757636824    1.548596142721   -0.000745563423
C       1.942671994981    0.308821586953   -0.234131078057
C       1.635519532197   -0.882405288035    0.374423039984
C      -0.351863049068   -1.392819326530   -0.507733101367
C      -1.261581320922   -0.473520484789   -0.028854546357
C      -1.552111389836    0.778481146806   -0.707492782894
O      -2.527043701484    1.514495597717   -0.428965212598
H       1.480673494761    2.369624252168   -0.673049157766
H      -0.327464778575    2.645516815240    0.937031348294
H       2.635217123642    0.303342666469   -1.072590499461
H       2.157649507902   -1.786517140373    0.093385653567
H       1.160249735492   -0.917624777169    1.343562798971
H       0.015466984929   -1.291238402544   -1.515549603366
H      -0.315332523515   -2.389146396563   -0.087800749642
H      -0.011338302948    1.008576440578    1.662674785550
H      -0.969911643634    1.006468614834   -1.613249200370
H      -2.463817271717    0.088898657477    1.518789707947
N      -1.949855913344   -0.726869117842    1.203212291238
H      -2.604722168949   -1.508206720237    1.134488914936

Ambimodal TS Water45-73
155
ANGSTROM
O       1.523100937716   -3.915660684613   -2.753876965613
H       2.250022022185   -3.370814905497   -2.416629659042
H       1.436655067800   -3.665723397611   -3.699250094154
O      -4.946983167887    1.022266357167    1.106043604174
H      -5.376893395833    1.840941306518    0.853107796245
H      -4.041027479857    1.080825294670    0.735147352481
O      -0.146636451604   -2.000871544879    6.035065621194
H      -0.049877626447   -1.051344988231    6.182698775665
H       0.714325673017   -2.309456643751    5.725269202287
O       1.131403833514    2.933003880937    3.806450267397
H       0.244986771865    3.179127350793    3.490630275695
H       0.977455765292    2.327544030526    4.554040714781
O      -0.888044930175   -3.964911402896    1.644654465336
H       0.032503629117   -4.114278943280    1.922709875615
H      -1.353637281420   -3.726533360031    2.460176846766
O       1.708265399596    5.261757787261   -0.117278466596
H       0.911243425050    4.883480600545   -0.515220139589
H       2.384252082732    5.223683741595   -0.808162620644
O      -2.730808449594   -3.709595457843   -0.467302068608
H      -2.238278743157   -3.882470927774   -1.294131914929
H      -2.111884570403   -3.906518931244    0.250130791025
O      -3.829839927912    3.565863729567   -0.842836674718
H      -3.430504701777    4.272502867521   -0.324209430936
H      -3.369741486533    2.753917876015   -0.543165570894
```

26935	O	4.232230154990	-2.156102398297	2.554828784923
26936	H	4.090477787887	-2.728732576143	1.790934228265
26937	H	4.582245367076	-1.318765707147	2.221659447990
26938	O	2.666932589947	0.764370888866	3.016811738960
26939	H	2.257437082140	1.634447229620	3.148097986020
26940	H	1.945853917341	0.166639013817	2.794032684494
26941	O	0.755767156015	0.932383549183	-3.063805105168
26942	H	1.604765582249	0.455145219171	-3.099218964743
26943	H	0.980838715704	1.883086908640	-3.117239300163
26944	O	-2.408169083645	3.071385037038	-3.098161777086
26945	H	-3.107381231442	3.363732627996	-2.480098569934
26946	H	-2.444481812251	2.099827114531	-3.074203064632
26947	O	3.140829124280	-0.326847159522	-3.448677742190
26948	H	3.024511800141	-0.364118717482	-4.411543325369
26949	H	3.860427946911	0.323118548945	-3.246421571074
26950	O	4.854971144792	0.488083950753	1.553633160862
26951	H	3.980207888796	0.568888840951	2.000722322278
26952	H	5.402163072343	1.124540315974	2.043961698444
26953	O	-4.682053492026	-2.961535655278	1.562627628771
26954	H	-5.369162795867	-2.481656410673	1.050305455743
26955	H	-4.097724870938	-3.355101889799	0.904209721980
26956	O	1.347526398106	3.561542507415	-3.292555536535
26957	H	2.217230625601	3.865550456196	-2.988156018590
26958	H	0.711179093672	3.888292905219	-2.636475838586
26959	O	-2.738559782081	0.300594608826	-3.106832668619
26960	H	-2.036451672374	-0.285838579647	-3.439436040389
26961	H	-3.471056941801	0.206649245673	-3.723621106081
26962	O	0.361135523403	-0.914304895397	3.252031595309
26963	H	1.068477499788	-1.500169928098	3.618564266152
26964	H	-0.434105307814	-1.488458873608	3.271946624508
26965	O	3.857917423467	-2.478637933670	-2.104788093151
26966	H	3.537795383191	-1.673448936518	-2.591366414697
26967	H	4.488150035279	-2.905131872808	-2.688792749936
26968	O	2.976347171577	3.937871043319	1.891271876350
26969	H	2.493749610912	4.514720311278	1.264523985407
26970	H	2.330759722593	3.687070290892	2.566154048356
26971	O	3.382312052072	-3.888243003191	0.365297747788
26972	H	3.700364356895	-3.440019405397	-0.427507617115
26973	H	2.702524656849	-4.515259214198	0.040014263812
26974	O	1.086660331077	-2.872468995141	-5.177553156255
26975	H	1.588732747730	-2.102478156400	-5.484142790855
26976	H	0.184136439402	-2.551477450918	-5.027682907681
26977	O	-1.526967790792	3.444315571710	3.154602038987
26978	H	-1.967071125046	3.860754270013	3.897339389363
26979	H	-1.734682557322	2.478553537948	3.236121192340
26980	O	-4.084584363617	-1.725118666978	-1.779165124329
26981	H	-3.518259900636	-0.940998123390	-1.837442772774
26982	H	-3.600943148411	-2.404282688779	-1.272015416222
26983	O	-1.386264154101	-1.634600599115	-4.590950138245
26984	H	-2.316011686364	-1.811976995021	-4.823806798738
26985	H	-1.081423403217	-0.868336915635	-5.116928211297
26986	O	2.194303315779	-0.424738212383	-6.032489746249
26987	H	1.307213755019	0.001841613951	-5.913245206496
26988	H	2.479506208234	-0.219762091639	-6.922431470811
26989	O	0.921273067574	0.669453680895	5.420630884053
26990	H	0.637271068917	0.167911367827	4.628754369291

26991	H	1.857901498141	0.480264077938	5.516681346319
26992	O	4.326151327032	2.629333076359	-0.144455694125
26993	H	4.620377840416	1.814601602747	0.298634550045
26994	H	3.852971250850	3.112073954905	0.555986724442
26995	O	3.636635076209	4.450313446510	-2.027749381185
26996	H	4.427329410518	4.798535389331	-2.440205650642
26997	H	3.930914063027	3.778128039951	-1.384598380835
26998	O	5.714145573888	-0.740015081802	-0.815905785956
26999	H	5.040917327505	-1.398545574588	-1.035772592433
27000	H	5.478058803615	-0.375922143144	0.050071906589
27001	O	-1.721751505306	-2.497867357878	3.857007136611
27002	H	-2.565432181527	-2.031993686241	3.833980319205
27003	H	-1.374445853917	-2.445998802999	4.768258088353
27004	O	5.059754090957	1.229616792056	-2.538673577113
27005	H	4.758411106873	1.857561495150	-1.869924541573
27006	H	5.510279989630	0.530405736231	-2.019838524329
27007	O	-3.911580953136	-0.824533856611	2.923865621589
27008	H	-4.504611715927	-0.166481005592	2.536341579346
27009	H	-4.191899463745	-1.677775974449	2.509263435318
27010	O	1.688169568498	-4.480809877356	2.510350764436
27011	H	1.793467843784	-5.433490582957	2.490526510826
27012	H	2.322736245339	-4.127982920188	1.864939058339
27013	O	-1.897321156690	0.895070803386	3.656795870602
27014	H	-1.126005364880	0.367981731386	3.420344451119
27015	H	-2.670663772808	0.342700265670	3.458343388742
27016	O	-1.941802961588	4.902110040505	0.874631452372
27017	H	-1.517412635252	5.731086479326	1.101626693296
27018	H	-1.827146978869	4.310699132454	1.644190842009
27019	O	-0.190277646575	0.595770033586	-5.467575512340
27020	H	-0.444341862825	1.520298772386	-5.676004161923
27021	H	0.100122541557	0.671673155633	-4.516482073407
27022	O	-1.246283427516	-3.803869844027	-2.709056705155
27023	H	-0.284668361983	-3.925820653808	-2.674932305039
27024	H	-1.383194426233	-3.067930245113	-3.316506572237
27025	O	1.237328836326	-5.336357241052	-0.424338076177
27026	H	0.375465413814	-5.098394007484	-0.070874209592
27027	H	1.242342920161	-5.055911730057	-1.353793719234
27028	O	-4.065744654723	-2.017950257534	-4.480350937170
27029	H	-4.162827669680	-2.004036377605	-3.502755769796
27030	H	-4.531503080536	-2.797547491920	-4.782143660120
27031	O	-6.121638849817	-1.276716272566	0.039977428378
27032	H	-5.831625211195	-0.433462326483	0.425418221774
27033	H	-5.564648882208	-1.412583755507	-0.743862173266
27034	O	-0.560647952569	4.141559904084	-1.409315344737
27035	H	-1.196894216712	3.758280354182	-2.048504809748
27036	H	-1.098570714682	4.363063646036	-0.631258623777
27037	O	5.327286327625	2.778575641017	2.973202756682
27038	H	5.145364314430	2.722466200279	3.911851041382
27039	H	4.607588930419	3.305212764161	2.591346400737
27040	O	-0.653421041640	3.222196970661	-5.299526663191
27041	H	0.136117056063	3.465911229030	-4.795919716508
27042	H	-1.384483096945	3.297163599647	-4.668364422917
27043	O	2.198101102768	-2.486112194278	4.320487077586
27044	H	3.028061145105	-2.245996515605	3.862281530568
27045	H	1.951470718038	-3.332693041091	3.915099170813
27046	C	0.272225654023	1.652763648224	0.903811555960

```
C      1.341103271964    1.572056315162    0.067159224259
C      2.069874066716    0.367292907695   -0.255136029437
C      1.752234249324   -0.887550215762    0.194585234771
C     -0.205415628805   -1.299730805338   -0.910781372189
C     -1.147723877468   -0.607137817492   -0.170311295973
C     -1.545519611368    0.762596381883   -0.514876489007
O     -2.534621162189    1.346857357282   -0.012276884779
H      1.616422644701    2.456713727683   -0.503170097522
H     -0.254901260170    2.583191832517    1.051877612067
H      2.809025730559    0.457880407992   -1.045864923061
H      2.309072241925   -1.745546292809   -0.150635652257
H      1.191905235566   -1.059113473147    1.101916403908
H      0.127519734567   -0.911208844528   -1.857740682311
H     -0.065370146604   -2.359248232494   -0.755953591562
H      0.016144728010    0.868479380469    1.599335105073
H     -1.057810803442    1.208387988843   -1.392823127095
H     -2.480424000612   -0.703516409752    1.419630149202
N     -1.680367113708   -1.137047174059    0.986216574346
H     -1.533896232674   -2.111064498388    1.194503825753

Cope TS Water45-1
155
ANGSTROM
C     -0.008475628431    1.247423993704    0.763197632321
C      1.289125572893    1.337577571225    0.057066411497
C      2.003905670668    0.248418477335   -0.258922860563
C      1.448740480294   -1.144366145197    0.013806566947
C     -0.003265272024   -1.274967629310   -0.630407659974
C     -1.064938759483   -0.339829392232   -0.033683546113
C     -1.507317445354    0.843030563427   -0.731227253283
O     -2.588109270677    1.476107960103   -0.484750721807
H      1.612529028980    2.323596594401   -0.256015052642
H     -0.528408826927    2.191151777393    0.907265419859
H      2.940733917894    0.327014575253   -0.793302660598
H      2.091121592029   -1.924971498227   -0.400841192811
H      1.350374742933   -1.328282984860    1.089129873985
H      0.090618813051   -1.082700890349   -1.703697595729
H     -0.344030389977   -2.307422981583   -0.496782257396
H      0.000075564778    0.686893744475    1.696376510079
H     -0.908325675837    1.178982436106   -1.583430590873
H     -2.606104478539   -0.325620880852    1.336279823197
N     -1.866749208128   -0.880868731467    0.937017340334
H     -1.534667722299   -1.621757895012    1.529514007608
O      4.091680589210    3.532442898281    0.812875447755
H      3.605918965936    4.061931596407    0.171064304180
H      3.441102367345    3.253162769963    1.474420542213
O     -3.804582030017    3.342468173121    1.064997779970
H     -3.365132940308    2.608702024483    0.600622801048
H     -3.659249058349    3.198071443923    2.034838570420
O     -5.788028553409    1.408183473804    3.510548754934
H     -5.136671506989    2.074878052532    3.755474234796
H     -6.135353466423    1.678831174283    2.632772554512
O      0.318439375289    1.907960390816   -3.112618224863
H      0.508853315970    2.853469112284   -3.158456020210
H      1.144460843742    1.423878603156   -3.287325126513
O     -0.000599343633    1.886322051685    4.826785386354
```

27103	H	-0.792740410395	1.804819216522	5.364043615434
27104	H	-0.044398974587	1.151559972230	4.198919277868
27105	O	2.139122592152	-3.469007535721	-2.787526708202
27106	H	2.794398778999	-2.772182083053	-2.598635062325
27107	H	2.487287838317	-3.977932583535	-3.521474516372
27108	O	1.302446166588	-1.656974462160	5.778956406353
27109	H	2.101181107228	-2.140654766346	5.498976796426
27110	H	1.650430014710	-0.782027680848	6.062238748635
27111	O	2.012798131188	2.981726529189	2.626516964960
27112	H	1.366674424043	3.449493050077	2.066982497985
27113	H	2.095179156725	3.488473711658	3.436628527202
27114	O	4.035387034839	-1.892330040131	2.358056088347
27115	H	3.426374353901	-2.595948291934	2.018537032791
27116	H	3.513521978136	-1.082711405309	2.527862756916
27117	O	-1.602409355072	0.869560217855	-4.607674784939
27118	H	-2.393341715575	1.424226481397	-4.557332063674
27119	H	-0.909214630602	1.330825948807	-4.079173800480
27120	O	-0.509342200920	-3.159192757188	-3.618555311795
27121	H	0.351450100874	-3.292910781528	-3.191084987732
27122	H	-1.105050717679	-3.846575249086	-3.277932396823
27123	O	1.329975154133	-5.070158837304	-0.619523244709
27124	H	1.619672564544	-4.519870380921	-1.359375021235
27125	H	1.824601768008	-4.765176527616	0.158616871499
27126	O	5.223679091533	1.625664960433	-0.807113348109
27127	H	4.961943009498	2.286564687738	-0.139243093197
27128	H	5.177276072128	0.763017692237	-0.376293762077
27129	O	-3.868156512086	2.407839182887	-3.919467092052
27130	H	-3.437482929047	2.943290610155	-3.224659783316
27131	H	-4.518568336254	2.968218258815	-4.342966435773
27132	O	-4.827439438002	-0.069992909873	-2.893650657233
27133	H	-4.024805997803	-0.595655815343	-3.032214919024
27134	H	-4.609437677533	0.809300122764	-3.236448895204
27135	O	-2.515365655074	3.723768436123	-1.959420913449
27136	H	-2.532006886828	2.888911453012	-1.434226279384
27137	H	-2.572376144264	4.428455334551	-1.296485736641
27138	O	-0.869453403382	4.252183083027	3.640146251673
27139	H	-0.464635715512	4.430052192713	2.782210910945
27140	H	-0.395681424640	3.498409647318	4.027930731364
27141	O	4.070546500535	-1.570889752534	-2.346689866736
27142	H	4.792252478335	-1.349783424336	-2.938362523514
27143	H	4.411007540246	-1.422790231823	-1.439823192872
27144	O	2.636189326012	0.303954353240	3.095932513291
27145	H	1.724194511257	-0.043759296023	3.183659055208
27146	H	2.544886638748	1.210176009053	2.768100601328
27147	O	0.510708285942	-1.000921644002	-5.177267311941
27148	H	0.143706291026	-1.804092303042	-4.785278805337
27149	H	-0.220908847540	-0.372749194116	-5.235377271245
27150	O	-1.245796891139	-5.920456553791	-0.333521624440
27151	H	-1.631033160539	-5.684297069427	-1.188594939948
27152	H	-0.291516403743	-5.730362120578	-0.406748446635
27153	O	-2.288977768056	0.454522844278	3.721782632013
27154	H	-3.081681092294	-0.031924810642	3.437068356199
27155	H	-1.564375587919	-0.187749553834	3.717895057931
27156	O	-2.243045739438	-1.140732385487	-2.989249119324
27157	H	-2.011744026731	-0.384000367556	-3.576246999604
27158	H	-1.660286520157	-1.864724594068	-3.277520936258

27159	O	3.466352974202	2.701684798978	-2.619329642611
27160	H	3.164006971948	1.946620341241	-3.138457439421
27161	H	4.180032554495	2.358335331664	-2.051270487127
27162	O	2.434455806104	-3.899417851872	1.650325787112
27163	H	2.530983344872	-4.515337920399	2.403107999074
27164	H	1.526464700972	-3.544637876953	1.779085113658
27165	O	0.197949409726	-0.738087134248	3.569195940422
27166	H	0.516368261949	-1.083402072672	4.441293641883
27167	H	0.076468652900	-1.546191415828	3.024578249881
27168	O	0.008785019361	-3.075254470476	2.307462887059
27169	H	-0.745325745860	-3.447858504414	1.790951600241
27170	H	-0.070851382361	-3.492494845912	3.192906942911
27171	O	-4.500568566621	-0.726056571932	2.620963376040
27172	H	-5.074538064675	-0.043856440690	3.048801836361
27173	H	-4.746796502789	-0.708499449372	1.687407159994
27174	O	-2.172339213689	-3.924153941551	1.100539044797
27175	H	-1.896812583636	-4.765717639316	0.658340348021
27176	H	-2.623275554282	-3.397069413203	0.417724276101
27177	O	-2.225545885772	5.289456738809	0.314287228494
27178	H	-2.856277841471	4.612909421081	0.682420910719
27179	H	-2.469835609251	6.127711324584	0.706166462478
27180	O	-2.559094667114	-4.637533934195	-2.466241692714
27181	H	-2.955467090140	-3.950191467421	-1.898950684849
27182	H	-3.270318473802	-4.991281996888	-3.001116531564
27183	O	2.184023193497	-5.005313819305	4.102237393703
27184	H	1.288182018298	-4.888214438415	4.438174912682
27185	H	2.732418100761	-4.346734790987	4.552934975957
27186	O	-0.152910715939	4.639646546493	-2.939624728759
27187	H	-0.993315429979	4.271960772347	-2.588996866354
27188	H	-0.384855863249	5.240340116272	-3.647180651980
27189	O	-3.400951933957	-2.499739537380	-0.937396327234
27190	H	-2.926172980421	-1.950122254285	-1.585950217033
27191	H	-4.046245677388	-1.891985104533	-0.541185173673
27192	O	2.371847535290	0.788874050823	5.827964222814
27193	H	1.613880504385	1.375201415492	5.685714715345
27194	H	2.756739070362	0.656914904378	4.944253657069
27195	O	-3.261180169611	2.973103166675	3.604814618689
27196	H	-2.502082214167	3.579710007630	3.711253466225
27197	H	-2.892154626411	2.076607096487	3.699716871906
27198	O	-2.652106193999	-2.693159481121	3.559151163657
27199	H	-3.441686441794	-2.148606368390	3.447339636590
27200	H	-2.657182865672	-3.298252062960	2.802450494331
27201	O	5.206652817960	-1.124203608776	0.084099428234
27202	H	6.131593345626	-1.362542241943	0.157516673776
27203	H	4.782654786647	-1.408120393112	0.930013683609
27204	O	-0.397582407609	-3.743504393698	4.863830535776
27205	H	0.011228104527	-3.072674324485	5.427976286951
27206	H	-1.316180685873	-3.465498959567	4.720489215070
27207	O	-4.752578928850	-0.127662245231	-0.219898344839
27208	H	-5.031842988583	-0.144962050031	-1.160931914988
27209	H	-3.959720119869	0.449934078830	-0.244279866489
27210	O	-6.222089394357	1.991830588207	0.913655813254
27211	H	-5.508789562261	2.648285391694	0.848477569491
27212	H	-5.886232485385	1.193673960416	0.473223831577
27213	O	1.992861262582	4.444181383625	-1.152773036257
27214	H	2.555785986224	3.838163452667	-1.680955657846

```
H      1.294901139541    4.706192373712   -1.769426988204
O      3.662974092968   -2.757139188465    4.890809009109
H      4.428986524663   -2.623311033267    5.448770736899
H      3.907370272283   -2.440241200955    3.990321779098
O      2.409619566297    0.287130253908   -3.733284918407
H      1.821475445679   -0.206698332028   -4.342103741225
H      2.834583601472   -0.369944059808   -3.165292810778
O      0.322364249919    4.474278660441    1.045201037588
H     -0.532114838906    4.787699778939    0.697039026787
H      0.944678190773    4.537197495854    0.296854986903

Cope TS Water45-2
155
ANGSTROM
C     -1.135718194380    0.966628248067    1.175300243475
C      0.199708642563    1.522945148055    0.826689596085
C      1.200656815089    0.812399331037    0.291772894408
C      1.009073415254   -0.630320801359   -0.163106137985
C     -0.327641303412   -0.725830991097   -1.008075833091
C     -1.580277429034   -0.514875161040   -0.160968879010
C     -2.478049272413    0.565801210156   -0.430910219645
O     -3.717991387434    0.636239307777   -0.080783773860
H      0.334317497950    2.580331541015    1.029897730965
H     -1.869472866872    1.728955498220    1.428905523078
H      2.156548701436    1.278579449522    0.083088880277
H      1.846298222188   -0.960130722631   -0.780232401980
H      0.932201372594   -1.315807404222    0.690465224516
H     -0.284751403852    0.005810283808   -1.820074890897
H     -0.390014957227   -1.725835073486   -1.447238470852
H     -1.125014477846    0.201394813678    1.950096755449
H     -2.103623431038    1.357230597976   -1.092453155844
H     -2.633894705339   -1.506072285443    1.304502285227
N     -1.994077152666   -1.633330843901    0.533935457568
H     -1.283064761328   -2.331836714736    0.708741295493
O     -4.803914583663   -3.239188499374    4.155866275975
H     -5.205253673587   -2.412112350785    3.869485953250
H     -4.570878446231   -3.721507040451    3.351642768514
O      1.697708177020    0.958911187217    3.606456519536
H      1.319317807779    0.327677999224    2.981627367333
H      1.242771201948    1.808708318120    3.448365294976
O      5.059562098336   -1.464256626921   -2.962479568296
H      5.258131006343   -0.662079413013   -3.490718410564
H      4.213194215422   -1.782329900334   -3.309769175783
O      4.990137507217    2.699563060677   -1.430509502443
H      4.769767678065    1.754894123557   -1.326774324819
H      4.729211742722    3.088034239605   -0.581146489231
O     -3.282595224823    3.612512604564    2.567223573914
H     -2.933805579582    3.753478063162    1.649428417607
H     -3.491863291172    4.484819425129    2.902274664310
O      1.615234586556    4.380628295613   -0.356698923605
H      2.354661872608    3.985890899761    0.132660094220
H      1.064761268295    4.836556286625    0.306275024055
O     -4.418556522568    2.797587989923   -1.500378343755
H     -4.254846042744    2.039593658359   -0.894025974145
H     -5.370569129985    2.855341559880   -1.595329165961
O     -0.203775430518    5.137546992077    1.570680905036
```

27271	H	-1.016006734229	4.960217582303	1.080665789211
27272	H	-0.125751665927	4.446051174389	2.246306293085
27273	O	4.102211762883	0.144383523776	-1.034951644247
27274	H	4.543527691177	-0.479115010776	-1.653698069356
27275	H	4.115728939611	-0.262106265184	-0.161760938908
27276	O	-2.858703073877	-0.644652287183	3.436639699157
27277	H	-3.822078479419	-0.614295336210	3.499264057776
27278	H	-2.571363696404	-1.327144155515	4.085633967901
27279	O	-5.913223164790	0.137103454774	-2.016127795644
27280	H	-5.257626662433	0.215960208957	-1.309289641529
27281	H	-5.454954600468	0.399378761153	-2.826313459216
27282	O	-2.259425019632	-2.730016888199	5.031065285369
27283	H	-3.192091890228	-3.005925773452	5.038097259674
27284	H	-1.856483748029	-3.248506436932	4.311627735914
27285	O	2.453261314869	1.087187366336	-3.115089690004
27286	H	2.819120068162	0.771928689977	-2.274948713775
27287	H	2.437103176279	2.072451223386	-3.053638440114
27288	O	4.149714264806	1.093145207694	2.382850369225
27289	H	4.210566230864	0.204397278200	2.004554226546
27290	H	3.372707039581	1.065385541746	2.966974822335
27291	O	0.843397561109	-4.440821468996	-2.534270593096
27292	H	0.396269283012	-4.161591258394	-1.699803209211
27293	H	1.454603938880	-5.128372740062	-2.253264350480
27294	O	2.143546878076	-2.933858162668	3.091522838433
27295	H	1.402321704070	-2.292805691739	2.976867377337
27296	H	2.086199458792	-3.155338132521	4.030506071885
27297	O	0.111808672519	3.168421578056	3.604614606891
27298	H	-0.682244652260	2.629298385465	3.843915572298
27299	H	0.331798186144	3.687428413922	4.379491641241
27300	O	-5.147682352132	1.692456175904	1.969984966415
27301	H	-4.652050192650	1.318560129200	1.218549952790
27302	H	-4.585316345127	2.408620017616	2.309355658096
27303	O	-0.583914484402	3.112729543461	-1.476244427461
27304	H	-0.762361249342	3.405220990996	-2.390366115443
27305	H	0.259510786533	3.519815565761	-1.214329858571
27306	O	-0.392844290824	-3.877350661003	-0.259196648138
27307	H	0.107045510284	-4.325413712747	0.460476003432
27308	H	-1.210302978936	-4.361649752563	-0.414128281899
27309	O	0.746053297918	-2.836331085057	5.568764685444
27310	H	0.700518718205	-1.897090902942	5.786880488108
27311	H	-0.156824074576	-3.149968512078	5.675457274760
27312	O	-0.010268984700	-1.390991470768	2.996059023450
27313	H	-0.128476569391	-1.117985946902	3.924209778733
27314	H	-0.576751720340	-2.173643027502	2.878395138822
27315	O	5.029161203504	0.978031969778	-4.078986117928
27316	H	4.062504565762	1.045427223332	-3.950535125689
27317	H	5.405245835898	1.683688124262	-3.545484177490
27318	O	2.364931119139	-2.301833426125	-3.057534278306
27319	H	1.800293363695	-3.101565624348	-3.016182521117
27320	H	1.817678769998	-1.612011230636	-3.452857514388
27321	O	2.742028052029	-5.480816175071	-0.481024011400
27322	H	3.463808832623	-6.104306786468	-0.399030091505
27323	H	3.149912068662	-4.597802353584	-0.609705539940
27324	O	-2.044023941036	1.769310896907	4.283880583007
27325	H	-2.294418687535	0.876918651802	3.946948345302
27326	H	-2.622070643477	2.387565365282	3.813290285908

27327	O	3.835288267501	3.346257550058	1.020991036020
27328	H	3.949835433502	2.473449466955	1.472466419968
27329	H	4.271145171440	3.989043888717	1.580944657245
27330	O	-3.080428172272	-4.036117531487	-1.018448249461
27331	H	-2.796089312367	-3.108022089123	-1.031527674959
27332	H	-3.889440962898	-4.064236546468	-1.539140029411
27333	O	0.020213551611	-0.095094603238	5.454112984764
27334	H	0.758701097453	0.367134388952	5.013889255116
27335	H	-0.728886952188	0.507952663315	5.445174726816
27336	O	0.498344700820	-0.408591640314	-4.187258849048
27337	H	-0.173962994337	0.164705699561	-4.586510458777
27338	H	1.174440361410	0.208945753213	-3.841375506620
27339	O	-4.971111713478	-1.579848124768	0.865367122918
27340	H	-4.466255266234	-0.814620862603	0.507544026187
27341	H	-5.458533770962	-1.244276902090	1.628426804595
27342	O	-1.126963261044	-2.637636717782	-3.824437549897
27343	H	-0.579032996694	-3.379206366104	-3.545963710558
27344	H	-0.521346681098	-1.899971543341	-4.009040995493
27345	O	-1.556622681064	1.220894435903	-5.175830863685
27346	H	-1.782767237360	1.013895235740	-6.082785713729
27347	H	-2.372624366398	1.077251696693	-4.638026307254
27348	O	3.852820091892	-1.511408393749	1.366530981395
27349	H	3.868393872417	-2.139104363904	0.624754870107
27350	H	3.364637539195	-1.968638269549	2.068671461043
27351	O	-1.352473066596	-3.765260980585	2.710750261795
27352	H	-2.221309834058	-3.912939674304	2.276320193353
27353	H	-0.744626584198	-4.468407519912	2.458119516150
27354	O	2.837056183700	3.675122024489	-2.778770990443
27355	H	2.336142723519	4.100230844737	-2.070963826089
27356	H	3.715031538186	3.477603054845	-2.394573033229
27357	O	1.050834890183	-4.977418055688	1.671672556577
27358	H	1.696931149299	-5.304327737944	1.023174035239
27359	H	1.516715698421	-4.282561246910	2.188022717759
27360	O	3.853753333774	-3.052622497427	-0.937710879596
27361	H	4.713886819019	-2.916879117021	-1.349582387154
27362	H	3.221099569836	-2.691371173500	-1.594638621684
27363	O	-2.460006770516	4.067713320310	0.092809106621
27364	H	-3.236321678142	3.921922643259	-0.465672769301
27365	H	-1.714201111193	3.688908928800	-0.450431701810
27366	O	-3.068546328900	-1.358141356664	-2.459286348669
27367	H	-3.896206646738	-1.868887257162	-2.383639652776
27368	H	-2.444420691579	-1.871784053882	-3.010520148648
27369	O	-3.778906058053	-3.918943560824	1.576170742265
27370	H	-3.559702547061	-4.248927693079	0.685117276032
27371	H	-4.197000893331	-3.055277418983	1.386235501020
27372	O	-5.687179052576	-0.472132240503	3.340524149441
27373	H	-6.426435386826	-0.338458545549	3.934149814477
27374	H	-5.539810409368	0.399531276329	2.881993385358
27375	O	-1.397103555997	3.715256324605	-3.996342627270
27376	H	-1.263705288289	2.887312165806	-4.488938529737
27377	H	-2.339383477907	3.891610364076	-4.004411838331
27378	O	-3.690277589733	0.956256442555	-3.603613870174
27379	H	-3.698455295202	1.661807663566	-2.938908623306
27380	H	-3.402085493731	0.124858277940	-3.150608095682
27381	O	-5.424300631692	-2.545453328194	-1.716326029845
27382	H	-5.920664351299	-1.770509934669	-2.027340262154

```
H     -5.365578029365   -2.404461950342   -0.757269481541

Cope TS Water45-3
155
ANGSTROM
O      1.922205438362   -0.764028278743    3.399035216395
H      2.536718911824   -0.289304551282    2.801682163116
H      1.552181435209   -1.532338855606    2.925539364795
O      0.572127548512   -3.000471646256    2.732771196467
H     -0.071793359146   -2.939507461798    3.458104455999
H      0.118236575351   -3.504886734811    2.025339840501
O     -6.099502175409   -2.495147658280    0.733869543082
H     -7.003765709475   -2.672445634332    0.990487199467
H     -5.761227126559   -1.796090300963    1.336857543814
O      2.203394128647   -6.081498211321   -1.291346151442
H      2.099336488983   -6.847508830323   -0.726788223267
H      2.623628596527   -5.382477597760   -0.738739520590
O     -0.688670118292    0.022426953469    3.915703557614
H      0.243050705724   -0.184433887004    3.729368612791
H     -1.053978997301   -0.808271617240    4.263099988308
O     -1.817153147808    3.712373483142    0.818316411020
H     -2.313442101483    4.370901654652    0.324956507327
H     -1.717657129273    4.025788499200    1.732370586964
O      1.362015327911    1.574750743545   -3.467571781930
H      0.550497853232    1.027560916615   -3.463031260496
H      1.200387192930    2.228694994274   -4.155839116116
O     -0.487606623080    3.293646726027   -2.200528397768
H     -0.509119528388    3.299957063079   -3.184066667137
H      0.426944394872    3.140697800246   -1.954031399021
O     -1.073323542833    0.354022644565   -3.563138976521
H     -1.442864193379   -0.549716588456   -3.598406603502
H     -1.826685497686    0.942834955310   -3.343855907454
O      1.544080605139   -1.008778715778   -5.663744740640
H      1.083015504161   -1.605490935520   -5.054029104878
H      2.340016502727   -0.719494540194   -5.196633142451
O     -3.778396102954   -2.958750106403    3.502522322926
H     -4.200657523701   -2.111563404042    3.292335149599
H     -3.674274915852   -3.418331943487    2.651212430946
O     -2.806389254381   -3.982211722853   -1.882755870130
H     -1.966033591074   -4.462039719115   -1.865837419482
H     -3.146092187362   -3.983974618386   -0.975096038783
O     -4.376102622376   -0.575484396727   -3.987735282166
H     -3.633155473858   -1.157691040929   -4.197835371836
H     -4.660742020223   -0.851267039997   -3.101688942248
O     -4.747254452962   -1.093119760122   -1.270054905606
H     -5.374714815634   -1.674440834513   -0.820840325627
H     -3.943297295297   -1.111635323421   -0.718254801856
O     -4.794298674106   -0.672855913504    2.233776380912
H     -4.009370635504   -0.746935592732    1.654043033712
H     -4.939781974160    0.276924511200    2.295648014785
O     -2.374852484540    5.129105795249   -1.799019757953
H     -1.864001545824    5.935914591488   -1.881788883406
H     -1.718660842641    4.398491302380   -1.771448627241
O     -3.774512442647    1.815308416475    1.329110367793
H     -4.347758956426    1.790348198145    0.534430330406
H     -3.014322198975    2.358407435608    1.050628155954
```

27439	O	-1.663122457457	4.175121066238	3.552633962897
27440	H	-2.094492256575	4.820390979540	4.112617208571
27441	H	-2.130986891249	3.311539317351	3.719123166992
27442	O	3.571852134256	0.288363293648	-4.146059147591
27443	H	4.049092888647	0.918344966189	-4.686762160195
27444	H	2.767715096041	0.758728689552	-3.810638867465
27445	O	0.644374727031	-2.934599332022	-3.764979510320
27446	H	1.454893708615	-2.752818371452	-3.249396766242
27447	H	0.898428580564	-3.640324967256	-4.407120460832
27448	O	5.703567040197	-2.887624702619	-0.557062084007
27449	H	5.383864614536	-2.115087503902	-1.066811215068
27450	H	6.411279488649	-3.278250046836	-1.070343710996
27451	O	-2.053656814487	-2.145279907047	-3.725209196956
27452	H	-2.451972247810	-2.747065158178	-3.066343914226
27453	H	-1.190785061671	-2.529567585299	-3.932270197658
27454	O	5.100559858012	1.253920255153	-0.116767217123
27455	H	4.645204638576	1.064284107589	0.728895829699
27456	H	6.037538608834	1.247900971136	0.084895189976
27457	O	-5.106181882377	1.623137833647	-1.009899398957
27458	H	-5.178323969197	0.659028432638	-1.117535315206
27459	H	-4.481360609672	1.896954052273	-1.701596043105
27460	O	-3.342837103241	1.721659291081	-3.162893766439
27461	H	-3.828666837688	0.992999128018	-3.616201812075
27462	H	-3.368277870731	2.513567664902	-3.720417659153
27463	O	2.926212456999	-2.788533311161	-2.329332519356
27464	H	2.858898074431	-3.186531037078	-1.446287424139
27465	H	3.491579510873	-2.005539539177	-2.219030139786
27466	O	2.328106555319	0.944536513688	5.621376642931
27467	H	2.025994773161	0.214832680138	5.064983155805
27468	H	1.832233242554	1.715562381385	5.299843032285
27469	O	-0.322802956661	1.013781252717	-6.070986095050
27470	H	0.408974927363	0.390574118386	-6.209839646754
27471	H	-0.763289769997	0.667477981818	-5.271332060313
27472	O	4.856264664944	0.750739437581	4.889549717182
27473	H	5.407885336362	1.366521862236	5.370398102996
27474	H	3.937117917616	0.865074375017	5.243603975304
27475	O	4.233699261805	-2.029573026310	4.258359786122
27476	H	4.752541830043	-1.326597145867	4.667680556002
27477	H	3.363202138269	-1.618907086532	4.100605866548
27478	O	3.552997493995	-5.142389358139	-3.577042878175
27479	H	3.477692079092	-4.240477117992	-3.213695462439
27480	H	3.145105797967	-5.708427608071	-2.906440656562
27481	O	0.545850236633	2.578128779071	4.329599287704
27482	H	-0.022034700244	1.799744752116	4.255349376325
27483	H	-0.008737204067	3.319821001172	4.053274874060
27484	O	-3.586440418858	-3.686250229399	0.813060046968
27485	H	-4.541868003899	-3.567871771607	0.700041704554
27486	H	-3.216214130937	-2.771718724735	0.744620502324
27487	O	-0.119019656050	-4.659696149491	-1.748276738013
27488	H	0.640815561883	-5.264564644934	-1.697358904895
27489	H	0.102598862191	-4.016281933656	-2.449049580472
27490	O	4.116714783463	0.413588153290	2.272975043411
27491	H	4.399793073608	0.761654820533	3.135966307573
27492	H	4.717808972618	-0.354080384020	2.127480096507
27493	O	5.760693250188	-1.649420165838	1.951034794415
27494	H	5.746053961082	-2.201554859714	1.155711069881

```
H      5.380245738376   -2.155446121800    2.684742698288
O     -1.421362003242   -2.542042807369    4.596055613368
H     -1.468957555964   -2.873036845508    5.492818026030
H     -2.308430620062   -2.717873776996    4.187136591093
O      3.317037889263   -4.080365787402    0.081954408622
H      4.240451417743   -3.823105380892   -0.071911723783
H      3.206123392634   -4.127922895802    1.058648447371
O      4.710230504748   -0.748508712249   -1.896291228599
H      4.432946740508   -0.352240824289   -2.742267234292
H      4.761110928717   -0.004535105553   -1.259298185183
O     -2.844024688893    1.838659349487    3.866319319146
H     -2.147797721055    1.166412149949    3.872583228664
H     -3.271701355110    1.773521531730    2.993828829594
O     -0.786796669773   -4.391129697870    0.898899908916
H     -1.748805980754   -4.369098817030    0.928647717843
H     -0.532040469636   -4.468239667064   -0.037889227106
O     -3.178575732658    4.256533220445   -4.253619417506
H     -2.964252670718    4.594481881083   -3.355260602724
H     -3.994564362566    4.681518099957   -4.518683288461
O      1.633827311502   -4.774036878776   -5.391082919675
H      2.392597833175   -5.029467294979   -4.813678714725
H      2.007775663590   -4.384617054543   -6.182505788328
O      3.047987524423   -4.132449150415    2.730275806820
H      2.126148024229   -3.825811846446    2.825886939313
H      3.569378447175   -3.598930705729    3.341726015163
O     -0.489971488067    3.425451350823   -4.890565807749
H     -0.446813573185    2.622012517436   -5.453574931270
H     -1.393605215857    3.754459641813   -4.970840474784
C     -0.225160147158   -1.170627359272   -0.857025121102
C      1.066450071434   -1.562353025434   -0.231338154417
C      1.966534342873   -0.632625973124    0.113601589357
C      1.619393933129    0.850418537979   -0.025671206335
C      0.218543792473    1.136891719673    0.670417213813
C     -0.978004945580    0.350295837536    0.085975266589
C     -1.579490126453   -0.755721567346    0.806014226956
O     -2.742537885576   -1.206707080945    0.593437035811
H      1.225586993283   -2.618190202750   -0.032211454419
H     -0.912204690575   -1.990738858060   -1.059171688891
H      2.920006735643   -0.893468014578    0.555886055897
H      2.381353011969    1.482366476871    0.437910652669
H      1.554082012579    1.131086721002   -1.085180535926
H      0.318848024004    0.917309517661    1.737805002550
H     -0.021370566279    2.201288978656    0.579919121273
H     -0.107265365820   -0.573411082290   -1.762060552231
H     -0.979908428202   -1.253033085601    1.582644504738
H     -2.623101682892    0.684156972307   -1.121956729949
N     -1.792775158138    1.098934698339   -0.736825456433
H     -1.410768118739    1.860105881649   -1.277998132900

Cope TS Water45-4
155
ANGSTROM
O      2.388882701993    3.892460511699   -0.372459218379
H      3.341530103264    3.669827808407   -0.309005665181
H      2.037345287632    3.271557228896   -1.020861031748
O      4.631861373478    2.269787014176    2.707661395331
```

27551	H	4.389466679570	1.327840728180	2.859816562867
27552	H	5.291755962019	2.493955670589	3.363755234753
27553	O	3.330756520183	0.573144025514	-4.729934345451
27554	H	2.677860794658	-0.133922316042	-4.566943789362
27555	H	4.201977295735	0.161500096293	-4.614910049972
27556	O	1.281251063476	0.090350532447	3.679579805581
27557	H	0.969574454156	1.009039297754	3.756593147308
27558	H	1.190987862166	-0.275974786063	4.588462949014
27559	O	4.873386652503	2.892377283971	-0.014535924896
27560	H	5.000191283068	1.996762582735	-0.372250000328
27561	H	4.887901544019	2.779115934220	0.947404639927
27562	O	-4.138235650519	-1.428255252913	2.899745420076
27563	H	-3.927255757885	-2.333029410857	3.176237343530
27564	H	-3.681248749856	-1.310660454136	2.044045047579
27565	O	-4.450601046562	-2.048302974204	-1.077959551967
27566	H	-4.726485682756	-2.876859673587	-0.683661025614
27567	H	-3.838386852945	-1.625473482390	-0.439297580111
27568	O	-2.501016206319	0.885613192592	-3.920475992677
27569	H	-3.219821669162	0.665033099417	-3.303437603531
27570	H	-2.586797921755	1.851196238317	-4.004339935453
27571	O	-1.977556178732	-0.326983678990	4.165143049236
27572	H	-1.836874746259	0.506995977586	3.702277785947
27573	H	-2.846177540020	-0.653030534642	3.867597642554
27574	O	3.499959052033	-2.335553316199	5.001376625516
27575	H	2.777372241663	-2.532184524116	4.357982985200
27576	H	3.970296938668	-3.155200062162	5.150602188900
27577	O	5.500759483232	-0.873804792543	-3.773372027277
27578	H	4.747960169053	-1.164501344398	-3.205999082575
27579	H	5.864400178553	-1.664290459654	-4.171305643037
27580	O	1.860534206981	4.244512334218	-3.724513108172
27581	H	2.167379748583	5.073650513822	-3.355258619264
27582	H	2.388022395334	3.531447653801	-3.291241119688
27583	O	5.204284626670	-1.499162632973	0.981094274764
27584	H	5.324418944958	-0.789085411333	0.330322072058
27585	H	4.771553486556	-1.085912935857	1.743951451571
27586	O	1.435520162425	-1.314976248580	-4.121230129209
27587	H	1.192450591402	-2.249197729709	-4.022879787795
27588	H	0.685017489436	-0.892810213132	-4.591584270176
27589	O	1.175813729060	-1.095813995191	6.090082567979
27590	H	2.018541289967	-1.564479856673	6.018509300426
27591	H	0.472084135759	-1.746515443787	5.954368042226
27592	O	3.809989001604	-3.316961342311	-0.533411711801
27593	H	4.441618617552	-2.784413046715	-0.011183032183
27594	H	3.164979191789	-3.642021677630	0.119454823794
27595	O	0.283212620581	-6.492461519023	-1.278827361675
27596	H	1.174303643180	-6.211135377964	-1.564293218509
27597	H	-0.341237577147	-5.904263613253	-1.722044972365
27598	O	-1.930764400848	2.136894855503	2.734209054215
27599	H	-1.934246348680	2.774825301331	1.994790646907
27600	H	-1.158587230443	2.396978639560	3.262938943914
27601	O	3.382272471856	-1.285616422149	-2.226141923714
27602	H	2.556919831415	-1.272895654315	-2.739645121616
27603	H	3.393448522009	-2.088216627566	-1.664807842226
27604	O	2.222076334190	-4.391745795057	1.412364467824
27605	H	2.785713756894	-5.099680851569	1.726596690572
27606	H	1.314036532012	-4.814179835462	1.285572315140

27607	O	0.177758441721	1.157579138239	-3.053010024114
27608	H	0.612497015793	1.528493934715	-3.831790091465
27609	H	-0.751373403051	1.052726650805	-3.303556532594
27610	O	1.182583298932	2.176262763021	-5.510655446171
27611	H	2.034541540434	1.718209730082	-5.487018632538
27612	H	1.343889491891	3.060211426166	-5.157739249535
27613	O	-1.886353039674	4.237021965839	0.992965292367
27614	H	-2.487498976744	4.074534780918	0.216949606447
27615	H	-2.330320361520	4.907012652064	1.515338121568
27616	O	-2.268112018228	-3.759900860890	0.778338652616
27617	H	-2.534737938650	-3.961161768054	1.692938650195
27618	H	-2.378022643735	-2.793384695152	0.712458749715
27619	O	1.497182753743	-2.633047637455	3.263396066446
27620	H	1.401810577513	-1.727707589614	2.935275033333
27621	H	1.763086981717	-3.211511116754	2.516609667852
27622	O	-3.688343946914	3.948049014145	-0.891539584358
27623	H	-4.257899536147	3.225557394561	-0.577573815578
27624	H	-3.433336474038	3.725644031052	-1.801436967044
27625	O	5.151056608355	0.382881363887	-1.102950381426
27626	H	5.782576094430	0.220089835279	-1.810465527447
27627	H	4.328312009087	-0.057536514610	-1.399654706474
27628	O	-0.452998429473	3.796809384820	-2.299927756231
27629	H	-0.347184319064	4.521757419416	-1.645595708075
27630	H	0.307856620990	3.865870745971	-2.901043013679
27631	O	3.859723218038	-0.251466216602	3.164737814850
27632	H	2.891702762359	-0.067978377282	3.261109025316
27633	H	4.054404657769	-0.844432790891	3.905087856795
27634	O	-0.820942067286	-2.680698195661	4.702261798085
27635	H	-0.033173350997	-2.764998147910	4.135148008410
27636	H	-1.161500168859	-1.771063199242	4.566103567539
27637	O	-5.341257278282	1.977234114833	0.110299922103
27638	H	-6.238083183130	2.290592821452	0.228890518271
27639	H	-5.017977349469	1.728332138928	1.015708430255
27640	O	-4.591531281430	1.385564697205	2.564736813178
27641	H	-4.625773523693	0.443605197839	2.773191004257
27642	H	-3.671523149775	1.666595268467	2.705217964481
27643	O	2.300610621474	3.785213148363	2.389924800987
27644	H	2.207407098316	3.860130087357	1.424320451714
27645	H	3.128883527303	3.301263456542	2.535675302822
27646	O	-2.855894668436	-3.860989490298	3.461159334465
27647	H	-3.086522336279	-4.655616251540	3.941748913152
27648	H	-2.083771191448	-3.463116755086	3.944069334586
27649	O	0.119736069117	5.542320513851	-0.371803822506
27650	H	-0.490694145242	5.117317408855	0.257074997849
27651	H	0.994294359906	5.162455165325	-0.209628310169
27652	O	-0.077934080715	-5.487455574412	1.142424519390
27653	H	-0.817034027609	-4.875375980363	1.003049328114
27654	H	0.018716255753	-5.995848204337	0.301183028267
27655	O	-2.735238222718	3.576428991439	-3.511208876428
27656	H	-2.855553498119	4.353947463430	-4.055501557200
27657	H	-1.844205353992	3.671064893712	-3.073728660906
27658	O	3.181683970783	2.228703448563	-2.618664067418
27659	H	4.072981026424	2.305545091684	-2.271522419964
27660	H	3.208757971661	1.576694729930	-3.354376548697
27661	O	0.704690585810	-3.908592791298	-3.606838308958
27662	H	0.018691426399	-3.954385870860	-2.910172671841

```
H      0.330548665913   -4.342959136542   -4.374840944844
O     -2.699171625018   -2.275093979145   -3.192302524046
H     -3.432854310782   -2.126246407623   -2.571406232327
H     -2.494954984191   -1.428630872309   -3.598066715063
O      2.610525844692   -5.329628783261   -2.170558662183
H      2.107744388737   -4.761414523159   -2.775090310390
H      3.171620443380   -4.738776010363   -1.652997550052
O      0.484377436227    2.698077730241    3.930892095695
H      0.591490423621    3.114738652526    4.786084912436
H      1.113666973001    3.152351351246    3.311695064855
O     -0.501688344701   -0.139763559503   -5.627461045304
H     -1.302316355264    0.143622263749   -5.163322886555
H     -0.028184766494    0.672367802580   -5.852035690861
O     -4.409962354971    0.524326518787   -2.015466787182
H     -4.787771454255    1.086399187805   -1.319055895264
H     -4.696342383419   -0.372907289035   -1.808625431591
O     -1.370975210994   -4.249831923081   -1.910828424996
H     -1.915407620361   -3.635699539798   -2.438683975012
H     -1.682814346389   -4.163347000732   -0.998674019895
C     -0.272123135772   -1.024261717907   -0.882766654175
C      0.880387489506   -1.595128877874   -0.138872586047
C      1.833250123532   -0.774640765592    0.326633760350
C      1.706781211339    0.727873151609    0.094506679579
C      0.305579486315    1.275076567688    0.616917611652
C     -0.954145330247    0.571471373879    0.058370919001
C     -1.540752206429   -0.517396520797    0.810463154901
O     -2.679921543637   -1.052938985474    0.662488489147
H      0.880216307516   -2.664259639021    0.058475807381
H     -1.041003887887   -1.720494655829   -1.207219158812
H      2.678617877017   -1.130744923304    0.901312445829
H      2.505893507826    1.270334174310    0.607357811171
H      1.770567552932    0.948072939037   -0.979410841801
H      0.305739880498    1.190713822291    1.706535007049
H      0.228835155606    2.336729966669    0.363469696393
H      0.024190651524   -0.381817983663   -1.715390363628
H     -0.911747161201   -0.930871648853    1.621102263745
H     -2.586446686756    0.973179086123   -1.138159045634
N     -1.733791621824    1.361441936807   -0.758686177416
H     -1.258718344059    2.004338798887   -1.378507636280

Cope TS Water45-5
155
ANGSTROM
O      3.007289842975   -3.043574942777   -2.207933711609
H      3.741000576433   -2.905674968604   -2.811928349223
H      2.713721583082   -3.977309129366   -2.335262659604
O     -2.117014061761   -1.406865236063   -4.457068274302
H     -1.299071877073   -1.898522240050   -4.336418143620
H     -2.636310638219   -1.520261332563   -3.645637302505
O      1.802610862966   -5.352602239738   -2.756475582547
H      1.208364858629   -5.104186646044   -3.469497831318
H      1.255278718638   -5.539105703128   -1.976950379724
O     -5.921590483322   -0.980108597814   -1.651004373020
H     -5.970946305716   -1.218265032083   -0.709069954328
H     -5.193693113517   -1.506180015872   -2.011292170233
O      4.143122649025   -3.101583144861    0.349306154240
```

27719	H	3.691295351255	-3.051806267398	-0.511822942165
27720	H	4.803716104957	-2.376305458254	0.328240975091
27721	O	-4.869387561015	2.510370677819	1.146876736874
27722	H	-3.920901157276	2.714864111067	1.312549264248
27723	H	-5.258112725145	3.334615553395	0.845747251356
27724	O	0.539654517616	-2.642138011422	-3.548598778173
27725	H	-0.133854126766	-3.090702400306	-3.008598959765
27726	H	1.368986917829	-2.704575453910	-3.048124165506
27727	O	4.826488209943	1.548228924920	1.463393836636
27728	H	5.088783812394	2.365192644748	1.891445960448
27729	H	4.111682456363	1.179999863426	2.010333426414
27730	O	-1.367409941333	-4.049230406516	-2.113473819107
27731	H	-1.777243285001	-4.800724273641	-2.544592495462
27732	H	-0.770334959489	-4.426202401737	-1.424304194375
27733	O	-0.440674218358	5.049710993208	1.190248965585
27734	H	0.422554831022	4.816507211778	0.825280616311
27735	H	-0.879915528347	5.542713814152	0.455941815990
27736	O	-1.262545266317	1.581006105634	3.590102001929
27737	H	-2.054955499052	1.128386118273	3.916146355948
27738	H	-0.522187769711	1.289943950099	4.149688914170
27739	O	3.580016463768	0.321827112770	-4.595121241690
27740	H	3.775443977645	1.282041367225	-4.579362566727
27741	H	2.611651981762	0.245392340984	-4.529605806653
27742	O	-1.202718074422	1.059863816916	-5.187467983731
27743	H	-1.684008634774	0.214039861167	-5.173728311298
27744	H	-1.734062055157	1.646765720177	-4.621769073175
27745	O	-0.279886024605	-2.636742850152	5.327112703309
27746	H	-0.386592622356	-3.211974564532	6.086538312305
27747	H	-1.209195322398	-2.479748636005	4.989551243929
27748	O	-3.590458330963	0.339145318175	4.462037956148
27749	H	-3.893707188902	0.562548708964	5.343345458639
27750	H	-4.360892762917	0.437266177945	3.865887311839
27751	O	-3.232786004924	-3.237568866647	1.977005387912
27752	H	-3.018462842707	-2.466545195669	1.399834573261
27753	H	-4.175876368528	-3.377489845635	1.859519641025
27754	O	4.050060939074	-0.198637558076	-2.091042476853
27755	H	3.312349341892	-0.780682165742	-1.884758138463
27756	H	4.005606154930	-0.051791011239	-3.076079164793
27757	O	-5.766783731183	0.733501345619	2.896961467393
27758	H	-6.638689363534	1.010476879053	3.180779413221
27759	H	-5.455278968936	1.399695526142	2.241366943267
27760	O	3.904309823017	-1.338581225500	4.706987468790
27761	H	3.131459384007	-1.269872194212	5.327000892347
27762	H	4.682217889248	-1.459439573363	5.250798859731
27763	O	0.898289099332	0.099266321007	-3.901484804802
27764	H	0.131072914737	0.400658831276	-4.448451002120
27765	H	0.810519618413	-0.866216612497	-3.864497075643
27766	O	0.264372731791	-5.245889038540	-0.430283811609
27767	H	1.142245106304	-5.289639503630	0.002225352086
27768	H	-0.365474521991	-5.369726523257	0.318049130086
27769	O	2.619170808290	-5.432574483695	0.866609427424
27770	H	3.303198672107	-4.763075244771	0.730709729110
27771	H	2.406348195538	-5.444469360552	1.802937547424
27772	O	-2.696330959983	-2.289999020453	4.461142703242
27773	H	-2.989950039527	-1.367201029916	4.401236840503
27774	H	-2.872454762335	-2.695761882980	3.587406773422

27775	O	-1.280111547703	-5.267881663707	1.721716787812
27776	H	-0.597686290474	-5.060202558815	2.385334783986
27777	H	-1.971737727126	-4.596992737302	1.819041275621
27778	O	2.052614306045	4.032443070126	-0.085196093064
27779	H	2.759663635474	3.628170009884	-0.612052289996
27780	H	2.304661890838	3.866897589648	0.844282132006
27781	O	-0.129110780450	3.608491262319	-1.738399322670
27782	H	0.236875828344	3.454958314944	-2.627941657061
27783	H	0.642479375154	3.762616283526	-1.166396062882
27784	O	-4.595502156701	1.329831868169	-1.437715333531
27785	H	-4.745521066939	1.480961959174	-0.493725513203
27786	H	-5.142701017529	0.542438931736	-1.658198116070
27787	O	-2.757355069334	2.462643480260	-3.434248377448
27788	H	-2.662907897762	3.242074092249	-2.874578773899
27789	H	-3.471561880525	1.941756522934	-3.049292520675
27790	O	3.821909358119	2.968540562066	-4.038502345044
27791	H	2.882366017371	3.199003382290	-4.080865267290
27792	H	4.061265986862	2.984290961855	-3.102683786835
27793	O	-1.516040904835	5.866215512864	-1.092701158345
27794	H	-2.424658193151	5.545376125312	-1.128358747525
27795	H	-0.990461414535	5.149553205487	-1.500953256515
27796	O	2.533614879641	3.405597915051	2.533456708160
27797	H	1.739012649734	3.723201189977	3.005197039281
27798	H	2.591288073878	2.461721513724	2.722171880892
27799	O	-3.489928798348	3.905910675000	-1.042890330192
27800	H	-2.886704028660	3.518475377854	-0.393819171052
27801	H	-4.058243278377	3.178953628468	-1.326410056278
27802	O	4.104578725530	2.413353424645	-1.229044562835
27803	H	4.581449366337	2.307844084626	-0.397086878673
27804	H	4.034360343604	1.501389716038	-1.575366384585
27805	O	0.156294580127	4.031059891861	3.710794229609
27806	H	-0.452540973532	3.279076554161	3.648675682272
27807	H	-0.118055047096	4.644768482967	3.013622530506
27808	O	5.592514761753	-0.850514670620	0.085364889984
27809	H	5.233951323614	-0.605722856526	-0.785019256922
27810	H	5.495980493688	-0.057998638768	0.628156420080
27811	O	1.770732020525	-0.964695032551	6.220836779735
27812	H	1.050292340118	-1.558717648974	5.956791561161
27813	H	1.458766634303	-0.065965589081	6.032593836207
27814	O	0.811721369563	-1.098857706742	3.043275021760
27815	H	0.404022000568	-1.431331715168	3.848575974227
27816	H	1.429679642074	-1.800343340103	2.753206753887
27817	O	2.799253571129	-2.857403214736	2.646610195052
27818	H	3.376128637932	-2.458431150733	3.315296123119
27819	H	3.310387471562	-2.915945227058	1.809644295886
27820	O	-5.449875685632	-1.392976580564	1.061172451901
27821	H	-5.713993895425	-0.756778267502	1.738370924506
27822	H	-4.533658227203	-1.164472249949	0.843483773572
27823	O	-3.343727980824	-2.025340220471	-1.989514094004
27824	H	-2.816969641905	-2.836403633163	-1.967416943205
27825	H	-3.195018654225	-1.585199165029	-1.133037300167
27826	O	0.730144768623	-4.469536579062	3.441603450808
27827	H	0.342150550600	-3.898706412953	4.121246204178
27828	H	1.494108892541	-3.974895806548	3.103940561181
27829	O	-2.309953351746	3.119535857300	1.510491014038
27830	H	-1.669956762707	3.856446708566	1.413036061759

```
H      -1.922226134440    2.506016810334    2.147948205345
O       1.036730239920    1.498449621971    5.127002147654
H       1.737339159258    1.329817695135    4.464821605767
H       0.999850316040    2.451900927562    5.244487604808
O       2.820754605707    0.653500636670    3.233906673973
H       3.395686297933    0.053552591698    3.744962463436
H       2.059232111449    0.081515725933    2.981193664066
O       1.016717523988    2.858507628072   -4.077517586490
H       0.425187954667    2.886853859318   -4.834472959752
H       1.108966432979    1.915700297038   -3.866761386086
C      -0.213750191729   -1.074886000292   -0.892259475847
C       1.033598367714   -1.513096328803   -0.221138482987
C       1.940683145523   -0.605235137143    0.167564001805
C       1.631799231621    0.876767223191    0.000854900440
C       0.239856414937    1.219998574213    0.683070109058
C      -0.977318417372    0.512101289550    0.055403456568
C      -1.617380990385   -0.579846427938    0.760416632120
O      -2.761632652321   -1.060507709035    0.518902991493
H       1.150683951688   -2.574776824448   -0.018089421216
H      -0.923799092117   -1.864542863388   -1.114390907696
H       2.859329716901   -0.874608564005    0.673250348723
H       2.408224090416    1.492431267364    0.453785517569
H       1.562186772621    1.151099325376   -1.060092983089
H       0.300847455757    0.960130195618    1.743651936936
H       0.080304611784    2.298192037500    0.600184827673
H      -0.057636713953   -0.450820847891   -1.773409583311
H      -1.038629435071   -1.036096671288    1.579574471513
H      -2.562373711828    0.944978128259   -1.212338059122
N      -1.704315037641    1.294725362887   -0.811883725398
H      -1.227937732949    2.008761793763   -1.348549926533

Cope TS Water45-6
155
ANGSTROM
O      -4.088138941576   -1.618303749471    3.086675948393
H      -3.706996173014   -1.480230845104    2.204171142527
H      -4.078509808875   -2.592112592361    3.227633629610
O      -2.483675742225    4.284113225444    0.849795092751
H      -2.526917749854    4.327137681402   -0.115211650338
H      -1.542998209910    4.403220858590    1.062360896600
O      -0.315622106713   -1.636447028636   -5.111335887903
H       0.472438261050   -1.935247159123   -4.618649262435
H      -0.019592937052   -0.841727566627   -5.587389547990
O       3.878881262196    1.057608371530   -2.136985478139
H       4.212901412920    0.883047614734   -1.237485520179
H       3.621090568036    1.999699349301   -2.179271990642
O      -2.038420494218   -0.796840674030    4.720051015364
H      -1.604793270653   -1.633996316131    4.990097983672
H      -2.784882331257   -1.054484463374    4.144554109125
O       1.686513902796   -2.243837898407   -3.397386087656
H       1.348724648209   -2.975317442122   -2.841261411005
H       2.653842177149   -2.234636990754   -3.347257497374
O      -4.445397928984    1.086910864782   -2.108639720006
H      -4.619338501075    0.196012258221   -2.423517720000
H      -4.770569924112    1.113847020162   -1.188369582176
O      -5.041241838717    0.920249095813    3.966204122779
```

27887	H	-4.898909602045	-0.011914374446	3.758737727085
27888	H	-4.531935446869	1.410244028918	3.300237645050
27889	O	-1.621158518487	2.251182126634	-4.651145414929
27890	H	-2.435400047257	2.768945027035	-4.456840719062
27891	H	-1.804344794971	1.379426252351	-4.238817101646
27892	O	2.608189761953	3.435155237915	-2.542932608000
27893	H	1.647211936979	3.274458785910	-2.496952327523
27894	H	2.793869701914	3.428539444230	-3.496351631845
27895	O	2.005715807453	-1.090397599231	3.327245648154
27896	H	1.387066882665	-0.440016418442	3.707582991050
27897	H	2.822463698815	-0.577044688522	3.096435703013
27898	O	-6.322122068714	-1.575944537670	1.253016017948
27899	H	-6.095318970240	-0.682718751506	0.948691667711
27900	H	-5.857484020970	-1.678992551329	2.093017503238
27901	O	-0.527934387157	-2.969848168630	5.066589357524
27902	H	0.351248653134	-2.882441000888	5.451103809042
27903	H	-0.385148647389	-3.043306968723	4.103632446444
27904	O	-3.670306445108	-1.692298864995	-2.089094406062
27905	H	-3.121253401347	-2.411199763673	-2.439419972506
27906	H	-3.399671362930	-1.545946582329	-1.165098779710
27907	O	-3.265553505238	1.324333022877	5.951574658470
27908	H	-4.079447921687	1.242205147919	5.408368763414
27909	H	-2.763850708351	0.521537092527	5.732827735574
27910	O	-2.199145263651	3.269860876314	4.587794707395
27911	H	-2.773853557804	4.024097661068	4.720546858642
27912	H	-2.543207841665	2.551467473424	5.193205165077
27913	O	-0.038867677983	2.659868596085	-2.590283819635
27914	H	-0.577259670258	2.650817639528	-3.423350062908
27915	H	0.347841317863	1.766491454938	-2.581313541273
27916	O	-3.186038180497	2.114435928545	2.256437574591
27917	H	-2.746257324849	2.410514172964	3.071809990175
27918	H	-3.027690535719	2.857865458560	1.637628777241
27919	O	1.597276647696	-5.054940736881	3.821485544634
27920	H	0.938372886422	-5.496663585424	4.361042154198
27921	H	1.998424460885	-4.362052201037	4.389255689863
27922	O	2.737585265803	4.120471787805	0.270185660512
27923	H	2.818528825254	3.967483965913	-0.678771806101
27924	H	1.797384156135	4.275962171009	0.449412536320
27925	O	1.392382482131	0.375608565976	-2.998259867306
27926	H	1.423751411989	-0.599358371217	-3.067362531832
27927	H	2.291457525104	0.638859387024	-2.725579442236
27928	O	-3.248679225287	-3.929524864307	0.495293017870
27929	H	-3.060844007531	-2.976045021665	0.592002597606
27930	H	-4.112287029943	-3.939667586280	0.016878012271
27931	O	-4.033778842023	-4.277844762236	3.030114754900
27932	H	-4.863179032879	-4.756125207729	3.029920840598
27933	H	-3.692160723111	-4.307570706989	2.107567396511
27934	O	4.313591783922	-1.350576369034	-3.344050796882
27935	H	4.183187896998	-0.454566170702	-2.977015650230
27936	H	4.846638529407	-1.246507572608	-4.132531505427
27937	O	0.178962448739	0.605482908096	4.434289000453
27938	H	0.378095353270	0.775896636350	5.356957423648
27939	H	-0.698368246388	0.150944806192	4.435341118862
27940	O	-3.759274871416	3.371163346953	-3.547330990735
27941	H	-3.295484927439	3.881486244110	-2.867130411803
27942	H	-4.114997638563	2.593313592329	-3.087467265377

27943	O	0.348110668013	-3.063383058913	2.459688787073
27944	H	0.975724794719	-2.320020101185	2.521004803366
27945	H	0.849909061022	-3.841004979080	2.772808302649
27946	O	1.791177270701	-5.927062651227	1.094755241534
27947	H	1.868125282690	-5.764730283841	2.042357910524
27948	H	0.889052553696	-5.681004198164	0.841748880550
27949	O	5.089279424851	0.151141919505	0.174983510608
27950	H	6.021985372998	0.368959199194	0.197959917540
27951	H	5.036130791202	-0.800088237654	-0.107212895548
27952	O	2.367025366854	-2.878745959082	5.218246528297
27953	H	3.251360765382	-2.792294574804	5.573941971879
27954	H	2.289755572628	-2.193154669099	4.500494138478
27955	O	0.390318661909	-4.236657873544	-2.156423028556
27956	H	0.115972110808	-4.385743192455	-1.229164232363
27957	H	-0.433084318086	-4.071454950518	-2.637629611108
27958	O	4.951534171873	-2.301093198510	-0.788562693400
27959	H	4.824209654054	-2.111744192382	-1.729872971403
27960	H	4.270625474254	-2.961818649262	-0.573702592426
27961	O	4.097704741005	0.409490470560	2.794855086788
27962	H	3.826772133447	1.343617011775	2.873971471072
27963	H	4.459701525054	0.321350957722	1.898120727253
27964	O	0.367418473077	3.262244960246	3.699506365610
27965	H	0.429444336673	2.294476491560	3.764048423198
27966	H	-0.509870834031	3.449854953249	4.080333403636
27967	O	-1.873708631663	4.571543244489	-1.840307283717
27968	H	-1.157083622195	3.932010155346	-1.994284589609
27969	H	-1.558171231123	5.421557836914	-2.147820009885
27970	O	2.918200890558	-4.156117582538	-0.626785344740
27971	H	2.373150365743	-4.395369192764	-1.381548840911
27972	H	2.697006527224	-4.818501659092	0.055895938120
27973	O	3.179875828360	2.987385799204	2.766849923597
27974	H	3.186520130728	3.425371004074	1.899141352917
27975	H	2.353876043905	3.236086185836	3.193894478012
27976	O	-4.857275677596	0.671747818007	0.529462188000
27977	H	-4.492204500841	1.351720993528	1.111799857448
27978	H	-4.159051874428	-0.007807600250	0.493478758597
27979	O	-1.913450978766	-3.348253356085	-3.561503499563
27980	H	-1.429186355816	-2.785634890365	-4.190173882788
27981	H	-2.356498181041	-4.020563276341	-4.081133604636
27982	O	-0.571756207623	-4.541835651070	0.361687202822
27983	H	-0.296516569495	-3.906125860539	1.048557948235
27984	H	-1.542291705638	-4.539133368294	0.372403770351
27985	O	-1.752480924777	-0.086004067459	-3.369968924200
27986	H	-1.300250155407	-0.686788558182	-3.988610440123
27987	H	-2.560528358039	-0.527050247187	-3.072319744520
27988	O	0.220299902521	4.614328550525	1.416346696138
27989	H	0.357400250872	5.526626473820	1.676155942723
27990	H	0.309459832080	4.081270560607	2.243787637271
27991	O	2.575435210864	2.975387577490	-5.275372298607
27992	H	1.959317383630	2.238106290260	-5.462258165662
27993	H	2.566808280295	3.538399495612	-6.048715279903
27994	O	-5.543322885533	-3.342106484569	-0.670092059382
27995	H	-5.228973545279	-2.740133202815	-1.357101529747
27996	H	-5.953101285665	-2.771783737229	0.013175710914
27997	O	0.695747482096	0.972442955001	-5.523948698477
27998	H	0.946242826010	0.761803846273	-4.596197086501

```
H     -0.116393543080    1.497424303658   -5.458671936860
C     -0.331061548988   -1.088312927658   -0.712463420327
C      1.039764267656   -1.399901969143   -0.200618959610
C      1.879767760930   -0.459495808022    0.238044788292
C      1.450552265156    1.002144769981    0.353577012309
C      0.022781032724    1.097278804490    1.027353194939
C     -1.113207360195    0.376849586745    0.264331430506
C     -1.750696345068   -0.766623930683    0.871437189555
O     -2.899381441012   -1.238216187896    0.578686078948
H      1.332769526569   -2.445415497505   -0.198811373569
H     -0.937260607001   -1.981436816319   -0.875211080734
H      2.870045331275   -0.721737293483    0.583873993292
H      2.167596842625    1.572373888110    0.948298415568
H      1.397955978171    1.473595101823   -0.634345425284
H      0.094293563030    0.693993615757    2.041886977564
H     -0.262752574897    2.150757040533    1.102488141899
H     -0.342351210953   -0.504848077371   -1.635436068590
H     -1.193880240195   -1.283302800146    1.668148086946
H     -2.610264870572    0.874292801835   -1.105195204107
N     -1.826590517888    1.224518357021   -0.572046486841
H     -1.292228539720    1.896623698397   -1.102312437075

Cope TS Water45-7
155
ANGSTROM
O     -0.521872243042   -4.139583403818    1.174401984355
H     -1.441313967293   -4.370794073729    1.384078571065
H     -0.421904136722   -4.249789015850    0.206641432442
O      3.724672044276   -1.563509564692    4.498113219140
H      2.938002752294   -1.065231961304    4.161932343101
H      3.934418331739   -1.192645216406    5.355030028634
O      1.528797788029    4.295410438767    1.498604973496
H      1.678046541180    4.248589366243    0.546409197732
H      0.571158167347    4.382390954556    1.631813141599
O      2.337631192997   -5.361132089321   -0.434246514563
H      2.120644270474   -5.783533718579    0.424888289903
H      1.482445183710   -5.225686213380   -0.859801819621
O      3.824247787115   -4.281907607364   -2.526781374683
H      3.038840369563   -4.292617022619   -3.085975621762
H      3.553233861688   -4.742435992905   -1.717852801969
O      1.092563925119    4.043075293304    4.905293503930
H      0.226014471297    3.611409635108    4.898746347586
H      1.036753546536    4.769750552700    4.279766339598
O     -2.281688128753    0.836616857054    3.120961298938
H     -3.237178245798    0.969203530020    3.329948368871
H     -2.039060405198   -0.004702658954    3.571140193370
O     -4.821530486272    1.499413008732    3.534324153870
H     -4.777801860104    2.162025333017    2.830386479112
H     -5.370726243197    0.774414274076    3.191718264447
O      1.273805995588   -3.066393766891   -3.191529752985
H      0.823036171222   -2.533877770305   -3.861411520704
H      1.850623140893   -2.458209885685   -2.706282594494
O      6.649592176733    0.230142354072    0.498104756667
H      6.460635048226    1.040147227941    0.983817933429
H      6.224093562755   -0.473853401860    1.018198633272
O     -5.014718071550    0.920561222282   -2.506620597356
```

28055	H	-4.434392860013	0.167237215340	-2.713899528208
28056	H	-4.560136949260	1.691616161840	-2.880417221553
28057	O	1.400568337162	-5.969434891117	2.007671298103
28058	H	1.925467307970	-5.544654024723	2.697031299580
28059	H	0.653654063803	-5.368735281137	1.833223111334
28060	O	0.494058303600	-1.171309150186	-5.088057213642
28061	H	1.410799293496	-0.827989109136	-4.984958544507
28062	H	0.287030258644	-1.133537768307	-6.022020292348
28063	O	-1.151245642355	4.216962973843	2.171331809319
28064	H	-1.207348681733	3.766111483944	3.028178833133
28065	H	-2.004022164214	4.097305926592	1.743792695995
28066	O	-0.774694659250	0.748360545546	-3.577572498043
28067	H	-0.421424845168	0.017318448056	-4.111540928356
28068	H	-0.218454025489	1.506707437090	-3.859882435726
28069	O	-3.164336273240	-4.613823714994	1.874795387210
28070	H	-3.361801618386	-5.324492259221	2.484536432862
28071	H	-3.462513905914	-3.775564195838	2.296983192322
28072	O	-1.827775282353	-1.448450911644	4.397714884122
28073	H	-1.060930298980	-1.952278014794	4.051631988578
28074	H	-2.618535571963	-1.851887683868	3.998671832938
28075	O	5.285886296588	0.350856179924	-1.752865947557
28076	H	5.744402666673	0.323443838383	-0.873136381856
28077	H	5.991520332140	0.425978590905	-2.396649666756
28078	O	-2.970144401711	2.696121463616	-3.349358451278
28079	H	-2.992476011724	3.135595928273	-2.464716204018
28080	H	-2.306770918153	1.991574275217	-3.317336723685
28081	O	-1.388072720898	2.756199033787	4.585871844675
28082	H	-1.689349192397	2.005720438015	3.987561087967
28083	H	-2.158650048908	3.015838196950	5.093428350751
28084	O	4.666059255485	1.526731900697	2.077018856520
28085	H	3.912610438463	1.801362032441	2.626777882294
28086	H	4.613197448724	0.558323183559	2.016724662188
28087	O	-0.261839764208	-4.492535522149	-1.507173087820
28088	H	-1.136303181876	-4.353178138912	-1.905450393027
28089	H	0.337830391496	-3.985945390526	-2.092690849363
28090	O	4.915733302849	-1.255699468677	2.013696742580
28091	H	4.478410885818	-1.996593277743	1.553442327046
28092	H	4.716628780982	-1.406355338476	2.951127401180
28093	O	2.482504846392	3.865415327243	-1.270729864533
28094	H	2.730836673101	3.179749329908	-1.909073470903
28095	H	3.255530019737	3.914665931515	-0.671874733426
28096	O	3.203890855291	1.797516475188	-2.960681670970
28097	H	3.025854548873	1.137581567468	-3.646082906078
28098	H	3.897219787089	1.402970100521	-2.407446390490
28099	O	-2.870280612653	4.019163071828	-1.024524994637
28100	H	-3.181094168693	3.555252923018	-0.228850521617
28101	H	-1.903749254159	4.050647604373	-0.958547875245
28102	O	-3.864264059211	-2.177241508848	2.720969405384
28103	H	-3.432391929268	-1.772721609313	1.927848759203
28104	H	-4.746349679214	-1.776971069554	2.727807730951
28105	O	-0.213286399544	3.612254705153	-1.562118985407
28106	H	0.739315051213	3.740127726412	-1.417864660847
28107	H	-0.406998443231	4.003160114751	-2.429465592175
28108	O	-4.792432891699	0.576686702741	0.114304693420
28109	H	-4.034854484483	-0.053334492000	0.141649044785
28110	H	-5.000696859546	0.717607491289	-0.840032867257

28111	O	-1.132617536280	4.540837042143	-4.015093487528
28112	H	-1.885532471497	3.905933673022	-3.948570342430
28113	H	-1.521035302626	5.414867542572	-4.053021771822
28114	O	1.618123793850	-0.188257946163	3.617064725120
28115	H	1.929134333885	0.703440568316	3.363360216171
28116	H	1.057251313681	-0.035687368265	4.409976579577
28117	O	-6.065700848619	-0.553880168234	2.150773662503
28118	H	-5.675283109932	-0.165099402280	1.330144558539
28119	H	-6.986335773033	-0.728250798802	1.956564050311
28120	O	3.044065587486	-0.472134764888	-4.653492307322
28121	H	3.733382244641	-0.736633395490	-5.262692669120
28122	H	3.223668064512	-0.950382836760	-3.795620136746
28123	O	-2.614278124445	-3.583171951029	-2.659125216024
28124	H	-3.188414110072	-3.541778888957	-1.845507361722
28125	H	-3.120516816239	-4.053301676890	-3.321400521052
28126	O	3.450432249613	-3.336733183197	1.144852842505
28127	H	3.194668491828	-4.015074980017	0.502513948381
28128	H	3.187489734537	-3.678013983136	2.018450193712
28129	O	-0.058783234561	0.376064474189	5.631465055949
28130	H	-0.422444163105	1.250807673822	5.440041356235
28131	H	-0.766249877476	-0.265673962813	5.482208986640
28132	O	0.856421942149	2.653846492541	-4.517725673500
28133	H	1.640066568891	2.754592166256	-3.967092694216
28134	H	0.355283664152	3.479361203049	-4.483867791533
28135	O	3.509959729086	-1.627982889121	-2.364494578389
28136	H	3.863715099625	-2.541899489618	-2.313319662430
28137	H	4.148991794513	-1.026532505485	-1.943642121015
28138	O	0.318262120977	-2.580225206830	3.218498430505
28139	H	0.840182058010	-1.783744672966	3.028558123957
28140	H	0.098208522894	-3.025805303959	2.378042992318
28141	O	4.436799341799	3.797951324736	0.593391349110
28142	H	4.591564023775	2.931290199872	1.014997432618
28143	H	4.074461902654	4.360248038442	1.281797956007
28144	O	-2.935073504525	-0.789755533243	-2.729533418814
28145	H	-2.191224876836	-0.332321263610	-3.143484847502
28146	H	-2.757871443178	-1.740686829308	-2.789378709738
28147	O	2.345056081125	2.373824702033	3.198266782347
28148	H	2.085817419952	2.846927396629	4.010587900203
28149	H	2.070532632858	2.984948345900	2.481850590977
28150	O	2.599735559428	-3.997215607396	3.692487125774
28151	H	1.718633799552	-3.576710942110	3.685546037762
28152	H	3.176896918522	-3.345851743912	4.116935127333
28153	O	-3.996274979239	-3.338528692234	-0.464538669601
28154	H	-3.769553541245	-3.971555289259	0.231290445040
28155	H	-3.626430932921	-2.486025405386	-0.169927786207
28156	O	-3.538831221682	2.616623451121	1.259065443514
28157	H	-2.809509242528	2.093805615836	1.616452756104
28158	H	-4.083519070650	1.955012567325	0.771515128745
28159	C	-0.226175499512	-1.184403161982	-0.818143774820
28160	C	1.077547362279	-1.497899997595	-0.193154412632
28161	C	1.946567403131	-0.536815014068	0.156493748273
28162	C	1.574474739084	0.931489521844	0.015798723015
28163	C	0.191827687003	1.200425718994	0.767386043616
28164	C	-1.010608840024	0.482965495261	0.152844525229
28165	C	-1.595793092182	-0.683748028978	0.754058631885
28166	O	-2.782636959014	-1.112718039844	0.549439802463

```
H      1.293401513335   -2.540639929315    0.017850151811
H     -0.881004464836   -2.029406211107   -1.013867178360
H      2.890769200233   -0.781256413151    0.623974738123
H      2.345104570965    1.578418405887    0.440432517598
H      1.444178960757    1.209033959438   -1.037935863671
H      0.308371089160    0.908992352159    1.815665129858
H     -0.012795658355    2.274995402219    0.731449625406
H     -0.177601246284   -0.538258200217   -1.694628377295
H     -1.021255973022   -1.177004687666    1.550469215966
H     -2.460203536538    0.759496072269   -1.262942978687
N     -1.738070649611    1.233582980407   -0.737883470781
H     -1.229962244788    1.943259172890   -1.253950366678

Cope TS Water45-8
155
ANGSTROM
C     -0.258836963130   -1.276328152334   -0.768423044147
C      1.073343178502   -1.584723454387   -0.203510573095
C      1.980050789971   -0.626477262471    0.031462332793
C      1.606571105672    0.839304506694   -0.128697904717
C      0.270570236717    1.129012079763    0.689717284438
C     -0.967807312555    0.465210758912    0.093025010223
C     -1.624034597852   -0.637605601021    0.736835922776
O     -2.806278567294   -1.050803347925    0.480959042355
H      1.275861079594   -2.624642384992    0.035083470264
H     -0.912769133618   -2.136444457232   -0.855309418164
H      2.958190923494   -0.855434483050    0.431748275709
H      2.399849633195    1.490303478536    0.236691519656
H      1.413839626688    1.104509587813   -1.173990379086
H      0.415729017889    0.798680697090    1.723144021079
H      0.105348711443    2.210246651667    0.700324915950
H     -0.249981973916   -0.721212904320   -1.706251232222
H     -1.112697666651   -1.102035095945    1.591205513158
H     -2.447970560518    0.804663726598   -1.291822642858
N     -1.658140150740    1.225471098855   -0.826532259495
H     -1.142532360129    1.876279209391   -1.412987123668
O     -0.896491415798    0.158849661412   -3.560603131518
H     -1.753579864902   -0.296248212585   -3.646339190061
H     -1.013053210206    1.037788014516   -3.981357298961
O      4.718384900539    0.335015112351   -1.341354787899
H      3.949439347028    0.092933148941   -1.903770444784
H      5.448531868941   -0.183862105192   -1.709662495082
O      4.149442503330    3.477186434733   -3.145725717889
H      4.473791086460    3.338694196470   -2.218798712496
H      4.300551780749    4.399504130725   -3.351252162714
O      1.726483298297    2.239089242613   -3.171916119255
H      1.578750452863    2.083761010288   -4.133677429688
H      2.565249101113    2.734849877870   -3.114709368233
O      4.747553099467    2.959829233260   -0.616994885736
H      4.659451851516    2.004817721048   -0.801164124711
H      3.927977934849    3.266111231905   -0.186367110738
O      0.639845592229    1.700969436502    4.469070756889
H      0.018900994084    2.345404623886    4.072719315789
H      1.477789013760    1.851371528619    4.006045883752
O      4.149230466095    1.306258531781   -5.020419961089
H      3.898649331150    0.582403539232   -4.424541703228
```

28223	H	4.344889234586	2.059890578105	-4.446212433370
28224	O	0.292930336445	-5.823231484367	-0.257792850812
28225	H	-0.058797754116	-5.768049555399	-1.175735219099
28226	H	0.415530964570	-6.754562575151	-0.069638595512
28227	O	-4.807703364759	2.932008709853	2.101599144823
28228	H	-4.509757047607	3.223802830298	2.976987062057
28229	H	-4.321233990317	3.456313704947	1.438286349456
28230	O	0.220032068219	-2.457569001661	3.022826282202
28231	H	-0.262169346808	-2.034119586766	3.749531596737
28232	H	0.940247793597	-1.824564254037	2.824104228150
28233	O	-0.371642723040	3.485277457985	-2.112178380313
28234	H	0.457824361259	3.064721725593	-2.427468315687
28235	H	-0.159282297891	3.947020001632	-1.292886234946
28236	O	-0.571932609866	-0.595161193799	4.929784856580
28237	H	-0.402717413766	-0.721298124803	5.864163201068
28238	H	-0.101745333371	0.247181446497	4.687235793288
28239	O	-3.162634228891	3.799272914944	-2.409116834560
28240	H	-2.202258428598	3.757297942332	-2.508713816841
28241	H	-3.510591513852	2.947696127339	-2.722854509139
28242	O	-2.579742906792	1.384397391645	2.188393726590
28243	H	-3.447619863508	1.802457519641	2.029125406130
28244	H	-2.689761338433	0.924378246402	3.041367943377
28245	O	4.082085150685	-2.539536934921	2.177828500366
28246	H	4.746365519074	-1.945467861208	1.769542502371
28247	H	3.741538200294	-3.094962742336	1.464465567017
28248	O	-4.369236644779	1.363017949646	-3.010953057320
28249	H	-4.598349591174	0.924788677549	-2.154374707458
28250	H	-5.200598635147	1.644309578589	-3.393988423357
28251	O	-0.374674805944	4.412450773337	0.741563464983
28252	H	-0.408448848308	4.247348120086	1.696640777031
28253	H	-1.314719217400	4.368047367736	0.482932063840
28254	O	2.337706924939	-0.784475297477	3.072764314303
28255	H	2.491462670823	-0.673424598522	4.012956822660
28256	H	3.021663848091	-1.421531367208	2.743124161967
28257	O	0.547918275302	-0.969940969392	-5.426997536934
28258	H	0.014585140185	-0.568645477360	-4.683758243352
28259	H	-0.080788446045	-1.224794404137	-6.102454324303
28260	O	-1.125658770579	3.345933689380	3.220105951051
28261	H	-1.589073344146	2.627879087942	2.722622967866
28262	H	-1.788874352741	3.662522450649	3.842322822757
28263	O	-4.999131596778	-2.308640164188	-2.248103695247
28264	H	-4.493370743119	-2.917534879044	-1.696994657928
28265	H	-5.148605360322	-1.514511304821	-1.698566055933
28266	O	-2.981971993529	-3.442863347256	-0.619322352829
28267	H	-3.027956542837	-2.546710982135	-0.213295864940
28268	H	-2.404632928267	-3.927894001824	-0.004248753917
28269	O	6.140044790784	-1.976701053231	-1.661975708827
28270	H	6.630718340154	-2.412885255667	-2.358164336030
28271	H	5.261338718850	-2.410601661336	-1.648063532233
28272	O	2.598017340757	-4.504814974416	0.548467798213
28273	H	1.858061377389	-4.963578115511	0.121427389963
28274	H	2.450929055486	-4.652636537385	1.505266391759
28275	O	-3.147204999405	0.265404436735	4.589862991647
28276	H	-2.277061023447	-0.136557065886	4.769599554669
28277	H	-3.662989892926	-0.390953104580	4.074513629256
28278	O	2.882698329518	1.940785426773	2.789896693024

28279	H	2.690323684865	1.000976974276	2.641855208593
28280	H	3.851105592009	2.019504653281	2.666153191765
28281	O	-1.260018820027	-4.210826028921	1.374088364245
28282	H	-0.721259220285	-3.595373289196	1.896721184145
28283	H	-0.640199738942	-4.814813540973	0.932937078048
28284	O	2.835907692222	-0.258336818528	-3.104320518494
28285	H	2.301333624654	-1.066391079248	-3.069928294710
28286	H	2.227415609771	0.501795931004	-3.073672919671
28287	O	2.482418999852	3.793174882680	0.719477355983
28288	H	1.597993094311	4.174046179218	0.677360196314
28289	H	2.530708399889	3.303297839202	1.557735198840
28290	O	-3.614846301818	3.005792201942	4.645289436232
28291	H	-4.114494802937	3.237637343296	5.427965996888
28292	H	-3.467663427071	2.041662976817	4.692819586146
28293	O	-1.575648684332	-3.183651172709	-2.954009726318
28294	H	-2.133820169879	-3.283892818559	-2.153064003788
28295	H	-2.061443810379	-2.575890236278	-3.524748799698
28296	O	-6.092970292805	0.505757512152	1.492746596573
28297	H	-5.618337041097	-0.123815239772	2.059827615897
28298	H	-5.802468369271	1.391179352987	1.756649947275
28299	O	1.573029514867	1.586417708729	-5.753461846983
28300	H	1.295646171712	0.651337363546	-5.762160073134
28301	H	2.553736088937	1.553094582727	-5.755582800918
28302	O	-4.862893790156	0.005250572136	-0.799471437060
28303	H	-4.050712261917	-0.318187497721	-0.346354234344
28304	H	-5.468179676106	0.237216931011	-0.057426179679
28305	O	5.456857563767	1.884604644119	1.968788405050
28306	H	5.674882883391	0.973309158155	1.727034741470
28307	H	5.459650542432	2.397561787540	1.146876909158
28308	O	-3.307853246233	-1.050762428208	-3.892075154931
28309	H	-3.946721244531	-1.601662870068	-3.382434720365
28310	H	-3.692370266871	-0.160122841243	-3.843695342045
28311	O	3.711633557959	-3.196292944131	-1.681231736026
28312	H	3.381047688135	-3.687169328942	-0.915741212608
28313	H	2.952791044603	-3.056269248330	-2.260752864809
28314	O	-0.807156826745	-5.765239174454	-2.700145382468
28315	H	-0.995193016826	-4.834993768910	-2.929913891797
28316	H	-1.635815829115	-6.236172767399	-2.800168044684
28317	O	-3.867306301228	-3.972007808579	2.395931943158
28318	H	-4.334897793912	-4.182157655446	1.584416377753
28319	H	-2.925867445766	-4.104941419226	2.201763565888
28320	O	-4.299501607859	-1.291197921925	2.727891162335
28321	H	-4.302098227041	-2.260408491417	2.822063150010
28322	H	-3.745994373891	-1.140663298678	1.933976583727
28323	O	-0.966057528368	2.509744957139	-4.854857871070
28324	H	-0.786656875460	3.193215251014	-4.201602040320
28325	H	-0.167642917659	2.410122993431	-5.391121430393
28326	O	-2.969251555841	3.656754315739	0.230595644741
28327	H	-3.182417709824	3.845685784057	-0.711245082026
28328	H	-2.632910334941	2.747814408297	0.227835223702
28329	O	1.304641970399	-2.584567525403	-3.302986246239
28330	H	0.411122190146	-2.816414940193	-3.014886295980
28331	H	1.198633189409	-2.253484236059	-4.209618724203
28332	O	5.661216392992	-0.769620407426	0.906888729442
28333	H	5.152115281598	-0.370318876123	0.167586798192
28334	H	6.349541245860	-1.272791293330	0.461093681597

```
O      2.103577233317   -4.470431048367    3.218103080213
H      2.892404224107   -3.962390182326    3.429011865541
H      1.357806976141   -3.842943117340    3.233880326682

Cope TS Water45-9
155
ANGSTROM
C     -0.295711544840   -1.203111020574   -0.763870534567
C      1.018090255319   -1.543586693530   -0.171939891778
C      1.934779574056   -0.601802126122    0.098161975557
C      1.566425353707    0.869211308024   -0.030063457264
C      0.228092402725    1.095163729768    0.806006278526
C     -1.013094858499    0.463981950020    0.171441450447
C     -1.668144487186   -0.651769042095    0.790675944783
O     -2.858344192114   -1.069609777119    0.549452076293
H      1.194806087221   -2.589759125204    0.058147310417
H     -0.963624359566   -2.044995962799   -0.926609222958
H      2.902528625457   -0.846926178144    0.516294484532
H      2.351935439988    1.518370619503    0.362468367429
H      1.375219554186    1.151504719688   -1.072059755198
H      0.379262867579    0.668955931845    1.800199881283
H      0.054671008827    2.166874212436    0.912849740300
H     -0.239528543647   -0.595172189671   -1.664335623445
H     -1.116548347366   -1.155648188819    1.593598188512
H     -2.432689315840    0.885168750059   -1.265525533103
N     -1.692185006818    1.289678295650   -0.709849087185
H     -1.118761116996    1.939128686413   -1.247242459005
O     -4.847884206770    1.137311351311    3.047259071922
H     -5.019565865464    1.065032136286    2.074192039519
H     -4.799719741483    2.079751818806    3.240623474230
O     -3.449325873009   -1.679659729277   -2.099256916847
H     -3.420294817863   -0.834128344012   -2.574741473003
H     -3.358551761029   -1.470155164133   -1.157032713147
O     -1.078889490318    2.886389606393    3.595802981628
H     -0.848859118245    3.341992120465    2.757153476016
H     -1.461235404943    2.013546929789    3.349342573384
O      1.811603711473    2.776340283305   -3.904033771010
H      1.110011635839    2.228345334432   -3.483970380782
H      1.571089905932    2.770940050179   -4.839166494780
O      5.022869001467    1.378293518980    1.297509670187
H      5.024172480051    0.420415144973    1.448832596809
H      4.300001749436    1.753014127956    1.828907449192
O      3.036040180390    2.635566010888    2.765165111274
H      3.494164771793    3.196884154334    3.392487272906
H      2.349068527282    2.151524537490    3.287759967563
O      1.808351821282   -4.075037656866   -2.134329920991
H      0.873353786040   -4.139534039723   -1.899444334467
H      2.010033922028   -3.137355421038   -2.270063027431
O      2.713598061781    0.018757136072   -5.907209508521
H      3.222752835861    0.288099530066   -5.130016130158
H      2.557266481817   -0.928782311433   -5.798091749959
O      2.643556903035    3.851545718647   -1.512620253094
H      3.397502671493    3.259081803165   -1.339619951669
H      2.486695646216    3.781719162367   -2.465453301527
O     -3.486378673164    3.164359662507    0.481306379962
H     -2.592387574854    2.817905337731    0.336059106956
```

28391	H	-3.557069340008	3.366595260192	1.436375122848
28392	O	-0.150822833974	3.580014899971	-1.382223433732
28393	H	-0.348535174052	3.822560174080	-0.459423923380
28394	H	0.809899834140	3.663507154927	-1.472032051540
28395	O	0.696957605254	-5.587663049042	1.531300767498
28396	H	0.282916056714	-4.880301456320	2.068286542685
28397	H	0.689301761764	-6.378696460471	2.069872306811
28398	O	1.782461141561	-4.001145850783	4.505471176347
28399	H	1.747149093106	-3.290441491332	5.160364138484
28400	H	0.880465687960	-4.091707835056	4.174730569533
28401	O	1.162252182809	1.417258952602	4.206299163477
28402	H	0.927680741930	0.572066651457	3.794576995789
28403	H	0.393955310717	2.012464833410	4.093864413052
28404	O	-0.657090059429	-4.618939000994	-0.793726565619
28405	H	-0.129172823432	-5.190328367891	-0.221420439553
28406	H	-1.398902672459	-4.335481178609	-0.230220715419
28407	O	-4.448892543366	-1.645856997468	2.747298075460
28408	H	-4.895988657045	-0.836171505433	3.022006742867
28409	H	-3.938066813866	-1.405092291683	1.948989962833
28410	O	3.034565326205	-1.604187467159	-2.594351846103
28411	H	3.780569531293	-1.907392474896	-2.039769489041
28412	H	3.227815321762	-0.673488852649	-2.826386525714
28413	O	-0.048708782902	1.041059822521	-3.127905593075
28414	H	0.098637608187	0.182752332191	-3.576474162712
28415	H	-0.806972190566	1.434613275386	-3.568620893375
28416	O	-4.925328340074	-4.238784254916	2.014873164159
28417	H	-4.939887129983	-3.347375695190	2.406672128583
28418	H	-5.673935084313	-4.290555589878	1.419221792545
28419	O	2.949563977762	-2.809354098091	2.367891975840
28420	H	2.941332827811	-3.403294084607	1.586338556763
28421	H	2.714837807674	-3.364495283992	3.139701864170
28422	O	-3.606063725780	3.772517727415	3.135707655387
28423	H	-2.714474436986	3.526276653013	3.469804106239
28424	H	-3.672399013783	4.725003680372	3.218054480707
28425	O	4.857920221145	-2.559363074899	-0.812104291750
28426	H	5.014363172569	-2.014327423920	-0.025968079860
28427	H	4.418778697317	-3.357734714149	-0.497559183870
28428	O	-1.693647579760	0.465528764774	-5.450068847940
28429	H	-1.239723009788	-0.362632375611	-5.203836103485
28430	H	-0.981226505780	1.033567720310	-5.792095911951
28431	O	1.373426832124	-1.492083970148	5.766501074917
28432	H	0.505072190225	-1.137408710196	6.077611068629
28433	H	2.024425780111	-0.829077717509	6.002720394352
28434	O	-1.562703055955	-3.332562230925	-3.183470601547
28435	H	-1.300862380767	-3.939997457619	-2.481531450668
28436	H	-2.337842585525	-2.842255680218	-2.850228496754
28437	O	-3.339928530350	0.820201064360	-3.386036062393
28438	H	-2.748465685322	0.708104043175	-4.170778449351
28439	H	-4.230529652933	0.842388365905	-3.741498094945
28440	O	-0.588626698785	-3.584044978412	2.907311950660
28441	H	-0.117261270977	-2.727828146646	2.811681680091
28442	H	-1.286068855787	-3.618768829114	2.211319433440
28443	O	-2.293631629119	-1.890482943980	4.372577133108
28444	H	-1.750536473711	-2.623971156161	4.043178104854
28445	H	-3.164879891950	-1.973441446135	3.938918390002
28446	O	-3.719614633976	3.473281259922	-2.361644032222

```
H     -3.816824484578    3.393570103714   -1.405514134432
H     -3.497609188431    2.591418166227   -2.689854316136
O      0.673340780140    1.699076631918   -6.240070790879
H      0.711356212221    1.944451884276   -7.164429159364
H      1.381138009618    1.019212567212   -6.113611166994
O     -4.873917504101    0.830135717613    0.421273006321
H     -4.170266315597    0.163065630818    0.344982794624
H     -4.453211934589    1.694980053678    0.260423757967
O      3.670989421569    0.903805921510   -3.366893149572
H      3.012620087076    1.606588382772   -3.557770676094
H      4.192129495562    1.246340021104   -2.624402884175
O      4.824029325967    2.192920310670   -1.199332033776
H      5.660577562349    2.627173868900   -1.368962779442
H      4.886884355331    1.836217695445   -0.277522720640
O      2.295379892659   -2.575899074652   -4.937272269146
H      2.250056971883   -3.531315756666   -4.872890135106
H      2.678666083653   -2.266488725740   -4.079935313831
O     -2.475018855936    5.862382490083    0.372739633508
H     -3.218966729988    5.249372691686    0.351090108106
H     -2.211102894769    5.948452627196   -0.575998922971
O     -0.986462848564   -0.382048103968    6.242063249348
H     -1.574115669280   -0.950903159538    5.715336820080
H     -1.029257471962    0.495409307134    5.853328245565
O     -0.646371829853    4.180562532699    1.261834723055
H     -1.261058271255    4.927075098830    1.051473485422
H      0.264759623237    4.546455719791    1.307641936651
O      1.962308181031    4.647472062228    1.116843801542
H      2.372811560956    3.926747633644    1.621201917944
H      2.241793873579    4.529287953454    0.197538826416
O     -2.224408156649    0.566926019264    3.109568414185
H     -2.119885533656   -0.257309324255    3.613718077837
H     -3.185436958153    0.770812299362    3.130790554559
O      0.841304250043   -1.274153892239    3.127770310741
H      1.635682402005   -1.629769107138    2.684868369256
H      1.010832071313   -1.409376193492    4.084058605580
O     -1.876163414242    5.536767595425   -2.206975084908
H     -2.625966998013    4.973158239736   -2.459184689125
H     -1.136304231275    4.913183932259   -2.096885738222
O      2.923753141538   -4.506770100712    0.270644696268
H      2.595145143836   -4.395011406227   -0.652039273805
H      2.270736103418   -5.086784992622    0.690798250377
O     -0.086850769055   -1.395513196304   -4.306147742673
H      0.728188640147   -1.810249083668   -4.641447541225
H     -0.611025358358   -2.121603526332   -3.906790020268
O      5.091391318810   -1.373688246289    1.742739509728
H      5.859524502333   -1.710970864663    2.204283097831
H      4.312666180996   -1.878152402140    2.073374497037
O     -2.483264112600   -3.697374430733    1.022275834974
H     -3.310365896374   -4.100141944135    1.351643619517
H     -2.723949748162   -2.778952156917    0.783286453023

Cope TS Water45-10
155
ANGSTROM
C     -0.232390493687   -1.209373038263   -0.740470349861
C      1.089257174832   -1.513720822416   -0.144247551846
```

28503	C	1.963416877399	-0.541636048049	0.154366867337
28504	C	1.555959969369	0.915319348604	-0.007197241935
28505	C	0.201206741652	1.151231457104	0.780988751733
28506	C	-0.999527520794	0.406062814571	0.184938456433
28507	C	-1.658769712545	-0.689403028760	0.861103629677
28508	O	-2.828109622571	-1.082755565771	0.586930605134
28509	H	1.298199516768	-2.559368015975	0.068014146171
28510	H	-0.882133423673	-2.073235333447	-0.855683989349
28511	H	2.932904136049	-0.730836124864	0.596858446374
28512	H	2.319309791316	1.584363273955	0.388647682632
28513	H	1.385078614437	1.173939626677	-1.059548707498
28514	H	0.343088803566	0.863347801508	1.826471388047
28515	H	-0.027811785301	2.219418917022	0.746443049243
28516	H	-0.206495268110	-0.637101656690	-1.666540789994
28517	H	-1.121216357969	-1.201254806827	1.670321509327
28518	H	-2.431883733251	0.678663893666	-1.265093587549
28519	N	-1.750778983229	1.163411358548	-0.687161654030
28520	H	-1.280928745828	1.895493788366	-1.201970013063
28521	O	-5.136456623052	0.580152833624	2.592284595777
28522	H	-5.150324134186	-0.397009392703	2.602974153804
28523	H	-5.008869034764	0.790825371002	1.643059089465
28524	O	-3.256687373916	-0.005704073747	-2.708936642989
28525	H	-2.911633001068	-0.829363291700	-3.106481551370
28526	H	-3.067042969057	0.721016934816	-3.327746387273
28527	O	1.404499297641	-4.837509693560	-1.573925577969
28528	H	2.053833144155	-4.180897419800	-1.849681143445
28529	H	1.677802855575	-5.115269473180	-0.676485904542
28530	O	-3.553854441500	0.518158568791	4.710470559605
28531	H	-4.075325199120	0.626287658558	3.878182223956
28532	H	-4.063412649704	-0.075482829611	5.262348964823
28533	O	3.027076392320	2.994000128182	2.439391354714
28534	H	3.513967530873	3.461974875250	3.119456749447
28535	H	2.075097624940	3.261178008723	2.556016042313
28536	O	1.988141957846	-5.164489946686	1.058533186217
28537	H	2.660028106810	-4.560378112406	1.412115458145
28538	H	1.165394884628	-4.952587913769	1.521184252850
28539	O	-2.970830475628	2.401787255866	-3.960823842990
28540	H	-2.089119666066	2.763283168196	-4.125010672857
28541	H	-3.256543179663	2.708809418541	-3.082453165517
28542	O	1.003415447303	-1.670971591605	3.010395252863
28543	H	1.596143649001	-2.020652079402	3.711505889291
28544	H	0.237936455249	-1.290006185241	3.482879540764
28545	O	-2.316230372712	-3.050836491169	3.962034989495
28546	H	-2.297522248315	-3.561688599989	4.771860387926
28547	H	-1.863811993048	-2.205966835853	4.157563522702
28548	O	3.556966418934	-2.971671618400	1.853376869098
28549	H	4.037532951806	-2.666095602716	1.070123674838
28550	H	2.736463085222	-2.464579861636	1.882696499106
28551	O	-4.720585746831	0.688529879982	-0.065895721344
28552	H	-5.373133171307	0.075909220209	-0.444577438034
28553	H	-3.927198442489	0.125067961775	0.074537809718
28554	O	-0.136287374377	0.754483544385	-3.215101104115
28555	H	-0.341656787845	1.685690608829	-3.058008775982
28556	H	-0.478871749440	0.548941672969	-4.111625164394
28557	O	4.470533594319	0.571428345486	-1.628910640927
28558	H	4.301917677074	1.383695498310	-1.132490629090

28559	H	3.805788818593	0.534872005335	-2.330777250787
28560	O	-4.065633210186	2.892273247860	-1.450490453119
28561	H	-4.917996943986	3.296272610914	-1.619907668489
28562	H	-4.271864347075	2.012769689055	-1.052898617514
28563	O	4.757458343795	-1.878834243817	-0.538340425726
28564	H	5.610190559520	-2.212625131171	-0.820207136443
28565	H	4.705834346993	-0.948503618781	-0.858140471149
28566	O	-0.237607302144	4.720106690539	0.420329700244
28567	H	0.106520652812	4.576525006733	1.314667651861
28568	H	0.514727776618	4.630633476667	-0.179345957659
28569	O	-1.082498070025	-0.659983982986	4.416173807610
28570	H	-1.885810068632	-0.120520331794	4.317267720407
28571	H	-0.474433502299	-0.098978885735	4.951799124830
28572	O	-0.433686561250	2.947160342730	-5.244556594021
28573	H	-0.553246513295	2.028711379654	-5.563597551361
28574	H	-0.618886337833	3.519396262687	-5.988580211030
28575	O	0.309823265129	-2.995450022751	-3.500430914054
28576	H	0.268377300056	-3.941414457683	-3.327425061290
28577	H	1.051612650353	-2.685024320424	-2.952128842845
28578	O	-2.418777098114	-2.374061673176	-3.606320714682
28579	H	-2.838020483067	-2.772034265291	-2.814585641803
28580	H	-1.468292694507	-2.550969179550	-3.539712899978
28581	O	-0.241338723755	3.671956721748	5.571651346162
28582	H	-1.197375728539	3.520279158986	5.412131036736
28583	H	-0.176629403504	4.034273317689	6.455457357441
28584	O	1.831950444444	4.028195590411	-1.516031911398
28585	H	2.030295038215	3.801483460527	-2.450860333638
28586	H	2.571475179102	3.657743818224	-1.011547169786
28587	O	0.549595293228	3.735263160591	2.931370059271
28588	H	0.465975931749	4.014890053374	3.853220563790
28589	H	-0.168335466172	3.048794935642	2.844880064956
28590	O	-0.742781640544	0.329152937304	-5.775268344859
28591	H	-0.040903002123	-0.319602514164	-5.931706627106
28592	H	-1.607093172799	-0.148332511742	-5.921502635027
28593	O	-1.527886331803	2.121193336247	2.863386906417
28594	H	-1.983562045060	2.410103179563	3.671462032337
28595	H	-2.038881360279	2.495998114285	2.121887029072
28596	O	-5.938098613561	0.097627109812	-2.804069116847
28597	H	-6.327885266195	-0.669482431092	-2.378950111383
28598	H	-4.971157728597	0.020216380686	-2.656234656086
28599	O	-5.867830296241	-1.805304267505	-0.531844317533
28600	H	-5.173165670487	-2.352729836799	-0.927700840396
28601	H	-5.871757725067	-2.038407863605	0.401479340366
28602	O	-0.322383039686	-3.904279887434	2.139801067082
28603	H	-1.125132009882	-3.824945509570	2.676371427697
28604	H	0.195325549721	-3.100754956686	2.344024383277
28605	O	3.090612151172	-2.269341303653	4.581122016408
28606	H	3.348198062375	-1.341138097652	4.618052963173
28607	H	3.596389559994	-2.674075661955	3.863690754704
28608	O	-2.967019401559	-1.004351287539	-5.988192162249
28609	H	-2.935195886482	-1.618742678450	-5.240513918822
28610	H	-3.730214304163	-0.407289499521	-5.828287492982
28611	O	-2.740620689639	3.574428862120	0.903227674837
28612	H	-3.128687869300	3.278576209852	0.061244291534
28613	H	-1.944043472722	4.081222538828	0.677763053794
28614	O	4.075319073647	3.044353860878	-0.105746445177

```
H      4.832494138962    3.629009317756   -0.159381023416
H      3.772522627981    3.041758949792    0.823479984658
O     -1.148398575196   -4.374564644595   -0.493518327586
H     -0.334948504026   -4.632279384622   -0.954081947403
H     -0.903207530120   -4.282156912057    0.441382137745
O      2.089495109828    3.017829963212   -3.997606862027
H      1.213516259103    3.060099381679   -4.418269108595
H      2.323152992384    2.079673855330   -3.949263506624
O      2.385927698177    0.164457389340   -3.619727752408
H      2.294795243537   -0.287070498740   -4.470640669315
H      1.445647016316    0.351075590786   -3.355652363127
O     -4.562086725703   -2.083165947674    2.373670118086
H     -4.005972162623   -2.549145151030    3.009864261406
H     -3.932114124292   -1.804943954653    1.677022166725
O     -2.845979590890    3.106704098645    5.105782016155
H     -3.507045622680    3.448350776348    4.462912416649
H     -3.119663651912    2.188641668340    5.270880259558
O      2.655475725234    0.502006470425    3.656569719335
H      2.097868906700   -0.103915641314    3.141475610324
H      2.820367282167    1.281161954613    3.103449346360
O     -0.724658041509    3.360164899096   -2.263165248190
H      0.171366584285    3.612419438446   -1.979431102345
H     -1.305459201863    4.062765982709   -1.967733781539
O      0.676169742016    0.991034157989    5.545129444923
H      1.456106839004    0.883967371840    4.971979336798
H      0.409184186279    1.921929680765    5.495536753037
O     -4.881895761692    0.780695467354   -5.306840440154
H     -4.318323623658    1.496947898263   -4.984125417594
H     -5.440063449550    0.527102050777   -4.553244846090
O      2.786919310445   -2.366788333801   -2.466495424272
H      2.754553766026   -1.470078707485   -2.842294050725
H      3.419807381090   -2.311499101456   -1.732871375909
O     -4.513014175205    3.455643544247    3.045329904251
H     -3.972642174851    3.622422943396    2.255508221402
H     -4.923329381030    2.592936897277    2.912793009734
O     -3.416520728141   -3.066954545245   -1.195878273765
H     -3.208664112482   -2.310735351398   -0.617370155250
H     -2.763680992497   -3.745823304096   -0.950357487459
O      1.300062815328   -1.550539699239   -5.596744251420
H      1.572688149003   -2.157254058926   -6.284933797024
H      0.876578751618   -2.093240269343   -4.899383793629

Cope TS Water45-11
155
ANGSTROM
C     -0.255243056136   -1.159531774980   -0.827313813820
C      0.983649572806   -1.571184813143   -0.127767959103
C      1.897358230139   -0.668207760720    0.256180829025
C      1.622045708072    0.815875379366    0.045428429036
C      0.217254099269    1.208557311357    0.683113394474
C     -1.020760123508    0.504291200384    0.093078720966
C     -1.626414669439   -0.613647700327    0.776903083359
O     -2.777949224897   -1.115673528233    0.579816467460
H      1.098368973863   -2.623957682821    0.110522607630
H     -0.955809228856   -1.955639908008   -1.054308327373
H      2.796071531071   -0.950439880801    0.790935484450
```

28671	H	2.396136365472	1.436937896968	0.504541270263
28672	H	1.580260180995	1.057754425364	-1.023730936046
28673	H	0.275615967000	1.011609960501	1.757035675514
28674	H	0.080844019975	2.284957378867	0.544747157000
28675	H	-0.105145854257	-0.544754841831	-1.714968133227
28676	H	-1.034191906633	-1.059115443014	1.589505446260
28677	H	-2.612267076614	0.925631220375	-1.177717167346
28678	N	-1.749834318396	1.271788387330	-0.773590063324
28679	H	-1.279126473083	1.979378654663	-1.312236027961
28680	O	-5.468037019766	-2.865685245413	-1.595857126272
28681	H	-4.789430226157	-3.393553227955	-1.152122683999
28682	H	-5.639977767916	-2.111292745482	-1.001926986768
28683	O	3.400669251711	-1.068415417988	3.556546779504
28684	H	2.456213582682	-0.859653751370	3.756703847097
28685	H	3.857209793952	-1.074273431803	4.399631070115
28686	O	-0.000063131028	-2.752780474363	2.649107085652
28687	H	0.869035444363	-3.197568462831	2.805758290625
28688	H	-0.647102451758	-3.119696325513	3.298131544678
28689	O	-2.781970244292	1.256361884341	2.404370990050
28690	H	-2.768654722421	0.882212047947	3.300925688608
28691	H	-2.353962541475	2.120891394627	2.424352742861
28692	O	-2.890855685840	2.115919443123	-4.239676926402
28693	H	-2.657156024699	2.978349924351	-3.866667155653
28694	H	-2.082702972254	1.566457320997	-4.182293083348
28695	O	3.429887516332	-0.953340548631	-2.693883145802
28696	H	3.035396791340	-1.795557463444	-2.370040323615
28697	H	4.056983787559	-1.216866630403	-3.371924294445
28698	O	5.232192169994	-2.200485497762	1.506451529659
28699	H	4.734245889574	-1.660903716491	2.129663694022
28700	H	5.396067921502	-1.643942709377	0.710851731939
28701	O	-3.066186616985	-3.612039916640	-0.433917778433
28702	H	-3.006094938402	-2.705314142102	-0.071689010241
28703	H	-2.388823592350	-4.114964823793	0.050710817604
28704	O	3.740011221838	2.017356319469	-2.123413476412
28705	H	3.577974519329	1.086815527384	-2.321326736177
28706	H	4.372674826473	2.027753305906	-1.387656476457
28707	O	-1.781067261345	-3.293336965719	-2.792375243743
28708	H	-2.374242221818	-3.569657009983	-2.064559816363
28709	H	-2.125780194071	-3.648489416683	-3.641243590800
28710	O	-1.326845343501	3.697754841862	2.156307728358
28711	H	-0.448213694863	4.087138738245	2.004026548609
28712	H	-1.815708548041	3.862843006630	1.328113627731
28713	O	4.213587578388	1.408201818696	2.483713558767
28714	H	3.569275129457	2.032050404985	2.882179048225
28715	H	3.934248016577	0.525807283184	2.765687663412
28716	O	0.133660702267	2.990454321813	-2.538440497308
28717	H	-0.542533411078	3.641801440069	-2.775802022863
28718	H	0.848895235891	3.049634064101	-3.231623104789
28719	O	-4.073367598247	-0.036581030142	-5.673546474967
28720	H	-3.870479398673	0.853430824168	-5.358434650513
28721	H	-4.249772217582	-0.568347029038	-4.880868269310
28722	O	-5.310694795825	-0.550329004579	-0.110414713131
28723	H	-5.277451443602	0.041505641408	-0.873066301967
28724	H	-4.370511298953	-0.692413170869	0.126897738418
28725	O	-0.201112130433	1.866411634192	3.978612202761
28726	H	-0.571685186263	2.467586425159	3.312181254400

28727	H	-0.765773314761	1.985818750281	4.766861192509
28728	O	-3.960088091940	-1.535899359192	-3.321355007623
28729	H	-3.085354076959	-1.933201460281	-3.188543547736
28730	H	-4.596671215895	-2.085703870226	-2.803710289779
28731	O	-4.183746696798	0.881053786990	-2.243175438083
28732	H	-3.844696897129	1.439737655049	-2.972927422326
28733	H	-4.125346002369	-0.028873480045	-2.611450894798
28734	O	1.031733203765	-1.627969498406	-6.413822369002
28735	H	0.961520924209	-2.142581057805	-5.590259710912
28736	H	0.113335515373	-1.419139031329	-6.631478237177
28737	O	0.322611058048	-4.711488369735	-1.639665174083
28738	H	-0.388289853932	-4.285655824838	-2.148184547747
28739	H	-0.007327861404	-4.765468020806	-0.730892678360
28740	O	-3.052805527292	-0.188650663194	4.732350424890
28741	H	-2.340056214712	-0.689039177883	5.170312115537
28742	H	-3.489262904872	-0.822595987920	4.134219566761
28743	O	-2.693590458452	4.390633182249	-0.155837169999
28744	H	-3.495511421415	3.780708577399	-0.255636348648
28745	H	-3.057416589720	5.232191094935	0.123996078708
28746	O	3.729509551420	-4.108268723724	0.537389115571
28747	H	4.250653094910	-4.907306748938	0.453990958841
28748	H	4.349573721895	-3.403418712460	0.869147473998
28749	O	-1.966005990927	-3.537726763635	4.232547109753
28750	H	-1.778285688618	-3.008989079353	5.018980568532
28751	H	-2.713685497907	-3.110696755964	3.781349195545
28752	O	-0.643415992378	0.656582218848	-3.716247853257
28753	H	-0.395387038244	1.419205689277	-3.162462508965
28754	H	0.190309255759	0.446814264140	-4.188909919876
28755	O	-0.968123673039	-1.502096312263	5.923107009599
28756	H	-0.223548784404	-1.213289298896	5.346413678030
28757	H	-0.766785741373	-1.201426271550	6.809272741665
28758	O	-5.360525243365	1.565776875932	1.659932973109
28759	H	-5.583686517018	0.747884554598	1.185912068410
28760	H	-4.438652408730	1.440589533857	1.965215199322
28761	O	-0.885581487586	-4.587786843165	0.898310408055
28762	H	-0.960998200458	-5.328619596052	1.500848238771
28763	H	-0.564002616860	-3.827046962420	1.436822808130
28764	O	0.712765189494	-2.281466839848	-3.795739877421
28765	H	-0.159596672581	-2.557324032285	-3.489951738983
28766	H	1.355106305234	-2.814841238136	-3.320111122941
28767	O	2.678794234509	-3.432017892630	-1.939329850218
28768	H	1.858818480019	-3.963235845194	-1.847510425396
28769	H	3.146577766355	-3.598480431498	-1.103649174327
28770	O	-2.651155869381	-3.617059507234	-5.265710890566
28771	H	-2.205307246135	-2.791367324876	-5.561327244570
28772	H	-3.585027348521	-3.483956973204	-5.437531966644
28773	O	-2.168228463458	4.440478187013	-2.806133329899
28774	H	-2.346471822315	4.395686074556	-1.837816451793
28775	H	-2.516175723267	5.275732056859	-3.118230607374
28776	O	-2.171928815564	2.163070265686	5.814877571758
28777	H	-2.758633489943	2.906720011916	5.669848122634
28778	H	-2.664860523627	1.366112644107	5.558191771002
28779	O	5.297571738105	1.895986117651	0.143119007288
28780	H	4.855615351445	1.734845463948	1.014862576223
28781	H	6.094352813383	2.389354669220	0.337667087606
28782	O	-4.811577409760	2.996286871839	-0.499909834149

```
H     -5.094061546296    2.555035424616    0.339733522946
H     -4.653792862002    2.282259955920   -1.133515571282
O      2.013102557697    3.974827950516   -0.851933059697
H      2.741776046357    3.415770371798   -1.151814914544
H      1.245304359772    3.674484621590   -1.375813518669
O      2.305322700079    2.968801359995    3.525242380626
H      1.992830724776    3.587098197074    2.843925551438
H      1.506700898323    2.519561113555    3.844416292719
O      1.225870062353    4.610038282800    1.574999252526
H      1.547848367082    4.331066643943    0.680692032931
H      1.476337651629    5.529553062843    1.667611509933
O      5.412707566420   -0.710406508383   -0.694106496351
H      5.446837385830    0.223597740709   -0.424797888664
H      4.661964844321   -0.794961461710   -1.302418268145
O      2.437840976159   -3.705635401758    2.927390279744
H      2.915336804751   -2.893172734721    3.148594408960
H      2.777527462113   -3.972570079958    2.053576887236
O     -3.863849069239   -2.012995627665    2.853263056531
H     -4.788927817956   -2.135530543567    2.635888681064
H     -3.438323288937   -1.678655873596    2.027120775384
O      0.810497050660   -0.663003302840    4.092296787532
H      0.435440074562    0.223616617039    3.931086006294
H      0.449197667809   -1.292505386978    3.423461285065
O      1.811208607896    0.294660662691   -4.755840832655
H      1.630081081006   -0.339181617905   -5.490040076023
H      2.214298446367   -0.226403818569   -4.046200407290
O     -1.653567803026   -1.162062279378   -5.796427800730
H     -1.313992635957   -0.754481368739   -4.989375073521
H     -2.517433216643   -0.724196506888   -5.960624883336
O      2.134643132906    2.945823274479   -4.217031482790
H      2.870112461934    2.856293806259   -3.594697998187
H      2.042979965109    2.065153636023   -4.622128318987

Cope TS Water45-12
155
ANGSTROM
C     -0.225563686099   -1.210852525891   -0.780267376638
C      1.048927900152   -1.558658706415   -0.120634106255
C      1.943208240041   -0.617409880549    0.220479778152
C      1.632766390110    0.858293883409    0.018866376376
C      0.264215340891    1.208815757454    0.755224951952
C     -0.960991749716    0.511872734676    0.177656950171
C     -1.548393547504   -0.653318286906    0.773625627877
O     -2.744528649092   -1.056322518358    0.550765122078
H      1.204777422750   -2.602565061802    0.131001343939
H     -0.898266331604   -2.038012840252   -0.989565221921
H      2.858007700505   -0.869838983588    0.735543305226
H      2.422056759521    1.495426003082    0.420822251108
H      1.508490480723    1.103596404563   -1.041467809938
H      0.379951615085    0.950546203307    1.811354491419
H      0.113306871339    2.289647824021    0.679526983188
H     -0.138525656238   -0.567920866497   -1.656112124008
H     -0.993510973195   -1.146813376549    1.583566900980
H     -2.493514105460    0.785424341208   -1.159069936259
N     -1.712176875952    1.252324343921   -0.710976309428
H     -1.245009218418    1.940290509876   -1.298113542695
```

28839	O	-2.753832503083	3.172628802525	1.153222198026
28840	H	-2.638103998408	2.679707388634	1.985038714995
28841	H	-2.448649704756	2.557541238669	0.463470191102
28842	O	4.988160634219	2.822432354965	-1.888227041337
28843	H	4.122160750547	2.920189892472	-2.326452170429
28844	H	4.905252288627	3.310001916977	-1.054359790861
28845	O	-0.545817001873	3.086216702629	-4.484931403164
28846	H	-0.485000827739	2.130116678491	-4.315952840864
28847	H	-0.529221704374	3.489752024388	-3.597502663750
28848	O	-5.266734248631	-0.032549802999	0.496887216685
28849	H	-5.230582706143	0.943414336236	0.473625505558
28850	H	-4.342689035145	-0.332662696803	0.406166746941
28851	O	-3.900131674501	-4.005834566414	-4.262516926927
28852	H	-3.472605324088	-3.345195907533	-4.875033772466
28853	H	-4.255083089925	-4.702335563955	-4.814103912547
28854	O	-3.086807330987	3.236519255581	-5.194909873295
28855	H	-3.215854615395	3.877495042712	-5.893241775318
28856	H	-2.113614959446	3.233993604863	-4.989972035511
28857	O	-0.720933441835	0.384601994664	-4.179064368126
28858	H	-1.592210633626	0.418975556378	-4.626303948084
28859	H	-0.630474508790	-0.551065535703	-3.916119117133
28860	O	-4.942279464561	-2.230296700683	-2.513514186941
28861	H	-4.642936431619	-2.933809866023	-3.118643266729
28862	H	-5.738191651837	-1.840185477283	-2.882827254984
28863	O	-1.509165563228	-4.493016297117	2.188053339434
28864	H	-2.118628957075	-4.279151787043	1.460343983209
28865	H	-0.659684787540	-4.703256260433	1.770128690104
28866	O	3.411317683734	-0.975997951717	3.877417639998
28867	H	2.484214173307	-1.034627460035	3.577491389422
28868	H	3.585171343666	-1.805207337243	4.324324267736
28869	O	-3.258801450547	0.477949776452	-5.083750295874
28870	H	-3.352530816765	1.418535889372	-5.302690443281
28871	H	-3.568207286191	0.386848975395	-4.157532630777
28872	O	-1.060744836553	3.707345501123	-1.919736135611
28873	H	-0.819160214194	4.181980643690	-1.108980771358
28874	H	-2.028523032866	3.757959607573	-2.004830880860
28875	O	4.544081535442	-2.541885075572	-1.336675217939
28876	H	4.833828674173	-1.632569578070	-1.505571954337
28877	H	4.473555049961	-2.604927120587	-0.369956117056
28878	O	2.032742409516	2.928558509771	-5.532504621787
28879	H	1.098939969974	3.158758915143	-5.409423153118
28880	H	2.053565847832	1.980705998655	-5.739057455957
28881	O	1.050917541937	3.638558909237	2.740099924305
28882	H	0.628179636561	3.006456030003	3.333253051172
28883	H	1.938954164334	3.288416722174	2.571267333261
28884	O	2.917636728556	1.532946969948	4.804815452981
28885	H	3.220450753725	0.627648852053	4.601209646637
28886	H	3.238287218485	2.067489864852	4.059837314915
28887	O	-2.680285080167	-2.207029190674	-5.725345936809
28888	H	-3.015285004165	-1.300498621369	-5.682313219567
28889	H	-1.860347280090	-2.207033325865	-5.189558600323
28890	O	2.406363457944	2.752730917752	-2.876818416171
28891	H	2.383825685569	2.978220535078	-3.831183794733
28892	H	2.408155127733	1.784030914561	-2.845038540254
28893	O	-5.694854249027	-2.901934650577	0.053650319868
28894	H	-5.753959133915	-2.038251158630	0.475528496194

28895	H	-5.506944806969	-2.708188523484	-0.880783999266
28896	O	0.340204339199	1.394271488928	4.500703577835
28897	H	-0.032450881108	1.481346547058	5.379449060024
28898	H	1.334500237184	1.477844161916	4.632087096834
28899	O	-3.687235380256	3.082441047543	-2.511735659900
28900	H	-3.592155677430	2.120938897947	-2.483589378440
28901	H	-3.665011224693	3.307060418630	-3.455872947434
28902	O	0.712903265517	-1.139613214331	3.352851751657
28903	H	-0.031102163683	-1.710658695944	3.672389570228
28904	H	0.546751893637	-0.275215184361	3.760897771369
28905	O	-3.612194412404	-1.026292494006	3.114090621460
28906	H	-4.568340855582	-1.000858947619	3.170612551205
28907	H	-3.401144813996	-1.160698358560	2.166772105883
28908	O	4.515215999798	-2.509314562541	1.470692892664
28909	H	5.190000309165	-3.107058077682	1.793773524713
28910	H	3.661135648662	-2.818184368285	1.872857676685
28911	O	-2.225468624146	1.397166848977	3.128300229943
28912	H	-2.701111250233	0.561000325658	3.263526006393
28913	H	-1.382368839854	1.330756403288	3.589043506204
28914	O	2.042326708020	4.669765115305	-0.897313981384
28915	H	1.194737897460	4.653871802643	-0.437733628536
28916	H	2.010792748200	4.012922718991	-1.612554537460
28917	O	-0.637694074717	-2.264350513315	-3.955671247868
28918	H	-0.968965220135	-2.899872078111	-3.293815780645
28919	H	0.211478657441	-2.584546450634	-4.301277356050
28920	O	1.738658251850	0.117153505361	-5.358622834442
28921	H	0.815692012372	0.257771652472	-5.072083019246
28922	H	2.252114394003	0.137515384853	-4.524181105896
28923	O	0.901056560167	-5.110228440457	0.914130622683
28924	H	0.833486362633	-4.987444330917	-0.071865559102
28925	H	1.077859923046	-6.041183112143	1.050944594884
28926	O	0.691538609562	-4.800618504795	-1.680159242887
28927	H	1.216716750740	-4.033537009601	-1.983141342540
28928	H	-0.232677145547	-4.615050057304	-1.909240798493
28929	O	4.996119390465	0.216813590397	1.474775791161
28930	H	4.748996963722	-0.708734240838	1.666762607738
28931	H	5.874764828162	0.344933724555	1.834300721635
28932	O	2.202978859101	-2.702294328769	-2.581973302756
28933	H	2.341151225575	-2.880121683253	-3.521922079146
28934	H	3.082089687037	-2.767803288309	-2.136965642445
28935	O	4.919617046870	0.226167104406	-1.350644903262
28936	H	4.870103636452	0.255861309899	-0.381813987536
28937	H	5.093679677106	1.159756902105	-1.619624962582
28938	O	4.281894549007	4.269703837189	0.404469321703
28939	H	4.655827570056	5.133005622364	0.580627794124
28940	H	3.411334079469	4.437308994418	-0.046908860026
28941	O	-1.399965324921	-2.549252342746	4.044708908415
28942	H	-2.221401821054	-2.064826853318	3.885988888147
28943	H	-1.440001555819	-3.331849173423	3.453155005052
28944	O	-1.898759482319	-3.988831038494	-2.289406603099
28945	H	-2.349132897432	-3.901841129424	-1.417722555215
28946	H	-2.600258262713	-4.192901192018	-2.925603552664
28947	O	-5.127545507835	2.638977443235	-0.078712628484
28948	H	-4.456665149054	2.994187834548	0.530965494689
28949	H	-4.827534337519	2.922028948749	-0.952795278918
28950	O	3.609853864003	2.498510329651	2.344601188908

```
H      3.940798235694    3.168456306030    1.717449663133
H      4.003138543497    1.656763493471    2.059839095163
O     -0.424902860210    4.559921299611    0.637363854615
H      0.136386445454    4.390364331855    1.418527424719
H     -1.323397189761    4.320041949178    0.926272153715
O     -6.439172229987    0.332867101107   -1.875296566367
H     -6.843969040709    1.193120923571   -1.751186851792
H     -6.171149848428    0.042448161939   -0.972054897295
O      2.289717534726   -3.409462624345    2.563726931975
H      1.818566357169   -4.043185622558    1.994610459600
H      1.655263906284   -2.705011230751    2.753579267199
O      2.684633711616   -0.020606127353   -2.860213454824
H      3.533737330035    0.047411382229   -2.375747138880
H      2.362307833914   -0.924309792157   -2.680199374452
O     -3.858052181754    0.245695612483   -2.465614598887
H     -4.819720799975    0.409730071663   -2.314139456665
H     -3.798257719804   -0.717387935718   -2.348148724197
O      1.745242313257   -2.568076429417   -5.323000878949
H      1.797522051527   -1.588205168981   -5.442326546058
H      1.820067761094   -2.953204164228   -6.195565464608
O     -3.080302878935   -3.630247466090    0.090900933183
H     -2.918482499067   -2.675105264834    0.269019747667
H     -4.051328918210   -3.696596313369    0.144294387417

Cope TS Water45-13
155
ANGSTROM
C     -0.227442819759   -1.182770393592   -0.805412554683
C      1.045617625285   -1.546472564778   -0.157338437710
C      1.931407946643   -0.621303559226    0.240841037463
C      1.612486008102    0.857347181300    0.084615196925
C      0.225106145401    1.182299841719    0.801284798060
C     -0.989208754881    0.512577115930    0.163599135615
C     -1.572963589628   -0.662532415927    0.752879081250
O     -2.751986638455   -1.110751806245    0.533989241792
H      1.195539733854   -2.600700499863    0.044854343131
H     -0.886920005248   -2.019360381119   -1.014162655936
H      2.839362001896   -0.890601933789    0.764587249509
H      2.389319349064    1.485830163248    0.523504561601
H      1.504393143187    1.126060226851   -0.972407038162
H      0.310052126667    0.877763535016    1.847926493844
H      0.070732113066    2.263637750077    0.766023292773
H     -0.148067676199   -0.535026450589   -1.678850816489
H     -1.008401053536   -1.138567369519    1.569158682902
H     -2.399585526020    0.826967065870   -1.287629921279
N     -1.676002482099    1.280174069475   -0.745862986815
H     -1.134440856487    1.959440668878   -1.264447103734
O     -3.454184751405   -3.082501333774    2.277215851207
H     -3.210013307612   -2.286616145298    1.760589988905
H     -4.188777882123   -2.815512166768    2.834968406499
O     -1.064123543414   -4.347434220197   -1.371821483249
H     -1.981829699222   -4.209649225721   -1.081470448627
H     -0.611236105217   -4.821182595138   -0.643466122791
O      3.106953911112    2.823024494220   -2.464698191678
H      3.001392619412    2.313686084523   -3.283894235061
H      3.596833783054    2.252564467629   -1.854830631692
```

29007	O	1.759517385387	-4.655729283065	-1.994435938659
29008	H	0.840709252617	-4.391781932900	-1.841765019150
29009	H	1.853572294062	-5.456832318132	-1.470044251196
29010	O	-3.690355970212	3.771269504991	-1.673050856838
29011	H	-4.265619573415	3.056892131283	-2.083539012473
29012	H	-4.092526497052	4.605016987380	-1.919905428849
29013	O	0.454373994542	3.167587516021	-1.941122629037
29014	H	1.387188927273	3.065818681190	-2.209644311216
29015	H	-0.052184496218	3.419807190272	-2.718894121391
29016	O	-1.099584836453	-2.728739618572	-3.598073751677
29017	H	-1.184843146980	-3.462496314632	-2.968747135614
29018	H	-0.131573190627	-2.558130922702	-3.648308436124
29019	O	-1.340316087788	-0.951219200527	-5.632605178494
29020	H	-1.418175628782	-1.695212608552	-5.008151455321
29021	H	-0.785930665458	-1.267019703027	-6.346769629465
29022	O	-3.306478565141	0.766921621656	-4.531314847883
29023	H	-3.155624390027	0.131385454909	-3.791584108974
29024	H	-2.889593865216	0.351167066746	-5.292768083991
29025	O	-3.055370902691	0.023743233869	3.714279182168
29026	H	-3.445037448423	0.380558212005	4.516511586405
29027	H	-3.822548672453	-0.390139737940	3.230733470359
29028	O	2.587096790223	-3.859676107650	1.506467046303
29029	H	3.119498263593	-3.557400175581	0.757402415432
29030	H	3.168758340989	-3.802233450367	2.297635259922
29031	O	2.359633702240	1.071813797285	-4.433175983644
29032	H	1.422256926269	0.913068754552	-4.133545029930
29033	H	2.387908647675	0.871737168321	-5.368336719072
29034	O	-1.890947124108	4.422662399936	0.485156207700
29035	H	-2.030184860831	3.667573070868	1.070106269461
29036	H	-2.346714805429	4.209010390381	-0.337099982565
29037	O	-2.862894573451	-0.959853514641	-2.564061986694
29038	H	-3.581398547098	-1.494610828068	-2.220429058963
29039	H	-2.191230345940	-1.585057544030	-2.909617780206
29040	O	3.769311895545	-2.997616861717	-0.979438110354
29041	H	3.172345394877	-3.670009848486	-1.349285377778
29042	H	3.652389384921	-2.228418495709	-1.554326636116
29043	O	4.832806376836	1.115117700967	-0.922328220703
29044	H	5.066953208837	0.290194786922	-0.407237698260
29045	H	5.661667499629	1.411648624854	-1.303469510003
29046	O	-0.274519652795	3.270698029789	3.871807675827
29047	H	-0.280000731634	4.051843363644	4.425532281863
29048	H	0.565263855116	3.299231212542	3.367253635447
29049	O	-1.820065312199	2.825425362062	-3.578286364298
29050	H	-2.423538180772	2.209908767245	-4.040447382731
29051	H	-2.356509070784	3.221993081924	-2.874284042584
29052	O	-4.706274347683	0.290508518725	-0.569725866651
29053	H	-4.778601172789	1.082998088702	-0.004907859910
29054	H	-3.918788091631	-0.192486591895	-0.217125942803
29055	O	-2.183064997247	-2.188124020088	5.488980717293
29056	H	-1.853114007555	-2.965415079653	4.997535865168
29057	H	-2.475176246746	-1.565382511732	4.812800669479
29058	O	0.671950079266	-2.340179841178	2.862433015677
29059	H	1.160994157027	-2.147176610149	3.686537018892
29060	H	1.285830199899	-2.862976806906	2.319971968010
29061	O	3.486071968464	4.940706209858	-0.554097144043
29062	H	3.379638934689	4.423737068597	-1.362071489145

29063	H	2.593182133643	5.088805604085	-0.211232661437
29064	O	1.557649566206	-2.484236134628	-3.763057181083
29065	H	2.077701054771	-1.830079722680	-3.276980045700
29066	H	1.820208746120	-3.342497116716	-3.407977797591
29067	O	-5.273357128479	-0.923218359782	2.713359555783
29068	H	-5.520573538358	-1.368574496911	1.873099426401
29069	H	-5.805832608290	-0.107347361379	2.721865606444
29070	O	-5.943879047361	-1.953526413875	0.303258932277
29071	H	-5.685493676104	-1.121896915224	-0.137065077944
29072	H	-5.347710805346	-2.629474346250	-0.043878809682
29073	O	-5.106194275694	1.873507955596	-2.729202641094
29074	H	-4.679157111529	1.517837834195	-3.520261902084
29075	H	-5.038874070155	1.178185500149	-2.044167743766
29076	O	4.538534761904	-0.604417703647	2.850996231292
29077	H	5.213185555194	-0.083427441561	3.288853360535
29078	H	3.687318894159	-0.146419859476	3.034946863888
29079	O	-2.397685151108	2.319890533055	2.343551511647
29080	H	-1.724534993241	2.728758250270	2.910721174322
29081	H	-2.621332805229	1.464440147417	2.754124049289
29082	O	-0.307041096118	0.560008526627	4.263758670364
29083	H	-1.152791852618	0.204085317384	3.963057822632
29084	H	-0.381015473510	1.527763296998	4.269172147644
29085	O	0.810791141592	4.636534820093	0.301584888827
29086	H	0.721755079894	4.094863161391	-0.507878142798
29087	H	-0.111672775750	4.850343877516	0.522497242729
29088	O	2.370126977710	0.751888460078	3.621702179606
29089	H	2.316187684856	1.635771557440	3.232470738736
29090	H	1.452091056762	0.475523773549	3.776136082210
29091	O	0.041056341571	-1.015904029106	6.522080131511
29092	H	-0.046689837299	-0.291473161935	5.883032978114
29093	H	-0.750102275715	-1.566502805595	6.361473596462
29094	O	-6.581716897140	1.449112330667	2.330233923743
29095	H	-5.960781817921	1.991630983322	1.818493191716
29096	H	-7.353458815972	1.321669463012	1.776494262679
29097	O	-1.285688655798	-3.959934274959	3.635299641412
29098	H	-2.066546614360	-3.736889492889	3.092355997823
29099	H	-0.557302264495	-3.368770965200	3.322056448097
29100	O	4.382192510289	3.052385754983	1.174770116960
29101	H	4.545862921395	2.324522801807	0.559296942360
29102	H	4.221835689551	3.836242730842	0.609837754471
29103	O	-0.113159672686	0.637795392404	-3.679525034336
29104	H	-0.645208593031	1.446240818378	-3.578493967506
29105	H	-0.555557338088	0.102305620436	-4.358971828960
29106	O	-4.619646088790	2.645581699162	0.782146650416
29107	H	-4.329337693320	3.171488760909	0.021070821705
29108	H	-3.851411393491	2.583268039851	1.375319018801
29109	O	1.988776010501	3.345107037567	2.404506301770
29110	H	2.928302520002	3.276656417978	2.139513677733
29111	H	1.576835061878	3.839465391028	1.666402754205
29112	O	2.128474812362	-1.820151998971	5.115668358286
29113	H	1.402194730231	-1.668741997646	5.768584565497
29114	H	2.507991356902	-0.950764114844	4.955383915248
29115	O	0.580184206868	-5.494127897726	0.450276895303
29116	H	0.110397750462	-5.916577184844	1.195320271842
29117	H	1.276093754953	-4.957455240556	0.864105076688
29118	O	5.464194048852	-1.086169706873	0.320675914111

```
H      5.154578289898   -1.005919878062    1.241345632696
H      5.042257545624   -1.872975135868   -0.050214851090
O     -0.620775268428   -6.379331116285    2.708162695002
H     -0.083422353848   -6.809795770806    3.373275230867
H     -0.908028186570   -5.522923630945    3.101813314518
O      3.384091611895   -0.666878250859   -2.588047916978
H      3.117933852684   -0.085690428751   -3.322263665536
H      3.823042107839   -0.084653689352   -1.950029335464
O     -3.554649491870   -3.450239828608   -0.505830177962
H     -3.206052694610   -2.574708971234   -0.233376089236
H     -3.643716187502   -3.931381035519    0.326098184474
O      4.023704557545   -3.234723265525    3.662116227725
H      3.370800521143   -3.022362932029    4.345360295950
H      4.355068495987   -2.372381147773    3.365501520419

Cope TS Water45-14
155
ANGSTROM
C     -0.158781213777   -1.156725220479   -0.710203302318
C      1.163691630484   -1.464636458628   -0.101913606009
C      2.036647994574   -0.486989113389    0.182576053046
C      1.612265850657    0.964682415909    0.005129548910
C      0.244171278349    1.186009533588    0.790354624584
C     -0.944888248149    0.436331094059    0.163118722608
C     -1.638127025432   -0.614691405880    0.886205283693
O     -2.776964802744   -1.070861767469    0.592315475799
H      1.375564468879   -2.501119662784    0.144806549123
H     -0.832574896757   -2.004610065429   -0.823949993397
H      3.004074922811   -0.690413014610    0.625643312667
H      2.376195116991    1.643860236169    0.384294691400
H      1.438703263922    1.201529946198   -1.052156809643
H      0.384323086351    0.873645065707    1.829359368532
H     -0.007769130634    2.251281297910    0.792224354366
H     -0.095624659719   -0.590752878210   -1.638354730803
H     -1.107977413609   -1.072773448823    1.735648644659
H     -2.463537956421    0.815681992614   -1.199470748634
N     -1.648635808542    1.195967106355   -0.748746170513
H     -1.147661954758    1.863417743390   -1.315676486636
O     -2.542638906858   -2.988798063976    3.347242600619
H     -1.708331552706   -3.089788984685    2.842297508376
H     -3.233704560946   -3.367982674630    2.785235791357
O     -4.189666246202    2.090117551249    0.649322359651
H     -3.570697876646    1.804562455387    1.339364578215
H     -3.852499916742    2.954369938216    0.361743254164
O     -4.516069709915    1.455842893725   -2.073840372890
H     -4.491500811686    1.699020849625   -1.133607541624
H     -4.517700874515    0.489666717193   -2.107496821675
O     -4.339799120244   -3.111125999122    1.231792272005
H     -3.653011095931   -2.427956682061    1.034319598890
H     -5.130723164018   -2.590482833618    1.410757948667
O     -0.161353508343   -3.496907632849    2.157634128297
H     -0.098724594875   -4.050862987031    1.369199275361
H      0.166749294236   -4.050751644246    2.902648336153
O      0.691880557251    3.258451023716    4.636456283931
H      1.158774020591    3.412340301345    3.791408573276
H      1.048759005678    3.875213446606    5.275739495845
```

29175	O	0.816702830826	3.448562219355	-3.811230362054
29176	H	1.644551246034	3.447515441174	-3.306852538053
29177	H	0.151047757792	3.830126355888	-3.217523704568
29178	O	1.908006311732	-0.751017658184	-4.491102496825
29179	H	1.234494340187	-0.677524244243	-5.212239553511
29180	H	1.818957530054	-1.634357546843	-4.112432834118
29181	O	0.218776352561	0.864681345263	-3.097423911046
29182	H	0.900415771147	0.362292782985	-3.577868783293
29183	H	0.278423153900	1.774193828523	-3.440216755411
29184	O	1.809971966491	-1.872142043617	3.274514529512
29185	H	1.931639117220	-0.930339287533	3.049749729058
29186	H	1.098668678892	-2.222665762071	2.712889037555
29187	O	3.863513590094	-3.085962175413	-1.906839367153
29188	H	3.084989224793	-3.018135803080	-2.469309709461
29189	H	3.543124900851	-3.392729087906	-1.037231429751
29190	O	-2.374251307190	2.243634180417	-3.645899846278
29191	H	-1.966656384785	2.914462102904	-3.088910717578
29192	H	-3.219806010342	2.023075211229	-3.218974572019
29193	O	-0.196999501162	-0.428875775061	-6.085897966418
29194	H	-0.236593790736	0.541190365422	-6.229612432070
29195	H	-0.952465968603	-0.574479556773	-5.499895087691
29196	O	0.236916701408	-4.975004783832	-0.264577418348
29197	H	-0.504002155792	-4.558251761016	-0.745347256083
29198	H	0.599723857935	-5.608012325182	-0.898858119507
29199	O	-3.040227647915	4.519126979027	-0.072165911964
29200	H	-2.277921547077	4.451949336834	0.553843380036
29201	H	-3.529660290572	5.307637851491	0.160815441945
29202	O	-0.980603127335	-3.061040908628	-4.429752750713
29203	H	-1.338313937630	-2.162440068187	-4.165238218549
29204	H	-0.875471925905	-3.016276722424	-5.382035370574
29205	O	0.538032234335	-1.339118650427	5.639205423152
29206	H	1.088407484738	-1.647261027085	4.895580410785
29207	H	-0.143665184107	-2.016222085506	5.765918831633
29208	O	1.231252377166	-3.128305162761	-3.029609969635
29209	H	0.422050324951	-3.137050297552	-3.604289898252
29210	H	0.921483999771	-2.802021238747	-2.176230652431
29211	O	4.963933755359	-0.662688087830	-1.261752177929
29212	H	4.621240053363	-1.516340079341	-1.586708194698
29213	H	5.310710069661	-0.870898202171	-0.378231280861
29214	O	5.715843714194	-1.373566685317	1.326784877701
29215	H	6.595233400524	-1.726063719578	1.462530053470
29216	H	5.089393296099	-2.032556861610	1.740225560934
29217	O	-1.345499787148	-3.404059651735	5.717555257033
29218	H	-1.866672893318	-3.244381939884	4.901847003275
29219	H	-1.975130522252	-3.490872454140	6.433335956131
29220	O	4.027283254549	-6.009266730939	-1.532431286312
29221	H	3.177106059849	-6.115681239247	-1.989375980351
29222	H	4.452938590934	-5.244591077932	-1.937489128926
29223	O	-0.255345092587	0.633183656081	3.985198209990
29224	H	0.077203352703	1.470458182032	4.327112563651
29225	H	-0.078158176723	-0.036323703420	4.677072213806
29226	O	-5.334631486429	-0.413116453779	1.377793477355
29227	H	-5.594503329819	0.506535404374	1.267244227605
29228	H	-4.437568980229	-0.444488226914	1.004967448761
29229	O	-3.762425641315	-1.214796017033	-1.907034669120
29230	H	-4.136043351136	-2.103008507252	-1.950417845119

29231	H	-3.384359300393	-1.137188722343	-1.005754341266
29232	O	4.781730287511	2.040998462599	-0.260408355979
29233	H	4.217483398199	2.542421616303	-0.866590710955
29234	H	4.914784744650	1.181356315986	-0.683903738926
29235	O	-1.960545977758	3.737619707517	3.811346508129
29236	H	-1.151502037762	3.499553592284	4.286725892297
29237	H	-2.360626757401	2.888683638490	3.526450187406
29238	O	-0.691429109032	2.200040585622	-5.868350251634
29239	H	-1.428824981414	2.163202232713	-5.235255486905
29240	H	-0.026786493443	2.748776698602	-5.434795985615
29241	O	2.241105713564	3.331191262960	2.367014253188
29242	H	3.164958957133	3.591153622163	2.362646795782
29243	H	2.240700337042	2.357719889223	2.514963130235
29244	O	1.440546191395	4.344946849113	-0.283299643071
29245	H	1.609741081727	3.961134997263	0.587917094617
29246	H	2.064664619828	3.937869636657	-0.896899025492
29247	O	2.575556012654	0.766456459663	3.155107061201
29248	H	3.524762513358	0.813160208880	2.848839666125
29249	H	2.588976808713	0.807061631057	4.113130712454
29250	O	3.194506512149	3.199046005947	-2.302683524602
29251	H	3.794075645640	3.800160879666	-2.747688785104
29252	H	3.365328225770	2.302109345287	-2.707909309346
29253	O	4.079746408425	-3.096463769570	2.409817843425
29254	H	3.308154088935	-2.649894905658	2.807601487548
29255	H	3.715227765050	-3.649871265415	1.698009587718
29256	O	-2.625867804153	1.284795453650	2.883540346768
29257	H	-3.247720525098	0.681903620411	3.344377705290
29258	H	-1.742988889970	0.998897127331	3.189319010996
29259	O	0.736065247482	-4.666700687102	4.383011523998
29260	H	1.547179870854	-4.197169249502	4.592313015937
29261	H	0.057726021951	-4.349263034409	5.001428164558
29262	O	-1.695150089652	-3.890033474746	-1.819979605703
29263	H	-2.649170302730	-3.943632415331	-1.646496191701
29264	H	-1.598282147765	-3.785639497247	-2.773907183289
29265	O	3.827085586315	0.883918648214	-3.305854650741
29266	H	3.209624553041	0.322348143339	-3.799609825922
29267	H	4.264248294640	0.315744833056	-2.654312344361
29268	O	-1.861074935407	-0.705000619515	-3.829742748512
29269	H	-1.275013909598	0.023750431113	-3.522637706213
29270	H	-2.608207712893	-0.757128897079	-3.209666830235
29271	O	4.997826153493	1.084518927513	2.274987335152
29272	H	5.344227581835	0.206561281817	2.029980708873
29273	H	4.948605083923	1.564270617687	1.427625958499
29274	O	-4.405627721072	-3.897373308186	-1.314762379589
29275	H	-4.422894942358	-3.693318141852	-0.348106704128
29276	H	-5.017923414741	-4.618404508154	-1.458755976268
29277	O	-4.153657346792	-0.694995962520	3.860687290990
29278	H	-4.741776461444	-0.676736982455	3.084596905244
29279	H	-3.555093121929	-1.446853063221	3.736787380448
29280	O	-0.890213118734	3.921318753290	-1.746236459569
29281	H	-0.169559310909	4.107522199531	-1.115877855305
29282	H	-1.708965277061	4.176888763069	-1.296603474368
29283	O	1.480895794355	-5.805510115528	-2.649755686469
29284	H	1.440239472120	-4.841175719870	-2.832103746479
29285	H	1.171075847252	-6.251142643689	-3.437947384133
29286	O	-0.923539316976	4.350807280116	1.483176985619

```
H     -0.286536760125    5.060372771216    1.562561905690
H     -1.304519082990    4.171379986652    2.381546320220
O      2.912192136950   -4.414952254574    0.313418548062
H      3.384541013103   -5.164039261442   -0.103811811474
H      1.968611232022   -4.635328080528    0.268872989169

Cope TS Water45-15
155
ANGSTROM
C     -0.311979731336   -1.227362614853   -0.703423278154
C      1.085084155033   -1.385008366707   -0.236307107939
C      1.903195752604   -0.351830537548    0.003159920449
C      1.403884369784    1.085776900076   -0.015865357823
C      0.063328037221    1.184953894784    0.829167497052
C     -1.117113854065    0.443449614762    0.203904485518
C     -1.719648808979   -0.705454995330    0.823444457832
O     -2.884429672891   -1.156105858435    0.556110287337
H      1.424687114831   -2.402346175375   -0.068249128048
H     -0.877390843027   -2.155046517364   -0.739957486369
H      2.922443277052   -0.516113058797    0.326313052500
H      2.146757588102    1.758942435583    0.416706561110
H      1.186047534441    1.429196978959   -1.033316684408
H      0.254953790082    0.803828829321    1.836536802048
H     -0.215123652637    2.239531740718    0.905290143556
H     -0.414449178716   -0.695830374713   -1.646696879047
H     -1.181553882102   -1.175602319634    1.662619224962
H     -2.605928883741    0.715958026252   -1.172886004599
N     -1.829569693153    1.170578820082   -0.718332219306
H     -1.312960485026    1.776757884878   -1.340422030752
O      1.268015069627    4.764763630563   -3.407853279286
H      0.969689665290    4.679054508395   -2.488499766104
H      2.150662762620    4.363969112623   -3.441206648327
O     -0.081185840469    1.304745218949   -3.065826025916
H     -0.046256609644    1.983334659358   -3.773098209759
H     -0.874011890632    0.768422428945   -3.254249947506
O      1.481432352802   -4.283498079970   -4.962371347159
H      1.275451975318   -3.477439725051   -5.455219942415
H      2.375027392990   -4.171839033909   -4.622282099954
O      3.725237940944    3.721693587453    1.880184791197
H      4.595628390876    4.063082381649    2.088203302653
H      3.671276900228    3.686496007860    0.904220229163
O      2.783401513234   -3.901687488058    1.024027074475
H      2.996897835666   -3.834923706246    1.958871557013
H      3.486325102305   -3.421310707099    0.556300140304
O     -4.965811898136    0.233489554508   -0.237569642470
H     -4.171882693035   -0.312360525727   -0.004651968774
H     -5.716307173987   -0.206285119585    0.163410299709
O      1.650698518136   -1.082886236716    2.892422793239
H      2.065080980053   -0.220875004880    2.741600940736
H      2.401788127924   -1.664386028580    3.150379375328
O      1.253781642458    5.080866950779    1.831119455742
H      2.103201850543    4.655809230857    2.021522424952
H      0.580102316985    4.604845667567    2.332910740376
O      4.657265293986   -2.256489807538   -0.209781466994
H      4.702420792878   -1.611001663571   -0.937438798227
H      4.873866450303   -1.736490376489    0.577295624505
```

29343	O	2.541621577429	0.831272634396	-3.295188906437
29344	H	2.685465957492	0.151525059818	-3.995359926635
29345	H	1.572596873050	0.908733432495	-3.171022368283
29346	O	0.646450106159	4.300493728416	-0.685457355167
29347	H	-0.288103684065	4.081414489034	-0.574273286597
29348	H	0.882657107033	4.779884315165	0.134996357555
29349	O	-2.387001406842	1.147094994787	3.200041740139
29350	H	-1.731869659409	0.568130094376	3.635011523932
29351	H	-1.964151996708	2.013348594224	3.128849125341
29352	O	0.393209289817	-3.630927484914	2.982935628608
29353	H	-0.254389524712	-3.949526673246	2.332596627098
29354	H	0.821633212625	-2.853040106909	2.605044792406
29355	O	-2.107314880656	3.861995483072	0.147934483802
29356	H	-2.826355044997	3.304045050682	0.524629761287
29357	H	-2.381976779397	3.958397923934	-0.779301940265
29358	O	0.164664474286	0.952055761935	-6.662005487611
29359	H	0.356152168642	1.287204336850	-7.538124462103
29360	H	0.242438687285	1.720416245495	-6.054085315649
29361	O	0.679146082623	2.226854108564	4.367441699763
29362	H	0.226311338053	1.390895640859	4.568053416727
29363	H	0.072207166197	2.750777580096	3.831326785150
29364	O	-1.166337146128	3.763042681563	2.724795158015
29365	H	-1.693690787215	4.398665935039	3.212603797135
29366	H	-1.443514826094	3.857877438025	1.791650406372
29367	O	0.633886166514	-1.727506597246	-5.973673786139
29368	H	0.552580415183	-0.800585584801	-6.245135124672
29369	H	-0.282360952943	-2.050485999919	-5.897421241010
29370	O	3.609089355286	3.240236569247	-3.475123052933
29371	H	3.196530821863	2.339614464532	-3.447703644333
29372	H	4.267333134593	3.221979520522	-4.168907460392
29373	O	3.692004390310	-0.245336309552	5.247831052533
29374	H	3.524942823751	0.418626979944	4.557139038557
29375	H	3.038387778804	-0.090742067508	5.930458930762
29376	O	-4.262122382737	-1.125924549969	2.874879486102
29377	H	-3.853018862852	-0.360870497971	3.292757589637
29378	H	-3.846584648349	-1.184065180112	1.993884057070
29379	O	-1.004959768286	-2.885653574471	5.079200315953
29380	H	-0.786341713543	-3.419886786943	5.842308530646
29381	H	-0.415019905896	-3.195766415186	4.340223768506
29382	O	4.692508947615	-0.326107995646	-2.184190656453
29383	H	3.830651160748	0.082378000270	-2.410523174865
29384	H	5.014764583235	-0.713462960834	-3.000471779887
29385	O	3.348068143817	3.538383858629	-0.785300758506
29386	H	2.393605587637	3.720121999591	-0.803148838378
29387	H	3.623439003479	3.565532294364	-1.716176977177
29388	O	-2.596628915228	3.242234483794	-5.051972279704
29389	H	-2.787999219288	3.426355890580	-4.113679512511
29390	H	-2.956066957397	2.372717467660	-5.249847136246
29391	O	-0.411237116482	-0.301372633118	4.398854903613
29392	H	0.337510128637	-0.634058606522	3.853296611254
29393	H	-0.759575163158	-1.095417477214	4.835269208237
29394	O	-1.975963768170	-2.608421768989	-5.813527196817
29395	H	-2.105786581871	-2.704780650380	-4.843992513738
29396	H	-2.112145981758	-3.477499832323	-6.191423589318
29397	O	-4.487736408418	1.438717177635	-2.655983651055
29398	H	-3.800476866846	0.831819244343	-2.994179097312

```
H     -4.820923333751    1.038992330655   -1.837562589825
O      5.268470564304    1.497973382131   -0.185289601688
H      5.244875034244    0.884604505561   -0.934968475298
H      4.579896104382    2.152578352488   -0.367348643608
O      3.110201874105   -1.244818591827   -4.805446286776
H      2.296453593471   -1.464083658653   -5.282572152477
H      3.174134702140   -1.897300705825   -4.079200048239
O     -3.230388511148    3.723376723731   -2.393662765468
H     -3.895965400187    4.411383467606   -2.430348089960
H     -3.727596047048    2.861163362363   -2.405612832674
O      2.916419866585    1.419105609131    3.143953663231
H      3.281747223966    2.200928947586    2.693206321539
H      2.126663840076    1.760161935791    3.621079223331
O      3.922084809051   -2.357558226572    3.481570862694
H      4.490023075520   -1.882589095061    2.858818704944
H      4.004913885936   -1.859698304604    4.311069247726
O      0.405499679737   -3.542514117174   -2.604417611178
H      0.442797917609   -4.168905330353   -1.849210644678
H      0.644582341166   -4.034579141046   -3.414777057560
O     -3.104858318382   -3.542282343973    3.372922402256
H     -3.649685157218   -2.743019733815    3.230269631350
H     -2.478046463274   -3.305829028748    4.076254370896
O     -3.140873243197   -3.492156149261   -0.791486885460
H     -2.652224312875   -4.059246847987   -0.178522828165
H     -3.212454242630   -2.641434120499   -0.322658702384
O     -4.038630430641    2.361961034689    1.251841232421
H     -3.627074270251    1.845953386528    1.956555002237
H     -4.439614757307    1.711005795978    0.650994839553
O     -2.081809386197   -2.740752921723   -3.148016217299
H     -2.597159284293   -3.088301096705   -2.390533533112
H     -1.147488164500   -2.984624120840   -2.952359457054
O     -2.414521740782    0.159670373528   -6.091293381742
H     -1.528569274398    0.473200900123   -6.351947202214
H     -2.424355697749   -0.790770584878   -6.269460942959
O      0.597343069651   -4.961680471047   -0.341132303451
H      1.398421424384   -4.713702518022    0.152276508694
H     -0.149554677845   -4.787593976834    0.242241699489
O      0.054091416389    3.071636205245   -5.061449122880
H     -0.899732261077    3.292420545332   -5.140566130925
H      0.475484126966    3.801563040220   -4.552140854639
O      4.877982468027   -0.278036333089    1.821538195346
H      4.275708285990    0.237300821238    2.371274341550
H      5.181404351986    0.357557871383    1.141757215686
O     -2.384290369079   -0.085828219791   -3.450777331288
H     -2.456600729102    0.016309608395   -4.427209408395
H     -2.321863620340   -1.050871063823   -3.288554739109
O      3.087427920738   -3.001797157147   -2.752048278218
H      3.563816708597   -2.992641777811   -1.917068886708
H      2.137176584306   -3.049232147151   -2.532125287883
O     -1.709560199060   -4.566151158129    1.417420489129
H     -1.856383678651   -5.504660725890    1.549297167373
H     -2.294438032918   -4.120780700974    2.099172493905

Cope TS Water45-16
155
ANGSTROM
```

29455	C	-0.272035003864	-1.273908261396	-0.727322524096
29456	C	1.062794025896	-1.558950027066	-0.155536024324
29457	C	1.942012175129	-0.594949143680	0.157401789673
29458	C	1.569171026517	0.877636759593	0.055150858984
29459	C	0.194301707187	1.145503901339	0.817573060362
29460	C	-1.016249957436	0.463276783276	0.181699987272
29461	C	-1.650766386235	-0.680491184391	0.787825258509
29462	O	-2.822541521928	-1.104478971376	0.527270429113
29463	H	1.294252021898	-2.604837733804	0.030293757420
29464	H	-0.914414218647	-2.143451977564	-0.846488008844
29465	H	2.918931269920	-0.831029375111	0.561515732140
29466	H	2.343985504871	1.511337420591	0.494351480850
29467	H	1.435899545430	1.185559810542	-0.988986210840
29468	H	0.309188459284	0.827760845224	1.859366558656
29469	H	0.011535012562	2.224700534062	0.801705599442
29470	H	-0.266414801865	-0.683884247285	-1.640557301697
29471	H	-1.121089702541	-1.151881520679	1.630987533404
29472	H	-2.399756569751	0.751815152648	-1.306357602203
29473	N	-1.677139166648	1.206248651463	-0.758658562644
29474	H	-1.146701810389	1.879898269432	-1.289603043331
29475	O	1.432494527142	0.141389776771	-3.341286879166
29476	H	2.011143673227	0.588969819126	-3.996934204040
29477	H	0.596814225523	0.647684773170	-3.347795407351
29478	O	-4.115293023445	3.277567591456	-0.281804036570
29479	H	-4.290850240074	2.349519799729	-0.072348618973
29480	H	-3.691912225903	3.267036320284	-1.155872237101
29481	O	-2.827535581569	-4.094312039092	-2.557561632148
29482	H	-2.370074477927	-4.317991791048	-1.731629467109
29483	H	-3.600444110218	-3.580391360875	-2.259528078767
29484	O	-3.129960660759	2.936711312267	-2.878571158058
29485	H	-3.920863982324	2.382289280265	-3.014450362954
29486	H	-2.366462025331	2.374234025306	-3.102082138713
29487	O	3.790813865826	-3.469268869609	0.824725868335
29488	H	3.784348774761	-3.183154607284	-0.109542417353
29489	H	3.269049321679	-4.300636791938	0.816626988090
29490	O	-3.072943920747	-0.430217968632	-2.740055624562
29491	H	-3.558006842154	-0.014322811863	-3.455915722786
29492	H	-2.409381962959	-1.023855492373	-3.135647995862
29493	O	-5.602212578231	0.302710157484	2.708324450426
29494	H	-5.968869973642	-0.247954093087	1.998156920255
29495	H	-6.108097422457	1.115935233776	2.718265857568
29496	O	-5.276167103009	1.280696839404	-2.803370827189
29497	H	-5.068534321919	0.980465190834	-1.888838100166
29498	H	-6.097774666901	1.768162486811	-2.743967250090
29499	O	4.938471671541	1.186099132617	-0.364671973532
29500	H	4.769985582949	1.881910904646	0.290671173903
29501	H	5.248163419983	0.417070534043	0.152049051176
29502	O	3.794035223930	-0.181855376274	3.273338366988
29503	H	3.175477813530	-0.899882096306	3.059328554646
29504	H	3.303286942488	0.647285828011	3.137330295819
29505	O	-4.771194482171	0.566916329045	-0.243087213656
29506	H	-5.491991361674	-0.028551512901	0.005521139016
29507	H	-3.958147658077	0.063941374453	-0.005319327100
29508	O	-1.381913952161	-4.354825475861	-0.222884447432
29509	H	-0.592983274666	-4.621417711831	-0.721157364469
29510	H	-1.074297500737	-4.183502700369	0.678998802919

29511	O	-0.945874356685	2.080110917192	5.612248931804
29512	H	-1.262129212327	2.574467058056	4.827285356287
29513	H	-1.276043091043	1.179177129798	5.500882056233
29514	O	-0.861771265810	1.565469477258	-3.665901440186
29515	H	-0.463035448781	2.366264302368	-4.049061660535
29516	H	-1.163192036666	1.005567644232	-4.410022394830
29517	O	2.329579007320	-2.544774846740	2.802426438170
29518	H	2.650423341517	-3.055413213625	3.548864930842
29519	H	2.875345038794	-2.830187642791	2.020780485337
29520	O	-1.674117322555	3.174197612418	3.231507035655
29521	H	-2.131875324009	2.355408766962	2.925498051803
29522	H	-0.813077830896	3.196992720020	2.792854667730
29523	O	-2.128629794520	-3.212355040222	3.943033165548
29524	H	-2.881570611411	-2.915545669330	3.368284128661
29525	H	-2.452217061646	-3.962288259632	4.442467007146
29526	O	2.464656386956	4.704863619912	0.287567281173
29527	H	2.183664513056	4.286099668128	-0.542819942154
29528	H	3.260504419587	4.232921825846	0.578363528447
29529	O	-2.527800672888	4.843337497979	1.205170019889
29530	H	-3.202436801041	4.321517374501	0.721155527534
29531	H	-2.329251025832	4.337433444636	2.012563377542
29532	O	1.455858376051	-2.587616021119	-3.196295076640
29533	H	2.327991569326	-2.750967436095	-2.797654774390
29534	H	1.409041842291	-1.613273831805	-3.271156217897
29535	O	-1.491670474012	-0.230486317801	-5.581147181075
29536	H	-0.839049814363	-0.348498595415	-6.272008933782
29537	H	-1.485103794217	-1.052436662479	-5.053702237932
29538	O	0.779217417779	-4.870025840856	-1.849280425456
29539	H	0.960982107188	-4.018393037451	-2.291971894430
29540	H	0.568200078957	-5.498028766511	-2.541095549499
29541	O	3.870194482710	-2.885214427418	-1.846730853847
29542	H	4.623358555557	-3.332429559470	-2.233389991424
29543	H	4.006542696641	-1.910179772268	-2.011319894476
29544	O	1.644927507373	2.348634694725	5.878754049845
29545	H	0.662088339852	2.213719094563	5.762198144789
29546	H	1.737350181284	2.950506122997	6.616932497571
29547	O	4.404688533470	3.133285246415	1.549086960053
29548	H	3.798800337397	2.802505114008	2.248039099189
29549	H	5.193070126085	3.449100906199	1.990922751420
29550	O	1.185871964675	3.403715845980	-1.827356080737
29551	H	0.458197289519	3.830331844475	-1.337402859661
29552	H	0.991982565005	3.589524525817	-2.762395651488
29553	O	-0.075346770712	-3.903592697442	2.216148200469
29554	H	-0.769190601938	-3.680021810645	2.861383552670
29555	H	0.677631719440	-3.323783882645	2.409213745142
29556	O	2.554638611721	2.289530873282	3.296014932866
29557	H	2.350945865435	2.325236924721	4.246901143291
29558	H	1.825895527369	2.784916981215	2.871576639875
29559	O	3.337705101240	1.398898166233	-4.733479171495
29560	H	4.048047857806	0.753367140962	-4.714595962389
29561	H	3.556781190992	2.033692801528	-4.015816934144
29562	O	1.892042857353	-5.367587210421	0.705489622704
29563	H	1.151982585758	-5.086887377237	1.261281776693
29564	H	1.579047557712	-5.305186841121	-0.208848128377
29565	O	-2.859567561623	0.890222945020	2.649049586696
29566	H	-2.533681348910	0.365392826071	3.410341991118

```
H      -3.823698344377    0.799361796888    2.641538952251
O       0.734588322391   -0.438379039915    3.712819310952
H       1.278557402172   -0.369786553307    4.530221444150
H       1.069368820478   -1.216188711961    3.253012203479
O       0.195513521444    3.984971671775   -4.314492564299
H      -0.528548892738    4.544277993620   -3.906735281737
H       0.576929523404    4.488997143947   -5.031891868900
O       4.033165180170   -0.363725822425   -2.418710409511
H       3.103143011461   -0.117419666961   -2.539340974518
H       4.376952649250    0.197042221168   -1.703585622439
O       2.484089738760   -0.263876676693    5.737320231716
H       3.241439266319   -0.224127886172    5.132747018170
H       2.278333860629    0.655134492484    5.976954362635
O      -0.613993975416    4.954069623036   -0.534575028927
H      -0.065801996166    5.688177860882   -0.254028271268
H      -1.353510038761    4.887628328767    0.140209264539
O      -1.737961848198   -0.518326342872    4.650316173738
H      -0.821778943334   -0.461762638498    4.292247327072
H      -1.944797467552   -1.463197309063    4.620778681122
O      -1.691417958308    5.252104438215   -3.043089015342
H      -2.395467619143    4.582527391755   -3.053191369790
H      -1.364185772629    5.252261456465   -2.123334205391
O      -6.190274202390   -1.655031847919    0.694820911964
H      -5.704331465510   -2.165343390605    1.361112909095
H      -5.826178175428   -1.980425895177   -0.151370280743
O       5.445713775988   -0.964167834086    1.235757543122
H       5.007907942503   -0.653266189621    2.050359852409
H       5.151544216627   -1.871536413528    1.104285653496
O      -4.111438271437   -2.205761518313    2.518586444582
H      -3.595839504651   -1.829220571539    1.753343839691
H      -4.426997575376   -1.440265274458    3.010797080282
O       3.848273581953    2.826600796784   -2.495739038086
H       2.949849097519    2.970991104642   -2.165951475089
H       4.318164237970    2.317942569729   -1.823302061539
O       0.793305514961    4.026761464199    2.207943645943
H       1.354978339294    4.308483257578    1.439004970422
H       0.644371496406    4.815370320197    2.731630188723
O      -4.452570705201   -2.276878742808   -1.323596899987
H      -4.196378007237   -1.548177258529   -1.922540941140
H      -3.807618185352   -2.185212138457   -0.602093172856
O      -1.365161248304   -2.292762936261   -3.818036281294
H      -1.901887461756   -3.034702166373   -3.436309158152
H      -0.443202961440   -2.482610231808   -3.606852615684

Cope TS Water45-17
155
ANGSTROM
C      -0.214474259503   -1.114847780128   -0.825253778117
C       1.089335890678   -1.483523709414   -0.220478376609
C       1.957728595488   -0.551357267276    0.196816752436
C       1.586355521086    0.927401674522    0.147679568381
C       0.192070301350    1.128628291867    0.877958663164
C      -0.991356888526    0.429261458052    0.197661959398
C      -1.596511485125   -0.726149047560    0.807112192980
O      -2.788427631764   -1.141740857462    0.597321018452
H       1.289601901506   -2.544605331280   -0.100771787463
```

29623	H	-0.877267056723	-1.946342152572	-1.055154889157
29624	H	2.899104143071	-0.811740067475	0.665012854154
29625	H	2.336682218805	1.542207109799	0.650926971928
29626	H	1.488756153614	1.278974000069	-0.888536072527
29627	H	0.295940188495	0.761156809562	1.903178451345
29628	H	-0.038169230496	2.196964009396	0.927116583694
29629	H	-0.137847420487	-0.456629431509	-1.692253416882
29630	H	-1.013481254689	-1.258305215304	1.571441401945
29631	H	-2.486508171824	0.811225409590	-1.173740437080
29632	N	-1.731826530272	1.232303966784	-0.644966538651
29633	H	-1.226283685725	1.931483543287	-1.174346302366
29634	O	3.745758069924	-5.154487333368	-1.254716349808
29635	H	2.909410969956	-4.891746401747	-1.657266513626
29636	H	3.506963076491	-5.619729350565	-0.447311060645
29637	O	0.957600690037	-4.826621052463	-2.036095788870
29638	H	-0.011062105327	-4.578414264748	-2.060469435807
29639	H	1.018090653564	-5.610502952926	-2.585456641676
29640	O	-0.021760290555	5.286563686715	4.198590163526
29641	H	-0.535703262790	5.991168989806	3.802844141104
29642	H	-0.654021312821	4.557702272682	4.384719038042
29643	O	4.384084941783	1.121456681935	-3.829778336492
29644	H	4.879827856681	0.806676014855	-4.586500807715
29645	H	4.094164116820	0.322690959530	-3.336780330302
29646	O	0.040647754669	0.525321136757	-3.570187008218
29647	H	0.743126112561	0.938391463915	-4.118258497040
29648	H	-0.181024503221	-0.306639967520	-4.013787964558
29649	O	4.172836812021	3.799477186830	2.452320408290
29650	H	4.385041582691	2.852284002023	2.650129974278
29651	H	4.746593555550	4.323135014934	3.011734083691
29652	O	-0.682363058884	-0.826282390590	4.805503320615
29653	H	-0.448505638986	0.094106870960	4.622396737133
29654	H	-1.668767703840	-0.862060675109	4.895835037787
29655	O	3.851697134995	3.622951696939	-0.280446592993
29656	H	4.110677920429	2.689417166779	-0.384545801240
29657	H	4.054449883946	3.845251444830	0.643066794633
29658	O	1.435852224005	3.674715459219	2.616818211238
29659	H	1.023096467054	4.352421167451	3.191966546805
29660	H	2.399144971658	3.808809185023	2.649610962934
29661	O	4.802803930126	-2.152427454249	3.340864987781
29662	H	5.301663612462	-1.645070672394	2.686204256984
29663	H	4.283981954823	-2.799002521137	2.835509376307
29664	O	2.959052959879	-3.421129503017	1.730839474859
29665	H	2.522203026163	-4.237902202644	1.409694153115
29666	H	2.245595326605	-2.880946774474	2.102142814328
29667	O	-4.283746481017	-2.152885342270	2.651103398287
29668	H	-5.169038058008	-1.860887187246	2.396715765030
29669	H	-3.703040931870	-1.854986098013	1.927442580178
29670	O	-0.817214516145	-1.888668973363	-4.641502628492
29671	H	-1.097422697266	-1.874031339300	-5.557093281626
29672	H	-1.624511838976	-1.926545520657	-4.097391354821
29673	O	5.628515229255	-0.618043720781	1.077690165071
29674	H	5.458060623270	-1.427216059894	0.569541511811
29675	H	5.429057103380	0.098392926148	0.452257773684
29676	O	-5.044562770358	-1.334251049375	-0.983361311616
29677	H	-4.976316185181	-0.583708406676	-1.588580048260
29678	H	-4.193662585444	-1.356973069019	-0.512475572727

29679	O	0.725120290393	1.501380172473	4.070235047599
29680	H	1.017235339185	2.249537378163	3.507147894299
29681	H	1.521042025098	0.991311805524	4.255923322171
29682	O	-1.572661707718	-4.278140332463	-2.162189483142
29683	H	-1.954588369775	-4.268910318193	-1.250888919209
29684	H	-1.900645490413	-3.476630593988	-2.585021453687
29685	O	1.906285278414	1.749627294202	-5.031172217518
29686	H	2.770292180453	1.585857731092	-4.622861213958
29687	H	1.655870652940	2.654727898398	-4.783110344896
29688	O	-1.590885014434	3.087697277049	4.209892584386
29689	H	-0.855318161393	2.464472092361	4.293793760636
29690	H	-1.776415751287	3.156433399834	3.251960786768
29691	O	-2.981075312683	-1.892355790466	-2.970973051169
29692	H	-3.780761719430	-2.356126238522	-2.702892535821
29693	H	-3.195097180125	-0.947061661268	-2.862390835050
29694	O	-2.345808237697	3.099313285947	1.630243031155
29695	H	-2.409634861419	3.634365473128	0.823492589858
29696	H	-3.010291168960	2.408188578431	1.536620009991
29697	O	4.713404029114	1.295414141695	3.028811622322
29698	H	4.034952779664	0.783269891800	3.499826193158
29699	H	5.136919250679	0.675677374060	2.420546327223
29700	O	-3.874929972466	0.676092249060	-2.557090479541
29701	H	-4.325357413917	1.374393411470	-2.028167240618
29702	H	-3.312348613927	1.185403558439	-3.184679155195
29703	O	4.519125671164	-2.702946638944	-0.384066841699
29704	H	4.540380268829	-3.564380403925	-0.837015106651
29705	H	3.920407022181	-2.863994353843	0.374121660184
29706	O	-0.483138218211	-4.099539518529	2.160219975282
29707	H	0.049910555150	-3.312936337207	2.357465260760
29708	H	-1.118233208053	-4.165457969240	2.900796267823
29709	O	1.220817617195	4.433441150501	-0.003072839162
29710	H	2.135719223643	4.209816571460	-0.241872740399
29711	H	1.140546896848	4.171599423676	0.932324621007
29712	O	-0.408186671398	3.579465895015	-1.898384581684
29713	H	0.175224788222	3.864350834141	-1.154174081868
29714	H	0.120852547815	3.766037136449	-2.691912670316
29715	O	4.757370912057	1.115770343657	-0.887963156776
29716	H	5.371743447537	1.325642948453	-1.595450593975
29717	H	4.144056840920	0.453436780453	-1.261498537630
29718	O	3.641803439416	-0.938161853235	-2.274489129689
29719	H	2.884040262132	-1.394705343806	-2.689116488128
29720	H	4.015198194017	-1.595706974570	-1.652072300538
29721	O	-2.418057301904	-3.778890452789	4.016746067367
29722	H	-3.184236770688	-3.494322132400	3.498284986556
29723	H	-2.337572279889	-3.135622118926	4.726365389027
29724	O	1.536157670065	-1.320068598908	6.317471921048
29725	H	1.557119177732	-2.238231302739	6.588709236060
29726	H	0.657637917254	-1.166432442693	5.918448704030
29727	O	1.696555228552	-2.466909291696	-3.370860625217
29728	H	1.492031055562	-3.265471478832	-2.861793055208
29729	H	0.890594638063	-2.244552111752	-3.852139919039
29730	O	1.381051405187	4.229496130063	-3.908481803138
29731	H	2.291401925536	4.163105746747	-3.521873240066
29732	H	1.349182042472	5.059724601379	-4.383346342615
29733	O	1.451895046106	-5.390232441197	0.688615563778
29734	H	0.687629484659	-5.170203872907	1.247457425849

```
H      1.174504901418   -5.203361357794   -0.222851069789
O     -2.465272746760   -3.914174196708    0.287242350762
H     -2.712065058055   -2.984117572422    0.397188317198
H     -1.763560102278   -4.081805848774    0.944040643449
O     -4.332406807551    1.013102079593    1.425062839079
H     -5.187764945048    0.565265425781    1.548960479097
H     -3.749842690095    0.313027530672    1.077666476881
O     -2.681803914871    4.420737451915   -0.789881329688
H     -2.698716500575    5.376101808354   -0.843313186691
H     -1.863291683926    4.124780016047   -1.248979165249
O     -3.295412374037   -0.947054629849    4.910542990774
H     -3.712205860594   -1.396613160821    4.152409586891
H     -3.591981472448   -0.015710929571    4.841422561798
O     -3.935142547610    1.573512017960    4.254713975035
H     -4.091770563102    1.519199324322    3.302412145821
H     -3.189710282018    2.184392717951    4.382061298489
O     -4.776461578446    2.525694095546   -0.873497189399
H     -4.117038448813    3.234811523629   -0.889696238009
H     -4.676083353094    2.084083811337   -0.013074925052
O      0.935812854382   -1.832029278187    2.929890811522
H      1.694827086280   -1.290289267563    3.227381736229
H      0.250379196683   -1.616136822478    3.594055701462
O     -2.078902586706    2.102029016325   -3.896177735285
H     -1.286288391753    1.520331543020   -3.811808004603
H     -1.865973110432    2.876538588036   -3.369978761831
O      3.841651992528    3.861420636012   -3.021004451404
H      3.893167603156    3.925868421135   -2.053676896646
H      4.090571639218    2.956780164500   -3.253126329747
O      2.932504767637   -0.472127645757    4.163655493025
H      3.654655720953   -1.119801340000    3.965395047297
H      2.601024444364   -0.716181103945    5.050707981987
O     -6.360237718348   -0.813553625742    1.285651091539
H     -7.313714620441   -0.807961325385    1.209792207667
H     -6.002144145836   -1.072453223026    0.402889568634

Cope TS Water45-18
155
ANGSTROM
C     -0.190175749418   -1.184674354191   -0.748073232192
C      1.119671621741   -1.504245080145   -0.129539510276
C      2.002800844394   -0.538684529388    0.160551765962
C      1.619120637961    0.924085651617   -0.023972550153
C      0.255113864449    1.187426095109    0.745251452345
C     -0.955790204407    0.449712268278    0.161302879336
C     -1.600667766714   -0.645161704606    0.841028106279
O     -2.772882327308   -1.066859612826    0.576358345693
H      1.315567001523   -2.546455936446    0.104503341734
H     -0.854979536728   -2.035677805532   -0.879322160928
H      2.956114619514   -0.751539607984    0.624067140261
H      2.384335017885    1.590651759892    0.377507062299
H      1.473784509265    1.169936472506   -1.083692128962
H      0.404197655117    0.902803106243    1.789765134626
H      0.037118120779    2.258083819475    0.711535358155
H     -0.137600010847   -0.610931588061   -1.674354949571
H     -1.056115658052   -1.135103958114    1.661009343204
H     -2.493905165677    0.782220292926   -1.167877008214
```

29791	N	-1.706959812025	1.215236192026	-0.712857660974
29792	H	-1.225358891083	1.912315450765	-1.269844463160
29793	O	0.164428951459	-3.843529260505	-2.904367562730
29794	H	0.750034487312	-3.131628373630	-2.586399428909
29795	H	-0.188515115752	-3.524814838289	-3.751291804984
29796	O	-2.287705068578	1.674279569980	2.374521947534
29797	H	-3.114018176794	1.608705188617	2.892254359527
29798	H	-2.030863644176	2.625676332543	2.304263078289
29799	O	1.340822111661	4.066575064297	1.965946825657
29800	H	2.053308734068	3.474988185811	2.286956642912
29801	H	1.570796147662	4.236466896234	1.039936311318
29802	O	4.119109148523	2.682524037079	-1.416754382226
29803	H	3.904242258322	1.843333781926	-1.856485683210
29804	H	4.551075895276	2.420714667181	-0.583654481100
29805	O	-4.735425109776	1.007222535587	3.314647621001
29806	H	-4.440125356694	0.273921119051	3.879575228525
29807	H	-5.119738626996	0.580258703037	2.531403119928
29808	O	-1.537694969036	3.420924333544	-5.116436696021
29809	H	-2.187406796593	2.889489814057	-4.615446415150
29810	H	-1.512988519266	4.273601573783	-4.678374837214
29811	O	1.168737204791	-5.597709678708	1.789428694705
29812	H	1.761769606671	-5.279253270249	1.083477946993
29813	H	0.891980073437	-4.804151755045	2.270952060306
29814	O	-1.615916771401	-4.586075231156	-1.008864117966
29815	H	-1.359583121784	-5.469604930931	-0.688642291322
29816	H	-0.938078616425	-4.340588866914	-1.670176293568
29817	O	2.306416327708	-2.144370275923	-2.752223572620
29818	H	2.778818759911	-2.988548494692	-2.650771243533
29819	H	2.189423747161	-2.024172406181	-3.716731150769
29820	O	3.249811874050	-4.161087526340	-5.640686456307
29821	H	4.124390512285	-3.942125908645	-5.963470372903
29822	H	2.734692844955	-3.335740637322	-5.654160413618
29823	O	2.716439458020	-2.346784319257	2.863724057663
29824	H	2.562333076178	-1.428954332153	3.141439307425
29825	H	1.830237756859	-2.724012857675	2.754382589037
29826	O	-2.089953772979	-4.516160517266	1.690101872795
29827	H	-1.947862100259	-4.336064649744	0.742914838645
29828	H	-1.890174838733	-5.453190095337	1.782536303818
29829	O	-0.380580676124	1.054489381715	4.169982128788
29830	H	-1.064052596141	1.162174195579	3.472826527150
29831	H	-0.214873646626	1.969528475469	4.481488499587
29832	O	-3.482198619557	-2.671973154515	-1.480653665839
29833	H	-2.974402517143	-3.489672383575	-1.341765601050
29834	H	-3.212840201347	-2.068147516174	-0.759661105757
29835	O	0.053885258564	-3.177190174713	2.796153309480
29836	H	-0.744300854934	-3.590839066557	2.423244200734
29837	H	-0.263815912655	-2.662188284133	3.563930457530
29838	O	-4.229929575028	3.668410393299	-1.640622353537
29839	H	-5.155937001488	3.582262732280	-1.886320649643
29840	H	-4.143548516494	3.125932517457	-0.826435666397
29841	O	-0.799157797346	-6.734292465431	0.471254273021
29842	H	-0.490885086843	-7.597563402434	0.198357029094
29843	H	-0.041866533062	-6.324899880541	0.974261999872
29844	O	-1.429393346192	4.156863753941	2.013844267504
29845	H	-1.696839935594	4.493478496807	1.134668165605
29846	H	-0.454847646088	4.147809867876	1.983924937400

29847	O	-4.055124028218	-2.727434094268	2.214310113308
29848	H	-3.517730093148	-2.140258904275	1.631976354807
29849	H	-3.546928057906	-3.553091004378	2.218945536772
29850	O	0.604702779881	2.022485757742	-4.295091332179
29851	H	0.085832823407	1.242261676593	-4.001278195501
29852	H	-0.040038126516	2.552984113230	-4.807852081637
29853	O	2.745256069122	0.780012916158	-5.193192053043
29854	H	1.970345902577	1.299301558202	-4.861457630110
29855	H	3.213402960977	1.348918646881	-5.803982517270
29856	O	-5.036656128348	0.023954803678	-3.741402829348
29857	H	-5.818664751254	0.336749656824	-4.196023776967
29858	H	-5.358700266390	-0.380540015170	-2.896858218459
29859	O	-0.801848472684	-2.726506370412	-5.262920835033
29860	H	-1.623488537609	-2.372419618031	-4.867490622707
29861	H	-1.067191902810	-3.341479385200	-5.947215572497
29862	O	-0.024005867022	3.704536871388	4.518539925620
29863	H	0.708974397410	3.909970881757	3.924949426226
29864	H	-0.801850723732	4.070010007128	4.082757468510
29865	O	2.805196078193	-4.831772608645	-3.020128712060
29866	H	3.059935323379	-4.779000826594	-3.958129543037
29867	H	1.835987321892	-4.865087139167	-3.019655478448
29868	O	-6.249320200563	1.670560870157	-1.483034761204
29869	H	-6.803557283802	0.888513671995	-1.478252062792
29870	H	-5.650246441550	1.574073056266	-0.729271310016
29871	O	2.108100304418	0.166381924814	3.875430912567
29872	H	2.403420223536	0.089206919189	4.783969298856
29873	H	1.163935672063	0.449878340424	3.927106453827
29874	O	-5.375531548974	-0.506205016574	0.998801364587
29875	H	-5.466530754266	-1.351118870525	1.455093145266
29876	H	-4.423051941997	-0.444954990857	0.785668760970
29877	O	-3.863275399968	-1.375407599544	4.464658952674
29878	H	-4.325782052533	-1.843500759718	5.159649791253
29879	H	-3.954811982992	-1.922059152065	3.640413987072
29880	O	2.131911165579	4.263148838156	-0.725017913662
29881	H	2.871253066828	3.652015401683	-0.984901999449
29882	H	2.409485026914	5.143155018200	-0.979734145181
29883	O	-2.945946689128	-1.770116544048	-3.955745032657
29884	H	-3.693827597638	-1.170261703751	-4.105022407572
29885	H	-3.107990565024	-2.159965124707	-3.068192902533
29886	O	3.695071947045	0.172918660310	-2.591315905371
29887	H	3.130664186776	-0.611961763446	-2.448554740078
29888	H	3.552902954559	0.408190668361	-3.520117387754
29889	O	-0.109640866692	3.319417064150	-1.957937100358
29890	H	0.676977124308	3.635358724370	-1.477053030262
29891	H	0.224542896525	2.934208085782	-2.786455047486
29892	O	-3.163087912780	2.001460491602	-3.444998389066
29893	H	-3.484271288101	2.648533351948	-2.779593826254
29894	H	-3.888697324397	1.365314080445	-3.577660586684
29895	O	1.852891630953	-1.797829683071	-5.400127129995
29896	H	2.094285071975	-0.855442265672	-5.452270087307
29897	H	0.888029665138	-1.875400973745	-5.432424304068
29898	O	3.302520631611	2.391432185523	2.758213528175
29899	H	3.969553252954	2.124552512813	2.108973549534
29900	H	2.921528100411	1.569277906231	3.111307877665
29901	O	-4.253839875585	2.091056435350	0.521292456628
29902	H	-3.377192205892	1.824201548238	0.862728638615

```
H     -4.745967867384    2.405613278484    1.283631444684
O     -1.131837957436    0.217039645763   -3.456905940783
H     -1.628277100324   -0.576772100444   -3.715382300072
H     -1.808407021232    0.917258637121   -3.390396675659
O      5.458764664890   -0.616277776450   -0.519236792894
H      4.917198515315   -0.413889203170   -1.297573103949
H      5.063177314588   -1.396577476840   -0.093160489545
O      5.227949893064    1.610246313202    0.858514868069
H      6.115330225342    1.877018865214    1.098799088737
H      5.317750890221    0.730054278624    0.403668726010
O     -1.073965936526   -1.583355465449    4.699140665161
H     -2.041192305158   -1.519105847624    4.658371117614
H     -0.763005623524   -0.667543398536    4.648739628056
O      4.442648797807   -2.784393360785    0.918746249754
H      3.787525763093   -2.587430956591    1.639925219701
H      5.151869388036   -3.270304323454    1.341576239623
O     -5.867108037140   -1.294148138279   -1.582435429402
H     -5.808892799614   -0.943323269303   -0.672631810555
H     -5.198875109741   -1.996392803091   -1.617161592553
O     -1.927473133961    4.886698609770   -0.558936127845
H     -2.786743589155    4.696822390637   -0.952577790084
H     -1.288518696321    4.372152836235   -1.076773867378
O      2.672493084890   -4.573796435930   -0.215375051473
H      2.875368144610   -4.812466404089   -1.126211948626
H      3.343123537851   -3.931586786155    0.071661136953

Cope TS Water45-19
155
ANGSTROM
C     -0.243893344692   -1.152001459313   -0.851362220933
C      1.132457284881   -1.521857024066   -0.452505704385
C      2.114988812176   -0.610368482507   -0.400538240033
C      1.802282672194    0.863406945106   -0.631079719408
C      0.624757659225    1.295438851156    0.348655207037
C     -0.721769739612    0.639561840716    0.010456778515
C     -1.303433213964   -0.347155334421    0.889594339093
O     -2.516321655741   -0.714535342444    0.912987103227
H      1.302771293995   -2.557161361312   -0.168153973995
H     -0.974516929817   -1.951617161042   -0.777418076198
H      3.122254311501   -0.872447373404   -0.100175530844
H      2.672094721400    1.491409766895   -0.437879381512
H      1.470168851165    1.049940088917   -1.660282622698
H      0.928034786625    1.051872694046    1.372165018657
H      0.498820771086    2.380314600302    0.279381096624
H     -0.329937968640   -0.661745940418   -1.820422893236
H     -0.634333523843   -0.779520387968    1.653955023278
H     -2.372902778282    0.984446988411   -1.181671995733
N     -1.507181837429    1.373544739911   -0.844756317345
H     -1.050630528477    1.997211556222   -1.503216931781
O      2.183329146788    3.035118150446    3.597214753473
H      1.720388523259    2.192653797096    3.716248607451
H      1.489668617955    3.702904242422    3.495237359329
O     -3.229249709818    2.460308707327    1.157505642700
H     -2.570738348686    2.255408032429    1.843139288783
H     -2.811782716201    3.166417325139    0.626451391621
O     -2.247670090486    4.161661274532   -3.557593683321
```

29959	H	-1.924664204266	4.336309976387	-4.442439961378
29960	H	-1.453597064167	3.927470712930	-3.020122436960
29961	O	-0.854608554399	-3.762218911005	1.707197339625
29962	H	-0.311536416433	-3.305724315293	2.376029778600
29963	H	-1.758624126095	-3.680697913043	2.043314211978
29964	O	1.939917620556	4.253251809426	-0.606846981946
29965	H	1.391478195012	4.896615083361	-0.136049568888
29966	H	2.553965875762	3.890536764858	0.064710224794
29967	O	-4.520116857983	-3.777867284742	0.216252210049
29968	H	-4.226601607960	-3.550967052612	1.114933708483
29969	H	-4.847239228680	-2.947170699522	-0.166327246162
29970	O	1.871082997397	-5.382294089823	-1.746057610746
29971	H	2.580751589786	-4.725372486360	-1.888252135497
29972	H	1.758822059209	-5.437955171878	-0.788367182267
29973	O	5.119863840937	0.880104495934	0.436676465991
29974	H	4.930551993654	0.796671580288	-0.519317180498
29975	H	5.999428917610	1.253569892506	0.510183455357
29976	O	-2.974964854771	0.746358561617	4.568384146415
29977	H	-2.464314317813	1.441893626248	4.119522308800
29978	H	-3.655838783788	0.474601415852	3.928751874650
29979	O	3.754506277585	-3.406394236371	-1.930267375993
29980	H	3.460840714269	-2.525119941583	-2.189826458406
29981	H	3.912641584812	-3.367643994786	-0.972471371646
29982	O	-2.257904295851	-3.723955794726	-4.873196203634
29983	H	-1.522743558117	-4.061894968572	-4.342585470793
29984	H	-2.872905461825	-3.328944513679	-4.233468004952
29985	O	-3.654683785739	5.697106006071	-1.797647919835
29986	H	-3.247971137110	5.215999106685	-2.537976604955
29987	H	-4.507589144026	5.248387794045	-1.610527594777
29988	O	-5.713505972377	4.240035292912	-0.851887706842
29989	H	-5.572333107812	4.400164183016	0.094295062144
29990	H	-5.566992968427	3.292332523391	-0.987195333095
29991	O	1.455824252288	-5.316323530479	3.734807828451
29992	H	1.143613171435	-4.402959686993	3.871538187818
29993	H	0.741114238357	-5.891702380717	4.011023335057
29994	O	-3.314267625205	-2.725595562892	2.476202015997
29995	H	-3.918274741853	-2.307486085937	3.092798253887
29996	H	-2.984883352223	-1.991545683794	1.903961174969
29997	O	0.753556951078	0.632649880364	3.733705150418
29998	H	0.152631306482	0.159069769856	4.337137246760
29999	H	1.542079690972	0.062322754086	3.666872117862
30000	O	-1.023615476422	-0.887115773243	5.172894976361
30001	H	-1.825157891207	-0.351604634048	4.916069016030
30002	H	-1.055053313281	-0.953054830947	6.127578419131
30003	O	-1.644618267158	-0.213105198457	-3.697235350644
30004	H	-1.775998538592	-0.562291282188	-4.617570064557
30005	H	-2.293578422493	0.515412702608	-3.593158692520
30006	O	4.711171101059	-1.304294956489	2.124476429532
30007	H	4.447613623331	-2.065260224291	1.578885141102
30008	H	4.821126574453	-0.541673309688	1.532092156506
30009	O	2.785102337447	-1.137095117116	3.860098718324
30010	H	3.241163639077	-1.117923551219	4.702253345089
30011	H	3.503110066461	-1.176958127053	3.168915745888
30012	O	3.888763897754	-3.592024987877	0.813505196473
30013	H	2.995342282967	-3.997168747753	0.921589699241
30014	H	4.512702928801	-4.271302346972	1.074865696446

30015	O	3.461158507469	3.276550213040	-2.830238925339
30016	H	3.018699390540	3.699246612869	-2.080656182770
30017	H	3.809708036835	2.433211642047	-2.510137807232
30018	O	-2.069734410503	-3.906972293572	-0.823036277703
30019	H	-1.556122651278	-3.944219849103	-0.004056661054
30020	H	-3.005156671516	-4.039031843878	-0.539136991032
30021	O	1.342913442546	3.095775023897	-4.535722683188
30022	H	2.209493411678	3.221358417189	-4.103883053381
30023	H	1.227990809534	2.133367141909	-4.631447628586
30024	O	-3.204242366908	-2.197071083314	-2.735314398811
30025	H	-2.590105740327	-1.491403990981	-3.032671475711
30026	H	-2.713149032952	-2.773206188128	-2.131455463371
30027	O	-4.864488610316	1.559674882406	-0.865040303837
30028	H	-4.981024507205	0.587277449709	-0.869943618381
30029	H	-4.251509384046	1.758377778520	-0.137372052060
30030	O	-0.002659044169	4.750317661365	3.175914807454
30031	H	0.073743078751	5.128233605868	2.273956145723
30032	H	-0.178076409821	5.479470422003	3.771186604662
30033	O	-1.735055433353	-1.354276701349	-6.064135060619
30034	H	-1.982273627113	-2.261401568202	-5.785565553907
30035	H	-0.811242432807	-1.407483267738	-6.344370933461
30036	O	-0.015954336002	5.801751627992	0.696337982977
30037	H	-0.912516787468	5.429913650967	0.482691307284
30038	H	-0.031222542579	6.715792131428	0.410007439340
30039	O	-2.462094426418	4.922970466325	0.340925744214
30040	H	-3.096422034687	5.210761176455	1.005363801689
30041	H	-2.858008067981	5.237128388235	-0.531529213681
30042	O	0.849632166345	0.421031817209	-4.404821526106
30043	H	-0.062087814124	0.264285175404	-4.064757455384
30044	H	0.896068240331	-0.075555780154	-5.234885612426
30045	O	-0.055473002514	3.343620106662	-2.328654773050
30046	H	0.437071723907	3.259544086275	-3.185958796407
30047	H	0.588390028734	3.722218226862	-1.710219795203
30048	O	1.053027803496	-1.537260987375	-6.388624643433
30049	H	1.534559236927	-1.752911901796	-7.186942019353
30050	H	1.272602348640	-2.219548207860	-5.725123584761
30051	O	-3.441911803083	1.699765092484	-3.226708191396
30052	H	-4.061056280074	1.728734368237	-2.477636416645
30053	H	-3.134248373209	2.608333811271	-3.374510147689
30054	O	-1.304259148701	2.323706295251	3.054036765231
30055	H	-0.935744215251	3.220684263982	3.123097823459
30056	H	-0.548061380298	1.724003240090	3.143302245187
30057	O	-4.604045654069	0.042002085030	2.486336546074
30058	H	-3.874223835694	-0.085969500550	1.854624732206
30059	H	-5.329115395834	0.407188994934	1.977982140865
30060	O	1.711175895312	-3.250142139217	-4.376631795388
30061	H	0.905403860899	-3.472275390425	-3.861906903112
30062	H	2.302415696342	-3.996329898846	-4.254634084143
30063	O	-0.280433363116	-4.275929962092	-2.905803577026
30064	H	0.420355808096	-4.834784744368	-2.507024513098
30065	H	-0.938438570412	-4.163620714330	-2.201056602213
30066	O	0.554447797283	-2.711125632813	3.786912692492
30067	H	1.381627614196	-2.196879654328	3.739089026158
30068	H	-0.075217295012	-2.168324150645	4.289285384006
30069	O	-4.959784832277	4.366658850119	1.882158838552
30070	H	-4.407386849843	3.566234766491	1.734234001741

```
H      -5.616074480818    4.137536686532    2.539333865504
O       1.581142005770   -4.926241129848    1.062020270462
H       1.678960567618   -5.252374835169    1.978807509601
H       0.693047088942   -4.536885730387    1.052808675181
O      -4.639059667971   -1.127844465861   -0.744682932850
H      -3.845196794819   -1.009813692687   -0.190626571116
H      -4.301599000100   -1.464020104754   -1.602647509371
O       4.647051457428    0.711856121576   -2.254938459858
H       5.345619331604    0.724135737655   -2.910993252862
H       3.984459557497    0.067126182193   -2.579242970914
O       2.837697738360   -0.989154758589   -3.300597340761
H       2.143649873143   -0.357262701775   -3.582604217521
H       2.573937590126   -1.813232798635   -3.745921988572
O       3.578630502424    3.185329942503    1.260251853905
H       4.058483105060    2.356600558933    1.134562377436
H       3.152105585625    3.132901419357    2.137874177618

Cope TS Water45-20
155
ANGSTROM
C      -0.286102443096   -1.063684037873   -0.842475951212
C       0.987524941909   -1.541641311911   -0.230175743069
C       1.924443504153   -0.666149062638    0.155735395952
C       1.636446049086    0.828819472428    0.049493843153
C       0.262654603089    1.139659247876    0.784146885929
C      -0.969596432516    0.441924086455    0.175260084838
C      -1.600390318855   -0.690617348678    0.812923879530
O      -2.798560917840   -1.076626724135    0.592593927170
H       1.093859545957   -2.609948691305   -0.075100349943
H      -0.997935885550   -1.842847334829   -1.112882761883
H       2.853174978347   -0.976438754808    0.620373807765
H       2.426019180466    1.423531424176    0.508661972731
H       1.552744035923    1.133823505589   -1.001188363696
H       0.375660175796    0.846424212282    1.831564843653
H       0.080480905872    2.218027160760    0.750548222656
H      -0.139951517652   -0.409144690589   -1.702738758418
H      -1.019258842720   -1.249769952376    1.555294269605
H      -2.583731665364    0.861452610225   -1.020578811579
N      -1.760308422664    1.276751289126   -0.602436245470
H      -1.278673388323    1.871757561860   -1.269824045636
O       1.052119438269    0.152113083943   -3.540089310807
H       1.134536816300    0.341772822182   -4.488537992195
H       1.932055447294   -0.159924614336   -3.213055974460
O      -0.169054268248    4.617058442900    3.178621726234
H      -0.887685206050    4.229292977370    2.670029861180
H       0.612470915717    4.594933069833    2.598416792249
O      -4.109749273916    1.205006484880    1.512515830076
H      -3.658582414820    0.552599733890    0.956414367567
H      -4.200440521737    0.769415557241    2.384090791660
O      -0.265442153819   -0.349700353558    3.651519331223
H      -0.804773076510    0.446646772976    3.814126365505
H       0.388616431785   -0.372253131852    4.376132184810
O      -5.587177073108    2.921822614160   -0.173591954706
H      -5.137566866743    2.346729094638    0.462571906503
H      -6.145328216009    2.329106674325   -0.694703461732
O      -3.440394820340   -3.159881299345    2.191797641771
```

30127	H	-4.357833773814	-3.026993701946	2.463323085943
30128	H	-3.222327536349	-2.356857470489	1.667507896931
30129	O	-0.458950735954	-2.054080979931	-3.969814409852
30130	H	-0.002757942279	-2.776838425628	-3.501643859322
30131	H	0.039146529000	-1.240300347798	-3.745235919071
30132	O	4.654128867628	1.282708680526	-1.300979531029
30133	H	4.648214781233	1.137794796752	-0.347553269083
30134	H	4.236878181637	0.515641733553	-1.726179214774
30135	O	1.882853273072	4.112039808008	1.444646289806
30136	H	1.533793854572	4.149778669739	0.539640514680
30137	H	2.849800473227	4.303700677562	1.357067895510
30138	O	-2.572910675828	3.322069941079	1.950508290047
30139	H	-3.116004361240	2.548337869856	1.661303022243
30140	H	-3.196721251305	4.030241689927	2.126337369182
30141	O	3.372355102716	-0.792602534880	-2.672568948725
30142	H	3.379673806897	-1.652750235262	-2.202939902413
30143	H	3.646901379776	-1.003524901089	-3.584827935505
30144	O	-3.677357278192	3.290166183322	-2.067813998453
30145	H	-4.405904312628	3.435284875345	-1.433966822407
30146	H	-3.645553032797	2.319827083795	-2.152381950732
30147	O	-4.145852231087	-0.264860725158	3.792434166546
30148	H	-3.459582267035	-0.880225312307	4.134520157817
30149	H	-4.863082814911	-0.835836043745	3.480004720838
30150	O	-3.252711575570	-3.525369718810	-0.655106414835
30151	H	-3.597118736303	-3.969546345645	0.127426730452
30152	H	-3.045337095223	-2.622923751609	-0.349785036767
30153	O	0.302185291815	0.830359974679	-6.115250040661
30154	H	0.500520310713	1.722059773327	-6.403263996118
30155	H	-0.663610686218	0.798068855935	-5.953126578234
30156	O	-0.709519728260	-3.742352619434	1.757957043527
30157	H	-0.197695865153	-4.553619533080	1.961829994029
30158	H	-1.610106468886	-3.884831461756	2.068448793395
30159	O	-2.161742294871	-2.044683283694	4.384760535089
30160	H	-2.468888663496	-2.593641347424	3.645088587866
30161	H	-1.440643951718	-1.461339435085	4.042291103425
30162	O	-3.030470994239	-1.135307671441	-4.037496413155
30163	H	-2.161273682169	-1.571407201086	-3.949388140082
30164	H	-2.884121938991	-0.460623538858	-4.723967205392
30165	O	-2.206106652329	3.185927374462	-4.415097289259
30166	H	-1.424735558766	2.976131195262	-3.863305830735
30167	H	-2.890138475276	3.416806077564	-3.769865813052
30168	O	-3.989658335526	0.564723660289	-2.180153597749
30169	H	-4.954086505481	0.602904107397	-2.229141549836
30170	H	-3.670798413960	-0.050947597512	-2.868846784197
30171	O	0.835527270823	4.303127926608	-1.131575007940
30172	H	-0.129905090700	4.347425885453	-0.881645221390
30173	H	1.065909736346	5.181111702950	-1.440905414399
30174	O	-6.725303731401	0.732332917362	-1.555770048760
30175	H	-6.401789961368	0.026346816577	-0.954375071652
30176	H	-7.525490649330	0.407103726738	-1.967354939355
30177	O	0.513922232136	2.732309521878	5.022867226926
30178	H	-0.331323138902	2.270462306726	5.034687924648
30179	H	0.330425987399	3.554513730717	4.526355966478
30180	O	4.609789813250	1.494824829744	1.673218756093
30181	H	5.232942581431	0.979814919660	2.186508353751
30182	H	3.854527422614	1.664071406770	2.265200889126

30183	O	1.339984976298	-0.710868585839	5.843523114209
30184	H	1.096089525266	-0.293784867838	6.669301693010
30185	H	1.010045855324	-1.643843235825	5.865975341059
30186	O	0.805839317994	-5.936661799728	1.493592986892
30187	H	1.630257678184	-5.443993138437	1.321194237587
30188	H	0.458586500273	-6.185424613585	0.632857313609
30189	O	-0.802887694086	-4.732870563924	-0.769270836605
30190	H	-1.705847616890	-4.466378486940	-1.004327310642
30191	H	-0.644880073710	-4.292702362939	0.092854287557
30192	O	-1.699476870416	4.356426008479	-0.514505458279
30193	H	-1.916645073157	3.927640690266	0.326798123142
30194	H	-2.350441513660	4.013860833201	-1.159888170744
30195	O	1.628557127816	-2.807259801116	2.970467772739
30196	H	2.121770027981	-1.967564181085	3.148327475268
30197	H	0.806943204203	-2.569988024288	2.524085094489
30198	O	-2.404532744557	0.949807112049	-5.721949767243
30199	H	-2.915102712193	1.133163112649	-6.510806164738
30200	H	-2.386619140991	1.803692518980	-5.195606886639
30201	O	2.903036539467	-4.189370598535	1.102501589004
30202	H	3.751744404140	-4.448097964050	1.463440988172
30203	H	2.469684090276	-3.634113187331	1.801869691853
30204	O	2.957145293997	-0.614821022282	3.603421196221
30205	H	2.702533478384	-0.626360374497	4.536385825371
30206	H	2.647831725181	0.248808572863	3.279620451252
30207	O	-2.002514173785	1.676630108781	4.150360747153
30208	H	-2.115258930852	2.327608480323	3.438052927678
30209	H	-2.838673213182	1.205507805192	4.240001084654
30210	O	2.283028796229	1.983404833350	3.112780784534
30211	H	1.775868983200	2.182841918244	3.926078631246
30212	H	2.084043714991	2.728797711238	2.515894111091
30213	O	4.461904859871	4.247678095059	0.903687121730
30214	H	4.749786763914	3.355184848305	1.142476538807
30215	H	4.352662461895	4.232272649463	-0.069153980547
30216	O	-5.496630528412	-1.261824192540	-0.244090693153
30217	H	-5.438692504257	-1.784704278350	-1.079930394872
30218	H	-4.585711353474	-0.970192457502	-0.085829135885
30219	O	3.295340642168	-3.284349103846	-1.603542904119
30220	H	2.469976020970	-3.569723258642	-2.027513466495
30221	H	3.215471234555	-3.535014837938	-0.672220955670
30222	O	3.698852813162	3.778079506775	-1.661729959936
30223	H	4.094179635842	2.881748881555	-1.659809104278
30224	H	2.755210366508	3.661867368763	-1.517770516548
30225	O	-4.991648371942	-2.533516076630	-2.540101783893
30226	H	-4.389609929798	-3.149509305457	-2.095043740040
30227	H	-4.441907228059	-2.070992590046	-3.191033963376
30228	O	0.306882533706	-3.111251292650	5.425163112933
30229	H	-0.615605629365	-2.915122693800	5.199788161407
30230	H	0.784955365839	-3.223240233673	4.583904639401
30231	O	0.853183540859	-4.084580985004	-2.703354447729
30232	H	0.940025849995	-4.851835279860	-3.269883219801
30233	H	0.254374809400	-4.344614675662	-1.958120213796
30234	O	-5.927770872679	-1.952247151812	2.355426685215
30235	H	-6.862619514112	-2.026336263146	2.545824228162
30236	H	-5.850891569556	-1.700005641791	1.410852368118
30237	O	1.044521343102	-1.871238910693	-6.275673527872
30238	H	0.829999349163	-0.957890493496	-6.507680661897

```
H      0.427613393689   -2.095806607838   -5.554922122286
O     -0.227399495187    2.399712690772   -2.783801001942
H      0.353598844335    3.043943291583   -2.350946619201
H      0.306379702887    1.629762885979   -3.056820751725
O      3.525149257842   -1.523073732728   -5.276370306979
H      2.633950254756   -1.688266729233   -5.659275801060
H      4.099552036180   -2.201527394188   -5.629450619472

Cope TS Water45-21
155
ANGSTROM
C     -0.203136041203   -1.165025344595   -0.740936514840
C      1.120011846562   -1.478162627645   -0.142827939201
C      2.003072337668   -0.513838637421    0.151218202986
C      1.611409924418    0.951777020129   -0.002101501496
C      0.253628438953    1.192573938534    0.784069912799
C     -0.956870432325    0.483933694823    0.155597885068
C     -1.616018639711   -0.592699657061    0.849651314198
O     -2.782757554563   -1.034510953931    0.618532191270
H      1.329877222847   -2.519679769337    0.078919507317
H     -0.867083876925   -2.017409309147   -0.868621673311
H      2.965138316174   -0.734482500154    0.595039553630
H      2.379938748870    1.616485048785    0.393577861138
H      1.444067380262    1.211274114453   -1.055313465052
H      0.386531435330    0.862924480621    1.818993264525
H      0.040181140984    2.265057036324    0.797531712277
H     -0.157030435807   -0.599760341379   -1.670469266354
H     -1.057456764924   -1.051985439098    1.677076404205
H     -2.481304391947    0.898961585862   -1.203960858477
N     -1.647970147607    1.256249625336   -0.755244997872
H     -1.128885228673    1.897282144356   -1.335049323684
O      3.559496788186    3.335808702634   -1.452309641735
H      2.660337201558    3.621596195094   -1.200554483417
H      3.984964267876    3.085437852259   -0.617190724314
O     -3.498338893970    0.056750725832    3.335548282987
H     -2.904144440897    0.697478757954    2.918920083694
H     -2.924290544122   -0.599709435288    3.769923600769
O     -1.830961987171    4.433539504991    3.214331537080
H     -2.088573380995    3.576440129990    2.796870664977
H     -1.031674821152    4.700114377163    2.736954038162
O      1.946987959355   -4.733389210107    1.037974308849
H      1.417697649391   -5.165896228630    1.723785428030
H      2.592088665707   -4.172878015077    1.491202777426
O     -2.825349326727   -1.348423163988   -3.509877934385
H     -2.169532024323   -1.998597190280   -3.845602394378
H     -3.205274854215   -1.790060574503   -2.731000525239
O     -4.932099223968    2.256442780939    4.118340546296
H     -5.312945330118    2.489599529355    3.265619789467
H     -4.560711608047    1.367149464243    3.991301679420
O      1.364047662564    0.080166876999   -3.423716081155
H      1.468652272238   -0.893736230632   -3.398882120072
H      0.459606404421    0.247079358390   -3.740109379333
O      4.138208803964   -4.731749403427   -1.906899700206
H      4.328864217272   -4.301469656922   -2.744637484702
H      3.181912783109   -4.874775422614   -1.878899164103
O     -2.525612216964    2.875758672657    5.368383627200
```

30295	H	-2.275601722889	3.653532713505	4.846538590166
30296	H	-3.445641608243	2.693532724642	5.111481725146
30297	O	-0.884755757306	-3.124447722123	-4.013074175240
30298	H	-1.034887273336	-3.741525235059	-3.285499389191
30299	H	0.056157256502	-2.900337923214	-3.974665407852
30300	O	1.768629460744	-2.558833327433	-3.306230880203
30301	H	2.737402668773	-2.414485004461	-3.326394256602
30302	H	1.622793581673	-3.229231825169	-2.610073680802
30303	O	1.135291089595	3.182616402660	3.753920040797
30304	H	0.976740353456	3.801265602781	3.018247960029
30305	H	1.072066977854	3.697821677383	4.559698551051
30306	O	4.600879392140	2.202854255990	0.944847317954
30307	H	5.408141555492	2.609894236771	1.261808105678
30308	H	4.885954351339	1.369717670186	0.486459075449
30309	O	-2.636885506347	2.328347343474	1.796157012085
30310	H	-3.605399601496	2.420182816982	1.728692781150
30311	H	-2.300989384237	2.766095082327	0.993256515729
30312	O	3.907587997082	0.910078065835	-2.634097372853
30313	H	3.848708159014	1.828200252536	-2.298970738518
30314	H	2.987759392192	0.622898412023	-2.740255441923
30315	O	-1.189827551017	0.623865048908	-4.212775967491
30316	H	-1.829347680364	-0.042303467020	-3.873508251344
30317	H	-1.330158152346	0.672381376835	-5.159096194105
30318	O	-1.548521572743	-4.371546285931	-1.489652417276
30319	H	-1.501706438934	-4.552498449617	-0.531341388276
30320	H	-2.391126927327	-3.906173722496	-1.596594084000
30321	O	-5.747037800251	-1.210585600945	2.306305832943
30322	H	-5.081390207389	-0.684863528452	2.784053958693
30323	H	-5.373476341781	-2.101258018155	2.283228261583
30324	O	0.509561998776	4.894039332102	1.738559503559
30325	H	0.364166082552	5.802240805979	1.455267590830
30326	H	0.679847403116	4.414450610943	0.905274109040
30327	O	3.650513156663	-2.950919091413	2.371052249731
30328	H	4.392270033568	-3.108832835829	2.955780751245
30329	H	3.104138357101	-2.254967621923	2.791040602489
30330	O	-5.123536304013	0.196096296918	0.137650620146
30331	H	-4.197815425300	-0.078520896506	0.299828873240
30332	H	-5.617245526989	-0.317591650925	0.813179083258
30333	O	4.355910017317	-1.898238958103	-3.002825356113
30334	H	4.497005058515	-2.074789286649	-2.059743251662
30335	H	4.418333334804	-0.941346629495	-3.101915427946
30336	O	2.065393164693	-1.096630675690	3.526699435907
30337	H	2.473287593384	-0.222973864076	3.334449666112
30338	H	1.548030926784	-0.967778376470	4.344118999137
30339	O	-0.397975383381	2.927989295750	-2.925315964188
30340	H	-0.644859348279	2.130404951305	-3.419844079385
30341	H	0.385814563677	3.295079236317	-3.400303851271
30342	O	-3.069029537773	3.603857174700	-2.446819528044
30343	H	-2.169542811868	3.476853722174	-2.789005960848
30344	H	-3.490230716424	2.731773350024	-2.467675059138
30345	O	1.902716258689	3.730273267063	-3.986946834074
30346	H	2.200676264975	3.015620066850	-4.588503890952
30347	H	2.588563601281	3.814869995480	-3.316752160043
30348	O	-1.908695404083	-1.976078061674	4.303338127432
30349	H	-1.204829502018	-2.263956056561	3.686019027874
30350	H	-1.420624061337	-1.609488101294	5.050247766078

```
O      0.995952305104    4.074510063590   -0.827230822171
H      0.447586498413    3.570456013059   -1.450582948850
H      0.713954852380    4.997348509305   -0.951195768825
O      2.759787328465    1.514253729154   -5.282909490349
H      3.673154770206    1.389990985089   -5.012216444022
H      2.238814153901    0.910177778923   -4.717616691294
O      0.143928560858   -5.668648036121    2.948177726932
H     -0.587850603844   -5.414598812197    2.346426788782
H      0.007930268720   -6.586145020565    3.182657014255
O      4.631279685613   -2.615019666281   -0.265741394721
H      4.556694453886   -3.489383960386   -0.697388468208
H      4.253777433121   -2.730630706550    0.618336777556
O      3.149425048295    1.315186395974    3.172974594707
H      3.627355447227    1.618279044508    2.382931790469
H      2.483032903744    1.989728492635    3.369642015318
O     -5.284446921516    2.626695357133    1.213476454595
H     -5.307322525507    1.720844155708    0.816465876561
H     -5.457966800451    3.230536261023    0.489068486589
O     -3.752078871812   -2.631860248253   -1.270064853966
H     -4.652225613802   -2.850991929002   -1.027166063426
H     -3.409627028647   -2.050692936765   -0.558137202547
O     -4.085863180458    0.964807521923   -2.330489852926
H     -4.682624512913    0.671059626384   -1.626565113979
H     -3.921480171671    0.210487318876   -2.907495158154
O      5.367101632050    0.100072288209   -0.442742700585
H      4.917092198127    0.323924867851   -1.278730982554
H      5.214286055836   -0.844720021495   -0.295738188430
O      0.246965902105   -0.589336166934    5.516533854912
H     -0.190585004904    0.110882574096    4.952985145539
H      0.444531286059   -0.172915105576    6.355036204191
O     -3.577960753088   -3.026957480530    2.253187454237
H     -3.246753005475   -2.822370061750    3.137244724252
H     -3.325243022431   -2.252759730406    1.701329549419
O      1.306477327960   -4.604430901554   -1.582263691641
H      0.348606594863   -4.714304315988   -1.638366805370
H      1.527472307184   -4.675120356498   -0.633351280398
O     -1.522426608236   -4.411445521686    1.232128017363
H     -0.885798754797   -3.704460216489    1.425009589400
H     -2.377329093750   -4.078008159524    1.585250298460
O     -0.865530912232    1.259364029842    4.051980407400
H     -0.170075147583    1.904386589548    3.848808174306
H     -1.532094484573    1.760202421065    4.581360675405
O     -3.955539701231    5.332194588957    1.794163776644
H     -3.240974595143    5.210693112214    2.452900576780
H     -4.667516430973    4.753552557566    2.076954096354
O     -0.502385478797    6.305718351441   -0.509746652433
H     -0.911351009980    6.901432078426   -1.136614791857
H     -1.171555669924    5.601138109936   -0.318131710140
O      0.133511025012   -2.781835583818    2.708474697345
H      0.854481418113   -2.139241890073    2.855735854421
H      0.421590303414   -3.613871732579    3.101207449091
O     -2.230647144980    4.327995889724   -0.035568992779
H     -2.931555809645    4.765676950796    0.488584536945
H     -2.619749143042    4.094776494586   -0.908868611692

Cope TS Water45-22
```

```
155
ANGSTROM
C     -0.203558671765   -1.028304749292   -0.714029774355
C      1.057769092585   -1.464059355565   -0.043870789520
C      1.950152738198   -0.559716515228    0.375326323291
C      1.659615660039    0.929853870585    0.197931074919
C      0.223888206637    1.287133011174    0.772048491385
C     -0.955356564705    0.499219663685    0.154843613000
C     -1.573050721305   -0.574051415587    0.899823099061
O     -2.737392983747   -1.053976555324    0.688792748342
H      1.186303902544   -2.529961137664    0.112625070398
H     -0.894041260828   -1.833145964838   -0.960738988842
H      2.862841288249   -0.840207132781    0.886701536955
H      2.404993651710    1.542661902574    0.707791354440
H      1.678490011569    1.195828935269   -0.866752145459
H      0.229795327926    1.122401532405    1.853642902243
H      0.034332028883    2.353666142346    0.609508781245
H     -0.025901749355   -0.429415637063   -1.606059687479
H     -0.991023685305   -1.028529620758    1.713872446147
H     -2.534587449892    0.835238822645   -1.150411130172
N     -1.730719562193    1.257612638771   -0.713102062113
H     -1.244980124878    1.894811731433   -1.333254912081
O     -6.357691359232   -1.725843096098    1.484196165724
H     -7.277981938567   -1.901737356690    1.677665692188
H     -6.053564845868   -2.496325854519    0.916723055242
O     -2.232735148876    1.580464841057    5.626853945489
H     -3.075252626624    1.290650709217    5.222035003771
H     -2.448615030574    2.086088125641    6.410409239284
O      2.212740221853   -4.727407410359   -1.439535815278
H      1.542450572957   -4.271921891243   -1.974648658369
H      1.736850784479   -5.153586167456   -0.718854622233
O     -2.947673055549   -3.753060652716    0.963154737960
H     -2.396865992477   -3.999405517770    1.744834698852
H     -2.884032090350   -2.785116462985    0.878914983424
O     -4.540893442766    1.057023875426   -2.166211376455
H     -4.248945546671    1.989551163744   -2.208423857604
H     -4.935484993712    0.956451116791   -1.285641273715
O     -3.492572037985    4.443399742184    0.662347871153
H     -3.958959606804    5.126368330555    1.143958112153
H     -3.539903103236    3.628134349811    1.202790812587
O     -0.761725533887    4.493461192223    0.014994021602
H     -1.659890757988    4.584972256064    0.360631391587
H     -0.862420509498    4.127667894020   -0.874247237764
O      0.538709838261   -2.264449650246    2.865942737825
H      0.719075816076   -2.391508989352    3.816537256315
H      1.276110128275   -2.725848422086    2.416555852258
O     -3.686380434063    2.185085703449    2.113359775761
H     -4.154666205571    1.620875653206    1.465953766096
H     -4.039580017134    1.927699805574    2.976208240697
O     -5.528368972818   -3.792298231550    0.155059213095
H     -4.680228439541   -3.982579000409    0.594089028724
H     -5.293667431413   -3.780534757378   -0.793585504418
O      2.643391203887   -3.674684844895    2.018089081633
H      3.116682383362   -3.277895640615    2.770212593929
H      3.197121680030   -3.511011896899    1.223290481088
O     -4.157095971811   -1.393104908538    3.036035956262
```

30463	H	-3.566400940799	-1.277471348317	2.270220120222
30464	H	-5.017313212399	-1.624025042981	2.634885801466
30465	O	-1.415450279985	-4.734691858361	-0.982648017681
30466	H	-0.704059450422	-5.095741151725	-0.432077514236
30467	H	-2.057345684789	-4.378964626297	-0.327730660997
30468	O	3.466213193391	-2.082766642560	4.112144063008
30469	H	2.985493676189	-1.233559966029	4.062863074777
30470	H	3.046249810603	-2.549844827400	4.841622189860
30471	O	4.103277057810	-3.238545337372	-0.179323395058
30472	H	3.557740969653	-3.789325843958	-0.774754853953
30473	H	4.190838980998	-2.373577254193	-0.597881787743
30474	O	0.547857952146	-5.223205814616	0.982055048176
30475	H	-0.114700936324	-5.224581060261	1.683773709076
30476	H	1.331931413210	-4.811438667482	1.378696137048
30477	O	0.471683665210	3.810448344235	-5.574768427455
30478	H	-0.381113023844	3.379748479343	-5.387156513562
30479	H	0.339356981561	4.343693621716	-6.358842708213
30480	O	1.852066438975	5.081824105428	-0.443529674415
30481	H	0.920977533253	5.006846772708	-0.171106682118
30482	H	1.892108390181	4.702663378536	-1.336652836994
30483	O	1.764299994412	3.832908932646	-2.950604589723
30484	H	2.469889694947	3.149057074757	-3.004434376412
30485	H	1.594415674827	4.123376125273	-3.852650095433
30486	O	4.810775824631	2.388889821565	-0.519173066555
30487	H	5.691796593455	2.735713581851	-0.375520472716
30488	H	4.221002386775	2.818780553268	0.136670618194
30489	O	1.737601760209	0.040020558742	3.888335886071
30490	H	1.823633855556	1.010134779846	3.731473211242
30491	H	1.366770767141	-0.362167895707	3.091190387834
30492	O	2.034285834130	1.544965392518	-5.440187945547
30493	H	2.923883147066	1.727810242811	-5.121279609507
30494	H	1.597562687105	2.399594377160	-5.592524724664
30495	O	4.920081704479	-0.433381236676	-0.632238493253
30496	H	5.234126131294	-0.705104680282	0.243392953588
30497	H	4.814626291324	0.529204289303	-0.586932757959
30498	O	-3.174543399694	-1.250506624356	-2.186490906076
30499	H	-3.775619160731	-0.494860629010	-2.355195977461
30500	H	-3.076904259305	-1.283863159418	-1.223010048670
30501	O	0.079555667927	-3.503489388881	-2.773020325686
30502	H	-0.117464056629	-3.884643898403	-3.629514828017
30503	H	-0.549211215491	-3.914899164213	-2.131907632683
30504	O	0.863604243569	0.904584320407	-3.058129400010
30505	H	1.364136375896	1.194733027919	-3.842347723199
30506	H	-0.037314970113	0.730383166975	-3.384808424900
30507	O	4.463959893286	-0.423680527889	-3.374724123830
30508	H	3.665484731098	-0.964634911869	-3.474936631271
30509	H	4.768099187841	-0.566186308050	-2.466244143801
30510	O	-3.603344512871	3.604342961479	-2.018622576598
30511	H	-3.705853268852	3.917522057296	-1.107473062735
30512	H	-2.649230122753	3.585467625672	-2.165477349864
30513	O	-1.908852228742	2.524197039504	-4.881087467320
30514	H	-1.831309957422	1.562768142708	-4.689062980720
30515	H	-2.831149686485	2.750129136895	-4.742573469127
30516	O	1.943215792981	-1.541881158210	-3.402317910381
30517	H	1.608748582338	-0.672411311376	-3.092607228549
30518	H	1.378606560083	-2.220465246521	-3.009678044866

```
O      3.826527258608    2.153559915449   -3.160478333400
H      4.204519843886    2.328118054142   -2.286405562962
H      4.029276697966    1.198849755482   -3.312466060765
O     -0.445813235086   -0.552702785985    5.414014220436
H     -0.987895868196    0.230956277867    5.580344591829
H      0.329647341926   -0.243119486749    4.901047149858
O      0.975088341909   -2.891499784874    5.528030305182
H      0.529666381759   -2.081305301659    5.821572982143
H      0.350602975675   -3.605632963956    5.672129301322
O      3.243440814654    3.712215284724    1.264835963841
H      3.719541143827    4.375589527974    1.766670203925
H      2.679752652576    4.222825584586    0.610649619402
O     -0.817120632596    3.297184305556   -2.499218588559
H     -1.129390334267    3.048397527857   -3.386719780747
H      0.155777673919    3.394115839218   -2.584055819863
O      5.324720903497   -1.606878714223    1.894360328823
H      4.855392658395   -1.697135237435    2.732675074878
H      5.238273855534   -2.450320778579    1.436003374972
O     -2.319197179093   -2.568639019739    4.879301671458
H     -1.679387633004   -1.853059620647    5.038329391852
H     -3.026015458387   -2.184663411190    4.343215812904
O     -4.967906266449    0.424973303769    0.484589392191
H     -4.145372565481   -0.103819547931    0.452020590067
H     -5.642583432844   -0.198726306899    0.799096628075
O     -4.537682726172    0.825789428117    4.404932679013
H     -4.362613026541   -0.016662948183    3.907616330571
H     -5.348603009352    0.684002795039    4.893892244300
O      1.713222684273    2.654564561873    3.414006766058
H      2.163260982606    2.999540870084    2.627493472247
H      0.769130187684    2.853580999798    3.314705043605
O     -1.478573455709    0.014750845796   -4.106087901491
H     -2.010343190649   -0.607996493180   -3.590215195500
H     -0.908091618548   -0.489627436043   -4.733948623201
O     -1.293087176588   -4.188741918084    2.972564634806
H     -0.627981807161   -3.476431720249    2.819297530502
H     -1.769386173714   -3.872689012938    3.762755482760
O     -4.210715359365   -3.802332630790   -2.231994638591
H     -3.865248203800   -2.900761938780   -2.359025592650
H     -3.447952640465   -4.359646736891   -2.052622282697
O      0.486899308965   -0.944157932193   -5.618521013907
H      1.085329631060   -1.335688648405   -4.949898761654
H      0.949406790991   -0.147267714368   -5.904698053828
O     -1.092953948712    2.687555071894    3.302266923512
H     -1.387943191246    2.359289021335    4.162759496550
H     -1.842357522959    2.579471972321    2.704086366560

Cope TS Water45-23
155
ANGSTROM
C     -0.222557661421   -1.134059696006   -0.798888474976
C      1.091636811146   -1.476761425503   -0.187950664001
C      1.979543682957   -0.528598962181    0.139235527685
C      1.597083788120    0.940837581594    0.003714659456
C      0.203505938503    1.189015426677    0.717299050814
C     -0.993806185933    0.442495454541    0.098652416217
C     -1.628433533952   -0.634211843932    0.821074080591
```

30575	O	-2.801179604810	-1.084304171234	0.608153381748
30576	H	1.281093101719	-2.527587748692	0.009102483943
30577	H	-0.884039945507	-1.983780775512	-0.948268701814
30578	H	2.941613062043	-0.765020586919	0.576891205338
30579	H	2.345585239311	1.591186008112	0.457438864413
30580	H	1.500272460874	1.225870370947	-1.048945453531
30581	H	0.312327216351	0.909605726799	1.769272934451
30582	H	-0.027363662177	2.255176352916	0.671387931000
30583	H	-0.152833162812	-0.564052564680	-1.726045672602
30584	H	-1.051494390219	-1.102417454584	1.629185126084
30585	H	-2.551969874888	0.782930971089	-1.216080254586
30586	N	-1.749942284809	1.203253658723	-0.769434165714
30587	H	-1.282047085179	1.876212304207	-1.366245486895
30588	O	-3.159812672151	3.443345449810	-2.315064923871
30589	H	-3.550158931854	4.096595040389	-2.895895798553
30590	H	-3.646470277534	3.489043102509	-1.451473927385
30591	O	-0.366986924633	-1.825397216969	3.751467950889
30592	H	-1.019812520955	-2.199787264087	4.360483294813
30593	H	-0.424652986110	-0.856570202642	3.908551335764
30594	O	-4.340789391566	3.393741134700	0.038738760740
30595	H	-3.617739101592	3.621274295596	0.640180393162
30596	H	-4.544722564410	2.452043902787	0.205997954676
30597	O	4.852809350660	-3.114022112615	2.597261245230
30598	H	4.802791882772	-3.434477180563	1.678890637382
30599	H	3.961014910694	-2.816522047205	2.832865611010
30600	O	-0.109981583736	2.812970568956	-2.481176126038
30601	H	-0.360567600211	3.353466688009	-3.255783004963
30602	H	0.307645164241	2.019322790516	-2.856528300432
30603	O	-1.424777599619	-3.308762206900	-3.200752291081
30604	H	-2.167354006197	-2.765541431042	-2.880743552372
30605	H	-1.125581405712	-3.835186099073	-2.441298266417
30606	O	1.335266128757	4.168453450116	-0.645712772355
30607	H	0.877218306323	3.599826908857	-1.297822244870
30608	H	2.283602572022	3.998212682646	-0.807753299466
30609	O	4.999763615117	-3.676049462590	-0.102587841216
30610	H	5.852603510209	-3.959896383994	-0.432541337042
30611	H	4.836164869175	-2.782514833635	-0.490531278471
30612	O	-3.026114207375	0.239742056273	5.606457391278
30613	H	-2.165193210590	0.567158961950	5.302862560523
30614	H	-2.944181991551	-0.725527213306	5.582796199420
30615	O	3.175081907021	0.624365301927	2.901757667382
30616	H	3.823993080223	0.460823647052	3.621285195408
30617	H	2.528489086800	1.270204305419	3.242085944564
30618	O	0.700983846590	-1.966030355131	-3.960898751816
30619	H	1.442429428606	-2.384566214325	-3.473470855945
30620	H	-0.118258927158	-2.461260783676	-3.703945367785
30621	O	4.746257653939	2.051249996234	1.274850285494
30622	H	4.137147358532	1.489698786047	1.814165927234
30623	H	5.179598317196	2.649816763561	1.883920746706
30624	O	2.325939901076	-2.012967486562	3.104289418043
30625	H	2.551184337708	-1.087338854465	2.899204190979
30626	H	1.413149135800	-1.995213704832	3.436522858981
30627	O	2.523287590069	-4.819269238846	-0.679799047848
30628	H	2.209897187332	-4.743672827694	0.250276799306
30629	H	3.482754693229	-4.742502129941	-0.581347590876
30630	O	1.121820969016	0.636395403940	-3.541484697970

30631	H	0.940852418878	-0.310716488058	-3.716213753430
30632	H	2.048675922595	0.694327517357	-3.225422051733
30633	O	-2.752148526969	-2.498458102653	4.986408668677
30634	H	-3.170528079191	-3.214813675528	5.463672779544
30635	H	-3.270303017743	-2.362623932793	4.161260476840
30636	O	6.361573455188	0.068711953719	0.287095720990
30637	H	6.507614395793	-0.388927774233	1.132717284317
30638	H	5.839992952915	0.858399980077	0.517726096372
30639	O	-4.856492758989	0.776104530703	0.536559192411
30640	H	-4.084369386524	0.181850924496	0.518619609580
30641	H	-5.137166864479	0.809388920622	1.457521013418
30642	O	6.586955476224	-1.193883244305	2.740428405388
30643	H	5.926155729637	-1.944018637811	2.664080930251
30644	H	7.426793592803	-1.596323988659	2.962431165793
30645	O	-4.646500642549	0.581951080832	3.471751407997
30646	H	-4.204070052621	0.603467867737	4.348552043520
30647	H	-4.705357647591	-0.361579601184	3.266678710157
30648	O	4.739093253491	-1.279170628226	-1.264159921344
30649	H	5.176752301423	-1.555833829989	-2.103192151493
30650	H	5.401318111736	-0.769167248144	-0.729560959950
30651	O	-3.346926629034	-3.828016824722	0.629642056345
30652	H	-3.204575151492	-2.894931491898	0.395911398151
30653	H	-3.233008097499	-4.327197506054	-0.181506775336
30654	O	-4.231485866601	0.876316977926	-2.394134565985
30655	H	-4.810343210172	0.970762064035	-1.630174577455
30656	H	-3.841466901156	1.754316691245	-2.553773283806
30657	O	-3.357467843756	-1.726386454523	-2.040313261329
30658	H	-3.740387053579	-0.947212738866	-2.468388558920
30659	H	-3.242865784759	-1.460585754389	-1.114708279312
30660	O	1.442435839032	2.523422490281	3.802393596442
30661	H	1.734773238340	3.073673190285	4.529738783114
30662	H	1.175010351525	3.151876381619	3.076887995569
30663	O	5.292231235488	-2.215047779731	-3.679334709108
30664	H	5.130834357646	-1.454935856612	-4.264932732501
30665	H	4.454228756751	-2.697391268218	-3.634914353915
30666	O	-2.400097809431	1.597918626658	2.358438963563
30667	H	-2.379211067776	2.540300007862	2.152590769139
30668	H	-3.285402446577	1.375861464563	2.712395670565
30669	O	-0.430912644616	-4.508922171862	-0.893980955588
30670	H	-0.533713612438	-4.343651661850	0.055431198249
30671	H	0.467627130233	-4.834982268383	-1.010037042055
30672	O	-2.108200438879	4.320652843082	1.537120854818
30673	H	-1.833850780779	4.600725332282	0.609322063012
30674	H	-2.456095069700	5.109201881717	1.955827649721
30675	O	4.690857127226	0.114258582205	-5.035825894023
30676	H	3.942707128098	0.122541794201	-5.697594700857
30677	H	5.405479897129	0.601577503265	-5.446048276721
30678	O	2.760017130142	0.217147534782	-6.787147747758
30679	H	2.220380330079	-0.588089287199	-6.826028949915
30680	H	2.130692603165	0.937711318203	-6.585247852200
30681	O	-0.679977825649	0.804519694810	4.202993953198
30682	H	-1.291916979862	1.107825462495	3.490812194307
30683	H	0.085379616747	1.401220359049	4.161911746108
30684	O	5.059219088552	-0.045040817252	4.669586411550
30685	H	5.707586903204	-0.354790932631	4.004274257960
30686	H	4.756426923457	-0.831389391547	5.128424587652

```
O       3.634662534773    0.824616274545   -2.642038939520
H       4.096688955587    0.671487843976   -3.488300883510
H       3.941126109535    0.120428356546   -2.039489110898
O      -1.376993636408    5.034858944858   -0.838795462853
H      -0.415017595456    4.949201588192   -0.884040010741
H      -1.748852661998    4.401040517283   -1.478913198435
O       1.783623835288   -4.467997962982    1.855863515396
H       0.822924279238   -4.505533189264    1.954384955136
H       2.051748472446   -3.628361600278    2.259433239314
O       0.684068797179    4.269491359795    2.005846950934
H      -0.283726262591    4.267043227668    1.941805185605
H       1.012717929810    4.263834454142    1.083299571610
O      -0.902140242337   -3.907671198770    1.863182476462
H      -1.848263997862   -3.958750133660    1.641474103531
H      -0.782287378905   -3.158805565414    2.466557726995
O      -4.148601938762   -2.091628705105    2.720654910554
H      -3.608733339498   -1.674281895047    2.014224071418
H      -4.465107713303   -2.910355698766    2.327664683223
O       0.997570279087   -1.933402281702   -6.568473997887
H       0.973758386743   -2.830760819171   -6.899744475161
H       0.920082709789   -1.989200654002   -5.581002722659
O       3.838361032225    3.222077275999   -1.162607681222
H       3.761725734075    2.459452519273   -1.757944448195
H       4.214226436692    2.872386737172   -0.343103876016
O       0.750269775957    1.853989986905   -5.899355271876
H       0.909479259430    1.455948163316   -5.014558421420
H       0.026535309769    1.317185555971   -6.280310394520
O       2.765079622044   -2.905722452380   -2.567047106538
H       3.099109421310   -2.212357789731   -1.983889642174
H       2.568393820692   -3.661249695999   -1.976025699275
O      -1.027408821646   -0.060124515145   -6.605536723884
H      -1.589443688746   -0.350804207222   -5.884721366160
H      -0.363504582263   -0.759795186354   -6.739971199199
O      -0.386074229110    4.076771454100   -4.852368860933
H       0.189203565830    4.824957503103   -5.013759238471
H      -0.005555669702    3.323457546810   -5.355450555183

Cope TS Water45-24
155
ANGSTROM
C      -0.236763704834   -1.167713687063   -0.844377132553
C       1.049547473403   -1.529834828962   -0.202014950465
C       1.936318235270   -0.605349045912    0.192528743617
C       1.617290898902    0.878869773337    0.082933229336
C       0.188828885583    1.194772472422    0.712147586957
C      -1.009155964500    0.452579944615    0.113880253680
C      -1.601444139520   -0.698086327921    0.749549371745
O      -2.787617662195   -1.119497410730    0.518285405102
H       1.224840660326   -2.584364313129   -0.019523914090
H      -0.906454668774   -1.997055992823   -1.058009830732
H       2.868493371096   -0.884589363401    0.668652853806
H       2.360476766900    1.474984412832    0.618387398054
H       1.602428488228    1.204123660112   -0.963368223412
H       0.243565425557    0.974136171055    1.780764549169
H      -0.002246252158    2.264631962126    0.588263259816
H      -0.144425737494   -0.528282196239   -1.721303107618
```

30743	H	-1.042126845564	-1.188568543332	1.557522651904
30744	H	-2.600044515146	0.769520567906	-1.143099986274
30745	N	-1.791779626388	1.207074031206	-0.730500530672
30746	H	-1.359245570435	1.889782701485	-1.338008953255
30747	O	-1.449055881219	-1.719809085782	-5.355536181645
30748	H	-2.080791258617	-1.720371286615	-6.074972355672
30749	H	-1.707238341650	-0.985776971438	-4.739139446977
30750	O	3.527722322539	6.290028176610	-0.290653720750
30751	H	3.769420167152	7.094759195632	-0.748941563841
30752	H	2.558256160891	6.297655409366	-0.210280914795
30753	O	4.859434355164	1.515380693699	-1.181276999251
30754	H	4.258334872631	2.266846743870	-1.325406890489
30755	H	4.424072374377	0.728083291535	-1.526932073301
30756	O	0.787719670386	4.066186186992	-2.309027101013
30757	H	1.722069547602	3.981348765223	-2.046107785527
30758	H	0.553209765942	3.151541564074	-2.564740644891
30759	O	-2.151173969503	-0.718360959992	4.269633168950
30760	H	-2.929037124852	-1.124393358058	3.860429328004
30761	H	-1.543935831557	-1.473124275640	4.415460309995
30762	O	-4.029211640696	4.761368532687	1.210601698660
30763	H	-3.094908320336	5.007350472854	1.137485710194
30764	H	-4.249798450966	4.264137822858	0.404648579258
30765	O	1.778864033212	-1.259793393561	5.790692500953
30766	H	2.414032278253	-0.550168235958	5.968271696523
30767	H	2.322209187418	-2.024157201289	5.484968479930
30768	O	3.135247459265	-3.226737828869	4.586917109643
30769	H	2.444626948387	-3.367869916496	3.915556720809
30770	H	3.906191048833	-2.949846719867	4.075315984764
30771	O	3.143061098231	-4.037739227594	1.008662376304
30772	H	3.644709597609	-4.831983755763	1.192845170937
30773	H	3.578665969856	-3.324233782087	1.528831080799
30774	O	3.187653027653	3.673439492410	-1.076919005096
30775	H	2.749921276376	3.499978184681	-0.226285855739
30776	H	3.591746394288	4.547276469798	-0.964701486752
30777	O	3.155622270637	-3.509720659780	-1.656421210205
30778	H	3.176663904147	-3.700346859713	-0.696339710914
30779	H	2.274215670583	-3.760693634763	-1.953760809418
30780	O	4.549837415006	-2.206054501575	2.382838028744
30781	H	4.215015822045	-1.332531067519	2.672363275435
30782	H	5.166566453786	-1.983476427286	1.658431729642
30783	O	-2.078263976684	-4.269131642069	2.780089073479
30784	H	-2.796918464578	-3.619137247828	2.699157400106
30785	H	-1.628691490624	-4.036496683257	3.602750356775
30786	O	1.818709709150	3.922240744648	1.338513313128
30787	H	1.131267034016	3.694853945702	1.989666709568
30788	H	1.473911394240	4.702703487867	0.862900066103
30789	O	0.235002552765	1.479245178621	-2.867257399508
30790	H	0.989369828003	0.962195839423	-3.214451115840
30791	H	-0.557244370659	1.126698659688	-3.296247145000
30792	O	0.966747158548	-2.511154845724	-4.098000851123
30793	H	0.159924998140	-2.433898456387	-4.618976568200
30794	H	0.760175299749	-3.095390713886	-3.352467811706
30795	O	4.435951248763	4.383089902611	1.871792623443
30796	H	3.478296429649	4.197075137106	1.849806162697
30797	H	4.560441378672	5.107718393261	1.252394979118
30798	O	1.106880150223	-2.979940388615	2.786361520437

30799	H	1.147407220441	-2.024047229399	2.606520949942
30800	H	0.811123238570	-3.434663373319	1.977216368757
30801	O	-4.334395856199	3.216947766633	3.393184354339
30802	H	-5.092169120198	2.677592685545	3.158735217192
30803	H	-4.242518222636	3.860875485494	2.653724266127
30804	O	5.816096369689	-1.126611615032	0.257143106629
30805	H	5.231608458344	-1.079602176105	-0.514619479264
30806	H	5.987490920921	-0.217939130400	0.522306628621
30807	O	0.913086479855	1.972658926608	4.795784668519
30808	H	0.922403697212	1.271448988417	4.108680219783
30809	H	0.313620593260	1.603268477025	5.482731300280
30810	O	-2.008953616527	3.474632528118	-2.502817661818
30811	H	-1.213791292139	4.006492738854	-2.393285453953
30812	H	-1.966809878231	3.129418469277	-3.409671758211
30813	O	1.174696508693	-0.308330873358	3.339029938526
30814	H	1.273802153595	-0.710394084463	4.229823694767
30815	H	2.088665352837	-0.086767784224	3.066887045668
30816	O	2.152199497876	-0.146333210781	-3.797184695808
30817	H	1.736816576331	-1.028698888159	-3.919425337111
30818	H	2.896222817826	-0.322051334789	-3.199764994161
30819	O	-2.141400723539	0.145464959964	-3.643586431315
30820	H	-2.711219937791	-0.368363587852	-3.023699327903
30821	H	-2.668754760389	0.873596820694	-3.986481673886
30822	O	-3.901539446176	-2.195110468546	2.644290138648
30823	H	-4.840751943276	-2.289310061578	2.487504736555
30824	H	-3.510295403562	-1.834324578473	1.809846969717
30825	O	-1.339160995746	5.552982286058	1.513642588696
30826	H	-1.542696459111	6.287081059400	2.095219366147
30827	H	-0.980927588602	4.844203606218	2.088492316246
30828	O	0.193746806496	-4.185824674497	-1.990322936915
30829	H	0.065612987599	-4.260686101721	-1.033551924658
30830	H	-0.701862849888	-4.096239303991	-2.362032326421
30831	O	-0.316323599270	3.551717174609	3.002269883401
30832	H	-1.006508331107	2.867497472872	2.861566491065
30833	H	0.156888651890	3.241384861295	3.796876840389
30834	O	-0.216081345462	-4.427916599823	0.864812334748
30835	H	-0.991071337458	-4.363641124834	1.473529619294
30836	H	0.127374618025	-5.315730747706	0.977784817069
30837	O	5.249354440667	1.784695998336	1.475856547489
30838	H	5.094615721857	2.724143215606	1.683852532391
30839	H	5.138386882051	1.710842181612	0.506356411818
30840	O	-5.136380386812	0.993351273402	-2.505945129733
30841	H	-4.874047840793	1.788039461732	-1.979463907763
30842	H	-6.078912899557	1.074272832497	-2.646356453221
30843	O	-0.560158821067	0.383931964776	6.332807910541
30844	H	0.107177752412	-0.314443808265	6.353219287364
30845	H	-1.262485046831	0.073094166673	5.743074702231
30846	O	-4.191785322070	1.179385193870	1.024764814925
30847	H	-3.902402119515	0.297625247485	0.743689833848
30848	H	-3.494783518419	1.470255751598	1.641067328486
30849	O	4.155295117469	-1.119600705150	-2.109429889438
30850	H	4.864969721489	-1.329485536350	-2.720318404886
30851	H	3.713341409708	-1.991695902266	-1.918684339383
30852	O	0.758380880494	5.872350146798	-0.229279063452
30853	H	0.620378209124	5.312454109754	-1.016219834796
30854	H	-0.083898296926	5.878215225707	0.259075064661

```
O     -3.695133154628   -1.239478646387   -1.996627076230
H     -4.411498300344   -0.583486781776   -2.081955018734
H     -3.428015649185   -1.253986136888   -1.051668025389
O     -0.589921671509   -2.879922289071    4.796831115377
H     -0.069726750016   -2.772973388963    5.595860746308
H      0.066936293107   -2.952082373155    4.062674938205
O      0.246290961956    0.639689598224   -5.804891880816
H      1.068019000280    0.571606955765   -5.301074690862
H     -0.118778721010   -0.253972386747   -5.824109878919
O     -2.195736717866   -3.386884419294   -3.126960698640
H     -2.819463693196   -2.787986311967   -2.695081078609
H     -1.956728513983   -2.965959406577   -3.964170014522
O     -1.687648417702    2.459154468638   -5.110167323377
H     -1.619751136177    3.190457525049   -5.723925495899
H     -0.956995082197    1.846733392086   -5.341539079053
O      3.749356594681    0.283697794972    3.031948012185
H      4.305639532261    0.863574163827    2.454273452557
H      3.857758482632    0.601767605601    3.941093967512
O      3.297107272612    1.121736877331    5.648154895517
H      3.782749479811    1.746967109890    6.184878539595
H      2.459266617951    1.570137543682    5.375778360166
O     -4.285148569101    2.991103436961   -0.946970210331
H     -4.157887072735    2.341839843506   -0.216290883100
H     -3.425805922757    3.159129794944   -1.367047610451
O     -2.231285733087    1.680523619864    2.895780983416
H     -2.137955457993    0.878952068596    3.440215781052
H     -2.937208677994    2.232877162448    3.305014299266
```
